# Investigating dynamic and energetic determinants of protein nucleic acid recognition: analysis of the zinc finger zif268-DNA complexes

**DOI:** 10.1186/1472-6807-10-42

**Published:** 2010-11-24

**Authors:** Rubben Torella, Elisabetta Moroni, Michele Caselle, Giulia Morra, Giorgio Colombo

**Affiliations:** 1 Istituto di Chimica del Riconoscimento Molecolare, CNR. Via Mario Bianco 9, 20131 Milano, Italy; 2 Università di Torino, Dipartimento Fisica Teorica, I-10125 Torino, Italy

## Abstract

**Background:**

Protein-DNA recognition underlies fundamental biological processes ranging from transcription to replication and modification. Herein, we present a computational study of the sequence modulation of internal dynamic properties and of intraprotein networks of aminoacid interactions that determine the stability and specificity of protein-DNA complexes.

**Results:**

To this aim, we apply novel theoretical approaches to analyze the dynamics and energetics of biological systems starting from MD trajectories. As model system, we chose different sequences of Zinc Fingers (ZF) of the Zif268 family bound with different sequences of DNA. The complexes differ for their experimental stability properties, but share the same overall 3 D structure and do not undergo structural modifications during the simulations. The results of our analysis suggest that the energy landscape for DNA binding may be populated by dynamically different states, even in the absence of major conformational changes. Energetic couplings between residues change in response to protein and/or DNA sequence variations thus modulating the selectivity of recognition and the relative importance of different regions for binding.

**Conclusions:**

The results show differences in the organization of the intra-protein energy-networks responsible for the stabilization of the protein conformations recognizing and binding DNA. These, in turn, are reflected into different modulation of the ZF's internal dynamics. The results also show a correlation between energetic and dynamic properties of the different proteins and their specificity/selectivity for DNA sequences. Finally, a dynamic and energetic model for the recognition of DNA by Zinc Fingers is proposed.

## Background

Protein-DNA recognition mechanisms underlie the functioning and regulation of several cellular processes ranging from transcription to replication, modification and restriction. Consequently, it is not surprising that questions on how to achieve a detailed molecular understanding of these phenomena have emerged since the first X-ray structures of complexes appeared.

One of the central problems involves the understanding of how a certain binding protein efficiently selects a specific target sequence from a large number of possible sites [1]. Initial studies concentrated on the specific hydrogen bonding between aminoacid side-chains and DNA bases [2]. This initial picture evolved to a more complex one [3] in which several additional factors have to be taken into account: electrostatics [4-9], the effects of localized water molecules [10,11] and general solvation effects [12-14], shape complementarity [15], DNA deformation have all been shown to play a critical role [16-23].

However, despite significant progress at the experimental and theoretical level, the molecular determinants of the events at the basis of protein-DNA recognition have not been fully characterized.

In this study, we apply all-atom, explicit solvent Molecular Dynamics (MD) simulations to protein-DNA complexes that show the same overall 3-Dimensional (3D) structures but differ for point mutations in either the protein or the DNA. Experimental data show that these sequence-differences have an impact on the affinity and specificity in recognition. Our goal here is to study the applicability of novel theoretical/computational approaches to map the principal energetic interactions and internal dynamic properties of complexes to investigate the determinants of stability, selectivity and specificity of different mutants with the same 3 D organization for selected DNA sequences.

As a model system we chose the Zinc Finger (ZF) proteins of the Zif268 family [24,25]. Zinc fingers represent one of the most recurrent motifs among eukaryotic DNA-binding proteins. ZFs specifically recognize and bind their target nucleotide sequences [1]. In particular, Zif268 (subsequently re-named Egr1) is a nuclear protein with transcriptional regulating functions: the transcripts activated by this molecule code for proteins required for cell differentiation and mitogenesis. The importance of this protein family increased after its relationships with p53-regulated apoptotic pathways were clarified [26-28].

Zinc Fingers of Zif268 belong to the C2H2 family (where Zn is coordinated by two Cys and two His residues) and are characterized by a modular structure featuring three repeated domains [24,25]. Each finger consists of about 30 aminoacids and contains a short β-sheet and one α-helix. The two secondary structures are held in a compact conformation by a small hydrophobic core and the presence of the Zn ion that coordinates two Cys residues from the β-sheet and two His residues from the α-helix.

Analyses of X-ray data of the Zif268-DNA complexes revealed that residues at the four specific positions, -1, 2, 3 and 6 (numbering with respect to the start of the α-helix) in helix 1 make most of the contacts to the DNA stretch [24,25]. To evaluate the effects of variations in the protein sequence on the DNA binding specificities, Rebar and Pabo used phage display approaches to prepare a library of variants randomizing the four critical aminoacids in the first Zinc Finger of Zif268 [24]. Affinity selections using DNA sequences with base variations in the region recognized by the protein, allowed to identify protein variants that could bind specifically to new DNA sites. Dissociation constants were then determined for each selected protein in complex with its DNA-target sequence [24]. Crystal structures of the complexes between wild type or mutated proteins with their target sequences were also obtained. Overall, the different structures showed a high degree of similarity [25].

**Figure 1:**
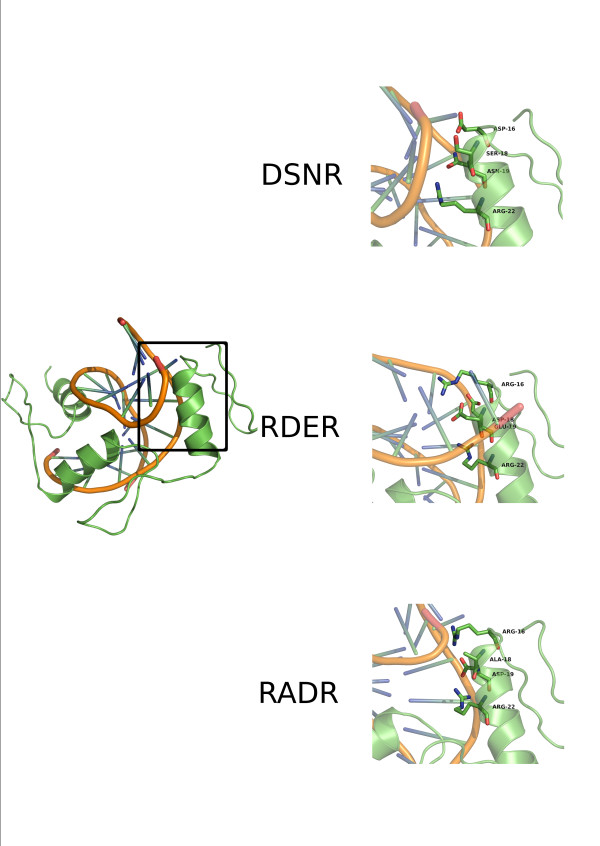
**X-ray structures of the complexes simulated**. The figure represents the initial structures of the complexes used in this study. Protein residues in contact with the DNA stretch and mutated are highlighted as sticks.

These experimental structures of different complexes were used as starting points for multiple copies of MD simulations. Three simulations were thus started from each crystal structure with different sets of random initial velocities. We thus obtained a total of 60 ns simulations for each complex in set of seven different systems, resulting in a total of 420 ns of MD simulation in explicit solvent. The complexes, their pdb names, the different aminoacid motifs on the helix and the respective DNA sequences that they bind are reported in Table 1 and Figure 1. Different groups of proteins are labeled according to the residues helix residues -1, 2, 3 and 6 that make direct contact with the DNA stretch. According to this definition, our study involves two variant proteins (DSNR and RADR) in complex with different DNA sequences. A sequence alignment of the proteins is also provided in Additional file 1, **Figure S1**, visualizing the high identity of the sequences and their differences.

**Table 1:** Complexes and Dissociation Constants.

PDB Code	Aminoacid sequence at positions -1, 2, 3, 6	Nucleotidic sequence at positions 8-11	Dissociation Constant (Kd, nM)
1A1F	DSNR	GACC	0.019

1A1G	DSNR	GCGT (wild type)	1.8

1A1I	RADR	GCAC	0.068

1A1J	RADR	GCGT (wild type)	0.035

1A1K	RADR	GACC	9.3

1A1L	RDER (wild type)	GCAC	5.6

1AAY	RDER (wild type)	GCGT (wild type)	2.7

This table reports on the names of the complexes, the specific sequences on Helix 1 that recognize DNA, DNA sequences, and the respective dissociation constants.

Analysis of the trajectories of each complex was then carried out to map the dynamic residue-residue coordination and rigidity distribution, the principal energetic interactions and the differences in their profiles.

The results showed differences in terms of the organization of the intra-protein energy-networks responsible for the selection and stabilization of the protein conformations recognizing DNA, and consequently for the specificity. Moreover, sequence differences in the protein and DNA-mutations are reflected into different modulation of the ZF's internal dynamics. The results also showed a correlation between energetic and dynamic properties of the different proteins and their selectivity/specificity for DNA sequences. Finally, we propose a dynamic and energetic model for the recognition of DNA by Zinc Fingers that may be useful to improve our understanding of the physico-chemical bases of protein-DNA recognition mechanisms.

## Results

### Structural Parameters

The time evolution of each protein's atom-positional Root Mean Square Deviation (RMSD) from the initial X-ray structures was evaluated after combining the data from the three independent trajectories for each complex, as described in Materials and Methods. In all cases, RMSD values stabilized around 0.2 nm, showing the substantial stability of the complexes in the simulation conditions (Additional file 1, **Figure S2**). The calculation of average RMSD values obtained by comparing all the structures visited by each trajectory with all the structures visited by the other trajectories also yielded an average value of 0.2nm, showing high degrees of structural similarity among the different complexes. The overall conservation of structural properties was also apparent in the secondary structure analysis. No major variation could be detected, indicating the absence of large conformational changes or folding-unfolding events.

### Protein Flexibility in Relation to Affinity and Specificity

Next, possible correlations between the dynamics of each protein and its DNA-binding characteristics were evaluated. First, Covariance analysis and Essential Dynamics (ED) were carried out on the trajectories of the complexes. ED identifies relevant low-energy displacements of groups of residues and emphasizes the amplitude and direction of dominant protein motions by projecting the trajectories on a subset of the principal eigenvalues and eigenvectors of the residue pair covariance matrix calculated from MD [29,30]. Using this approach, protein regions responsible for the most relevant collective motions could be identified, and the information used to illuminate the effects of the protein and/or DNA sequences on recognition and binding.

**Figure 2:**
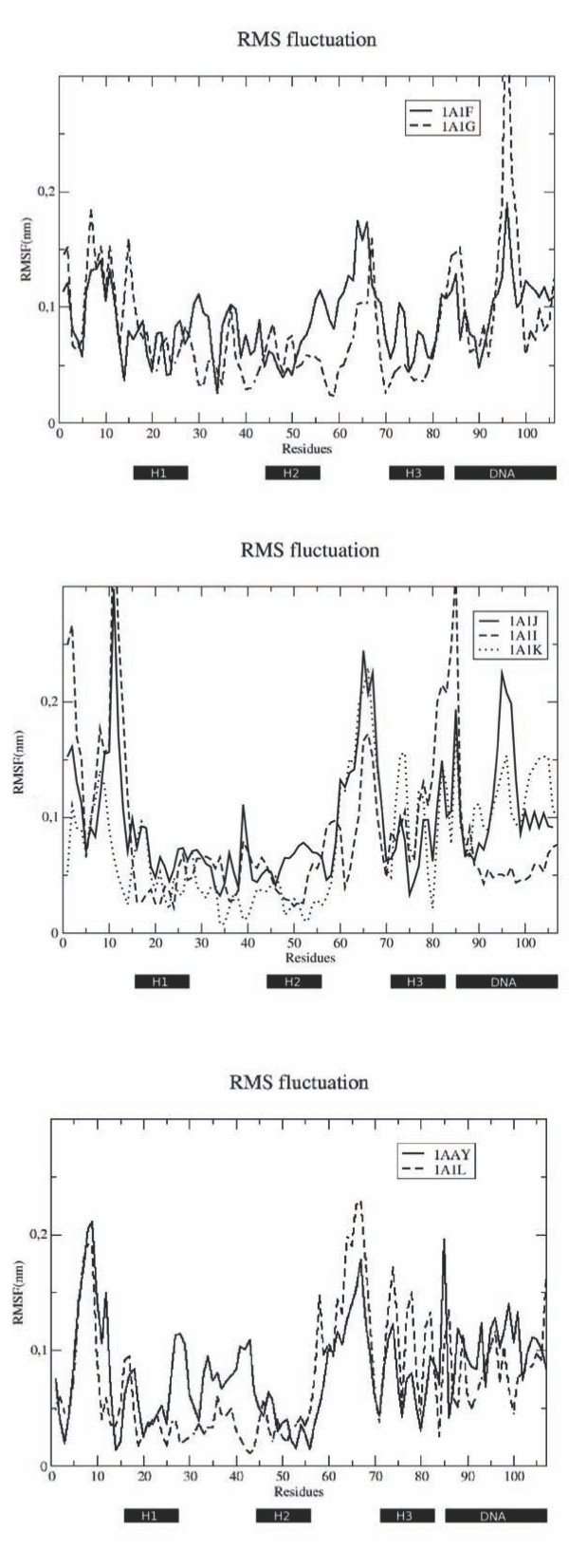
**RMSF of the complexes during the MD simulations**.Residue-based Root Mean Square Fluctuations. For the proteins the calculation is carried out on the Cα atoms; DNA fluctuation, the fluctuations of the C1 atoms are considered. Fluctuations are in nm.

The residue-based Root Mean Square Fluctuations (RMSF) were calculated by projecting each trajectory on the essential subspace defined by the eigenvectors responsible for 90% of the total variance (Figure 2). Differences emerge within different complex families. In the case of the DSNR binding sequence, the protein in the complex 1A1F shows generally larger fluctuations compared to 1A1G. Interestingly, for these two proteins higher fluctuations characterize the protein with higher affinity for DNA (Figure 2).

Differential fluctuations can also be noticed in the analysis of RADR sequences. Consistently with the previous observation, the proteins with higher fluctuations display better DNA-binding properties in their respective complexes (1A1I and 1A1J), compared to 1A1K. (Figure 2).

The trend of flexible protein and rigid DNA was also conserved also in the REDR sequences (Figure 2).

The picture emerging from the analysis of these data is that, within each single group of ZF, higher dynamics appears to be correlated with lower Kd values. In order to gain more quantitative insights into how protein flexibility determines specificity in general, and not limited to a single protein sequence, we defined a measure of global protein flexibility and explored their correlations with the value of the dissociation constant Kd (Table 2).

**Table 2:** Fluctuations and Specificity.

PDB Code	Aminoacid sequence at positions -1, 2, 3, 6	Kd (nM)	Total Protein Flexibility (RMSF sum (nm))	Total Complex Flexibility (RMSF sum (nm))
1A1F	DSNR	0.019	7.18	9.49

1A1G	DSNR	1.8	6.05	8.70

1A1I	RADR	0.068	7.72	9.03

1A1J	RADR	0.035	7.6	10.1

1A1K	RADR	9.3	5.87	8.38

1A1L	RDER (wild type)	5.6	6.10	7.97

1AAY	RDER (wild type)	2.7	6.47	8.68

	**Correlation Log(Kd)/RMSF**		**-0.92**	**-0.88**

The complexes simulated, the dissociation constants, the total flexibility of each system evaluated as a sum of the Root Mean Square Fluctuations (RMSFs) of the proteins and of the complexes are indicated. Correlations between Log(Kd) and RMSFs are indicated in the last row of the table.

First, we summed up all the residue-based RMSF values for each protein and plotted the resulting value of the flexibility parameter against the respective log(Kd) value. The log of Kd was chosen to calculate the correlations, as this quantity is directly related to differences in free energy or entropy between different complexes (*vide infra)*. It is to be noted that the direct linear correlation of Kd values with calculated parameters yields comparable correlation values, in all cases. Interestingly, the calculation of correlation between log(Kd) and the RMSF flexibility parameter yielded a correlation coefficient of -0.92. When considering also the fluctuations of the DNA stretch in the calculation, the correlation coefficient resulted -0.88 (Table 2).

**Figure 3:**
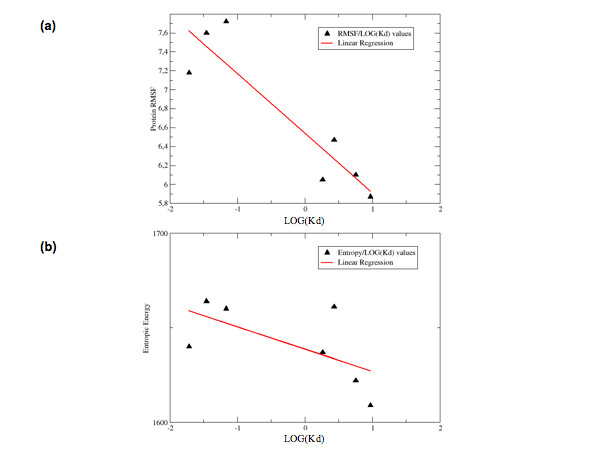
**Correlation between Flexibility and Entropy values and Log(Kd)**. **(a) **Complex flexibility calculated on the basis of RMSF are plotted against the Log(Kd). **(b) **Complex entropies calculated according to the Schlitter's approximation are plotted against the Log(Kd). The line representing the best linear fit is represented.

The correlations described for Table 2 are reported pictorially in Figure 3a.

A modulation of internal flexibility may also be related to a modulation of the conformational entropy. Indeed, upon evaluating each protein's conformational entropy with the Schlitter's approximation [31], and plotting it against the respective log(Kd), a correlation coefficient of -0.63 was found (Figure 3b). It worth noting, at this point, that these quantitative correlations can be obtained only after combining the statistics from different trajectories. This underlines the importance of significant sampling, even when starting simulations from well-defined X-ray structures.

Our results indicate a clear, non-trivial anticorrelation between affinity and flexibility for different sequences, suggesting that in general ZF proteins may specifically select oligonucleotide sequences by adapting their conformational ensemble to the rather rigid DNA target. In this framework, the possibility to access a diverse and larger pool of conformations for more flexible proteins (compared to rigid ones) favors the selection of the fine-tuned interaction motifs necessary to stabilize a certain complex.

### Protein Internal Dynamics, Coordination and Rigidity

Differential flexibility plays a key role in helping different proteins select different, and relatively rigid, DNA targets [6,32]. Analysis of helix 1, the secondary structure element where mutations are located and which should directly sense the binding to DNA, does not highlight significant differences among mean fluctuations in different proteins. In contrast, differences can be noticed in the fluctuations of helix 2 and 3 (Figure 2). This suggests that the structural and conformational effects of protein-DNA interaction directly involving helix 1 can be transmitted long-range to different regions of the protein. Variations due to changes in the side-chain interactions may thus be reflected in the collective modification of the dynamics allowing a certain protein sequence to adapt to a specific DNA stretch.

To gain more insights into these points, we applied a novel method for the analysis of the dynamic connectivity within a protein. Our approach aims at the quantitative description of the degree of internal coordination between residue pairs in the presence of dynamics.

Internal dynamic coordination is recapitulated by means of the ICRM (Internal Coordination and Rigidity Matrix) matrix (See Materials and Methods). According to the definitions presented in Materials and Methods, the elements of the matrix, *Rij*, describe how residue pairs are *dynamically connected: *high *Rij *values are due to low distance fluctuations and therefore detect residue pairs characterized by high dynamical coordination. On the other hand low *Rij *values describe poorly correlated moving pairs. Coordination between neighbouring residues may be a simple consequence of local interactions, while strong coordination between residues located at high distances sheds light on long-range correlations. In this model, the lower the distance fluctuation between two residues, the better their coordination. Groups of locally highly coordinated residues identify protein's rigid sub-structures that may have specific functional roles.

**Figure 4:**
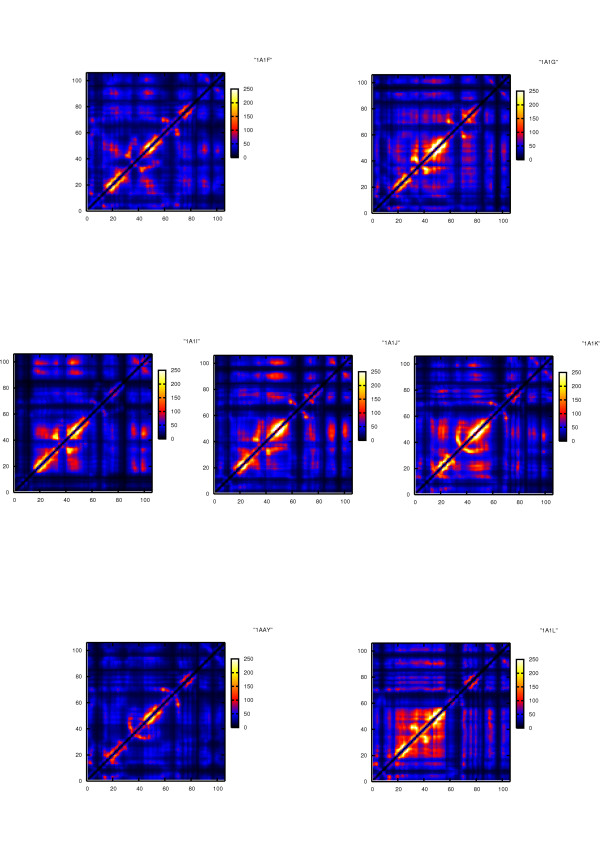
**ICRM matrices for complexes simulated in this study**. The diagonal and the nearest neighbors (i.e., the Rij values for i = j, i = j ± 1) have been normalized to zero in order to avoid divergence to infinity during calculations. Units are [nm]-2.

The representative matrices for each complex are displayed in Figure 4. The results show that the protein's coordination pattern changes significantly as a function of the sequence of the protein and of the bound DNA. In general, the proteins of a certain family (defined based on the aminoacid sequence that binds to DNA) with higher affinity for a certain DNA stretch show a lower degree of internal coordination (1AIF, 1A1J, 1A1Y). In these cases, internal coordination is concentrated at the level of the three α-helices, with the connecting loops showing little dynamic coordination with the rest of the system. In contrast, the dynamics of proteins with lower affinities shows higher internal coordination, also involving the helix-connecting loops.

Dynamic coordination and rigidity (flexibility) can be unequally distributed among different secondary structures within each protein, and this distribution may change when considering different systems.

**Figure 5:**
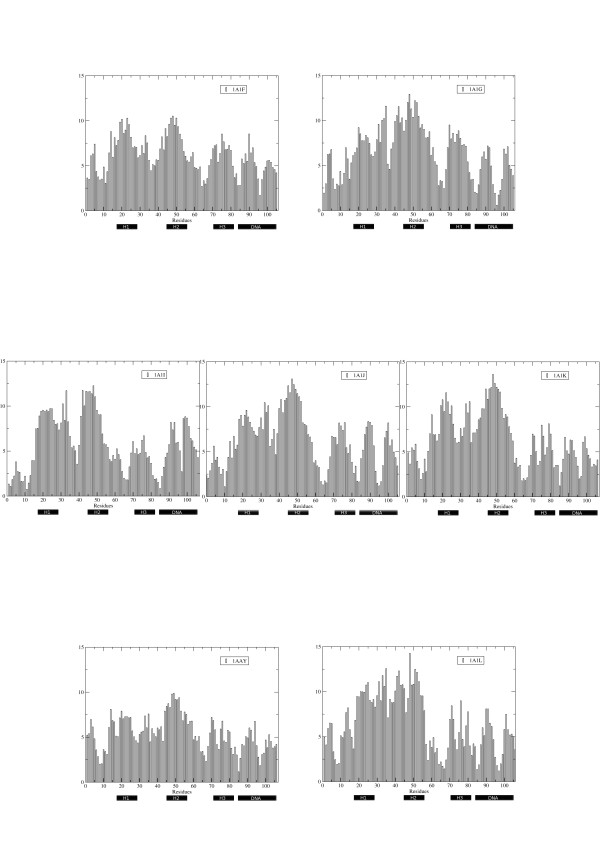
**Residue-based rigidity profiles**. Profile of the main eigenvector derived from the eigenvector decomposition of the ICRM matrix for each of the simulated complexes.

To investigate these aspects and extract the principal features of internal dynamics, the matrices were simplified through eigenvector decomposition. The resulting main eigenvalue can be considered as a general parameter related to the overall degree of dynamic connectivity within a protein, while the corresponding eigenvector reports on the role of each residue in defining the global dynamic properties of the matrix. In this context, the principal eigenvector provides a direct and compact way to combine the linear sequence description with information on global dynamic properties. Higher values of the component indicate a higher contribution of the residue to the global rigidity (coordination) of the complex (Figure 5).

In this frame of thought, the components of the principal eigenvector weighted by the respective eigenvalue were calculated for each complex. Figure 5 reports on the different collective coordination profiles, ordered based on the respective protein sequence. Dynamic differences between the complexes seem to be especially evident for the helices 2, 3 and for the loop connecting helix 1 and 2. This observation is consistent with a model where the effects of mutations on helix 1 are propagated long-range across the protein structure, modulating the recognition and binding properties of the different mutants.

Overall, these data suggest that ZF proteins may exploit flexibility-rigidity modulation to facilitate DNA recognition and binding. The affinity for a DNA stretch may thus be linked to differences in the underlying dynamics as a function of small changes in the sequence and of the identity of the binding partner. These considerations corroborate the hypotheses of Elrod-Ericksson and co-workers who postulated flexibility as a necessary property for a protein to bind and optimally adapt to the relatively rigid DNA structure [25].

### Energetics of Complexes

Next, we set out to calculate the principal energetic interactions that are involved in the stabilization of each complex, and are ultimately responsible for specificity. To this end, we applied the energy decomposition approach [33-39]. This method was introduced to extract the major contributions to energetic stability of the native structure of a protein from all-atom molecular dynamics (MD) simulations, and its results have been verified and benchmarked against a diverse set of experimental data [33-39]. For a system of *N *residues, the matrix of average non-bonded interactions between pairs of residues is built from an MD trajectory. All the matrices with average energy values and corresponding error bars are provided in Additional files 2, 3, 4, 5, 6, 7 and 8. The energy map is then simplified through eigenvalue decomposition. Analysis of the *N *components of the eigenvector associated with the lowest eigenvalue (called first eigenvector and first eigenvalue, respectively) was shown to single out those residues (hot sites) behaving as strongly interacting and possible stabilizing centers. The eigenvector associated with the main eigenvalue provides a compact description of the participation of each aminoacid to the global stabilization of a structure or a complex. The properties of this first eigenvector (labelled Sequence Eigenvector, SE) ultimately depend on the sequence.

Thus, the approximated energy obtained after the matrix decomposition (see Materials and Methods) accounts for the main attractive interactions that stabilize a certain state.

According to our approximation, any two residues *i *and *j *in a system interact with an energy $\lambda_{1}w_{i}^{1}w_{j}^{1}$ , where λ_1 _is the first eigenvalue and *wi*1 is the component of the associated first eigenvector contributed by residue *i*. The summation over all possible residue-pairs of the energetic couplings provides the effective approximation to the stabilization energy. The contribution of a specific residue *i *to the overall effective stabilization energy is thus the product of *wi*1 with all other *wj*.1

In this framework, it is possible to calculate the effective approximation to the total stabilization energy as a sum of intraprotein (or protein-protein), protein-DNA and intra-DNA (or DNA-DNA) stabilization energy, as reported in Table 3.

**Table 3:** Energetics of Complexes.

PDB Code	Protein-Protein	Protein-DNA	DNA-DNA	Total Stabilization Energy	Intra-protein
1A1F	-58,47	-414,60	-734,92	-1207,99	-513,38

1A1G	-64,77	-432,7	-722,54	-1220,01	-390,68

1A1I	-65,75	-433,5	-714,52	-1213,77	-367,53

1A1J	-66,12	-438,35	-726,55	-1231,02	-440,24

1A1K	-61,61	-423,81	-728,83	-1214,25	-137,19

1A1L	-89,11	-501,37	-705,23	-1295,71	-210,41

1AAY	-64,44	-432,67	-726,34	-1223,45	-342,07

Energetic contributions calculated according to the Energy Decomposition Method due to intraprotein, protein-DNA and intra-DNA contributions are indicated. The last column expresses intra-protein stabilization energy calculated only on the proteins, in the presence of the respective DNA. All the energetic values are in **Kcal/mol**

Analysis of the results shows that the highest contributions to the global stabilization of the complexes are provided by DNA-DNA (60% of the total stabilizing energy) and protein-DNA interactions (35%).

**Figure 6:**
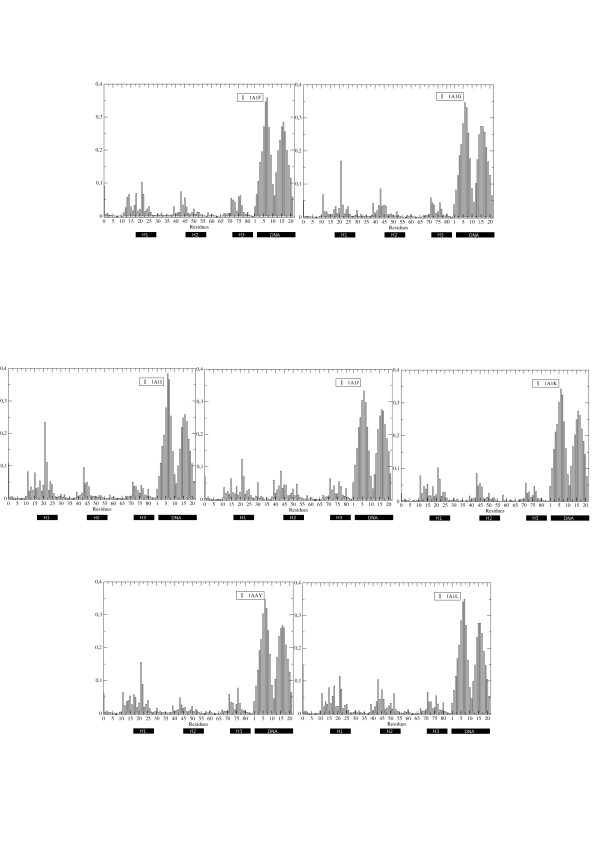
**Residue-based energetics calculated on the whole protein-DNA complex**. Profile of the main eigenvector extracted from the interaction energy matrix for each of the whole protein-DNA complexes.

These properties are reflected at the level of the components of the first eigenvectors derived from the decompositions of the energy matrices for each system. In all cases, the components due to the DNA bases show dominant values with respect to the protein residues, with little apparent modulation of the energy component profile that can be ascribed to different protein sequences (Figure 6). DNA-DNA and Protein-DNA interactions contributing to the overall stabilization are electrostatic in nature and involve the negative charges on the nucleic acid and the positive charges on protein groups. The high intensity of these interactions prevails on the energetic effects of subtle changes within the oligonucleotide stretch or at the protein-DNA interface. As a consequence, no specific correlation could be found between these terms and the relative Kd values for different sequences.

**Figure 7:**
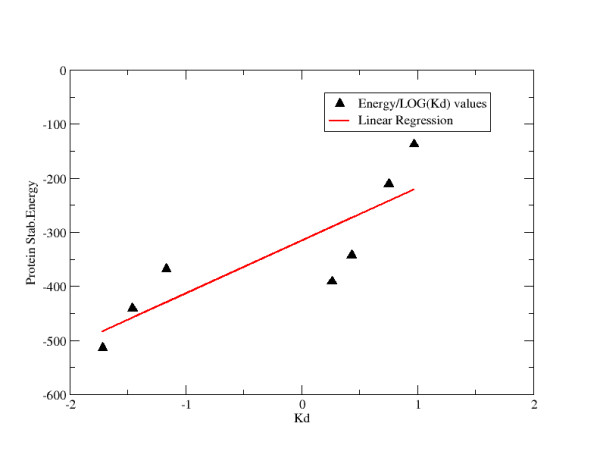
**Correlation between Intraprotein Energy values and Log(Kd)**. Intraprotein stabilization energy values (Kcal/mol) are plotted against the Log(Kd). The line representing the best linear fit is represented.

The contributions involving intraprotein residue-residue pair interactions account for about 5% of the total stabilization energy. Strikingly, the correlation between the contributions to the stabilization energy due only to intraprotein residue-residue interactions and the dissociation constant (log(Kd)) yielded a value as high as 0.85 (Figure 7), indicating that this energetic descriptor is able to catch the main determinants of specificity in the ZF-DNA recognition (Table 3). Once more it is important to underline that this quantitative result could be obtained only after calculating averaged contributions on the combined trajectories in each complex.

From the physico-chemical point of view, this result indicates that the internal energetic distribution of the protein reorganizes specifically in response to the presence of a certain DNA stretch and in response to specific sequence mutations.

**Figure 8:**
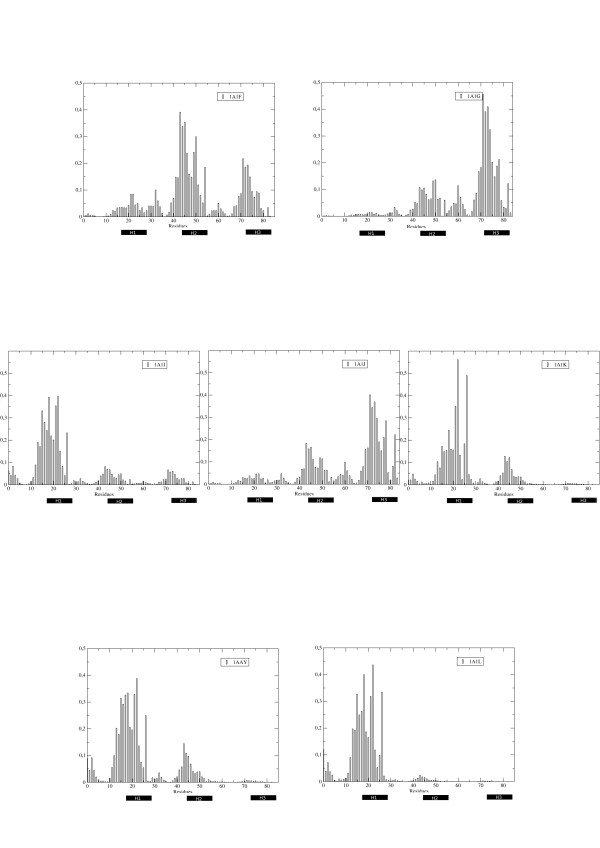
**Residue-based energetics contributed by intraprotein interactions**. Profile of the main eigenvector extracted from the energy matrix calculated only for intraprotein interactions for each of the simulated complexes.

**Figure 9:**
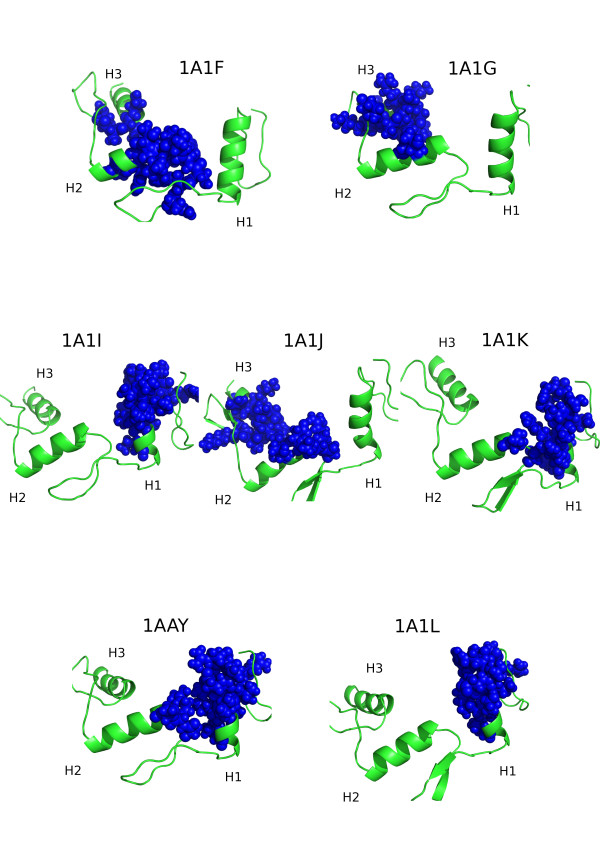
**Structural representation of the hotspot distribution in each complex**. The residues corresponding to the highest components of the energy eigenvectors depicted in Figure 7 are plotted as space filling representations on the 3 D structures of each protein. The final results show different contiguous networks of strongly interacting residues, which in turn differentially modulate affinity for the DNA stretch.

Consistently with what reported for the dynamic characterization of the complexes, analyses of the matrices of intraprotein residue-residue energy couplings showed specific patterns of modulation of the internal interaction networks (Figure 8; Figure 9), reflecting DNA effects on the organization of the internal distribution of interactions in the protein. Eigenvector decomposition shows that mutations on helix 1 influence distant regions of the protein and are not limited to local perturbation effects (Figure 8; Figure 9).

Summarizing, the results of our energy analysis suggest that recognition and binding properties are linked to a specific distribution of the stabilization energy. As expected, the global stabilization of the complex is mainly due to electrostatic interactions bringing the protein and DNA together. Modulation of affinity for a specific DNA sequence is further regulated through a redistribution of the stabilizing intraprotein interactions that strongly depends on the protein sequence.

The energy contributions we calculate with the energy decomposition method are to be considered as the effective energies approximating the free energy of binding in a situation where the unbound state is set to a common reference state (ensemble of unbound states) in which the non-bonded energy is equal for all sequences. Given the high degree of similarities of the sequences, this is a viable hypothesis, and was already shown to hold for the study of folding-unfolding of related proteins [33].

## Discussion

In this paper, we have concentrated on protein-DNA recognition, using MD simulations to investigate the global mechanisms by which Zinc Fingers bind to and modulate their affinities for given oligonucleotide sequences. DNA-binding proteins are in general able to efficiently find their binding sites, amongst large numbers of alternative genomic sequences [1,40,41]. Conformational dynamics and specific energetic factors underlie the process. In some cases one of the two factors may be prevalent, so that either enthalpic or entropic factors mainly determine the complex formation reaction.

In order to evaluate these contributions and shed light on the molecular details underlying protein-DNA recognition and specificity, we have applied novel methods to the analysis of simulation data. To this end, we have extended our analysis of signal transduction [42,43] and protein energetics [33-39,42] to obtain a compact description of the effects of mutations in the sequence of either the protein or the target DNA (or both) on internal collective dynamics and on interaction networks responsible for the stabilization of a certain complex.

The results of our analysis, based on the statistics obtained from the combination of multiple trajectories for each system, show quantitative correlations between the degree of protein flexibility and intraprotein residue-residue coupling energies with Kd values for the protein-DNA complexes under study.

The emerging picture is that selectivity and specificity are strictly correlated to lower rigidity and higher conformational entropy of the protein. In particular, the loop regions connecting different secondary structures emerge as the least coordinated and rigid motifs in the proteins showing the highest affinities. The possibility to visit a higher number of conformational states on the binding energy landscape may represent an advantage for the protein in the adaptation to the rigid-body like structure of DNA, and would allow the search for the best possible set of stabilizing interactions. Moreover, our results show that sequence variations in helix 1 determine long-range effects altering the dynamics of helices 2 and 3, suggesting a cooperative perturbation of the conformational dynamics that extends beyond the point of mutation and influences the dynamics of the whole protein in the complex.

From the energetic point of view, the application of the energy decomposition method to the trajectories of the different complexes revealed that the modulation of specific intraprotein interaction networks in response to the presence of a certain DNA-stretch quantitatively impacts on the binding affinities. Interestingly, the proteins with higher affinities (1A1F, 1A1J and 1AAY) are characterized by a more spread distribution of stabilizing residues, distributed all along the sequence. A diffuse network of strongly interacting residues once more suggests the importance of cooperative effects in determining complex stabilization and specificity.

These observations are consistent with the recent observations by Miyazono et al [44]. The authors demonstrated, through X-ray crystallography and binding assays, the fundamental role played by extended regions in determining the specificity of homeodomain proteins for DNA and the cooperativity of the binding mechanisms. Extended, flexible regions were suggested to promote the diversity of recognition mechanisms necessary for DNA-recognition, and their deletion was shown to dramatically decrease the cooperative character of the DNA-binding event.

Overall, our calculations and models suggest that both flexibility (i.e. entropic factors) and energy modulation (enthalpic factors) contribute to the affinity and selectivity of the Zinc Fingers examined here for their target DNA sequences. The two factors are strictly interconnected: the distribution and modulation of dominant interactions reverberates in the corresponding dynamics of the complexes. Indeed, from the thermodynamic point of view, Kd is related to the complexation reaction free energy (ΔG), which is ultimately determined by the combination of internal energy and entropy. The good correlations with Kd obtained for intraprotein energy, entropy and flexibility reflect this point.

From the mechanistic point of view, our calculations suggest that binding to DNA and selection of a certain sequence can be part of a hierarchical process: at the first level, electrostatic interactions due to the charged nature of the oligonucleotide stretch and of the binding site on the protein contribute to stabilize the complex. These interactions are typically long-range and would not allow a fine-tuned discrimination of sequences. Interestingly, all variations at the dynamic and/or energetic level do not involve any significant collective DNA-protein deformations, in line with what was already highlighted by Lavery and coworkers [17,32].

Specificity and selectivity, in turn, can be achieved through the modulation of interactions of differential intensity among specific subsets of residues. In other words, specific energetic interaction patterns throughout the protein structure can determine the accessibility of different sub-sets of conformations that allow the protein to optimally adapt to the structure and sequence of the target DNA.

## Conclusions

Our analyses of collective properties allowed us to appreciate the pervasive effects of perturbations in one limited region of the protein on the global dynamic and energetic determinants of recognition and binding.

Overall, the results of our MD simulations and analyses have suggested that the energy landscape for DNA binding may be populated by dynamically different states, even in the absence of major conformational changes. Energetic couplings between residues may change in response to sequence variations thus modulating the importance of different regions for binding, and the consequent dynamics of protein-DNA complex formation.

From the applicative point of view [45], given the quantitative agreement with experimental data, we conjecture that the approach presented here may be used for the computational design and modification of (small) proteins specific for given DNA sequences. Iterative cycles of *in silico *mutations and evaluation of the dynamic and energetic properties with the methods presented here, would allow to select protein sequences with dynamic and energetic profiles that have maximal similarity with the ones of known proteins with high affinity and specificity.

## Methods

### MD set-up and simulations

All MD simulations were performed using the AMBER 9.0 package [46] with the ff03 force field. Each protein was solvated in a cubic box large enough to contain 0.8 nm of solvent around the complex. The TIP3P water model was used for solvation [47]. A 1 nm non-bonded cutoff was used for van der Waals interactions, while the Particle Mesh Ewald summation method (PME) was used to deal with long-range Coulomb interactions [48]. The Berendsen thermostat was used to control temperature and pressure [49]. Charges on sidechains were chosen to correspond to a pH value of 7. Na+ counterions were added to ensure electroneutrality. All the structural and dynamical analyses were performed with analyses programs from the GROMACS package, after the trajectories were translated to the suitable format.

In the Zif268 protein, each of the three zinc ions is coordinated to two cysteines and two histidines, one ion for each helix. The zinc was modeled as covalently bound to the ligands, and the parameters used to describe the metal, its charge and bonding properties were the ones developed by Merz and coworkers [50].

In our work, 7 protein-DNA complexes were considered: they differ, with respect of the aminoacids placed on residues 16, 18, 19, 22 and of the nucleotides placed on the position 8, 9, 10, 11 and their complementary (Table 1) [24].

Every complex was initially minimized in vacuo by multiple minimizations (200 steps steepest descent plus 200 steps conjugate gradient). After this, each system was solvated. Multiple solute equilibration processes were performed, in order to reorganize the water molecules (100 steps steepest descent + 50 ps equilibration dynamics at constant P and T = 100K).

After the solute equilibration, another system minimization was performed (200 steps, steepest descent); afterwards, the temperature of the system was slowly brought to the desired value of 300K in 3 steps, with subsequent 100 ps NVT equilibration processes at 100K, 200K and 300K).

Finally, a last 100 ps equilibration NPT process was performed. From this final structure a set of 3 different 20 ns MD simulations was performed for each system. Three different sets of initial velocities obtained from a Maxwellian velocity distribution at the desired temperature of 300K were used to yield three different production runs for each complex.

Overall, this protocol resulted in 3 runs for each of the seven complexes studies, providing a total of 420 ns of simulation time. All the analyses were carried out on the combined trajectories obtained by concatenating each of the 3 runs for each complex, after eliminating the first 5 ns of each trajectory to allow for equilibration.

### Covariance Analysis

Covariance matrices were built by averaging motions of Cα atoms of the aminoacids and of the C1 atoms of the deoxyribose ring of the nucleotides, deviating from the mean structure, with the latter calculated over the trajectory. The essential directions of correlated motions during dynamics were then calculated by means of the Essential Dynamics method [51], or principal component analysis of the 3N × 3N covariance matrix *Cij*.

The covariance matrix was also used to calculate the entropic content of the complexes using the Schlitter's approach [31].

### Analysis of Internal Coordination and Rigidity

In order to provide a simple and sequence-related one-dimensional descriptor of the contribution of each residue in defining the dynamic connectivity within the protein, we introduced an analysis based upon signal propagation; a concept originally developed based on elastic network models [52], and then extended to all-atom MD simulation trajectories [42]. Herein we have used a new, compact and efficient metrics to quantitatively describe the degree of internal dynamic coordination between residue pairs in the presence of dynamics by means of a matrix called ICRM (Internal Coordination and Rigidity Matrix) whose elements Rij are defined for every pair of residues i,j as follows:

(1) $$R_{ij}=\left\{\begin{array}{cc} \frac{1}{\sum_{k=1}^{3}\langle \Delta x^{k}ij^{2}\rangle -{\langle \Delta x^{k}ij\rangle }^{2}} & \left|i-j\right|>1\\ 0 & \left|i-j\right|\leq 1 \end{array}\right\}$$

Where Δx_ij_^k ^represents the instant value of the *k*-th cartesian component of the distance between Cα of residues i and j and yields the fluctuation of the distance component when averaged over the trajectory. We set R_ij _= 0 on the diagonal and for nearest-neighbours to avoid divergence. In this way, the ICRM matrix describes how residue pairs are *dynamically connected: *high R_ij _values are due to low distance fluctuations and therefore detect residue pairs characterized by high dynamical coordination. On the other hand low R_ij _values describe poorly correlated moving pairs (i.e., they are poorly coordinated and are characterized by low communication propensity due to high distance fluctuations). Coordination between neighbouring residues may be a trivial consequence of local interactions, while strong coordination between residues located at high distances sheds light on long range correlations. Hence, to summarize, the lower the distance fluctuation between two residues, the better they are coordinated and behaving like two points of a rigid body. Groups of locally highly coordinated residues identify protein's rigid sub-structures, such as secondary structure elements. On the other hand, residue pairs at long distance having low distance fluctuations can be due to mutually coordinated protein sub-domains possibly related to long range correlations.

### Energy Decomposition Method

The energy decomposition method (EDM) [33-39] aims at the identification of crucial residues (hotspots) for the stabilization of a certain structure and its energetic stability. As first step, the method computes the matrix of non-bonded interaction energies (namely, van der Waals and electrostatic interactions) between pairs of residues. This matrix is afterwards diagonalized and, from the analysis of the eigenvector associated with the lowest eigenvalue, it is possible to determine those residues that behave as strongly interacting and stabilizing centers.

Herein, the structures sampled every 1 ns for each complex were taken into account and the average non-bonded interaction energy matrix was computed from averaging non-bonded pair interactions over all the protein structures saved. The solvent is directly taken into account using the GBSA method [53] in the calculation of the non-bonded interactions. This type of averaging calculation allows to obtain strong correlations with experimental free-energy related values, in contrast to the simple use of representative structures of main clusters.

Let us indicate with M the non-bonded interaction energy matrix without the diagonal elements, namely without the self-interaction terms. This matrix can be diagonalized and expressed in terms of its eigenvalues and eigenvectors:

(2) $$M_{ij}=\sum_{k=1}^{N}\lambda_{k}w_{ik}w_{jk}$$

where:

- *N *is the sum of the aminoacids and nucleotides in the complex;

- λ_k _is the *k*-th eigenvalue;

- **W***_ik _*is the *i*-th component of the *k*-th eigenvector;

Eigenvalues and eigenvectors are usually labeled following an increasing order. Therefore, λ_1 _is the lowest eigenvalue and, from now on, we will refer to the first eigenvector as the eigenvector corresponding to the eigenvalue λ_1_.

The total non-bonded energy is defined as:

(3) $$E_{nb}=\frac{1}{2}\sum_{i,j=1}^{N}M_{ij}=\frac{1}{2}\sum_{i.j=1}^{N}\sum_{k=1}^{N}\lambda_{k}w_{ik}w_{jk}=\frac{1}{2}\sum_{k=1}^{N}\lambda_{k}W_{k}$$

with $W_{k}=\sum_{i,j=1}^{N}w_{ik}w_{jk}$. If λ_1_*W*_1 _is much larger than λ_k_*W*_k _for *k*≠1, the sum over *i *and *j *of **M***_ij _*is dominated by the contribution due to the first eigenvalue and eigenvector, such that the total non-bonded energy can be approximated by:

(4) $$E_{nb}\approx E_{nb}^{app}=\frac{\lambda_{1}}{2}\sum_{i,j=1}^{N}w_{i1}w_{j1}=\frac{\lambda_{1}W_{1}}{2}$$

As mentioned above, the eigenvector associated with the lowest eigenvalue is used to identify the most stabilizing aminoacids. In particular, considering its squared components as the weights of the corresponding residues in the structural stabilization, we can define "hot spots" those residues with a weight higher than a threshold *t*. This threshold is chosen equal to the squared component of a normalized "flat eigenvector" (namely, a normalized vector whose components provide the same contribution for each site). This corresponds to a case in which each residue equally contributes to the structural stability and, therefore, the threshold *t *is equal to *1/N*, where *N *is the number of the eigenvector components.

## Abbreviations

ZF: Zinc Fingers; MD: Molecular Dynamics; ICRM Matrix: Internal Coordination and Rigidity Matrix.

## Authors' contributions

All authors contributed to the research presented. EM, RT performed research, Analyzed Data, Interpreted Data. MC was critically involved in developing research. GM, GC designed research, methods, wrote the paper. All authors read and approved the final manuscript.

## Acknowledgements

This work was supported by a grant from AIRC (Associazione Italiana Ricerca sul Cancro) to GC, from the Fondazione Cariplo grant Nr. 2008.2198, and from the Italian Ministry of Health GR-2007 programme.

## Supplementary Material

Additional file 1 **Sequence alignment and structural time evolution of proteins in simulations**. **Figure S1: **The sequence alignment of the protein residues, made with Clustal-W. **Figure S2**.The time dependent RMSD evolution of all trajectories over the combined molecular dynamic trajectories. Click here for file

Additional file 2 **Average pair interaction energies for 1A1F Complex and their standard deviations**. Click here for file

Additional file 3 **Average pair interaction energies for 1A1G Complex and their standard deviations**. Click here for file

Additional file 4 **Average pair interaction energies for 1A1I Complex and their standard deviations**. Click here for file

Additional file 5 **Average pair interaction energies for 1A1J Complex and their standard deviations**. Click here for file

Additional file 6 **Average pair interaction energies for 1A1K Complex and their standard deviations**. Click here for file

Additional file 7 **Average pair interaction energies for 1A1L Complex and their standard deviations**. Click here for file

Additional file 8 **Average pair interaction energies for 1AAY Complex and their standard deviations**. Click here for file
