# Supplementary material for: Investigating dynamic and energetic determinants of protein nucleic acid recognition: analysis of the zinc finger zif268-DNA complexes
Source: BMC Struct Biol. 2010 Nov 24;10:42. doi: 10.1186/1472-6807-10-42 (PMC3002361; doi:10.1186/1472-6807-10-42)
Supplement: Additional file 1 — Sequence alignment and structural time evolution of proteins in simulations. Figure S1: The sequence alignment of the protein residues, made with Clustal-W. Figure S2.The time dependent RMSD evolution of all trajectories over the combined molecular dynamic trajectories. [file 1472-6807-10-42-S1.PDF]

Supplementary Material.

The supplementary material file contains:

Supplementary Material Figure S1. The sequence alignment of the protein residues, made with Clustal-W.

Supplementary Material Figure S2. The time dependent RMSD evolution of all trajectories over the combined molecular dynamic trajectories.

```
CLUSTAL 2.0.12 multiple sequence alignment


RADR          RPYACPVESCDRRFSRSADLTRHIRIHTGQKPFQCRICMRNFSRSRSDHLTTHIRTHTGEKP 60
RDER          RPYACPVESCDRRFSRSDELTRHIRIHTGQKPFQCRICMRNFSRSRSDHLTTHIRTHTGEKP 60
DSNR          RPYACPVESCDRRFSDSSNLTRHIRIHTGQKPFQCRICMRNFSRSRSDHLTTHIRTHTGEKP 60
              *************** * :*****************************************

RADR          FACDICGRKFARSDERKRHTKIHL 84
RDER          FACDICGRKFARSDERKRHTKIHL 84
DSNR          FACDICGRKFARSDERKRHTKIHL 84
              ************************
```

Supplementary Material Figure S1. The sequence alignment of the protein residues, made with Clustal-W.

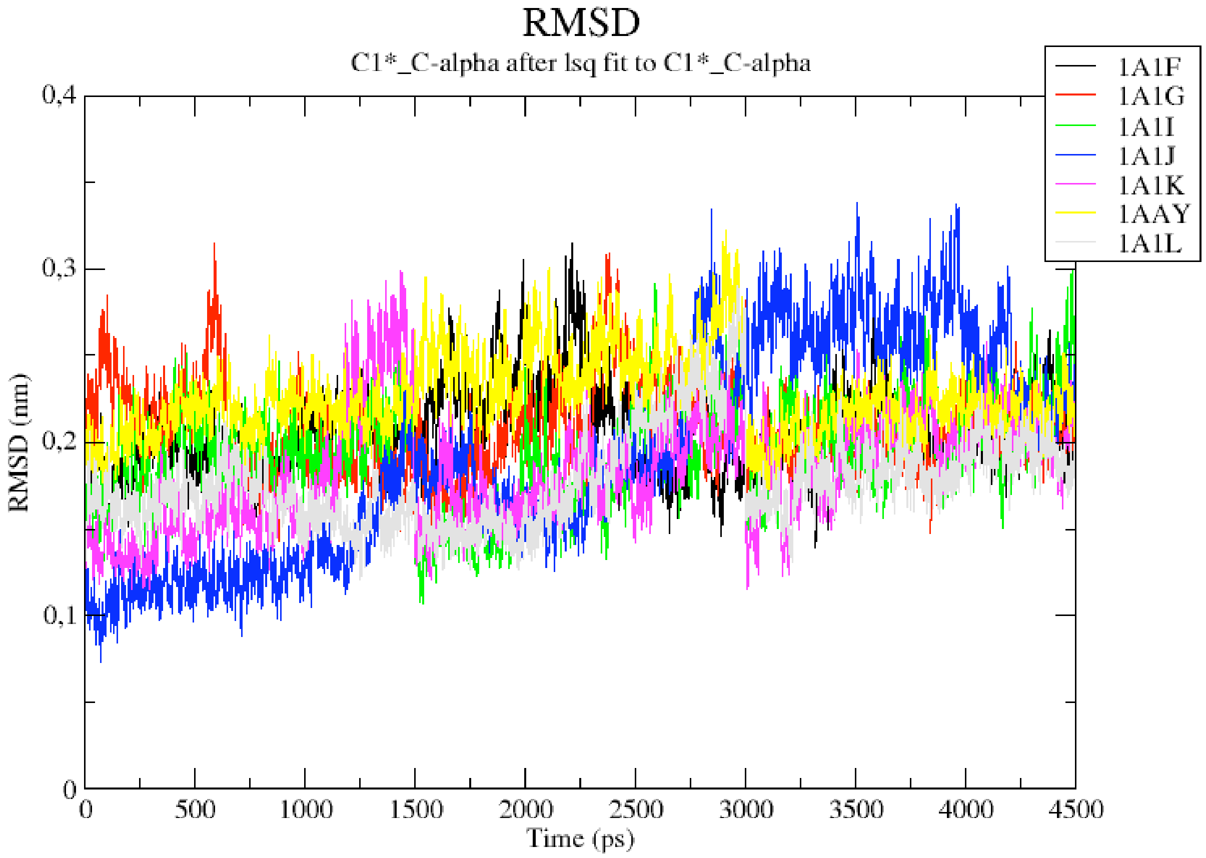

Supplementary Material Figure S2. The time dependent RMSD evolution of all trajectories over the combined molecular dynamic trajectories.
