# Supplementary material for: Investigating dynamic and energetic determinants of protein nucleic acid recognition: analysis of the zinc finger zif268-DNA complexes
Source: BMC Struct Biol. 2010 Nov 24;10:42. doi: 10.1186/1472-6807-10-42 (PMC3002361; doi:10.1186/1472-6807-10-42)
Supplement: Additional file 2 — Average pair interaction energies for 1A1F Complex and their standard deviations. [file 1472-6807-10-42-S2.PDF]

=>> 1A1F

Residue ->	Residue	TGBTOT	SD
1 ->	1	-250.94	4.32
1 ->	2	-0.91	0.36
1 ->	3	-1.17	0.21
1 ->	4	-0.15	0.06
1 ->	5	-0.12	0.01
1 ->	6	-0.00	0.00
1 ->	7	-0.00	0.00
1 ->	8	-0.05	0.02
1 ->	9	-0.00	0.00
1 ->	10	-0.09	0.01
1 ->	11	-0.14	0.02
1 ->	12	0.15	0.49
1 ->	13	-4.36	1.92
1 ->	14	-2.47	0.75
1 ->	15	-2.09	1.32
1 ->	16	-0.52	0.15
1 ->	17	-0.06	0.02
1 ->	18	0.01	0.01
1 ->	19	0.19	0.09
1 ->	20	-0.04	0.01
1 ->	21	-0.00	0.00
1 ->	22	0.11	0.02
1 ->	23	0.00	0.00
1 ->	24	-0.00	0.00
1 ->	25	0.03	0.00
1 ->	26	0.00	0.00
1 ->	27	-0.01	0.00
1 ->	28	0.00	0.00
1 ->	29	0.00	0.00
1 ->	30	-0.00	0.00
1 ->	31	0.02	0.00
1 ->	32	0.00	0.00
1 ->	33	0.00	0.00
1 ->	34	0.00	0.00
1 ->	35	-0.01	0.00
1 ->	36	0.00	0.00
1 ->	37	0.00	0.00
1 ->	38	-0.01	0.00
1 ->	39	-0.00	0.00
1 ->	40	0.01	0.00
1 ->	41	0.00	0.00
1 ->	42	0.00	0.00
1 ->	43	-0.00	0.00
1 ->	44	0.05	0.01
1 ->	45	0.00	0.00
1 ->	46	-0.03	0.00

1 ->	47	0.00	0.00
1 ->	48	0.00	0.00
1 ->	49	0.00	0.00
1 ->	50	0.00	0.00
1 ->	51	0.00	0.00
1 ->	52	0.00	0.00
1 ->	53	0.01	0.00
1 ->	54	0.00	0.00
1 ->	55	0.00	0.00
1 ->	56	0.00	0.00
1 ->	57	0.00	0.00
1 ->	58	-0.01	0.00
1 ->	59	0.01	0.00
1 ->	60	0.00	0.00
1 ->	61	0.00	0.00
1 ->	62	-0.00	0.00
1 ->	63	-0.01	0.00
1 ->	64	-0.01	0.00
1 ->	65	-0.00	0.00
1 ->	66	-0.00	0.00
1 ->	67	0.00	0.00
1 ->	68	0.01	0.00
1 ->	69	0.00	0.00
1 ->	70	0.00	0.00
1 ->	71	-0.00	0.00
1 ->	72	0.02	0.00
1 ->	73	0.00	0.00
1 ->	74	-0.03	0.00
1 ->	75	-0.01	0.00
1 ->	76	0.02	0.00
1 ->	77	0.03	0.01
1 ->	78	0.02	0.00
1 ->	79	0.00	0.00
1 ->	80	0.00	0.00
1 ->	81	0.03	0.01
1 ->	82	0.00	0.00
1 ->	83	0.00	0.00
1 ->	84	-0.01	0.00
1 ->	85	-0.00	0.00
1 ->	86	-0.01	0.00
1 ->	87	-0.01	0.00
1 ->	88	-0.01	0.00
1 ->	89	-0.03	0.01
1 ->	90	-0.11	0.07
1 ->	91	-2.79	2.32
1 ->	92	-10.25	2.61
1 ->	93	-0.95	1.80
1 ->	94	-0.07	0.02
1 ->	95	-0.02	0.01
1 ->	96	-0.01	0.00

1	->	97	-0.02	0.00
1	->	98	-0.03	0.00
1	->	99	-0.03	0.00
1	->	100	-0.03	0.01
1	->	101	-0.05	0.01
1	->	102	-0.07	0.01
1	->	103	-0.06	0.02
1	->	104	-0.06	0.02
1	->	105	-0.04	0.01
1	->	106	-0.01	0.00
2	->	1	-0.91	0.35
2	->	2	1.38	0.64
2	->	3	-12.89	0.53
2	->	4	-0.40	0.23
2	->	5	-0.02	0.00
2	->	6	-0.00	0.00
2	->	7	-0.00	0.00
2	->	8	0.00	0.00
2	->	9	-0.00	0.00
2	->	10	-0.00	0.00
2	->	11	-0.00	0.00
2	->	12	-0.01	0.01
2	->	13	-1.53	0.83
2	->	14	-0.79	0.38
2	->	15	-0.67	0.41
2	->	16	-0.18	0.15
2	->	17	-0.05	0.03
2	->	18	-0.01	0.00
2	->	19	-0.00	0.00
2	->	20	-0.04	0.02
2	->	21	-0.00	0.00
2	->	22	-0.00	0.00
2	->	23	-0.00	0.00
2	->	24	-0.00	0.00
2	->	25	-0.00	0.00
2	->	26	-0.00	0.00
2	->	27	-0.00	0.00
2	->	28	-0.00	0.00
2	->	29	-0.00	0.00
2	->	30	-0.00	0.00
2	->	31	-0.00	0.00
2	->	32	-0.00	0.00
2	->	33	0.00	0.00
2	->	34	0.00	0.00
2	->	35	-0.00	0.00
2	->	36	0.00	0.00
2	->	37	0.00	0.00
2	->	38	-0.00	0.00
2	->	39	0.00	0.00
2	->	40	-0.00	0.00

2	->	41	0.00	0.00
2	->	42	0.00	0.00
2	->	43	0.00	0.00
2	->	44	0.00	0.00
2	->	45	0.00	0.00
2	->	46	-0.00	0.00
2	->	47	0.00	0.00
2	->	48	0.00	0.00
2	->	49	0.00	0.00
2	->	50	0.00	0.00
2	->	51	0.00	0.00
2	->	52	0.00	0.00
2	->	53	0.00	0.00
2	->	54	0.00	0.00
2	->	55	0.00	0.00
2	->	56	0.00	0.00
2	->	57	0.00	0.00
2	->	58	-0.00	0.00
2	->	59	0.00	0.00
2	->	60	0.00	0.00
2	->	61	0.00	0.00
2	->	62	0.00	0.00
2	->	63	-0.00	0.00
2	->	64	-0.00	0.00
2	->	65	0.00	0.00
2	->	66	-0.00	0.00
2	->	67	0.00	0.00
2	->	68	0.00	0.00
2	->	69	0.00	0.00
2	->	70	0.00	0.00
2	->	71	0.00	0.00
2	->	72	0.00	0.00
2	->	73	0.00	0.00
2	->	74	-0.00	0.00
2	->	75	-0.00	0.00
2	->	76	0.00	0.00
2	->	77	0.00	0.00
2	->	78	0.00	0.00
2	->	79	0.00	0.00
2	->	80	0.00	0.00
2	->	81	0.00	0.00
2	->	82	0.00	0.00
2	->	83	0.00	0.00
2	->	84	-0.00	0.00
2	->	85	-0.00	0.00
2	->	86	-0.00	0.00
2	->	87	-0.00	0.00
2	->	88	-0.00	0.00
2	->	89	-0.00	0.00
2	->	90	-0.00	0.00

2 ->	91	-0.01	0.01
2 ->	92	-0.04	0.03
2 ->	93	-0.02	0.01
2 ->	94	-0.01	0.00
2 ->	95	-0.00	0.00
2 ->	96	-0.00	0.00
2 ->	97	-0.00	0.00
2 ->	98	-0.00	0.00
2 ->	99	-0.00	0.00
2 ->	100	-0.00	0.00
2 ->	101	-0.00	0.00
2 ->	102	-0.00	0.00
2 ->	103	-0.00	0.00
2 ->	104	-0.00	0.00
2 ->	105	-0.00	0.00
2 ->	106	0.00	0.00
3 ->	1	-1.18	0.21
3 ->	2	-12.99	0.51
3 ->	3	19.13	1.04
3 ->	4	-9.66	0.45
3 ->	5	-0.51	0.06
3 ->	6	-0.03	0.00
3 ->	7	0.01	0.01
3 ->	8	-0.01	0.01
3 ->	9	-0.00	0.00
3 ->	10	-0.01	0.00
3 ->	11	-0.01	0.00
3 ->	12	-0.17	0.06
3 ->	13	-3.14	0.58
3 ->	14	-3.66	0.49
3 ->	15	-1.00	0.38
3 ->	16	-1.82	0.58
3 ->	17	-1.92	0.59
3 ->	18	-0.04	0.02
3 ->	19	-0.08	0.04
3 ->	20	-1.53	0.40
3 ->	21	-0.04	0.01
3 ->	22	-0.01	0.01
3 ->	23	-0.02	0.01
3 ->	24	-0.02	0.01
3 ->	25	-0.00	0.00
3 ->	26	-0.00	0.00
3 ->	27	-0.01	0.00
3 ->	28	-0.00	0.00
3 ->	29	0.00	0.00
3 ->	30	-0.00	0.00
3 ->	31	0.00	0.00
3 ->	32	0.00	0.00
3 ->	33	-0.00	0.00
3 ->	34	-0.00	0.00

3	->	35	-0.00	0.00
3	->	36	-0.00	0.00
3	->	37	0.00	0.00
3	->	38	-0.00	0.00
3	->	39	0.00	0.00
3	->	40	-0.00	0.00
3	->	41	0.00	0.00
3	->	42	0.00	0.00
3	->	43	-0.00	0.00
3	->	44	-0.00	0.00
3	->	45	-0.00	0.00
3	->	46	-0.00	0.00
3	->	47	-0.00	0.00
3	->	48	0.00	0.00
3	->	49	0.00	0.00
3	->	50	-0.00	0.00
3	->	51	0.00	0.00
3	->	52	0.00	0.00
3	->	53	-0.00	0.00
3	->	54	-0.00	0.00
3	->	55	0.00	0.00
3	->	56	0.00	0.00
3	->	57	0.00	0.00
3	->	58	0.00	0.00
3	->	59	-0.00	0.00
3	->	60	0.00	0.00
3	->	61	-0.00	0.00
3	->	62	0.00	0.00
3	->	63	0.00	0.00
3	->	64	0.00	0.00
3	->	65	0.00	0.00
3	->	66	0.00	0.00
3	->	67	0.00	0.00
3	->	68	-0.00	0.00
3	->	69	-0.00	0.00
3	->	70	-0.00	0.00
3	->	71	0.00	0.00
3	->	72	-0.00	0.00
3	->	73	0.00	0.00
3	->	74	0.00	0.00
3	->	75	0.00	0.00
3	->	76	-0.00	0.00
3	->	77	-0.00	0.00
3	->	78	-0.00	0.00
3	->	79	0.00	0.00
3	->	80	0.00	0.00
3	->	81	-0.00	0.00
3	->	82	0.00	0.00
3	->	83	0.00	0.00
3	->	84	0.00	0.00

3	->	85	0.00	0.00
3	->	86	0.00	0.00
3	->	87	0.00	0.00
3	->	88	0.00	0.00
3	->	89	-0.00	0.00
3	->	90	-0.00	0.00
3	->	91	-0.01	0.02
3	->	92	-0.00	0.04
3	->	93	-0.02	0.01
3	->	94	-0.02	0.01
3	->	95	-0.01	0.00
3	->	96	-0.01	0.01
3	->	97	-0.01	0.00
3	->	98	-0.01	0.00
3	->	99	-0.00	0.00
3	->	100	-0.00	0.00
3	->	101	-0.00	0.00
3	->	102	-0.00	0.00
3	->	103	-0.00	0.00
3	->	104	0.00	0.00
3	->	105	0.00	0.00
3	->	106	0.00	0.00
4	->	1	-0.15	0.06
4	->	2	-0.42	0.24
4	->	3	-9.63	0.45
4	->	4	22.15	0.92
4	->	5	-12.80	0.49
4	->	6	-0.31	0.06
4	->	7	0.08	0.06
4	->	8	-0.06	0.06
4	->	9	-0.01	0.00
4	->	10	-0.10	0.03
4	->	11	-0.03	0.01
4	->	12	-0.13	0.10
4	->	13	-1.98	0.42
4	->	14	-0.75	0.26
4	->	15	-0.00	0.01
4	->	16	-0.00	0.01
4	->	17	-0.02	0.01
4	->	18	-0.00	0.00
4	->	19	-0.00	0.02
4	->	20	-1.19	0.27
4	->	21	-0.02	0.01
4	->	22	0.01	0.01
4	->	23	-0.09	0.03
4	->	24	-0.08	0.04
4	->	25	-0.00	0.00
4	->	26	-0.00	0.00
4	->	27	-0.07	0.02
4	->	28	-0.00	0.00

4 ->	29	0.00	0.00
4 ->	30	-0.00	0.00
4 ->	31	0.00	0.00
4 ->	32	-0.00	0.00
4 ->	33	0.00	0.00
4 ->	34	-0.00	0.00
4 ->	35	-0.00	0.00
4 ->	36	-0.00	0.00
4 ->	37	0.00	0.00
4 ->	38	-0.00	0.00
4 ->	39	0.00	0.00
4 ->	40	0.00	0.00
4 ->	41	-0.00	0.00
4 ->	42	0.00	0.00
4 ->	43	0.00	0.00
4 ->	44	-0.00	0.00
4 ->	45	0.00	0.00
4 ->	46	-0.00	0.00
4 ->	47	0.00	0.00
4 ->	48	0.00	0.00
4 ->	49	0.00	0.00
4 ->	50	-0.00	0.00
4 ->	51	0.00	0.00
4 ->	52	-0.00	0.00
4 ->	53	0.00	0.00
4 ->	54	-0.00	0.00
4 ->	55	0.00	0.00
4 ->	56	0.00	0.00
4 ->	57	0.00	0.00
4 ->	58	-0.00	0.00
4 ->	59	0.00	0.00
4 ->	60	0.00	0.00
4 ->	61	0.00	0.00
4 ->	62	0.00	0.00
4 ->	63	0.00	0.00
4 ->	64	0.00	0.00
4 ->	65	0.00	0.00
4 ->	66	-0.00	0.00
4 ->	67	0.00	0.00
4 ->	68	0.00	0.00
4 ->	69	0.00	0.00
4 ->	70	0.00	0.00
4 ->	71	0.00	0.00
4 ->	72	0.00	0.00
4 ->	73	0.00	0.00
4 ->	74	-0.00	0.00
4 ->	75	-0.00	0.00
4 ->	76	0.00	0.00
4 ->	77	0.00	0.00
4 ->	78	0.00	0.00

4	->	79	0.00	0.00
4	->	80	0.00	0.00
4	->	81	0.00	0.00
4	->	82	0.00	0.00
4	->	83	0.00	0.00
4	->	84	-0.00	0.00
4	->	85	0.00	0.00
4	->	86	-0.00	0.00
4	->	87	-0.00	0.00
4	->	88	-0.00	0.00
4	->	89	-0.00	0.00
4	->	90	-0.01	0.01
4	->	91	-0.03	0.01
4	->	92	0.00	0.01
4	->	93	-0.00	0.00
4	->	94	-0.00	0.00
4	->	95	-0.00	0.00
4	->	96	-0.00	0.00
4	->	97	-0.00	0.00
4	->	98	-0.00	0.00
4	->	99	-0.00	0.00
4	->	100	0.00	0.00
4	->	101	-0.00	0.00
4	->	102	-0.00	0.00
4	->	103	-0.00	0.00
4	->	104	-0.00	0.00
4	->	105	-0.00	0.00
4	->	106	-0.00	0.00
5	->	1	-0.12	0.01
5	->	2	-0.02	0.00
5	->	3	-0.51	0.06
5	->	4	-12.86	0.49
5	->	5	-28.88	4.00
5	->	6	-1.80	0.45
5	->	7	-6.16	2.45
5	->	8	0.16	0.14
5	->	9	-0.03	0.10
5	->	10	1.98	0.71
5	->	11	0.09	0.02
5	->	12	-0.14	0.05
5	->	13	-0.36	0.10
5	->	14	-0.51	0.21
5	->	15	0.01	0.01
5	->	16	0.04	0.01
5	->	17	-0.01	0.01
5	->	18	-0.00	0.00
5	->	19	-0.10	0.02
5	->	20	-1.08	0.20
5	->	21	0.00	0.01
5	->	22	-0.14	0.04

5	->	23	1.26	0.35
5	->	24	-0.77	0.25
5	->	25	-0.06	0.01
5	->	26	-0.08	0.03
5	->	27	2.52	1.07
5	->	28	-0.02	0.01
5	->	29	-0.01	0.00
5	->	30	-0.01	0.00
5	->	31	-0.03	0.00
5	->	32	-0.00	0.00
5	->	33	0.00	0.00
5	->	34	-0.00	0.00
5	->	35	0.01	0.00
5	->	36	-0.00	0.00
5	->	37	-0.00	0.00
5	->	38	0.01	0.00
5	->	39	0.00	0.00
5	->	40	-0.01	0.00
5	->	41	-0.00	0.00
5	->	42	-0.00	0.00
5	->	43	-0.00	0.00
5	->	44	-0.03	0.00
5	->	45	-0.00	0.00
5	->	46	0.02	0.00
5	->	47	-0.00	0.00
5	->	48	-0.00	0.00
5	->	49	-0.00	0.00
5	->	50	-0.00	0.00
5	->	51	-0.00	0.00
5	->	52	-0.00	0.00
5	->	53	-0.01	0.00
5	->	54	-0.00	0.00
5	->	55	0.00	0.00
5	->	56	-0.00	0.00
5	->	57	-0.00	0.00
5	->	58	0.00	0.00
5	->	59	-0.00	0.00
5	->	60	-0.00	0.00
5	->	61	-0.00	0.00
5	->	62	0.00	0.00
5	->	63	0.00	0.00
5	->	64	0.00	0.00
5	->	65	0.00	0.00
5	->	66	0.00	0.00
5	->	67	0.00	0.00
5	->	68	-0.00	0.00
5	->	69	-0.00	0.00
5	->	70	-0.00	0.00
5	->	71	0.00	0.00
5	->	72	-0.01	0.00

5	->	73	-0.00	0.00
5	->	74	0.01	0.00
5	->	75	0.00	0.00
5	->	76	-0.00	0.00
5	->	77	-0.01	0.00
5	->	78	-0.01	0.00
5	->	79	-0.00	0.00
5	->	80	-0.00	0.00
5	->	81	-0.00	0.00
5	->	82	-0.00	0.00
5	->	83	-0.00	0.00
5	->	84	0.00	0.00
5	->	85	0.00	0.00
5	->	86	0.00	0.00
5	->	87	0.01	0.00
5	->	88	0.01	0.00
5	->	89	0.02	0.00
5	->	90	0.09	0.03
5	->	91	0.16	0.05
5	->	92	0.06	0.01
5	->	93	0.02	0.00
5	->	94	0.01	0.00
5	->	95	0.00	0.00
5	->	96	0.00	0.00
5	->	97	0.01	0.00
5	->	98	0.02	0.00
5	->	99	0.01	0.00
5	->	100	0.01	0.00
5	->	101	0.01	0.00
5	->	102	0.01	0.00
5	->	103	0.01	0.00
5	->	104	0.01	0.00
5	->	105	0.01	0.00
5	->	106	0.01	0.00
6	->	1	-0.00	0.00
6	->	2	-0.00	0.00
6	->	3	-0.03	0.00
6	->	4	-0.32	0.06
6	->	5	-1.91	0.46
6	->	6	0.80	0.59
6	->	7	-13.09	0.49
6	->	8	-0.62	0.29
6	->	9	-0.04	0.01
6	->	10	-0.06	0.03
6	->	11	-0.00	0.00
6	->	12	-0.01	0.00
6	->	13	-0.02	0.01
6	->	14	-0.02	0.01
6	->	15	-0.00	0.00
6	->	16	-0.00	0.00

6	->	17	-0.01	0.00
6	->	18	-0.00	0.00
6	->	19	-0.01	0.00
6	->	20	-0.19	0.11
6	->	21	-0.02	0.01
6	->	22	-0.01	0.00
6	->	23	-0.08	0.03
6	->	24	-1.19	0.34
6	->	25	-0.02	0.01
6	->	26	-0.01	0.00
6	->	27	-0.37	0.18
6	->	28	-0.07	0.09
6	->	29	-0.00	0.00
6	->	30	-0.00	0.00
6	->	31	0.00	0.00
6	->	32	-0.00	0.00
6	->	33	-0.00	0.00
6	->	34	0.00	0.00
6	->	35	-0.00	0.00
6	->	36	0.00	0.00
6	->	37	0.00	0.00
6	->	38	-0.00	0.00
6	->	39	0.00	0.00
6	->	40	0.00	0.00
6	->	41	0.00	0.00
6	->	42	-0.00	0.00
6	->	43	-0.00	0.00
6	->	44	0.00	0.00
6	->	45	0.00	0.00
6	->	46	-0.00	0.00
6	->	47	0.00	0.00
6	->	48	0.00	0.00
6	->	49	0.00	0.00
6	->	50	0.00	0.00
6	->	51	0.00	0.00
6	->	52	0.00	0.00
6	->	53	0.00	0.00
6	->	54	0.00	0.00
6	->	55	0.00	0.00
6	->	56	0.00	0.00
6	->	57	0.00	0.00
6	->	58	-0.00	0.00
6	->	59	0.00	0.00
6	->	60	0.00	0.00
6	->	61	0.00	0.00
6	->	62	0.00	0.00
6	->	63	-0.00	0.00
6	->	64	0.00	0.00
6	->	65	0.00	0.00
6	->	66	-0.00	0.00

6 ->	67	0.00	0.00
6 ->	68	0.00	0.00
6 ->	69	0.00	0.00
6 ->	70	0.00	0.00
6 ->	71	0.00	0.00
6 ->	72	0.00	0.00
6 ->	73	0.00	0.00
6 ->	74	-0.00	0.00
6 ->	75	-0.00	0.00
6 ->	76	0.00	0.00
6 ->	77	0.00	0.00
6 ->	78	0.00	0.00
6 ->	79	0.00	0.00
6 ->	80	0.00	0.00
6 ->	81	0.00	0.00
6 ->	82	0.00	0.00
6 ->	83	0.00	0.00
6 ->	84	-0.00	0.00
6 ->	85	-0.00	0.00
6 ->	86	-0.00	0.00
6 ->	87	-0.00	0.00
6 ->	88	-0.00	0.00
6 ->	89	-0.00	0.00
6 ->	90	-0.00	0.00
6 ->	91	-0.01	0.00
6 ->	92	-0.00	0.00
6 ->	93	-0.00	0.00
6 ->	94	-0.00	0.00
6 ->	95	-0.00	0.00
6 ->	96	-0.00	0.00
6 ->	97	-0.00	0.00
6 ->	98	-0.00	0.00
6 ->	99	-0.00	0.00
6 ->	100	-0.00	0.00
6 ->	101	-0.00	0.00
6 ->	102	-0.00	0.00
6 ->	103	-0.00	0.00
6 ->	104	-0.00	0.00
6 ->	105	-0.00	0.00
6 ->	106	-0.00	0.00
7 ->	1	-0.00	0.00
7 ->	2	-0.00	0.00
7 ->	3	0.01	0.01
7 ->	4	0.08	0.06
7 ->	5	-6.25	2.45
7 ->	6	-13.22	0.49
7 ->	7	26.20	1.66
7 ->	8	-11.39	0.36
7 ->	9	-1.34	0.43
7 ->	10	-1.64	0.99

7 ->	11	-0.04	0.02
7 ->	12	-0.01	0.01
7 ->	13	-0.01	0.01
7 ->	14	-0.04	0.02
7 ->	15	-0.00	0.00
7 ->	16	-0.00	0.00
7 ->	17	-0.00	0.00
7 ->	18	-0.00	0.00
7 ->	19	0.01	0.01
7 ->	20	-0.02	0.01
7 ->	21	-0.01	0.01
7 ->	22	0.01	0.01
7 ->	23	-0.37	0.17
7 ->	24	-0.10	0.05
7 ->	25	-0.00	0.01
7 ->	26	0.01	0.01
7 ->	27	-2.49	0.99
7 ->	28	-0.05	0.03
7 ->	29	-0.00	0.00
7 ->	30	-0.00	0.00
7 ->	31	-0.00	0.00
7 ->	32	-0.00	0.00
7 ->	33	-0.00	0.00
7 ->	34	0.00	0.00
7 ->	35	0.00	0.00
7 ->	36	0.00	0.00
7 ->	37	0.00	0.00
7 ->	38	0.00	0.00
7 ->	39	0.00	0.00
7 ->	40	-0.00	0.00
7 ->	41	0.00	0.00
7 ->	42	-0.00	0.00
7 ->	43	-0.00	0.00
7 ->	44	-0.00	0.00
7 ->	45	0.00	0.00
7 ->	46	-0.00	0.00
7 ->	47	-0.00	0.00
7 ->	48	0.00	0.00
7 ->	49	0.00	0.00
7 ->	50	0.00	0.00
7 ->	51	0.00	0.00
7 ->	52	0.00	0.00
7 ->	53	0.00	0.00
7 ->	54	0.00	0.00
7 ->	55	0.00	0.00
7 ->	56	0.00	0.00
7 ->	57	0.00	0.00
7 ->	58	-0.00	0.00
7 ->	59	-0.00	0.00
7 ->	60	0.00	0.00

7	->	61	0.00	0.00
7	->	62	0.00	0.00
7	->	63	0.00	0.00
7	->	64	0.00	0.00
7	->	65	0.00	0.00
7	->	66	0.00	0.00
7	->	67	0.00	0.00
7	->	68	0.00	0.00
7	->	69	0.00	0.00
7	->	70	0.00	0.00
7	->	71	0.00	0.00
7	->	72	0.00	0.00
7	->	73	0.00	0.00
7	->	74	0.00	0.00
7	->	75	0.00	0.00
7	->	76	0.00	0.00
7	->	77	-0.00	0.00
7	->	78	-0.00	0.00
7	->	79	0.00	0.00
7	->	80	0.00	0.00
7	->	81	-0.00	0.00
7	->	82	0.00	0.00
7	->	83	0.00	0.00
7	->	84	0.00	0.00
7	->	85	0.00	0.00
7	->	86	-0.00	0.00
7	->	87	0.00	0.00
7	->	88	-0.00	0.00
7	->	89	-0.00	0.00
7	->	90	-0.02	0.01
7	->	91	-0.02	0.01
7	->	92	-0.00	0.00
7	->	93	-0.00	0.00
7	->	94	-0.00	0.00
7	->	95	-0.00	0.00
7	->	96	-0.00	0.00
7	->	97	-0.00	0.00
7	->	98	-0.00	0.00
7	->	99	-0.00	0.00
7	->	100	-0.00	0.00
7	->	101	0.00	0.00
7	->	102	-0.00	0.00
7	->	103	0.00	0.00
7	->	104	-0.00	0.00
7	->	105	-0.00	0.00
7	->	106	0.00	0.00
8	->	1	-0.05	0.02
8	->	2	0.00	0.00
8	->	3	-0.01	0.01
8	->	4	-0.06	0.06

8	->	5	0.15	0.15
8	->	6	-0.65	0.31
8	->	7	-11.42	0.37
8	->	8	-70.74	2.31
8	->	9	-18.55	0.44
8	->	10	-0.60	0.19
8	->	11	0.01	0.02
8	->	12	-0.05	0.01
8	->	13	-0.24	0.88
8	->	14	-0.01	0.00
8	->	15	-0.00	0.00
8	->	16	0.01	0.00
8	->	17	0.00	0.00
8	->	18	0.00	0.00
8	->	19	-0.00	0.00
8	->	20	-0.01	0.00
8	->	21	0.00	0.00
8	->	22	-0.01	0.00
8	->	23	0.00	0.01
8	->	24	-0.01	0.00
8	->	25	-0.01	0.00
8	->	26	-0.00	0.00
8	->	27	0.00	0.04
8	->	28	-0.00	0.00
8	->	29	-0.00	0.00
8	->	30	-0.00	0.00
8	->	31	-0.01	0.00
8	->	32	-0.00	0.00
8	->	33	0.00	0.00
8	->	34	-0.00	0.00
8	->	35	0.00	0.00
8	->	36	-0.00	0.00
8	->	37	-0.00	0.00
8	->	38	0.00	0.00
8	->	39	0.00	0.00
8	->	40	-0.01	0.00
8	->	41	-0.00	0.00
8	->	42	-0.00	0.00
8	->	43	-0.00	0.00
8	->	44	-0.01	0.00
8	->	45	-0.00	0.00
8	->	46	0.01	0.00
8	->	47	-0.00	0.00
8	->	48	-0.00	0.00
8	->	49	-0.00	0.00
8	->	50	-0.00	0.00
8	->	51	-0.00	0.00
8	->	52	-0.00	0.00
8	->	53	-0.00	0.00
8	->	54	-0.00	0.00

8	->	55	0.00	0.00
8	->	56	-0.00	0.00
8	->	57	-0.00	0.00
8	->	58	0.00	0.00
8	->	59	-0.00	0.00
8	->	60	-0.00	0.00
8	->	61	-0.00	0.00
8	->	62	0.00	0.00
8	->	63	0.00	0.00
8	->	64	0.00	0.00
8	->	65	0.00	0.00
8	->	66	0.00	0.00
8	->	67	0.00	0.00
8	->	68	-0.00	0.00
8	->	69	-0.00	0.00
8	->	70	-0.00	0.00
8	->	71	-0.00	0.00
8	->	72	-0.00	0.00
8	->	73	-0.00	0.00
8	->	74	0.00	0.00
8	->	75	0.00	0.00
8	->	76	-0.00	0.00
8	->	77	-0.00	0.00
8	->	78	-0.00	0.00
8	->	79	-0.00	0.00
8	->	80	-0.00	0.00
8	->	81	-0.00	0.00
8	->	82	-0.00	0.00
8	->	83	-0.00	0.00
8	->	84	0.00	0.00
8	->	85	0.00	0.00
8	->	86	0.00	0.00
8	->	87	0.00	0.00
8	->	88	0.00	0.00
8	->	89	0.01	0.00
8	->	90	0.02	0.00
8	->	91	0.02	0.00
8	->	92	0.02	0.00
8	->	93	0.01	0.00
8	->	94	0.00	0.00
8	->	95	0.00	0.00
8	->	96	0.00	0.00
8	->	97	0.01	0.00
8	->	98	0.01	0.00
8	->	99	0.00	0.00
8	->	100	0.00	0.00
8	->	101	0.00	0.00
8	->	102	0.00	0.00
8	->	103	0.00	0.00
8	->	104	0.01	0.00

8	->	105	0.01	0.00
8	->	106	0.00	0.00
9	->	1	-0.00	0.00
9	->	2	-0.00	0.00
9	->	3	-0.00	0.00
9	->	4	-0.01	0.00
9	->	5	-0.03	0.10
9	->	6	-0.04	0.01
9	->	7	-1.35	0.43
9	->	8	-18.64	0.44
9	->	9	14.67	1.85
9	->	10	-9.74	0.61
9	->	11	-0.34	0.08
9	->	12	-0.04	0.01
9	->	13	-0.01	0.01
9	->	14	-0.01	0.00
9	->	15	-0.00	0.00
9	->	16	-0.00	0.00
9	->	17	-0.00	0.00
9	->	18	-0.00	0.00
9	->	19	-0.00	0.00
9	->	20	-0.00	0.00
9	->	21	-0.00	0.00
9	->	22	-0.00	0.00
9	->	23	-0.02	0.03
9	->	24	-0.00	0.00
9	->	25	-0.00	0.00
9	->	26	-0.01	0.01
9	->	27	-0.14	0.13
9	->	28	-0.00	0.00
9	->	29	-0.00	0.00
9	->	30	-0.00	0.00
9	->	31	0.00	0.00
9	->	32	0.00	0.00
9	->	33	-0.00	0.00
9	->	34	0.00	0.00
9	->	35	-0.00	0.00
9	->	36	0.00	0.00
9	->	37	0.00	0.00
9	->	38	-0.00	0.00
9	->	39	0.00	0.00
9	->	40	0.00	0.00
9	->	41	-0.00	0.00
9	->	42	-0.00	0.00
9	->	43	-0.00	0.00
9	->	44	-0.00	0.00
9	->	45	0.00	0.00
9	->	46	-0.00	0.00
9	->	47	-0.00	0.00
9	->	48	0.00	0.00

9	->	49	0.00	0.00
9	->	50	0.00	0.00
9	->	51	-0.00	0.00
9	->	52	0.00	0.00
9	->	53	0.00	0.00
9	->	54	0.00	0.00
9	->	55	0.00	0.00
9	->	56	0.00	0.00
9	->	57	0.00	0.00
9	->	58	0.00	0.00
9	->	59	0.00	0.00
9	->	60	0.00	0.00
9	->	61	0.00	0.00
9	->	62	0.00	0.00
9	->	63	0.00	0.00
9	->	64	0.00	0.00
9	->	65	0.00	0.00
9	->	66	0.00	0.00
9	->	67	0.00	0.00
9	->	68	-0.00	0.00
9	->	69	0.00	0.00
9	->	70	-0.00	0.00
9	->	71	-0.00	0.00
9	->	72	-0.00	0.00
9	->	73	0.00	0.00
9	->	74	0.00	0.00
9	->	75	0.00	0.00
9	->	76	-0.00	0.00
9	->	77	-0.00	0.00
9	->	78	-0.00	0.00
9	->	79	0.00	0.00
9	->	80	0.00	0.00
9	->	81	-0.00	0.00
9	->	82	0.00	0.00
9	->	83	0.00	0.00
9	->	84	0.00	0.00
9	->	85	0.00	0.00
9	->	86	0.00	0.00
9	->	87	0.00	0.00
9	->	88	-0.00	0.00
9	->	89	-0.00	0.00
9	->	90	-0.01	0.01
9	->	91	-0.01	0.00
9	->	92	-0.00	0.00
9	->	93	0.00	0.00
9	->	94	0.00	0.00
9	->	95	0.00	0.00
9	->	96	-0.00	0.00
9	->	97	-0.00	0.00
9	->	98	-0.00	0.00

9	->	99	-0.00	0.00
9	->	100	0.00	0.00
9	->	101	0.00	0.00
9	->	102	0.00	0.00
9	->	103	0.00	0.00
9	->	104	0.00	0.00
9	->	105	0.00	0.00
9	->	106	0.00	0.00
10	->	1	-0.09	0.01
10	->	2	-0.00	0.00
10	->	3	-0.01	0.00
10	->	4	-0.10	0.03
10	->	5	1.96	0.71
10	->	6	-0.06	0.03
10	->	7	-1.63	0.98
10	->	8	-0.61	0.19
10	->	9	-9.82	0.61
10	->	10	-37.93	2.58
10	->	11	-14.23	0.30
10	->	12	-1.06	0.22
10	->	13	-0.19	0.11
10	->	14	-0.04	0.04
10	->	15	-0.00	0.00
10	->	16	0.03	0.00
10	->	17	0.00	0.00
10	->	18	0.00	0.00
10	->	19	-0.02	0.01
10	->	20	-0.01	0.01
10	->	21	0.00	0.00
10	->	22	-0.07	0.01
10	->	23	1.53	0.31
10	->	24	0.03	0.01
10	->	25	-0.04	0.00
10	->	26	-0.05	0.01
10	->	27	2.26	0.87
10	->	28	-0.01	0.00
10	->	29	-0.00	0.00
10	->	30	-0.00	0.00
10	->	31	-0.03	0.00
10	->	32	-0.00	0.00
10	->	33	0.00	0.00
10	->	34	-0.00	0.00
10	->	35	0.01	0.00
10	->	36	-0.00	0.00
10	->	37	-0.00	0.00
10	->	38	0.01	0.00
10	->	39	0.00	0.00
10	->	40	-0.02	0.01
10	->	41	0.00	0.00
10	->	42	-0.00	0.00

10 ->	43	-0.00	0.00
10 ->	44	-0.03	0.00
10 ->	45	-0.00	0.00
10 ->	46	0.02	0.00
10 ->	47	-0.00	0.00
10 ->	48	-0.00	0.00
10 ->	49	-0.00	0.00
10 ->	50	-0.00	0.00
10 ->	51	-0.00	0.00
10 ->	52	-0.00	0.00
10 ->	53	-0.01	0.00
10 ->	54	-0.00	0.00
10 ->	55	0.00	0.00
10 ->	56	-0.00	0.00
10 ->	57	-0.00	0.00
10 ->	58	0.00	0.00
10 ->	59	-0.00	0.00
10 ->	60	-0.00	0.00
10 ->	61	-0.00	0.00
10 ->	62	0.00	0.00
10 ->	63	0.00	0.00
10 ->	64	0.00	0.00
10 ->	65	0.00	0.00
10 ->	66	0.00	0.00
10 ->	67	0.00	0.00
10 ->	68	-0.00	0.00
10 ->	69	-0.00	0.00
10 ->	70	-0.00	0.00
10 ->	71	0.00	0.00
10 ->	72	-0.01	0.00
10 ->	73	-0.00	0.00
10 ->	74	0.01	0.00
10 ->	75	0.01	0.00
10 ->	76	-0.00	0.00
10 ->	77	-0.01	0.00
10 ->	78	-0.01	0.00
10 ->	79	-0.00	0.00
10 ->	80	-0.00	0.00
10 ->	81	-0.01	0.00
10 ->	82	-0.00	0.00
10 ->	83	-0.00	0.00
10 ->	84	0.00	0.00
10 ->	85	0.00	0.00
10 ->	86	0.00	0.00
10 ->	87	0.01	0.00
10 ->	88	0.01	0.00
10 ->	89	0.04	0.01
10 ->	90	0.16	0.07
10 ->	91	0.22	0.09
10 ->	92	0.04	0.01

10	->	93	0.02	0.00
10	->	94	0.01	0.00
10	->	95	0.00	0.00
10	->	96	0.00	0.00
10	->	97	0.01	0.00
10	->	98	0.01	0.00
10	->	99	0.01	0.00
10	->	100	0.01	0.00
10	->	101	0.01	0.00
10	->	102	0.01	0.00
10	->	103	0.01	0.00
10	->	104	0.02	0.00
10	->	105	0.02	0.01
10	->	106	0.01	0.00
11	->	1	-0.14	0.02
11	->	2	-0.00	0.00
11	->	3	-0.01	0.00
11	->	4	-0.03	0.01
11	->	5	0.09	0.02
11	->	6	-0.00	0.00
11	->	7	-0.04	0.02
11	->	8	0.01	0.02
11	->	9	-0.36	0.08
11	->	10	-14.35	0.30
11	->	11	-35.18	2.96
11	->	12	-10.06	2.65
11	->	13	-0.64	0.46
11	->	14	-0.03	0.01
11	->	15	-0.01	0.00
11	->	16	0.02	0.00
11	->	17	-0.00	0.00
11	->	18	0.00	0.00
11	->	19	-0.00	0.00
11	->	20	-0.00	0.00
11	->	21	-0.00	0.00
11	->	22	-0.03	0.01
11	->	23	0.00	0.01
11	->	24	-0.00	0.00
11	->	25	-0.01	0.00
11	->	26	-0.00	0.00
11	->	27	0.02	0.01
11	->	28	-0.00	0.00
11	->	29	-0.00	0.00
11	->	30	-0.00	0.00
11	->	31	-0.01	0.00
11	->	32	-0.00	0.00
11	->	33	0.00	0.00
11	->	34	-0.00	0.00
11	->	35	0.00	0.00
11	->	36	-0.00	0.00

11	->	37	-0.00	0.00
11	->	38	0.00	0.00
11	->	39	0.00	0.00
11	->	40	-0.01	0.00
11	->	41	0.00	0.00
11	->	42	-0.00	0.00
11	->	43	-0.00	0.00
11	->	44	-0.02	0.01
11	->	45	-0.00	0.00
11	->	46	0.01	0.00
11	->	47	-0.00	0.00
11	->	48	-0.00	0.00
11	->	49	-0.00	0.00
11	->	50	-0.00	0.00
11	->	51	-0.00	0.00
11	->	52	-0.00	0.00
11	->	53	-0.00	0.00
11	->	54	-0.00	0.00
11	->	55	0.00	0.00
11	->	56	-0.00	0.00
11	->	57	-0.00	0.00
11	->	58	0.00	0.00
11	->	59	-0.00	0.00
11	->	60	-0.00	0.00
11	->	61	-0.00	0.00
11	->	62	0.00	0.00
11	->	63	0.00	0.00
11	->	64	0.00	0.00
11	->	65	0.00	0.00
11	->	66	0.00	0.00
11	->	67	-0.00	0.00
11	->	68	-0.00	0.00
11	->	69	-0.00	0.00
11	->	70	-0.00	0.00
11	->	71	-0.00	0.00
11	->	72	-0.01	0.00
11	->	73	-0.00	0.00
11	->	74	0.01	0.00
11	->	75	0.00	0.00
11	->	76	-0.00	0.00
11	->	77	-0.01	0.00
11	->	78	-0.01	0.00
11	->	79	-0.00	0.00
11	->	80	-0.00	0.00
11	->	81	-0.01	0.00
11	->	82	-0.00	0.00
11	->	83	-0.00	0.00
11	->	84	0.00	0.00
11	->	85	0.00	0.00
11	->	86	0.00	0.00

11	->	87	0.01	0.00
11	->	88	0.01	0.00
11	->	89	0.02	0.01
11	->	90	0.03	0.04
11	->	91	0.01	0.02
11	->	92	0.03	0.00
11	->	93	0.02	0.00
11	->	94	0.01	0.00
11	->	95	0.00	0.00
11	->	96	0.00	0.00
11	->	97	0.01	0.00
11	->	98	0.01	0.00
11	->	99	0.01	0.00
11	->	100	0.01	0.00
11	->	101	0.01	0.00
11	->	102	0.01	0.00
11	->	103	0.02	0.01
11	->	104	0.03	0.01
11	->	105	0.03	0.02
11	->	106	0.01	0.00
12	->	1	0.15	0.49
12	->	2	-0.01	0.01
12	->	3	-0.17	0.05
12	->	4	-0.12	0.09
12	->	5	-0.13	0.05
12	->	6	-0.01	0.00
12	->	7	-0.01	0.01
12	->	8	-0.05	0.01
12	->	9	-0.04	0.01
12	->	10	-1.06	0.22
12	->	11	-10.23	2.71
12	->	12	-110.55	3.58
12	->	13	-9.28	0.47
12	->	14	-1.00	0.28
12	->	15	-0.07	0.03
12	->	16	-0.05	0.01
12	->	17	-0.01	0.00
12	->	18	-0.00	0.00
12	->	19	0.04	0.03
12	->	20	-0.03	0.01
12	->	21	-0.00	0.00
12	->	22	0.07	0.03
12	->	23	-0.09	0.09
12	->	24	-0.01	0.01
12	->	25	0.02	0.01
12	->	26	-0.00	0.00
12	->	27	-0.05	0.04
12	->	28	0.00	0.00
12	->	29	0.00	0.00
12	->	30	-0.00	0.00

12	->	31	0.02	0.01
12	->	32	0.00	0.00
12	->	33	-0.00	0.00
12	->	34	0.00	0.00
12	->	35	-0.01	0.00
12	->	36	0.00	0.00
12	->	37	0.00	0.00
12	->	38	-0.01	0.00
12	->	39	-0.00	0.00
12	->	40	0.02	0.01
12	->	41	-0.00	0.00
12	->	42	0.00	0.00
12	->	43	-0.00	0.00
12	->	44	0.05	0.02
12	->	45	-0.00	0.00
12	->	46	-0.03	0.01
12	->	47	0.00	0.00
12	->	48	0.00	0.00
12	->	49	0.00	0.00
12	->	50	0.00	0.00
12	->	51	0.00	0.00
12	->	52	0.00	0.00
12	->	53	0.01	0.00
12	->	54	0.00	0.00
12	->	55	0.00	0.00
12	->	56	0.00	0.00
12	->	57	0.00	0.00
12	->	58	-0.00	0.00
12	->	59	0.01	0.00
12	->	60	0.00	0.00
12	->	61	0.00	0.00
12	->	62	-0.00	0.00
12	->	63	-0.00	0.00
12	->	64	-0.00	0.00
12	->	65	-0.00	0.00
12	->	66	-0.00	0.00
12	->	67	0.00	0.00
12	->	68	0.01	0.00
12	->	69	0.00	0.00
12	->	70	0.00	0.00
12	->	71	0.00	0.00
12	->	72	0.02	0.01
12	->	73	0.00	0.00
12	->	74	-0.02	0.01
12	->	75	-0.01	0.00
12	->	76	0.01	0.00
12	->	77	0.02	0.00
12	->	78	0.02	0.01
12	->	79	0.00	0.00
12	->	80	0.00	0.00

12	->	81	0.02	0.01
12	->	82	0.00	0.00
12	->	83	0.00	0.00
12	->	84	-0.00	0.00
12	->	85	-0.00	0.00
12	->	86	-0.01	0.00
12	->	87	-0.01	0.01
12	->	88	-0.02	0.01
12	->	89	-0.10	0.09
12	->	90	-3.07	2.82
12	->	91	-4.66	3.31
12	->	92	-0.54	0.63
12	->	93	-0.05	0.01
12	->	94	-0.02	0.00
12	->	95	-0.01	0.00
12	->	96	-0.00	0.00
12	->	97	-0.01	0.00
12	->	98	-0.01	0.00
12	->	99	-0.01	0.00
12	->	100	-0.01	0.00
12	->	101	-0.02	0.01
12	->	102	-0.05	0.02
12	->	103	-0.07	0.03
12	->	104	-0.12	0.07
12	->	105	-0.10	0.10
12	->	106	-0.02	0.01
13	->	1	-4.35	1.92
13	->	2	-1.56	0.84
13	->	3	-3.15	0.58
13	->	4	-2.00	0.42
13	->	5	-0.36	0.10
13	->	6	-0.02	0.01
13	->	7	-0.01	0.01
13	->	8	-0.24	0.89
13	->	9	-0.01	0.01
13	->	10	-0.20	0.11
13	->	11	-0.66	0.48
13	->	12	-9.38	0.46
13	->	13	-107.74	2.59
13	->	14	-12.28	0.39
13	->	15	-0.53	0.13
13	->	16	-0.20	0.04
13	->	17	-0.04	0.01
13	->	18	0.01	0.01
13	->	19	0.16	0.05
13	->	20	-0.07	0.02
13	->	21	-0.01	0.01
13	->	22	0.05	0.01
13	->	23	-0.05	0.01
13	->	24	-0.01	0.00

13	->	25	0.01	0.00
13	->	26	0.00	0.00
13	->	27	-0.02	0.01
13	->	28	0.00	0.00
13	->	29	0.00	0.00
13	->	30	0.00	0.00
13	->	31	0.01	0.00
13	->	32	0.00	0.00
13	->	33	-0.00	0.00
13	->	34	0.00	0.00
13	->	35	-0.00	0.00
13	->	36	0.00	0.00
13	->	37	0.00	0.00
13	->	38	-0.00	0.00
13	->	39	-0.00	0.00
13	->	40	0.01	0.00
13	->	41	0.00	0.00
13	->	42	0.00	0.00
13	->	43	-0.00	0.00
13	->	44	0.02	0.00
13	->	45	0.00	0.00
13	->	46	-0.01	0.00
13	->	47	0.00	0.00
13	->	48	0.00	0.00
13	->	49	0.00	0.00
13	->	50	0.00	0.00
13	->	51	0.00	0.00
13	->	52	0.00	0.00
13	->	53	0.00	0.00
13	->	54	0.00	0.00
13	->	55	-0.00	0.00
13	->	56	0.00	0.00
13	->	57	0.00	0.00
13	->	58	-0.00	0.00
13	->	59	0.00	0.00
13	->	60	0.00	0.00
13	->	61	0.00	0.00
13	->	62	-0.00	0.00
13	->	63	-0.00	0.00
13	->	64	-0.00	0.00
13	->	65	-0.00	0.00
13	->	66	-0.00	0.00
13	->	67	0.00	0.00
13	->	68	0.00	0.00
13	->	69	0.00	0.00
13	->	70	0.00	0.00
13	->	71	-0.00	0.00
13	->	72	0.01	0.00
13	->	73	0.00	0.00
13	->	74	-0.01	0.00

13 ->	75	-0.00	0.00
13 ->	76	0.00	0.00
13 ->	77	0.01	0.00
13 ->	78	0.00	0.00
13 ->	79	0.00	0.00
13 ->	80	0.00	0.00
13 ->	81	0.00	0.00
13 ->	82	0.00	0.00
13 ->	83	0.00	0.00
13 ->	84	-0.00	0.00
13 ->	85	-0.00	0.00
13 ->	86	-0.00	0.00
13 ->	87	-0.00	0.00
13 ->	88	-0.00	0.00
13 ->	89	-0.01	0.00
13 ->	90	-0.06	0.02
13 ->	91	-0.42	0.37
13 ->	92	-0.55	0.35
13 ->	93	-0.04	0.01
13 ->	94	-0.01	0.00
13 ->	95	-0.01	0.00
13 ->	96	-0.00	0.00
13 ->	97	-0.01	0.00
13 ->	98	-0.01	0.00
13 ->	99	-0.01	0.00
13 ->	100	-0.01	0.00
13 ->	101	-0.01	0.00
13 ->	102	-0.01	0.00
13 ->	103	-0.01	0.00
13 ->	104	-0.01	0.00
13 ->	105	-0.01	0.00
13 ->	106	-0.00	0.00
14 ->	1	-2.45	0.75
14 ->	2	-0.79	0.38
14 ->	3	-3.66	0.49
14 ->	4	-0.75	0.26
14 ->	5	-0.50	0.20
14 ->	6	-0.02	0.01
14 ->	7	-0.04	0.02
14 ->	8	-0.01	0.00
14 ->	9	-0.01	0.00
14 ->	10	-0.04	0.04
14 ->	11	-0.03	0.01
14 ->	12	-1.02	0.29
14 ->	13	-12.35	0.39
14 ->	14	29.36	1.21
14 ->	15	-16.34	0.60
14 ->	16	-1.46	0.24
14 ->	17	-0.22	0.06
14 ->	18	-0.04	0.02

14	->	19	-1.41	0.42
14	->	20	-2.88	0.47
14	->	21	-0.07	0.02
14	->	22	-0.28	0.09
14	->	23	-2.08	0.32
14	->	24	-0.09	0.03
14	->	25	-0.02	0.01
14	->	26	-0.04	0.01
14	->	27	-0.01	0.02
14	->	28	-0.00	0.00
14	->	29	-0.00	0.00
14	->	30	-0.00	0.00
14	->	31	-0.00	0.00
14	->	32	-0.00	0.00
14	->	33	-0.00	0.00
14	->	34	0.00	0.00
14	->	35	-0.00	0.00
14	->	36	0.00	0.00
14	->	37	0.00	0.00
14	->	38	-0.00	0.00
14	->	39	0.00	0.00
14	->	40	0.00	0.00
14	->	41	0.00	0.00
14	->	42	-0.00	0.00
14	->	43	-0.00	0.00
14	->	44	-0.01	0.01
14	->	45	-0.00	0.00
14	->	46	-0.00	0.00
14	->	47	-0.00	0.00
14	->	48	0.00	0.00
14	->	49	0.00	0.00
14	->	50	0.00	0.00
14	->	51	0.00	0.00
14	->	52	0.00	0.00
14	->	53	0.00	0.00
14	->	54	0.00	0.00
14	->	55	0.00	0.00
14	->	56	0.00	0.00
14	->	57	0.00	0.00
14	->	58	-0.00	0.00
14	->	59	0.00	0.00
14	->	60	0.00	0.00
14	->	61	0.00	0.00
14	->	62	-0.00	0.00
14	->	63	-0.00	0.00
14	->	64	-0.00	0.00
14	->	65	0.00	0.00
14	->	66	-0.00	0.00
14	->	67	0.00	0.00
14	->	68	0.00	0.00

14 ->	69	0.00	0.00
14 ->	70	0.00	0.00
14 ->	71	0.00	0.00
14 ->	72	0.00	0.00
14 ->	73	0.00	0.00
14 ->	74	-0.00	0.00
14 ->	75	-0.00	0.00
14 ->	76	0.00	0.00
14 ->	77	0.00	0.00
14 ->	78	0.00	0.00
14 ->	79	0.00	0.00
14 ->	80	0.00	0.00
14 ->	81	0.00	0.00
14 ->	82	0.00	0.00
14 ->	83	0.00	0.00
14 ->	84	-0.00	0.00
14 ->	85	-0.00	0.00
14 ->	86	-0.00	0.00
14 ->	87	-0.00	0.00
14 ->	88	-0.00	0.00
14 ->	89	-0.01	0.00
14 ->	90	-0.06	0.05
14 ->	91	-2.90	0.93
14 ->	92	-3.98	1.71
14 ->	93	-0.11	0.04
14 ->	94	-0.01	0.01
14 ->	95	-0.00	0.00
14 ->	96	-0.00	0.00
14 ->	97	-0.01	0.00
14 ->	98	-0.01	0.00
14 ->	99	-0.01	0.00
14 ->	100	-0.01	0.00
14 ->	101	-0.01	0.00
14 ->	102	-0.01	0.00
14 ->	103	-0.01	0.00
14 ->	104	-0.00	0.00
14 ->	105	-0.00	0.00
14 ->	106	-0.00	0.00
15 ->	1	-2.06	1.29
15 ->	2	-0.66	0.40
15 ->	3	-0.99	0.38
15 ->	4	-0.00	0.01
15 ->	5	0.01	0.01
15 ->	6	-0.00	0.00
15 ->	7	-0.00	0.00
15 ->	8	-0.00	0.00
15 ->	9	-0.00	0.00
15 ->	10	-0.00	0.00
15 ->	11	-0.01	0.00
15 ->	12	-0.07	0.03

15	->	13	-0.53	0.13
15	->	14	-16.37	0.59
15	->	15	23.53	2.76
15	->	16	-10.82	0.62
15	->	17	-0.27	0.07
15	->	18	-0.01	0.03
15	->	19	-0.62	1.12
15	->	20	-0.07	0.02
15	->	21	-0.01	0.00
15	->	22	-0.04	0.08
15	->	23	-0.01	0.00
15	->	24	-0.00	0.00
15	->	25	-0.00	0.00
15	->	26	-0.00	0.00
15	->	27	0.00	0.00
15	->	28	0.00	0.00
15	->	29	0.00	0.00
15	->	30	-0.00	0.00
15	->	31	0.00	0.00
15	->	32	0.00	0.00
15	->	33	-0.00	0.00
15	->	34	0.00	0.00
15	->	35	-0.00	0.00
15	->	36	0.00	0.00
15	->	37	0.00	0.00
15	->	38	-0.00	0.00
15	->	39	-0.00	0.00
15	->	40	0.00	0.00
15	->	41	-0.00	0.00
15	->	42	0.00	0.00
15	->	43	-0.00	0.00
15	->	44	-0.00	0.01
15	->	45	-0.00	0.00
15	->	46	-0.00	0.00
15	->	47	-0.00	0.00
15	->	48	0.00	0.00
15	->	49	0.00	0.00
15	->	50	0.00	0.00
15	->	51	0.00	0.00
15	->	52	0.00	0.00
15	->	53	0.00	0.00
15	->	54	0.00	0.00
15	->	55	0.00	0.00
15	->	56	0.00	0.00
15	->	57	0.00	0.00
15	->	58	-0.00	0.00
15	->	59	0.00	0.00
15	->	60	0.00	0.00
15	->	61	0.00	0.00
15	->	62	0.00	0.00

15	->	63	-0.00	0.00
15	->	64	-0.00	0.00
15	->	65	0.00	0.00
15	->	66	-0.00	0.00
15	->	67	0.00	0.00
15	->	68	0.00	0.00
15	->	69	0.00	0.00
15	->	70	0.00	0.00
15	->	71	0.00	0.00
15	->	72	0.00	0.00
15	->	73	-0.00	0.00
15	->	74	-0.00	0.00
15	->	75	-0.00	0.00
15	->	76	0.00	0.00
15	->	77	0.00	0.00
15	->	78	0.00	0.00
15	->	79	0.00	0.00
15	->	80	0.00	0.00
15	->	81	0.00	0.00
15	->	82	0.00	0.00
15	->	83	0.00	0.00
15	->	84	-0.00	0.00
15	->	85	-0.00	0.00
15	->	86	-0.00	0.00
15	->	87	-0.00	0.00
15	->	88	-0.00	0.00
15	->	89	-0.00	0.00
15	->	90	-0.01	0.01
15	->	91	-0.86	0.54
15	->	92	-8.13	4.21
15	->	93	-0.57	0.32
15	->	94	-0.04	0.04
15	->	95	-0.01	0.00
15	->	96	-0.00	0.00
15	->	97	-0.00	0.00
15	->	98	-0.01	0.00
15	->	99	-0.00	0.00
15	->	100	-0.00	0.00
15	->	101	-0.00	0.00
15	->	102	-0.01	0.00
15	->	103	-0.00	0.00
15	->	104	-0.00	0.00
15	->	105	-0.00	0.00
15	->	106	-0.00	0.00
16	->	1	-0.52	0.15
16	->	2	-0.18	0.15
16	->	3	-1.85	0.58
16	->	4	-0.00	0.01
16	->	5	0.04	0.01
16	->	6	-0.00	0.00

16	->	7	-0.00	0.00
16	->	8	0.01	0.00
16	->	9	-0.00	0.00
16	->	10	0.03	0.00
16	->	11	0.02	0.00
16	->	12	-0.05	0.01
16	->	13	-0.20	0.04
16	->	14	-1.46	0.24
16	->	15	-10.93	0.62
16	->	16	-19.69	1.98
16	->	17	-17.00	0.56
16	->	18	-4.83	1.33
16	->	19	-4.51	0.79
16	->	20	-1.94	0.45
16	->	21	-0.21	0.09
16	->	22	-0.35	0.12
16	->	23	-0.06	0.01
16	->	24	-0.02	0.01
16	->	25	-0.05	0.01
16	->	26	-0.01	0.00
16	->	27	-0.00	0.00
16	->	28	-0.00	0.00
16	->	29	-0.00	0.00
16	->	30	0.00	0.00
16	->	31	-0.01	0.00
16	->	32	-0.00	0.00
16	->	33	-0.00	0.00
16	->	34	-0.00	0.00
16	->	35	0.01	0.00
16	->	36	-0.00	0.00
16	->	37	-0.00	0.00
16	->	38	0.01	0.00
16	->	39	0.00	0.00
16	->	40	-0.01	0.00
16	->	41	-0.00	0.00
16	->	42	-0.00	0.00
16	->	43	0.00	0.00
16	->	44	-0.06	0.01
16	->	45	-0.00	0.00
16	->	46	0.04	0.01
16	->	47	-0.00	0.00
16	->	48	-0.00	0.00
16	->	49	-0.00	0.00
16	->	50	-0.00	0.00
16	->	51	-0.00	0.00
16	->	52	-0.00	0.00
16	->	53	-0.01	0.00
16	->	54	-0.00	0.00
16	->	55	-0.00	0.00
16	->	56	-0.00	0.00

16	->	57	-0.00	0.00
16	->	58	0.01	0.00
16	->	59	-0.00	0.00
16	->	60	-0.00	0.00
16	->	61	-0.00	0.00
16	->	62	0.00	0.00
16	->	63	0.00	0.00
16	->	64	0.00	0.00
16	->	65	0.00	0.00
16	->	66	0.00	0.00
16	->	67	-0.00	0.00
16	->	68	-0.00	0.00
16	->	69	-0.00	0.00
16	->	70	-0.00	0.00
16	->	71	0.00	0.00
16	->	72	-0.01	0.00
16	->	73	-0.00	0.00
16	->	74	0.01	0.00
16	->	75	0.01	0.00
16	->	76	-0.01	0.00
16	->	77	-0.02	0.00
16	->	78	-0.01	0.00
16	->	79	-0.00	0.00
16	->	80	-0.00	0.00
16	->	81	-0.01	0.00
16	->	82	-0.00	0.00
16	->	83	-0.00	0.00
16	->	84	0.00	0.00
16	->	85	0.00	0.00
16	->	86	0.00	0.00
16	->	87	0.01	0.00
16	->	88	0.01	0.00
16	->	89	0.01	0.00
16	->	90	0.03	0.01
16	->	91	0.14	0.13
16	->	92	0.27	0.40
16	->	93	-0.48	0.38
16	->	94	-3.59	1.59
16	->	95	-0.52	0.56
16	->	96	-0.05	0.07
16	->	97	0.11	0.07
16	->	98	0.13	0.12
16	->	99	0.02	0.01
16	->	100	0.03	0.00
16	->	101	0.02	0.00
16	->	102	0.02	0.00
16	->	103	0.02	0.00
16	->	104	0.01	0.00
16	->	105	0.01	0.00
16	->	106	0.00	0.00

17 ->	1	-0.06	0.02
17 ->	2	-0.05	0.03
17 ->	3	-1.94	0.62
17 ->	4	-0.02	0.01
17 ->	5	-0.01	0.01
17 ->	6	-0.01	0.00
17 ->	7	-0.00	0.00
17 ->	8	0.00	0.00
17 ->	9	-0.00	0.00
17 ->	10	0.00	0.00
17 ->	11	-0.00	0.00
17 ->	12	-0.01	0.00
17 ->	13	-0.04	0.01
17 ->	14	-0.22	0.06
17 ->	15	-0.27	0.08
17 ->	16	-17.06	0.57
17 ->	17	19.82	2.10
17 ->	18	-15.43	0.47
17 ->	19	-1.05	0.20
17 ->	20	-2.19	0.49
17 ->	21	-2.76	1.64
17 ->	22	-0.11	0.07
17 ->	23	-0.04	0.02
17 ->	24	-0.06	0.02
17 ->	25	-0.02	0.01
17 ->	26	-0.00	0.00
17 ->	27	-0.00	0.00
17 ->	28	-0.00	0.00
17 ->	29	-0.00	0.00
17 ->	30	-0.00	0.00
17 ->	31	-0.00	0.00
17 ->	32	-0.00	0.00
17 ->	33	-0.00	0.00
17 ->	34	0.00	0.00
17 ->	35	0.00	0.00
17 ->	36	-0.00	0.00
17 ->	37	0.00	0.00
17 ->	38	0.00	0.00
17 ->	39	0.00	0.00
17 ->	40	-0.00	0.00
17 ->	41	0.00	0.00
17 ->	42	0.00	0.00
17 ->	43	0.00	0.00
17 ->	44	-0.00	0.00
17 ->	45	-0.00	0.00
17 ->	46	0.00	0.00
17 ->	47	-0.00	0.00
17 ->	48	-0.00	0.00
17 ->	49	-0.00	0.00
17 ->	50	-0.00	0.00

17	->	51	-0.00	0.00
17	->	52	-0.00	0.00
17	->	53	-0.00	0.00
17	->	54	0.00	0.00
17	->	55	0.00	0.00
17	->	56	-0.00	0.00
17	->	57	-0.00	0.00
17	->	58	0.00	0.00
17	->	59	-0.00	0.00
17	->	60	0.00	0.00
17	->	61	0.00	0.00
17	->	62	0.00	0.00
17	->	63	0.00	0.00
17	->	64	0.00	0.00
17	->	65	0.00	0.00
17	->	66	0.00	0.00
17	->	67	0.00	0.00
17	->	68	-0.00	0.00
17	->	69	-0.00	0.00
17	->	70	-0.00	0.00
17	->	71	0.00	0.00
17	->	72	-0.00	0.00
17	->	73	0.00	0.00
17	->	74	0.00	0.00
17	->	75	0.00	0.00
17	->	76	-0.00	0.00
17	->	77	-0.00	0.00
17	->	78	-0.00	0.00
17	->	79	-0.00	0.00
17	->	80	0.00	0.00
17	->	81	-0.00	0.00
17	->	82	-0.00	0.00
17	->	83	0.00	0.00
17	->	84	0.00	0.00
17	->	85	0.00	0.00
17	->	86	0.00	0.00
17	->	87	0.00	0.00
17	->	88	0.00	0.00
17	->	89	0.00	0.00
17	->	90	-0.00	0.00
17	->	91	0.01	0.02
17	->	92	0.07	0.06
17	->	93	-0.02	0.01
17	->	94	-0.06	0.04
17	->	95	-0.03	0.03
17	->	96	-0.70	1.09
17	->	97	-0.09	0.04
17	->	98	-0.06	0.03
17	->	99	-0.01	0.00
17	->	100	-0.00	0.00

17	->	101	-0.00	0.00
17	->	102	-0.00	0.00
17	->	103	-0.00	0.00
17	->	104	0.00	0.00
17	->	105	0.00	0.00
17	->	106	0.00	0.00
18	->	1	0.01	0.01
18	->	2	-0.01	0.00
18	->	3	-0.04	0.02
18	->	4	-0.00	0.00
18	->	5	-0.00	0.00
18	->	6	-0.00	0.00
18	->	7	-0.00	0.00
18	->	8	0.00	0.00
18	->	9	-0.00	0.00
18	->	10	0.00	0.00
18	->	11	0.00	0.00
18	->	12	-0.00	0.00
18	->	13	0.01	0.01
18	->	14	-0.04	0.02
18	->	15	-0.01	0.03
18	->	16	-4.89	1.34
18	->	17	-15.49	0.47
18	->	18	26.53	2.12
18	->	19	-10.53	0.60
18	->	20	-1.11	0.20
18	->	21	-2.43	0.43
18	->	22	-2.32	0.66
18	->	23	-0.14	0.08
18	->	24	-0.06	0.03
18	->	25	-0.08	0.06
18	->	26	-0.01	0.00
18	->	27	-0.01	0.00
18	->	28	-0.00	0.00
18	->	29	-0.00	0.00
18	->	30	-0.00	0.00
18	->	31	-0.00	0.00
18	->	32	-0.00	0.00
18	->	33	-0.01	0.00
18	->	34	-0.00	0.00
18	->	35	0.00	0.00
18	->	36	-0.00	0.00
18	->	37	-0.00	0.00
18	->	38	0.00	0.00
18	->	39	0.00	0.00
18	->	40	-0.00	0.00
18	->	41	0.00	0.00
18	->	42	0.00	0.00
18	->	43	-0.00	0.00
18	->	44	0.01	0.01

18 ->	45	-0.01	0.01
18 ->	46	-0.02	0.01
18 ->	47	-0.01	0.00
18 ->	48	-0.00	0.00
18 ->	49	-0.00	0.00
18 ->	50	-0.00	0.00
18 ->	51	-0.00	0.00
18 ->	52	-0.00	0.00
18 ->	53	0.00	0.00
18 ->	54	0.00	0.00
18 ->	55	0.00	0.00
18 ->	56	-0.00	0.00
18 ->	57	0.00	0.00
18 ->	58	-0.00	0.00
18 ->	59	0.00	0.00
18 ->	60	0.00	0.00
18 ->	61	0.00	0.00
18 ->	62	-0.00	0.00
18 ->	63	-0.00	0.00
18 ->	64	-0.00	0.00
18 ->	65	-0.00	0.00
18 ->	66	-0.00	0.00
18 ->	67	0.00	0.00
18 ->	68	0.00	0.00
18 ->	69	0.00	0.00
18 ->	70	-0.00	0.00
18 ->	71	0.00	0.00
18 ->	72	0.00	0.00
18 ->	73	0.00	0.00
18 ->	74	-0.00	0.00
18 ->	75	-0.00	0.00
18 ->	76	0.00	0.00
18 ->	77	0.00	0.00
18 ->	78	0.00	0.00
18 ->	79	-0.00	0.00
18 ->	80	0.00	0.00
18 ->	81	0.00	0.00
18 ->	82	0.00	0.00
18 ->	83	0.00	0.00
18 ->	84	-0.00	0.00
18 ->	85	-0.00	0.00
18 ->	86	0.00	0.00
18 ->	87	0.00	0.00
18 ->	88	0.00	0.00
18 ->	89	0.00	0.00
18 ->	90	-0.01	0.01
18 ->	91	-0.08	0.06
18 ->	92	-0.21	0.09
18 ->	93	-0.12	0.10
18 ->	94	-0.18	0.73

18 ->	95	-0.23	0.22
18 ->	96	-0.76	0.76
18 ->	97	-2.78	1.20
18 ->	98	-1.79	0.76
18 ->	99	-0.10	0.05
18 ->	100	-0.01	0.00
18 ->	101	-0.01	0.00
18 ->	102	-0.00	0.00
18 ->	103	-0.00	0.00
18 ->	104	0.00	0.00
18 ->	105	0.00	0.00
18 ->	106	0.00	0.00
19 ->	1	0.19	0.09
19 ->	2	-0.00	0.00
19 ->	3	-0.08	0.04
19 ->	4	-0.00	0.02
19 ->	5	-0.10	0.02
19 ->	6	-0.01	0.00
19 ->	7	0.01	0.01
19 ->	8	-0.00	0.00
19 ->	9	-0.00	0.00
19 ->	10	-0.02	0.01
19 ->	11	-0.00	0.00
19 ->	12	0.04	0.03
19 ->	13	0.16	0.05
19 ->	14	-1.41	0.42
19 ->	15	-0.61	1.12
19 ->	16	-4.52	0.80
19 ->	17	-1.06	0.20
19 ->	18	-10.57	0.60
19 ->	19	-27.77	2.34
19 ->	20	-6.02	0.48
19 ->	21	-0.73	0.18
19 ->	22	-4.37	1.67
19 ->	23	-1.74	0.72
19 ->	24	-0.01	0.04
19 ->	25	-0.00	0.02
19 ->	26	-0.01	0.02
19 ->	27	-0.04	0.01
19 ->	28	-0.01	0.00
19 ->	29	-0.00	0.00
19 ->	30	-0.00	0.00
19 ->	31	-0.00	0.00
19 ->	32	-0.00	0.00
19 ->	33	-0.00	0.00
19 ->	34	-0.00	0.00
19 ->	35	0.00	0.00
19 ->	36	-0.00	0.00
19 ->	37	-0.00	0.00
19 ->	38	0.00	0.00

19	->	39	0.00	0.00
19	->	40	-0.00	0.00
19	->	41	0.00	0.00
19	->	42	0.00	0.00
19	->	43	0.00	0.00
19	->	44	0.00	0.04
19	->	45	-0.01	0.00
19	->	46	-0.01	0.01
19	->	47	-0.01	0.00
19	->	48	-0.00	0.00
19	->	49	-0.00	0.00
19	->	50	-0.00	0.00
19	->	51	-0.00	0.00
19	->	52	-0.00	0.00
19	->	53	-0.00	0.00
19	->	54	-0.00	0.00
19	->	55	0.00	0.00
19	->	56	0.00	0.00
19	->	57	-0.00	0.00
19	->	58	0.00	0.00
19	->	59	-0.00	0.00
19	->	60	-0.00	0.00
19	->	61	-0.00	0.00
19	->	62	0.00	0.00
19	->	63	0.00	0.00
19	->	64	0.00	0.00
19	->	65	0.00	0.00
19	->	66	0.00	0.00
19	->	67	0.00	0.00
19	->	68	-0.00	0.00
19	->	69	-0.00	0.00
19	->	70	0.00	0.00
19	->	71	0.00	0.00
19	->	72	-0.00	0.00
19	->	73	-0.00	0.00
19	->	74	0.00	0.00
19	->	75	0.00	0.00
19	->	76	-0.00	0.00
19	->	77	0.00	0.00
19	->	78	-0.00	0.00
19	->	79	-0.00	0.00
19	->	80	0.00	0.00
19	->	81	0.00	0.00
19	->	82	0.00	0.00
19	->	83	0.00	0.00
19	->	84	-0.00	0.00
19	->	85	0.00	0.00
19	->	86	0.00	0.00
19	->	87	0.00	0.00
19	->	88	0.00	0.00

19	->	89	-0.00	0.00
19	->	90	-0.12	0.05
19	->	91	-1.95	0.94
19	->	92	-4.87	2.04
19	->	93	-3.92	2.14
19	->	94	-0.56	0.41
19	->	95	-0.04	0.02
19	->	96	-0.03	0.02
19	->	97	0.01	0.02
19	->	98	0.15	0.18
19	->	99	0.04	0.08
19	->	100	-0.03	0.02
19	->	101	-0.03	0.01
19	->	102	-0.02	0.01
19	->	103	-0.01	0.00
19	->	104	-0.00	0.00
19	->	105	-0.00	0.00
19	->	106	0.00	0.00
20	->	1	-0.04	0.01
20	->	2	-0.04	0.02
20	->	3	-1.53	0.40
20	->	4	-1.22	0.27
20	->	5	-1.07	0.20
20	->	6	-0.19	0.11
20	->	7	-0.02	0.01
20	->	8	-0.01	0.00
20	->	9	-0.00	0.00
20	->	10	-0.01	0.01
20	->	11	-0.00	0.00
20	->	12	-0.03	0.01
20	->	13	-0.07	0.02
20	->	14	-2.95	0.49
20	->	15	-0.07	0.02
20	->	16	-1.97	0.45
20	->	17	-2.20	0.48
20	->	18	-1.11	0.20
20	->	19	-6.07	0.48
20	->	20	22.80	1.20
20	->	21	-6.49	0.47
20	->	22	-1.53	0.32
20	->	23	-2.90	0.45
20	->	24	-3.99	0.64
20	->	25	-0.14	0.06
20	->	26	-0.09	0.04
20	->	27	-0.06	0.02
20	->	28	-0.02	0.00
20	->	29	-0.00	0.00
20	->	30	-0.00	0.00
20	->	31	-0.00	0.00
20	->	32	-0.00	0.00

20	->	33	-0.00	0.00
20	->	34	-0.00	0.00
20	->	35	0.00	0.00
20	->	36	-0.00	0.00
20	->	37	0.00	0.00
20	->	38	0.00	0.00
20	->	39	0.00	0.00
20	->	40	-0.00	0.00
20	->	41	0.00	0.00
20	->	42	-0.00	0.00
20	->	43	-0.01	0.01
20	->	44	-0.02	0.01
20	->	45	-0.00	0.00
20	->	46	-0.00	0.00
20	->	47	-0.00	0.00
20	->	48	-0.00	0.00
20	->	49	-0.00	0.00
20	->	50	-0.00	0.00
20	->	51	-0.00	0.00
20	->	52	-0.00	0.00
20	->	53	-0.00	0.00
20	->	54	-0.00	0.00
20	->	55	0.00	0.00
20	->	56	-0.00	0.00
20	->	57	0.00	0.00
20	->	58	0.00	0.00
20	->	59	-0.00	0.00
20	->	60	0.00	0.00
20	->	61	0.00	0.00
20	->	62	0.00	0.00
20	->	63	0.00	0.00
20	->	64	0.00	0.00
20	->	65	0.00	0.00
20	->	66	0.00	0.00
20	->	67	0.00	0.00
20	->	68	-0.00	0.00
20	->	69	-0.00	0.00
20	->	70	0.00	0.00
20	->	71	-0.00	0.00
20	->	72	-0.00	0.00
20	->	73	-0.00	0.00
20	->	74	0.00	0.00
20	->	75	0.00	0.00
20	->	76	0.00	0.00
20	->	77	0.00	0.00
20	->	78	-0.00	0.00
20	->	79	0.00	0.00
20	->	80	0.00	0.00
20	->	81	-0.00	0.00
20	->	82	0.00	0.00

20	->	83	0.00	0.00
20	->	84	0.00	0.00
20	->	85	0.00	0.00
20	->	86	0.00	0.00
20	->	87	0.00	0.00
20	->	88	-0.00	0.00
20	->	89	-0.00	0.00
20	->	90	0.01	0.01
20	->	91	0.02	0.05
20	->	92	0.06	0.04
20	->	93	-0.02	0.01
20	->	94	-0.01	0.01
20	->	95	-0.00	0.00
20	->	96	-0.02	0.03
20	->	97	-0.03	0.01
20	->	98	-0.02	0.02
20	->	99	-0.00	0.00
20	->	100	-0.00	0.00
20	->	101	-0.00	0.00
20	->	102	-0.00	0.00
20	->	103	-0.00	0.00
20	->	104	-0.00	0.00
20	->	105	0.00	0.00
20	->	106	0.00	0.00
21	->	1	-0.00	0.00
21	->	2	-0.00	0.00
21	->	3	-0.04	0.01
21	->	4	-0.02	0.01
21	->	5	0.00	0.01
21	->	6	-0.02	0.01
21	->	7	-0.01	0.01
21	->	8	0.00	0.00
21	->	9	-0.00	0.00
21	->	10	0.00	0.00
21	->	11	-0.00	0.00
21	->	12	-0.00	0.00
21	->	13	-0.01	0.01
21	->	14	-0.07	0.02
21	->	15	-0.01	0.00
21	->	16	-0.21	0.09
21	->	17	-2.77	1.63
21	->	18	-2.44	0.43
21	->	19	-0.74	0.18
21	->	20	-6.50	0.47
21	->	21	-21.15	2.88
21	->	22	-10.69	0.38
21	->	23	-0.91	0.18
21	->	24	-3.06	0.54
21	->	25	-2.89	0.92
21	->	26	-0.12	0.06

21	->	27	-0.05	0.02
21	->	28	-0.03	0.02
21	->	29	-0.01	0.00
21	->	30	-0.05	0.02
21	->	31	-0.00	0.00
21	->	32	-0.01	0.01
21	->	33	-0.02	0.02
21	->	34	-0.00	0.00
21	->	35	0.00	0.00
21	->	36	-0.00	0.00
21	->	37	-0.00	0.00
21	->	38	0.00	0.00
21	->	39	0.00	0.00
21	->	40	-0.00	0.00
21	->	41	0.00	0.00
21	->	42	0.00	0.00
21	->	43	-0.04	0.05
21	->	44	0.03	0.03
21	->	45	-0.01	0.01
21	->	46	-0.02	0.01
21	->	47	-0.01	0.00
21	->	48	-0.00	0.00
21	->	49	-0.00	0.00
21	->	50	-0.00	0.00
21	->	51	-0.00	0.00
21	->	52	-0.00	0.00
21	->	53	0.00	0.00
21	->	54	0.00	0.00
21	->	55	0.00	0.00
21	->	56	0.00	0.00
21	->	57	0.00	0.00
21	->	58	-0.00	0.00
21	->	59	0.00	0.00
21	->	60	0.00	0.00
21	->	61	0.00	0.00
21	->	62	-0.00	0.00
21	->	63	-0.00	0.00
21	->	64	-0.00	0.00
21	->	65	-0.00	0.00
21	->	66	-0.00	0.00
21	->	67	0.00	0.00
21	->	68	0.00	0.00
21	->	69	0.00	0.00
21	->	70	0.00	0.00
21	->	71	0.00	0.00
21	->	72	0.00	0.00
21	->	73	0.00	0.00
21	->	74	-0.00	0.00
21	->	75	-0.00	0.00
21	->	76	0.00	0.00

21 ->	77	0.00	0.00
21 ->	78	0.00	0.00
21 ->	79	0.00	0.00
21 ->	80	0.00	0.00
21 ->	81	0.00	0.00
21 ->	82	0.00	0.00
21 ->	83	0.00	0.00
21 ->	84	-0.00	0.00
21 ->	85	0.00	0.00
21 ->	86	-0.00	0.00
21 ->	87	0.00	0.00
21 ->	88	-0.00	0.00
21 ->	89	-0.00	0.00
21 ->	90	-0.01	0.01
21 ->	91	-0.06	0.04
21 ->	92	-0.04	0.02
21 ->	93	-0.00	0.01
21 ->	94	-0.00	0.01
21 ->	95	-0.01	0.00
21 ->	96	-0.31	0.46
21 ->	97	-0.30	0.28
21 ->	98	-0.16	0.11
21 ->	99	-0.02	0.01
21 ->	100	-0.00	0.00
21 ->	101	-0.00	0.00
21 ->	102	-0.00	0.00
21 ->	103	-0.00	0.00
21 ->	104	-0.00	0.00
21 ->	105	0.00	0.00
21 ->	106	0.00	0.00
22 ->	1	0.11	0.02
22 ->	2	-0.00	0.00
22 ->	3	-0.01	0.01
22 ->	4	0.01	0.01
22 ->	5	-0.14	0.04
22 ->	6	-0.01	0.00
22 ->	7	0.01	0.01
22 ->	8	-0.01	0.00
22 ->	9	-0.00	0.00
22 ->	10	-0.07	0.01
22 ->	11	-0.03	0.01
22 ->	12	0.07	0.03
22 ->	13	0.05	0.01
22 ->	14	-0.28	0.09
22 ->	15	-0.04	0.08
22 ->	16	-0.35	0.12
22 ->	17	-0.11	0.07
22 ->	18	-2.35	0.66
22 ->	19	-4.41	1.67
22 ->	20	-1.53	0.31

22	->	21	-10.71	0.38
22	->	22	-96.43	2.53
22	->	23	-19.61	0.47
22	->	24	-0.72	0.17
22	->	25	-2.29	0.68
22	->	26	-1.72	0.96
22	->	27	-0.10	0.04
22	->	28	-0.02	0.01
22	->	29	-0.00	0.01
22	->	30	-0.03	0.01
22	->	31	0.02	0.01
22	->	32	-0.01	0.01
22	->	33	-0.04	0.03
22	->	34	-0.00	0.00
22	->	35	-0.02	0.00
22	->	36	0.01	0.00
22	->	37	0.00	0.00
22	->	38	-0.01	0.00
22	->	39	-0.00	0.00
22	->	40	0.02	0.01
22	->	41	0.01	0.00
22	->	42	0.01	0.01
22	->	43	-0.30	0.32
22	->	44	-0.68	0.99
22	->	45	-0.10	0.05
22	->	46	-1.10	0.47
22	->	47	-0.13	0.04
22	->	48	-0.01	0.01
22	->	49	0.00	0.00
22	->	50	-0.00	0.00
22	->	51	0.00	0.00
22	->	52	0.00	0.00
22	->	53	0.03	0.00
22	->	54	0.00	0.00
22	->	55	0.00	0.00
22	->	56	0.00	0.00
22	->	57	0.00	0.00
22	->	58	-0.01	0.00
22	->	59	0.01	0.00
22	->	60	0.00	0.00
22	->	61	-0.00	0.00
22	->	62	-0.00	0.00
22	->	63	-0.01	0.00
22	->	64	-0.00	0.00
22	->	65	-0.00	0.00
22	->	66	-0.00	0.00
22	->	67	-0.00	0.00
22	->	68	0.01	0.00
22	->	69	0.01	0.00
22	->	70	0.00	0.00

22 ->	71	-0.00	0.00
22 ->	72	0.04	0.00
22 ->	73	0.00	0.00
22 ->	74	-0.04	0.00
22 ->	75	-0.02	0.00
22 ->	76	0.02	0.01
22 ->	77	0.02	0.00
22 ->	78	0.02	0.00
22 ->	79	0.00	0.00
22 ->	80	0.00	0.00
22 ->	81	0.01	0.00
22 ->	82	0.00	0.00
22 ->	83	0.00	0.00
22 ->	84	-0.00	0.00
22 ->	85	-0.00	0.00
22 ->	86	-0.01	0.00
22 ->	87	-0.02	0.00
22 ->	88	-0.03	0.00
22 ->	89	-0.09	0.03
22 ->	90	-0.51	0.23
22 ->	91	-3.84	1.04
22 ->	92	-8.54	1.45
22 ->	93	-0.09	0.80
22 ->	94	-0.03	0.13
22 ->	95	-0.04	0.02
22 ->	96	-0.03	0.02
22 ->	97	-0.13	0.06
22 ->	98	-1.52	1.97
22 ->	99	-2.54	1.62
22 ->	100	0.21	0.25
22 ->	101	-0.17	0.09
22 ->	102	-0.09	0.03
22 ->	103	-0.04	0.01
22 ->	104	-0.03	0.00
22 ->	105	-0.02	0.00
22 ->	106	-0.01	0.00
23 ->	1	0.00	0.00
23 ->	2	-0.00	0.00
23 ->	3	-0.02	0.01
23 ->	4	-0.09	0.03
23 ->	5	1.23	0.36
23 ->	6	-0.08	0.03
23 ->	7	-0.37	0.17
23 ->	8	0.00	0.01
23 ->	9	-0.02	0.03
23 ->	10	1.48	0.31
23 ->	11	0.00	0.01
23 ->	12	-0.09	0.08
23 ->	13	-0.05	0.01
23 ->	14	-2.10	0.32

23	->	15	-0.01	0.00
23	->	16	-0.06	0.01
23	->	17	-0.04	0.02
23	->	18	-0.14	0.08
23	->	19	-1.77	0.72
23	->	20	-2.91	0.45
23	->	21	-0.91	0.18
23	->	22	-19.65	0.47
23	->	23	10.73	1.04
23	->	24	-10.33	0.49
23	->	25	-1.33	0.18
23	->	26	-3.56	0.70
23	->	27	24.99	9.60
23	->	28	-0.21	0.06
23	->	29	-0.04	0.02
23	->	30	-0.03	0.01
23	->	31	-0.01	0.00
23	->	32	-0.00	0.00
23	->	33	-0.00	0.00
23	->	34	-0.00	0.00
23	->	35	-0.00	0.00
23	->	36	0.00	0.00
23	->	37	0.00	0.00
23	->	38	-0.00	0.00
23	->	39	0.00	0.00
23	->	40	-0.00	0.00
23	->	41	-0.00	0.00
23	->	42	-0.01	0.01
23	->	43	-0.04	0.01
23	->	44	-0.09	0.04
23	->	45	0.00	0.00
23	->	46	0.00	0.01
23	->	47	-0.00	0.00
23	->	48	-0.00	0.00
23	->	49	-0.00	0.00
23	->	50	-0.00	0.00
23	->	51	-0.00	0.00
23	->	52	0.00	0.00
23	->	53	0.00	0.00
23	->	54	0.00	0.00
23	->	55	0.00	0.00
23	->	56	0.00	0.00
23	->	57	0.00	0.00
23	->	58	-0.00	0.00
23	->	59	0.00	0.00
23	->	60	0.00	0.00
23	->	61	0.00	0.00
23	->	62	0.00	0.00
23	->	63	-0.00	0.00
23	->	64	-0.00	0.00

23	->	65	0.00	0.00
23	->	66	-0.00	0.00
23	->	67	0.00	0.00
23	->	68	0.00	0.00
23	->	69	0.00	0.00
23	->	70	0.00	0.00
23	->	71	-0.00	0.00
23	->	72	0.00	0.00
23	->	73	0.00	0.00
23	->	74	-0.00	0.00
23	->	75	-0.00	0.00
23	->	76	0.00	0.00
23	->	77	0.00	0.00
23	->	78	0.00	0.00
23	->	79	0.00	0.00
23	->	80	0.00	0.00
23	->	81	0.00	0.00
23	->	82	0.00	0.00
23	->	83	0.00	0.00
23	->	84	-0.00	0.00
23	->	85	-0.00	0.00
23	->	86	-0.00	0.00
23	->	87	-0.00	0.00
23	->	88	-0.01	0.00
23	->	89	-0.03	0.01
23	->	90	-0.83	0.36
23	->	91	-4.66	1.74
23	->	92	-0.06	0.02
23	->	93	-0.01	0.00
23	->	94	-0.01	0.00
23	->	95	-0.00	0.00
23	->	96	-0.00	0.00
23	->	97	-0.01	0.00
23	->	98	-0.01	0.01
23	->	99	-0.01	0.00
23	->	100	-0.01	0.00
23	->	101	-0.01	0.00
23	->	102	-0.01	0.00
23	->	103	-0.01	0.00
23	->	104	-0.01	0.00
23	->	105	-0.00	0.00
23	->	106	-0.00	0.00
24	->	1	-0.00	0.00
24	->	2	-0.00	0.00
24	->	3	-0.02	0.01
24	->	4	-0.08	0.04
24	->	5	-0.76	0.25
24	->	6	-1.19	0.35
24	->	7	-0.10	0.05
24	->	8	-0.01	0.00

24	->	9	-0.00	0.00
24	->	10	0.03	0.01
24	->	11	-0.00	0.00
24	->	12	-0.01	0.01
24	->	13	-0.01	0.00
24	->	14	-0.09	0.03
24	->	15	-0.00	0.00
24	->	16	-0.02	0.01
24	->	17	-0.06	0.02
24	->	18	-0.06	0.03
24	->	19	-0.01	0.04
24	->	20	-3.96	0.64
24	->	21	-3.09	0.53
24	->	22	-0.73	0.17
24	->	23	-10.38	0.49
24	->	24	24.05	1.56
24	->	25	-10.28	0.38
24	->	26	-1.04	0.21
24	->	27	-2.69	0.41
24	->	28	-5.46	0.74
24	->	29	-0.15	0.06
24	->	30	-0.42	0.20
24	->	31	-0.01	0.01
24	->	32	-0.01	0.00
24	->	33	-0.01	0.00
24	->	34	-0.00	0.00
24	->	35	0.00	0.00
24	->	36	-0.00	0.00
24	->	37	-0.00	0.00
24	->	38	0.00	0.00
24	->	39	0.00	0.00
24	->	40	-0.00	0.00
24	->	41	0.00	0.00
24	->	42	-0.00	0.00
24	->	43	-0.02	0.01
24	->	44	-0.02	0.01
24	->	45	-0.00	0.00
24	->	46	-0.00	0.00
24	->	47	-0.00	0.00
24	->	48	-0.00	0.00
24	->	49	-0.00	0.00
24	->	50	0.00	0.00
24	->	51	0.00	0.00
24	->	52	0.00	0.00
24	->	53	0.00	0.00
24	->	54	-0.00	0.00
24	->	55	0.00	0.00
24	->	56	-0.00	0.00
24	->	57	0.00	0.00
24	->	58	-0.00	0.00

24	->	59	0.00	0.00
24	->	60	0.00	0.00
24	->	61	0.00	0.00
24	->	62	-0.00	0.00
24	->	63	-0.00	0.00
24	->	64	-0.00	0.00
24	->	65	0.00	0.00
24	->	66	-0.00	0.00
24	->	67	0.00	0.00
24	->	68	-0.00	0.00
24	->	69	0.00	0.00
24	->	70	-0.00	0.00
24	->	71	-0.00	0.00
24	->	72	0.00	0.00
24	->	73	0.00	0.00
24	->	74	-0.00	0.00
24	->	75	-0.00	0.00
24	->	76	0.00	0.00
24	->	77	0.00	0.00
24	->	78	0.00	0.00
24	->	79	0.00	0.00
24	->	80	0.00	0.00
24	->	81	0.00	0.00
24	->	82	0.00	0.00
24	->	83	0.00	0.00
24	->	84	-0.00	0.00
24	->	85	0.00	0.00
24	->	86	0.00	0.00
24	->	87	0.00	0.00
24	->	88	-0.00	0.00
24	->	89	0.00	0.00
24	->	90	0.01	0.01
24	->	91	0.06	0.03
24	->	92	-0.00	0.01
24	->	93	-0.00	0.00
24	->	94	-0.00	0.00
24	->	95	-0.00	0.00
24	->	96	-0.01	0.01
24	->	97	-0.01	0.01
24	->	98	-0.02	0.01
24	->	99	-0.00	0.00
24	->	100	-0.00	0.00
24	->	101	-0.00	0.00
24	->	102	-0.00	0.00
24	->	103	-0.00	0.00
24	->	104	-0.00	0.00
24	->	105	-0.00	0.00
24	->	106	0.00	0.00
25	->	1	0.03	0.00
25	->	2	-0.00	0.00

25	->	3	-0.00	0.00
25	->	4	-0.00	0.00
25	->	5	-0.06	0.01
25	->	6	-0.02	0.01
25	->	7	-0.00	0.01
25	->	8	-0.01	0.00
25	->	9	-0.00	0.00
25	->	10	-0.04	0.00
25	->	11	-0.01	0.00
25	->	12	0.02	0.01
25	->	13	0.01	0.00
25	->	14	-0.02	0.01
25	->	15	-0.00	0.00
25	->	16	-0.05	0.01
25	->	17	-0.02	0.01
25	->	18	-0.08	0.06
25	->	19	-0.00	0.02
25	->	20	-0.14	0.06
25	->	21	-2.92	0.91
25	->	22	-2.31	0.65
25	->	23	-1.34	0.18
25	->	24	-10.30	0.38
25	->	25	-105.17	2.80
25	->	26	-12.46	0.57
25	->	27	-0.66	0.14
25	->	28	-1.24	0.36
25	->	29	-0.89	0.32
25	->	30	-5.07	1.94
25	->	31	0.02	0.10
25	->	32	-0.85	0.52
25	->	33	-0.85	0.49
25	->	34	-0.01	0.00
25	->	35	-0.03	0.00
25	->	36	0.02	0.01
25	->	37	0.00	0.00
25	->	38	-0.02	0.00
25	->	39	-0.00	0.00
25	->	40	0.03	0.02
25	->	41	0.03	0.01
25	->	42	0.03	0.04
25	->	43	-2.17	1.36
25	->	44	-0.14	0.18
25	->	45	-0.15	0.08
25	->	46	-0.13	0.04
25	->	47	-0.01	0.01
25	->	48	-0.01	0.00
25	->	49	0.00	0.00
25	->	50	0.00	0.00
25	->	51	0.00	0.00
25	->	52	0.00	0.00

25	->	53	0.02	0.00
25	->	54	0.00	0.00
25	->	55	-0.00	0.00
25	->	56	0.00	0.00
25	->	57	0.00	0.00
25	->	58	-0.01	0.00
25	->	59	0.01	0.00
25	->	60	0.00	0.00
25	->	61	0.00	0.00
25	->	62	-0.00	0.00
25	->	63	-0.00	0.00
25	->	64	-0.00	0.00
25	->	65	-0.00	0.00
25	->	66	-0.00	0.00
25	->	67	-0.00	0.00
25	->	68	0.00	0.00
25	->	69	0.00	0.00
25	->	70	0.00	0.00
25	->	71	-0.00	0.00
25	->	72	0.02	0.00
25	->	73	0.00	0.00
25	->	74	-0.01	0.00
25	->	75	-0.01	0.00
25	->	76	0.01	0.00
25	->	77	0.01	0.00
25	->	78	0.01	0.00
25	->	79	0.00	0.00
25	->	80	0.00	0.00
25	->	81	0.00	0.00
25	->	82	0.00	0.00
25	->	83	-0.00	0.00
25	->	84	-0.00	0.00
25	->	85	-0.00	0.00
25	->	86	-0.01	0.00
25	->	87	-0.01	0.00
25	->	88	-0.02	0.00
25	->	89	-0.06	0.02
25	->	90	-0.12	0.06
25	->	91	-0.11	0.04
25	->	92	-0.05	0.01
25	->	93	-0.02	0.00
25	->	94	-0.02	0.00
25	->	95	-0.02	0.00
25	->	96	-0.03	0.02
25	->	97	-0.78	0.85
25	->	98	-3.29	4.22
25	->	99	-0.08	0.04
25	->	100	-0.02	0.00
25	->	101	-0.01	0.00
25	->	102	-0.01	0.00

25 ->	103	-0.01	0.00
25 ->	104	-0.01	0.00
25 ->	105	-0.01	0.00
25 ->	106	-0.00	0.00
26 ->	1	0.00	0.00
26 ->	2	-0.00	0.00
26 ->	3	-0.00	0.00
26 ->	4	-0.00	0.00
26 ->	5	-0.08	0.03
26 ->	6	-0.01	0.00
26 ->	7	0.01	0.01
26 ->	8	-0.00	0.00
26 ->	9	-0.01	0.01
26 ->	10	-0.05	0.01
26 ->	11	-0.00	0.00
26 ->	12	-0.00	0.00
26 ->	13	0.00	0.00
26 ->	14	-0.04	0.01
26 ->	15	-0.00	0.00
26 ->	16	-0.01	0.00
26 ->	17	-0.00	0.00
26 ->	18	-0.01	0.00
26 ->	19	-0.01	0.02
26 ->	20	-0.09	0.04
26 ->	21	-0.12	0.06
26 ->	22	-1.74	0.95
26 ->	23	-3.57	0.71
26 ->	24	-1.05	0.21
26 ->	25	-12.51	0.57
26 ->	26	20.47	1.64
26 ->	27	-21.49	0.39
26 ->	28	-0.71	0.15
26 ->	29	-1.53	0.37
26 ->	30	-0.53	0.28
26 ->	31	-0.30	0.33
26 ->	32	-0.03	0.01
26 ->	33	-0.01	0.00
26 ->	34	-0.00	0.00
26 ->	35	-0.00	0.00
26 ->	36	-0.00	0.00
26 ->	37	0.00	0.00
26 ->	38	-0.00	0.00
26 ->	39	-0.00	0.00
26 ->	40	-0.03	0.04
26 ->	41	-0.02	0.01
26 ->	42	-0.03	0.02
26 ->	43	-1.69	0.55
26 ->	44	-0.52	0.38
26 ->	45	-0.00	0.00
26 ->	46	-0.01	0.00

26	->	47	-0.04	0.04
26	->	48	-0.00	0.00
26	->	49	-0.00	0.00
26	->	50	-0.00	0.00
26	->	51	-0.00	0.00
26	->	52	-0.00	0.00
26	->	53	0.00	0.00
26	->	54	0.00	0.00
26	->	55	-0.00	0.00
26	->	56	0.00	0.00
26	->	57	0.00	0.00
26	->	58	-0.00	0.00
26	->	59	0.00	0.00
26	->	60	0.00	0.00
26	->	61	0.00	0.00
26	->	62	-0.00	0.00
26	->	63	-0.00	0.00
26	->	64	-0.00	0.00
26	->	65	0.00	0.00
26	->	66	-0.00	0.00
26	->	67	0.00	0.00
26	->	68	0.00	0.00
26	->	69	0.00	0.00
26	->	70	0.00	0.00
26	->	71	-0.00	0.00
26	->	72	0.00	0.00
26	->	73	0.00	0.00
26	->	74	-0.00	0.00
26	->	75	-0.00	0.00
26	->	76	0.00	0.00
26	->	77	0.00	0.00
26	->	78	0.00	0.00
26	->	79	0.00	0.00
26	->	80	0.00	0.00
26	->	81	0.00	0.00
26	->	82	0.00	0.00
26	->	83	0.00	0.00
26	->	84	-0.00	0.00
26	->	85	-0.00	0.00
26	->	86	-0.00	0.00
26	->	87	-0.00	0.00
26	->	88	-0.01	0.00
26	->	89	-0.11	0.05
26	->	90	-1.14	0.89
26	->	91	-0.28	0.28
26	->	92	-0.01	0.00
26	->	93	-0.01	0.00
26	->	94	-0.00	0.00
26	->	95	-0.00	0.00
26	->	96	-0.00	0.00

26	->	97	-0.01	0.00
26	->	98	-0.01	0.00
26	->	99	-0.01	0.00
26	->	100	-0.00	0.00
26	->	101	-0.00	0.00
26	->	102	-0.00	0.00
26	->	103	-0.00	0.00
26	->	104	-0.00	0.00
26	->	105	-0.00	0.00
26	->	106	-0.00	0.00
27	->	1	-0.01	0.00
27	->	2	-0.00	0.00
27	->	3	-0.01	0.00
27	->	4	-0.07	0.02
27	->	5	2.50	1.07
27	->	6	-0.36	0.18
27	->	7	-2.47	0.97
27	->	8	0.00	0.04
27	->	9	-0.15	0.14
27	->	10	2.20	0.86
27	->	11	0.02	0.01
27	->	12	-0.05	0.04
27	->	13	-0.02	0.01
27	->	14	-0.01	0.02
27	->	15	0.00	0.00
27	->	16	-0.00	0.00
27	->	17	-0.00	0.00
27	->	18	-0.01	0.00
27	->	19	-0.04	0.01
27	->	20	-0.06	0.02
27	->	21	-0.05	0.02
27	->	22	-0.10	0.04
27	->	23	24.98	9.60
27	->	24	-2.67	0.40
27	->	25	-0.67	0.14
27	->	26	-21.57	0.39
27	->	27	4.08	1.30
27	->	28	-4.96	0.37
27	->	29	-0.46	0.15
27	->	30	-0.04	0.03
27	->	31	-0.03	0.01
27	->	32	-0.00	0.00
27	->	33	-0.00	0.00
27	->	34	-0.00	0.00
27	->	35	-0.00	0.00
27	->	36	0.00	0.00
27	->	37	0.00	0.00
27	->	38	-0.00	0.00
27	->	39	-0.00	0.00
27	->	40	-0.00	0.00

27	->	41	-0.01	0.00
27	->	42	-0.01	0.01
27	->	43	-0.00	0.01
27	->	44	-0.01	0.01
27	->	45	0.00	0.00
27	->	46	-0.00	0.00
27	->	47	-0.00	0.00
27	->	48	-0.00	0.00
27	->	49	0.00	0.00
27	->	50	0.00	0.00
27	->	51	0.00	0.00
27	->	52	0.00	0.00
27	->	53	0.00	0.00
27	->	54	0.00	0.00
27	->	55	-0.00	0.00
27	->	56	0.00	0.00
27	->	57	0.00	0.00
27	->	58	-0.00	0.00
27	->	59	0.00	0.00
27	->	60	0.00	0.00
27	->	61	0.00	0.00
27	->	62	0.00	0.00
27	->	63	-0.00	0.00
27	->	64	-0.00	0.00
27	->	65	0.00	0.00
27	->	66	-0.00	0.00
27	->	67	0.00	0.00
27	->	68	0.00	0.00
27	->	69	0.00	0.00
27	->	70	0.00	0.00
27	->	71	-0.00	0.00
27	->	72	0.00	0.00
27	->	73	0.00	0.00
27	->	74	-0.00	0.00
27	->	75	-0.00	0.00
27	->	76	0.00	0.00
27	->	77	0.00	0.00
27	->	78	0.00	0.00
27	->	79	0.00	0.00
27	->	80	0.00	0.00
27	->	81	0.00	0.00
27	->	82	0.00	0.00
27	->	83	-0.00	0.00
27	->	84	0.00	0.00
27	->	85	-0.00	0.00
27	->	86	-0.00	0.00
27	->	87	-0.00	0.00
27	->	88	-0.00	0.00
27	->	89	0.00	0.01
27	->	90	0.04	0.03

27	->	91	0.10	0.06
27	->	92	0.00	0.00
27	->	93	-0.00	0.00
27	->	94	-0.00	0.00
27	->	95	-0.00	0.00
27	->	96	-0.00	0.00
27	->	97	-0.00	0.00
27	->	98	-0.00	0.00
27	->	99	-0.00	0.00
27	->	100	-0.00	0.00
27	->	101	-0.00	0.00
27	->	102	-0.00	0.00
27	->	103	-0.00	0.00
27	->	104	-0.00	0.00
27	->	105	-0.00	0.00
27	->	106	-0.00	0.00
28	->	1	0.00	0.00
28	->	2	-0.00	0.00
28	->	3	-0.00	0.00
28	->	4	-0.00	0.00
28	->	5	-0.02	0.01
28	->	6	-0.07	0.09
28	->	7	-0.05	0.03
28	->	8	-0.00	0.00
28	->	9	-0.00	0.00
28	->	10	-0.01	0.00
28	->	11	-0.00	0.00
28	->	12	0.00	0.00
28	->	13	0.00	0.00
28	->	14	-0.00	0.00
28	->	15	0.00	0.00
28	->	16	-0.00	0.00
28	->	17	-0.00	0.00
28	->	18	-0.00	0.00
28	->	19	-0.01	0.00
28	->	20	-0.02	0.00
28	->	21	-0.03	0.02
28	->	22	-0.02	0.01
28	->	23	-0.21	0.07
28	->	24	-5.47	0.73
28	->	25	-1.25	0.35
28	->	26	-0.73	0.15
28	->	27	-5.11	0.37
28	->	28	-22.32	1.46
28	->	29	-11.61	0.48
28	->	30	-3.81	1.29
28	->	31	-0.04	0.01
28	->	32	-0.02	0.01
28	->	33	-0.00	0.00
28	->	34	-0.00	0.00

28 ->	35	0.00	0.00
28 ->	36	-0.00	0.00
28 ->	37	0.00	0.00
28 ->	38	0.00	0.00
28 ->	39	-0.00	0.00
28 ->	40	-0.00	0.00
28 ->	41	-0.00	0.00
28 ->	42	-0.01	0.00
28 ->	43	-0.04	0.02
28 ->	44	-0.00	0.00
28 ->	45	-0.00	0.00
28 ->	46	-0.00	0.00
28 ->	47	-0.00	0.00
28 ->	48	-0.00	0.00
28 ->	49	0.00	0.00
28 ->	50	0.00	0.00
28 ->	51	0.00	0.00
28 ->	52	0.00	0.00
28 ->	53	0.00	0.00
28 ->	54	0.00	0.00
28 ->	55	0.00	0.00
28 ->	56	0.00	0.00
28 ->	57	0.00	0.00
28 ->	58	0.00	0.00
28 ->	59	0.00	0.00
28 ->	60	0.00	0.00
28 ->	61	0.00	0.00
28 ->	62	0.00	0.00
28 ->	63	-0.00	0.00
28 ->	64	0.00	0.00
28 ->	65	0.00	0.00
28 ->	66	-0.00	0.00
28 ->	67	0.00	0.00
28 ->	68	0.00	0.00
28 ->	69	0.00	0.00
28 ->	70	0.00	0.00
28 ->	71	0.00	0.00
28 ->	72	0.00	0.00
28 ->	73	0.00	0.00
28 ->	74	-0.00	0.00
28 ->	75	-0.00	0.00
28 ->	76	0.00	0.00
28 ->	77	0.00	0.00
28 ->	78	0.00	0.00
28 ->	79	0.00	0.00
28 ->	80	0.00	0.00
28 ->	81	0.00	0.00
28 ->	82	0.00	0.00
28 ->	83	0.00	0.00
28 ->	84	-0.00	0.00

28	->	85	0.00	0.00
28	->	86	-0.00	0.00
28	->	87	-0.00	0.00
28	->	88	-0.00	0.00
28	->	89	-0.00	0.00
28	->	90	-0.01	0.00
28	->	91	-0.01	0.00
28	->	92	-0.00	0.00
28	->	93	-0.00	0.00
28	->	94	-0.00	0.00
28	->	95	-0.00	0.00
28	->	96	-0.00	0.00
28	->	97	-0.00	0.00
28	->	98	-0.00	0.00
28	->	99	-0.00	0.00
28	->	100	-0.00	0.00
28	->	101	-0.00	0.00
28	->	102	-0.00	0.00
28	->	103	-0.00	0.00
28	->	104	-0.00	0.00
28	->	105	-0.00	0.00
28	->	106	-0.00	0.00
29	->	1	0.00	0.00
29	->	2	-0.00	0.00
29	->	3	0.00	0.00
29	->	4	0.00	0.00
29	->	5	-0.01	0.00
29	->	6	-0.00	0.00
29	->	7	-0.00	0.00
29	->	8	-0.00	0.00
29	->	9	-0.00	0.00
29	->	10	-0.00	0.00
29	->	11	-0.00	0.00
29	->	12	0.00	0.00
29	->	13	0.00	0.00
29	->	14	-0.00	0.00
29	->	15	0.00	0.00
29	->	16	-0.00	0.00
29	->	17	-0.00	0.00
29	->	18	-0.00	0.00
29	->	19	-0.00	0.00
29	->	20	-0.00	0.00
29	->	21	-0.01	0.00
29	->	22	-0.00	0.01
29	->	23	-0.04	0.02
29	->	24	-0.15	0.06
29	->	25	-0.89	0.32
29	->	26	-1.57	0.37
29	->	27	-0.49	0.15
29	->	28	-11.68	0.47

29	->	29	12.40	0.67
29	->	30	-14.53	0.44
29	->	31	-1.37	0.58
29	->	32	-0.03	0.00
29	->	33	-0.01	0.00
29	->	34	-0.00	0.00
29	->	35	0.00	0.00
29	->	36	-0.00	0.00
29	->	37	0.00	0.00
29	->	38	0.00	0.00
29	->	39	-0.00	0.00
29	->	40	-0.00	0.00
29	->	41	-0.00	0.01
29	->	42	0.01	0.01
29	->	43	-0.16	0.15
29	->	44	-0.01	0.00
29	->	45	-0.00	0.00
29	->	46	-0.00	0.00
29	->	47	-0.00	0.00
29	->	48	-0.00	0.00
29	->	49	0.00	0.00
29	->	50	0.00	0.00
29	->	51	-0.00	0.00
29	->	52	0.00	0.00
29	->	53	0.00	0.00
29	->	54	0.00	0.00
29	->	55	0.00	0.00
29	->	56	0.00	0.00
29	->	57	0.00	0.00
29	->	58	-0.00	0.00
29	->	59	0.00	0.00
29	->	60	0.00	0.00
29	->	61	0.00	0.00
29	->	62	-0.00	0.00
29	->	63	-0.00	0.00
29	->	64	-0.00	0.00
29	->	65	0.00	0.00
29	->	66	-0.00	0.00
29	->	67	0.00	0.00
29	->	68	0.00	0.00
29	->	69	0.00	0.00
29	->	70	0.00	0.00
29	->	71	0.00	0.00
29	->	72	0.00	0.00
29	->	73	0.00	0.00
29	->	74	-0.00	0.00
29	->	75	-0.00	0.00
29	->	76	0.00	0.00
29	->	77	0.00	0.00
29	->	78	0.00	0.00

29	->	79	0.00	0.00
29	->	80	0.00	0.00
29	->	81	0.00	0.00
29	->	82	0.00	0.00
29	->	83	0.00	0.00
29	->	84	-0.00	0.00
29	->	85	0.00	0.00
29	->	86	-0.00	0.00
29	->	87	-0.00	0.00
29	->	88	-0.00	0.00
29	->	89	-0.01	0.00
29	->	90	-0.01	0.00
29	->	91	-0.01	0.00
29	->	92	-0.00	0.00
29	->	93	-0.00	0.00
29	->	94	-0.00	0.00
29	->	95	-0.00	0.00
29	->	96	-0.00	0.00
29	->	97	-0.00	0.00
29	->	98	-0.00	0.00
29	->	99	-0.00	0.00
29	->	100	-0.00	0.00
29	->	101	-0.00	0.00
29	->	102	-0.00	0.00
29	->	103	-0.00	0.00
29	->	104	-0.00	0.00
29	->	105	-0.00	0.00
29	->	106	-0.00	0.00
30	->	1	-0.00	0.00
30	->	2	-0.00	0.00
30	->	3	-0.00	0.00
30	->	4	-0.00	0.00
30	->	5	-0.01	0.00
30	->	6	-0.00	0.00
30	->	7	-0.00	0.00
30	->	8	-0.00	0.00
30	->	9	-0.00	0.00
30	->	10	-0.00	0.00
30	->	11	-0.00	0.00
30	->	12	-0.00	0.00
30	->	13	0.00	0.00
30	->	14	-0.00	0.00
30	->	15	-0.00	0.00
30	->	16	0.00	0.00
30	->	17	-0.00	0.00
30	->	18	-0.00	0.00
30	->	19	-0.00	0.00
30	->	20	-0.00	0.00
30	->	21	-0.05	0.02
30	->	22	-0.03	0.01

30 ->	23	-0.03	0.01
30 ->	24	-0.42	0.19
30 ->	25	-5.04	1.91
30 ->	26	-0.53	0.28
30 ->	27	-0.04	0.03
30 ->	28	-3.83	1.29
30 ->	29	-14.67	0.44
30 ->	30	-52.75	2.13
30 ->	31	-11.00	0.27
30 ->	32	-1.23	0.27
30 ->	33	-0.12	0.03
30 ->	34	-0.01	0.00
30 ->	35	-0.00	0.00
30 ->	36	-0.00	0.00
30 ->	37	-0.00	0.00
30 ->	38	0.00	0.00
30 ->	39	-0.00	0.00
30 ->	40	-0.00	0.01
30 ->	41	-0.01	0.03
30 ->	42	-0.32	0.10
30 ->	43	-2.67	1.47
30 ->	44	0.00	0.01
30 ->	45	0.00	0.01
30 ->	46	0.00	0.00
30 ->	47	-0.00	0.00
30 ->	48	-0.00	0.00
30 ->	49	-0.00	0.00
30 ->	50	-0.00	0.00
30 ->	51	-0.00	0.00
30 ->	52	-0.00	0.00
30 ->	53	-0.00	0.00
30 ->	54	-0.00	0.00
30 ->	55	0.00	0.00
30 ->	56	-0.00	0.00
30 ->	57	-0.00	0.00
30 ->	58	0.00	0.00
30 ->	59	-0.00	0.00
30 ->	60	-0.00	0.00
30 ->	61	-0.00	0.00
30 ->	62	0.00	0.00
30 ->	63	0.00	0.00
30 ->	64	0.00	0.00
30 ->	65	0.00	0.00
30 ->	66	0.00	0.00
30 ->	67	0.00	0.00
30 ->	68	-0.00	0.00
30 ->	69	-0.00	0.00
30 ->	70	-0.00	0.00
30 ->	71	0.00	0.00
30 ->	72	-0.00	0.00

30	->	73	-0.00	0.00
30	->	74	0.00	0.00
30	->	75	0.00	0.00
30	->	76	-0.00	0.00
30	->	77	-0.00	0.00
30	->	78	-0.00	0.00
30	->	79	-0.00	0.00
30	->	80	0.00	0.00
30	->	81	-0.00	0.00
30	->	82	-0.00	0.00
30	->	83	-0.00	0.00
30	->	84	0.00	0.00
30	->	85	0.00	0.00
30	->	86	0.00	0.00
30	->	87	0.00	0.00
30	->	88	0.00	0.00
30	->	89	-0.00	0.00
30	->	90	-0.00	0.00
30	->	91	-0.00	0.00
30	->	92	0.00	0.00
30	->	93	-0.00	0.00
30	->	94	0.00	0.00
30	->	95	0.00	0.00
30	->	96	-0.00	0.00
30	->	97	-0.01	0.01
30	->	98	-0.00	0.04
30	->	99	0.00	0.00
30	->	100	0.00	0.00
30	->	101	0.00	0.00
30	->	102	0.00	0.00
30	->	103	0.00	0.00
30	->	104	0.00	0.00
30	->	105	0.00	0.00
30	->	106	0.00	0.00
31	->	1	0.02	0.00
31	->	2	-0.00	0.00
31	->	3	0.00	0.00
31	->	4	0.00	0.00
31	->	5	-0.03	0.00
31	->	6	0.00	0.00
31	->	7	-0.00	0.00
31	->	8	-0.01	0.00
31	->	9	0.00	0.00
31	->	10	-0.03	0.00
31	->	11	-0.01	0.00
31	->	12	0.02	0.01
31	->	13	0.01	0.00
31	->	14	-0.00	0.00
31	->	15	0.00	0.00
31	->	16	-0.01	0.00

31	->	17	-0.00	0.00
31	->	18	-0.00	0.00
31	->	19	-0.00	0.00
31	->	20	-0.00	0.00
31	->	21	-0.00	0.00
31	->	22	0.02	0.01
31	->	23	-0.01	0.00
31	->	24	-0.01	0.01
31	->	25	0.02	0.10
31	->	26	-0.30	0.34
31	->	27	-0.03	0.01
31	->	28	-0.04	0.01
31	->	29	-1.45	0.60
31	->	30	-11.03	0.27
31	->	31	9.32	2.33
31	->	32	-0.30	0.40
31	->	33	-0.89	0.28
31	->	34	-0.18	0.19
31	->	35	-0.09	0.02
31	->	36	0.02	0.01
31	->	37	0.00	0.00
31	->	38	-0.06	0.01
31	->	39	-0.02	0.02
31	->	40	0.10	0.14
31	->	41	-2.56	0.97
31	->	42	-3.17	0.44
31	->	43	-2.34	0.60
31	->	44	-0.01	0.05
31	->	45	-0.06	0.02
31	->	46	-0.07	0.01
31	->	47	0.00	0.01
31	->	48	-0.02	0.01
31	->	49	-0.00	0.00
31	->	50	-0.00	0.00
31	->	51	-0.00	0.00
31	->	52	-0.00	0.00
31	->	53	0.02	0.00
31	->	54	0.00	0.00
31	->	55	-0.01	0.00
31	->	56	0.00	0.00
31	->	57	0.00	0.00
31	->	58	-0.01	0.00
31	->	59	0.01	0.00
31	->	60	0.00	0.00
31	->	61	0.00	0.00
31	->	62	-0.00	0.00
31	->	63	-0.00	0.00
31	->	64	-0.00	0.00
31	->	65	-0.00	0.00
31	->	66	-0.00	0.00

31	->	67	-0.00	0.00
31	->	68	0.00	0.00
31	->	69	0.00	0.00
31	->	70	0.00	0.00
31	->	71	-0.00	0.00
31	->	72	0.02	0.01
31	->	73	0.00	0.00
31	->	74	-0.01	0.00
31	->	75	-0.01	0.00
31	->	76	0.01	0.00
31	->	77	0.01	0.00
31	->	78	0.01	0.00
31	->	79	0.00	0.00
31	->	80	0.00	0.00
31	->	81	0.00	0.00
31	->	82	0.00	0.00
31	->	83	0.00	0.00
31	->	84	-0.00	0.00
31	->	85	-0.00	0.00
31	->	86	-0.01	0.00
31	->	87	-0.03	0.01
31	->	88	-0.09	0.06
31	->	89	-0.32	0.46
31	->	90	-0.13	0.14
31	->	91	-0.04	0.01
31	->	92	-0.02	0.00
31	->	93	-0.01	0.00
31	->	94	-0.01	0.00
31	->	95	-0.00	0.00
31	->	96	-0.00	0.00
31	->	97	-0.02	0.01
31	->	98	-0.06	0.02
31	->	99	-0.02	0.01
31	->	100	-0.01	0.00
31	->	101	-0.01	0.00
31	->	102	-0.01	0.00
31	->	103	-0.01	0.00
31	->	104	-0.01	0.00
31	->	105	-0.01	0.00
31	->	106	-0.01	0.00
32	->	1	0.00	0.00
32	->	2	-0.00	0.00
32	->	3	0.00	0.00
32	->	4	-0.00	0.00
32	->	5	-0.00	0.00
32	->	6	-0.00	0.00
32	->	7	-0.00	0.00
32	->	8	-0.00	0.00
32	->	9	0.00	0.00
32	->	10	-0.00	0.00

32 ->	11	-0.00	0.00
32 ->	12	0.00	0.00
32 ->	13	0.00	0.00
32 ->	14	-0.00	0.00
32 ->	15	0.00	0.00
32 ->	16	-0.00	0.00
32 ->	17	-0.00	0.00
32 ->	18	-0.00	0.00
32 ->	19	-0.00	0.00
32 ->	20	-0.00	0.00
32 ->	21	-0.01	0.01
32 ->	22	-0.01	0.01
32 ->	23	-0.00	0.00
32 ->	24	-0.01	0.00
32 ->	25	-0.86	0.53
32 ->	26	-0.03	0.01
32 ->	27	-0.00	0.00
32 ->	28	-0.02	0.01
32 ->	29	-0.03	0.00
32 ->	30	-1.24	0.28
32 ->	31	-0.36	0.40
32 ->	32	1.64	0.59
32 ->	33	-10.56	0.51
32 ->	34	-0.79	0.42
32 ->	35	-0.02	0.01
32 ->	36	-0.02	0.01
32 ->	37	-0.00	0.00
32 ->	38	0.00	0.00
32 ->	39	-0.01	0.00
32 ->	40	-0.01	0.01
32 ->	41	-0.31	0.24
32 ->	42	-0.86	0.16
32 ->	43	-0.58	0.20
32 ->	44	-0.10	0.04
32 ->	45	-0.06	0.02
32 ->	46	-0.02	0.00
32 ->	47	-0.01	0.00
32 ->	48	-0.04	0.01
32 ->	49	-0.00	0.00
32 ->	50	-0.00	0.00
32 ->	51	-0.00	0.00
32 ->	52	-0.00	0.00
32 ->	53	-0.00	0.00
32 ->	54	-0.00	0.00
32 ->	55	-0.00	0.00
32 ->	56	-0.00	0.00
32 ->	57	0.00	0.00
32 ->	58	0.00	0.00
32 ->	59	-0.00	0.00
32 ->	60	-0.00	0.00

32	->	61	0.00	0.00
32	->	62	0.00	0.00
32	->	63	0.00	0.00
32	->	64	-0.00	0.00
32	->	65	0.00	0.00
32	->	66	0.00	0.00
32	->	67	0.00	0.00
32	->	68	0.00	0.00
32	->	69	-0.00	0.00
32	->	70	0.00	0.00
32	->	71	0.00	0.00
32	->	72	0.00	0.00
32	->	73	0.00	0.00
32	->	74	-0.00	0.00
32	->	75	-0.00	0.00
32	->	76	0.00	0.00
32	->	77	0.00	0.00
32	->	78	0.00	0.00
32	->	79	0.00	0.00
32	->	80	0.00	0.00
32	->	81	0.00	0.00
32	->	82	0.00	0.00
32	->	83	0.00	0.00
32	->	84	-0.00	0.00
32	->	85	0.00	0.00
32	->	86	0.00	0.00
32	->	87	-0.00	0.00
32	->	88	-0.00	0.00
32	->	89	-0.00	0.00
32	->	90	-0.01	0.00
32	->	91	-0.00	0.00
32	->	92	-0.00	0.00
32	->	93	-0.00	0.00
32	->	94	-0.00	0.00
32	->	95	-0.00	0.00
32	->	96	-0.00	0.00
32	->	97	-0.02	0.01
32	->	98	-0.10	0.08
32	->	99	-0.01	0.00
32	->	100	-0.00	0.00
32	->	101	-0.00	0.00
32	->	102	-0.00	0.00
32	->	103	-0.00	0.00
32	->	104	-0.00	0.00
32	->	105	-0.00	0.00
32	->	106	-0.00	0.00
33	->	1	0.00	0.00
33	->	2	0.00	0.00
33	->	3	-0.00	0.00
33	->	4	0.00	0.00

33	->	5	0.00	0.00
33	->	6	-0.00	0.00
33	->	7	-0.00	0.00
33	->	8	0.00	0.00
33	->	9	-0.00	0.00
33	->	10	0.00	0.00
33	->	11	0.00	0.00
33	->	12	-0.00	0.00
33	->	13	-0.00	0.00
33	->	14	-0.00	0.00
33	->	15	-0.00	0.00
33	->	16	-0.00	0.00
33	->	17	-0.00	0.00
33	->	18	-0.01	0.00
33	->	19	-0.00	0.00
33	->	20	-0.00	0.00
33	->	21	-0.02	0.02
33	->	22	-0.04	0.03
33	->	23	-0.00	0.00
33	->	24	-0.01	0.00
33	->	25	-0.83	0.48
33	->	26	-0.01	0.00
33	->	27	-0.00	0.00
33	->	28	-0.00	0.00
33	->	29	-0.01	0.00
33	->	30	-0.12	0.03
33	->	31	-0.90	0.28
33	->	32	-10.67	0.51
33	->	33	27.47	1.15
33	->	34	-13.09	0.52
33	->	35	-0.45	0.11
33	->	36	-0.02	0.03
33	->	37	-0.00	0.00
33	->	38	-0.01	0.01
33	->	39	-0.02	0.01
33	->	40	-0.24	0.09
33	->	41	-2.51	0.46
33	->	42	-3.77	0.49
33	->	43	-0.95	0.18
33	->	44	-2.06	0.55
33	->	45	-2.28	0.31
33	->	46	0.02	0.03
33	->	47	-0.06	0.03
33	->	48	-1.48	0.33
33	->	49	-0.04	0.01
33	->	50	-0.01	0.00
33	->	51	-0.02	0.01
33	->	52	-0.02	0.01
33	->	53	-0.00	0.00
33	->	54	-0.00	0.00

33	->	55	-0.01	0.00
33	->	56	-0.00	0.00
33	->	57	-0.00	0.00
33	->	58	-0.00	0.00
33	->	59	0.00	0.00
33	->	60	-0.00	0.00
33	->	61	-0.00	0.00
33	->	62	-0.00	0.00
33	->	63	-0.00	0.00
33	->	64	-0.00	0.00
33	->	65	0.00	0.00
33	->	66	-0.00	0.00
33	->	67	0.00	0.00
33	->	68	0.00	0.00
33	->	69	0.00	0.00
33	->	70	-0.00	0.00
33	->	71	-0.00	0.00
33	->	72	-0.00	0.00
33	->	73	-0.00	0.00
33	->	74	0.00	0.00
33	->	75	0.00	0.00
33	->	76	0.00	0.00
33	->	77	0.00	0.00
33	->	78	-0.00	0.00
33	->	79	-0.00	0.00
33	->	80	0.00	0.00
33	->	81	0.00	0.00
33	->	82	0.00	0.00
33	->	83	0.00	0.00
33	->	84	0.00	0.00
33	->	85	-0.00	0.00
33	->	86	-0.00	0.00
33	->	87	-0.00	0.00
33	->	88	-0.00	0.00
33	->	89	-0.01	0.00
33	->	90	-0.01	0.00
33	->	91	-0.00	0.00
33	->	92	-0.00	0.00
33	->	93	-0.00	0.00
33	->	94	-0.00	0.00
33	->	95	-0.00	0.00
33	->	96	-0.01	0.00
33	->	97	-0.08	0.07
33	->	98	-1.15	0.59
33	->	99	-0.06	0.04
33	->	100	-0.01	0.00
33	->	101	-0.00	0.00
33	->	102	-0.00	0.00
33	->	103	-0.00	0.00
33	->	104	-0.00	0.00

33	->	105	-0.00	0.00
33	->	106	0.00	0.00
34	->	1	0.00	0.00
34	->	2	0.00	0.00
34	->	3	-0.00	0.00
34	->	4	-0.00	0.00
34	->	5	-0.00	0.00
34	->	6	0.00	0.00
34	->	7	0.00	0.00
34	->	8	-0.00	0.00
34	->	9	0.00	0.00
34	->	10	-0.00	0.00
34	->	11	-0.00	0.00
34	->	12	0.00	0.00
34	->	13	0.00	0.00
34	->	14	0.00	0.00
34	->	15	0.00	0.00
34	->	16	-0.00	0.00
34	->	17	0.00	0.00
34	->	18	-0.00	0.00
34	->	19	-0.00	0.00
34	->	20	-0.00	0.00
34	->	21	-0.00	0.00
34	->	22	-0.00	0.00
34	->	23	-0.00	0.00
34	->	24	-0.00	0.00
34	->	25	-0.01	0.00
34	->	26	-0.00	0.00
34	->	27	-0.00	0.00
34	->	28	-0.00	0.00
34	->	29	-0.00	0.00
34	->	30	-0.01	0.00
34	->	31	-0.19	0.20
34	->	32	-0.83	0.43
34	->	33	-13.09	0.52
34	->	34	-55.93	2.49
34	->	35	-12.15	0.96
34	->	36	-1.19	1.52
34	->	37	-0.00	0.04
34	->	38	-0.13	0.41
34	->	39	-0.77	1.21
34	->	40	-0.70	0.80
34	->	41	-2.43	0.80
34	->	42	-0.58	0.27
34	->	43	-0.03	0.01
34	->	44	-0.03	0.01
34	->	45	-0.02	0.01
34	->	46	0.00	0.01
34	->	47	0.00	0.01
34	->	48	-1.32	0.31

34	->	49	-0.01	0.01
34	->	50	-0.00	0.00
34	->	51	-0.07	0.03
34	->	52	-0.08	0.04
34	->	53	-0.01	0.00
34	->	54	-0.00	0.00
34	->	55	-0.06	0.02
34	->	56	-0.00	0.00
34	->	57	-0.00	0.00
34	->	58	0.00	0.00
34	->	59	-0.00	0.00
34	->	60	-0.00	0.00
34	->	61	0.00	0.00
34	->	62	0.00	0.00
34	->	63	0.00	0.00
34	->	64	0.00	0.00
34	->	65	0.00	0.00
34	->	66	0.00	0.00
34	->	67	0.00	0.00
34	->	68	-0.00	0.00
34	->	69	-0.00	0.00
34	->	70	0.00	0.00
34	->	71	0.00	0.00
34	->	72	-0.00	0.00
34	->	73	-0.00	0.00
34	->	74	0.00	0.00
34	->	75	0.00	0.00
34	->	76	-0.00	0.00
34	->	77	-0.00	0.00
34	->	78	-0.00	0.00
34	->	79	-0.00	0.00
34	->	80	0.00	0.00
34	->	81	-0.00	0.00
34	->	82	-0.00	0.00
34	->	83	0.00	0.00
34	->	84	0.00	0.00
34	->	85	0.00	0.00
34	->	86	0.00	0.00
34	->	87	-0.00	0.00
34	->	88	-0.01	0.00
34	->	89	-0.01	0.00
34	->	90	-0.00	0.00
34	->	91	-0.00	0.00
34	->	92	-0.00	0.00
34	->	93	0.00	0.00
34	->	94	0.00	0.00
34	->	95	0.00	0.00
34	->	96	-0.00	0.00
34	->	97	-0.00	0.00
34	->	98	-0.00	0.00

34	->	99	-0.00	0.00
34	->	100	0.00	0.00
34	->	101	-0.00	0.00
34	->	102	0.00	0.00
34	->	103	0.00	0.00
34	->	104	-0.00	0.00
34	->	105	-0.00	0.00
34	->	106	-0.00	0.00
35	->	1	-0.01	0.00
35	->	2	-0.00	0.00
35	->	3	-0.00	0.00
35	->	4	-0.00	0.00
35	->	5	0.01	0.00
35	->	6	-0.00	0.00
35	->	7	0.00	0.00
35	->	8	0.00	0.00
35	->	9	-0.00	0.00
35	->	10	0.01	0.00
35	->	11	0.00	0.00
35	->	12	-0.01	0.00
35	->	13	-0.00	0.00
35	->	14	-0.00	0.00
35	->	15	-0.00	0.00
35	->	16	0.01	0.00
35	->	17	0.00	0.00
35	->	18	0.00	0.00
35	->	19	0.00	0.00
35	->	20	0.00	0.00
35	->	21	0.00	0.00
35	->	22	-0.02	0.00
35	->	23	-0.00	0.00
35	->	24	0.00	0.00
35	->	25	-0.03	0.00
35	->	26	-0.00	0.00
35	->	27	-0.00	0.00
35	->	28	0.00	0.00
35	->	29	0.00	0.00
35	->	30	-0.00	0.00
35	->	31	-0.09	0.02
35	->	32	-0.02	0.01
35	->	33	-0.45	0.11
35	->	34	-12.17	0.95
35	->	35	-26.47	2.72
35	->	36	-9.59	0.90
35	->	37	-5.68	1.64
35	->	38	1.56	0.92
35	->	39	-0.23	0.34
35	->	40	-1.15	0.90
35	->	41	-0.74	0.65
35	->	42	-0.42	0.29

35 ->	43	0.01	0.01
35 ->	44	-0.05	0.01
35 ->	45	-0.01	0.01
35 ->	46	0.04	0.01
35 ->	47	-0.09	0.02
35 ->	48	-1.11	0.18
35 ->	49	0.01	0.01
35 ->	50	-0.04	0.02
35 ->	51	1.56	0.28
35 ->	52	-0.34	0.44
35 ->	53	-0.05	0.02
35 ->	54	-0.07	0.05
35 ->	55	3.49	0.61
35 ->	56	-0.02	0.01
35 ->	57	-0.00	0.00
35 ->	58	0.03	0.01
35 ->	59	-0.02	0.00
35 ->	60	-0.00	0.00
35 ->	61	0.00	0.00
35 ->	62	0.00	0.00
35 ->	63	0.01	0.00
35 ->	64	0.00	0.00
35 ->	65	0.00	0.00
35 ->	66	0.00	0.00
35 ->	67	0.00	0.00
35 ->	68	-0.01	0.00
35 ->	69	-0.01	0.00
35 ->	70	-0.00	0.00
35 ->	71	-0.00	0.00
35 ->	72	-0.03	0.00
35 ->	73	-0.00	0.00
35 ->	74	0.02	0.00
35 ->	75	0.02	0.00
35 ->	76	-0.01	0.00
35 ->	77	-0.01	0.00
35 ->	78	-0.01	0.00
35 ->	79	-0.00	0.00
35 ->	80	-0.00	0.00
35 ->	81	-0.00	0.00
35 ->	82	-0.00	0.00
35 ->	83	0.00	0.00
35 ->	84	0.00	0.00
35 ->	85	0.00	0.00
35 ->	86	0.02	0.00
35 ->	87	0.06	0.02
35 ->	88	0.09	0.03
35 ->	89	0.04	0.01
35 ->	90	0.02	0.01
35 ->	91	0.01	0.00
35 ->	92	0.01	0.00

35 ->	93	0.00	0.00
35 ->	94	0.00	0.00
35 ->	95	0.00	0.00
35 ->	96	0.00	0.00
35 ->	97	0.01	0.00
35 ->	98	0.03	0.00
35 ->	99	0.02	0.00
35 ->	100	0.01	0.00
35 ->	101	0.01	0.00
35 ->	102	0.01	0.00
35 ->	103	0.01	0.00
35 ->	104	0.01	0.00
35 ->	105	0.00	0.00
35 ->	106	0.01	0.00
36 ->	1	0.00	0.00
36 ->	2	0.00	0.00
36 ->	3	-0.00	0.00
36 ->	4	-0.00	0.00
36 ->	5	-0.00	0.00
36 ->	6	0.00	0.00
36 ->	7	0.00	0.00
36 ->	8	-0.00	0.00
36 ->	9	0.00	0.00
36 ->	10	-0.00	0.00
36 ->	11	-0.00	0.00
36 ->	12	0.00	0.00
36 ->	13	0.00	0.00
36 ->	14	0.00	0.00
36 ->	15	0.00	0.00
36 ->	16	-0.00	0.00
36 ->	17	-0.00	0.00
36 ->	18	-0.00	0.00
36 ->	19	-0.00	0.00
36 ->	20	-0.00	0.00
36 ->	21	-0.00	0.00
36 ->	22	0.01	0.00
36 ->	23	0.00	0.00
36 ->	24	-0.00	0.00
36 ->	25	0.02	0.01
36 ->	26	-0.00	0.00
36 ->	27	0.00	0.00
36 ->	28	-0.00	0.00
36 ->	29	-0.00	0.00
36 ->	30	-0.00	0.00
36 ->	31	0.02	0.01
36 ->	32	-0.02	0.01
36 ->	33	-0.02	0.03
36 ->	34	-1.21	1.54
36 ->	35	-9.70	0.92
36 ->	36	-116.49	2.01

36	->	37	-13.32	0.69
36	->	38	-0.74	0.20
36	->	39	-0.25	0.47
36	->	40	-0.01	0.03
36	->	41	-0.03	0.02
36	->	42	-0.02	0.01
36	->	43	-0.00	0.00
36	->	44	0.01	0.00
36	->	45	-0.01	0.00
36	->	46	-0.03	0.01
36	->	47	-0.01	0.00
36	->	48	-0.11	0.08
36	->	49	-0.02	0.02
36	->	50	-0.01	0.00
36	->	51	-0.08	0.04
36	->	52	-0.68	1.04
36	->	53	0.02	0.01
36	->	54	-0.00	0.00
36	->	55	-0.31	0.15
36	->	56	-0.11	0.17
36	->	57	-0.00	0.00
36	->	58	-0.07	0.11
36	->	59	0.02	0.01
36	->	60	0.00	0.00
36	->	61	-0.00	0.00
36	->	62	-0.00	0.00
36	->	63	-0.00	0.00
36	->	64	-0.00	0.00
36	->	65	-0.00	0.00
36	->	66	-0.00	0.00
36	->	67	-0.00	0.00
36	->	68	0.01	0.00
36	->	69	0.01	0.01
36	->	70	-0.00	0.00
36	->	71	-0.00	0.00
36	->	72	0.01	0.00
36	->	73	0.00	0.00
36	->	74	-0.01	0.00
36	->	75	-0.01	0.01
36	->	76	0.01	0.00
36	->	77	0.00	0.00
36	->	78	0.01	0.00
36	->	79	0.00	0.00
36	->	80	0.00	0.00
36	->	81	0.00	0.00
36	->	82	0.00	0.00
36	->	83	-0.00	0.00
36	->	84	-0.00	0.00
36	->	85	-0.00	0.00
36	->	86	-0.01	0.00

36	->	87	-0.03	0.01
36	->	88	-0.03	0.01
36	->	89	-0.02	0.01
36	->	90	-0.01	0.00
36	->	91	-0.01	0.00
36	->	92	-0.00	0.00
36	->	93	-0.00	0.00
36	->	94	-0.00	0.00
36	->	95	-0.00	0.00
36	->	96	-0.00	0.00
36	->	97	-0.02	0.01
36	->	98	-0.03	0.01
36	->	99	-0.03	0.01
36	->	100	-0.02	0.01
36	->	101	-0.01	0.00
36	->	102	-0.00	0.00
36	->	103	-0.00	0.00
36	->	104	-0.00	0.00
36	->	105	-0.00	0.00
36	->	106	-0.00	0.00
37	->	1	0.00	0.00
37	->	2	0.00	0.00
37	->	3	0.00	0.00
37	->	4	0.00	0.00
37	->	5	-0.00	0.00
37	->	6	0.00	0.00
37	->	7	0.00	0.00
37	->	8	-0.00	0.00
37	->	9	0.00	0.00
37	->	10	-0.00	0.00
37	->	11	-0.00	0.00
37	->	12	0.00	0.00
37	->	13	0.00	0.00
37	->	14	0.00	0.00
37	->	15	0.00	0.00
37	->	16	-0.00	0.00
37	->	17	0.00	0.00
37	->	18	-0.00	0.00
37	->	19	-0.00	0.00
37	->	20	0.00	0.00
37	->	21	-0.00	0.00
37	->	22	0.00	0.00
37	->	23	0.00	0.00
37	->	24	-0.00	0.00
37	->	25	0.00	0.00
37	->	26	0.00	0.00
37	->	27	0.00	0.00
37	->	28	0.00	0.00
37	->	29	0.00	0.00
37	->	30	-0.00	0.00

37 ->	31	0.00	0.00
37 ->	32	-0.00	0.00
37 ->	33	-0.00	0.00
37 ->	34	-0.00	0.04
37 ->	35	-5.76	1.65
37 ->	36	-13.46	0.68
37 ->	37	18.44	1.89
37 ->	38	-14.56	0.77
37 ->	39	-0.38	0.14
37 ->	40	-0.03	0.03
37 ->	41	-0.02	0.01
37 ->	42	-0.03	0.01
37 ->	43	-0.00	0.00
37 ->	44	0.00	0.00
37 ->	45	-0.00	0.00
37 ->	46	-0.00	0.00
37 ->	47	0.00	0.00
37 ->	48	-0.03	0.01
37 ->	49	-0.01	0.00
37 ->	50	-0.00	0.00
37 ->	51	-0.42	0.12
37 ->	52	-0.28	0.31
37 ->	53	-0.01	0.01
37 ->	54	-0.01	0.01
37 ->	55	-3.62	0.74
37 ->	56	-0.25	0.28
37 ->	57	-0.01	0.00
37 ->	58	-0.01	0.00
37 ->	59	0.00	0.00
37 ->	60	-0.00	0.00
37 ->	61	-0.00	0.00
37 ->	62	-0.00	0.00
37 ->	63	-0.00	0.00
37 ->	64	-0.00	0.00
37 ->	65	-0.00	0.00
37 ->	66	-0.00	0.00
37 ->	67	-0.00	0.00
37 ->	68	0.00	0.00
37 ->	69	0.00	0.00
37 ->	70	-0.00	0.00
37 ->	71	-0.00	0.00
37 ->	72	0.00	0.00
37 ->	73	0.00	0.00
37 ->	74	-0.00	0.00
37 ->	75	-0.00	0.00
37 ->	76	0.00	0.00
37 ->	77	0.00	0.00
37 ->	78	0.00	0.00
37 ->	79	0.00	0.00
37 ->	80	0.00	0.00

37	->	81	0.00	0.00
37	->	82	0.00	0.00
37	->	83	0.00	0.00
37	->	84	-0.00	0.00
37	->	85	-0.00	0.00
37	->	86	-0.00	0.00
37	->	87	-0.01	0.01
37	->	88	-0.02	0.01
37	->	89	-0.00	0.00
37	->	90	-0.00	0.00
37	->	91	-0.00	0.00
37	->	92	-0.00	0.00
37	->	93	-0.00	0.00
37	->	94	-0.00	0.00
37	->	95	-0.00	0.00
37	->	96	-0.00	0.00
37	->	97	-0.00	0.00
37	->	98	-0.00	0.00
37	->	99	-0.00	0.00
37	->	100	-0.00	0.00
37	->	101	-0.00	0.00
37	->	102	-0.00	0.00
37	->	103	-0.00	0.00
37	->	104	-0.00	0.00
37	->	105	-0.00	0.00
37	->	106	-0.00	0.00
38	->	1	-0.01	0.00
38	->	2	-0.00	0.00
38	->	3	-0.00	0.00
38	->	4	-0.00	0.00
38	->	5	0.01	0.00
38	->	6	-0.00	0.00
38	->	7	0.00	0.00
38	->	8	0.00	0.00
38	->	9	-0.00	0.00
38	->	10	0.01	0.00
38	->	11	0.00	0.00
38	->	12	-0.01	0.00
38	->	13	-0.00	0.00
38	->	14	-0.00	0.00
38	->	15	-0.00	0.00
38	->	16	0.01	0.00
38	->	17	0.00	0.00
38	->	18	0.00	0.00
38	->	19	0.00	0.00
38	->	20	0.00	0.00
38	->	21	0.00	0.00
38	->	22	-0.01	0.00
38	->	23	-0.00	0.00
38	->	24	0.00	0.00

38	->	25	-0.02	0.00
38	->	26	-0.00	0.00
38	->	27	-0.00	0.00
38	->	28	0.00	0.00
38	->	29	0.00	0.00
38	->	30	0.00	0.00
38	->	31	-0.06	0.01
38	->	32	0.00	0.00
38	->	33	-0.01	0.01
38	->	34	-0.13	0.41
38	->	35	1.54	0.92
38	->	36	-0.75	0.20
38	->	37	-14.63	0.77
38	->	38	-35.40	3.30
38	->	39	-11.55	0.55
38	->	40	-2.22	2.01
38	->	41	0.02	0.04
38	->	42	-0.01	0.03
38	->	43	-0.00	0.00
38	->	44	-0.02	0.00
38	->	45	0.00	0.00
38	->	46	0.03	0.00
38	->	47	-0.01	0.01
38	->	48	-0.02	0.01
38	->	49	0.00	0.00
38	->	50	-0.01	0.00
38	->	51	1.71	0.32
38	->	52	0.03	0.01
38	->	53	-0.04	0.01
38	->	54	-0.05	0.03
38	->	55	2.60	0.81
38	->	56	-0.01	0.00
38	->	57	-0.00	0.00
38	->	58	0.03	0.00
38	->	59	-0.03	0.00
38	->	60	-0.00	0.00
38	->	61	0.00	0.00
38	->	62	0.00	0.00
38	->	63	0.01	0.00
38	->	64	0.00	0.00
38	->	65	0.00	0.00
38	->	66	0.01	0.00
38	->	67	0.00	0.00
38	->	68	-0.01	0.00
38	->	69	-0.01	0.00
38	->	70	-0.00	0.00
38	->	71	-0.00	0.00
38	->	72	-0.03	0.00
38	->	73	-0.00	0.00
38	->	74	0.02	0.00

38	->	75	0.02	0.00
38	->	76	-0.01	0.00
38	->	77	-0.01	0.00
38	->	78	-0.01	0.00
38	->	79	-0.00	0.00
38	->	80	-0.00	0.00
38	->	81	-0.01	0.00
38	->	82	-0.00	0.00
38	->	83	0.00	0.00
38	->	84	0.00	0.00
38	->	85	0.00	0.00
38	->	86	0.02	0.00
38	->	87	0.09	0.03
38	->	88	0.14	0.06
38	->	89	0.05	0.01
38	->	90	0.02	0.01
38	->	91	0.01	0.00
38	->	92	0.01	0.00
38	->	93	0.00	0.00
38	->	94	0.00	0.00
38	->	95	0.00	0.00
38	->	96	0.00	0.00
38	->	97	0.01	0.00
38	->	98	0.02	0.00
38	->	99	0.02	0.00
38	->	100	0.01	0.00
38	->	101	0.01	0.00
38	->	102	0.01	0.00
38	->	103	0.01	0.00
38	->	104	0.01	0.00
38	->	105	0.00	0.00
38	->	106	0.01	0.00
39	->	1	-0.00	0.00
39	->	2	0.00	0.00
39	->	3	0.00	0.00
39	->	4	0.00	0.00
39	->	5	0.00	0.00
39	->	6	0.00	0.00
39	->	7	0.00	0.00
39	->	8	0.00	0.00
39	->	9	0.00	0.00
39	->	10	0.00	0.00
39	->	11	0.00	0.00
39	->	12	-0.00	0.00
39	->	13	-0.00	0.00
39	->	14	0.00	0.00
39	->	15	-0.00	0.00
39	->	16	0.00	0.00
39	->	17	0.00	0.00
39	->	18	0.00	0.00

39	->	19	0.00	0.00
39	->	20	0.00	0.00
39	->	21	0.00	0.00
39	->	22	-0.00	0.00
39	->	23	0.00	0.00
39	->	24	0.00	0.00
39	->	25	-0.00	0.00
39	->	26	-0.00	0.00
39	->	27	-0.00	0.00
39	->	28	-0.00	0.00
39	->	29	-0.00	0.00
39	->	30	-0.00	0.00
39	->	31	-0.02	0.02
39	->	32	-0.01	0.00
39	->	33	-0.02	0.01
39	->	34	-0.77	1.21
39	->	35	-0.23	0.35
39	->	36	-0.25	0.47
39	->	37	-0.38	0.15
39	->	38	-11.64	0.53
39	->	39	10.16	1.55
39	->	40	-9.00	0.77
39	->	41	-0.86	0.49
39	->	42	-0.07	0.02
39	->	43	-0.01	0.00
39	->	44	-0.00	0.00
39	->	45	-0.00	0.00
39	->	46	-0.00	0.00
39	->	47	0.00	0.00
39	->	48	-0.01	0.00
39	->	49	-0.00	0.00
39	->	50	-0.00	0.00
39	->	51	-0.04	0.04
39	->	52	-0.01	0.00
39	->	53	-0.00	0.00
39	->	54	-0.00	0.00
39	->	55	0.01	0.03
39	->	56	-0.00	0.00
39	->	57	0.00	0.00
39	->	58	-0.00	0.00
39	->	59	0.00	0.00
39	->	60	-0.00	0.00
39	->	61	0.00	0.00
39	->	62	0.00	0.00
39	->	63	-0.00	0.00
39	->	64	-0.00	0.00
39	->	65	0.00	0.00
39	->	66	0.00	0.00
39	->	67	-0.00	0.00
39	->	68	-0.00	0.00

39	->	69	0.00	0.00
39	->	70	-0.00	0.00
39	->	71	0.00	0.00
39	->	72	-0.00	0.00
39	->	73	0.00	0.00
39	->	74	0.00	0.00
39	->	75	-0.00	0.00
39	->	76	-0.00	0.00
39	->	77	-0.00	0.00
39	->	78	-0.00	0.00
39	->	79	-0.00	0.00
39	->	80	0.00	0.00
39	->	81	-0.00	0.00
39	->	82	-0.00	0.00
39	->	83	0.00	0.00
39	->	84	0.00	0.00
39	->	85	0.00	0.00
39	->	86	-0.00	0.00
39	->	87	-0.00	0.00
39	->	88	-0.01	0.00
39	->	89	-0.00	0.00
39	->	90	-0.00	0.00
39	->	91	0.00	0.00
39	->	92	0.00	0.00
39	->	93	0.00	0.00
39	->	94	0.00	0.00
39	->	95	0.00	0.00
39	->	96	0.00	0.00
39	->	97	0.00	0.00
39	->	98	-0.00	0.00
39	->	99	-0.00	0.00
39	->	100	0.00	0.00
39	->	101	0.00	0.00
39	->	102	0.00	0.00
39	->	103	0.00	0.00
39	->	104	0.00	0.00
39	->	105	0.00	0.00
39	->	106	-0.00	0.00
40	->	1	0.01	0.00
40	->	2	-0.00	0.00
40	->	3	-0.00	0.00
40	->	4	0.00	0.00
40	->	5	-0.01	0.00
40	->	6	0.00	0.00
40	->	7	-0.00	0.00
40	->	8	-0.01	0.00
40	->	9	0.00	0.00
40	->	10	-0.02	0.01
40	->	11	-0.01	0.00
40	->	12	0.02	0.01

40	->	13	0.01	0.00
40	->	14	0.00	0.00
40	->	15	0.00	0.00
40	->	16	-0.01	0.00
40	->	17	-0.00	0.00
40	->	18	-0.00	0.00
40	->	19	-0.00	0.00
40	->	20	-0.00	0.00
40	->	21	-0.00	0.00
40	->	22	0.02	0.01
40	->	23	-0.00	0.00
40	->	24	-0.00	0.00
40	->	25	0.03	0.02
40	->	26	-0.02	0.04
40	->	27	-0.00	0.00
40	->	28	-0.00	0.00
40	->	29	-0.00	0.00
40	->	30	-0.00	0.01
40	->	31	0.10	0.14
40	->	32	-0.01	0.01
40	->	33	-0.24	0.09
40	->	34	-0.69	0.79
40	->	35	-1.14	0.91
40	->	36	-0.01	0.03
40	->	37	-0.03	0.03
40	->	38	-2.29	2.04
40	->	39	-9.08	0.75
40	->	40	-107.33	3.57
40	->	41	-15.91	1.04
40	->	42	-3.22	1.50
40	->	43	-0.17	0.18
40	->	44	0.03	0.01
40	->	45	-0.01	0.01
40	->	46	-0.05	0.01
40	->	47	-0.01	0.03
40	->	48	-0.12	0.04
40	->	49	-0.01	0.00
40	->	50	-0.00	0.01
40	->	51	-0.75	0.69
40	->	52	-0.03	0.02
40	->	53	0.02	0.01
40	->	54	-0.01	0.01
40	->	55	-0.17	0.15
40	->	56	0.00	0.00
40	->	57	0.00	0.00
40	->	58	-0.02	0.00
40	->	59	0.02	0.01
40	->	60	0.00	0.00
40	->	61	-0.00	0.00
40	->	62	-0.00	0.00

40	->	63	-0.01	0.00
40	->	64	-0.00	0.00
40	->	65	-0.00	0.00
40	->	66	-0.01	0.00
40	->	67	-0.00	0.00
40	->	68	0.01	0.00
40	->	69	0.01	0.00
40	->	70	0.00	0.00
40	->	71	-0.00	0.00
40	->	72	0.05	0.02
40	->	73	0.00	0.00
40	->	74	-0.03	0.01
40	->	75	-0.02	0.01
40	->	76	0.01	0.00
40	->	77	0.01	0.00
40	->	78	0.02	0.00
40	->	79	0.00	0.00
40	->	80	0.00	0.00
40	->	81	0.01	0.00
40	->	82	0.00	0.00
40	->	83	0.00	0.00
40	->	84	-0.00	0.00
40	->	85	-0.01	0.00
40	->	86	-0.03	0.01
40	->	87	-0.49	0.55
40	->	88	-3.97	3.69
40	->	89	-4.23	3.94
40	->	90	-0.12	0.12
40	->	91	-0.03	0.01
40	->	92	-0.01	0.00
40	->	93	-0.01	0.00
40	->	94	-0.00	0.00
40	->	95	-0.00	0.00
40	->	96	-0.00	0.00
40	->	97	-0.01	0.00
40	->	98	-0.02	0.01
40	->	99	-0.02	0.01
40	->	100	-0.01	0.00
40	->	101	-0.01	0.00
40	->	102	-0.01	0.00
40	->	103	-0.01	0.00
40	->	104	-0.02	0.00
40	->	105	-0.01	0.00
40	->	106	-0.02	0.00
41	->	1	0.00	0.00
41	->	2	0.00	0.00
41	->	3	0.00	0.00
41	->	4	-0.00	0.00
41	->	5	-0.00	0.00
41	->	6	0.00	0.00

41	->	7	0.00	0.00
41	->	8	-0.00	0.00
41	->	9	-0.00	0.00
41	->	10	0.00	0.00
41	->	11	0.00	0.00
41	->	12	-0.00	0.00
41	->	13	0.00	0.00
41	->	14	0.00	0.00
41	->	15	-0.00	0.00
41	->	16	-0.00	0.00
41	->	17	0.00	0.00
41	->	18	0.00	0.00
41	->	19	0.00	0.00
41	->	20	0.00	0.00
41	->	21	0.00	0.00
41	->	22	0.01	0.00
41	->	23	-0.00	0.00
41	->	24	0.00	0.00
41	->	25	0.03	0.01
41	->	26	-0.02	0.01
41	->	27	-0.01	0.00
41	->	28	-0.00	0.00
41	->	29	-0.00	0.01
41	->	30	-0.01	0.03
41	->	31	-2.53	0.94
41	->	32	-0.31	0.25
41	->	33	-2.52	0.46
41	->	34	-2.40	0.78
41	->	35	-0.74	0.64
41	->	36	-0.03	0.02
41	->	37	-0.02	0.01
41	->	38	0.02	0.04
41	->	39	-0.88	0.49
41	->	40	-15.92	1.04
41	->	41	-30.86	2.03
41	->	42	-8.63	0.54
41	->	43	-0.55	0.07
41	->	44	0.01	0.04
41	->	45	-0.04	0.01
41	->	46	-0.03	0.01
41	->	47	0.06	0.03
41	->	48	-0.10	0.04
41	->	49	-0.01	0.00
41	->	50	-0.00	0.01
41	->	51	-0.03	0.02
41	->	52	-0.01	0.01
41	->	53	-0.00	0.00
41	->	54	-0.00	0.00
41	->	55	-0.01	0.01
41	->	56	-0.00	0.00

41	->	57	-0.00	0.00
41	->	58	0.00	0.00
41	->	59	-0.00	0.00
41	->	60	-0.00	0.00
41	->	61	0.00	0.00
41	->	62	0.00	0.00
41	->	63	0.00	0.00
41	->	64	0.00	0.00
41	->	65	0.00	0.00
41	->	66	0.00	0.00
41	->	67	0.00	0.00
41	->	68	-0.00	0.00
41	->	69	-0.00	0.00
41	->	70	-0.00	0.00
41	->	71	0.00	0.00
41	->	72	-0.00	0.00
41	->	73	-0.00	0.00
41	->	74	0.00	0.00
41	->	75	0.00	0.00
41	->	76	-0.00	0.00
41	->	77	-0.00	0.00
41	->	78	-0.00	0.00
41	->	79	-0.00	0.00
41	->	80	0.00	0.00
41	->	81	-0.00	0.00
41	->	82	-0.00	0.00
41	->	83	0.00	0.00
41	->	84	0.00	0.00
41	->	85	0.00	0.00
41	->	86	0.00	0.00
41	->	87	-0.00	0.00
41	->	88	-0.02	0.01
41	->	89	-0.04	0.03
41	->	90	-0.01	0.01
41	->	91	-0.00	0.00
41	->	92	-0.00	0.00
41	->	93	-0.00	0.00
41	->	94	-0.00	0.00
41	->	95	-0.00	0.00
41	->	96	-0.00	0.00
41	->	97	-0.00	0.00
41	->	98	-0.01	0.01
41	->	99	-0.00	0.00
41	->	100	-0.00	0.00
41	->	101	-0.00	0.00
41	->	102	0.00	0.00
41	->	103	-0.00	0.00
41	->	104	0.00	0.00
41	->	105	-0.00	0.00
41	->	106	0.00	0.00

42	->	1	0.00	0.00
42	->	2	0.00	0.00
42	->	3	0.00	0.00
42	->	4	0.00	0.00
42	->	5	-0.00	0.00
42	->	6	-0.00	0.00
42	->	7	-0.00	0.00
42	->	8	-0.00	0.00
42	->	9	-0.00	0.00
42	->	10	-0.00	0.00
42	->	11	-0.00	0.00
42	->	12	0.00	0.00
42	->	13	0.00	0.00
42	->	14	-0.00	0.00
42	->	15	0.00	0.00
42	->	16	-0.00	0.00
42	->	17	0.00	0.00
42	->	18	0.00	0.00
42	->	19	0.00	0.00
42	->	20	-0.00	0.00
42	->	21	0.00	0.00
42	->	22	0.01	0.01
42	->	23	-0.01	0.01
42	->	24	-0.00	0.00
42	->	25	0.03	0.04
42	->	26	-0.03	0.02
42	->	27	-0.01	0.01
42	->	28	-0.01	0.00
42	->	29	0.01	0.01
42	->	30	-0.32	0.10
42	->	31	-3.15	0.44
42	->	32	-0.86	0.16
42	->	33	-3.78	0.49
42	->	34	-0.59	0.27
42	->	35	-0.41	0.29
42	->	36	-0.02	0.01
42	->	37	-0.03	0.01
42	->	38	-0.01	0.03
42	->	39	-0.07	0.02
42	->	40	-3.19	1.47
42	->	41	-8.70	0.54
42	->	42	28.57	1.15
42	->	43	-17.15	0.34
42	->	44	-1.18	0.27
42	->	45	-0.26	0.06
42	->	46	-0.14	0.03
42	->	47	-1.72	0.67
42	->	48	-2.76	0.49
42	->	49	-0.06	0.02
42	->	50	-0.09	0.04

42 ->	51	-1.85	0.44
42 ->	52	-0.06	0.02
42 ->	53	-0.01	0.01
42 ->	54	-0.02	0.01
42 ->	55	-0.00	0.01
42 ->	56	-0.00	0.00
42 ->	57	-0.00	0.00
42 ->	58	-0.00	0.00
42 ->	59	-0.00	0.00
42 ->	60	-0.00	0.00
42 ->	61	-0.00	0.00
42 ->	62	0.00	0.00
42 ->	63	-0.00	0.00
42 ->	64	-0.00	0.00
42 ->	65	-0.00	0.00
42 ->	66	-0.00	0.00
42 ->	67	0.00	0.00
42 ->	68	0.00	0.00
42 ->	69	0.00	0.00
42 ->	70	-0.00	0.00
42 ->	71	-0.00	0.00
42 ->	72	-0.01	0.00
42 ->	73	-0.00	0.00
42 ->	74	-0.00	0.00
42 ->	75	-0.00	0.00
42 ->	76	0.00	0.00
42 ->	77	0.00	0.00
42 ->	78	0.00	0.00
42 ->	79	0.00	0.00
42 ->	80	0.00	0.00
42 ->	81	0.00	0.00
42 ->	82	0.00	0.00
42 ->	83	0.00	0.00
42 ->	84	-0.00	0.00
42 ->	85	-0.00	0.00
42 ->	86	-0.00	0.00
42 ->	87	0.00	0.01
42 ->	88	-0.17	0.12
42 ->	89	-0.23	0.16
42 ->	90	-0.06	0.05
42 ->	91	-0.02	0.01
42 ->	92	-0.01	0.00
42 ->	93	-0.00	0.00
42 ->	94	-0.00	0.00
42 ->	95	-0.00	0.00
42 ->	96	-0.00	0.00
42 ->	97	-0.01	0.00
42 ->	98	-0.02	0.01
42 ->	99	-0.01	0.01
42 ->	100	-0.00	0.00

42	->	101	-0.00	0.00
42	->	102	-0.00	0.00
42	->	103	-0.00	0.00
42	->	104	-0.00	0.00
42	->	105	-0.00	0.00
42	->	106	-0.00	0.00
43	->	1	-0.00	0.00
43	->	2	0.00	0.00
43	->	3	-0.00	0.00
43	->	4	0.00	0.00
43	->	5	-0.00	0.00
43	->	6	-0.00	0.00
43	->	7	-0.00	0.00
43	->	8	-0.00	0.00
43	->	9	-0.00	0.00
43	->	10	-0.00	0.00
43	->	11	-0.00	0.00
43	->	12	-0.00	0.00
43	->	13	-0.00	0.00
43	->	14	-0.00	0.00
43	->	15	-0.00	0.00
43	->	16	0.00	0.00
43	->	17	0.00	0.00
43	->	18	-0.00	0.00
43	->	19	0.00	0.00
43	->	20	-0.01	0.01
43	->	21	-0.04	0.05
43	->	22	-0.30	0.32
43	->	23	-0.04	0.01
43	->	24	-0.02	0.01
43	->	25	-2.13	1.36
43	->	26	-1.65	0.54
43	->	27	-0.00	0.01
43	->	28	-0.04	0.02
43	->	29	-0.15	0.15
43	->	30	-2.68	1.46
43	->	31	-2.29	0.59
43	->	32	-0.57	0.20
43	->	33	-0.94	0.17
43	->	34	-0.03	0.01
43	->	35	0.01	0.01
43	->	36	-0.00	0.00
43	->	37	-0.00	0.00
43	->	38	-0.00	0.00
43	->	39	-0.01	0.00
43	->	40	-0.17	0.18
43	->	41	-0.55	0.07
43	->	42	-17.14	0.34
43	->	43	23.61	2.91
43	->	44	-6.45	0.60

43	->	45	-0.31	0.08
43	->	46	-0.17	0.03
43	->	47	-0.13	0.10
43	->	48	-0.04	0.01
43	->	49	-0.00	0.00
43	->	50	-0.01	0.00
43	->	51	-0.01	0.00
43	->	52	-0.00	0.00
43	->	53	-0.00	0.00
43	->	54	-0.00	0.00
43	->	55	0.00	0.00
43	->	56	0.00	0.00
43	->	57	0.00	0.00
43	->	58	-0.00	0.00
43	->	59	0.00	0.00
43	->	60	0.00	0.00
43	->	61	0.00	0.00
43	->	62	0.00	0.00
43	->	63	-0.00	0.00
43	->	64	0.00	0.00
43	->	65	-0.00	0.00
43	->	66	-0.00	0.00
43	->	67	-0.00	0.00
43	->	68	0.00	0.00
43	->	69	0.00	0.00
43	->	70	0.00	0.00
43	->	71	0.00	0.00
43	->	72	0.00	0.00
43	->	73	0.00	0.00
43	->	74	-0.00	0.00
43	->	75	-0.00	0.00
43	->	76	0.00	0.00
43	->	77	0.00	0.00
43	->	78	0.00	0.00
43	->	79	0.00	0.00
43	->	80	0.00	0.00
43	->	81	0.00	0.00
43	->	82	0.00	0.00
43	->	83	0.00	0.00
43	->	84	-0.00	0.00
43	->	85	-0.00	0.00
43	->	86	-0.00	0.00
43	->	87	-0.00	0.00
43	->	88	-0.00	0.01
43	->	89	-0.02	0.03
43	->	90	-0.08	0.09
43	->	91	-0.03	0.02
43	->	92	-0.01	0.00
43	->	93	0.00	0.00
43	->	94	-0.00	0.00

43	->	95	-0.00	0.00
43	->	96	-0.00	0.00
43	->	97	-0.01	0.01
43	->	98	-0.03	0.02
43	->	99	-0.02	0.02
43	->	100	0.00	0.00
43	->	101	-0.00	0.00
43	->	102	-0.00	0.00
43	->	103	-0.00	0.00
43	->	104	-0.00	0.00
43	->	105	-0.00	0.00
43	->	106	-0.00	0.00
44	->	1	0.05	0.01
44	->	2	0.00	0.00
44	->	3	-0.00	0.00
44	->	4	-0.00	0.00
44	->	5	-0.03	0.00
44	->	6	0.00	0.00
44	->	7	-0.00	0.00
44	->	8	-0.01	0.00
44	->	9	-0.00	0.00
44	->	10	-0.03	0.00
44	->	11	-0.02	0.01
44	->	12	0.05	0.02
44	->	13	0.02	0.00
44	->	14	-0.01	0.01
44	->	15	-0.00	0.01
44	->	16	-0.06	0.01
44	->	17	-0.00	0.00
44	->	18	0.01	0.01
44	->	19	0.00	0.04
44	->	20	-0.02	0.01
44	->	21	0.03	0.03
44	->	22	-0.70	0.99
44	->	23	-0.09	0.04
44	->	24	-0.02	0.01
44	->	25	-0.14	0.18
44	->	26	-0.53	0.39
44	->	27	-0.01	0.01
44	->	28	-0.00	0.00
44	->	29	-0.01	0.00
44	->	30	0.00	0.01
44	->	31	-0.01	0.05
44	->	32	-0.10	0.04
44	->	33	-2.07	0.55
44	->	34	-0.03	0.01
44	->	35	-0.05	0.01
44	->	36	0.01	0.00
44	->	37	0.00	0.00
44	->	38	-0.02	0.00

44 ->	39	-0.00	0.00
44 ->	40	0.03	0.01
44 ->	41	0.01	0.04
44 ->	42	-1.19	0.26
44 ->	43	-6.55	0.60
44 ->	44	-93.45	1.86
44 ->	45	-21.62	0.40
44 ->	46	-15.91	1.30
44 ->	47	-5.06	0.75
44 ->	48	-1.09	0.36
44 ->	49	0.01	0.05
44 ->	50	-0.07	0.03
44 ->	51	-0.01	0.01
44 ->	52	-0.00	0.01
44 ->	53	0.04	0.01
44 ->	54	-0.00	0.00
44 ->	55	-0.01	0.00
44 ->	56	0.00	0.00
44 ->	57	0.00	0.00
44 ->	58	-0.02	0.00
44 ->	59	0.02	0.00
44 ->	60	0.00	0.00
44 ->	61	-0.00	0.00
44 ->	62	-0.00	0.00
44 ->	63	-0.01	0.00
44 ->	64	-0.01	0.00
44 ->	65	-0.00	0.00
44 ->	66	-0.01	0.00
44 ->	67	-0.00	0.00
44 ->	68	0.01	0.00
44 ->	69	0.01	0.00
44 ->	70	0.00	0.00
44 ->	71	0.00	0.00
44 ->	72	0.09	0.02
44 ->	73	-0.00	0.01
44 ->	74	-0.08	0.02
44 ->	75	-0.03	0.00
44 ->	76	0.02	0.01
44 ->	77	0.03	0.00
44 ->	78	0.02	0.00
44 ->	79	0.00	0.00
44 ->	80	0.00	0.00
44 ->	81	0.02	0.00
44 ->	82	0.00	0.00
44 ->	83	-0.00	0.00
44 ->	84	-0.00	0.00
44 ->	85	-0.00	0.00
44 ->	86	-0.02	0.00
44 ->	87	-0.04	0.01
44 ->	88	-0.08	0.01

44	->	89	-0.28	0.16
44	->	90	-2.33	0.63
44	->	91	-9.12	0.76
44	->	92	-1.47	0.60
44	->	93	-0.04	0.05
44	->	94	-0.04	0.01
44	->	95	-0.02	0.00
44	->	96	-0.01	0.00
44	->	97	-0.07	0.02
44	->	98	-0.26	0.16
44	->	99	-0.71	0.47
44	->	100	0.93	0.44
44	->	101	0.29	0.32
44	->	102	-0.10	0.07
44	->	103	-0.07	0.03
44	->	104	-0.04	0.01
44	->	105	-0.02	0.00
44	->	106	-0.01	0.00
45	->	1	0.00	0.00
45	->	2	0.00	0.00
45	->	3	-0.00	0.00
45	->	4	0.00	0.00
45	->	5	-0.00	0.00
45	->	6	0.00	0.00
45	->	7	0.00	0.00
45	->	8	-0.00	0.00
45	->	9	0.00	0.00
45	->	10	-0.00	0.00
45	->	11	-0.00	0.00
45	->	12	-0.00	0.00
45	->	13	0.00	0.00
45	->	14	-0.00	0.00
45	->	15	-0.00	0.00
45	->	16	-0.00	0.00
45	->	17	-0.00	0.00
45	->	18	-0.01	0.01
45	->	19	-0.01	0.00
45	->	20	-0.00	0.00
45	->	21	-0.01	0.01
45	->	22	-0.10	0.05
45	->	23	0.00	0.00
45	->	24	-0.00	0.00
45	->	25	-0.15	0.08
45	->	26	-0.00	0.00
45	->	27	0.00	0.00
45	->	28	-0.00	0.00
45	->	29	-0.00	0.00
45	->	30	0.00	0.01
45	->	31	-0.06	0.02
45	->	32	-0.06	0.02

45	->	33	-2.32	0.32
45	->	34	-0.02	0.01
45	->	35	-0.01	0.01
45	->	36	-0.01	0.00
45	->	37	-0.00	0.00
45	->	38	0.00	0.00
45	->	39	-0.00	0.00
45	->	40	-0.01	0.01
45	->	41	-0.04	0.01
45	->	42	-0.26	0.06
45	->	43	-0.31	0.08
45	->	44	-21.63	0.40
45	->	45	23.26	1.98
45	->	46	-10.46	0.62
45	->	47	-0.94	0.17
45	->	48	-2.21	0.36
45	->	49	-2.85	1.41
45	->	50	-0.08	0.04
45	->	51	-0.04	0.02
45	->	52	-0.06	0.03
45	->	53	-0.02	0.01
45	->	54	-0.00	0.00
45	->	55	-0.00	0.00
45	->	56	-0.00	0.00
45	->	57	-0.00	0.00
45	->	58	0.00	0.00
45	->	59	-0.00	0.00
45	->	60	-0.00	0.00
45	->	61	-0.00	0.00
45	->	62	0.00	0.00
45	->	63	0.00	0.00
45	->	64	0.00	0.00
45	->	65	0.00	0.00
45	->	66	0.00	0.00
45	->	67	0.00	0.00
45	->	68	-0.00	0.00
45	->	69	-0.00	0.00
45	->	70	0.00	0.00
45	->	71	0.00	0.00
45	->	72	-0.00	0.00
45	->	73	-0.00	0.00
45	->	74	-0.00	0.00
45	->	75	0.00	0.00
45	->	76	-0.00	0.00
45	->	77	-0.00	0.00
45	->	78	-0.00	0.00
45	->	79	-0.00	0.00
45	->	80	-0.00	0.00
45	->	81	-0.00	0.00
45	->	82	-0.00	0.00

45	->	83	0.00	0.00
45	->	84	0.00	0.00
45	->	85	0.00	0.00
45	->	86	0.00	0.00
45	->	87	-0.00	0.00
45	->	88	-0.00	0.00
45	->	89	-0.00	0.00
45	->	90	-0.00	0.00
45	->	91	-0.00	0.01
45	->	92	-0.00	0.00
45	->	93	-0.01	0.00
45	->	94	-0.00	0.00
45	->	95	-0.00	0.00
45	->	96	-0.01	0.00
45	->	97	-0.05	0.08
45	->	98	-1.70	0.51
45	->	99	-2.36	2.15
45	->	100	-0.05	0.02
45	->	101	-0.01	0.00
45	->	102	-0.00	0.00
45	->	103	-0.00	0.00
45	->	104	-0.00	0.00
45	->	105	-0.00	0.00
45	->	106	0.00	0.00
46	->	1	-0.03	0.00
46	->	2	-0.00	0.00
46	->	3	-0.00	0.00
46	->	4	-0.00	0.00
46	->	5	0.02	0.00
46	->	6	-0.00	0.00
46	->	7	-0.00	0.00
46	->	8	0.01	0.00
46	->	9	-0.00	0.00
46	->	10	0.02	0.00
46	->	11	0.01	0.00
46	->	12	-0.03	0.01
46	->	13	-0.01	0.00
46	->	14	-0.00	0.00
46	->	15	-0.00	0.00
46	->	16	0.04	0.01
46	->	17	0.00	0.00
46	->	18	-0.02	0.01
46	->	19	-0.01	0.01
46	->	20	-0.00	0.00
46	->	21	-0.02	0.01
46	->	22	-1.10	0.47
46	->	23	0.00	0.01
46	->	24	-0.00	0.00
46	->	25	-0.13	0.04
46	->	26	-0.01	0.00

46	->	27	-0.00	0.00
46	->	28	-0.00	0.00
46	->	29	-0.00	0.00
46	->	30	0.00	0.00
46	->	31	-0.07	0.01
46	->	32	-0.02	0.00
46	->	33	0.02	0.03
46	->	34	0.00	0.01
46	->	35	0.04	0.01
46	->	36	-0.03	0.01
46	->	37	-0.00	0.00
46	->	38	0.03	0.00
46	->	39	-0.00	0.00
46	->	40	-0.05	0.01
46	->	41	-0.03	0.01
46	->	42	-0.14	0.03
46	->	43	-0.17	0.03
46	->	44	-15.85	1.31
46	->	45	-10.52	0.62
46	->	46	-10.44	2.32
46	->	47	-13.32	0.70
46	->	48	-1.44	0.26
46	->	49	-2.78	0.44
46	->	50	-1.57	0.76
46	->	51	-0.14	0.08
46	->	52	-0.07	0.04
46	->	53	-0.16	0.05
46	->	54	-0.01	0.00
46	->	55	-0.01	0.00
46	->	56	-0.01	0.00
46	->	57	-0.00	0.00
46	->	58	0.03	0.01
46	->	59	-0.03	0.00
46	->	60	-0.01	0.00
46	->	61	-0.01	0.01
46	->	62	0.00	0.00
46	->	63	0.01	0.00
46	->	64	0.01	0.00
46	->	65	0.00	0.00
46	->	66	0.01	0.00
46	->	67	0.00	0.00
46	->	68	-0.01	0.00
46	->	69	-0.02	0.00
46	->	70	-0.00	0.00
46	->	71	-0.01	0.00
46	->	72	-0.11	0.03
46	->	73	-0.01	0.00
46	->	74	0.06	0.01
46	->	75	0.03	0.00
46	->	76	-0.03	0.01

46	->	77	-0.03	0.00
46	->	78	-0.03	0.00
46	->	79	-0.00	0.00
46	->	80	-0.00	0.00
46	->	81	-0.02	0.00
46	->	82	-0.00	0.00
46	->	83	0.00	0.00
46	->	84	0.00	0.00
46	->	85	0.00	0.00
46	->	86	0.02	0.00
46	->	87	0.02	0.00
46	->	88	0.03	0.01
46	->	89	-0.03	0.04
46	->	90	0.11	0.06
46	->	91	0.64	0.20
46	->	92	0.23	0.12
46	->	93	-0.02	0.04
46	->	94	0.03	0.01
46	->	95	0.02	0.00
46	->	96	0.00	0.00
46	->	97	0.02	0.01
46	->	98	-0.16	0.11
46	->	99	-1.96	0.74
46	->	100	-5.13	1.18
46	->	101	-1.03	0.73
46	->	102	-0.04	0.05
46	->	103	0.01	0.01
46	->	104	0.02	0.00
46	->	105	0.01	0.00
46	->	106	0.01	0.00
47	->	1	0.00	0.00
47	->	2	0.00	0.00
47	->	3	-0.00	0.00
47	->	4	0.00	0.00
47	->	5	-0.00	0.00
47	->	6	0.00	0.00
47	->	7	-0.00	0.00
47	->	8	-0.00	0.00
47	->	9	-0.00	0.00
47	->	10	-0.00	0.00
47	->	11	-0.00	0.00
47	->	12	0.00	0.00
47	->	13	0.00	0.00
47	->	14	-0.00	0.00
47	->	15	-0.00	0.00
47	->	16	-0.00	0.00
47	->	17	-0.00	0.00
47	->	18	-0.01	0.00
47	->	19	-0.01	0.00
47	->	20	-0.00	0.00

47	->	21	-0.01	0.00
47	->	22	-0.13	0.04
47	->	23	-0.00	0.00
47	->	24	-0.00	0.00
47	->	25	-0.01	0.01
47	->	26	-0.04	0.04
47	->	27	-0.00	0.00
47	->	28	-0.00	0.00
47	->	29	-0.00	0.00
47	->	30	-0.00	0.00
47	->	31	0.00	0.01
47	->	32	-0.01	0.00
47	->	33	-0.06	0.03
47	->	34	0.00	0.01
47	->	35	-0.09	0.02
47	->	36	-0.01	0.00
47	->	37	0.00	0.00
47	->	38	-0.01	0.01
47	->	39	0.00	0.00
47	->	40	-0.01	0.03
47	->	41	0.06	0.03
47	->	42	-1.76	0.67
47	->	43	-0.13	0.10
47	->	44	-5.00	0.74
47	->	45	-0.94	0.17
47	->	46	-13.39	0.69
47	->	47	20.84	1.45
47	->	48	-6.30	0.51
47	->	49	-0.94	0.17
47	->	50	-1.72	0.40
47	->	51	-2.08	0.55
47	->	52	-0.04	0.04
47	->	53	-0.03	0.02
47	->	54	-0.02	0.01
47	->	55	-0.04	0.01
47	->	56	-0.00	0.00
47	->	57	-0.00	0.00
47	->	58	0.00	0.00
47	->	59	-0.01	0.00
47	->	60	-0.00	0.00
47	->	61	-0.00	0.00
47	->	62	0.00	0.00
47	->	63	0.00	0.00
47	->	64	0.00	0.00
47	->	65	0.00	0.00
47	->	66	0.00	0.00
47	->	67	0.00	0.00
47	->	68	-0.00	0.00
47	->	69	-0.00	0.00
47	->	70	-0.00	0.00

47	->	71	-0.01	0.00
47	->	72	-0.12	0.05
47	->	73	-0.00	0.00
47	->	74	-0.01	0.02
47	->	75	-0.00	0.00
47	->	76	-0.00	0.00
47	->	77	-0.00	0.00
47	->	78	-0.00	0.00
47	->	79	-0.00	0.00
47	->	80	0.00	0.00
47	->	81	0.00	0.00
47	->	82	0.00	0.00
47	->	83	0.00	0.00
47	->	84	0.00	0.00
47	->	85	-0.00	0.00
47	->	86	-0.01	0.00
47	->	87	-0.04	0.01
47	->	88	-0.22	0.06
47	->	89	-2.63	0.90
47	->	90	-3.34	0.72
47	->	91	-0.56	0.36
47	->	92	-0.04	0.02
47	->	93	-0.01	0.00
47	->	94	-0.00	0.00
47	->	95	-0.00	0.00
47	->	96	-0.00	0.00
47	->	97	-0.01	0.00
47	->	98	-0.03	0.01
47	->	99	-0.08	0.06
47	->	100	-0.22	0.11
47	->	101	-0.28	0.20
47	->	102	-0.09	0.07
47	->	103	-0.09	0.05
47	->	104	-0.02	0.00
47	->	105	-0.01	0.00
47	->	106	-0.00	0.00
48	->	1	0.00	0.00
48	->	2	0.00	0.00
48	->	3	0.00	0.00
48	->	4	0.00	0.00
48	->	5	-0.00	0.00
48	->	6	0.00	0.00
48	->	7	0.00	0.00
48	->	8	-0.00	0.00
48	->	9	0.00	0.00
48	->	10	-0.00	0.00
48	->	11	-0.00	0.00
48	->	12	0.00	0.00
48	->	13	0.00	0.00
48	->	14	0.00	0.00

48	->	15	0.00	0.00
48	->	16	-0.00	0.00
48	->	17	-0.00	0.00
48	->	18	-0.00	0.00
48	->	19	-0.00	0.00
48	->	20	-0.00	0.00
48	->	21	-0.00	0.00
48	->	22	-0.01	0.01
48	->	23	-0.00	0.00
48	->	24	-0.00	0.00
48	->	25	-0.01	0.00
48	->	26	-0.00	0.00
48	->	27	-0.00	0.00
48	->	28	-0.00	0.00
48	->	29	-0.00	0.00
48	->	30	-0.00	0.00
48	->	31	-0.02	0.01
48	->	32	-0.04	0.01
48	->	33	-1.47	0.34
48	->	34	-1.34	0.32
48	->	35	-1.11	0.19
48	->	36	-0.11	0.08
48	->	37	-0.03	0.01
48	->	38	-0.02	0.01
48	->	39	-0.01	0.00
48	->	40	-0.12	0.04
48	->	41	-0.10	0.04
48	->	42	-2.83	0.51
48	->	43	-0.04	0.01
48	->	44	-1.11	0.36
48	->	45	-2.23	0.36
48	->	46	-1.44	0.26
48	->	47	-6.35	0.51
48	->	48	22.49	1.10
48	->	49	-6.16	0.49
48	->	50	-0.93	0.18
48	->	51	-2.86	0.33
48	->	52	-4.19	0.63
48	->	53	-0.18	0.08
48	->	54	-0.08	0.04
48	->	55	-0.09	0.03
48	->	56	-0.02	0.01
48	->	57	-0.00	0.00
48	->	58	-0.00	0.00
48	->	59	-0.00	0.00
48	->	60	-0.00	0.00
48	->	61	-0.00	0.00
48	->	62	0.00	0.00
48	->	63	0.00	0.00
48	->	64	0.00	0.00

48	->	65	0.00	0.00
48	->	66	0.00	0.00
48	->	67	0.00	0.00
48	->	68	-0.00	0.00
48	->	69	-0.00	0.00
48	->	70	0.00	0.00
48	->	71	-0.01	0.00
48	->	72	-0.01	0.01
48	->	73	-0.00	0.00
48	->	74	-0.00	0.00
48	->	75	0.00	0.00
48	->	76	-0.00	0.00
48	->	77	-0.00	0.00
48	->	78	-0.00	0.00
48	->	79	-0.00	0.00
48	->	80	-0.00	0.00
48	->	81	-0.00	0.00
48	->	82	-0.00	0.00
48	->	83	0.00	0.00
48	->	84	0.00	0.00
48	->	85	-0.00	0.00
48	->	86	-0.00	0.00
48	->	87	0.00	0.01
48	->	88	-0.00	0.02
48	->	89	-0.02	0.01
48	->	90	-0.00	0.00
48	->	91	-0.00	0.00
48	->	92	-0.00	0.00
48	->	93	-0.00	0.00
48	->	94	-0.00	0.00
48	->	95	-0.00	0.00
48	->	96	-0.00	0.00
48	->	97	-0.01	0.01
48	->	98	-0.05	0.07
48	->	99	-0.08	0.04
48	->	100	-0.03	0.02
48	->	101	-0.01	0.00
48	->	102	-0.00	0.00
48	->	103	-0.00	0.00
48	->	104	-0.00	0.00
48	->	105	-0.00	0.00
48	->	106	-0.00	0.00
49	->	1	0.00	0.00
49	->	2	0.00	0.00
49	->	3	0.00	0.00
49	->	4	0.00	0.00
49	->	5	-0.00	0.00
49	->	6	0.00	0.00
49	->	7	0.00	0.00
49	->	8	-0.00	0.00

49	->	9	0.00	0.00
49	->	10	-0.00	0.00
49	->	11	-0.00	0.00
49	->	12	0.00	0.00
49	->	13	0.00	0.00
49	->	14	0.00	0.00
49	->	15	0.00	0.00
49	->	16	-0.00	0.00
49	->	17	-0.00	0.00
49	->	18	-0.00	0.00
49	->	19	-0.00	0.00
49	->	20	-0.00	0.00
49	->	21	-0.00	0.00
49	->	22	0.00	0.00
49	->	23	-0.00	0.00
49	->	24	-0.00	0.00
49	->	25	0.00	0.00
49	->	26	-0.00	0.00
49	->	27	0.00	0.00
49	->	28	0.00	0.00
49	->	29	0.00	0.00
49	->	30	-0.00	0.00
49	->	31	-0.00	0.00
49	->	32	-0.00	0.00
49	->	33	-0.04	0.01
49	->	34	-0.01	0.01
49	->	35	0.01	0.01
49	->	36	-0.02	0.02
49	->	37	-0.01	0.00
49	->	38	0.00	0.00
49	->	39	-0.00	0.00
49	->	40	-0.01	0.00
49	->	41	-0.01	0.00
49	->	42	-0.06	0.02
49	->	43	-0.00	0.00
49	->	44	0.01	0.05
49	->	45	-2.86	1.41
49	->	46	-2.80	0.45
49	->	47	-0.95	0.17
49	->	48	-6.16	0.50
49	->	49	-21.02	2.06
49	->	50	-8.10	0.34
49	->	51	-0.84	0.17
49	->	52	-2.77	0.58
49	->	53	-3.17	1.03
49	->	54	-0.13	0.06
49	->	55	-0.04	0.02
49	->	56	-0.03	0.01
49	->	57	-0.01	0.00
49	->	58	-0.03	0.02

49	->	59	-0.00	0.00
49	->	60	-0.02	0.01
49	->	61	-0.03	0.02
49	->	62	-0.00	0.00
49	->	63	0.00	0.00
49	->	64	0.00	0.00
49	->	65	0.00	0.00
49	->	66	0.00	0.00
49	->	67	0.00	0.00
49	->	68	-0.00	0.00
49	->	69	-0.00	0.00
49	->	70	0.00	0.00
49	->	71	-0.05	0.03
49	->	72	0.03	0.02
49	->	73	-0.02	0.01
49	->	74	-0.02	0.01
49	->	75	-0.00	0.00
49	->	76	-0.00	0.00
49	->	77	-0.00	0.00
49	->	78	-0.00	0.00
49	->	79	-0.00	0.00
49	->	80	0.00	0.00
49	->	81	0.00	0.00
49	->	82	0.00	0.00
49	->	83	0.00	0.00
49	->	84	-0.00	0.00
49	->	85	-0.00	0.00
49	->	86	-0.00	0.00
49	->	87	-0.02	0.01
49	->	88	-0.03	0.02
49	->	89	-0.02	0.01
49	->	90	-0.01	0.00
49	->	91	-0.01	0.00
49	->	92	-0.00	0.00
49	->	93	-0.00	0.00
49	->	94	-0.00	0.00
49	->	95	-0.00	0.00
49	->	96	-0.00	0.00
49	->	97	-0.01	0.00
49	->	98	-0.05	0.07
49	->	99	-0.21	0.19
49	->	100	-0.26	0.20
49	->	101	-0.02	0.01
49	->	102	-0.00	0.00
49	->	103	-0.00	0.00
49	->	104	-0.00	0.00
49	->	105	-0.00	0.00
49	->	106	-0.00	0.00
50	->	1	0.00	0.00
50	->	2	0.00	0.00

50 ->	3	-0.00	0.00
50 ->	4	-0.00	0.00
50 ->	5	-0.00	0.00
50 ->	6	0.00	0.00
50 ->	7	0.00	0.00
50 ->	8	-0.00	0.00
50 ->	9	0.00	0.00
50 ->	10	-0.00	0.00
50 ->	11	-0.00	0.00
50 ->	12	0.00	0.00
50 ->	13	0.00	0.00
50 ->	14	0.00	0.00
50 ->	15	0.00	0.00
50 ->	16	-0.00	0.00
50 ->	17	-0.00	0.00
50 ->	18	-0.00	0.00
50 ->	19	-0.00	0.00
50 ->	20	-0.00	0.00
50 ->	21	-0.00	0.00
50 ->	22	-0.00	0.00
50 ->	23	-0.00	0.00
50 ->	24	0.00	0.00
50 ->	25	0.00	0.00
50 ->	26	-0.00	0.00
50 ->	27	0.00	0.00
50 ->	28	0.00	0.00
50 ->	29	0.00	0.00
50 ->	30	-0.00	0.00
50 ->	31	-0.00	0.00
50 ->	32	-0.00	0.00
50 ->	33	-0.01	0.00
50 ->	34	-0.00	0.00
50 ->	35	-0.04	0.02
50 ->	36	-0.01	0.00
50 ->	37	-0.00	0.00
50 ->	38	-0.01	0.00
50 ->	39	-0.00	0.00
50 ->	40	-0.00	0.01
50 ->	41	-0.00	0.01
50 ->	42	-0.09	0.04
50 ->	43	-0.01	0.00
50 ->	44	-0.07	0.03
50 ->	45	-0.08	0.04
50 ->	46	-1.58	0.76
50 ->	47	-1.71	0.40
50 ->	48	-0.93	0.18
50 ->	49	-8.13	0.34
50 ->	50	-22.45	1.53
50 ->	51	-22.10	0.50
50 ->	52	-0.94	0.17

50	->	53	-3.55	0.57
50	->	54	-1.83	0.83
50	->	55	-0.12	0.06
50	->	56	-0.05	0.02
50	->	57	-0.03	0.01
50	->	58	-0.02	0.01
50	->	59	-0.03	0.01
50	->	60	-0.03	0.01
50	->	61	-0.03	0.02
50	->	62	-0.00	0.00
50	->	63	-0.00	0.00
50	->	64	0.00	0.00
50	->	65	-0.00	0.00
50	->	66	0.00	0.00
50	->	67	0.00	0.00
50	->	68	-0.00	0.00
50	->	69	-0.01	0.00
50	->	70	-0.02	0.01
50	->	71	-0.44	0.28
50	->	72	-1.44	0.35
50	->	73	-0.02	0.04
50	->	74	-0.03	0.02
50	->	75	-0.02	0.01
50	->	76	-0.00	0.00
50	->	77	0.00	0.00
50	->	78	-0.00	0.00
50	->	79	-0.00	0.00
50	->	80	0.00	0.00
50	->	81	0.00	0.00
50	->	82	0.00	0.00
50	->	83	0.00	0.00
50	->	84	-0.00	0.00
50	->	85	-0.00	0.00
50	->	86	-0.01	0.01
50	->	87	-0.09	0.06
50	->	88	-0.23	0.17
50	->	89	-0.32	0.30
50	->	90	-0.02	0.01
50	->	91	-0.01	0.00
50	->	92	-0.01	0.00
50	->	93	-0.00	0.00
50	->	94	-0.00	0.00
50	->	95	-0.00	0.00
50	->	96	-0.00	0.00
50	->	97	-0.00	0.00
50	->	98	-0.01	0.00
50	->	99	-0.02	0.01
50	->	100	-0.04	0.04
50	->	101	-0.05	0.04
50	->	102	-0.03	0.02

50	->	103	-0.02	0.01
50	->	104	-0.01	0.00
50	->	105	-0.00	0.00
50	->	106	-0.00	0.00
51	->	1	0.00	0.00
51	->	2	0.00	0.00
51	->	3	0.00	0.00
51	->	4	0.00	0.00
51	->	5	-0.00	0.00
51	->	6	0.00	0.00
51	->	7	0.00	0.00
51	->	8	-0.00	0.00
51	->	9	-0.00	0.00
51	->	10	-0.00	0.00
51	->	11	-0.00	0.00
51	->	12	0.00	0.00
51	->	13	0.00	0.00
51	->	14	0.00	0.00
51	->	15	0.00	0.00
51	->	16	-0.00	0.00
51	->	17	-0.00	0.00
51	->	18	-0.00	0.00
51	->	19	-0.00	0.00
51	->	20	-0.00	0.00
51	->	21	-0.00	0.00
51	->	22	0.00	0.00
51	->	23	-0.00	0.00
51	->	24	0.00	0.00
51	->	25	0.00	0.00
51	->	26	-0.00	0.00
51	->	27	0.00	0.00
51	->	28	0.00	0.00
51	->	29	-0.00	0.00
51	->	30	-0.00	0.00
51	->	31	-0.00	0.00
51	->	32	-0.00	0.00
51	->	33	-0.02	0.01
51	->	34	-0.07	0.03
51	->	35	1.54	0.29
51	->	36	-0.08	0.04
51	->	37	-0.42	0.12
51	->	38	1.66	0.33
51	->	39	-0.04	0.04
51	->	40	-0.73	0.66
51	->	41	-0.03	0.02
51	->	42	-1.88	0.44
51	->	43	-0.01	0.00
51	->	44	-0.01	0.01
51	->	45	-0.04	0.02
51	->	46	-0.14	0.08

51	->	47	-2.11	0.55
51	->	48	-2.86	0.33
51	->	49	-0.84	0.17
51	->	50	-22.15	0.50
51	->	51	10.27	1.20
51	->	52	-10.29	0.44
51	->	53	-1.25	0.21
51	->	54	-3.12	0.75
51	->	55	22.07	14.57
51	->	56	-0.21	0.05
51	->	57	-0.03	0.01
51	->	58	-0.02	0.01
51	->	59	-0.01	0.00
51	->	60	-0.01	0.00
51	->	61	-0.00	0.00
51	->	62	-0.00	0.00
51	->	63	-0.00	0.00
51	->	64	-0.00	0.00
51	->	65	-0.00	0.00
51	->	66	-0.00	0.00
51	->	67	0.00	0.00
51	->	68	-0.00	0.00
51	->	69	-0.00	0.00
51	->	70	-0.01	0.00
51	->	71	-0.02	0.01
51	->	72	-0.07	0.03
51	->	73	0.00	0.00
51	->	74	-0.00	0.00
51	->	75	-0.01	0.00
51	->	76	0.00	0.00
51	->	77	0.00	0.00
51	->	78	0.00	0.00
51	->	79	0.00	0.00
51	->	80	0.00	0.00
51	->	81	0.00	0.00
51	->	82	0.00	0.00
51	->	83	-0.00	0.00
51	->	84	-0.00	0.00
51	->	85	-0.00	0.00
51	->	86	-0.02	0.01
51	->	87	-0.55	0.36
51	->	88	-4.00	2.90
51	->	89	-0.08	0.04
51	->	90	-0.01	0.00
51	->	91	-0.01	0.00
51	->	92	-0.00	0.00
51	->	93	-0.00	0.00
51	->	94	-0.00	0.00
51	->	95	-0.00	0.00
51	->	96	-0.00	0.00

51	->	97	-0.00	0.00
51	->	98	-0.01	0.00
51	->	99	-0.01	0.00
51	->	100	-0.01	0.00
51	->	101	-0.00	0.00
51	->	102	-0.00	0.00
51	->	103	-0.01	0.00
51	->	104	-0.00	0.00
51	->	105	-0.00	0.00
51	->	106	-0.00	0.00
52	->	1	0.00	0.00
52	->	2	0.00	0.00
52	->	3	0.00	0.00
52	->	4	-0.00	0.00
52	->	5	-0.00	0.00
52	->	6	0.00	0.00
52	->	7	0.00	0.00
52	->	8	-0.00	0.00
52	->	9	0.00	0.00
52	->	10	-0.00	0.00
52	->	11	-0.00	0.00
52	->	12	0.00	0.00
52	->	13	0.00	0.00
52	->	14	0.00	0.00
52	->	15	0.00	0.00
52	->	16	-0.00	0.00
52	->	17	-0.00	0.00
52	->	18	-0.00	0.00
52	->	19	-0.00	0.00
52	->	20	-0.00	0.00
52	->	21	-0.00	0.00
52	->	22	0.00	0.00
52	->	23	0.00	0.00
52	->	24	0.00	0.00
52	->	25	0.00	0.00
52	->	26	-0.00	0.00
52	->	27	0.00	0.00
52	->	28	0.00	0.00
52	->	29	0.00	0.00
52	->	30	-0.00	0.00
52	->	31	-0.00	0.00
52	->	32	-0.00	0.00
52	->	33	-0.02	0.01
52	->	34	-0.08	0.04
52	->	35	-0.34	0.44
52	->	36	-0.68	1.04
52	->	37	-0.28	0.32
52	->	38	0.03	0.01
52	->	39	-0.01	0.00
52	->	40	-0.03	0.02

52 ->	41	-0.01	0.01
52 ->	42	-0.06	0.02
52 ->	43	-0.00	0.00
52 ->	44	-0.00	0.01
52 ->	45	-0.06	0.03
52 ->	46	-0.07	0.04
52 ->	47	-0.04	0.04
52 ->	48	-4.16	0.62
52 ->	49	-2.79	0.58
52 ->	50	-0.94	0.17
52 ->	51	-10.32	0.44
52 ->	52	23.79	1.45
52 ->	53	-10.11	0.37
52 ->	54	-1.09	0.27
52 ->	55	-2.56	0.38
52 ->	56	-5.61	1.13
52 ->	57	-0.19	0.10
52 ->	58	-0.33	0.16
52 ->	59	-0.01	0.01
52 ->	60	-0.01	0.00
52 ->	61	-0.01	0.00
52 ->	62	-0.00	0.00
52 ->	63	0.00	0.00
52 ->	64	0.00	0.00
52 ->	65	0.00	0.00
52 ->	66	0.00	0.00
52 ->	67	0.00	0.00
52 ->	68	-0.00	0.00
52 ->	69	0.00	0.00
52 ->	70	0.00	0.00
52 ->	71	-0.02	0.01
52 ->	72	-0.02	0.01
52 ->	73	-0.00	0.00
52 ->	74	-0.00	0.00
52 ->	75	-0.00	0.00
52 ->	76	-0.00	0.00
52 ->	77	0.00	0.00
52 ->	78	-0.00	0.00
52 ->	79	0.00	0.00
52 ->	80	-0.00	0.00
52 ->	81	0.00	0.00
52 ->	82	0.00	0.00
52 ->	83	0.00	0.00
52 ->	84	-0.00	0.00
52 ->	85	-0.00	0.00
52 ->	86	-0.00	0.00
52 ->	87	0.00	0.02
52 ->	88	0.04	0.03
52 ->	89	-0.01	0.00
52 ->	90	-0.00	0.00

52 ->	91	-0.00	0.00
52 ->	92	-0.00	0.00
52 ->	93	-0.00	0.00
52 ->	94	-0.00	0.00
52 ->	95	-0.00	0.00
52 ->	96	-0.00	0.00
52 ->	97	-0.00	0.00
52 ->	98	-0.01	0.01
52 ->	99	-0.02	0.01
52 ->	100	-0.02	0.01
52 ->	101	-0.00	0.00
52 ->	102	-0.00	0.00
52 ->	103	-0.00	0.00
52 ->	104	-0.00	0.00
52 ->	105	-0.00	0.00
52 ->	106	-0.00	0.00
53 ->	1	0.01	0.00
53 ->	2	0.00	0.00
53 ->	3	-0.00	0.00
53 ->	4	0.00	0.00
53 ->	5	-0.01	0.00
53 ->	6	0.00	0.00
53 ->	7	0.00	0.00
53 ->	8	-0.00	0.00
53 ->	9	0.00	0.00
53 ->	10	-0.01	0.00
53 ->	11	-0.00	0.00
53 ->	12	0.01	0.00
53 ->	13	0.00	0.00
53 ->	14	0.00	0.00
53 ->	15	0.00	0.00
53 ->	16	-0.01	0.00
53 ->	17	-0.00	0.00
53 ->	18	0.00	0.00
53 ->	19	-0.00	0.00
53 ->	20	-0.00	0.00
53 ->	21	0.00	0.00
53 ->	22	0.03	0.00
53 ->	23	0.00	0.00
53 ->	24	0.00	0.00
53 ->	25	0.02	0.00
53 ->	26	0.00	0.00
53 ->	27	0.00	0.00
53 ->	28	0.00	0.00
53 ->	29	0.00	0.00
53 ->	30	-0.00	0.00
53 ->	31	0.02	0.00
53 ->	32	-0.00	0.00
53 ->	33	-0.00	0.00
53 ->	34	-0.01	0.00

53	->	35	-0.05	0.02
53	->	36	0.02	0.01
53	->	37	-0.01	0.01
53	->	38	-0.04	0.01
53	->	39	-0.00	0.00
53	->	40	0.02	0.01
53	->	41	-0.00	0.00
53	->	42	-0.01	0.01
53	->	43	-0.00	0.00
53	->	44	0.04	0.01
53	->	45	-0.02	0.01
53	->	46	-0.16	0.05
53	->	47	-0.03	0.02
53	->	48	-0.18	0.08
53	->	49	-3.21	1.03
53	->	50	-3.58	0.57
53	->	51	-1.25	0.21
53	->	52	-10.14	0.37
53	->	53	-104.69	2.72
53	->	54	-5.32	0.66
53	->	55	-0.77	0.15
53	->	56	-1.45	0.26
53	->	57	-1.36	0.35
53	->	58	-3.86	1.01
53	->	59	-0.01	0.12
53	->	60	-1.60	0.29
53	->	61	-0.82	0.31
53	->	62	-0.01	0.00
53	->	63	-0.03	0.00
53	->	64	-0.02	0.00
53	->	65	-0.00	0.00
53	->	66	-0.02	0.00
53	->	67	-0.00	0.00
53	->	68	0.03	0.01
53	->	69	0.08	0.02
53	->	70	0.02	0.05
53	->	71	-3.39	1.49
53	->	72	-0.35	0.24
53	->	73	-0.18	0.12
53	->	74	-0.12	0.05
53	->	75	-0.14	0.10
53	->	76	0.04	0.02
53	->	77	0.03	0.01
53	->	78	0.03	0.01
53	->	79	0.00	0.00
53	->	80	0.00	0.00
53	->	81	0.02	0.00
53	->	82	0.00	0.00
53	->	83	-0.00	0.00
53	->	84	-0.01	0.00

53 ->	85	-0.01	0.01
53 ->	86	-0.11	0.05
53 ->	87	-0.36	0.23
53 ->	88	-0.15	0.10
53 ->	89	-0.04	0.01
53 ->	90	-0.03	0.00
53 ->	91	-0.02	0.00
53 ->	92	-0.01	0.00
53 ->	93	-0.02	0.00
53 ->	94	-0.02	0.00
53 ->	95	-0.01	0.00
53 ->	96	-0.00	0.00
53 ->	97	-0.02	0.00
53 ->	98	-0.04	0.01
53 ->	99	-0.31	0.44
53 ->	100	-1.68	2.83
53 ->	101	-0.11	0.05
53 ->	102	-0.03	0.01
53 ->	103	-0.02	0.00
53 ->	104	-0.01	0.00
53 ->	105	-0.01	0.00
53 ->	106	-0.01	0.00
54 ->	1	0.00	0.00
54 ->	2	0.00	0.00
54 ->	3	-0.00	0.00
54 ->	4	-0.00	0.00
54 ->	5	-0.00	0.00
54 ->	6	0.00	0.00
54 ->	7	0.00	0.00
54 ->	8	-0.00	0.00
54 ->	9	0.00	0.00
54 ->	10	-0.00	0.00
54 ->	11	-0.00	0.00
54 ->	12	0.00	0.00
54 ->	13	0.00	0.00
54 ->	14	0.00	0.00
54 ->	15	0.00	0.00
54 ->	16	-0.00	0.00
54 ->	17	0.00	0.00
54 ->	18	0.00	0.00
54 ->	19	-0.00	0.00
54 ->	20	-0.00	0.00
54 ->	21	0.00	0.00
54 ->	22	0.00	0.00
54 ->	23	0.00	0.00
54 ->	24	-0.00	0.00
54 ->	25	0.00	0.00
54 ->	26	0.00	0.00
54 ->	27	0.00	0.00
54 ->	28	0.00	0.00

54	->	29	0.00	0.00
54	->	30	-0.00	0.00
54	->	31	0.00	0.00
54	->	32	-0.00	0.00
54	->	33	-0.00	0.00
54	->	34	-0.00	0.00
54	->	35	-0.07	0.05
54	->	36	-0.00	0.00
54	->	37	-0.01	0.01
54	->	38	-0.05	0.03
54	->	39	-0.00	0.00
54	->	40	-0.01	0.01
54	->	41	-0.00	0.00
54	->	42	-0.02	0.01
54	->	43	-0.00	0.00
54	->	44	-0.00	0.00
54	->	45	-0.00	0.00
54	->	46	-0.01	0.00
54	->	47	-0.02	0.01
54	->	48	-0.08	0.04
54	->	49	-0.13	0.06
54	->	50	-1.84	0.82
54	->	51	-3.11	0.76
54	->	52	-1.10	0.27
54	->	53	-5.36	0.66
54	->	54	-23.50	2.08
54	->	55	-22.28	0.53
54	->	56	-0.57	0.15
54	->	57	-0.93	0.34
54	->	58	-0.15	0.09
54	->	59	-0.14	0.10
54	->	60	-0.02	0.01
54	->	61	-0.00	0.00
54	->	62	-0.00	0.00
54	->	63	0.00	0.00
54	->	64	0.00	0.00
54	->	65	0.00	0.00
54	->	66	0.00	0.00
54	->	67	0.00	0.00
54	->	68	-0.01	0.00
54	->	69	-0.01	0.00
54	->	70	-0.03	0.01
54	->	71	-0.30	0.31
54	->	72	-0.16	0.19
54	->	73	0.00	0.00
54	->	74	-0.00	0.00
54	->	75	-0.02	0.04
54	->	76	-0.00	0.00
54	->	77	0.00	0.00
54	->	78	-0.00	0.00

54	->	79	-0.00	0.00
54	->	80	0.00	0.00
54	->	81	0.00	0.00
54	->	82	0.00	0.00
54	->	83	0.00	0.00
54	->	84	0.00	0.00
54	->	85	-0.01	0.00
54	->	86	-0.13	0.12
54	->	87	-2.17	1.42
54	->	88	-1.08	1.68
54	->	89	-0.02	0.01
54	->	90	-0.00	0.00
54	->	91	-0.00	0.00
54	->	92	-0.00	0.00
54	->	93	-0.00	0.00
54	->	94	-0.00	0.00
54	->	95	-0.00	0.00
54	->	96	-0.00	0.00
54	->	97	-0.00	0.00
54	->	98	-0.00	0.00
54	->	99	-0.00	0.00
54	->	100	-0.00	0.00
54	->	101	-0.00	0.00
54	->	102	-0.00	0.00
54	->	103	-0.00	0.00
54	->	104	-0.00	0.00
54	->	105	-0.00	0.00
54	->	106	-0.00	0.00
55	->	1	0.00	0.00
55	->	2	0.00	0.00
55	->	3	0.00	0.00
55	->	4	0.00	0.00
55	->	5	0.00	0.00
55	->	6	0.00	0.00
55	->	7	0.00	0.00
55	->	8	0.00	0.00
55	->	9	0.00	0.00
55	->	10	0.00	0.00
55	->	11	0.00	0.00
55	->	12	0.00	0.00
55	->	13	-0.00	0.00
55	->	14	0.00	0.00
55	->	15	0.00	0.00
55	->	16	-0.00	0.00
55	->	17	0.00	0.00
55	->	18	0.00	0.00
55	->	19	0.00	0.00
55	->	20	0.00	0.00
55	->	21	0.00	0.00
55	->	22	0.00	0.00

55	->	23	0.00	0.00
55	->	24	0.00	0.00
55	->	25	-0.00	0.00
55	->	26	-0.00	0.00
55	->	27	-0.00	0.00
55	->	28	0.00	0.00
55	->	29	0.00	0.00
55	->	30	0.00	0.00
55	->	31	-0.01	0.00
55	->	32	-0.00	0.00
55	->	33	-0.01	0.00
55	->	34	-0.06	0.02
55	->	35	3.46	0.62
55	->	36	-0.31	0.15
55	->	37	-3.59	0.72
55	->	38	2.57	0.80
55	->	39	0.01	0.03
55	->	40	-0.17	0.15
55	->	41	-0.01	0.01
55	->	42	-0.00	0.01
55	->	43	0.00	0.00
55	->	44	-0.01	0.00
55	->	45	-0.00	0.00
55	->	46	-0.01	0.00
55	->	47	-0.04	0.01
55	->	48	-0.09	0.03
55	->	49	-0.04	0.02
55	->	50	-0.12	0.06
55	->	51	22.05	14.57
55	->	52	-2.55	0.38
55	->	53	-0.77	0.15
55	->	54	-22.38	0.52
55	->	55	4.80	1.23
55	->	56	-4.97	0.44
55	->	57	-0.50	0.11
55	->	58	-0.04	0.02
55	->	59	-0.03	0.02
55	->	60	-0.00	0.00
55	->	61	-0.00	0.00
55	->	62	0.00	0.00
55	->	63	-0.00	0.00
55	->	64	-0.00	0.00
55	->	65	-0.00	0.00
55	->	66	-0.00	0.00
55	->	67	0.00	0.00
55	->	68	-0.00	0.00
55	->	69	-0.00	0.00
55	->	70	-0.01	0.00
55	->	71	-0.01	0.00
55	->	72	-0.01	0.01

55	->	73	-0.00	0.00
55	->	74	-0.00	0.00
55	->	75	-0.00	0.00
55	->	76	0.00	0.00
55	->	77	0.00	0.00
55	->	78	0.00	0.00
55	->	79	0.00	0.00
55	->	80	0.00	0.00
55	->	81	0.00	0.00
55	->	82	0.00	0.00
55	->	83	0.00	0.00
55	->	84	-0.00	0.00
55	->	85	-0.00	0.00
55	->	86	0.01	0.01
55	->	87	0.06	0.06
55	->	88	0.04	0.05
55	->	89	-0.00	0.00
55	->	90	-0.00	0.00
55	->	91	-0.00	0.00
55	->	92	-0.00	0.00
55	->	93	-0.00	0.00
55	->	94	-0.00	0.00
55	->	95	-0.00	0.00
55	->	96	-0.00	0.00
55	->	97	-0.00	0.00
55	->	98	-0.00	0.00
55	->	99	-0.00	0.00
55	->	100	-0.00	0.00
55	->	101	-0.00	0.00
55	->	102	-0.00	0.00
55	->	103	-0.00	0.00
55	->	104	-0.00	0.00
55	->	105	-0.00	0.00
55	->	106	-0.00	0.00
56	->	1	0.00	0.00
56	->	2	0.00	0.00
56	->	3	0.00	0.00
56	->	4	0.00	0.00
56	->	5	-0.00	0.00
56	->	6	0.00	0.00
56	->	7	0.00	0.00
56	->	8	-0.00	0.00
56	->	9	0.00	0.00
56	->	10	-0.00	0.00
56	->	11	-0.00	0.00
56	->	12	0.00	0.00
56	->	13	0.00	0.00
56	->	14	0.00	0.00
56	->	15	0.00	0.00
56	->	16	-0.00	0.00

56 ->	17	-0.00	0.00
56 ->	18	-0.00	0.00
56 ->	19	0.00	0.00
56 ->	20	-0.00	0.00
56 ->	21	0.00	0.00
56 ->	22	0.00	0.00
56 ->	23	0.00	0.00
56 ->	24	-0.00	0.00
56 ->	25	0.00	0.00
56 ->	26	0.00	0.00
56 ->	27	0.00	0.00
56 ->	28	0.00	0.00
56 ->	29	0.00	0.00
56 ->	30	-0.00	0.00
56 ->	31	0.00	0.00
56 ->	32	-0.00	0.00
56 ->	33	-0.00	0.00
56 ->	34	-0.00	0.00
56 ->	35	-0.02	0.01
56 ->	36	-0.11	0.17
56 ->	37	-0.26	0.28
56 ->	38	-0.01	0.00
56 ->	39	-0.00	0.00
56 ->	40	0.00	0.00
56 ->	41	-0.00	0.00
56 ->	42	-0.00	0.00
56 ->	43	0.00	0.00
56 ->	44	0.00	0.00
56 ->	45	-0.00	0.00
56 ->	46	-0.01	0.00
56 ->	47	-0.00	0.00
56 ->	48	-0.02	0.01
56 ->	49	-0.03	0.01
56 ->	50	-0.05	0.02
56 ->	51	-0.21	0.05
56 ->	52	-5.62	1.12
56 ->	53	-1.46	0.26
56 ->	54	-0.58	0.15
56 ->	55	-5.13	0.45
56 ->	56	-22.96	1.50
56 ->	57	-11.64	0.51
56 ->	58	-2.00	0.68
56 ->	59	-0.03	0.02
56 ->	60	-0.02	0.00
56 ->	61	-0.00	0.00
56 ->	62	-0.00	0.00
56 ->	63	0.00	0.00
56 ->	64	0.00	0.00
56 ->	65	0.00	0.00
56 ->	66	0.00	0.00

56	->	67	0.00	0.00
56	->	68	-0.00	0.00
56	->	69	-0.01	0.00
56	->	70	-0.00	0.00
56	->	71	-0.02	0.00
56	->	72	-0.00	0.00
56	->	73	-0.00	0.00
56	->	74	-0.00	0.00
56	->	75	-0.00	0.00
56	->	76	-0.00	0.00
56	->	77	0.00	0.00
56	->	78	0.00	0.00
56	->	79	0.00	0.00
56	->	80	0.00	0.00
56	->	81	0.00	0.00
56	->	82	0.00	0.00
56	->	83	0.00	0.00
56	->	84	0.00	0.00
56	->	85	-0.00	0.00
56	->	86	-0.01	0.01
56	->	87	-0.03	0.02
56	->	88	-0.01	0.01
56	->	89	-0.00	0.00
56	->	90	-0.00	0.00
56	->	91	-0.00	0.00
56	->	92	-0.00	0.00
56	->	93	-0.00	0.00
56	->	94	-0.00	0.00
56	->	95	-0.00	0.00
56	->	96	-0.00	0.00
56	->	97	-0.00	0.00
56	->	98	-0.00	0.00
56	->	99	-0.00	0.00
56	->	100	-0.00	0.00
56	->	101	-0.00	0.00
56	->	102	-0.00	0.00
56	->	103	-0.00	0.00
56	->	104	-0.00	0.00
56	->	105	-0.00	0.00
56	->	106	-0.00	0.00
57	->	1	0.00	0.00
57	->	2	0.00	0.00
57	->	3	0.00	0.00
57	->	4	0.00	0.00
57	->	5	-0.00	0.00
57	->	6	0.00	0.00
57	->	7	0.00	0.00
57	->	8	-0.00	0.00
57	->	9	0.00	0.00
57	->	10	-0.00	0.00

57	->	11	-0.00	0.00
57	->	12	0.00	0.00
57	->	13	0.00	0.00
57	->	14	0.00	0.00
57	->	15	0.00	0.00
57	->	16	-0.00	0.00
57	->	17	-0.00	0.00
57	->	18	0.00	0.00
57	->	19	-0.00	0.00
57	->	20	0.00	0.00
57	->	21	0.00	0.00
57	->	22	0.00	0.00
57	->	23	0.00	0.00
57	->	24	0.00	0.00
57	->	25	0.00	0.00
57	->	26	0.00	0.00
57	->	27	0.00	0.00
57	->	28	0.00	0.00
57	->	29	0.00	0.00
57	->	30	-0.00	0.00
57	->	31	0.00	0.00
57	->	32	0.00	0.00
57	->	33	-0.00	0.00
57	->	34	-0.00	0.00
57	->	35	-0.00	0.00
57	->	36	-0.00	0.00
57	->	37	-0.01	0.00
57	->	38	-0.00	0.00
57	->	39	0.00	0.00
57	->	40	0.00	0.00
57	->	41	-0.00	0.00
57	->	42	-0.00	0.00
57	->	43	0.00	0.00
57	->	44	0.00	0.00
57	->	45	-0.00	0.00
57	->	46	-0.00	0.00
57	->	47	-0.00	0.00
57	->	48	-0.00	0.00
57	->	49	-0.01	0.00
57	->	50	-0.03	0.01
57	->	51	-0.03	0.01
57	->	52	-0.19	0.10
57	->	53	-1.37	0.36
57	->	54	-0.97	0.35
57	->	55	-0.51	0.11
57	->	56	-11.71	0.51
57	->	57	12.16	0.64
57	->	58	-15.09	0.49
57	->	59	-1.19	0.50
57	->	60	-0.03	0.00

57	->	61	-0.01	0.00
57	->	62	-0.00	0.00
57	->	63	0.00	0.00
57	->	64	0.00	0.00
57	->	65	0.00	0.00
57	->	66	0.00	0.00
57	->	67	-0.00	0.00
57	->	68	-0.00	0.00
57	->	69	-0.02	0.01
57	->	70	-0.00	0.01
57	->	71	-0.04	0.01
57	->	72	-0.01	0.00
57	->	73	-0.00	0.00
57	->	74	-0.00	0.00
57	->	75	-0.01	0.01
57	->	76	-0.00	0.00
57	->	77	0.00	0.00
57	->	78	0.00	0.00
57	->	79	-0.00	0.00
57	->	80	-0.00	0.00
57	->	81	0.00	0.00
57	->	82	0.00	0.00
57	->	83	0.00	0.00
57	->	84	0.00	0.00
57	->	85	-0.00	0.00
57	->	86	-0.03	0.02
57	->	87	-0.08	0.09
57	->	88	-0.01	0.00
57	->	89	-0.00	0.00
57	->	90	-0.00	0.00
57	->	91	-0.00	0.00
57	->	92	-0.00	0.00
57	->	93	-0.00	0.00
57	->	94	-0.00	0.00
57	->	95	-0.00	0.00
57	->	96	-0.00	0.00
57	->	97	-0.00	0.00
57	->	98	-0.00	0.00
57	->	99	-0.00	0.00
57	->	100	-0.00	0.00
57	->	101	-0.00	0.00
57	->	102	-0.00	0.00
57	->	103	-0.00	0.00
57	->	104	-0.00	0.00
57	->	105	-0.00	0.00
57	->	106	-0.00	0.00
58	->	1	-0.01	0.00
58	->	2	-0.00	0.00
58	->	3	0.00	0.00
58	->	4	-0.00	0.00

58	->	5	0.00	0.00
58	->	6	-0.00	0.00
58	->	7	-0.00	0.00
58	->	8	0.00	0.00
58	->	9	0.00	0.00
58	->	10	0.00	0.00
58	->	11	0.00	0.00
58	->	12	-0.00	0.00
58	->	13	-0.00	0.00
58	->	14	-0.00	0.00
58	->	15	-0.00	0.00
58	->	16	0.01	0.00
58	->	17	0.00	0.00
58	->	18	-0.00	0.00
58	->	19	0.00	0.00
58	->	20	0.00	0.00
58	->	21	-0.00	0.00
58	->	22	-0.01	0.00
58	->	23	-0.00	0.00
58	->	24	-0.00	0.00
58	->	25	-0.01	0.00
58	->	26	-0.00	0.00
58	->	27	-0.00	0.00
58	->	28	0.00	0.00
58	->	29	-0.00	0.00
58	->	30	0.00	0.00
58	->	31	-0.01	0.00
58	->	32	0.00	0.00
58	->	33	-0.00	0.00
58	->	34	0.00	0.00
58	->	35	0.03	0.01
58	->	36	-0.07	0.11
58	->	37	-0.01	0.00
58	->	38	0.03	0.00
58	->	39	-0.00	0.00
58	->	40	-0.02	0.00
58	->	41	0.00	0.00
58	->	42	-0.00	0.00
58	->	43	-0.00	0.00
58	->	44	-0.02	0.00
58	->	45	0.00	0.00
58	->	46	0.03	0.01
58	->	47	0.00	0.00
58	->	48	-0.00	0.00
58	->	49	-0.03	0.02
58	->	50	-0.02	0.01
58	->	51	-0.02	0.01
58	->	52	-0.33	0.15
58	->	53	-3.83	0.99
58	->	54	-0.15	0.09

58	->	55	-0.04	0.02
58	->	56	-2.03	0.67
58	->	57	-15.23	0.49
58	->	58	-62.71	2.28
58	->	59	-11.95	0.67
58	->	60	-1.48	0.43
58	->	61	-0.10	0.02
58	->	62	-0.01	0.00
58	->	63	0.02	0.00
58	->	64	0.01	0.00
58	->	65	0.00	0.00
58	->	66	0.02	0.00
58	->	67	0.00	0.00
58	->	68	-0.03	0.01
58	->	69	-0.07	0.09
58	->	70	-0.26	0.07
58	->	71	-0.70	0.24
58	->	72	-0.04	0.01
58	->	73	-0.01	0.00
58	->	74	0.03	0.00
58	->	75	0.05	0.02
58	->	76	-0.04	0.01
58	->	77	-0.02	0.00
58	->	78	-0.02	0.00
58	->	79	-0.00	0.00
58	->	80	-0.00	0.00
58	->	81	-0.01	0.00
58	->	82	-0.00	0.00
58	->	83	0.00	0.00
58	->	84	0.01	0.00
58	->	85	0.00	0.00
58	->	86	0.03	0.01
58	->	87	0.04	0.02
58	->	88	0.03	0.00
58	->	89	0.01	0.00
58	->	90	0.01	0.00
58	->	91	0.01	0.00
58	->	92	0.01	0.00
58	->	93	0.01	0.00
58	->	94	0.01	0.00
58	->	95	0.01	0.00
58	->	96	0.00	0.00
58	->	97	0.01	0.00
58	->	98	0.02	0.00
58	->	99	0.03	0.01
58	->	100	0.04	0.01
58	->	101	0.02	0.00
58	->	102	0.01	0.00
58	->	103	0.01	0.00
58	->	104	0.01	0.00

58	->	105	0.00	0.00
58	->	106	0.00	0.00
59	->	1	0.01	0.00
59	->	2	0.00	0.00
59	->	3	-0.00	0.00
59	->	4	0.00	0.00
59	->	5	-0.00	0.00
59	->	6	0.00	0.00
59	->	7	-0.00	0.00
59	->	8	-0.00	0.00
59	->	9	0.00	0.00
59	->	10	-0.00	0.00
59	->	11	-0.00	0.00
59	->	12	0.01	0.00
59	->	13	0.00	0.00
59	->	14	0.00	0.00
59	->	15	0.00	0.00
59	->	16	-0.00	0.00
59	->	17	-0.00	0.00
59	->	18	0.00	0.00
59	->	19	-0.00	0.00
59	->	20	-0.00	0.00
59	->	21	0.00	0.00
59	->	22	0.01	0.00
59	->	23	0.00	0.00
59	->	24	0.00	0.00
59	->	25	0.01	0.00
59	->	26	0.00	0.00
59	->	27	0.00	0.00
59	->	28	0.00	0.00
59	->	29	0.00	0.00
59	->	30	-0.00	0.00
59	->	31	0.01	0.00
59	->	32	-0.00	0.00
59	->	33	0.00	0.00
59	->	34	-0.00	0.00
59	->	35	-0.02	0.00
59	->	36	0.02	0.01
59	->	37	0.00	0.00
59	->	38	-0.03	0.00
59	->	39	0.00	0.00
59	->	40	0.02	0.01
59	->	41	-0.00	0.00
59	->	42	-0.00	0.00
59	->	43	0.00	0.00
59	->	44	0.02	0.00
59	->	45	-0.00	0.00
59	->	46	-0.03	0.00
59	->	47	-0.01	0.00
59	->	48	-0.00	0.00

59 ->	49	-0.00	0.00
59 ->	50	-0.03	0.01
59 ->	51	-0.01	0.00
59 ->	52	-0.01	0.01
59 ->	53	-0.01	0.12
59 ->	54	-0.14	0.10
59 ->	55	-0.03	0.02
59 ->	56	-0.03	0.02
59 ->	57	-1.26	0.51
59 ->	58	-12.00	0.68
59 ->	59	11.61	2.94
59 ->	60	-0.31	0.39
59 ->	61	-0.97	0.28
59 ->	62	-0.15	0.03
59 ->	63	-0.08	0.01
59 ->	64	-0.03	0.00
59 ->	65	-0.00	0.00
59 ->	66	-0.06	0.01
59 ->	67	-0.01	0.01
59 ->	68	0.17	0.17
59 ->	69	-2.03	0.73
59 ->	70	-3.23	0.39
59 ->	71	-1.77	0.39
59 ->	72	-0.04	0.04
59 ->	73	-0.06	0.02
59 ->	74	-0.07	0.01
59 ->	75	-0.59	0.62
59 ->	76	0.03	0.01
59 ->	77	0.01	0.00
59 ->	78	0.05	0.01
59 ->	79	-0.00	0.00
59 ->	80	-0.00	0.00
59 ->	81	0.02	0.00
59 ->	82	0.00	0.00
59 ->	83	-0.00	0.00
59 ->	84	-0.01	0.00
59 ->	85	-0.25	0.35
59 ->	86	-1.93	2.62
59 ->	87	-0.43	0.46
59 ->	88	-0.05	0.01
59 ->	89	-0.02	0.00
59 ->	90	-0.01	0.00
59 ->	91	-0.01	0.00
59 ->	92	-0.01	0.00
59 ->	93	-0.01	0.00
59 ->	94	-0.01	0.00
59 ->	95	-0.00	0.00
59 ->	96	-0.00	0.00
59 ->	97	-0.00	0.00
59 ->	98	-0.01	0.00

59	->	99	-0.02	0.01
59	->	100	-0.04	0.03
59	->	101	-0.03	0.01
59	->	102	-0.02	0.00
59	->	103	-0.02	0.00
59	->	104	-0.01	0.00
59	->	105	-0.01	0.00
59	->	106	-0.01	0.00
60	->	1	0.00	0.00
60	->	2	0.00	0.00
60	->	3	0.00	0.00
60	->	4	0.00	0.00
60	->	5	-0.00	0.00
60	->	6	0.00	0.00
60	->	7	0.00	0.00
60	->	8	-0.00	0.00
60	->	9	0.00	0.00
60	->	10	-0.00	0.00
60	->	11	-0.00	0.00
60	->	12	0.00	0.00
60	->	13	0.00	0.00
60	->	14	0.00	0.00
60	->	15	0.00	0.00
60	->	16	-0.00	0.00
60	->	17	0.00	0.00
60	->	18	0.00	0.00
60	->	19	-0.00	0.00
60	->	20	0.00	0.00
60	->	21	0.00	0.00
60	->	22	0.00	0.00
60	->	23	0.00	0.00
60	->	24	0.00	0.00
60	->	25	0.00	0.00
60	->	26	0.00	0.00
60	->	27	0.00	0.00
60	->	28	0.00	0.00
60	->	29	0.00	0.00
60	->	30	-0.00	0.00
60	->	31	0.00	0.00
60	->	32	-0.00	0.00
60	->	33	-0.00	0.00
60	->	34	-0.00	0.00
60	->	35	-0.00	0.00
60	->	36	0.00	0.00
60	->	37	-0.00	0.00
60	->	38	-0.00	0.00
60	->	39	-0.00	0.00
60	->	40	0.00	0.00
60	->	41	-0.00	0.00
60	->	42	-0.00	0.00

60	->	43	0.00	0.00
60	->	44	0.00	0.00
60	->	45	-0.00	0.00
60	->	46	-0.01	0.00
60	->	47	-0.00	0.00
60	->	48	-0.00	0.00
60	->	49	-0.02	0.01
60	->	50	-0.03	0.01
60	->	51	-0.01	0.00
60	->	52	-0.01	0.00
60	->	53	-1.59	0.30
60	->	54	-0.02	0.01
60	->	55	-0.00	0.00
60	->	56	-0.02	0.00
60	->	57	-0.03	0.00
60	->	58	-1.52	0.44
60	->	59	-0.36	0.39
60	->	60	1.80	0.61
60	->	61	-10.29	0.49
60	->	62	-0.35	0.12
60	->	63	-0.02	0.00
60	->	64	-0.00	0.00
60	->	65	-0.00	0.00
60	->	66	0.00	0.00
60	->	67	-0.00	0.00
60	->	68	-0.02	0.01
60	->	69	-0.53	0.72
60	->	70	-0.88	0.17
60	->	71	-0.80	0.19
60	->	72	-0.13	0.08
60	->	73	-0.05	0.03
60	->	74	-0.01	0.00
60	->	75	-0.02	0.01
60	->	76	-0.03	0.01
60	->	77	-0.01	0.00
60	->	78	-0.00	0.00
60	->	79	-0.00	0.00
60	->	80	-0.00	0.00
60	->	81	-0.00	0.00
60	->	82	-0.00	0.00
60	->	83	-0.00	0.00
60	->	84	0.00	0.00
60	->	85	-0.00	0.00
60	->	86	-0.01	0.00
60	->	87	-0.01	0.00
60	->	88	-0.00	0.00
60	->	89	-0.00	0.00
60	->	90	-0.00	0.00
60	->	91	-0.00	0.00
60	->	92	-0.00	0.00

60	->	93	-0.00	0.00
60	->	94	-0.00	0.00
60	->	95	-0.00	0.00
60	->	96	-0.00	0.00
60	->	97	-0.00	0.00
60	->	98	-0.00	0.00
60	->	99	-0.01	0.01
60	->	100	-0.05	0.06
60	->	101	-0.02	0.01
60	->	102	-0.00	0.00
60	->	103	-0.00	0.00
60	->	104	-0.00	0.00
60	->	105	-0.00	0.00
60	->	106	-0.00	0.00
61	->	1	0.00	0.00
61	->	2	0.00	0.00
61	->	3	-0.00	0.00
61	->	4	0.00	0.00
61	->	5	-0.00	0.00
61	->	6	0.00	0.00
61	->	7	0.00	0.00
61	->	8	-0.00	0.00
61	->	9	0.00	0.00
61	->	10	-0.00	0.00
61	->	11	-0.00	0.00
61	->	12	0.00	0.00
61	->	13	0.00	0.00
61	->	14	0.00	0.00
61	->	15	0.00	0.00
61	->	16	-0.00	0.00
61	->	17	0.00	0.00
61	->	18	0.00	0.00
61	->	19	-0.00	0.00
61	->	20	0.00	0.00
61	->	21	0.00	0.00
61	->	22	-0.00	0.00
61	->	23	0.00	0.00
61	->	24	0.00	0.00
61	->	25	0.00	0.00
61	->	26	0.00	0.00
61	->	27	0.00	0.00
61	->	28	0.00	0.00
61	->	29	0.00	0.00
61	->	30	-0.00	0.00
61	->	31	0.00	0.00
61	->	32	0.00	0.00
61	->	33	-0.00	0.00
61	->	34	0.00	0.00
61	->	35	0.00	0.00
61	->	36	-0.00	0.00

61	->	37	-0.00	0.00
61	->	38	0.00	0.00
61	->	39	0.00	0.00
61	->	40	-0.00	0.00
61	->	41	0.00	0.00
61	->	42	-0.00	0.00
61	->	43	0.00	0.00
61	->	44	-0.00	0.00
61	->	45	-0.00	0.00
61	->	46	-0.01	0.01
61	->	47	-0.00	0.00
61	->	48	-0.00	0.00
61	->	49	-0.03	0.02
61	->	50	-0.03	0.02
61	->	51	-0.00	0.00
61	->	52	-0.01	0.00
61	->	53	-0.82	0.30
61	->	54	-0.00	0.00
61	->	55	-0.00	0.00
61	->	56	-0.00	0.00
61	->	57	-0.01	0.00
61	->	58	-0.10	0.02
61	->	59	-0.98	0.28
61	->	60	-10.42	0.49
61	->	61	27.26	1.46
61	->	62	-10.59	0.41
61	->	63	-0.38	0.06
61	->	64	-0.05	0.01
61	->	65	-0.01	0.00
61	->	66	-0.01	0.00
61	->	67	-0.02	0.01
61	->	68	-0.30	0.06
61	->	69	-2.79	0.53
61	->	70	-3.72	0.45
61	->	71	-0.98	0.30
61	->	72	-1.81	0.77
61	->	73	-1.78	0.83
61	->	74	-0.00	0.02
61	->	75	-0.12	0.05
61	->	76	-1.25	0.81
61	->	77	-0.04	0.03
61	->	78	-0.01	0.00
61	->	79	-0.02	0.00
61	->	80	-0.01	0.01
61	->	81	-0.00	0.00
61	->	82	-0.00	0.00
61	->	83	-0.01	0.00
61	->	84	-0.00	0.00
61	->	85	-0.00	0.00
61	->	86	-0.01	0.00

61	->	87	-0.01	0.00
61	->	88	-0.00	0.00
61	->	89	-0.00	0.00
61	->	90	-0.00	0.00
61	->	91	-0.00	0.00
61	->	92	-0.01	0.00
61	->	93	-0.00	0.00
61	->	94	-0.00	0.00
61	->	95	-0.00	0.00
61	->	96	-0.00	0.00
61	->	97	-0.00	0.00
61	->	98	-0.00	0.00
61	->	99	-0.02	0.02
61	->	100	-0.56	0.83
61	->	101	-0.25	0.26
61	->	102	-0.02	0.01
61	->	103	-0.01	0.00
61	->	104	-0.00	0.00
61	->	105	-0.00	0.00
61	->	106	-0.00	0.00
62	->	1	-0.00	0.00
62	->	2	0.00	0.00
62	->	3	0.00	0.00
62	->	4	0.00	0.00
62	->	5	0.00	0.00
62	->	6	0.00	0.00
62	->	7	0.00	0.00
62	->	8	0.00	0.00
62	->	9	0.00	0.00
62	->	10	0.00	0.00
62	->	11	0.00	0.00
62	->	12	-0.00	0.00
62	->	13	-0.00	0.00
62	->	14	-0.00	0.00
62	->	15	0.00	0.00
62	->	16	0.00	0.00
62	->	17	0.00	0.00
62	->	18	-0.00	0.00
62	->	19	0.00	0.00
62	->	20	0.00	0.00
62	->	21	-0.00	0.00
62	->	22	-0.00	0.00
62	->	23	0.00	0.00
62	->	24	-0.00	0.00
62	->	25	-0.00	0.00
62	->	26	-0.00	0.00
62	->	27	0.00	0.00
62	->	28	0.00	0.00
62	->	29	-0.00	0.00
62	->	30	0.00	0.00

62	->	31	-0.00	0.00
62	->	32	0.00	0.00
62	->	33	-0.00	0.00
62	->	34	0.00	0.00
62	->	35	0.00	0.00
62	->	36	-0.00	0.00
62	->	37	-0.00	0.00
62	->	38	0.00	0.00
62	->	39	0.00	0.00
62	->	40	-0.00	0.00
62	->	41	0.00	0.00
62	->	42	0.00	0.00
62	->	43	0.00	0.00
62	->	44	-0.00	0.00
62	->	45	0.00	0.00
62	->	46	0.00	0.00
62	->	47	0.00	0.00
62	->	48	0.00	0.00
62	->	49	-0.00	0.00
62	->	50	-0.00	0.00
62	->	51	-0.00	0.00
62	->	52	-0.00	0.00
62	->	53	-0.01	0.00
62	->	54	-0.00	0.00
62	->	55	0.00	0.00
62	->	56	-0.00	0.00
62	->	57	-0.00	0.00
62	->	58	-0.01	0.00
62	->	59	-0.15	0.03
62	->	60	-0.37	0.13
62	->	61	-10.58	0.41
62	->	62	22.68	1.11
62	->	63	-11.87	0.33
62	->	64	-0.46	0.09
62	->	65	-0.04	0.03
62	->	66	-0.13	0.06
62	->	67	-0.20	0.16
62	->	68	-1.42	0.52
62	->	69	-1.30	0.29
62	->	70	-1.10	0.47
62	->	71	-0.02	0.01
62	->	72	-0.02	0.01
62	->	73	-0.01	0.01
62	->	74	0.00	0.00
62	->	75	-0.01	0.01
62	->	76	-2.16	1.69
62	->	77	-0.02	0.01
62	->	78	0.00	0.01
62	->	79	-0.08	0.02
62	->	80	-0.03	0.02

62	->	81	-0.01	0.00
62	->	82	-0.00	0.00
62	->	83	-0.04	0.01
62	->	84	-0.00	0.00
62	->	85	-0.00	0.00
62	->	86	-0.01	0.00
62	->	87	-0.00	0.00
62	->	88	-0.00	0.00
62	->	89	0.00	0.00
62	->	90	0.00	0.00
62	->	91	0.00	0.00
62	->	92	0.00	0.00
62	->	93	0.00	0.00
62	->	94	0.00	0.00
62	->	95	0.00	0.00
62	->	96	0.00	0.00
62	->	97	0.00	0.00
62	->	98	0.00	0.00
62	->	99	0.00	0.00
62	->	100	0.00	0.00
62	->	101	0.00	0.01
62	->	102	0.00	0.00
62	->	103	0.00	0.00
62	->	104	0.00	0.00
62	->	105	0.00	0.00
62	->	106	0.00	0.00
63	->	1	-0.01	0.00
63	->	2	-0.00	0.00
63	->	3	0.00	0.00
63	->	4	0.00	0.00
63	->	5	0.00	0.00
63	->	6	-0.00	0.00
63	->	7	0.00	0.00
63	->	8	0.00	0.00
63	->	9	0.00	0.00
63	->	10	0.00	0.00
63	->	11	0.00	0.00
63	->	12	-0.00	0.00
63	->	13	-0.00	0.00
63	->	14	-0.00	0.00
63	->	15	-0.00	0.00
63	->	16	0.00	0.00
63	->	17	0.00	0.00
63	->	18	-0.00	0.00
63	->	19	0.00	0.00
63	->	20	0.00	0.00
63	->	21	-0.00	0.00
63	->	22	-0.01	0.00
63	->	23	-0.00	0.00
63	->	24	-0.00	0.00

63	->	25	-0.00	0.00
63	->	26	-0.00	0.00
63	->	27	-0.00	0.00
63	->	28	-0.00	0.00
63	->	29	-0.00	0.00
63	->	30	0.00	0.00
63	->	31	-0.00	0.00
63	->	32	0.00	0.00
63	->	33	-0.00	0.00
63	->	34	0.00	0.00
63	->	35	0.01	0.00
63	->	36	-0.00	0.00
63	->	37	-0.00	0.00
63	->	38	0.01	0.00
63	->	39	-0.00	0.00
63	->	40	-0.01	0.00
63	->	41	0.00	0.00
63	->	42	-0.00	0.00
63	->	43	-0.00	0.00
63	->	44	-0.01	0.00
63	->	45	0.00	0.00
63	->	46	0.01	0.00
63	->	47	0.00	0.00
63	->	48	0.00	0.00
63	->	49	0.00	0.00
63	->	50	-0.00	0.00
63	->	51	-0.00	0.00
63	->	52	0.00	0.00
63	->	53	-0.03	0.00
63	->	54	0.00	0.00
63	->	55	-0.00	0.00
63	->	56	0.00	0.00
63	->	57	0.00	0.00
63	->	58	0.02	0.00
63	->	59	-0.08	0.01
63	->	60	-0.02	0.00
63	->	61	-0.38	0.06
63	->	62	-11.93	0.34
63	->	63	-27.74	1.89
63	->	64	-14.46	1.39
63	->	65	1.34	1.67
63	->	66	2.66	0.40
63	->	67	-1.32	0.77
63	->	68	-2.42	0.78
63	->	69	-0.59	0.19
63	->	70	-0.84	0.31
63	->	71	0.00	0.01
63	->	72	-0.04	0.01
63	->	73	-0.00	0.01
63	->	74	0.04	0.01

63	->	75	-0.01	0.02
63	->	76	-1.63	0.91
63	->	77	-0.02	0.02
63	->	78	-0.17	0.05
63	->	79	2.75	0.74
63	->	80	0.14	0.23
63	->	81	-0.01	0.02
63	->	82	-0.08	0.03
63	->	83	2.20	0.26
63	->	84	0.06	0.01
63	->	85	0.01	0.00
63	->	86	0.04	0.01
63	->	87	0.01	0.00
63	->	88	0.01	0.00
63	->	89	0.01	0.00
63	->	90	0.01	0.00
63	->	91	0.00	0.00
63	->	92	0.00	0.00
63	->	93	0.01	0.00
63	->	94	0.01	0.00
63	->	95	0.00	0.00
63	->	96	0.00	0.00
63	->	97	0.00	0.00
63	->	98	0.00	0.00
63	->	99	0.01	0.00
63	->	100	0.02	0.01
63	->	101	0.04	0.01
63	->	102	0.03	0.01
63	->	103	0.02	0.00
63	->	104	0.01	0.00
63	->	105	0.01	0.00
63	->	106	0.00	0.00
64	->	1	-0.01	0.00
64	->	2	-0.00	0.00
64	->	3	0.00	0.00
64	->	4	0.00	0.00
64	->	5	0.00	0.00
64	->	6	0.00	0.00
64	->	7	0.00	0.00
64	->	8	0.00	0.00
64	->	9	0.00	0.00
64	->	10	0.00	0.00
64	->	11	0.00	0.00
64	->	12	-0.00	0.00
64	->	13	-0.00	0.00
64	->	14	-0.00	0.00
64	->	15	-0.00	0.00
64	->	16	0.00	0.00
64	->	17	0.00	0.00
64	->	18	-0.00	0.00

64	->	19	0.00	0.00
64	->	20	0.00	0.00
64	->	21	-0.00	0.00
64	->	22	-0.00	0.00
64	->	23	-0.00	0.00
64	->	24	-0.00	0.00
64	->	25	-0.00	0.00
64	->	26	-0.00	0.00
64	->	27	-0.00	0.00
64	->	28	0.00	0.00
64	->	29	-0.00	0.00
64	->	30	0.00	0.00
64	->	31	-0.00	0.00
64	->	32	-0.00	0.00
64	->	33	-0.00	0.00
64	->	34	0.00	0.00
64	->	35	0.00	0.00
64	->	36	-0.00	0.00
64	->	37	-0.00	0.00
64	->	38	0.00	0.00
64	->	39	-0.00	0.00
64	->	40	-0.00	0.00
64	->	41	0.00	0.00
64	->	42	-0.00	0.00
64	->	43	0.00	0.00
64	->	44	-0.01	0.00
64	->	45	0.00	0.00
64	->	46	0.01	0.00
64	->	47	0.00	0.00
64	->	48	0.00	0.00
64	->	49	0.00	0.00
64	->	50	0.00	0.00
64	->	51	-0.00	0.00
64	->	52	0.00	0.00
64	->	53	-0.02	0.00
64	->	54	0.00	0.00
64	->	55	-0.00	0.00
64	->	56	0.00	0.00
64	->	57	0.00	0.00
64	->	58	0.01	0.00
64	->	59	-0.03	0.00
64	->	60	-0.00	0.00
64	->	61	-0.05	0.01
64	->	62	-0.46	0.09
64	->	63	-14.58	1.41
64	->	64	-34.98	2.36
64	->	65	-12.51	0.46
64	->	66	-0.37	0.31
64	->	67	-0.41	0.38
64	->	68	-0.04	0.02

64	->	69	-0.08	0.02
64	->	70	-0.01	0.02
64	->	71	0.00	0.00
64	->	72	-0.02	0.00
64	->	73	-0.00	0.00
64	->	74	0.02	0.00
64	->	75	0.02	0.00
64	->	76	-1.30	1.76
64	->	77	-0.04	0.02
64	->	78	-0.03	0.01
64	->	79	-0.05	0.07
64	->	80	-0.10	0.14
64	->	81	-0.03	0.01
64	->	82	-0.01	0.00
64	->	83	-0.15	0.10
64	->	84	0.04	0.01
64	->	85	0.00	0.00
64	->	86	0.02	0.00
64	->	87	0.01	0.00
64	->	88	0.01	0.00
64	->	89	0.00	0.00
64	->	90	0.00	0.00
64	->	91	0.00	0.00
64	->	92	0.00	0.00
64	->	93	0.01	0.00
64	->	94	0.01	0.00
64	->	95	0.00	0.00
64	->	96	0.00	0.00
64	->	97	0.00	0.00
64	->	98	0.00	0.00
64	->	99	0.01	0.00
64	->	100	0.02	0.01
64	->	101	0.03	0.01
64	->	102	0.02	0.01
64	->	103	0.01	0.00
64	->	104	0.01	0.00
64	->	105	0.01	0.00
64	->	106	0.00	0.00
65	->	1	-0.00	0.00
65	->	2	0.00	0.00
65	->	3	0.00	0.00
65	->	4	0.00	0.00
65	->	5	0.00	0.00
65	->	6	0.00	0.00
65	->	7	0.00	0.00
65	->	8	0.00	0.00
65	->	9	0.00	0.00
65	->	10	0.00	0.00
65	->	11	0.00	0.00
65	->	12	-0.00	0.00

65	->	13	-0.00	0.00
65	->	14	0.00	0.00
65	->	15	0.00	0.00
65	->	16	0.00	0.00
65	->	17	0.00	0.00
65	->	18	-0.00	0.00
65	->	19	0.00	0.00
65	->	20	0.00	0.00
65	->	21	-0.00	0.00
65	->	22	-0.00	0.00
65	->	23	0.00	0.00
65	->	24	0.00	0.00
65	->	25	-0.00	0.00
65	->	26	0.00	0.00
65	->	27	0.00	0.00
65	->	28	0.00	0.00
65	->	29	0.00	0.00
65	->	30	0.00	0.00
65	->	31	-0.00	0.00
65	->	32	0.00	0.00
65	->	33	0.00	0.00
65	->	34	0.00	0.00
65	->	35	0.00	0.00
65	->	36	-0.00	0.00
65	->	37	-0.00	0.00
65	->	38	0.00	0.00
65	->	39	0.00	0.00
65	->	40	-0.00	0.00
65	->	41	0.00	0.00
65	->	42	-0.00	0.00
65	->	43	-0.00	0.00
65	->	44	-0.00	0.00
65	->	45	0.00	0.00
65	->	46	0.00	0.00
65	->	47	0.00	0.00
65	->	48	0.00	0.00
65	->	49	0.00	0.00
65	->	50	-0.00	0.00
65	->	51	-0.00	0.00
65	->	52	0.00	0.00
65	->	53	-0.00	0.00
65	->	54	0.00	0.00
65	->	55	-0.00	0.00
65	->	56	0.00	0.00
65	->	57	0.00	0.00
65	->	58	0.00	0.00
65	->	59	-0.00	0.00
65	->	60	-0.00	0.00
65	->	61	-0.01	0.00
65	->	62	-0.04	0.03

65	->	63	1.27	1.68
65	->	64	-12.70	0.47
65	->	65	22.61	1.52
65	->	66	-8.46	0.97
65	->	67	-0.42	0.18
65	->	68	-0.24	0.06
65	->	69	-0.01	0.00
65	->	70	-0.02	0.01
65	->	71	-0.00	0.00
65	->	72	-0.00	0.00
65	->	73	-0.00	0.00
65	->	74	0.00	0.00
65	->	75	-0.00	0.00
65	->	76	-0.05	0.03
65	->	77	-0.01	0.00
65	->	78	-0.02	0.00
65	->	79	0.70	0.11
65	->	80	-0.12	0.15
65	->	81	-0.01	0.01
65	->	82	-0.05	0.02
65	->	83	0.52	0.74
65	->	84	-0.06	0.09
65	->	85	-0.00	0.00
65	->	86	0.00	0.00
65	->	87	0.00	0.00
65	->	88	0.00	0.00
65	->	89	0.00	0.00
65	->	90	0.00	0.00
65	->	91	0.00	0.00
65	->	92	0.00	0.00
65	->	93	0.00	0.00
65	->	94	0.00	0.00
65	->	95	0.00	0.00
65	->	96	0.00	0.00
65	->	97	0.00	0.00
65	->	98	0.00	0.00
65	->	99	0.00	0.00
65	->	100	0.00	0.00
65	->	101	-0.00	0.00
65	->	102	-0.00	0.00
65	->	103	0.00	0.00
65	->	104	0.00	0.00
65	->	105	0.00	0.00
65	->	106	0.00	0.00
66	->	1	-0.00	0.00
66	->	2	-0.00	0.00
66	->	3	0.00	0.00
66	->	4	-0.00	0.00
66	->	5	0.00	0.00
66	->	6	-0.00	0.00

66	->	7	0.00	0.00
66	->	8	0.00	0.00
66	->	9	0.00	0.00
66	->	10	0.00	0.00
66	->	11	0.00	0.00
66	->	12	-0.00	0.00
66	->	13	-0.00	0.00
66	->	14	-0.00	0.00
66	->	15	-0.00	0.00
66	->	16	0.00	0.00
66	->	17	0.00	0.00
66	->	18	-0.00	0.00
66	->	19	0.00	0.00
66	->	20	0.00	0.00
66	->	21	-0.00	0.00
66	->	22	-0.00	0.00
66	->	23	-0.00	0.00
66	->	24	-0.00	0.00
66	->	25	-0.00	0.00
66	->	26	-0.00	0.00
66	->	27	-0.00	0.00
66	->	28	-0.00	0.00
66	->	29	-0.00	0.00
66	->	30	0.00	0.00
66	->	31	-0.00	0.00
66	->	32	0.00	0.00
66	->	33	-0.00	0.00
66	->	34	0.00	0.00
66	->	35	0.00	0.00
66	->	36	-0.00	0.00
66	->	37	-0.00	0.00
66	->	38	0.01	0.00
66	->	39	0.00	0.00
66	->	40	-0.01	0.00
66	->	41	0.00	0.00
66	->	42	-0.00	0.00
66	->	43	-0.00	0.00
66	->	44	-0.01	0.00
66	->	45	0.00	0.00
66	->	46	0.01	0.00
66	->	47	0.00	0.00
66	->	48	0.00	0.00
66	->	49	0.00	0.00
66	->	50	0.00	0.00
66	->	51	-0.00	0.00
66	->	52	0.00	0.00
66	->	53	-0.02	0.00
66	->	54	0.00	0.00
66	->	55	-0.00	0.00
66	->	56	0.00	0.00

66	->	57	0.00	0.00
66	->	58	0.02	0.00
66	->	59	-0.06	0.01
66	->	60	0.00	0.00
66	->	61	-0.01	0.00
66	->	62	-0.13	0.06
66	->	63	2.67	0.40
66	->	64	-0.38	0.32
66	->	65	-8.38	0.97
66	->	66	-32.96	2.83
66	->	67	-8.98	0.47
66	->	68	-3.42	0.61
66	->	69	-0.03	0.04
66	->	70	0.03	0.03
66	->	71	-0.00	0.00
66	->	72	-0.02	0.00
66	->	73	0.00	0.00
66	->	74	0.03	0.00
66	->	75	0.05	0.01
66	->	76	-0.09	0.03
66	->	77	-0.03	0.01
66	->	78	-0.08	0.01
66	->	79	3.41	0.72
66	->	80	0.03	0.02
66	->	81	-0.03	0.01
66	->	82	-0.07	0.04
66	->	83	1.95	0.25
66	->	84	0.05	0.00
66	->	85	0.02	0.01
66	->	86	0.05	0.01
66	->	87	0.02	0.00
66	->	88	0.01	0.00
66	->	89	0.01	0.00
66	->	90	0.00	0.00
66	->	91	0.00	0.00
66	->	92	0.00	0.00
66	->	93	0.00	0.00
66	->	94	0.00	0.00
66	->	95	0.00	0.00
66	->	96	0.00	0.00
66	->	97	0.00	0.00
66	->	98	0.00	0.00
66	->	99	0.01	0.00
66	->	100	0.01	0.00
66	->	101	0.02	0.01
66	->	102	0.02	0.00
66	->	103	0.02	0.00
66	->	104	0.01	0.00
66	->	105	0.01	0.00
66	->	106	0.00	0.00

67	->	1	0.00	0.00
67	->	2	0.00	0.00
67	->	3	0.00	0.00
67	->	4	0.00	0.00
67	->	5	0.00	0.00
67	->	6	0.00	0.00
67	->	7	0.00	0.00
67	->	8	0.00	0.00
67	->	9	0.00	0.00
67	->	10	0.00	0.00
67	->	11	-0.00	0.00
67	->	12	0.00	0.00
67	->	13	0.00	0.00
67	->	14	0.00	0.00
67	->	15	0.00	0.00
67	->	16	-0.00	0.00
67	->	17	0.00	0.00
67	->	18	0.00	0.00
67	->	19	0.00	0.00
67	->	20	0.00	0.00
67	->	21	0.00	0.00
67	->	22	-0.00	0.00
67	->	23	0.00	0.00
67	->	24	0.00	0.00
67	->	25	-0.00	0.00
67	->	26	0.00	0.00
67	->	27	0.00	0.00
67	->	28	0.00	0.00
67	->	29	0.00	0.00
67	->	30	0.00	0.00
67	->	31	-0.00	0.00
67	->	32	0.00	0.00
67	->	33	0.00	0.00
67	->	34	0.00	0.00
67	->	35	0.00	0.00
67	->	36	-0.00	0.00
67	->	37	-0.00	0.00
67	->	38	0.00	0.00
67	->	39	-0.00	0.00
67	->	40	-0.00	0.00
67	->	41	0.00	0.00
67	->	42	0.00	0.00
67	->	43	-0.00	0.00
67	->	44	-0.00	0.00
67	->	45	0.00	0.00
67	->	46	0.00	0.00
67	->	47	0.00	0.00
67	->	48	0.00	0.00
67	->	49	0.00	0.00
67	->	50	0.00	0.00

67	->	51	0.00	0.00
67	->	52	0.00	0.00
67	->	53	-0.00	0.00
67	->	54	0.00	0.00
67	->	55	0.00	0.00
67	->	56	0.00	0.00
67	->	57	-0.00	0.00
67	->	58	0.00	0.00
67	->	59	-0.01	0.01
67	->	60	-0.00	0.00
67	->	61	-0.02	0.01
67	->	62	-0.20	0.16
67	->	63	-1.32	0.78
67	->	64	-0.41	0.39
67	->	65	-0.42	0.18
67	->	66	-9.06	0.48
67	->	67	11.75	0.77
67	->	68	-8.82	0.33
67	->	69	-1.02	0.74
67	->	70	-0.11	0.03
67	->	71	-0.00	0.00
67	->	72	-0.00	0.00
67	->	73	-0.00	0.00
67	->	74	-0.00	0.00
67	->	75	0.00	0.00
67	->	76	-0.01	0.01
67	->	77	-0.00	0.00
67	->	78	0.00	0.00
67	->	79	-0.09	0.05
67	->	80	-0.01	0.00
67	->	81	-0.00	0.00
67	->	82	-0.00	0.00
67	->	83	-0.04	0.04
67	->	84	-0.00	0.00
67	->	85	-0.00	0.00
67	->	86	-0.00	0.00
67	->	87	-0.00	0.00
67	->	88	0.00	0.00
67	->	89	0.00	0.00
67	->	90	0.00	0.00
67	->	91	0.00	0.00
67	->	92	-0.00	0.00
67	->	93	0.00	0.00
67	->	94	-0.00	0.00
67	->	95	0.00	0.00
67	->	96	0.00	0.00
67	->	97	0.00	0.00
67	->	98	0.00	0.00
67	->	99	0.00	0.00
67	->	100	0.00	0.00

67	->	101	-0.00	0.00
67	->	102	-0.00	0.00
67	->	103	-0.00	0.00
67	->	104	-0.00	0.00
67	->	105	-0.00	0.00
67	->	106	-0.00	0.00
68	->	1	0.01	0.00
68	->	2	0.00	0.00
68	->	3	-0.00	0.00
68	->	4	0.00	0.00
68	->	5	-0.00	0.00
68	->	6	0.00	0.00
68	->	7	0.00	0.00
68	->	8	-0.00	0.00
68	->	9	-0.00	0.00
68	->	10	-0.00	0.00
68	->	11	-0.00	0.00
68	->	12	0.01	0.00
68	->	13	0.00	0.00
68	->	14	0.00	0.00
68	->	15	0.00	0.00
68	->	16	-0.00	0.00
68	->	17	-0.00	0.00
68	->	18	0.00	0.00
68	->	19	-0.00	0.00
68	->	20	-0.00	0.00
68	->	21	0.00	0.00
68	->	22	0.01	0.00
68	->	23	0.00	0.00
68	->	24	-0.00	0.00
68	->	25	0.00	0.00
68	->	26	0.00	0.00
68	->	27	0.00	0.00
68	->	28	0.00	0.00
68	->	29	0.00	0.00
68	->	30	-0.00	0.00
68	->	31	0.00	0.00
68	->	32	0.00	0.00
68	->	33	0.00	0.00
68	->	34	-0.00	0.00
68	->	35	-0.01	0.00
68	->	36	0.01	0.00
68	->	37	0.00	0.00
68	->	38	-0.01	0.00
68	->	39	-0.00	0.00
68	->	40	0.01	0.00
68	->	41	-0.00	0.00
68	->	42	0.00	0.00
68	->	43	0.00	0.00
68	->	44	0.01	0.00

68	->	45	-0.00	0.00
68	->	46	-0.01	0.00
68	->	47	-0.00	0.00
68	->	48	-0.00	0.00
68	->	49	-0.00	0.00
68	->	50	-0.00	0.00
68	->	51	-0.00	0.00
68	->	52	-0.00	0.00
68	->	53	0.03	0.01
68	->	54	-0.01	0.00
68	->	55	-0.00	0.00
68	->	56	-0.00	0.00
68	->	57	-0.00	0.00
68	->	58	-0.03	0.01
68	->	59	0.17	0.17
68	->	60	-0.02	0.01
68	->	61	-0.30	0.06
68	->	62	-1.41	0.53
68	->	63	-2.43	0.79
68	->	64	-0.04	0.02
68	->	65	-0.24	0.06
68	->	66	-3.53	0.62
68	->	67	-8.94	0.32
68	->	68	-105.15	4.06
68	->	69	-13.70	1.50
68	->	70	-3.69	0.70
68	->	71	-0.07	0.03
68	->	72	0.03	0.01
68	->	73	-0.01	0.01
68	->	74	-0.06	0.01
68	->	75	-2.04	3.34
68	->	76	-0.11	0.06
68	->	77	0.01	0.01
68	->	78	0.11	0.04
68	->	79	-2.01	0.65
68	->	80	-0.04	0.01
68	->	81	0.03	0.01
68	->	82	-0.04	0.05
68	->	83	-0.22	0.06
68	->	84	-0.03	0.00
68	->	85	-1.93	1.87
68	->	86	-3.11	2.67
68	->	87	-0.08	0.08
68	->	88	-0.03	0.01
68	->	89	-0.01	0.00
68	->	90	-0.01	0.00
68	->	91	-0.01	0.00
68	->	92	-0.00	0.00
68	->	93	-0.01	0.00
68	->	94	-0.00	0.00

68	->	95	-0.00	0.00
68	->	96	-0.00	0.00
68	->	97	-0.00	0.00
68	->	98	-0.00	0.00
68	->	99	-0.01	0.00
68	->	100	-0.02	0.01
68	->	101	-0.02	0.01
68	->	102	-0.02	0.01
68	->	103	-0.02	0.01
68	->	104	-0.01	0.01
68	->	105	-0.02	0.01
68	->	106	-0.01	0.00
69	->	1	0.00	0.00
69	->	2	0.00	0.00
69	->	3	-0.00	0.00
69	->	4	0.00	0.00
69	->	5	-0.00	0.00
69	->	6	0.00	0.00
69	->	7	0.00	0.00
69	->	8	-0.00	0.00
69	->	9	0.00	0.00
69	->	10	-0.00	0.00
69	->	11	-0.00	0.00
69	->	12	0.00	0.00
69	->	13	0.00	0.00
69	->	14	0.00	0.00
69	->	15	0.00	0.00
69	->	16	-0.00	0.00
69	->	17	-0.00	0.00
69	->	18	0.00	0.00
69	->	19	-0.00	0.00
69	->	20	-0.00	0.00
69	->	21	0.00	0.00
69	->	22	0.01	0.00
69	->	23	0.00	0.00
69	->	24	0.00	0.00
69	->	25	0.00	0.00
69	->	26	0.00	0.00
69	->	27	0.00	0.00
69	->	28	0.00	0.00
69	->	29	0.00	0.00
69	->	30	-0.00	0.00
69	->	31	0.00	0.00
69	->	32	-0.00	0.00
69	->	33	0.00	0.00
69	->	34	-0.00	0.00
69	->	35	-0.01	0.00
69	->	36	0.01	0.01
69	->	37	0.00	0.00
69	->	38	-0.01	0.00

69	->	39	0.00	0.00
69	->	40	0.01	0.00
69	->	41	-0.00	0.00
69	->	42	0.00	0.00
69	->	43	0.00	0.00
69	->	44	0.01	0.00
69	->	45	-0.00	0.00
69	->	46	-0.02	0.00
69	->	47	-0.00	0.00
69	->	48	-0.00	0.00
69	->	49	-0.00	0.00
69	->	50	-0.01	0.00
69	->	51	-0.00	0.00
69	->	52	0.00	0.00
69	->	53	0.08	0.02
69	->	54	-0.01	0.00
69	->	55	-0.00	0.00
69	->	56	-0.01	0.00
69	->	57	-0.02	0.01
69	->	58	-0.07	0.09
69	->	59	-2.05	0.72
69	->	60	-0.53	0.73
69	->	61	-2.80	0.53
69	->	62	-1.33	0.29
69	->	63	-0.59	0.19
69	->	64	-0.08	0.02
69	->	65	-0.01	0.00
69	->	66	-0.03	0.04
69	->	67	-1.08	0.74
69	->	68	-13.69	1.51
69	->	69	9.93	2.50
69	->	70	-10.51	0.60
69	->	71	-0.42	0.06
69	->	72	-0.00	0.03
69	->	73	-0.04	0.02
69	->	74	-0.05	0.01
69	->	75	-0.21	0.20
69	->	76	0.08	0.04
69	->	77	-0.01	0.01
69	->	78	0.04	0.01
69	->	79	-0.02	0.01
69	->	80	-0.01	0.01
69	->	81	0.01	0.00
69	->	82	-0.00	0.00
69	->	83	-0.01	0.01
69	->	84	-0.01	0.00
69	->	85	-0.04	0.04
69	->	86	-0.14	0.12
69	->	87	-0.04	0.01
69	->	88	-0.02	0.00

69	->	89	-0.01	0.00
69	->	90	-0.01	0.00
69	->	91	-0.00	0.00
69	->	92	-0.00	0.00
69	->	93	-0.01	0.00
69	->	94	-0.01	0.00
69	->	95	-0.00	0.00
69	->	96	-0.00	0.00
69	->	97	-0.00	0.00
69	->	98	-0.01	0.00
69	->	99	-0.02	0.01
69	->	100	-0.03	0.02
69	->	101	-0.04	0.02
69	->	102	-0.02	0.00
69	->	103	-0.01	0.00
69	->	104	-0.01	0.00
69	->	105	-0.01	0.00
69	->	106	-0.00	0.00
70	->	1	0.00	0.00
70	->	2	0.00	0.00
70	->	3	-0.00	0.00
70	->	4	0.00	0.00
70	->	5	-0.00	0.00
70	->	6	0.00	0.00
70	->	7	0.00	0.00
70	->	8	-0.00	0.00
70	->	9	-0.00	0.00
70	->	10	-0.00	0.00
70	->	11	-0.00	0.00
70	->	12	0.00	0.00
70	->	13	0.00	0.00
70	->	14	0.00	0.00
70	->	15	0.00	0.00
70	->	16	-0.00	0.00
70	->	17	-0.00	0.00
70	->	18	-0.00	0.00
70	->	19	0.00	0.00
70	->	20	0.00	0.00
70	->	21	0.00	0.00
70	->	22	0.00	0.00
70	->	23	0.00	0.00
70	->	24	-0.00	0.00
70	->	25	0.00	0.00
70	->	26	0.00	0.00
70	->	27	0.00	0.00
70	->	28	0.00	0.00
70	->	29	0.00	0.00
70	->	30	-0.00	0.00
70	->	31	0.00	0.00
70	->	32	0.00	0.00

70	->	33	-0.00	0.00
70	->	34	0.00	0.00
70	->	35	-0.00	0.00
70	->	36	-0.00	0.00
70	->	37	-0.00	0.00
70	->	38	-0.00	0.00
70	->	39	-0.00	0.00
70	->	40	0.00	0.00
70	->	41	-0.00	0.00
70	->	42	-0.00	0.00
70	->	43	0.00	0.00
70	->	44	0.00	0.00
70	->	45	0.00	0.00
70	->	46	-0.00	0.00
70	->	47	-0.00	0.00
70	->	48	0.00	0.00
70	->	49	0.00	0.00
70	->	50	-0.02	0.01
70	->	51	-0.01	0.00
70	->	52	0.00	0.00
70	->	53	0.02	0.05
70	->	54	-0.03	0.01
70	->	55	-0.01	0.00
70	->	56	-0.00	0.00
70	->	57	-0.00	0.01
70	->	58	-0.26	0.07
70	->	59	-3.22	0.40
70	->	60	-0.87	0.17
70	->	61	-3.73	0.45
70	->	62	-1.10	0.47
70	->	63	-0.83	0.31
70	->	64	-0.01	0.02
70	->	65	-0.02	0.01
70	->	66	0.03	0.03
70	->	67	-0.11	0.03
70	->	68	-3.64	0.69
70	->	69	-10.57	0.61
70	->	70	29.82	1.07
70	->	71	-9.78	0.38
70	->	72	-1.01	0.18
70	->	73	-0.22	0.05
70	->	74	-0.13	0.03
70	->	75	-3.66	1.40
70	->	76	-2.87	0.49
70	->	77	-0.05	0.02
70	->	78	-0.24	0.07
70	->	79	-1.85	0.35
70	->	80	-0.05	0.03
70	->	81	-0.01	0.00
70	->	82	-0.03	0.01

70	->	83	-0.01	0.01
70	->	84	-0.00	0.00
70	->	85	-0.12	0.22
70	->	86	-0.28	0.29
70	->	87	-0.08	0.04
70	->	88	-0.01	0.01
70	->	89	-0.00	0.00
70	->	90	-0.00	0.00
70	->	91	-0.00	0.00
70	->	92	-0.00	0.00
70	->	93	-0.00	0.00
70	->	94	-0.00	0.00
70	->	95	0.00	0.00
70	->	96	0.00	0.00
70	->	97	0.00	0.00
70	->	98	-0.00	0.00
70	->	99	-0.00	0.00
70	->	100	-0.01	0.01
70	->	101	-0.02	0.02
70	->	102	-0.01	0.00
70	->	103	-0.01	0.00
70	->	104	-0.00	0.00
70	->	105	-0.00	0.00
70	->	106	-0.00	0.00
71	->	1	-0.00	0.00
71	->	2	0.00	0.00
71	->	3	0.00	0.00
71	->	4	0.00	0.00
71	->	5	0.00	0.00
71	->	6	0.00	0.00
71	->	7	0.00	0.00
71	->	8	-0.00	0.00
71	->	9	-0.00	0.00
71	->	10	0.00	0.00
71	->	11	-0.00	0.00
71	->	12	0.00	0.00
71	->	13	-0.00	0.00
71	->	14	0.00	0.00
71	->	15	0.00	0.00
71	->	16	0.00	0.00
71	->	17	0.00	0.00
71	->	18	0.00	0.00
71	->	19	0.00	0.00
71	->	20	-0.00	0.00
71	->	21	0.00	0.00
71	->	22	-0.00	0.00
71	->	23	-0.00	0.00
71	->	24	-0.00	0.00
71	->	25	-0.00	0.00
71	->	26	-0.00	0.00

71	->	27	-0.00	0.00
71	->	28	0.00	0.00
71	->	29	0.00	0.00
71	->	30	0.00	0.00
71	->	31	-0.00	0.00
71	->	32	0.00	0.00
71	->	33	-0.00	0.00
71	->	34	0.00	0.00
71	->	35	-0.00	0.00
71	->	36	-0.00	0.00
71	->	37	-0.00	0.00
71	->	38	-0.00	0.00
71	->	39	0.00	0.00
71	->	40	-0.00	0.00
71	->	41	0.00	0.00
71	->	42	-0.00	0.00
71	->	43	0.00	0.00
71	->	44	0.00	0.00
71	->	45	0.00	0.00
71	->	46	-0.01	0.00
71	->	47	-0.01	0.00
71	->	48	-0.01	0.00
71	->	49	-0.05	0.03
71	->	50	-0.43	0.27
71	->	51	-0.02	0.01
71	->	52	-0.02	0.01
71	->	53	-3.35	1.49
71	->	54	-0.30	0.30
71	->	55	-0.01	0.00
71	->	56	-0.02	0.00
71	->	57	-0.04	0.01
71	->	58	-0.71	0.24
71	->	59	-1.74	0.39
71	->	60	-0.79	0.19
71	->	61	-0.98	0.30
71	->	62	-0.02	0.01
71	->	63	0.00	0.01
71	->	64	0.00	0.00
71	->	65	-0.00	0.00
71	->	66	-0.00	0.00
71	->	67	-0.00	0.00
71	->	68	-0.07	0.03
71	->	69	-0.42	0.06
71	->	70	-9.78	0.36
71	->	71	23.77	1.07
71	->	72	-7.87	0.45
71	->	73	-0.32	0.07
71	->	74	-0.18	0.06
71	->	75	-2.55	2.18
71	->	76	-0.03	0.02

71	->	77	-0.01	0.00
71	->	78	-0.00	0.01
71	->	79	-0.00	0.00
71	->	80	-0.00	0.00
71	->	81	0.00	0.00
71	->	82	-0.00	0.00
71	->	83	-0.00	0.00
71	->	84	-0.00	0.00
71	->	85	-0.00	0.01
71	->	86	-0.07	0.05
71	->	87	-0.14	0.11
71	->	88	-0.03	0.01
71	->	89	-0.01	0.00
71	->	90	-0.00	0.00
71	->	91	0.00	0.00
71	->	92	0.00	0.00
71	->	93	-0.00	0.00
71	->	94	0.00	0.00
71	->	95	0.00	0.00
71	->	96	0.00	0.00
71	->	97	0.00	0.00
71	->	98	0.00	0.00
71	->	99	-0.00	0.00
71	->	100	-0.01	0.02
71	->	101	-0.03	0.02
71	->	102	-0.01	0.00
71	->	103	0.00	0.00
71	->	104	-0.00	0.00
71	->	105	-0.00	0.00
71	->	106	-0.00	0.00
72	->	1	0.02	0.00
72	->	2	0.00	0.00
72	->	3	-0.00	0.00
72	->	4	0.00	0.00
72	->	5	-0.01	0.00
72	->	6	0.00	0.00
72	->	7	0.00	0.00
72	->	8	-0.00	0.00
72	->	9	-0.00	0.00
72	->	10	-0.01	0.00
72	->	11	-0.01	0.00
72	->	12	0.02	0.01
72	->	13	0.01	0.00
72	->	14	0.00	0.00
72	->	15	0.00	0.00
72	->	16	-0.01	0.00
72	->	17	-0.00	0.00
72	->	18	0.00	0.00
72	->	19	-0.00	0.00
72	->	20	-0.00	0.00

72	->	21	0.00	0.00
72	->	22	0.04	0.00
72	->	23	0.00	0.00
72	->	24	0.00	0.00
72	->	25	0.02	0.00
72	->	26	0.00	0.00
72	->	27	0.00	0.00
72	->	28	0.00	0.00
72	->	29	0.00	0.00
72	->	30	-0.00	0.00
72	->	31	0.02	0.01
72	->	32	0.00	0.00
72	->	33	-0.00	0.00
72	->	34	-0.00	0.00
72	->	35	-0.03	0.00
72	->	36	0.01	0.00
72	->	37	0.00	0.00
72	->	38	-0.03	0.00
72	->	39	-0.00	0.00
72	->	40	0.05	0.02
72	->	41	-0.00	0.00
72	->	42	-0.01	0.00
72	->	43	0.00	0.00
72	->	44	0.09	0.02
72	->	45	-0.00	0.00
72	->	46	-0.11	0.03
72	->	47	-0.12	0.05
72	->	48	-0.01	0.01
72	->	49	0.03	0.02
72	->	50	-1.47	0.36
72	->	51	-0.07	0.03
72	->	52	-0.02	0.01
72	->	53	-0.36	0.25
72	->	54	-0.16	0.19
72	->	55	-0.01	0.01
72	->	56	-0.00	0.00
72	->	57	-0.01	0.00
72	->	58	-0.04	0.01
72	->	59	-0.04	0.04
72	->	60	-0.13	0.08
72	->	61	-1.83	0.76
72	->	62	-0.02	0.01
72	->	63	-0.04	0.01
72	->	64	-0.02	0.00
72	->	65	-0.00	0.00
72	->	66	-0.02	0.00
72	->	67	-0.00	0.00
72	->	68	0.03	0.01
72	->	69	-0.00	0.03
72	->	70	-1.01	0.18

72	->	71	-7.95	0.45
72	->	72	-94.32	2.41
72	->	73	-21.56	0.50
72	->	74	-14.22	2.28
72	->	75	-4.44	1.40
72	->	76	-0.85	0.37
72	->	77	0.08	0.04
72	->	78	0.33	0.29
72	->	79	-0.01	0.01
72	->	80	-0.01	0.01
72	->	81	0.04	0.01
72	->	82	-0.00	0.00
72	->	83	-0.00	0.00
72	->	84	-0.01	0.00
72	->	85	-0.04	0.01
72	->	86	-0.23	0.07
72	->	87	-2.69	0.78
72	->	88	-8.75	0.85
72	->	89	-2.05	0.78
72	->	90	-0.12	0.07
72	->	91	-0.01	0.03
72	->	92	-0.01	0.01
72	->	93	-0.02	0.00
72	->	94	-0.02	0.00
72	->	95	-0.01	0.00
72	->	96	-0.00	0.00
72	->	97	-0.01	0.00
72	->	98	-0.02	0.00
72	->	99	-0.05	0.01
72	->	100	-0.13	0.07
72	->	101	-0.41	0.24
72	->	102	-0.27	0.15
72	->	103	-0.04	0.79
72	->	104	0.37	0.29
72	->	105	0.12	0.15
72	->	106	-0.05	0.01
73	->	1	0.00	0.00
73	->	2	0.00	0.00
73	->	3	0.00	0.00
73	->	4	0.00	0.00
73	->	5	-0.00	0.00
73	->	6	0.00	0.00
73	->	7	0.00	0.00
73	->	8	-0.00	0.00
73	->	9	0.00	0.00
73	->	10	-0.00	0.00
73	->	11	-0.00	0.00
73	->	12	0.00	0.00
73	->	13	0.00	0.00
73	->	14	0.00	0.00

73	->	15	-0.00	0.00
73	->	16	-0.00	0.00
73	->	17	0.00	0.00
73	->	18	0.00	0.00
73	->	19	-0.00	0.00
73	->	20	-0.00	0.00
73	->	21	0.00	0.00
73	->	22	0.00	0.00
73	->	23	0.00	0.00
73	->	24	0.00	0.00
73	->	25	0.00	0.00
73	->	26	0.00	0.00
73	->	27	0.00	0.00
73	->	28	0.00	0.00
73	->	29	0.00	0.00
73	->	30	-0.00	0.00
73	->	31	0.00	0.00
73	->	32	0.00	0.00
73	->	33	-0.00	0.00
73	->	34	-0.00	0.00
73	->	35	-0.00	0.00
73	->	36	0.00	0.00
73	->	37	0.00	0.00
73	->	38	-0.00	0.00
73	->	39	0.00	0.00
73	->	40	0.00	0.00
73	->	41	-0.00	0.00
73	->	42	-0.00	0.00
73	->	43	0.00	0.00
73	->	44	-0.00	0.01
73	->	45	-0.00	0.00
73	->	46	-0.01	0.00
73	->	47	-0.00	0.00
73	->	48	-0.00	0.00
73	->	49	-0.02	0.01
73	->	50	-0.02	0.04
73	->	51	0.00	0.00
73	->	52	-0.00	0.00
73	->	53	-0.18	0.12
73	->	54	0.00	0.00
73	->	55	-0.00	0.00
73	->	56	-0.00	0.00
73	->	57	-0.00	0.00
73	->	58	-0.01	0.00
73	->	59	-0.06	0.02
73	->	60	-0.05	0.03
73	->	61	-1.81	0.85
73	->	62	-0.01	0.01
73	->	63	-0.00	0.01
73	->	64	-0.00	0.00

73	->	65	-0.00	0.00
73	->	66	0.00	0.00
73	->	67	-0.00	0.00
73	->	68	-0.01	0.01
73	->	69	-0.04	0.02
73	->	70	-0.22	0.05
73	->	71	-0.32	0.07
73	->	72	-21.56	0.50
73	->	73	23.00	2.46
73	->	74	-10.65	0.64
73	->	75	-0.83	0.18
73	->	76	-2.78	0.65
73	->	77	-1.77	0.83
73	->	78	-0.11	0.08
73	->	79	-0.04	0.02
73	->	80	-0.02	0.01
73	->	81	-0.01	0.01
73	->	82	-0.00	0.00
73	->	83	-0.00	0.00
73	->	84	0.00	0.00
73	->	85	-0.00	0.00
73	->	86	-0.00	0.00
73	->	87	-0.01	0.00
73	->	88	-0.01	0.01
73	->	89	-0.01	0.01
73	->	90	-0.00	0.01
73	->	91	-0.00	0.03
73	->	92	-0.02	0.02
73	->	93	-0.01	0.00
73	->	94	-0.00	0.00
73	->	95	-0.00	0.00
73	->	96	-0.00	0.00
73	->	97	-0.00	0.00
73	->	98	-0.00	0.00
73	->	99	-0.01	0.01
73	->	100	-0.54	0.49
73	->	101	-3.46	3.02
73	->	102	-0.48	0.30
73	->	103	-0.07	0.03
73	->	104	-0.01	0.00
73	->	105	-0.00	0.00
73	->	106	-0.00	0.00
74	->	1	-0.03	0.00
74	->	2	-0.00	0.00
74	->	3	0.00	0.00
74	->	4	-0.00	0.00
74	->	5	0.01	0.00
74	->	6	-0.00	0.00
74	->	7	0.00	0.00
74	->	8	0.00	0.00

74	->	9	0.00	0.00
74	->	10	0.01	0.00
74	->	11	0.01	0.00
74	->	12	-0.02	0.01
74	->	13	-0.01	0.00
74	->	14	-0.00	0.00
74	->	15	-0.00	0.00
74	->	16	0.01	0.00
74	->	17	0.00	0.00
74	->	18	-0.00	0.00
74	->	19	0.00	0.00
74	->	20	0.00	0.00
74	->	21	-0.00	0.00
74	->	22	-0.04	0.00
74	->	23	-0.00	0.00
74	->	24	-0.00	0.00
74	->	25	-0.01	0.00
74	->	26	-0.00	0.00
74	->	27	-0.00	0.00
74	->	28	-0.00	0.00
74	->	29	-0.00	0.00
74	->	30	0.00	0.00
74	->	31	-0.01	0.00
74	->	32	-0.00	0.00
74	->	33	0.00	0.00
74	->	34	0.00	0.00
74	->	35	0.02	0.00
74	->	36	-0.01	0.00
74	->	37	-0.00	0.00
74	->	38	0.02	0.00
74	->	39	0.00	0.00
74	->	40	-0.03	0.01
74	->	41	0.00	0.00
74	->	42	-0.00	0.00
74	->	43	-0.00	0.00
74	->	44	-0.08	0.02
74	->	45	-0.00	0.00
74	->	46	0.06	0.01
74	->	47	-0.01	0.02
74	->	48	-0.00	0.00
74	->	49	-0.02	0.01
74	->	50	-0.03	0.02
74	->	51	-0.00	0.00
74	->	52	-0.00	0.00
74	->	53	-0.12	0.05
74	->	54	-0.00	0.00
74	->	55	-0.00	0.00
74	->	56	-0.00	0.00
74	->	57	-0.00	0.00
74	->	58	0.03	0.00

74	->	59	-0.07	0.01
74	->	60	-0.01	0.00
74	->	61	-0.00	0.02
74	->	62	0.00	0.00
74	->	63	0.04	0.01
74	->	64	0.02	0.00
74	->	65	0.00	0.00
74	->	66	0.03	0.00
74	->	67	-0.00	0.00
74	->	68	-0.06	0.01
74	->	69	-0.05	0.01
74	->	70	-0.13	0.03
74	->	71	-0.18	0.06
74	->	72	-14.13	2.27
74	->	73	-10.72	0.65
74	->	74	-14.51	2.64
74	->	75	-13.39	0.62
74	->	76	-1.61	0.33
74	->	77	-2.52	0.42
74	->	78	-2.88	1.68
74	->	79	-0.12	0.06
74	->	80	-0.04	0.02
74	->	81	-0.11	0.04
74	->	82	-0.01	0.01
74	->	83	-0.01	0.00
74	->	84	0.02	0.00
74	->	85	-0.00	0.01
74	->	86	0.01	0.04
74	->	87	-0.50	0.49
74	->	88	0.46	0.18
74	->	89	0.15	0.14
74	->	90	-0.05	0.06
74	->	91	-0.04	0.03
74	->	92	-0.01	0.01
74	->	93	0.02	0.00
74	->	94	0.02	0.00
74	->	95	0.01	0.00
74	->	96	0.00	0.00
74	->	97	0.01	0.00
74	->	98	0.01	0.00
74	->	99	0.02	0.00
74	->	100	0.04	0.04
74	->	101	-0.08	0.19
74	->	102	-1.85	0.82
74	->	103	-3.41	1.36
74	->	104	-0.55	0.26
74	->	105	0.02	0.04
74	->	106	0.01	0.00
75	->	1	-0.01	0.00
75	->	2	-0.00	0.00

75	->	3	0.00	0.00
75	->	4	-0.00	0.00
75	->	5	0.00	0.00
75	->	6	-0.00	0.00
75	->	7	0.00	0.00
75	->	8	0.00	0.00
75	->	9	0.00	0.00
75	->	10	0.01	0.00
75	->	11	0.00	0.00
75	->	12	-0.01	0.00
75	->	13	-0.00	0.00
75	->	14	-0.00	0.00
75	->	15	-0.00	0.00
75	->	16	0.01	0.00
75	->	17	0.00	0.00
75	->	18	-0.00	0.00
75	->	19	0.00	0.00
75	->	20	0.00	0.00
75	->	21	-0.00	0.00
75	->	22	-0.02	0.00
75	->	23	-0.00	0.00
75	->	24	-0.00	0.00
75	->	25	-0.01	0.00
75	->	26	-0.00	0.00
75	->	27	-0.00	0.00
75	->	28	-0.00	0.00
75	->	29	-0.00	0.00
75	->	30	0.00	0.00
75	->	31	-0.01	0.00
75	->	32	-0.00	0.00
75	->	33	0.00	0.00
75	->	34	0.00	0.00
75	->	35	0.02	0.00
75	->	36	-0.01	0.01
75	->	37	-0.00	0.00
75	->	38	0.02	0.00
75	->	39	-0.00	0.00
75	->	40	-0.02	0.01
75	->	41	0.00	0.00
75	->	42	-0.00	0.00
75	->	43	-0.00	0.00
75	->	44	-0.03	0.00
75	->	45	0.00	0.00
75	->	46	0.03	0.00
75	->	47	-0.00	0.00
75	->	48	0.00	0.00
75	->	49	-0.00	0.00
75	->	50	-0.02	0.01
75	->	51	-0.01	0.00
75	->	52	-0.00	0.00

75	->	53	-0.14	0.10
75	->	54	-0.02	0.04
75	->	55	-0.00	0.00
75	->	56	-0.00	0.00
75	->	57	-0.01	0.01
75	->	58	0.05	0.02
75	->	59	-0.58	0.62
75	->	60	-0.02	0.01
75	->	61	-0.12	0.05
75	->	62	-0.01	0.01
75	->	63	-0.01	0.02
75	->	64	0.02	0.00
75	->	65	-0.00	0.00
75	->	66	0.05	0.01
75	->	67	0.00	0.00
75	->	68	-2.02	3.32
75	->	69	-0.21	0.20
75	->	70	-3.65	1.39
75	->	71	-2.51	2.16
75	->	72	-4.42	1.39
75	->	73	-0.85	0.18
75	->	74	-13.47	0.63
75	->	75	-46.54	4.72
75	->	76	-7.51	0.53
75	->	77	-1.15	0.20
75	->	78	-5.80	3.96
75	->	79	-3.19	0.84
75	->	80	-0.16	0.07
75	->	81	-0.09	0.03
75	->	82	-0.05	0.03
75	->	83	-0.02	0.01
75	->	84	0.02	0.00
75	->	85	-0.08	0.11
75	->	86	-0.36	0.83
75	->	87	-1.37	1.99
75	->	88	0.09	0.10
75	->	89	0.01	0.01
75	->	90	0.01	0.01
75	->	91	0.01	0.00
75	->	92	0.01	0.00
75	->	93	0.01	0.00
75	->	94	0.01	0.00
75	->	95	0.00	0.00
75	->	96	0.00	0.00
75	->	97	0.00	0.00
75	->	98	0.01	0.00
75	->	99	0.01	0.00
75	->	100	0.01	0.01
75	->	101	-0.00	0.03
75	->	102	-0.02	0.03

75	->	103	-0.02	0.05
75	->	104	-0.09	0.18
75	->	105	0.06	0.13
75	->	106	0.00	0.01
76	->	1	0.02	0.00
76	->	2	0.00	0.00
76	->	3	-0.00	0.00
76	->	4	0.00	0.00
76	->	5	-0.00	0.00
76	->	6	0.00	0.00
76	->	7	0.00	0.00
76	->	8	-0.00	0.00
76	->	9	-0.00	0.00
76	->	10	-0.00	0.00
76	->	11	-0.00	0.00
76	->	12	0.01	0.00
76	->	13	0.00	0.00
76	->	14	0.00	0.00
76	->	15	0.00	0.00
76	->	16	-0.01	0.00
76	->	17	-0.00	0.00
76	->	18	0.00	0.00
76	->	19	-0.00	0.00
76	->	20	0.00	0.00
76	->	21	0.00	0.00
76	->	22	0.02	0.01
76	->	23	0.00	0.00
76	->	24	0.00	0.00
76	->	25	0.01	0.00
76	->	26	0.00	0.00
76	->	27	0.00	0.00
76	->	28	0.00	0.00
76	->	29	0.00	0.00
76	->	30	-0.00	0.00
76	->	31	0.01	0.00
76	->	32	0.00	0.00
76	->	33	0.00	0.00
76	->	34	-0.00	0.00
76	->	35	-0.01	0.00
76	->	36	0.01	0.00
76	->	37	0.00	0.00
76	->	38	-0.01	0.00
76	->	39	-0.00	0.00
76	->	40	0.01	0.00
76	->	41	-0.00	0.00
76	->	42	0.00	0.00
76	->	43	0.00	0.00
76	->	44	0.02	0.01
76	->	45	-0.00	0.00
76	->	46	-0.03	0.01

76	->	47	-0.00	0.00
76	->	48	-0.00	0.00
76	->	49	-0.00	0.00
76	->	50	-0.00	0.00
76	->	51	0.00	0.00
76	->	52	-0.00	0.00
76	->	53	0.04	0.02
76	->	54	-0.00	0.00
76	->	55	0.00	0.00
76	->	56	-0.00	0.00
76	->	57	-0.00	0.00
76	->	58	-0.04	0.01
76	->	59	0.03	0.01
76	->	60	-0.03	0.01
76	->	61	-1.26	0.82
76	->	62	-2.19	1.69
76	->	63	-1.63	0.90
76	->	64	-1.30	1.78
76	->	65	-0.05	0.03
76	->	66	-0.09	0.03
76	->	67	-0.01	0.01
76	->	68	-0.11	0.06
76	->	69	0.08	0.04
76	->	70	-2.93	0.50
76	->	71	-0.03	0.02
76	->	72	-0.86	0.37
76	->	73	-2.81	0.64
76	->	74	-1.62	0.33
76	->	75	-7.55	0.53
76	->	76	-104.51	2.83
76	->	77	-11.96	0.72
76	->	78	-0.85	0.21
76	->	79	-2.18	0.42
76	->	80	-3.22	1.39
76	->	81	0.00	0.05
76	->	82	-0.04	0.02
76	->	83	-0.07	0.02
76	->	84	-0.05	0.01
76	->	85	-0.02	0.01
76	->	86	-0.04	0.01
76	->	87	-0.03	0.00
76	->	88	-0.02	0.00
76	->	89	-0.02	0.00
76	->	90	-0.01	0.00
76	->	91	-0.01	0.00
76	->	92	-0.01	0.01
76	->	93	-0.02	0.01
76	->	94	-0.02	0.01
76	->	95	-0.01	0.00
76	->	96	-0.00	0.00

76	->	97	-0.00	0.00
76	->	98	-0.01	0.00
76	->	99	-0.03	0.02
76	->	100	-0.34	0.50
76	->	101	-2.45	4.02
76	->	102	-0.91	1.57
76	->	103	-0.11	0.04
76	->	104	-0.02	0.00
76	->	105	-0.02	0.00
76	->	106	-0.01	0.00
77	->	1	0.03	0.01
77	->	2	0.00	0.00
77	->	3	-0.00	0.00
77	->	4	0.00	0.00
77	->	5	-0.01	0.00
77	->	6	0.00	0.00
77	->	7	-0.00	0.00
77	->	8	-0.00	0.00
77	->	9	-0.00	0.00
77	->	10	-0.01	0.00
77	->	11	-0.01	0.00
77	->	12	0.02	0.00
77	->	13	0.01	0.00
77	->	14	0.00	0.00
77	->	15	0.00	0.00
77	->	16	-0.02	0.00
77	->	17	-0.00	0.00
77	->	18	0.00	0.00
77	->	19	0.00	0.00
77	->	20	0.00	0.00
77	->	21	0.00	0.00
77	->	22	0.02	0.00
77	->	23	0.00	0.00
77	->	24	0.00	0.00
77	->	25	0.01	0.00
77	->	26	0.00	0.00
77	->	27	0.00	0.00
77	->	28	0.00	0.00
77	->	29	0.00	0.00
77	->	30	-0.00	0.00
77	->	31	0.01	0.00
77	->	32	0.00	0.00
77	->	33	0.00	0.00
77	->	34	-0.00	0.00
77	->	35	-0.01	0.00
77	->	36	0.00	0.00
77	->	37	0.00	0.00
77	->	38	-0.01	0.00
77	->	39	-0.00	0.00
77	->	40	0.01	0.00

77 ->	41	-0.00	0.00
77 ->	42	0.00	0.00
77 ->	43	0.00	0.00
77 ->	44	0.03	0.00
77 ->	45	-0.00	0.00
77 ->	46	-0.03	0.00
77 ->	47	-0.00	0.00
77 ->	48	-0.00	0.00
77 ->	49	-0.00	0.00
77 ->	50	0.00	0.00
77 ->	51	0.00	0.00
77 ->	52	0.00	0.00
77 ->	53	0.03	0.01
77 ->	54	0.00	0.00
77 ->	55	0.00	0.00
77 ->	56	0.00	0.00
77 ->	57	0.00	0.00
77 ->	58	-0.02	0.00
77 ->	59	0.01	0.00
77 ->	60	-0.01	0.00
77 ->	61	-0.04	0.03
77 ->	62	-0.02	0.01
77 ->	63	-0.02	0.02
77 ->	64	-0.04	0.02
77 ->	65	-0.01	0.00
77 ->	66	-0.03	0.01
77 ->	67	-0.00	0.00
77 ->	68	0.01	0.01
77 ->	69	-0.01	0.01
77 ->	70	-0.05	0.02
77 ->	71	-0.01	0.00
77 ->	72	0.08	0.04
77 ->	73	-1.81	0.84
77 ->	74	-2.55	0.43
77 ->	75	-1.16	0.20
77 ->	76	-11.99	0.74
77 ->	77	13.62	4.16
77 ->	78	-6.62	0.54
77 ->	79	-0.88	0.21
77 ->	80	-2.07	0.48
77 ->	81	-1.47	0.85
77 ->	82	-0.06	0.06
77 ->	83	-0.04	0.02
77 ->	84	-0.07	0.02
77 ->	85	-0.02	0.01
77 ->	86	-0.04	0.01
77 ->	87	-0.01	0.01
77 ->	88	-0.03	0.00
77 ->	89	-0.02	0.00
77 ->	90	-0.01	0.01

77	->	91	-0.00	0.01
77	->	92	-0.03	0.01
77	->	93	-0.05	0.01
77	->	94	-0.03	0.01
77	->	95	-0.01	0.00
77	->	96	-0.00	0.00
77	->	97	-0.01	0.00
77	->	98	-0.01	0.00
77	->	99	-0.02	0.00
77	->	100	-0.06	0.03
77	->	101	-0.96	0.54
77	->	102	-7.71	3.46
77	->	103	-3.90	2.61
77	->	104	-0.07	0.02
77	->	105	-0.04	0.01
77	->	106	-0.01	0.00
78	->	1	0.02	0.00
78	->	2	0.00	0.00
78	->	3	-0.00	0.00
78	->	4	0.00	0.00
78	->	5	-0.01	0.00
78	->	6	0.00	0.00
78	->	7	-0.00	0.00
78	->	8	-0.00	0.00
78	->	9	-0.00	0.00
78	->	10	-0.01	0.00
78	->	11	-0.01	0.00
78	->	12	0.02	0.01
78	->	13	0.00	0.00
78	->	14	0.00	0.00
78	->	15	0.00	0.00
78	->	16	-0.01	0.00
78	->	17	-0.00	0.00
78	->	18	0.00	0.00
78	->	19	-0.00	0.00
78	->	20	-0.00	0.00
78	->	21	0.00	0.00
78	->	22	0.02	0.00
78	->	23	0.00	0.00
78	->	24	0.00	0.00
78	->	25	0.01	0.00
78	->	26	0.00	0.00
78	->	27	0.00	0.00
78	->	28	0.00	0.00
78	->	29	0.00	0.00
78	->	30	-0.00	0.00
78	->	31	0.01	0.00
78	->	32	0.00	0.00
78	->	33	-0.00	0.00
78	->	34	-0.00	0.00

78	->	35	-0.01	0.00
78	->	36	0.01	0.00
78	->	37	0.00	0.00
78	->	38	-0.01	0.00
78	->	39	-0.00	0.00
78	->	40	0.02	0.00
78	->	41	-0.00	0.00
78	->	42	0.00	0.00
78	->	43	0.00	0.00
78	->	44	0.02	0.00
78	->	45	-0.00	0.00
78	->	46	-0.03	0.00
78	->	47	-0.00	0.00
78	->	48	-0.00	0.00
78	->	49	-0.00	0.00
78	->	50	-0.00	0.00
78	->	51	0.00	0.00
78	->	52	-0.00	0.00
78	->	53	0.03	0.01
78	->	54	-0.00	0.00
78	->	55	0.00	0.00
78	->	56	0.00	0.00
78	->	57	0.00	0.00
78	->	58	-0.02	0.00
78	->	59	0.05	0.01
78	->	60	-0.00	0.00
78	->	61	-0.01	0.00
78	->	62	0.00	0.01
78	->	63	-0.17	0.05
78	->	64	-0.03	0.01
78	->	65	-0.02	0.00
78	->	66	-0.08	0.01
78	->	67	0.00	0.00
78	->	68	0.11	0.04
78	->	69	0.04	0.01
78	->	70	-0.24	0.07
78	->	71	-0.00	0.01
78	->	72	0.33	0.29
78	->	73	-0.11	0.08
78	->	74	-2.93	1.69
78	->	75	-5.84	4.00
78	->	76	-0.85	0.21
78	->	77	-6.65	0.54
78	->	78	-103.21	3.07
78	->	79	-19.64	0.43
78	->	80	-0.72	0.17
78	->	81	-2.16	0.48
78	->	82	-1.56	1.13
78	->	83	-0.13	0.05
78	->	84	-0.06	0.02

78	->	85	-1.69	1.30
78	->	86	-6.10	2.63
78	->	87	-0.25	0.95
78	->	88	-0.24	0.37
78	->	89	-0.04	0.02
78	->	90	-0.03	0.01
78	->	91	-0.02	0.00
78	->	92	-0.02	0.00
78	->	93	-0.01	0.00
78	->	94	-0.01	0.00
78	->	95	-0.00	0.00
78	->	96	-0.00	0.00
78	->	97	-0.00	0.00
78	->	98	-0.01	0.00
78	->	99	-0.01	0.00
78	->	100	-0.02	0.01
78	->	101	-0.05	0.01
78	->	102	-0.10	0.04
78	->	103	-0.25	0.23
78	->	104	-0.65	0.85
78	->	105	-2.63	2.44
78	->	106	-0.04	0.30
79	->	1	0.00	0.00
79	->	2	0.00	0.00
79	->	3	0.00	0.00
79	->	4	0.00	0.00
79	->	5	-0.00	0.00
79	->	6	0.00	0.00
79	->	7	0.00	0.00
79	->	8	-0.00	0.00
79	->	9	0.00	0.00
79	->	10	-0.00	0.00
79	->	11	-0.00	0.00
79	->	12	0.00	0.00
79	->	13	0.00	0.00
79	->	14	0.00	0.00
79	->	15	0.00	0.00
79	->	16	-0.00	0.00
79	->	17	-0.00	0.00
79	->	18	-0.00	0.00
79	->	19	-0.00	0.00
79	->	20	0.00	0.00
79	->	21	0.00	0.00
79	->	22	0.00	0.00
79	->	23	0.00	0.00
79	->	24	0.00	0.00
79	->	25	0.00	0.00
79	->	26	0.00	0.00
79	->	27	0.00	0.00
79	->	28	0.00	0.00

79	->	29	0.00	0.00
79	->	30	-0.00	0.00
79	->	31	0.00	0.00
79	->	32	0.00	0.00
79	->	33	-0.00	0.00
79	->	34	-0.00	0.00
79	->	35	-0.00	0.00
79	->	36	0.00	0.00
79	->	37	0.00	0.00
79	->	38	-0.00	0.00
79	->	39	-0.00	0.00
79	->	40	0.00	0.00
79	->	41	-0.00	0.00
79	->	42	0.00	0.00
79	->	43	0.00	0.00
79	->	44	0.00	0.00
79	->	45	-0.00	0.00
79	->	46	-0.00	0.00
79	->	47	-0.00	0.00
79	->	48	-0.00	0.00
79	->	49	-0.00	0.00
79	->	50	-0.00	0.00
79	->	51	0.00	0.00
79	->	52	0.00	0.00
79	->	53	0.00	0.00
79	->	54	-0.00	0.00
79	->	55	0.00	0.00
79	->	56	0.00	0.00
79	->	57	-0.00	0.00
79	->	58	-0.00	0.00
79	->	59	-0.00	0.00
79	->	60	-0.00	0.00
79	->	61	-0.02	0.00
79	->	62	-0.08	0.02
79	->	63	2.72	0.74
79	->	64	-0.05	0.07
79	->	65	0.70	0.11
79	->	66	3.37	0.73
79	->	67	-0.09	0.05
79	->	68	-1.97	0.63
79	->	69	-0.02	0.01
79	->	70	-1.85	0.35
79	->	71	-0.00	0.00
79	->	72	-0.01	0.01
79	->	73	-0.04	0.02
79	->	74	-0.12	0.06
79	->	75	-3.22	0.84
79	->	76	-2.19	0.42
79	->	77	-0.89	0.21
79	->	78	-19.68	0.43

79	->	79	9.89	1.40
79	->	80	-5.04	0.52
79	->	81	-1.13	0.26
79	->	82	-3.82	0.73
79	->	83	16.73	10.30
79	->	84	-0.03	0.04
79	->	85	-0.09	0.12
79	->	86	-0.05	0.02
79	->	87	-0.01	0.00
79	->	88	-0.01	0.00
79	->	89	-0.00	0.00
79	->	90	-0.00	0.00
79	->	91	-0.00	0.00
79	->	92	-0.00	0.00
79	->	93	-0.00	0.00
79	->	94	-0.00	0.00
79	->	95	-0.00	0.00
79	->	96	-0.00	0.00
79	->	97	-0.00	0.00
79	->	98	-0.00	0.00
79	->	99	-0.00	0.00
79	->	100	-0.00	0.00
79	->	101	-0.01	0.01
79	->	102	-0.02	0.01
79	->	103	-0.01	0.01
79	->	104	-0.01	0.00
79	->	105	-0.01	0.00
79	->	106	-0.00	0.00
80	->	1	0.00	0.00
80	->	2	0.00	0.00
80	->	3	0.00	0.00
80	->	4	0.00	0.00
80	->	5	-0.00	0.00
80	->	6	0.00	0.00
80	->	7	0.00	0.00
80	->	8	-0.00	0.00
80	->	9	0.00	0.00
80	->	10	-0.00	0.00
80	->	11	-0.00	0.00
80	->	12	0.00	0.00
80	->	13	0.00	0.00
80	->	14	0.00	0.00
80	->	15	0.00	0.00
80	->	16	-0.00	0.00
80	->	17	0.00	0.00
80	->	18	0.00	0.00
80	->	19	0.00	0.00
80	->	20	0.00	0.00
80	->	21	0.00	0.00
80	->	22	0.00	0.00

80	->	23	0.00	0.00
80	->	24	0.00	0.00
80	->	25	0.00	0.00
80	->	26	0.00	0.00
80	->	27	0.00	0.00
80	->	28	0.00	0.00
80	->	29	0.00	0.00
80	->	30	0.00	0.00
80	->	31	0.00	0.00
80	->	32	0.00	0.00
80	->	33	0.00	0.00
80	->	34	0.00	0.00
80	->	35	-0.00	0.00
80	->	36	0.00	0.00
80	->	37	0.00	0.00
80	->	38	-0.00	0.00
80	->	39	0.00	0.00
80	->	40	0.00	0.00
80	->	41	0.00	0.00
80	->	42	0.00	0.00
80	->	43	0.00	0.00
80	->	44	0.00	0.00
80	->	45	-0.00	0.00
80	->	46	-0.00	0.00
80	->	47	0.00	0.00
80	->	48	-0.00	0.00
80	->	49	0.00	0.00
80	->	50	0.00	0.00
80	->	51	0.00	0.00
80	->	52	-0.00	0.00
80	->	53	0.00	0.00
80	->	54	0.00	0.00
80	->	55	0.00	0.00
80	->	56	0.00	0.00
80	->	57	-0.00	0.00
80	->	58	-0.00	0.00
80	->	59	-0.00	0.00
80	->	60	-0.00	0.00
80	->	61	-0.01	0.01
80	->	62	-0.03	0.02
80	->	63	0.13	0.24
80	->	64	-0.10	0.14
80	->	65	-0.11	0.14
80	->	66	0.03	0.02
80	->	67	-0.01	0.00
80	->	68	-0.04	0.01
80	->	69	-0.01	0.01
80	->	70	-0.05	0.03
80	->	71	-0.00	0.00
80	->	72	-0.01	0.01

80 ->	73	-0.02	0.01
80 ->	74	-0.04	0.02
80 ->	75	-0.16	0.07
80 ->	76	-3.20	1.39
80 ->	77	-2.07	0.47
80 ->	78	-0.72	0.17
80 ->	79	-5.07	0.52
80 ->	80	-23.47	1.69
80 ->	81	-15.32	0.43
80 ->	82	-0.76	0.20
80 ->	83	-2.82	0.88
80 ->	84	-0.76	0.73
80 ->	85	-0.00	0.00
80 ->	86	-0.01	0.00
80 ->	87	-0.00	0.00
80 ->	88	-0.00	0.00
80 ->	89	-0.00	0.00
80 ->	90	-0.00	0.00
80 ->	91	-0.00	0.00
80 ->	92	-0.00	0.00
80 ->	93	-0.00	0.00
80 ->	94	-0.00	0.00
80 ->	95	-0.00	0.00
80 ->	96	-0.00	0.00
80 ->	97	-0.00	0.00
80 ->	98	-0.00	0.00
80 ->	99	-0.00	0.00
80 ->	100	-0.00	0.00
80 ->	101	-0.01	0.01
80 ->	102	-0.03	0.03
80 ->	103	-0.02	0.01
80 ->	104	-0.00	0.00
80 ->	105	-0.00	0.00
80 ->	106	-0.00	0.00
81 ->	1	0.03	0.01
81 ->	2	0.00	0.00
81 ->	3	-0.00	0.00
81 ->	4	0.00	0.00
81 ->	5	-0.00	0.00
81 ->	6	0.00	0.00
81 ->	7	-0.00	0.00
81 ->	8	-0.00	0.00
81 ->	9	-0.00	0.00
81 ->	10	-0.01	0.00
81 ->	11	-0.01	0.00
81 ->	12	0.02	0.01
81 ->	13	0.00	0.00
81 ->	14	0.00	0.00
81 ->	15	0.00	0.00
81 ->	16	-0.01	0.00

81 ->	17	-0.00	0.00
81 ->	18	0.00	0.00
81 ->	19	0.00	0.00
81 ->	20	-0.00	0.00
81 ->	21	0.00	0.00
81 ->	22	0.01	0.00
81 ->	23	0.00	0.00
81 ->	24	0.00	0.00
81 ->	25	0.00	0.00
81 ->	26	0.00	0.00
81 ->	27	0.00	0.00
81 ->	28	0.00	0.00
81 ->	29	0.00	0.00
81 ->	30	-0.00	0.00
81 ->	31	0.00	0.00
81 ->	32	0.00	0.00
81 ->	33	0.00	0.00
81 ->	34	-0.00	0.00
81 ->	35	-0.00	0.00
81 ->	36	0.00	0.00
81 ->	37	0.00	0.00
81 ->	38	-0.01	0.00
81 ->	39	-0.00	0.00
81 ->	40	0.01	0.00
81 ->	41	-0.00	0.00
81 ->	42	0.00	0.00
81 ->	43	0.00	0.00
81 ->	44	0.02	0.00
81 ->	45	-0.00	0.00
81 ->	46	-0.02	0.00
81 ->	47	0.00	0.00
81 ->	48	-0.00	0.00
81 ->	49	0.00	0.00
81 ->	50	0.00	0.00
81 ->	51	0.00	0.00
81 ->	52	0.00	0.00
81 ->	53	0.02	0.00
81 ->	54	0.00	0.00
81 ->	55	0.00	0.00
81 ->	56	0.00	0.00
81 ->	57	0.00	0.00
81 ->	58	-0.01	0.00
81 ->	59	0.02	0.00
81 ->	60	-0.00	0.00
81 ->	61	-0.00	0.00
81 ->	62	-0.01	0.00
81 ->	63	-0.01	0.02
81 ->	64	-0.03	0.01
81 ->	65	-0.01	0.01
81 ->	66	-0.03	0.01

81	->	67	-0.00	0.00
81	->	68	0.03	0.01
81	->	69	0.01	0.00
81	->	70	-0.01	0.00
81	->	71	0.00	0.00
81	->	72	0.04	0.01
81	->	73	-0.01	0.01
81	->	74	-0.11	0.04
81	->	75	-0.09	0.03
81	->	76	0.00	0.05
81	->	77	-1.48	0.84
81	->	78	-2.19	0.47
81	->	79	-1.14	0.26
81	->	80	-15.37	0.42
81	->	81	5.71	3.06
81	->	82	-11.38	0.57
81	->	83	-0.65	0.27
81	->	84	-1.81	0.72
81	->	85	-0.03	0.02
81	->	86	-0.05	0.02
81	->	87	-0.02	0.00
81	->	88	-0.02	0.00
81	->	89	-0.02	0.00
81	->	90	-0.01	0.00
81	->	91	-0.01	0.00
81	->	92	-0.02	0.01
81	->	93	-0.02	0.01
81	->	94	-0.01	0.00
81	->	95	-0.01	0.00
81	->	96	-0.00	0.00
81	->	97	-0.00	0.00
81	->	98	-0.00	0.00
81	->	99	-0.01	0.00
81	->	100	-0.02	0.00
81	->	101	-0.05	0.02
81	->	102	-0.31	0.36
81	->	103	-1.93	2.73
81	->	104	-0.20	0.21
81	->	105	-0.06	0.02
81	->	106	-0.01	0.00
82	->	1	0.00	0.00
82	->	2	0.00	0.00
82	->	3	0.00	0.00
82	->	4	0.00	0.00
82	->	5	-0.00	0.00
82	->	6	0.00	0.00
82	->	7	0.00	0.00
82	->	8	-0.00	0.00
82	->	9	0.00	0.00
82	->	10	-0.00	0.00

82	->	11	-0.00	0.00
82	->	12	0.00	0.00
82	->	13	0.00	0.00
82	->	14	0.00	0.00
82	->	15	0.00	0.00
82	->	16	-0.00	0.00
82	->	17	-0.00	0.00
82	->	18	0.00	0.00
82	->	19	0.00	0.00
82	->	20	0.00	0.00
82	->	21	0.00	0.00
82	->	22	0.00	0.00
82	->	23	0.00	0.00
82	->	24	0.00	0.00
82	->	25	0.00	0.00
82	->	26	0.00	0.00
82	->	27	0.00	0.00
82	->	28	0.00	0.00
82	->	29	0.00	0.00
82	->	30	-0.00	0.00
82	->	31	0.00	0.00
82	->	32	0.00	0.00
82	->	33	0.00	0.00
82	->	34	-0.00	0.00
82	->	35	-0.00	0.00
82	->	36	0.00	0.00
82	->	37	0.00	0.00
82	->	38	-0.00	0.00
82	->	39	-0.00	0.00
82	->	40	0.00	0.00
82	->	41	-0.00	0.00
82	->	42	0.00	0.00
82	->	43	0.00	0.00
82	->	44	0.00	0.00
82	->	45	-0.00	0.00
82	->	46	-0.00	0.00
82	->	47	0.00	0.00
82	->	48	-0.00	0.00
82	->	49	0.00	0.00
82	->	50	0.00	0.00
82	->	51	0.00	0.00
82	->	52	0.00	0.00
82	->	53	0.00	0.00
82	->	54	0.00	0.00
82	->	55	0.00	0.00
82	->	56	0.00	0.00
82	->	57	0.00	0.00
82	->	58	-0.00	0.00
82	->	59	0.00	0.00
82	->	60	-0.00	0.00

82 ->	61	-0.00	0.00
82 ->	62	-0.00	0.00
82 ->	63	-0.08	0.03
82 ->	64	-0.01	0.00
82 ->	65	-0.05	0.02
82 ->	66	-0.07	0.04
82 ->	67	-0.00	0.00
82 ->	68	-0.05	0.05
82 ->	69	-0.00	0.00
82 ->	70	-0.03	0.01
82 ->	71	-0.00	0.00
82 ->	72	-0.00	0.00
82 ->	73	-0.00	0.00
82 ->	74	-0.01	0.01
82 ->	75	-0.05	0.03
82 ->	76	-0.04	0.02
82 ->	77	-0.06	0.06
82 ->	78	-1.57	1.11
82 ->	79	-3.84	0.73
82 ->	80	-0.78	0.20
82 ->	81	-11.48	0.57
82 ->	82	17.35	2.02
82 ->	83	-22.02	0.75
82 ->	84	-0.96	0.36
82 ->	85	-0.09	0.15
82 ->	86	-0.02	0.02
82 ->	87	-0.00	0.00
82 ->	88	-0.00	0.00
82 ->	89	-0.00	0.00
82 ->	90	-0.00	0.00
82 ->	91	-0.00	0.00
82 ->	92	-0.00	0.00
82 ->	93	-0.00	0.00
82 ->	94	-0.00	0.00
82 ->	95	-0.00	0.00
82 ->	96	-0.00	0.00
82 ->	97	0.00	0.00
82 ->	98	-0.00	0.00
82 ->	99	-0.00	0.00
82 ->	100	-0.00	0.00
82 ->	101	-0.00	0.00
82 ->	102	-0.00	0.00
82 ->	103	-0.01	0.00
82 ->	104	-0.01	0.01
82 ->	105	-0.01	0.01
82 ->	106	-0.00	0.00
83 ->	1	0.00	0.00
83 ->	2	0.00	0.00
83 ->	3	0.00	0.00
83 ->	4	0.00	0.00

83	->	5	-0.00	0.00
83	->	6	0.00	0.00
83	->	7	0.00	0.00
83	->	8	-0.00	0.00
83	->	9	0.00	0.00
83	->	10	-0.00	0.00
83	->	11	-0.00	0.00
83	->	12	0.00	0.00
83	->	13	0.00	0.00
83	->	14	0.00	0.00
83	->	15	0.00	0.00
83	->	16	-0.00	0.00
83	->	17	0.00	0.00
83	->	18	0.00	0.00
83	->	19	0.00	0.00
83	->	20	0.00	0.00
83	->	21	0.00	0.00
83	->	22	0.00	0.00
83	->	23	0.00	0.00
83	->	24	0.00	0.00
83	->	25	-0.00	0.00
83	->	26	0.00	0.00
83	->	27	-0.00	0.00
83	->	28	0.00	0.00
83	->	29	0.00	0.00
83	->	30	-0.00	0.00
83	->	31	0.00	0.00
83	->	32	0.00	0.00
83	->	33	0.00	0.00
83	->	34	0.00	0.00
83	->	35	0.00	0.00
83	->	36	-0.00	0.00
83	->	37	0.00	0.00
83	->	38	0.00	0.00
83	->	39	0.00	0.00
83	->	40	0.00	0.00
83	->	41	0.00	0.00
83	->	42	0.00	0.00
83	->	43	0.00	0.00
83	->	44	-0.00	0.00
83	->	45	0.00	0.00
83	->	46	0.00	0.00
83	->	47	0.00	0.00
83	->	48	0.00	0.00
83	->	49	0.00	0.00
83	->	50	0.00	0.00
83	->	51	-0.00	0.00
83	->	52	0.00	0.00
83	->	53	-0.00	0.00
83	->	54	0.00	0.00

83 ->	55	0.00	0.00
83 ->	56	0.00	0.00
83 ->	57	0.00	0.00
83 ->	58	0.00	0.00
83 ->	59	-0.00	0.00
83 ->	60	-0.00	0.00
83 ->	61	-0.01	0.00
83 ->	62	-0.04	0.01
83 ->	63	2.18	0.26
83 ->	64	-0.15	0.10
83 ->	65	0.55	0.75
83 ->	66	1.95	0.25
83 ->	67	-0.04	0.04
83 ->	68	-0.22	0.06
83 ->	69	-0.01	0.01
83 ->	70	-0.01	0.01
83 ->	71	-0.00	0.00
83 ->	72	-0.00	0.00
83 ->	73	-0.00	0.00
83 ->	74	-0.01	0.00
83 ->	75	-0.02	0.01
83 ->	76	-0.07	0.02
83 ->	77	-0.04	0.02
83 ->	78	-0.13	0.05
83 ->	79	16.72	10.30
83 ->	80	-2.83	0.88
83 ->	81	-0.66	0.27
83 ->	82	-22.12	0.74
83 ->	83	4.16	1.37
83 ->	84	-5.00	0.56
83 ->	85	-0.01	0.00
83 ->	86	-0.00	0.00
83 ->	87	-0.00	0.00
83 ->	88	-0.00	0.00
83 ->	89	-0.00	0.00
83 ->	90	-0.00	0.00
83 ->	91	-0.00	0.00
83 ->	92	-0.00	0.00
83 ->	93	-0.00	0.00
83 ->	94	-0.00	0.00
83 ->	95	0.00	0.00
83 ->	96	0.00	0.00
83 ->	97	-0.00	0.00
83 ->	98	0.00	0.00
83 ->	99	0.00	0.00
83 ->	100	0.00	0.00
83 ->	101	-0.00	0.00
83 ->	102	-0.00	0.00
83 ->	103	-0.00	0.00
83 ->	104	-0.00	0.00

83 ->	105	-0.00	0.00
83 ->	106	-0.00	0.00
84 ->	1	-0.01	0.00
84 ->	2	-0.00	0.00
84 ->	3	0.00	0.00
84 ->	4	-0.00	0.00
84 ->	5	0.00	0.00
84 ->	6	-0.00	0.00
84 ->	7	0.00	0.00
84 ->	8	0.00	0.00
84 ->	9	0.00	0.00
84 ->	10	0.00	0.00
84 ->	11	0.00	0.00
84 ->	12	-0.00	0.00
84 ->	13	-0.00	0.00
84 ->	14	-0.00	0.00
84 ->	15	-0.00	0.00
84 ->	16	0.00	0.00
84 ->	17	0.00	0.00
84 ->	18	-0.00	0.00
84 ->	19	-0.00	0.00
84 ->	20	0.00	0.00
84 ->	21	-0.00	0.00
84 ->	22	-0.00	0.00
84 ->	23	-0.00	0.00
84 ->	24	-0.00	0.00
84 ->	25	-0.00	0.00
84 ->	26	-0.00	0.00
84 ->	27	0.00	0.00
84 ->	28	-0.00	0.00
84 ->	29	-0.00	0.00
84 ->	30	0.00	0.00
84 ->	31	-0.00	0.00
84 ->	32	-0.00	0.00
84 ->	33	0.00	0.00
84 ->	34	0.00	0.00
84 ->	35	0.00	0.00
84 ->	36	-0.00	0.00
84 ->	37	-0.00	0.00
84 ->	38	0.00	0.00
84 ->	39	0.00	0.00
84 ->	40	-0.00	0.00
84 ->	41	0.00	0.00
84 ->	42	-0.00	0.00
84 ->	43	-0.00	0.00
84 ->	44	-0.00	0.00
84 ->	45	0.00	0.00
84 ->	46	0.00	0.00
84 ->	47	0.00	0.00
84 ->	48	0.00	0.00

84	->	49	-0.00	0.00
84	->	50	-0.00	0.00
84	->	51	-0.00	0.00
84	->	52	-0.00	0.00
84	->	53	-0.01	0.00
84	->	54	0.00	0.00
84	->	55	-0.00	0.00
84	->	56	0.00	0.00
84	->	57	0.00	0.00
84	->	58	0.01	0.00
84	->	59	-0.01	0.00
84	->	60	0.00	0.00
84	->	61	-0.00	0.00
84	->	62	-0.00	0.00
84	->	63	0.06	0.01
84	->	64	0.04	0.01
84	->	65	-0.06	0.09
84	->	66	0.05	0.00
84	->	67	-0.00	0.00
84	->	68	-0.03	0.00
84	->	69	-0.01	0.00
84	->	70	-0.00	0.00
84	->	71	-0.00	0.00
84	->	72	-0.01	0.00
84	->	73	0.00	0.00
84	->	74	0.02	0.00
84	->	75	0.02	0.00
84	->	76	-0.05	0.01
84	->	77	-0.07	0.02
84	->	78	-0.06	0.02
84	->	79	-0.03	0.04
84	->	80	-0.78	0.73
84	->	81	-1.84	0.72
84	->	82	-0.99	0.37
84	->	83	-5.11	0.56
84	->	84	-90.52	1.80
84	->	85	0.01	0.00
84	->	86	0.02	0.00
84	->	87	0.01	0.00
84	->	88	0.01	0.00
84	->	89	0.00	0.00
84	->	90	0.00	0.00
84	->	91	0.00	0.00
84	->	92	0.00	0.00
84	->	93	0.01	0.00
84	->	94	0.01	0.00
84	->	95	0.00	0.00
84	->	96	0.00	0.00
84	->	97	0.00	0.00
84	->	98	0.00	0.00

84	->	99	0.00	0.00
84	->	100	0.01	0.00
84	->	101	0.01	0.00
84	->	102	0.02	0.00
84	->	103	0.02	0.00
84	->	104	0.01	0.00
84	->	105	0.01	0.00
84	->	106	0.00	0.00
85	->	1	-0.00	0.00
85	->	2	-0.00	0.00
85	->	3	0.00	0.00
85	->	4	0.00	0.00
85	->	5	0.00	0.00
85	->	6	-0.00	0.00
85	->	7	0.00	0.00
85	->	8	0.00	0.00
85	->	9	0.00	0.00
85	->	10	0.00	0.00
85	->	11	0.00	0.00
85	->	12	-0.00	0.00
85	->	13	-0.00	0.00
85	->	14	-0.00	0.00
85	->	15	-0.00	0.00
85	->	16	0.00	0.00
85	->	17	0.00	0.00
85	->	18	-0.00	0.00
85	->	19	0.00	0.00
85	->	20	0.00	0.00
85	->	21	0.00	0.00
85	->	22	-0.00	0.00
85	->	23	-0.00	0.00
85	->	24	0.00	0.00
85	->	25	-0.00	0.00
85	->	26	-0.00	0.00
85	->	27	-0.00	0.00
85	->	28	0.00	0.00
85	->	29	0.00	0.00
85	->	30	0.00	0.00
85	->	31	-0.00	0.00
85	->	32	0.00	0.00
85	->	33	-0.00	0.00
85	->	34	0.00	0.00
85	->	35	0.00	0.00
85	->	36	-0.00	0.00
85	->	37	-0.00	0.00
85	->	38	0.00	0.00
85	->	39	0.00	0.00
85	->	40	-0.01	0.00
85	->	41	0.00	0.00
85	->	42	-0.00	0.00

85 ->	43	-0.00	0.00
85 ->	44	-0.00	0.00
85 ->	45	0.00	0.00
85 ->	46	0.00	0.00
85 ->	47	-0.00	0.00
85 ->	48	-0.00	0.00
85 ->	49	-0.00	0.00
85 ->	50	-0.00	0.00
85 ->	51	-0.00	0.00
85 ->	52	-0.00	0.00
85 ->	53	-0.01	0.01
85 ->	54	-0.01	0.00
85 ->	55	-0.00	0.00
85 ->	56	-0.00	0.00
85 ->	57	-0.00	0.00
85 ->	58	0.00	0.00
85 ->	59	-0.24	0.35
85 ->	60	-0.00	0.00
85 ->	61	-0.00	0.00
85 ->	62	-0.00	0.00
85 ->	63	0.01	0.00
85 ->	64	0.00	0.00
85 ->	65	-0.00	0.00
85 ->	66	0.02	0.01
85 ->	67	-0.00	0.00
85 ->	68	-1.94	1.87
85 ->	69	-0.04	0.04
85 ->	70	-0.12	0.23
85 ->	71	-0.00	0.01
85 ->	72	-0.04	0.01
85 ->	73	-0.00	0.00
85 ->	74	-0.00	0.01
85 ->	75	-0.08	0.12
85 ->	76	-0.02	0.01
85 ->	77	-0.02	0.01
85 ->	78	-1.68	1.29
85 ->	79	-0.09	0.12
85 ->	80	-0.00	0.00
85 ->	81	-0.03	0.02
85 ->	82	-0.09	0.15
85 ->	83	-0.01	0.00
85 ->	84	0.01	0.00
85 ->	85	-85.85	2.63
85 ->	86	-35.56	0.87
85 ->	87	-0.31	0.07
85 ->	88	-0.03	0.01
85 ->	89	-0.00	0.00
85 ->	90	0.00	0.00
85 ->	91	0.00	0.00
85 ->	92	0.00	0.00

85 ->	93	0.00	0.00
85 ->	94	0.00	0.00
85 ->	95	0.00	0.00
85 ->	96	0.00	0.00
85 ->	97	0.00	0.00
85 ->	98	0.00	0.00
85 ->	99	0.00	0.00
85 ->	100	0.00	0.00
85 ->	101	0.00	0.00
85 ->	102	0.00	0.00
85 ->	103	-0.00	0.00
85 ->	104	-0.03	0.01
85 ->	105	-0.19	0.08
85 ->	106	-2.06	1.26
86 ->	1	-0.01	0.00
86 ->	2	-0.00	0.00
86 ->	3	0.00	0.00
86 ->	4	-0.00	0.00
86 ->	5	0.00	0.00
86 ->	6	-0.00	0.00
86 ->	7	-0.00	0.00
86 ->	8	0.00	0.00
86 ->	9	0.00	0.00
86 ->	10	0.00	0.00
86 ->	11	0.00	0.00
86 ->	12	-0.01	0.00
86 ->	13	-0.00	0.00
86 ->	14	-0.00	0.00
86 ->	15	-0.00	0.00
86 ->	16	0.00	0.00
86 ->	17	0.00	0.00
86 ->	18	0.00	0.00
86 ->	19	0.00	0.00
86 ->	20	0.00	0.00
86 ->	21	-0.00	0.00
86 ->	22	-0.01	0.00
86 ->	23	-0.00	0.00
86 ->	24	0.00	0.00
86 ->	25	-0.01	0.00
86 ->	26	-0.00	0.00
86 ->	27	-0.00	0.00
86 ->	28	-0.00	0.00
86 ->	29	-0.00	0.00
86 ->	30	0.00	0.00
86 ->	31	-0.01	0.00
86 ->	32	0.00	0.00
86 ->	33	-0.00	0.00
86 ->	34	0.00	0.00
86 ->	35	0.02	0.00
86 ->	36	-0.01	0.00

86	->	37	-0.00	0.00
86	->	38	0.02	0.00
86	->	39	-0.00	0.00
86	->	40	-0.03	0.01
86	->	41	0.00	0.00
86	->	42	-0.00	0.00
86	->	43	-0.00	0.00
86	->	44	-0.02	0.00
86	->	45	0.00	0.00
86	->	46	0.02	0.00
86	->	47	-0.01	0.00
86	->	48	-0.00	0.00
86	->	49	-0.00	0.00
86	->	50	-0.01	0.01
86	->	51	-0.02	0.01
86	->	52	-0.00	0.00
86	->	53	-0.11	0.05
86	->	54	-0.13	0.12
86	->	55	0.01	0.01
86	->	56	-0.01	0.01
86	->	57	-0.03	0.02
86	->	58	0.03	0.01
86	->	59	-1.90	2.57
86	->	60	-0.01	0.00
86	->	61	-0.01	0.00
86	->	62	-0.01	0.00
86	->	63	0.04	0.01
86	->	64	0.02	0.00
86	->	65	0.00	0.00
86	->	66	0.05	0.01
86	->	67	-0.00	0.00
86	->	68	-3.05	2.64
86	->	69	-0.14	0.11
86	->	70	-0.27	0.28
86	->	71	-0.07	0.05
86	->	72	-0.23	0.07
86	->	73	-0.00	0.00
86	->	74	0.01	0.04
86	->	75	-0.40	0.85
86	->	76	-0.04	0.01
86	->	77	-0.04	0.01
86	->	78	-6.09	2.63
86	->	79	-0.05	0.02
86	->	80	-0.01	0.00
86	->	81	-0.05	0.02
86	->	82	-0.02	0.02
86	->	83	-0.00	0.00
86	->	84	0.02	0.00
86	->	85	-35.41	0.86
86	->	86	-173.92	3.84

86	->	87	-36.23	0.76
86	->	88	-0.33	0.07
86	->	89	-0.02	0.01
86	->	90	0.01	0.00
86	->	91	0.01	0.00
86	->	92	0.01	0.00
86	->	93	0.01	0.00
86	->	94	0.01	0.00
86	->	95	0.00	0.00
86	->	96	0.00	0.00
86	->	97	0.00	0.00
86	->	98	0.00	0.00
86	->	99	0.01	0.00
86	->	100	0.01	0.00
86	->	101	0.02	0.00
86	->	102	0.01	0.00
86	->	103	-0.01	0.01
86	->	104	-0.10	0.05
86	->	105	-0.91	0.63
86	->	106	-10.59	0.59
87	->	1	-0.01	0.00
87	->	2	-0.00	0.00
87	->	3	0.00	0.00
87	->	4	-0.00	0.00
87	->	5	0.01	0.00
87	->	6	-0.00	0.00
87	->	7	0.00	0.00
87	->	8	0.00	0.00
87	->	9	0.00	0.00
87	->	10	0.01	0.00
87	->	11	0.01	0.00
87	->	12	-0.01	0.01
87	->	13	-0.00	0.00
87	->	14	-0.00	0.00
87	->	15	-0.00	0.00
87	->	16	0.01	0.00
87	->	17	0.00	0.00
87	->	18	0.00	0.00
87	->	19	0.00	0.00
87	->	20	0.00	0.00
87	->	21	0.00	0.00
87	->	22	-0.02	0.00
87	->	23	-0.00	0.00
87	->	24	0.00	0.00
87	->	25	-0.01	0.00
87	->	26	-0.00	0.00
87	->	27	-0.00	0.00
87	->	28	-0.00	0.00
87	->	29	-0.00	0.00
87	->	30	0.00	0.00

87 ->	31	-0.03	0.01
87 ->	32	-0.00	0.00
87 ->	33	-0.00	0.00
87 ->	34	-0.00	0.00
87 ->	35	0.06	0.02
87 ->	36	-0.03	0.01
87 ->	37	-0.01	0.01
87 ->	38	0.09	0.03
87 ->	39	-0.00	0.00
87 ->	40	-0.49	0.55
87 ->	41	-0.00	0.00
87 ->	42	0.00	0.01
87 ->	43	-0.00	0.00
87 ->	44	-0.04	0.01
87 ->	45	-0.00	0.00
87 ->	46	0.02	0.00
87 ->	47	-0.04	0.01
87 ->	48	0.00	0.01
87 ->	49	-0.02	0.01
87 ->	50	-0.10	0.07
87 ->	51	-0.55	0.36
87 ->	52	0.00	0.02
87 ->	53	-0.36	0.23
87 ->	54	-2.11	1.39
87 ->	55	0.06	0.06
87 ->	56	-0.03	0.02
87 ->	57	-0.08	0.09
87 ->	58	0.04	0.02
87 ->	59	-0.42	0.45
87 ->	60	-0.01	0.00
87 ->	61	-0.01	0.00
87 ->	62	-0.00	0.00
87 ->	63	0.01	0.00
87 ->	64	0.01	0.00
87 ->	65	0.00	0.00
87 ->	66	0.02	0.00
87 ->	67	-0.00	0.00
87 ->	68	-0.08	0.08
87 ->	69	-0.04	0.01
87 ->	70	-0.08	0.04
87 ->	71	-0.13	0.09
87 ->	72	-2.66	0.77
87 ->	73	-0.01	0.00
87 ->	74	-0.50	0.49
87 ->	75	-1.38	2.01
87 ->	76	-0.03	0.00
87 ->	77	-0.01	0.01
87 ->	78	-0.24	0.95
87 ->	79	-0.01	0.00
87 ->	80	-0.00	0.00

87	->	81	-0.02	0.00
87	->	82	-0.00	0.00
87	->	83	-0.00	0.00
87	->	84	0.01	0.00
87	->	85	-0.31	0.07
87	->	86	-36.03	0.77
87	->	87	-209.05	3.64
87	->	88	-33.04	1.43
87	->	89	-0.27	0.08
87	->	90	0.00	0.01
87	->	91	0.01	0.00
87	->	92	0.01	0.00
87	->	93	0.00	0.00
87	->	94	0.00	0.00
87	->	95	0.00	0.00
87	->	96	0.00	0.00
87	->	97	0.00	0.00
87	->	98	0.01	0.00
87	->	99	0.01	0.00
87	->	100	0.01	0.00
87	->	101	0.00	0.00
87	->	102	-0.02	0.01
87	->	103	-0.11	0.05
87	->	104	-0.53	0.29
87	->	105	-11.30	0.83
87	->	106	0.10	0.16
88	->	1	-0.01	0.00
88	->	2	-0.00	0.00
88	->	3	0.00	0.00
88	->	4	-0.00	0.00
88	->	5	0.01	0.00
88	->	6	-0.00	0.00
88	->	7	-0.00	0.00
88	->	8	0.00	0.00
88	->	9	-0.00	0.00
88	->	10	0.01	0.00
88	->	11	0.01	0.00
88	->	12	-0.02	0.01
88	->	13	-0.00	0.00
88	->	14	-0.00	0.00
88	->	15	-0.00	0.00
88	->	16	0.01	0.00
88	->	17	0.00	0.00
88	->	18	0.00	0.00
88	->	19	0.00	0.00
88	->	20	-0.00	0.00
88	->	21	-0.00	0.00
88	->	22	-0.03	0.00
88	->	23	-0.01	0.00
88	->	24	-0.00	0.00

88	->	25	-0.02	0.00
88	->	26	-0.01	0.00
88	->	27	-0.00	0.00
88	->	28	-0.00	0.00
88	->	29	-0.00	0.00
88	->	30	0.00	0.00
88	->	31	-0.09	0.06
88	->	32	-0.00	0.00
88	->	33	-0.00	0.00
88	->	34	-0.01	0.00
88	->	35	0.09	0.03
88	->	36	-0.03	0.01
88	->	37	-0.02	0.01
88	->	38	0.14	0.06
88	->	39	-0.01	0.00
88	->	40	-3.95	3.65
88	->	41	-0.02	0.01
88	->	42	-0.17	0.12
88	->	43	-0.00	0.01
88	->	44	-0.08	0.01
88	->	45	-0.00	0.00
88	->	46	0.03	0.01
88	->	47	-0.22	0.06
88	->	48	-0.00	0.02
88	->	49	-0.03	0.02
88	->	50	-0.23	0.17
88	->	51	-3.92	2.85
88	->	52	0.04	0.03
88	->	53	-0.15	0.10
88	->	54	-1.07	1.66
88	->	55	0.04	0.05
88	->	56	-0.01	0.01
88	->	57	-0.01	0.00
88	->	58	0.03	0.00
88	->	59	-0.05	0.01
88	->	60	-0.00	0.00
88	->	61	-0.00	0.00
88	->	62	-0.00	0.00
88	->	63	0.01	0.00
88	->	64	0.01	0.00
88	->	65	0.00	0.00
88	->	66	0.01	0.00
88	->	67	0.00	0.00
88	->	68	-0.03	0.01
88	->	69	-0.02	0.00
88	->	70	-0.01	0.01
88	->	71	-0.03	0.01
88	->	72	-8.75	0.85
88	->	73	-0.01	0.01
88	->	74	0.46	0.18

88	->	75	0.09	0.10
88	->	76	-0.02	0.00
88	->	77	-0.03	0.00
88	->	78	-0.24	0.37
88	->	79	-0.01	0.00
88	->	80	-0.00	0.00
88	->	81	-0.02	0.00
88	->	82	-0.00	0.00
88	->	83	-0.00	0.00
88	->	84	0.01	0.00
88	->	85	-0.03	0.01
88	->	86	-0.33	0.07
88	->	87	-32.85	1.41
88	->	88	-168.10	2.93
88	->	89	-35.51	0.90
88	->	90	-0.32	0.07
88	->	91	-0.01	0.01
88	->	92	0.01	0.00
88	->	93	0.01	0.00
88	->	94	0.01	0.00
88	->	95	0.00	0.00
88	->	96	0.00	0.00
88	->	97	0.01	0.00
88	->	98	0.01	0.00
88	->	99	0.02	0.00
88	->	100	0.01	0.00
88	->	101	-0.00	0.00
88	->	102	-0.08	0.03
88	->	103	-1.67	0.60
88	->	104	-11.39	0.67
88	->	105	-4.37	0.89
88	->	106	-0.26	0.08
89	->	1	-0.03	0.01
89	->	2	-0.00	0.00
89	->	3	-0.00	0.00
89	->	4	-0.00	0.00
89	->	5	0.02	0.00
89	->	6	-0.00	0.00
89	->	7	-0.00	0.00
89	->	8	0.01	0.00
89	->	9	-0.00	0.00
89	->	10	0.04	0.01
89	->	11	0.02	0.01
89	->	12	-0.10	0.09
89	->	13	-0.01	0.00
89	->	14	-0.01	0.00
89	->	15	-0.00	0.00
89	->	16	0.01	0.00
89	->	17	0.00	0.00
89	->	18	0.00	0.00

89	->	19	-0.00	0.00
89	->	20	-0.00	0.00
89	->	21	-0.00	0.00
89	->	22	-0.09	0.03
89	->	23	-0.03	0.01
89	->	24	0.00	0.00
89	->	25	-0.06	0.02
89	->	26	-0.11	0.05
89	->	27	0.00	0.01
89	->	28	-0.00	0.00
89	->	29	-0.01	0.00
89	->	30	-0.00	0.00
89	->	31	-0.32	0.45
89	->	32	-0.00	0.00
89	->	33	-0.01	0.00
89	->	34	-0.01	0.00
89	->	35	0.04	0.01
89	->	36	-0.02	0.01
89	->	37	-0.00	0.00
89	->	38	0.05	0.01
89	->	39	-0.00	0.00
89	->	40	-4.17	3.90
89	->	41	-0.04	0.03
89	->	42	-0.21	0.15
89	->	43	-0.02	0.03
89	->	44	-0.28	0.16
89	->	45	-0.00	0.00
89	->	46	-0.03	0.04
89	->	47	-2.63	0.91
89	->	48	-0.02	0.01
89	->	49	-0.02	0.01
89	->	50	-0.32	0.30
89	->	51	-0.08	0.04
89	->	52	-0.01	0.00
89	->	53	-0.04	0.01
89	->	54	-0.02	0.01
89	->	55	-0.00	0.00
89	->	56	-0.00	0.00
89	->	57	-0.00	0.00
89	->	58	0.01	0.00
89	->	59	-0.02	0.00
89	->	60	-0.00	0.00
89	->	61	-0.00	0.00
89	->	62	0.00	0.00
89	->	63	0.01	0.00
89	->	64	0.00	0.00
89	->	65	0.00	0.00
89	->	66	0.01	0.00
89	->	67	0.00	0.00
89	->	68	-0.01	0.00

89	->	69	-0.01	0.00
89	->	70	-0.00	0.00
89	->	71	-0.01	0.00
89	->	72	-2.02	0.77
89	->	73	-0.01	0.01
89	->	74	0.15	0.14
89	->	75	0.01	0.01
89	->	76	-0.02	0.00
89	->	77	-0.02	0.00
89	->	78	-0.04	0.02
89	->	79	-0.00	0.00
89	->	80	-0.00	0.00
89	->	81	-0.02	0.00
89	->	82	-0.00	0.00
89	->	83	-0.00	0.00
89	->	84	0.00	0.00
89	->	85	-0.00	0.00
89	->	86	-0.02	0.01
89	->	87	-0.27	0.07
89	->	88	-35.30	0.88
89	->	89	-90.75	4.20
89	->	90	-33.32	0.79
89	->	91	-0.19	0.05
89	->	92	0.00	0.01
89	->	93	0.01	0.00
89	->	94	0.01	0.00
89	->	95	0.00	0.00
89	->	96	0.00	0.00
89	->	97	0.01	0.00
89	->	98	0.01	0.00
89	->	99	0.01	0.00
89	->	100	-0.00	0.01
89	->	101	-0.06	0.03
89	->	102	-0.54	0.22
89	->	103	-6.06	0.55
89	->	104	-0.64	0.26
89	->	105	-0.22	0.07
89	->	106	-0.02	0.02
90	->	1	-0.11	0.07
90	->	2	-0.00	0.00
90	->	3	-0.00	0.00
90	->	4	-0.01	0.01
90	->	5	0.09	0.03
90	->	6	-0.00	0.00
90	->	7	-0.02	0.01
90	->	8	0.02	0.00
90	->	9	-0.01	0.01
90	->	10	0.16	0.07
90	->	11	0.03	0.04
90	->	12	-3.09	2.82

90 ->	13	-0.06	0.02
90 ->	14	-0.06	0.05
90 ->	15	-0.01	0.01
90 ->	16	0.03	0.01
90 ->	17	-0.00	0.00
90 ->	18	-0.01	0.01
90 ->	19	-0.12	0.05
90 ->	20	0.01	0.01
90 ->	21	-0.01	0.01
90 ->	22	-0.51	0.23
90 ->	23	-0.83	0.36
90 ->	24	0.01	0.01
90 ->	25	-0.12	0.06
90 ->	26	-1.09	0.85
90 ->	27	0.04	0.03
90 ->	28	-0.01	0.00
90 ->	29	-0.01	0.00
90 ->	30	-0.00	0.00
90 ->	31	-0.13	0.14
90 ->	32	-0.01	0.00
90 ->	33	-0.01	0.00
90 ->	34	-0.00	0.00
90 ->	35	0.02	0.01
90 ->	36	-0.01	0.00
90 ->	37	-0.00	0.00
90 ->	38	0.02	0.01
90 ->	39	-0.00	0.00
90 ->	40	-0.12	0.12
90 ->	41	-0.01	0.01
90 ->	42	-0.06	0.05
90 ->	43	-0.08	0.09
90 ->	44	-2.33	0.62
90 ->	45	-0.00	0.00
90 ->	46	0.11	0.06
90 ->	47	-3.31	0.71
90 ->	48	-0.00	0.00
90 ->	49	-0.01	0.00
90 ->	50	-0.02	0.01
90 ->	51	-0.01	0.00
90 ->	52	-0.00	0.00
90 ->	53	-0.03	0.00
90 ->	54	-0.00	0.00
90 ->	55	-0.00	0.00
90 ->	56	-0.00	0.00
90 ->	57	-0.00	0.00
90 ->	58	0.01	0.00
90 ->	59	-0.01	0.00
90 ->	60	-0.00	0.00
90 ->	61	-0.00	0.00
90 ->	62	0.00	0.00

90	->	63	0.01	0.00
90	->	64	0.00	0.00
90	->	65	0.00	0.00
90	->	66	0.00	0.00
90	->	67	0.00	0.00
90	->	68	-0.01	0.00
90	->	69	-0.01	0.00
90	->	70	-0.00	0.00
90	->	71	-0.00	0.00
90	->	72	-0.12	0.07
90	->	73	-0.00	0.01
90	->	74	-0.05	0.06
90	->	75	0.01	0.01
90	->	76	-0.01	0.00
90	->	77	-0.01	0.01
90	->	78	-0.03	0.01
90	->	79	-0.00	0.00
90	->	80	-0.00	0.00
90	->	81	-0.01	0.00
90	->	82	-0.00	0.00
90	->	83	-0.00	0.00
90	->	84	0.00	0.00
90	->	85	0.00	0.00
90	->	86	0.01	0.00
90	->	87	0.00	0.01
90	->	88	-0.32	0.07
90	->	89	-33.08	0.79
90	->	90	-173.00	2.65
90	->	91	-33.46	0.81
90	->	92	-0.25	0.12
90	->	93	-0.02	0.01
90	->	94	0.00	0.00
90	->	95	0.00	0.00
90	->	96	0.00	0.00
90	->	97	0.01	0.00
90	->	98	0.02	0.01
90	->	99	-0.00	0.01
90	->	100	-0.08	0.03
90	->	101	-1.32	0.50
90	->	102	-11.65	0.69
90	->	103	-3.78	0.59
90	->	104	-0.29	0.09
90	->	105	-0.03	0.02
90	->	106	0.01	0.00
91	->	1	-2.79	2.34
91	->	2	-0.01	0.01
91	->	3	-0.01	0.02
91	->	4	-0.03	0.01
91	->	5	0.16	0.05
91	->	6	-0.01	0.00

91	->	7	-0.02	0.01
91	->	8	0.02	0.00
91	->	9	-0.01	0.00
91	->	10	0.22	0.09
91	->	11	0.01	0.02
91	->	12	-4.62	3.29
91	->	13	-0.42	0.37
91	->	14	-2.89	0.92
91	->	15	-0.86	0.53
91	->	16	0.14	0.13
91	->	17	0.01	0.02
91	->	18	-0.08	0.06
91	->	19	-1.96	0.94
91	->	20	0.02	0.05
91	->	21	-0.06	0.04
91	->	22	-3.83	1.03
91	->	23	-4.58	1.73
91	->	24	0.06	0.03
91	->	25	-0.11	0.04
91	->	26	-0.27	0.27
91	->	27	0.10	0.06
91	->	28	-0.01	0.00
91	->	29	-0.01	0.00
91	->	30	-0.00	0.00
91	->	31	-0.04	0.01
91	->	32	-0.00	0.00
91	->	33	-0.00	0.00
91	->	34	-0.00	0.00
91	->	35	0.01	0.00
91	->	36	-0.01	0.00
91	->	37	-0.00	0.00
91	->	38	0.01	0.00
91	->	39	0.00	0.00
91	->	40	-0.03	0.01
91	->	41	-0.00	0.00
91	->	42	-0.02	0.01
91	->	43	-0.03	0.02
91	->	44	-9.10	0.76
91	->	45	-0.00	0.01
91	->	46	0.64	0.20
91	->	47	-0.56	0.36
91	->	48	-0.00	0.00
91	->	49	-0.01	0.00
91	->	50	-0.01	0.00
91	->	51	-0.01	0.00
91	->	52	-0.00	0.00
91	->	53	-0.02	0.00
91	->	54	-0.00	0.00
91	->	55	-0.00	0.00
91	->	56	-0.00	0.00

91	->	57	-0.00	0.00
91	->	58	0.01	0.00
91	->	59	-0.01	0.00
91	->	60	-0.00	0.00
91	->	61	-0.00	0.00
91	->	62	0.00	0.00
91	->	63	0.00	0.00
91	->	64	0.00	0.00
91	->	65	0.00	0.00
91	->	66	0.00	0.00
91	->	67	0.00	0.00
91	->	68	-0.01	0.00
91	->	69	-0.00	0.00
91	->	70	-0.00	0.00
91	->	71	0.00	0.00
91	->	72	-0.01	0.03
91	->	73	-0.00	0.03
91	->	74	-0.04	0.03
91	->	75	0.01	0.00
91	->	76	-0.01	0.00
91	->	77	-0.00	0.01
91	->	78	-0.02	0.00
91	->	79	-0.00	0.00
91	->	80	-0.00	0.00
91	->	81	-0.01	0.00
91	->	82	-0.00	0.00
91	->	83	-0.00	0.00
91	->	84	0.00	0.00
91	->	85	0.00	0.00
91	->	86	0.01	0.00
91	->	87	0.01	0.00
91	->	88	-0.01	0.01
91	->	89	-0.19	0.05
91	->	90	-33.34	0.80
91	->	91	-160.04	3.62
91	->	92	-32.47	1.38
91	->	93	-0.34	0.11
91	->	94	-0.04	0.02
91	->	95	-0.00	0.00
91	->	96	-0.00	0.00
91	->	97	0.01	0.00
91	->	98	0.00	0.02
91	->	99	-0.07	0.08
91	->	100	-0.97	0.47
91	->	101	-11.62	0.94
91	->	102	-3.31	0.70
91	->	103	-0.33	0.08
91	->	104	-0.03	0.02
91	->	105	0.01	0.00
91	->	106	0.01	0.00

92	->	1	-10.22	2.60
92	->	2	-0.04	0.03
92	->	3	-0.00	0.04
92	->	4	0.00	0.01
92	->	5	0.06	0.01
92	->	6	-0.00	0.00
92	->	7	-0.00	0.00
92	->	8	0.02	0.00
92	->	9	-0.00	0.00
92	->	10	0.04	0.01
92	->	11	0.03	0.00
92	->	12	-0.53	0.63
92	->	13	-0.55	0.35
92	->	14	-3.96	1.71
92	->	15	-8.13	4.21
92	->	16	0.27	0.40
92	->	17	0.07	0.06
92	->	18	-0.21	0.09
92	->	19	-4.89	2.04
92	->	20	0.06	0.04
92	->	21	-0.04	0.02
92	->	22	-8.52	1.45
92	->	23	-0.06	0.02
92	->	24	-0.00	0.01
92	->	25	-0.05	0.01
92	->	26	-0.01	0.00
92	->	27	0.00	0.00
92	->	28	-0.00	0.00
92	->	29	-0.00	0.00
92	->	30	0.00	0.00
92	->	31	-0.02	0.00
92	->	32	-0.00	0.00
92	->	33	-0.00	0.00
92	->	34	-0.00	0.00
92	->	35	0.01	0.00
92	->	36	-0.00	0.00
92	->	37	-0.00	0.00
92	->	38	0.01	0.00
92	->	39	0.00	0.00
92	->	40	-0.01	0.00
92	->	41	-0.00	0.00
92	->	42	-0.01	0.00
92	->	43	-0.01	0.00
92	->	44	-1.47	0.60
92	->	45	-0.00	0.00
92	->	46	0.23	0.12
92	->	47	-0.04	0.02
92	->	48	-0.00	0.00
92	->	49	-0.00	0.00
92	->	50	-0.01	0.00

92	->	51	-0.00	0.00
92	->	52	-0.00	0.00
92	->	53	-0.01	0.00
92	->	54	-0.00	0.00
92	->	55	-0.00	0.00
92	->	56	-0.00	0.00
92	->	57	-0.00	0.00
92	->	58	0.01	0.00
92	->	59	-0.01	0.00
92	->	60	-0.00	0.00
92	->	61	-0.01	0.00
92	->	62	0.00	0.00
92	->	63	0.00	0.00
92	->	64	0.00	0.00
92	->	65	0.00	0.00
92	->	66	0.00	0.00
92	->	67	-0.00	0.00
92	->	68	-0.00	0.00
92	->	69	-0.00	0.00
92	->	70	-0.00	0.00
92	->	71	0.00	0.00
92	->	72	-0.01	0.01
92	->	73	-0.02	0.02
92	->	74	-0.01	0.01
92	->	75	0.01	0.00
92	->	76	-0.01	0.01
92	->	77	-0.03	0.01
92	->	78	-0.02	0.00
92	->	79	-0.00	0.00
92	->	80	-0.00	0.00
92	->	81	-0.02	0.01
92	->	82	-0.00	0.00
92	->	83	-0.00	0.00
92	->	84	0.00	0.00
92	->	85	0.00	0.00
92	->	86	0.01	0.00
92	->	87	0.01	0.00
92	->	88	0.01	0.00
92	->	89	0.00	0.01
92	->	90	-0.25	0.12
92	->	91	-32.41	1.36
92	->	92	-156.62	4.91
92	->	93	-36.36	0.88
92	->	94	-0.47	0.08
92	->	95	-0.04	0.01
92	->	96	-0.00	0.00
92	->	97	0.01	0.01
92	->	98	-0.04	0.06
92	->	99	-0.27	0.33
92	->	100	-11.73	0.73

92	->	101	-3.91	0.72
92	->	102	-0.39	0.10
92	->	103	-0.05	0.02
92	->	104	0.01	0.00
92	->	105	0.02	0.00
92	->	106	0.01	0.00
93	->	1	-0.93	1.78
93	->	2	-0.02	0.01
93	->	3	-0.02	0.01
93	->	4	-0.00	0.00
93	->	5	0.02	0.00
93	->	6	-0.00	0.00
93	->	7	-0.00	0.00
93	->	8	0.01	0.00
93	->	9	0.00	0.00
93	->	10	0.02	0.00
93	->	11	0.02	0.00
93	->	12	-0.05	0.01
93	->	13	-0.04	0.01
93	->	14	-0.11	0.04
93	->	15	-0.53	0.31
93	->	16	-0.47	0.38
93	->	17	-0.02	0.01
93	->	18	-0.12	0.10
93	->	19	-3.92	2.15
93	->	20	-0.02	0.01
93	->	21	-0.00	0.01
93	->	22	-0.09	0.79
93	->	23	-0.01	0.00
93	->	24	-0.00	0.00
93	->	25	-0.02	0.00
93	->	26	-0.01	0.00
93	->	27	-0.00	0.00
93	->	28	-0.00	0.00
93	->	29	-0.00	0.00
93	->	30	-0.00	0.00
93	->	31	-0.01	0.00
93	->	32	-0.00	0.00
93	->	33	-0.00	0.00
93	->	34	0.00	0.00
93	->	35	0.00	0.00
93	->	36	-0.00	0.00
93	->	37	-0.00	0.00
93	->	38	0.00	0.00
93	->	39	0.00	0.00
93	->	40	-0.01	0.00
93	->	41	-0.00	0.00
93	->	42	-0.00	0.00
93	->	43	0.00	0.00
93	->	44	-0.04	0.05

93	->	45	-0.01	0.00
93	->	46	-0.02	0.04
93	->	47	-0.01	0.00
93	->	48	-0.00	0.00
93	->	49	-0.00	0.00
93	->	50	-0.00	0.00
93	->	51	-0.00	0.00
93	->	52	-0.00	0.00
93	->	53	-0.02	0.00
93	->	54	-0.00	0.00
93	->	55	-0.00	0.00
93	->	56	-0.00	0.00
93	->	57	-0.00	0.00
93	->	58	0.01	0.00
93	->	59	-0.01	0.00
93	->	60	-0.00	0.00
93	->	61	-0.00	0.00
93	->	62	0.00	0.00
93	->	63	0.01	0.00
93	->	64	0.01	0.00
93	->	65	0.00	0.00
93	->	66	0.00	0.00
93	->	67	0.00	0.00
93	->	68	-0.01	0.00
93	->	69	-0.01	0.00
93	->	70	-0.00	0.00
93	->	71	-0.00	0.00
93	->	72	-0.02	0.00
93	->	73	-0.01	0.00
93	->	74	0.02	0.00
93	->	75	0.01	0.00
93	->	76	-0.02	0.01
93	->	77	-0.05	0.01
93	->	78	-0.01	0.00
93	->	79	-0.00	0.00
93	->	80	-0.00	0.00
93	->	81	-0.02	0.01
93	->	82	-0.00	0.00
93	->	83	-0.00	0.00
93	->	84	0.01	0.00
93	->	85	0.00	0.00
93	->	86	0.01	0.00
93	->	87	0.00	0.00
93	->	88	0.01	0.00
93	->	89	0.01	0.00
93	->	90	-0.02	0.01
93	->	91	-0.34	0.12
93	->	92	-36.11	0.87
93	->	93	-131.85	3.16
93	->	94	-34.61	0.71

93	->	95	-0.25	0.05
93	->	96	-0.02	0.01
93	->	97	-0.09	0.04
93	->	98	-2.34	0.67
93	->	99	-6.10	0.64
93	->	100	-0.83	0.43
93	->	101	-0.22	0.09
93	->	102	-0.07	0.06
93	->	103	0.01	0.00
93	->	104	0.02	0.00
93	->	105	0.01	0.00
93	->	106	0.00	0.00
94	->	1	-0.07	0.02
94	->	2	-0.01	0.00
94	->	3	-0.02	0.01
94	->	4	-0.00	0.00
94	->	5	0.01	0.00
94	->	6	-0.00	0.00
94	->	7	-0.00	0.00
94	->	8	0.00	0.00
94	->	9	0.00	0.00
94	->	10	0.01	0.00
94	->	11	0.01	0.00
94	->	12	-0.02	0.00
94	->	13	-0.01	0.00
94	->	14	-0.01	0.01
94	->	15	-0.04	0.04
94	->	16	-3.66	1.61
94	->	17	-0.06	0.04
94	->	18	-0.17	0.73
94	->	19	-0.56	0.41
94	->	20	-0.01	0.01
94	->	21	-0.00	0.01
94	->	22	-0.03	0.13
94	->	23	-0.01	0.00
94	->	24	-0.00	0.00
94	->	25	-0.02	0.00
94	->	26	-0.00	0.00
94	->	27	-0.00	0.00
94	->	28	-0.00	0.00
94	->	29	-0.00	0.00
94	->	30	0.00	0.00
94	->	31	-0.01	0.00
94	->	32	-0.00	0.00
94	->	33	-0.00	0.00
94	->	34	0.00	0.00
94	->	35	0.00	0.00
94	->	36	-0.00	0.00
94	->	37	-0.00	0.00
94	->	38	0.00	0.00

94 ->	39	0.00	0.00
94 ->	40	-0.00	0.00
94 ->	41	-0.00	0.00
94 ->	42	-0.00	0.00
94 ->	43	-0.00	0.00
94 ->	44	-0.04	0.01
94 ->	45	-0.00	0.00
94 ->	46	0.03	0.01
94 ->	47	-0.00	0.00
94 ->	48	-0.00	0.00
94 ->	49	-0.00	0.00
94 ->	50	-0.00	0.00
94 ->	51	-0.00	0.00
94 ->	52	-0.00	0.00
94 ->	53	-0.02	0.00
94 ->	54	-0.00	0.00
94 ->	55	-0.00	0.00
94 ->	56	-0.00	0.00
94 ->	57	-0.00	0.00
94 ->	58	0.01	0.00
94 ->	59	-0.01	0.00
94 ->	60	-0.00	0.00
94 ->	61	-0.00	0.00
94 ->	62	0.00	0.00
94 ->	63	0.01	0.00
94 ->	64	0.01	0.00
94 ->	65	0.00	0.00
94 ->	66	0.00	0.00
94 ->	67	-0.00	0.00
94 ->	68	-0.00	0.00
94 ->	69	-0.01	0.00
94 ->	70	-0.00	0.00
94 ->	71	0.00	0.00
94 ->	72	-0.02	0.00
94 ->	73	-0.00	0.00
94 ->	74	0.02	0.00
94 ->	75	0.01	0.00
94 ->	76	-0.02	0.01
94 ->	77	-0.03	0.01
94 ->	78	-0.01	0.00
94 ->	79	-0.00	0.00
94 ->	80	-0.00	0.00
94 ->	81	-0.01	0.00
94 ->	82	-0.00	0.00
94 ->	83	-0.00	0.00
94 ->	84	0.01	0.00
94 ->	85	0.00	0.00
94 ->	86	0.01	0.00
94 ->	87	0.00	0.00
94 ->	88	0.01	0.00

94	->	89	0.01	0.00
94	->	90	0.00	0.00
94	->	91	-0.04	0.02
94	->	92	-0.47	0.08
94	->	93	-34.41	0.71
94	->	94	-216.86	2.70
94	->	95	-32.79	0.75
94	->	96	-0.11	0.05
94	->	97	-0.93	0.53
94	->	98	-11.29	0.79
94	->	99	-0.73	0.33
94	->	100	0.02	0.05
94	->	101	-0.01	0.02
94	->	102	0.02	0.01
94	->	103	0.02	0.00
94	->	104	0.01	0.00
94	->	105	0.01	0.00
94	->	106	0.00	0.00
95	->	1	-0.02	0.01
95	->	2	-0.00	0.00
95	->	3	-0.01	0.00
95	->	4	-0.00	0.00
95	->	5	0.00	0.00
95	->	6	-0.00	0.00
95	->	7	-0.00	0.00
95	->	8	0.00	0.00
95	->	9	0.00	0.00
95	->	10	0.00	0.00
95	->	11	0.00	0.00
95	->	12	-0.01	0.00
95	->	13	-0.01	0.00
95	->	14	-0.00	0.00
95	->	15	-0.01	0.00
95	->	16	-0.52	0.57
95	->	17	-0.03	0.03
95	->	18	-0.23	0.22
95	->	19	-0.04	0.02
95	->	20	-0.00	0.00
95	->	21	-0.01	0.00
95	->	22	-0.04	0.02
95	->	23	-0.00	0.00
95	->	24	-0.00	0.00
95	->	25	-0.02	0.00
95	->	26	-0.00	0.00
95	->	27	-0.00	0.00
95	->	28	-0.00	0.00
95	->	29	-0.00	0.00
95	->	30	0.00	0.00
95	->	31	-0.00	0.00
95	->	32	-0.00	0.00

95	->	33	-0.00	0.00
95	->	34	0.00	0.00
95	->	35	0.00	0.00
95	->	36	-0.00	0.00
95	->	37	-0.00	0.00
95	->	38	0.00	0.00
95	->	39	0.00	0.00
95	->	40	-0.00	0.00
95	->	41	-0.00	0.00
95	->	42	-0.00	0.00
95	->	43	-0.00	0.00
95	->	44	-0.02	0.00
95	->	45	-0.00	0.00
95	->	46	0.02	0.00
95	->	47	-0.00	0.00
95	->	48	-0.00	0.00
95	->	49	-0.00	0.00
95	->	50	-0.00	0.00
95	->	51	-0.00	0.00
95	->	52	-0.00	0.00
95	->	53	-0.01	0.00
95	->	54	-0.00	0.00
95	->	55	-0.00	0.00
95	->	56	-0.00	0.00
95	->	57	-0.00	0.00
95	->	58	0.01	0.00
95	->	59	-0.00	0.00
95	->	60	-0.00	0.00
95	->	61	-0.00	0.00
95	->	62	0.00	0.00
95	->	63	0.00	0.00
95	->	64	0.00	0.00
95	->	65	0.00	0.00
95	->	66	0.00	0.00
95	->	67	0.00	0.00
95	->	68	-0.00	0.00
95	->	69	-0.00	0.00
95	->	70	0.00	0.00
95	->	71	0.00	0.00
95	->	72	-0.01	0.00
95	->	73	-0.00	0.00
95	->	74	0.01	0.00
95	->	75	0.00	0.00
95	->	76	-0.01	0.00
95	->	77	-0.01	0.00
95	->	78	-0.00	0.00
95	->	79	-0.00	0.00
95	->	80	-0.00	0.00
95	->	81	-0.01	0.00
95	->	82	-0.00	0.00

95	->	83	0.00	0.00
95	->	84	0.00	0.00
95	->	85	0.00	0.00
95	->	86	0.00	0.00
95	->	87	0.00	0.00
95	->	88	0.00	0.00
95	->	89	0.00	0.00
95	->	90	0.00	0.00
95	->	91	-0.00	0.00
95	->	92	-0.04	0.01
95	->	93	-0.25	0.05
95	->	94	-32.49	0.75
95	->	95	-215.14	3.01
95	->	96	-2.80	1.65
95	->	97	-10.57	0.73
95	->	98	-2.52	1.01
95	->	99	-0.14	0.07
95	->	100	-0.01	0.05
95	->	101	0.01	0.01
95	->	102	0.01	0.00
95	->	103	0.01	0.00
95	->	104	0.00	0.00
95	->	105	0.00	0.00
95	->	106	0.00	0.00
96	->	1	-0.01	0.00
96	->	2	-0.00	0.00
96	->	3	-0.01	0.01
96	->	4	-0.00	0.00
96	->	5	0.00	0.00
96	->	6	-0.00	0.00
96	->	7	-0.00	0.00
96	->	8	0.00	0.00
96	->	9	-0.00	0.00
96	->	10	0.00	0.00
96	->	11	0.00	0.00
96	->	12	-0.00	0.00
96	->	13	-0.00	0.00
96	->	14	-0.00	0.00
96	->	15	-0.00	0.00
96	->	16	-0.05	0.07
96	->	17	-0.69	1.08
96	->	18	-0.76	0.75
96	->	19	-0.03	0.02
96	->	20	-0.02	0.03
96	->	21	-0.31	0.46
96	->	22	-0.03	0.02
96	->	23	-0.00	0.00
96	->	24	-0.01	0.01
96	->	25	-0.03	0.02
96	->	26	-0.00	0.00

96	->	27	-0.00	0.00
96	->	28	-0.00	0.00
96	->	29	-0.00	0.00
96	->	30	-0.00	0.00
96	->	31	-0.00	0.00
96	->	32	-0.00	0.00
96	->	33	-0.01	0.00
96	->	34	-0.00	0.00
96	->	35	0.00	0.00
96	->	36	-0.00	0.00
96	->	37	-0.00	0.00
96	->	38	0.00	0.00
96	->	39	0.00	0.00
96	->	40	-0.00	0.00
96	->	41	-0.00	0.00
96	->	42	-0.00	0.00
96	->	43	-0.00	0.00
96	->	44	-0.01	0.00
96	->	45	-0.01	0.00
96	->	46	0.00	0.00
96	->	47	-0.00	0.00
96	->	48	-0.00	0.00
96	->	49	-0.00	0.00
96	->	50	-0.00	0.00
96	->	51	-0.00	0.00
96	->	52	-0.00	0.00
96	->	53	-0.00	0.00
96	->	54	-0.00	0.00
96	->	55	-0.00	0.00
96	->	56	-0.00	0.00
96	->	57	-0.00	0.00
96	->	58	0.00	0.00
96	->	59	-0.00	0.00
96	->	60	-0.00	0.00
96	->	61	-0.00	0.00
96	->	62	0.00	0.00
96	->	63	0.00	0.00
96	->	64	0.00	0.00
96	->	65	0.00	0.00
96	->	66	0.00	0.00
96	->	67	0.00	0.00
96	->	68	-0.00	0.00
96	->	69	-0.00	0.00
96	->	70	0.00	0.00
96	->	71	0.00	0.00
96	->	72	-0.00	0.00
96	->	73	-0.00	0.00
96	->	74	0.00	0.00
96	->	75	0.00	0.00
96	->	76	-0.00	0.00

96	->	77	-0.00	0.00
96	->	78	-0.00	0.00
96	->	79	-0.00	0.00
96	->	80	-0.00	0.00
96	->	81	-0.00	0.00
96	->	82	-0.00	0.00
96	->	83	0.00	0.00
96	->	84	0.00	0.00
96	->	85	0.00	0.00
96	->	86	0.00	0.00
96	->	87	0.00	0.00
96	->	88	0.00	0.00
96	->	89	0.00	0.00
96	->	90	0.00	0.00
96	->	91	-0.00	0.00
96	->	92	-0.00	0.00
96	->	93	-0.02	0.01
96	->	94	-0.11	0.05
96	->	95	-2.73	1.62
96	->	96	-47.81	2.84
96	->	97	-36.50	1.44
96	->	98	-0.42	0.11
96	->	99	-0.06	0.05
96	->	100	-0.00	0.00
96	->	101	0.00	0.00
96	->	102	0.00	0.00
96	->	103	0.00	0.00
96	->	104	0.00	0.00
96	->	105	0.00	0.00
96	->	106	0.00	0.00
97	->	1	-0.02	0.00
97	->	2	-0.00	0.00
97	->	3	-0.01	0.00
97	->	4	-0.00	0.00
97	->	5	0.01	0.00
97	->	6	-0.00	0.00
97	->	7	-0.00	0.00
97	->	8	0.01	0.00
97	->	9	-0.00	0.00
97	->	10	0.01	0.00
97	->	11	0.01	0.00
97	->	12	-0.01	0.00
97	->	13	-0.01	0.00
97	->	14	-0.01	0.00
97	->	15	-0.00	0.00
97	->	16	0.11	0.07
97	->	17	-0.09	0.04
97	->	18	-2.76	1.20
97	->	19	0.01	0.02
97	->	20	-0.03	0.01

97	->	21	-0.29	0.27
97	->	22	-0.13	0.06
97	->	23	-0.01	0.00
97	->	24	-0.01	0.01
97	->	25	-0.79	0.85
97	->	26	-0.01	0.00
97	->	27	-0.00	0.00
97	->	28	-0.00	0.00
97	->	29	-0.00	0.00
97	->	30	-0.01	0.01
97	->	31	-0.02	0.01
97	->	32	-0.02	0.01
97	->	33	-0.08	0.08
97	->	34	-0.00	0.00
97	->	35	0.01	0.00
97	->	36	-0.02	0.01
97	->	37	-0.00	0.00
97	->	38	0.01	0.00
97	->	39	0.00	0.00
97	->	40	-0.01	0.00
97	->	41	-0.00	0.00
97	->	42	-0.01	0.00
97	->	43	-0.01	0.01
97	->	44	-0.07	0.02
97	->	45	-0.05	0.08
97	->	46	0.02	0.01
97	->	47	-0.01	0.00
97	->	48	-0.01	0.01
97	->	49	-0.01	0.00
97	->	50	-0.00	0.00
97	->	51	-0.00	0.00
97	->	52	-0.00	0.00
97	->	53	-0.02	0.00
97	->	54	-0.00	0.00
97	->	55	-0.00	0.00
97	->	56	-0.00	0.00
97	->	57	-0.00	0.00
97	->	58	0.01	0.00
97	->	59	-0.00	0.00
97	->	60	-0.00	0.00
97	->	61	-0.00	0.00
97	->	62	0.00	0.00
97	->	63	0.00	0.00
97	->	64	0.00	0.00
97	->	65	0.00	0.00
97	->	66	0.00	0.00
97	->	67	0.00	0.00
97	->	68	-0.00	0.00
97	->	69	-0.00	0.00
97	->	70	0.00	0.00

97	->	71	0.00	0.00
97	->	72	-0.01	0.00
97	->	73	-0.00	0.00
97	->	74	0.01	0.00
97	->	75	0.00	0.00
97	->	76	-0.00	0.00
97	->	77	-0.01	0.00
97	->	78	-0.00	0.00
97	->	79	-0.00	0.00
97	->	80	-0.00	0.00
97	->	81	-0.00	0.00
97	->	82	0.00	0.00
97	->	83	-0.00	0.00
97	->	84	0.00	0.00
97	->	85	0.00	0.00
97	->	86	0.00	0.00
97	->	87	0.00	0.00
97	->	88	0.01	0.00
97	->	89	0.01	0.00
97	->	90	0.01	0.00
97	->	91	0.01	0.00
97	->	92	0.01	0.01
97	->	93	-0.09	0.04
97	->	94	-0.93	0.53
97	->	95	-10.65	0.73
97	->	96	-36.26	1.44
97	->	97	-180.89	2.88
97	->	98	-36.24	0.88
97	->	99	-0.38	0.11
97	->	100	-0.02	0.01
97	->	101	0.00	0.00
97	->	102	0.00	0.00
97	->	103	0.00	0.00
97	->	104	0.00	0.00
97	->	105	0.00	0.00
97	->	106	0.00	0.00
98	->	1	-0.03	0.00
98	->	2	-0.00	0.00
98	->	3	-0.01	0.00
98	->	4	-0.00	0.00
98	->	5	0.02	0.00
98	->	6	-0.00	0.00
98	->	7	-0.00	0.00
98	->	8	0.01	0.00
98	->	9	-0.00	0.00
98	->	10	0.01	0.00
98	->	11	0.01	0.00
98	->	12	-0.01	0.00
98	->	13	-0.01	0.00
98	->	14	-0.01	0.00

98	->	15	-0.01	0.00
98	->	16	0.13	0.12
98	->	17	-0.06	0.03
98	->	18	-1.77	0.76
98	->	19	0.15	0.18
98	->	20	-0.02	0.02
98	->	21	-0.16	0.11
98	->	22	-1.50	1.93
98	->	23	-0.01	0.01
98	->	24	-0.02	0.01
98	->	25	-3.27	4.20
98	->	26	-0.01	0.00
98	->	27	-0.00	0.00
98	->	28	-0.00	0.00
98	->	29	-0.00	0.00
98	->	30	-0.00	0.04
98	->	31	-0.06	0.02
98	->	32	-0.09	0.08
98	->	33	-1.14	0.58
98	->	34	-0.00	0.00
98	->	35	0.03	0.00
98	->	36	-0.03	0.01
98	->	37	-0.00	0.00
98	->	38	0.02	0.00
98	->	39	-0.00	0.00
98	->	40	-0.02	0.01
98	->	41	-0.01	0.01
98	->	42	-0.02	0.01
98	->	43	-0.03	0.02
98	->	44	-0.26	0.16
98	->	45	-1.74	0.51
98	->	46	-0.16	0.11
98	->	47	-0.03	0.01
98	->	48	-0.05	0.08
98	->	49	-0.05	0.08
98	->	50	-0.01	0.00
98	->	51	-0.01	0.00
98	->	52	-0.01	0.01
98	->	53	-0.04	0.01
98	->	54	-0.00	0.00
98	->	55	-0.00	0.00
98	->	56	-0.00	0.00
98	->	57	-0.00	0.00
98	->	58	0.02	0.00
98	->	59	-0.01	0.00
98	->	60	-0.00	0.00
98	->	61	-0.00	0.00
98	->	62	0.00	0.00
98	->	63	0.00	0.00
98	->	64	0.00	0.00

98	->	65	0.00	0.00
98	->	66	0.00	0.00
98	->	67	0.00	0.00
98	->	68	-0.00	0.00
98	->	69	-0.01	0.00
98	->	70	-0.00	0.00
98	->	71	0.00	0.00
98	->	72	-0.02	0.00
98	->	73	-0.00	0.00
98	->	74	0.01	0.00
98	->	75	0.01	0.00
98	->	76	-0.01	0.00
98	->	77	-0.01	0.00
98	->	78	-0.01	0.00
98	->	79	-0.00	0.00
98	->	80	-0.00	0.00
98	->	81	-0.00	0.00
98	->	82	-0.00	0.00
98	->	83	0.00	0.00
98	->	84	0.00	0.00
98	->	85	0.00	0.00
98	->	86	0.00	0.00
98	->	87	0.01	0.00
98	->	88	0.01	0.00
98	->	89	0.01	0.00
98	->	90	0.02	0.01
98	->	91	0.00	0.02
98	->	92	-0.04	0.06
98	->	93	-2.34	0.67
98	->	94	-11.32	0.79
98	->	95	-2.52	1.00
98	->	96	-0.42	0.11
98	->	97	-36.03	0.86
98	->	98	-175.17	5.32
98	->	99	-34.80	0.61
98	->	100	-0.34	0.07
98	->	101	-0.04	0.01
98	->	102	-0.00	0.00
98	->	103	0.00	0.00
98	->	104	0.01	0.00
98	->	105	0.00	0.00
98	->	106	0.00	0.00
99	->	1	-0.03	0.00
99	->	2	-0.00	0.00
99	->	3	-0.00	0.00
99	->	4	-0.00	0.00
99	->	5	0.01	0.00
99	->	6	-0.00	0.00
99	->	7	-0.00	0.00
99	->	8	0.00	0.00

99	->	9	-0.00	0.00
99	->	10	0.01	0.00
99	->	11	0.01	0.00
99	->	12	-0.01	0.00
99	->	13	-0.01	0.00
99	->	14	-0.01	0.00
99	->	15	-0.00	0.00
99	->	16	0.02	0.01
99	->	17	-0.01	0.00
99	->	18	-0.10	0.05
99	->	19	0.04	0.08
99	->	20	-0.00	0.00
99	->	21	-0.02	0.01
99	->	22	-2.52	1.61
99	->	23	-0.01	0.00
99	->	24	-0.00	0.00
99	->	25	-0.08	0.04
99	->	26	-0.01	0.00
99	->	27	-0.00	0.00
99	->	28	-0.00	0.00
99	->	29	-0.00	0.00
99	->	30	0.00	0.00
99	->	31	-0.02	0.01
99	->	32	-0.01	0.00
99	->	33	-0.06	0.04
99	->	34	-0.00	0.00
99	->	35	0.02	0.00
99	->	36	-0.03	0.01
99	->	37	-0.00	0.00
99	->	38	0.02	0.00
99	->	39	-0.00	0.00
99	->	40	-0.02	0.01
99	->	41	-0.00	0.00
99	->	42	-0.01	0.01
99	->	43	-0.02	0.02
99	->	44	-0.70	0.47
99	->	45	-2.33	2.12
99	->	46	-1.93	0.75
99	->	47	-0.08	0.06
99	->	48	-0.08	0.04
99	->	49	-0.21	0.19
99	->	50	-0.02	0.01
99	->	51	-0.01	0.00
99	->	52	-0.02	0.01
99	->	53	-0.31	0.43
99	->	54	-0.00	0.00
99	->	55	-0.00	0.00
99	->	56	-0.00	0.00
99	->	57	-0.00	0.00
99	->	58	0.03	0.01

99	->	59	-0.02	0.01
99	->	60	-0.01	0.01
99	->	61	-0.02	0.02
99	->	62	0.00	0.00
99	->	63	0.01	0.00
99	->	64	0.01	0.00
99	->	65	0.00	0.00
99	->	66	0.01	0.00
99	->	67	0.00	0.00
99	->	68	-0.01	0.00
99	->	69	-0.02	0.01
99	->	70	-0.00	0.00
99	->	71	-0.00	0.00
99	->	72	-0.05	0.01
99	->	73	-0.01	0.01
99	->	74	0.02	0.00
99	->	75	0.01	0.00
99	->	76	-0.03	0.02
99	->	77	-0.02	0.00
99	->	78	-0.01	0.00
99	->	79	-0.00	0.00
99	->	80	-0.00	0.00
99	->	81	-0.01	0.00
99	->	82	-0.00	0.00
99	->	83	0.00	0.00
99	->	84	0.00	0.00
99	->	85	0.00	0.00
99	->	86	0.01	0.00
99	->	87	0.01	0.00
99	->	88	0.02	0.00
99	->	89	0.01	0.00
99	->	90	-0.00	0.01
99	->	91	-0.07	0.08
99	->	92	-0.27	0.33
99	->	93	-6.10	0.64
99	->	94	-0.73	0.33
99	->	95	-0.14	0.07
99	->	96	-0.06	0.05
99	->	97	-0.38	0.11
99	->	98	-34.59	0.61
99	->	99	-92.78	3.35
99	->	100	-34.05	1.24
99	->	101	-0.23	0.06
99	->	102	-0.01	0.01
99	->	103	0.01	0.00
99	->	104	0.01	0.00
99	->	105	0.00	0.00
99	->	106	0.00	0.00
100	->	1	-0.03	0.01
100	->	2	-0.00	0.00

100	->	3	-0.00	0.00
100	->	4	0.00	0.00
100	->	5	0.01	0.00
100	->	6	-0.00	0.00
100	->	7	-0.00	0.00
100	->	8	0.00	0.00
100	->	9	0.00	0.00
100	->	10	0.01	0.00
100	->	11	0.01	0.00
100	->	12	-0.01	0.00
100	->	13	-0.01	0.00
100	->	14	-0.01	0.00
100	->	15	-0.00	0.00
100	->	16	0.03	0.00
100	->	17	-0.00	0.00
100	->	18	-0.01	0.00
100	->	19	-0.03	0.02
100	->	20	-0.00	0.00
100	->	21	-0.00	0.00
100	->	22	0.21	0.25
100	->	23	-0.01	0.00
100	->	24	-0.00	0.00
100	->	25	-0.02	0.00
100	->	26	-0.00	0.00
100	->	27	-0.00	0.00
100	->	28	-0.00	0.00
100	->	29	-0.00	0.00
100	->	30	0.00	0.00
100	->	31	-0.01	0.00
100	->	32	-0.00	0.00
100	->	33	-0.01	0.00
100	->	34	0.00	0.00
100	->	35	0.01	0.00
100	->	36	-0.02	0.01
100	->	37	-0.00	0.00
100	->	38	0.01	0.00
100	->	39	0.00	0.00
100	->	40	-0.01	0.00
100	->	41	-0.00	0.00
100	->	42	-0.00	0.00
100	->	43	0.00	0.00
100	->	44	0.93	0.44
100	->	45	-0.05	0.02
100	->	46	-5.16	1.19
100	->	47	-0.23	0.11
100	->	48	-0.03	0.02
100	->	49	-0.24	0.19
100	->	50	-0.04	0.04
100	->	51	-0.01	0.00
100	->	52	-0.02	0.01

100 ->	53	-1.66	2.80
100 ->	54	-0.00	0.00
100 ->	55	-0.00	0.00
100 ->	56	-0.00	0.00
100 ->	57	-0.00	0.00
100 ->	58	0.04	0.01
100 ->	59	-0.04	0.03
100 ->	60	-0.05	0.06
100 ->	61	-0.58	0.85
100 ->	62	0.00	0.00
100 ->	63	0.02	0.01
100 ->	64	0.02	0.01
100 ->	65	0.00	0.00
100 ->	66	0.01	0.00
100 ->	67	0.00	0.00
100 ->	68	-0.02	0.01
100 ->	69	-0.03	0.02
100 ->	70	-0.01	0.01
100 ->	71	-0.01	0.02
100 ->	72	-0.13	0.07
100 ->	73	-0.55	0.50
100 ->	74	0.04	0.04
100 ->	75	0.01	0.01
100 ->	76	-0.34	0.50
100 ->	77	-0.06	0.03
100 ->	78	-0.02	0.01
100 ->	79	-0.00	0.00
100 ->	80	-0.00	0.00
100 ->	81	-0.02	0.00
100 ->	82	-0.00	0.00
100 ->	83	0.00	0.00
100 ->	84	0.01	0.00
100 ->	85	0.00	0.00
100 ->	86	0.01	0.00
100 ->	87	0.01	0.00
100 ->	88	0.01	0.00
100 ->	89	-0.00	0.01
100 ->	90	-0.08	0.03
100 ->	91	-0.97	0.47
100 ->	92	-11.69	0.73
100 ->	93	-0.83	0.44
100 ->	94	0.02	0.05
100 ->	95	-0.01	0.05
100 ->	96	-0.00	0.00
100 ->	97	-0.02	0.01
100 ->	98	-0.34	0.07
100 ->	99	-33.85	1.22
100 ->	100	-211.98	4.51
100 ->	101	-32.16	1.12
100 ->	102	-0.11	0.04

100 ->	103	-0.00	0.01
100 ->	104	0.01	0.00
100 ->	105	0.01	0.00
100 ->	106	0.00	0.00
101 ->	1	-0.05	0.01
101 ->	2	-0.00	0.00
101 ->	3	-0.00	0.00
101 ->	4	-0.00	0.00
101 ->	5	0.01	0.00
101 ->	6	-0.00	0.00
101 ->	7	0.00	0.00
101 ->	8	0.00	0.00
101 ->	9	0.00	0.00
101 ->	10	0.01	0.00
101 ->	11	0.01	0.00
101 ->	12	-0.02	0.01
101 ->	13	-0.01	0.00
101 ->	14	-0.01	0.00
101 ->	15	-0.00	0.00
101 ->	16	0.02	0.00
101 ->	17	-0.00	0.00
101 ->	18	-0.01	0.00
101 ->	19	-0.03	0.01
101 ->	20	-0.00	0.00
101 ->	21	-0.00	0.00
101 ->	22	-0.17	0.09
101 ->	23	-0.01	0.00
101 ->	24	-0.00	0.00
101 ->	25	-0.01	0.00
101 ->	26	-0.00	0.00
101 ->	27	-0.00	0.00
101 ->	28	-0.00	0.00
101 ->	29	-0.00	0.00
101 ->	30	0.00	0.00
101 ->	31	-0.01	0.00
101 ->	32	-0.00	0.00
101 ->	33	-0.00	0.00
101 ->	34	-0.00	0.00
101 ->	35	0.01	0.00
101 ->	36	-0.01	0.00
101 ->	37	-0.00	0.00
101 ->	38	0.01	0.00
101 ->	39	0.00	0.00
101 ->	40	-0.01	0.00
101 ->	41	-0.00	0.00
101 ->	42	-0.00	0.00
101 ->	43	-0.00	0.00
101 ->	44	0.30	0.32
101 ->	45	-0.01	0.00
101 ->	46	-1.03	0.73

101 ->	47	-0.28	0.20
101 ->	48	-0.01	0.00
101 ->	49	-0.02	0.01
101 ->	50	-0.05	0.03
101 ->	51	-0.00	0.00
101 ->	52	-0.00	0.00
101 ->	53	-0.11	0.05
101 ->	54	-0.00	0.00
101 ->	55	-0.00	0.00
101 ->	56	-0.00	0.00
101 ->	57	-0.00	0.00
101 ->	58	0.02	0.00
101 ->	59	-0.03	0.01
101 ->	60	-0.02	0.01
101 ->	61	-0.23	0.25
101 ->	62	0.00	0.01
101 ->	63	0.04	0.01
101 ->	64	0.03	0.01
101 ->	65	-0.00	0.00
101 ->	66	0.02	0.01
101 ->	67	-0.00	0.00
101 ->	68	-0.02	0.01
101 ->	69	-0.04	0.02
101 ->	70	-0.02	0.02
101 ->	71	-0.03	0.02
101 ->	72	-0.41	0.24
101 ->	73	-3.46	3.00
101 ->	74	-0.08	0.19
101 ->	75	-0.00	0.03
101 ->	76	-2.43	3.99
101 ->	77	-0.96	0.54
101 ->	78	-0.05	0.01
101 ->	79	-0.01	0.01
101 ->	80	-0.01	0.01
101 ->	81	-0.05	0.02
101 ->	82	-0.00	0.00
101 ->	83	-0.00	0.00
101 ->	84	0.01	0.00
101 ->	85	0.00	0.00
101 ->	86	0.02	0.00
101 ->	87	0.00	0.00
101 ->	88	-0.00	0.00
101 ->	89	-0.06	0.03
101 ->	90	-1.32	0.51
101 ->	91	-11.59	0.94
101 ->	92	-3.93	0.72
101 ->	93	-0.22	0.09
101 ->	94	-0.01	0.02
101 ->	95	0.01	0.01
101 ->	96	0.00	0.00

101	->	97	0.00	0.00
101	->	98	-0.04	0.01
101	->	99	-0.23	0.06
101	->	100	-31.93	1.08
101	->	101	-210.34	5.55
101	->	102	-31.98	0.79
101	->	103	-0.20	0.05
101	->	104	0.00	0.00
101	->	105	0.01	0.00
101	->	106	0.00	0.00
102	->	1	-0.07	0.01
102	->	2	-0.00	0.00
102	->	3	-0.00	0.00
102	->	4	-0.00	0.00
102	->	5	0.01	0.00
102	->	6	-0.00	0.00
102	->	7	-0.00	0.00
102	->	8	0.00	0.00
102	->	9	0.00	0.00
102	->	10	0.01	0.00
102	->	11	0.01	0.00
102	->	12	-0.05	0.02
102	->	13	-0.01	0.00
102	->	14	-0.01	0.00
102	->	15	-0.01	0.00
102	->	16	0.02	0.00
102	->	17	-0.00	0.00
102	->	18	-0.00	0.00
102	->	19	-0.02	0.01
102	->	20	-0.00	0.00
102	->	21	-0.00	0.00
102	->	22	-0.09	0.03
102	->	23	-0.01	0.00
102	->	24	-0.00	0.00
102	->	25	-0.01	0.00
102	->	26	-0.00	0.00
102	->	27	-0.00	0.00
102	->	28	-0.00	0.00
102	->	29	-0.00	0.00
102	->	30	0.00	0.00
102	->	31	-0.01	0.00
102	->	32	-0.00	0.00
102	->	33	-0.00	0.00
102	->	34	0.00	0.00
102	->	35	0.01	0.00
102	->	36	-0.00	0.00
102	->	37	-0.00	0.00
102	->	38	0.01	0.00
102	->	39	0.00	0.00
102	->	40	-0.01	0.00

102	->	41	0.00	0.00
102	->	42	-0.00	0.00
102	->	43	-0.00	0.00
102	->	44	-0.10	0.07
102	->	45	-0.00	0.00
102	->	46	-0.04	0.05
102	->	47	-0.09	0.07
102	->	48	-0.00	0.00
102	->	49	-0.00	0.00
102	->	50	-0.03	0.02
102	->	51	-0.00	0.00
102	->	52	-0.00	0.00
102	->	53	-0.03	0.01
102	->	54	-0.00	0.00
102	->	55	-0.00	0.00
102	->	56	-0.00	0.00
102	->	57	-0.00	0.00
102	->	58	0.01	0.00
102	->	59	-0.02	0.00
102	->	60	-0.00	0.00
102	->	61	-0.02	0.01
102	->	62	0.00	0.00
102	->	63	0.03	0.01
102	->	64	0.02	0.01
102	->	65	-0.00	0.00
102	->	66	0.02	0.00
102	->	67	-0.00	0.00
102	->	68	-0.02	0.01
102	->	69	-0.02	0.00
102	->	70	-0.01	0.00
102	->	71	-0.01	0.00
102	->	72	-0.27	0.15
102	->	73	-0.47	0.30
102	->	74	-1.84	0.81
102	->	75	-0.02	0.03
102	->	76	-0.90	1.56
102	->	77	-7.60	3.41
102	->	78	-0.10	0.04
102	->	79	-0.02	0.01
102	->	80	-0.03	0.03
102	->	81	-0.31	0.36
102	->	82	-0.00	0.00
102	->	83	-0.00	0.00
102	->	84	0.02	0.00
102	->	85	0.00	0.00
102	->	86	0.01	0.00
102	->	87	-0.02	0.01
102	->	88	-0.08	0.03
102	->	89	-0.54	0.23
102	->	90	-11.62	0.70

102	->	91	-3.32	0.70
102	->	92	-0.39	0.10
102	->	93	-0.07	0.06
102	->	94	0.02	0.01
102	->	95	0.01	0.00
102	->	96	0.00	0.00
102	->	97	0.00	0.00
102	->	98	-0.00	0.00
102	->	99	-0.01	0.01
102	->	100	-0.11	0.04
102	->	101	-31.75	0.78
102	->	102	-208.92	4.60
102	->	103	-33.04	1.03
102	->	104	-0.12	0.03
102	->	105	0.01	0.01
102	->	106	0.00	0.00
103	->	1	-0.06	0.02
103	->	2	-0.00	0.00
103	->	3	-0.00	0.00
103	->	4	-0.00	0.00
103	->	5	0.01	0.00
103	->	6	-0.00	0.00
103	->	7	0.00	0.00
103	->	8	0.00	0.00
103	->	9	0.00	0.00
103	->	10	0.01	0.00
103	->	11	0.02	0.01
103	->	12	-0.07	0.03
103	->	13	-0.01	0.00
103	->	14	-0.01	0.00
103	->	15	-0.00	0.00
103	->	16	0.02	0.00
103	->	17	-0.00	0.00
103	->	18	-0.00	0.00
103	->	19	-0.01	0.00
103	->	20	-0.00	0.00
103	->	21	-0.00	0.00
103	->	22	-0.04	0.01
103	->	23	-0.01	0.00
103	->	24	-0.00	0.00
103	->	25	-0.01	0.00
103	->	26	-0.00	0.00
103	->	27	-0.00	0.00
103	->	28	-0.00	0.00
103	->	29	-0.00	0.00
103	->	30	0.00	0.00
103	->	31	-0.01	0.00
103	->	32	-0.00	0.00
103	->	33	-0.00	0.00
103	->	34	0.00	0.00

103	->	35	0.01	0.00
103	->	36	-0.00	0.00
103	->	37	-0.00	0.00
103	->	38	0.01	0.00
103	->	39	0.00	0.00
103	->	40	-0.01	0.00
103	->	41	-0.00	0.00
103	->	42	-0.00	0.00
103	->	43	-0.00	0.00
103	->	44	-0.07	0.03
103	->	45	-0.00	0.00
103	->	46	0.01	0.01
103	->	47	-0.09	0.05
103	->	48	-0.00	0.00
103	->	49	-0.00	0.00
103	->	50	-0.02	0.01
103	->	51	-0.01	0.00
103	->	52	-0.00	0.00
103	->	53	-0.02	0.00
103	->	54	-0.00	0.00
103	->	55	-0.00	0.00
103	->	56	-0.00	0.00
103	->	57	-0.00	0.00
103	->	58	0.01	0.00
103	->	59	-0.02	0.00
103	->	60	-0.00	0.00
103	->	61	-0.01	0.00
103	->	62	0.00	0.00
103	->	63	0.02	0.00
103	->	64	0.01	0.00
103	->	65	0.00	0.00
103	->	66	0.02	0.00
103	->	67	-0.00	0.00
103	->	68	-0.02	0.01
103	->	69	-0.01	0.00
103	->	70	-0.01	0.00
103	->	71	0.00	0.00
103	->	72	-0.04	0.78
103	->	73	-0.07	0.03
103	->	74	-3.44	1.37
103	->	75	-0.02	0.05
103	->	76	-0.11	0.04
103	->	77	-3.83	2.58
103	->	78	-0.25	0.23
103	->	79	-0.01	0.01
103	->	80	-0.02	0.01
103	->	81	-1.91	2.70
103	->	82	-0.01	0.00
103	->	83	-0.00	0.00
103	->	84	0.02	0.00

103	->	85	-0.00	0.00
103	->	86	-0.01	0.01
103	->	87	-0.11	0.05
103	->	88	-1.66	0.59
103	->	89	-6.08	0.55
103	->	90	-3.76	0.59
103	->	91	-0.33	0.08
103	->	92	-0.05	0.02
103	->	93	0.01	0.00
103	->	94	0.02	0.00
103	->	95	0.01	0.00
103	->	96	0.00	0.00
103	->	97	0.00	0.00
103	->	98	0.00	0.00
103	->	99	0.01	0.00
103	->	100	-0.00	0.01
103	->	101	-0.20	0.05
103	->	102	-32.84	1.01
103	->	103	-128.73	3.90
103	->	104	-34.11	1.01
103	->	105	-0.28	0.05
103	->	106	-0.02	0.01
104	->	1	-0.06	0.02
104	->	2	-0.00	0.00
104	->	3	0.00	0.00
104	->	4	-0.00	0.00
104	->	5	0.01	0.00
104	->	6	-0.00	0.00
104	->	7	-0.00	0.00
104	->	8	0.01	0.00
104	->	9	0.00	0.00
104	->	10	0.02	0.00
104	->	11	0.03	0.01
104	->	12	-0.12	0.07
104	->	13	-0.01	0.00
104	->	14	-0.00	0.00
104	->	15	-0.00	0.00
104	->	16	0.01	0.00
104	->	17	0.00	0.00
104	->	18	0.00	0.00
104	->	19	-0.00	0.00
104	->	20	-0.00	0.00
104	->	21	-0.00	0.00
104	->	22	-0.03	0.00
104	->	23	-0.01	0.00
104	->	24	-0.00	0.00
104	->	25	-0.01	0.00
104	->	26	-0.00	0.00
104	->	27	-0.00	0.00
104	->	28	-0.00	0.00

104 ->	29	-0.00	0.00
104 ->	30	0.00	0.00
104 ->	31	-0.01	0.00
104 ->	32	-0.00	0.00
104 ->	33	-0.00	0.00
104 ->	34	-0.00	0.00
104 ->	35	0.01	0.00
104 ->	36	-0.00	0.00
104 ->	37	-0.00	0.00
104 ->	38	0.01	0.00
104 ->	39	0.00	0.00
104 ->	40	-0.02	0.00
104 ->	41	0.00	0.00
104 ->	42	-0.00	0.00
104 ->	43	-0.00	0.00
104 ->	44	-0.04	0.01
104 ->	45	-0.00	0.00
104 ->	46	0.02	0.00
104 ->	47	-0.02	0.00
104 ->	48	-0.00	0.00
104 ->	49	-0.00	0.00
104 ->	50	-0.01	0.00
104 ->	51	-0.00	0.00
104 ->	52	-0.00	0.00
104 ->	53	-0.01	0.00
104 ->	54	-0.00	0.00
104 ->	55	-0.00	0.00
104 ->	56	-0.00	0.00
104 ->	57	-0.00	0.00
104 ->	58	0.01	0.00
104 ->	59	-0.01	0.00
104 ->	60	-0.00	0.00
104 ->	61	-0.00	0.00
104 ->	62	0.00	0.00
104 ->	63	0.01	0.00
104 ->	64	0.01	0.00
104 ->	65	0.00	0.00
104 ->	66	0.01	0.00
104 ->	67	-0.00	0.00
104 ->	68	-0.01	0.01
104 ->	69	-0.01	0.00
104 ->	70	-0.00	0.00
104 ->	71	-0.00	0.00
104 ->	72	0.38	0.29
104 ->	73	-0.01	0.00
104 ->	74	-0.55	0.27
104 ->	75	-0.09	0.18
104 ->	76	-0.02	0.00
104 ->	77	-0.07	0.02
104 ->	78	-0.65	0.84

104	->	79	-0.01	0.00
104	->	80	-0.00	0.00
104	->	81	-0.19	0.20
104	->	82	-0.01	0.01
104	->	83	-0.00	0.00
104	->	84	0.01	0.00
104	->	85	-0.03	0.01
104	->	86	-0.10	0.05
104	->	87	-0.52	0.29
104	->	88	-11.34	0.67
104	->	89	-0.64	0.26
104	->	90	-0.29	0.09
104	->	91	-0.03	0.02
104	->	92	0.01	0.00
104	->	93	0.02	0.00
104	->	94	0.01	0.00
104	->	95	0.00	0.00
104	->	96	0.00	0.00
104	->	97	0.00	0.00
104	->	98	0.01	0.00
104	->	99	0.01	0.00
104	->	100	0.01	0.00
104	->	101	0.00	0.00
104	->	102	-0.12	0.03
104	->	103	-33.85	1.00
104	->	104	-219.04	2.29
104	->	105	-33.78	0.85
104	->	106	-0.18	0.04
105	->	1	-0.04	0.01
105	->	2	-0.00	0.00
105	->	3	0.00	0.00
105	->	4	-0.00	0.00
105	->	5	0.01	0.00
105	->	6	-0.00	0.00
105	->	7	-0.00	0.00
105	->	8	0.01	0.00
105	->	9	0.00	0.00
105	->	10	0.02	0.01
105	->	11	0.03	0.02
105	->	12	-0.10	0.10
105	->	13	-0.01	0.00
105	->	14	-0.00	0.00
105	->	15	-0.00	0.00
105	->	16	0.01	0.00
105	->	17	0.00	0.00
105	->	18	0.00	0.00
105	->	19	-0.00	0.00
105	->	20	0.00	0.00
105	->	21	0.00	0.00
105	->	22	-0.02	0.00

105 ->	23	-0.00	0.00
105 ->	24	-0.00	0.00
105 ->	25	-0.01	0.00
105 ->	26	-0.00	0.00
105 ->	27	-0.00	0.00
105 ->	28	-0.00	0.00
105 ->	29	-0.00	0.00
105 ->	30	0.00	0.00
105 ->	31	-0.01	0.00
105 ->	32	-0.00	0.00
105 ->	33	-0.00	0.00
105 ->	34	-0.00	0.00
105 ->	35	0.00	0.00
105 ->	36	-0.00	0.00
105 ->	37	-0.00	0.00
105 ->	38	0.00	0.00
105 ->	39	0.00	0.00
105 ->	40	-0.01	0.00
105 ->	41	-0.00	0.00
105 ->	42	-0.00	0.00
105 ->	43	-0.00	0.00
105 ->	44	-0.02	0.00
105 ->	45	-0.00	0.00
105 ->	46	0.01	0.00
105 ->	47	-0.01	0.00
105 ->	48	-0.00	0.00
105 ->	49	-0.00	0.00
105 ->	50	-0.00	0.00
105 ->	51	-0.00	0.00
105 ->	52	-0.00	0.00
105 ->	53	-0.01	0.00
105 ->	54	-0.00	0.00
105 ->	55	-0.00	0.00
105 ->	56	-0.00	0.00
105 ->	57	-0.00	0.00
105 ->	58	0.00	0.00
105 ->	59	-0.01	0.00
105 ->	60	-0.00	0.00
105 ->	61	-0.00	0.00
105 ->	62	0.00	0.00
105 ->	63	0.01	0.00
105 ->	64	0.01	0.00
105 ->	65	0.00	0.00
105 ->	66	0.01	0.00
105 ->	67	-0.00	0.00
105 ->	68	-0.02	0.01
105 ->	69	-0.01	0.00
105 ->	70	-0.00	0.00
105 ->	71	-0.00	0.00
105 ->	72	0.12	0.15

105	->	73	-0.00	0.00
105	->	74	0.02	0.04
105	->	75	0.06	0.13
105	->	76	-0.02	0.00
105	->	77	-0.04	0.01
105	->	78	-2.62	2.45
105	->	79	-0.01	0.00
105	->	80	-0.00	0.00
105	->	81	-0.06	0.02
105	->	82	-0.01	0.01
105	->	83	-0.00	0.00
105	->	84	0.01	0.00
105	->	85	-0.19	0.08
105	->	86	-0.91	0.63
105	->	87	-11.32	0.83
105	->	88	-4.36	0.89
105	->	89	-0.22	0.07
105	->	90	-0.03	0.02
105	->	91	0.01	0.00
105	->	92	0.02	0.00
105	->	93	0.01	0.00
105	->	94	0.01	0.00
105	->	95	0.00	0.00
105	->	96	0.00	0.00
105	->	97	0.00	0.00
105	->	98	0.00	0.00
105	->	99	0.00	0.00
105	->	100	0.01	0.00
105	->	101	0.01	0.00
105	->	102	0.01	0.01
105	->	103	-0.28	0.05
105	->	104	-33.48	0.84
105	->	105	-182.37	2.49
105	->	106	-36.09	1.02
106	->	1	-0.01	0.00
106	->	2	0.00	0.00
106	->	3	0.00	0.00
106	->	4	-0.00	0.00
106	->	5	0.01	0.00
106	->	6	-0.00	0.00
106	->	7	0.00	0.00
106	->	8	0.00	0.00
106	->	9	0.00	0.00
106	->	10	0.01	0.00
106	->	11	0.01	0.00
106	->	12	-0.02	0.01
106	->	13	-0.00	0.00
106	->	14	-0.00	0.00
106	->	15	-0.00	0.00
106	->	16	0.00	0.00

106	->	17	0.00	0.00
106	->	18	0.00	0.00
106	->	19	0.00	0.00
106	->	20	0.00	0.00
106	->	21	0.00	0.00
106	->	22	-0.01	0.00
106	->	23	-0.00	0.00
106	->	24	0.00	0.00
106	->	25	-0.00	0.00
106	->	26	-0.00	0.00
106	->	27	-0.00	0.00
106	->	28	-0.00	0.00
106	->	29	-0.00	0.00
106	->	30	0.00	0.00
106	->	31	-0.01	0.00
106	->	32	-0.00	0.00
106	->	33	0.00	0.00
106	->	34	-0.00	0.00
106	->	35	0.01	0.00
106	->	36	-0.00	0.00
106	->	37	-0.00	0.00
106	->	38	0.01	0.00
106	->	39	-0.00	0.00
106	->	40	-0.02	0.00
106	->	41	0.00	0.00
106	->	42	-0.00	0.00
106	->	43	-0.00	0.00
106	->	44	-0.01	0.00
106	->	45	0.00	0.00
106	->	46	0.01	0.00
106	->	47	-0.00	0.00
106	->	48	-0.00	0.00
106	->	49	-0.00	0.00
106	->	50	-0.00	0.00
106	->	51	-0.00	0.00
106	->	52	-0.00	0.00
106	->	53	-0.01	0.00
106	->	54	-0.00	0.00
106	->	55	-0.00	0.00
106	->	56	-0.00	0.00
106	->	57	-0.00	0.00
106	->	58	0.00	0.00
106	->	59	-0.01	0.00
106	->	60	-0.00	0.00
106	->	61	-0.00	0.00
106	->	62	0.00	0.00
106	->	63	0.00	0.00
106	->	64	0.00	0.00
106	->	65	0.00	0.00
106	->	66	0.00	0.00

106	->	67	-0.00	0.00
106	->	68	-0.01	0.00
106	->	69	-0.00	0.00
106	->	70	-0.00	0.00
106	->	71	-0.00	0.00
106	->	72	-0.05	0.01
106	->	73	-0.00	0.00
106	->	74	0.01	0.00
106	->	75	0.00	0.01
106	->	76	-0.01	0.00
106	->	77	-0.01	0.00
106	->	78	-0.04	0.30
106	->	79	-0.00	0.00
106	->	80	-0.00	0.00
106	->	81	-0.01	0.00
106	->	82	-0.00	0.00
106	->	83	-0.00	0.00
106	->	84	0.00	0.00
106	->	85	-2.12	1.27
106	->	86	-10.53	0.58
106	->	87	0.10	0.16
106	->	88	-0.26	0.08
106	->	89	-0.02	0.02
106	->	90	0.01	0.00
106	->	91	0.01	0.00
106	->	92	0.01	0.00
106	->	93	0.00	0.00
106	->	94	0.00	0.00
106	->	95	0.00	0.00
106	->	96	0.00	0.00
106	->	97	0.00	0.00
106	->	98	0.00	0.00
106	->	99	0.00	0.00
106	->	100	0.00	0.00
106	->	101	0.00	0.00
106	->	102	0.00	0.00
106	->	103	-0.02	0.01
106	->	104	-0.18	0.04
106	->	105	-35.84	1.01
106	->	106	-216.63	3.15
