# Supplementary material for: Investigating dynamic and energetic determinants of protein nucleic acid recognition: analysis of the zinc finger zif268-DNA complexes
Source: BMC Struct Biol. 2010 Nov 24;10:42. doi: 10.1186/1472-6807-10-42 (PMC3002361; doi:10.1186/1472-6807-10-42)
Supplement: Additional file 3 — Average pair interaction energies for 1A1G Complex and their standard deviations. [file 1472-6807-10-42-S3.PDF]

=>> 1A1G

Residue ->	Residue	TGBTOT	SD
1 ->	1	-253.98	8.02
1 ->	2	-1.54	1.08
1 ->	3	-1.80	1.49
1 ->	4	-0.12	0.05
1 ->	5	-0.10	0.02
1 ->	6	-0.00	0.00
1 ->	7	-0.00	0.00
1 ->	8	-0.04	0.01
1 ->	9	-0.00	0.00
1 ->	10	-0.07	0.02
1 ->	11	-0.13	0.04
1 ->	12	0.22	0.51
1 ->	13	-2.03	1.77
1 ->	14	-1.94	1.34
1 ->	15	-3.73	2.25
1 ->	16	-0.66	0.89
1 ->	17	-0.08	0.11
1 ->	18	0.00	0.01
1 ->	19	0.12	0.08
1 ->	20	-0.04	0.02
1 ->	21	0.00	0.00
1 ->	22	0.14	0.07
1 ->	23	0.00	0.00
1 ->	24	0.00	0.00
1 ->	25	0.04	0.01
1 ->	26	0.00	0.00
1 ->	27	-0.01	0.00
1 ->	28	0.00	0.00
1 ->	29	0.00	0.00
1 ->	30	-0.00	0.00
1 ->	31	0.02	0.00
1 ->	32	0.00	0.00
1 ->	33	0.00	0.00
1 ->	34	0.00	0.00
1 ->	35	-0.01	0.00
1 ->	36	0.00	0.00
1 ->	37	-0.00	0.00
1 ->	38	-0.00	0.00
1 ->	39	-0.00	0.00
1 ->	40	0.01	0.00
1 ->	41	0.00	0.00
1 ->	42	0.00	0.00
1 ->	43	-0.00	0.00
1 ->	44	0.05	0.01
1 ->	45	0.00	0.00
1 ->	46	-0.03	0.01

1 ->	47	0.00	0.00
1 ->	48	0.00	0.00
1 ->	49	0.00	0.00
1 ->	50	0.00	0.00
1 ->	51	0.00	0.00
1 ->	52	0.00	0.00
1 ->	53	0.01	0.00
1 ->	54	0.00	0.00
1 ->	55	0.00	0.00
1 ->	56	0.00	0.00
1 ->	57	0.00	0.00
1 ->	58	-0.01	0.00
1 ->	59	0.01	0.00
1 ->	60	0.00	0.00
1 ->	61	0.00	0.00
1 ->	62	-0.00	0.00
1 ->	63	-0.00	0.00
1 ->	64	-0.00	0.00
1 ->	65	-0.00	0.00
1 ->	66	-0.00	0.00
1 ->	67	0.00	0.00
1 ->	68	0.00	0.00
1 ->	69	0.00	0.00
1 ->	70	0.00	0.00
1 ->	71	-0.00	0.00
1 ->	72	0.02	0.01
1 ->	73	0.00	0.00
1 ->	74	-0.02	0.01
1 ->	75	-0.01	0.00
1 ->	76	0.01	0.00
1 ->	77	0.02	0.01
1 ->	78	0.01	0.00
1 ->	79	0.00	0.00
1 ->	80	0.00	0.00
1 ->	81	0.01	0.01
1 ->	82	0.00	0.00
1 ->	83	0.00	0.00
1 ->	84	-0.00	0.00
1 ->	85	-0.00	0.00
1 ->	86	-0.01	0.00
1 ->	87	-0.01	0.00
1 ->	88	-0.01	0.00
1 ->	89	-0.02	0.01
1 ->	90	-0.04	0.01
1 ->	91	-0.57	0.69
1 ->	92	-4.38	4.60
1 ->	93	-4.60	4.28
1 ->	94	-0.12	0.04
1 ->	95	-0.04	0.01
1 ->	96	-0.01	0.01

1	->	97	-0.03	0.01
1	->	98	-0.02	0.00
1	->	99	-0.02	0.00
1	->	100	-0.03	0.01
1	->	101	-0.04	0.01
1	->	102	-0.05	0.02
1	->	103	-0.04	0.02
1	->	104	-0.03	0.01
1	->	105	-0.02	0.01
1	->	106	-0.01	0.00
2	->	1	-1.53	1.09
2	->	2	1.86	0.96
2	->	3	-12.40	0.87
2	->	4	-0.30	0.08
2	->	5	-0.02	0.01
2	->	6	-0.00	0.00
2	->	7	-0.00	0.00
2	->	8	0.00	0.00
2	->	9	-0.00	0.00
2	->	10	-0.00	0.00
2	->	11	-0.01	0.01
2	->	12	-0.01	0.01
2	->	13	-1.73	1.06
2	->	14	-1.26	0.27
2	->	15	-1.06	0.30
2	->	16	-0.21	0.16
2	->	17	-0.04	0.03
2	->	18	-0.01	0.00
2	->	19	0.01	0.02
2	->	20	-0.04	0.01
2	->	21	-0.00	0.00
2	->	22	0.00	0.00
2	->	23	-0.00	0.00
2	->	24	-0.00	0.00
2	->	25	-0.00	0.00
2	->	26	-0.00	0.00
2	->	27	-0.00	0.00
2	->	28	-0.00	0.00
2	->	29	0.00	0.00
2	->	30	-0.00	0.00
2	->	31	0.00	0.00
2	->	32	-0.00	0.00
2	->	33	0.00	0.00
2	->	34	0.00	0.00
2	->	35	-0.00	0.00
2	->	36	0.00	0.00
2	->	37	0.00	0.00
2	->	38	-0.00	0.00
2	->	39	0.00	0.00
2	->	40	0.00	0.00

2 ->	41	0.00	0.00
2 ->	42	0.00	0.00
2 ->	43	0.00	0.00
2 ->	44	0.00	0.00
2 ->	45	0.00	0.00
2 ->	46	-0.00	0.00
2 ->	47	0.00	0.00
2 ->	48	0.00	0.00
2 ->	49	0.00	0.00
2 ->	50	0.00	0.00
2 ->	51	0.00	0.00
2 ->	52	0.00	0.00
2 ->	53	0.00	0.00
2 ->	54	0.00	0.00
2 ->	55	0.00	0.00
2 ->	56	0.00	0.00
2 ->	57	0.00	0.00
2 ->	58	-0.00	0.00
2 ->	59	0.00	0.00
2 ->	60	0.00	0.00
2 ->	61	0.00	0.00
2 ->	62	0.00	0.00
2 ->	63	-0.00	0.00
2 ->	64	-0.00	0.00
2 ->	65	0.00	0.00
2 ->	66	-0.00	0.00
2 ->	67	0.00	0.00
2 ->	68	0.00	0.00
2 ->	69	0.00	0.00
2 ->	70	0.00	0.00
2 ->	71	0.00	0.00
2 ->	72	0.00	0.00
2 ->	73	0.00	0.00
2 ->	74	-0.00	0.00
2 ->	75	-0.00	0.00
2 ->	76	0.00	0.00
2 ->	77	0.00	0.00
2 ->	78	0.00	0.00
2 ->	79	0.00	0.00
2 ->	80	0.00	0.00
2 ->	81	0.00	0.00
2 ->	82	0.00	0.00
2 ->	83	0.00	0.00
2 ->	84	-0.00	0.00
2 ->	85	-0.00	0.00
2 ->	86	0.00	0.00
2 ->	87	-0.00	0.00
2 ->	88	-0.00	0.00
2 ->	89	-0.00	0.00
2 ->	90	-0.00	0.00

2	->	91	-0.01	0.01
2	->	92	-0.05	0.07
2	->	93	-0.02	0.01
2	->	94	-0.01	0.00
2	->	95	-0.00	0.00
2	->	96	-0.00	0.00
2	->	97	-0.00	0.00
2	->	98	-0.00	0.00
2	->	99	-0.00	0.00
2	->	100	-0.00	0.00
2	->	101	-0.00	0.00
2	->	102	-0.00	0.00
2	->	103	-0.00	0.00
2	->	104	-0.00	0.00
2	->	105	-0.00	0.00
2	->	106	-0.00	0.00
3	->	1	-1.79	1.46
3	->	2	-12.50	0.87
3	->	3	19.31	1.31
3	->	4	-10.02	0.60
3	->	5	-0.50	0.07
3	->	6	-0.03	0.01
3	->	7	0.01	0.01
3	->	8	-0.01	0.00
3	->	9	-0.00	0.00
3	->	10	-0.02	0.01
3	->	11	-0.01	0.01
3	->	12	-0.20	0.04
3	->	13	-3.26	0.62
3	->	14	-3.78	0.50
3	->	15	-1.01	0.47
3	->	16	-1.46	0.64
3	->	17	-1.40	0.75
3	->	18	-0.04	0.02
3	->	19	-0.09	0.07
3	->	20	-1.56	0.49
3	->	21	-0.03	0.01
3	->	22	-0.01	0.02
3	->	23	-0.02	0.01
3	->	24	-0.02	0.01
3	->	25	-0.00	0.00
3	->	26	-0.00	0.00
3	->	27	-0.01	0.00
3	->	28	-0.00	0.00
3	->	29	0.00	0.00
3	->	30	-0.00	0.00
3	->	31	-0.00	0.00
3	->	32	-0.00	0.00
3	->	33	-0.00	0.00
3	->	34	0.00	0.00

3	->	35	0.00	0.00
3	->	36	0.00	0.00
3	->	37	0.00	0.00
3	->	38	0.00	0.00
3	->	39	0.00	0.00
3	->	40	-0.00	0.00
3	->	41	0.00	0.00
3	->	42	-0.00	0.00
3	->	43	-0.00	0.00
3	->	44	-0.00	0.00
3	->	45	-0.00	0.00
3	->	46	0.00	0.00
3	->	47	-0.00	0.00
3	->	48	0.00	0.00
3	->	49	-0.00	0.00
3	->	50	-0.00	0.00
3	->	51	-0.00	0.00
3	->	52	0.00	0.00
3	->	53	-0.00	0.00
3	->	54	0.00	0.00
3	->	55	0.00	0.00
3	->	56	0.00	0.00
3	->	57	0.00	0.00
3	->	58	0.00	0.00
3	->	59	-0.00	0.00
3	->	60	0.00	0.00
3	->	61	0.00	0.00
3	->	62	0.00	0.00
3	->	63	0.00	0.00
3	->	64	0.00	0.00
3	->	65	0.00	0.00
3	->	66	0.00	0.00
3	->	67	0.00	0.00
3	->	68	-0.00	0.00
3	->	69	-0.00	0.00
3	->	70	0.00	0.00
3	->	71	0.00	0.00
3	->	72	-0.00	0.00
3	->	73	0.00	0.00
3	->	74	0.00	0.00
3	->	75	0.00	0.00
3	->	76	-0.00	0.00
3	->	77	-0.00	0.00
3	->	78	-0.00	0.00
3	->	79	0.00	0.00
3	->	80	0.00	0.00
3	->	81	-0.00	0.00
3	->	82	-0.00	0.00
3	->	83	0.00	0.00
3	->	84	0.00	0.00

3	->	85	0.00	0.00
3	->	86	0.00	0.00
3	->	87	0.00	0.00
3	->	88	0.00	0.00
3	->	89	-0.00	0.00
3	->	90	-0.00	0.00
3	->	91	-0.00	0.01
3	->	92	0.00	0.02
3	->	93	-0.01	0.00
3	->	94	-0.01	0.00
3	->	95	-0.01	0.00
3	->	96	-0.02	0.01
3	->	97	-0.01	0.01
3	->	98	-0.00	0.00
3	->	99	-0.00	0.00
3	->	100	-0.00	0.00
3	->	101	-0.00	0.00
3	->	102	-0.00	0.00
3	->	103	0.00	0.00
3	->	104	0.00	0.00
3	->	105	0.00	0.00
3	->	106	0.00	0.00
4	->	1	-0.12	0.05
4	->	2	-0.31	0.08
4	->	3	-9.99	0.59
4	->	4	22.47	1.13
4	->	5	-12.58	0.40
4	->	6	-0.37	0.14
4	->	7	0.06	0.06
4	->	8	-0.06	0.03
4	->	9	-0.01	0.01
4	->	10	-0.13	0.06
4	->	11	-0.04	0.03
4	->	12	-0.16	0.11
4	->	13	-2.10	0.40
4	->	14	-0.68	0.30
4	->	15	0.00	0.01
4	->	16	-0.00	0.01
4	->	17	-0.01	0.00
4	->	18	-0.00	0.00
4	->	19	-0.00	0.02
4	->	20	-1.06	0.28
4	->	21	-0.01	0.01
4	->	22	0.01	0.01
4	->	23	-0.08	0.03
4	->	24	-0.06	0.03
4	->	25	-0.01	0.00
4	->	26	-0.00	0.00
4	->	27	-0.07	0.03
4	->	28	-0.00	0.00

4 ->	29	0.00	0.00
4 ->	30	-0.00	0.00
4 ->	31	0.00	0.00
4 ->	32	-0.00	0.00
4 ->	33	-0.00	0.00
4 ->	34	0.00	0.00
4 ->	35	0.00	0.00
4 ->	36	-0.00	0.00
4 ->	37	0.00	0.00
4 ->	38	0.00	0.00
4 ->	39	0.00	0.00
4 ->	40	0.00	0.00
4 ->	41	0.00	0.00
4 ->	42	0.00	0.00
4 ->	43	0.00	0.00
4 ->	44	-0.00	0.00
4 ->	45	-0.00	0.00
4 ->	46	0.00	0.00
4 ->	47	0.00	0.00
4 ->	48	0.00	0.00
4 ->	49	0.00	0.00
4 ->	50	-0.00	0.00
4 ->	51	-0.00	0.00
4 ->	52	0.00	0.00
4 ->	53	-0.00	0.00
4 ->	54	0.00	0.00
4 ->	55	0.00	0.00
4 ->	56	0.00	0.00
4 ->	57	0.00	0.00
4 ->	58	0.00	0.00
4 ->	59	0.00	0.00
4 ->	60	0.00	0.00
4 ->	61	0.00	0.00
4 ->	62	0.00	0.00
4 ->	63	0.00	0.00
4 ->	64	0.00	0.00
4 ->	65	0.00	0.00
4 ->	66	-0.00	0.00
4 ->	67	0.00	0.00
4 ->	68	0.00	0.00
4 ->	69	-0.00	0.00
4 ->	70	0.00	0.00
4 ->	71	0.00	0.00
4 ->	72	0.00	0.00
4 ->	73	0.00	0.00
4 ->	74	-0.00	0.00
4 ->	75	0.00	0.00
4 ->	76	0.00	0.00
4 ->	77	0.00	0.00
4 ->	78	0.00	0.00

4	->	79	0.00	0.00
4	->	80	0.00	0.00
4	->	81	0.00	0.00
4	->	82	0.00	0.00
4	->	83	0.00	0.00
4	->	84	0.00	0.00
4	->	85	-0.00	0.00
4	->	86	-0.00	0.00
4	->	87	-0.00	0.00
4	->	88	-0.00	0.00
4	->	89	-0.00	0.00
4	->	90	-0.00	0.00
4	->	91	-0.02	0.01
4	->	92	-0.01	0.01
4	->	93	-0.00	0.00
4	->	94	-0.00	0.00
4	->	95	0.00	0.00
4	->	96	-0.00	0.00
4	->	97	0.00	0.00
4	->	98	-0.00	0.00
4	->	99	0.00	0.00
4	->	100	0.00	0.00
4	->	101	-0.00	0.00
4	->	102	-0.00	0.00
4	->	103	-0.00	0.00
4	->	104	-0.00	0.00
4	->	105	-0.00	0.00
4	->	106	-0.00	0.00
5	->	1	-0.10	0.02
5	->	2	-0.02	0.01
5	->	3	-0.50	0.07
5	->	4	-12.62	0.41
5	->	5	-28.13	3.21
5	->	6	-1.80	0.50
5	->	7	-6.50	2.32
5	->	8	0.15	0.18
5	->	9	-0.05	0.10
5	->	10	2.12	0.65
5	->	11	0.09	0.02
5	->	12	-0.21	0.11
5	->	13	-0.42	0.12
5	->	14	-0.55	0.22
5	->	15	0.01	0.01
5	->	16	0.04	0.01
5	->	17	-0.00	0.00
5	->	18	-0.00	0.00
5	->	19	-0.10	0.02
5	->	20	-0.96	0.25
5	->	21	-0.00	0.01
5	->	22	-0.21	0.06

5 ->	23	1.79	0.70
5 ->	24	-0.58	0.40
5 ->	25	-0.06	0.01
5 ->	26	-0.07	0.03
5 ->	27	2.72	1.05
5 ->	28	-0.02	0.01
5 ->	29	-0.00	0.00
5 ->	30	-0.00	0.00
5 ->	31	-0.02	0.00
5 ->	32	-0.00	0.00
5 ->	33	0.00	0.00
5 ->	34	-0.00	0.00
5 ->	35	0.00	0.00
5 ->	36	-0.00	0.00
5 ->	37	0.00	0.00
5 ->	38	0.00	0.00
5 ->	39	0.00	0.00
5 ->	40	-0.01	0.00
5 ->	41	-0.00	0.00
5 ->	42	-0.00	0.00
5 ->	43	-0.00	0.00
5 ->	44	-0.03	0.00
5 ->	45	-0.00	0.00
5 ->	46	0.01	0.00
5 ->	47	-0.00	0.00
5 ->	48	-0.00	0.00
5 ->	49	-0.00	0.00
5 ->	50	-0.00	0.00
5 ->	51	-0.00	0.00
5 ->	52	-0.00	0.00
5 ->	53	-0.00	0.00
5 ->	54	-0.00	0.00
5 ->	55	0.00	0.00
5 ->	56	-0.00	0.00
5 ->	57	-0.00	0.00
5 ->	58	0.00	0.00
5 ->	59	-0.00	0.00
5 ->	60	-0.00	0.00
5 ->	61	-0.00	0.00
5 ->	62	0.00	0.00
5 ->	63	0.00	0.00
5 ->	64	0.00	0.00
5 ->	65	0.00	0.00
5 ->	66	0.00	0.00
5 ->	67	0.00	0.00
5 ->	68	-0.00	0.00
5 ->	69	-0.00	0.00
5 ->	70	-0.00	0.00
5 ->	71	0.00	0.00
5 ->	72	-0.01	0.00

5	->	73	-0.00	0.00
5	->	74	0.01	0.00
5	->	75	0.00	0.00
5	->	76	-0.00	0.00
5	->	77	-0.01	0.00
5	->	78	-0.00	0.00
5	->	79	-0.00	0.00
5	->	80	-0.00	0.00
5	->	81	-0.00	0.00
5	->	82	-0.00	0.00
5	->	83	-0.00	0.00
5	->	84	0.00	0.00
5	->	85	0.00	0.00
5	->	86	0.00	0.00
5	->	87	0.00	0.00
5	->	88	0.01	0.00
5	->	89	0.02	0.00
5	->	90	0.05	0.01
5	->	91	0.15	0.06
5	->	92	0.07	0.02
5	->	93	0.02	0.00
5	->	94	0.01	0.00
5	->	95	0.01	0.00
5	->	96	0.00	0.00
5	->	97	0.01	0.01
5	->	98	0.01	0.00
5	->	99	0.01	0.00
5	->	100	0.01	0.00
5	->	101	0.01	0.00
5	->	102	0.01	0.00
5	->	103	0.01	0.00
5	->	104	0.01	0.00
5	->	105	0.01	0.00
5	->	106	0.01	0.00
6	->	1	-0.00	0.00
6	->	2	-0.00	0.00
6	->	3	-0.03	0.01
6	->	4	-0.38	0.15
6	->	5	-1.92	0.50
6	->	6	0.76	0.47
6	->	7	-13.15	0.41
6	->	8	-0.58	0.21
6	->	9	-0.04	0.02
6	->	10	-0.07	0.02
6	->	11	-0.01	0.00
6	->	12	-0.01	0.00
6	->	13	-0.02	0.01
6	->	14	-0.03	0.03
6	->	15	-0.00	0.00
6	->	16	-0.00	0.00

6 ->	17	-0.01	0.00
6 ->	18	-0.00	0.00
6 ->	19	-0.01	0.00
6 ->	20	-0.33	0.26
6 ->	21	-0.02	0.01
6 ->	22	-0.01	0.00
6 ->	23	-0.12	0.06
6 ->	24	-1.18	0.35
6 ->	25	-0.01	0.01
6 ->	26	-0.01	0.00
6 ->	27	-0.37	0.24
6 ->	28	-0.06	0.08
6 ->	29	-0.00	0.00
6 ->	30	-0.00	0.00
6 ->	31	0.00	0.00
6 ->	32	-0.00	0.00
6 ->	33	-0.00	0.00
6 ->	34	0.00	0.00
6 ->	35	-0.00	0.00
6 ->	36	0.00	0.00
6 ->	37	0.00	0.00
6 ->	38	-0.00	0.00
6 ->	39	0.00	0.00
6 ->	40	0.00	0.00
6 ->	41	0.00	0.00
6 ->	42	-0.00	0.00
6 ->	43	-0.00	0.00
6 ->	44	0.00	0.00
6 ->	45	0.00	0.00
6 ->	46	-0.00	0.00
6 ->	47	0.00	0.00
6 ->	48	0.00	0.00
6 ->	49	0.00	0.00
6 ->	50	0.00	0.00
6 ->	51	0.00	0.00
6 ->	52	0.00	0.00
6 ->	53	0.00	0.00
6 ->	54	0.00	0.00
6 ->	55	0.00	0.00
6 ->	56	0.00	0.00
6 ->	57	0.00	0.00
6 ->	58	-0.00	0.00
6 ->	59	0.00	0.00
6 ->	60	0.00	0.00
6 ->	61	0.00	0.00
6 ->	62	0.00	0.00
6 ->	63	-0.00	0.00
6 ->	64	-0.00	0.00
6 ->	65	0.00	0.00
6 ->	66	-0.00	0.00

6	->	67	0.00	0.00
6	->	68	0.00	0.00
6	->	69	0.00	0.00
6	->	70	0.00	0.00
6	->	71	0.00	0.00
6	->	72	0.00	0.00
6	->	73	0.00	0.00
6	->	74	-0.00	0.00
6	->	75	-0.00	0.00
6	->	76	0.00	0.00
6	->	77	0.00	0.00
6	->	78	0.00	0.00
6	->	79	0.00	0.00
6	->	80	0.00	0.00
6	->	81	0.00	0.00
6	->	82	0.00	0.00
6	->	83	0.00	0.00
6	->	84	-0.00	0.00
6	->	85	0.00	0.00
6	->	86	-0.00	0.00
6	->	87	-0.00	0.00
6	->	88	-0.00	0.00
6	->	89	-0.00	0.00
6	->	90	-0.00	0.00
6	->	91	-0.00	0.00
6	->	92	-0.00	0.00
6	->	93	-0.00	0.00
6	->	94	-0.00	0.00
6	->	95	-0.00	0.00
6	->	96	-0.00	0.00
6	->	97	-0.00	0.00
6	->	98	-0.00	0.00
6	->	99	-0.00	0.00
6	->	100	-0.00	0.00
6	->	101	-0.00	0.00
6	->	102	-0.00	0.00
6	->	103	-0.00	0.00
6	->	104	-0.00	0.00
6	->	105	-0.00	0.00
6	->	106	-0.00	0.00
7	->	1	-0.00	0.00
7	->	2	-0.00	0.00
7	->	3	0.01	0.01
7	->	4	0.06	0.06
7	->	5	-6.59	2.33
7	->	6	-13.29	0.41
7	->	7	26.43	1.26
7	->	8	-11.49	0.40
7	->	9	-1.32	0.38
7	->	10	-1.81	0.88

7 ->	11	-0.04	0.02
7 ->	12	-0.02	0.01
7 ->	13	-0.01	0.01
7 ->	14	-0.05	0.02
7 ->	15	-0.00	0.00
7 ->	16	-0.00	0.00
7 ->	17	-0.00	0.00
7 ->	18	-0.00	0.00
7 ->	19	0.01	0.01
7 ->	20	-0.03	0.01
7 ->	21	-0.01	0.00
7 ->	22	0.03	0.02
7 ->	23	-0.39	0.16
7 ->	24	-0.09	0.06
7 ->	25	-0.00	0.00
7 ->	26	0.00	0.01
7 ->	27	-2.32	1.19
7 ->	28	-0.04	0.02
7 ->	29	-0.00	0.00
7 ->	30	-0.00	0.00
7 ->	31	-0.00	0.00
7 ->	32	-0.00	0.00
7 ->	33	0.00	0.00
7 ->	34	0.00	0.00
7 ->	35	0.00	0.00
7 ->	36	0.00	0.00
7 ->	37	0.00	0.00
7 ->	38	-0.00	0.00
7 ->	39	0.00	0.00
7 ->	40	0.00	0.00
7 ->	41	0.00	0.00
7 ->	42	0.00	0.00
7 ->	43	-0.00	0.00
7 ->	44	-0.00	0.00
7 ->	45	0.00	0.00
7 ->	46	-0.00	0.00
7 ->	47	0.00	0.00
7 ->	48	0.00	0.00
7 ->	49	0.00	0.00
7 ->	50	0.00	0.00
7 ->	51	0.00	0.00
7 ->	52	0.00	0.00
7 ->	53	0.00	0.00
7 ->	54	0.00	0.00
7 ->	55	0.00	0.00
7 ->	56	0.00	0.00
7 ->	57	0.00	0.00
7 ->	58	-0.00	0.00
7 ->	59	0.00	0.00
7 ->	60	0.00	0.00

7	->	61	0.00	0.00
7	->	62	0.00	0.00
7	->	63	0.00	0.00
7	->	64	0.00	0.00
7	->	65	0.00	0.00
7	->	66	0.00	0.00
7	->	67	0.00	0.00
7	->	68	0.00	0.00
7	->	69	0.00	0.00
7	->	70	0.00	0.00
7	->	71	0.00	0.00
7	->	72	0.00	0.00
7	->	73	0.00	0.00
7	->	74	-0.00	0.00
7	->	75	0.00	0.00
7	->	76	0.00	0.00
7	->	77	-0.00	0.00
7	->	78	-0.00	0.00
7	->	79	0.00	0.00
7	->	80	-0.00	0.00
7	->	81	-0.00	0.00
7	->	82	0.00	0.00
7	->	83	0.00	0.00
7	->	84	0.00	0.00
7	->	85	0.00	0.00
7	->	86	0.00	0.00
7	->	87	-0.00	0.00
7	->	88	-0.00	0.00
7	->	89	-0.00	0.00
7	->	90	-0.01	0.01
7	->	91	-0.03	0.02
7	->	92	-0.01	0.00
7	->	93	-0.00	0.00
7	->	94	-0.00	0.00
7	->	95	-0.00	0.00
7	->	96	-0.00	0.00
7	->	97	-0.00	0.00
7	->	98	-0.00	0.00
7	->	99	-0.00	0.00
7	->	100	-0.00	0.00
7	->	101	-0.00	0.00
7	->	102	-0.00	0.00
7	->	103	0.00	0.00
7	->	104	0.00	0.00
7	->	105	-0.00	0.00
7	->	106	0.00	0.00
8	->	1	-0.04	0.01
8	->	2	0.00	0.00
8	->	3	-0.01	0.00
8	->	4	-0.06	0.03

8	->	5	0.15	0.19
8	->	6	-0.61	0.23
8	->	7	-11.52	0.40
8	->	8	-71.07	2.19
8	->	9	-18.88	1.27
8	->	10	-0.61	0.21
8	->	11	-0.00	0.03
8	->	12	-0.04	0.01
8	->	13	-0.13	0.13
8	->	14	-0.01	0.00
8	->	15	-0.00	0.00
8	->	16	0.01	0.00
8	->	17	0.00	0.00
8	->	18	0.00	0.00
8	->	19	-0.00	0.00
8	->	20	-0.00	0.00
8	->	21	0.00	0.00
8	->	22	-0.02	0.00
8	->	23	0.01	0.01
8	->	24	-0.01	0.00
8	->	25	-0.01	0.00
8	->	26	-0.00	0.00
8	->	27	0.02	0.02
8	->	28	-0.00	0.00
8	->	29	-0.00	0.00
8	->	30	-0.00	0.00
8	->	31	-0.01	0.00
8	->	32	-0.00	0.00
8	->	33	0.00	0.00
8	->	34	-0.00	0.00
8	->	35	0.00	0.00
8	->	36	-0.00	0.00
8	->	37	0.00	0.00
8	->	38	0.00	0.00
8	->	39	0.00	0.00
8	->	40	-0.00	0.00
8	->	41	-0.00	0.00
8	->	42	-0.00	0.00
8	->	43	-0.00	0.00
8	->	44	-0.01	0.00
8	->	45	-0.00	0.00
8	->	46	0.00	0.00
8	->	47	-0.00	0.00
8	->	48	-0.00	0.00
8	->	49	-0.00	0.00
8	->	50	-0.00	0.00
8	->	51	-0.00	0.00
8	->	52	-0.00	0.00
8	->	53	-0.00	0.00
8	->	54	-0.00	0.00

8	->	55	0.00	0.00
8	->	56	-0.00	0.00
8	->	57	-0.00	0.00
8	->	58	0.00	0.00
8	->	59	-0.00	0.00
8	->	60	-0.00	0.00
8	->	61	-0.00	0.00
8	->	62	0.00	0.00
8	->	63	0.00	0.00
8	->	64	0.00	0.00
8	->	65	0.00	0.00
8	->	66	0.00	0.00
8	->	67	0.00	0.00
8	->	68	-0.00	0.00
8	->	69	-0.00	0.00
8	->	70	-0.00	0.00
8	->	71	0.00	0.00
8	->	72	-0.00	0.00
8	->	73	-0.00	0.00
8	->	74	0.00	0.00
8	->	75	0.00	0.00
8	->	76	-0.00	0.00
8	->	77	-0.00	0.00
8	->	78	-0.00	0.00
8	->	79	-0.00	0.00
8	->	80	-0.00	0.00
8	->	81	-0.00	0.00
8	->	82	-0.00	0.00
8	->	83	-0.00	0.00
8	->	84	0.00	0.00
8	->	85	0.00	0.00
8	->	86	0.00	0.00
8	->	87	0.00	0.00
8	->	88	0.00	0.00
8	->	89	0.01	0.00
8	->	90	0.01	0.00
8	->	91	0.02	0.00
8	->	92	0.02	0.00
8	->	93	0.01	0.00
8	->	94	0.01	0.00
8	->	95	0.00	0.00
8	->	96	0.00	0.00
8	->	97	0.00	0.00
8	->	98	0.00	0.00
8	->	99	0.00	0.00
8	->	100	0.00	0.00
8	->	101	0.00	0.00
8	->	102	0.00	0.00
8	->	103	0.00	0.00
8	->	104	0.01	0.00

8	->	105	0.01	0.00
8	->	106	0.00	0.00
9	->	1	-0.00	0.00
9	->	2	-0.00	0.00
9	->	3	-0.00	0.00
9	->	4	-0.01	0.01
9	->	5	-0.06	0.10
9	->	6	-0.04	0.02
9	->	7	-1.32	0.37
9	->	8	-18.99	1.29
9	->	9	15.25	1.89
9	->	10	-9.72	0.60
9	->	11	-0.36	0.12
9	->	12	-0.04	0.02
9	->	13	-0.02	0.01
9	->	14	-0.01	0.01
9	->	15	-0.00	0.00
9	->	16	-0.00	0.00
9	->	17	-0.00	0.00
9	->	18	-0.00	0.00
9	->	19	-0.00	0.00
9	->	20	-0.00	0.00
9	->	21	-0.00	0.00
9	->	22	-0.00	0.00
9	->	23	-0.03	0.03
9	->	24	-0.00	0.00
9	->	25	-0.00	0.00
9	->	26	-0.00	0.00
9	->	27	-0.08	0.06
9	->	28	-0.00	0.00
9	->	29	-0.00	0.00
9	->	30	-0.00	0.00
9	->	31	0.00	0.00
9	->	32	0.00	0.00
9	->	33	-0.00	0.00
9	->	34	0.00	0.00
9	->	35	-0.00	0.00
9	->	36	0.00	0.00
9	->	37	0.00	0.00
9	->	38	0.00	0.00
9	->	39	0.00	0.00
9	->	40	-0.00	0.00
9	->	41	0.00	0.00
9	->	42	0.00	0.00
9	->	43	0.00	0.00
9	->	44	-0.00	0.00
9	->	45	0.00	0.00
9	->	46	0.00	0.00
9	->	47	-0.00	0.00
9	->	48	0.00	0.00

9	->	49	0.00	0.00
9	->	50	0.00	0.00
9	->	51	0.00	0.00
9	->	52	0.00	0.00
9	->	53	-0.00	0.00
9	->	54	0.00	0.00
9	->	55	-0.00	0.00
9	->	56	0.00	0.00
9	->	57	0.00	0.00
9	->	58	0.00	0.00
9	->	59	-0.00	0.00
9	->	60	0.00	0.00
9	->	61	0.00	0.00
9	->	62	0.00	0.00
9	->	63	0.00	0.00
9	->	64	0.00	0.00
9	->	65	0.00	0.00
9	->	66	0.00	0.00
9	->	67	0.00	0.00
9	->	68	-0.00	0.00
9	->	69	-0.00	0.00
9	->	70	0.00	0.00
9	->	71	0.00	0.00
9	->	72	-0.00	0.00
9	->	73	0.00	0.00
9	->	74	0.00	0.00
9	->	75	0.00	0.00
9	->	76	-0.00	0.00
9	->	77	-0.00	0.00
9	->	78	-0.00	0.00
9	->	79	0.00	0.00
9	->	80	0.00	0.00
9	->	81	-0.00	0.00
9	->	82	0.00	0.00
9	->	83	0.00	0.00
9	->	84	0.00	0.00
9	->	85	0.00	0.00
9	->	86	0.00	0.00
9	->	87	-0.00	0.00
9	->	88	0.00	0.00
9	->	89	-0.00	0.00
9	->	90	-0.00	0.00
9	->	91	-0.01	0.00
9	->	92	-0.00	0.00
9	->	93	0.00	0.00
9	->	94	0.00	0.00
9	->	95	0.00	0.00
9	->	96	0.00	0.00
9	->	97	-0.00	0.00
9	->	98	0.00	0.00

9	->	99	0.00	0.00
9	->	100	0.00	0.00
9	->	101	0.00	0.00
9	->	102	0.00	0.00
9	->	103	0.00	0.00
9	->	104	0.00	0.00
9	->	105	0.00	0.00
9	->	106	0.00	0.00
10	->	1	-0.07	0.02
10	->	2	-0.00	0.00
10	->	3	-0.02	0.01
10	->	4	-0.13	0.06
10	->	5	2.11	0.65
10	->	6	-0.07	0.02
10	->	7	-1.79	0.87
10	->	8	-0.63	0.21
10	->	9	-9.81	0.60
10	->	10	-36.43	2.19
10	->	11	-14.19	0.25
10	->	12	-1.36	0.66
10	->	13	-0.42	0.57
10	->	14	-0.15	0.22
10	->	15	-0.00	0.00
10	->	16	0.03	0.00
10	->	17	0.00	0.00
10	->	18	0.00	0.00
10	->	19	-0.02	0.01
10	->	20	-0.01	0.01
10	->	21	0.00	0.00
10	->	22	-0.11	0.03
10	->	23	1.92	0.70
10	->	24	0.03	0.01
10	->	25	-0.04	0.00
10	->	26	-0.05	0.02
10	->	27	2.43	0.95
10	->	28	-0.01	0.00
10	->	29	-0.00	0.00
10	->	30	-0.00	0.00
10	->	31	-0.02	0.00
10	->	32	-0.00	0.00
10	->	33	0.00	0.00
10	->	34	-0.00	0.00
10	->	35	0.00	0.00
10	->	36	-0.00	0.00
10	->	37	0.00	0.00
10	->	38	0.00	0.00
10	->	39	0.00	0.00
10	->	40	-0.01	0.00
10	->	41	-0.00	0.00
10	->	42	-0.00	0.00

10 ->	43	-0.00	0.00
10 ->	44	-0.02	0.00
10 ->	45	-0.00	0.00
10 ->	46	0.01	0.00
10 ->	47	-0.00	0.00
10 ->	48	-0.00	0.00
10 ->	49	-0.00	0.00
10 ->	50	-0.00	0.00
10 ->	51	-0.00	0.00
10 ->	52	-0.00	0.00
10 ->	53	-0.00	0.00
10 ->	54	-0.00	0.00
10 ->	55	0.00	0.00
10 ->	56	-0.00	0.00
10 ->	57	-0.00	0.00
10 ->	58	0.00	0.00
10 ->	59	-0.00	0.00
10 ->	60	-0.00	0.00
10 ->	61	-0.00	0.00
10 ->	62	0.00	0.00
10 ->	63	0.00	0.00
10 ->	64	0.00	0.00
10 ->	65	0.00	0.00
10 ->	66	0.00	0.00
10 ->	67	-0.00	0.00
10 ->	68	-0.00	0.00
10 ->	69	-0.00	0.00
10 ->	70	-0.00	0.00
10 ->	71	0.00	0.00
10 ->	72	-0.01	0.00
10 ->	73	-0.00	0.00
10 ->	74	0.01	0.00
10 ->	75	0.00	0.00
10 ->	76	-0.00	0.00
10 ->	77	-0.01	0.00
10 ->	78	-0.01	0.00
10 ->	79	-0.00	0.00
10 ->	80	-0.00	0.00
10 ->	81	-0.01	0.00
10 ->	82	-0.00	0.00
10 ->	83	-0.00	0.00
10 ->	84	0.00	0.00
10 ->	85	0.00	0.00
10 ->	86	0.00	0.00
10 ->	87	0.00	0.00
10 ->	88	0.01	0.00
10 ->	89	0.02	0.00
10 ->	90	0.07	0.02
10 ->	91	0.17	0.07
10 ->	92	0.05	0.01

10	->	93	0.01	0.00
10	->	94	0.01	0.00
10	->	95	0.00	0.00
10	->	96	0.00	0.00
10	->	97	0.01	0.00
10	->	98	0.00	0.00
10	->	99	0.01	0.00
10	->	100	0.01	0.00
10	->	101	0.01	0.00
10	->	102	0.01	0.00
10	->	103	0.01	0.00
10	->	104	0.02	0.00
10	->	105	0.02	0.01
10	->	106	0.01	0.00
11	->	1	-0.13	0.04
11	->	2	-0.01	0.01
11	->	3	-0.01	0.01
11	->	4	-0.04	0.03
11	->	5	0.09	0.02
11	->	6	-0.01	0.00
11	->	7	-0.04	0.02
11	->	8	-0.00	0.03
11	->	9	-0.39	0.13
11	->	10	-14.30	0.26
11	->	11	-36.40	1.29
11	->	12	-8.82	0.55
11	->	13	-1.18	1.14
11	->	14	-0.05	0.01
11	->	15	-0.01	0.00
11	->	16	0.02	0.00
11	->	17	0.00	0.00
11	->	18	0.00	0.00
11	->	19	-0.00	0.00
11	->	20	-0.01	0.00
11	->	21	-0.00	0.00
11	->	22	-0.03	0.01
11	->	23	0.00	0.02
11	->	24	-0.00	0.00
11	->	25	-0.01	0.00
11	->	26	-0.00	0.00
11	->	27	0.02	0.01
11	->	28	-0.00	0.00
11	->	29	-0.00	0.00
11	->	30	0.00	0.00
11	->	31	-0.01	0.00
11	->	32	-0.00	0.00
11	->	33	0.00	0.00
11	->	34	-0.00	0.00
11	->	35	0.00	0.00
11	->	36	-0.00	0.00

11	->	37	0.00	0.00
11	->	38	0.00	0.00
11	->	39	0.00	0.00
11	->	40	-0.00	0.00
11	->	41	-0.00	0.00
11	->	42	-0.00	0.00
11	->	43	-0.00	0.00
11	->	44	-0.01	0.00
11	->	45	-0.00	0.00
11	->	46	0.01	0.00
11	->	47	-0.00	0.00
11	->	48	-0.00	0.00
11	->	49	-0.00	0.00
11	->	50	-0.00	0.00
11	->	51	-0.00	0.00
11	->	52	-0.00	0.00
11	->	53	-0.00	0.00
11	->	54	-0.00	0.00
11	->	55	-0.00	0.00
11	->	56	-0.00	0.00
11	->	57	-0.00	0.00
11	->	58	0.00	0.00
11	->	59	-0.00	0.00
11	->	60	-0.00	0.00
11	->	61	-0.00	0.00
11	->	62	0.00	0.00
11	->	63	0.00	0.00
11	->	64	0.00	0.00
11	->	65	0.00	0.00
11	->	66	0.00	0.00
11	->	67	0.00	0.00
11	->	68	-0.00	0.00
11	->	69	-0.00	0.00
11	->	70	-0.00	0.00
11	->	71	0.00	0.00
11	->	72	-0.01	0.00
11	->	73	-0.00	0.00
11	->	74	0.01	0.00
11	->	75	0.00	0.00
11	->	76	-0.00	0.00
11	->	77	-0.01	0.00
11	->	78	-0.01	0.00
11	->	79	-0.00	0.00
11	->	80	-0.00	0.00
11	->	81	-0.01	0.00
11	->	82	-0.00	0.00
11	->	83	-0.00	0.00
11	->	84	0.00	0.00
11	->	85	0.00	0.00
11	->	86	0.00	0.00

11	->	87	0.00	0.00
11	->	88	0.00	0.00
11	->	89	0.01	0.00
11	->	90	0.02	0.00
11	->	91	0.02	0.01
11	->	92	0.03	0.01
11	->	93	0.02	0.01
11	->	94	0.01	0.00
11	->	95	0.00	0.00
11	->	96	0.00	0.00
11	->	97	0.01	0.00
11	->	98	0.00	0.00
11	->	99	0.00	0.00
11	->	100	0.01	0.00
11	->	101	0.01	0.00
11	->	102	0.01	0.00
11	->	103	0.02	0.01
11	->	104	0.02	0.01
11	->	105	0.02	0.01
11	->	106	0.01	0.00
12	->	1	0.22	0.50
12	->	2	-0.01	0.01
12	->	3	-0.20	0.04
12	->	4	-0.16	0.11
12	->	5	-0.20	0.11
12	->	6	-0.01	0.00
12	->	7	-0.02	0.01
12	->	8	-0.04	0.01
12	->	9	-0.04	0.02
12	->	10	-1.37	0.68
12	->	11	-8.93	0.55
12	->	12	-108.36	2.80
12	->	13	-9.26	0.73
12	->	14	-2.39	0.92
12	->	15	-0.11	0.04
12	->	16	-0.07	0.01
12	->	17	-0.01	0.00
12	->	18	-0.00	0.00
12	->	19	0.09	0.07
12	->	20	-0.06	0.02
12	->	21	-0.00	0.00
12	->	22	0.45	0.28
12	->	23	-0.35	0.40
12	->	24	-0.02	0.01
12	->	25	0.03	0.01
12	->	26	-0.02	0.03
12	->	27	-0.07	0.05
12	->	28	0.00	0.00
12	->	29	0.00	0.00
12	->	30	-0.00	0.00

12	->	31	0.02	0.01
12	->	32	0.00	0.00
12	->	33	-0.00	0.00
12	->	34	0.00	0.00
12	->	35	-0.01	0.00
12	->	36	0.00	0.00
12	->	37	-0.00	0.00
12	->	38	-0.01	0.00
12	->	39	-0.00	0.00
12	->	40	0.01	0.00
12	->	41	0.00	0.00
12	->	42	0.00	0.00
12	->	43	0.00	0.00
12	->	44	0.04	0.01
12	->	45	0.00	0.00
12	->	46	-0.03	0.00
12	->	47	0.00	0.00
12	->	48	0.00	0.00
12	->	49	0.00	0.00
12	->	50	0.00	0.00
12	->	51	0.00	0.00
12	->	52	0.00	0.00
12	->	53	0.01	0.00
12	->	54	0.00	0.00
12	->	55	0.00	0.00
12	->	56	0.00	0.00
12	->	57	0.00	0.00
12	->	58	-0.00	0.00
12	->	59	0.00	0.00
12	->	60	0.00	0.00
12	->	61	0.00	0.00
12	->	62	-0.00	0.00
12	->	63	-0.00	0.00
12	->	64	-0.00	0.00
12	->	65	-0.00	0.00
12	->	66	-0.00	0.00
12	->	67	0.00	0.00
12	->	68	0.00	0.00
12	->	69	0.00	0.00
12	->	70	0.00	0.00
12	->	71	-0.00	0.00
12	->	72	0.02	0.00
12	->	73	0.00	0.00
12	->	74	-0.02	0.01
12	->	75	-0.01	0.00
12	->	76	0.01	0.00
12	->	77	0.02	0.01
12	->	78	0.02	0.00
12	->	79	0.00	0.00
12	->	80	0.00	0.00

12	->	81	0.01	0.00
12	->	82	0.00	0.00
12	->	83	0.00	0.00
12	->	84	-0.00	0.00
12	->	85	-0.00	0.00
12	->	86	-0.01	0.00
12	->	87	-0.01	0.00
12	->	88	-0.01	0.00
12	->	89	-0.04	0.01
12	->	90	-0.52	0.49
12	->	91	-6.84	2.43
12	->	92	-2.79	2.48
12	->	93	-0.08	0.04
12	->	94	-0.03	0.01
12	->	95	-0.01	0.00
12	->	96	-0.00	0.00
12	->	97	-0.01	0.00
12	->	98	-0.01	0.00
12	->	99	-0.01	0.00
12	->	100	-0.02	0.00
12	->	101	-0.03	0.01
12	->	102	-0.06	0.03
12	->	103	-0.07	0.05
12	->	104	-0.06	0.03
12	->	105	-0.04	0.01
12	->	106	-0.01	0.00
13	->	1	-2.02	1.77
13	->	2	-1.75	1.06
13	->	3	-3.27	0.62
13	->	4	-2.12	0.39
13	->	5	-0.42	0.12
13	->	6	-0.02	0.01
13	->	7	-0.01	0.01
13	->	8	-0.13	0.13
13	->	9	-0.02	0.01
13	->	10	-0.43	0.57
13	->	11	-1.21	1.15
13	->	12	-9.33	0.73
13	->	13	-109.04	2.57
13	->	14	-12.50	0.49
13	->	15	-0.74	0.10
13	->	16	-0.18	0.04
13	->	17	-0.03	0.01
13	->	18	0.01	0.01
13	->	19	0.19	0.08
13	->	20	-0.09	0.02
13	->	21	-0.01	0.01
13	->	22	0.13	0.06
13	->	23	-0.04	0.02
13	->	24	-0.01	0.01

13	->	25	0.02	0.00
13	->	26	-0.00	0.00
13	->	27	-0.02	0.01
13	->	28	0.00	0.00
13	->	29	0.00	0.00
13	->	30	-0.00	0.00
13	->	31	0.01	0.00
13	->	32	0.00	0.00
13	->	33	0.00	0.00
13	->	34	0.00	0.00
13	->	35	-0.00	0.00
13	->	36	0.00	0.00
13	->	37	-0.00	0.00
13	->	38	-0.00	0.00
13	->	39	-0.00	0.00
13	->	40	0.00	0.00
13	->	41	0.00	0.00
13	->	42	0.00	0.00
13	->	43	0.00	0.00
13	->	44	0.01	0.00
13	->	45	0.00	0.00
13	->	46	-0.01	0.00
13	->	47	0.00	0.00
13	->	48	0.00	0.00
13	->	49	0.00	0.00
13	->	50	0.00	0.00
13	->	51	0.00	0.00
13	->	52	0.00	0.00
13	->	53	0.00	0.00
13	->	54	0.00	0.00
13	->	55	0.00	0.00
13	->	56	0.00	0.00
13	->	57	0.00	0.00
13	->	58	-0.00	0.00
13	->	59	0.00	0.00
13	->	60	0.00	0.00
13	->	61	0.00	0.00
13	->	62	-0.00	0.00
13	->	63	-0.00	0.00
13	->	64	-0.00	0.00
13	->	65	-0.00	0.00
13	->	66	-0.00	0.00
13	->	67	0.00	0.00
13	->	68	0.00	0.00
13	->	69	0.00	0.00
13	->	70	0.00	0.00
13	->	71	-0.00	0.00
13	->	72	0.00	0.00
13	->	73	0.00	0.00
13	->	74	-0.00	0.00

13	->	75	-0.00	0.00
13	->	76	0.00	0.00
13	->	77	0.00	0.00
13	->	78	0.00	0.00
13	->	79	0.00	0.00
13	->	80	0.00	0.00
13	->	81	0.00	0.00
13	->	82	0.00	0.00
13	->	83	0.00	0.00
13	->	84	-0.00	0.00
13	->	85	-0.00	0.00
13	->	86	-0.00	0.00
13	->	87	-0.00	0.00
13	->	88	-0.00	0.00
13	->	89	-0.01	0.00
13	->	90	-0.03	0.01
13	->	91	-0.18	0.07
13	->	92	-0.41	0.23
13	->	93	-0.05	0.02
13	->	94	-0.02	0.00
13	->	95	-0.01	0.00
13	->	96	-0.00	0.00
13	->	97	-0.01	0.00
13	->	98	-0.00	0.00
13	->	99	-0.01	0.00
13	->	100	-0.01	0.00
13	->	101	-0.01	0.00
13	->	102	-0.01	0.00
13	->	103	-0.01	0.00
13	->	104	-0.01	0.00
13	->	105	-0.01	0.00
13	->	106	-0.00	0.00
14	->	1	-1.93	1.33
14	->	2	-1.26	0.27
14	->	3	-3.79	0.50
14	->	4	-0.68	0.30
14	->	5	-0.53	0.22
14	->	6	-0.03	0.03
14	->	7	-0.05	0.02
14	->	8	-0.01	0.00
14	->	9	-0.01	0.01
14	->	10	-0.15	0.23
14	->	11	-0.05	0.01
14	->	12	-2.39	0.90
14	->	13	-12.58	0.49
14	->	14	29.77	1.05
14	->	15	-16.93	0.41
14	->	16	-1.18	0.26
14	->	17	-0.16	0.06
14	->	18	-0.06	0.02

14 ->	19	-1.36	0.47
14 ->	20	-2.70	0.46
14 ->	21	-0.07	0.02
14 ->	22	-0.59	0.29
14 ->	23	-2.30	0.39
14 ->	24	-0.08	0.04
14 ->	25	-0.02	0.01
14 ->	26	-0.04	0.01
14 ->	27	-0.00	0.02
14 ->	28	-0.00	0.00
14 ->	29	-0.00	0.00
14 ->	30	-0.00	0.00
14 ->	31	0.00	0.00
14 ->	32	-0.00	0.00
14 ->	33	-0.00	0.00
14 ->	34	0.00	0.00
14 ->	35	-0.00	0.00
14 ->	36	0.00	0.00
14 ->	37	-0.00	0.00
14 ->	38	-0.00	0.00
14 ->	39	0.00	0.00
14 ->	40	0.00	0.00
14 ->	41	0.00	0.00
14 ->	42	-0.00	0.00
14 ->	43	-0.00	0.00
14 ->	44	-0.00	0.00
14 ->	45	-0.00	0.00
14 ->	46	-0.00	0.00
14 ->	47	-0.00	0.00
14 ->	48	0.00	0.00
14 ->	49	0.00	0.00
14 ->	50	0.00	0.00
14 ->	51	0.00	0.00
14 ->	52	0.00	0.00
14 ->	53	0.00	0.00
14 ->	54	0.00	0.00
14 ->	55	0.00	0.00
14 ->	56	0.00	0.00
14 ->	57	0.00	0.00
14 ->	58	-0.00	0.00
14 ->	59	0.00	0.00
14 ->	60	0.00	0.00
14 ->	61	0.00	0.00
14 ->	62	0.00	0.00
14 ->	63	-0.00	0.00
14 ->	64	-0.00	0.00
14 ->	65	0.00	0.00
14 ->	66	-0.00	0.00
14 ->	67	-0.00	0.00
14 ->	68	0.00	0.00

14	->	69	0.00	0.00
14	->	70	0.00	0.00
14	->	71	0.00	0.00
14	->	72	0.00	0.00
14	->	73	0.00	0.00
14	->	74	-0.00	0.00
14	->	75	-0.00	0.00
14	->	76	0.00	0.00
14	->	77	0.00	0.00
14	->	78	0.00	0.00
14	->	79	0.00	0.00
14	->	80	0.00	0.00
14	->	81	0.00	0.00
14	->	82	0.00	0.00
14	->	83	0.00	0.00
14	->	84	-0.00	0.00
14	->	85	-0.00	0.00
14	->	86	-0.00	0.00
14	->	87	-0.00	0.00
14	->	88	-0.00	0.00
14	->	89	-0.00	0.00
14	->	90	-0.01	0.02
14	->	91	-0.79	0.58
14	->	92	-2.10	1.44
14	->	93	-0.11	0.08
14	->	94	-0.01	0.01
14	->	95	-0.00	0.00
14	->	96	-0.01	0.00
14	->	97	-0.01	0.00
14	->	98	-0.00	0.00
14	->	99	-0.00	0.00
14	->	100	-0.00	0.00
14	->	101	-0.00	0.00
14	->	102	-0.00	0.00
14	->	103	-0.00	0.00
14	->	104	-0.00	0.00
14	->	105	-0.00	0.00
14	->	106	-0.00	0.00
15	->	1	-3.67	2.22
15	->	2	-1.05	0.30
15	->	3	-1.00	0.46
15	->	4	0.00	0.01
15	->	5	0.01	0.01
15	->	6	-0.00	0.00
15	->	7	-0.00	0.00
15	->	8	-0.00	0.00
15	->	9	-0.00	0.00
15	->	10	-0.00	0.00
15	->	11	-0.01	0.00
15	->	12	-0.11	0.04

15	->	13	-0.74	0.10
15	->	14	-16.93	0.40
15	->	15	21.03	2.46
15	->	16	-11.28	0.49
15	->	17	-0.29	0.06
15	->	18	-0.02	0.02
15	->	19	-0.22	0.29
15	->	20	-0.08	0.04
15	->	21	-0.01	0.00
15	->	22	0.01	0.13
15	->	23	-0.01	0.00
15	->	24	-0.00	0.00
15	->	25	-0.00	0.00
15	->	26	-0.00	0.00
15	->	27	0.00	0.00
15	->	28	0.00	0.00
15	->	29	0.00	0.00
15	->	30	-0.00	0.00
15	->	31	0.00	0.00
15	->	32	0.00	0.00
15	->	33	-0.00	0.00
15	->	34	0.00	0.00
15	->	35	-0.00	0.00
15	->	36	0.00	0.00
15	->	37	0.00	0.00
15	->	38	-0.00	0.00
15	->	39	0.00	0.00
15	->	40	0.00	0.00
15	->	41	0.00	0.00
15	->	42	-0.00	0.00
15	->	43	-0.00	0.00
15	->	44	0.00	0.00
15	->	45	0.00	0.00
15	->	46	-0.00	0.00
15	->	47	-0.00	0.00
15	->	48	0.00	0.00
15	->	49	0.00	0.00
15	->	50	0.00	0.00
15	->	51	0.00	0.00
15	->	52	0.00	0.00
15	->	53	0.00	0.00
15	->	54	0.00	0.00
15	->	55	0.00	0.00
15	->	56	0.00	0.00
15	->	57	0.00	0.00
15	->	58	-0.00	0.00
15	->	59	0.00	0.00
15	->	60	0.00	0.00
15	->	61	0.00	0.00
15	->	62	-0.00	0.00

15	->	63	-0.00	0.00
15	->	64	-0.00	0.00
15	->	65	0.00	0.00
15	->	66	-0.00	0.00
15	->	67	0.00	0.00
15	->	68	0.00	0.00
15	->	69	0.00	0.00
15	->	70	0.00	0.00
15	->	71	0.00	0.00
15	->	72	0.00	0.00
15	->	73	0.00	0.00
15	->	74	-0.00	0.00
15	->	75	-0.00	0.00
15	->	76	0.00	0.00
15	->	77	0.00	0.00
15	->	78	0.00	0.00
15	->	79	0.00	0.00
15	->	80	0.00	0.00
15	->	81	0.00	0.00
15	->	82	0.00	0.00
15	->	83	0.00	0.00
15	->	84	-0.00	0.00
15	->	85	-0.00	0.00
15	->	86	-0.00	0.00
15	->	87	-0.00	0.00
15	->	88	-0.00	0.00
15	->	89	-0.00	0.00
15	->	90	-0.00	0.01
15	->	91	-0.19	0.40
15	->	92	-3.00	4.52
15	->	93	-0.62	1.05
15	->	94	-0.03	0.02
15	->	95	-0.02	0.03
15	->	96	-0.01	0.01
15	->	97	-0.01	0.02
15	->	98	-0.00	0.00
15	->	99	-0.00	0.00
15	->	100	-0.00	0.00
15	->	101	-0.00	0.00
15	->	102	-0.00	0.00
15	->	103	-0.00	0.00
15	->	104	-0.00	0.00
15	->	105	-0.00	0.00
15	->	106	-0.00	0.00
16	->	1	-0.66	0.88
16	->	2	-0.21	0.16
16	->	3	-1.48	0.65
16	->	4	-0.00	0.01
16	->	5	0.04	0.01
16	->	6	-0.00	0.00

16	->	7	-0.00	0.00
16	->	8	0.01	0.00
16	->	9	-0.00	0.00
16	->	10	0.03	0.00
16	->	11	0.02	0.00
16	->	12	-0.07	0.01
16	->	13	-0.18	0.04
16	->	14	-1.18	0.26
16	->	15	-11.39	0.49
16	->	16	-21.67	2.46
16	->	17	-17.26	0.49
16	->	18	-6.52	1.69
16	->	19	-4.22	1.15
16	->	20	-1.84	0.37
16	->	21	-0.23	0.07
16	->	22	-0.35	0.12
16	->	23	-0.05	0.02
16	->	24	-0.02	0.00
16	->	25	-0.07	0.01
16	->	26	-0.01	0.00
16	->	27	-0.00	0.00
16	->	28	-0.00	0.00
16	->	29	-0.00	0.00
16	->	30	0.00	0.00
16	->	31	-0.02	0.00
16	->	32	-0.00	0.00
16	->	33	-0.00	0.00
16	->	34	-0.00	0.00
16	->	35	0.01	0.00
16	->	36	-0.01	0.00
16	->	37	0.00	0.00
16	->	38	0.01	0.00
16	->	39	0.00	0.00
16	->	40	-0.01	0.00
16	->	41	-0.00	0.00
16	->	42	-0.00	0.00
16	->	43	0.00	0.00
16	->	44	-0.08	0.04
16	->	45	-0.01	0.00
16	->	46	0.04	0.01
16	->	47	-0.00	0.00
16	->	48	-0.00	0.00
16	->	49	-0.00	0.00
16	->	50	-0.00	0.00
16	->	51	-0.00	0.00
16	->	52	-0.00	0.00
16	->	53	-0.01	0.00
16	->	54	-0.00	0.00
16	->	55	-0.00	0.00
16	->	56	-0.00	0.00

16	->	57	-0.00	0.00
16	->	58	0.01	0.00
16	->	59	-0.00	0.00
16	->	60	-0.00	0.00
16	->	61	-0.00	0.00
16	->	62	0.00	0.00
16	->	63	0.00	0.00
16	->	64	0.00	0.00
16	->	65	0.00	0.00
16	->	66	0.00	0.00
16	->	67	0.00	0.00
16	->	68	-0.00	0.00
16	->	69	-0.00	0.00
16	->	70	-0.00	0.00
16	->	71	0.00	0.00
16	->	72	-0.01	0.00
16	->	73	-0.00	0.00
16	->	74	0.01	0.00
16	->	75	0.01	0.00
16	->	76	-0.01	0.00
16	->	77	-0.01	0.00
16	->	78	-0.01	0.00
16	->	79	-0.00	0.00
16	->	80	-0.00	0.00
16	->	81	-0.01	0.00
16	->	82	-0.00	0.00
16	->	83	0.00	0.00
16	->	84	0.00	0.00
16	->	85	0.00	0.00
16	->	86	0.00	0.00
16	->	87	0.01	0.00
16	->	88	0.01	0.00
16	->	89	0.01	0.00
16	->	90	0.03	0.00
16	->	91	0.10	0.04
16	->	92	0.25	0.24
16	->	93	-0.16	0.34
16	->	94	0.02	0.06
16	->	95	-0.02	0.06
16	->	96	-0.30	0.72
16	->	97	-1.06	1.31
16	->	98	-0.17	0.27
16	->	99	0.01	0.02
16	->	100	0.02	0.00
16	->	101	0.02	0.00
16	->	102	0.02	0.00
16	->	103	0.01	0.00
16	->	104	0.01	0.00
16	->	105	0.01	0.00
16	->	106	0.00	0.00

17	->	1	-0.08	0.11
17	->	2	-0.04	0.03
17	->	3	-1.41	0.77
17	->	4	-0.01	0.00
17	->	5	-0.00	0.00
17	->	6	-0.01	0.00
17	->	7	-0.00	0.00
17	->	8	0.00	0.00
17	->	9	-0.00	0.00
17	->	10	0.00	0.00
17	->	11	0.00	0.00
17	->	12	-0.01	0.00
17	->	13	-0.03	0.01
17	->	14	-0.16	0.06
17	->	15	-0.29	0.06
17	->	16	-17.31	0.49
17	->	17	19.70	1.51
17	->	18	-15.19	0.40
17	->	19	-1.08	0.24
17	->	20	-2.31	0.35
17	->	21	-3.40	1.32
17	->	22	-0.14	0.07
17	->	23	-0.05	0.03
17	->	24	-0.07	0.02
17	->	25	-0.03	0.01
17	->	26	-0.00	0.00
17	->	27	-0.00	0.00
17	->	28	-0.00	0.00
17	->	29	-0.00	0.00
17	->	30	-0.00	0.00
17	->	31	-0.00	0.00
17	->	32	-0.00	0.00
17	->	33	-0.00	0.00
17	->	34	0.00	0.00
17	->	35	0.00	0.00
17	->	36	-0.00	0.00
17	->	37	0.00	0.00
17	->	38	0.00	0.00
17	->	39	0.00	0.00
17	->	40	-0.00	0.00
17	->	41	-0.00	0.00
17	->	42	-0.00	0.00
17	->	43	-0.00	0.00
17	->	44	-0.00	0.00
17	->	45	-0.00	0.00
17	->	46	-0.00	0.00
17	->	47	-0.00	0.00
17	->	48	-0.00	0.00
17	->	49	-0.00	0.00
17	->	50	-0.00	0.00

17	->	51	-0.00	0.00
17	->	52	-0.00	0.00
17	->	53	-0.00	0.00
17	->	54	-0.00	0.00
17	->	55	0.00	0.00
17	->	56	-0.00	0.00
17	->	57	0.00	0.00
17	->	58	0.00	0.00
17	->	59	-0.00	0.00
17	->	60	0.00	0.00
17	->	61	0.00	0.00
17	->	62	0.00	0.00
17	->	63	0.00	0.00
17	->	64	0.00	0.00
17	->	65	0.00	0.00
17	->	66	0.00	0.00
17	->	67	0.00	0.00
17	->	68	-0.00	0.00
17	->	69	-0.00	0.00
17	->	70	-0.00	0.00
17	->	71	0.00	0.00
17	->	72	-0.00	0.00
17	->	73	0.00	0.00
17	->	74	0.00	0.00
17	->	75	0.00	0.00
17	->	76	-0.00	0.00
17	->	77	-0.00	0.00
17	->	78	-0.00	0.00
17	->	79	-0.00	0.00
17	->	80	0.00	0.00
17	->	81	-0.00	0.00
17	->	82	0.00	0.00
17	->	83	0.00	0.00
17	->	84	0.00	0.00
17	->	85	0.00	0.00
17	->	86	0.00	0.00
17	->	87	0.00	0.00
17	->	88	0.00	0.00
17	->	89	0.00	0.00
17	->	90	0.00	0.00
17	->	91	-0.00	0.01
17	->	92	0.01	0.03
17	->	93	-0.01	0.01
17	->	94	-0.01	0.01
17	->	95	-0.01	0.01
17	->	96	-0.60	0.83
17	->	97	-0.11	0.12
17	->	98	-0.01	0.01
17	->	99	-0.00	0.00
17	->	100	-0.00	0.00

17	->	101	-0.00	0.00
17	->	102	-0.00	0.00
17	->	103	0.00	0.00
17	->	104	0.00	0.00
17	->	105	0.00	0.00
17	->	106	0.00	0.00
18	->	1	0.00	0.01
18	->	2	-0.01	0.00
18	->	3	-0.04	0.02
18	->	4	-0.00	0.00
18	->	5	-0.00	0.00
18	->	6	-0.00	0.00
18	->	7	-0.00	0.00
18	->	8	0.00	0.00
18	->	9	-0.00	0.00
18	->	10	0.00	0.00
18	->	11	0.00	0.00
18	->	12	-0.00	0.00
18	->	13	0.01	0.01
18	->	14	-0.06	0.02
18	->	15	-0.02	0.02
18	->	16	-6.60	1.70
18	->	17	-15.24	0.40
18	->	18	24.99	2.65
18	->	19	-10.32	0.63
18	->	20	-1.03	0.17
18	->	21	-2.49	0.41
18	->	22	-2.45	0.50
18	->	23	-0.10	0.06
18	->	24	-0.05	0.03
18	->	25	-0.13	0.11
18	->	26	-0.01	0.00
18	->	27	-0.01	0.00
18	->	28	-0.00	0.00
18	->	29	-0.00	0.00
18	->	30	-0.00	0.00
18	->	31	-0.00	0.00
18	->	32	-0.01	0.00
18	->	33	-0.01	0.01
18	->	34	-0.00	0.00
18	->	35	0.00	0.00
18	->	36	-0.00	0.00
18	->	37	0.00	0.00
18	->	38	0.00	0.00
18	->	39	0.00	0.00
18	->	40	-0.00	0.00
18	->	41	-0.00	0.00
18	->	42	-0.00	0.00
18	->	43	-0.01	0.01
18	->	44	-0.04	0.07

18	->	45	-0.01	0.00
18	->	46	-0.01	0.01
18	->	47	-0.01	0.00
18	->	48	-0.00	0.00
18	->	49	-0.00	0.00
18	->	50	-0.00	0.00
18	->	51	-0.00	0.00
18	->	52	-0.00	0.00
18	->	53	-0.00	0.00
18	->	54	-0.00	0.00
18	->	55	0.00	0.00
18	->	56	-0.00	0.00
18	->	57	0.00	0.00
18	->	58	0.00	0.00
18	->	59	-0.00	0.00
18	->	60	-0.00	0.00
18	->	61	0.00	0.00
18	->	62	0.00	0.00
18	->	63	0.00	0.00
18	->	64	0.00	0.00
18	->	65	0.00	0.00
18	->	66	0.00	0.00
18	->	67	0.00	0.00
18	->	68	-0.00	0.00
18	->	69	-0.00	0.00
18	->	70	-0.00	0.00
18	->	71	0.00	0.00
18	->	72	-0.00	0.00
18	->	73	0.00	0.00
18	->	74	0.00	0.00
18	->	75	0.00	0.00
18	->	76	-0.00	0.00
18	->	77	-0.00	0.00
18	->	78	-0.00	0.00
18	->	79	-0.00	0.00
18	->	80	-0.00	0.00
18	->	81	-0.00	0.00
18	->	82	0.00	0.00
18	->	83	0.00	0.00
18	->	84	0.00	0.00
18	->	85	0.00	0.00
18	->	86	0.00	0.00
18	->	87	0.00	0.00
18	->	88	0.00	0.00
18	->	89	-0.00	0.00
18	->	90	-0.01	0.01
18	->	91	-0.05	0.03
18	->	92	-0.15	0.07
18	->	93	-0.25	0.27
18	->	94	-0.02	0.07

18	->	95	-0.04	0.03
18	->	96	-0.78	1.04
18	->	97	-1.58	1.29
18	->	98	-0.81	1.06
18	->	99	-0.03	0.03
18	->	100	-0.01	0.00
18	->	101	-0.00	0.00
18	->	102	-0.00	0.00
18	->	103	-0.00	0.00
18	->	104	0.00	0.00
18	->	105	0.00	0.00
18	->	106	0.00	0.00
19	->	1	0.12	0.08
19	->	2	0.01	0.02
19	->	3	-0.09	0.07
19	->	4	-0.00	0.02
19	->	5	-0.10	0.02
19	->	6	-0.01	0.00
19	->	7	0.01	0.01
19	->	8	-0.00	0.00
19	->	9	-0.00	0.00
19	->	10	-0.02	0.01
19	->	11	-0.00	0.00
19	->	12	0.09	0.07
19	->	13	0.19	0.08
19	->	14	-1.36	0.47
19	->	15	-0.22	0.29
19	->	16	-4.23	1.16
19	->	17	-1.08	0.24
19	->	18	-10.38	0.63
19	->	19	-28.79	2.47
19	->	20	-5.84	0.45
19	->	21	-0.74	0.16
19	->	22	-2.72	1.65
19	->	23	-2.09	0.54
19	->	24	-0.03	0.05
19	->	25	-0.01	0.04
19	->	26	-0.02	0.01
19	->	27	-0.04	0.01
19	->	28	-0.01	0.00
19	->	29	-0.00	0.00
19	->	30	0.00	0.00
19	->	31	-0.00	0.00
19	->	32	-0.00	0.00
19	->	33	-0.01	0.00
19	->	34	-0.00	0.00
19	->	35	0.00	0.00
19	->	36	-0.00	0.00
19	->	37	0.00	0.00
19	->	38	0.00	0.00

19	->	39	0.00	0.00
19	->	40	-0.00	0.00
19	->	41	0.00	0.00
19	->	42	0.00	0.00
19	->	43	-0.00	0.01
19	->	44	-0.04	0.09
19	->	45	-0.01	0.00
19	->	46	-0.01	0.01
19	->	47	-0.01	0.00
19	->	48	-0.00	0.00
19	->	49	-0.00	0.00
19	->	50	-0.00	0.00
19	->	51	-0.00	0.00
19	->	52	-0.00	0.00
19	->	53	-0.00	0.00
19	->	54	-0.00	0.00
19	->	55	-0.00	0.00
19	->	56	-0.00	0.00
19	->	57	0.00	0.00
19	->	58	0.00	0.00
19	->	59	-0.00	0.00
19	->	60	-0.00	0.00
19	->	61	0.00	0.00
19	->	62	-0.00	0.00
19	->	63	0.00	0.00
19	->	64	0.00	0.00
19	->	65	0.00	0.00
19	->	66	0.00	0.00
19	->	67	0.00	0.00
19	->	68	-0.00	0.00
19	->	69	-0.00	0.00
19	->	70	-0.00	0.00
19	->	71	0.00	0.00
19	->	72	-0.00	0.00
19	->	73	-0.00	0.00
19	->	74	0.00	0.00
19	->	75	0.00	0.00
19	->	76	-0.00	0.00
19	->	77	0.00	0.00
19	->	78	-0.00	0.00
19	->	79	-0.00	0.00
19	->	80	-0.00	0.00
19	->	81	-0.00	0.00
19	->	82	0.00	0.00
19	->	83	0.00	0.00
19	->	84	0.00	0.00
19	->	85	0.00	0.00
19	->	86	0.00	0.00
19	->	87	0.00	0.00
19	->	88	0.00	0.00

19	->	89	-0.01	0.00
19	->	90	-0.09	0.07
19	->	91	-1.16	0.62
19	->	92	-6.06	3.09
19	->	93	-1.42	1.41
19	->	94	-0.04	0.05
19	->	95	-0.01	0.01
19	->	96	-0.03	0.03
19	->	97	-0.07	0.06
19	->	98	-0.13	0.14
19	->	99	-0.00	0.02
19	->	100	-0.01	0.01
19	->	101	-0.02	0.01
19	->	102	-0.01	0.01
19	->	103	-0.00	0.00
19	->	104	-0.00	0.00
19	->	105	-0.00	0.00
19	->	106	0.00	0.00
20	->	1	-0.04	0.02
20	->	2	-0.04	0.01
20	->	3	-1.56	0.50
20	->	4	-1.09	0.28
20	->	5	-0.96	0.25
20	->	6	-0.33	0.26
20	->	7	-0.03	0.01
20	->	8	-0.00	0.00
20	->	9	-0.00	0.00
20	->	10	-0.01	0.01
20	->	11	-0.01	0.00
20	->	12	-0.06	0.02
20	->	13	-0.09	0.02
20	->	14	-2.75	0.47
20	->	15	-0.08	0.04
20	->	16	-1.86	0.37
20	->	17	-2.32	0.35
20	->	18	-1.03	0.17
20	->	19	-5.89	0.45
20	->	20	22.23	1.31
20	->	21	-6.22	0.55
20	->	22	-1.67	0.30
20	->	23	-2.91	0.53
20	->	24	-4.10	0.50
20	->	25	-0.18	0.08
20	->	26	-0.09	0.04
20	->	27	-0.06	0.02
20	->	28	-0.02	0.01
20	->	29	-0.00	0.00
20	->	30	-0.01	0.00
20	->	31	-0.00	0.00
20	->	32	-0.00	0.00

20	->	33	-0.01	0.00
20	->	34	-0.00	0.00
20	->	35	0.00	0.00
20	->	36	-0.00	0.00
20	->	37	0.00	0.00
20	->	38	0.00	0.00
20	->	39	0.00	0.00
20	->	40	-0.00	0.00
20	->	41	0.00	0.00
20	->	42	0.00	0.00
20	->	43	-0.01	0.00
20	->	44	-0.02	0.01
20	->	45	-0.00	0.00
20	->	46	-0.00	0.00
20	->	47	-0.00	0.00
20	->	48	-0.00	0.00
20	->	49	-0.00	0.00
20	->	50	-0.00	0.00
20	->	51	-0.00	0.00
20	->	52	0.00	0.00
20	->	53	-0.00	0.00
20	->	54	0.00	0.00
20	->	55	0.00	0.00
20	->	56	-0.00	0.00
20	->	57	-0.00	0.00
20	->	58	0.00	0.00
20	->	59	-0.00	0.00
20	->	60	0.00	0.00
20	->	61	0.00	0.00
20	->	62	0.00	0.00
20	->	63	0.00	0.00
20	->	64	-0.00	0.00
20	->	65	0.00	0.00
20	->	66	0.00	0.00
20	->	67	0.00	0.00
20	->	68	-0.00	0.00
20	->	69	-0.00	0.00
20	->	70	-0.00	0.00
20	->	71	0.00	0.00
20	->	72	-0.00	0.00
20	->	73	0.00	0.00
20	->	74	0.00	0.00
20	->	75	0.00	0.00
20	->	76	0.00	0.00
20	->	77	-0.00	0.00
20	->	78	-0.00	0.00
20	->	79	0.00	0.00
20	->	80	-0.00	0.00
20	->	81	-0.00	0.00
20	->	82	0.00	0.00

20	->	83	-0.00	0.00
20	->	84	0.00	0.00
20	->	85	0.00	0.00
20	->	86	0.00	0.00
20	->	87	0.00	0.00
20	->	88	-0.00	0.00
20	->	89	-0.00	0.00
20	->	90	0.01	0.01
20	->	91	0.11	0.08
20	->	92	0.15	0.08
20	->	93	-0.01	0.01
20	->	94	-0.01	0.00
20	->	95	-0.00	0.00
20	->	96	-0.05	0.08
20	->	97	-0.03	0.02
20	->	98	-0.01	0.01
20	->	99	-0.00	0.00
20	->	100	-0.00	0.00
20	->	101	-0.00	0.00
20	->	102	-0.00	0.00
20	->	103	-0.00	0.00
20	->	104	0.00	0.00
20	->	105	0.00	0.00
20	->	106	0.00	0.00
21	->	1	0.00	0.00
21	->	2	-0.00	0.00
21	->	3	-0.03	0.01
21	->	4	-0.01	0.01
21	->	5	-0.00	0.01
21	->	6	-0.02	0.01
21	->	7	-0.01	0.00
21	->	8	0.00	0.00
21	->	9	-0.00	0.00
21	->	10	0.00	0.00
21	->	11	-0.00	0.00
21	->	12	-0.00	0.00
21	->	13	-0.01	0.01
21	->	14	-0.07	0.02
21	->	15	-0.01	0.00
21	->	16	-0.23	0.07
21	->	17	-3.41	1.31
21	->	18	-2.51	0.41
21	->	19	-0.75	0.16
21	->	20	-6.23	0.55
21	->	21	-19.99	2.41
21	->	22	-10.45	0.38
21	->	23	-0.87	0.17
21	->	24	-2.84	0.38
21	->	25	-4.09	0.53
21	->	26	-0.13	0.06

21	->	27	-0.06	0.02
21	->	28	-0.03	0.02
21	->	29	-0.01	0.01
21	->	30	-0.07	0.04
21	->	31	-0.00	0.00
21	->	32	-0.02	0.01
21	->	33	-0.03	0.01
21	->	34	-0.00	0.00
21	->	35	-0.00	0.00
21	->	36	0.00	0.00
21	->	37	0.00	0.00
21	->	38	0.00	0.00
21	->	39	0.00	0.00
21	->	40	-0.00	0.00
21	->	41	0.00	0.00
21	->	42	0.00	0.00
21	->	43	-0.05	0.03
21	->	44	0.03	0.02
21	->	45	-0.01	0.01
21	->	46	-0.02	0.01
21	->	47	-0.01	0.00
21	->	48	-0.00	0.00
21	->	49	-0.00	0.00
21	->	50	-0.00	0.00
21	->	51	-0.00	0.00
21	->	52	-0.00	0.00
21	->	53	0.00	0.00
21	->	54	0.00	0.00
21	->	55	0.00	0.00
21	->	56	0.00	0.00
21	->	57	0.00	0.00
21	->	58	-0.00	0.00
21	->	59	0.00	0.00
21	->	60	0.00	0.00
21	->	61	0.00	0.00
21	->	62	-0.00	0.00
21	->	63	-0.00	0.00
21	->	64	-0.00	0.00
21	->	65	-0.00	0.00
21	->	66	-0.00	0.00
21	->	67	0.00	0.00
21	->	68	0.00	0.00
21	->	69	0.00	0.00
21	->	70	0.00	0.00
21	->	71	-0.00	0.00
21	->	72	0.00	0.00
21	->	73	0.00	0.00
21	->	74	-0.00	0.00
21	->	75	-0.00	0.00
21	->	76	0.00	0.00

21	->	77	0.00	0.00
21	->	78	0.00	0.00
21	->	79	0.00	0.00
21	->	80	0.00	0.00
21	->	81	0.00	0.00
21	->	82	0.00	0.00
21	->	83	0.00	0.00
21	->	84	-0.00	0.00
21	->	85	-0.00	0.00
21	->	86	-0.00	0.00
21	->	87	0.00	0.00
21	->	88	-0.00	0.00
21	->	89	-0.01	0.00
21	->	90	-0.03	0.01
21	->	91	-0.11	0.05
21	->	92	-0.08	0.05
21	->	93	-0.01	0.00
21	->	94	-0.01	0.00
21	->	95	-0.00	0.00
21	->	96	-0.58	1.06
21	->	97	-0.26	0.36
21	->	98	-0.02	0.01
21	->	99	-0.01	0.01
21	->	100	-0.00	0.00
21	->	101	-0.00	0.00
21	->	102	-0.00	0.00
21	->	103	-0.00	0.00
21	->	104	-0.00	0.00
21	->	105	0.00	0.00
21	->	106	0.00	0.00
22	->	1	0.14	0.07
22	->	2	0.00	0.00
22	->	3	-0.01	0.02
22	->	4	0.01	0.01
22	->	5	-0.21	0.06
22	->	6	-0.01	0.00
22	->	7	0.03	0.02
22	->	8	-0.02	0.00
22	->	9	-0.00	0.00
22	->	10	-0.11	0.03
22	->	11	-0.03	0.01
22	->	12	0.45	0.28
22	->	13	0.13	0.06
22	->	14	-0.61	0.30
22	->	15	0.01	0.13
22	->	16	-0.35	0.12
22	->	17	-0.14	0.07
22	->	18	-2.49	0.49
22	->	19	-2.75	1.66
22	->	20	-1.68	0.29

22	->	21	-10.46	0.38
22	->	22	-93.41	5.92
22	->	23	-19.32	0.71
22	->	24	-0.77	0.18
22	->	25	-2.31	0.46
22	->	26	-2.05	0.96
22	->	27	-0.17	0.07
22	->	28	-0.02	0.02
22	->	29	-0.00	0.01
22	->	30	-0.05	0.02
22	->	31	0.05	0.02
22	->	32	-0.03	0.02
22	->	33	-0.06	0.04
22	->	34	-0.00	0.00
22	->	35	-0.02	0.00
22	->	36	0.01	0.00
22	->	37	-0.00	0.00
22	->	38	-0.01	0.00
22	->	39	-0.00	0.00
22	->	40	0.02	0.00
22	->	41	0.01	0.00
22	->	42	0.02	0.01
22	->	43	-0.55	0.58
22	->	44	-0.70	0.89
22	->	45	-0.06	0.05
22	->	46	-0.21	0.09
22	->	47	-0.11	0.07
22	->	48	-0.01	0.00
22	->	49	0.00	0.00
22	->	50	0.00	0.00
22	->	51	0.00	0.00
22	->	52	0.00	0.00
22	->	53	0.02	0.00
22	->	54	0.00	0.00
22	->	55	0.00	0.00
22	->	56	0.00	0.00
22	->	57	0.00	0.00
22	->	58	-0.01	0.00
22	->	59	0.01	0.00
22	->	60	0.00	0.00
22	->	61	0.00	0.00
22	->	62	-0.00	0.00
22	->	63	-0.01	0.00
22	->	64	-0.00	0.00
22	->	65	-0.00	0.00
22	->	66	-0.00	0.00
22	->	67	-0.00	0.00
22	->	68	0.01	0.00
22	->	69	0.01	0.00
22	->	70	0.00	0.00

22	->	71	-0.00	0.00
22	->	72	0.03	0.01
22	->	73	0.00	0.00
22	->	74	-0.03	0.00
22	->	75	-0.01	0.00
22	->	76	0.01	0.00
22	->	77	0.02	0.00
22	->	78	0.02	0.00
22	->	79	0.00	0.00
22	->	80	0.00	0.00
22	->	81	0.01	0.00
22	->	82	0.00	0.00
22	->	83	0.00	0.00
22	->	84	-0.00	0.00
22	->	85	-0.00	0.00
22	->	86	-0.01	0.00
22	->	87	-0.01	0.00
22	->	88	-0.03	0.01
22	->	89	-0.19	0.08
22	->	90	-2.71	1.22
22	->	91	-15.03	6.75
22	->	92	-10.65	5.89
22	->	93	-0.07	0.07
22	->	94	-0.06	0.01
22	->	95	-0.02	0.00
22	->	96	-0.03	0.03
22	->	97	-0.09	0.06
22	->	98	-0.05	0.04
22	->	99	-0.09	0.05
22	->	100	-0.01	0.02
22	->	101	-0.05	0.02
22	->	102	-0.07	0.02
22	->	103	-0.04	0.01
22	->	104	-0.03	0.01
22	->	105	-0.02	0.01
22	->	106	-0.01	0.00
23	->	1	0.00	0.00
23	->	2	-0.00	0.00
23	->	3	-0.02	0.01
23	->	4	-0.08	0.03
23	->	5	1.76	0.71
23	->	6	-0.12	0.06
23	->	7	-0.39	0.16
23	->	8	0.01	0.01
23	->	9	-0.03	0.03
23	->	10	1.88	0.69
23	->	11	0.00	0.02
23	->	12	-0.35	0.39
23	->	13	-0.04	0.02
23	->	14	-2.34	0.38

23	->	15	-0.01	0.00
23	->	16	-0.05	0.02
23	->	17	-0.05	0.03
23	->	18	-0.10	0.06
23	->	19	-2.11	0.54
23	->	20	-2.92	0.52
23	->	21	-0.88	0.17
23	->	22	-19.35	0.70
23	->	23	11.44	1.47
23	->	24	-10.29	0.48
23	->	25	-1.34	0.22
23	->	26	-3.42	0.89
23	->	27	22.45	12.57
23	->	28	-0.23	0.06
23	->	29	-0.04	0.02
23	->	30	-0.03	0.01
23	->	31	-0.01	0.00
23	->	32	-0.01	0.00
23	->	33	-0.00	0.00
23	->	34	-0.00	0.00
23	->	35	-0.00	0.00
23	->	36	0.00	0.00
23	->	37	0.00	0.00
23	->	38	-0.00	0.00
23	->	39	0.00	0.00
23	->	40	-0.00	0.00
23	->	41	-0.00	0.00
23	->	42	-0.01	0.00
23	->	43	-0.02	0.01
23	->	44	-0.05	0.02
23	->	45	0.00	0.00
23	->	46	-0.00	0.00
23	->	47	-0.00	0.00
23	->	48	-0.00	0.00
23	->	49	0.00	0.00
23	->	50	0.00	0.00
23	->	51	0.00	0.00
23	->	52	0.00	0.00
23	->	53	0.00	0.00
23	->	54	0.00	0.00
23	->	55	0.00	0.00
23	->	56	0.00	0.00
23	->	57	0.00	0.00
23	->	58	-0.00	0.00
23	->	59	0.00	0.00
23	->	60	0.00	0.00
23	->	61	0.00	0.00
23	->	62	-0.00	0.00
23	->	63	-0.00	0.00
23	->	64	-0.00	0.00

23	->	65	0.00	0.00
23	->	66	-0.00	0.00
23	->	67	0.00	0.00
23	->	68	0.00	0.00
23	->	69	0.00	0.00
23	->	70	0.00	0.00
23	->	71	-0.00	0.00
23	->	72	0.00	0.00
23	->	73	0.00	0.00
23	->	74	-0.00	0.00
23	->	75	-0.00	0.00
23	->	76	0.00	0.00
23	->	77	0.00	0.00
23	->	78	0.00	0.00
23	->	79	0.00	0.00
23	->	80	0.00	0.00
23	->	81	0.00	0.00
23	->	82	0.00	0.00
23	->	83	0.00	0.00
23	->	84	-0.00	0.00
23	->	85	-0.00	0.00
23	->	86	-0.00	0.00
23	->	87	-0.00	0.00
23	->	88	-0.00	0.00
23	->	89	-0.02	0.01
23	->	90	-0.73	0.47
23	->	91	-5.12	3.02
23	->	92	-0.19	0.14
23	->	93	-0.01	0.00
23	->	94	-0.01	0.00
23	->	95	-0.00	0.00
23	->	96	-0.00	0.00
23	->	97	-0.00	0.00
23	->	98	-0.00	0.00
23	->	99	-0.01	0.00
23	->	100	-0.00	0.00
23	->	101	-0.00	0.00
23	->	102	-0.01	0.00
23	->	103	-0.01	0.00
23	->	104	-0.00	0.00
23	->	105	-0.00	0.00
23	->	106	-0.00	0.00
24	->	1	0.00	0.00
24	->	2	-0.00	0.00
24	->	3	-0.02	0.01
24	->	4	-0.06	0.03
24	->	5	-0.58	0.40
24	->	6	-1.18	0.36
24	->	7	-0.09	0.06
24	->	8	-0.01	0.00

24	->	9	-0.00	0.00
24	->	10	0.03	0.01
24	->	11	-0.00	0.00
24	->	12	-0.02	0.01
24	->	13	-0.01	0.01
24	->	14	-0.08	0.04
24	->	15	-0.00	0.00
24	->	16	-0.02	0.00
24	->	17	-0.07	0.02
24	->	18	-0.05	0.03
24	->	19	-0.03	0.05
24	->	20	-4.08	0.49
24	->	21	-2.86	0.38
24	->	22	-0.77	0.18
24	->	23	-10.34	0.48
24	->	24	24.02	1.61
24	->	25	-10.18	0.37
24	->	26	-1.07	0.21
24	->	27	-2.81	0.44
24	->	28	-5.10	1.05
24	->	29	-0.16	0.10
24	->	30	-0.43	0.26
24	->	31	-0.01	0.01
24	->	32	-0.01	0.00
24	->	33	-0.01	0.00
24	->	34	-0.00	0.00
24	->	35	0.00	0.00
24	->	36	-0.00	0.00
24	->	37	-0.00	0.00
24	->	38	0.00	0.00
24	->	39	0.00	0.00
24	->	40	-0.00	0.00
24	->	41	0.00	0.00
24	->	42	0.00	0.00
24	->	43	-0.01	0.01
24	->	44	-0.01	0.01
24	->	45	-0.00	0.00
24	->	46	-0.00	0.00
24	->	47	-0.00	0.00
24	->	48	-0.00	0.00
24	->	49	0.00	0.00
24	->	50	0.00	0.00
24	->	51	0.00	0.00
24	->	52	0.00	0.00
24	->	53	0.00	0.00
24	->	54	0.00	0.00
24	->	55	0.00	0.00
24	->	56	0.00	0.00
24	->	57	0.00	0.00
24	->	58	-0.00	0.00

24	->	59	0.00	0.00
24	->	60	0.00	0.00
24	->	61	0.00	0.00
24	->	62	0.00	0.00
24	->	63	-0.00	0.00
24	->	64	-0.00	0.00
24	->	65	0.00	0.00
24	->	66	-0.00	0.00
24	->	67	0.00	0.00
24	->	68	0.00	0.00
24	->	69	0.00	0.00
24	->	70	0.00	0.00
24	->	71	-0.00	0.00
24	->	72	0.00	0.00
24	->	73	0.00	0.00
24	->	74	-0.00	0.00
24	->	75	-0.00	0.00
24	->	76	0.00	0.00
24	->	77	0.00	0.00
24	->	78	-0.00	0.00
24	->	79	0.00	0.00
24	->	80	0.00	0.00
24	->	81	0.00	0.00
24	->	82	0.00	0.00
24	->	83	0.00	0.00
24	->	84	-0.00	0.00
24	->	85	0.00	0.00
24	->	86	0.00	0.00
24	->	87	0.00	0.00
24	->	88	-0.00	0.00
24	->	89	-0.00	0.00
24	->	90	0.01	0.02
24	->	91	0.10	0.07
24	->	92	0.00	0.01
24	->	93	-0.00	0.00
24	->	94	-0.00	0.00
24	->	95	-0.00	0.00
24	->	96	-0.01	0.01
24	->	97	-0.01	0.01
24	->	98	-0.00	0.00
24	->	99	-0.00	0.00
24	->	100	-0.00	0.00
24	->	101	-0.00	0.00
24	->	102	-0.00	0.00
24	->	103	-0.00	0.00
24	->	104	0.00	0.00
24	->	105	0.00	0.00
24	->	106	0.00	0.00
25	->	1	0.04	0.01
25	->	2	-0.00	0.00

25	->	3	-0.00	0.00
25	->	4	-0.01	0.00
25	->	5	-0.06	0.01
25	->	6	-0.01	0.01
25	->	7	-0.00	0.00
25	->	8	-0.01	0.00
25	->	9	-0.00	0.00
25	->	10	-0.04	0.00
25	->	11	-0.01	0.00
25	->	12	0.03	0.01
25	->	13	0.02	0.00
25	->	14	-0.02	0.01
25	->	15	-0.00	0.00
25	->	16	-0.07	0.01
25	->	17	-0.03	0.01
25	->	18	-0.13	0.11
25	->	19	-0.01	0.04
25	->	20	-0.18	0.08
25	->	21	-4.13	0.55
25	->	22	-2.34	0.45
25	->	23	-1.35	0.22
25	->	24	-10.21	0.38
25	->	25	-104.35	3.62
25	->	26	-12.52	0.53
25	->	27	-0.69	0.17
25	->	28	-1.51	0.33
25	->	29	-1.22	0.48
25	->	30	-4.66	0.75
25	->	31	0.01	0.12
25	->	32	-1.64	0.26
25	->	33	-0.92	0.41
25	->	34	-0.01	0.00
25	->	35	-0.03	0.00
25	->	36	0.02	0.00
25	->	37	-0.00	0.00
25	->	38	-0.02	0.00
25	->	39	-0.00	0.00
25	->	40	0.02	0.01
25	->	41	0.03	0.01
25	->	42	0.01	0.02
25	->	43	-1.55	1.53
25	->	44	-0.15	0.25
25	->	45	-0.09	0.07
25	->	46	-0.10	0.05
25	->	47	-0.03	0.02
25	->	48	-0.01	0.00
25	->	49	0.00	0.00
25	->	50	0.00	0.00
25	->	51	0.00	0.00
25	->	52	0.00	0.00

25	->	53	0.02	0.00
25	->	54	0.00	0.00
25	->	55	-0.00	0.00
25	->	56	0.00	0.00
25	->	57	0.00	0.00
25	->	58	-0.01	0.00
25	->	59	0.01	0.00
25	->	60	0.00	0.00
25	->	61	0.00	0.00
25	->	62	-0.00	0.00
25	->	63	-0.00	0.00
25	->	64	-0.00	0.00
25	->	65	-0.00	0.00
25	->	66	-0.00	0.00
25	->	67	-0.00	0.00
25	->	68	0.00	0.00
25	->	69	0.00	0.00
25	->	70	0.00	0.00
25	->	71	-0.00	0.00
25	->	72	0.02	0.00
25	->	73	0.00	0.00
25	->	74	-0.01	0.00
25	->	75	-0.01	0.00
25	->	76	0.01	0.00
25	->	77	0.01	0.00
25	->	78	0.01	0.00
25	->	79	0.00	0.00
25	->	80	0.00	0.00
25	->	81	0.00	0.00
25	->	82	0.00	0.00
25	->	83	0.00	0.00
25	->	84	-0.00	0.00
25	->	85	-0.00	0.00
25	->	86	-0.01	0.00
25	->	87	-0.01	0.00
25	->	88	-0.03	0.01
25	->	89	-0.11	0.03
25	->	90	-0.33	0.12
25	->	91	-0.27	0.11
25	->	92	-0.08	0.02
25	->	93	-0.02	0.00
25	->	94	-0.02	0.01
25	->	95	-0.01	0.00
25	->	96	-0.55	0.83
25	->	97	-3.36	4.73
25	->	98	-0.08	0.04
25	->	99	-0.05	0.02
25	->	100	-0.01	0.00
25	->	101	-0.01	0.00
25	->	102	-0.01	0.00

25	->	103	-0.01	0.00
25	->	104	-0.01	0.00
25	->	105	-0.01	0.00
25	->	106	-0.01	0.00
26	->	1	0.00	0.00
26	->	2	-0.00	0.00
26	->	3	-0.00	0.00
26	->	4	-0.00	0.00
26	->	5	-0.07	0.03
26	->	6	-0.01	0.00
26	->	7	0.00	0.01
26	->	8	-0.00	0.00
26	->	9	-0.00	0.00
26	->	10	-0.05	0.02
26	->	11	-0.00	0.00
26	->	12	-0.02	0.03
26	->	13	-0.00	0.00
26	->	14	-0.04	0.01
26	->	15	-0.00	0.00
26	->	16	-0.01	0.00
26	->	17	-0.00	0.00
26	->	18	-0.01	0.00
26	->	19	-0.02	0.01
26	->	20	-0.09	0.04
26	->	21	-0.13	0.06
26	->	22	-2.07	0.94
26	->	23	-3.43	0.89
26	->	24	-1.08	0.21
26	->	25	-12.56	0.53
26	->	26	19.35	1.64
26	->	27	-21.77	0.45
26	->	28	-0.65	0.14
26	->	29	-1.19	0.46
26	->	30	-0.38	0.15
26	->	31	-0.16	0.10
26	->	32	-0.03	0.01
26	->	33	-0.00	0.00
26	->	34	-0.00	0.00
26	->	35	-0.00	0.00
26	->	36	0.00	0.00
26	->	37	0.00	0.00
26	->	38	0.00	0.00
26	->	39	-0.00	0.00
26	->	40	-0.00	0.00
26	->	41	-0.02	0.00
26	->	42	-0.02	0.01
26	->	43	-0.42	0.31
26	->	44	-0.05	0.02
26	->	45	0.00	0.00
26	->	46	-0.00	0.00

26	->	47	-0.01	0.01
26	->	48	-0.00	0.00
26	->	49	-0.00	0.00
26	->	50	-0.00	0.00
26	->	51	-0.00	0.00
26	->	52	0.00	0.00
26	->	53	0.00	0.00
26	->	54	0.00	0.00
26	->	55	0.00	0.00
26	->	56	0.00	0.00
26	->	57	0.00	0.00
26	->	58	-0.00	0.00
26	->	59	0.00	0.00
26	->	60	0.00	0.00
26	->	61	0.00	0.00
26	->	62	0.00	0.00
26	->	63	-0.00	0.00
26	->	64	-0.00	0.00
26	->	65	0.00	0.00
26	->	66	-0.00	0.00
26	->	67	0.00	0.00
26	->	68	0.00	0.00
26	->	69	0.00	0.00
26	->	70	0.00	0.00
26	->	71	0.00	0.00
26	->	72	0.00	0.00
26	->	73	0.00	0.00
26	->	74	-0.00	0.00
26	->	75	-0.00	0.00
26	->	76	0.00	0.00
26	->	77	0.00	0.00
26	->	78	0.00	0.00
26	->	79	0.00	0.00
26	->	80	0.00	0.00
26	->	81	0.00	0.00
26	->	82	0.00	0.00
26	->	83	0.00	0.00
26	->	84	-0.00	0.00
26	->	85	-0.00	0.00
26	->	86	-0.00	0.00
26	->	87	-0.00	0.00
26	->	88	-0.01	0.00
26	->	89	-0.14	0.07
26	->	90	-2.64	0.77
26	->	91	-0.89	0.39
26	->	92	-0.02	0.01
26	->	93	-0.00	0.00
26	->	94	-0.00	0.00
26	->	95	-0.00	0.00
26	->	96	-0.00	0.00

26 ->	97	-0.00	0.00
26 ->	98	-0.00	0.00
26 ->	99	-0.00	0.00
26 ->	100	-0.00	0.00
26 ->	101	-0.00	0.00
26 ->	102	-0.00	0.00
26 ->	103	-0.00	0.00
26 ->	104	-0.00	0.00
26 ->	105	-0.00	0.00
26 ->	106	-0.00	0.00
27 ->	1	-0.01	0.00
27 ->	2	-0.00	0.00
27 ->	3	-0.01	0.00
27 ->	4	-0.07	0.03
27 ->	5	2.70	1.05
27 ->	6	-0.36	0.24
27 ->	7	-2.30	1.18
27 ->	8	0.02	0.02
27 ->	9	-0.08	0.06
27 ->	10	2.37	0.95
27 ->	11	0.02	0.01
27 ->	12	-0.07	0.05
27 ->	13	-0.02	0.01
27 ->	14	-0.00	0.02
27 ->	15	0.00	0.00
27 ->	16	-0.00	0.00
27 ->	17	-0.00	0.00
27 ->	18	-0.01	0.00
27 ->	19	-0.04	0.01
27 ->	20	-0.06	0.02
27 ->	21	-0.06	0.02
27 ->	22	-0.17	0.07
27 ->	23	22.45	12.58
27 ->	24	-2.79	0.43
27 ->	25	-0.69	0.17
27 ->	26	-21.86	0.45
27 ->	27	3.61	1.77
27 ->	28	-4.95	0.40
27 ->	29	-0.53	0.10
27 ->	30	-0.04	0.03
27 ->	31	-0.02	0.01
27 ->	32	-0.00	0.00
27 ->	33	-0.00	0.00
27 ->	34	-0.00	0.00
27 ->	35	-0.00	0.00
27 ->	36	0.00	0.00
27 ->	37	-0.00	0.00
27 ->	38	-0.00	0.00
27 ->	39	-0.00	0.00
27 ->	40	-0.00	0.00

27	->	41	-0.00	0.00
27	->	42	-0.01	0.00
27	->	43	-0.01	0.01
27	->	44	-0.00	0.00
27	->	45	0.00	0.00
27	->	46	-0.00	0.00
27	->	47	-0.00	0.00
27	->	48	0.00	0.00
27	->	49	0.00	0.00
27	->	50	0.00	0.00
27	->	51	0.00	0.00
27	->	52	0.00	0.00
27	->	53	0.00	0.00
27	->	54	0.00	0.00
27	->	55	0.00	0.00
27	->	56	0.00	0.00
27	->	57	0.00	0.00
27	->	58	-0.00	0.00
27	->	59	0.00	0.00
27	->	60	0.00	0.00
27	->	61	0.00	0.00
27	->	62	0.00	0.00
27	->	63	-0.00	0.00
27	->	64	-0.00	0.00
27	->	65	0.00	0.00
27	->	66	-0.00	0.00
27	->	67	0.00	0.00
27	->	68	0.00	0.00
27	->	69	0.00	0.00
27	->	70	0.00	0.00
27	->	71	0.00	0.00
27	->	72	0.00	0.00
27	->	73	0.00	0.00
27	->	74	-0.00	0.00
27	->	75	-0.00	0.00
27	->	76	0.00	0.00
27	->	77	0.00	0.00
27	->	78	0.00	0.00
27	->	79	0.00	0.00
27	->	80	0.00	0.00
27	->	81	0.00	0.00
27	->	82	0.00	0.00
27	->	83	0.00	0.00
27	->	84	-0.00	0.00
27	->	85	-0.00	0.00
27	->	86	-0.00	0.00
27	->	87	-0.00	0.00
27	->	88	-0.00	0.00
27	->	89	0.01	0.01
27	->	90	0.04	0.03

27	->	91	0.12	0.09
27	->	92	0.01	0.01
27	->	93	-0.00	0.00
27	->	94	-0.00	0.00
27	->	95	-0.00	0.00
27	->	96	-0.00	0.00
27	->	97	-0.00	0.00
27	->	98	-0.00	0.00
27	->	99	-0.00	0.00
27	->	100	-0.00	0.00
27	->	101	-0.00	0.00
27	->	102	-0.00	0.00
27	->	103	-0.00	0.00
27	->	104	-0.00	0.00
27	->	105	-0.00	0.00
27	->	106	-0.00	0.00
28	->	1	0.00	0.00
28	->	2	-0.00	0.00
28	->	3	-0.00	0.00
28	->	4	-0.00	0.00
28	->	5	-0.02	0.01
28	->	6	-0.07	0.08
28	->	7	-0.04	0.02
28	->	8	-0.00	0.00
28	->	9	-0.00	0.00
28	->	10	-0.01	0.00
28	->	11	-0.00	0.00
28	->	12	0.00	0.00
28	->	13	0.00	0.00
28	->	14	-0.00	0.00
28	->	15	0.00	0.00
28	->	16	-0.00	0.00
28	->	17	-0.00	0.00
28	->	18	-0.00	0.00
28	->	19	-0.01	0.00
28	->	20	-0.02	0.01
28	->	21	-0.03	0.02
28	->	22	-0.02	0.02
28	->	23	-0.23	0.06
28	->	24	-5.12	1.05
28	->	25	-1.52	0.33
28	->	26	-0.67	0.13
28	->	27	-5.11	0.40
28	->	28	-22.76	1.78
28	->	29	-11.57	0.50
28	->	30	-3.64	1.25
28	->	31	-0.03	0.01
28	->	32	-0.02	0.00
28	->	33	-0.00	0.00
28	->	34	-0.00	0.00

28	->	35	0.00	0.00
28	->	36	-0.00	0.00
28	->	37	0.00	0.00
28	->	38	0.00	0.00
28	->	39	0.00	0.00
28	->	40	-0.00	0.00
28	->	41	-0.00	0.00
28	->	42	-0.00	0.00
28	->	43	-0.02	0.01
28	->	44	-0.00	0.00
28	->	45	-0.00	0.00
28	->	46	-0.00	0.00
28	->	47	-0.00	0.00
28	->	48	-0.00	0.00
28	->	49	0.00	0.00
28	->	50	0.00	0.00
28	->	51	0.00	0.00
28	->	52	-0.00	0.00
28	->	53	0.00	0.00
28	->	54	0.00	0.00
28	->	55	0.00	0.00
28	->	56	0.00	0.00
28	->	57	0.00	0.00
28	->	58	-0.00	0.00
28	->	59	0.00	0.00
28	->	60	0.00	0.00
28	->	61	0.00	0.00
28	->	62	-0.00	0.00
28	->	63	-0.00	0.00
28	->	64	-0.00	0.00
28	->	65	0.00	0.00
28	->	66	-0.00	0.00
28	->	67	0.00	0.00
28	->	68	0.00	0.00
28	->	69	0.00	0.00
28	->	70	0.00	0.00
28	->	71	0.00	0.00
28	->	72	0.00	0.00
28	->	73	0.00	0.00
28	->	74	-0.00	0.00
28	->	75	-0.00	0.00
28	->	76	0.00	0.00
28	->	77	0.00	0.00
28	->	78	0.00	0.00
28	->	79	0.00	0.00
28	->	80	0.00	0.00
28	->	81	0.00	0.00
28	->	82	0.00	0.00
28	->	83	0.00	0.00
28	->	84	-0.00	0.00

28	->	85	-0.00	0.00
28	->	86	0.00	0.00
28	->	87	-0.00	0.00
28	->	88	-0.00	0.00
28	->	89	-0.00	0.00
28	->	90	-0.02	0.01
28	->	91	-0.01	0.01
28	->	92	-0.00	0.00
28	->	93	-0.00	0.00
28	->	94	-0.00	0.00
28	->	95	-0.00	0.00
28	->	96	-0.00	0.00
28	->	97	-0.00	0.00
28	->	98	-0.00	0.00
28	->	99	-0.00	0.00
28	->	100	-0.00	0.00
28	->	101	-0.00	0.00
28	->	102	-0.00	0.00
28	->	103	-0.00	0.00
28	->	104	-0.00	0.00
28	->	105	-0.00	0.00
28	->	106	-0.00	0.00
29	->	1	0.00	0.00
29	->	2	0.00	0.00
29	->	3	0.00	0.00
29	->	4	0.00	0.00
29	->	5	-0.00	0.00
29	->	6	-0.00	0.00
29	->	7	-0.00	0.00
29	->	8	-0.00	0.00
29	->	9	-0.00	0.00
29	->	10	-0.00	0.00
29	->	11	-0.00	0.00
29	->	12	0.00	0.00
29	->	13	0.00	0.00
29	->	14	-0.00	0.00
29	->	15	0.00	0.00
29	->	16	-0.00	0.00
29	->	17	-0.00	0.00
29	->	18	-0.00	0.00
29	->	19	-0.00	0.00
29	->	20	-0.00	0.00
29	->	21	-0.01	0.01
29	->	22	-0.00	0.01
29	->	23	-0.04	0.02
29	->	24	-0.16	0.10
29	->	25	-1.23	0.48
29	->	26	-1.23	0.47
29	->	27	-0.54	0.10
29	->	28	-11.63	0.51

29	->	29	12.03	0.69
29	->	30	-14.46	0.56
29	->	31	-0.72	0.34
29	->	32	-0.03	0.00
29	->	33	-0.01	0.00
29	->	34	-0.00	0.00
29	->	35	0.00	0.00
29	->	36	-0.00	0.00
29	->	37	0.00	0.00
29	->	38	0.00	0.00
29	->	39	-0.00	0.00
29	->	40	-0.00	0.00
29	->	41	0.00	0.01
29	->	42	0.00	0.01
29	->	43	-0.03	0.04
29	->	44	-0.01	0.00
29	->	45	-0.00	0.00
29	->	46	-0.00	0.00
29	->	47	-0.00	0.00
29	->	48	-0.00	0.00
29	->	49	0.00	0.00
29	->	50	0.00	0.00
29	->	51	0.00	0.00
29	->	52	0.00	0.00
29	->	53	0.00	0.00
29	->	54	0.00	0.00
29	->	55	0.00	0.00
29	->	56	0.00	0.00
29	->	57	0.00	0.00
29	->	58	-0.00	0.00
29	->	59	0.00	0.00
29	->	60	0.00	0.00
29	->	61	0.00	0.00
29	->	62	0.00	0.00
29	->	63	-0.00	0.00
29	->	64	-0.00	0.00
29	->	65	0.00	0.00
29	->	66	-0.00	0.00
29	->	67	0.00	0.00
29	->	68	0.00	0.00
29	->	69	0.00	0.00
29	->	70	0.00	0.00
29	->	71	0.00	0.00
29	->	72	0.00	0.00
29	->	73	0.00	0.00
29	->	74	-0.00	0.00
29	->	75	-0.00	0.00
29	->	76	0.00	0.00
29	->	77	0.00	0.00
29	->	78	0.00	0.00

29	->	79	0.00	0.00
29	->	80	0.00	0.00
29	->	81	0.00	0.00
29	->	82	0.00	0.00
29	->	83	0.00	0.00
29	->	84	-0.00	0.00
29	->	85	0.00	0.00
29	->	86	-0.00	0.00
29	->	87	-0.00	0.00
29	->	88	-0.00	0.00
29	->	89	-0.01	0.01
29	->	90	-0.03	0.01
29	->	91	-0.01	0.00
29	->	92	-0.00	0.00
29	->	93	-0.00	0.00
29	->	94	-0.00	0.00
29	->	95	-0.00	0.00
29	->	96	-0.00	0.00
29	->	97	-0.00	0.00
29	->	98	-0.00	0.00
29	->	99	-0.00	0.00
29	->	100	-0.00	0.00
29	->	101	-0.00	0.00
29	->	102	-0.00	0.00
29	->	103	-0.00	0.00
29	->	104	-0.00	0.00
29	->	105	-0.00	0.00
29	->	106	-0.00	0.00
30	->	1	-0.00	0.00
30	->	2	-0.00	0.00
30	->	3	-0.00	0.00
30	->	4	-0.00	0.00
30	->	5	-0.00	0.00
30	->	6	-0.00	0.00
30	->	7	-0.00	0.00
30	->	8	-0.00	0.00
30	->	9	-0.00	0.00
30	->	10	-0.00	0.00
30	->	11	0.00	0.00
30	->	12	-0.00	0.00
30	->	13	-0.00	0.00
30	->	14	-0.00	0.00
30	->	15	-0.00	0.00
30	->	16	0.00	0.00
30	->	17	-0.00	0.00
30	->	18	-0.00	0.00
30	->	19	0.00	0.00
30	->	20	-0.01	0.00
30	->	21	-0.07	0.04
30	->	22	-0.05	0.02

30	->	23	-0.03	0.01
30	->	24	-0.43	0.25
30	->	25	-4.62	0.74
30	->	26	-0.38	0.15
30	->	27	-0.04	0.03
30	->	28	-3.65	1.25
30	->	29	-14.63	0.55
30	->	30	-53.60	2.13
30	->	31	-11.18	0.53
30	->	32	-1.32	0.33
30	->	33	-0.10	0.02
30	->	34	-0.01	0.00
30	->	35	-0.00	0.00
30	->	36	-0.00	0.00
30	->	37	0.00	0.00
30	->	38	0.00	0.00
30	->	39	-0.00	0.00
30	->	40	0.01	0.00
30	->	41	0.05	0.04
30	->	42	-0.18	0.06
30	->	43	-0.67	0.80
30	->	44	-0.01	0.01
30	->	45	-0.01	0.00
30	->	46	0.00	0.00
30	->	47	-0.01	0.00
30	->	48	-0.00	0.00
30	->	49	-0.00	0.00
30	->	50	-0.00	0.00
30	->	51	-0.00	0.00
30	->	52	-0.00	0.00
30	->	53	-0.00	0.00
30	->	54	-0.00	0.00
30	->	55	-0.00	0.00
30	->	56	-0.00	0.00
30	->	57	-0.00	0.00
30	->	58	0.00	0.00
30	->	59	-0.00	0.00
30	->	60	-0.00	0.00
30	->	61	-0.00	0.00
30	->	62	0.00	0.00
30	->	63	0.00	0.00
30	->	64	0.00	0.00
30	->	65	0.00	0.00
30	->	66	0.00	0.00
30	->	67	0.00	0.00
30	->	68	-0.00	0.00
30	->	69	-0.00	0.00
30	->	70	-0.00	0.00
30	->	71	0.00	0.00
30	->	72	-0.00	0.00

30	->	73	-0.00	0.00
30	->	74	0.00	0.00
30	->	75	0.00	0.00
30	->	76	-0.00	0.00
30	->	77	-0.00	0.00
30	->	78	-0.00	0.00
30	->	79	-0.00	0.00
30	->	80	-0.00	0.00
30	->	81	-0.00	0.00
30	->	82	0.00	0.00
30	->	83	-0.00	0.00
30	->	84	0.00	0.00
30	->	85	0.00	0.00
30	->	86	0.00	0.00
30	->	87	0.00	0.00
30	->	88	-0.00	0.00
30	->	89	-0.01	0.01
30	->	90	-0.00	0.01
30	->	91	-0.00	0.00
30	->	92	0.00	0.00
30	->	93	0.00	0.00
30	->	94	0.00	0.00
30	->	95	0.00	0.00
30	->	96	-0.00	0.00
30	->	97	-0.00	0.01
30	->	98	0.00	0.00
30	->	99	0.00	0.00
30	->	100	0.00	0.00
30	->	101	0.00	0.00
30	->	102	0.00	0.00
30	->	103	0.00	0.00
30	->	104	0.00	0.00
30	->	105	-0.00	0.00
30	->	106	0.00	0.00
31	->	1	0.02	0.00
31	->	2	0.00	0.00
31	->	3	-0.00	0.00
31	->	4	0.00	0.00
31	->	5	-0.02	0.00
31	->	6	0.00	0.00
31	->	7	-0.00	0.00
31	->	8	-0.01	0.00
31	->	9	0.00	0.00
31	->	10	-0.02	0.00
31	->	11	-0.01	0.00
31	->	12	0.02	0.01
31	->	13	0.01	0.00
31	->	14	0.00	0.00
31	->	15	0.00	0.00
31	->	16	-0.02	0.00

31 ->	17	-0.00	0.00
31 ->	18	-0.00	0.00
31 ->	19	-0.00	0.00
31 ->	20	-0.00	0.00
31 ->	21	-0.00	0.00
31 ->	22	0.05	0.02
31 ->	23	-0.01	0.00
31 ->	24	-0.01	0.01
31 ->	25	0.01	0.12
31 ->	26	-0.16	0.10
31 ->	27	-0.02	0.01
31 ->	28	-0.03	0.01
31 ->	29	-0.75	0.37
31 ->	30	-11.24	0.54
31 ->	31	12.97	4.36
31 ->	32	-0.25	0.36
31 ->	33	-0.84	0.31
31 ->	34	-0.14	0.05
31 ->	35	-0.08	0.01
31 ->	36	0.01	0.00
31 ->	37	-0.00	0.00
31 ->	38	-0.05	0.01
31 ->	39	-0.01	0.00
31 ->	40	0.23	0.24
31 ->	41	-2.49	0.92
31 ->	42	-3.09	0.41
31 ->	43	-3.04	1.26
31 ->	44	-0.07	0.05
31 ->	45	-0.08	0.02
31 ->	46	-0.07	0.01
31 ->	47	-0.08	0.09
31 ->	48	-0.04	0.02
31 ->	49	-0.00	0.00
31 ->	50	0.00	0.01
31 ->	51	0.00	0.00
31 ->	52	-0.00	0.00
31 ->	53	0.02	0.00
31 ->	54	0.00	0.00
31 ->	55	-0.00	0.00
31 ->	56	0.00	0.00
31 ->	57	0.00	0.00
31 ->	58	-0.01	0.00
31 ->	59	0.01	0.00
31 ->	60	0.00	0.00
31 ->	61	-0.00	0.00
31 ->	62	-0.00	0.00
31 ->	63	-0.00	0.00
31 ->	64	-0.00	0.00
31 ->	65	-0.00	0.00
31 ->	66	-0.00	0.00

31	->	67	-0.00	0.00
31	->	68	0.01	0.00
31	->	69	0.01	0.00
31	->	70	0.00	0.00
31	->	71	0.00	0.00
31	->	72	0.03	0.01
31	->	73	0.00	0.00
31	->	74	-0.02	0.00
31	->	75	-0.02	0.00
31	->	76	0.01	0.00
31	->	77	0.01	0.00
31	->	78	0.02	0.00
31	->	79	0.00	0.00
31	->	80	0.00	0.00
31	->	81	0.01	0.00
31	->	82	0.00	0.00
31	->	83	0.00	0.00
31	->	84	-0.00	0.00
31	->	85	-0.00	0.00
31	->	86	-0.01	0.00
31	->	87	-0.04	0.02
31	->	88	-0.48	0.53
31	->	89	-3.30	3.31
31	->	90	-1.32	1.89
31	->	91	-0.07	0.02
31	->	92	-0.03	0.00
31	->	93	-0.01	0.00
31	->	94	-0.01	0.00
31	->	95	-0.00	0.00
31	->	96	-0.01	0.01
31	->	97	-0.05	0.04
31	->	98	-0.03	0.01
31	->	99	-0.03	0.01
31	->	100	-0.01	0.00
31	->	101	-0.01	0.00
31	->	102	-0.01	0.00
31	->	103	-0.02	0.00
31	->	104	-0.02	0.01
31	->	105	-0.02	0.01
31	->	106	-0.02	0.01
32	->	1	0.00	0.00
32	->	2	-0.00	0.00
32	->	3	-0.00	0.00
32	->	4	-0.00	0.00
32	->	5	-0.00	0.00
32	->	6	-0.00	0.00
32	->	7	-0.00	0.00
32	->	8	-0.00	0.00
32	->	9	0.00	0.00
32	->	10	-0.00	0.00

32	->	11	-0.00	0.00
32	->	12	0.00	0.00
32	->	13	0.00	0.00
32	->	14	-0.00	0.00
32	->	15	0.00	0.00
32	->	16	-0.00	0.00
32	->	17	-0.00	0.00
32	->	18	-0.01	0.00
32	->	19	-0.00	0.00
32	->	20	-0.00	0.00
32	->	21	-0.02	0.01
32	->	22	-0.03	0.02
32	->	23	-0.01	0.00
32	->	24	-0.01	0.00
32	->	25	-1.64	0.26
32	->	26	-0.03	0.01
32	->	27	-0.00	0.00
32	->	28	-0.02	0.00
32	->	29	-0.03	0.00
32	->	30	-1.35	0.34
32	->	31	-0.28	0.36
32	->	32	1.63	0.57
32	->	33	-10.50	0.52
32	->	34	-0.54	0.21
32	->	35	-0.01	0.00
32	->	36	-0.01	0.01
32	->	37	-0.00	0.00
32	->	38	0.00	0.00
32	->	39	-0.01	0.00
32	->	40	-0.02	0.01
32	->	41	-0.28	0.17
32	->	42	-0.77	0.17
32	->	43	-0.64	0.23
32	->	44	-0.13	0.06
32	->	45	-0.06	0.02
32	->	46	-0.01	0.00
32	->	47	-0.01	0.00
32	->	48	-0.04	0.01
32	->	49	-0.00	0.00
32	->	50	-0.00	0.00
32	->	51	-0.00	0.00
32	->	52	-0.00	0.00
32	->	53	-0.00	0.00
32	->	54	0.00	0.00
32	->	55	-0.00	0.00
32	->	56	-0.00	0.00
32	->	57	-0.00	0.00
32	->	58	0.00	0.00
32	->	59	-0.00	0.00
32	->	60	0.00	0.00

32	->	61	0.00	0.00
32	->	62	-0.00	0.00
32	->	63	-0.00	0.00
32	->	64	-0.00	0.00
32	->	65	0.00	0.00
32	->	66	-0.00	0.00
32	->	67	0.00	0.00
32	->	68	0.00	0.00
32	->	69	-0.00	0.00
32	->	70	0.00	0.00
32	->	71	0.00	0.00
32	->	72	0.00	0.00
32	->	73	0.00	0.00
32	->	74	-0.00	0.00
32	->	75	-0.00	0.00
32	->	76	0.00	0.00
32	->	77	0.00	0.00
32	->	78	0.00	0.00
32	->	79	0.00	0.00
32	->	80	0.00	0.00
32	->	81	0.00	0.00
32	->	82	0.00	0.00
32	->	83	0.00	0.00
32	->	84	-0.00	0.00
32	->	85	-0.00	0.00
32	->	86	0.00	0.00
32	->	87	-0.00	0.00
32	->	88	-0.00	0.00
32	->	89	-0.01	0.00
32	->	90	-0.01	0.00
32	->	91	-0.01	0.00
32	->	92	-0.00	0.00
32	->	93	-0.00	0.00
32	->	94	-0.00	0.00
32	->	95	-0.00	0.00
32	->	96	-0.01	0.01
32	->	97	-0.06	0.08
32	->	98	-0.02	0.01
32	->	99	-0.00	0.00
32	->	100	-0.00	0.00
32	->	101	-0.00	0.00
32	->	102	-0.00	0.00
32	->	103	-0.00	0.00
32	->	104	-0.00	0.00
32	->	105	-0.00	0.00
32	->	106	-0.00	0.00
33	->	1	0.00	0.00
33	->	2	0.00	0.00
33	->	3	-0.00	0.00
33	->	4	-0.00	0.00

33	->	5	0.00	0.00
33	->	6	-0.00	0.00
33	->	7	0.00	0.00
33	->	8	0.00	0.00
33	->	9	-0.00	0.00
33	->	10	0.00	0.00
33	->	11	0.00	0.00
33	->	12	-0.00	0.00
33	->	13	0.00	0.00
33	->	14	-0.00	0.00
33	->	15	-0.00	0.00
33	->	16	-0.00	0.00
33	->	17	-0.00	0.00
33	->	18	-0.01	0.01
33	->	19	-0.01	0.00
33	->	20	-0.01	0.00
33	->	21	-0.03	0.01
33	->	22	-0.06	0.04
33	->	23	-0.00	0.00
33	->	24	-0.01	0.00
33	->	25	-0.91	0.41
33	->	26	-0.00	0.00
33	->	27	-0.00	0.00
33	->	28	-0.00	0.00
33	->	29	-0.01	0.00
33	->	30	-0.10	0.02
33	->	31	-0.86	0.31
33	->	32	-10.63	0.50
33	->	33	27.87	1.34
33	->	34	-12.90	0.35
33	->	35	-0.43	0.06
33	->	36	-0.03	0.01
33	->	37	-0.01	0.00
33	->	38	-0.01	0.00
33	->	39	-0.02	0.01
33	->	40	-0.29	0.07
33	->	41	-2.64	0.66
33	->	42	-3.46	0.47
33	->	43	-0.95	0.22
33	->	44	-2.55	0.50
33	->	45	-2.17	0.53
33	->	46	0.01	0.02
33	->	47	-0.11	0.06
33	->	48	-1.42	0.40
33	->	49	-0.04	0.01
33	->	50	-0.01	0.00
33	->	51	-0.02	0.01
33	->	52	-0.02	0.00
33	->	53	-0.00	0.00
33	->	54	-0.00	0.00

33	->	55	-0.01	0.00
33	->	56	-0.00	0.00
33	->	57	-0.00	0.00
33	->	58	-0.00	0.00
33	->	59	0.00	0.00
33	->	60	0.00	0.00
33	->	61	-0.00	0.00
33	->	62	-0.00	0.00
33	->	63	-0.00	0.00
33	->	64	-0.00	0.00
33	->	65	-0.00	0.00
33	->	66	0.00	0.00
33	->	67	-0.00	0.00
33	->	68	0.00	0.00
33	->	69	0.00	0.00
33	->	70	0.00	0.00
33	->	71	-0.00	0.00
33	->	72	-0.00	0.00
33	->	73	0.00	0.00
33	->	74	-0.00	0.00
33	->	75	-0.00	0.00
33	->	76	0.00	0.00
33	->	77	0.00	0.00
33	->	78	-0.00	0.00
33	->	79	0.00	0.00
33	->	80	0.00	0.00
33	->	81	-0.00	0.00
33	->	82	0.00	0.00
33	->	83	0.00	0.00
33	->	84	-0.00	0.00
33	->	85	0.00	0.00
33	->	86	-0.00	0.00
33	->	87	-0.00	0.00
33	->	88	-0.01	0.01
33	->	89	-0.01	0.01
33	->	90	-0.01	0.00
33	->	91	-0.00	0.00
33	->	92	-0.00	0.00
33	->	93	-0.00	0.00
33	->	94	-0.01	0.00
33	->	95	-0.00	0.00
33	->	96	-0.02	0.02
33	->	97	-0.66	0.85
33	->	98	-0.33	0.26
33	->	99	-0.03	0.01
33	->	100	-0.01	0.00
33	->	101	-0.00	0.00
33	->	102	-0.00	0.00
33	->	103	-0.00	0.00
33	->	104	-0.00	0.00

33	->	105	-0.00	0.00
33	->	106	0.00	0.00
34	->	1	0.00	0.00
34	->	2	0.00	0.00
34	->	3	0.00	0.00
34	->	4	0.00	0.00
34	->	5	-0.00	0.00
34	->	6	0.00	0.00
34	->	7	0.00	0.00
34	->	8	-0.00	0.00
34	->	9	0.00	0.00
34	->	10	-0.00	0.00
34	->	11	-0.00	0.00
34	->	12	0.00	0.00
34	->	13	0.00	0.00
34	->	14	0.00	0.00
34	->	15	0.00	0.00
34	->	16	-0.00	0.00
34	->	17	0.00	0.00
34	->	18	-0.00	0.00
34	->	19	-0.00	0.00
34	->	20	-0.00	0.00
34	->	21	-0.00	0.00
34	->	22	-0.00	0.00
34	->	23	-0.00	0.00
34	->	24	-0.00	0.00
34	->	25	-0.01	0.00
34	->	26	-0.00	0.00
34	->	27	-0.00	0.00
34	->	28	-0.00	0.00
34	->	29	-0.00	0.00
34	->	30	-0.01	0.00
34	->	31	-0.14	0.05
34	->	32	-0.56	0.23
34	->	33	-12.90	0.35
34	->	34	-54.26	2.19
34	->	35	-12.03	0.78
34	->	36	-1.03	1.13
34	->	37	-0.06	0.05
34	->	38	-0.13	0.10
34	->	39	-2.62	0.84
34	->	40	-2.29	0.39
34	->	41	-3.42	1.14
34	->	42	-0.91	0.23
34	->	43	-0.04	0.01
34	->	44	-0.02	0.01
34	->	45	-0.02	0.01
34	->	46	-0.01	0.00
34	->	47	0.03	0.01
34	->	48	-1.18	0.28

34 ->	49	-0.02	0.00
34 ->	50	0.00	0.01
34 ->	51	-0.12	0.03
34 ->	52	-0.04	0.01
34 ->	53	-0.00	0.00
34 ->	54	-0.00	0.00
34 ->	55	-0.05	0.01
34 ->	56	-0.00	0.00
34 ->	57	0.00	0.00
34 ->	58	0.00	0.00
34 ->	59	0.00	0.00
34 ->	60	-0.00	0.00
34 ->	61	0.00	0.00
34 ->	62	0.00	0.00
34 ->	63	0.00	0.00
34 ->	64	-0.00	0.00
34 ->	65	0.00	0.00
34 ->	66	-0.00	0.00
34 ->	67	0.00	0.00
34 ->	68	0.00	0.00
34 ->	69	-0.00	0.00
34 ->	70	0.00	0.00
34 ->	71	0.00	0.00
34 ->	72	0.00	0.00
34 ->	73	-0.00	0.00
34 ->	74	-0.00	0.00
34 ->	75	-0.00	0.00
34 ->	76	0.00	0.00
34 ->	77	0.00	0.00
34 ->	78	0.00	0.00
34 ->	79	0.00	0.00
34 ->	80	0.00	0.00
34 ->	81	0.00	0.00
34 ->	82	0.00	0.00
34 ->	83	0.00	0.00
34 ->	84	-0.00	0.00
34 ->	85	-0.00	0.00
34 ->	86	-0.00	0.00
34 ->	87	-0.01	0.01
34 ->	88	-0.03	0.02
34 ->	89	-0.02	0.01
34 ->	90	-0.01	0.00
34 ->	91	-0.00	0.00
34 ->	92	-0.00	0.00
34 ->	93	0.00	0.00
34 ->	94	-0.00	0.00
34 ->	95	0.00	0.00
34 ->	96	-0.00	0.00
34 ->	97	-0.00	0.00
34 ->	98	-0.00	0.01

34	->	99	-0.00	0.00
34	->	100	-0.00	0.00
34	->	101	-0.00	0.00
34	->	102	-0.00	0.00
34	->	103	-0.00	0.00
34	->	104	-0.00	0.00
34	->	105	-0.00	0.00
34	->	106	-0.00	0.00
35	->	1	-0.01	0.00
35	->	2	-0.00	0.00
35	->	3	0.00	0.00
35	->	4	0.00	0.00
35	->	5	0.00	0.00
35	->	6	-0.00	0.00
35	->	7	0.00	0.00
35	->	8	0.00	0.00
35	->	9	-0.00	0.00
35	->	10	0.00	0.00
35	->	11	0.00	0.00
35	->	12	-0.01	0.00
35	->	13	-0.00	0.00
35	->	14	-0.00	0.00
35	->	15	-0.00	0.00
35	->	16	0.01	0.00
35	->	17	0.00	0.00
35	->	18	0.00	0.00
35	->	19	0.00	0.00
35	->	20	0.00	0.00
35	->	21	-0.00	0.00
35	->	22	-0.02	0.00
35	->	23	-0.00	0.00
35	->	24	0.00	0.00
35	->	25	-0.03	0.00
35	->	26	-0.00	0.00
35	->	27	-0.00	0.00
35	->	28	0.00	0.00
35	->	29	0.00	0.00
35	->	30	-0.00	0.00
35	->	31	-0.08	0.01
35	->	32	-0.01	0.00
35	->	33	-0.44	0.06
35	->	34	-12.10	0.77
35	->	35	-25.74	3.38
35	->	36	-9.62	0.83
35	->	37	0.55	1.89
35	->	38	2.55	0.90
35	->	39	-2.48	0.54
35	->	40	-2.73	0.35
35	->	41	-0.33	0.07
35	->	42	-0.60	0.16

35	->	43	0.01	0.01
35	->	44	-0.05	0.01
35	->	45	-0.00	0.01
35	->	46	0.04	0.01
35	->	47	-0.12	0.02
35	->	48	-1.14	0.23
35	->	49	0.02	0.01
35	->	50	-0.07	0.02
35	->	51	2.81	0.73
35	->	52	-0.31	0.21
35	->	53	-0.05	0.02
35	->	54	-0.06	0.05
35	->	55	2.13	0.58
35	->	56	-0.02	0.01
35	->	57	-0.00	0.00
35	->	58	0.03	0.01
35	->	59	-0.03	0.00
35	->	60	-0.00	0.00
35	->	61	0.00	0.00
35	->	62	0.00	0.00
35	->	63	0.01	0.00
35	->	64	0.00	0.00
35	->	65	0.00	0.00
35	->	66	0.01	0.00
35	->	67	0.00	0.00
35	->	68	-0.01	0.00
35	->	69	-0.01	0.00
35	->	70	-0.00	0.00
35	->	71	-0.00	0.00
35	->	72	-0.03	0.00
35	->	73	-0.00	0.00
35	->	74	0.02	0.00
35	->	75	0.02	0.00
35	->	76	-0.01	0.00
35	->	77	-0.01	0.00
35	->	78	-0.01	0.00
35	->	79	-0.00	0.00
35	->	80	-0.00	0.00
35	->	81	-0.00	0.00
35	->	82	-0.00	0.00
35	->	83	0.00	0.00
35	->	84	0.00	0.00
35	->	85	0.00	0.00
35	->	86	0.02	0.00
35	->	87	0.06	0.02
35	->	88	0.11	0.06
35	->	89	0.04	0.01
35	->	90	0.02	0.00
35	->	91	0.01	0.00
35	->	92	0.01	0.00

35	->	93	0.00	0.00
35	->	94	0.00	0.00
35	->	95	0.00	0.00
35	->	96	0.00	0.00
35	->	97	0.02	0.01
35	->	98	0.03	0.01
35	->	99	0.03	0.01
35	->	100	0.01	0.00
35	->	101	0.01	0.00
35	->	102	0.01	0.00
35	->	103	0.01	0.00
35	->	104	0.01	0.00
35	->	105	0.00	0.00
35	->	106	0.01	0.00
36	->	1	0.00	0.00
36	->	2	0.00	0.00
36	->	3	0.00	0.00
36	->	4	-0.00	0.00
36	->	5	-0.00	0.00
36	->	6	0.00	0.00
36	->	7	0.00	0.00
36	->	8	-0.00	0.00
36	->	9	0.00	0.00
36	->	10	-0.00	0.00
36	->	11	-0.00	0.00
36	->	12	0.00	0.00
36	->	13	0.00	0.00
36	->	14	0.00	0.00
36	->	15	0.00	0.00
36	->	16	-0.01	0.00
36	->	17	-0.00	0.00
36	->	18	-0.00	0.00
36	->	19	-0.00	0.00
36	->	20	-0.00	0.00
36	->	21	0.00	0.00
36	->	22	0.01	0.00
36	->	23	0.00	0.00
36	->	24	-0.00	0.00
36	->	25	0.02	0.00
36	->	26	0.00	0.00
36	->	27	0.00	0.00
36	->	28	-0.00	0.00
36	->	29	-0.00	0.00
36	->	30	-0.00	0.00
36	->	31	0.01	0.00
36	->	32	-0.01	0.01
36	->	33	-0.03	0.01
36	->	34	-1.06	1.15
36	->	35	-9.67	0.85
36	->	36	-116.20	2.38

36	->	37	-14.14	0.92
36	->	38	-0.88	0.25
36	->	39	-1.11	0.74
36	->	40	-0.02	0.03
36	->	41	-0.03	0.02
36	->	42	-0.02	0.01
36	->	43	-0.00	0.00
36	->	44	0.01	0.00
36	->	45	-0.01	0.01
36	->	46	-0.03	0.01
36	->	47	-0.01	0.00
36	->	48	-0.16	0.14
36	->	49	-0.02	0.04
36	->	50	-0.01	0.00
36	->	51	-0.11	0.05
36	->	52	-0.51	0.88
36	->	53	0.02	0.01
36	->	54	-0.00	0.00
36	->	55	-0.20	0.12
36	->	56	-0.03	0.09
36	->	57	0.00	0.00
36	->	58	-0.04	0.02
36	->	59	0.02	0.01
36	->	60	0.00	0.00
36	->	61	-0.00	0.00
36	->	62	-0.00	0.00
36	->	63	-0.00	0.00
36	->	64	-0.00	0.00
36	->	65	-0.00	0.00
36	->	66	-0.00	0.00
36	->	67	-0.00	0.00
36	->	68	0.01	0.00
36	->	69	0.01	0.00
36	->	70	-0.00	0.00
36	->	71	-0.00	0.00
36	->	72	0.01	0.00
36	->	73	0.00	0.00
36	->	74	-0.01	0.00
36	->	75	-0.01	0.00
36	->	76	0.01	0.00
36	->	77	0.00	0.00
36	->	78	0.01	0.00
36	->	79	0.00	0.00
36	->	80	0.00	0.00
36	->	81	0.00	0.00
36	->	82	0.00	0.00
36	->	83	-0.00	0.00
36	->	84	-0.00	0.00
36	->	85	-0.00	0.00
36	->	86	-0.01	0.00

36	->	87	-0.02	0.00
36	->	88	-0.03	0.00
36	->	89	-0.02	0.00
36	->	90	-0.01	0.00
36	->	91	-0.01	0.00
36	->	92	-0.01	0.00
36	->	93	-0.00	0.00
36	->	94	-0.00	0.00
36	->	95	-0.00	0.00
36	->	96	-0.00	0.00
36	->	97	-0.03	0.01
36	->	98	-0.05	0.02
36	->	99	-0.04	0.02
36	->	100	-0.02	0.01
36	->	101	-0.01	0.00
36	->	102	-0.00	0.00
36	->	103	-0.00	0.00
36	->	104	-0.00	0.00
36	->	105	-0.00	0.00
36	->	106	-0.00	0.00
37	->	1	-0.00	0.00
37	->	2	0.00	0.00
37	->	3	0.00	0.00
37	->	4	0.00	0.00
37	->	5	0.00	0.00
37	->	6	0.00	0.00
37	->	7	0.00	0.00
37	->	8	0.00	0.00
37	->	9	0.00	0.00
37	->	10	0.00	0.00
37	->	11	0.00	0.00
37	->	12	-0.00	0.00
37	->	13	-0.00	0.00
37	->	14	-0.00	0.00
37	->	15	0.00	0.00
37	->	16	0.00	0.00
37	->	17	0.00	0.00
37	->	18	0.00	0.00
37	->	19	0.00	0.00
37	->	20	0.00	0.00
37	->	21	0.00	0.00
37	->	22	-0.00	0.00
37	->	23	0.00	0.00
37	->	24	-0.00	0.00
37	->	25	-0.00	0.00
37	->	26	0.00	0.00
37	->	27	-0.00	0.00
37	->	28	0.00	0.00
37	->	29	0.00	0.00
37	->	30	0.00	0.00

37	->	31	-0.00	0.00
37	->	32	-0.00	0.00
37	->	33	-0.01	0.00
37	->	34	-0.06	0.05
37	->	35	0.47	1.89
37	->	36	-14.25	0.90
37	->	37	20.56	2.57
37	->	38	-9.96	3.14
37	->	39	-0.83	0.36
37	->	40	-0.16	0.09
37	->	41	-0.01	0.00
37	->	42	-0.02	0.01
37	->	43	-0.00	0.00
37	->	44	-0.00	0.00
37	->	45	-0.00	0.00
37	->	46	-0.00	0.00
37	->	47	-0.00	0.00
37	->	48	-0.03	0.01
37	->	49	-0.00	0.00
37	->	50	-0.01	0.00
37	->	51	0.46	0.42
37	->	52	-0.15	0.23
37	->	53	-0.01	0.01
37	->	54	-0.03	0.02
37	->	55	0.58	0.71
37	->	56	-0.03	0.04
37	->	57	-0.00	0.00
37	->	58	-0.00	0.00
37	->	59	-0.00	0.00
37	->	60	-0.00	0.00
37	->	61	0.00	0.00
37	->	62	0.00	0.00
37	->	63	0.00	0.00
37	->	64	0.00	0.00
37	->	65	0.00	0.00
37	->	66	0.00	0.00
37	->	67	0.00	0.00
37	->	68	-0.00	0.00
37	->	69	-0.00	0.00
37	->	70	-0.00	0.00
37	->	71	-0.00	0.00
37	->	72	-0.00	0.00
37	->	73	0.00	0.00
37	->	74	0.00	0.00
37	->	75	0.00	0.00
37	->	76	-0.00	0.00
37	->	77	-0.00	0.00
37	->	78	-0.00	0.00
37	->	79	-0.00	0.00
37	->	80	0.00	0.00

37	->	81	-0.00	0.00
37	->	82	0.00	0.00
37	->	83	0.00	0.00
37	->	84	0.00	0.00
37	->	85	-0.00	0.00
37	->	86	-0.00	0.00
37	->	87	-0.00	0.00
37	->	88	-0.00	0.01
37	->	89	-0.00	0.00
37	->	90	0.00	0.00
37	->	91	0.00	0.00
37	->	92	0.00	0.00
37	->	93	0.00	0.00
37	->	94	0.00	0.00
37	->	95	0.00	0.00
37	->	96	-0.00	0.00
37	->	97	-0.00	0.00
37	->	98	-0.00	0.00
37	->	99	-0.00	0.00
37	->	100	-0.00	0.00
37	->	101	0.00	0.00
37	->	102	0.00	0.00
37	->	103	0.00	0.00
37	->	104	0.00	0.00
37	->	105	-0.00	0.00
37	->	106	0.00	0.00
38	->	1	-0.00	0.00
38	->	2	-0.00	0.00
38	->	3	0.00	0.00
38	->	4	0.00	0.00
38	->	5	0.00	0.00
38	->	6	-0.00	0.00
38	->	7	-0.00	0.00
38	->	8	0.00	0.00
38	->	9	0.00	0.00
38	->	10	0.00	0.00
38	->	11	0.00	0.00
38	->	12	-0.01	0.00
38	->	13	-0.00	0.00
38	->	14	-0.00	0.00
38	->	15	-0.00	0.00
38	->	16	0.01	0.00
38	->	17	0.00	0.00
38	->	18	0.00	0.00
38	->	19	0.00	0.00
38	->	20	0.00	0.00
38	->	21	0.00	0.00
38	->	22	-0.01	0.00
38	->	23	-0.00	0.00
38	->	24	0.00	0.00

38	->	25	-0.02	0.00
38	->	26	0.00	0.00
38	->	27	-0.00	0.00
38	->	28	0.00	0.00
38	->	29	0.00	0.00
38	->	30	0.00	0.00
38	->	31	-0.05	0.01
38	->	32	0.00	0.00
38	->	33	-0.01	0.00
38	->	34	-0.13	0.10
38	->	35	2.55	0.91
38	->	36	-0.89	0.25
38	->	37	-9.94	3.19
38	->	38	-34.37	3.24
38	->	39	-11.71	0.57
38	->	40	-3.40	0.70
38	->	41	0.02	0.02
38	->	42	-0.01	0.03
38	->	43	0.00	0.00
38	->	44	-0.02	0.00
38	->	45	0.00	0.00
38	->	46	0.03	0.00
38	->	47	-0.02	0.01
38	->	48	-0.01	0.01
38	->	49	0.00	0.00
38	->	50	-0.02	0.01
38	->	51	3.34	0.74
38	->	52	0.04	0.02
38	->	53	-0.04	0.01
38	->	54	-0.04	0.03
38	->	55	1.81	0.41
38	->	56	-0.01	0.00
38	->	57	-0.00	0.00
38	->	58	0.02	0.00
38	->	59	-0.03	0.00
38	->	60	-0.00	0.00
38	->	61	0.00	0.00
38	->	62	0.00	0.00
38	->	63	0.01	0.00
38	->	64	0.00	0.00
38	->	65	0.00	0.00
38	->	66	0.01	0.00
38	->	67	0.00	0.00
38	->	68	-0.01	0.00
38	->	69	-0.01	0.00
38	->	70	-0.00	0.00
38	->	71	-0.00	0.00
38	->	72	-0.03	0.00
38	->	73	-0.00	0.00
38	->	74	0.02	0.00

38	->	75	0.02	0.00
38	->	76	-0.01	0.00
38	->	77	-0.01	0.00
38	->	78	-0.01	0.00
38	->	79	-0.00	0.00
38	->	80	-0.00	0.00
38	->	81	-0.00	0.00
38	->	82	-0.00	0.00
38	->	83	0.00	0.00
38	->	84	0.00	0.00
38	->	85	0.00	0.00
38	->	86	0.02	0.01
38	->	87	0.09	0.04
38	->	88	0.19	0.13
38	->	89	0.05	0.01
38	->	90	0.03	0.00
38	->	91	0.01	0.00
38	->	92	0.01	0.00
38	->	93	0.00	0.00
38	->	94	0.00	0.00
38	->	95	0.00	0.00
38	->	96	0.00	0.00
38	->	97	0.01	0.00
38	->	98	0.02	0.00
38	->	99	0.02	0.00
38	->	100	0.01	0.00
38	->	101	0.01	0.00
38	->	102	0.00	0.00
38	->	103	0.01	0.00
38	->	104	0.01	0.00
38	->	105	0.00	0.00
38	->	106	0.01	0.00
39	->	1	-0.00	0.00
39	->	2	0.00	0.00
39	->	3	0.00	0.00
39	->	4	0.00	0.00
39	->	5	0.00	0.00
39	->	6	0.00	0.00
39	->	7	0.00	0.00
39	->	8	0.00	0.00
39	->	9	0.00	0.00
39	->	10	0.00	0.00
39	->	11	0.00	0.00
39	->	12	-0.00	0.00
39	->	13	-0.00	0.00
39	->	14	0.00	0.00
39	->	15	0.00	0.00
39	->	16	0.00	0.00
39	->	17	0.00	0.00
39	->	18	0.00	0.00

39	->	19	0.00	0.00
39	->	20	0.00	0.00
39	->	21	0.00	0.00
39	->	22	-0.00	0.00
39	->	23	0.00	0.00
39	->	24	0.00	0.00
39	->	25	-0.00	0.00
39	->	26	-0.00	0.00
39	->	27	-0.00	0.00
39	->	28	0.00	0.00
39	->	29	-0.00	0.00
39	->	30	-0.00	0.00
39	->	31	-0.01	0.00
39	->	32	-0.01	0.00
39	->	33	-0.02	0.01
39	->	34	-2.62	0.84
39	->	35	-2.50	0.54
39	->	36	-1.11	0.72
39	->	37	-0.85	0.36
39	->	38	-11.82	0.56
39	->	39	12.47	1.14
39	->	40	-9.45	0.53
39	->	41	-0.43	0.37
39	->	42	-0.11	0.02
39	->	43	-0.01	0.00
39	->	44	-0.00	0.00
39	->	45	-0.00	0.00
39	->	46	-0.00	0.00
39	->	47	0.00	0.00
39	->	48	-0.02	0.01
39	->	49	-0.00	0.00
39	->	50	-0.00	0.00
39	->	51	-0.10	0.04
39	->	52	-0.01	0.00
39	->	53	-0.00	0.00
39	->	54	-0.00	0.00
39	->	55	-0.03	0.02
39	->	56	-0.00	0.00
39	->	57	0.00	0.00
39	->	58	-0.00	0.00
39	->	59	0.00	0.00
39	->	60	0.00	0.00
39	->	61	0.00	0.00
39	->	62	0.00	0.00
39	->	63	-0.00	0.00
39	->	64	-0.00	0.00
39	->	65	0.00	0.00
39	->	66	-0.00	0.00
39	->	67	-0.00	0.00
39	->	68	0.00	0.00

39	->	69	0.00	0.00
39	->	70	-0.00	0.00
39	->	71	0.00	0.00
39	->	72	0.00	0.00
39	->	73	0.00	0.00
39	->	74	-0.00	0.00
39	->	75	-0.00	0.00
39	->	76	0.00	0.00
39	->	77	0.00	0.00
39	->	78	0.00	0.00
39	->	79	0.00	0.00
39	->	80	0.00	0.00
39	->	81	0.00	0.00
39	->	82	0.00	0.00
39	->	83	0.00	0.00
39	->	84	-0.00	0.00
39	->	85	-0.00	0.00
39	->	86	-0.00	0.00
39	->	87	-0.00	0.00
39	->	88	-0.02	0.01
39	->	89	-0.00	0.00
39	->	90	-0.00	0.00
39	->	91	0.00	0.00
39	->	92	0.00	0.00
39	->	93	-0.00	0.00
39	->	94	-0.00	0.00
39	->	95	-0.00	0.00
39	->	96	0.00	0.00
39	->	97	-0.00	0.00
39	->	98	-0.00	0.00
39	->	99	-0.00	0.00
39	->	100	-0.00	0.00
39	->	101	-0.00	0.00
39	->	102	-0.00	0.00
39	->	103	0.00	0.00
39	->	104	0.00	0.00
39	->	105	-0.00	0.00
39	->	106	-0.00	0.00
40	->	1	0.01	0.00
40	->	2	0.00	0.00
40	->	3	-0.00	0.00
40	->	4	0.00	0.00
40	->	5	-0.01	0.00
40	->	6	0.00	0.00
40	->	7	0.00	0.00
40	->	8	-0.00	0.00
40	->	9	-0.00	0.00
40	->	10	-0.01	0.00
40	->	11	-0.00	0.00
40	->	12	0.01	0.00

40 ->	13	0.00	0.00
40 ->	14	0.00	0.00
40 ->	15	0.00	0.00
40 ->	16	-0.01	0.00
40 ->	17	-0.00	0.00
40 ->	18	-0.00	0.00
40 ->	19	-0.00	0.00
40 ->	20	-0.00	0.00
40 ->	21	-0.00	0.00
40 ->	22	0.02	0.00
40 ->	23	-0.00	0.00
40 ->	24	-0.00	0.00
40 ->	25	0.02	0.01
40 ->	26	-0.00	0.00
40 ->	27	-0.00	0.00
40 ->	28	-0.00	0.00
40 ->	29	-0.00	0.00
40 ->	30	0.01	0.00
40 ->	31	0.23	0.24
40 ->	32	-0.02	0.01
40 ->	33	-0.29	0.07
40 ->	34	-2.27	0.39
40 ->	35	-2.73	0.35
40 ->	36	-0.02	0.03
40 ->	37	-0.16	0.09
40 ->	38	-3.51	0.71
40 ->	39	-9.50	0.53
40 ->	40	-103.77	2.97
40 ->	41	-15.59	0.72
40 ->	42	-3.81	0.86
40 ->	43	-0.10	0.06
40 ->	44	0.02	0.01
40 ->	45	-0.01	0.01
40 ->	46	-0.05	0.01
40 ->	47	-0.01	0.03
40 ->	48	-0.18	0.06
40 ->	49	-0.01	0.00
40 ->	50	0.02	0.02
40 ->	51	-1.44	0.63
40 ->	52	-0.05	0.02
40 ->	53	0.03	0.01
40 ->	54	-0.03	0.02
40 ->	55	-0.22	0.07
40 ->	56	0.00	0.00
40 ->	57	0.00	0.00
40 ->	58	-0.02	0.00
40 ->	59	0.03	0.01
40 ->	60	0.00	0.00
40 ->	61	-0.00	0.00
40 ->	62	-0.00	0.00

40	->	63	-0.01	0.00
40	->	64	-0.00	0.00
40	->	65	-0.00	0.00
40	->	66	-0.01	0.00
40	->	67	-0.00	0.00
40	->	68	0.02	0.01
40	->	69	0.01	0.00
40	->	70	0.00	0.00
40	->	71	-0.00	0.00
40	->	72	0.06	0.02
40	->	73	-0.00	0.00
40	->	74	-0.03	0.00
40	->	75	-0.04	0.01
40	->	76	0.01	0.00
40	->	77	0.01	0.00
40	->	78	0.02	0.00
40	->	79	0.00	0.00
40	->	80	0.00	0.00
40	->	81	0.01	0.00
40	->	82	0.00	0.00
40	->	83	-0.00	0.00
40	->	84	-0.00	0.00
40	->	85	-0.01	0.00
40	->	86	-0.05	0.02
40	->	87	-1.00	0.62
40	->	88	-7.69	3.12
40	->	89	-2.57	2.77
40	->	90	-0.07	0.02
40	->	91	-0.03	0.01
40	->	92	-0.01	0.00
40	->	93	-0.01	0.00
40	->	94	-0.00	0.00
40	->	95	-0.00	0.00
40	->	96	-0.00	0.00
40	->	97	-0.01	0.01
40	->	98	-0.02	0.01
40	->	99	-0.02	0.00
40	->	100	-0.01	0.00
40	->	101	-0.01	0.00
40	->	102	-0.01	0.00
40	->	103	-0.01	0.00
40	->	104	-0.02	0.00
40	->	105	-0.01	0.01
40	->	106	-0.03	0.01
41	->	1	0.00	0.00
41	->	2	0.00	0.00
41	->	3	0.00	0.00
41	->	4	0.00	0.00
41	->	5	-0.00	0.00
41	->	6	0.00	0.00

41	->	7	0.00	0.00
41	->	8	-0.00	0.00
41	->	9	0.00	0.00
41	->	10	-0.00	0.00
41	->	11	-0.00	0.00
41	->	12	0.00	0.00
41	->	13	0.00	0.00
41	->	14	0.00	0.00
41	->	15	0.00	0.00
41	->	16	-0.00	0.00
41	->	17	-0.00	0.00
41	->	18	-0.00	0.00
41	->	19	0.00	0.00
41	->	20	0.00	0.00
41	->	21	0.00	0.00
41	->	22	0.01	0.00
41	->	23	-0.00	0.00
41	->	24	0.00	0.00
41	->	25	0.03	0.01
41	->	26	-0.02	0.00
41	->	27	-0.00	0.00
41	->	28	-0.00	0.00
41	->	29	0.00	0.01
41	->	30	0.05	0.04
41	->	31	-2.47	0.92
41	->	32	-0.28	0.17
41	->	33	-2.65	0.66
41	->	34	-3.35	1.11
41	->	35	-0.33	0.07
41	->	36	-0.03	0.02
41	->	37	-0.01	0.00
41	->	38	0.02	0.02
41	->	39	-0.44	0.38
41	->	40	-15.56	0.72
41	->	41	-31.32	2.08
41	->	42	-8.71	0.46
41	->	43	-0.57	0.09
41	->	44	-0.02	0.04
41	->	45	-0.04	0.01
41	->	46	-0.02	0.01
41	->	47	0.03	0.03
41	->	48	-0.08	0.02
41	->	49	-0.01	0.00
41	->	50	0.01	0.01
41	->	51	-0.02	0.01
41	->	52	-0.01	0.00
41	->	53	-0.00	0.00
41	->	54	-0.00	0.00
41	->	55	-0.01	0.00
41	->	56	-0.00	0.00

41	->	57	-0.00	0.00
41	->	58	0.00	0.00
41	->	59	-0.00	0.00
41	->	60	-0.00	0.00
41	->	61	0.00	0.00
41	->	62	0.00	0.00
41	->	63	0.00	0.00
41	->	64	0.00	0.00
41	->	65	0.00	0.00
41	->	66	0.00	0.00
41	->	67	0.00	0.00
41	->	68	-0.00	0.00
41	->	69	-0.00	0.00
41	->	70	0.00	0.00
41	->	71	0.00	0.00
41	->	72	-0.00	0.00
41	->	73	-0.00	0.00
41	->	74	0.00	0.00
41	->	75	0.00	0.00
41	->	76	-0.00	0.00
41	->	77	-0.00	0.00
41	->	78	-0.00	0.00
41	->	79	-0.00	0.00
41	->	80	0.00	0.00
41	->	81	-0.00	0.00
41	->	82	0.00	0.00
41	->	83	0.00	0.00
41	->	84	0.00	0.00
41	->	85	0.00	0.00
41	->	86	-0.00	0.00
41	->	87	-0.01	0.01
41	->	88	-0.06	0.05
41	->	89	-0.10	0.08
41	->	90	-0.04	0.04
41	->	91	-0.00	0.00
41	->	92	-0.00	0.00
41	->	93	-0.00	0.00
41	->	94	-0.00	0.00
41	->	95	-0.00	0.00
41	->	96	-0.00	0.00
41	->	97	-0.01	0.01
41	->	98	-0.01	0.01
41	->	99	-0.00	0.00
41	->	100	-0.00	0.00
41	->	101	-0.00	0.00
41	->	102	-0.00	0.00
41	->	103	-0.00	0.00
41	->	104	-0.00	0.00
41	->	105	-0.00	0.00
41	->	106	-0.00	0.00

42	->	1	0.00	0.00
42	->	2	0.00	0.00
42	->	3	-0.00	0.00
42	->	4	0.00	0.00
42	->	5	-0.00	0.00
42	->	6	-0.00	0.00
42	->	7	0.00	0.00
42	->	8	-0.00	0.00
42	->	9	0.00	0.00
42	->	10	-0.00	0.00
42	->	11	-0.00	0.00
42	->	12	0.00	0.00
42	->	13	0.00	0.00
42	->	14	-0.00	0.00
42	->	15	-0.00	0.00
42	->	16	-0.00	0.00
42	->	17	-0.00	0.00
42	->	18	-0.00	0.00
42	->	19	0.00	0.00
42	->	20	0.00	0.00
42	->	21	0.00	0.00
42	->	22	0.02	0.01
42	->	23	-0.01	0.00
42	->	24	0.00	0.00
42	->	25	0.01	0.02
42	->	26	-0.02	0.01
42	->	27	-0.01	0.00
42	->	28	-0.00	0.00
42	->	29	0.00	0.01
42	->	30	-0.18	0.06
42	->	31	-3.07	0.41
42	->	32	-0.77	0.17
42	->	33	-3.46	0.47
42	->	34	-0.91	0.23
42	->	35	-0.58	0.16
42	->	36	-0.02	0.01
42	->	37	-0.02	0.01
42	->	38	-0.01	0.03
42	->	39	-0.11	0.02
42	->	40	-3.75	0.85
42	->	41	-8.79	0.47
42	->	42	29.24	1.05
42	->	43	-17.22	0.43
42	->	44	-0.97	0.20
42	->	45	-0.21	0.06
42	->	46	-0.12	0.03
42	->	47	-2.25	0.39
42	->	48	-2.50	0.43
42	->	49	-0.06	0.01
42	->	50	-0.15	0.06

42	->	51	-1.99	0.31
42	->	52	-0.06	0.02
42	->	53	-0.02	0.01
42	->	54	-0.02	0.01
42	->	55	-0.01	0.01
42	->	56	-0.00	0.00
42	->	57	-0.00	0.00
42	->	58	-0.00	0.00
42	->	59	-0.00	0.00
42	->	60	-0.00	0.00
42	->	61	-0.00	0.00
42	->	62	-0.00	0.00
42	->	63	-0.00	0.00
42	->	64	-0.00	0.00
42	->	65	0.00	0.00
42	->	66	-0.00	0.00
42	->	67	0.00	0.00
42	->	68	0.00	0.00
42	->	69	0.00	0.00
42	->	70	-0.00	0.00
42	->	71	-0.00	0.00
42	->	72	-0.01	0.01
42	->	73	-0.00	0.00
42	->	74	-0.00	0.00
42	->	75	-0.00	0.00
42	->	76	0.00	0.00
42	->	77	0.00	0.00
42	->	78	0.00	0.00
42	->	79	0.00	0.00
42	->	80	0.00	0.00
42	->	81	0.00	0.00
42	->	82	0.00	0.00
42	->	83	0.00	0.00
42	->	84	-0.00	0.00
42	->	85	-0.00	0.00
42	->	86	-0.01	0.00
42	->	87	-0.02	0.03
42	->	88	-0.69	0.79
42	->	89	-0.66	0.48
42	->	90	-0.25	0.18
42	->	91	-0.02	0.01
42	->	92	-0.01	0.00
42	->	93	-0.00	0.00
42	->	94	-0.00	0.00
42	->	95	-0.00	0.00
42	->	96	-0.00	0.00
42	->	97	-0.01	0.01
42	->	98	-0.02	0.01
42	->	99	-0.01	0.00
42	->	100	-0.00	0.00

42	->	101	-0.00	0.00
42	->	102	-0.00	0.00
42	->	103	-0.00	0.00
42	->	104	-0.00	0.00
42	->	105	-0.01	0.00
42	->	106	-0.00	0.00
43	->	1	-0.00	0.00
43	->	2	0.00	0.00
43	->	3	-0.00	0.00
43	->	4	0.00	0.00
43	->	5	-0.00	0.00
43	->	6	-0.00	0.00
43	->	7	-0.00	0.00
43	->	8	-0.00	0.00
43	->	9	0.00	0.00
43	->	10	-0.00	0.00
43	->	11	-0.00	0.00
43	->	12	0.00	0.00
43	->	13	0.00	0.00
43	->	14	-0.00	0.00
43	->	15	-0.00	0.00
43	->	16	0.00	0.00
43	->	17	-0.00	0.00
43	->	18	-0.01	0.01
43	->	19	-0.00	0.01
43	->	20	-0.01	0.00
43	->	21	-0.05	0.03
43	->	22	-0.54	0.57
43	->	23	-0.02	0.01
43	->	24	-0.01	0.01
43	->	25	-1.51	1.53
43	->	26	-0.41	0.29
43	->	27	-0.01	0.01
43	->	28	-0.02	0.01
43	->	29	-0.03	0.04
43	->	30	-0.68	0.79
43	->	31	-2.98	1.25
43	->	32	-0.63	0.22
43	->	33	-0.94	0.22
43	->	34	-0.04	0.01
43	->	35	0.01	0.01
43	->	36	-0.00	0.00
43	->	37	-0.00	0.00
43	->	38	0.00	0.00
43	->	39	-0.01	0.00
43	->	40	-0.10	0.06
43	->	41	-0.57	0.09
43	->	42	-17.21	0.43
43	->	43	22.97	2.05
43	->	44	-6.36	0.44

43	->	45	-0.23	0.05
43	->	46	-0.16	0.07
43	->	47	-1.11	0.80
43	->	48	-0.04	0.02
43	->	49	-0.00	0.00
43	->	50	-0.01	0.00
43	->	51	-0.01	0.01
43	->	52	-0.00	0.00
43	->	53	-0.00	0.00
43	->	54	-0.00	0.00
43	->	55	0.00	0.00
43	->	56	0.00	0.00
43	->	57	0.00	0.00
43	->	58	-0.00	0.00
43	->	59	0.00	0.00
43	->	60	0.00	0.00
43	->	61	0.00	0.00
43	->	62	-0.00	0.00
43	->	63	-0.00	0.00
43	->	64	-0.00	0.00
43	->	65	0.00	0.00
43	->	66	-0.00	0.00
43	->	67	0.00	0.00
43	->	68	0.00	0.00
43	->	69	0.00	0.00
43	->	70	0.00	0.00
43	->	71	0.00	0.00
43	->	72	0.00	0.00
43	->	73	-0.00	0.00
43	->	74	-0.00	0.00
43	->	75	-0.00	0.00
43	->	76	0.00	0.00
43	->	77	0.00	0.00
43	->	78	0.00	0.00
43	->	79	0.00	0.00
43	->	80	0.00	0.00
43	->	81	0.00	0.00
43	->	82	0.00	0.00
43	->	83	0.00	0.00
43	->	84	-0.00	0.00
43	->	85	-0.00	0.00
43	->	86	-0.00	0.00
43	->	87	-0.00	0.00
43	->	88	0.01	0.03
43	->	89	-0.43	0.38
43	->	90	-2.90	2.25
43	->	91	-0.07	0.02
43	->	92	-0.02	0.01
43	->	93	-0.00	0.00
43	->	94	0.00	0.00

43	->	95	-0.00	0.00
43	->	96	-0.01	0.01
43	->	97	-0.01	0.02
43	->	98	-0.02	0.01
43	->	99	-0.01	0.01
43	->	100	0.00	0.00
43	->	101	-0.00	0.00
43	->	102	-0.00	0.00
43	->	103	-0.00	0.00
43	->	104	-0.00	0.00
43	->	105	-0.01	0.00
43	->	106	-0.00	0.00
44	->	1	0.05	0.01
44	->	2	0.00	0.00
44	->	3	-0.00	0.00
44	->	4	-0.00	0.00
44	->	5	-0.03	0.00
44	->	6	0.00	0.00
44	->	7	-0.00	0.00
44	->	8	-0.01	0.00
44	->	9	-0.00	0.00
44	->	10	-0.02	0.00
44	->	11	-0.01	0.00
44	->	12	0.04	0.01
44	->	13	0.01	0.00
44	->	14	-0.00	0.00
44	->	15	0.00	0.00
44	->	16	-0.08	0.04
44	->	17	-0.00	0.00
44	->	18	-0.04	0.07
44	->	19	-0.04	0.09
44	->	20	-0.02	0.01
44	->	21	0.03	0.02
44	->	22	-0.73	0.91
44	->	23	-0.05	0.02
44	->	24	-0.01	0.01
44	->	25	-0.16	0.26
44	->	26	-0.05	0.02
44	->	27	-0.00	0.00
44	->	28	-0.00	0.00
44	->	29	-0.01	0.00
44	->	30	-0.01	0.01
44	->	31	-0.07	0.05
44	->	32	-0.13	0.06
44	->	33	-2.57	0.51
44	->	34	-0.02	0.01
44	->	35	-0.05	0.01
44	->	36	0.01	0.00
44	->	37	-0.00	0.00
44	->	38	-0.02	0.00

44	->	39	-0.00	0.00
44	->	40	0.02	0.01
44	->	41	-0.02	0.04
44	->	42	-0.97	0.20
44	->	43	-6.48	0.45
44	->	44	-94.63	3.28
44	->	45	-21.25	0.64
44	->	46	-12.41	4.84
44	->	47	-5.01	0.62
44	->	48	-1.24	0.45
44	->	49	-0.04	0.05
44	->	50	-0.06	0.04
44	->	51	-0.01	0.01
44	->	52	-0.00	0.00
44	->	53	0.05	0.01
44	->	54	-0.00	0.00
44	->	55	-0.00	0.00
44	->	56	0.00	0.00
44	->	57	0.00	0.00
44	->	58	-0.02	0.00
44	->	59	0.02	0.00
44	->	60	0.00	0.00
44	->	61	-0.00	0.00
44	->	62	-0.00	0.00
44	->	63	-0.01	0.00
44	->	64	-0.01	0.00
44	->	65	-0.00	0.00
44	->	66	-0.01	0.00
44	->	67	-0.00	0.00
44	->	68	0.01	0.00
44	->	69	0.01	0.00
44	->	70	0.00	0.00
44	->	71	0.00	0.00
44	->	72	0.15	0.14
44	->	73	-0.00	0.01
44	->	74	-0.09	0.04
44	->	75	-0.03	0.01
44	->	76	0.02	0.00
44	->	77	0.03	0.01
44	->	78	0.02	0.00
44	->	79	0.00	0.00
44	->	80	0.00	0.00
44	->	81	0.02	0.00
44	->	82	0.00	0.00
44	->	83	0.00	0.00
44	->	84	-0.00	0.00
44	->	85	-0.00	0.00
44	->	86	-0.02	0.00
44	->	87	-0.03	0.01
44	->	88	-0.07	0.02

44	->	89	-0.26	0.10
44	->	90	-2.17	1.67
44	->	91	-6.60	2.78
44	->	92	-4.01	2.02
44	->	93	-0.06	0.13
44	->	94	-0.01	0.02
44	->	95	-0.02	0.00
44	->	96	-0.03	0.02
44	->	97	-0.21	0.13
44	->	98	-0.44	0.27
44	->	99	-1.73	1.93
44	->	100	0.60	0.53
44	->	101	0.27	0.52
44	->	102	-0.12	0.10
44	->	103	-0.07	0.04
44	->	104	-0.04	0.01
44	->	105	-0.02	0.00
44	->	106	-0.01	0.00
45	->	1	0.00	0.00
45	->	2	0.00	0.00
45	->	3	-0.00	0.00
45	->	4	-0.00	0.00
45	->	5	-0.00	0.00
45	->	6	0.00	0.00
45	->	7	0.00	0.00
45	->	8	-0.00	0.00
45	->	9	0.00	0.00
45	->	10	-0.00	0.00
45	->	11	-0.00	0.00
45	->	12	0.00	0.00
45	->	13	0.00	0.00
45	->	14	-0.00	0.00
45	->	15	0.00	0.00
45	->	16	-0.01	0.00
45	->	17	-0.00	0.00
45	->	18	-0.01	0.00
45	->	19	-0.01	0.00
45	->	20	-0.00	0.00
45	->	21	-0.01	0.01
45	->	22	-0.06	0.05
45	->	23	0.00	0.00
45	->	24	-0.00	0.00
45	->	25	-0.09	0.07
45	->	26	0.00	0.00
45	->	27	0.00	0.00
45	->	28	-0.00	0.00
45	->	29	-0.00	0.00
45	->	30	-0.01	0.00
45	->	31	-0.08	0.02
45	->	32	-0.06	0.02

45	->	33	-2.21	0.54
45	->	34	-0.02	0.01
45	->	35	-0.00	0.01
45	->	36	-0.01	0.01
45	->	37	-0.00	0.00
45	->	38	0.00	0.00
45	->	39	-0.00	0.00
45	->	40	-0.01	0.01
45	->	41	-0.04	0.01
45	->	42	-0.21	0.06
45	->	43	-0.23	0.05
45	->	44	-21.25	0.64
45	->	45	24.22	2.59
45	->	46	-10.54	0.60
45	->	47	-1.07	0.16
45	->	48	-2.35	0.31
45	->	49	-2.38	1.46
45	->	50	-0.11	0.06
45	->	51	-0.05	0.03
45	->	52	-0.06	0.03
45	->	53	-0.01	0.01
45	->	54	-0.00	0.00
45	->	55	-0.00	0.00
45	->	56	-0.00	0.00
45	->	57	-0.00	0.00
45	->	58	0.00	0.00
45	->	59	-0.00	0.00
45	->	60	-0.00	0.00
45	->	61	-0.00	0.00
45	->	62	0.00	0.00
45	->	63	0.00	0.00
45	->	64	0.00	0.00
45	->	65	0.00	0.00
45	->	66	0.00	0.00
45	->	67	0.00	0.00
45	->	68	-0.00	0.00
45	->	69	-0.00	0.00
45	->	70	0.00	0.00
45	->	71	-0.00	0.00
45	->	72	0.00	0.01
45	->	73	-0.00	0.00
45	->	74	-0.00	0.00
45	->	75	0.00	0.00
45	->	76	-0.00	0.00
45	->	77	-0.00	0.00
45	->	78	-0.00	0.00
45	->	79	-0.00	0.00
45	->	80	-0.00	0.00
45	->	81	-0.00	0.00
45	->	82	-0.00	0.00

45	->	83	0.00	0.00
45	->	84	0.00	0.00
45	->	85	0.00	0.00
45	->	86	0.00	0.00
45	->	87	-0.00	0.00
45	->	88	-0.00	0.01
45	->	89	-0.00	0.01
45	->	90	0.00	0.01
45	->	91	-0.00	0.01
45	->	92	-0.00	0.01
45	->	93	-0.01	0.01
45	->	94	-0.00	0.02
45	->	95	-0.00	0.00
45	->	96	-0.01	0.01
45	->	97	-0.49	0.47
45	->	98	-3.53	2.02
45	->	99	-0.65	0.78
45	->	100	-0.02	0.02
45	->	101	-0.01	0.00
45	->	102	-0.00	0.00
45	->	103	-0.00	0.00
45	->	104	-0.00	0.00
45	->	105	-0.00	0.00
45	->	106	0.00	0.00
46	->	1	-0.03	0.01
46	->	2	-0.00	0.00
46	->	3	0.00	0.00
46	->	4	0.00	0.00
46	->	5	0.01	0.00
46	->	6	-0.00	0.00
46	->	7	-0.00	0.00
46	->	8	0.00	0.00
46	->	9	0.00	0.00
46	->	10	0.01	0.00
46	->	11	0.01	0.00
46	->	12	-0.03	0.00
46	->	13	-0.01	0.00
46	->	14	-0.00	0.00
46	->	15	-0.00	0.00
46	->	16	0.04	0.01
46	->	17	-0.00	0.00
46	->	18	-0.01	0.01
46	->	19	-0.01	0.01
46	->	20	-0.00	0.00
46	->	21	-0.02	0.01
46	->	22	-0.21	0.09
46	->	23	-0.00	0.00
46	->	24	-0.00	0.00
46	->	25	-0.10	0.05
46	->	26	-0.00	0.00

46	->	27	-0.00	0.00
46	->	28	-0.00	0.00
46	->	29	-0.00	0.00
46	->	30	0.00	0.00
46	->	31	-0.07	0.01
46	->	32	-0.01	0.00
46	->	33	0.01	0.02
46	->	34	-0.01	0.00
46	->	35	0.04	0.01
46	->	36	-0.03	0.01
46	->	37	-0.00	0.00
46	->	38	0.03	0.00
46	->	39	-0.00	0.00
46	->	40	-0.05	0.01
46	->	41	-0.02	0.01
46	->	42	-0.12	0.03
46	->	43	-0.16	0.07
46	->	44	-12.33	4.82
46	->	45	-10.59	0.60
46	->	46	-14.53	4.39
46	->	47	-13.40	0.58
46	->	48	-1.22	0.17
46	->	49	-2.68	0.56
46	->	50	-2.12	1.30
46	->	51	-0.11	0.06
46	->	52	-0.06	0.03
46	->	53	-0.22	0.14
46	->	54	-0.01	0.00
46	->	55	-0.01	0.00
46	->	56	-0.01	0.00
46	->	57	-0.00	0.00
46	->	58	0.03	0.01
46	->	59	-0.03	0.01
46	->	60	-0.01	0.00
46	->	61	-0.01	0.00
46	->	62	0.00	0.00
46	->	63	0.01	0.00
46	->	64	0.01	0.00
46	->	65	0.00	0.00
46	->	66	0.01	0.00
46	->	67	0.00	0.00
46	->	68	-0.02	0.00
46	->	69	-0.02	0.00
46	->	70	-0.00	0.00
46	->	71	-0.01	0.01
46	->	72	-0.33	0.42
46	->	73	-0.01	0.01
46	->	74	0.07	0.02
46	->	75	0.04	0.01
46	->	76	-0.03	0.01

46	->	77	-0.03	0.01
46	->	78	-0.03	0.01
46	->	79	-0.00	0.00
46	->	80	-0.00	0.00
46	->	81	-0.02	0.00
46	->	82	-0.00	0.00
46	->	83	0.00	0.00
46	->	84	0.01	0.00
46	->	85	0.00	0.00
46	->	86	0.02	0.00
46	->	87	0.02	0.01
46	->	88	0.02	0.03
46	->	89	-0.07	0.10
46	->	90	0.22	0.26
46	->	91	0.33	0.19
46	->	92	0.14	0.11
46	->	93	-0.00	0.03
46	->	94	-0.03	0.03
46	->	95	0.01	0.00
46	->	96	0.01	0.01
46	->	97	0.05	0.04
46	->	98	-0.58	0.65
46	->	99	-1.50	0.86
46	->	100	-4.56	1.17
46	->	101	-0.70	0.51
46	->	102	-0.05	0.10
46	->	103	0.01	0.01
46	->	104	0.02	0.00
46	->	105	0.02	0.00
46	->	106	0.01	0.00
47	->	1	0.00	0.00
47	->	2	0.00	0.00
47	->	3	-0.00	0.00
47	->	4	0.00	0.00
47	->	5	-0.00	0.00
47	->	6	0.00	0.00
47	->	7	0.00	0.00
47	->	8	-0.00	0.00
47	->	9	-0.00	0.00
47	->	10	-0.00	0.00
47	->	11	-0.00	0.00
47	->	12	0.00	0.00
47	->	13	0.00	0.00
47	->	14	-0.00	0.00
47	->	15	-0.00	0.00
47	->	16	-0.00	0.00
47	->	17	-0.00	0.00
47	->	18	-0.01	0.00
47	->	19	-0.01	0.00
47	->	20	-0.00	0.00

47	->	21	-0.01	0.00
47	->	22	-0.11	0.07
47	->	23	-0.00	0.00
47	->	24	-0.00	0.00
47	->	25	-0.03	0.02
47	->	26	-0.01	0.01
47	->	27	-0.00	0.00
47	->	28	-0.00	0.00
47	->	29	-0.00	0.00
47	->	30	-0.01	0.00
47	->	31	-0.08	0.09
47	->	32	-0.01	0.00
47	->	33	-0.11	0.06
47	->	34	0.03	0.01
47	->	35	-0.12	0.02
47	->	36	-0.01	0.00
47	->	37	-0.00	0.00
47	->	38	-0.02	0.01
47	->	39	0.00	0.00
47	->	40	-0.01	0.03
47	->	41	0.03	0.03
47	->	42	-2.26	0.38
47	->	43	-1.09	0.78
47	->	44	-4.93	0.61
47	->	45	-1.08	0.16
47	->	46	-13.48	0.59
47	->	47	20.38	1.37
47	->	48	-6.34	0.52
47	->	49	-0.92	0.16
47	->	50	-1.91	0.44
47	->	51	-2.00	0.61
47	->	52	-0.02	0.04
47	->	53	-0.03	0.03
47	->	54	-0.02	0.01
47	->	55	-0.03	0.01
47	->	56	-0.00	0.00
47	->	57	-0.00	0.00
47	->	58	0.00	0.00
47	->	59	-0.00	0.00
47	->	60	-0.00	0.00
47	->	61	-0.00	0.00
47	->	62	0.00	0.00
47	->	63	0.00	0.00
47	->	64	0.00	0.00
47	->	65	0.00	0.00
47	->	66	0.00	0.00
47	->	67	0.00	0.00
47	->	68	-0.00	0.00
47	->	69	-0.00	0.00
47	->	70	-0.00	0.00

47	->	71	-0.01	0.00
47	->	72	-0.15	0.16
47	->	73	-0.00	0.00
47	->	74	-0.00	0.01
47	->	75	-0.00	0.00
47	->	76	-0.00	0.00
47	->	77	0.00	0.00
47	->	78	-0.00	0.00
47	->	79	-0.00	0.00
47	->	80	0.00	0.00
47	->	81	0.00	0.00
47	->	82	0.00	0.00
47	->	83	0.00	0.00
47	->	84	-0.00	0.00
47	->	85	-0.00	0.00
47	->	86	-0.01	0.00
47	->	87	-0.05	0.02
47	->	88	-0.31	0.13
47	->	89	-2.85	0.61
47	->	90	-2.36	1.01
47	->	91	-0.24	0.15
47	->	92	-0.03	0.01
47	->	93	-0.01	0.00
47	->	94	-0.02	0.01
47	->	95	-0.00	0.00
47	->	96	-0.00	0.00
47	->	97	-0.01	0.01
47	->	98	-0.03	0.02
47	->	99	-0.05	0.05
47	->	100	-0.14	0.04
47	->	101	-0.07	0.05
47	->	102	-0.03	0.01
47	->	103	-0.04	0.01
47	->	104	-0.01	0.00
47	->	105	-0.01	0.01
47	->	106	-0.00	0.00
48	->	1	0.00	0.00
48	->	2	0.00	0.00
48	->	3	0.00	0.00
48	->	4	0.00	0.00
48	->	5	-0.00	0.00
48	->	6	0.00	0.00
48	->	7	0.00	0.00
48	->	8	-0.00	0.00
48	->	9	0.00	0.00
48	->	10	-0.00	0.00
48	->	11	-0.00	0.00
48	->	12	0.00	0.00
48	->	13	0.00	0.00
48	->	14	0.00	0.00

48	->	15	0.00	0.00
48	->	16	-0.00	0.00
48	->	17	-0.00	0.00
48	->	18	-0.00	0.00
48	->	19	-0.00	0.00
48	->	20	-0.00	0.00
48	->	21	-0.00	0.00
48	->	22	-0.01	0.00
48	->	23	-0.00	0.00
48	->	24	-0.00	0.00
48	->	25	-0.01	0.00
48	->	26	-0.00	0.00
48	->	27	0.00	0.00
48	->	28	-0.00	0.00
48	->	29	-0.00	0.00
48	->	30	-0.00	0.00
48	->	31	-0.04	0.02
48	->	32	-0.04	0.01
48	->	33	-1.42	0.41
48	->	34	-1.20	0.29
48	->	35	-1.13	0.24
48	->	36	-0.16	0.15
48	->	37	-0.03	0.01
48	->	38	-0.01	0.01
48	->	39	-0.02	0.01
48	->	40	-0.18	0.06
48	->	41	-0.08	0.02
48	->	42	-2.57	0.45
48	->	43	-0.04	0.02
48	->	44	-1.25	0.46
48	->	45	-2.37	0.31
48	->	46	-1.22	0.17
48	->	47	-6.38	0.52
48	->	48	21.84	1.27
48	->	49	-6.35	0.56
48	->	50	-0.98	0.16
48	->	51	-2.89	0.38
48	->	52	-4.12	0.67
48	->	53	-0.18	0.08
48	->	54	-0.07	0.03
48	->	55	-0.07	0.02
48	->	56	-0.02	0.01
48	->	57	-0.00	0.00
48	->	58	-0.00	0.00
48	->	59	-0.00	0.00
48	->	60	-0.00	0.00
48	->	61	-0.00	0.00
48	->	62	-0.00	0.00
48	->	63	0.00	0.00
48	->	64	0.00	0.00

48	->	65	0.00	0.00
48	->	66	0.00	0.00
48	->	67	0.00	0.00
48	->	68	-0.00	0.00
48	->	69	-0.00	0.00
48	->	70	-0.00	0.00
48	->	71	-0.01	0.00
48	->	72	-0.02	0.01
48	->	73	-0.00	0.00
48	->	74	-0.00	0.00
48	->	75	0.00	0.00
48	->	76	-0.00	0.00
48	->	77	-0.00	0.00
48	->	78	-0.00	0.00
48	->	79	-0.00	0.00
48	->	80	-0.00	0.00
48	->	81	-0.00	0.00
48	->	82	-0.00	0.00
48	->	83	0.00	0.00
48	->	84	0.00	0.00
48	->	85	-0.00	0.00
48	->	86	-0.00	0.00
48	->	87	0.00	0.01
48	->	88	0.03	0.05
48	->	89	-0.01	0.01
48	->	90	0.00	0.01
48	->	91	-0.00	0.00
48	->	92	-0.00	0.00
48	->	93	-0.00	0.00
48	->	94	-0.00	0.00
48	->	95	-0.00	0.00
48	->	96	-0.00	0.00
48	->	97	-0.03	0.02
48	->	98	-0.15	0.11
48	->	99	-0.16	0.13
48	->	100	-0.02	0.01
48	->	101	-0.01	0.00
48	->	102	-0.00	0.00
48	->	103	-0.00	0.00
48	->	104	-0.00	0.00
48	->	105	-0.00	0.00
48	->	106	-0.00	0.00
49	->	1	0.00	0.00
49	->	2	0.00	0.00
49	->	3	-0.00	0.00
49	->	4	0.00	0.00
49	->	5	-0.00	0.00
49	->	6	0.00	0.00
49	->	7	0.00	0.00
49	->	8	-0.00	0.00

49	->	9	0.00	0.00
49	->	10	-0.00	0.00
49	->	11	-0.00	0.00
49	->	12	0.00	0.00
49	->	13	0.00	0.00
49	->	14	0.00	0.00
49	->	15	0.00	0.00
49	->	16	-0.00	0.00
49	->	17	-0.00	0.00
49	->	18	-0.00	0.00
49	->	19	-0.00	0.00
49	->	20	-0.00	0.00
49	->	21	-0.00	0.00
49	->	22	0.00	0.00
49	->	23	0.00	0.00
49	->	24	0.00	0.00
49	->	25	0.00	0.00
49	->	26	-0.00	0.00
49	->	27	0.00	0.00
49	->	28	0.00	0.00
49	->	29	0.00	0.00
49	->	30	-0.00	0.00
49	->	31	-0.00	0.00
49	->	32	-0.00	0.00
49	->	33	-0.04	0.01
49	->	34	-0.02	0.00
49	->	35	0.02	0.01
49	->	36	-0.02	0.03
49	->	37	-0.00	0.00
49	->	38	0.00	0.00
49	->	39	-0.00	0.00
49	->	40	-0.01	0.00
49	->	41	-0.01	0.00
49	->	42	-0.06	0.01
49	->	43	-0.00	0.00
49	->	44	-0.04	0.05
49	->	45	-2.40	1.46
49	->	46	-2.70	0.57
49	->	47	-0.93	0.16
49	->	48	-6.36	0.56
49	->	49	-19.80	1.82
49	->	50	-7.91	0.53
49	->	51	-0.91	0.15
49	->	52	-2.69	0.50
49	->	53	-3.59	0.84
49	->	54	-0.17	0.07
49	->	55	-0.06	0.03
49	->	56	-0.03	0.02
49	->	57	-0.01	0.00
49	->	58	-0.03	0.02

49	->	59	-0.00	0.00
49	->	60	-0.01	0.01
49	->	61	-0.02	0.01
49	->	62	-0.00	0.00
49	->	63	0.00	0.00
49	->	64	0.00	0.00
49	->	65	0.00	0.00
49	->	66	0.00	0.00
49	->	67	0.00	0.00
49	->	68	-0.00	0.00
49	->	69	0.00	0.00
49	->	70	0.00	0.00
49	->	71	-0.05	0.04
49	->	72	0.06	0.06
49	->	73	-0.01	0.01
49	->	74	-0.02	0.01
49	->	75	-0.01	0.02
49	->	76	-0.00	0.00
49	->	77	0.00	0.00
49	->	78	-0.00	0.00
49	->	79	-0.00	0.00
49	->	80	-0.00	0.00
49	->	81	0.00	0.00
49	->	82	0.00	0.00
49	->	83	0.00	0.00
49	->	84	0.00	0.00
49	->	85	-0.00	0.00
49	->	86	-0.01	0.00
49	->	87	-0.03	0.02
49	->	88	-0.06	0.05
49	->	89	-0.02	0.01
49	->	90	-0.01	0.00
49	->	91	-0.01	0.00
49	->	92	-0.00	0.00
49	->	93	-0.00	0.00
49	->	94	-0.01	0.00
49	->	95	-0.00	0.00
49	->	96	-0.00	0.00
49	->	97	-0.01	0.01
49	->	98	-0.48	0.45
49	->	99	-2.64	2.12
49	->	100	-0.21	0.16
49	->	101	-0.01	0.01
49	->	102	-0.00	0.00
49	->	103	-0.00	0.00
49	->	104	-0.00	0.00
49	->	105	-0.00	0.00
49	->	106	-0.00	0.00
50	->	1	0.00	0.00
50	->	2	0.00	0.00

50	->	3	-0.00	0.00
50	->	4	-0.00	0.00
50	->	5	-0.00	0.00
50	->	6	0.00	0.00
50	->	7	0.00	0.00
50	->	8	-0.00	0.00
50	->	9	0.00	0.00
50	->	10	-0.00	0.00
50	->	11	-0.00	0.00
50	->	12	0.00	0.00
50	->	13	0.00	0.00
50	->	14	0.00	0.00
50	->	15	0.00	0.00
50	->	16	-0.00	0.00
50	->	17	-0.00	0.00
50	->	18	-0.00	0.00
50	->	19	-0.00	0.00
50	->	20	-0.00	0.00
50	->	21	-0.00	0.00
50	->	22	0.00	0.00
50	->	23	0.00	0.00
50	->	24	0.00	0.00
50	->	25	0.00	0.00
50	->	26	-0.00	0.00
50	->	27	0.00	0.00
50	->	28	0.00	0.00
50	->	29	0.00	0.00
50	->	30	-0.00	0.00
50	->	31	0.00	0.01
50	->	32	-0.00	0.00
50	->	33	-0.01	0.00
50	->	34	0.00	0.01
50	->	35	-0.07	0.02
50	->	36	-0.01	0.00
50	->	37	-0.01	0.00
50	->	38	-0.02	0.01
50	->	39	-0.00	0.00
50	->	40	0.02	0.02
50	->	41	0.01	0.01
50	->	42	-0.15	0.06
50	->	43	-0.01	0.00
50	->	44	-0.06	0.04
50	->	45	-0.11	0.06
50	->	46	-2.13	1.30
50	->	47	-1.92	0.44
50	->	48	-0.99	0.16
50	->	49	-7.94	0.53
50	->	50	-20.79	2.92
50	->	51	-21.69	0.48
50	->	52	-0.86	0.16

50	->	53	-3.55	0.79
50	->	54	-2.53	1.51
50	->	55	-0.15	0.07
50	->	56	-0.04	0.02
50	->	57	-0.03	0.01
50	->	58	-0.02	0.01
50	->	59	-0.04	0.02
50	->	60	-0.02	0.01
50	->	61	-0.01	0.01
50	->	62	-0.00	0.00
50	->	63	0.00	0.00
50	->	64	0.00	0.00
50	->	65	0.00	0.00
50	->	66	0.00	0.00
50	->	67	0.00	0.00
50	->	68	-0.00	0.00
50	->	69	-0.01	0.00
50	->	70	-0.03	0.02
50	->	71	-0.36	0.25
50	->	72	-2.09	1.67
50	->	73	0.00	0.01
50	->	74	0.01	0.04
50	->	75	-0.03	0.04
50	->	76	-0.00	0.00
50	->	77	-0.00	0.00
50	->	78	-0.00	0.00
50	->	79	-0.00	0.00
50	->	80	0.00	0.00
50	->	81	0.00	0.00
50	->	82	-0.00	0.00
50	->	83	0.00	0.00
50	->	84	0.00	0.00
50	->	85	-0.00	0.00
50	->	86	-0.02	0.02
50	->	87	-0.45	0.50
50	->	88	-0.76	0.85
50	->	89	-0.35	0.26
50	->	90	-0.02	0.01
50	->	91	-0.01	0.01
50	->	92	-0.01	0.00
50	->	93	-0.00	0.00
50	->	94	-0.00	0.00
50	->	95	-0.00	0.00
50	->	96	-0.00	0.00
50	->	97	-0.00	0.00
50	->	98	-0.00	0.01
50	->	99	-0.01	0.05
50	->	100	-0.02	0.04
50	->	101	-0.03	0.01
50	->	102	-0.02	0.01

50 ->	103	-0.02	0.01
50 ->	104	-0.01	0.00
50 ->	105	-0.00	0.00
50 ->	106	-0.00	0.00
51 ->	1	0.00	0.00
51 ->	2	0.00	0.00
51 ->	3	-0.00	0.00
51 ->	4	-0.00	0.00
51 ->	5	-0.00	0.00
51 ->	6	0.00	0.00
51 ->	7	0.00	0.00
51 ->	8	-0.00	0.00
51 ->	9	0.00	0.00
51 ->	10	-0.00	0.00
51 ->	11	-0.00	0.00
51 ->	12	0.00	0.00
51 ->	13	0.00	0.00
51 ->	14	0.00	0.00
51 ->	15	0.00	0.00
51 ->	16	-0.00	0.00
51 ->	17	-0.00	0.00
51 ->	18	-0.00	0.00
51 ->	19	-0.00	0.00
51 ->	20	-0.00	0.00
51 ->	21	-0.00	0.00
51 ->	22	0.00	0.00
51 ->	23	0.00	0.00
51 ->	24	0.00	0.00
51 ->	25	0.00	0.00
51 ->	26	-0.00	0.00
51 ->	27	0.00	0.00
51 ->	28	0.00	0.00
51 ->	29	0.00	0.00
51 ->	30	-0.00	0.00
51 ->	31	0.00	0.00
51 ->	32	-0.00	0.00
51 ->	33	-0.02	0.01
51 ->	34	-0.12	0.03
51 ->	35	2.77	0.73
51 ->	36	-0.11	0.05
51 ->	37	0.46	0.42
51 ->	38	3.30	0.75
51 ->	39	-0.10	0.04
51 ->	40	-1.40	0.61
51 ->	41	-0.02	0.01
51 ->	42	-2.01	0.30
51 ->	43	-0.01	0.01
51 ->	44	-0.01	0.01
51 ->	45	-0.05	0.03
51 ->	46	-0.11	0.06

51	->	47	-2.04	0.60
51	->	48	-2.89	0.38
51	->	49	-0.92	0.15
51	->	50	-21.73	0.49
51	->	51	11.11	1.28
51	->	52	-10.26	0.51
51	->	53	-1.24	0.24
51	->	54	-2.81	0.84
51	->	55	20.10	11.65
51	->	56	-0.21	0.08
51	->	57	-0.03	0.02
51	->	58	-0.02	0.01
51	->	59	-0.01	0.01
51	->	60	-0.00	0.00
51	->	61	-0.00	0.00
51	->	62	-0.00	0.00
51	->	63	-0.00	0.00
51	->	64	-0.00	0.00
51	->	65	0.00	0.00
51	->	66	-0.00	0.00
51	->	67	0.00	0.00
51	->	68	-0.00	0.00
51	->	69	-0.00	0.00
51	->	70	-0.01	0.00
51	->	71	-0.02	0.01
51	->	72	-0.07	0.04
51	->	73	0.00	0.00
51	->	74	-0.00	0.00
51	->	75	-0.00	0.01
51	->	76	0.00	0.00
51	->	77	0.00	0.00
51	->	78	0.00	0.00
51	->	79	0.00	0.00
51	->	80	0.00	0.00
51	->	81	0.00	0.00
51	->	82	0.00	0.00
51	->	83	0.00	0.00
51	->	84	-0.00	0.00
51	->	85	-0.00	0.00
51	->	86	-0.02	0.01
51	->	87	-0.65	0.75
51	->	88	-3.06	3.48
51	->	89	-0.06	0.02
51	->	90	-0.01	0.00
51	->	91	-0.01	0.00
51	->	92	-0.00	0.00
51	->	93	-0.00	0.00
51	->	94	-0.00	0.00
51	->	95	-0.00	0.00
51	->	96	-0.00	0.00

51	->	97	-0.00	0.00
51	->	98	-0.01	0.01
51	->	99	-0.02	0.01
51	->	100	-0.00	0.00
51	->	101	-0.00	0.00
51	->	102	-0.00	0.00
51	->	103	-0.01	0.00
51	->	104	-0.00	0.00
51	->	105	-0.01	0.00
51	->	106	-0.00	0.00
52	->	1	0.00	0.00
52	->	2	0.00	0.00
52	->	3	0.00	0.00
52	->	4	0.00	0.00
52	->	5	-0.00	0.00
52	->	6	0.00	0.00
52	->	7	0.00	0.00
52	->	8	-0.00	0.00
52	->	9	0.00	0.00
52	->	10	-0.00	0.00
52	->	11	-0.00	0.00
52	->	12	0.00	0.00
52	->	13	0.00	0.00
52	->	14	0.00	0.00
52	->	15	0.00	0.00
52	->	16	-0.00	0.00
52	->	17	-0.00	0.00
52	->	18	-0.00	0.00
52	->	19	-0.00	0.00
52	->	20	0.00	0.00
52	->	21	-0.00	0.00
52	->	22	0.00	0.00
52	->	23	0.00	0.00
52	->	24	0.00	0.00
52	->	25	0.00	0.00
52	->	26	0.00	0.00
52	->	27	0.00	0.00
52	->	28	-0.00	0.00
52	->	29	0.00	0.00
52	->	30	-0.00	0.00
52	->	31	-0.00	0.00
52	->	32	-0.00	0.00
52	->	33	-0.02	0.00
52	->	34	-0.04	0.01
52	->	35	-0.32	0.22
52	->	36	-0.51	0.88
52	->	37	-0.15	0.23
52	->	38	0.04	0.02
52	->	39	-0.01	0.00
52	->	40	-0.05	0.02

52	->	41	-0.01	0.00
52	->	42	-0.06	0.02
52	->	43	-0.00	0.00
52	->	44	-0.00	0.00
52	->	45	-0.06	0.03
52	->	46	-0.06	0.03
52	->	47	-0.02	0.04
52	->	48	-4.09	0.66
52	->	49	-2.71	0.51
52	->	50	-0.86	0.16
52	->	51	-10.31	0.52
52	->	52	23.34	1.69
52	->	53	-10.23	0.50
52	->	54	-1.05	0.24
52	->	55	-3.04	0.64
52	->	56	-4.68	1.82
52	->	57	-0.18	0.09
52	->	58	-0.26	0.12
52	->	59	-0.01	0.01
52	->	60	-0.01	0.00
52	->	61	-0.01	0.00
52	->	62	-0.00	0.00
52	->	63	0.00	0.00
52	->	64	0.00	0.00
52	->	65	0.00	0.00
52	->	66	0.00	0.00
52	->	67	0.00	0.00
52	->	68	-0.00	0.00
52	->	69	-0.00	0.00
52	->	70	0.00	0.00
52	->	71	-0.02	0.01
52	->	72	-0.01	0.01
52	->	73	-0.00	0.00
52	->	74	-0.00	0.00
52	->	75	-0.00	0.01
52	->	76	-0.00	0.00
52	->	77	0.00	0.00
52	->	78	-0.00	0.00
52	->	79	0.00	0.00
52	->	80	0.00	0.00
52	->	81	0.00	0.00
52	->	82	0.00	0.00
52	->	83	0.00	0.00
52	->	84	0.00	0.00
52	->	85	-0.00	0.00
52	->	86	-0.00	0.00
52	->	87	-0.00	0.02
52	->	88	0.05	0.06
52	->	89	-0.01	0.00
52	->	90	-0.00	0.00

52 ->	91	-0.00	0.00
52 ->	92	-0.00	0.00
52 ->	93	-0.00	0.00
52 ->	94	-0.00	0.00
52 ->	95	-0.00	0.00
52 ->	96	-0.00	0.00
52 ->	97	-0.01	0.00
52 ->	98	-0.03	0.02
52 ->	99	-0.08	0.07
52 ->	100	-0.02	0.01
52 ->	101	-0.00	0.00
52 ->	102	-0.00	0.00
52 ->	103	-0.00	0.00
52 ->	104	-0.00	0.00
52 ->	105	-0.00	0.00
52 ->	106	-0.00	0.00
53 ->	1	0.01	0.00
53 ->	2	0.00	0.00
53 ->	3	-0.00	0.00
53 ->	4	-0.00	0.00
53 ->	5	-0.00	0.00
53 ->	6	0.00	0.00
53 ->	7	0.00	0.00
53 ->	8	-0.00	0.00
53 ->	9	-0.00	0.00
53 ->	10	-0.00	0.00
53 ->	11	-0.00	0.00
53 ->	12	0.01	0.00
53 ->	13	0.00	0.00
53 ->	14	0.00	0.00
53 ->	15	0.00	0.00
53 ->	16	-0.01	0.00
53 ->	17	-0.00	0.00
53 ->	18	-0.00	0.00
53 ->	19	-0.00	0.00
53 ->	20	-0.00	0.00
53 ->	21	0.00	0.00
53 ->	22	0.02	0.00
53 ->	23	0.00	0.00
53 ->	24	0.00	0.00
53 ->	25	0.02	0.00
53 ->	26	0.00	0.00
53 ->	27	0.00	0.00
53 ->	28	0.00	0.00
53 ->	29	0.00	0.00
53 ->	30	-0.00	0.00
53 ->	31	0.02	0.00
53 ->	32	-0.00	0.00
53 ->	33	-0.00	0.00
53 ->	34	-0.00	0.00

53	->	35	-0.05	0.02
53	->	36	0.02	0.01
53	->	37	-0.01	0.01
53	->	38	-0.04	0.01
53	->	39	-0.00	0.00
53	->	40	0.03	0.01
53	->	41	-0.00	0.00
53	->	42	-0.02	0.01
53	->	43	-0.00	0.00
53	->	44	0.05	0.01
53	->	45	-0.01	0.01
53	->	46	-0.22	0.14
53	->	47	-0.03	0.03
53	->	48	-0.18	0.08
53	->	49	-3.63	0.84
53	->	50	-3.57	0.78
53	->	51	-1.25	0.24
53	->	52	-10.26	0.51
53	->	53	-103.35	4.18
53	->	54	-5.24	0.54
53	->	55	-0.63	0.20
53	->	56	-1.44	0.30
53	->	57	-1.26	0.36
53	->	58	-3.85	0.99
53	->	59	0.02	0.15
53	->	60	-1.12	0.62
53	->	61	-0.50	0.36
53	->	62	-0.01	0.01
53	->	63	-0.03	0.01
53	->	64	-0.02	0.00
53	->	65	-0.00	0.00
53	->	66	-0.02	0.00
53	->	67	-0.00	0.00
53	->	68	0.03	0.01
53	->	69	0.08	0.03
53	->	70	0.05	0.05
53	->	71	-3.24	1.99
53	->	72	-0.32	0.27
53	->	73	-0.16	0.10
53	->	74	-0.13	0.05
53	->	75	-0.19	0.13
53	->	76	0.04	0.01
53	->	77	0.03	0.01
53	->	78	0.03	0.01
53	->	79	0.00	0.00
53	->	80	0.00	0.00
53	->	81	0.01	0.00
53	->	82	0.00	0.00
53	->	83	-0.00	0.00
53	->	84	-0.01	0.00

53 ->	85	-0.01	0.01
53 ->	86	-0.10	0.05
53 ->	87	-0.24	0.12
53 ->	88	-0.13	0.06
53 ->	89	-0.04	0.01
53 ->	90	-0.03	0.00
53 ->	91	-0.02	0.00
53 ->	92	-0.02	0.00
53 ->	93	-0.02	0.00
53 ->	94	-0.01	0.00
53 ->	95	-0.01	0.00
53 ->	96	-0.01	0.00
53 ->	97	-0.03	0.01
53 ->	98	-0.14	0.09
53 ->	99	-1.93	1.52
53 ->	100	-7.03	3.79
53 ->	101	-0.09	0.03
53 ->	102	-0.03	0.00
53 ->	103	-0.02	0.00
53 ->	104	-0.01	0.00
53 ->	105	-0.01	0.00
53 ->	106	-0.01	0.00
54 ->	1	0.00	0.00
54 ->	2	0.00	0.00
54 ->	3	0.00	0.00
54 ->	4	0.00	0.00
54 ->	5	-0.00	0.00
54 ->	6	0.00	0.00
54 ->	7	0.00	0.00
54 ->	8	-0.00	0.00
54 ->	9	0.00	0.00
54 ->	10	-0.00	0.00
54 ->	11	-0.00	0.00
54 ->	12	0.00	0.00
54 ->	13	0.00	0.00
54 ->	14	0.00	0.00
54 ->	15	0.00	0.00
54 ->	16	-0.00	0.00
54 ->	17	-0.00	0.00
54 ->	18	-0.00	0.00
54 ->	19	-0.00	0.00
54 ->	20	0.00	0.00
54 ->	21	0.00	0.00
54 ->	22	0.00	0.00
54 ->	23	0.00	0.00
54 ->	24	0.00	0.00
54 ->	25	0.00	0.00
54 ->	26	0.00	0.00
54 ->	27	0.00	0.00
54 ->	28	0.00	0.00

54 ->	29	0.00	0.00
54 ->	30	-0.00	0.00
54 ->	31	0.00	0.00
54 ->	32	0.00	0.00
54 ->	33	-0.00	0.00
54 ->	34	-0.00	0.00
54 ->	35	-0.06	0.05
54 ->	36	-0.00	0.00
54 ->	37	-0.03	0.02
54 ->	38	-0.04	0.03
54 ->	39	-0.00	0.00
54 ->	40	-0.03	0.02
54 ->	41	-0.00	0.00
54 ->	42	-0.02	0.01
54 ->	43	-0.00	0.00
54 ->	44	-0.00	0.00
54 ->	45	-0.00	0.00
54 ->	46	-0.01	0.00
54 ->	47	-0.02	0.01
54 ->	48	-0.07	0.03
54 ->	49	-0.17	0.07
54 ->	50	-2.54	1.51
54 ->	51	-2.82	0.84
54 ->	52	-1.05	0.24
54 ->	53	-5.28	0.54
54 ->	54	-23.24	1.69
54 ->	55	-22.32	0.34
54 ->	56	-0.62	0.17
54 ->	57	-1.00	0.38
54 ->	58	-0.19	0.10
54 ->	59	-0.26	0.24
54 ->	60	-0.01	0.01
54 ->	61	-0.00	0.00
54 ->	62	-0.00	0.00
54 ->	63	-0.00	0.00
54 ->	64	-0.00	0.00
54 ->	65	-0.00	0.00
54 ->	66	0.00	0.00
54 ->	67	-0.00	0.00
54 ->	68	-0.01	0.01
54 ->	69	-0.02	0.01
54 ->	70	-0.03	0.01
54 ->	71	-0.36	0.30
54 ->	72	-0.07	0.05
54 ->	73	0.00	0.00
54 ->	74	-0.00	0.00
54 ->	75	-0.02	0.04
54 ->	76	-0.00	0.00
54 ->	77	0.00	0.00
54 ->	78	-0.00	0.00

54	->	79	-0.00	0.00
54	->	80	0.00	0.00
54	->	81	0.00	0.00
54	->	82	-0.00	0.00
54	->	83	0.00	0.00
54	->	84	0.00	0.00
54	->	85	-0.01	0.01
54	->	86	-0.14	0.23
54	->	87	-2.13	1.23
54	->	88	-0.04	0.15
54	->	89	-0.01	0.00
54	->	90	-0.00	0.00
54	->	91	-0.00	0.00
54	->	92	-0.00	0.00
54	->	93	-0.00	0.00
54	->	94	-0.00	0.00
54	->	95	-0.00	0.00
54	->	96	-0.00	0.00
54	->	97	-0.00	0.00
54	->	98	-0.00	0.00
54	->	99	-0.00	0.00
54	->	100	-0.00	0.00
54	->	101	-0.00	0.00
54	->	102	-0.00	0.00
54	->	103	-0.00	0.00
54	->	104	-0.00	0.00
54	->	105	-0.01	0.00
54	->	106	-0.00	0.00
55	->	1	0.00	0.00
55	->	2	0.00	0.00
55	->	3	0.00	0.00
55	->	4	0.00	0.00
55	->	5	0.00	0.00
55	->	6	0.00	0.00
55	->	7	0.00	0.00
55	->	8	0.00	0.00
55	->	9	-0.00	0.00
55	->	10	0.00	0.00
55	->	11	-0.00	0.00
55	->	12	0.00	0.00
55	->	13	0.00	0.00
55	->	14	0.00	0.00
55	->	15	0.00	0.00
55	->	16	-0.00	0.00
55	->	17	0.00	0.00
55	->	18	0.00	0.00
55	->	19	-0.00	0.00
55	->	20	0.00	0.00
55	->	21	0.00	0.00
55	->	22	0.00	0.00

55	->	23	0.00	0.00
55	->	24	0.00	0.00
55	->	25	-0.00	0.00
55	->	26	0.00	0.00
55	->	27	0.00	0.00
55	->	28	0.00	0.00
55	->	29	0.00	0.00
55	->	30	-0.00	0.00
55	->	31	-0.00	0.00
55	->	32	-0.00	0.00
55	->	33	-0.01	0.00
55	->	34	-0.05	0.01
55	->	35	2.10	0.59
55	->	36	-0.20	0.12
55	->	37	0.60	0.72
55	->	38	1.81	0.42
55	->	39	-0.03	0.02
55	->	40	-0.22	0.07
55	->	41	-0.01	0.00
55	->	42	-0.01	0.01
55	->	43	0.00	0.00
55	->	44	-0.00	0.00
55	->	45	-0.00	0.00
55	->	46	-0.01	0.00
55	->	47	-0.03	0.01
55	->	48	-0.07	0.02
55	->	49	-0.06	0.03
55	->	50	-0.15	0.07
55	->	51	20.09	11.66
55	->	52	-3.02	0.65
55	->	53	-0.63	0.19
55	->	54	-22.42	0.34
55	->	55	3.09	1.25
55	->	56	-5.02	0.43
55	->	57	-0.51	0.14
55	->	58	-0.05	0.03
55	->	59	-0.04	0.02
55	->	60	-0.00	0.00
55	->	61	-0.00	0.00
55	->	62	0.00	0.00
55	->	63	-0.00	0.00
55	->	64	-0.00	0.00
55	->	65	-0.00	0.00
55	->	66	-0.00	0.00
55	->	67	-0.00	0.00
55	->	68	-0.00	0.00
55	->	69	-0.00	0.00
55	->	70	-0.01	0.00
55	->	71	-0.01	0.00
55	->	72	-0.00	0.00

55	->	73	-0.00	0.00
55	->	74	-0.00	0.00
55	->	75	-0.00	0.00
55	->	76	0.00	0.00
55	->	77	0.00	0.00
55	->	78	0.00	0.00
55	->	79	0.00	0.00
55	->	80	0.00	0.00
55	->	81	0.00	0.00
55	->	82	0.00	0.00
55	->	83	0.00	0.00
55	->	84	-0.00	0.00
55	->	85	-0.00	0.00
55	->	86	0.00	0.01
55	->	87	-0.03	0.07
55	->	88	0.02	0.05
55	->	89	-0.00	0.00
55	->	90	-0.00	0.00
55	->	91	-0.00	0.00
55	->	92	-0.00	0.00
55	->	93	-0.00	0.00
55	->	94	-0.00	0.00
55	->	95	-0.00	0.00
55	->	96	-0.00	0.00
55	->	97	-0.00	0.00
55	->	98	-0.00	0.00
55	->	99	-0.01	0.00
55	->	100	-0.00	0.00
55	->	101	-0.00	0.00
55	->	102	-0.00	0.00
55	->	103	-0.00	0.00
55	->	104	-0.00	0.00
55	->	105	-0.00	0.00
55	->	106	-0.00	0.00
56	->	1	0.00	0.00
56	->	2	0.00	0.00
56	->	3	0.00	0.00
56	->	4	0.00	0.00
56	->	5	-0.00	0.00
56	->	6	0.00	0.00
56	->	7	0.00	0.00
56	->	8	-0.00	0.00
56	->	9	0.00	0.00
56	->	10	-0.00	0.00
56	->	11	-0.00	0.00
56	->	12	0.00	0.00
56	->	13	0.00	0.00
56	->	14	0.00	0.00
56	->	15	0.00	0.00
56	->	16	-0.00	0.00

56	->	17	-0.00	0.00
56	->	18	-0.00	0.00
56	->	19	-0.00	0.00
56	->	20	-0.00	0.00
56	->	21	0.00	0.00
56	->	22	0.00	0.00
56	->	23	0.00	0.00
56	->	24	0.00	0.00
56	->	25	0.00	0.00
56	->	26	0.00	0.00
56	->	27	0.00	0.00
56	->	28	0.00	0.00
56	->	29	0.00	0.00
56	->	30	-0.00	0.00
56	->	31	0.00	0.00
56	->	32	-0.00	0.00
56	->	33	-0.00	0.00
56	->	34	-0.00	0.00
56	->	35	-0.02	0.01
56	->	36	-0.03	0.09
56	->	37	-0.03	0.04
56	->	38	-0.01	0.00
56	->	39	-0.00	0.00
56	->	40	0.00	0.00
56	->	41	-0.00	0.00
56	->	42	-0.00	0.00
56	->	43	0.00	0.00
56	->	44	0.00	0.00
56	->	45	-0.00	0.00
56	->	46	-0.01	0.00
56	->	47	-0.00	0.00
56	->	48	-0.02	0.01
56	->	49	-0.03	0.02
56	->	50	-0.04	0.02
56	->	51	-0.21	0.08
56	->	52	-4.69	1.82
56	->	53	-1.45	0.30
56	->	54	-0.63	0.16
56	->	55	-5.18	0.43
56	->	56	-24.23	2.47
56	->	57	-11.50	0.32
56	->	58	-2.44	1.17
56	->	59	-0.04	0.02
56	->	60	-0.01	0.00
56	->	61	-0.00	0.00
56	->	62	-0.00	0.00
56	->	63	0.00	0.00
56	->	64	0.00	0.00
56	->	65	0.00	0.00
56	->	66	0.00	0.00

56	->	67	0.00	0.00
56	->	68	-0.00	0.00
56	->	69	-0.01	0.00
56	->	70	-0.00	0.00
56	->	71	-0.01	0.00
56	->	72	-0.00	0.00
56	->	73	-0.00	0.00
56	->	74	-0.00	0.00
56	->	75	-0.00	0.00
56	->	76	-0.00	0.00
56	->	77	0.00	0.00
56	->	78	0.00	0.00
56	->	79	0.00	0.00
56	->	80	0.00	0.00
56	->	81	0.00	0.00
56	->	82	-0.00	0.00
56	->	83	0.00	0.00
56	->	84	0.00	0.00
56	->	85	-0.00	0.00
56	->	86	-0.01	0.00
56	->	87	-0.02	0.01
56	->	88	-0.01	0.00
56	->	89	-0.00	0.00
56	->	90	-0.00	0.00
56	->	91	-0.00	0.00
56	->	92	-0.00	0.00
56	->	93	-0.00	0.00
56	->	94	-0.00	0.00
56	->	95	-0.00	0.00
56	->	96	-0.00	0.00
56	->	97	-0.00	0.00
56	->	98	-0.00	0.00
56	->	99	-0.01	0.00
56	->	100	-0.00	0.00
56	->	101	-0.00	0.00
56	->	102	-0.00	0.00
56	->	103	-0.00	0.00
56	->	104	-0.00	0.00
56	->	105	-0.00	0.00
56	->	106	-0.00	0.00
57	->	1	0.00	0.00
57	->	2	0.00	0.00
57	->	3	0.00	0.00
57	->	4	0.00	0.00
57	->	5	-0.00	0.00
57	->	6	0.00	0.00
57	->	7	0.00	0.00
57	->	8	-0.00	0.00
57	->	9	0.00	0.00
57	->	10	-0.00	0.00

57	->	11	-0.00	0.00
57	->	12	0.00	0.00
57	->	13	0.00	0.00
57	->	14	0.00	0.00
57	->	15	0.00	0.00
57	->	16	-0.00	0.00
57	->	17	0.00	0.00
57	->	18	0.00	0.00
57	->	19	0.00	0.00
57	->	20	-0.00	0.00
57	->	21	0.00	0.00
57	->	22	0.00	0.00
57	->	23	0.00	0.00
57	->	24	0.00	0.00
57	->	25	0.00	0.00
57	->	26	0.00	0.00
57	->	27	0.00	0.00
57	->	28	0.00	0.00
57	->	29	0.00	0.00
57	->	30	-0.00	0.00
57	->	31	0.00	0.00
57	->	32	-0.00	0.00
57	->	33	-0.00	0.00
57	->	34	0.00	0.00
57	->	35	-0.00	0.00
57	->	36	0.00	0.00
57	->	37	-0.00	0.00
57	->	38	-0.00	0.00
57	->	39	0.00	0.00
57	->	40	0.00	0.00
57	->	41	-0.00	0.00
57	->	42	-0.00	0.00
57	->	43	0.00	0.00
57	->	44	0.00	0.00
57	->	45	-0.00	0.00
57	->	46	-0.00	0.00
57	->	47	-0.00	0.00
57	->	48	-0.00	0.00
57	->	49	-0.01	0.00
57	->	50	-0.03	0.01
57	->	51	-0.03	0.02
57	->	52	-0.18	0.09
57	->	53	-1.28	0.36
57	->	54	-1.04	0.39
57	->	55	-0.53	0.13
57	->	56	-11.58	0.32
57	->	57	12.38	0.60
57	->	58	-14.86	0.61
57	->	59	-1.28	0.63
57	->	60	-0.03	0.00

57	->	61	-0.01	0.00
57	->	62	-0.00	0.00
57	->	63	0.00	0.00
57	->	64	0.00	0.00
57	->	65	0.00	0.00
57	->	66	0.00	0.00
57	->	67	-0.00	0.00
57	->	68	-0.00	0.00
57	->	69	-0.03	0.02
57	->	70	-0.00	0.01
57	->	71	-0.03	0.01
57	->	72	-0.01	0.00
57	->	73	-0.00	0.00
57	->	74	-0.00	0.00
57	->	75	-0.01	0.00
57	->	76	-0.00	0.00
57	->	77	0.00	0.00
57	->	78	0.00	0.00
57	->	79	-0.00	0.00
57	->	80	0.00	0.00
57	->	81	0.00	0.00
57	->	82	-0.00	0.00
57	->	83	0.00	0.00
57	->	84	0.00	0.00
57	->	85	-0.00	0.00
57	->	86	-0.02	0.01
57	->	87	-0.04	0.02
57	->	88	-0.01	0.00
57	->	89	-0.00	0.00
57	->	90	-0.00	0.00
57	->	91	-0.00	0.00
57	->	92	-0.00	0.00
57	->	93	-0.00	0.00
57	->	94	-0.00	0.00
57	->	95	-0.00	0.00
57	->	96	-0.00	0.00
57	->	97	-0.00	0.00
57	->	98	-0.00	0.00
57	->	99	-0.00	0.00
57	->	100	-0.00	0.00
57	->	101	-0.00	0.00
57	->	102	-0.00	0.00
57	->	103	-0.00	0.00
57	->	104	-0.00	0.00
57	->	105	-0.00	0.00
57	->	106	-0.00	0.00
58	->	1	-0.01	0.00
58	->	2	-0.00	0.00
58	->	3	0.00	0.00
58	->	4	0.00	0.00

58	->	5	0.00	0.00
58	->	6	-0.00	0.00
58	->	7	-0.00	0.00
58	->	8	0.00	0.00
58	->	9	0.00	0.00
58	->	10	0.00	0.00
58	->	11	0.00	0.00
58	->	12	-0.00	0.00
58	->	13	-0.00	0.00
58	->	14	-0.00	0.00
58	->	15	-0.00	0.00
58	->	16	0.01	0.00
58	->	17	0.00	0.00
58	->	18	0.00	0.00
58	->	19	0.00	0.00
58	->	20	0.00	0.00
58	->	21	-0.00	0.00
58	->	22	-0.01	0.00
58	->	23	-0.00	0.00
58	->	24	-0.00	0.00
58	->	25	-0.01	0.00
58	->	26	-0.00	0.00
58	->	27	-0.00	0.00
58	->	28	-0.00	0.00
58	->	29	-0.00	0.00
58	->	30	0.00	0.00
58	->	31	-0.01	0.00
58	->	32	0.00	0.00
58	->	33	-0.00	0.00
58	->	34	0.00	0.00
58	->	35	0.03	0.01
58	->	36	-0.04	0.02
58	->	37	-0.00	0.00
58	->	38	0.02	0.00
58	->	39	-0.00	0.00
58	->	40	-0.02	0.00
58	->	41	0.00	0.00
58	->	42	-0.00	0.00
58	->	43	-0.00	0.00
58	->	44	-0.02	0.00
58	->	45	0.00	0.00
58	->	46	0.03	0.01
58	->	47	0.00	0.00
58	->	48	-0.00	0.00
58	->	49	-0.03	0.02
58	->	50	-0.02	0.01
58	->	51	-0.02	0.01
58	->	52	-0.26	0.12
58	->	53	-3.83	0.98
58	->	54	-0.20	0.10

58 ->	55	-0.05	0.03
58 ->	56	-2.46	1.14
58 ->	57	-15.00	0.62
58 ->	58	-63.60	2.43
58 ->	59	-11.77	0.51
58 ->	60	-1.23	0.33
58 ->	61	-0.09	0.02
58 ->	62	-0.01	0.00
58 ->	63	0.02	0.00
58 ->	64	0.01	0.00
58 ->	65	0.00	0.00
58 ->	66	0.02	0.00
58 ->	67	0.00	0.00
58 ->	68	-0.03	0.01
58 ->	69	-0.05	0.07
58 ->	70	-0.26	0.09
58 ->	71	-0.64	0.26
58 ->	72	-0.03	0.01
58 ->	73	-0.01	0.00
58 ->	74	0.03	0.00
58 ->	75	0.05	0.01
58 ->	76	-0.04	0.01
58 ->	77	-0.02	0.00
58 ->	78	-0.02	0.00
58 ->	79	-0.00	0.00
58 ->	80	-0.00	0.00
58 ->	81	-0.01	0.00
58 ->	82	-0.00	0.00
58 ->	83	0.00	0.00
58 ->	84	0.01	0.00
58 ->	85	0.00	0.00
58 ->	86	0.02	0.01
58 ->	87	0.03	0.01
58 ->	88	0.03	0.00
58 ->	89	0.01	0.00
58 ->	90	0.01	0.00
58 ->	91	0.01	0.00
58 ->	92	0.01	0.00
58 ->	93	0.01	0.00
58 ->	94	0.00	0.00
58 ->	95	0.01	0.00
58 ->	96	0.00	0.00
58 ->	97	0.01	0.00
58 ->	98	0.03	0.01
58 ->	99	0.04	0.02
58 ->	100	0.04	0.02
58 ->	101	0.02	0.01
58 ->	102	0.01	0.00
58 ->	103	0.01	0.00
58 ->	104	0.00	0.00

58	->	105	0.00	0.00
58	->	106	0.00	0.00
59	->	1	0.01	0.00
59	->	2	0.00	0.00
59	->	3	-0.00	0.00
59	->	4	0.00	0.00
59	->	5	-0.00	0.00
59	->	6	0.00	0.00
59	->	7	0.00	0.00
59	->	8	-0.00	0.00
59	->	9	-0.00	0.00
59	->	10	-0.00	0.00
59	->	11	-0.00	0.00
59	->	12	0.00	0.00
59	->	13	0.00	0.00
59	->	14	0.00	0.00
59	->	15	0.00	0.00
59	->	16	-0.00	0.00
59	->	17	-0.00	0.00
59	->	18	-0.00	0.00
59	->	19	-0.00	0.00
59	->	20	-0.00	0.00
59	->	21	0.00	0.00
59	->	22	0.01	0.00
59	->	23	0.00	0.00
59	->	24	0.00	0.00
59	->	25	0.01	0.00
59	->	26	0.00	0.00
59	->	27	0.00	0.00
59	->	28	0.00	0.00
59	->	29	0.00	0.00
59	->	30	-0.00	0.00
59	->	31	0.01	0.00
59	->	32	-0.00	0.00
59	->	33	0.00	0.00
59	->	34	0.00	0.00
59	->	35	-0.03	0.00
59	->	36	0.02	0.01
59	->	37	-0.00	0.00
59	->	38	-0.03	0.00
59	->	39	0.00	0.00
59	->	40	0.03	0.01
59	->	41	-0.00	0.00
59	->	42	-0.00	0.00
59	->	43	0.00	0.00
59	->	44	0.02	0.00
59	->	45	-0.00	0.00
59	->	46	-0.03	0.01
59	->	47	-0.00	0.00
59	->	48	-0.00	0.00

59	->	49	-0.00	0.00
59	->	50	-0.04	0.02
59	->	51	-0.01	0.01
59	->	52	-0.01	0.01
59	->	53	0.02	0.15
59	->	54	-0.26	0.23
59	->	55	-0.04	0.02
59	->	56	-0.04	0.02
59	->	57	-1.36	0.64
59	->	58	-11.83	0.52
59	->	59	12.30	2.85
59	->	60	-0.34	0.35
59	->	61	-0.83	0.28
59	->	62	-0.15	0.04
59	->	63	-0.09	0.01
59	->	64	-0.03	0.00
59	->	65	-0.01	0.00
59	->	66	-0.06	0.01
59	->	67	-0.01	0.01
59	->	68	0.17	0.17
59	->	69	-2.59	0.99
59	->	70	-3.27	0.46
59	->	71	-1.83	0.33
59	->	72	-0.05	0.05
59	->	73	-0.07	0.02
59	->	74	-0.07	0.01
59	->	75	-0.59	0.33
59	->	76	0.04	0.01
59	->	77	0.01	0.00
59	->	78	0.05	0.01
59	->	79	-0.00	0.00
59	->	80	-0.00	0.00
59	->	81	0.02	0.00
59	->	82	0.00	0.00
59	->	83	-0.01	0.00
59	->	84	-0.01	0.00
59	->	85	-0.11	0.20
59	->	86	-1.13	1.48
59	->	87	-1.16	1.70
59	->	88	-0.06	0.01
59	->	89	-0.02	0.00
59	->	90	-0.01	0.00
59	->	91	-0.01	0.00
59	->	92	-0.01	0.00
59	->	93	-0.01	0.00
59	->	94	-0.01	0.00
59	->	95	-0.00	0.00
59	->	96	-0.00	0.00
59	->	97	-0.01	0.00
59	->	98	-0.02	0.00

59	->	99	-0.05	0.03
59	->	100	-0.08	0.04
59	->	101	-0.03	0.01
59	->	102	-0.02	0.00
59	->	103	-0.01	0.00
59	->	104	-0.01	0.00
59	->	105	-0.01	0.00
59	->	106	-0.01	0.00
60	->	1	0.00	0.00
60	->	2	0.00	0.00
60	->	3	0.00	0.00
60	->	4	0.00	0.00
60	->	5	-0.00	0.00
60	->	6	0.00	0.00
60	->	7	0.00	0.00
60	->	8	-0.00	0.00
60	->	9	0.00	0.00
60	->	10	-0.00	0.00
60	->	11	-0.00	0.00
60	->	12	0.00	0.00
60	->	13	0.00	0.00
60	->	14	0.00	0.00
60	->	15	0.00	0.00
60	->	16	-0.00	0.00
60	->	17	0.00	0.00
60	->	18	-0.00	0.00
60	->	19	-0.00	0.00
60	->	20	0.00	0.00
60	->	21	0.00	0.00
60	->	22	0.00	0.00
60	->	23	0.00	0.00
60	->	24	0.00	0.00
60	->	25	0.00	0.00
60	->	26	0.00	0.00
60	->	27	0.00	0.00
60	->	28	0.00	0.00
60	->	29	0.00	0.00
60	->	30	-0.00	0.00
60	->	31	0.00	0.00
60	->	32	0.00	0.00
60	->	33	0.00	0.00
60	->	34	-0.00	0.00
60	->	35	-0.00	0.00
60	->	36	0.00	0.00
60	->	37	-0.00	0.00
60	->	38	-0.00	0.00
60	->	39	0.00	0.00
60	->	40	0.00	0.00
60	->	41	-0.00	0.00
60	->	42	-0.00	0.00

60	->	43	0.00	0.00
60	->	44	0.00	0.00
60	->	45	-0.00	0.00
60	->	46	-0.01	0.00
60	->	47	-0.00	0.00
60	->	48	-0.00	0.00
60	->	49	-0.01	0.01
60	->	50	-0.02	0.01
60	->	51	-0.00	0.00
60	->	52	-0.01	0.00
60	->	53	-1.10	0.62
60	->	54	-0.01	0.01
60	->	55	-0.00	0.00
60	->	56	-0.01	0.00
60	->	57	-0.03	0.00
60	->	58	-1.25	0.34
60	->	59	-0.37	0.36
60	->	60	1.48	0.60
60	->	61	-10.47	0.52
60	->	62	-0.45	0.18
60	->	63	-0.02	0.00
60	->	64	-0.00	0.00
60	->	65	-0.00	0.00
60	->	66	0.00	0.00
60	->	67	-0.00	0.00
60	->	68	-0.01	0.01
60	->	69	-0.67	0.63
60	->	70	-0.91	0.14
60	->	71	-0.81	0.20
60	->	72	-0.15	0.06
60	->	73	-0.06	0.02
60	->	74	-0.02	0.00
60	->	75	-0.02	0.01
60	->	76	-0.04	0.02
60	->	77	-0.01	0.00
60	->	78	-0.00	0.00
60	->	79	-0.00	0.00
60	->	80	-0.00	0.00
60	->	81	-0.00	0.00
60	->	82	-0.00	0.00
60	->	83	-0.00	0.00
60	->	84	0.00	0.00
60	->	85	-0.00	0.00
60	->	86	-0.01	0.00
60	->	87	-0.01	0.00
60	->	88	-0.00	0.00
60	->	89	-0.00	0.00
60	->	90	-0.00	0.00
60	->	91	-0.00	0.00
60	->	92	-0.00	0.00

60	->	93	-0.00	0.00
60	->	94	-0.00	0.00
60	->	95	-0.00	0.00
60	->	96	-0.00	0.00
60	->	97	-0.00	0.00
60	->	98	-0.00	0.00
60	->	99	-0.04	0.03
60	->	100	-0.14	0.12
60	->	101	-0.02	0.01
60	->	102	-0.00	0.00
60	->	103	-0.00	0.00
60	->	104	-0.00	0.00
60	->	105	-0.00	0.00
60	->	106	-0.00	0.00
61	->	1	0.00	0.00
61	->	2	0.00	0.00
61	->	3	0.00	0.00
61	->	4	0.00	0.00
61	->	5	-0.00	0.00
61	->	6	0.00	0.00
61	->	7	0.00	0.00
61	->	8	-0.00	0.00
61	->	9	0.00	0.00
61	->	10	-0.00	0.00
61	->	11	-0.00	0.00
61	->	12	0.00	0.00
61	->	13	0.00	0.00
61	->	14	0.00	0.00
61	->	15	0.00	0.00
61	->	16	-0.00	0.00
61	->	17	0.00	0.00
61	->	18	0.00	0.00
61	->	19	0.00	0.00
61	->	20	0.00	0.00
61	->	21	0.00	0.00
61	->	22	0.00	0.00
61	->	23	0.00	0.00
61	->	24	0.00	0.00
61	->	25	0.00	0.00
61	->	26	0.00	0.00
61	->	27	0.00	0.00
61	->	28	0.00	0.00
61	->	29	0.00	0.00
61	->	30	-0.00	0.00
61	->	31	-0.00	0.00
61	->	32	0.00	0.00
61	->	33	-0.00	0.00
61	->	34	0.00	0.00
61	->	35	0.00	0.00
61	->	36	-0.00	0.00

61	->	37	0.00	0.00
61	->	38	0.00	0.00
61	->	39	0.00	0.00
61	->	40	-0.00	0.00
61	->	41	0.00	0.00
61	->	42	-0.00	0.00
61	->	43	0.00	0.00
61	->	44	-0.00	0.00
61	->	45	-0.00	0.00
61	->	46	-0.01	0.00
61	->	47	-0.00	0.00
61	->	48	-0.00	0.00
61	->	49	-0.02	0.01
61	->	50	-0.01	0.01
61	->	51	-0.00	0.00
61	->	52	-0.01	0.00
61	->	53	-0.49	0.36
61	->	54	-0.00	0.00
61	->	55	-0.00	0.00
61	->	56	-0.00	0.00
61	->	57	-0.01	0.00
61	->	58	-0.09	0.02
61	->	59	-0.84	0.28
61	->	60	-10.60	0.52
61	->	61	27.43	1.26
61	->	62	-10.90	0.46
61	->	63	-0.40	0.07
61	->	64	-0.05	0.01
61	->	65	-0.01	0.00
61	->	66	-0.01	0.00
61	->	67	-0.02	0.01
61	->	68	-0.23	0.08
61	->	69	-2.93	0.52
61	->	70	-3.65	0.40
61	->	71	-0.98	0.23
61	->	72	-2.22	0.65
61	->	73	-1.92	0.58
61	->	74	0.01	0.02
61	->	75	-0.15	0.05
61	->	76	-1.42	0.92
61	->	77	-0.03	0.02
61	->	78	-0.01	0.01
61	->	79	-0.02	0.01
61	->	80	-0.01	0.01
61	->	81	-0.00	0.00
61	->	82	-0.00	0.00
61	->	83	-0.01	0.00
61	->	84	-0.00	0.00
61	->	85	-0.00	0.00
61	->	86	-0.01	0.00

61	->	87	-0.01	0.00
61	->	88	-0.00	0.00
61	->	89	-0.00	0.00
61	->	90	-0.00	0.00
61	->	91	-0.00	0.00
61	->	92	-0.01	0.00
61	->	93	-0.00	0.00
61	->	94	-0.00	0.00
61	->	95	-0.00	0.00
61	->	96	-0.00	0.00
61	->	97	-0.00	0.00
61	->	98	-0.00	0.00
61	->	99	-0.02	0.03
61	->	100	-1.05	0.73
61	->	101	-0.40	0.28
61	->	102	-0.02	0.00
61	->	103	-0.01	0.00
61	->	104	-0.00	0.00
61	->	105	-0.00	0.00
61	->	106	-0.00	0.00
62	->	1	-0.00	0.00
62	->	2	0.00	0.00
62	->	3	0.00	0.00
62	->	4	0.00	0.00
62	->	5	0.00	0.00
62	->	6	0.00	0.00
62	->	7	0.00	0.00
62	->	8	0.00	0.00
62	->	9	0.00	0.00
62	->	10	0.00	0.00
62	->	11	0.00	0.00
62	->	12	-0.00	0.00
62	->	13	-0.00	0.00
62	->	14	0.00	0.00
62	->	15	-0.00	0.00
62	->	16	0.00	0.00
62	->	17	0.00	0.00
62	->	18	0.00	0.00
62	->	19	-0.00	0.00
62	->	20	0.00	0.00
62	->	21	-0.00	0.00
62	->	22	-0.00	0.00
62	->	23	-0.00	0.00
62	->	24	0.00	0.00
62	->	25	-0.00	0.00
62	->	26	0.00	0.00
62	->	27	0.00	0.00
62	->	28	-0.00	0.00
62	->	29	0.00	0.00
62	->	30	0.00	0.00

62	->	31	-0.00	0.00
62	->	32	-0.00	0.00
62	->	33	-0.00	0.00
62	->	34	0.00	0.00
62	->	35	0.00	0.00
62	->	36	-0.00	0.00
62	->	37	0.00	0.00
62	->	38	0.00	0.00
62	->	39	0.00	0.00
62	->	40	-0.00	0.00
62	->	41	0.00	0.00
62	->	42	-0.00	0.00
62	->	43	-0.00	0.00
62	->	44	-0.00	0.00
62	->	45	0.00	0.00
62	->	46	0.00	0.00
62	->	47	0.00	0.00
62	->	48	-0.00	0.00
62	->	49	-0.00	0.00
62	->	50	-0.00	0.00
62	->	51	-0.00	0.00
62	->	52	-0.00	0.00
62	->	53	-0.01	0.01
62	->	54	-0.00	0.00
62	->	55	0.00	0.00
62	->	56	-0.00	0.00
62	->	57	-0.00	0.00
62	->	58	-0.01	0.00
62	->	59	-0.15	0.04
62	->	60	-0.47	0.19
62	->	61	-10.89	0.46
62	->	62	23.73	1.16
62	->	63	-11.59	0.67
62	->	64	-0.40	0.10
62	->	65	-0.04	0.03
62	->	66	-0.13	0.06
62	->	67	-0.11	0.19
62	->	68	-0.87	0.63
62	->	69	-1.48	0.35
62	->	70	-0.91	0.52
62	->	71	-0.01	0.01
62	->	72	-0.03	0.01
62	->	73	-0.02	0.01
62	->	74	0.01	0.01
62	->	75	-0.02	0.02
62	->	76	-3.23	1.51
62	->	77	-0.01	0.01
62	->	78	-0.00	0.01
62	->	79	-0.08	0.02
62	->	80	-0.02	0.02

62	->	81	-0.01	0.00
62	->	82	-0.00	0.00
62	->	83	-0.04	0.01
62	->	84	-0.00	0.00
62	->	85	-0.00	0.00
62	->	86	-0.00	0.00
62	->	87	-0.00	0.00
62	->	88	-0.00	0.00
62	->	89	0.00	0.00
62	->	90	0.00	0.00
62	->	91	0.00	0.00
62	->	92	0.00	0.00
62	->	93	0.00	0.00
62	->	94	0.00	0.00
62	->	95	0.00	0.00
62	->	96	0.00	0.00
62	->	97	0.00	0.00
62	->	98	0.00	0.00
62	->	99	0.00	0.00
62	->	100	-0.00	0.00
62	->	101	0.00	0.01
62	->	102	0.00	0.00
62	->	103	0.00	0.00
62	->	104	0.00	0.00
62	->	105	0.00	0.00
62	->	106	0.00	0.00
63	->	1	-0.00	0.00
63	->	2	-0.00	0.00
63	->	3	0.00	0.00
63	->	4	0.00	0.00
63	->	5	0.00	0.00
63	->	6	-0.00	0.00
63	->	7	0.00	0.00
63	->	8	0.00	0.00
63	->	9	0.00	0.00
63	->	10	0.00	0.00
63	->	11	0.00	0.00
63	->	12	-0.00	0.00
63	->	13	-0.00	0.00
63	->	14	-0.00	0.00
63	->	15	-0.00	0.00
63	->	16	0.00	0.00
63	->	17	0.00	0.00
63	->	18	0.00	0.00
63	->	19	0.00	0.00
63	->	20	0.00	0.00
63	->	21	-0.00	0.00
63	->	22	-0.01	0.00
63	->	23	-0.00	0.00
63	->	24	-0.00	0.00

63	->	25	-0.00	0.00
63	->	26	-0.00	0.00
63	->	27	-0.00	0.00
63	->	28	-0.00	0.00
63	->	29	-0.00	0.00
63	->	30	0.00	0.00
63	->	31	-0.00	0.00
63	->	32	-0.00	0.00
63	->	33	-0.00	0.00
63	->	34	0.00	0.00
63	->	35	0.01	0.00
63	->	36	-0.00	0.00
63	->	37	0.00	0.00
63	->	38	0.01	0.00
63	->	39	-0.00	0.00
63	->	40	-0.01	0.00
63	->	41	0.00	0.00
63	->	42	-0.00	0.00
63	->	43	-0.00	0.00
63	->	44	-0.01	0.00
63	->	45	0.00	0.00
63	->	46	0.01	0.00
63	->	47	0.00	0.00
63	->	48	0.00	0.00
63	->	49	0.00	0.00
63	->	50	0.00	0.00
63	->	51	-0.00	0.00
63	->	52	0.00	0.00
63	->	53	-0.03	0.01
63	->	54	-0.00	0.00
63	->	55	-0.00	0.00
63	->	56	0.00	0.00
63	->	57	0.00	0.00
63	->	58	0.02	0.00
63	->	59	-0.09	0.01
63	->	60	-0.02	0.00
63	->	61	-0.41	0.07
63	->	62	-11.62	0.68
63	->	63	-28.95	2.72
63	->	64	-15.25	1.48
63	->	65	1.35	1.40
63	->	66	2.39	0.60
63	->	67	-0.69	0.76
63	->	68	-1.73	1.05
63	->	69	-0.68	0.16
63	->	70	-0.79	0.37
63	->	71	0.01	0.01
63	->	72	-0.04	0.01
63	->	73	-0.00	0.01
63	->	74	0.04	0.01

63	->	75	-0.01	0.02
63	->	76	-1.89	0.70
63	->	77	-0.02	0.02
63	->	78	-0.15	0.04
63	->	79	2.32	0.84
63	->	80	0.10	0.20
63	->	81	-0.01	0.02
63	->	82	-0.07	0.03
63	->	83	2.22	0.29
63	->	84	0.06	0.01
63	->	85	0.01	0.00
63	->	86	0.04	0.01
63	->	87	0.02	0.01
63	->	88	0.01	0.00
63	->	89	0.01	0.00
63	->	90	0.01	0.00
63	->	91	0.00	0.00
63	->	92	0.00	0.00
63	->	93	0.01	0.00
63	->	94	0.01	0.00
63	->	95	0.00	0.00
63	->	96	0.00	0.00
63	->	97	0.00	0.00
63	->	98	0.01	0.00
63	->	99	0.01	0.00
63	->	100	0.02	0.00
63	->	101	0.03	0.01
63	->	102	0.03	0.01
63	->	103	0.02	0.01
63	->	104	0.01	0.00
63	->	105	0.01	0.00
63	->	106	0.00	0.00
64	->	1	-0.00	0.00
64	->	2	-0.00	0.00
64	->	3	0.00	0.00
64	->	4	0.00	0.00
64	->	5	0.00	0.00
64	->	6	-0.00	0.00
64	->	7	0.00	0.00
64	->	8	0.00	0.00
64	->	9	0.00	0.00
64	->	10	0.00	0.00
64	->	11	0.00	0.00
64	->	12	-0.00	0.00
64	->	13	-0.00	0.00
64	->	14	-0.00	0.00
64	->	15	-0.00	0.00
64	->	16	0.00	0.00
64	->	17	0.00	0.00
64	->	18	0.00	0.00

64	->	19	0.00	0.00
64	->	20	-0.00	0.00
64	->	21	-0.00	0.00
64	->	22	-0.00	0.00
64	->	23	-0.00	0.00
64	->	24	-0.00	0.00
64	->	25	-0.00	0.00
64	->	26	-0.00	0.00
64	->	27	-0.00	0.00
64	->	28	-0.00	0.00
64	->	29	-0.00	0.00
64	->	30	0.00	0.00
64	->	31	-0.00	0.00
64	->	32	-0.00	0.00
64	->	33	-0.00	0.00
64	->	34	-0.00	0.00
64	->	35	0.00	0.00
64	->	36	-0.00	0.00
64	->	37	0.00	0.00
64	->	38	0.00	0.00
64	->	39	-0.00	0.00
64	->	40	-0.00	0.00
64	->	41	0.00	0.00
64	->	42	-0.00	0.00
64	->	43	-0.00	0.00
64	->	44	-0.01	0.00
64	->	45	0.00	0.00
64	->	46	0.01	0.00
64	->	47	0.00	0.00
64	->	48	0.00	0.00
64	->	49	0.00	0.00
64	->	50	0.00	0.00
64	->	51	-0.00	0.00
64	->	52	0.00	0.00
64	->	53	-0.02	0.00
64	->	54	-0.00	0.00
64	->	55	-0.00	0.00
64	->	56	0.00	0.00
64	->	57	0.00	0.00
64	->	58	0.01	0.00
64	->	59	-0.03	0.00
64	->	60	-0.00	0.00
64	->	61	-0.05	0.01
64	->	62	-0.40	0.10
64	->	63	-15.40	1.51
64	->	64	-34.76	2.05
64	->	65	-12.28	0.67
64	->	66	-0.46	0.35
64	->	67	-0.21	0.36
64	->	68	-0.05	0.02

64	->	69	-0.08	0.02
64	->	70	-0.02	0.02
64	->	71	0.00	0.00
64	->	72	-0.02	0.00
64	->	73	-0.00	0.00
64	->	74	0.02	0.00
64	->	75	0.02	0.00
64	->	76	-1.02	1.11
64	->	77	-0.04	0.01
64	->	78	-0.03	0.01
64	->	79	-0.07	0.06
64	->	80	-0.12	0.20
64	->	81	-0.03	0.01
64	->	82	-0.01	0.00
64	->	83	-0.20	0.13
64	->	84	0.03	0.01
64	->	85	0.00	0.00
64	->	86	0.02	0.00
64	->	87	0.01	0.00
64	->	88	0.01	0.00
64	->	89	0.00	0.00
64	->	90	0.00	0.00
64	->	91	0.00	0.00
64	->	92	0.00	0.00
64	->	93	0.01	0.00
64	->	94	0.01	0.00
64	->	95	0.00	0.00
64	->	96	0.00	0.00
64	->	97	0.00	0.00
64	->	98	0.00	0.00
64	->	99	0.01	0.00
64	->	100	0.02	0.01
64	->	101	0.03	0.01
64	->	102	0.02	0.01
64	->	103	0.01	0.00
64	->	104	0.01	0.00
64	->	105	0.01	0.00
64	->	106	0.00	0.00
65	->	1	-0.00	0.00
65	->	2	0.00	0.00
65	->	3	0.00	0.00
65	->	4	0.00	0.00
65	->	5	0.00	0.00
65	->	6	0.00	0.00
65	->	7	0.00	0.00
65	->	8	0.00	0.00
65	->	9	0.00	0.00
65	->	10	0.00	0.00
65	->	11	0.00	0.00
65	->	12	-0.00	0.00

65 ->	13	-0.00	0.00
65 ->	14	0.00	0.00
65 ->	15	0.00	0.00
65 ->	16	0.00	0.00
65 ->	17	0.00	0.00
65 ->	18	0.00	0.00
65 ->	19	0.00	0.00
65 ->	20	0.00	0.00
65 ->	21	-0.00	0.00
65 ->	22	-0.00	0.00
65 ->	23	0.00	0.00
65 ->	24	0.00	0.00
65 ->	25	-0.00	0.00
65 ->	26	0.00	0.00
65 ->	27	0.00	0.00
65 ->	28	0.00	0.00
65 ->	29	0.00	0.00
65 ->	30	0.00	0.00
65 ->	31	-0.00	0.00
65 ->	32	0.00	0.00
65 ->	33	-0.00	0.00
65 ->	34	0.00	0.00
65 ->	35	0.00	0.00
65 ->	36	-0.00	0.00
65 ->	37	0.00	0.00
65 ->	38	0.00	0.00
65 ->	39	0.00	0.00
65 ->	40	-0.00	0.00
65 ->	41	0.00	0.00
65 ->	42	0.00	0.00
65 ->	43	0.00	0.00
65 ->	44	-0.00	0.00
65 ->	45	0.00	0.00
65 ->	46	0.00	0.00
65 ->	47	0.00	0.00
65 ->	48	0.00	0.00
65 ->	49	0.00	0.00
65 ->	50	0.00	0.00
65 ->	51	0.00	0.00
65 ->	52	0.00	0.00
65 ->	53	-0.00	0.00
65 ->	54	-0.00	0.00
65 ->	55	-0.00	0.00
65 ->	56	0.00	0.00
65 ->	57	0.00	0.00
65 ->	58	0.00	0.00
65 ->	59	-0.01	0.00
65 ->	60	-0.00	0.00
65 ->	61	-0.01	0.00
65 ->	62	-0.04	0.03

65	->	63	1.27	1.41
65	->	64	-12.47	0.67
65	->	65	22.15	1.65
65	->	66	-8.83	1.13
65	->	67	-0.38	0.24
65	->	68	-0.21	0.08
65	->	69	-0.01	0.01
65	->	70	-0.03	0.01
65	->	71	-0.00	0.00
65	->	72	-0.00	0.00
65	->	73	-0.00	0.00
65	->	74	0.00	0.00
65	->	75	-0.00	0.00
65	->	76	-0.04	0.03
65	->	77	-0.01	0.00
65	->	78	-0.01	0.00
65	->	79	0.64	0.12
65	->	80	-0.14	0.19
65	->	81	-0.01	0.00
65	->	82	-0.05	0.02
65	->	83	0.24	0.80
65	->	84	-0.08	0.07
65	->	85	-0.00	0.00
65	->	86	0.00	0.00
65	->	87	0.00	0.00
65	->	88	0.00	0.00
65	->	89	0.00	0.00
65	->	90	0.00	0.00
65	->	91	0.00	0.00
65	->	92	0.00	0.00
65	->	93	0.00	0.00
65	->	94	0.00	0.00
65	->	95	0.00	0.00
65	->	96	0.00	0.00
65	->	97	0.00	0.00
65	->	98	0.00	0.00
65	->	99	0.00	0.00
65	->	100	0.00	0.00
65	->	101	-0.00	0.00
65	->	102	-0.00	0.00
65	->	103	0.00	0.00
65	->	104	-0.00	0.00
65	->	105	0.00	0.00
65	->	106	0.00	0.00
66	->	1	-0.00	0.00
66	->	2	-0.00	0.00
66	->	3	0.00	0.00
66	->	4	-0.00	0.00
66	->	5	0.00	0.00
66	->	6	-0.00	0.00

66	->	7	0.00	0.00
66	->	8	0.00	0.00
66	->	9	0.00	0.00
66	->	10	0.00	0.00
66	->	11	0.00	0.00
66	->	12	-0.00	0.00
66	->	13	-0.00	0.00
66	->	14	-0.00	0.00
66	->	15	-0.00	0.00
66	->	16	0.00	0.00
66	->	17	0.00	0.00
66	->	18	0.00	0.00
66	->	19	0.00	0.00
66	->	20	0.00	0.00
66	->	21	-0.00	0.00
66	->	22	-0.00	0.00
66	->	23	-0.00	0.00
66	->	24	-0.00	0.00
66	->	25	-0.00	0.00
66	->	26	-0.00	0.00
66	->	27	-0.00	0.00
66	->	28	-0.00	0.00
66	->	29	-0.00	0.00
66	->	30	0.00	0.00
66	->	31	-0.00	0.00
66	->	32	-0.00	0.00
66	->	33	0.00	0.00
66	->	34	-0.00	0.00
66	->	35	0.01	0.00
66	->	36	-0.00	0.00
66	->	37	0.00	0.00
66	->	38	0.01	0.00
66	->	39	-0.00	0.00
66	->	40	-0.01	0.00
66	->	41	0.00	0.00
66	->	42	-0.00	0.00
66	->	43	-0.00	0.00
66	->	44	-0.01	0.00
66	->	45	0.00	0.00
66	->	46	0.01	0.00
66	->	47	0.00	0.00
66	->	48	0.00	0.00
66	->	49	0.00	0.00
66	->	50	0.00	0.00
66	->	51	-0.00	0.00
66	->	52	0.00	0.00
66	->	53	-0.02	0.00
66	->	54	0.00	0.00
66	->	55	-0.00	0.00
66	->	56	0.00	0.00

66 ->	57	0.00	0.00
66 ->	58	0.02	0.00
66 ->	59	-0.06	0.01
66 ->	60	0.00	0.00
66 ->	61	-0.01	0.00
66 ->	62	-0.13	0.06
66 ->	63	2.41	0.60
66 ->	64	-0.46	0.35
66 ->	65	-8.73	1.13
66 ->	66	-32.90	3.60
66 ->	67	-8.91	0.49
66 ->	68	-3.44	2.05
66 ->	69	-0.04	0.05
66 ->	70	0.02	0.03
66 ->	71	-0.00	0.00
66 ->	72	-0.02	0.00
66 ->	73	0.00	0.00
66 ->	74	0.03	0.00
66 ->	75	0.05	0.01
66 ->	76	-0.10	0.03
66 ->	77	-0.03	0.01
66 ->	78	-0.07	0.01
66 ->	79	2.78	0.84
66 ->	80	0.03	0.01
66 ->	81	-0.03	0.01
66 ->	82	-0.06	0.02
66 ->	83	1.98	0.37
66 ->	84	0.05	0.00
66 ->	85	0.01	0.00
66 ->	86	0.06	0.01
66 ->	87	0.02	0.01
66 ->	88	0.01	0.00
66 ->	89	0.01	0.00
66 ->	90	0.00	0.00
66 ->	91	0.00	0.00
66 ->	92	0.00	0.00
66 ->	93	0.00	0.00
66 ->	94	0.00	0.00
66 ->	95	0.00	0.00
66 ->	96	0.00	0.00
66 ->	97	0.00	0.00
66 ->	98	0.00	0.00
66 ->	99	0.01	0.00
66 ->	100	0.02	0.00
66 ->	101	0.02	0.00
66 ->	102	0.02	0.00
66 ->	103	0.01	0.00
66 ->	104	0.01	0.00
66 ->	105	0.01	0.00
66 ->	106	0.00	0.00

67	->	1	0.00	0.00
67	->	2	0.00	0.00
67	->	3	0.00	0.00
67	->	4	0.00	0.00
67	->	5	0.00	0.00
67	->	6	0.00	0.00
67	->	7	0.00	0.00
67	->	8	0.00	0.00
67	->	9	0.00	0.00
67	->	10	-0.00	0.00
67	->	11	0.00	0.00
67	->	12	0.00	0.00
67	->	13	0.00	0.00
67	->	14	-0.00	0.00
67	->	15	0.00	0.00
67	->	16	0.00	0.00
67	->	17	0.00	0.00
67	->	18	0.00	0.00
67	->	19	0.00	0.00
67	->	20	0.00	0.00
67	->	21	0.00	0.00
67	->	22	-0.00	0.00
67	->	23	0.00	0.00
67	->	24	0.00	0.00
67	->	25	-0.00	0.00
67	->	26	0.00	0.00
67	->	27	0.00	0.00
67	->	28	0.00	0.00
67	->	29	0.00	0.00
67	->	30	0.00	0.00
67	->	31	-0.00	0.00
67	->	32	0.00	0.00
67	->	33	-0.00	0.00
67	->	34	0.00	0.00
67	->	35	0.00	0.00
67	->	36	-0.00	0.00
67	->	37	0.00	0.00
67	->	38	0.00	0.00
67	->	39	-0.00	0.00
67	->	40	-0.00	0.00
67	->	41	0.00	0.00
67	->	42	0.00	0.00
67	->	43	0.00	0.00
67	->	44	-0.00	0.00
67	->	45	0.00	0.00
67	->	46	0.00	0.00
67	->	47	0.00	0.00
67	->	48	0.00	0.00
67	->	49	0.00	0.00
67	->	50	0.00	0.00

67	->	51	0.00	0.00
67	->	52	0.00	0.00
67	->	53	-0.00	0.00
67	->	54	-0.00	0.00
67	->	55	-0.00	0.00
67	->	56	0.00	0.00
67	->	57	-0.00	0.00
67	->	58	0.00	0.00
67	->	59	-0.01	0.01
67	->	60	-0.00	0.00
67	->	61	-0.02	0.01
67	->	62	-0.11	0.18
67	->	63	-0.70	0.76
67	->	64	-0.21	0.37
67	->	65	-0.38	0.24
67	->	66	-8.97	0.49
67	->	67	11.30	0.85
67	->	68	-8.62	0.34
67	->	69	-0.95	0.62
67	->	70	-0.08	0.03
67	->	71	-0.00	0.00
67	->	72	-0.00	0.00
67	->	73	-0.00	0.00
67	->	74	-0.00	0.00
67	->	75	0.00	0.00
67	->	76	-0.01	0.00
67	->	77	-0.00	0.00
67	->	78	-0.00	0.00
67	->	79	-0.06	0.04
67	->	80	-0.00	0.00
67	->	81	-0.00	0.00
67	->	82	-0.00	0.00
67	->	83	-0.02	0.04
67	->	84	-0.00	0.00
67	->	85	-0.00	0.00
67	->	86	-0.00	0.00
67	->	87	-0.00	0.00
67	->	88	0.00	0.00
67	->	89	0.00	0.00
67	->	90	0.00	0.00
67	->	91	0.00	0.00
67	->	92	0.00	0.00
67	->	93	0.00	0.00
67	->	94	0.00	0.00
67	->	95	0.00	0.00
67	->	96	0.00	0.00
67	->	97	0.00	0.00
67	->	98	0.00	0.00
67	->	99	0.00	0.00
67	->	100	0.00	0.00

67	->	101	0.00	0.00
67	->	102	-0.00	0.00
67	->	103	-0.00	0.00
67	->	104	-0.00	0.00
67	->	105	-0.00	0.00
67	->	106	-0.00	0.00
68	->	1	0.00	0.00
68	->	2	0.00	0.00
68	->	3	-0.00	0.00
68	->	4	0.00	0.00
68	->	5	-0.00	0.00
68	->	6	0.00	0.00
68	->	7	0.00	0.00
68	->	8	-0.00	0.00
68	->	9	-0.00	0.00
68	->	10	-0.00	0.00
68	->	11	-0.00	0.00
68	->	12	0.00	0.00
68	->	13	0.00	0.00
68	->	14	0.00	0.00
68	->	15	0.00	0.00
68	->	16	-0.00	0.00
68	->	17	-0.00	0.00
68	->	18	-0.00	0.00
68	->	19	-0.00	0.00
68	->	20	-0.00	0.00
68	->	21	0.00	0.00
68	->	22	0.01	0.00
68	->	23	0.00	0.00
68	->	24	0.00	0.00
68	->	25	0.00	0.00
68	->	26	0.00	0.00
68	->	27	0.00	0.00
68	->	28	0.00	0.00
68	->	29	0.00	0.00
68	->	30	-0.00	0.00
68	->	31	0.01	0.00
68	->	32	0.00	0.00
68	->	33	0.00	0.00
68	->	34	0.00	0.00
68	->	35	-0.01	0.00
68	->	36	0.01	0.00
68	->	37	-0.00	0.00
68	->	38	-0.01	0.00
68	->	39	0.00	0.00
68	->	40	0.02	0.01
68	->	41	-0.00	0.00
68	->	42	0.00	0.00
68	->	43	0.00	0.00
68	->	44	0.01	0.00

68	->	45	-0.00	0.00
68	->	46	-0.02	0.00
68	->	47	-0.00	0.00
68	->	48	-0.00	0.00
68	->	49	-0.00	0.00
68	->	50	-0.00	0.00
68	->	51	-0.00	0.00
68	->	52	-0.00	0.00
68	->	53	0.03	0.01
68	->	54	-0.01	0.01
68	->	55	-0.00	0.00
68	->	56	-0.00	0.00
68	->	57	-0.00	0.00
68	->	58	-0.03	0.01
68	->	59	0.17	0.16
68	->	60	-0.01	0.01
68	->	61	-0.23	0.08
68	->	62	-0.87	0.63
68	->	63	-1.73	1.06
68	->	64	-0.05	0.02
68	->	65	-0.21	0.08
68	->	66	-3.54	2.06
68	->	67	-8.75	0.34
68	->	68	-106.86	3.19
68	->	69	-13.10	0.67
68	->	70	-3.26	0.80
68	->	71	-0.07	0.02
68	->	72	0.03	0.02
68	->	73	-0.01	0.00
68	->	74	-0.05	0.01
68	->	75	-0.50	0.52
68	->	76	-0.07	0.08
68	->	77	0.01	0.01
68	->	78	0.08	0.02
68	->	79	-1.45	0.69
68	->	80	-0.03	0.01
68	->	81	0.02	0.01
68	->	82	-0.03	0.02
68	->	83	-0.22	0.16
68	->	84	-0.03	0.01
68	->	85	-1.48	1.22
68	->	86	-5.46	4.14
68	->	87	-0.26	0.59
68	->	88	-0.04	0.01
68	->	89	-0.02	0.00
68	->	90	-0.01	0.00
68	->	91	-0.01	0.00
68	->	92	-0.00	0.00
68	->	93	-0.00	0.00
68	->	94	-0.00	0.00

68	->	95	-0.00	0.00
68	->	96	-0.00	0.00
68	->	97	-0.00	0.00
68	->	98	-0.01	0.00
68	->	99	-0.01	0.00
68	->	100	-0.02	0.00
68	->	101	-0.02	0.00
68	->	102	-0.02	0.00
68	->	103	-0.02	0.00
68	->	104	-0.01	0.00
68	->	105	-0.02	0.00
68	->	106	-0.02	0.01
69	->	1	0.00	0.00
69	->	2	0.00	0.00
69	->	3	-0.00	0.00
69	->	4	-0.00	0.00
69	->	5	-0.00	0.00
69	->	6	0.00	0.00
69	->	7	0.00	0.00
69	->	8	-0.00	0.00
69	->	9	-0.00	0.00
69	->	10	-0.00	0.00
69	->	11	-0.00	0.00
69	->	12	0.00	0.00
69	->	13	0.00	0.00
69	->	14	0.00	0.00
69	->	15	0.00	0.00
69	->	16	-0.00	0.00
69	->	17	-0.00	0.00
69	->	18	-0.00	0.00
69	->	19	-0.00	0.00
69	->	20	-0.00	0.00
69	->	21	0.00	0.00
69	->	22	0.01	0.00
69	->	23	0.00	0.00
69	->	24	0.00	0.00
69	->	25	0.00	0.00
69	->	26	0.00	0.00
69	->	27	0.00	0.00
69	->	28	0.00	0.00
69	->	29	0.00	0.00
69	->	30	-0.00	0.00
69	->	31	0.01	0.00
69	->	32	-0.00	0.00
69	->	33	0.00	0.00
69	->	34	-0.00	0.00
69	->	35	-0.01	0.00
69	->	36	0.01	0.00
69	->	37	-0.00	0.00
69	->	38	-0.01	0.00

69	->	39	0.00	0.00
69	->	40	0.01	0.00
69	->	41	-0.00	0.00
69	->	42	0.00	0.00
69	->	43	0.00	0.00
69	->	44	0.01	0.00
69	->	45	-0.00	0.00
69	->	46	-0.02	0.00
69	->	47	-0.00	0.00
69	->	48	-0.00	0.00
69	->	49	0.00	0.00
69	->	50	-0.01	0.00
69	->	51	-0.00	0.00
69	->	52	-0.00	0.00
69	->	53	0.08	0.03
69	->	54	-0.02	0.01
69	->	55	-0.00	0.00
69	->	56	-0.01	0.00
69	->	57	-0.03	0.02
69	->	58	-0.05	0.06
69	->	59	-2.60	0.98
69	->	60	-0.68	0.64
69	->	61	-2.95	0.52
69	->	62	-1.50	0.35
69	->	63	-0.68	0.16
69	->	64	-0.08	0.02
69	->	65	-0.01	0.01
69	->	66	-0.04	0.05
69	->	67	-1.01	0.62
69	->	68	-13.10	0.67
69	->	69	10.02	1.94
69	->	70	-10.67	0.55
69	->	71	-0.41	0.06
69	->	72	0.00	0.03
69	->	73	-0.04	0.02
69	->	74	-0.05	0.01
69	->	75	-0.33	0.18
69	->	76	0.11	0.06
69	->	77	-0.00	0.01
69	->	78	0.04	0.02
69	->	79	-0.03	0.02
69	->	80	-0.01	0.01
69	->	81	0.01	0.00
69	->	82	-0.00	0.00
69	->	83	-0.02	0.01
69	->	84	-0.01	0.00
69	->	85	-0.03	0.03
69	->	86	-0.11	0.06
69	->	87	-0.06	0.04
69	->	88	-0.02	0.00

69	->	89	-0.01	0.00
69	->	90	-0.01	0.00
69	->	91	-0.00	0.00
69	->	92	-0.00	0.00
69	->	93	-0.01	0.00
69	->	94	-0.01	0.00
69	->	95	-0.00	0.00
69	->	96	-0.00	0.00
69	->	97	-0.01	0.00
69	->	98	-0.01	0.00
69	->	99	-0.03	0.01
69	->	100	-0.05	0.01
69	->	101	-0.04	0.01
69	->	102	-0.02	0.00
69	->	103	-0.01	0.00
69	->	104	-0.01	0.00
69	->	105	-0.01	0.00
69	->	106	-0.00	0.00
70	->	1	0.00	0.00
70	->	2	0.00	0.00
70	->	3	0.00	0.00
70	->	4	0.00	0.00
70	->	5	-0.00	0.00
70	->	6	0.00	0.00
70	->	7	0.00	0.00
70	->	8	-0.00	0.00
70	->	9	0.00	0.00
70	->	10	-0.00	0.00
70	->	11	-0.00	0.00
70	->	12	0.00	0.00
70	->	13	0.00	0.00
70	->	14	0.00	0.00
70	->	15	0.00	0.00
70	->	16	-0.00	0.00
70	->	17	-0.00	0.00
70	->	18	-0.00	0.00
70	->	19	-0.00	0.00
70	->	20	-0.00	0.00
70	->	21	0.00	0.00
70	->	22	0.00	0.00
70	->	23	0.00	0.00
70	->	24	0.00	0.00
70	->	25	0.00	0.00
70	->	26	0.00	0.00
70	->	27	0.00	0.00
70	->	28	0.00	0.00
70	->	29	0.00	0.00
70	->	30	-0.00	0.00
70	->	31	0.00	0.00
70	->	32	0.00	0.00

70	->	33	0.00	0.00
70	->	34	0.00	0.00
70	->	35	-0.00	0.00
70	->	36	-0.00	0.00
70	->	37	-0.00	0.00
70	->	38	-0.00	0.00
70	->	39	-0.00	0.00
70	->	40	0.00	0.00
70	->	41	0.00	0.00
70	->	42	-0.00	0.00
70	->	43	0.00	0.00
70	->	44	0.00	0.00
70	->	45	0.00	0.00
70	->	46	-0.00	0.00
70	->	47	-0.00	0.00
70	->	48	-0.00	0.00
70	->	49	0.00	0.00
70	->	50	-0.03	0.02
70	->	51	-0.01	0.00
70	->	52	0.00	0.00
70	->	53	0.05	0.05
70	->	54	-0.03	0.01
70	->	55	-0.01	0.00
70	->	56	-0.00	0.00
70	->	57	-0.00	0.01
70	->	58	-0.26	0.09
70	->	59	-3.25	0.46
70	->	60	-0.91	0.14
70	->	61	-3.66	0.40
70	->	62	-0.91	0.52
70	->	63	-0.79	0.37
70	->	64	-0.02	0.02
70	->	65	-0.03	0.01
70	->	66	0.02	0.03
70	->	67	-0.08	0.03
70	->	68	-3.22	0.78
70	->	69	-10.73	0.55
70	->	70	30.10	1.14
70	->	71	-9.79	0.27
70	->	72	-0.91	0.22
70	->	73	-0.22	0.09
70	->	74	-0.14	0.03
70	->	75	-4.58	1.13
70	->	76	-2.52	0.48
70	->	77	-0.05	0.02
70	->	78	-0.22	0.07
70	->	79	-2.07	0.46
70	->	80	-0.05	0.04
70	->	81	-0.00	0.00
70	->	82	-0.04	0.01

70	->	83	-0.01	0.01
70	->	84	-0.00	0.00
70	->	85	-0.15	0.29
70	->	86	-0.24	0.18
70	->	87	-0.12	0.09
70	->	88	-0.02	0.01
70	->	89	-0.01	0.00
70	->	90	-0.00	0.00
70	->	91	-0.00	0.00
70	->	92	-0.00	0.00
70	->	93	-0.00	0.00
70	->	94	-0.00	0.00
70	->	95	0.00	0.00
70	->	96	0.00	0.00
70	->	97	0.00	0.00
70	->	98	-0.00	0.00
70	->	99	-0.01	0.01
70	->	100	-0.03	0.02
70	->	101	-0.02	0.01
70	->	102	-0.01	0.00
70	->	103	-0.00	0.00
70	->	104	-0.01	0.00
70	->	105	-0.00	0.00
70	->	106	-0.00	0.00
71	->	1	-0.00	0.00
71	->	2	0.00	0.00
71	->	3	0.00	0.00
71	->	4	0.00	0.00
71	->	5	0.00	0.00
71	->	6	0.00	0.00
71	->	7	0.00	0.00
71	->	8	0.00	0.00
71	->	9	0.00	0.00
71	->	10	0.00	0.00
71	->	11	0.00	0.00
71	->	12	-0.00	0.00
71	->	13	-0.00	0.00
71	->	14	0.00	0.00
71	->	15	0.00	0.00
71	->	16	0.00	0.00
71	->	17	0.00	0.00
71	->	18	0.00	0.00
71	->	19	0.00	0.00
71	->	20	0.00	0.00
71	->	21	-0.00	0.00
71	->	22	-0.00	0.00
71	->	23	-0.00	0.00
71	->	24	-0.00	0.00
71	->	25	-0.00	0.00
71	->	26	0.00	0.00

71	->	27	0.00	0.00
71	->	28	0.00	0.00
71	->	29	0.00	0.00
71	->	30	0.00	0.00
71	->	31	0.00	0.00
71	->	32	0.00	0.00
71	->	33	-0.00	0.00
71	->	34	0.00	0.00
71	->	35	-0.00	0.00
71	->	36	-0.00	0.00
71	->	37	-0.00	0.00
71	->	38	-0.00	0.00
71	->	39	0.00	0.00
71	->	40	-0.00	0.00
71	->	41	0.00	0.00
71	->	42	-0.00	0.00
71	->	43	0.00	0.00
71	->	44	0.00	0.00
71	->	45	-0.00	0.00
71	->	46	-0.01	0.01
71	->	47	-0.01	0.00
71	->	48	-0.01	0.00
71	->	49	-0.05	0.04
71	->	50	-0.36	0.25
71	->	51	-0.02	0.01
71	->	52	-0.02	0.01
71	->	53	-3.20	1.99
71	->	54	-0.36	0.29
71	->	55	-0.01	0.00
71	->	56	-0.01	0.00
71	->	57	-0.03	0.01
71	->	58	-0.66	0.26
71	->	59	-1.79	0.33
71	->	60	-0.80	0.20
71	->	61	-0.97	0.23
71	->	62	-0.01	0.01
71	->	63	0.01	0.01
71	->	64	0.00	0.00
71	->	65	-0.00	0.00
71	->	66	-0.00	0.00
71	->	67	-0.00	0.00
71	->	68	-0.07	0.02
71	->	69	-0.41	0.06
71	->	70	-9.79	0.27
71	->	71	23.60	1.23
71	->	72	-7.85	0.46
71	->	73	-0.31	0.06
71	->	74	-0.19	0.06
71	->	75	-3.87	1.59
71	->	76	-0.04	0.03

71	->	77	-0.01	0.00
71	->	78	-0.00	0.01
71	->	79	-0.00	0.00
71	->	80	-0.00	0.00
71	->	81	0.00	0.00
71	->	82	-0.00	0.00
71	->	83	0.00	0.00
71	->	84	-0.00	0.00
71	->	85	-0.01	0.00
71	->	86	-0.10	0.09
71	->	87	-0.24	0.25
71	->	88	-0.03	0.01
71	->	89	-0.01	0.00
71	->	90	-0.00	0.00
71	->	91	0.00	0.00
71	->	92	0.00	0.00
71	->	93	-0.00	0.00
71	->	94	0.00	0.00
71	->	95	0.00	0.00
71	->	96	0.00	0.00
71	->	97	0.00	0.00
71	->	98	-0.00	0.00
71	->	99	-0.00	0.02
71	->	100	0.02	0.06
71	->	101	-0.03	0.02
71	->	102	-0.01	0.00
71	->	103	0.00	0.00
71	->	104	-0.00	0.00
71	->	105	-0.00	0.00
71	->	106	-0.00	0.00
72	->	1	0.02	0.01
72	->	2	0.00	0.00
72	->	3	-0.00	0.00
72	->	4	0.00	0.00
72	->	5	-0.01	0.00
72	->	6	0.00	0.00
72	->	7	0.00	0.00
72	->	8	-0.00	0.00
72	->	9	-0.00	0.00
72	->	10	-0.01	0.00
72	->	11	-0.01	0.00
72	->	12	0.02	0.00
72	->	13	0.00	0.00
72	->	14	0.00	0.00
72	->	15	0.00	0.00
72	->	16	-0.01	0.00
72	->	17	-0.00	0.00
72	->	18	-0.00	0.00
72	->	19	-0.00	0.00
72	->	20	-0.00	0.00

72	->	21	0.00	0.00
72	->	22	0.03	0.01
72	->	23	0.00	0.00
72	->	24	0.00	0.00
72	->	25	0.02	0.00
72	->	26	0.00	0.00
72	->	27	0.00	0.00
72	->	28	0.00	0.00
72	->	29	0.00	0.00
72	->	30	-0.00	0.00
72	->	31	0.03	0.01
72	->	32	0.00	0.00
72	->	33	-0.00	0.00
72	->	34	0.00	0.00
72	->	35	-0.03	0.00
72	->	36	0.01	0.00
72	->	37	-0.00	0.00
72	->	38	-0.03	0.00
72	->	39	0.00	0.00
72	->	40	0.06	0.02
72	->	41	-0.00	0.00
72	->	42	-0.01	0.01
72	->	43	0.00	0.00
72	->	44	0.15	0.14
72	->	45	0.00	0.01
72	->	46	-0.33	0.42
72	->	47	-0.15	0.16
72	->	48	-0.02	0.01
72	->	49	0.06	0.06
72	->	50	-2.12	1.67
72	->	51	-0.07	0.04
72	->	52	-0.01	0.01
72	->	53	-0.32	0.27
72	->	54	-0.07	0.05
72	->	55	-0.00	0.00
72	->	56	-0.00	0.00
72	->	57	-0.01	0.00
72	->	58	-0.03	0.01
72	->	59	-0.05	0.05
72	->	60	-0.15	0.06
72	->	61	-2.23	0.65
72	->	62	-0.03	0.01
72	->	63	-0.04	0.01
72	->	64	-0.02	0.00
72	->	65	-0.00	0.00
72	->	66	-0.02	0.00
72	->	67	-0.00	0.00
72	->	68	0.03	0.02
72	->	69	0.00	0.03
72	->	70	-0.91	0.22

72	->	71	-7.94	0.47
72	->	72	-93.83	1.98
72	->	73	-21.57	0.56
72	->	74	-14.22	2.79
72	->	75	-4.99	2.28
72	->	76	-1.01	0.64
72	->	77	0.08	0.04
72	->	78	0.20	0.09
72	->	79	-0.01	0.01
72	->	80	-0.01	0.01
72	->	81	0.03	0.01
72	->	82	-0.00	0.00
72	->	83	-0.00	0.00
72	->	84	-0.01	0.00
72	->	85	-0.03	0.01
72	->	86	-0.20	0.13
72	->	87	-2.01	0.89
72	->	88	-8.00	2.88
72	->	89	-2.99	1.27
72	->	90	-0.25	0.44
72	->	91	-0.02	0.05
72	->	92	-0.01	0.01
72	->	93	-0.03	0.01
72	->	94	-0.02	0.00
72	->	95	-0.01	0.00
72	->	96	-0.00	0.00
72	->	97	-0.02	0.00
72	->	98	-0.03	0.01
72	->	99	-0.21	0.23
72	->	100	-0.56	0.54
72	->	101	-0.55	0.26
72	->	102	-0.27	0.39
72	->	103	0.46	0.55
72	->	104	0.18	0.27
72	->	105	0.15	0.17
72	->	106	-0.05	0.02
73	->	1	0.00	0.00
73	->	2	0.00	0.00
73	->	3	0.00	0.00
73	->	4	0.00	0.00
73	->	5	-0.00	0.00
73	->	6	0.00	0.00
73	->	7	0.00	0.00
73	->	8	-0.00	0.00
73	->	9	0.00	0.00
73	->	10	-0.00	0.00
73	->	11	-0.00	0.00
73	->	12	0.00	0.00
73	->	13	0.00	0.00
73	->	14	0.00	0.00

73	->	15	0.00	0.00
73	->	16	-0.00	0.00
73	->	17	0.00	0.00
73	->	18	0.00	0.00
73	->	19	-0.00	0.00
73	->	20	0.00	0.00
73	->	21	0.00	0.00
73	->	22	0.00	0.00
73	->	23	0.00	0.00
73	->	24	0.00	0.00
73	->	25	0.00	0.00
73	->	26	0.00	0.00
73	->	27	0.00	0.00
73	->	28	0.00	0.00
73	->	29	0.00	0.00
73	->	30	-0.00	0.00
73	->	31	0.00	0.00
73	->	32	0.00	0.00
73	->	33	0.00	0.00
73	->	34	-0.00	0.00
73	->	35	-0.00	0.00
73	->	36	0.00	0.00
73	->	37	0.00	0.00
73	->	38	-0.00	0.00
73	->	39	0.00	0.00
73	->	40	-0.00	0.00
73	->	41	-0.00	0.00
73	->	42	-0.00	0.00
73	->	43	-0.00	0.00
73	->	44	-0.00	0.01
73	->	45	-0.00	0.00
73	->	46	-0.01	0.01
73	->	47	-0.00	0.00
73	->	48	-0.00	0.00
73	->	49	-0.01	0.01
73	->	50	0.00	0.01
73	->	51	0.00	0.00
73	->	52	-0.00	0.00
73	->	53	-0.16	0.10
73	->	54	0.00	0.00
73	->	55	-0.00	0.00
73	->	56	-0.00	0.00
73	->	57	-0.00	0.00
73	->	58	-0.01	0.00
73	->	59	-0.07	0.02
73	->	60	-0.06	0.02
73	->	61	-1.96	0.59
73	->	62	-0.02	0.01
73	->	63	-0.00	0.01
73	->	64	-0.00	0.00

73	->	65	-0.00	0.00
73	->	66	0.00	0.00
73	->	67	-0.00	0.00
73	->	68	-0.01	0.00
73	->	69	-0.04	0.02
73	->	70	-0.22	0.09
73	->	71	-0.32	0.06
73	->	72	-21.57	0.56
73	->	73	24.17	2.35
73	->	74	-10.73	0.53
73	->	75	-0.78	0.18
73	->	76	-2.56	0.94
73	->	77	-1.36	0.83
73	->	78	-0.08	0.06
73	->	79	-0.04	0.03
73	->	80	-0.01	0.01
73	->	81	-0.01	0.00
73	->	82	-0.00	0.00
73	->	83	-0.00	0.00
73	->	84	0.00	0.00
73	->	85	-0.00	0.00
73	->	86	-0.00	0.00
73	->	87	-0.01	0.00
73	->	88	-0.01	0.01
73	->	89	-0.01	0.00
73	->	90	-0.00	0.01
73	->	91	-0.01	0.02
73	->	92	-0.02	0.01
73	->	93	-0.01	0.00
73	->	94	-0.01	0.00
73	->	95	-0.00	0.00
73	->	96	-0.00	0.00
73	->	97	-0.00	0.00
73	->	98	-0.00	0.00
73	->	99	0.01	0.06
73	->	100	-1.00	0.51
73	->	101	-4.79	1.70
73	->	102	-0.50	0.89
73	->	103	-0.05	0.03
73	->	104	-0.01	0.00
73	->	105	-0.00	0.00
73	->	106	-0.00	0.00
74	->	1	-0.02	0.01
74	->	2	-0.00	0.00
74	->	3	0.00	0.00
74	->	4	-0.00	0.00
74	->	5	0.01	0.00
74	->	6	-0.00	0.00
74	->	7	-0.00	0.00
74	->	8	0.00	0.00

74	->	9	0.00	0.00
74	->	10	0.01	0.00
74	->	11	0.01	0.00
74	->	12	-0.02	0.01
74	->	13	-0.00	0.00
74	->	14	-0.00	0.00
74	->	15	-0.00	0.00
74	->	16	0.01	0.00
74	->	17	0.00	0.00
74	->	18	0.00	0.00
74	->	19	0.00	0.00
74	->	20	0.00	0.00
74	->	21	-0.00	0.00
74	->	22	-0.03	0.00
74	->	23	-0.00	0.00
74	->	24	-0.00	0.00
74	->	25	-0.01	0.00
74	->	26	-0.00	0.00
74	->	27	-0.00	0.00
74	->	28	-0.00	0.00
74	->	29	-0.00	0.00
74	->	30	0.00	0.00
74	->	31	-0.02	0.00
74	->	32	-0.00	0.00
74	->	33	-0.00	0.00
74	->	34	-0.00	0.00
74	->	35	0.02	0.00
74	->	36	-0.01	0.00
74	->	37	0.00	0.00
74	->	38	0.02	0.00
74	->	39	-0.00	0.00
74	->	40	-0.03	0.00
74	->	41	0.00	0.00
74	->	42	-0.00	0.00
74	->	43	-0.00	0.00
74	->	44	-0.09	0.04
74	->	45	-0.00	0.00
74	->	46	0.07	0.02
74	->	47	-0.00	0.01
74	->	48	-0.00	0.00
74	->	49	-0.02	0.01
74	->	50	0.01	0.04
74	->	51	-0.00	0.00
74	->	52	-0.00	0.00
74	->	53	-0.13	0.05
74	->	54	-0.00	0.00
74	->	55	-0.00	0.00
74	->	56	-0.00	0.00
74	->	57	-0.00	0.00
74	->	58	0.03	0.00

74	->	59	-0.07	0.01
74	->	60	-0.02	0.00
74	->	61	0.01	0.02
74	->	62	0.01	0.01
74	->	63	0.04	0.01
74	->	64	0.02	0.00
74	->	65	0.00	0.00
74	->	66	0.03	0.00
74	->	67	-0.00	0.00
74	->	68	-0.05	0.01
74	->	69	-0.05	0.01
74	->	70	-0.14	0.03
74	->	71	-0.19	0.06
74	->	72	-14.14	2.79
74	->	73	-10.79	0.53
74	->	74	-14.69	2.61
74	->	75	-13.70	0.61
74	->	76	-1.54	0.33
74	->	77	-2.51	0.50
74	->	78	-2.20	0.51
74	->	79	-0.12	0.06
74	->	80	-0.04	0.01
74	->	81	-0.10	0.03
74	->	82	-0.01	0.00
74	->	83	-0.01	0.00
74	->	84	0.02	0.00
74	->	85	0.00	0.01
74	->	86	0.01	0.03
74	->	87	-0.37	0.24
74	->	88	0.52	0.23
74	->	89	0.21	0.13
74	->	90	-0.04	0.06
74	->	91	-0.04	0.04
74	->	92	-0.01	0.01
74	->	93	0.02	0.00
74	->	94	0.01	0.00
74	->	95	0.01	0.00
74	->	96	0.00	0.00
74	->	97	0.01	0.00
74	->	98	0.02	0.00
74	->	99	0.06	0.06
74	->	100	0.06	0.06
74	->	101	-0.29	0.31
74	->	102	-1.89	1.05
74	->	103	-3.70	1.25
74	->	104	-0.68	0.25
74	->	105	0.01	0.04
74	->	106	0.01	0.00
75	->	1	-0.01	0.00
75	->	2	-0.00	0.00

75	->	3	0.00	0.00
75	->	4	0.00	0.00
75	->	5	0.00	0.00
75	->	6	-0.00	0.00
75	->	7	0.00	0.00
75	->	8	0.00	0.00
75	->	9	0.00	0.00
75	->	10	0.00	0.00
75	->	11	0.00	0.00
75	->	12	-0.01	0.00
75	->	13	-0.00	0.00
75	->	14	-0.00	0.00
75	->	15	-0.00	0.00
75	->	16	0.01	0.00
75	->	17	0.00	0.00
75	->	18	0.00	0.00
75	->	19	0.00	0.00
75	->	20	0.00	0.00
75	->	21	-0.00	0.00
75	->	22	-0.01	0.00
75	->	23	-0.00	0.00
75	->	24	-0.00	0.00
75	->	25	-0.01	0.00
75	->	26	-0.00	0.00
75	->	27	-0.00	0.00
75	->	28	-0.00	0.00
75	->	29	-0.00	0.00
75	->	30	0.00	0.00
75	->	31	-0.02	0.00
75	->	32	-0.00	0.00
75	->	33	-0.00	0.00
75	->	34	-0.00	0.00
75	->	35	0.02	0.00
75	->	36	-0.01	0.00
75	->	37	0.00	0.00
75	->	38	0.02	0.00
75	->	39	-0.00	0.00
75	->	40	-0.04	0.01
75	->	41	0.00	0.00
75	->	42	-0.00	0.00
75	->	43	-0.00	0.00
75	->	44	-0.03	0.01
75	->	45	0.00	0.00
75	->	46	0.04	0.01
75	->	47	-0.00	0.00
75	->	48	0.00	0.00
75	->	49	-0.01	0.02
75	->	50	-0.03	0.04
75	->	51	-0.00	0.01
75	->	52	-0.00	0.01

75	->	53	-0.19	0.13
75	->	54	-0.02	0.04
75	->	55	-0.00	0.00
75	->	56	-0.00	0.00
75	->	57	-0.01	0.00
75	->	58	0.05	0.01
75	->	59	-0.59	0.33
75	->	60	-0.02	0.01
75	->	61	-0.15	0.05
75	->	62	-0.02	0.02
75	->	63	-0.01	0.02
75	->	64	0.02	0.00
75	->	65	-0.00	0.00
75	->	66	0.05	0.01
75	->	67	0.00	0.00
75	->	68	-0.50	0.51
75	->	69	-0.33	0.18
75	->	70	-4.55	1.12
75	->	71	-3.81	1.58
75	->	72	-4.95	2.27
75	->	73	-0.79	0.18
75	->	74	-13.78	0.61
75	->	75	-45.45	4.50
75	->	76	-7.58	0.54
75	->	77	-1.25	0.21
75	->	78	-3.68	0.79
75	->	79	-3.33	0.66
75	->	80	-0.17	0.06
75	->	81	-0.08	0.02
75	->	82	-0.04	0.02
75	->	83	-0.02	0.01
75	->	84	0.02	0.00
75	->	85	-0.08	0.11
75	->	86	-0.39	0.63
75	->	87	-0.89	0.58
75	->	88	0.05	0.04
75	->	89	0.01	0.01
75	->	90	0.01	0.00
75	->	91	0.01	0.00
75	->	92	0.01	0.00
75	->	93	0.01	0.00
75	->	94	0.01	0.00
75	->	95	0.00	0.00
75	->	96	0.00	0.00
75	->	97	0.01	0.00
75	->	98	0.01	0.00
75	->	99	0.02	0.01
75	->	100	0.02	0.01
75	->	101	-0.00	0.02
75	->	102	-0.01	0.02

75	->	103	-0.02	0.03
75	->	104	-0.05	0.04
75	->	105	0.00	0.01
75	->	106	0.01	0.00
76	->	1	0.01	0.00
76	->	2	0.00	0.00
76	->	3	-0.00	0.00
76	->	4	0.00	0.00
76	->	5	-0.00	0.00
76	->	6	0.00	0.00
76	->	7	0.00	0.00
76	->	8	-0.00	0.00
76	->	9	-0.00	0.00
76	->	10	-0.00	0.00
76	->	11	-0.00	0.00
76	->	12	0.01	0.00
76	->	13	0.00	0.00
76	->	14	0.00	0.00
76	->	15	0.00	0.00
76	->	16	-0.01	0.00
76	->	17	-0.00	0.00
76	->	18	-0.00	0.00
76	->	19	-0.00	0.00
76	->	20	0.00	0.00
76	->	21	0.00	0.00
76	->	22	0.01	0.00
76	->	23	0.00	0.00
76	->	24	0.00	0.00
76	->	25	0.01	0.00
76	->	26	0.00	0.00
76	->	27	0.00	0.00
76	->	28	0.00	0.00
76	->	29	0.00	0.00
76	->	30	-0.00	0.00
76	->	31	0.01	0.00
76	->	32	0.00	0.00
76	->	33	0.00	0.00
76	->	34	0.00	0.00
76	->	35	-0.01	0.00
76	->	36	0.01	0.00
76	->	37	-0.00	0.00
76	->	38	-0.01	0.00
76	->	39	0.00	0.00
76	->	40	0.01	0.00
76	->	41	-0.00	0.00
76	->	42	0.00	0.00
76	->	43	0.00	0.00
76	->	44	0.02	0.00
76	->	45	-0.00	0.00
76	->	46	-0.03	0.01

76	->	47	-0.00	0.00
76	->	48	-0.00	0.00
76	->	49	-0.00	0.00
76	->	50	-0.00	0.00
76	->	51	0.00	0.00
76	->	52	-0.00	0.00
76	->	53	0.04	0.01
76	->	54	-0.00	0.00
76	->	55	0.00	0.00
76	->	56	-0.00	0.00
76	->	57	-0.00	0.00
76	->	58	-0.04	0.01
76	->	59	0.04	0.01
76	->	60	-0.04	0.02
76	->	61	-1.42	0.93
76	->	62	-3.26	1.49
76	->	63	-1.89	0.70
76	->	64	-1.01	1.12
76	->	65	-0.04	0.03
76	->	66	-0.10	0.03
76	->	67	-0.01	0.00
76	->	68	-0.07	0.08
76	->	69	0.11	0.06
76	->	70	-2.56	0.49
76	->	71	-0.04	0.03
76	->	72	-1.03	0.64
76	->	73	-2.59	0.94
76	->	74	-1.55	0.33
76	->	75	-7.61	0.54
76	->	76	-105.74	2.37
76	->	77	-11.75	0.54
76	->	78	-0.89	0.19
76	->	79	-2.18	0.41
76	->	80	-3.08	1.36
76	->	81	-0.01	0.05
76	->	82	-0.05	0.02
76	->	83	-0.08	0.02
76	->	84	-0.05	0.02
76	->	85	-0.02	0.01
76	->	86	-0.05	0.02
76	->	87	-0.03	0.01
76	->	88	-0.02	0.00
76	->	89	-0.02	0.00
76	->	90	-0.01	0.00
76	->	91	-0.01	0.00
76	->	92	-0.01	0.00
76	->	93	-0.02	0.01
76	->	94	-0.02	0.01
76	->	95	-0.01	0.00
76	->	96	-0.00	0.00

76	->	97	-0.01	0.00
76	->	98	-0.01	0.00
76	->	99	-0.03	0.01
76	->	100	-0.12	0.09
76	->	101	-0.53	0.97
76	->	102	-0.22	0.12
76	->	103	-0.07	0.04
76	->	104	-0.02	0.01
76	->	105	-0.02	0.00
76	->	106	-0.01	0.00
77	->	1	0.02	0.01
77	->	2	0.00	0.00
77	->	3	-0.00	0.00
77	->	4	0.00	0.00
77	->	5	-0.01	0.00
77	->	6	0.00	0.00
77	->	7	-0.00	0.00
77	->	8	-0.00	0.00
77	->	9	-0.00	0.00
77	->	10	-0.01	0.00
77	->	11	-0.01	0.00
77	->	12	0.02	0.01
77	->	13	0.00	0.00
77	->	14	0.00	0.00
77	->	15	0.00	0.00
77	->	16	-0.01	0.00
77	->	17	-0.00	0.00
77	->	18	-0.00	0.00
77	->	19	0.00	0.00
77	->	20	-0.00	0.00
77	->	21	0.00	0.00
77	->	22	0.02	0.00
77	->	23	0.00	0.00
77	->	24	0.00	0.00
77	->	25	0.01	0.00
77	->	26	0.00	0.00
77	->	27	0.00	0.00
77	->	28	0.00	0.00
77	->	29	0.00	0.00
77	->	30	-0.00	0.00
77	->	31	0.01	0.00
77	->	32	0.00	0.00
77	->	33	0.00	0.00
77	->	34	0.00	0.00
77	->	35	-0.01	0.00
77	->	36	0.00	0.00
77	->	37	-0.00	0.00
77	->	38	-0.01	0.00
77	->	39	0.00	0.00
77	->	40	0.01	0.00

77 ->	41	-0.00	0.00
77 ->	42	0.00	0.00
77 ->	43	0.00	0.00
77 ->	44	0.03	0.01
77 ->	45	-0.00	0.00
77 ->	46	-0.03	0.01
77 ->	47	0.00	0.00
77 ->	48	-0.00	0.00
77 ->	49	0.00	0.00
77 ->	50	-0.00	0.00
77 ->	51	0.00	0.00
77 ->	52	0.00	0.00
77 ->	53	0.03	0.01
77 ->	54	0.00	0.00
77 ->	55	0.00	0.00
77 ->	56	0.00	0.00
77 ->	57	0.00	0.00
77 ->	58	-0.02	0.00
77 ->	59	0.01	0.00
77 ->	60	-0.01	0.00
77 ->	61	-0.03	0.02
77 ->	62	-0.01	0.01
77 ->	63	-0.02	0.02
77 ->	64	-0.04	0.01
77 ->	65	-0.01	0.00
77 ->	66	-0.03	0.01
77 ->	67	-0.00	0.00
77 ->	68	0.01	0.01
77 ->	69	-0.00	0.01
77 ->	70	-0.05	0.02
77 ->	71	-0.01	0.00
77 ->	72	0.08	0.04
77 ->	73	-1.39	0.84
77 ->	74	-2.53	0.50
77 ->	75	-1.26	0.21
77 ->	76	-11.78	0.54
77 ->	77	11.47	3.75
77 ->	78	-6.51	0.48
77 ->	79	-0.86	0.20
77 ->	80	-1.95	0.39
77 ->	81	-1.45	0.56
77 ->	82	-0.05	0.03
77 ->	83	-0.03	0.01
77 ->	84	-0.06	0.02
77 ->	85	-0.02	0.01
77 ->	86	-0.05	0.03
77 ->	87	-0.01	0.01
77 ->	88	-0.03	0.01
77 ->	89	-0.02	0.00
77 ->	90	-0.01	0.01

77	->	91	-0.01	0.01
77	->	92	-0.02	0.01
77	->	93	-0.04	0.01
77	->	94	-0.03	0.01
77	->	95	-0.01	0.00
77	->	96	-0.00	0.00
77	->	97	-0.01	0.00
77	->	98	-0.01	0.00
77	->	99	-0.02	0.01
77	->	100	-0.10	0.13
77	->	101	-1.15	1.16
77	->	102	-6.69	3.11
77	->	103	-2.51	2.63
77	->	104	-0.07	0.02
77	->	105	-0.05	0.02
77	->	106	-0.01	0.00
78	->	1	0.01	0.00
78	->	2	0.00	0.00
78	->	3	-0.00	0.00
78	->	4	0.00	0.00
78	->	5	-0.00	0.00
78	->	6	0.00	0.00
78	->	7	-0.00	0.00
78	->	8	-0.00	0.00
78	->	9	-0.00	0.00
78	->	10	-0.01	0.00
78	->	11	-0.01	0.00
78	->	12	0.02	0.00
78	->	13	0.00	0.00
78	->	14	0.00	0.00
78	->	15	0.00	0.00
78	->	16	-0.01	0.00
78	->	17	-0.00	0.00
78	->	18	-0.00	0.00
78	->	19	-0.00	0.00
78	->	20	-0.00	0.00
78	->	21	0.00	0.00
78	->	22	0.02	0.00
78	->	23	0.00	0.00
78	->	24	-0.00	0.00
78	->	25	0.01	0.00
78	->	26	0.00	0.00
78	->	27	0.00	0.00
78	->	28	0.00	0.00
78	->	29	0.00	0.00
78	->	30	-0.00	0.00
78	->	31	0.02	0.00
78	->	32	0.00	0.00
78	->	33	-0.00	0.00
78	->	34	0.00	0.00

78	->	35	-0.01	0.00
78	->	36	0.01	0.00
78	->	37	-0.00	0.00
78	->	38	-0.01	0.00
78	->	39	0.00	0.00
78	->	40	0.02	0.00
78	->	41	-0.00	0.00
78	->	42	0.00	0.00
78	->	43	0.00	0.00
78	->	44	0.02	0.00
78	->	45	-0.00	0.00
78	->	46	-0.03	0.01
78	->	47	-0.00	0.00
78	->	48	-0.00	0.00
78	->	49	-0.00	0.00
78	->	50	-0.00	0.00
78	->	51	0.00	0.00
78	->	52	-0.00	0.00
78	->	53	0.03	0.01
78	->	54	-0.00	0.00
78	->	55	0.00	0.00
78	->	56	0.00	0.00
78	->	57	0.00	0.00
78	->	58	-0.02	0.00
78	->	59	0.05	0.01
78	->	60	-0.00	0.00
78	->	61	-0.01	0.01
78	->	62	-0.00	0.01
78	->	63	-0.15	0.04
78	->	64	-0.03	0.01
78	->	65	-0.01	0.00
78	->	66	-0.07	0.01
78	->	67	-0.00	0.00
78	->	68	0.08	0.02
78	->	69	0.04	0.02
78	->	70	-0.22	0.07
78	->	71	-0.00	0.01
78	->	72	0.20	0.09
78	->	73	-0.08	0.06
78	->	74	-2.24	0.51
78	->	75	-3.71	0.79
78	->	76	-0.90	0.19
78	->	77	-6.55	0.48
78	->	78	-103.74	2.27
78	->	79	-19.72	0.40
78	->	80	-0.64	0.16
78	->	81	-1.89	0.61
78	->	82	-1.21	0.81
78	->	83	-0.11	0.04
78	->	84	-0.06	0.01

78	->	85	-1.38	1.35
78	->	86	-5.72	2.34
78	->	87	-0.76	0.81
78	->	88	-0.16	0.08
78	->	89	-0.05	0.01
78	->	90	-0.03	0.00
78	->	91	-0.02	0.00
78	->	92	-0.02	0.00
78	->	93	-0.01	0.00
78	->	94	-0.01	0.00
78	->	95	-0.00	0.00
78	->	96	-0.00	0.00
78	->	97	-0.01	0.00
78	->	98	-0.01	0.00
78	->	99	-0.02	0.00
78	->	100	-0.03	0.01
78	->	101	-0.05	0.01
78	->	102	-0.09	0.02
78	->	103	-0.23	0.18
78	->	104	-1.41	1.14
78	->	105	-3.77	2.69
78	->	106	-0.24	0.60
79	->	1	0.00	0.00
79	->	2	0.00	0.00
79	->	3	0.00	0.00
79	->	4	0.00	0.00
79	->	5	-0.00	0.00
79	->	6	0.00	0.00
79	->	7	0.00	0.00
79	->	8	-0.00	0.00
79	->	9	0.00	0.00
79	->	10	-0.00	0.00
79	->	11	-0.00	0.00
79	->	12	0.00	0.00
79	->	13	0.00	0.00
79	->	14	0.00	0.00
79	->	15	0.00	0.00
79	->	16	-0.00	0.00
79	->	17	-0.00	0.00
79	->	18	-0.00	0.00
79	->	19	-0.00	0.00
79	->	20	0.00	0.00
79	->	21	0.00	0.00
79	->	22	0.00	0.00
79	->	23	0.00	0.00
79	->	24	0.00	0.00
79	->	25	0.00	0.00
79	->	26	0.00	0.00
79	->	27	0.00	0.00
79	->	28	0.00	0.00

79	->	29	0.00	0.00
79	->	30	-0.00	0.00
79	->	31	0.00	0.00
79	->	32	0.00	0.00
79	->	33	0.00	0.00
79	->	34	0.00	0.00
79	->	35	-0.00	0.00
79	->	36	0.00	0.00
79	->	37	-0.00	0.00
79	->	38	-0.00	0.00
79	->	39	0.00	0.00
79	->	40	0.00	0.00
79	->	41	-0.00	0.00
79	->	42	0.00	0.00
79	->	43	0.00	0.00
79	->	44	0.00	0.00
79	->	45	-0.00	0.00
79	->	46	-0.00	0.00
79	->	47	-0.00	0.00
79	->	48	-0.00	0.00
79	->	49	-0.00	0.00
79	->	50	-0.00	0.00
79	->	51	0.00	0.00
79	->	52	0.00	0.00
79	->	53	0.00	0.00
79	->	54	-0.00	0.00
79	->	55	0.00	0.00
79	->	56	0.00	0.00
79	->	57	-0.00	0.00
79	->	58	-0.00	0.00
79	->	59	-0.00	0.00
79	->	60	-0.00	0.00
79	->	61	-0.02	0.01
79	->	62	-0.08	0.02
79	->	63	2.29	0.84
79	->	64	-0.07	0.06
79	->	65	0.64	0.12
79	->	66	2.75	0.85
79	->	67	-0.06	0.04
79	->	68	-1.41	0.66
79	->	69	-0.03	0.02
79	->	70	-2.07	0.45
79	->	71	-0.00	0.00
79	->	72	-0.01	0.01
79	->	73	-0.04	0.03
79	->	74	-0.12	0.06
79	->	75	-3.36	0.65
79	->	76	-2.19	0.41
79	->	77	-0.87	0.19
79	->	78	-19.76	0.41

79	->	79	9.51	1.29
79	->	80	-5.00	0.48
79	->	81	-1.09	0.26
79	->	82	-3.92	0.72
79	->	83	20.34	12.71
79	->	84	-0.04	0.05
79	->	85	-0.17	0.21
79	->	86	-0.08	0.12
79	->	87	-0.02	0.01
79	->	88	-0.01	0.00
79	->	89	-0.00	0.00
79	->	90	-0.00	0.00
79	->	91	-0.00	0.00
79	->	92	-0.00	0.00
79	->	93	-0.00	0.00
79	->	94	-0.00	0.00
79	->	95	-0.00	0.00
79	->	96	-0.00	0.00
79	->	97	-0.00	0.00
79	->	98	-0.00	0.00
79	->	99	-0.00	0.00
79	->	100	-0.01	0.00
79	->	101	-0.01	0.00
79	->	102	-0.01	0.01
79	->	103	-0.01	0.00
79	->	104	-0.01	0.00
79	->	105	-0.01	0.00
79	->	106	-0.01	0.00
80	->	1	0.00	0.00
80	->	2	0.00	0.00
80	->	3	0.00	0.00
80	->	4	0.00	0.00
80	->	5	-0.00	0.00
80	->	6	0.00	0.00
80	->	7	-0.00	0.00
80	->	8	-0.00	0.00
80	->	9	0.00	0.00
80	->	10	-0.00	0.00
80	->	11	-0.00	0.00
80	->	12	0.00	0.00
80	->	13	0.00	0.00
80	->	14	0.00	0.00
80	->	15	0.00	0.00
80	->	16	-0.00	0.00
80	->	17	0.00	0.00
80	->	18	-0.00	0.00
80	->	19	-0.00	0.00
80	->	20	-0.00	0.00
80	->	21	0.00	0.00
80	->	22	0.00	0.00

80	->	23	0.00	0.00
80	->	24	0.00	0.00
80	->	25	0.00	0.00
80	->	26	0.00	0.00
80	->	27	0.00	0.00
80	->	28	0.00	0.00
80	->	29	0.00	0.00
80	->	30	-0.00	0.00
80	->	31	0.00	0.00
80	->	32	0.00	0.00
80	->	33	0.00	0.00
80	->	34	0.00	0.00
80	->	35	-0.00	0.00
80	->	36	0.00	0.00
80	->	37	0.00	0.00
80	->	38	-0.00	0.00
80	->	39	0.00	0.00
80	->	40	0.00	0.00
80	->	41	0.00	0.00
80	->	42	0.00	0.00
80	->	43	0.00	0.00
80	->	44	0.00	0.00
80	->	45	-0.00	0.00
80	->	46	-0.00	0.00
80	->	47	0.00	0.00
80	->	48	-0.00	0.00
80	->	49	-0.00	0.00
80	->	50	0.00	0.00
80	->	51	0.00	0.00
80	->	52	0.00	0.00
80	->	53	0.00	0.00
80	->	54	0.00	0.00
80	->	55	0.00	0.00
80	->	56	0.00	0.00
80	->	57	0.00	0.00
80	->	58	-0.00	0.00
80	->	59	-0.00	0.00
80	->	60	-0.00	0.00
80	->	61	-0.01	0.01
80	->	62	-0.02	0.02
80	->	63	0.10	0.20
80	->	64	-0.12	0.20
80	->	65	-0.14	0.19
80	->	66	0.03	0.01
80	->	67	-0.00	0.00
80	->	68	-0.03	0.01
80	->	69	-0.01	0.01
80	->	70	-0.05	0.04
80	->	71	-0.00	0.00
80	->	72	-0.01	0.01

80	->	73	-0.01	0.01
80	->	74	-0.04	0.01
80	->	75	-0.17	0.06
80	->	76	-3.06	1.36
80	->	77	-1.96	0.39
80	->	78	-0.65	0.16
80	->	79	-5.04	0.48
80	->	80	-23.53	1.96
80	->	81	-15.26	0.39
80	->	82	-0.84	0.22
80	->	83	-2.59	0.75
80	->	84	-1.14	0.88
80	->	85	-0.01	0.01
80	->	86	-0.01	0.00
80	->	87	-0.00	0.00
80	->	88	-0.00	0.00
80	->	89	-0.00	0.00
80	->	90	-0.00	0.00
80	->	91	-0.00	0.00
80	->	92	-0.00	0.00
80	->	93	-0.00	0.00
80	->	94	-0.00	0.00
80	->	95	-0.00	0.00
80	->	96	-0.00	0.00
80	->	97	-0.00	0.00
80	->	98	-0.00	0.00
80	->	99	-0.00	0.00
80	->	100	-0.00	0.00
80	->	101	-0.01	0.00
80	->	102	-0.02	0.01
80	->	103	-0.02	0.02
80	->	104	-0.01	0.00
80	->	105	-0.00	0.00
80	->	106	-0.00	0.00
81	->	1	0.01	0.01
81	->	2	0.00	0.00
81	->	3	-0.00	0.00
81	->	4	0.00	0.00
81	->	5	-0.00	0.00
81	->	6	0.00	0.00
81	->	7	-0.00	0.00
81	->	8	-0.00	0.00
81	->	9	-0.00	0.00
81	->	10	-0.01	0.00
81	->	11	-0.01	0.00
81	->	12	0.01	0.00
81	->	13	0.00	0.00
81	->	14	0.00	0.00
81	->	15	0.00	0.00
81	->	16	-0.01	0.00

81	->	17	-0.00	0.00
81	->	18	-0.00	0.00
81	->	19	-0.00	0.00
81	->	20	-0.00	0.00
81	->	21	0.00	0.00
81	->	22	0.01	0.00
81	->	23	0.00	0.00
81	->	24	0.00	0.00
81	->	25	0.00	0.00
81	->	26	0.00	0.00
81	->	27	0.00	0.00
81	->	28	0.00	0.00
81	->	29	0.00	0.00
81	->	30	-0.00	0.00
81	->	31	0.01	0.00
81	->	32	0.00	0.00
81	->	33	-0.00	0.00
81	->	34	0.00	0.00
81	->	35	-0.00	0.00
81	->	36	0.00	0.00
81	->	37	-0.00	0.00
81	->	38	-0.00	0.00
81	->	39	0.00	0.00
81	->	40	0.01	0.00
81	->	41	-0.00	0.00
81	->	42	0.00	0.00
81	->	43	0.00	0.00
81	->	44	0.02	0.00
81	->	45	-0.00	0.00
81	->	46	-0.02	0.00
81	->	47	0.00	0.00
81	->	48	-0.00	0.00
81	->	49	0.00	0.00
81	->	50	0.00	0.00
81	->	51	0.00	0.00
81	->	52	0.00	0.00
81	->	53	0.01	0.00
81	->	54	0.00	0.00
81	->	55	0.00	0.00
81	->	56	0.00	0.00
81	->	57	0.00	0.00
81	->	58	-0.01	0.00
81	->	59	0.02	0.00
81	->	60	-0.00	0.00
81	->	61	-0.00	0.00
81	->	62	-0.01	0.00
81	->	63	-0.01	0.02
81	->	64	-0.03	0.01
81	->	65	-0.01	0.00
81	->	66	-0.03	0.01

81	->	67	-0.00	0.00
81	->	68	0.02	0.01
81	->	69	0.01	0.00
81	->	70	-0.00	0.00
81	->	71	0.00	0.00
81	->	72	0.03	0.01
81	->	73	-0.01	0.00
81	->	74	-0.10	0.03
81	->	75	-0.08	0.02
81	->	76	-0.01	0.05
81	->	77	-1.47	0.55
81	->	78	-1.92	0.60
81	->	79	-1.10	0.26
81	->	80	-15.29	0.38
81	->	81	4.75	2.75
81	->	82	-11.34	0.58
81	->	83	-0.69	0.25
81	->	84	-1.66	0.67
81	->	85	-0.07	0.21
81	->	86	-0.06	0.04
81	->	87	-0.02	0.01
81	->	88	-0.02	0.00
81	->	89	-0.02	0.00
81	->	90	-0.01	0.00
81	->	91	-0.01	0.00
81	->	92	-0.01	0.00
81	->	93	-0.01	0.00
81	->	94	-0.01	0.00
81	->	95	-0.00	0.00
81	->	96	-0.00	0.00
81	->	97	-0.00	0.00
81	->	98	-0.00	0.00
81	->	99	-0.01	0.00
81	->	100	-0.02	0.01
81	->	101	-0.04	0.02
81	->	102	-0.20	0.30
81	->	103	-1.08	2.21
81	->	104	-0.35	0.81
81	->	105	-0.07	0.04
81	->	106	-0.02	0.02
82	->	1	0.00	0.00
82	->	2	0.00	0.00
82	->	3	-0.00	0.00
82	->	4	0.00	0.00
82	->	5	-0.00	0.00
82	->	6	0.00	0.00
82	->	7	0.00	0.00
82	->	8	-0.00	0.00
82	->	9	0.00	0.00
82	->	10	-0.00	0.00

82 ->	11	-0.00	0.00
82 ->	12	0.00	0.00
82 ->	13	0.00	0.00
82 ->	14	0.00	0.00
82 ->	15	0.00	0.00
82 ->	16	-0.00	0.00
82 ->	17	0.00	0.00
82 ->	18	0.00	0.00
82 ->	19	0.00	0.00
82 ->	20	0.00	0.00
82 ->	21	0.00	0.00
82 ->	22	0.00	0.00
82 ->	23	0.00	0.00
82 ->	24	0.00	0.00
82 ->	25	0.00	0.00
82 ->	26	0.00	0.00
82 ->	27	0.00	0.00
82 ->	28	0.00	0.00
82 ->	29	0.00	0.00
82 ->	30	0.00	0.00
82 ->	31	0.00	0.00
82 ->	32	0.00	0.00
82 ->	33	0.00	0.00
82 ->	34	0.00	0.00
82 ->	35	-0.00	0.00
82 ->	36	0.00	0.00
82 ->	37	0.00	0.00
82 ->	38	-0.00	0.00
82 ->	39	0.00	0.00
82 ->	40	0.00	0.00
82 ->	41	0.00	0.00
82 ->	42	0.00	0.00
82 ->	43	0.00	0.00
82 ->	44	0.00	0.00
82 ->	45	-0.00	0.00
82 ->	46	-0.00	0.00
82 ->	47	0.00	0.00
82 ->	48	-0.00	0.00
82 ->	49	0.00	0.00
82 ->	50	-0.00	0.00
82 ->	51	0.00	0.00
82 ->	52	0.00	0.00
82 ->	53	0.00	0.00
82 ->	54	-0.00	0.00
82 ->	55	0.00	0.00
82 ->	56	-0.00	0.00
82 ->	57	-0.00	0.00
82 ->	58	-0.00	0.00
82 ->	59	0.00	0.00
82 ->	60	-0.00	0.00

82	->	61	-0.00	0.00
82	->	62	-0.00	0.00
82	->	63	-0.07	0.03
82	->	64	-0.01	0.00
82	->	65	-0.05	0.02
82	->	66	-0.06	0.02
82	->	67	-0.00	0.00
82	->	68	-0.03	0.02
82	->	69	-0.00	0.00
82	->	70	-0.04	0.01
82	->	71	-0.00	0.00
82	->	72	-0.00	0.00
82	->	73	-0.00	0.00
82	->	74	-0.01	0.00
82	->	75	-0.04	0.02
82	->	76	-0.05	0.02
82	->	77	-0.05	0.03
82	->	78	-1.23	0.80
82	->	79	-3.94	0.72
82	->	80	-0.85	0.22
82	->	81	-11.44	0.58
82	->	82	17.37	1.78
82	->	83	-21.94	0.83
82	->	84	-0.99	0.36
82	->	85	-0.39	0.67
82	->	86	-0.05	0.05
82	->	87	-0.01	0.00
82	->	88	-0.00	0.00
82	->	89	-0.00	0.00
82	->	90	-0.00	0.00
82	->	91	-0.00	0.00
82	->	92	-0.00	0.00
82	->	93	-0.00	0.00
82	->	94	-0.00	0.00
82	->	95	-0.00	0.00
82	->	96	-0.00	0.00
82	->	97	-0.00	0.00
82	->	98	-0.00	0.00
82	->	99	-0.00	0.00
82	->	100	-0.00	0.00
82	->	101	-0.00	0.00
82	->	102	-0.00	0.00
82	->	103	-0.01	0.00
82	->	104	-0.01	0.00
82	->	105	-0.01	0.00
82	->	106	-0.01	0.01
83	->	1	0.00	0.00
83	->	2	0.00	0.00
83	->	3	0.00	0.00
83	->	4	0.00	0.00

83	->	5	-0.00	0.00
83	->	6	0.00	0.00
83	->	7	0.00	0.00
83	->	8	-0.00	0.00
83	->	9	0.00	0.00
83	->	10	-0.00	0.00
83	->	11	-0.00	0.00
83	->	12	0.00	0.00
83	->	13	0.00	0.00
83	->	14	0.00	0.00
83	->	15	0.00	0.00
83	->	16	0.00	0.00
83	->	17	0.00	0.00
83	->	18	0.00	0.00
83	->	19	0.00	0.00
83	->	20	-0.00	0.00
83	->	21	0.00	0.00
83	->	22	0.00	0.00
83	->	23	0.00	0.00
83	->	24	0.00	0.00
83	->	25	0.00	0.00
83	->	26	0.00	0.00
83	->	27	0.00	0.00
83	->	28	0.00	0.00
83	->	29	0.00	0.00
83	->	30	-0.00	0.00
83	->	31	0.00	0.00
83	->	32	0.00	0.00
83	->	33	0.00	0.00
83	->	34	0.00	0.00
83	->	35	0.00	0.00
83	->	36	-0.00	0.00
83	->	37	0.00	0.00
83	->	38	0.00	0.00
83	->	39	0.00	0.00
83	->	40	-0.00	0.00
83	->	41	0.00	0.00
83	->	42	0.00	0.00
83	->	43	0.00	0.00
83	->	44	0.00	0.00
83	->	45	0.00	0.00
83	->	46	0.00	0.00
83	->	47	0.00	0.00
83	->	48	0.00	0.00
83	->	49	0.00	0.00
83	->	50	0.00	0.00
83	->	51	0.00	0.00
83	->	52	0.00	0.00
83	->	53	-0.00	0.00
83	->	54	0.00	0.00

83	->	55	0.00	0.00
83	->	56	0.00	0.00
83	->	57	0.00	0.00
83	->	58	0.00	0.00
83	->	59	-0.01	0.00
83	->	60	-0.00	0.00
83	->	61	-0.01	0.00
83	->	62	-0.04	0.01
83	->	63	2.19	0.29
83	->	64	-0.20	0.13
83	->	65	0.28	0.79
83	->	66	1.97	0.37
83	->	67	-0.02	0.04
83	->	68	-0.22	0.16
83	->	69	-0.02	0.01
83	->	70	-0.01	0.01
83	->	71	0.00	0.00
83	->	72	-0.00	0.00
83	->	73	-0.00	0.00
83	->	74	-0.01	0.00
83	->	75	-0.02	0.01
83	->	76	-0.08	0.02
83	->	77	-0.03	0.01
83	->	78	-0.11	0.04
83	->	79	20.33	12.71
83	->	80	-2.60	0.75
83	->	81	-0.69	0.25
83	->	82	-22.04	0.83
83	->	83	4.80	1.60
83	->	84	-5.07	0.49
83	->	85	-0.01	0.01
83	->	86	-0.01	0.00
83	->	87	-0.00	0.00
83	->	88	-0.00	0.00
83	->	89	-0.00	0.00
83	->	90	-0.00	0.00
83	->	91	-0.00	0.00
83	->	92	-0.00	0.00
83	->	93	-0.00	0.00
83	->	94	-0.00	0.00
83	->	95	0.00	0.00
83	->	96	0.00	0.00
83	->	97	0.00	0.00
83	->	98	0.00	0.00
83	->	99	0.00	0.00
83	->	100	-0.00	0.00
83	->	101	-0.00	0.00
83	->	102	-0.00	0.00
83	->	103	-0.00	0.00
83	->	104	-0.00	0.00

83	->	105	-0.00	0.00
83	->	106	-0.00	0.00
84	->	1	-0.00	0.00
84	->	2	-0.00	0.00
84	->	3	0.00	0.00
84	->	4	0.00	0.00
84	->	5	0.00	0.00
84	->	6	-0.00	0.00
84	->	7	0.00	0.00
84	->	8	0.00	0.00
84	->	9	0.00	0.00
84	->	10	0.00	0.00
84	->	11	0.00	0.00
84	->	12	-0.00	0.00
84	->	13	-0.00	0.00
84	->	14	-0.00	0.00
84	->	15	-0.00	0.00
84	->	16	0.00	0.00
84	->	17	0.00	0.00
84	->	18	0.00	0.00
84	->	19	0.00	0.00
84	->	20	0.00	0.00
84	->	21	-0.00	0.00
84	->	22	-0.00	0.00
84	->	23	-0.00	0.00
84	->	24	-0.00	0.00
84	->	25	-0.00	0.00
84	->	26	-0.00	0.00
84	->	27	-0.00	0.00
84	->	28	-0.00	0.00
84	->	29	-0.00	0.00
84	->	30	0.00	0.00
84	->	31	-0.00	0.00
84	->	32	-0.00	0.00
84	->	33	-0.00	0.00
84	->	34	-0.00	0.00
84	->	35	0.00	0.00
84	->	36	-0.00	0.00
84	->	37	0.00	0.00
84	->	38	0.00	0.00
84	->	39	-0.00	0.00
84	->	40	-0.00	0.00
84	->	41	0.00	0.00
84	->	42	-0.00	0.00
84	->	43	-0.00	0.00
84	->	44	-0.00	0.00
84	->	45	0.00	0.00
84	->	46	0.01	0.00
84	->	47	-0.00	0.00
84	->	48	0.00	0.00

84	->	49	0.00	0.00
84	->	50	0.00	0.00
84	->	51	-0.00	0.00
84	->	52	0.00	0.00
84	->	53	-0.01	0.00
84	->	54	0.00	0.00
84	->	55	-0.00	0.00
84	->	56	0.00	0.00
84	->	57	0.00	0.00
84	->	58	0.01	0.00
84	->	59	-0.01	0.00
84	->	60	0.00	0.00
84	->	61	-0.00	0.00
84	->	62	-0.00	0.00
84	->	63	0.06	0.01
84	->	64	0.03	0.01
84	->	65	-0.08	0.07
84	->	66	0.05	0.00
84	->	67	-0.00	0.00
84	->	68	-0.03	0.01
84	->	69	-0.01	0.00
84	->	70	-0.00	0.00
84	->	71	-0.00	0.00
84	->	72	-0.01	0.00
84	->	73	0.00	0.00
84	->	74	0.02	0.00
84	->	75	0.02	0.00
84	->	76	-0.05	0.02
84	->	77	-0.06	0.02
84	->	78	-0.06	0.01
84	->	79	-0.04	0.05
84	->	80	-1.16	0.88
84	->	81	-1.68	0.67
84	->	82	-1.01	0.37
84	->	83	-5.18	0.48
84	->	84	-90.00	2.07
84	->	85	0.01	0.00
84	->	86	0.02	0.00
84	->	87	0.01	0.00
84	->	88	0.01	0.00
84	->	89	0.00	0.00
84	->	90	0.00	0.00
84	->	91	0.00	0.00
84	->	92	0.00	0.00
84	->	93	0.00	0.00
84	->	94	0.00	0.00
84	->	95	0.00	0.00
84	->	96	0.00	0.00
84	->	97	0.00	0.00
84	->	98	0.00	0.00

84	->	99	0.00	0.00
84	->	100	0.01	0.00
84	->	101	0.01	0.00
84	->	102	0.02	0.00
84	->	103	0.02	0.01
84	->	104	0.01	0.01
84	->	105	0.01	0.00
84	->	106	0.00	0.00
85	->	1	-0.00	0.00
85	->	2	-0.00	0.00
85	->	3	0.00	0.00
85	->	4	-0.00	0.00
85	->	5	0.00	0.00
85	->	6	0.00	0.00
85	->	7	0.00	0.00
85	->	8	0.00	0.00
85	->	9	0.00	0.00
85	->	10	0.00	0.00
85	->	11	0.00	0.00
85	->	12	-0.00	0.00
85	->	13	-0.00	0.00
85	->	14	-0.00	0.00
85	->	15	-0.00	0.00
85	->	16	0.00	0.00
85	->	17	0.00	0.00
85	->	18	0.00	0.00
85	->	19	0.00	0.00
85	->	20	0.00	0.00
85	->	21	-0.00	0.00
85	->	22	-0.00	0.00
85	->	23	-0.00	0.00
85	->	24	0.00	0.00
85	->	25	-0.00	0.00
85	->	26	-0.00	0.00
85	->	27	-0.00	0.00
85	->	28	-0.00	0.00
85	->	29	0.00	0.00
85	->	30	0.00	0.00
85	->	31	-0.00	0.00
85	->	32	-0.00	0.00
85	->	33	0.00	0.00
85	->	34	-0.00	0.00
85	->	35	0.00	0.00
85	->	36	-0.00	0.00
85	->	37	-0.00	0.00
85	->	38	0.00	0.00
85	->	39	-0.00	0.00
85	->	40	-0.01	0.00
85	->	41	0.00	0.00
85	->	42	-0.00	0.00

85	->	43	-0.00	0.00
85	->	44	-0.00	0.00
85	->	45	0.00	0.00
85	->	46	0.00	0.00
85	->	47	-0.00	0.00
85	->	48	-0.00	0.00
85	->	49	-0.00	0.00
85	->	50	-0.00	0.00
85	->	51	-0.00	0.00
85	->	52	-0.00	0.00
85	->	53	-0.01	0.01
85	->	54	-0.01	0.01
85	->	55	-0.00	0.00
85	->	56	-0.00	0.00
85	->	57	-0.00	0.00
85	->	58	0.00	0.00
85	->	59	-0.11	0.20
85	->	60	-0.00	0.00
85	->	61	-0.00	0.00
85	->	62	-0.00	0.00
85	->	63	0.01	0.00
85	->	64	0.00	0.00
85	->	65	-0.00	0.00
85	->	66	0.01	0.00
85	->	67	-0.00	0.00
85	->	68	-1.48	1.23
85	->	69	-0.03	0.03
85	->	70	-0.15	0.29
85	->	71	-0.01	0.00
85	->	72	-0.03	0.01
85	->	73	-0.00	0.00
85	->	74	0.00	0.01
85	->	75	-0.08	0.11
85	->	76	-0.02	0.01
85	->	77	-0.02	0.01
85	->	78	-1.36	1.33
85	->	79	-0.17	0.21
85	->	80	-0.01	0.01
85	->	81	-0.07	0.21
85	->	82	-0.38	0.66
85	->	83	-0.01	0.01
85	->	84	0.01	0.00
85	->	85	-86.45	3.02
85	->	86	-35.27	1.18
85	->	87	-0.27	0.08
85	->	88	-0.04	0.01
85	->	89	-0.00	0.00
85	->	90	0.00	0.00
85	->	91	0.00	0.00
85	->	92	0.00	0.00

85	->	93	0.00	0.00
85	->	94	0.00	0.00
85	->	95	0.00	0.00
85	->	96	0.00	0.00
85	->	97	0.00	0.00
85	->	98	0.00	0.00
85	->	99	0.00	0.00
85	->	100	0.00	0.00
85	->	101	0.00	0.00
85	->	102	0.00	0.00
85	->	103	-0.00	0.00
85	->	104	-0.03	0.01
85	->	105	-0.22	0.07
85	->	106	-2.76	1.54
86	->	1	-0.01	0.00
86	->	2	0.00	0.00
86	->	3	0.00	0.00
86	->	4	-0.00	0.00
86	->	5	0.00	0.00
86	->	6	-0.00	0.00
86	->	7	0.00	0.00
86	->	8	0.00	0.00
86	->	9	0.00	0.00
86	->	10	0.00	0.00
86	->	11	0.00	0.00
86	->	12	-0.01	0.00
86	->	13	-0.00	0.00
86	->	14	-0.00	0.00
86	->	15	-0.00	0.00
86	->	16	0.00	0.00
86	->	17	0.00	0.00
86	->	18	0.00	0.00
86	->	19	0.00	0.00
86	->	20	0.00	0.00
86	->	21	-0.00	0.00
86	->	22	-0.01	0.00
86	->	23	-0.00	0.00
86	->	24	0.00	0.00
86	->	25	-0.01	0.00
86	->	26	-0.00	0.00
86	->	27	-0.00	0.00
86	->	28	0.00	0.00
86	->	29	-0.00	0.00
86	->	30	0.00	0.00
86	->	31	-0.01	0.00
86	->	32	0.00	0.00
86	->	33	-0.00	0.00
86	->	34	-0.00	0.00
86	->	35	0.02	0.00
86	->	36	-0.01	0.00

86	->	37	-0.00	0.00
86	->	38	0.02	0.01
86	->	39	-0.00	0.00
86	->	40	-0.05	0.02
86	->	41	-0.00	0.00
86	->	42	-0.01	0.00
86	->	43	-0.00	0.00
86	->	44	-0.02	0.00
86	->	45	0.00	0.00
86	->	46	0.02	0.00
86	->	47	-0.01	0.00
86	->	48	-0.00	0.00
86	->	49	-0.01	0.00
86	->	50	-0.02	0.02
86	->	51	-0.02	0.01
86	->	52	-0.00	0.00
86	->	53	-0.10	0.05
86	->	54	-0.14	0.23
86	->	55	0.00	0.01
86	->	56	-0.01	0.00
86	->	57	-0.02	0.01
86	->	58	0.02	0.01
86	->	59	-1.12	1.45
86	->	60	-0.01	0.00
86	->	61	-0.01	0.00
86	->	62	-0.00	0.00
86	->	63	0.04	0.01
86	->	64	0.02	0.00
86	->	65	0.00	0.00
86	->	66	0.06	0.01
86	->	67	-0.00	0.00
86	->	68	-5.41	4.14
86	->	69	-0.11	0.06
86	->	70	-0.23	0.17
86	->	71	-0.10	0.09
86	->	72	-0.20	0.13
86	->	73	-0.00	0.00
86	->	74	0.01	0.03
86	->	75	-0.44	0.65
86	->	76	-0.05	0.02
86	->	77	-0.05	0.03
86	->	78	-5.69	2.36
86	->	79	-0.08	0.12
86	->	80	-0.01	0.00
86	->	81	-0.06	0.04
86	->	82	-0.04	0.05
86	->	83	-0.01	0.00
86	->	84	0.02	0.00
86	->	85	-35.10	1.17
86	->	86	-173.11	4.57

86	->	87	-35.97	1.00
86	->	88	-0.39	0.10
86	->	89	-0.02	0.01
86	->	90	0.01	0.00
86	->	91	0.01	0.00
86	->	92	0.00	0.00
86	->	93	0.00	0.00
86	->	94	0.00	0.00
86	->	95	0.00	0.00
86	->	96	0.00	0.00
86	->	97	0.00	0.00
86	->	98	0.01	0.00
86	->	99	0.01	0.00
86	->	100	0.01	0.01
86	->	101	0.01	0.00
86	->	102	0.01	0.00
86	->	103	-0.01	0.01
86	->	104	-0.12	0.06
86	->	105	-1.27	0.74
86	->	106	-10.71	0.75
87	->	1	-0.01	0.00
87	->	2	-0.00	0.00
87	->	3	0.00	0.00
87	->	4	-0.00	0.00
87	->	5	0.00	0.00
87	->	6	-0.00	0.00
87	->	7	-0.00	0.00
87	->	8	0.00	0.00
87	->	9	-0.00	0.00
87	->	10	0.00	0.00
87	->	11	0.00	0.00
87	->	12	-0.01	0.00
87	->	13	-0.00	0.00
87	->	14	-0.00	0.00
87	->	15	-0.00	0.00
87	->	16	0.01	0.00
87	->	17	0.00	0.00
87	->	18	0.00	0.00
87	->	19	0.00	0.00
87	->	20	0.00	0.00
87	->	21	0.00	0.00
87	->	22	-0.01	0.00
87	->	23	-0.00	0.00
87	->	24	0.00	0.00
87	->	25	-0.01	0.00
87	->	26	-0.00	0.00
87	->	27	-0.00	0.00
87	->	28	-0.00	0.00
87	->	29	-0.00	0.00
87	->	30	0.00	0.00

87	->	31	-0.04	0.02
87	->	32	-0.00	0.00
87	->	33	-0.00	0.00
87	->	34	-0.01	0.01
87	->	35	0.06	0.02
87	->	36	-0.02	0.00
87	->	37	-0.00	0.00
87	->	38	0.09	0.04
87	->	39	-0.00	0.00
87	->	40	-1.00	0.62
87	->	41	-0.01	0.01
87	->	42	-0.02	0.03
87	->	43	-0.00	0.00
87	->	44	-0.03	0.01
87	->	45	-0.00	0.00
87	->	46	0.02	0.01
87	->	47	-0.05	0.02
87	->	48	0.00	0.01
87	->	49	-0.03	0.02
87	->	50	-0.46	0.51
87	->	51	-0.65	0.75
87	->	52	-0.00	0.02
87	->	53	-0.24	0.12
87	->	54	-2.12	1.21
87	->	55	-0.03	0.06
87	->	56	-0.02	0.01
87	->	57	-0.04	0.02
87	->	58	0.03	0.01
87	->	59	-1.14	1.67
87	->	60	-0.01	0.00
87	->	61	-0.01	0.00
87	->	62	-0.00	0.00
87	->	63	0.02	0.01
87	->	64	0.01	0.00
87	->	65	0.00	0.00
87	->	66	0.02	0.01
87	->	67	-0.00	0.00
87	->	68	-0.26	0.58
87	->	69	-0.06	0.04
87	->	70	-0.12	0.09
87	->	71	-0.23	0.23
87	->	72	-1.99	0.88
87	->	73	-0.01	0.00
87	->	74	-0.37	0.24
87	->	75	-0.90	0.59
87	->	76	-0.03	0.01
87	->	77	-0.01	0.01
87	->	78	-0.75	0.81
87	->	79	-0.02	0.01
87	->	80	-0.00	0.00

87	->	81	-0.02	0.01
87	->	82	-0.01	0.00
87	->	83	-0.00	0.00
87	->	84	0.01	0.00
87	->	85	-0.27	0.08
87	->	86	-35.76	1.00
87	->	87	-210.98	4.05
87	->	88	-31.88	1.10
87	->	89	-0.27	0.08
87	->	90	0.00	0.01
87	->	91	0.01	0.00
87	->	92	0.01	0.00
87	->	93	0.00	0.00
87	->	94	0.00	0.00
87	->	95	0.00	0.00
87	->	96	0.00	0.00
87	->	97	0.01	0.00
87	->	98	0.01	0.00
87	->	99	0.01	0.00
87	->	100	0.01	0.00
87	->	101	-0.00	0.00
87	->	102	-0.02	0.01
87	->	103	-0.09	0.04
87	->	104	-0.48	0.25
87	->	105	-11.21	0.83
87	->	106	0.15	0.13
88	->	1	-0.01	0.00
88	->	2	-0.00	0.00
88	->	3	0.00	0.00
88	->	4	-0.00	0.00
88	->	5	0.01	0.00
88	->	6	-0.00	0.00
88	->	7	-0.00	0.00
88	->	8	0.00	0.00
88	->	9	0.00	0.00
88	->	10	0.01	0.00
88	->	11	0.00	0.00
88	->	12	-0.01	0.00
88	->	13	-0.00	0.00
88	->	14	-0.00	0.00
88	->	15	-0.00	0.00
88	->	16	0.01	0.00
88	->	17	0.00	0.00
88	->	18	0.00	0.00
88	->	19	0.00	0.00
88	->	20	-0.00	0.00
88	->	21	-0.00	0.00
88	->	22	-0.03	0.01
88	->	23	-0.00	0.00
88	->	24	-0.00	0.00

88	->	25	-0.03	0.01
88	->	26	-0.01	0.00
88	->	27	-0.00	0.00
88	->	28	-0.00	0.00
88	->	29	-0.00	0.00
88	->	30	-0.00	0.00
88	->	31	-0.48	0.53
88	->	32	-0.00	0.00
88	->	33	-0.01	0.01
88	->	34	-0.03	0.02
88	->	35	0.11	0.06
88	->	36	-0.03	0.00
88	->	37	-0.00	0.01
88	->	38	0.19	0.13
88	->	39	-0.02	0.01
88	->	40	-7.65	3.08
88	->	41	-0.06	0.05
88	->	42	-0.69	0.80
88	->	43	0.01	0.03
88	->	44	-0.07	0.02
88	->	45	-0.00	0.01
88	->	46	0.02	0.03
88	->	47	-0.31	0.13
88	->	48	0.03	0.05
88	->	49	-0.06	0.05
88	->	50	-0.75	0.84
88	->	51	-3.01	3.44
88	->	52	0.05	0.06
88	->	53	-0.13	0.06
88	->	54	-0.04	0.15
88	->	55	0.02	0.05
88	->	56	-0.01	0.00
88	->	57	-0.01	0.00
88	->	58	0.03	0.00
88	->	59	-0.06	0.01
88	->	60	-0.00	0.00
88	->	61	-0.00	0.00
88	->	62	-0.00	0.00
88	->	63	0.01	0.00
88	->	64	0.01	0.00
88	->	65	0.00	0.00
88	->	66	0.01	0.00
88	->	67	0.00	0.00
88	->	68	-0.04	0.01
88	->	69	-0.02	0.00
88	->	70	-0.02	0.01
88	->	71	-0.03	0.01
88	->	72	-7.98	2.89
88	->	73	-0.01	0.01
88	->	74	0.52	0.23

88	->	75	0.05	0.04
88	->	76	-0.02	0.00
88	->	77	-0.03	0.01
88	->	78	-0.16	0.08
88	->	79	-0.01	0.00
88	->	80	-0.00	0.00
88	->	81	-0.02	0.00
88	->	82	-0.00	0.00
88	->	83	-0.00	0.00
88	->	84	0.01	0.00
88	->	85	-0.04	0.01
88	->	86	-0.39	0.10
88	->	87	-31.70	1.09
88	->	88	-165.41	6.12
88	->	89	-34.76	1.08
88	->	90	-0.25	0.08
88	->	91	-0.01	0.01
88	->	92	0.01	0.00
88	->	93	0.01	0.00
88	->	94	0.01	0.00
88	->	95	0.00	0.00
88	->	96	0.00	0.00
88	->	97	0.01	0.00
88	->	98	0.02	0.00
88	->	99	0.02	0.01
88	->	100	0.01	0.00
88	->	101	-0.01	0.01
88	->	102	-0.11	0.04
88	->	103	-2.10	0.71
88	->	104	-11.41	0.77
88	->	105	-5.63	1.27
88	->	106	-0.25	0.07
89	->	1	-0.02	0.01
89	->	2	-0.00	0.00
89	->	3	-0.00	0.00
89	->	4	-0.00	0.00
89	->	5	0.02	0.00
89	->	6	-0.00	0.00
89	->	7	-0.00	0.00
89	->	8	0.01	0.00
89	->	9	-0.00	0.00
89	->	10	0.02	0.00
89	->	11	0.01	0.00
89	->	12	-0.04	0.01
89	->	13	-0.01	0.00
89	->	14	-0.00	0.00
89	->	15	-0.00	0.00
89	->	16	0.01	0.00
89	->	17	0.00	0.00
89	->	18	-0.00	0.00

89	->	19	-0.01	0.00
89	->	20	-0.00	0.00
89	->	21	-0.01	0.00
89	->	22	-0.19	0.08
89	->	23	-0.02	0.01
89	->	24	-0.00	0.00
89	->	25	-0.11	0.03
89	->	26	-0.14	0.07
89	->	27	0.01	0.01
89	->	28	-0.00	0.00
89	->	29	-0.01	0.01
89	->	30	-0.01	0.01
89	->	31	-3.26	3.28
89	->	32	-0.01	0.00
89	->	33	-0.01	0.01
89	->	34	-0.02	0.01
89	->	35	0.04	0.01
89	->	36	-0.02	0.00
89	->	37	-0.00	0.00
89	->	38	0.05	0.01
89	->	39	-0.00	0.00
89	->	40	-2.53	2.75
89	->	41	-0.10	0.08
89	->	42	-0.64	0.47
89	->	43	-0.44	0.38
89	->	44	-0.26	0.10
89	->	45	-0.00	0.01
89	->	46	-0.07	0.10
89	->	47	-2.88	0.61
89	->	48	-0.01	0.01
89	->	49	-0.02	0.01
89	->	50	-0.35	0.25
89	->	51	-0.06	0.02
89	->	52	-0.01	0.00
89	->	53	-0.04	0.01
89	->	54	-0.01	0.00
89	->	55	-0.00	0.00
89	->	56	-0.00	0.00
89	->	57	-0.00	0.00
89	->	58	0.01	0.00
89	->	59	-0.02	0.00
89	->	60	-0.00	0.00
89	->	61	-0.00	0.00
89	->	62	0.00	0.00
89	->	63	0.01	0.00
89	->	64	0.00	0.00
89	->	65	0.00	0.00
89	->	66	0.01	0.00
89	->	67	0.00	0.00
89	->	68	-0.02	0.00

89	->	69	-0.01	0.00
89	->	70	-0.01	0.00
89	->	71	-0.01	0.00
89	->	72	-2.96	1.29
89	->	73	-0.01	0.00
89	->	74	0.21	0.13
89	->	75	0.01	0.01
89	->	76	-0.02	0.00
89	->	77	-0.02	0.00
89	->	78	-0.05	0.01
89	->	79	-0.00	0.00
89	->	80	-0.00	0.00
89	->	81	-0.02	0.00
89	->	82	-0.00	0.00
89	->	83	-0.00	0.00
89	->	84	0.00	0.00
89	->	85	-0.00	0.00
89	->	86	-0.02	0.01
89	->	87	-0.27	0.08
89	->	88	-34.57	1.06
89	->	89	-87.78	4.43
89	->	90	-33.12	1.03
89	->	91	-0.12	0.08
89	->	92	0.01	0.01
89	->	93	0.01	0.00
89	->	94	0.01	0.00
89	->	95	0.00	0.00
89	->	96	0.00	0.00
89	->	97	0.01	0.00
89	->	98	0.01	0.00
89	->	99	0.01	0.00
89	->	100	-0.01	0.01
89	->	101	-0.07	0.03
89	->	102	-0.68	0.29
89	->	103	-6.00	0.71
89	->	104	-0.68	0.34
89	->	105	-0.27	0.13
89	->	106	-0.03	0.03
90	->	1	-0.04	0.01
90	->	2	-0.00	0.00
90	->	3	-0.00	0.00
90	->	4	-0.00	0.00
90	->	5	0.05	0.01
90	->	6	-0.00	0.00
90	->	7	-0.01	0.01
90	->	8	0.01	0.00
90	->	9	-0.00	0.00
90	->	10	0.07	0.02
90	->	11	0.02	0.00
90	->	12	-0.52	0.49

90	->	13	-0.03	0.01
90	->	14	-0.01	0.02
90	->	15	-0.00	0.01
90	->	16	0.03	0.00
90	->	17	0.00	0.00
90	->	18	-0.01	0.01
90	->	19	-0.09	0.07
90	->	20	0.01	0.01
90	->	21	-0.03	0.01
90	->	22	-2.71	1.23
90	->	23	-0.73	0.46
90	->	24	0.01	0.02
90	->	25	-0.33	0.12
90	->	26	-2.60	0.74
90	->	27	0.04	0.03
90	->	28	-0.02	0.01
90	->	29	-0.03	0.01
90	->	30	-0.00	0.01
90	->	31	-1.31	1.88
90	->	32	-0.01	0.00
90	->	33	-0.01	0.00
90	->	34	-0.01	0.00
90	->	35	0.02	0.00
90	->	36	-0.01	0.00
90	->	37	0.00	0.00
90	->	38	0.03	0.00
90	->	39	-0.00	0.00
90	->	40	-0.07	0.02
90	->	41	-0.04	0.04
90	->	42	-0.25	0.18
90	->	43	-2.87	2.24
90	->	44	-2.15	1.65
90	->	45	0.00	0.01
90	->	46	0.21	0.26
90	->	47	-2.35	1.00
90	->	48	0.00	0.01
90	->	49	-0.01	0.00
90	->	50	-0.02	0.01
90	->	51	-0.01	0.00
90	->	52	-0.00	0.00
90	->	53	-0.03	0.00
90	->	54	-0.00	0.00
90	->	55	-0.00	0.00
90	->	56	-0.00	0.00
90	->	57	-0.00	0.00
90	->	58	0.01	0.00
90	->	59	-0.01	0.00
90	->	60	-0.00	0.00
90	->	61	-0.00	0.00
90	->	62	0.00	0.00

90	->	63	0.01	0.00
90	->	64	0.00	0.00
90	->	65	0.00	0.00
90	->	66	0.00	0.00
90	->	67	0.00	0.00
90	->	68	-0.01	0.00
90	->	69	-0.01	0.00
90	->	70	-0.00	0.00
90	->	71	-0.00	0.00
90	->	72	-0.25	0.44
90	->	73	-0.00	0.01
90	->	74	-0.04	0.06
90	->	75	0.01	0.00
90	->	76	-0.01	0.00
90	->	77	-0.01	0.01
90	->	78	-0.03	0.00
90	->	79	-0.00	0.00
90	->	80	-0.00	0.00
90	->	81	-0.01	0.00
90	->	82	-0.00	0.00
90	->	83	-0.00	0.00
90	->	84	0.00	0.00
90	->	85	0.00	0.00
90	->	86	0.01	0.00
90	->	87	0.00	0.01
90	->	88	-0.25	0.08
90	->	89	-32.92	1.00
90	->	90	-169.71	3.88
90	->	91	-32.58	1.19
90	->	92	-0.16	0.13
90	->	93	-0.02	0.01
90	->	94	0.01	0.00
90	->	95	0.01	0.00
90	->	96	0.00	0.00
90	->	97	0.02	0.01
90	->	98	0.01	0.00
90	->	99	0.01	0.01
90	->	100	-0.10	0.08
90	->	101	-1.01	0.61
90	->	102	-11.40	0.78
90	->	103	-3.58	0.58
90	->	104	-0.31	0.08
90	->	105	-0.04	0.03
90	->	106	0.01	0.00
91	->	1	-0.57	0.69
91	->	2	-0.01	0.01
91	->	3	-0.00	0.01
91	->	4	-0.02	0.01
91	->	5	0.15	0.06
91	->	6	-0.00	0.00

91	->	7	-0.03	0.02
91	->	8	0.02	0.00
91	->	9	-0.01	0.00
91	->	10	0.17	0.07
91	->	11	0.02	0.01
91	->	12	-6.85	2.41
91	->	13	-0.18	0.07
91	->	14	-0.80	0.58
91	->	15	-0.19	0.40
91	->	16	0.10	0.04
91	->	17	-0.00	0.01
91	->	18	-0.05	0.03
91	->	19	-1.16	0.62
91	->	20	0.11	0.08
91	->	21	-0.11	0.05
91	->	22	-14.99	6.73
91	->	23	-5.05	2.99
91	->	24	0.10	0.07
91	->	25	-0.27	0.11
91	->	26	-0.87	0.37
91	->	27	0.12	0.09
91	->	28	-0.01	0.01
91	->	29	-0.01	0.00
91	->	30	-0.00	0.00
91	->	31	-0.07	0.02
91	->	32	-0.01	0.00
91	->	33	-0.00	0.00
91	->	34	-0.00	0.00
91	->	35	0.01	0.00
91	->	36	-0.01	0.00
91	->	37	0.00	0.00
91	->	38	0.01	0.00
91	->	39	0.00	0.00
91	->	40	-0.03	0.01
91	->	41	-0.00	0.00
91	->	42	-0.02	0.01
91	->	43	-0.07	0.02
91	->	44	-6.59	2.79
91	->	45	-0.00	0.01
91	->	46	0.33	0.19
91	->	47	-0.24	0.14
91	->	48	-0.00	0.00
91	->	49	-0.01	0.00
91	->	50	-0.01	0.01
91	->	51	-0.01	0.00
91	->	52	-0.00	0.00
91	->	53	-0.02	0.00
91	->	54	-0.00	0.00
91	->	55	-0.00	0.00
91	->	56	-0.00	0.00

91	->	57	-0.00	0.00
91	->	58	0.01	0.00
91	->	59	-0.01	0.00
91	->	60	-0.00	0.00
91	->	61	-0.00	0.00
91	->	62	0.00	0.00
91	->	63	0.00	0.00
91	->	64	0.00	0.00
91	->	65	0.00	0.00
91	->	66	0.00	0.00
91	->	67	0.00	0.00
91	->	68	-0.01	0.00
91	->	69	-0.00	0.00
91	->	70	-0.00	0.00
91	->	71	0.00	0.00
91	->	72	-0.02	0.05
91	->	73	-0.01	0.02
91	->	74	-0.04	0.04
91	->	75	0.01	0.00
91	->	76	-0.01	0.00
91	->	77	-0.01	0.01
91	->	78	-0.02	0.00
91	->	79	-0.00	0.00
91	->	80	-0.00	0.00
91	->	81	-0.01	0.00
91	->	82	-0.00	0.00
91	->	83	-0.00	0.00
91	->	84	0.00	0.00
91	->	85	0.00	0.00
91	->	86	0.01	0.00
91	->	87	0.01	0.00
91	->	88	-0.01	0.01
91	->	89	-0.12	0.08
91	->	90	-32.48	1.16
91	->	91	-158.56	5.01
91	->	92	-30.88	1.73
91	->	93	-0.30	0.08
91	->	94	0.00	0.01
91	->	95	0.00	0.00
91	->	96	0.00	0.00
91	->	97	0.02	0.01
91	->	98	-0.01	0.01
91	->	99	-0.00	0.04
91	->	100	-0.89	0.42
91	->	101	-11.59	0.86
91	->	102	-4.14	0.87
91	->	103	-0.36	0.09
91	->	104	-0.03	0.01
91	->	105	0.01	0.00
91	->	106	0.01	0.00

92	->	1	-4.37	4.57
92	->	2	-0.05	0.07
92	->	3	0.00	0.02
92	->	4	-0.01	0.01
92	->	5	0.07	0.02
92	->	6	-0.00	0.00
92	->	7	-0.01	0.00
92	->	8	0.02	0.00
92	->	9	-0.00	0.00
92	->	10	0.05	0.01
92	->	11	0.03	0.01
92	->	12	-2.76	2.46
92	->	13	-0.41	0.23
92	->	14	-2.05	1.44
92	->	15	-3.00	4.50
92	->	16	0.25	0.24
92	->	17	0.01	0.03
92	->	18	-0.15	0.07
92	->	19	-6.06	3.09
92	->	20	0.15	0.08
92	->	21	-0.08	0.05
92	->	22	-10.62	5.88
92	->	23	-0.19	0.14
92	->	24	0.00	0.01
92	->	25	-0.08	0.02
92	->	26	-0.02	0.01
92	->	27	0.01	0.01
92	->	28	-0.00	0.00
92	->	29	-0.00	0.00
92	->	30	0.00	0.00
92	->	31	-0.03	0.00
92	->	32	-0.00	0.00
92	->	33	-0.00	0.00
92	->	34	-0.00	0.00
92	->	35	0.01	0.00
92	->	36	-0.01	0.00
92	->	37	0.00	0.00
92	->	38	0.01	0.00
92	->	39	0.00	0.00
92	->	40	-0.01	0.00
92	->	41	-0.00	0.00
92	->	42	-0.01	0.00
92	->	43	-0.02	0.01
92	->	44	-3.98	2.03
92	->	45	-0.00	0.01
92	->	46	0.14	0.11
92	->	47	-0.03	0.01
92	->	48	-0.00	0.00
92	->	49	-0.00	0.00
92	->	50	-0.01	0.00

92	->	51	-0.00	0.00
92	->	52	-0.00	0.00
92	->	53	-0.02	0.00
92	->	54	-0.00	0.00
92	->	55	-0.00	0.00
92	->	56	-0.00	0.00
92	->	57	-0.00	0.00
92	->	58	0.01	0.00
92	->	59	-0.01	0.00
92	->	60	-0.00	0.00
92	->	61	-0.01	0.00
92	->	62	0.00	0.00
92	->	63	0.00	0.00
92	->	64	0.00	0.00
92	->	65	0.00	0.00
92	->	66	0.00	0.00
92	->	67	0.00	0.00
92	->	68	-0.00	0.00
92	->	69	-0.00	0.00
92	->	70	-0.00	0.00
92	->	71	0.00	0.00
92	->	72	-0.01	0.01
92	->	73	-0.02	0.01
92	->	74	-0.01	0.01
92	->	75	0.01	0.00
92	->	76	-0.01	0.00
92	->	77	-0.02	0.01
92	->	78	-0.02	0.00
92	->	79	-0.00	0.00
92	->	80	-0.00	0.00
92	->	81	-0.01	0.00
92	->	82	-0.00	0.00
92	->	83	-0.00	0.00
92	->	84	0.00	0.00
92	->	85	0.00	0.00
92	->	86	0.00	0.00
92	->	87	0.01	0.00
92	->	88	0.01	0.00
92	->	89	0.01	0.01
92	->	90	-0.16	0.13
92	->	91	-30.74	1.72
92	->	92	-159.57	3.57
92	->	93	-35.47	1.23
92	->	94	-0.25	0.07
92	->	95	-0.01	0.01
92	->	96	-0.00	0.01
92	->	97	-0.01	0.01
92	->	98	-0.14	0.08
92	->	99	-0.29	0.54
92	->	100	-11.67	0.67

92	->	101	-3.87	0.74
92	->	102	-0.40	0.16
92	->	103	-0.04	0.03
92	->	104	0.02	0.00
92	->	105	0.01	0.00
92	->	106	0.01	0.00
93	->	1	-4.54	4.23
93	->	2	-0.02	0.01
93	->	3	-0.01	0.00
93	->	4	-0.00	0.00
93	->	5	0.02	0.00
93	->	6	-0.00	0.00
93	->	7	-0.00	0.00
93	->	8	0.01	0.00
93	->	9	0.00	0.00
93	->	10	0.01	0.00
93	->	11	0.02	0.01
93	->	12	-0.08	0.04
93	->	13	-0.05	0.02
93	->	14	-0.11	0.08
93	->	15	-0.60	1.03
93	->	16	-0.16	0.34
93	->	17	-0.01	0.01
93	->	18	-0.25	0.28
93	->	19	-1.43	1.41
93	->	20	-0.01	0.01
93	->	21	-0.01	0.00
93	->	22	-0.07	0.07
93	->	23	-0.01	0.00
93	->	24	-0.00	0.00
93	->	25	-0.02	0.00
93	->	26	-0.00	0.00
93	->	27	-0.00	0.00
93	->	28	-0.00	0.00
93	->	29	-0.00	0.00
93	->	30	0.00	0.00
93	->	31	-0.01	0.00
93	->	32	-0.00	0.00
93	->	33	-0.00	0.00
93	->	34	0.00	0.00
93	->	35	0.00	0.00
93	->	36	-0.00	0.00
93	->	37	0.00	0.00
93	->	38	0.00	0.00
93	->	39	-0.00	0.00
93	->	40	-0.01	0.00
93	->	41	-0.00	0.00
93	->	42	-0.00	0.00
93	->	43	-0.00	0.00
93	->	44	-0.06	0.13

93	->	45	-0.01	0.01
93	->	46	-0.00	0.03
93	->	47	-0.01	0.00
93	->	48	-0.00	0.00
93	->	49	-0.00	0.00
93	->	50	-0.00	0.00
93	->	51	-0.00	0.00
93	->	52	-0.00	0.00
93	->	53	-0.02	0.00
93	->	54	-0.00	0.00
93	->	55	-0.00	0.00
93	->	56	-0.00	0.00
93	->	57	-0.00	0.00
93	->	58	0.01	0.00
93	->	59	-0.01	0.00
93	->	60	-0.00	0.00
93	->	61	-0.00	0.00
93	->	62	0.00	0.00
93	->	63	0.01	0.00
93	->	64	0.01	0.00
93	->	65	0.00	0.00
93	->	66	0.00	0.00
93	->	67	0.00	0.00
93	->	68	-0.00	0.00
93	->	69	-0.01	0.00
93	->	70	-0.00	0.00
93	->	71	-0.00	0.00
93	->	72	-0.03	0.01
93	->	73	-0.01	0.00
93	->	74	0.02	0.00
93	->	75	0.01	0.00
93	->	76	-0.02	0.01
93	->	77	-0.04	0.01
93	->	78	-0.01	0.00
93	->	79	-0.00	0.00
93	->	80	-0.00	0.00
93	->	81	-0.01	0.00
93	->	82	-0.00	0.00
93	->	83	-0.00	0.00
93	->	84	0.00	0.00
93	->	85	0.00	0.00
93	->	86	0.00	0.00
93	->	87	0.00	0.00
93	->	88	0.01	0.00
93	->	89	0.01	0.00
93	->	90	-0.02	0.01
93	->	91	-0.30	0.08
93	->	92	-35.29	1.22
93	->	93	-213.18	4.33
93	->	94	-33.46	1.22

93	->	95	-0.24	0.07
93	->	96	-0.02	0.01
93	->	97	-0.07	0.04
93	->	98	-0.47	0.33
93	->	99	-11.45	0.64
93	->	100	0.26	0.36
93	->	101	-0.07	0.10
93	->	102	-0.02	0.04
93	->	103	0.01	0.00
93	->	104	0.01	0.00
93	->	105	0.01	0.00
93	->	106	0.00	0.00
94	->	1	-0.12	0.04
94	->	2	-0.01	0.00
94	->	3	-0.01	0.00
94	->	4	-0.00	0.00
94	->	5	0.01	0.00
94	->	6	-0.00	0.00
94	->	7	-0.00	0.00
94	->	8	0.01	0.00
94	->	9	0.00	0.00
94	->	10	0.01	0.00
94	->	11	0.01	0.00
94	->	12	-0.03	0.01
94	->	13	-0.02	0.00
94	->	14	-0.01	0.01
94	->	15	-0.03	0.02
94	->	16	0.02	0.06
94	->	17	-0.01	0.01
94	->	18	-0.02	0.07
94	->	19	-0.04	0.05
94	->	20	-0.01	0.00
94	->	21	-0.01	0.00
94	->	22	-0.06	0.01
94	->	23	-0.01	0.00
94	->	24	-0.00	0.00
94	->	25	-0.02	0.01
94	->	26	-0.00	0.00
94	->	27	-0.00	0.00
94	->	28	-0.00	0.00
94	->	29	-0.00	0.00
94	->	30	0.00	0.00
94	->	31	-0.01	0.00
94	->	32	-0.00	0.00
94	->	33	-0.01	0.00
94	->	34	-0.00	0.00
94	->	35	0.00	0.00
94	->	36	-0.00	0.00
94	->	37	0.00	0.00
94	->	38	0.00	0.00

94 ->	39	-0.00	0.00
94 ->	40	-0.00	0.00
94 ->	41	-0.00	0.00
94 ->	42	-0.00	0.00
94 ->	43	0.00	0.00
94 ->	44	-0.01	0.02
94 ->	45	-0.00	0.02
94 ->	46	-0.03	0.03
94 ->	47	-0.02	0.01
94 ->	48	-0.00	0.00
94 ->	49	-0.01	0.00
94 ->	50	-0.00	0.00
94 ->	51	-0.00	0.00
94 ->	52	-0.00	0.00
94 ->	53	-0.01	0.00
94 ->	54	-0.00	0.00
94 ->	55	-0.00	0.00
94 ->	56	-0.00	0.00
94 ->	57	-0.00	0.00
94 ->	58	0.00	0.00
94 ->	59	-0.01	0.00
94 ->	60	-0.00	0.00
94 ->	61	-0.00	0.00
94 ->	62	0.00	0.00
94 ->	63	0.01	0.00
94 ->	64	0.01	0.00
94 ->	65	0.00	0.00
94 ->	66	0.00	0.00
94 ->	67	0.00	0.00
94 ->	68	-0.00	0.00
94 ->	69	-0.01	0.00
94 ->	70	-0.00	0.00
94 ->	71	0.00	0.00
94 ->	72	-0.02	0.00
94 ->	73	-0.01	0.00
94 ->	74	0.01	0.00
94 ->	75	0.01	0.00
94 ->	76	-0.02	0.01
94 ->	77	-0.03	0.01
94 ->	78	-0.01	0.00
94 ->	79	-0.00	0.00
94 ->	80	-0.00	0.00
94 ->	81	-0.01	0.00
94 ->	82	-0.00	0.00
94 ->	83	-0.00	0.00
94 ->	84	0.00	0.00
94 ->	85	0.00	0.00
94 ->	86	0.00	0.00
94 ->	87	0.00	0.00
94 ->	88	0.01	0.00

94	->	89	0.01	0.00
94	->	90	0.01	0.00
94	->	91	0.00	0.01
94	->	92	-0.25	0.07
94	->	93	-33.15	1.22
94	->	94	-182.85	2.72
94	->	95	-35.70	1.08
94	->	96	-0.17	0.08
94	->	97	-2.46	0.92
94	->	98	-10.95	0.72
94	->	99	-3.99	1.23
94	->	100	-0.28	0.11
94	->	101	-0.06	0.04
94	->	102	0.01	0.00
94	->	103	0.02	0.00
94	->	104	0.01	0.00
94	->	105	0.01	0.00
94	->	106	0.00	0.00
95	->	1	-0.04	0.02
95	->	2	-0.00	0.00
95	->	3	-0.01	0.00
95	->	4	0.00	0.00
95	->	5	0.01	0.00
95	->	6	-0.00	0.00
95	->	7	-0.00	0.00
95	->	8	0.00	0.00
95	->	9	0.00	0.00
95	->	10	0.00	0.00
95	->	11	0.00	0.00
95	->	12	-0.01	0.00
95	->	13	-0.01	0.00
95	->	14	-0.00	0.00
95	->	15	-0.02	0.03
95	->	16	-0.02	0.06
95	->	17	-0.01	0.01
95	->	18	-0.04	0.03
95	->	19	-0.01	0.01
95	->	20	-0.00	0.00
95	->	21	-0.00	0.00
95	->	22	-0.02	0.00
95	->	23	-0.00	0.00
95	->	24	-0.00	0.00
95	->	25	-0.01	0.00
95	->	26	-0.00	0.00
95	->	27	-0.00	0.00
95	->	28	-0.00	0.00
95	->	29	-0.00	0.00
95	->	30	0.00	0.00
95	->	31	-0.00	0.00
95	->	32	-0.00	0.00

95 ->	33	-0.00	0.00
95 ->	34	0.00	0.00
95 ->	35	0.00	0.00
95 ->	36	-0.00	0.00
95 ->	37	0.00	0.00
95 ->	38	0.00	0.00
95 ->	39	-0.00	0.00
95 ->	40	-0.00	0.00
95 ->	41	-0.00	0.00
95 ->	42	-0.00	0.00
95 ->	43	-0.00	0.00
95 ->	44	-0.02	0.00
95 ->	45	-0.00	0.00
95 ->	46	0.01	0.00
95 ->	47	-0.00	0.00
95 ->	48	-0.00	0.00
95 ->	49	-0.00	0.00
95 ->	50	-0.00	0.00
95 ->	51	-0.00	0.00
95 ->	52	-0.00	0.00
95 ->	53	-0.01	0.00
95 ->	54	-0.00	0.00
95 ->	55	-0.00	0.00
95 ->	56	-0.00	0.00
95 ->	57	-0.00	0.00
95 ->	58	0.01	0.00
95 ->	59	-0.00	0.00
95 ->	60	-0.00	0.00
95 ->	61	-0.00	0.00
95 ->	62	0.00	0.00
95 ->	63	0.00	0.00
95 ->	64	0.00	0.00
95 ->	65	0.00	0.00
95 ->	66	0.00	0.00
95 ->	67	0.00	0.00
95 ->	68	-0.00	0.00
95 ->	69	-0.00	0.00
95 ->	70	0.00	0.00
95 ->	71	0.00	0.00
95 ->	72	-0.01	0.00
95 ->	73	-0.00	0.00
95 ->	74	0.01	0.00
95 ->	75	0.00	0.00
95 ->	76	-0.01	0.00
95 ->	77	-0.01	0.00
95 ->	78	-0.00	0.00
95 ->	79	-0.00	0.00
95 ->	80	-0.00	0.00
95 ->	81	-0.00	0.00
95 ->	82	-0.00	0.00

95	->	83	0.00	0.00
95	->	84	0.00	0.00
95	->	85	0.00	0.00
95	->	86	0.00	0.00
95	->	87	0.00	0.00
95	->	88	0.00	0.00
95	->	89	0.00	0.00
95	->	90	0.01	0.00
95	->	91	0.00	0.00
95	->	92	-0.01	0.01
95	->	93	-0.24	0.07
95	->	94	-35.44	1.08
95	->	95	-95.89	2.74
95	->	96	-3.05	2.05
95	->	97	-5.52	0.67
95	->	98	-0.46	0.15
95	->	99	-0.17	0.09
95	->	100	-0.02	0.02
95	->	101	0.01	0.01
95	->	102	0.01	0.00
95	->	103	0.01	0.00
95	->	104	0.00	0.00
95	->	105	0.00	0.00
95	->	106	0.00	0.00
96	->	1	-0.01	0.01
96	->	2	-0.00	0.00
96	->	3	-0.02	0.01
96	->	4	-0.00	0.00
96	->	5	0.00	0.00
96	->	6	-0.00	0.00
96	->	7	-0.00	0.00
96	->	8	0.00	0.00
96	->	9	0.00	0.00
96	->	10	0.00	0.00
96	->	11	0.00	0.00
96	->	12	-0.00	0.00
96	->	13	-0.00	0.00
96	->	14	-0.01	0.00
96	->	15	-0.01	0.01
96	->	16	-0.30	0.73
96	->	17	-0.60	0.83
96	->	18	-0.77	1.03
96	->	19	-0.03	0.03
96	->	20	-0.05	0.08
96	->	21	-0.58	1.06
96	->	22	-0.03	0.03
96	->	23	-0.00	0.00
96	->	24	-0.01	0.01
96	->	25	-0.55	0.83
96	->	26	-0.00	0.00

96	->	27	-0.00	0.00
96	->	28	-0.00	0.00
96	->	29	-0.00	0.00
96	->	30	-0.00	0.00
96	->	31	-0.01	0.01
96	->	32	-0.01	0.01
96	->	33	-0.02	0.02
96	->	34	-0.00	0.00
96	->	35	0.00	0.00
96	->	36	-0.00	0.00
96	->	37	-0.00	0.00
96	->	38	0.00	0.00
96	->	39	0.00	0.00
96	->	40	-0.00	0.00
96	->	41	-0.00	0.00
96	->	42	-0.00	0.00
96	->	43	-0.01	0.01
96	->	44	-0.03	0.02
96	->	45	-0.01	0.01
96	->	46	0.01	0.01
96	->	47	-0.00	0.00
96	->	48	-0.00	0.00
96	->	49	-0.00	0.00
96	->	50	-0.00	0.00
96	->	51	-0.00	0.00
96	->	52	-0.00	0.00
96	->	53	-0.01	0.00
96	->	54	-0.00	0.00
96	->	55	-0.00	0.00
96	->	56	-0.00	0.00
96	->	57	-0.00	0.00
96	->	58	0.00	0.00
96	->	59	-0.00	0.00
96	->	60	-0.00	0.00
96	->	61	-0.00	0.00
96	->	62	0.00	0.00
96	->	63	0.00	0.00
96	->	64	0.00	0.00
96	->	65	0.00	0.00
96	->	66	0.00	0.00
96	->	67	0.00	0.00
96	->	68	-0.00	0.00
96	->	69	-0.00	0.00
96	->	70	0.00	0.00
96	->	71	0.00	0.00
96	->	72	-0.00	0.00
96	->	73	-0.00	0.00
96	->	74	0.00	0.00
96	->	75	0.00	0.00
96	->	76	-0.00	0.00

96	->	77	-0.00	0.00
96	->	78	-0.00	0.00
96	->	79	-0.00	0.00
96	->	80	-0.00	0.00
96	->	81	-0.00	0.00
96	->	82	-0.00	0.00
96	->	83	0.00	0.00
96	->	84	0.00	0.00
96	->	85	0.00	0.00
96	->	86	0.00	0.00
96	->	87	0.00	0.00
96	->	88	0.00	0.00
96	->	89	0.00	0.00
96	->	90	0.00	0.00
96	->	91	0.00	0.00
96	->	92	-0.00	0.01
96	->	93	-0.02	0.01
96	->	94	-0.17	0.08
96	->	95	-2.99	2.02
96	->	96	-50.02	4.00
96	->	97	-34.27	1.63
96	->	98	-0.24	0.10
96	->	99	-0.03	0.01
96	->	100	-0.00	0.00
96	->	101	0.00	0.00
96	->	102	0.00	0.00
96	->	103	0.00	0.00
96	->	104	0.00	0.00
96	->	105	0.00	0.00
96	->	106	0.00	0.00
97	->	1	-0.03	0.01
97	->	2	-0.00	0.00
97	->	3	-0.01	0.01
97	->	4	0.00	0.00
97	->	5	0.01	0.01
97	->	6	-0.00	0.00
97	->	7	-0.00	0.00
97	->	8	0.00	0.00
97	->	9	-0.00	0.00
97	->	10	0.01	0.00
97	->	11	0.01	0.00
97	->	12	-0.01	0.00
97	->	13	-0.01	0.00
97	->	14	-0.01	0.00
97	->	15	-0.01	0.02
97	->	16	-1.08	1.34
97	->	17	-0.11	0.12
97	->	18	-1.57	1.30
97	->	19	-0.07	0.07
97	->	20	-0.03	0.02

97	->	21	-0.26	0.35
97	->	22	-0.09	0.06
97	->	23	-0.00	0.00
97	->	24	-0.01	0.01
97	->	25	-3.33	4.68
97	->	26	-0.00	0.00
97	->	27	-0.00	0.00
97	->	28	-0.00	0.00
97	->	29	-0.00	0.00
97	->	30	-0.00	0.01
97	->	31	-0.05	0.04
97	->	32	-0.06	0.08
97	->	33	-0.67	0.86
97	->	34	-0.00	0.00
97	->	35	0.02	0.01
97	->	36	-0.03	0.01
97	->	37	-0.00	0.00
97	->	38	0.01	0.00
97	->	39	-0.00	0.00
97	->	40	-0.01	0.01
97	->	41	-0.01	0.01
97	->	42	-0.01	0.01
97	->	43	-0.01	0.02
97	->	44	-0.21	0.13
97	->	45	-0.49	0.47
97	->	46	0.05	0.04
97	->	47	-0.01	0.01
97	->	48	-0.03	0.02
97	->	49	-0.01	0.01
97	->	50	-0.00	0.00
97	->	51	-0.00	0.00
97	->	52	-0.01	0.00
97	->	53	-0.03	0.01
97	->	54	-0.00	0.00
97	->	55	-0.00	0.00
97	->	56	-0.00	0.00
97	->	57	-0.00	0.00
97	->	58	0.01	0.00
97	->	59	-0.01	0.00
97	->	60	-0.00	0.00
97	->	61	-0.00	0.00
97	->	62	0.00	0.00
97	->	63	0.00	0.00
97	->	64	0.00	0.00
97	->	65	0.00	0.00
97	->	66	0.00	0.00
97	->	67	0.00	0.00
97	->	68	-0.00	0.00
97	->	69	-0.01	0.00
97	->	70	0.00	0.00

97	->	71	0.00	0.00
97	->	72	-0.02	0.00
97	->	73	-0.00	0.00
97	->	74	0.01	0.00
97	->	75	0.01	0.00
97	->	76	-0.01	0.00
97	->	77	-0.01	0.00
97	->	78	-0.01	0.00
97	->	79	-0.00	0.00
97	->	80	-0.00	0.00
97	->	81	-0.00	0.00
97	->	82	-0.00	0.00
97	->	83	0.00	0.00
97	->	84	0.00	0.00
97	->	85	0.00	0.00
97	->	86	0.00	0.00
97	->	87	0.01	0.00
97	->	88	0.01	0.00
97	->	89	0.01	0.00
97	->	90	0.02	0.01
97	->	91	0.02	0.01
97	->	92	-0.01	0.01
97	->	93	-0.07	0.04
97	->	94	-2.46	0.92
97	->	95	-5.55	0.67
97	->	96	-34.06	1.60
97	->	97	-129.65	5.66
97	->	98	-34.88	1.14
97	->	99	-0.33	0.08
97	->	100	-0.01	0.01
97	->	101	0.00	0.00
97	->	102	0.01	0.00
97	->	103	0.01	0.00
97	->	104	0.00	0.00
97	->	105	0.00	0.00
97	->	106	0.00	0.00
98	->	1	-0.02	0.00
98	->	2	-0.00	0.00
98	->	3	-0.00	0.00
98	->	4	-0.00	0.00
98	->	5	0.01	0.00
98	->	6	-0.00	0.00
98	->	7	-0.00	0.00
98	->	8	0.00	0.00
98	->	9	0.00	0.00
98	->	10	0.00	0.00
98	->	11	0.00	0.00
98	->	12	-0.01	0.00
98	->	13	-0.00	0.00
98	->	14	-0.00	0.00

98	->	15	-0.00	0.00
98	->	16	-0.17	0.27
98	->	17	-0.01	0.01
98	->	18	-0.81	1.07
98	->	19	-0.13	0.14
98	->	20	-0.01	0.01
98	->	21	-0.02	0.01
98	->	22	-0.05	0.04
98	->	23	-0.00	0.00
98	->	24	-0.00	0.00
98	->	25	-0.08	0.04
98	->	26	-0.00	0.00
98	->	27	-0.00	0.00
98	->	28	-0.00	0.00
98	->	29	-0.00	0.00
98	->	30	0.00	0.00
98	->	31	-0.03	0.01
98	->	32	-0.02	0.01
98	->	33	-0.32	0.25
98	->	34	-0.00	0.01
98	->	35	0.03	0.01
98	->	36	-0.05	0.02
98	->	37	-0.00	0.00
98	->	38	0.02	0.00
98	->	39	-0.00	0.00
98	->	40	-0.02	0.01
98	->	41	-0.01	0.01
98	->	42	-0.02	0.01
98	->	43	-0.02	0.01
98	->	44	-0.44	0.27
98	->	45	-3.54	1.99
98	->	46	-0.59	0.65
98	->	47	-0.03	0.02
98	->	48	-0.15	0.11
98	->	49	-0.48	0.46
98	->	50	-0.00	0.01
98	->	51	-0.01	0.01
98	->	52	-0.03	0.02
98	->	53	-0.14	0.09
98	->	54	-0.00	0.00
98	->	55	-0.00	0.00
98	->	56	-0.00	0.00
98	->	57	-0.00	0.00
98	->	58	0.03	0.01
98	->	59	-0.02	0.00
98	->	60	-0.00	0.00
98	->	61	-0.00	0.00
98	->	62	0.00	0.00
98	->	63	0.01	0.00
98	->	64	0.00	0.00

98	->	65	0.00	0.00
98	->	66	0.00	0.00
98	->	67	0.00	0.00
98	->	68	-0.01	0.00
98	->	69	-0.01	0.00
98	->	70	-0.00	0.00
98	->	71	-0.00	0.00
98	->	72	-0.03	0.01
98	->	73	-0.00	0.00
98	->	74	0.02	0.00
98	->	75	0.01	0.00
98	->	76	-0.01	0.00
98	->	77	-0.01	0.00
98	->	78	-0.01	0.00
98	->	79	-0.00	0.00
98	->	80	-0.00	0.00
98	->	81	-0.00	0.00
98	->	82	-0.00	0.00
98	->	83	0.00	0.00
98	->	84	0.00	0.00
98	->	85	0.00	0.00
98	->	86	0.01	0.00
98	->	87	0.01	0.00
98	->	88	0.02	0.00
98	->	89	0.01	0.00
98	->	90	0.01	0.00
98	->	91	-0.01	0.01
98	->	92	-0.14	0.09
98	->	93	-0.47	0.33
98	->	94	-10.92	0.72
98	->	95	-0.46	0.15
98	->	96	-0.24	0.10
98	->	97	-34.67	1.11
98	->	98	-213.05	4.35
98	->	99	-34.04	0.83
98	->	100	-0.12	0.06
98	->	101	-0.01	0.01
98	->	102	0.00	0.00
98	->	103	0.00	0.00
98	->	104	0.00	0.00
98	->	105	0.00	0.00
98	->	106	0.00	0.00
99	->	1	-0.02	0.00
99	->	2	-0.00	0.00
99	->	3	-0.00	0.00
99	->	4	0.00	0.00
99	->	5	0.01	0.00
99	->	6	-0.00	0.00
99	->	7	-0.00	0.00
99	->	8	0.00	0.00

99	->	9	0.00	0.00
99	->	10	0.01	0.00
99	->	11	0.00	0.00
99	->	12	-0.01	0.00
99	->	13	-0.01	0.00
99	->	14	-0.00	0.00
99	->	15	-0.00	0.00
99	->	16	0.01	0.02
99	->	17	-0.00	0.00
99	->	18	-0.03	0.03
99	->	19	-0.00	0.02
99	->	20	-0.00	0.00
99	->	21	-0.01	0.01
99	->	22	-0.09	0.05
99	->	23	-0.01	0.00
99	->	24	-0.00	0.00
99	->	25	-0.05	0.02
99	->	26	-0.00	0.00
99	->	27	-0.00	0.00
99	->	28	-0.00	0.00
99	->	29	-0.00	0.00
99	->	30	0.00	0.00
99	->	31	-0.03	0.01
99	->	32	-0.00	0.00
99	->	33	-0.03	0.01
99	->	34	-0.00	0.00
99	->	35	0.03	0.01
99	->	36	-0.04	0.02
99	->	37	-0.00	0.00
99	->	38	0.02	0.00
99	->	39	-0.00	0.00
99	->	40	-0.02	0.00
99	->	41	-0.00	0.00
99	->	42	-0.01	0.00
99	->	43	-0.01	0.01
99	->	44	-1.72	1.93
99	->	45	-0.64	0.77
99	->	46	-1.49	0.85
99	->	47	-0.05	0.05
99	->	48	-0.16	0.13
99	->	49	-2.61	2.09
99	->	50	-0.01	0.05
99	->	51	-0.02	0.01
99	->	52	-0.07	0.07
99	->	53	-1.94	1.52
99	->	54	-0.00	0.00
99	->	55	-0.01	0.00
99	->	56	-0.01	0.00
99	->	57	-0.00	0.00
99	->	58	0.04	0.02

99	->	59	-0.05	0.03
99	->	60	-0.04	0.03
99	->	61	-0.02	0.03
99	->	62	0.00	0.00
99	->	63	0.01	0.00
99	->	64	0.01	0.00
99	->	65	0.00	0.00
99	->	66	0.01	0.00
99	->	67	0.00	0.00
99	->	68	-0.01	0.00
99	->	69	-0.03	0.01
99	->	70	-0.01	0.01
99	->	71	-0.00	0.02
99	->	72	-0.21	0.23
99	->	73	0.01	0.06
99	->	74	0.06	0.06
99	->	75	0.02	0.01
99	->	76	-0.03	0.01
99	->	77	-0.02	0.01
99	->	78	-0.02	0.00
99	->	79	-0.00	0.00
99	->	80	-0.00	0.00
99	->	81	-0.01	0.00
99	->	82	-0.00	0.00
99	->	83	0.00	0.00
99	->	84	0.00	0.00
99	->	85	0.00	0.00
99	->	86	0.01	0.00
99	->	87	0.01	0.00
99	->	88	0.02	0.01
99	->	89	0.01	0.00
99	->	90	0.01	0.01
99	->	91	-0.00	0.04
99	->	92	-0.29	0.54
99	->	93	-11.48	0.64
99	->	94	-3.99	1.23
99	->	95	-0.17	0.10
99	->	96	-0.03	0.01
99	->	97	-0.33	0.08
99	->	98	-33.80	0.82
99	->	99	-173.63	4.12
99	->	100	-36.28	0.99
99	->	101	-0.39	0.11
99	->	102	-0.04	0.01
99	->	103	0.00	0.00
99	->	104	0.01	0.00
99	->	105	0.00	0.00
99	->	106	0.00	0.00
100	->	1	-0.03	0.01
100	->	2	-0.00	0.00

100	->	3	-0.00	0.00
100	->	4	0.00	0.00
100	->	5	0.01	0.00
100	->	6	-0.00	0.00
100	->	7	-0.00	0.00
100	->	8	0.00	0.00
100	->	9	0.00	0.00
100	->	10	0.01	0.00
100	->	11	0.01	0.00
100	->	12	-0.02	0.00
100	->	13	-0.01	0.00
100	->	14	-0.00	0.00
100	->	15	-0.00	0.00
100	->	16	0.02	0.00
100	->	17	-0.00	0.00
100	->	18	-0.01	0.00
100	->	19	-0.01	0.01
100	->	20	-0.00	0.00
100	->	21	-0.00	0.00
100	->	22	-0.01	0.02
100	->	23	-0.00	0.00
100	->	24	-0.00	0.00
100	->	25	-0.01	0.00
100	->	26	-0.00	0.00
100	->	27	-0.00	0.00
100	->	28	-0.00	0.00
100	->	29	-0.00	0.00
100	->	30	0.00	0.00
100	->	31	-0.01	0.00
100	->	32	-0.00	0.00
100	->	33	-0.01	0.00
100	->	34	-0.00	0.00
100	->	35	0.01	0.00
100	->	36	-0.02	0.01
100	->	37	-0.00	0.00
100	->	38	0.01	0.00
100	->	39	-0.00	0.00
100	->	40	-0.01	0.00
100	->	41	-0.00	0.00
100	->	42	-0.00	0.00
100	->	43	0.00	0.00
100	->	44	0.60	0.53
100	->	45	-0.02	0.02
100	->	46	-4.62	1.17
100	->	47	-0.14	0.04
100	->	48	-0.02	0.01
100	->	49	-0.21	0.16
100	->	50	-0.02	0.04
100	->	51	-0.00	0.00
100	->	52	-0.02	0.01

100	->	53	-6.97	3.77
100	->	54	-0.00	0.00
100	->	55	-0.00	0.00
100	->	56	-0.00	0.00
100	->	57	-0.00	0.00
100	->	58	0.04	0.02
100	->	59	-0.08	0.04
100	->	60	-0.14	0.11
100	->	61	-1.07	0.74
100	->	62	-0.00	0.00
100	->	63	0.02	0.00
100	->	64	0.02	0.01
100	->	65	0.00	0.00
100	->	66	0.02	0.00
100	->	67	0.00	0.00
100	->	68	-0.02	0.00
100	->	69	-0.05	0.01
100	->	70	-0.03	0.02
100	->	71	0.02	0.06
100	->	72	-0.56	0.52
100	->	73	-1.02	0.52
100	->	74	0.06	0.06
100	->	75	0.02	0.01
100	->	76	-0.12	0.09
100	->	77	-0.10	0.13
100	->	78	-0.03	0.01
100	->	79	-0.01	0.00
100	->	80	-0.00	0.00
100	->	81	-0.02	0.01
100	->	82	-0.00	0.00
100	->	83	-0.00	0.00
100	->	84	0.01	0.00
100	->	85	0.00	0.00
100	->	86	0.01	0.01
100	->	87	0.01	0.00
100	->	88	0.01	0.00
100	->	89	-0.01	0.01
100	->	90	-0.10	0.08
100	->	91	-0.90	0.43
100	->	92	-11.65	0.67
100	->	93	0.26	0.36
100	->	94	-0.28	0.11
100	->	95	-0.02	0.02
100	->	96	-0.00	0.00
100	->	97	-0.01	0.01
100	->	98	-0.12	0.06
100	->	99	-36.13	0.98
100	->	100	-207.01	5.61
100	->	101	-31.91	0.73
100	->	102	-0.12	0.04

100	->	103	-0.01	0.01
100	->	104	0.01	0.00
100	->	105	0.01	0.00
100	->	106	0.00	0.00
101	->	1	-0.04	0.01
101	->	2	-0.00	0.00
101	->	3	-0.00	0.00
101	->	4	-0.00	0.00
101	->	5	0.01	0.00
101	->	6	-0.00	0.00
101	->	7	-0.00	0.00
101	->	8	0.00	0.00
101	->	9	0.00	0.00
101	->	10	0.01	0.00
101	->	11	0.01	0.00
101	->	12	-0.03	0.01
101	->	13	-0.01	0.00
101	->	14	-0.00	0.00
101	->	15	-0.00	0.00
101	->	16	0.02	0.00
101	->	17	-0.00	0.00
101	->	18	-0.00	0.00
101	->	19	-0.02	0.01
101	->	20	-0.00	0.00
101	->	21	-0.00	0.00
101	->	22	-0.05	0.02
101	->	23	-0.00	0.00
101	->	24	-0.00	0.00
101	->	25	-0.01	0.00
101	->	26	-0.00	0.00
101	->	27	-0.00	0.00
101	->	28	-0.00	0.00
101	->	29	-0.00	0.00
101	->	30	0.00	0.00
101	->	31	-0.01	0.00
101	->	32	-0.00	0.00
101	->	33	-0.00	0.00
101	->	34	-0.00	0.00
101	->	35	0.01	0.00
101	->	36	-0.01	0.00
101	->	37	0.00	0.00
101	->	38	0.01	0.00
101	->	39	-0.00	0.00
101	->	40	-0.01	0.00
101	->	41	-0.00	0.00
101	->	42	-0.00	0.00
101	->	43	-0.00	0.00
101	->	44	0.27	0.51
101	->	45	-0.01	0.00
101	->	46	-0.71	0.52

101 ->	47	-0.08	0.05
101 ->	48	-0.01	0.00
101 ->	49	-0.01	0.01
101 ->	50	-0.03	0.01
101 ->	51	-0.00	0.00
101 ->	52	-0.00	0.00
101 ->	53	-0.09	0.03
101 ->	54	-0.00	0.00
101 ->	55	-0.00	0.00
101 ->	56	-0.00	0.00
101 ->	57	-0.00	0.00
101 ->	58	0.02	0.01
101 ->	59	-0.03	0.01
101 ->	60	-0.02	0.01
101 ->	61	-0.38	0.27
101 ->	62	0.00	0.01
101 ->	63	0.03	0.01
101 ->	64	0.03	0.01
101 ->	65	-0.00	0.00
101 ->	66	0.02	0.00
101 ->	67	0.00	0.00
101 ->	68	-0.02	0.00
101 ->	69	-0.04	0.01
101 ->	70	-0.02	0.01
101 ->	71	-0.03	0.02
101 ->	72	-0.55	0.26
101 ->	73	-4.76	1.68
101 ->	74	-0.29	0.31
101 ->	75	-0.00	0.02
101 ->	76	-0.52	0.96
101 ->	77	-1.15	1.17
101 ->	78	-0.05	0.01
101 ->	79	-0.01	0.00
101 ->	80	-0.01	0.00
101 ->	81	-0.04	0.02
101 ->	82	-0.00	0.00
101 ->	83	-0.00	0.00
101 ->	84	0.01	0.00
101 ->	85	0.00	0.00
101 ->	86	0.01	0.00
101 ->	87	-0.00	0.00
101 ->	88	-0.01	0.01
101 ->	89	-0.07	0.03
101 ->	90	-1.02	0.62
101 ->	91	-11.56	0.85
101 ->	92	-3.88	0.75
101 ->	93	-0.07	0.10
101 ->	94	-0.06	0.04
101 ->	95	0.01	0.01
101 ->	96	0.00	0.00

101	->	97	0.00	0.00
101	->	98	-0.01	0.01
101	->	99	-0.39	0.11
101	->	100	-31.69	0.72
101	->	101	-210.37	3.48
101	->	102	-31.54	1.06
101	->	103	-0.20	0.05
101	->	104	0.00	0.01
101	->	105	0.01	0.00
101	->	106	0.00	0.00
102	->	1	-0.05	0.02
102	->	2	-0.00	0.00
102	->	3	-0.00	0.00
102	->	4	-0.00	0.00
102	->	5	0.01	0.00
102	->	6	-0.00	0.00
102	->	7	-0.00	0.00
102	->	8	0.00	0.00
102	->	9	0.00	0.00
102	->	10	0.01	0.00
102	->	11	0.01	0.00
102	->	12	-0.06	0.03
102	->	13	-0.01	0.00
102	->	14	-0.00	0.00
102	->	15	-0.00	0.00
102	->	16	0.02	0.00
102	->	17	-0.00	0.00
102	->	18	-0.00	0.00
102	->	19	-0.01	0.01
102	->	20	-0.00	0.00
102	->	21	-0.00	0.00
102	->	22	-0.07	0.02
102	->	23	-0.01	0.00
102	->	24	-0.00	0.00
102	->	25	-0.01	0.00
102	->	26	-0.00	0.00
102	->	27	-0.00	0.00
102	->	28	-0.00	0.00
102	->	29	-0.00	0.00
102	->	30	0.00	0.00
102	->	31	-0.01	0.00
102	->	32	-0.00	0.00
102	->	33	-0.00	0.00
102	->	34	-0.00	0.00
102	->	35	0.01	0.00
102	->	36	-0.00	0.00
102	->	37	0.00	0.00
102	->	38	0.00	0.00
102	->	39	-0.00	0.00
102	->	40	-0.01	0.00

102	->	41	-0.00	0.00
102	->	42	-0.00	0.00
102	->	43	-0.00	0.00
102	->	44	-0.12	0.10
102	->	45	-0.00	0.00
102	->	46	-0.05	0.10
102	->	47	-0.03	0.02
102	->	48	-0.00	0.00
102	->	49	-0.00	0.00
102	->	50	-0.02	0.01
102	->	51	-0.00	0.00
102	->	52	-0.00	0.00
102	->	53	-0.03	0.00
102	->	54	-0.00	0.00
102	->	55	-0.00	0.00
102	->	56	-0.00	0.00
102	->	57	-0.00	0.00
102	->	58	0.01	0.00
102	->	59	-0.02	0.00
102	->	60	-0.00	0.00
102	->	61	-0.02	0.00
102	->	62	0.00	0.00
102	->	63	0.03	0.01
102	->	64	0.02	0.01
102	->	65	-0.00	0.00
102	->	66	0.02	0.00
102	->	67	-0.00	0.00
102	->	68	-0.02	0.00
102	->	69	-0.02	0.00
102	->	70	-0.01	0.00
102	->	71	-0.01	0.00
102	->	72	-0.26	0.38
102	->	73	-0.49	0.87
102	->	74	-1.88	1.07
102	->	75	-0.01	0.02
102	->	76	-0.22	0.12
102	->	77	-6.58	3.07
102	->	78	-0.09	0.02
102	->	79	-0.01	0.01
102	->	80	-0.02	0.01
102	->	81	-0.20	0.30
102	->	82	-0.00	0.00
102	->	83	-0.00	0.00
102	->	84	0.02	0.00
102	->	85	0.00	0.00
102	->	86	0.01	0.00
102	->	87	-0.02	0.01
102	->	88	-0.11	0.04
102	->	89	-0.68	0.28
102	->	90	-11.39	0.78

102	->	91	-4.16	0.88
102	->	92	-0.40	0.16
102	->	93	-0.02	0.04
102	->	94	0.01	0.00
102	->	95	0.01	0.00
102	->	96	0.00	0.00
102	->	97	0.01	0.00
102	->	98	0.00	0.00
102	->	99	-0.04	0.01
102	->	100	-0.12	0.04
102	->	101	-31.34	1.05
102	->	102	-209.66	2.84
102	->	103	-33.34	0.78
102	->	104	-0.12	0.04
102	->	105	0.00	0.01
102	->	106	0.00	0.00
103	->	1	-0.04	0.02
103	->	2	-0.00	0.00
103	->	3	0.00	0.00
103	->	4	-0.00	0.00
103	->	5	0.01	0.00
103	->	6	-0.00	0.00
103	->	7	0.00	0.00
103	->	8	0.00	0.00
103	->	9	0.00	0.00
103	->	10	0.01	0.00
103	->	11	0.02	0.01
103	->	12	-0.07	0.05
103	->	13	-0.01	0.00
103	->	14	-0.00	0.00
103	->	15	-0.00	0.00
103	->	16	0.01	0.00
103	->	17	0.00	0.00
103	->	18	-0.00	0.00
103	->	19	-0.00	0.00
103	->	20	-0.00	0.00
103	->	21	-0.00	0.00
103	->	22	-0.04	0.01
103	->	23	-0.01	0.00
103	->	24	-0.00	0.00
103	->	25	-0.01	0.00
103	->	26	-0.00	0.00
103	->	27	-0.00	0.00
103	->	28	-0.00	0.00
103	->	29	-0.00	0.00
103	->	30	0.00	0.00
103	->	31	-0.02	0.00
103	->	32	-0.00	0.00
103	->	33	-0.00	0.00
103	->	34	-0.00	0.00

103	->	35	0.01	0.00
103	->	36	-0.00	0.00
103	->	37	0.00	0.00
103	->	38	0.01	0.00
103	->	39	0.00	0.00
103	->	40	-0.01	0.00
103	->	41	-0.00	0.00
103	->	42	-0.00	0.00
103	->	43	-0.00	0.00
103	->	44	-0.07	0.04
103	->	45	-0.00	0.00
103	->	46	0.01	0.01
103	->	47	-0.04	0.01
103	->	48	-0.00	0.00
103	->	49	-0.00	0.00
103	->	50	-0.02	0.01
103	->	51	-0.01	0.00
103	->	52	-0.00	0.00
103	->	53	-0.02	0.00
103	->	54	-0.00	0.00
103	->	55	-0.00	0.00
103	->	56	-0.00	0.00
103	->	57	-0.00	0.00
103	->	58	0.01	0.00
103	->	59	-0.01	0.00
103	->	60	-0.00	0.00
103	->	61	-0.01	0.00
103	->	62	0.00	0.00
103	->	63	0.02	0.01
103	->	64	0.01	0.00
103	->	65	0.00	0.00
103	->	66	0.01	0.00
103	->	67	-0.00	0.00
103	->	68	-0.02	0.00
103	->	69	-0.01	0.00
103	->	70	-0.00	0.00
103	->	71	0.00	0.00
103	->	72	0.46	0.55
103	->	73	-0.05	0.03
103	->	74	-3.73	1.26
103	->	75	-0.02	0.03
103	->	76	-0.07	0.04
103	->	77	-2.46	2.59
103	->	78	-0.23	0.18
103	->	79	-0.01	0.00
103	->	80	-0.02	0.02
103	->	81	-1.08	2.20
103	->	82	-0.01	0.00
103	->	83	-0.00	0.00
103	->	84	0.02	0.01

103	->	85	-0.00	0.00
103	->	86	-0.01	0.01
103	->	87	-0.09	0.04
103	->	88	-2.10	0.71
103	->	89	-6.02	0.70
103	->	90	-3.56	0.59
103	->	91	-0.36	0.09
103	->	92	-0.04	0.03
103	->	93	0.01	0.00
103	->	94	0.02	0.00
103	->	95	0.01	0.00
103	->	96	0.00	0.00
103	->	97	0.01	0.00
103	->	98	0.00	0.00
103	->	99	0.00	0.00
103	->	100	-0.01	0.01
103	->	101	-0.20	0.05
103	->	102	-33.11	0.76
103	->	103	-130.78	3.68
103	->	104	-34.60	0.90
103	->	105	-0.31	0.08
103	->	106	-0.02	0.01
104	->	1	-0.03	0.01
104	->	2	-0.00	0.00
104	->	3	0.00	0.00
104	->	4	-0.00	0.00
104	->	5	0.01	0.00
104	->	6	-0.00	0.00
104	->	7	0.00	0.00
104	->	8	0.01	0.00
104	->	9	0.00	0.00
104	->	10	0.02	0.00
104	->	11	0.02	0.01
104	->	12	-0.06	0.03
104	->	13	-0.01	0.00
104	->	14	-0.00	0.00
104	->	15	-0.00	0.00
104	->	16	0.01	0.00
104	->	17	0.00	0.00
104	->	18	0.00	0.00
104	->	19	-0.00	0.00
104	->	20	0.00	0.00
104	->	21	-0.00	0.00
104	->	22	-0.03	0.01
104	->	23	-0.00	0.00
104	->	24	0.00	0.00
104	->	25	-0.01	0.00
104	->	26	-0.00	0.00
104	->	27	-0.00	0.00
104	->	28	-0.00	0.00

104	->	29	-0.00	0.00
104	->	30	0.00	0.00
104	->	31	-0.02	0.01
104	->	32	-0.00	0.00
104	->	33	-0.00	0.00
104	->	34	-0.00	0.00
104	->	35	0.01	0.00
104	->	36	-0.00	0.00
104	->	37	0.00	0.00
104	->	38	0.01	0.00
104	->	39	0.00	0.00
104	->	40	-0.02	0.00
104	->	41	-0.00	0.00
104	->	42	-0.00	0.00
104	->	43	-0.00	0.00
104	->	44	-0.04	0.01
104	->	45	-0.00	0.00
104	->	46	0.02	0.00
104	->	47	-0.01	0.00
104	->	48	-0.00	0.00
104	->	49	-0.00	0.00
104	->	50	-0.01	0.00
104	->	51	-0.00	0.00
104	->	52	-0.00	0.00
104	->	53	-0.01	0.00
104	->	54	-0.00	0.00
104	->	55	-0.00	0.00
104	->	56	-0.00	0.00
104	->	57	-0.00	0.00
104	->	58	0.00	0.00
104	->	59	-0.01	0.00
104	->	60	-0.00	0.00
104	->	61	-0.00	0.00
104	->	62	0.00	0.00
104	->	63	0.01	0.00
104	->	64	0.01	0.00
104	->	65	-0.00	0.00
104	->	66	0.01	0.00
104	->	67	-0.00	0.00
104	->	68	-0.01	0.00
104	->	69	-0.01	0.00
104	->	70	-0.01	0.00
104	->	71	-0.00	0.00
104	->	72	0.18	0.27
104	->	73	-0.01	0.00
104	->	74	-0.69	0.26
104	->	75	-0.05	0.04
104	->	76	-0.02	0.01
104	->	77	-0.07	0.02
104	->	78	-1.39	1.12

104	->	79	-0.01	0.00
104	->	80	-0.01	0.00
104	->	81	-0.34	0.79
104	->	82	-0.01	0.00
104	->	83	-0.00	0.00
104	->	84	0.01	0.01
104	->	85	-0.03	0.01
104	->	86	-0.12	0.06
104	->	87	-0.48	0.25
104	->	88	-11.36	0.77
104	->	89	-0.68	0.34
104	->	90	-0.31	0.08
104	->	91	-0.03	0.01
104	->	92	0.02	0.00
104	->	93	0.01	0.00
104	->	94	0.01	0.00
104	->	95	0.00	0.00
104	->	96	0.00	0.00
104	->	97	0.00	0.00
104	->	98	0.00	0.00
104	->	99	0.01	0.00
104	->	100	0.01	0.00
104	->	101	0.00	0.01
104	->	102	-0.12	0.04
104	->	103	-34.37	0.90
104	->	104	-217.48	3.13
104	->	105	-33.38	0.92
104	->	106	-0.18	0.05
105	->	1	-0.02	0.01
105	->	2	-0.00	0.00
105	->	3	0.00	0.00
105	->	4	-0.00	0.00
105	->	5	0.01	0.00
105	->	6	-0.00	0.00
105	->	7	-0.00	0.00
105	->	8	0.01	0.00
105	->	9	0.00	0.00
105	->	10	0.02	0.01
105	->	11	0.02	0.01
105	->	12	-0.04	0.01
105	->	13	-0.01	0.00
105	->	14	-0.00	0.00
105	->	15	-0.00	0.00
105	->	16	0.01	0.00
105	->	17	0.00	0.00
105	->	18	0.00	0.00
105	->	19	-0.00	0.00
105	->	20	0.00	0.00
105	->	21	0.00	0.00
105	->	22	-0.02	0.01

105	->	23	-0.00	0.00
105	->	24	0.00	0.00
105	->	25	-0.01	0.00
105	->	26	-0.00	0.00
105	->	27	-0.00	0.00
105	->	28	-0.00	0.00
105	->	29	-0.00	0.00
105	->	30	-0.00	0.00
105	->	31	-0.02	0.01
105	->	32	-0.00	0.00
105	->	33	-0.00	0.00
105	->	34	-0.00	0.00
105	->	35	0.00	0.00
105	->	36	-0.00	0.00
105	->	37	-0.00	0.00
105	->	38	0.00	0.00
105	->	39	-0.00	0.00
105	->	40	-0.01	0.01
105	->	41	-0.00	0.00
105	->	42	-0.01	0.00
105	->	43	-0.01	0.00
105	->	44	-0.02	0.00
105	->	45	-0.00	0.00
105	->	46	0.02	0.00
105	->	47	-0.01	0.01
105	->	48	-0.00	0.00
105	->	49	-0.00	0.00
105	->	50	-0.00	0.00
105	->	51	-0.01	0.00
105	->	52	-0.00	0.00
105	->	53	-0.01	0.00
105	->	54	-0.01	0.00
105	->	55	-0.00	0.00
105	->	56	-0.00	0.00
105	->	57	-0.00	0.00
105	->	58	0.00	0.00
105	->	59	-0.01	0.00
105	->	60	-0.00	0.00
105	->	61	-0.00	0.00
105	->	62	0.00	0.00
105	->	63	0.01	0.00
105	->	64	0.01	0.00
105	->	65	0.00	0.00
105	->	66	0.01	0.00
105	->	67	-0.00	0.00
105	->	68	-0.02	0.00
105	->	69	-0.01	0.00
105	->	70	-0.00	0.00
105	->	71	-0.00	0.00
105	->	72	0.15	0.17

105	->	73	-0.00	0.00
105	->	74	0.01	0.04
105	->	75	0.00	0.01
105	->	76	-0.02	0.00
105	->	77	-0.05	0.02
105	->	78	-3.76	2.69
105	->	79	-0.01	0.00
105	->	80	-0.00	0.00
105	->	81	-0.07	0.04
105	->	82	-0.01	0.00
105	->	83	-0.00	0.00
105	->	84	0.01	0.00
105	->	85	-0.22	0.07
105	->	86	-1.26	0.74
105	->	87	-11.23	0.83
105	->	88	-5.63	1.27
105	->	89	-0.27	0.13
105	->	90	-0.04	0.03
105	->	91	0.01	0.00
105	->	92	0.01	0.00
105	->	93	0.01	0.00
105	->	94	0.01	0.00
105	->	95	0.00	0.00
105	->	96	0.00	0.00
105	->	97	0.00	0.00
105	->	98	0.00	0.00
105	->	99	0.00	0.00
105	->	100	0.01	0.00
105	->	101	0.01	0.00
105	->	102	0.00	0.01
105	->	103	-0.31	0.08
105	->	104	-33.08	0.91
105	->	105	-180.72	3.19
105	->	106	-35.94	1.11
106	->	1	-0.01	0.00
106	->	2	-0.00	0.00
106	->	3	0.00	0.00
106	->	4	-0.00	0.00
106	->	5	0.01	0.00
106	->	6	-0.00	0.00
106	->	7	0.00	0.00
106	->	8	0.00	0.00
106	->	9	0.00	0.00
106	->	10	0.01	0.00
106	->	11	0.01	0.00
106	->	12	-0.01	0.00
106	->	13	-0.00	0.00
106	->	14	-0.00	0.00
106	->	15	-0.00	0.00
106	->	16	0.00	0.00

106	->	17	0.00	0.00
106	->	18	0.00	0.00
106	->	19	0.00	0.00
106	->	20	0.00	0.00
106	->	21	0.00	0.00
106	->	22	-0.01	0.00
106	->	23	-0.00	0.00
106	->	24	0.00	0.00
106	->	25	-0.01	0.00
106	->	26	-0.00	0.00
106	->	27	-0.00	0.00
106	->	28	-0.00	0.00
106	->	29	-0.00	0.00
106	->	30	0.00	0.00
106	->	31	-0.02	0.01
106	->	32	-0.00	0.00
106	->	33	0.00	0.00
106	->	34	-0.00	0.00
106	->	35	0.01	0.00
106	->	36	-0.00	0.00
106	->	37	0.00	0.00
106	->	38	0.01	0.00
106	->	39	-0.00	0.00
106	->	40	-0.03	0.01
106	->	41	-0.00	0.00
106	->	42	-0.00	0.00
106	->	43	-0.00	0.00
106	->	44	-0.01	0.00
106	->	45	0.00	0.00
106	->	46	0.01	0.00
106	->	47	-0.00	0.00
106	->	48	-0.00	0.00
106	->	49	-0.00	0.00
106	->	50	-0.00	0.00
106	->	51	-0.00	0.00
106	->	52	-0.00	0.00
106	->	53	-0.01	0.00
106	->	54	-0.00	0.00
106	->	55	-0.00	0.00
106	->	56	-0.00	0.00
106	->	57	-0.00	0.00
106	->	58	0.00	0.00
106	->	59	-0.01	0.00
106	->	60	-0.00	0.00
106	->	61	-0.00	0.00
106	->	62	0.00	0.00
106	->	63	0.00	0.00
106	->	64	0.00	0.00
106	->	65	0.00	0.00
106	->	66	0.00	0.00

106	->	67	-0.00	0.00
106	->	68	-0.02	0.01
106	->	69	-0.00	0.00
106	->	70	-0.00	0.00
106	->	71	-0.00	0.00
106	->	72	-0.05	0.02
106	->	73	-0.00	0.00
106	->	74	0.01	0.00
106	->	75	0.01	0.00
106	->	76	-0.01	0.00
106	->	77	-0.01	0.00
106	->	78	-0.23	0.58
106	->	79	-0.01	0.00
106	->	80	-0.00	0.00
106	->	81	-0.02	0.02
106	->	82	-0.01	0.01
106	->	83	-0.00	0.00
106	->	84	0.00	0.00
106	->	85	-2.85	1.57
106	->	86	-10.66	0.75
106	->	87	0.15	0.13
106	->	88	-0.25	0.07
106	->	89	-0.03	0.03
106	->	90	0.01	0.00
106	->	91	0.01	0.00
106	->	92	0.01	0.00
106	->	93	0.00	0.00
106	->	94	0.00	0.00
106	->	95	0.00	0.00
106	->	96	0.00	0.00
106	->	97	0.00	0.00
106	->	98	0.00	0.00
106	->	99	0.00	0.00
106	->	100	0.00	0.00
106	->	101	0.00	0.00
106	->	102	0.00	0.00
106	->	103	-0.02	0.01
106	->	104	-0.18	0.05
106	->	105	-35.69	1.12
106	->	106	-215.14	3.17
