# Supplementary material for: Investigating dynamic and energetic determinants of protein nucleic acid recognition: analysis of the zinc finger zif268-DNA complexes
Source: BMC Struct Biol. 2010 Nov 24;10:42. doi: 10.1186/1472-6807-10-42 (PMC3002361; doi:10.1186/1472-6807-10-42)
Supplement: Additional file 4 — Average pair interaction energies for 1A1I Complex and their standard deviations. [file 1472-6807-10-42-S4.PDF]

=>> 1A1I

Residue ->	Residue	TGBTOT	SD
1 ->	1	-251.19	8.67
1 ->	2	-0.92	0.36
1 ->	3	-1.16	0.33
1 ->	4	-0.12	0.03
1 ->	5	-0.17	0.03
1 ->	6	-0.00	0.00
1 ->	7	0.00	0.00
1 ->	8	-0.05	0.02
1 ->	9	-0.00	0.00
1 ->	10	-0.10	0.01
1 ->	11	-0.13	0.02
1 ->	12	0.50	0.87
1 ->	13	-3.81	1.98
1 ->	14	-2.99	1.08
1 ->	15	-4.62	1.44
1 ->	16	0.68	1.13
1 ->	17	-0.04	0.02
1 ->	18	0.06	0.09
1 ->	19	-3.02	4.53
1 ->	20	-0.07	0.06
1 ->	21	0.01	0.01
1 ->	22	0.67	0.78
1 ->	23	0.00	0.03
1 ->	24	-0.00	0.01
1 ->	25	0.04	0.01
1 ->	26	0.00	0.00
1 ->	27	-0.01	0.00
1 ->	28	0.00	0.00
1 ->	29	0.00	0.00
1 ->	30	-0.00	0.00
1 ->	31	0.02	0.00
1 ->	32	0.00	0.00
1 ->	33	-0.00	0.00
1 ->	34	0.00	0.00
1 ->	35	-0.01	0.00
1 ->	36	0.00	0.00
1 ->	37	0.00	0.00
1 ->	38	-0.00	0.00
1 ->	39	-0.00	0.00
1 ->	40	0.01	0.00
1 ->	41	0.00	0.00
1 ->	42	0.00	0.00
1 ->	43	0.00	0.00
1 ->	44	0.05	0.02
1 ->	45	0.00	0.00
1 ->	46	-0.03	0.01

1 ->	47	0.00	0.00
1 ->	48	0.00	0.00
1 ->	49	0.00	0.00
1 ->	50	0.00	0.00
1 ->	51	0.00	0.00
1 ->	52	0.00	0.00
1 ->	53	0.01	0.00
1 ->	54	0.00	0.00
1 ->	55	0.00	0.00
1 ->	56	0.00	0.00
1 ->	57	0.00	0.00
1 ->	58	-0.00	0.00
1 ->	59	0.00	0.00
1 ->	60	0.00	0.00
1 ->	61	0.00	0.00
1 ->	62	-0.00	0.00
1 ->	63	-0.00	0.00
1 ->	64	-0.00	0.00
1 ->	65	-0.00	0.00
1 ->	66	-0.00	0.00
1 ->	67	0.00	0.00
1 ->	68	0.00	0.00
1 ->	69	0.00	0.00
1 ->	70	0.00	0.00
1 ->	71	-0.00	0.00
1 ->	72	0.02	0.00
1 ->	73	0.00	0.00
1 ->	74	-0.02	0.01
1 ->	75	-0.01	0.00
1 ->	76	0.01	0.00
1 ->	77	0.02	0.01
1 ->	78	0.01	0.01
1 ->	79	0.00	0.00
1 ->	80	0.00	0.00
1 ->	81	0.02	0.01
1 ->	82	0.00	0.00
1 ->	83	0.00	0.00
1 ->	84	0.00	0.00
1 ->	85	0.00	0.00
1 ->	86	-0.00	0.00
1 ->	87	-0.01	0.00
1 ->	88	-0.01	0.00
1 ->	89	-0.01	0.00
1 ->	90	-0.02	0.01
1 ->	91	-0.08	0.05
1 ->	92	-1.21	1.25
1 ->	93	-7.20	7.64
1 ->	94	-0.79	1.02
1 ->	95	-0.07	0.01
1 ->	96	-0.02	0.00

1 ->	97	-0.01	0.01
1 ->	98	-0.04	0.01
1 ->	99	-0.03	0.01
1 ->	100	-0.01	0.01
1 ->	101	-0.02	0.01
1 ->	102	-0.03	0.01
1 ->	103	-0.04	0.02
1 ->	104	-0.03	0.01
1 ->	105	-0.03	0.01
1 ->	106	-0.02	0.01
1 ->	107	-0.01	0.00
2 ->	1	-0.92	0.36
2 ->	2	1.75	0.71
2 ->	3	-12.37	0.74
2 ->	4	-0.37	0.15
2 ->	5	-0.02	0.01
2 ->	6	-0.01	0.00
2 ->	7	-0.00	0.00
2 ->	8	0.00	0.00
2 ->	9	-0.00	0.00
2 ->	10	-0.00	0.00
2 ->	11	-0.01	0.00
2 ->	12	-0.02	0.01
2 ->	13	-1.91	0.95
2 ->	14	-1.00	0.18
2 ->	15	-0.96	0.43
2 ->	16	-0.09	0.05
2 ->	17	-0.02	0.02
2 ->	18	-0.00	0.00
2 ->	19	-0.02	0.01
2 ->	20	-0.03	0.01
2 ->	21	-0.00	0.00
2 ->	22	-0.00	0.00
2 ->	23	-0.00	0.00
2 ->	24	-0.00	0.00
2 ->	25	-0.00	0.00
2 ->	26	-0.00	0.00
2 ->	27	-0.00	0.00
2 ->	28	-0.00	0.00
2 ->	29	0.00	0.00
2 ->	30	-0.00	0.00
2 ->	31	-0.00	0.00
2 ->	32	-0.00	0.00
2 ->	33	0.00	0.00
2 ->	34	-0.00	0.00
2 ->	35	-0.00	0.00
2 ->	36	0.00	0.00
2 ->	37	0.00	0.00
2 ->	38	0.00	0.00
2 ->	39	0.00	0.00

2 ->	40	-0.00	0.00
2 ->	41	-0.00	0.00
2 ->	42	0.00	0.00
2 ->	43	0.00	0.00
2 ->	44	0.00	0.00
2 ->	45	0.00	0.00
2 ->	46	-0.00	0.00
2 ->	47	0.00	0.00
2 ->	48	0.00	0.00
2 ->	49	0.00	0.00
2 ->	50	0.00	0.00
2 ->	51	0.00	0.00
2 ->	52	0.00	0.00
2 ->	53	0.00	0.00
2 ->	54	0.00	0.00
2 ->	55	0.00	0.00
2 ->	56	0.00	0.00
2 ->	57	0.00	0.00
2 ->	58	-0.00	0.00
2 ->	59	0.00	0.00
2 ->	60	0.00	0.00
2 ->	61	0.00	0.00
2 ->	62	0.00	0.00
2 ->	63	-0.00	0.00
2 ->	64	-0.00	0.00
2 ->	65	0.00	0.00
2 ->	66	-0.00	0.00
2 ->	67	0.00	0.00
2 ->	68	0.00	0.00
2 ->	69	0.00	0.00
2 ->	70	0.00	0.00
2 ->	71	-0.00	0.00
2 ->	72	0.00	0.00
2 ->	73	0.00	0.00
2 ->	74	-0.00	0.00
2 ->	75	-0.00	0.00
2 ->	76	0.00	0.00
2 ->	77	0.00	0.00
2 ->	78	0.00	0.00
2 ->	79	0.00	0.00
2 ->	80	0.00	0.00
2 ->	81	0.00	0.00
2 ->	82	0.00	0.00
2 ->	83	0.00	0.00
2 ->	84	0.00	0.00
2 ->	85	-0.00	0.00
2 ->	86	-0.00	0.00
2 ->	87	-0.00	0.00
2 ->	88	-0.00	0.00
2 ->	89	-0.00	0.00

2	->	90	0.00	0.00
2	->	91	-0.00	0.00
2	->	92	-0.00	0.00
2	->	93	-0.01	0.00
2	->	94	-0.01	0.00
2	->	95	-0.00	0.00
2	->	96	-0.00	0.00
2	->	97	-0.00	0.00
2	->	98	-0.00	0.00
2	->	99	-0.00	0.00
2	->	100	-0.00	0.00
2	->	101	-0.00	0.00
2	->	102	-0.00	0.00
2	->	103	-0.00	0.00
2	->	104	-0.00	0.00
2	->	105	-0.00	0.00
2	->	106	-0.00	0.00
2	->	107	-0.00	0.00
3	->	1	-1.16	0.33
3	->	2	-12.46	0.75
3	->	3	18.74	1.63
3	->	4	-10.32	0.82
3	->	5	-0.59	0.24
3	->	6	-0.05	0.04
3	->	7	0.00	0.01
3	->	8	-0.01	0.01
3	->	9	-0.00	0.00
3	->	10	-0.01	0.01
3	->	11	-0.04	0.05
3	->	12	-0.20	0.06
3	->	13	-3.25	0.64
3	->	14	-3.69	0.53
3	->	15	-1.37	0.44
3	->	16	-1.16	0.82
3	->	17	-0.80	0.77
3	->	18	-0.02	0.01
3	->	19	-0.06	0.04
3	->	20	-1.66	0.44
3	->	21	-0.03	0.01
3	->	22	-0.01	0.01
3	->	23	-0.02	0.01
3	->	24	-0.04	0.10
3	->	25	-0.00	0.00
3	->	26	-0.00	0.00
3	->	27	-0.01	0.01
3	->	28	-0.00	0.00
3	->	29	-0.00	0.00
3	->	30	-0.00	0.00
3	->	31	-0.00	0.00
3	->	32	-0.00	0.00

3 ->	33	-0.00	0.00
3 ->	34	-0.00	0.00
3 ->	35	-0.00	0.00
3 ->	36	-0.00	0.00
3 ->	37	0.00	0.00
3 ->	38	0.00	0.00
3 ->	39	0.00	0.00
3 ->	40	-0.00	0.00
3 ->	41	0.00	0.00
3 ->	42	0.00	0.00
3 ->	43	-0.00	0.00
3 ->	44	-0.00	0.00
3 ->	45	0.00	0.00
3 ->	46	0.00	0.00
3 ->	47	-0.00	0.00
3 ->	48	0.00	0.00
3 ->	49	0.00	0.00
3 ->	50	-0.00	0.00
3 ->	51	0.00	0.00
3 ->	52	0.00	0.00
3 ->	53	-0.00	0.00
3 ->	54	0.00	0.00
3 ->	55	0.00	0.00
3 ->	56	0.00	0.00
3 ->	57	0.00	0.00
3 ->	58	0.00	0.00
3 ->	59	-0.00	0.00
3 ->	60	0.00	0.00
3 ->	61	0.00	0.00
3 ->	62	0.00	0.00
3 ->	63	0.00	0.00
3 ->	64	0.00	0.00
3 ->	65	0.00	0.00
3 ->	66	0.00	0.00
3 ->	67	0.00	0.00
3 ->	68	-0.00	0.00
3 ->	69	0.00	0.00
3 ->	70	0.00	0.00
3 ->	71	0.00	0.00
3 ->	72	-0.00	0.00
3 ->	73	0.00	0.00
3 ->	74	0.00	0.00
3 ->	75	0.00	0.00
3 ->	76	-0.00	0.00
3 ->	77	-0.00	0.00
3 ->	78	-0.00	0.00
3 ->	79	0.00	0.00
3 ->	80	0.00	0.00
3 ->	81	-0.00	0.00
3 ->	82	0.00	0.00

3	->	83	0.00	0.00
3	->	84	-0.00	0.00
3	->	85	-0.00	0.00
3	->	86	0.00	0.00
3	->	87	0.00	0.00
3	->	88	0.00	0.00
3	->	89	0.00	0.00
3	->	90	-0.00	0.00
3	->	91	-0.00	0.00
3	->	92	-0.00	0.01
3	->	93	0.00	0.01
3	->	94	-0.01	0.00
3	->	95	-0.01	0.00
3	->	96	-0.01	0.01
3	->	97	-0.06	0.17
3	->	98	-0.02	0.02
3	->	99	-0.00	0.00
3	->	100	-0.00	0.00
3	->	101	-0.00	0.00
3	->	102	-0.00	0.00
3	->	103	0.00	0.00
3	->	104	0.00	0.00
3	->	105	0.00	0.00
3	->	106	0.00	0.00
3	->	107	0.00	0.00
4	->	1	-0.12	0.03
4	->	2	-0.38	0.16
4	->	3	-10.28	0.81
4	->	4	22.44	1.07
4	->	5	-12.77	0.75
4	->	6	-0.48	0.18
4	->	7	0.06	0.04
4	->	8	-0.09	0.13
4	->	9	-0.01	0.01
4	->	10	-0.08	0.04
4	->	11	-0.63	0.72
4	->	12	-0.15	0.14
4	->	13	-2.06	0.48
4	->	14	-0.55	0.23
4	->	15	-0.01	0.01
4	->	16	-0.02	0.01
4	->	17	-0.01	0.00
4	->	18	0.00	0.00
4	->	19	-0.00	0.01
4	->	20	-0.66	0.36
4	->	21	-0.01	0.00
4	->	22	0.01	0.01
4	->	23	-0.07	0.03
4	->	24	-0.06	0.06
4	->	25	-0.01	0.00

4	->	26	-0.00	0.00
4	->	27	-0.08	0.03
4	->	28	-0.00	0.00
4	->	29	-0.00	0.00
4	->	30	-0.00	0.00
4	->	31	-0.00	0.00
4	->	32	-0.00	0.00
4	->	33	-0.00	0.00
4	->	34	-0.00	0.00
4	->	35	0.00	0.00
4	->	36	-0.00	0.00
4	->	37	0.00	0.00
4	->	38	0.00	0.00
4	->	39	0.00	0.00
4	->	40	-0.00	0.00
4	->	41	-0.00	0.00
4	->	42	0.00	0.00
4	->	43	0.00	0.00
4	->	44	-0.00	0.00
4	->	45	-0.00	0.00
4	->	46	0.00	0.00
4	->	47	0.00	0.00
4	->	48	0.00	0.00
4	->	49	-0.00	0.00
4	->	50	-0.00	0.00
4	->	51	0.00	0.00
4	->	52	-0.00	0.00
4	->	53	-0.00	0.00
4	->	54	0.00	0.00
4	->	55	0.00	0.00
4	->	56	0.00	0.00
4	->	57	0.00	0.00
4	->	58	-0.00	0.00
4	->	59	0.00	0.00
4	->	60	0.00	0.00
4	->	61	0.00	0.00
4	->	62	0.00	0.00
4	->	63	-0.00	0.00
4	->	64	0.00	0.00
4	->	65	0.00	0.00
4	->	66	-0.00	0.00
4	->	67	0.00	0.00
4	->	68	0.00	0.00
4	->	69	0.00	0.00
4	->	70	0.00	0.00
4	->	71	0.00	0.00
4	->	72	-0.00	0.00
4	->	73	0.00	0.00
4	->	74	0.00	0.00
4	->	75	0.00	0.00

4	->	76	-0.00	0.00
4	->	77	0.00	0.00
4	->	78	0.00	0.00
4	->	79	0.00	0.00
4	->	80	0.00	0.00
4	->	81	0.00	0.00
4	->	82	0.00	0.00
4	->	83	0.00	0.00
4	->	84	0.00	0.00
4	->	85	-0.00	0.00
4	->	86	-0.00	0.00
4	->	87	0.00	0.00
4	->	88	-0.00	0.00
4	->	89	-0.00	0.00
4	->	90	-0.00	0.00
4	->	91	-0.01	0.00
4	->	92	-0.01	0.01
4	->	93	-0.00	0.00
4	->	94	-0.00	0.00
4	->	95	-0.00	0.00
4	->	96	-0.00	0.00
4	->	97	-0.00	0.00
4	->	98	0.00	0.00
4	->	99	0.00	0.00
4	->	100	0.00	0.00
4	->	101	0.00	0.00
4	->	102	0.00	0.00
4	->	103	-0.00	0.00
4	->	104	-0.00	0.00
4	->	105	-0.00	0.00
4	->	106	-0.00	0.00
4	->	107	-0.00	0.00
5	->	1	-0.17	0.03
5	->	2	-0.02	0.01
5	->	3	-0.59	0.24
5	->	4	-12.83	0.75
5	->	5	-25.66	1.88
5	->	6	-1.61	0.44
5	->	7	-5.21	2.08
5	->	8	0.14	0.13
5	->	9	-0.07	0.06
5	->	10	5.20	1.95
5	->	11	-0.98	0.88
5	->	12	-0.45	0.39
5	->	13	-0.70	0.28
5	->	14	-0.70	0.38
5	->	15	0.02	0.01
5	->	16	-0.04	0.01
5	->	17	-0.00	0.00
5	->	18	-0.02	0.01

5 ->	19	0.02	0.02
5 ->	20	-0.65	0.35
5 ->	21	-0.01	0.01
5 ->	22	-0.28	0.07
5 ->	23	1.80	0.52
5 ->	24	-0.39	0.36
5 ->	25	-0.06	0.02
5 ->	26	-0.09	0.03
5 ->	27	2.94	0.70
5 ->	28	-0.02	0.01
5 ->	29	-0.01	0.00
5 ->	30	-0.00	0.00
5 ->	31	-0.02	0.00
5 ->	32	-0.00	0.00
5 ->	33	0.00	0.00
5 ->	34	-0.00	0.00
5 ->	35	0.01	0.00
5 ->	36	-0.00	0.00
5 ->	37	-0.00	0.00
5 ->	38	0.00	0.00
5 ->	39	0.00	0.00
5 ->	40	-0.01	0.00
5 ->	41	-0.00	0.00
5 ->	42	-0.00	0.00
5 ->	43	-0.00	0.00
5 ->	44	-0.03	0.00
5 ->	45	-0.00	0.00
5 ->	46	0.01	0.00
5 ->	47	-0.00	0.00
5 ->	48	-0.00	0.00
5 ->	49	-0.00	0.00
5 ->	50	-0.00	0.00
5 ->	51	-0.00	0.00
5 ->	52	-0.00	0.00
5 ->	53	-0.00	0.00
5 ->	54	-0.00	0.00
5 ->	55	0.00	0.00
5 ->	56	-0.00	0.00
5 ->	57	-0.00	0.00
5 ->	58	0.00	0.00
5 ->	59	-0.00	0.00
5 ->	60	-0.00	0.00
5 ->	61	-0.00	0.00
5 ->	62	0.00	0.00
5 ->	63	0.00	0.00
5 ->	64	0.00	0.00
5 ->	65	0.00	0.00
5 ->	66	0.00	0.00
5 ->	67	0.00	0.00
5 ->	68	-0.00	0.00

5	->	69	-0.00	0.00
5	->	70	-0.00	0.00
5	->	71	0.00	0.00
5	->	72	-0.01	0.00
5	->	73	-0.00	0.00
5	->	74	0.01	0.00
5	->	75	0.00	0.00
5	->	76	-0.00	0.00
5	->	77	-0.01	0.00
5	->	78	-0.01	0.00
5	->	79	-0.00	0.00
5	->	80	-0.00	0.00
5	->	81	-0.00	0.00
5	->	82	-0.00	0.00
5	->	83	-0.00	0.00
5	->	84	0.00	0.00
5	->	85	-0.00	0.00
5	->	86	0.00	0.00
5	->	87	0.00	0.00
5	->	88	0.01	0.00
5	->	89	0.01	0.00
5	->	90	0.02	0.00
5	->	91	0.09	0.04
5	->	92	0.22	0.11
5	->	93	0.06	0.03
5	->	94	0.01	0.00
5	->	95	0.01	0.00
5	->	96	0.00	0.00
5	->	97	0.00	0.00
5	->	98	0.02	0.01
5	->	99	0.02	0.00
5	->	100	0.01	0.00
5	->	101	0.01	0.00
5	->	102	0.01	0.00
5	->	103	0.01	0.00
5	->	104	0.01	0.00
5	->	105	0.01	0.00
5	->	106	0.01	0.00
5	->	107	0.01	0.00
6	->	1	-0.00	0.00
6	->	2	-0.01	0.00
6	->	3	-0.05	0.04
6	->	4	-0.50	0.20
6	->	5	-1.70	0.45
6	->	6	1.25	0.84
6	->	7	-13.08	0.47
6	->	8	-0.44	0.21
6	->	9	-0.04	0.01
6	->	10	-0.07	0.03
6	->	11	-0.10	0.10

6	->	12	-0.03	0.02
6	->	13	-0.03	0.01
6	->	14	-0.10	0.19
6	->	15	-0.00	0.00
6	->	16	-0.00	0.00
6	->	17	-0.01	0.00
6	->	18	-0.00	0.00
6	->	19	-0.01	0.01
6	->	20	-0.43	0.46
6	->	21	-0.02	0.01
6	->	22	-0.00	0.01
6	->	23	-0.19	0.25
6	->	24	-1.43	0.33
6	->	25	-0.03	0.01
6	->	26	-0.01	0.01
6	->	27	-0.54	0.39
6	->	28	-0.19	0.22
6	->	29	-0.00	0.00
6	->	30	-0.01	0.01
6	->	31	-0.00	0.00
6	->	32	-0.00	0.00
6	->	33	-0.00	0.00
6	->	34	0.00	0.00
6	->	35	-0.00	0.00
6	->	36	0.00	0.00
6	->	37	0.00	0.00
6	->	38	-0.00	0.00
6	->	39	0.00	0.00
6	->	40	0.00	0.00
6	->	41	0.00	0.00
6	->	42	-0.00	0.00
6	->	43	-0.00	0.00
6	->	44	0.00	0.00
6	->	45	0.00	0.00
6	->	46	-0.00	0.00
6	->	47	0.00	0.00
6	->	48	0.00	0.00
6	->	49	0.00	0.00
6	->	50	0.00	0.00
6	->	51	0.00	0.00
6	->	52	0.00	0.00
6	->	53	0.00	0.00
6	->	54	0.00	0.00
6	->	55	0.00	0.00
6	->	56	0.00	0.00
6	->	57	0.00	0.00
6	->	58	-0.00	0.00
6	->	59	0.00	0.00
6	->	60	0.00	0.00
6	->	61	0.00	0.00

6	->	62	-0.00	0.00
6	->	63	-0.00	0.00
6	->	64	-0.00	0.00
6	->	65	0.00	0.00
6	->	66	0.00	0.00
6	->	67	0.00	0.00
6	->	68	0.00	0.00
6	->	69	0.00	0.00
6	->	70	0.00	0.00
6	->	71	0.00	0.00
6	->	72	0.00	0.00
6	->	73	0.00	0.00
6	->	74	-0.00	0.00
6	->	75	-0.00	0.00
6	->	76	0.00	0.00
6	->	77	0.00	0.00
6	->	78	0.00	0.00
6	->	79	0.00	0.00
6	->	80	0.00	0.00
6	->	81	0.00	0.00
6	->	82	0.00	0.00
6	->	83	0.00	0.00
6	->	84	0.00	0.00
6	->	85	0.00	0.00
6	->	86	0.00	0.00
6	->	87	-0.00	0.00
6	->	88	-0.00	0.00
6	->	89	-0.00	0.00
6	->	90	-0.00	0.00
6	->	91	-0.00	0.00
6	->	92	-0.01	0.01
6	->	93	-0.00	0.00
6	->	94	-0.00	0.00
6	->	95	-0.00	0.00
6	->	96	-0.00	0.00
6	->	97	-0.00	0.00
6	->	98	-0.01	0.00
6	->	99	-0.00	0.00
6	->	100	-0.00	0.00
6	->	101	-0.00	0.00
6	->	102	-0.00	0.00
6	->	103	-0.00	0.00
6	->	104	-0.00	0.00
6	->	105	-0.00	0.00
6	->	106	-0.00	0.00
6	->	107	-0.00	0.00
7	->	1	0.00	0.00
7	->	2	-0.00	0.00
7	->	3	0.00	0.01
7	->	4	0.06	0.04

7	->	5	-5.28	2.09
7	->	6	-13.22	0.46
7	->	7	26.87	1.48
7	->	8	-11.25	0.49
7	->	9	-1.51	0.35
7	->	10	-1.95	0.82
7	->	11	-0.43	0.35
7	->	12	-0.04	0.02
7	->	13	-0.01	0.01
7	->	14	-0.04	0.02
7	->	15	-0.00	0.00
7	->	16	-0.00	0.00
7	->	17	-0.00	0.00
7	->	18	0.00	0.00
7	->	19	0.00	0.00
7	->	20	-0.02	0.01
7	->	21	-0.01	0.01
7	->	22	0.03	0.02
7	->	23	-0.35	0.18
7	->	24	-0.26	0.31
7	->	25	-0.00	0.01
7	->	26	0.01	0.01
7	->	27	-2.47	0.56
7	->	28	-0.15	0.15
7	->	29	-0.01	0.00
7	->	30	-0.00	0.00
7	->	31	-0.00	0.00
7	->	32	-0.00	0.00
7	->	33	-0.00	0.00
7	->	34	0.00	0.00
7	->	35	0.00	0.00
7	->	36	-0.00	0.00
7	->	37	0.00	0.00
7	->	38	0.00	0.00
7	->	39	0.00	0.00
7	->	40	-0.00	0.00
7	->	41	0.00	0.00
7	->	42	0.00	0.00
7	->	43	-0.00	0.00
7	->	44	-0.00	0.00
7	->	45	-0.00	0.00
7	->	46	-0.00	0.00
7	->	47	-0.00	0.00
7	->	48	0.00	0.00
7	->	49	0.00	0.00
7	->	50	0.00	0.00
7	->	51	0.00	0.00
7	->	52	-0.00	0.00
7	->	53	-0.00	0.00
7	->	54	0.00	0.00

7 ->	55	0.00	0.00
7 ->	56	0.00	0.00
7 ->	57	0.00	0.00
7 ->	58	-0.00	0.00
7 ->	59	0.00	0.00
7 ->	60	0.00	0.00
7 ->	61	0.00	0.00
7 ->	62	0.00	0.00
7 ->	63	0.00	0.00
7 ->	64	0.00	0.00
7 ->	65	0.00	0.00
7 ->	66	0.00	0.00
7 ->	67	0.00	0.00
7 ->	68	0.00	0.00
7 ->	69	0.00	0.00
7 ->	70	0.00	0.00
7 ->	71	0.00	0.00
7 ->	72	-0.00	0.00
7 ->	73	-0.00	0.00
7 ->	74	0.00	0.00
7 ->	75	0.00	0.00
7 ->	76	-0.00	0.00
7 ->	77	0.00	0.00
7 ->	78	-0.00	0.00
7 ->	79	0.00	0.00
7 ->	80	0.00	0.00
7 ->	81	-0.00	0.00
7 ->	82	0.00	0.00
7 ->	83	0.00	0.00
7 ->	84	0.00	0.00
7 ->	85	0.00	0.00
7 ->	86	0.00	0.00
7 ->	87	0.00	0.00
7 ->	88	0.00	0.00
7 ->	89	-0.00	0.00
7 ->	90	-0.00	0.00
7 ->	91	-0.02	0.01
7 ->	92	-0.03	0.03
7 ->	93	-0.00	0.00
7 ->	94	-0.00	0.00
7 ->	95	-0.00	0.00
7 ->	96	0.00	0.00
7 ->	97	-0.00	0.00
7 ->	98	-0.00	0.00
7 ->	99	-0.00	0.00
7 ->	100	-0.00	0.00
7 ->	101	0.00	0.00
7 ->	102	-0.00	0.00
7 ->	103	0.00	0.00
7 ->	104	0.00	0.00

7	->	105	0.00	0.00
7	->	106	0.00	0.00
7	->	107	0.00	0.00
8	->	1	-0.05	0.02
8	->	2	0.00	0.00
8	->	3	-0.01	0.01
8	->	4	-0.09	0.13
8	->	5	0.14	0.13
8	->	6	-0.45	0.22
8	->	7	-11.26	0.49
8	->	8	-70.19	2.19
8	->	9	-18.54	0.46
8	->	10	-0.49	0.23
8	->	11	-0.57	0.56
8	->	12	-0.07	0.02
8	->	13	-0.25	0.91
8	->	14	-0.01	0.00
8	->	15	0.00	0.00
8	->	16	-0.01	0.00
8	->	17	0.00	0.00
8	->	18	0.00	0.00
8	->	19	0.02	0.01
8	->	20	-0.00	0.00
8	->	21	0.00	0.00
8	->	22	-0.02	0.01
8	->	23	0.00	0.01
8	->	24	-0.01	0.01
8	->	25	-0.01	0.00
8	->	26	-0.01	0.00
8	->	27	-0.03	0.07
8	->	28	-0.01	0.00
8	->	29	-0.00	0.00
8	->	30	-0.00	0.00
8	->	31	-0.01	0.00
8	->	32	-0.00	0.00
8	->	33	0.00	0.00
8	->	34	-0.00	0.00
8	->	35	0.00	0.00
8	->	36	-0.00	0.00
8	->	37	-0.00	0.00
8	->	38	0.00	0.00
8	->	39	0.00	0.00
8	->	40	-0.00	0.00
8	->	41	-0.00	0.00
8	->	42	-0.00	0.00
8	->	43	-0.00	0.00
8	->	44	-0.01	0.00
8	->	45	-0.00	0.00
8	->	46	0.00	0.00
8	->	47	-0.00	0.00

8 ->	48	-0.00	0.00
8 ->	49	-0.00	0.00
8 ->	50	-0.00	0.00
8 ->	51	-0.00	0.00
8 ->	52	-0.00	0.00
8 ->	53	-0.00	0.00
8 ->	54	-0.00	0.00
8 ->	55	0.00	0.00
8 ->	56	-0.00	0.00
8 ->	57	-0.00	0.00
8 ->	58	0.00	0.00
8 ->	59	-0.00	0.00
8 ->	60	-0.00	0.00
8 ->	61	-0.00	0.00
8 ->	62	0.00	0.00
8 ->	63	0.00	0.00
8 ->	64	0.00	0.00
8 ->	65	0.00	0.00
8 ->	66	0.00	0.00
8 ->	67	0.00	0.00
8 ->	68	-0.00	0.00
8 ->	69	-0.00	0.00
8 ->	70	-0.00	0.00
8 ->	71	0.00	0.00
8 ->	72	-0.00	0.00
8 ->	73	-0.00	0.00
8 ->	74	0.00	0.00
8 ->	75	0.00	0.00
8 ->	76	-0.00	0.00
8 ->	77	-0.00	0.00
8 ->	78	-0.00	0.00
8 ->	79	-0.00	0.00
8 ->	80	-0.00	0.00
8 ->	81	-0.00	0.00
8 ->	82	-0.00	0.00
8 ->	83	-0.00	0.00
8 ->	84	0.00	0.00
8 ->	85	-0.00	0.00
8 ->	86	0.00	0.00
8 ->	87	0.00	0.00
8 ->	88	0.00	0.00
8 ->	89	0.00	0.00
8 ->	90	0.01	0.00
8 ->	91	0.02	0.00
8 ->	92	0.02	0.01
8 ->	93	0.02	0.01
8 ->	94	0.01	0.00
8 ->	95	0.00	0.00
8 ->	96	0.00	0.00
8 ->	97	0.00	0.00

8 ->	98	0.01	0.00
8 ->	99	0.01	0.00
8 ->	100	0.00	0.00
8 ->	101	0.00	0.00
8 ->	102	0.00	0.00
8 ->	103	0.00	0.00
8 ->	104	0.00	0.00
8 ->	105	0.01	0.00
8 ->	106	0.01	0.00
8 ->	107	0.00	0.00
9 ->	1	-0.00	0.00
9 ->	2	-0.00	0.00
9 ->	3	-0.00	0.00
9 ->	4	-0.01	0.01
9 ->	5	-0.07	0.06
9 ->	6	-0.04	0.01
9 ->	7	-1.50	0.34
9 ->	8	-18.64	0.46
9 ->	9	14.35	1.28
9 ->	10	-8.90	0.75
9 ->	11	-0.41	0.13
9 ->	12	-0.08	0.14
9 ->	13	-0.02	0.01
9 ->	14	-0.01	0.00
9 ->	15	-0.00	0.00
9 ->	16	-0.00	0.00
9 ->	17	-0.00	0.00
9 ->	18	-0.00	0.00
9 ->	19	-0.00	0.00
9 ->	20	-0.00	0.00
9 ->	21	-0.00	0.00
9 ->	22	-0.00	0.00
9 ->	23	-0.05	0.04
9 ->	24	-0.01	0.01
9 ->	25	-0.00	0.00
9 ->	26	-0.01	0.02
9 ->	27	-0.42	0.56
9 ->	28	-0.01	0.01
9 ->	29	-0.00	0.00
9 ->	30	-0.00	0.00
9 ->	31	0.00	0.00
9 ->	32	0.00	0.00
9 ->	33	-0.00	0.00
9 ->	34	0.00	0.00
9 ->	35	-0.00	0.00
9 ->	36	0.00	0.00
9 ->	37	0.00	0.00
9 ->	38	-0.00	0.00
9 ->	39	-0.00	0.00
9 ->	40	0.00	0.00

9	->	41	0.00	0.00
9	->	42	0.00	0.00
9	->	43	-0.00	0.00
9	->	44	0.00	0.00
9	->	45	0.00	0.00
9	->	46	-0.00	0.00
9	->	47	-0.00	0.00
9	->	48	0.00	0.00
9	->	49	0.00	0.00
9	->	50	0.00	0.00
9	->	51	-0.00	0.00
9	->	52	0.00	0.00
9	->	53	0.00	0.00
9	->	54	0.00	0.00
9	->	55	0.00	0.00
9	->	56	0.00	0.00
9	->	57	0.00	0.00
9	->	58	-0.00	0.00
9	->	59	0.00	0.00
9	->	60	0.00	0.00
9	->	61	0.00	0.00
9	->	62	0.00	0.00
9	->	63	0.00	0.00
9	->	64	0.00	0.00
9	->	65	0.00	0.00
9	->	66	0.00	0.00
9	->	67	0.00	0.00
9	->	68	-0.00	0.00
9	->	69	-0.00	0.00
9	->	70	0.00	0.00
9	->	71	0.00	0.00
9	->	72	-0.00	0.00
9	->	73	0.00	0.00
9	->	74	0.00	0.00
9	->	75	0.00	0.00
9	->	76	-0.00	0.00
9	->	77	-0.00	0.00
9	->	78	-0.00	0.00
9	->	79	0.00	0.00
9	->	80	0.00	0.00
9	->	81	-0.00	0.00
9	->	82	0.00	0.00
9	->	83	0.00	0.00
9	->	84	0.00	0.00
9	->	85	0.00	0.00
9	->	86	0.00	0.00
9	->	87	0.00	0.00
9	->	88	-0.00	0.00
9	->	89	-0.00	0.00
9	->	90	-0.00	0.00

9	->	91	-0.01	0.01
9	->	92	-0.01	0.01
9	->	93	0.00	0.00
9	->	94	0.00	0.00
9	->	95	0.00	0.00
9	->	96	0.00	0.00
9	->	97	-0.00	0.00
9	->	98	-0.00	0.00
9	->	99	-0.00	0.00
9	->	100	-0.00	0.00
9	->	101	0.00	0.00
9	->	102	0.00	0.00
9	->	103	0.00	0.00
9	->	104	0.00	0.00
9	->	105	0.00	0.00
9	->	106	0.00	0.00
9	->	107	0.00	0.00
10	->	1	-0.10	0.01
10	->	2	-0.00	0.00
10	->	3	-0.01	0.01
10	->	4	-0.08	0.04
10	->	5	5.19	1.95
10	->	6	-0.07	0.03
10	->	7	-1.94	0.81
10	->	8	-0.52	0.23
10	->	9	-9.03	0.74
10	->	10	-25.45	8.67
10	->	11	-13.72	0.51
10	->	12	-3.91	4.58
10	->	13	-0.20	0.28
10	->	14	-0.04	0.03
10	->	15	-0.00	0.00
10	->	16	-0.03	0.00
10	->	17	0.00	0.00
10	->	18	-0.00	0.00
10	->	19	0.04	0.01
10	->	20	-0.00	0.01
10	->	21	-0.00	0.00
10	->	22	-0.17	0.05
10	->	23	2.26	0.55
10	->	24	0.03	0.01
10	->	25	-0.04	0.01
10	->	26	-0.08	0.03
10	->	27	3.32	0.54
10	->	28	-0.01	0.00
10	->	29	-0.01	0.00
10	->	30	-0.00	0.00
10	->	31	-0.03	0.00
10	->	32	-0.00	0.00
10	->	33	0.00	0.00

10	->	34	-0.00	0.00
10	->	35	0.01	0.00
10	->	36	-0.00	0.00
10	->	37	-0.00	0.00
10	->	38	0.01	0.00
10	->	39	0.00	0.00
10	->	40	-0.01	0.00
10	->	41	-0.00	0.00
10	->	42	-0.00	0.00
10	->	43	-0.00	0.00
10	->	44	-0.03	0.01
10	->	45	0.00	0.00
10	->	46	0.01	0.00
10	->	47	-0.00	0.00
10	->	48	-0.00	0.00
10	->	49	-0.00	0.00
10	->	50	-0.00	0.00
10	->	51	-0.00	0.00
10	->	52	-0.00	0.00
10	->	53	-0.00	0.00
10	->	54	-0.00	0.00
10	->	55	0.00	0.00
10	->	56	-0.00	0.00
10	->	57	-0.00	0.00
10	->	58	0.00	0.00
10	->	59	-0.00	0.00
10	->	60	-0.00	0.00
10	->	61	-0.00	0.00
10	->	62	0.00	0.00
10	->	63	0.00	0.00
10	->	64	0.00	0.00
10	->	65	0.00	0.00
10	->	66	0.00	0.00
10	->	67	0.00	0.00
10	->	68	-0.00	0.00
10	->	69	-0.00	0.00
10	->	70	-0.00	0.00
10	->	71	-0.00	0.00
10	->	72	-0.01	0.00
10	->	73	-0.00	0.00
10	->	74	0.01	0.00
10	->	75	0.01	0.00
10	->	76	-0.00	0.00
10	->	77	-0.01	0.00
10	->	78	-0.01	0.00
10	->	79	-0.00	0.00
10	->	80	-0.00	0.00
10	->	81	-0.01	0.00
10	->	82	-0.00	0.00
10	->	83	-0.00	0.00

10	->	84	-0.00	0.00
10	->	85	-0.00	0.00
10	->	86	0.00	0.00
10	->	87	0.00	0.00
10	->	88	0.01	0.00
10	->	89	0.01	0.00
10	->	90	0.03	0.01
10	->	91	0.14	0.07
10	->	92	0.30	0.17
10	->	93	0.06	0.01
10	->	94	0.02	0.00
10	->	95	0.01	0.00
10	->	96	0.00	0.00
10	->	97	0.00	0.00
10	->	98	0.02	0.00
10	->	99	0.01	0.00
10	->	100	0.01	0.00
10	->	101	0.01	0.00
10	->	102	0.01	0.00
10	->	103	0.01	0.00
10	->	104	0.01	0.00
10	->	105	0.02	0.00
10	->	106	0.02	0.01
10	->	107	0.01	0.00
11	->	1	-0.13	0.02
11	->	2	-0.01	0.00
11	->	3	-0.04	0.05
11	->	4	-0.63	0.71
11	->	5	-1.00	0.90
11	->	6	-0.10	0.10
11	->	7	-0.44	0.36
11	->	8	-0.58	0.56
11	->	9	-0.41	0.13
11	->	10	-13.79	0.54
11	->	11	-32.26	2.87
11	->	12	-9.09	0.68
11	->	13	-1.19	1.23
11	->	14	-0.07	0.03
11	->	15	-0.00	0.00
11	->	16	-0.02	0.00
11	->	17	0.00	0.00
11	->	18	-0.00	0.00
11	->	19	0.03	0.00
11	->	20	-0.01	0.01
11	->	21	-0.00	0.00
11	->	22	-0.04	0.01
11	->	23	0.05	0.05
11	->	24	-0.01	0.01
11	->	25	-0.02	0.00
11	->	26	-0.01	0.01

11	->	27	0.16	0.11
11	->	28	-0.00	0.00
11	->	29	-0.00	0.00
11	->	30	-0.00	0.00
11	->	31	-0.01	0.00
11	->	32	-0.00	0.00
11	->	33	0.00	0.00
11	->	34	-0.00	0.00
11	->	35	0.00	0.00
11	->	36	-0.00	0.00
11	->	37	-0.00	0.00
11	->	38	0.00	0.00
11	->	39	0.00	0.00
11	->	40	-0.01	0.00
11	->	41	-0.00	0.00
11	->	42	-0.00	0.00
11	->	43	-0.00	0.00
11	->	44	-0.01	0.00
11	->	45	0.00	0.00
11	->	46	0.01	0.00
11	->	47	-0.00	0.00
11	->	48	-0.00	0.00
11	->	49	-0.00	0.00
11	->	50	-0.00	0.00
11	->	51	-0.00	0.00
11	->	52	-0.00	0.00
11	->	53	-0.00	0.00
11	->	54	-0.00	0.00
11	->	55	0.00	0.00
11	->	56	-0.00	0.00
11	->	57	-0.00	0.00
11	->	58	0.00	0.00
11	->	59	-0.00	0.00
11	->	60	-0.00	0.00
11	->	61	-0.00	0.00
11	->	62	0.00	0.00
11	->	63	0.00	0.00
11	->	64	0.00	0.00
11	->	65	0.00	0.00
11	->	66	0.00	0.00
11	->	67	0.00	0.00
11	->	68	-0.00	0.00
11	->	69	-0.00	0.00
11	->	70	-0.00	0.00
11	->	71	-0.00	0.00
11	->	72	-0.01	0.00
11	->	73	-0.00	0.00
11	->	74	0.01	0.00
11	->	75	0.00	0.00
11	->	76	-0.00	0.00

11	->	77	-0.01	0.00
11	->	78	-0.01	0.00
11	->	79	-0.00	0.00
11	->	80	-0.00	0.00
11	->	81	-0.01	0.00
11	->	82	-0.00	0.00
11	->	83	-0.00	0.00
11	->	84	-0.00	0.00
11	->	85	-0.00	0.00
11	->	86	0.00	0.00
11	->	87	0.00	0.00
11	->	88	0.00	0.00
11	->	89	0.00	0.00
11	->	90	0.01	0.00
11	->	91	0.02	0.01
11	->	92	0.03	0.02
11	->	93	0.03	0.01
11	->	94	0.01	0.00
11	->	95	0.01	0.00
11	->	96	0.00	0.00
11	->	97	0.00	0.00
11	->	98	0.01	0.00
11	->	99	0.01	0.00
11	->	100	0.01	0.00
11	->	101	0.00	0.00
11	->	102	0.01	0.00
11	->	103	0.01	0.00
11	->	104	0.01	0.00
11	->	105	0.01	0.01
11	->	106	0.01	0.01
11	->	107	0.01	0.00
12	->	1	0.49	0.87
12	->	2	-0.02	0.01
12	->	3	-0.20	0.06
12	->	4	-0.15	0.13
12	->	5	-0.45	0.39
12	->	6	-0.03	0.02
12	->	7	-0.04	0.02
12	->	8	-0.07	0.02
12	->	9	-0.09	0.15
12	->	10	-3.99	4.61
12	->	11	-9.17	0.65
12	->	12	-109.89	4.31
12	->	13	-9.34	0.51
12	->	14	-1.74	0.81
12	->	15	-0.08	0.04
12	->	16	0.05	0.04
12	->	17	-0.00	0.00
12	->	18	0.01	0.01
12	->	19	-0.12	0.10

12	->	20	-0.05	0.02
12	->	21	-0.00	0.00
12	->	22	0.41	0.63
12	->	23	-0.27	0.22
12	->	24	-0.02	0.02
12	->	25	0.02	0.01
12	->	26	-0.01	0.02
12	->	27	-0.19	0.18
12	->	28	0.00	0.00
12	->	29	0.00	0.00
12	->	30	-0.00	0.00
12	->	31	0.02	0.01
12	->	32	0.00	0.00
12	->	33	-0.00	0.00
12	->	34	0.00	0.00
12	->	35	-0.00	0.00
12	->	36	0.00	0.00
12	->	37	0.00	0.00
12	->	38	-0.00	0.00
12	->	39	-0.00	0.00
12	->	40	0.01	0.00
12	->	41	0.00	0.00
12	->	42	0.00	0.00
12	->	43	0.00	0.00
12	->	44	0.03	0.01
12	->	45	-0.00	0.00
12	->	46	-0.02	0.01
12	->	47	0.00	0.00
12	->	48	0.00	0.00
12	->	49	0.00	0.00
12	->	50	0.00	0.00
12	->	51	0.00	0.00
12	->	52	0.00	0.00
12	->	53	0.01	0.00
12	->	54	0.00	0.00
12	->	55	-0.00	0.00
12	->	56	0.00	0.00
12	->	57	0.00	0.00
12	->	58	-0.00	0.00
12	->	59	0.00	0.00
12	->	60	0.00	0.00
12	->	61	0.00	0.00
12	->	62	-0.00	0.00
12	->	63	-0.00	0.00
12	->	64	-0.00	0.00
12	->	65	-0.00	0.00
12	->	66	-0.00	0.00
12	->	67	0.00	0.00
12	->	68	0.00	0.00
12	->	69	0.00	0.00

12	->	70	0.00	0.00
12	->	71	0.00	0.00
12	->	72	0.01	0.01
12	->	73	0.00	0.00
12	->	74	-0.01	0.01
12	->	75	-0.01	0.00
12	->	76	0.00	0.00
12	->	77	0.01	0.01
12	->	78	0.01	0.01
12	->	79	0.00	0.00
12	->	80	0.00	0.00
12	->	81	0.01	0.01
12	->	82	0.00	0.00
12	->	83	0.00	0.00
12	->	84	0.00	0.00
12	->	85	0.00	0.00
12	->	86	-0.00	0.00
12	->	87	-0.00	0.00
12	->	88	-0.01	0.00
12	->	89	-0.01	0.01
12	->	90	-0.04	0.04
12	->	91	-0.88	1.74
12	->	92	-3.52	4.80
12	->	93	-1.84	2.93
12	->	94	-0.05	0.03
12	->	95	-0.02	0.01
12	->	96	-0.01	0.00
12	->	97	-0.00	0.00
12	->	98	-0.01	0.00
12	->	99	-0.01	0.00
12	->	100	-0.01	0.00
12	->	101	-0.01	0.00
12	->	102	-0.02	0.01
12	->	103	-0.03	0.01
12	->	104	-0.03	0.02
12	->	105	-0.04	0.02
12	->	106	-0.03	0.01
12	->	107	-0.01	0.01
13	->	1	-3.80	2.00
13	->	2	-1.94	0.95
13	->	3	-3.27	0.64
13	->	4	-2.08	0.48
13	->	5	-0.70	0.28
13	->	6	-0.03	0.01
13	->	7	-0.01	0.01
13	->	8	-0.26	0.93
13	->	9	-0.02	0.01
13	->	10	-0.20	0.28
13	->	11	-1.23	1.25
13	->	12	-9.39	0.50

13	->	13	-108.41	2.43
13	->	14	-12.39	0.59
13	->	15	-0.65	0.10
13	->	16	0.04	0.04
13	->	17	-0.02	0.01
13	->	18	0.02	0.01
13	->	19	-0.21	0.22
13	->	20	-0.07	0.02
13	->	21	-0.01	0.01
13	->	22	0.11	0.07
13	->	23	-0.05	0.03
13	->	24	-0.01	0.01
13	->	25	0.01	0.00
13	->	26	0.00	0.00
13	->	27	-0.03	0.01
13	->	28	0.00	0.00
13	->	29	0.00	0.00
13	->	30	-0.00	0.00
13	->	31	0.01	0.00
13	->	32	0.00	0.00
13	->	33	-0.00	0.00
13	->	34	0.00	0.00
13	->	35	-0.00	0.00
13	->	36	0.00	0.00
13	->	37	0.00	0.00
13	->	38	-0.00	0.00
13	->	39	-0.00	0.00
13	->	40	0.00	0.00
13	->	41	0.00	0.00
13	->	42	0.00	0.00
13	->	43	-0.00	0.00
13	->	44	0.01	0.00
13	->	45	0.00	0.00
13	->	46	-0.01	0.00
13	->	47	0.00	0.00
13	->	48	0.00	0.00
13	->	49	0.00	0.00
13	->	50	0.00	0.00
13	->	51	0.00	0.00
13	->	52	0.00	0.00
13	->	53	0.00	0.00
13	->	54	0.00	0.00
13	->	55	-0.00	0.00
13	->	56	0.00	0.00
13	->	57	0.00	0.00
13	->	58	-0.00	0.00
13	->	59	0.00	0.00
13	->	60	0.00	0.00
13	->	61	0.00	0.00
13	->	62	-0.00	0.00

13	->	63	-0.00	0.00
13	->	64	-0.00	0.00
13	->	65	-0.00	0.00
13	->	66	-0.00	0.00
13	->	67	0.00	0.00
13	->	68	0.00	0.00
13	->	69	0.00	0.00
13	->	70	0.00	0.00
13	->	71	-0.00	0.00
13	->	72	0.00	0.00
13	->	73	0.00	0.00
13	->	74	-0.00	0.00
13	->	75	-0.00	0.00
13	->	76	0.00	0.00
13	->	77	0.00	0.00
13	->	78	0.00	0.00
13	->	79	0.00	0.00
13	->	80	0.00	0.00
13	->	81	0.00	0.00
13	->	82	0.00	0.00
13	->	83	0.00	0.00
13	->	84	0.00	0.00
13	->	85	0.00	0.00
13	->	86	-0.00	0.00
13	->	87	-0.00	0.00
13	->	88	-0.00	0.00
13	->	89	-0.00	0.00
13	->	90	-0.01	0.00
13	->	91	-0.03	0.01
13	->	92	-0.11	0.08
13	->	93	-0.11	0.13
13	->	94	-0.04	0.02
13	->	95	-0.01	0.00
13	->	96	-0.00	0.00
13	->	97	-0.00	0.00
13	->	98	-0.02	0.01
13	->	99	-0.01	0.00
13	->	100	-0.01	0.00
13	->	101	-0.01	0.00
13	->	102	-0.01	0.00
13	->	103	-0.01	0.00
13	->	104	-0.01	0.00
13	->	105	-0.01	0.00
13	->	106	-0.01	0.00
13	->	107	-0.00	0.00
14	->	1	-2.99	1.09
14	->	2	-0.99	0.18
14	->	3	-3.71	0.54
14	->	4	-0.55	0.23
14	->	5	-0.69	0.37

14	->	6	-0.10	0.19
14	->	7	-0.04	0.02
14	->	8	-0.01	0.00
14	->	9	-0.01	0.00
14	->	10	-0.04	0.04
14	->	11	-0.07	0.03
14	->	12	-1.76	0.79
14	->	13	-12.44	0.59
14	->	14	28.95	1.61
14	->	15	-16.97	0.49
14	->	16	-0.62	0.42
14	->	17	-0.15	0.05
14	->	18	-0.06	0.03
14	->	19	-2.54	1.15
14	->	20	-2.22	0.58
14	->	21	-0.06	0.02
14	->	22	-0.83	0.41
14	->	23	-2.17	0.38
14	->	24	-0.07	0.03
14	->	25	-0.01	0.01
14	->	26	-0.03	0.01
14	->	27	-0.02	0.02
14	->	28	-0.00	0.00
14	->	29	-0.00	0.00
14	->	30	-0.00	0.00
14	->	31	-0.00	0.00
14	->	32	-0.00	0.00
14	->	33	-0.00	0.00
14	->	34	0.00	0.00
14	->	35	-0.00	0.00
14	->	36	0.00	0.00
14	->	37	0.00	0.00
14	->	38	-0.00	0.00
14	->	39	-0.00	0.00
14	->	40	0.00	0.00
14	->	41	0.00	0.00
14	->	42	-0.00	0.00
14	->	43	-0.00	0.00
14	->	44	-0.00	0.00
14	->	45	0.00	0.00
14	->	46	-0.00	0.00
14	->	47	-0.00	0.00
14	->	48	0.00	0.00
14	->	49	0.00	0.00
14	->	50	0.00	0.00
14	->	51	0.00	0.00
14	->	52	0.00	0.00
14	->	53	0.00	0.00
14	->	54	0.00	0.00
14	->	55	0.00	0.00

14	->	56	0.00	0.00
14	->	57	0.00	0.00
14	->	58	-0.00	0.00
14	->	59	0.00	0.00
14	->	60	0.00	0.00
14	->	61	0.00	0.00
14	->	62	0.00	0.00
14	->	63	-0.00	0.00
14	->	64	-0.00	0.00
14	->	65	0.00	0.00
14	->	66	-0.00	0.00
14	->	67	0.00	0.00
14	->	68	0.00	0.00
14	->	69	0.00	0.00
14	->	70	0.00	0.00
14	->	71	0.00	0.00
14	->	72	0.00	0.00
14	->	73	0.00	0.00
14	->	74	-0.00	0.00
14	->	75	-0.00	0.00
14	->	76	0.00	0.00
14	->	77	0.00	0.00
14	->	78	0.00	0.00
14	->	79	0.00	0.00
14	->	80	0.00	0.00
14	->	81	0.00	0.00
14	->	82	0.00	0.00
14	->	83	0.00	0.00
14	->	84	0.00	0.00
14	->	85	0.00	0.00
14	->	86	-0.00	0.00
14	->	87	-0.00	0.00
14	->	88	-0.00	0.00
14	->	89	-0.00	0.00
14	->	90	-0.00	0.00
14	->	91	0.02	0.03
14	->	92	-0.42	0.45
14	->	93	-0.66	0.65
14	->	94	-0.10	0.05
14	->	95	-0.01	0.00
14	->	96	-0.00	0.00
14	->	97	-0.01	0.00
14	->	98	-0.01	0.00
14	->	99	-0.01	0.00
14	->	100	-0.01	0.00
14	->	101	-0.00	0.00
14	->	102	-0.00	0.00
14	->	103	-0.00	0.00
14	->	104	-0.00	0.00
14	->	105	-0.00	0.00

14	->	106	-0.00	0.00
14	->	107	-0.00	0.00
15	->	1	-4.56	1.44
15	->	2	-0.94	0.42
15	->	3	-1.35	0.44
15	->	4	-0.01	0.01
15	->	5	0.02	0.01
15	->	6	-0.00	0.00
15	->	7	-0.00	0.00
15	->	8	0.00	0.00
15	->	9	-0.00	0.00
15	->	10	-0.00	0.00
15	->	11	-0.00	0.00
15	->	12	-0.08	0.04
15	->	13	-0.65	0.10
15	->	14	-16.97	0.48
15	->	15	20.20	2.16
15	->	16	-7.07	1.05
15	->	17	-0.34	0.07
15	->	18	0.00	0.03
15	->	19	-1.48	2.20
15	->	20	-0.21	0.28
15	->	21	-0.00	0.01
15	->	22	-0.01	0.04
15	->	23	-0.01	0.00
15	->	24	-0.00	0.00
15	->	25	-0.00	0.00
15	->	26	-0.00	0.00
15	->	27	0.00	0.00
15	->	28	-0.00	0.00
15	->	29	0.00	0.00
15	->	30	0.00	0.00
15	->	31	0.00	0.00
15	->	32	0.00	0.00
15	->	33	0.00	0.00
15	->	34	0.00	0.00
15	->	35	-0.00	0.00
15	->	36	-0.00	0.00
15	->	37	0.00	0.00
15	->	38	-0.00	0.00
15	->	39	0.00	0.00
15	->	40	0.00	0.00
15	->	41	-0.00	0.00
15	->	42	0.00	0.00
15	->	43	0.00	0.00
15	->	44	0.00	0.00
15	->	45	-0.00	0.00
15	->	46	-0.00	0.00
15	->	47	0.00	0.00
15	->	48	0.00	0.00

15	->	49	0.00	0.00
15	->	50	0.00	0.00
15	->	51	0.00	0.00
15	->	52	0.00	0.00
15	->	53	0.00	0.00
15	->	54	0.00	0.00
15	->	55	0.00	0.00
15	->	56	0.00	0.00
15	->	57	0.00	0.00
15	->	58	-0.00	0.00
15	->	59	0.00	0.00
15	->	60	0.00	0.00
15	->	61	0.00	0.00
15	->	62	-0.00	0.00
15	->	63	-0.00	0.00
15	->	64	-0.00	0.00
15	->	65	-0.00	0.00
15	->	66	-0.00	0.00
15	->	67	0.00	0.00
15	->	68	0.00	0.00
15	->	69	0.00	0.00
15	->	70	0.00	0.00
15	->	71	0.00	0.00
15	->	72	0.00	0.00
15	->	73	0.00	0.00
15	->	74	-0.00	0.00
15	->	75	-0.00	0.00
15	->	76	0.00	0.00
15	->	77	0.00	0.00
15	->	78	0.00	0.00
15	->	79	0.00	0.00
15	->	80	0.00	0.00
15	->	81	0.00	0.00
15	->	82	0.00	0.00
15	->	83	0.00	0.00
15	->	84	0.00	0.00
15	->	85	-0.00	0.00
15	->	86	-0.00	0.00
15	->	87	-0.00	0.00
15	->	88	-0.00	0.00
15	->	89	-0.00	0.00
15	->	90	-0.00	0.00
15	->	91	0.00	0.00
15	->	92	0.01	0.02
15	->	93	0.01	0.09
15	->	94	-0.01	0.15
15	->	95	-0.01	0.01
15	->	96	-0.01	0.00
15	->	97	-0.01	0.00
15	->	98	-0.01	0.01

15	->	99	-0.00	0.00
15	->	100	-0.00	0.00
15	->	101	-0.00	0.00
15	->	102	-0.00	0.00
15	->	103	-0.00	0.00
15	->	104	-0.00	0.00
15	->	105	-0.00	0.00
15	->	106	-0.00	0.00
15	->	107	-0.00	0.00
16	->	1	0.68	1.13
16	->	2	-0.09	0.05
16	->	3	-1.17	0.82
16	->	4	-0.02	0.01
16	->	5	-0.04	0.01
16	->	6	-0.00	0.00
16	->	7	-0.00	0.00
16	->	8	-0.01	0.00
16	->	9	-0.00	0.00
16	->	10	-0.03	0.00
16	->	11	-0.02	0.00
16	->	12	0.05	0.04
16	->	13	0.04	0.04
16	->	14	-0.62	0.42
16	->	15	-7.23	1.07
16	->	16	-97.81	3.42
16	->	17	-21.52	0.34
16	->	18	-0.66	0.28
16	->	19	-13.72	3.26
16	->	20	-1.96	0.63
16	->	21	-0.10	0.08
16	->	22	0.47	0.27
16	->	23	-0.02	0.01
16	->	24	-0.01	0.01
16	->	25	0.03	0.00
16	->	26	0.00	0.00
16	->	27	-0.00	0.00
16	->	28	0.00	0.00
16	->	29	0.00	0.00
16	->	30	-0.00	0.00
16	->	31	0.01	0.00
16	->	32	0.00	0.00
16	->	33	-0.00	0.00
16	->	34	0.00	0.00
16	->	35	-0.00	0.00
16	->	36	0.00	0.00
16	->	37	0.00	0.00
16	->	38	-0.00	0.00
16	->	39	-0.00	0.00
16	->	40	0.01	0.00
16	->	41	0.00	0.00

16 ->	42	0.00	0.00
16 ->	43	-0.00	0.00
16 ->	44	0.06	0.01
16 ->	45	-0.00	0.00
16 ->	46	-0.03	0.01
16 ->	47	0.00	0.00
16 ->	48	0.00	0.00
16 ->	49	0.00	0.00
16 ->	50	0.00	0.00
16 ->	51	0.00	0.00
16 ->	52	0.00	0.00
16 ->	53	0.01	0.00
16 ->	54	0.00	0.00
16 ->	55	0.00	0.00
16 ->	56	0.00	0.00
16 ->	57	0.00	0.00
16 ->	58	-0.00	0.00
16 ->	59	0.00	0.00
16 ->	60	0.00	0.00
16 ->	61	0.00	0.00
16 ->	62	-0.00	0.00
16 ->	63	-0.00	0.00
16 ->	64	-0.00	0.00
16 ->	65	-0.00	0.00
16 ->	66	-0.00	0.00
16 ->	67	-0.00	0.00
16 ->	68	0.00	0.00
16 ->	69	0.00	0.00
16 ->	70	0.00	0.00
16 ->	71	-0.00	0.00
16 ->	72	0.02	0.00
16 ->	73	0.00	0.00
16 ->	74	-0.02	0.00
16 ->	75	-0.01	0.00
16 ->	76	0.01	0.00
16 ->	77	0.02	0.00
16 ->	78	0.01	0.00
16 ->	79	0.00	0.00
16 ->	80	0.00	0.00
16 ->	81	0.01	0.00
16 ->	82	0.00	0.00
16 ->	83	0.00	0.00
16 ->	84	0.00	0.00
16 ->	85	0.00	0.00
16 ->	86	-0.00	0.00
16 ->	87	-0.00	0.00
16 ->	88	-0.01	0.00
16 ->	89	-0.01	0.00
16 ->	90	-0.02	0.00
16 ->	91	-0.05	0.03

16	->	92	-0.27	0.23
16	->	93	-3.01	1.29
16	->	94	-11.73	2.06
16	->	95	-0.55	0.28
16	->	96	-0.12	0.07
16	->	97	-0.08	0.05
16	->	98	-0.40	0.17
16	->	99	-0.09	0.04
16	->	100	0.03	0.02
16	->	101	-0.05	0.01
16	->	102	-0.05	0.01
16	->	103	-0.04	0.01
16	->	104	-0.03	0.00
16	->	105	-0.02	0.00
16	->	106	-0.01	0.00
16	->	107	-0.00	0.00
17	->	1	-0.04	0.02
17	->	2	-0.02	0.02
17	->	3	-0.81	0.78
17	->	4	-0.01	0.00
17	->	5	-0.00	0.00
17	->	6	-0.01	0.00
17	->	7	-0.00	0.00
17	->	8	0.00	0.00
17	->	9	-0.00	0.00
17	->	10	0.00	0.00
17	->	11	0.00	0.00
17	->	12	-0.00	0.00
17	->	13	-0.02	0.01
17	->	14	-0.15	0.05
17	->	15	-0.34	0.07
17	->	16	-21.52	0.35
17	->	17	21.14	2.35
17	->	18	-9.38	0.41
17	->	19	-0.64	0.18
17	->	20	-2.14	0.36
17	->	21	-4.23	1.05
17	->	22	-0.17	0.10
17	->	23	-0.04	0.03
17	->	24	-0.05	0.02
17	->	25	-0.02	0.01
17	->	26	-0.00	0.00
17	->	27	-0.00	0.00
17	->	28	-0.00	0.00
17	->	29	-0.00	0.00
17	->	30	-0.00	0.00
17	->	31	-0.00	0.00
17	->	32	-0.00	0.00
17	->	33	-0.00	0.00
17	->	34	-0.00	0.00

17 ->	35	0.00	0.00
17 ->	36	-0.00	0.00
17 ->	37	-0.00	0.00
17 ->	38	0.00	0.00
17 ->	39	0.00	0.00
17 ->	40	-0.00	0.00
17 ->	41	-0.00	0.00
17 ->	42	0.00	0.00
17 ->	43	-0.00	0.00
17 ->	44	0.00	0.01
17 ->	45	-0.00	0.00
17 ->	46	-0.00	0.00
17 ->	47	-0.00	0.00
17 ->	48	-0.00	0.00
17 ->	49	-0.00	0.00
17 ->	50	-0.00	0.00
17 ->	51	-0.00	0.00
17 ->	52	-0.00	0.00
17 ->	53	-0.00	0.00
17 ->	54	-0.00	0.00
17 ->	55	0.00	0.00
17 ->	56	-0.00	0.00
17 ->	57	0.00	0.00
17 ->	58	0.00	0.00
17 ->	59	-0.00	0.00
17 ->	60	-0.00	0.00
17 ->	61	0.00	0.00
17 ->	62	0.00	0.00
17 ->	63	0.00	0.00
17 ->	64	0.00	0.00
17 ->	65	0.00	0.00
17 ->	66	0.00	0.00
17 ->	67	0.00	0.00
17 ->	68	-0.00	0.00
17 ->	69	-0.00	0.00
17 ->	70	0.00	0.00
17 ->	71	0.00	0.00
17 ->	72	-0.00	0.00
17 ->	73	0.00	0.00
17 ->	74	0.00	0.00
17 ->	75	0.00	0.00
17 ->	76	-0.00	0.00
17 ->	77	-0.00	0.00
17 ->	78	-0.00	0.00
17 ->	79	-0.00	0.00
17 ->	80	0.00	0.00
17 ->	81	-0.00	0.00
17 ->	82	-0.00	0.00
17 ->	83	0.00	0.00
17 ->	84	0.00	0.00

17	->	85	0.00	0.00
17	->	86	0.00	0.00
17	->	87	0.00	0.00
17	->	88	0.00	0.00
17	->	89	0.00	0.00
17	->	90	0.00	0.00
17	->	91	-0.00	0.00
17	->	92	-0.01	0.01
17	->	93	-0.02	0.01
17	->	94	-0.07	0.03
17	->	95	-0.01	0.01
17	->	96	-0.05	0.05
17	->	97	-0.88	0.84
17	->	98	-2.31	1.24
17	->	99	-0.12	0.05
17	->	100	-0.00	0.01
17	->	101	-0.00	0.00
17	->	102	-0.00	0.00
17	->	103	-0.00	0.00
17	->	104	0.00	0.00
17	->	105	0.00	0.00
17	->	106	0.00	0.00
17	->	107	0.00	0.00
18	->	1	0.06	0.09
18	->	2	-0.00	0.00
18	->	3	-0.02	0.01
18	->	4	0.00	0.00
18	->	5	-0.02	0.01
18	->	6	-0.00	0.00
18	->	7	0.00	0.00
18	->	8	0.00	0.00
18	->	9	-0.00	0.00
18	->	10	-0.00	0.00
18	->	11	-0.00	0.00
18	->	12	0.01	0.01
18	->	13	0.02	0.01
18	->	14	-0.06	0.03
18	->	15	0.00	0.03
18	->	16	-0.67	0.28
18	->	17	-9.40	0.41
18	->	18	24.17	0.66
18	->	19	-14.56	0.57
18	->	20	-0.82	0.16
18	->	21	-1.71	0.53
18	->	22	-1.56	0.46
18	->	23	-0.02	0.06
18	->	24	-0.01	0.01
18	->	25	-0.02	0.02
18	->	26	-0.00	0.00
18	->	27	-0.01	0.00

18	->	28	-0.00	0.00
18	->	29	-0.00	0.00
18	->	30	-0.00	0.00
18	->	31	-0.00	0.00
18	->	32	-0.00	0.00
18	->	33	-0.00	0.00
18	->	34	-0.00	0.00
18	->	35	0.00	0.00
18	->	36	-0.00	0.00
18	->	37	-0.00	0.00
18	->	38	0.00	0.00
18	->	39	0.00	0.00
18	->	40	-0.00	0.00
18	->	41	0.00	0.00
18	->	42	0.00	0.00
18	->	43	-0.00	0.00
18	->	44	-0.06	0.08
18	->	45	-0.01	0.00
18	->	46	-0.01	0.01
18	->	47	-0.01	0.00
18	->	48	-0.00	0.00
18	->	49	-0.00	0.00
18	->	50	-0.00	0.00
18	->	51	-0.00	0.00
18	->	52	-0.00	0.00
18	->	53	-0.00	0.00
18	->	54	-0.00	0.00
18	->	55	0.00	0.00
18	->	56	-0.00	0.00
18	->	57	-0.00	0.00
18	->	58	0.00	0.00
18	->	59	-0.00	0.00
18	->	60	0.00	0.00
18	->	61	-0.00	0.00
18	->	62	0.00	0.00
18	->	63	0.00	0.00
18	->	64	0.00	0.00
18	->	65	0.00	0.00
18	->	66	0.00	0.00
18	->	67	0.00	0.00
18	->	68	-0.00	0.00
18	->	69	-0.00	0.00
18	->	70	0.00	0.00
18	->	71	0.00	0.00
18	->	72	-0.00	0.00
18	->	73	0.00	0.00
18	->	74	0.00	0.00
18	->	75	0.00	0.00
18	->	76	-0.00	0.00
18	->	77	0.00	0.00

18	->	78	-0.00	0.00
18	->	79	-0.00	0.00
18	->	80	0.00	0.00
18	->	81	-0.00	0.00
18	->	82	0.00	0.00
18	->	83	0.00	0.00
18	->	84	0.00	0.00
18	->	85	-0.00	0.00
18	->	86	0.00	0.00
18	->	87	0.00	0.00
18	->	88	0.00	0.00
18	->	89	0.00	0.00
18	->	90	0.00	0.00
18	->	91	-0.03	0.01
18	->	92	-0.10	0.05
18	->	93	-0.25	0.16
18	->	94	-2.57	1.26
18	->	95	-0.35	0.30
18	->	96	-0.12	0.07
18	->	97	-0.08	0.06
18	->	98	-1.67	0.24
18	->	99	-1.06	0.35
18	->	100	-0.16	0.18
18	->	101	-0.02	0.01
18	->	102	-0.01	0.00
18	->	103	-0.00	0.00
18	->	104	-0.00	0.00
18	->	105	0.00	0.00
18	->	106	0.00	0.00
18	->	107	0.00	0.00
19	->	1	-3.01	4.51
19	->	2	-0.02	0.01
19	->	3	-0.06	0.04
19	->	4	-0.00	0.01
19	->	5	0.02	0.02
19	->	6	-0.01	0.01
19	->	7	0.00	0.00
19	->	8	0.02	0.01
19	->	9	-0.00	0.00
19	->	10	0.04	0.01
19	->	11	0.03	0.00
19	->	12	-0.12	0.10
19	->	13	-0.21	0.22
19	->	14	-2.57	1.13
19	->	15	-1.47	2.19
19	->	16	-13.65	3.27
19	->	17	-0.65	0.18
19	->	18	-14.62	0.57
19	->	19	-6.58	4.54
19	->	20	-7.95	0.41

19 ->	21	-1.27	0.26
19 ->	22	-9.12	3.23
19 ->	23	-3.06	0.55
19 ->	24	-0.14	0.06
19 ->	25	-0.14	0.04
19 ->	26	-0.06	0.03
19 ->	27	-0.01	0.01
19 ->	28	-0.01	0.00
19 ->	29	-0.00	0.00
19 ->	30	0.00	0.00
19 ->	31	-0.03	0.01
19 ->	32	-0.00	0.00
19 ->	33	-0.00	0.00
19 ->	34	-0.00	0.00
19 ->	35	0.01	0.00
19 ->	36	-0.01	0.00
19 ->	37	-0.00	0.00
19 ->	38	0.01	0.00
19 ->	39	0.00	0.00
19 ->	40	-0.01	0.00
19 ->	41	-0.00	0.00
19 ->	42	-0.00	0.00
19 ->	43	-0.01	0.01
19 ->	44	-0.26	0.12
19 ->	45	0.00	0.00
19 ->	46	0.05	0.01
19 ->	47	-0.00	0.00
19 ->	48	-0.00	0.00
19 ->	49	-0.00	0.00
19 ->	50	-0.00	0.00
19 ->	51	-0.00	0.00
19 ->	52	-0.00	0.00
19 ->	53	-0.01	0.00
19 ->	54	-0.00	0.00
19 ->	55	-0.00	0.00
19 ->	56	-0.00	0.00
19 ->	57	-0.00	0.00
19 ->	58	0.00	0.00
19 ->	59	-0.01	0.00
19 ->	60	-0.00	0.00
19 ->	61	-0.00	0.00
19 ->	62	0.00	0.00
19 ->	63	0.00	0.00
19 ->	64	0.00	0.00
19 ->	65	0.00	0.00
19 ->	66	0.00	0.00
19 ->	67	0.00	0.00
19 ->	68	-0.00	0.00
19 ->	69	-0.00	0.00
19 ->	70	-0.00	0.00

19	->	71	0.00	0.00
19	->	72	-0.02	0.00
19	->	73	-0.00	0.00
19	->	74	0.02	0.00
19	->	75	0.01	0.00
19	->	76	-0.01	0.00
19	->	77	-0.02	0.00
19	->	78	-0.01	0.00
19	->	79	-0.00	0.00
19	->	80	-0.00	0.00
19	->	81	-0.01	0.00
19	->	82	-0.00	0.00
19	->	83	-0.00	0.00
19	->	84	-0.00	0.00
19	->	85	-0.00	0.00
19	->	86	0.00	0.00
19	->	87	0.01	0.00
19	->	88	0.01	0.00
19	->	89	0.01	0.00
19	->	90	0.02	0.00
19	->	91	0.21	0.17
19	->	92	0.32	0.43
19	->	93	-0.24	1.29
19	->	94	-2.76	1.18
19	->	95	-0.15	0.07
19	->	96	0.01	0.01
19	->	97	-0.00	0.01
19	->	98	0.05	0.04
19	->	99	0.14	0.04
19	->	100	-0.09	0.08
19	->	101	0.02	0.01
19	->	102	0.04	0.01
19	->	103	0.03	0.01
19	->	104	0.02	0.00
19	->	105	0.02	0.00
19	->	106	0.01	0.00
19	->	107	0.01	0.00
20	->	1	-0.07	0.06
20	->	2	-0.03	0.01
20	->	3	-1.65	0.45
20	->	4	-0.67	0.36
20	->	5	-0.63	0.35
20	->	6	-0.42	0.45
20	->	7	-0.02	0.01
20	->	8	-0.00	0.00
20	->	9	-0.00	0.00
20	->	10	-0.00	0.01
20	->	11	-0.01	0.01
20	->	12	-0.05	0.02
20	->	13	-0.07	0.02

20 ->	14	-2.26	0.59
20 ->	15	-0.21	0.29
20 ->	16	-1.97	0.63
20 ->	17	-2.15	0.36
20 ->	18	-0.82	0.16
20 ->	19	-7.99	0.41
20 ->	20	21.58	1.33
20 ->	21	-6.20	0.67
20 ->	22	-1.73	0.27
20 ->	23	-2.54	0.38
20 ->	24	-4.17	0.63
20 ->	25	-0.21	0.13
20 ->	26	-0.09	0.03
20 ->	27	-0.06	0.03
20 ->	28	-0.02	0.01
20 ->	29	-0.00	0.00
20 ->	30	-0.01	0.00
20 ->	31	-0.00	0.00
20 ->	32	-0.00	0.00
20 ->	33	-0.00	0.00
20 ->	34	-0.00	0.00
20 ->	35	0.00	0.00
20 ->	36	-0.00	0.00
20 ->	37	-0.00	0.00
20 ->	38	0.00	0.00
20 ->	39	0.00	0.00
20 ->	40	-0.00	0.00
20 ->	41	0.00	0.00
20 ->	42	0.00	0.00
20 ->	43	-0.01	0.01
20 ->	44	-0.01	0.01
20 ->	45	-0.00	0.00
20 ->	46	-0.00	0.00
20 ->	47	-0.00	0.00
20 ->	48	-0.00	0.00
20 ->	49	-0.00	0.00
20 ->	50	-0.00	0.00
20 ->	51	-0.00	0.00
20 ->	52	-0.00	0.00
20 ->	53	-0.00	0.00
20 ->	54	-0.00	0.00
20 ->	55	0.00	0.00
20 ->	56	-0.00	0.00
20 ->	57	0.00	0.00
20 ->	58	0.00	0.00
20 ->	59	-0.00	0.00
20 ->	60	0.00	0.00
20 ->	61	0.00	0.00
20 ->	62	0.00	0.00
20 ->	63	0.00	0.00

20	->	64	0.00	0.00
20	->	65	0.00	0.00
20	->	66	0.00	0.00
20	->	67	0.00	0.00
20	->	68	-0.00	0.00
20	->	69	-0.00	0.00
20	->	70	0.00	0.00
20	->	71	0.00	0.00
20	->	72	-0.00	0.00
20	->	73	0.00	0.00
20	->	74	0.00	0.00
20	->	75	0.00	0.00
20	->	76	-0.00	0.00
20	->	77	-0.00	0.00
20	->	78	-0.00	0.00
20	->	79	0.00	0.00
20	->	80	0.00	0.00
20	->	81	-0.00	0.00
20	->	82	0.00	0.00
20	->	83	0.00	0.00
20	->	84	0.00	0.00
20	->	85	0.00	0.00
20	->	86	0.00	0.00
20	->	87	0.00	0.00
20	->	88	0.00	0.00
20	->	89	-0.00	0.00
20	->	90	0.00	0.00
20	->	91	0.02	0.02
20	->	92	0.10	0.09
20	->	93	0.06	0.09
20	->	94	-0.07	0.03
20	->	95	-0.01	0.00
20	->	96	-0.01	0.00
20	->	97	-0.03	0.03
20	->	98	-0.11	0.05
20	->	99	-0.03	0.01
20	->	100	-0.02	0.01
20	->	101	-0.00	0.00
20	->	102	-0.00	0.00
20	->	103	-0.00	0.00
20	->	104	-0.00	0.00
20	->	105	0.00	0.00
20	->	106	0.00	0.00
20	->	107	0.00	0.00
21	->	1	0.01	0.01
21	->	2	-0.00	0.00
21	->	3	-0.03	0.01
21	->	4	-0.01	0.00
21	->	5	-0.01	0.01
21	->	6	-0.02	0.01

21	->	7	-0.01	0.01
21	->	8	0.00	0.00
21	->	9	-0.00	0.00
21	->	10	-0.00	0.00
21	->	11	-0.00	0.00
21	->	12	-0.00	0.00
21	->	13	-0.01	0.01
21	->	14	-0.06	0.02
21	->	15	-0.00	0.01
21	->	16	-0.10	0.08
21	->	17	-4.24	1.05
21	->	18	-1.73	0.54
21	->	19	-1.28	0.26
21	->	20	-6.21	0.67
21	->	21	-19.22	1.98
21	->	22	-10.35	0.51
21	->	23	-0.66	0.18
21	->	24	-2.05	0.64
21	->	25	-3.17	0.75
21	->	26	-0.10	0.08
21	->	27	-0.04	0.02
21	->	28	-0.02	0.01
21	->	29	-0.00	0.00
21	->	30	-0.04	0.03
21	->	31	-0.00	0.00
21	->	32	-0.02	0.01
21	->	33	-0.02	0.01
21	->	34	-0.00	0.00
21	->	35	0.00	0.00
21	->	36	-0.00	0.00
21	->	37	-0.00	0.00
21	->	38	0.00	0.00
21	->	39	0.00	0.00
21	->	40	-0.00	0.00
21	->	41	0.00	0.00
21	->	42	0.00	0.00
21	->	43	-0.06	0.04
21	->	44	0.08	0.04
21	->	45	-0.02	0.01
21	->	46	-0.02	0.01
21	->	47	-0.01	0.01
21	->	48	-0.00	0.00
21	->	49	-0.00	0.00
21	->	50	-0.00	0.00
21	->	51	-0.00	0.00
21	->	52	-0.00	0.00
21	->	53	0.00	0.00
21	->	54	-0.00	0.00
21	->	55	0.00	0.00
21	->	56	-0.00	0.00

21	->	57	0.00	0.00
21	->	58	-0.00	0.00
21	->	59	0.00	0.00
21	->	60	0.00	0.00
21	->	61	0.00	0.00
21	->	62	-0.00	0.00
21	->	63	-0.00	0.00
21	->	64	-0.00	0.00
21	->	65	0.00	0.00
21	->	66	-0.00	0.00
21	->	67	0.00	0.00
21	->	68	0.00	0.00
21	->	69	0.00	0.00
21	->	70	0.00	0.00
21	->	71	-0.00	0.00
21	->	72	0.00	0.00
21	->	73	0.00	0.00
21	->	74	-0.00	0.00
21	->	75	-0.00	0.00
21	->	76	0.00	0.00
21	->	77	0.00	0.00
21	->	78	0.00	0.00
21	->	79	0.00	0.00
21	->	80	0.00	0.00
21	->	81	0.00	0.00
21	->	82	0.00	0.00
21	->	83	0.00	0.00
21	->	84	0.00	0.00
21	->	85	0.00	0.00
21	->	86	0.00	0.00
21	->	87	0.00	0.00
21	->	88	0.00	0.00
21	->	89	-0.00	0.00
21	->	90	-0.00	0.00
21	->	91	-0.06	0.03
21	->	92	-0.12	0.05
21	->	93	-0.08	0.04
21	->	94	0.04	0.05
21	->	95	-0.00	0.01
21	->	96	-0.01	0.00
21	->	97	-0.09	0.07
21	->	98	-1.79	0.50
21	->	99	-0.76	0.33
21	->	100	-0.10	0.05
21	->	101	-0.00	0.00
21	->	102	-0.00	0.00
21	->	103	-0.00	0.00
21	->	104	-0.00	0.00
21	->	105	-0.00	0.00
21	->	106	-0.00	0.00

21	->	107	0.00	0.00
22	->	1	0.67	0.78
22	->	2	-0.00	0.00
22	->	3	-0.01	0.01
22	->	4	0.01	0.01
22	->	5	-0.28	0.07
22	->	6	-0.00	0.01
22	->	7	0.03	0.02
22	->	8	-0.02	0.01
22	->	9	-0.00	0.00
22	->	10	-0.17	0.05
22	->	11	-0.04	0.01
22	->	12	0.41	0.63
22	->	13	0.11	0.07
22	->	14	-0.85	0.42
22	->	15	-0.01	0.04
22	->	16	0.47	0.27
22	->	17	-0.17	0.10
22	->	18	-1.59	0.46
22	->	19	-9.15	3.23
22	->	20	-1.74	0.27
22	->	21	-10.36	0.50
22	->	22	-90.79	4.71
22	->	23	-19.02	0.63
22	->	24	-0.73	0.23
22	->	25	-2.00	0.54
22	->	26	-2.17	1.03
22	->	27	-0.22	0.06
22	->	28	-0.02	0.02
22	->	29	-0.00	0.01
22	->	30	-0.03	0.01
22	->	31	0.03	0.01
22	->	32	-0.01	0.00
22	->	33	-0.02	0.01
22	->	34	-0.00	0.00
22	->	35	-0.01	0.00
22	->	36	0.01	0.00
22	->	37	0.00	0.00
22	->	38	-0.01	0.00
22	->	39	-0.00	0.00
22	->	40	0.02	0.00
22	->	41	0.01	0.00
22	->	42	0.01	0.01
22	->	43	-0.22	0.21
22	->	44	-0.28	0.53
22	->	45	-0.03	0.01
22	->	46	-0.16	0.07
22	->	47	-0.09	0.06
22	->	48	-0.01	0.00
22	->	49	0.00	0.00

22	->	50	0.00	0.00
22	->	51	0.00	0.00
22	->	52	0.00	0.00
22	->	53	0.01	0.00
22	->	54	0.00	0.00
22	->	55	-0.00	0.00
22	->	56	0.00	0.00
22	->	57	0.00	0.00
22	->	58	-0.01	0.00
22	->	59	0.01	0.00
22	->	60	0.00	0.00
22	->	61	0.00	0.00
22	->	62	-0.00	0.00
22	->	63	-0.00	0.00
22	->	64	-0.00	0.00
22	->	65	-0.00	0.00
22	->	66	-0.00	0.00
22	->	67	-0.00	0.00
22	->	68	0.01	0.00
22	->	69	0.00	0.00
22	->	70	0.00	0.00
22	->	71	-0.00	0.00
22	->	72	0.03	0.00
22	->	73	0.00	0.00
22	->	74	-0.03	0.00
22	->	75	-0.02	0.00
22	->	76	0.01	0.00
22	->	77	0.02	0.00
22	->	78	0.02	0.00
22	->	79	0.00	0.00
22	->	80	0.00	0.00
22	->	81	0.01	0.01
22	->	82	0.00	0.00
22	->	83	0.00	0.00
22	->	84	0.00	0.00
22	->	85	0.00	0.00
22	->	86	-0.00	0.00
22	->	87	-0.01	0.00
22	->	88	-0.02	0.00
22	->	89	-0.02	0.00
22	->	90	-0.11	0.03
22	->	91	-4.71	1.67
22	->	92	-16.17	4.18
22	->	93	-8.48	6.51
22	->	94	-0.45	0.49
22	->	95	-0.03	0.02
22	->	96	-0.02	0.00
22	->	97	-0.02	0.01
22	->	98	-0.14	0.05
22	->	99	-0.32	0.14

22	->	100	-0.35	0.19
22	->	101	0.01	0.04
22	->	102	-0.07	0.04
22	->	103	-0.08	0.02
22	->	104	-0.05	0.01
22	->	105	-0.04	0.01
22	->	106	-0.02	0.00
22	->	107	-0.01	0.00
23	->	1	0.00	0.03
23	->	2	-0.00	0.00
23	->	3	-0.02	0.01
23	->	4	-0.07	0.03
23	->	5	1.77	0.53
23	->	6	-0.19	0.24
23	->	7	-0.34	0.17
23	->	8	0.00	0.01
23	->	9	-0.05	0.04
23	->	10	2.21	0.54
23	->	11	0.05	0.05
23	->	12	-0.26	0.22
23	->	13	-0.05	0.03
23	->	14	-2.20	0.38
23	->	15	-0.01	0.00
23	->	16	-0.02	0.01
23	->	17	-0.04	0.03
23	->	18	-0.02	0.06
23	->	19	-3.08	0.55
23	->	20	-2.55	0.37
23	->	21	-0.67	0.18
23	->	22	-19.06	0.63
23	->	23	11.71	1.54
23	->	24	-10.34	0.55
23	->	25	-1.32	0.25
23	->	26	-3.31	0.69
23	->	27	22.50	10.85
23	->	28	-0.23	0.08
23	->	29	-0.04	0.02
23	->	30	-0.03	0.01
23	->	31	-0.01	0.00
23	->	32	-0.01	0.00
23	->	33	-0.00	0.00
23	->	34	-0.00	0.00
23	->	35	-0.00	0.00
23	->	36	0.00	0.00
23	->	37	0.00	0.00
23	->	38	0.00	0.00
23	->	39	0.00	0.00
23	->	40	-0.00	0.00
23	->	41	-0.00	0.00
23	->	42	-0.01	0.00

23 ->	43	-0.02	0.01
23 ->	44	-0.08	0.04
23 ->	45	0.00	0.00
23 ->	46	0.00	0.00
23 ->	47	-0.00	0.00
23 ->	48	-0.00	0.00
23 ->	49	0.00	0.00
23 ->	50	0.00	0.00
23 ->	51	0.00	0.00
23 ->	52	0.00	0.00
23 ->	53	0.00	0.00
23 ->	54	0.00	0.00
23 ->	55	0.00	0.00
23 ->	56	0.00	0.00
23 ->	57	0.00	0.00
23 ->	58	-0.00	0.00
23 ->	59	0.00	0.00
23 ->	60	0.00	0.00
23 ->	61	0.00	0.00
23 ->	62	-0.00	0.00
23 ->	63	-0.00	0.00
23 ->	64	-0.00	0.00
23 ->	65	0.00	0.00
23 ->	66	-0.00	0.00
23 ->	67	0.00	0.00
23 ->	68	0.00	0.00
23 ->	69	0.00	0.00
23 ->	70	0.00	0.00
23 ->	71	-0.00	0.00
23 ->	72	0.00	0.00
23 ->	73	0.00	0.00
23 ->	74	-0.00	0.00
23 ->	75	-0.00	0.00
23 ->	76	0.00	0.00
23 ->	77	0.00	0.00
23 ->	78	0.00	0.00
23 ->	79	0.00	0.00
23 ->	80	0.00	0.00
23 ->	81	0.00	0.00
23 ->	82	0.00	0.00
23 ->	83	0.00	0.00
23 ->	84	0.00	0.00
23 ->	85	0.00	0.00
23 ->	86	-0.00	0.00
23 ->	87	-0.00	0.00
23 ->	88	-0.00	0.00
23 ->	89	-0.00	0.00
23 ->	90	-0.02	0.01
23 ->	91	-1.02	0.55
23 ->	92	-6.23	3.17

23	->	93	-0.15	0.16
23	->	94	-0.01	0.01
23	->	95	-0.01	0.00
23	->	96	-0.00	0.00
23	->	97	-0.00	0.00
23	->	98	-0.01	0.01
23	->	99	-0.01	0.01
23	->	100	-0.00	0.01
23	->	101	-0.01	0.00
23	->	102	-0.01	0.00
23	->	103	-0.01	0.00
23	->	104	-0.01	0.00
23	->	105	-0.00	0.00
23	->	106	-0.00	0.00
23	->	107	-0.00	0.00
24	->	1	-0.00	0.01
24	->	2	-0.00	0.00
24	->	3	-0.04	0.11
24	->	4	-0.06	0.06
24	->	5	-0.39	0.36
24	->	6	-1.44	0.33
24	->	7	-0.26	0.31
24	->	8	-0.01	0.01
24	->	9	-0.01	0.01
24	->	10	0.03	0.01
24	->	11	-0.01	0.01
24	->	12	-0.02	0.02
24	->	13	-0.01	0.01
24	->	14	-0.07	0.03
24	->	15	-0.00	0.00
24	->	16	-0.01	0.01
24	->	17	-0.05	0.02
24	->	18	-0.01	0.01
24	->	19	-0.14	0.06
24	->	20	-4.16	0.62
24	->	21	-2.07	0.65
24	->	22	-0.73	0.23
24	->	23	-10.39	0.55
24	->	24	23.33	1.85
24	->	25	-10.22	0.59
24	->	26	-1.06	0.19
24	->	27	-2.49	0.49
24	->	28	-5.27	1.05
24	->	29	-0.16	0.11
24	->	30	-0.50	0.28
24	->	31	-0.00	0.01
24	->	32	-0.01	0.00
24	->	33	-0.01	0.00
24	->	34	-0.00	0.00
24	->	35	0.00	0.00

24	->	36	-0.00	0.00
24	->	37	0.00	0.00
24	->	38	0.00	0.00
24	->	39	0.00	0.00
24	->	40	-0.00	0.00
24	->	41	0.00	0.00
24	->	42	0.00	0.00
24	->	43	-0.03	0.02
24	->	44	-0.01	0.01
24	->	45	-0.00	0.00
24	->	46	-0.00	0.00
24	->	47	-0.00	0.00
24	->	48	-0.00	0.00
24	->	49	0.00	0.00
24	->	50	0.00	0.00
24	->	51	0.00	0.00
24	->	52	-0.00	0.00
24	->	53	0.00	0.00
24	->	54	0.00	0.00
24	->	55	0.00	0.00
24	->	56	-0.00	0.00
24	->	57	0.00	0.00
24	->	58	0.00	0.00
24	->	59	-0.00	0.00
24	->	60	0.00	0.00
24	->	61	0.00	0.00
24	->	62	-0.00	0.00
24	->	63	-0.00	0.00
24	->	64	-0.00	0.00
24	->	65	0.00	0.00
24	->	66	-0.00	0.00
24	->	67	0.00	0.00
24	->	68	-0.00	0.00
24	->	69	0.00	0.00
24	->	70	0.00	0.00
24	->	71	0.00	0.00
24	->	72	-0.00	0.00
24	->	73	0.00	0.00
24	->	74	-0.00	0.00
24	->	75	0.00	0.00
24	->	76	0.00	0.00
24	->	77	0.00	0.00
24	->	78	0.00	0.00
24	->	79	0.00	0.00
24	->	80	0.00	0.00
24	->	81	0.00	0.00
24	->	82	0.00	0.00
24	->	83	0.00	0.00
24	->	84	0.00	0.00
24	->	85	0.00	0.00

24 ->	86	0.00	0.00
24 ->	87	0.00	0.00
24 ->	88	0.00	0.00
24 ->	89	-0.00	0.00
24 ->	90	-0.00	0.00
24 ->	91	0.02	0.02
24 ->	92	0.11	0.07
24 ->	93	0.00	0.01
24 ->	94	-0.01	0.00
24 ->	95	-0.00	0.00
24 ->	96	-0.00	0.00
24 ->	97	-0.01	0.01
24 ->	98	-0.05	0.02
24 ->	99	-0.02	0.01
24 ->	100	-0.01	0.00
24 ->	101	-0.00	0.00
24 ->	102	-0.00	0.00
24 ->	103	-0.00	0.00
24 ->	104	-0.00	0.00
24 ->	105	-0.00	0.00
24 ->	106	-0.00	0.00
24 ->	107	0.00	0.00
25 ->	1	0.04	0.01
25 ->	2	-0.00	0.00
25 ->	3	-0.00	0.00
25 ->	4	-0.01	0.00
25 ->	5	-0.06	0.02
25 ->	6	-0.03	0.01
25 ->	7	-0.00	0.01
25 ->	8	-0.01	0.00
25 ->	9	-0.00	0.00
25 ->	10	-0.04	0.01
25 ->	11	-0.02	0.00
25 ->	12	0.02	0.01
25 ->	13	0.01	0.00
25 ->	14	-0.01	0.01
25 ->	15	-0.00	0.00
25 ->	16	0.03	0.00
25 ->	17	-0.02	0.01
25 ->	18	-0.02	0.02
25 ->	19	-0.14	0.04
25 ->	20	-0.21	0.13
25 ->	21	-3.21	0.75
25 ->	22	-2.04	0.53
25 ->	23	-1.33	0.25
25 ->	24	-10.26	0.60
25 ->	25	-99.37	2.52
25 ->	26	-12.63	0.52
25 ->	27	-0.71	0.16
25 ->	28	-1.43	0.40

25	->	29	-1.15	0.47
25	->	30	-4.16	1.09
25	->	31	0.12	0.10
25	->	32	-1.24	0.48
25	->	33	-0.53	0.18
25	->	34	-0.01	0.00
25	->	35	-0.03	0.00
25	->	36	0.02	0.01
25	->	37	0.00	0.00
25	->	38	-0.02	0.00
25	->	39	-0.00	0.00
25	->	40	0.03	0.01
25	->	41	0.05	0.02
25	->	42	0.04	0.06
25	->	43	-5.37	1.11
25	->	44	-0.50	0.23
25	->	45	-0.28	0.07
25	->	46	-0.20	0.06
25	->	47	-0.08	0.05
25	->	48	-0.01	0.01
25	->	49	0.00	0.00
25	->	50	0.00	0.00
25	->	51	0.00	0.00
25	->	52	0.00	0.00
25	->	53	0.02	0.00
25	->	54	0.00	0.00
25	->	55	-0.00	0.00
25	->	56	0.00	0.00
25	->	57	0.00	0.00
25	->	58	-0.01	0.00
25	->	59	0.01	0.00
25	->	60	0.00	0.00
25	->	61	0.00	0.00
25	->	62	-0.00	0.00
25	->	63	-0.00	0.00
25	->	64	-0.00	0.00
25	->	65	-0.00	0.00
25	->	66	-0.00	0.00
25	->	67	-0.00	0.00
25	->	68	0.01	0.00
25	->	69	0.00	0.00
25	->	70	0.00	0.00
25	->	71	-0.00	0.00
25	->	72	0.02	0.00
25	->	73	0.00	0.00
25	->	74	-0.02	0.00
25	->	75	-0.01	0.00
25	->	76	0.01	0.00
25	->	77	0.01	0.00
25	->	78	0.01	0.00

25	->	79	0.00	0.00
25	->	80	0.00	0.00
25	->	81	0.01	0.00
25	->	82	0.00	0.00
25	->	83	0.00	0.00
25	->	84	0.00	0.00
25	->	85	0.00	0.00
25	->	86	-0.00	0.00
25	->	87	-0.01	0.00
25	->	88	-0.02	0.00
25	->	89	-0.03	0.01
25	->	90	-0.09	0.02
25	->	91	-0.32	0.08
25	->	92	-0.28	0.13
25	->	93	-0.07	0.02
25	->	94	-0.01	0.01
25	->	95	-0.02	0.00
25	->	96	-0.02	0.00
25	->	97	-0.05	0.02
25	->	98	-4.27	1.53
25	->	99	-8.56	1.50
25	->	100	-0.22	0.06
25	->	101	-0.02	0.01
25	->	102	-0.02	0.00
25	->	103	-0.01	0.00
25	->	104	-0.01	0.00
25	->	105	-0.01	0.00
25	->	106	-0.01	0.00
25	->	107	-0.01	0.00
26	->	1	0.00	0.00
26	->	2	-0.00	0.00
26	->	3	-0.00	0.00
26	->	4	-0.00	0.00
26	->	5	-0.09	0.03
26	->	6	-0.01	0.01
26	->	7	0.01	0.01
26	->	8	-0.01	0.00
26	->	9	-0.01	0.02
26	->	10	-0.08	0.03
26	->	11	-0.01	0.01
26	->	12	-0.01	0.02
26	->	13	0.00	0.00
26	->	14	-0.03	0.01
26	->	15	-0.00	0.00
26	->	16	0.00	0.00
26	->	17	-0.00	0.00
26	->	18	-0.00	0.00
26	->	19	-0.06	0.03
26	->	20	-0.09	0.03
26	->	21	-0.10	0.08

26	->	22	-2.17	1.02
26	->	23	-3.33	0.69
26	->	24	-1.07	0.19
26	->	25	-12.69	0.52
26	->	26	20.71	1.61
26	->	27	-21.59	0.40
26	->	28	-0.66	0.21
26	->	29	-1.29	0.43
26	->	30	-0.28	0.16
26	->	31	-0.18	0.11
26	->	32	-0.02	0.01
26	->	33	-0.00	0.00
26	->	34	-0.00	0.00
26	->	35	0.00	0.00
26	->	36	-0.00	0.00
26	->	37	0.00	0.00
26	->	38	0.00	0.00
26	->	39	-0.00	0.00
26	->	40	-0.01	0.00
26	->	41	-0.02	0.00
26	->	42	-0.02	0.01
26	->	43	-0.72	0.39
26	->	44	-0.09	0.08
26	->	45	0.00	0.00
26	->	46	-0.00	0.00
26	->	47	-0.03	0.03
26	->	48	-0.00	0.00
26	->	49	-0.00	0.00
26	->	50	-0.00	0.00
26	->	51	-0.00	0.00
26	->	52	0.00	0.00
26	->	53	0.00	0.00
26	->	54	0.00	0.00
26	->	55	0.00	0.00
26	->	56	0.00	0.00
26	->	57	0.00	0.00
26	->	58	-0.00	0.00
26	->	59	0.00	0.00
26	->	60	0.00	0.00
26	->	61	0.00	0.00
26	->	62	0.00	0.00
26	->	63	-0.00	0.00
26	->	64	-0.00	0.00
26	->	65	0.00	0.00
26	->	66	-0.00	0.00
26	->	67	0.00	0.00
26	->	68	0.00	0.00
26	->	69	0.00	0.00
26	->	70	0.00	0.00
26	->	71	-0.00	0.00

26	->	72	-0.00	0.00
26	->	73	0.00	0.00
26	->	74	-0.00	0.00
26	->	75	-0.00	0.00
26	->	76	0.00	0.00
26	->	77	0.00	0.00
26	->	78	0.00	0.00
26	->	79	0.00	0.00
26	->	80	0.00	0.00
26	->	81	0.00	0.00
26	->	82	0.00	0.00
26	->	83	0.00	0.00
26	->	84	0.00	0.00
26	->	85	0.00	0.00
26	->	86	0.00	0.00
26	->	87	-0.00	0.00
26	->	88	-0.00	0.00
26	->	89	-0.01	0.01
26	->	90	-0.18	0.12
26	->	91	-2.94	0.85
26	->	92	-1.08	0.44
26	->	93	-0.03	0.01
26	->	94	-0.01	0.00
26	->	95	-0.00	0.00
26	->	96	-0.00	0.00
26	->	97	-0.00	0.00
26	->	98	-0.00	0.00
26	->	99	-0.01	0.00
26	->	100	-0.01	0.00
26	->	101	-0.00	0.00
26	->	102	-0.00	0.00
26	->	103	-0.00	0.00
26	->	104	-0.01	0.00
26	->	105	-0.00	0.00
26	->	106	-0.00	0.00
26	->	107	-0.00	0.00
27	->	1	-0.01	0.00
27	->	2	-0.00	0.00
27	->	3	-0.01	0.01
27	->	4	-0.08	0.03
27	->	5	2.92	0.70
27	->	6	-0.53	0.38
27	->	7	-2.45	0.55
27	->	8	-0.03	0.07
27	->	9	-0.43	0.56
27	->	10	3.30	0.55
27	->	11	0.16	0.11
27	->	12	-0.19	0.18
27	->	13	-0.03	0.01
27	->	14	-0.02	0.02

27 ->	15	0.00	0.00
27 ->	16	-0.00	0.00
27 ->	17	-0.00	0.00
27 ->	18	-0.01	0.00
27 ->	19	-0.01	0.01
27 ->	20	-0.06	0.03
27 ->	21	-0.04	0.02
27 ->	22	-0.22	0.06
27 ->	23	22.47	10.85
27 ->	24	-2.49	0.48
27 ->	25	-0.71	0.16
27 ->	26	-21.67	0.40
27 ->	27	5.54	1.11
27 ->	28	-5.00	0.47
27 ->	29	-0.50	0.14
27 ->	30	-0.03	0.03
27 ->	31	-0.03	0.02
27 ->	32	-0.00	0.00
27 ->	33	-0.00	0.00
27 ->	34	-0.00	0.00
27 ->	35	-0.00	0.00
27 ->	36	0.00	0.00
27 ->	37	0.00	0.00
27 ->	38	-0.00	0.00
27 ->	39	-0.00	0.00
27 ->	40	-0.00	0.00
27 ->	41	-0.00	0.00
27 ->	42	-0.01	0.00
27 ->	43	-0.00	0.01
27 ->	44	-0.00	0.00
27 ->	45	0.00	0.00
27 ->	46	-0.00	0.00
27 ->	47	-0.00	0.00
27 ->	48	0.00	0.00
27 ->	49	0.00	0.00
27 ->	50	0.00	0.00
27 ->	51	0.00	0.00
27 ->	52	0.00	0.00
27 ->	53	0.00	0.00
27 ->	54	0.00	0.00
27 ->	55	0.00	0.00
27 ->	56	0.00	0.00
27 ->	57	0.00	0.00
27 ->	58	-0.00	0.00
27 ->	59	0.00	0.00
27 ->	60	0.00	0.00
27 ->	61	0.00	0.00
27 ->	62	0.00	0.00
27 ->	63	-0.00	0.00
27 ->	64	-0.00	0.00

27 ->	65	0.00	0.00
27 ->	66	-0.00	0.00
27 ->	67	0.00	0.00
27 ->	68	0.00	0.00
27 ->	69	0.00	0.00
27 ->	70	0.00	0.00
27 ->	71	0.00	0.00
27 ->	72	0.00	0.00
27 ->	73	0.00	0.00
27 ->	74	-0.00	0.00
27 ->	75	-0.00	0.00
27 ->	76	0.00	0.00
27 ->	77	0.00	0.00
27 ->	78	0.00	0.00
27 ->	79	0.00	0.00
27 ->	80	0.00	0.00
27 ->	81	0.00	0.00
27 ->	82	0.00	0.00
27 ->	83	0.00	0.00
27 ->	84	0.00	0.00
27 ->	85	0.00	0.00
27 ->	86	-0.00	0.00
27 ->	87	-0.00	0.00
27 ->	88	-0.00	0.00
27 ->	89	-0.00	0.00
27 ->	90	0.00	0.01
27 ->	91	0.07	0.05
27 ->	92	0.20	0.14
27 ->	93	0.01	0.01
27 ->	94	-0.00	0.00
27 ->	95	-0.00	0.00
27 ->	96	-0.00	0.00
27 ->	97	-0.00	0.00
27 ->	98	-0.00	0.00
27 ->	99	-0.00	0.00
27 ->	100	-0.00	0.00
27 ->	101	-0.00	0.00
27 ->	102	-0.00	0.00
27 ->	103	-0.00	0.00
27 ->	104	-0.00	0.00
27 ->	105	-0.00	0.00
27 ->	106	-0.00	0.00
27 ->	107	-0.00	0.00
28 ->	1	0.00	0.00
28 ->	2	-0.00	0.00
28 ->	3	-0.00	0.00
28 ->	4	-0.00	0.00
28 ->	5	-0.02	0.01
28 ->	6	-0.19	0.22
28 ->	7	-0.15	0.15

28	->	8	-0.01	0.00
28	->	9	-0.01	0.01
28	->	10	-0.01	0.00
28	->	11	-0.00	0.00
28	->	12	0.00	0.00
28	->	13	0.00	0.00
28	->	14	-0.00	0.00
28	->	15	-0.00	0.00
28	->	16	0.00	0.00
28	->	17	-0.00	0.00
28	->	18	-0.00	0.00
28	->	19	-0.01	0.00
28	->	20	-0.02	0.01
28	->	21	-0.02	0.01
28	->	22	-0.02	0.02
28	->	23	-0.23	0.08
28	->	24	-5.27	1.05
28	->	25	-1.44	0.40
28	->	26	-0.68	0.21
28	->	27	-5.16	0.47
28	->	28	-22.65	1.65
28	->	29	-11.58	0.47
28	->	30	-3.02	1.35
28	->	31	-0.05	0.02
28	->	32	-0.02	0.00
28	->	33	-0.00	0.00
28	->	34	-0.00	0.00
28	->	35	0.00	0.00
28	->	36	-0.00	0.00
28	->	37	0.00	0.00
28	->	38	0.00	0.00
28	->	39	-0.00	0.00
28	->	40	-0.00	0.00
28	->	41	-0.00	0.00
28	->	42	-0.00	0.00
28	->	43	-0.02	0.01
28	->	44	-0.00	0.00
28	->	45	-0.00	0.00
28	->	46	-0.00	0.00
28	->	47	-0.00	0.00
28	->	48	-0.00	0.00
28	->	49	0.00	0.00
28	->	50	0.00	0.00
28	->	51	0.00	0.00
28	->	52	0.00	0.00
28	->	53	0.00	0.00
28	->	54	-0.00	0.00
28	->	55	0.00	0.00
28	->	56	0.00	0.00
28	->	57	0.00	0.00

28	->	58	-0.00	0.00
28	->	59	0.00	0.00
28	->	60	0.00	0.00
28	->	61	0.00	0.00
28	->	62	0.00	0.00
28	->	63	-0.00	0.00
28	->	64	-0.00	0.00
28	->	65	0.00	0.00
28	->	66	-0.00	0.00
28	->	67	0.00	0.00
28	->	68	0.00	0.00
28	->	69	0.00	0.00
28	->	70	0.00	0.00
28	->	71	0.00	0.00
28	->	72	0.00	0.00
28	->	73	0.00	0.00
28	->	74	-0.00	0.00
28	->	75	-0.00	0.00
28	->	76	0.00	0.00
28	->	77	0.00	0.00
28	->	78	0.00	0.00
28	->	79	0.00	0.00
28	->	80	0.00	0.00
28	->	81	0.00	0.00
28	->	82	0.00	0.00
28	->	83	0.00	0.00
28	->	84	0.00	0.00
28	->	85	0.00	0.00
28	->	86	-0.00	0.00
28	->	87	-0.00	0.00
28	->	88	-0.00	0.00
28	->	89	-0.00	0.00
28	->	90	-0.01	0.00
28	->	91	-0.02	0.01
28	->	92	-0.02	0.01
28	->	93	-0.00	0.00
28	->	94	-0.00	0.00
28	->	95	-0.00	0.00
28	->	96	-0.00	0.00
28	->	97	-0.00	0.00
28	->	98	-0.00	0.00
28	->	99	-0.00	0.00
28	->	100	-0.00	0.00
28	->	101	-0.00	0.00
28	->	102	-0.00	0.00
28	->	103	-0.00	0.00
28	->	104	-0.00	0.00
28	->	105	-0.00	0.00
28	->	106	-0.00	0.00
28	->	107	-0.00	0.00

29	->	1	0.00	0.00
29	->	2	0.00	0.00
29	->	3	-0.00	0.00
29	->	4	-0.00	0.00
29	->	5	-0.01	0.00
29	->	6	-0.00	0.00
29	->	7	-0.01	0.00
29	->	8	-0.00	0.00
29	->	9	-0.00	0.00
29	->	10	-0.01	0.00
29	->	11	-0.00	0.00
29	->	12	0.00	0.00
29	->	13	0.00	0.00
29	->	14	-0.00	0.00
29	->	15	0.00	0.00
29	->	16	0.00	0.00
29	->	17	-0.00	0.00
29	->	18	-0.00	0.00
29	->	19	-0.00	0.00
29	->	20	-0.00	0.00
29	->	21	-0.00	0.00
29	->	22	-0.00	0.01
29	->	23	-0.04	0.02
29	->	24	-0.16	0.11
29	->	25	-1.16	0.47
29	->	26	-1.34	0.44
29	->	27	-0.51	0.14
29	->	28	-11.64	0.47
29	->	29	12.24	0.57
29	->	30	-14.42	0.49
29	->	31	-1.07	0.34
29	->	32	-0.03	0.00
29	->	33	-0.01	0.00
29	->	34	-0.00	0.00
29	->	35	0.00	0.00
29	->	36	-0.00	0.00
29	->	37	0.00	0.00
29	->	38	0.00	0.00
29	->	39	-0.00	0.00
29	->	40	-0.01	0.01
29	->	41	-0.00	0.01
29	->	42	-0.00	0.01
29	->	43	-0.04	0.04
29	->	44	-0.01	0.00
29	->	45	-0.00	0.00
29	->	46	-0.00	0.00
29	->	47	-0.00	0.00
29	->	48	-0.00	0.00
29	->	49	0.00	0.00
29	->	50	0.00	0.00

29	->	51	-0.00	0.00
29	->	52	-0.00	0.00
29	->	53	0.00	0.00
29	->	54	0.00	0.00
29	->	55	0.00	0.00
29	->	56	-0.00	0.00
29	->	57	-0.00	0.00
29	->	58	0.00	0.00
29	->	59	0.00	0.00
29	->	60	0.00	0.00
29	->	61	0.00	0.00
29	->	62	0.00	0.00
29	->	63	-0.00	0.00
29	->	64	-0.00	0.00
29	->	65	0.00	0.00
29	->	66	-0.00	0.00
29	->	67	0.00	0.00
29	->	68	0.00	0.00
29	->	69	-0.00	0.00
29	->	70	0.00	0.00
29	->	71	0.00	0.00
29	->	72	0.00	0.00
29	->	73	0.00	0.00
29	->	74	-0.00	0.00
29	->	75	-0.00	0.00
29	->	76	0.00	0.00
29	->	77	0.00	0.00
29	->	78	0.00	0.00
29	->	79	0.00	0.00
29	->	80	0.00	0.00
29	->	81	0.00	0.00
29	->	82	0.00	0.00
29	->	83	0.00	0.00
29	->	84	0.00	0.00
29	->	85	0.00	0.00
29	->	86	-0.00	0.00
29	->	87	-0.00	0.00
29	->	88	-0.00	0.00
29	->	89	-0.00	0.00
29	->	90	-0.01	0.01
29	->	91	-0.03	0.02
29	->	92	-0.01	0.00
29	->	93	-0.00	0.00
29	->	94	-0.00	0.00
29	->	95	-0.00	0.00
29	->	96	-0.00	0.00
29	->	97	-0.00	0.00
29	->	98	-0.00	0.00
29	->	99	-0.00	0.00
29	->	100	-0.00	0.00

29 ->	101	-0.00	0.00
29 ->	102	-0.00	0.00
29 ->	103	-0.00	0.00
29 ->	104	-0.00	0.00
29 ->	105	-0.00	0.00
29 ->	106	-0.00	0.00
29 ->	107	-0.00	0.00
30 ->	1	-0.00	0.00
30 ->	2	-0.00	0.00
30 ->	3	-0.00	0.00
30 ->	4	-0.00	0.00
30 ->	5	-0.00	0.00
30 ->	6	-0.01	0.01
30 ->	7	-0.00	0.00
30 ->	8	-0.00	0.00
30 ->	9	-0.00	0.00
30 ->	10	-0.00	0.00
30 ->	11	-0.00	0.00
30 ->	12	-0.00	0.00
30 ->	13	-0.00	0.00
30 ->	14	-0.00	0.00
30 ->	15	0.00	0.00
30 ->	16	-0.00	0.00
30 ->	17	-0.00	0.00
30 ->	18	-0.00	0.00
30 ->	19	0.00	0.00
30 ->	20	-0.01	0.00
30 ->	21	-0.04	0.03
30 ->	22	-0.03	0.01
30 ->	23	-0.03	0.01
30 ->	24	-0.50	0.28
30 ->	25	-4.14	1.07
30 ->	26	-0.29	0.16
30 ->	27	-0.03	0.03
30 ->	28	-3.04	1.35
30 ->	29	-14.58	0.49
30 ->	30	-53.21	2.67
30 ->	31	-11.32	0.75
30 ->	32	-1.41	0.44
30 ->	33	-0.09	0.02
30 ->	34	-0.01	0.00
30 ->	35	-0.00	0.00
30 ->	36	-0.00	0.00
30 ->	37	-0.00	0.00
30 ->	38	0.00	0.00
30 ->	39	-0.00	0.00
30 ->	40	0.00	0.00
30 ->	41	0.03	0.03
30 ->	42	-0.25	0.07
30 ->	43	-1.00	0.97

30 ->	44	0.00	0.01
30 ->	45	-0.01	0.00
30 ->	46	0.00	0.00
30 ->	47	-0.01	0.00
30 ->	48	-0.01	0.00
30 ->	49	-0.00	0.00
30 ->	50	-0.00	0.00
30 ->	51	-0.00	0.00
30 ->	52	-0.00	0.00
30 ->	53	-0.00	0.00
30 ->	54	-0.00	0.00
30 ->	55	-0.00	0.00
30 ->	56	0.00	0.00
30 ->	57	-0.00	0.00
30 ->	58	0.00	0.00
30 ->	59	-0.00	0.00
30 ->	60	-0.00	0.00
30 ->	61	-0.00	0.00
30 ->	62	0.00	0.00
30 ->	63	0.00	0.00
30 ->	64	0.00	0.00
30 ->	65	0.00	0.00
30 ->	66	0.00	0.00
30 ->	67	0.00	0.00
30 ->	68	-0.00	0.00
30 ->	69	-0.00	0.00
30 ->	70	-0.00	0.00
30 ->	71	0.00	0.00
30 ->	72	-0.00	0.00
30 ->	73	-0.00	0.00
30 ->	74	0.00	0.00
30 ->	75	0.00	0.00
30 ->	76	-0.00	0.00
30 ->	77	-0.00	0.00
30 ->	78	-0.00	0.00
30 ->	79	-0.00	0.00
30 ->	80	-0.00	0.00
30 ->	81	-0.00	0.00
30 ->	82	0.00	0.00
30 ->	83	0.00	0.00
30 ->	84	0.00	0.00
30 ->	85	0.00	0.00
30 ->	86	0.00	0.00
30 ->	87	0.00	0.00
30 ->	88	0.00	0.00
30 ->	89	-0.00	0.00
30 ->	90	-0.00	0.00
30 ->	91	-0.01	0.01
30 ->	92	-0.00	0.00
30 ->	93	0.00	0.00

30	->	94	-0.00	0.00
30	->	95	0.00	0.00
30	->	96	0.00	0.00
30	->	97	-0.00	0.00
30	->	98	-0.01	0.01
30	->	99	-0.01	0.01
30	->	100	0.00	0.00
30	->	101	0.00	0.00
30	->	102	0.00	0.00
30	->	103	0.00	0.00
30	->	104	0.00	0.00
30	->	105	0.00	0.00
30	->	106	0.00	0.00
30	->	107	0.00	0.00
31	->	1	0.02	0.00
31	->	2	-0.00	0.00
31	->	3	-0.00	0.00
31	->	4	-0.00	0.00
31	->	5	-0.02	0.00
31	->	6	-0.00	0.00
31	->	7	-0.00	0.00
31	->	8	-0.01	0.00
31	->	9	0.00	0.00
31	->	10	-0.03	0.00
31	->	11	-0.01	0.00
31	->	12	0.02	0.01
31	->	13	0.01	0.00
31	->	14	-0.00	0.00
31	->	15	0.00	0.00
31	->	16	0.01	0.00
31	->	17	-0.00	0.00
31	->	18	-0.00	0.00
31	->	19	-0.03	0.01
31	->	20	-0.00	0.00
31	->	21	-0.00	0.00
31	->	22	0.03	0.01
31	->	23	-0.01	0.00
31	->	24	-0.00	0.01
31	->	25	0.12	0.10
31	->	26	-0.18	0.11
31	->	27	-0.03	0.02
31	->	28	-0.05	0.02
31	->	29	-1.13	0.36
31	->	30	-11.37	0.77
31	->	31	11.04	2.21
31	->	32	-0.17	0.41
31	->	33	-0.86	0.26
31	->	34	-0.25	0.27
31	->	35	-0.10	0.02
31	->	36	0.01	0.00

31	->	37	0.00	0.00
31	->	38	-0.05	0.01
31	->	39	-0.02	0.01
31	->	40	0.13	0.38
31	->	41	-2.22	0.77
31	->	42	-3.42	0.45
31	->	43	-2.41	1.17
31	->	44	-0.07	0.04
31	->	45	-0.08	0.02
31	->	46	-0.08	0.02
31	->	47	-0.03	0.04
31	->	48	-0.04	0.01
31	->	49	-0.00	0.00
31	->	50	-0.00	0.00
31	->	51	-0.00	0.00
31	->	52	-0.00	0.00
31	->	53	0.02	0.00
31	->	54	0.00	0.00
31	->	55	-0.01	0.00
31	->	56	0.00	0.00
31	->	57	0.00	0.00
31	->	58	-0.01	0.00
31	->	59	0.01	0.00
31	->	60	0.00	0.00
31	->	61	-0.00	0.00
31	->	62	-0.00	0.00
31	->	63	-0.00	0.00
31	->	64	-0.00	0.00
31	->	65	-0.00	0.00
31	->	66	-0.00	0.00
31	->	67	-0.00	0.00
31	->	68	0.01	0.00
31	->	69	0.01	0.00
31	->	70	0.00	0.00
31	->	71	0.00	0.00
31	->	72	0.03	0.00
31	->	73	0.00	0.00
31	->	74	-0.02	0.00
31	->	75	-0.02	0.00
31	->	76	0.01	0.00
31	->	77	0.01	0.00
31	->	78	0.02	0.00
31	->	79	0.00	0.00
31	->	80	0.00	0.00
31	->	81	0.01	0.00
31	->	82	0.00	0.00
31	->	83	0.00	0.00
31	->	84	0.00	0.00
31	->	85	0.00	0.00
31	->	86	-0.00	0.00

31	->	87	-0.01	0.00
31	->	88	-0.05	0.02
31	->	89	-0.24	0.24
31	->	90	-1.30	1.62
31	->	91	-0.33	0.24
31	->	92	-0.06	0.01
31	->	93	-0.03	0.00
31	->	94	-0.01	0.00
31	->	95	-0.01	0.00
31	->	96	-0.01	0.00
31	->	97	-0.01	0.00
31	->	98	-0.07	0.02
31	->	99	-0.11	0.03
31	->	100	-0.04	0.00
31	->	101	-0.01	0.00
31	->	102	-0.01	0.00
31	->	103	-0.01	0.00
31	->	104	-0.01	0.00
31	->	105	-0.02	0.00
31	->	106	-0.01	0.00
31	->	107	-0.02	0.00
32	->	1	0.00	0.00
32	->	2	-0.00	0.00
32	->	3	-0.00	0.00
32	->	4	-0.00	0.00
32	->	5	-0.00	0.00
32	->	6	-0.00	0.00
32	->	7	-0.00	0.00
32	->	8	-0.00	0.00
32	->	9	0.00	0.00
32	->	10	-0.00	0.00
32	->	11	-0.00	0.00
32	->	12	0.00	0.00
32	->	13	0.00	0.00
32	->	14	-0.00	0.00
32	->	15	0.00	0.00
32	->	16	0.00	0.00
32	->	17	-0.00	0.00
32	->	18	-0.00	0.00
32	->	19	-0.00	0.00
32	->	20	-0.00	0.00
32	->	21	-0.02	0.01
32	->	22	-0.01	0.00
32	->	23	-0.01	0.00
32	->	24	-0.01	0.00
32	->	25	-1.22	0.47
32	->	26	-0.02	0.01
32	->	27	-0.00	0.00
32	->	28	-0.02	0.00
32	->	29	-0.03	0.00

32	->	30	-1.44	0.45
32	->	31	-0.20	0.41
32	->	32	1.71	0.50
32	->	33	-10.19	0.48
32	->	34	-0.78	0.50
32	->	35	-0.02	0.00
32	->	36	-0.02	0.03
32	->	37	-0.00	0.00
32	->	38	-0.00	0.00
32	->	39	-0.01	0.00
32	->	40	-0.01	0.01
32	->	41	-0.32	0.25
32	->	42	-0.87	0.11
32	->	43	-0.86	0.31
32	->	44	-0.20	0.08
32	->	45	-0.07	0.02
32	->	46	-0.02	0.00
32	->	47	-0.01	0.01
32	->	48	-0.05	0.01
32	->	49	-0.01	0.00
32	->	50	-0.00	0.00
32	->	51	-0.00	0.00
32	->	52	-0.00	0.00
32	->	53	-0.00	0.00
32	->	54	-0.00	0.00
32	->	55	-0.00	0.00
32	->	56	-0.00	0.00
32	->	57	-0.00	0.00
32	->	58	0.00	0.00
32	->	59	-0.00	0.00
32	->	60	0.00	0.00
32	->	61	0.00	0.00
32	->	62	0.00	0.00
32	->	63	-0.00	0.00
32	->	64	0.00	0.00
32	->	65	0.00	0.00
32	->	66	-0.00	0.00
32	->	67	0.00	0.00
32	->	68	0.00	0.00
32	->	69	-0.00	0.00
32	->	70	0.00	0.00
32	->	71	0.00	0.00
32	->	72	-0.00	0.00
32	->	73	0.00	0.00
32	->	74	-0.00	0.00
32	->	75	-0.00	0.00
32	->	76	0.00	0.00
32	->	77	0.00	0.00
32	->	78	0.00	0.00
32	->	79	0.00	0.00

32	->	80	0.00	0.00
32	->	81	0.00	0.00
32	->	82	0.00	0.00
32	->	83	0.00	0.00
32	->	84	0.00	0.00
32	->	85	0.00	0.00
32	->	86	0.00	0.00
32	->	87	0.00	0.00
32	->	88	-0.00	0.00
32	->	89	-0.00	0.00
32	->	90	-0.01	0.00
32	->	91	-0.01	0.00
32	->	92	-0.01	0.00
32	->	93	-0.00	0.00
32	->	94	-0.00	0.00
32	->	95	-0.00	0.00
32	->	96	-0.00	0.00
32	->	97	-0.00	0.00
32	->	98	-0.05	0.02
32	->	99	-0.18	0.09
32	->	100	-0.01	0.00
32	->	101	-0.00	0.00
32	->	102	-0.00	0.00
32	->	103	-0.00	0.00
32	->	104	-0.00	0.00
32	->	105	-0.00	0.00
32	->	106	-0.00	0.00
32	->	107	-0.00	0.00
33	->	1	-0.00	0.00
33	->	2	0.00	0.00
33	->	3	-0.00	0.00
33	->	4	-0.00	0.00
33	->	5	0.00	0.00
33	->	6	-0.00	0.00
33	->	7	-0.00	0.00
33	->	8	0.00	0.00
33	->	9	-0.00	0.00
33	->	10	0.00	0.00
33	->	11	0.00	0.00
33	->	12	-0.00	0.00
33	->	13	-0.00	0.00
33	->	14	-0.00	0.00
33	->	15	0.00	0.00
33	->	16	-0.00	0.00
33	->	17	-0.00	0.00
33	->	18	-0.00	0.00
33	->	19	-0.00	0.00
33	->	20	-0.00	0.00
33	->	21	-0.02	0.01
33	->	22	-0.02	0.01

33 ->	23	-0.00	0.00
33 ->	24	-0.01	0.00
33 ->	25	-0.52	0.18
33 ->	26	-0.00	0.00
33 ->	27	-0.00	0.00
33 ->	28	-0.00	0.00
33 ->	29	-0.01	0.00
33 ->	30	-0.09	0.02
33 ->	31	-0.87	0.26
33 ->	32	-10.33	0.49
33 ->	33	27.79	1.20
33 ->	34	-13.07	0.31
33 ->	35	-0.44	0.08
33 ->	36	-0.03	0.02
33 ->	37	-0.00	0.00
33 ->	38	-0.01	0.01
33 ->	39	-0.02	0.01
33 ->	40	-0.24	0.08
33 ->	41	-2.74	0.58
33 ->	42	-3.59	0.43
33 ->	43	-0.83	0.22
33 ->	44	-2.53	0.35
33 ->	45	-2.39	0.40
33 ->	46	0.02	0.02
33 ->	47	-0.10	0.04
33 ->	48	-1.65	0.36
33 ->	49	-0.05	0.02
33 ->	50	-0.01	0.00
33 ->	51	-0.02	0.01
33 ->	52	-0.02	0.01
33 ->	53	-0.00	0.00
33 ->	54	-0.00	0.00
33 ->	55	-0.01	0.00
33 ->	56	-0.00	0.00
33 ->	57	0.00	0.00
33 ->	58	-0.00	0.00
33 ->	59	-0.00	0.00
33 ->	60	-0.00	0.00
33 ->	61	-0.00	0.00
33 ->	62	0.00	0.00
33 ->	63	-0.00	0.00
33 ->	64	-0.00	0.00
33 ->	65	0.00	0.00
33 ->	66	0.00	0.00
33 ->	67	0.00	0.00
33 ->	68	-0.00	0.00
33 ->	69	0.00	0.00
33 ->	70	0.00	0.00
33 ->	71	-0.00	0.00
33 ->	72	-0.00	0.00

33	->	73	-0.00	0.00
33	->	74	-0.00	0.00
33	->	75	0.00	0.00
33	->	76	0.00	0.00
33	->	77	-0.00	0.00
33	->	78	-0.00	0.00
33	->	79	-0.00	0.00
33	->	80	0.00	0.00
33	->	81	-0.00	0.00
33	->	82	0.00	0.00
33	->	83	0.00	0.00
33	->	84	0.00	0.00
33	->	85	0.00	0.00
33	->	86	0.00	0.00
33	->	87	-0.00	0.00
33	->	88	-0.00	0.00
33	->	89	-0.00	0.00
33	->	90	-0.01	0.00
33	->	91	-0.01	0.00
33	->	92	-0.00	0.00
33	->	93	-0.00	0.00
33	->	94	-0.00	0.00
33	->	95	-0.00	0.00
33	->	96	-0.00	0.00
33	->	97	-0.01	0.00
33	->	98	-0.02	0.02
33	->	99	-1.48	0.33
33	->	100	-0.11	0.08
33	->	101	-0.02	0.00
33	->	102	-0.00	0.00
33	->	103	-0.00	0.00
33	->	104	-0.00	0.00
33	->	105	-0.00	0.00
33	->	106	-0.00	0.00
33	->	107	0.00	0.00
34	->	1	0.00	0.00
34	->	2	-0.00	0.00
34	->	3	-0.00	0.00
34	->	4	-0.00	0.00
34	->	5	-0.00	0.00
34	->	6	0.00	0.00
34	->	7	0.00	0.00
34	->	8	-0.00	0.00
34	->	9	0.00	0.00
34	->	10	-0.00	0.00
34	->	11	-0.00	0.00
34	->	12	0.00	0.00
34	->	13	0.00	0.00
34	->	14	0.00	0.00
34	->	15	0.00	0.00

34 ->	16	0.00	0.00
34 ->	17	-0.00	0.00
34 ->	18	-0.00	0.00
34 ->	19	-0.00	0.00
34 ->	20	-0.00	0.00
34 ->	21	-0.00	0.00
34 ->	22	-0.00	0.00
34 ->	23	-0.00	0.00
34 ->	24	-0.00	0.00
34 ->	25	-0.01	0.00
34 ->	26	-0.00	0.00
34 ->	27	-0.00	0.00
34 ->	28	-0.00	0.00
34 ->	29	-0.00	0.00
34 ->	30	-0.01	0.00
34 ->	31	-0.25	0.28
34 ->	32	-0.81	0.51
34 ->	33	-13.07	0.30
34 ->	34	-56.03	2.73
34 ->	35	-11.76	1.14
34 ->	36	-1.39	1.43
34 ->	37	-0.01	0.10
34 ->	38	-0.05	0.20
34 ->	39	-0.75	1.25
34 ->	40	-0.72	0.90
34 ->	41	-2.89	0.86
34 ->	42	-0.70	0.35
34 ->	43	-0.03	0.01
34 ->	44	-0.02	0.01
34 ->	45	-0.02	0.01
34 ->	46	-0.00	0.00
34 ->	47	-0.00	0.02
34 ->	48	-1.34	0.25
34 ->	49	-0.01	0.01
34 ->	50	-0.00	0.00
34 ->	51	-0.06	0.02
34 ->	52	-0.05	0.02
34 ->	53	-0.01	0.00
34 ->	54	-0.00	0.00
34 ->	55	-0.06	0.02
34 ->	56	-0.00	0.00
34 ->	57	-0.00	0.00
34 ->	58	0.00	0.00
34 ->	59	-0.00	0.00
34 ->	60	-0.00	0.00
34 ->	61	0.00	0.00
34 ->	62	0.00	0.00
34 ->	63	0.00	0.00
34 ->	64	0.00	0.00
34 ->	65	0.00	0.00

34	->	66	0.00	0.00
34	->	67	0.00	0.00
34	->	68	-0.00	0.00
34	->	69	-0.00	0.00
34	->	70	0.00	0.00
34	->	71	0.00	0.00
34	->	72	-0.00	0.00
34	->	73	-0.00	0.00
34	->	74	0.00	0.00
34	->	75	-0.00	0.00
34	->	76	-0.00	0.00
34	->	77	-0.00	0.00
34	->	78	0.00	0.00
34	->	79	-0.00	0.00
34	->	80	0.00	0.00
34	->	81	0.00	0.00
34	->	82	0.00	0.00
34	->	83	0.00	0.00
34	->	84	0.00	0.00
34	->	85	-0.00	0.00
34	->	86	0.00	0.00
34	->	87	-0.00	0.00
34	->	88	-0.00	0.01
34	->	89	-0.01	0.01
34	->	90	-0.01	0.00
34	->	91	-0.00	0.00
34	->	92	-0.00	0.00
34	->	93	-0.00	0.00
34	->	94	0.00	0.00
34	->	95	0.00	0.00
34	->	96	0.00	0.00
34	->	97	-0.00	0.00
34	->	98	-0.00	0.00
34	->	99	-0.00	0.00
34	->	100	-0.00	0.00
34	->	101	0.00	0.00
34	->	102	-0.00	0.00
34	->	103	-0.00	0.00
34	->	104	-0.00	0.00
34	->	105	-0.00	0.00
34	->	106	-0.00	0.00
34	->	107	-0.00	0.00
35	->	1	-0.01	0.00
35	->	2	-0.00	0.00
35	->	3	-0.00	0.00
35	->	4	0.00	0.00
35	->	5	0.01	0.00
35	->	6	-0.00	0.00
35	->	7	0.00	0.00
35	->	8	0.00	0.00

35	->	9	-0.00	0.00
35	->	10	0.01	0.00
35	->	11	0.00	0.00
35	->	12	-0.00	0.00
35	->	13	-0.00	0.00
35	->	14	-0.00	0.00
35	->	15	-0.00	0.00
35	->	16	-0.00	0.00
35	->	17	0.00	0.00
35	->	18	0.00	0.00
35	->	19	0.01	0.00
35	->	20	0.00	0.00
35	->	21	0.00	0.00
35	->	22	-0.01	0.00
35	->	23	-0.00	0.00
35	->	24	0.00	0.00
35	->	25	-0.03	0.00
35	->	26	0.00	0.00
35	->	27	-0.00	0.00
35	->	28	0.00	0.00
35	->	29	0.00	0.00
35	->	30	-0.00	0.00
35	->	31	-0.10	0.02
35	->	32	-0.02	0.00
35	->	33	-0.44	0.08
35	->	34	-11.81	1.13
35	->	35	-25.07	2.83
35	->	36	-9.34	0.74
35	->	37	-5.90	1.50
35	->	38	-0.06	1.82
35	->	39	-1.98	1.78
35	->	40	-1.28	0.79
35	->	41	-0.86	0.60
35	->	42	-0.52	0.20
35	->	43	0.01	0.01
35	->	44	-0.04	0.01
35	->	45	-0.00	0.01
35	->	46	0.04	0.01
35	->	47	-0.11	0.02
35	->	48	-1.08	0.24
35	->	49	0.01	0.01
35	->	50	-0.04	0.02
35	->	51	1.54	0.28
35	->	52	-0.19	0.23
35	->	53	-0.05	0.01
35	->	54	-0.08	0.04
35	->	55	3.41	0.63
35	->	56	-0.01	0.01
35	->	57	-0.00	0.00
35	->	58	0.03	0.01

35	->	59	-0.03	0.01
35	->	60	-0.00	0.00
35	->	61	0.00	0.00
35	->	62	-0.00	0.00
35	->	63	0.01	0.00
35	->	64	0.00	0.00
35	->	65	0.00	0.00
35	->	66	0.01	0.00
35	->	67	0.00	0.00
35	->	68	-0.01	0.00
35	->	69	-0.01	0.00
35	->	70	-0.00	0.00
35	->	71	-0.00	0.00
35	->	72	-0.03	0.00
35	->	73	-0.00	0.00
35	->	74	0.02	0.00
35	->	75	0.02	0.01
35	->	76	-0.01	0.00
35	->	77	-0.01	0.00
35	->	78	-0.01	0.00
35	->	79	-0.00	0.00
35	->	80	-0.00	0.00
35	->	81	-0.00	0.00
35	->	82	-0.00	0.00
35	->	83	0.00	0.00
35	->	84	0.00	0.00
35	->	85	-0.00	0.00
35	->	86	0.00	0.00
35	->	87	0.02	0.01
35	->	88	0.06	0.02
35	->	89	0.08	0.03
35	->	90	0.04	0.01
35	->	91	0.02	0.00
35	->	92	0.01	0.00
35	->	93	0.01	0.00
35	->	94	0.00	0.00
35	->	95	0.00	0.00
35	->	96	0.00	0.00
35	->	97	0.00	0.00
35	->	98	0.01	0.00
35	->	99	0.02	0.00
35	->	100	0.03	0.00
35	->	101	0.01	0.00
35	->	102	0.01	0.00
35	->	103	0.01	0.00
35	->	104	0.01	0.00
35	->	105	0.01	0.00
35	->	106	0.00	0.00
35	->	107	0.01	0.00
36	->	1	0.00	0.00

36	->	2	0.00	0.00
36	->	3	-0.00	0.00
36	->	4	-0.00	0.00
36	->	5	-0.00	0.00
36	->	6	0.00	0.00
36	->	7	-0.00	0.00
36	->	8	-0.00	0.00
36	->	9	0.00	0.00
36	->	10	-0.00	0.00
36	->	11	-0.00	0.00
36	->	12	0.00	0.00
36	->	13	0.00	0.00
36	->	14	0.00	0.00
36	->	15	-0.00	0.00
36	->	16	0.00	0.00
36	->	17	-0.00	0.00
36	->	18	-0.00	0.00
36	->	19	-0.01	0.00
36	->	20	-0.00	0.00
36	->	21	-0.00	0.00
36	->	22	0.01	0.00
36	->	23	0.00	0.00
36	->	24	-0.00	0.00
36	->	25	0.02	0.01
36	->	26	-0.00	0.00
36	->	27	0.00	0.00
36	->	28	-0.00	0.00
36	->	29	-0.00	0.00
36	->	30	-0.00	0.00
36	->	31	0.01	0.00
36	->	32	-0.02	0.03
36	->	33	-0.03	0.02
36	->	34	-1.42	1.44
36	->	35	-9.40	0.76
36	->	36	-116.85	1.98
36	->	37	-13.66	0.69
36	->	38	-0.68	0.23
36	->	39	-0.74	0.78
36	->	40	-0.01	0.01
36	->	41	-0.03	0.02
36	->	42	-0.02	0.01
36	->	43	-0.00	0.00
36	->	44	0.01	0.00
36	->	45	-0.01	0.00
36	->	46	-0.03	0.01
36	->	47	-0.01	0.00
36	->	48	-0.14	0.11
36	->	49	-0.02	0.04
36	->	50	-0.01	0.00
36	->	51	-0.06	0.03

36	->	52	-0.36	0.58
36	->	53	0.02	0.01
36	->	54	-0.00	0.00
36	->	55	-0.23	0.07
36	->	56	-0.03	0.05
36	->	57	-0.00	0.00
36	->	58	-0.04	0.02
36	->	59	0.02	0.00
36	->	60	0.00	0.00
36	->	61	-0.00	0.00
36	->	62	-0.00	0.00
36	->	63	-0.00	0.00
36	->	64	-0.00	0.00
36	->	65	-0.00	0.00
36	->	66	-0.00	0.00
36	->	67	-0.00	0.00
36	->	68	0.01	0.00
36	->	69	0.01	0.00
36	->	70	-0.00	0.00
36	->	71	-0.00	0.00
36	->	72	0.01	0.00
36	->	73	0.00	0.00
36	->	74	-0.01	0.00
36	->	75	-0.01	0.00
36	->	76	0.01	0.00
36	->	77	0.00	0.00
36	->	78	0.01	0.00
36	->	79	0.00	0.00
36	->	80	0.00	0.00
36	->	81	0.00	0.00
36	->	82	0.00	0.00
36	->	83	-0.00	0.00
36	->	84	0.00	0.00
36	->	85	0.00	0.00
36	->	86	-0.00	0.00
36	->	87	-0.01	0.00
36	->	88	-0.02	0.01
36	->	89	-0.03	0.01
36	->	90	-0.02	0.00
36	->	91	-0.01	0.00
36	->	92	-0.01	0.00
36	->	93	-0.00	0.00
36	->	94	-0.00	0.00
36	->	95	-0.00	0.00
36	->	96	-0.00	0.00
36	->	97	-0.00	0.00
36	->	98	-0.01	0.00
36	->	99	-0.03	0.01
36	->	100	-0.04	0.02
36	->	101	-0.02	0.01

36	->	102	-0.01	0.00
36	->	103	-0.00	0.00
36	->	104	-0.00	0.00
36	->	105	-0.00	0.00
36	->	106	-0.00	0.00
36	->	107	-0.00	0.00
37	->	1	0.00	0.00
37	->	2	0.00	0.00
37	->	3	0.00	0.00
37	->	4	0.00	0.00
37	->	5	-0.00	0.00
37	->	6	0.00	0.00
37	->	7	0.00	0.00
37	->	8	-0.00	0.00
37	->	9	0.00	0.00
37	->	10	-0.00	0.00
37	->	11	-0.00	0.00
37	->	12	0.00	0.00
37	->	13	0.00	0.00
37	->	14	0.00	0.00
37	->	15	0.00	0.00
37	->	16	0.00	0.00
37	->	17	-0.00	0.00
37	->	18	-0.00	0.00
37	->	19	-0.00	0.00
37	->	20	-0.00	0.00
37	->	21	-0.00	0.00
37	->	22	0.00	0.00
37	->	23	0.00	0.00
37	->	24	0.00	0.00
37	->	25	0.00	0.00
37	->	26	0.00	0.00
37	->	27	0.00	0.00
37	->	28	0.00	0.00
37	->	29	0.00	0.00
37	->	30	-0.00	0.00
37	->	31	0.00	0.00
37	->	32	-0.00	0.00
37	->	33	-0.00	0.00
37	->	34	-0.02	0.10
37	->	35	-5.98	1.51
37	->	36	-13.79	0.68
37	->	37	19.06	1.81
37	->	38	-14.12	0.77
37	->	39	-0.61	0.38
37	->	40	-0.03	0.04
37	->	41	-0.02	0.01
37	->	42	-0.04	0.01
37	->	43	-0.00	0.00
37	->	44	0.00	0.00

37	->	45	-0.00	0.00
37	->	46	-0.00	0.00
37	->	47	0.00	0.00
37	->	48	-0.04	0.01
37	->	49	-0.01	0.00
37	->	50	-0.00	0.00
37	->	51	-0.46	0.11
37	->	52	-0.52	0.41
37	->	53	-0.01	0.01
37	->	54	-0.01	0.01
37	->	55	-3.76	0.44
37	->	56	-0.18	0.18
37	->	57	-0.01	0.00
37	->	58	-0.01	0.00
37	->	59	-0.00	0.00
37	->	60	-0.00	0.00
37	->	61	-0.00	0.00
37	->	62	-0.00	0.00
37	->	63	-0.00	0.00
37	->	64	-0.00	0.00
37	->	65	0.00	0.00
37	->	66	-0.00	0.00
37	->	67	-0.00	0.00
37	->	68	0.00	0.00
37	->	69	0.00	0.00
37	->	70	-0.00	0.00
37	->	71	-0.00	0.00
37	->	72	0.00	0.00
37	->	73	0.00	0.00
37	->	74	-0.00	0.00
37	->	75	-0.00	0.00
37	->	76	0.00	0.00
37	->	77	0.00	0.00
37	->	78	0.00	0.00
37	->	79	0.00	0.00
37	->	80	0.00	0.00
37	->	81	0.00	0.00
37	->	82	0.00	0.00
37	->	83	-0.00	0.00
37	->	84	0.00	0.00
37	->	85	0.00	0.00
37	->	86	-0.00	0.00
37	->	87	-0.00	0.00
37	->	88	-0.01	0.01
37	->	89	-0.01	0.01
37	->	90	-0.00	0.00
37	->	91	-0.00	0.00
37	->	92	-0.00	0.00
37	->	93	-0.00	0.00
37	->	94	-0.00	0.00

37	->	95	-0.00	0.00
37	->	96	-0.00	0.00
37	->	97	-0.00	0.00
37	->	98	-0.00	0.00
37	->	99	-0.00	0.00
37	->	100	-0.00	0.00
37	->	101	-0.00	0.00
37	->	102	-0.00	0.00
37	->	103	-0.00	0.00
37	->	104	-0.00	0.00
37	->	105	-0.00	0.00
37	->	106	-0.00	0.00
37	->	107	-0.00	0.00
38	->	1	-0.00	0.00
38	->	2	0.00	0.00
38	->	3	0.00	0.00
38	->	4	0.00	0.00
38	->	5	0.00	0.00
38	->	6	-0.00	0.00
38	->	7	0.00	0.00
38	->	8	0.00	0.00
38	->	9	-0.00	0.00
38	->	10	0.01	0.00
38	->	11	0.00	0.00
38	->	12	-0.00	0.00
38	->	13	-0.00	0.00
38	->	14	-0.00	0.00
38	->	15	-0.00	0.00
38	->	16	-0.00	0.00
38	->	17	0.00	0.00
38	->	18	0.00	0.00
38	->	19	0.01	0.00
38	->	20	0.00	0.00
38	->	21	0.00	0.00
38	->	22	-0.01	0.00
38	->	23	0.00	0.00
38	->	24	0.00	0.00
38	->	25	-0.02	0.00
38	->	26	0.00	0.00
38	->	27	-0.00	0.00
38	->	28	0.00	0.00
38	->	29	0.00	0.00
38	->	30	0.00	0.00
38	->	31	-0.05	0.01
38	->	32	-0.00	0.00
38	->	33	-0.01	0.01
38	->	34	-0.05	0.20
38	->	35	-0.09	1.83
38	->	36	-0.68	0.23
38	->	37	-14.18	0.76

38	->	38	-36.57	3.03
38	->	39	-11.39	0.64
38	->	40	-2.63	3.02
38	->	41	-0.03	0.05
38	->	42	-0.02	0.03
38	->	43	-0.00	0.00
38	->	44	-0.02	0.00
38	->	45	0.00	0.00
38	->	46	0.03	0.00
38	->	47	-0.01	0.01
38	->	48	-0.01	0.01
38	->	49	-0.00	0.00
38	->	50	-0.01	0.01
38	->	51	1.50	0.34
38	->	52	0.01	0.01
38	->	53	-0.04	0.00
38	->	54	-0.06	0.02
38	->	55	2.31	0.73
38	->	56	-0.01	0.00
38	->	57	-0.00	0.00
38	->	58	0.02	0.00
38	->	59	-0.04	0.01
38	->	60	-0.00	0.00
38	->	61	0.00	0.00
38	->	62	-0.00	0.00
38	->	63	0.01	0.00
38	->	64	0.00	0.00
38	->	65	0.00	0.00
38	->	66	0.01	0.00
38	->	67	0.00	0.00
38	->	68	-0.02	0.01
38	->	69	-0.01	0.00
38	->	70	-0.00	0.00
38	->	71	-0.00	0.00
38	->	72	-0.03	0.00
38	->	73	-0.00	0.00
38	->	74	0.02	0.00
38	->	75	0.02	0.01
38	->	76	-0.01	0.00
38	->	77	-0.01	0.00
38	->	78	-0.01	0.00
38	->	79	-0.00	0.00
38	->	80	-0.00	0.00
38	->	81	-0.01	0.00
38	->	82	-0.00	0.00
38	->	83	0.00	0.00
38	->	84	-0.00	0.00
38	->	85	-0.00	0.00
38	->	86	0.00	0.00
38	->	87	0.03	0.01

38	->	88	0.09	0.04
38	->	89	0.13	0.09
38	->	90	0.04	0.01
38	->	91	0.02	0.00
38	->	92	0.01	0.00
38	->	93	0.01	0.00
38	->	94	0.00	0.00
38	->	95	0.00	0.00
38	->	96	0.00	0.00
38	->	97	0.00	0.00
38	->	98	0.01	0.00
38	->	99	0.01	0.00
38	->	100	0.02	0.00
38	->	101	0.01	0.00
38	->	102	0.01	0.00
38	->	103	0.01	0.00
38	->	104	0.01	0.00
38	->	105	0.01	0.00
38	->	106	0.00	0.00
38	->	107	0.01	0.00
39	->	1	-0.00	0.00
39	->	2	0.00	0.00
39	->	3	0.00	0.00
39	->	4	0.00	0.00
39	->	5	0.00	0.00
39	->	6	0.00	0.00
39	->	7	0.00	0.00
39	->	8	0.00	0.00
39	->	9	-0.00	0.00
39	->	10	0.00	0.00
39	->	11	0.00	0.00
39	->	12	-0.00	0.00
39	->	13	-0.00	0.00
39	->	14	-0.00	0.00
39	->	15	0.00	0.00
39	->	16	-0.00	0.00
39	->	17	0.00	0.00
39	->	18	0.00	0.00
39	->	19	0.00	0.00
39	->	20	0.00	0.00
39	->	21	0.00	0.00
39	->	22	-0.00	0.00
39	->	23	0.00	0.00
39	->	24	0.00	0.00
39	->	25	-0.00	0.00
39	->	26	-0.00	0.00
39	->	27	-0.00	0.00
39	->	28	-0.00	0.00
39	->	29	-0.00	0.00
39	->	30	-0.00	0.00

39 ->	31	-0.02	0.01
39 ->	32	-0.01	0.00
39 ->	33	-0.02	0.01
39 ->	34	-0.75	1.25
39 ->	35	-2.01	1.80
39 ->	36	-0.72	0.76
39 ->	37	-0.62	0.38
39 ->	38	-11.47	0.65
39 ->	39	11.06	1.40
39 ->	40	-9.33	0.75
39 ->	41	-0.77	0.52
39 ->	42	-0.09	0.02
39 ->	43	-0.01	0.00
39 ->	44	-0.00	0.00
39 ->	45	-0.00	0.00
39 ->	46	-0.00	0.00
39 ->	47	0.00	0.00
39 ->	48	-0.02	0.01
39 ->	49	-0.00	0.00
39 ->	50	-0.00	0.00
39 ->	51	-0.04	0.04
39 ->	52	-0.01	0.00
39 ->	53	-0.00	0.00
39 ->	54	-0.00	0.00
39 ->	55	-0.02	0.06
39 ->	56	-0.00	0.00
39 ->	57	0.00	0.00
39 ->	58	-0.00	0.00
39 ->	59	-0.00	0.00
39 ->	60	0.00	0.00
39 ->	61	0.00	0.00
39 ->	62	0.00	0.00
39 ->	63	-0.00	0.00
39 ->	64	0.00	0.00
39 ->	65	0.00	0.00
39 ->	66	-0.00	0.00
39 ->	67	0.00	0.00
39 ->	68	0.00	0.00
39 ->	69	0.00	0.00
39 ->	70	0.00	0.00
39 ->	71	-0.00	0.00
39 ->	72	-0.00	0.00
39 ->	73	-0.00	0.00
39 ->	74	0.00	0.00
39 ->	75	-0.00	0.00
39 ->	76	0.00	0.00
39 ->	77	0.00	0.00
39 ->	78	0.00	0.00
39 ->	79	0.00	0.00
39 ->	80	0.00	0.00

39	->	81	0.00	0.00
39	->	82	0.00	0.00
39	->	83	0.00	0.00
39	->	84	0.00	0.00
39	->	85	-0.00	0.00
39	->	86	-0.00	0.00
39	->	87	-0.00	0.00
39	->	88	-0.01	0.01
39	->	89	-0.01	0.01
39	->	90	-0.00	0.00
39	->	91	-0.00	0.00
39	->	92	0.00	0.00
39	->	93	0.00	0.00
39	->	94	0.00	0.00
39	->	95	0.00	0.00
39	->	96	0.00	0.00
39	->	97	0.00	0.00
39	->	98	0.00	0.00
39	->	99	-0.00	0.00
39	->	100	-0.00	0.00
39	->	101	-0.00	0.00
39	->	102	0.00	0.00
39	->	103	-0.00	0.00
39	->	104	0.00	0.00
39	->	105	0.00	0.00
39	->	106	-0.00	0.00
39	->	107	-0.00	0.00
40	->	1	0.01	0.00
40	->	2	-0.00	0.00
40	->	3	-0.00	0.00
40	->	4	-0.00	0.00
40	->	5	-0.01	0.00
40	->	6	0.00	0.00
40	->	7	-0.00	0.00
40	->	8	-0.00	0.00
40	->	9	0.00	0.00
40	->	10	-0.01	0.00
40	->	11	-0.01	0.00
40	->	12	0.01	0.00
40	->	13	0.00	0.00
40	->	14	0.00	0.00
40	->	15	0.00	0.00
40	->	16	0.01	0.00
40	->	17	-0.00	0.00
40	->	18	-0.00	0.00
40	->	19	-0.01	0.00
40	->	20	-0.00	0.00
40	->	21	-0.00	0.00
40	->	22	0.02	0.00
40	->	23	-0.00	0.00

40	->	24	-0.00	0.00
40	->	25	0.03	0.01
40	->	26	-0.01	0.00
40	->	27	-0.00	0.00
40	->	28	-0.00	0.00
40	->	29	-0.01	0.01
40	->	30	0.00	0.00
40	->	31	0.13	0.37
40	->	32	-0.01	0.01
40	->	33	-0.24	0.08
40	->	34	-0.71	0.90
40	->	35	-1.27	0.79
40	->	36	-0.01	0.01
40	->	37	-0.03	0.04
40	->	38	-2.73	3.06
40	->	39	-9.41	0.73
40	->	40	-108.53	3.27
40	->	41	-15.59	0.72
40	->	42	-2.37	1.22
40	->	43	-0.08	0.04
40	->	44	0.02	0.01
40	->	45	-0.01	0.00
40	->	46	-0.04	0.01
40	->	47	-0.00	0.02
40	->	48	-0.10	0.04
40	->	49	-0.01	0.00
40	->	50	-0.00	0.01
40	->	51	-0.46	0.45
40	->	52	-0.02	0.01
40	->	53	0.02	0.01
40	->	54	-0.01	0.04
40	->	55	-0.14	0.18
40	->	56	0.00	0.00
40	->	57	0.00	0.00
40	->	58	-0.01	0.00
40	->	59	0.03	0.01
40	->	60	0.00	0.00
40	->	61	-0.00	0.00
40	->	62	0.00	0.00
40	->	63	-0.01	0.00
40	->	64	-0.00	0.00
40	->	65	-0.00	0.00
40	->	66	-0.01	0.00
40	->	67	-0.00	0.00
40	->	68	0.02	0.01
40	->	69	0.01	0.00
40	->	70	0.00	0.00
40	->	71	-0.00	0.00
40	->	72	0.04	0.02
40	->	73	-0.00	0.00

40	->	74	-0.02	0.01
40	->	75	-0.03	0.01
40	->	76	0.01	0.00
40	->	77	0.01	0.00
40	->	78	0.02	0.01
40	->	79	0.00	0.00
40	->	80	0.00	0.00
40	->	81	0.01	0.00
40	->	82	0.00	0.00
40	->	83	0.00	0.00
40	->	84	0.00	0.00
40	->	85	0.00	0.00
40	->	86	-0.01	0.00
40	->	87	-0.04	0.04
40	->	88	-0.53	0.54
40	->	89	-4.23	3.52
40	->	90	-1.99	2.45
40	->	91	-0.07	0.03
40	->	92	-0.03	0.01
40	->	93	-0.01	0.00
40	->	94	-0.01	0.00
40	->	95	-0.00	0.00
40	->	96	-0.00	0.00
40	->	97	-0.00	0.00
40	->	98	-0.01	0.00
40	->	99	-0.02	0.00
40	->	100	-0.02	0.00
40	->	101	-0.01	0.00
40	->	102	-0.01	0.00
40	->	103	-0.01	0.00
40	->	104	-0.01	0.00
40	->	105	-0.02	0.01
40	->	106	-0.01	0.01
40	->	107	-0.03	0.01
41	->	1	0.00	0.00
41	->	2	-0.00	0.00
41	->	3	0.00	0.00
41	->	4	-0.00	0.00
41	->	5	-0.00	0.00
41	->	6	0.00	0.00
41	->	7	0.00	0.00
41	->	8	-0.00	0.00
41	->	9	0.00	0.00
41	->	10	-0.00	0.00
41	->	11	-0.00	0.00
41	->	12	0.00	0.00
41	->	13	0.00	0.00
41	->	14	0.00	0.00
41	->	15	-0.00	0.00
41	->	16	0.00	0.00

41	->	17	-0.00	0.00
41	->	18	0.00	0.00
41	->	19	-0.00	0.00
41	->	20	0.00	0.00
41	->	21	0.00	0.00
41	->	22	0.01	0.00
41	->	23	-0.00	0.00
41	->	24	0.00	0.00
41	->	25	0.05	0.02
41	->	26	-0.02	0.00
41	->	27	-0.00	0.00
41	->	28	-0.00	0.00
41	->	29	-0.00	0.01
41	->	30	0.03	0.03
41	->	31	-2.21	0.77
41	->	32	-0.32	0.26
41	->	33	-2.75	0.58
41	->	34	-2.82	0.86
41	->	35	-0.85	0.60
41	->	36	-0.03	0.02
41	->	37	-0.02	0.01
41	->	38	-0.03	0.05
41	->	39	-0.79	0.52
41	->	40	-15.58	0.71
41	->	41	-30.32	1.93
41	->	42	-8.80	0.41
41	->	43	-0.54	0.07
41	->	44	-0.03	0.02
41	->	45	-0.05	0.01
41	->	46	-0.03	0.02
41	->	47	0.06	0.04
41	->	48	-0.12	0.04
41	->	49	-0.01	0.01
41	->	50	-0.00	0.01
41	->	51	-0.03	0.02
41	->	52	-0.01	0.00
41	->	53	-0.00	0.00
41	->	54	-0.00	0.00
41	->	55	-0.02	0.01
41	->	56	-0.00	0.00
41	->	57	-0.00	0.00
41	->	58	0.00	0.00
41	->	59	-0.00	0.00
41	->	60	-0.00	0.00
41	->	61	0.00	0.00
41	->	62	0.00	0.00
41	->	63	0.00	0.00
41	->	64	0.00	0.00
41	->	65	0.00	0.00
41	->	66	0.00	0.00

41	->	67	0.00	0.00
41	->	68	-0.00	0.00
41	->	69	-0.00	0.00
41	->	70	0.00	0.00
41	->	71	-0.00	0.00
41	->	72	-0.00	0.00
41	->	73	-0.00	0.00
41	->	74	0.00	0.00
41	->	75	0.00	0.00
41	->	76	-0.00	0.00
41	->	77	-0.00	0.00
41	->	78	-0.00	0.00
41	->	79	-0.00	0.00
41	->	80	0.00	0.00
41	->	81	-0.00	0.00
41	->	82	0.00	0.00
41	->	83	0.00	0.00
41	->	84	0.00	0.00
41	->	85	-0.00	0.00
41	->	86	0.00	0.00
41	->	87	-0.00	0.00
41	->	88	-0.01	0.01
41	->	89	-0.04	0.03
41	->	90	-0.06	0.04
41	->	91	-0.02	0.01
41	->	92	-0.00	0.00
41	->	93	-0.00	0.00
41	->	94	-0.00	0.00
41	->	95	-0.00	0.00
41	->	96	-0.00	0.00
41	->	97	-0.00	0.00
41	->	98	-0.01	0.00
41	->	99	-0.02	0.01
41	->	100	-0.01	0.00
41	->	101	-0.00	0.00
41	->	102	-0.00	0.00
41	->	103	-0.00	0.00
41	->	104	-0.00	0.00
41	->	105	-0.00	0.00
41	->	106	-0.00	0.00
41	->	107	0.00	0.00
42	->	1	0.00	0.00
42	->	2	0.00	0.00
42	->	3	0.00	0.00
42	->	4	0.00	0.00
42	->	5	-0.00	0.00
42	->	6	-0.00	0.00
42	->	7	0.00	0.00
42	->	8	-0.00	0.00
42	->	9	0.00	0.00

42	->	10	-0.00	0.00
42	->	11	-0.00	0.00
42	->	12	0.00	0.00
42	->	13	0.00	0.00
42	->	14	-0.00	0.00
42	->	15	0.00	0.00
42	->	16	0.00	0.00
42	->	17	0.00	0.00
42	->	18	0.00	0.00
42	->	19	-0.00	0.00
42	->	20	0.00	0.00
42	->	21	0.00	0.00
42	->	22	0.01	0.01
42	->	23	-0.01	0.00
42	->	24	0.00	0.00
42	->	25	0.04	0.06
42	->	26	-0.02	0.01
42	->	27	-0.01	0.00
42	->	28	-0.00	0.00
42	->	29	-0.00	0.01
42	->	30	-0.25	0.07
42	->	31	-3.40	0.45
42	->	32	-0.87	0.11
42	->	33	-3.60	0.43
42	->	34	-0.70	0.35
42	->	35	-0.52	0.20
42	->	36	-0.02	0.01
42	->	37	-0.04	0.01
42	->	38	-0.02	0.03
42	->	39	-0.09	0.02
42	->	40	-2.36	1.20
42	->	41	-8.88	0.41
42	->	42	28.65	1.30
42	->	43	-17.22	0.30
42	->	44	-1.01	0.19
42	->	45	-0.19	0.05
42	->	46	-0.16	0.05
42	->	47	-2.41	0.41
42	->	48	-2.53	0.37
42	->	49	-0.07	0.01
42	->	50	-0.09	0.04
42	->	51	-2.01	0.39
42	->	52	-0.05	0.02
42	->	53	-0.02	0.00
42	->	54	-0.02	0.01
42	->	55	-0.01	0.01
42	->	56	-0.00	0.00
42	->	57	-0.00	0.00
42	->	58	-0.00	0.00
42	->	59	-0.00	0.00

42	->	60	-0.00	0.00
42	->	61	-0.00	0.00
42	->	62	-0.00	0.00
42	->	63	-0.00	0.00
42	->	64	-0.00	0.00
42	->	65	0.00	0.00
42	->	66	-0.00	0.00
42	->	67	-0.00	0.00
42	->	68	0.00	0.00
42	->	69	-0.00	0.00
42	->	70	-0.00	0.00
42	->	71	-0.00	0.00
42	->	72	-0.01	0.01
42	->	73	-0.00	0.00
42	->	74	-0.00	0.00
42	->	75	-0.00	0.00
42	->	76	0.00	0.00
42	->	77	0.00	0.00
42	->	78	0.00	0.00
42	->	79	0.00	0.00
42	->	80	0.00	0.00
42	->	81	0.00	0.00
42	->	82	0.00	0.00
42	->	83	0.00	0.00
42	->	84	0.00	0.00
42	->	85	0.00	0.00
42	->	86	-0.00	0.00
42	->	87	-0.00	0.00
42	->	88	-0.01	0.03
42	->	89	-0.33	0.27
42	->	90	-0.27	0.23
42	->	91	-0.09	0.03
42	->	92	-0.02	0.01
42	->	93	-0.01	0.00
42	->	94	-0.00	0.00
42	->	95	-0.00	0.00
42	->	96	-0.00	0.00
42	->	97	-0.00	0.00
42	->	98	-0.02	0.01
42	->	99	-0.04	0.01
42	->	100	-0.02	0.00
42	->	101	-0.00	0.00
42	->	102	-0.00	0.00
42	->	103	-0.00	0.00
42	->	104	-0.00	0.00
42	->	105	-0.00	0.00
42	->	106	-0.00	0.00
42	->	107	-0.00	0.00
43	->	1	0.00	0.00
43	->	2	0.00	0.00

43	->	3	-0.00	0.00
43	->	4	0.00	0.00
43	->	5	-0.00	0.00
43	->	6	-0.00	0.00
43	->	7	-0.00	0.00
43	->	8	-0.00	0.00
43	->	9	-0.00	0.00
43	->	10	-0.00	0.00
43	->	11	-0.00	0.00
43	->	12	0.00	0.00
43	->	13	-0.00	0.00
43	->	14	-0.00	0.00
43	->	15	0.00	0.00
43	->	16	-0.00	0.00
43	->	17	-0.00	0.00
43	->	18	-0.00	0.00
43	->	19	-0.01	0.01
43	->	20	-0.01	0.01
43	->	21	-0.06	0.04
43	->	22	-0.22	0.20
43	->	23	-0.02	0.01
43	->	24	-0.03	0.02
43	->	25	-5.35	1.10
43	->	26	-0.69	0.37
43	->	27	-0.00	0.01
43	->	28	-0.02	0.01
43	->	29	-0.04	0.04
43	->	30	-1.01	0.96
43	->	31	-2.36	1.16
43	->	32	-0.85	0.30
43	->	33	-0.82	0.22
43	->	34	-0.03	0.01
43	->	35	0.01	0.01
43	->	36	-0.00	0.00
43	->	37	-0.00	0.00
43	->	38	-0.00	0.00
43	->	39	-0.01	0.00
43	->	40	-0.08	0.04
43	->	41	-0.54	0.07
43	->	42	-17.20	0.30
43	->	43	22.76	1.83
43	->	44	-6.40	0.56
43	->	45	-0.19	0.05
43	->	46	-0.18	0.11
43	->	47	-1.00	0.79
43	->	48	-0.03	0.01
43	->	49	-0.00	0.00
43	->	50	-0.01	0.00
43	->	51	-0.01	0.00
43	->	52	-0.00	0.00

43	->	53	-0.00	0.00
43	->	54	-0.00	0.00
43	->	55	0.00	0.00
43	->	56	-0.00	0.00
43	->	57	0.00	0.00
43	->	58	-0.00	0.00
43	->	59	0.00	0.00
43	->	60	0.00	0.00
43	->	61	0.00	0.00
43	->	62	0.00	0.00
43	->	63	-0.00	0.00
43	->	64	-0.00	0.00
43	->	65	0.00	0.00
43	->	66	-0.00	0.00
43	->	67	0.00	0.00
43	->	68	0.00	0.00
43	->	69	0.00	0.00
43	->	70	0.00	0.00
43	->	71	-0.00	0.00
43	->	72	0.00	0.00
43	->	73	-0.00	0.00
43	->	74	-0.00	0.00
43	->	75	-0.00	0.00
43	->	76	0.00	0.00
43	->	77	0.00	0.00
43	->	78	0.00	0.00
43	->	79	0.00	0.00
43	->	80	0.00	0.00
43	->	81	0.00	0.00
43	->	82	0.00	0.00
43	->	83	0.00	0.00
43	->	84	0.00	0.00
43	->	85	0.00	0.00
43	->	86	-0.00	0.00
43	->	87	-0.00	0.00
43	->	88	-0.00	0.00
43	->	89	-0.01	0.01
43	->	90	-0.03	0.05
43	->	91	-0.14	0.09
43	->	92	-0.05	0.02
43	->	93	-0.02	0.01
43	->	94	-0.00	0.00
43	->	95	-0.00	0.00
43	->	96	-0.00	0.00
43	->	97	-0.00	0.00
43	->	98	0.05	0.04
43	->	99	0.13	0.08
43	->	100	-0.05	0.02
43	->	101	0.01	0.01
43	->	102	-0.00	0.00

43	->	103	-0.00	0.00
43	->	104	-0.00	0.00
43	->	105	-0.00	0.00
43	->	106	-0.00	0.00
43	->	107	-0.00	0.00
44	->	1	0.05	0.02
44	->	2	0.00	0.00
44	->	3	-0.00	0.00
44	->	4	-0.00	0.00
44	->	5	-0.03	0.00
44	->	6	0.00	0.00
44	->	7	-0.00	0.00
44	->	8	-0.01	0.00
44	->	9	0.00	0.00
44	->	10	-0.03	0.01
44	->	11	-0.01	0.00
44	->	12	0.03	0.01
44	->	13	0.01	0.00
44	->	14	-0.00	0.00
44	->	15	0.00	0.00
44	->	16	0.06	0.01
44	->	17	0.00	0.01
44	->	18	-0.06	0.08
44	->	19	-0.26	0.12
44	->	20	-0.01	0.01
44	->	21	0.08	0.04
44	->	22	-0.29	0.52
44	->	23	-0.08	0.04
44	->	24	-0.01	0.01
44	->	25	-0.51	0.23
44	->	26	-0.09	0.08
44	->	27	-0.00	0.00
44	->	28	-0.00	0.00
44	->	29	-0.01	0.00
44	->	30	0.00	0.01
44	->	31	-0.07	0.04
44	->	32	-0.20	0.08
44	->	33	-2.54	0.35
44	->	34	-0.02	0.01
44	->	35	-0.04	0.01
44	->	36	0.01	0.00
44	->	37	0.00	0.00
44	->	38	-0.02	0.00
44	->	39	-0.00	0.00
44	->	40	0.02	0.01
44	->	41	-0.03	0.02
44	->	42	-1.00	0.19
44	->	43	-6.46	0.55
44	->	44	-91.35	2.75
44	->	45	-21.62	0.55

44 ->	46	-12.68	6.18
44 ->	47	-4.44	1.45
44 ->	48	-0.83	0.45
44 ->	49	0.01	0.05
44 ->	50	-0.05	0.03
44 ->	51	-0.00	0.01
44 ->	52	0.00	0.01
44 ->	53	0.04	0.01
44 ->	54	0.00	0.00
44 ->	55	-0.00	0.00
44 ->	56	0.00	0.00
44 ->	57	0.00	0.00
44 ->	58	-0.02	0.00
44 ->	59	0.02	0.00
44 ->	60	0.00	0.00
44 ->	61	-0.00	0.00
44 ->	62	-0.00	0.00
44 ->	63	-0.01	0.00
44 ->	64	-0.01	0.00
44 ->	65	-0.00	0.00
44 ->	66	-0.01	0.00
44 ->	67	-0.00	0.00
44 ->	68	0.01	0.00
44 ->	69	0.01	0.00
44 ->	70	0.00	0.00
44 ->	71	0.00	0.00
44 ->	72	0.11	0.04
44 ->	73	-0.00	0.01
44 ->	74	-0.08	0.03
44 ->	75	-0.03	0.01
44 ->	76	0.02	0.00
44 ->	77	0.03	0.01
44 ->	78	0.02	0.00
44 ->	79	0.00	0.00
44 ->	80	0.00	0.00
44 ->	81	0.02	0.01
44 ->	82	0.00	0.00
44 ->	83	0.00	0.00
44 ->	84	0.00	0.00
44 ->	85	0.00	0.00
44 ->	86	-0.00	0.00
44 ->	87	-0.02	0.00
44 ->	88	-0.03	0.01
44 ->	89	-0.05	0.02
44 ->	90	-0.12	0.07
44 ->	91	-1.90	1.38
44 ->	92	-6.55	1.53
44 ->	93	-4.25	1.93
44 ->	94	-0.13	0.23
44 ->	95	-0.04	0.02

44	->	96	-0.03	0.01
44	->	97	-0.02	0.00
44	->	98	-0.46	0.13
44	->	99	-1.71	0.47
44	->	100	-5.23	2.06
44	->	101	0.71	0.83
44	->	102	0.38	0.65
44	->	103	-0.11	0.14
44	->	104	-0.05	0.03
44	->	105	-0.03	0.01
44	->	106	-0.02	0.00
44	->	107	-0.01	0.00
45	->	1	0.00	0.00
45	->	2	0.00	0.00
45	->	3	0.00	0.00
45	->	4	-0.00	0.00
45	->	5	-0.00	0.00
45	->	6	0.00	0.00
45	->	7	-0.00	0.00
45	->	8	-0.00	0.00
45	->	9	0.00	0.00
45	->	10	0.00	0.00
45	->	11	0.00	0.00
45	->	12	-0.00	0.00
45	->	13	0.00	0.00
45	->	14	0.00	0.00
45	->	15	-0.00	0.00
45	->	16	-0.00	0.00
45	->	17	-0.00	0.00
45	->	18	-0.01	0.00
45	->	19	0.00	0.00
45	->	20	-0.00	0.00
45	->	21	-0.02	0.01
45	->	22	-0.03	0.01
45	->	23	0.00	0.00
45	->	24	-0.00	0.00
45	->	25	-0.28	0.07
45	->	26	0.00	0.00
45	->	27	0.00	0.00
45	->	28	-0.00	0.00
45	->	29	-0.00	0.00
45	->	30	-0.01	0.00
45	->	31	-0.08	0.02
45	->	32	-0.07	0.02
45	->	33	-2.44	0.41
45	->	34	-0.02	0.01
45	->	35	-0.00	0.01
45	->	36	-0.01	0.00
45	->	37	-0.00	0.00
45	->	38	0.00	0.00

45	->	39	-0.00	0.00
45	->	40	-0.01	0.00
45	->	41	-0.05	0.01
45	->	42	-0.19	0.05
45	->	43	-0.19	0.05
45	->	44	-21.63	0.55
45	->	45	24.68	1.30
45	->	46	-10.04	0.54
45	->	47	-0.93	0.17
45	->	48	-2.30	0.40
45	->	49	-3.33	1.36
45	->	50	-0.10	0.05
45	->	51	-0.05	0.03
45	->	52	-0.05	0.02
45	->	53	-0.02	0.01
45	->	54	-0.00	0.00
45	->	55	-0.00	0.00
45	->	56	-0.00	0.00
45	->	57	-0.00	0.00
45	->	58	0.00	0.00
45	->	59	-0.00	0.00
45	->	60	-0.00	0.00
45	->	61	-0.00	0.00
45	->	62	0.00	0.00
45	->	63	0.00	0.00
45	->	64	0.00	0.00
45	->	65	0.00	0.00
45	->	66	0.00	0.00
45	->	67	0.00	0.00
45	->	68	-0.00	0.00
45	->	69	-0.00	0.00
45	->	70	0.00	0.00
45	->	71	-0.00	0.00
45	->	72	-0.01	0.00
45	->	73	-0.00	0.00
45	->	74	0.00	0.00
45	->	75	0.00	0.00
45	->	76	-0.00	0.00
45	->	77	-0.00	0.00
45	->	78	-0.00	0.00
45	->	79	-0.00	0.00
45	->	80	-0.00	0.00
45	->	81	-0.00	0.00
45	->	82	-0.00	0.00
45	->	83	0.00	0.00
45	->	84	0.00	0.00
45	->	85	-0.00	0.00
45	->	86	0.00	0.00
45	->	87	0.00	0.00
45	->	88	0.00	0.00

45	->	89	0.00	0.00
45	->	90	-0.00	0.00
45	->	91	0.00	0.00
45	->	92	0.00	0.01
45	->	93	-0.00	0.00
45	->	94	-0.01	0.00
45	->	95	-0.00	0.00
45	->	96	-0.00	0.00
45	->	97	-0.00	0.00
45	->	98	0.05	0.05
45	->	99	-1.58	0.34
45	->	100	-5.22	0.99
45	->	101	-0.09	0.03
45	->	102	-0.01	0.01
45	->	103	-0.00	0.00
45	->	104	-0.00	0.00
45	->	105	0.00	0.00
45	->	106	0.00	0.00
45	->	107	0.00	0.00
46	->	1	-0.03	0.01
46	->	2	-0.00	0.00
46	->	3	0.00	0.00
46	->	4	0.00	0.00
46	->	5	0.01	0.00
46	->	6	-0.00	0.00
46	->	7	-0.00	0.00
46	->	8	0.00	0.00
46	->	9	-0.00	0.00
46	->	10	0.01	0.00
46	->	11	0.01	0.00
46	->	12	-0.02	0.01
46	->	13	-0.01	0.00
46	->	14	-0.00	0.00
46	->	15	-0.00	0.00
46	->	16	-0.03	0.01
46	->	17	-0.00	0.00
46	->	18	-0.01	0.01
46	->	19	0.05	0.01
46	->	20	-0.00	0.00
46	->	21	-0.02	0.01
46	->	22	-0.16	0.07
46	->	23	0.00	0.00
46	->	24	-0.00	0.00
46	->	25	-0.20	0.06
46	->	26	-0.00	0.00
46	->	27	-0.00	0.00
46	->	28	-0.00	0.00
46	->	29	-0.00	0.00
46	->	30	0.00	0.00
46	->	31	-0.08	0.02

46	->	32	-0.02	0.00
46	->	33	0.02	0.02
46	->	34	-0.00	0.00
46	->	35	0.04	0.01
46	->	36	-0.03	0.01
46	->	37	-0.00	0.00
46	->	38	0.03	0.00
46	->	39	-0.00	0.00
46	->	40	-0.04	0.01
46	->	41	-0.03	0.02
46	->	42	-0.16	0.05
46	->	43	-0.18	0.11
46	->	44	-12.64	6.15
46	->	45	-10.10	0.53
46	->	46	-13.88	6.90
46	->	47	-13.10	0.73
46	->	48	-1.36	0.20
46	->	49	-2.84	0.46
46	->	50	-2.19	1.08
46	->	51	-0.19	0.11
46	->	52	-0.07	0.04
46	->	53	-0.17	0.06
46	->	54	-0.01	0.00
46	->	55	-0.01	0.00
46	->	56	-0.01	0.00
46	->	57	-0.00	0.00
46	->	58	0.03	0.01
46	->	59	-0.04	0.01
46	->	60	-0.01	0.00
46	->	61	-0.01	0.01
46	->	62	0.00	0.00
46	->	63	0.01	0.00
46	->	64	0.01	0.00
46	->	65	0.00	0.00
46	->	66	0.01	0.00
46	->	67	0.00	0.00
46	->	68	-0.02	0.01
46	->	69	-0.02	0.01
46	->	70	-0.00	0.00
46	->	71	-0.02	0.01
46	->	72	-0.18	0.10
46	->	73	-0.01	0.01
46	->	74	0.06	0.01
46	->	75	0.04	0.01
46	->	76	-0.03	0.01
46	->	77	-0.04	0.01
46	->	78	-0.03	0.00
46	->	79	-0.00	0.00
46	->	80	-0.00	0.00
46	->	81	-0.02	0.00

46	->	82	-0.00	0.00
46	->	83	-0.00	0.00
46	->	84	-0.00	0.00
46	->	85	-0.00	0.00
46	->	86	0.00	0.00
46	->	87	0.02	0.00
46	->	88	0.02	0.01
46	->	89	0.03	0.01
46	->	90	-0.01	0.04
46	->	91	0.12	0.11
46	->	92	0.37	0.25
46	->	93	0.13	0.15
46	->	94	0.02	0.02
46	->	95	0.02	0.01
46	->	96	0.02	0.00
46	->	97	0.01	0.00
46	->	98	0.10	0.04
46	->	99	0.04	0.06
46	->	100	-1.52	0.48
46	->	101	-3.84	1.58
46	->	102	-1.68	1.87
46	->	103	-0.11	0.15
46	->	104	0.01	0.01
46	->	105	0.02	0.00
46	->	106	0.01	0.00
46	->	107	0.01	0.00
47	->	1	0.00	0.00
47	->	2	0.00	0.00
47	->	3	-0.00	0.00
47	->	4	0.00	0.00
47	->	5	-0.00	0.00
47	->	6	0.00	0.00
47	->	7	-0.00	0.00
47	->	8	-0.00	0.00
47	->	9	-0.00	0.00
47	->	10	-0.00	0.00
47	->	11	-0.00	0.00
47	->	12	0.00	0.00
47	->	13	0.00	0.00
47	->	14	-0.00	0.00
47	->	15	0.00	0.00
47	->	16	0.00	0.00
47	->	17	-0.00	0.00
47	->	18	-0.01	0.00
47	->	19	-0.00	0.00
47	->	20	-0.00	0.00
47	->	21	-0.01	0.01
47	->	22	-0.09	0.06
47	->	23	-0.00	0.00
47	->	24	-0.00	0.00

47 ->	25	-0.08	0.05
47 ->	26	-0.03	0.03
47 ->	27	-0.00	0.00
47 ->	28	-0.00	0.00
47 ->	29	-0.00	0.00
47 ->	30	-0.01	0.00
47 ->	31	-0.03	0.04
47 ->	32	-0.01	0.01
47 ->	33	-0.10	0.04
47 ->	34	-0.00	0.02
47 ->	35	-0.11	0.02
47 ->	36	-0.01	0.00
47 ->	37	0.00	0.00
47 ->	38	-0.01	0.01
47 ->	39	0.00	0.00
47 ->	40	-0.00	0.02
47 ->	41	0.06	0.04
47 ->	42	-2.42	0.41
47 ->	43	-0.99	0.77
47 ->	44	-4.36	1.42
47 ->	45	-0.93	0.17
47 ->	46	-13.16	0.73
47 ->	47	19.46	1.00
47 ->	48	-6.51	0.55
47 ->	49	-0.80	0.15
47 ->	50	-1.46	0.34
47 ->	51	-1.52	0.66
47 ->	52	-0.00	0.03
47 ->	53	-0.01	0.02
47 ->	54	-0.02	0.01
47 ->	55	-0.03	0.01
47 ->	56	-0.00	0.00
47 ->	57	-0.00	0.00
47 ->	58	0.00	0.00
47 ->	59	-0.00	0.00
47 ->	60	-0.00	0.00
47 ->	61	-0.00	0.00
47 ->	62	0.00	0.00
47 ->	63	0.00	0.00
47 ->	64	0.00	0.00
47 ->	65	-0.00	0.00
47 ->	66	0.00	0.00
47 ->	67	0.00	0.00
47 ->	68	-0.00	0.00
47 ->	69	-0.00	0.00
47 ->	70	-0.00	0.00
47 ->	71	-0.01	0.01
47 ->	72	-0.12	0.06
47 ->	73	-0.00	0.00
47 ->	74	-0.01	0.01

47	->	75	-0.01	0.00
47	->	76	-0.00	0.00
47	->	77	-0.00	0.00
47	->	78	-0.00	0.00
47	->	79	-0.00	0.00
47	->	80	0.00	0.00
47	->	81	0.00	0.00
47	->	82	0.00	0.00
47	->	83	0.00	0.00
47	->	84	-0.00	0.00
47	->	85	0.00	0.00
47	->	86	-0.00	0.00
47	->	87	-0.01	0.01
47	->	88	-0.05	0.03
47	->	89	-0.19	0.08
47	->	90	-1.49	0.60
47	->	91	-2.98	0.62
47	->	92	-0.41	0.44
47	->	93	-0.05	0.03
47	->	94	-0.01	0.00
47	->	95	-0.00	0.00
47	->	96	-0.00	0.00
47	->	97	-0.00	0.00
47	->	98	0.01	0.01
47	->	99	-0.02	0.02
47	->	100	-0.08	0.02
47	->	101	-0.20	0.07
47	->	102	-0.18	0.16
47	->	103	-0.06	0.05
47	->	104	-0.04	0.03
47	->	105	-0.01	0.00
47	->	106	-0.01	0.00
47	->	107	-0.00	0.00
48	->	1	0.00	0.00
48	->	2	0.00	0.00
48	->	3	0.00	0.00
48	->	4	0.00	0.00
48	->	5	-0.00	0.00
48	->	6	0.00	0.00
48	->	7	0.00	0.00
48	->	8	-0.00	0.00
48	->	9	0.00	0.00
48	->	10	-0.00	0.00
48	->	11	-0.00	0.00
48	->	12	0.00	0.00
48	->	13	0.00	0.00
48	->	14	0.00	0.00
48	->	15	0.00	0.00
48	->	16	0.00	0.00
48	->	17	-0.00	0.00

48	->	18	-0.00	0.00
48	->	19	-0.00	0.00
48	->	20	-0.00	0.00
48	->	21	-0.00	0.00
48	->	22	-0.01	0.00
48	->	23	-0.00	0.00
48	->	24	-0.00	0.00
48	->	25	-0.01	0.01
48	->	26	-0.00	0.00
48	->	27	0.00	0.00
48	->	28	-0.00	0.00
48	->	29	-0.00	0.00
48	->	30	-0.01	0.00
48	->	31	-0.04	0.01
48	->	32	-0.05	0.01
48	->	33	-1.65	0.36
48	->	34	-1.37	0.26
48	->	35	-1.07	0.25
48	->	36	-0.14	0.11
48	->	37	-0.04	0.01
48	->	38	-0.01	0.01
48	->	39	-0.02	0.01
48	->	40	-0.10	0.04
48	->	41	-0.12	0.04
48	->	42	-2.58	0.37
48	->	43	-0.03	0.01
48	->	44	-0.84	0.45
48	->	45	-2.31	0.40
48	->	46	-1.37	0.20
48	->	47	-6.56	0.55
48	->	48	22.00	1.34
48	->	49	-6.35	0.52
48	->	50	-0.94	0.12
48	->	51	-2.86	0.47
48	->	52	-3.85	0.58
48	->	53	-0.21	0.07
48	->	54	-0.09	0.04
48	->	55	-0.08	0.03
48	->	56	-0.02	0.01
48	->	57	-0.00	0.00
48	->	58	-0.00	0.00
48	->	59	-0.01	0.00
48	->	60	-0.00	0.00
48	->	61	-0.00	0.00
48	->	62	-0.00	0.00
48	->	63	0.00	0.00
48	->	64	0.00	0.00
48	->	65	0.00	0.00
48	->	66	0.00	0.00
48	->	67	0.00	0.00

48	->	68	-0.00	0.00
48	->	69	-0.00	0.00
48	->	70	-0.00	0.00
48	->	71	-0.01	0.01
48	->	72	-0.02	0.01
48	->	73	-0.00	0.00
48	->	74	-0.00	0.00
48	->	75	0.00	0.00
48	->	76	-0.00	0.00
48	->	77	-0.00	0.00
48	->	78	-0.00	0.00
48	->	79	-0.00	0.00
48	->	80	0.00	0.00
48	->	81	-0.00	0.00
48	->	82	0.00	0.00
48	->	83	0.00	0.00
48	->	84	0.00	0.00
48	->	85	-0.00	0.00
48	->	86	-0.00	0.00
48	->	87	0.00	0.00
48	->	88	0.00	0.01
48	->	89	0.00	0.01
48	->	90	-0.01	0.01
48	->	91	0.00	0.00
48	->	92	-0.00	0.00
48	->	93	-0.00	0.00
48	->	94	-0.00	0.00
48	->	95	-0.00	0.00
48	->	96	-0.00	0.00
48	->	97	-0.00	0.00
48	->	98	-0.01	0.00
48	->	99	-0.04	0.01
48	->	100	-0.10	0.02
48	->	101	-0.02	0.01
48	->	102	-0.01	0.00
48	->	103	-0.00	0.00
48	->	104	-0.00	0.00
48	->	105	-0.00	0.00
48	->	106	-0.00	0.00
48	->	107	-0.00	0.00
49	->	1	0.00	0.00
49	->	2	0.00	0.00
49	->	3	0.00	0.00
49	->	4	-0.00	0.00
49	->	5	-0.00	0.00
49	->	6	0.00	0.00
49	->	7	0.00	0.00
49	->	8	-0.00	0.00
49	->	9	0.00	0.00
49	->	10	-0.00	0.00

49	->	11	-0.00	0.00
49	->	12	0.00	0.00
49	->	13	0.00	0.00
49	->	14	0.00	0.00
49	->	15	0.00	0.00
49	->	16	0.00	0.00
49	->	17	-0.00	0.00
49	->	18	-0.00	0.00
49	->	19	-0.00	0.00
49	->	20	-0.00	0.00
49	->	21	-0.00	0.00
49	->	22	0.00	0.00
49	->	23	0.00	0.00
49	->	24	0.00	0.00
49	->	25	0.00	0.00
49	->	26	-0.00	0.00
49	->	27	0.00	0.00
49	->	28	0.00	0.00
49	->	29	0.00	0.00
49	->	30	-0.00	0.00
49	->	31	-0.00	0.00
49	->	32	-0.01	0.00
49	->	33	-0.05	0.02
49	->	34	-0.01	0.01
49	->	35	0.01	0.01
49	->	36	-0.02	0.04
49	->	37	-0.01	0.00
49	->	38	-0.00	0.00
49	->	39	-0.00	0.00
49	->	40	-0.01	0.00
49	->	41	-0.01	0.01
49	->	42	-0.07	0.01
49	->	43	-0.00	0.00
49	->	44	0.01	0.05
49	->	45	-3.33	1.36
49	->	46	-2.85	0.46
49	->	47	-0.81	0.15
49	->	48	-6.36	0.52
49	->	49	-20.99	2.23
49	->	50	-8.13	0.47
49	->	51	-0.80	0.19
49	->	52	-2.44	0.68
49	->	53	-3.14	0.82
49	->	54	-0.13	0.06
49	->	55	-0.04	0.02
49	->	56	-0.02	0.01
49	->	57	-0.01	0.00
49	->	58	-0.02	0.02
49	->	59	-0.00	0.01
49	->	60	-0.02	0.01

49	->	61	-0.02	0.01
49	->	62	-0.00	0.00
49	->	63	0.00	0.00
49	->	64	0.00	0.00
49	->	65	0.00	0.00
49	->	66	0.00	0.00
49	->	67	0.00	0.00
49	->	68	-0.00	0.00
49	->	69	-0.00	0.00
49	->	70	0.00	0.00
49	->	71	-0.06	0.04
49	->	72	0.02	0.02
49	->	73	-0.02	0.01
49	->	74	-0.02	0.01
49	->	75	-0.01	0.01
49	->	76	-0.00	0.00
49	->	77	0.00	0.00
49	->	78	0.00	0.00
49	->	79	-0.00	0.00
49	->	80	-0.00	0.00
49	->	81	0.00	0.00
49	->	82	0.00	0.00
49	->	83	0.00	0.00
49	->	84	0.00	0.00
49	->	85	0.00	0.00
49	->	86	-0.00	0.00
49	->	87	-0.01	0.01
49	->	88	-0.02	0.01
49	->	89	-0.02	0.02
49	->	90	-0.01	0.00
49	->	91	-0.01	0.00
49	->	92	-0.01	0.00
49	->	93	-0.00	0.00
49	->	94	-0.00	0.00
49	->	95	-0.00	0.00
49	->	96	-0.00	0.00
49	->	97	-0.00	0.00
49	->	98	-0.01	0.00
49	->	99	-0.02	0.01
49	->	100	-0.20	0.22
49	->	101	-0.14	0.12
49	->	102	-0.01	0.01
49	->	103	-0.00	0.00
49	->	104	-0.00	0.00
49	->	105	-0.00	0.00
49	->	106	-0.00	0.00
49	->	107	-0.00	0.00
50	->	1	0.00	0.00
50	->	2	0.00	0.00
50	->	3	-0.00	0.00

50	->	4	-0.00	0.00
50	->	5	-0.00	0.00
50	->	6	0.00	0.00
50	->	7	0.00	0.00
50	->	8	-0.00	0.00
50	->	9	0.00	0.00
50	->	10	-0.00	0.00
50	->	11	-0.00	0.00
50	->	12	0.00	0.00
50	->	13	0.00	0.00
50	->	14	0.00	0.00
50	->	15	0.00	0.00
50	->	16	0.00	0.00
50	->	17	-0.00	0.00
50	->	18	-0.00	0.00
50	->	19	-0.00	0.00
50	->	20	-0.00	0.00
50	->	21	-0.00	0.00
50	->	22	0.00	0.00
50	->	23	0.00	0.00
50	->	24	0.00	0.00
50	->	25	0.00	0.00
50	->	26	-0.00	0.00
50	->	27	0.00	0.00
50	->	28	0.00	0.00
50	->	29	0.00	0.00
50	->	30	-0.00	0.00
50	->	31	-0.00	0.00
50	->	32	-0.00	0.00
50	->	33	-0.01	0.00
50	->	34	-0.00	0.00
50	->	35	-0.04	0.02
50	->	36	-0.01	0.00
50	->	37	-0.00	0.00
50	->	38	-0.01	0.01
50	->	39	-0.00	0.00
50	->	40	-0.00	0.01
50	->	41	-0.00	0.01
50	->	42	-0.09	0.04
50	->	43	-0.01	0.00
50	->	44	-0.05	0.03
50	->	45	-0.10	0.05
50	->	46	-2.20	1.08
50	->	47	-1.45	0.34
50	->	48	-0.94	0.11
50	->	49	-8.15	0.47
50	->	50	-22.42	1.98
50	->	51	-22.15	0.40
50	->	52	-0.86	0.15
50	->	53	-3.27	0.55

50	->	54	-1.61	0.97
50	->	55	-0.10	0.05
50	->	56	-0.04	0.02
50	->	57	-0.02	0.01
50	->	58	-0.03	0.02
50	->	59	-0.12	0.10
50	->	60	-0.06	0.08
50	->	61	-0.05	0.03
50	->	62	-0.00	0.00
50	->	63	-0.00	0.00
50	->	64	0.00	0.00
50	->	65	0.00	0.00
50	->	66	0.00	0.00
50	->	67	0.00	0.00
50	->	68	-0.00	0.00
50	->	69	-0.01	0.01
50	->	70	-0.04	0.02
50	->	71	-1.07	0.54
50	->	72	-1.44	0.37
50	->	73	-0.04	0.04
50	->	74	-0.05	0.05
50	->	75	-0.04	0.03
50	->	76	-0.00	0.00
50	->	77	0.00	0.00
50	->	78	-0.00	0.00
50	->	79	-0.00	0.00
50	->	80	-0.00	0.00
50	->	81	0.00	0.00
50	->	82	-0.00	0.00
50	->	83	0.00	0.00
50	->	84	0.00	0.00
50	->	85	0.00	0.00
50	->	86	-0.00	0.00
50	->	87	-0.01	0.01
50	->	88	-0.09	0.09
50	->	89	-0.11	0.09
50	->	90	-0.11	0.22
50	->	91	-0.02	0.01
50	->	92	-0.01	0.00
50	->	93	-0.01	0.00
50	->	94	-0.00	0.00
50	->	95	-0.00	0.00
50	->	96	-0.00	0.00
50	->	97	-0.00	0.00
50	->	98	-0.00	0.00
50	->	99	-0.01	0.00
50	->	100	-0.02	0.02
50	->	101	-0.04	0.02
50	->	102	-0.04	0.03
50	->	103	-0.03	0.01

50	->	104	-0.01	0.01
50	->	105	-0.00	0.00
50	->	106	-0.00	0.00
50	->	107	-0.00	0.00
51	->	1	0.00	0.00
51	->	2	0.00	0.00
51	->	3	0.00	0.00
51	->	4	0.00	0.00
51	->	5	-0.00	0.00
51	->	6	0.00	0.00
51	->	7	0.00	0.00
51	->	8	-0.00	0.00
51	->	9	-0.00	0.00
51	->	10	-0.00	0.00
51	->	11	-0.00	0.00
51	->	12	0.00	0.00
51	->	13	0.00	0.00
51	->	14	0.00	0.00
51	->	15	0.00	0.00
51	->	16	0.00	0.00
51	->	17	-0.00	0.00
51	->	18	-0.00	0.00
51	->	19	-0.00	0.00
51	->	20	-0.00	0.00
51	->	21	-0.00	0.00
51	->	22	0.00	0.00
51	->	23	0.00	0.00
51	->	24	0.00	0.00
51	->	25	0.00	0.00
51	->	26	-0.00	0.00
51	->	27	0.00	0.00
51	->	28	0.00	0.00
51	->	29	-0.00	0.00
51	->	30	-0.00	0.00
51	->	31	-0.00	0.00
51	->	32	-0.00	0.00
51	->	33	-0.02	0.01
51	->	34	-0.06	0.02
51	->	35	1.52	0.29
51	->	36	-0.06	0.03
51	->	37	-0.46	0.11
51	->	38	1.45	0.35
51	->	39	-0.04	0.04
51	->	40	-0.46	0.44
51	->	41	-0.03	0.02
51	->	42	-2.03	0.39
51	->	43	-0.01	0.00
51	->	44	-0.00	0.01
51	->	45	-0.05	0.03
51	->	46	-0.19	0.11

51	->	47	-1.56	0.67
51	->	48	-2.86	0.47
51	->	49	-0.81	0.19
51	->	50	-22.20	0.40
51	->	51	9.30	1.35
51	->	52	-10.21	0.51
51	->	53	-1.34	0.27
51	->	54	-3.38	0.76
51	->	55	19.18	10.25
51	->	56	-0.22	0.06
51	->	57	-0.04	0.02
51	->	58	-0.03	0.01
51	->	59	-0.03	0.03
51	->	60	-0.01	0.00
51	->	61	-0.00	0.00
51	->	62	-0.00	0.00
51	->	63	-0.00	0.00
51	->	64	-0.00	0.00
51	->	65	-0.00	0.00
51	->	66	-0.00	0.00
51	->	67	0.00	0.00
51	->	68	0.00	0.00
51	->	69	-0.00	0.00
51	->	70	-0.01	0.01
51	->	71	-0.06	0.04
51	->	72	-0.07	0.04
51	->	73	0.00	0.00
51	->	74	-0.00	0.00
51	->	75	-0.01	0.01
51	->	76	0.00	0.00
51	->	77	0.00	0.00
51	->	78	0.00	0.00
51	->	79	-0.00	0.00
51	->	80	0.00	0.00
51	->	81	0.00	0.00
51	->	82	0.00	0.00
51	->	83	0.00	0.00
51	->	84	0.00	0.00
51	->	85	0.00	0.00
51	->	86	-0.00	0.00
51	->	87	-0.03	0.02
51	->	88	-0.38	0.32
51	->	89	-1.50	2.17
51	->	90	-0.04	0.03
51	->	91	-0.01	0.00
51	->	92	-0.01	0.00
51	->	93	-0.00	0.00
51	->	94	-0.00	0.00
51	->	95	-0.00	0.00
51	->	96	-0.00	0.00

51	->	97	-0.00	0.00
51	->	98	-0.00	0.00
51	->	99	-0.01	0.00
51	->	100	-0.01	0.00
51	->	101	-0.01	0.00
51	->	102	-0.01	0.00
51	->	103	-0.00	0.00
51	->	104	-0.01	0.00
51	->	105	-0.00	0.00
51	->	106	-0.00	0.00
51	->	107	-0.00	0.00
52	->	1	0.00	0.00
52	->	2	0.00	0.00
52	->	3	0.00	0.00
52	->	4	-0.00	0.00
52	->	5	-0.00	0.00
52	->	6	0.00	0.00
52	->	7	-0.00	0.00
52	->	8	-0.00	0.00
52	->	9	0.00	0.00
52	->	10	-0.00	0.00
52	->	11	-0.00	0.00
52	->	12	0.00	0.00
52	->	13	0.00	0.00
52	->	14	0.00	0.00
52	->	15	0.00	0.00
52	->	16	0.00	0.00
52	->	17	-0.00	0.00
52	->	18	-0.00	0.00
52	->	19	-0.00	0.00
52	->	20	-0.00	0.00
52	->	21	-0.00	0.00
52	->	22	0.00	0.00
52	->	23	0.00	0.00
52	->	24	-0.00	0.00
52	->	25	0.00	0.00
52	->	26	0.00	0.00
52	->	27	0.00	0.00
52	->	28	0.00	0.00
52	->	29	-0.00	0.00
52	->	30	-0.00	0.00
52	->	31	-0.00	0.00
52	->	32	-0.00	0.00
52	->	33	-0.02	0.01
52	->	34	-0.05	0.02
52	->	35	-0.19	0.23
52	->	36	-0.36	0.58
52	->	37	-0.51	0.39
52	->	38	0.01	0.01
52	->	39	-0.01	0.00

52	->	40	-0.02	0.01
52	->	41	-0.01	0.00
52	->	42	-0.05	0.02
52	->	43	-0.00	0.00
52	->	44	0.00	0.01
52	->	45	-0.05	0.02
52	->	46	-0.07	0.04
52	->	47	-0.00	0.03
52	->	48	-3.83	0.57
52	->	49	-2.45	0.69
52	->	50	-0.87	0.15
52	->	51	-10.24	0.51
52	->	52	22.70	1.86
52	->	53	-10.43	0.47
52	->	54	-1.09	0.23
52	->	55	-2.61	0.35
52	->	56	-5.11	1.35
52	->	57	-0.16	0.10
52	->	58	-0.29	0.12
52	->	59	-0.01	0.02
52	->	60	-0.01	0.00
52	->	61	-0.01	0.00
52	->	62	-0.00	0.00
52	->	63	0.00	0.00
52	->	64	0.00	0.00
52	->	65	0.00	0.00
52	->	66	0.00	0.00
52	->	67	0.00	0.00
52	->	68	-0.00	0.00
52	->	69	-0.00	0.00
52	->	70	-0.00	0.00
52	->	71	-0.02	0.01
52	->	72	-0.02	0.01
52	->	73	-0.00	0.00
52	->	74	-0.00	0.00
52	->	75	-0.00	0.00
52	->	76	-0.00	0.00
52	->	77	0.00	0.00
52	->	78	-0.00	0.00
52	->	79	-0.00	0.00
52	->	80	0.00	0.00
52	->	81	0.00	0.00
52	->	82	0.00	0.00
52	->	83	0.00	0.00
52	->	84	0.00	0.00
52	->	85	-0.00	0.00
52	->	86	-0.00	0.00
52	->	87	0.00	0.01
52	->	88	0.01	0.02
52	->	89	0.02	0.03

52	->	90	-0.00	0.00
52	->	91	-0.00	0.00
52	->	92	-0.00	0.00
52	->	93	-0.00	0.00
52	->	94	-0.00	0.00
52	->	95	-0.00	0.00
52	->	96	-0.00	0.00
52	->	97	-0.00	0.00
52	->	98	-0.00	0.00
52	->	99	-0.01	0.00
52	->	100	-0.02	0.01
52	->	101	-0.01	0.00
52	->	102	-0.00	0.00
52	->	103	-0.00	0.00
52	->	104	-0.00	0.00
52	->	105	-0.00	0.00
52	->	106	-0.00	0.00
52	->	107	-0.00	0.00
53	->	1	0.01	0.00
53	->	2	0.00	0.00
53	->	3	-0.00	0.00
53	->	4	-0.00	0.00
53	->	5	-0.00	0.00
53	->	6	0.00	0.00
53	->	7	-0.00	0.00
53	->	8	-0.00	0.00
53	->	9	0.00	0.00
53	->	10	-0.00	0.00
53	->	11	-0.00	0.00
53	->	12	0.01	0.00
53	->	13	0.00	0.00
53	->	14	0.00	0.00
53	->	15	0.00	0.00
53	->	16	0.01	0.00
53	->	17	-0.00	0.00
53	->	18	-0.00	0.00
53	->	19	-0.01	0.00
53	->	20	-0.00	0.00
53	->	21	0.00	0.00
53	->	22	0.01	0.00
53	->	23	0.00	0.00
53	->	24	0.00	0.00
53	->	25	0.02	0.00
53	->	26	0.00	0.00
53	->	27	0.00	0.00
53	->	28	0.00	0.00
53	->	29	0.00	0.00
53	->	30	-0.00	0.00
53	->	31	0.02	0.00
53	->	32	-0.00	0.00

53	->	33	-0.00	0.00
53	->	34	-0.01	0.00
53	->	35	-0.05	0.01
53	->	36	0.02	0.01
53	->	37	-0.01	0.01
53	->	38	-0.04	0.00
53	->	39	-0.00	0.00
53	->	40	0.02	0.01
53	->	41	-0.00	0.00
53	->	42	-0.02	0.00
53	->	43	-0.00	0.00
53	->	44	0.04	0.01
53	->	45	-0.02	0.01
53	->	46	-0.17	0.06
53	->	47	-0.01	0.02
53	->	48	-0.21	0.07
53	->	49	-3.17	0.83
53	->	50	-3.29	0.55
53	->	51	-1.35	0.27
53	->	52	-10.47	0.47
53	->	53	-105.89	3.12
53	->	54	-5.30	0.61
53	->	55	-0.69	0.15
53	->	56	-1.51	0.42
53	->	57	-1.18	0.31
53	->	58	-4.81	2.35
53	->	59	-0.40	0.48
53	->	60	-1.60	0.50
53	->	61	-0.46	0.40
53	->	62	-0.01	0.00
53	->	63	-0.03	0.00
53	->	64	-0.02	0.00
53	->	65	-0.00	0.00
53	->	66	-0.02	0.00
53	->	67	-0.00	0.00
53	->	68	0.03	0.01
53	->	69	0.09	0.03
53	->	70	0.01	0.06
53	->	71	-2.26	2.23
53	->	72	-0.19	0.25
53	->	73	-0.13	0.16
53	->	74	-0.09	0.06
53	->	75	-0.10	0.08
53	->	76	0.03	0.01
53	->	77	0.03	0.01
53	->	78	0.02	0.01
53	->	79	0.00	0.00
53	->	80	0.00	0.00
53	->	81	0.01	0.00
53	->	82	0.00	0.00

53	->	83	-0.00	0.00
53	->	84	0.00	0.00
53	->	85	0.00	0.00
53	->	86	-0.01	0.01
53	->	87	-0.10	0.08
53	->	88	-0.23	0.17
53	->	89	-0.09	0.08
53	->	90	-0.03	0.01
53	->	91	-0.02	0.01
53	->	92	-0.02	0.00
53	->	93	-0.01	0.00
53	->	94	-0.01	0.00
53	->	95	-0.02	0.00
53	->	96	-0.01	0.00
53	->	97	-0.00	0.00
53	->	98	-0.01	0.00
53	->	99	-0.03	0.01
53	->	100	-0.14	0.20
53	->	101	-0.38	1.22
53	->	102	-0.07	0.06
53	->	103	-0.02	0.01
53	->	104	-0.02	0.00
53	->	105	-0.01	0.00
53	->	106	-0.01	0.00
53	->	107	-0.01	0.00
54	->	1	0.00	0.00
54	->	2	0.00	0.00
54	->	3	0.00	0.00
54	->	4	0.00	0.00
54	->	5	-0.00	0.00
54	->	6	0.00	0.00
54	->	7	0.00	0.00
54	->	8	-0.00	0.00
54	->	9	0.00	0.00
54	->	10	-0.00	0.00
54	->	11	-0.00	0.00
54	->	12	0.00	0.00
54	->	13	0.00	0.00
54	->	14	0.00	0.00
54	->	15	0.00	0.00
54	->	16	0.00	0.00
54	->	17	-0.00	0.00
54	->	18	-0.00	0.00
54	->	19	-0.00	0.00
54	->	20	-0.00	0.00
54	->	21	-0.00	0.00
54	->	22	0.00	0.00
54	->	23	0.00	0.00
54	->	24	0.00	0.00
54	->	25	0.00	0.00

54	->	26	0.00	0.00
54	->	27	0.00	0.00
54	->	28	-0.00	0.00
54	->	29	0.00	0.00
54	->	30	-0.00	0.00
54	->	31	0.00	0.00
54	->	32	-0.00	0.00
54	->	33	-0.00	0.00
54	->	34	-0.00	0.00
54	->	35	-0.08	0.04
54	->	36	-0.00	0.00
54	->	37	-0.01	0.01
54	->	38	-0.06	0.02
54	->	39	-0.00	0.00
54	->	40	-0.01	0.04
54	->	41	-0.00	0.00
54	->	42	-0.02	0.01
54	->	43	-0.00	0.00
54	->	44	0.00	0.00
54	->	45	-0.00	0.00
54	->	46	-0.01	0.00
54	->	47	-0.02	0.01
54	->	48	-0.09	0.04
54	->	49	-0.13	0.06
54	->	50	-1.62	0.97
54	->	51	-3.40	0.77
54	->	52	-1.10	0.23
54	->	53	-5.33	0.61
54	->	54	-22.62	2.14
54	->	55	-22.25	0.40
54	->	56	-0.60	0.18
54	->	57	-1.15	0.34
54	->	58	-0.24	0.12
54	->	59	-1.40	1.38
54	->	60	-0.03	0.02
54	->	61	-0.00	0.00
54	->	62	-0.00	0.00
54	->	63	0.00	0.00
54	->	64	0.00	0.00
54	->	65	0.00	0.00
54	->	66	0.00	0.00
54	->	67	-0.00	0.00
54	->	68	-0.02	0.03
54	->	69	-0.05	0.03
54	->	70	-0.10	0.06
54	->	71	-0.62	0.35
54	->	72	-0.08	0.09
54	->	73	0.00	0.00
54	->	74	-0.00	0.00
54	->	75	0.02	0.04

54	->	76	-0.00	0.00
54	->	77	-0.00	0.00
54	->	78	-0.00	0.00
54	->	79	-0.00	0.00
54	->	80	0.00	0.00
54	->	81	0.00	0.00
54	->	82	-0.00	0.00
54	->	83	0.00	0.00
54	->	84	0.00	0.00
54	->	85	-0.00	0.00
54	->	86	-0.00	0.00
54	->	87	-0.10	0.13
54	->	88	-1.34	1.39
54	->	89	-0.32	0.77
54	->	90	-0.01	0.01
54	->	91	-0.00	0.00
54	->	92	-0.00	0.00
54	->	93	-0.00	0.00
54	->	94	-0.00	0.00
54	->	95	-0.00	0.00
54	->	96	-0.00	0.00
54	->	97	-0.00	0.00
54	->	98	-0.00	0.00
54	->	99	-0.00	0.00
54	->	100	-0.00	0.00
54	->	101	-0.00	0.00
54	->	102	-0.00	0.00
54	->	103	-0.00	0.00
54	->	104	-0.00	0.00
54	->	105	-0.00	0.00
54	->	106	-0.00	0.00
54	->	107	-0.00	0.00
55	->	1	0.00	0.00
55	->	2	0.00	0.00
55	->	3	0.00	0.00
55	->	4	0.00	0.00
55	->	5	0.00	0.00
55	->	6	0.00	0.00
55	->	7	0.00	0.00
55	->	8	0.00	0.00
55	->	9	0.00	0.00
55	->	10	0.00	0.00
55	->	11	0.00	0.00
55	->	12	-0.00	0.00
55	->	13	-0.00	0.00
55	->	14	0.00	0.00
55	->	15	0.00	0.00
55	->	16	0.00	0.00
55	->	17	0.00	0.00
55	->	18	0.00	0.00

55	->	19	-0.00	0.00
55	->	20	0.00	0.00
55	->	21	0.00	0.00
55	->	22	-0.00	0.00
55	->	23	0.00	0.00
55	->	24	0.00	0.00
55	->	25	-0.00	0.00
55	->	26	0.00	0.00
55	->	27	0.00	0.00
55	->	28	0.00	0.00
55	->	29	0.00	0.00
55	->	30	-0.00	0.00
55	->	31	-0.01	0.00
55	->	32	-0.00	0.00
55	->	33	-0.01	0.00
55	->	34	-0.06	0.02
55	->	35	3.38	0.63
55	->	36	-0.23	0.07
55	->	37	-3.72	0.43
55	->	38	2.27	0.73
55	->	39	-0.02	0.06
55	->	40	-0.14	0.17
55	->	41	-0.02	0.01
55	->	42	-0.01	0.01
55	->	43	0.00	0.00
55	->	44	-0.00	0.00
55	->	45	-0.00	0.00
55	->	46	-0.01	0.00
55	->	47	-0.03	0.01
55	->	48	-0.08	0.03
55	->	49	-0.04	0.02
55	->	50	-0.10	0.05
55	->	51	19.15	10.26
55	->	52	-2.60	0.35
55	->	53	-0.70	0.15
55	->	54	-22.34	0.41
55	->	55	4.75	1.22
55	->	56	-5.03	0.46
55	->	57	-0.49	0.14
55	->	58	-0.05	0.03
55	->	59	-0.10	0.07
55	->	60	-0.00	0.00
55	->	61	-0.00	0.00
55	->	62	-0.00	0.00
55	->	63	-0.00	0.00
55	->	64	-0.00	0.00
55	->	65	-0.00	0.00
55	->	66	-0.00	0.00
55	->	67	-0.00	0.00
55	->	68	-0.00	0.00

55	->	69	-0.01	0.01
55	->	70	-0.01	0.00
55	->	71	-0.01	0.01
55	->	72	-0.01	0.00
55	->	73	0.00	0.00
55	->	74	-0.00	0.00
55	->	75	-0.00	0.00
55	->	76	0.00	0.00
55	->	77	0.00	0.00
55	->	78	0.00	0.00
55	->	79	0.00	0.00
55	->	80	0.00	0.00
55	->	81	0.00	0.00
55	->	82	0.00	0.00
55	->	83	-0.00	0.00
55	->	84	-0.00	0.00
55	->	85	0.00	0.00
55	->	86	-0.00	0.00
55	->	87	0.01	0.01
55	->	88	0.03	0.03
55	->	89	0.00	0.04
55	->	90	-0.00	0.00
55	->	91	-0.00	0.00
55	->	92	-0.00	0.00
55	->	93	0.00	0.00
55	->	94	-0.00	0.00
55	->	95	-0.00	0.00
55	->	96	-0.00	0.00
55	->	97	-0.00	0.00
55	->	98	0.00	0.00
55	->	99	-0.00	0.00
55	->	100	-0.00	0.00
55	->	101	-0.00	0.00
55	->	102	-0.00	0.00
55	->	103	-0.00	0.00
55	->	104	-0.00	0.00
55	->	105	-0.00	0.00
55	->	106	-0.00	0.00
55	->	107	-0.00	0.00
56	->	1	0.00	0.00
56	->	2	0.00	0.00
56	->	3	0.00	0.00
56	->	4	0.00	0.00
56	->	5	-0.00	0.00
56	->	6	0.00	0.00
56	->	7	0.00	0.00
56	->	8	-0.00	0.00
56	->	9	0.00	0.00
56	->	10	-0.00	0.00
56	->	11	-0.00	0.00

56	->	12	0.00	0.00
56	->	13	0.00	0.00
56	->	14	0.00	0.00
56	->	15	0.00	0.00
56	->	16	0.00	0.00
56	->	17	-0.00	0.00
56	->	18	-0.00	0.00
56	->	19	-0.00	0.00
56	->	20	-0.00	0.00
56	->	21	-0.00	0.00
56	->	22	0.00	0.00
56	->	23	0.00	0.00
56	->	24	-0.00	0.00
56	->	25	0.00	0.00
56	->	26	0.00	0.00
56	->	27	0.00	0.00
56	->	28	0.00	0.00
56	->	29	-0.00	0.00
56	->	30	0.00	0.00
56	->	31	0.00	0.00
56	->	32	-0.00	0.00
56	->	33	-0.00	0.00
56	->	34	-0.00	0.00
56	->	35	-0.01	0.01
56	->	36	-0.03	0.05
56	->	37	-0.18	0.18
56	->	38	-0.01	0.00
56	->	39	-0.00	0.00
56	->	40	0.00	0.00
56	->	41	-0.00	0.00
56	->	42	-0.00	0.00
56	->	43	-0.00	0.00
56	->	44	0.00	0.00
56	->	45	-0.00	0.00
56	->	46	-0.01	0.00
56	->	47	-0.00	0.00
56	->	48	-0.02	0.01
56	->	49	-0.02	0.01
56	->	50	-0.04	0.02
56	->	51	-0.22	0.06
56	->	52	-5.11	1.35
56	->	53	-1.51	0.42
56	->	54	-0.62	0.17
56	->	55	-5.18	0.47
56	->	56	-23.31	1.34
56	->	57	-11.64	0.52
56	->	58	-2.33	1.01
56	->	59	-0.05	0.02
56	->	60	-0.02	0.01
56	->	61	-0.00	0.00

56	->	62	-0.00	0.00
56	->	63	0.00	0.00
56	->	64	0.00	0.00
56	->	65	0.00	0.00
56	->	66	0.00	0.00
56	->	67	-0.00	0.00
56	->	68	-0.00	0.00
56	->	69	-0.01	0.01
56	->	70	-0.00	0.00
56	->	71	-0.01	0.01
56	->	72	-0.00	0.00
56	->	73	-0.00	0.00
56	->	74	-0.00	0.00
56	->	75	-0.00	0.00
56	->	76	-0.00	0.00
56	->	77	0.00	0.00
56	->	78	0.00	0.00
56	->	79	0.00	0.00
56	->	80	0.00	0.00
56	->	81	0.00	0.00
56	->	82	0.00	0.00
56	->	83	0.00	0.00
56	->	84	0.00	0.00
56	->	85	0.00	0.00
56	->	86	-0.00	0.00
56	->	87	-0.01	0.01
56	->	88	-0.02	0.01
56	->	89	-0.01	0.00
56	->	90	-0.00	0.00
56	->	91	-0.00	0.00
56	->	92	-0.00	0.00
56	->	93	-0.00	0.00
56	->	94	-0.00	0.00
56	->	95	-0.00	0.00
56	->	96	-0.00	0.00
56	->	97	-0.00	0.00
56	->	98	-0.00	0.00
56	->	99	-0.00	0.00
56	->	100	-0.00	0.00
56	->	101	-0.00	0.00
56	->	102	-0.00	0.00
56	->	103	-0.00	0.00
56	->	104	-0.00	0.00
56	->	105	-0.00	0.00
56	->	106	-0.00	0.00
56	->	107	-0.00	0.00
57	->	1	0.00	0.00
57	->	2	0.00	0.00
57	->	3	0.00	0.00
57	->	4	0.00	0.00

57	->	5	-0.00	0.00
57	->	6	0.00	0.00
57	->	7	0.00	0.00
57	->	8	-0.00	0.00
57	->	9	0.00	0.00
57	->	10	-0.00	0.00
57	->	11	-0.00	0.00
57	->	12	0.00	0.00
57	->	13	0.00	0.00
57	->	14	0.00	0.00
57	->	15	0.00	0.00
57	->	16	0.00	0.00
57	->	17	0.00	0.00
57	->	18	-0.00	0.00
57	->	19	-0.00	0.00
57	->	20	0.00	0.00
57	->	21	0.00	0.00
57	->	22	0.00	0.00
57	->	23	0.00	0.00
57	->	24	0.00	0.00
57	->	25	0.00	0.00
57	->	26	0.00	0.00
57	->	27	0.00	0.00
57	->	28	0.00	0.00
57	->	29	-0.00	0.00
57	->	30	-0.00	0.00
57	->	31	0.00	0.00
57	->	32	-0.00	0.00
57	->	33	0.00	0.00
57	->	34	-0.00	0.00
57	->	35	-0.00	0.00
57	->	36	-0.00	0.00
57	->	37	-0.01	0.00
57	->	38	-0.00	0.00
57	->	39	0.00	0.00
57	->	40	0.00	0.00
57	->	41	-0.00	0.00
57	->	42	-0.00	0.00
57	->	43	0.00	0.00
57	->	44	0.00	0.00
57	->	45	-0.00	0.00
57	->	46	-0.00	0.00
57	->	47	-0.00	0.00
57	->	48	-0.00	0.00
57	->	49	-0.01	0.00
57	->	50	-0.02	0.01
57	->	51	-0.04	0.02
57	->	52	-0.16	0.10
57	->	53	-1.18	0.31
57	->	54	-1.19	0.35

57	->	55	-0.51	0.13
57	->	56	-11.71	0.52
57	->	57	12.27	0.70
57	->	58	-14.92	0.45
57	->	59	-1.09	0.55
57	->	60	-0.04	0.01
57	->	61	-0.01	0.00
57	->	62	-0.00	0.00
57	->	63	0.00	0.00
57	->	64	0.00	0.00
57	->	65	0.00	0.00
57	->	66	0.00	0.00
57	->	67	-0.00	0.00
57	->	68	-0.00	0.00
57	->	69	-0.28	0.64
57	->	70	-0.00	0.01
57	->	71	-0.03	0.01
57	->	72	-0.01	0.00
57	->	73	-0.00	0.00
57	->	74	-0.00	0.00
57	->	75	-0.00	0.00
57	->	76	-0.00	0.00
57	->	77	0.00	0.00
57	->	78	0.00	0.00
57	->	79	-0.00	0.00
57	->	80	0.00	0.00
57	->	81	0.00	0.00
57	->	82	-0.00	0.00
57	->	83	0.00	0.00
57	->	84	0.00	0.00
57	->	85	-0.00	0.00
57	->	86	-0.00	0.00
57	->	87	-0.02	0.02
57	->	88	-0.05	0.12
57	->	89	-0.01	0.00
57	->	90	-0.00	0.00
57	->	91	-0.00	0.00
57	->	92	-0.00	0.00
57	->	93	-0.00	0.00
57	->	94	-0.00	0.00
57	->	95	-0.00	0.00
57	->	96	-0.00	0.00
57	->	97	-0.00	0.00
57	->	98	-0.00	0.00
57	->	99	-0.00	0.00
57	->	100	-0.00	0.00
57	->	101	-0.00	0.00
57	->	102	-0.00	0.00
57	->	103	-0.00	0.00
57	->	104	-0.00	0.00

57	->	105	-0.00	0.00
57	->	106	-0.00	0.00
57	->	107	-0.00	0.00
58	->	1	-0.00	0.00
58	->	2	-0.00	0.00
58	->	3	0.00	0.00
58	->	4	-0.00	0.00
58	->	5	0.00	0.00
58	->	6	-0.00	0.00
58	->	7	-0.00	0.00
58	->	8	0.00	0.00
58	->	9	-0.00	0.00
58	->	10	0.00	0.00
58	->	11	0.00	0.00
58	->	12	-0.00	0.00
58	->	13	-0.00	0.00
58	->	14	-0.00	0.00
58	->	15	-0.00	0.00
58	->	16	-0.00	0.00
58	->	17	0.00	0.00
58	->	18	0.00	0.00
58	->	19	0.00	0.00
58	->	20	0.00	0.00
58	->	21	-0.00	0.00
58	->	22	-0.01	0.00
58	->	23	-0.00	0.00
58	->	24	0.00	0.00
58	->	25	-0.01	0.00
58	->	26	-0.00	0.00
58	->	27	-0.00	0.00
58	->	28	-0.00	0.00
58	->	29	0.00	0.00
58	->	30	0.00	0.00
58	->	31	-0.01	0.00
58	->	32	0.00	0.00
58	->	33	-0.00	0.00
58	->	34	0.00	0.00
58	->	35	0.03	0.01
58	->	36	-0.04	0.02
58	->	37	-0.01	0.00
58	->	38	0.02	0.00
58	->	39	-0.00	0.00
58	->	40	-0.01	0.00
58	->	41	0.00	0.00
58	->	42	-0.00	0.00
58	->	43	-0.00	0.00
58	->	44	-0.02	0.00
58	->	45	0.00	0.00
58	->	46	0.03	0.01
58	->	47	0.00	0.00

58	->	48	-0.00	0.00
58	->	49	-0.02	0.02
58	->	50	-0.03	0.02
58	->	51	-0.03	0.01
58	->	52	-0.29	0.12
58	->	53	-4.80	2.35
58	->	54	-0.24	0.12
58	->	55	-0.05	0.03
58	->	56	-2.35	0.98
58	->	57	-15.06	0.45
58	->	58	-62.54	2.88
58	->	59	-12.25	0.70
58	->	60	-1.07	0.38
58	->	61	-0.07	0.03
58	->	62	-0.01	0.00
58	->	63	0.02	0.00
58	->	64	0.01	0.00
58	->	65	0.00	0.00
58	->	66	0.02	0.01
58	->	67	0.00	0.00
58	->	68	-0.02	0.01
58	->	69	-0.40	1.18
58	->	70	-0.16	0.13
58	->	71	-0.36	0.35
58	->	72	-0.03	0.01
58	->	73	-0.01	0.00
58	->	74	0.02	0.01
58	->	75	0.03	0.01
58	->	76	-0.03	0.01
58	->	77	-0.02	0.00
58	->	78	-0.02	0.00
58	->	79	-0.00	0.00
58	->	80	-0.00	0.00
58	->	81	-0.01	0.00
58	->	82	-0.00	0.00
58	->	83	0.00	0.00
58	->	84	-0.00	0.00
58	->	85	-0.00	0.00
58	->	86	0.00	0.00
58	->	87	0.02	0.01
58	->	88	0.03	0.02
58	->	89	0.02	0.01
58	->	90	0.01	0.00
58	->	91	0.01	0.00
58	->	92	0.01	0.00
58	->	93	0.00	0.00
58	->	94	0.01	0.00
58	->	95	0.01	0.00
58	->	96	0.01	0.00
58	->	97	0.00	0.00

58	->	98	0.01	0.00
58	->	99	0.01	0.00
58	->	100	0.03	0.01
58	->	101	0.03	0.01
58	->	102	0.02	0.01
58	->	103	0.01	0.00
58	->	104	0.01	0.00
58	->	105	0.00	0.00
58	->	106	0.00	0.00
58	->	107	0.00	0.00
59	->	1	0.00	0.00
59	->	2	0.00	0.00
59	->	3	-0.00	0.00
59	->	4	0.00	0.00
59	->	5	-0.00	0.00
59	->	6	0.00	0.00
59	->	7	0.00	0.00
59	->	8	-0.00	0.00
59	->	9	0.00	0.00
59	->	10	-0.00	0.00
59	->	11	-0.00	0.00
59	->	12	0.00	0.00
59	->	13	0.00	0.00
59	->	14	0.00	0.00
59	->	15	0.00	0.00
59	->	16	0.00	0.00
59	->	17	-0.00	0.00
59	->	18	-0.00	0.00
59	->	19	-0.01	0.00
59	->	20	-0.00	0.00
59	->	21	0.00	0.00
59	->	22	0.01	0.00
59	->	23	0.00	0.00
59	->	24	-0.00	0.00
59	->	25	0.01	0.00
59	->	26	0.00	0.00
59	->	27	0.00	0.00
59	->	28	0.00	0.00
59	->	29	0.00	0.00
59	->	30	-0.00	0.00
59	->	31	0.01	0.00
59	->	32	-0.00	0.00
59	->	33	-0.00	0.00
59	->	34	-0.00	0.00
59	->	35	-0.03	0.01
59	->	36	0.02	0.00
59	->	37	-0.00	0.00
59	->	38	-0.04	0.01
59	->	39	-0.00	0.00
59	->	40	0.03	0.01

59	->	41	-0.00	0.00
59	->	42	-0.00	0.00
59	->	43	0.00	0.00
59	->	44	0.02	0.00
59	->	45	-0.00	0.00
59	->	46	-0.04	0.01
59	->	47	-0.00	0.00
59	->	48	-0.01	0.00
59	->	49	-0.00	0.01
59	->	50	-0.12	0.10
59	->	51	-0.03	0.03
59	->	52	-0.01	0.02
59	->	53	-0.41	0.49
59	->	54	-1.40	1.39
59	->	55	-0.10	0.07
59	->	56	-0.05	0.02
59	->	57	-1.16	0.56
59	->	58	-12.30	0.70
59	->	59	13.45	4.02
59	->	60	-0.23	0.54
59	->	61	-1.03	0.28
59	->	62	-0.15	0.04
59	->	63	-0.08	0.01
59	->	64	-0.03	0.00
59	->	65	-0.00	0.00
59	->	66	-0.06	0.01
59	->	67	-0.01	0.01
59	->	68	0.26	0.21
59	->	69	-2.20	0.81
59	->	70	-2.76	0.81
59	->	71	-1.89	0.38
59	->	72	-0.04	0.04
59	->	73	-0.06	0.02
59	->	74	-0.07	0.01
59	->	75	-0.40	0.40
59	->	76	0.04	0.01
59	->	77	0.01	0.01
59	->	78	0.04	0.01
59	->	79	-0.00	0.00
59	->	80	-0.00	0.00
59	->	81	0.02	0.00
59	->	82	0.00	0.00
59	->	83	-0.00	0.00
59	->	84	0.00	0.00
59	->	85	0.00	0.00
59	->	86	-0.13	0.20
59	->	87	-1.35	1.67
59	->	88	-1.96	2.97
59	->	89	-0.07	0.03
59	->	90	-0.02	0.01

59	->	91	-0.02	0.00
59	->	92	-0.01	0.00
59	->	93	-0.01	0.00
59	->	94	-0.01	0.00
59	->	95	-0.01	0.00
59	->	96	-0.00	0.00
59	->	97	-0.00	0.00
59	->	98	-0.00	0.00
59	->	99	-0.01	0.00
59	->	100	-0.02	0.01
59	->	101	-0.02	0.01
59	->	102	-0.03	0.01
59	->	103	-0.02	0.00
59	->	104	-0.02	0.00
59	->	105	-0.01	0.00
59	->	106	-0.01	0.00
59	->	107	-0.01	0.00
60	->	1	0.00	0.00
60	->	2	0.00	0.00
60	->	3	0.00	0.00
60	->	4	0.00	0.00
60	->	5	-0.00	0.00
60	->	6	0.00	0.00
60	->	7	0.00	0.00
60	->	8	-0.00	0.00
60	->	9	0.00	0.00
60	->	10	-0.00	0.00
60	->	11	-0.00	0.00
60	->	12	0.00	0.00
60	->	13	0.00	0.00
60	->	14	0.00	0.00
60	->	15	0.00	0.00
60	->	16	0.00	0.00
60	->	17	-0.00	0.00
60	->	18	0.00	0.00
60	->	19	-0.00	0.00
60	->	20	0.00	0.00
60	->	21	0.00	0.00
60	->	22	0.00	0.00
60	->	23	0.00	0.00
60	->	24	0.00	0.00
60	->	25	0.00	0.00
60	->	26	0.00	0.00
60	->	27	0.00	0.00
60	->	28	0.00	0.00
60	->	29	0.00	0.00
60	->	30	-0.00	0.00
60	->	31	0.00	0.00
60	->	32	0.00	0.00
60	->	33	-0.00	0.00

60 ->	34	-0.00	0.00
60 ->	35	-0.00	0.00
60 ->	36	0.00	0.00
60 ->	37	-0.00	0.00
60 ->	38	-0.00	0.00
60 ->	39	0.00	0.00
60 ->	40	0.00	0.00
60 ->	41	-0.00	0.00
60 ->	42	-0.00	0.00
60 ->	43	0.00	0.00
60 ->	44	0.00	0.00
60 ->	45	-0.00	0.00
60 ->	46	-0.01	0.00
60 ->	47	-0.00	0.00
60 ->	48	-0.00	0.00
60 ->	49	-0.02	0.01
60 ->	50	-0.07	0.08
60 ->	51	-0.01	0.00
60 ->	52	-0.01	0.00
60 ->	53	-1.59	0.52
60 ->	54	-0.03	0.02
60 ->	55	-0.00	0.00
60 ->	56	-0.02	0.01
60 ->	57	-0.04	0.01
60 ->	58	-1.10	0.39
60 ->	59	-0.27	0.54
60 ->	60	1.89	0.98
60 ->	61	-10.70	0.55
60 ->	62	-0.37	0.15
60 ->	63	-0.01	0.01
60 ->	64	-0.00	0.00
60 ->	65	-0.00	0.00
60 ->	66	0.00	0.00
60 ->	67	-0.00	0.00
60 ->	68	-0.02	0.04
60 ->	69	-0.50	0.72
60 ->	70	-0.93	0.22
60 ->	71	-0.85	0.27
60 ->	72	-0.14	0.11
60 ->	73	-0.06	0.03
60 ->	74	-0.01	0.00
60 ->	75	-0.02	0.01
60 ->	76	-0.04	0.02
60 ->	77	-0.01	0.00
60 ->	78	-0.00	0.00
60 ->	79	-0.00	0.00
60 ->	80	-0.00	0.00
60 ->	81	-0.00	0.00
60 ->	82	-0.00	0.00
60 ->	83	-0.00	0.00

60	->	84	-0.00	0.00
60	->	85	0.00	0.00
60	->	86	-0.00	0.00
60	->	87	-0.01	0.00
60	->	88	-0.01	0.00
60	->	89	-0.00	0.00
60	->	90	-0.00	0.00
60	->	91	-0.00	0.00
60	->	92	-0.00	0.00
60	->	93	-0.00	0.00
60	->	94	-0.00	0.00
60	->	95	-0.00	0.00
60	->	96	-0.00	0.00
60	->	97	-0.00	0.00
60	->	98	-0.00	0.00
60	->	99	-0.00	0.00
60	->	100	-0.01	0.00
60	->	101	-0.02	0.01
60	->	102	-0.01	0.01
60	->	103	-0.00	0.00
60	->	104	-0.00	0.00
60	->	105	-0.00	0.00
60	->	106	-0.00	0.00
60	->	107	-0.00	0.00
61	->	1	0.00	0.00
61	->	2	0.00	0.00
61	->	3	0.00	0.00
61	->	4	0.00	0.00
61	->	5	-0.00	0.00
61	->	6	0.00	0.00
61	->	7	0.00	0.00
61	->	8	-0.00	0.00
61	->	9	0.00	0.00
61	->	10	-0.00	0.00
61	->	11	-0.00	0.00
61	->	12	0.00	0.00
61	->	13	0.00	0.00
61	->	14	0.00	0.00
61	->	15	0.00	0.00
61	->	16	0.00	0.00
61	->	17	0.00	0.00
61	->	18	-0.00	0.00
61	->	19	-0.00	0.00
61	->	20	0.00	0.00
61	->	21	0.00	0.00
61	->	22	0.00	0.00
61	->	23	0.00	0.00
61	->	24	0.00	0.00
61	->	25	0.00	0.00
61	->	26	0.00	0.00

61	->	27	0.00	0.00
61	->	28	0.00	0.00
61	->	29	0.00	0.00
61	->	30	-0.00	0.00
61	->	31	-0.00	0.00
61	->	32	0.00	0.00
61	->	33	-0.00	0.00
61	->	34	0.00	0.00
61	->	35	0.00	0.00
61	->	36	-0.00	0.00
61	->	37	-0.00	0.00
61	->	38	0.00	0.00
61	->	39	0.00	0.00
61	->	40	-0.00	0.00
61	->	41	0.00	0.00
61	->	42	-0.00	0.00
61	->	43	0.00	0.00
61	->	44	-0.00	0.00
61	->	45	-0.00	0.00
61	->	46	-0.01	0.01
61	->	47	-0.00	0.00
61	->	48	-0.00	0.00
61	->	49	-0.02	0.01
61	->	50	-0.05	0.03
61	->	51	-0.00	0.00
61	->	52	-0.01	0.00
61	->	53	-0.46	0.39
61	->	54	-0.00	0.00
61	->	55	-0.00	0.00
61	->	56	-0.00	0.00
61	->	57	-0.01	0.00
61	->	58	-0.07	0.03
61	->	59	-1.04	0.27
61	->	60	-10.82	0.55
61	->	61	27.04	1.21
61	->	62	-10.58	0.42
61	->	63	-0.38	0.06
61	->	64	-0.05	0.01
61	->	65	-0.00	0.00
61	->	66	-0.01	0.00
61	->	67	-0.02	0.01
61	->	68	-0.30	0.08
61	->	69	-2.83	0.65
61	->	70	-3.53	0.59
61	->	71	-0.87	0.23
61	->	72	-1.75	0.70
61	->	73	-1.96	0.77
61	->	74	-0.00	0.02
61	->	75	-0.10	0.05
61	->	76	-1.01	0.78

61 ->	77	-0.04	0.03
61 ->	78	-0.01	0.00
61 ->	79	-0.02	0.01
61 ->	80	-0.01	0.01
61 ->	81	-0.00	0.00
61 ->	82	-0.00	0.00
61 ->	83	-0.01	0.00
61 ->	84	-0.00	0.00
61 ->	85	-0.00	0.00
61 ->	86	-0.00	0.00
61 ->	87	-0.01	0.00
61 ->	88	-0.01	0.00
61 ->	89	-0.00	0.00
61 ->	90	-0.00	0.00
61 ->	91	-0.00	0.00
61 ->	92	-0.00	0.00
61 ->	93	-0.00	0.00
61 ->	94	-0.00	0.00
61 ->	95	-0.00	0.00
61 ->	96	-0.00	0.00
61 ->	97	-0.00	0.00
61 ->	98	-0.00	0.00
61 ->	99	-0.00	0.00
61 ->	100	-0.00	0.00
61 ->	101	-0.06	0.07
61 ->	102	-0.09	0.11
61 ->	103	-0.02	0.01
61 ->	104	-0.01	0.00
61 ->	105	-0.00	0.00
61 ->	106	-0.00	0.00
61 ->	107	-0.00	0.00
62 ->	1	-0.00	0.00
62 ->	2	0.00	0.00
62 ->	3	0.00	0.00
62 ->	4	0.00	0.00
62 ->	5	0.00	0.00
62 ->	6	-0.00	0.00
62 ->	7	0.00	0.00
62 ->	8	0.00	0.00
62 ->	9	0.00	0.00
62 ->	10	0.00	0.00
62 ->	11	0.00	0.00
62 ->	12	-0.00	0.00
62 ->	13	-0.00	0.00
62 ->	14	0.00	0.00
62 ->	15	-0.00	0.00
62 ->	16	-0.00	0.00
62 ->	17	0.00	0.00
62 ->	18	0.00	0.00
62 ->	19	0.00	0.00

62	->	20	0.00	0.00
62	->	21	-0.00	0.00
62	->	22	-0.00	0.00
62	->	23	-0.00	0.00
62	->	24	-0.00	0.00
62	->	25	-0.00	0.00
62	->	26	0.00	0.00
62	->	27	0.00	0.00
62	->	28	0.00	0.00
62	->	29	0.00	0.00
62	->	30	0.00	0.00
62	->	31	-0.00	0.00
62	->	32	0.00	0.00
62	->	33	0.00	0.00
62	->	34	0.00	0.00
62	->	35	-0.00	0.00
62	->	36	-0.00	0.00
62	->	37	-0.00	0.00
62	->	38	-0.00	0.00
62	->	39	0.00	0.00
62	->	40	0.00	0.00
62	->	41	0.00	0.00
62	->	42	-0.00	0.00
62	->	43	0.00	0.00
62	->	44	-0.00	0.00
62	->	45	0.00	0.00
62	->	46	0.00	0.00
62	->	47	0.00	0.00
62	->	48	-0.00	0.00
62	->	49	-0.00	0.00
62	->	50	-0.00	0.00
62	->	51	-0.00	0.00
62	->	52	-0.00	0.00
62	->	53	-0.01	0.00
62	->	54	-0.00	0.00
62	->	55	-0.00	0.00
62	->	56	-0.00	0.00
62	->	57	-0.00	0.00
62	->	58	-0.01	0.00
62	->	59	-0.15	0.04
62	->	60	-0.38	0.16
62	->	61	-10.56	0.43
62	->	62	22.91	1.32
62	->	63	-11.95	0.39
62	->	64	-0.43	0.10
62	->	65	-0.02	0.03
62	->	66	-0.14	0.06
62	->	67	-0.15	0.21
62	->	68	-1.39	0.42
62	->	69	-1.42	0.32

62	->	70	-1.05	0.40
62	->	71	-0.01	0.01
62	->	72	-0.02	0.01
62	->	73	-0.02	0.01
62	->	74	0.00	0.00
62	->	75	-0.01	0.01
62	->	76	-3.09	1.58
62	->	77	-0.01	0.01
62	->	78	0.00	0.01
62	->	79	-0.09	0.02
62	->	80	-0.03	0.02
62	->	81	-0.01	0.00
62	->	82	-0.00	0.00
62	->	83	-0.04	0.01
62	->	84	-0.00	0.00
62	->	85	-0.00	0.00
62	->	86	-0.00	0.00
62	->	87	-0.01	0.01
62	->	88	-0.00	0.00
62	->	89	-0.00	0.00
62	->	90	0.00	0.00
62	->	91	0.00	0.00
62	->	92	0.00	0.00
62	->	93	0.00	0.00
62	->	94	0.00	0.00
62	->	95	0.00	0.00
62	->	96	0.00	0.00
62	->	97	0.00	0.00
62	->	98	0.00	0.00
62	->	99	0.00	0.00
62	->	100	0.00	0.00
62	->	101	0.00	0.00
62	->	102	0.00	0.00
62	->	103	0.00	0.00
62	->	104	0.00	0.00
62	->	105	-0.00	0.00
62	->	106	0.00	0.00
62	->	107	-0.00	0.00
63	->	1	-0.00	0.00
63	->	2	-0.00	0.00
63	->	3	0.00	0.00
63	->	4	-0.00	0.00
63	->	5	0.00	0.00
63	->	6	-0.00	0.00
63	->	7	0.00	0.00
63	->	8	0.00	0.00
63	->	9	0.00	0.00
63	->	10	0.00	0.00
63	->	11	0.00	0.00
63	->	12	-0.00	0.00

63	->	13	-0.00	0.00
63	->	14	-0.00	0.00
63	->	15	-0.00	0.00
63	->	16	-0.00	0.00
63	->	17	0.00	0.00
63	->	18	0.00	0.00
63	->	19	0.00	0.00
63	->	20	0.00	0.00
63	->	21	-0.00	0.00
63	->	22	-0.00	0.00
63	->	23	-0.00	0.00
63	->	24	-0.00	0.00
63	->	25	-0.00	0.00
63	->	26	-0.00	0.00
63	->	27	-0.00	0.00
63	->	28	-0.00	0.00
63	->	29	-0.00	0.00
63	->	30	0.00	0.00
63	->	31	-0.00	0.00
63	->	32	-0.00	0.00
63	->	33	-0.00	0.00
63	->	34	0.00	0.00
63	->	35	0.01	0.00
63	->	36	-0.00	0.00
63	->	37	-0.00	0.00
63	->	38	0.01	0.00
63	->	39	-0.00	0.00
63	->	40	-0.01	0.00
63	->	41	0.00	0.00
63	->	42	-0.00	0.00
63	->	43	-0.00	0.00
63	->	44	-0.01	0.00
63	->	45	0.00	0.00
63	->	46	0.01	0.00
63	->	47	0.00	0.00
63	->	48	0.00	0.00
63	->	49	0.00	0.00
63	->	50	-0.00	0.00
63	->	51	-0.00	0.00
63	->	52	0.00	0.00
63	->	53	-0.03	0.00
63	->	54	0.00	0.00
63	->	55	-0.00	0.00
63	->	56	0.00	0.00
63	->	57	0.00	0.00
63	->	58	0.02	0.00
63	->	59	-0.08	0.01
63	->	60	-0.01	0.01
63	->	61	-0.38	0.06
63	->	62	-11.98	0.39

63	->	63	-26.61	2.07
63	->	64	-14.41	1.27
63	->	65	0.98	1.38
63	->	66	2.99	0.44
63	->	67	-1.08	0.74
63	->	68	-2.49	0.60
63	->	69	-0.57	0.13
63	->	70	-0.80	0.29
63	->	71	0.01	0.01
63	->	72	-0.04	0.01
63	->	73	-0.00	0.01
63	->	74	0.04	0.01
63	->	75	-0.00	0.02
63	->	76	-2.02	0.67
63	->	77	-0.03	0.02
63	->	78	-0.18	0.04
63	->	79	2.98	0.87
63	->	80	0.13	0.19
63	->	81	-0.00	0.02
63	->	82	-0.07	0.02
63	->	83	2.31	0.29
63	->	84	-0.00	0.01
63	->	85	-0.00	0.01
63	->	86	0.02	0.02
63	->	87	0.08	0.07
63	->	88	0.02	0.01
63	->	89	0.01	0.00
63	->	90	0.01	0.00
63	->	91	0.01	0.00
63	->	92	0.00	0.00
63	->	93	0.00	0.00
63	->	94	0.01	0.00
63	->	95	0.01	0.00
63	->	96	0.00	0.00
63	->	97	0.00	0.00
63	->	98	0.00	0.00
63	->	99	0.00	0.00
63	->	100	0.01	0.00
63	->	101	0.01	0.00
63	->	102	0.02	0.00
63	->	103	0.03	0.01
63	->	104	0.02	0.01
63	->	105	0.01	0.00
63	->	106	0.01	0.00
63	->	107	0.00	0.00
64	->	1	-0.00	0.00
64	->	2	-0.00	0.00
64	->	3	0.00	0.00
64	->	4	0.00	0.00
64	->	5	0.00	0.00

64	->	6	-0.00	0.00
64	->	7	0.00	0.00
64	->	8	0.00	0.00
64	->	9	0.00	0.00
64	->	10	0.00	0.00
64	->	11	0.00	0.00
64	->	12	-0.00	0.00
64	->	13	-0.00	0.00
64	->	14	-0.00	0.00
64	->	15	-0.00	0.00
64	->	16	-0.00	0.00
64	->	17	0.00	0.00
64	->	18	0.00	0.00
64	->	19	0.00	0.00
64	->	20	0.00	0.00
64	->	21	-0.00	0.00
64	->	22	-0.00	0.00
64	->	23	-0.00	0.00
64	->	24	-0.00	0.00
64	->	25	-0.00	0.00
64	->	26	-0.00	0.00
64	->	27	-0.00	0.00
64	->	28	-0.00	0.00
64	->	29	-0.00	0.00
64	->	30	0.00	0.00
64	->	31	-0.00	0.00
64	->	32	0.00	0.00
64	->	33	-0.00	0.00
64	->	34	0.00	0.00
64	->	35	0.00	0.00
64	->	36	-0.00	0.00
64	->	37	-0.00	0.00
64	->	38	0.00	0.00
64	->	39	0.00	0.00
64	->	40	-0.00	0.00
64	->	41	0.00	0.00
64	->	42	-0.00	0.00
64	->	43	-0.00	0.00
64	->	44	-0.01	0.00
64	->	45	0.00	0.00
64	->	46	0.01	0.00
64	->	47	0.00	0.00
64	->	48	0.00	0.00
64	->	49	0.00	0.00
64	->	50	0.00	0.00
64	->	51	-0.00	0.00
64	->	52	0.00	0.00
64	->	53	-0.02	0.00
64	->	54	0.00	0.00
64	->	55	-0.00	0.00

64	->	56	0.00	0.00
64	->	57	0.00	0.00
64	->	58	0.01	0.00
64	->	59	-0.03	0.00
64	->	60	-0.00	0.00
64	->	61	-0.05	0.01
64	->	62	-0.43	0.10
64	->	63	-14.54	1.29
64	->	64	-32.96	3.66
64	->	65	-12.26	0.51
64	->	66	-0.27	0.23
64	->	67	-0.25	0.30
64	->	68	-0.05	0.03
64	->	69	-0.08	0.02
64	->	70	-0.01	0.02
64	->	71	0.00	0.00
64	->	72	-0.02	0.00
64	->	73	-0.00	0.00
64	->	74	0.02	0.00
64	->	75	0.02	0.01
64	->	76	-2.74	2.21
64	->	77	-0.04	0.01
64	->	78	-0.03	0.01
64	->	79	-0.07	0.07
64	->	80	-0.18	0.16
64	->	81	-0.03	0.01
64	->	82	-0.01	0.00
64	->	83	-0.20	0.14
64	->	84	-0.02	0.02
64	->	85	0.00	0.01
64	->	86	0.01	0.00
64	->	87	0.03	0.01
64	->	88	0.01	0.00
64	->	89	0.01	0.00
64	->	90	0.00	0.00
64	->	91	0.00	0.00
64	->	92	0.00	0.00
64	->	93	0.00	0.00
64	->	94	0.01	0.00
64	->	95	0.01	0.00
64	->	96	0.00	0.00
64	->	97	0.00	0.00
64	->	98	0.00	0.00
64	->	99	0.00	0.00
64	->	100	0.01	0.00
64	->	101	0.01	0.00
64	->	102	0.02	0.01
64	->	103	0.02	0.01
64	->	104	0.02	0.00
64	->	105	0.01	0.00

64	->	106	0.01	0.00
64	->	107	0.00	0.00
65	->	1	-0.00	0.00
65	->	2	0.00	0.00
65	->	3	0.00	0.00
65	->	4	0.00	0.00
65	->	5	0.00	0.00
65	->	6	0.00	0.00
65	->	7	0.00	0.00
65	->	8	0.00	0.00
65	->	9	0.00	0.00
65	->	10	0.00	0.00
65	->	11	0.00	0.00
65	->	12	-0.00	0.00
65	->	13	-0.00	0.00
65	->	14	0.00	0.00
65	->	15	-0.00	0.00
65	->	16	-0.00	0.00
65	->	17	0.00	0.00
65	->	18	0.00	0.00
65	->	19	0.00	0.00
65	->	20	0.00	0.00
65	->	21	0.00	0.00
65	->	22	-0.00	0.00
65	->	23	0.00	0.00
65	->	24	0.00	0.00
65	->	25	-0.00	0.00
65	->	26	0.00	0.00
65	->	27	0.00	0.00
65	->	28	0.00	0.00
65	->	29	0.00	0.00
65	->	30	0.00	0.00
65	->	31	-0.00	0.00
65	->	32	0.00	0.00
65	->	33	0.00	0.00
65	->	34	0.00	0.00
65	->	35	0.00	0.00
65	->	36	-0.00	0.00
65	->	37	0.00	0.00
65	->	38	0.00	0.00
65	->	39	0.00	0.00
65	->	40	-0.00	0.00
65	->	41	0.00	0.00
65	->	42	0.00	0.00
65	->	43	0.00	0.00
65	->	44	-0.00	0.00
65	->	45	0.00	0.00
65	->	46	0.00	0.00
65	->	47	-0.00	0.00
65	->	48	0.00	0.00

65	->	49	0.00	0.00
65	->	50	0.00	0.00
65	->	51	-0.00	0.00
65	->	52	0.00	0.00
65	->	53	-0.00	0.00
65	->	54	0.00	0.00
65	->	55	-0.00	0.00
65	->	56	0.00	0.00
65	->	57	0.00	0.00
65	->	58	0.00	0.00
65	->	59	-0.00	0.00
65	->	60	-0.00	0.00
65	->	61	-0.00	0.00
65	->	62	-0.02	0.03
65	->	63	0.91	1.38
65	->	64	-12.46	0.53
65	->	65	21.84	1.43
65	->	66	-8.55	1.06
65	->	67	-0.38	0.18
65	->	68	-0.22	0.06
65	->	69	-0.01	0.00
65	->	70	-0.03	0.01
65	->	71	-0.00	0.00
65	->	72	-0.00	0.00
65	->	73	-0.00	0.00
65	->	74	0.00	0.00
65	->	75	-0.00	0.00
65	->	76	-0.05	0.03
65	->	77	-0.01	0.00
65	->	78	-0.02	0.00
65	->	79	0.72	0.10
65	->	80	-0.09	0.09
65	->	81	-0.01	0.00
65	->	82	-0.04	0.01
65	->	83	0.81	0.58
65	->	84	-0.08	0.20
65	->	85	-0.03	0.07
65	->	86	-0.00	0.00
65	->	87	0.00	0.01
65	->	88	0.00	0.00
65	->	89	0.00	0.00
65	->	90	0.00	0.00
65	->	91	0.00	0.00
65	->	92	0.00	0.00
65	->	93	0.00	0.00
65	->	94	0.00	0.00
65	->	95	0.00	0.00
65	->	96	0.00	0.00
65	->	97	0.00	0.00
65	->	98	0.00	0.00

65	->	99	0.00	0.00
65	->	100	0.00	0.00
65	->	101	0.00	0.00
65	->	102	0.00	0.00
65	->	103	0.00	0.00
65	->	104	0.00	0.00
65	->	105	0.00	0.00
65	->	106	0.00	0.00
65	->	107	0.00	0.00
66	->	1	-0.00	0.00
66	->	2	-0.00	0.00
66	->	3	0.00	0.00
66	->	4	-0.00	0.00
66	->	5	0.00	0.00
66	->	6	0.00	0.00
66	->	7	0.00	0.00
66	->	8	0.00	0.00
66	->	9	0.00	0.00
66	->	10	0.00	0.00
66	->	11	0.00	0.00
66	->	12	-0.00	0.00
66	->	13	-0.00	0.00
66	->	14	-0.00	0.00
66	->	15	-0.00	0.00
66	->	16	-0.00	0.00
66	->	17	0.00	0.00
66	->	18	0.00	0.00
66	->	19	0.00	0.00
66	->	20	0.00	0.00
66	->	21	-0.00	0.00
66	->	22	-0.00	0.00
66	->	23	-0.00	0.00
66	->	24	-0.00	0.00
66	->	25	-0.00	0.00
66	->	26	-0.00	0.00
66	->	27	-0.00	0.00
66	->	28	-0.00	0.00
66	->	29	-0.00	0.00
66	->	30	0.00	0.00
66	->	31	-0.00	0.00
66	->	32	-0.00	0.00
66	->	33	0.00	0.00
66	->	34	0.00	0.00
66	->	35	0.01	0.00
66	->	36	-0.00	0.00
66	->	37	-0.00	0.00
66	->	38	0.01	0.00
66	->	39	-0.00	0.00
66	->	40	-0.01	0.00
66	->	41	0.00	0.00

66	->	42	-0.00	0.00
66	->	43	-0.00	0.00
66	->	44	-0.01	0.00
66	->	45	0.00	0.00
66	->	46	0.01	0.00
66	->	47	0.00	0.00
66	->	48	0.00	0.00
66	->	49	0.00	0.00
66	->	50	0.00	0.00
66	->	51	-0.00	0.00
66	->	52	0.00	0.00
66	->	53	-0.02	0.00
66	->	54	0.00	0.00
66	->	55	-0.00	0.00
66	->	56	0.00	0.00
66	->	57	0.00	0.00
66	->	58	0.02	0.01
66	->	59	-0.06	0.01
66	->	60	0.00	0.00
66	->	61	-0.01	0.00
66	->	62	-0.14	0.06
66	->	63	2.99	0.44
66	->	64	-0.27	0.24
66	->	65	-8.47	1.06
66	->	66	-32.02	2.97
66	->	67	-8.92	0.42
66	->	68	-3.43	0.68
66	->	69	-0.03	0.03
66	->	70	0.04	0.03
66	->	71	-0.00	0.00
66	->	72	-0.02	0.00
66	->	73	0.00	0.00
66	->	74	0.03	0.00
66	->	75	0.04	0.01
66	->	76	-0.11	0.02
66	->	77	-0.03	0.00
66	->	78	-0.08	0.01
66	->	79	3.57	0.68
66	->	80	0.04	0.02
66	->	81	-0.03	0.01
66	->	82	-0.06	0.02
66	->	83	2.09	0.29
66	->	84	-0.00	0.00
66	->	85	-0.01	0.03
66	->	86	0.03	0.05
66	->	87	0.14	0.18
66	->	88	0.02	0.01
66	->	89	0.02	0.00
66	->	90	0.01	0.00
66	->	91	0.01	0.00

66	->	92	0.00	0.00
66	->	93	0.00	0.00
66	->	94	0.00	0.00
66	->	95	0.00	0.00
66	->	96	0.00	0.00
66	->	97	0.00	0.00
66	->	98	0.00	0.00
66	->	99	0.00	0.00
66	->	100	0.01	0.00
66	->	101	0.01	0.00
66	->	102	0.01	0.00
66	->	103	0.02	0.00
66	->	104	0.02	0.00
66	->	105	0.01	0.00
66	->	106	0.01	0.00
66	->	107	0.01	0.00
67	->	1	0.00	0.00
67	->	2	0.00	0.00
67	->	3	0.00	0.00
67	->	4	0.00	0.00
67	->	5	0.00	0.00
67	->	6	0.00	0.00
67	->	7	0.00	0.00
67	->	8	0.00	0.00
67	->	9	0.00	0.00
67	->	10	0.00	0.00
67	->	11	0.00	0.00
67	->	12	0.00	0.00
67	->	13	0.00	0.00
67	->	14	0.00	0.00
67	->	15	0.00	0.00
67	->	16	-0.00	0.00
67	->	17	0.00	0.00
67	->	18	0.00	0.00
67	->	19	0.00	0.00
67	->	20	0.00	0.00
67	->	21	0.00	0.00
67	->	22	-0.00	0.00
67	->	23	0.00	0.00
67	->	24	0.00	0.00
67	->	25	-0.00	0.00
67	->	26	0.00	0.00
67	->	27	0.00	0.00
67	->	28	0.00	0.00
67	->	29	0.00	0.00
67	->	30	0.00	0.00
67	->	31	-0.00	0.00
67	->	32	0.00	0.00
67	->	33	0.00	0.00
67	->	34	0.00	0.00

67	->	35	0.00	0.00
67	->	36	-0.00	0.00
67	->	37	-0.00	0.00
67	->	38	0.00	0.00
67	->	39	0.00	0.00
67	->	40	-0.00	0.00
67	->	41	0.00	0.00
67	->	42	-0.00	0.00
67	->	43	0.00	0.00
67	->	44	-0.00	0.00
67	->	45	0.00	0.00
67	->	46	0.00	0.00
67	->	47	0.00	0.00
67	->	48	0.00	0.00
67	->	49	0.00	0.00
67	->	50	0.00	0.00
67	->	51	0.00	0.00
67	->	52	0.00	0.00
67	->	53	-0.00	0.00
67	->	54	-0.00	0.00
67	->	55	-0.00	0.00
67	->	56	-0.00	0.00
67	->	57	-0.00	0.00
67	->	58	0.00	0.00
67	->	59	-0.01	0.01
67	->	60	-0.00	0.00
67	->	61	-0.02	0.01
67	->	62	-0.15	0.20
67	->	63	-1.09	0.74
67	->	64	-0.26	0.31
67	->	65	-0.38	0.18
67	->	66	-9.00	0.42
67	->	67	11.59	0.76
67	->	68	-8.71	0.33
67	->	69	-0.79	0.46
67	->	70	-0.09	0.03
67	->	71	-0.00	0.00
67	->	72	-0.00	0.00
67	->	73	-0.00	0.00
67	->	74	-0.00	0.00
67	->	75	0.00	0.00
67	->	76	-0.01	0.00
67	->	77	-0.00	0.00
67	->	78	0.00	0.00
67	->	79	-0.08	0.05
67	->	80	-0.01	0.00
67	->	81	-0.00	0.00
67	->	82	-0.00	0.00
67	->	83	-0.02	0.03
67	->	84	-0.00	0.00

67	->	85	-0.00	0.00
67	->	86	-0.01	0.01
67	->	87	-0.01	0.01
67	->	88	-0.00	0.00
67	->	89	0.00	0.00
67	->	90	0.00	0.00
67	->	91	0.00	0.00
67	->	92	0.00	0.00
67	->	93	-0.00	0.00
67	->	94	-0.00	0.00
67	->	95	-0.00	0.00
67	->	96	0.00	0.00
67	->	97	0.00	0.00
67	->	98	0.00	0.00
67	->	99	0.00	0.00
67	->	100	0.00	0.00
67	->	101	0.00	0.00
67	->	102	0.00	0.00
67	->	103	-0.00	0.00
67	->	104	-0.00	0.00
67	->	105	-0.00	0.00
67	->	106	-0.00	0.00
67	->	107	0.00	0.00
68	->	1	0.00	0.00
68	->	2	0.00	0.00
68	->	3	-0.00	0.00
68	->	4	0.00	0.00
68	->	5	-0.00	0.00
68	->	6	0.00	0.00
68	->	7	0.00	0.00
68	->	8	-0.00	0.00
68	->	9	-0.00	0.00
68	->	10	-0.00	0.00
68	->	11	-0.00	0.00
68	->	12	0.00	0.00
68	->	13	0.00	0.00
68	->	14	0.00	0.00
68	->	15	0.00	0.00
68	->	16	0.00	0.00
68	->	17	-0.00	0.00
68	->	18	-0.00	0.00
68	->	19	-0.00	0.00
68	->	20	-0.00	0.00
68	->	21	0.00	0.00
68	->	22	0.01	0.00
68	->	23	0.00	0.00
68	->	24	-0.00	0.00
68	->	25	0.01	0.00
68	->	26	0.00	0.00
68	->	27	0.00	0.00

68	->	28	0.00	0.00
68	->	29	0.00	0.00
68	->	30	-0.00	0.00
68	->	31	0.01	0.00
68	->	32	0.00	0.00
68	->	33	-0.00	0.00
68	->	34	-0.00	0.00
68	->	35	-0.01	0.00
68	->	36	0.01	0.00
68	->	37	0.00	0.00
68	->	38	-0.02	0.01
68	->	39	0.00	0.00
68	->	40	0.02	0.01
68	->	41	-0.00	0.00
68	->	42	0.00	0.00
68	->	43	0.00	0.00
68	->	44	0.01	0.00
68	->	45	-0.00	0.00
68	->	46	-0.02	0.01
68	->	47	-0.00	0.00
68	->	48	-0.00	0.00
68	->	49	-0.00	0.00
68	->	50	-0.00	0.00
68	->	51	0.00	0.00
68	->	52	-0.00	0.00
68	->	53	0.03	0.01
68	->	54	-0.02	0.03
68	->	55	-0.00	0.00
68	->	56	-0.00	0.00
68	->	57	-0.00	0.00
68	->	58	-0.02	0.01
68	->	59	0.26	0.20
68	->	60	-0.02	0.04
68	->	61	-0.30	0.08
68	->	62	-1.38	0.42
68	->	63	-2.50	0.60
68	->	64	-0.05	0.03
68	->	65	-0.23	0.06
68	->	66	-3.53	0.68
68	->	67	-8.85	0.33
68	->	68	-103.64	4.83
68	->	69	-13.57	1.43
68	->	70	-3.77	0.80
68	->	71	-0.08	0.04
68	->	72	0.05	0.04
68	->	73	-0.02	0.01
68	->	74	-0.06	0.02
68	->	75	-1.86	3.74
68	->	76	-0.12	0.06
68	->	77	0.01	0.01

68	->	78	0.14	0.08
68	->	79	-1.85	0.61
68	->	80	-0.04	0.02
68	->	81	0.02	0.00
68	->	82	-0.04	0.05
68	->	83	-0.23	0.07
68	->	84	-0.00	0.00
68	->	85	0.00	0.01
68	->	86	-1.88	1.19
68	->	87	-6.06	4.39
68	->	88	-0.35	0.55
68	->	89	-0.05	0.02
68	->	90	-0.02	0.01
68	->	91	-0.01	0.00
68	->	92	-0.01	0.00
68	->	93	-0.01	0.00
68	->	94	-0.01	0.00
68	->	95	-0.00	0.00
68	->	96	-0.00	0.00
68	->	97	-0.00	0.00
68	->	98	-0.00	0.00
68	->	99	-0.00	0.00
68	->	100	-0.01	0.00
68	->	101	-0.01	0.00
68	->	102	-0.02	0.01
68	->	103	-0.02	0.01
68	->	104	-0.02	0.01
68	->	105	-0.02	0.00
68	->	106	-0.02	0.01
68	->	107	-0.02	0.00
69	->	1	0.00	0.00
69	->	2	0.00	0.00
69	->	3	0.00	0.00
69	->	4	0.00	0.00
69	->	5	-0.00	0.00
69	->	6	0.00	0.00
69	->	7	0.00	0.00
69	->	8	-0.00	0.00
69	->	9	-0.00	0.00
69	->	10	-0.00	0.00
69	->	11	-0.00	0.00
69	->	12	0.00	0.00
69	->	13	0.00	0.00
69	->	14	0.00	0.00
69	->	15	0.00	0.00
69	->	16	0.00	0.00
69	->	17	-0.00	0.00
69	->	18	-0.00	0.00
69	->	19	-0.00	0.00
69	->	20	-0.00	0.00

69	->	21	0.00	0.00
69	->	22	0.00	0.00
69	->	23	0.00	0.00
69	->	24	0.00	0.00
69	->	25	0.00	0.00
69	->	26	0.00	0.00
69	->	27	0.00	0.00
69	->	28	0.00	0.00
69	->	29	-0.00	0.00
69	->	30	-0.00	0.00
69	->	31	0.01	0.00
69	->	32	-0.00	0.00
69	->	33	0.00	0.00
69	->	34	-0.00	0.00
69	->	35	-0.01	0.00
69	->	36	0.01	0.00
69	->	37	0.00	0.00
69	->	38	-0.01	0.00
69	->	39	0.00	0.00
69	->	40	0.01	0.00
69	->	41	-0.00	0.00
69	->	42	-0.00	0.00
69	->	43	0.00	0.00
69	->	44	0.01	0.00
69	->	45	-0.00	0.00
69	->	46	-0.02	0.01
69	->	47	-0.00	0.00
69	->	48	-0.00	0.00
69	->	49	-0.00	0.00
69	->	50	-0.01	0.01
69	->	51	-0.00	0.00
69	->	52	-0.00	0.00
69	->	53	0.09	0.03
69	->	54	-0.05	0.03
69	->	55	-0.01	0.01
69	->	56	-0.01	0.01
69	->	57	-0.29	0.64
69	->	58	-0.40	1.19
69	->	59	-2.21	0.80
69	->	60	-0.50	0.72
69	->	61	-2.85	0.65
69	->	62	-1.45	0.33
69	->	63	-0.57	0.13
69	->	64	-0.08	0.02
69	->	65	-0.01	0.00
69	->	66	-0.03	0.03
69	->	67	-0.85	0.47
69	->	68	-13.55	1.44
69	->	69	10.30	2.73
69	->	70	-10.52	0.39

69	->	71	-0.45	0.06
69	->	72	-0.00	0.03
69	->	73	-0.04	0.02
69	->	74	-0.05	0.01
69	->	75	-0.16	0.18
69	->	76	0.09	0.05
69	->	77	-0.00	0.01
69	->	78	0.04	0.01
69	->	79	-0.02	0.01
69	->	80	-0.01	0.01
69	->	81	0.01	0.00
69	->	82	-0.00	0.00
69	->	83	-0.01	0.00
69	->	84	-0.00	0.00
69	->	85	-0.00	0.00
69	->	86	-0.05	0.05
69	->	87	-0.16	0.13
69	->	88	-0.07	0.04
69	->	89	-0.02	0.01
69	->	90	-0.01	0.00
69	->	91	-0.01	0.00
69	->	92	-0.00	0.00
69	->	93	-0.00	0.00
69	->	94	-0.00	0.00
69	->	95	-0.00	0.00
69	->	96	-0.00	0.00
69	->	97	-0.00	0.00
69	->	98	-0.00	0.00
69	->	99	-0.01	0.00
69	->	100	-0.01	0.00
69	->	101	-0.02	0.01
69	->	102	-0.02	0.01
69	->	103	-0.02	0.00
69	->	104	-0.01	0.00
69	->	105	-0.01	0.00
69	->	106	-0.01	0.00
69	->	107	-0.00	0.00
70	->	1	0.00	0.00
70	->	2	0.00	0.00
70	->	3	0.00	0.00
70	->	4	0.00	0.00
70	->	5	-0.00	0.00
70	->	6	0.00	0.00
70	->	7	0.00	0.00
70	->	8	-0.00	0.00
70	->	9	0.00	0.00
70	->	10	-0.00	0.00
70	->	11	-0.00	0.00
70	->	12	0.00	0.00
70	->	13	0.00	0.00

70	->	14	0.00	0.00
70	->	15	0.00	0.00
70	->	16	0.00	0.00
70	->	17	0.00	0.00
70	->	18	0.00	0.00
70	->	19	-0.00	0.00
70	->	20	0.00	0.00
70	->	21	0.00	0.00
70	->	22	0.00	0.00
70	->	23	0.00	0.00
70	->	24	0.00	0.00
70	->	25	0.00	0.00
70	->	26	0.00	0.00
70	->	27	0.00	0.00
70	->	28	0.00	0.00
70	->	29	0.00	0.00
70	->	30	-0.00	0.00
70	->	31	0.00	0.00
70	->	32	0.00	0.00
70	->	33	0.00	0.00
70	->	34	0.00	0.00
70	->	35	-0.00	0.00
70	->	36	-0.00	0.00
70	->	37	-0.00	0.00
70	->	38	-0.00	0.00
70	->	39	0.00	0.00
70	->	40	0.00	0.00
70	->	41	0.00	0.00
70	->	42	-0.00	0.00
70	->	43	0.00	0.00
70	->	44	0.00	0.00
70	->	45	0.00	0.00
70	->	46	-0.00	0.00
70	->	47	-0.00	0.00
70	->	48	-0.00	0.00
70	->	49	0.00	0.00
70	->	50	-0.04	0.02
70	->	51	-0.01	0.01
70	->	52	-0.00	0.00
70	->	53	0.01	0.06
70	->	54	-0.10	0.06
70	->	55	-0.01	0.00
70	->	56	-0.00	0.00
70	->	57	-0.00	0.01
70	->	58	-0.16	0.13
70	->	59	-2.74	0.81
70	->	60	-0.93	0.22
70	->	61	-3.54	0.60
70	->	62	-1.04	0.40
70	->	63	-0.79	0.29

70	->	64	-0.01	0.02
70	->	65	-0.03	0.01
70	->	66	0.04	0.03
70	->	67	-0.09	0.03
70	->	68	-3.72	0.78
70	->	69	-10.58	0.39
70	->	70	29.71	1.22
70	->	71	-9.88	0.36
70	->	72	-1.10	0.23
70	->	73	-0.25	0.06
70	->	74	-0.15	0.03
70	->	75	-3.49	1.47
70	->	76	-2.87	0.51
70	->	77	-0.05	0.02
70	->	78	-0.23	0.07
70	->	79	-1.81	0.29
70	->	80	-0.05	0.03
70	->	81	-0.00	0.00
70	->	82	-0.03	0.01
70	->	83	-0.00	0.01
70	->	84	-0.00	0.00
70	->	85	-0.00	0.00
70	->	86	-0.11	0.22
70	->	87	-0.24	0.22
70	->	88	-0.12	0.07
70	->	89	-0.02	0.01
70	->	90	-0.01	0.00
70	->	91	-0.00	0.00
70	->	92	-0.00	0.00
70	->	93	-0.00	0.00
70	->	94	-0.00	0.00
70	->	95	-0.00	0.00
70	->	96	0.00	0.00
70	->	97	-0.00	0.00
70	->	98	-0.00	0.00
70	->	99	-0.00	0.00
70	->	100	-0.00	0.00
70	->	101	-0.00	0.00
70	->	102	-0.01	0.01
70	->	103	-0.01	0.01
70	->	104	-0.01	0.00
70	->	105	-0.01	0.00
70	->	106	-0.00	0.00
70	->	107	-0.00	0.00
71	->	1	-0.00	0.00
71	->	2	-0.00	0.00
71	->	3	0.00	0.00
71	->	4	0.00	0.00
71	->	5	0.00	0.00
71	->	6	0.00	0.00

71	->	7	0.00	0.00
71	->	8	0.00	0.00
71	->	9	0.00	0.00
71	->	10	-0.00	0.00
71	->	11	-0.00	0.00
71	->	12	0.00	0.00
71	->	13	-0.00	0.00
71	->	14	0.00	0.00
71	->	15	0.00	0.00
71	->	16	-0.00	0.00
71	->	17	0.00	0.00
71	->	18	0.00	0.00
71	->	19	0.00	0.00
71	->	20	0.00	0.00
71	->	21	-0.00	0.00
71	->	22	-0.00	0.00
71	->	23	-0.00	0.00
71	->	24	0.00	0.00
71	->	25	-0.00	0.00
71	->	26	-0.00	0.00
71	->	27	0.00	0.00
71	->	28	0.00	0.00
71	->	29	0.00	0.00
71	->	30	0.00	0.00
71	->	31	0.00	0.00
71	->	32	0.00	0.00
71	->	33	-0.00	0.00
71	->	34	0.00	0.00
71	->	35	-0.00	0.00
71	->	36	-0.00	0.00
71	->	37	-0.00	0.00
71	->	38	-0.00	0.00
71	->	39	-0.00	0.00
71	->	40	-0.00	0.00
71	->	41	-0.00	0.00
71	->	42	-0.00	0.00
71	->	43	-0.00	0.00
71	->	44	0.00	0.00
71	->	45	-0.00	0.00
71	->	46	-0.02	0.01
71	->	47	-0.01	0.01
71	->	48	-0.01	0.01
71	->	49	-0.06	0.04
71	->	50	-1.05	0.53
71	->	51	-0.06	0.04
71	->	52	-0.02	0.01
71	->	53	-2.23	2.21
71	->	54	-0.61	0.35
71	->	55	-0.01	0.01
71	->	56	-0.01	0.01

71	->	57	-0.03	0.01
71	->	58	-0.37	0.36
71	->	59	-1.86	0.37
71	->	60	-0.83	0.25
71	->	61	-0.87	0.23
71	->	62	-0.01	0.01
71	->	63	0.01	0.01
71	->	64	0.00	0.00
71	->	65	-0.00	0.00
71	->	66	-0.00	0.00
71	->	67	-0.00	0.00
71	->	68	-0.08	0.04
71	->	69	-0.45	0.06
71	->	70	-9.88	0.36
71	->	71	23.15	1.08
71	->	72	-7.99	0.49
71	->	73	-0.34	0.05
71	->	74	-0.20	0.04
71	->	75	-2.10	1.93
71	->	76	-0.03	0.02
71	->	77	-0.01	0.00
71	->	78	-0.00	0.01
71	->	79	-0.00	0.00
71	->	80	-0.00	0.00
71	->	81	0.00	0.00
71	->	82	-0.00	0.00
71	->	83	-0.00	0.00
71	->	84	-0.00	0.00
71	->	85	0.00	0.00
71	->	86	-0.00	0.00
71	->	87	-0.08	0.11
71	->	88	-0.15	0.13
71	->	89	-0.03	0.02
71	->	90	-0.01	0.01
71	->	91	-0.00	0.00
71	->	92	0.00	0.00
71	->	93	0.00	0.00
71	->	94	0.00	0.00
71	->	95	0.00	0.00
71	->	96	0.00	0.00
71	->	97	0.00	0.00
71	->	98	0.00	0.00
71	->	99	0.00	0.00
71	->	100	-0.00	0.00
71	->	101	-0.01	0.01
71	->	102	-0.02	0.02
71	->	103	-0.01	0.01
71	->	104	0.00	0.00
71	->	105	-0.00	0.00
71	->	106	-0.00	0.00

71	->	107	-0.00	0.00
72	->	1	0.02	0.00
72	->	2	0.00	0.00
72	->	3	-0.00	0.00
72	->	4	-0.00	0.00
72	->	5	-0.01	0.00
72	->	6	0.00	0.00
72	->	7	-0.00	0.00
72	->	8	-0.00	0.00
72	->	9	-0.00	0.00
72	->	10	-0.01	0.00
72	->	11	-0.01	0.00
72	->	12	0.01	0.01
72	->	13	0.00	0.00
72	->	14	0.00	0.00
72	->	15	0.00	0.00
72	->	16	0.02	0.00
72	->	17	-0.00	0.00
72	->	18	-0.00	0.00
72	->	19	-0.02	0.00
72	->	20	-0.00	0.00
72	->	21	0.00	0.00
72	->	22	0.03	0.00
72	->	23	0.00	0.00
72	->	24	-0.00	0.00
72	->	25	0.02	0.00
72	->	26	-0.00	0.00
72	->	27	0.00	0.00
72	->	28	0.00	0.00
72	->	29	0.00	0.00
72	->	30	-0.00	0.00
72	->	31	0.03	0.00
72	->	32	-0.00	0.00
72	->	33	-0.00	0.00
72	->	34	-0.00	0.00
72	->	35	-0.03	0.00
72	->	36	0.01	0.00
72	->	37	0.00	0.00
72	->	38	-0.03	0.00
72	->	39	-0.00	0.00
72	->	40	0.04	0.02
72	->	41	-0.00	0.00
72	->	42	-0.01	0.01
72	->	43	0.00	0.00
72	->	44	0.11	0.04
72	->	45	-0.01	0.00
72	->	46	-0.19	0.10
72	->	47	-0.12	0.06
72	->	48	-0.02	0.01
72	->	49	0.02	0.02

72	->	50	-1.48	0.38
72	->	51	-0.07	0.04
72	->	52	-0.02	0.01
72	->	53	-0.20	0.26
72	->	54	-0.08	0.09
72	->	55	-0.01	0.00
72	->	56	-0.00	0.00
72	->	57	-0.01	0.00
72	->	58	-0.03	0.01
72	->	59	-0.04	0.04
72	->	60	-0.14	0.11
72	->	61	-1.76	0.70
72	->	62	-0.02	0.01
72	->	63	-0.04	0.01
72	->	64	-0.02	0.00
72	->	65	-0.00	0.00
72	->	66	-0.02	0.00
72	->	67	-0.00	0.00
72	->	68	0.05	0.04
72	->	69	-0.00	0.03
72	->	70	-1.09	0.23
72	->	71	-8.09	0.49
72	->	72	-93.99	2.19
72	->	73	-21.58	0.49
72	->	74	-14.78	1.48
72	->	75	-4.72	1.30
72	->	76	-0.74	0.46
72	->	77	0.12	0.08
72	->	78	0.32	0.18
72	->	79	-0.00	0.01
72	->	80	-0.01	0.01
72	->	81	0.04	0.01
72	->	82	-0.00	0.00
72	->	83	-0.00	0.00
72	->	84	-0.00	0.00
72	->	85	0.00	0.01
72	->	86	-0.03	0.01
72	->	87	-0.37	0.42
72	->	88	-2.19	1.31
72	->	89	-8.40	1.27
72	->	90	-2.07	0.96
72	->	91	-0.14	0.16
72	->	92	-0.02	0.03
72	->	93	-0.02	0.01
72	->	94	-0.02	0.00
72	->	95	-0.02	0.00
72	->	96	-0.01	0.00
72	->	97	-0.00	0.00
72	->	98	-0.01	0.00
72	->	99	-0.02	0.00

72	->	100	-0.05	0.01
72	->	101	-0.09	0.05
72	->	102	-0.30	0.25
72	->	103	-0.59	0.64
72	->	104	-0.12	0.96
72	->	105	0.43	0.30
72	->	106	0.08	0.15
72	->	107	-0.05	0.01
73	->	1	0.00	0.00
73	->	2	0.00	0.00
73	->	3	0.00	0.00
73	->	4	0.00	0.00
73	->	5	-0.00	0.00
73	->	6	0.00	0.00
73	->	7	-0.00	0.00
73	->	8	-0.00	0.00
73	->	9	0.00	0.00
73	->	10	-0.00	0.00
73	->	11	-0.00	0.00
73	->	12	0.00	0.00
73	->	13	0.00	0.00
73	->	14	0.00	0.00
73	->	15	0.00	0.00
73	->	16	0.00	0.00
73	->	17	0.00	0.00
73	->	18	0.00	0.00
73	->	19	-0.00	0.00
73	->	20	0.00	0.00
73	->	21	0.00	0.00
73	->	22	0.00	0.00
73	->	23	0.00	0.00
73	->	24	0.00	0.00
73	->	25	0.00	0.00
73	->	26	0.00	0.00
73	->	27	0.00	0.00
73	->	28	0.00	0.00
73	->	29	0.00	0.00
73	->	30	-0.00	0.00
73	->	31	0.00	0.00
73	->	32	0.00	0.00
73	->	33	-0.00	0.00
73	->	34	-0.00	0.00
73	->	35	-0.00	0.00
73	->	36	0.00	0.00
73	->	37	0.00	0.00
73	->	38	-0.00	0.00
73	->	39	-0.00	0.00
73	->	40	-0.00	0.00
73	->	41	-0.00	0.00
73	->	42	-0.00	0.00

73	->	43	-0.00	0.00
73	->	44	-0.00	0.01
73	->	45	-0.00	0.00
73	->	46	-0.01	0.01
73	->	47	-0.00	0.00
73	->	48	-0.00	0.00
73	->	49	-0.02	0.01
73	->	50	-0.03	0.04
73	->	51	0.00	0.00
73	->	52	-0.00	0.00
73	->	53	-0.13	0.16
73	->	54	0.00	0.00
73	->	55	0.00	0.00
73	->	56	-0.00	0.00
73	->	57	-0.00	0.00
73	->	58	-0.01	0.00
73	->	59	-0.06	0.02
73	->	60	-0.06	0.03
73	->	61	-1.99	0.80
73	->	62	-0.02	0.01
73	->	63	-0.00	0.01
73	->	64	-0.00	0.00
73	->	65	-0.00	0.00
73	->	66	0.00	0.00
73	->	67	-0.00	0.00
73	->	68	-0.02	0.01
73	->	69	-0.04	0.02
73	->	70	-0.25	0.06
73	->	71	-0.34	0.05
73	->	72	-21.57	0.50
73	->	73	20.42	1.80
73	->	74	-10.57	0.66
73	->	75	-0.76	0.25
73	->	76	-2.31	1.02
73	->	77	-1.89	1.41
73	->	78	-0.10	0.08
73	->	79	-0.04	0.03
73	->	80	-0.02	0.01
73	->	81	-0.01	0.01
73	->	82	-0.00	0.00
73	->	83	-0.00	0.00
73	->	84	-0.00	0.00
73	->	85	-0.00	0.00
73	->	86	-0.00	0.00
73	->	87	0.00	0.01
73	->	88	-0.01	0.00
73	->	89	-0.01	0.00
73	->	90	-0.01	0.00
73	->	91	-0.00	0.01
73	->	92	-0.01	0.01

73	->	93	-0.01	0.01
73	->	94	-0.00	0.00
73	->	95	-0.00	0.00
73	->	96	-0.00	0.00
73	->	97	-0.00	0.00
73	->	98	-0.00	0.00
73	->	99	-0.00	0.00
73	->	100	-0.01	0.00
73	->	101	-0.18	0.31
73	->	102	-1.44	1.90
73	->	103	-0.62	1.15
73	->	104	-0.07	0.05
73	->	105	-0.01	0.00
73	->	106	-0.00	0.00
73	->	107	-0.00	0.00
74	->	1	-0.02	0.01
74	->	2	-0.00	0.00
74	->	3	0.00	0.00
74	->	4	0.00	0.00
74	->	5	0.01	0.00
74	->	6	-0.00	0.00
74	->	7	0.00	0.00
74	->	8	0.00	0.00
74	->	9	0.00	0.00
74	->	10	0.01	0.00
74	->	11	0.01	0.00
74	->	12	-0.01	0.01
74	->	13	-0.00	0.00
74	->	14	-0.00	0.00
74	->	15	-0.00	0.00
74	->	16	-0.02	0.00
74	->	17	0.00	0.00
74	->	18	0.00	0.00
74	->	19	0.02	0.00
74	->	20	0.00	0.00
74	->	21	-0.00	0.00
74	->	22	-0.03	0.00
74	->	23	-0.00	0.00
74	->	24	-0.00	0.00
74	->	25	-0.02	0.00
74	->	26	-0.00	0.00
74	->	27	-0.00	0.00
74	->	28	-0.00	0.00
74	->	29	-0.00	0.00
74	->	30	0.00	0.00
74	->	31	-0.02	0.00
74	->	32	-0.00	0.00
74	->	33	-0.00	0.00
74	->	34	0.00	0.00
74	->	35	0.02	0.00

74 ->	36	-0.01	0.00
74 ->	37	-0.00	0.00
74 ->	38	0.02	0.00
74 ->	39	0.00	0.00
74 ->	40	-0.02	0.01
74 ->	41	0.00	0.00
74 ->	42	-0.00	0.00
74 ->	43	-0.00	0.00
74 ->	44	-0.08	0.03
74 ->	45	0.00	0.00
74 ->	46	0.06	0.01
74 ->	47	-0.01	0.01
74 ->	48	-0.00	0.00
74 ->	49	-0.02	0.01
74 ->	50	-0.05	0.05
74 ->	51	-0.00	0.00
74 ->	52	-0.00	0.00
74 ->	53	-0.09	0.06
74 ->	54	-0.00	0.00
74 ->	55	-0.00	0.00
74 ->	56	-0.00	0.00
74 ->	57	-0.00	0.00
74 ->	58	0.02	0.01
74 ->	59	-0.07	0.01
74 ->	60	-0.01	0.00
74 ->	61	-0.00	0.02
74 ->	62	0.00	0.00
74 ->	63	0.04	0.01
74 ->	64	0.02	0.00
74 ->	65	0.00	0.00
74 ->	66	0.03	0.00
74 ->	67	-0.00	0.00
74 ->	68	-0.06	0.02
74 ->	69	-0.05	0.01
74 ->	70	-0.15	0.03
74 ->	71	-0.20	0.04
74 ->	72	-14.70	1.48
74 ->	73	-10.64	0.67
74 ->	74	-14.25	2.35
74 ->	75	-13.41	0.73
74 ->	76	-1.40	0.42
74 ->	77	-2.64	0.68
74 ->	78	-2.16	0.63
74 ->	79	-0.12	0.06
74 ->	80	-0.03	0.01
74 ->	81	-0.12	0.04
74 ->	82	-0.01	0.00
74 ->	83	-0.01	0.00
74 ->	84	-0.00	0.00
74 ->	85	-0.00	0.01

74	->	86	-0.00	0.01
74	->	87	0.03	0.03
74	->	88	-0.50	1.00
74	->	89	0.42	0.16
74	->	90	0.11	0.09
74	->	91	-0.08	0.09
74	->	92	-0.03	0.03
74	->	93	0.00	0.01
74	->	94	0.02	0.00
74	->	95	0.02	0.00
74	->	96	0.01	0.00
74	->	97	0.00	0.00
74	->	98	0.01	0.00
74	->	99	0.02	0.00
74	->	100	0.02	0.00
74	->	101	0.02	0.02
74	->	102	-0.17	0.28
74	->	103	-1.82	0.82
74	->	104	-2.86	1.48
74	->	105	-0.75	0.78
74	->	106	0.02	0.03
74	->	107	0.01	0.00
75	->	1	-0.01	0.00
75	->	2	-0.00	0.00
75	->	3	0.00	0.00
75	->	4	0.00	0.00
75	->	5	0.00	0.00
75	->	6	-0.00	0.00
75	->	7	0.00	0.00
75	->	8	0.00	0.00
75	->	9	0.00	0.00
75	->	10	0.01	0.00
75	->	11	0.00	0.00
75	->	12	-0.01	0.00
75	->	13	-0.00	0.00
75	->	14	-0.00	0.00
75	->	15	-0.00	0.00
75	->	16	-0.01	0.00
75	->	17	0.00	0.00
75	->	18	0.00	0.00
75	->	19	0.01	0.00
75	->	20	0.00	0.00
75	->	21	-0.00	0.00
75	->	22	-0.02	0.00
75	->	23	-0.00	0.00
75	->	24	0.00	0.00
75	->	25	-0.01	0.00
75	->	26	-0.00	0.00
75	->	27	-0.00	0.00
75	->	28	-0.00	0.00

75 ->	29	-0.00	0.00
75 ->	30	0.00	0.00
75 ->	31	-0.02	0.00
75 ->	32	-0.00	0.00
75 ->	33	0.00	0.00
75 ->	34	-0.00	0.00
75 ->	35	0.02	0.01
75 ->	36	-0.01	0.00
75 ->	37	-0.00	0.00
75 ->	38	0.02	0.01
75 ->	39	-0.00	0.00
75 ->	40	-0.03	0.01
75 ->	41	0.00	0.00
75 ->	42	-0.00	0.00
75 ->	43	-0.00	0.00
75 ->	44	-0.03	0.01
75 ->	45	0.00	0.00
75 ->	46	0.04	0.01
75 ->	47	-0.01	0.00
75 ->	48	0.00	0.00
75 ->	49	-0.01	0.01
75 ->	50	-0.04	0.03
75 ->	51	-0.01	0.01
75 ->	52	-0.00	0.00
75 ->	53	-0.10	0.08
75 ->	54	0.02	0.04
75 ->	55	-0.00	0.00
75 ->	56	-0.00	0.00
75 ->	57	-0.00	0.00
75 ->	58	0.03	0.01
75 ->	59	-0.40	0.40
75 ->	60	-0.02	0.01
75 ->	61	-0.10	0.05
75 ->	62	-0.01	0.01
75 ->	63	-0.00	0.02
75 ->	64	0.02	0.01
75 ->	65	-0.00	0.00
75 ->	66	0.04	0.01
75 ->	67	0.00	0.00
75 ->	68	-1.85	3.72
75 ->	69	-0.16	0.18
75 ->	70	-3.49	1.45
75 ->	71	-2.07	1.91
75 ->	72	-4.68	1.28
75 ->	73	-0.77	0.25
75 ->	74	-13.50	0.73
75 ->	75	-45.75	4.23
75 ->	76	-7.59	0.52
75 ->	77	-1.32	0.22
75 ->	78	-5.83	4.25

75	->	79	-3.46	0.56
75	->	80	-0.16	0.05
75	->	81	-0.10	0.03
75	->	82	-0.05	0.02
75	->	83	-0.02	0.00
75	->	84	-0.00	0.00
75	->	85	-0.01	0.01
75	->	86	-0.08	0.09
75	->	87	-0.85	0.78
75	->	88	-1.94	2.19
75	->	89	0.08	0.12
75	->	90	0.01	0.01
75	->	91	0.01	0.01
75	->	92	0.01	0.00
75	->	93	0.01	0.00
75	->	94	0.01	0.00
75	->	95	0.01	0.00
75	->	96	0.00	0.00
75	->	97	0.00	0.00
75	->	98	0.01	0.00
75	->	99	0.01	0.00
75	->	100	0.02	0.00
75	->	101	0.01	0.00
75	->	102	0.01	0.01
75	->	103	-0.00	0.02
75	->	104	-0.02	0.04
75	->	105	-0.21	0.33
75	->	106	0.10	0.19
75	->	107	0.01	0.01
76	->	1	0.01	0.00
76	->	2	0.00	0.00
76	->	3	-0.00	0.00
76	->	4	-0.00	0.00
76	->	5	-0.00	0.00
76	->	6	0.00	0.00
76	->	7	-0.00	0.00
76	->	8	-0.00	0.00
76	->	9	-0.00	0.00
76	->	10	-0.00	0.00
76	->	11	-0.00	0.00
76	->	12	0.00	0.00
76	->	13	0.00	0.00
76	->	14	0.00	0.00
76	->	15	0.00	0.00
76	->	16	0.01	0.00
76	->	17	-0.00	0.00
76	->	18	-0.00	0.00
76	->	19	-0.01	0.00
76	->	20	-0.00	0.00
76	->	21	0.00	0.00

76	->	22	0.01	0.00
76	->	23	0.00	0.00
76	->	24	0.00	0.00
76	->	25	0.01	0.00
76	->	26	0.00	0.00
76	->	27	0.00	0.00
76	->	28	0.00	0.00
76	->	29	0.00	0.00
76	->	30	-0.00	0.00
76	->	31	0.01	0.00
76	->	32	0.00	0.00
76	->	33	0.00	0.00
76	->	34	-0.00	0.00
76	->	35	-0.01	0.00
76	->	36	0.01	0.00
76	->	37	0.00	0.00
76	->	38	-0.01	0.00
76	->	39	0.00	0.00
76	->	40	0.01	0.00
76	->	41	-0.00	0.00
76	->	42	0.00	0.00
76	->	43	0.00	0.00
76	->	44	0.02	0.00
76	->	45	-0.00	0.00
76	->	46	-0.03	0.01
76	->	47	-0.00	0.00
76	->	48	-0.00	0.00
76	->	49	-0.00	0.00
76	->	50	-0.00	0.00
76	->	51	0.00	0.00
76	->	52	-0.00	0.00
76	->	53	0.03	0.01
76	->	54	-0.00	0.00
76	->	55	0.00	0.00
76	->	56	-0.00	0.00
76	->	57	-0.00	0.00
76	->	58	-0.03	0.01
76	->	59	0.04	0.01
76	->	60	-0.04	0.02
76	->	61	-1.01	0.78
76	->	62	-3.13	1.58
76	->	63	-2.02	0.67
76	->	64	-2.74	2.23
76	->	65	-0.05	0.03
76	->	66	-0.11	0.02
76	->	67	-0.01	0.00
76	->	68	-0.12	0.06
76	->	69	0.09	0.05
76	->	70	-2.93	0.51
76	->	71	-0.03	0.02

76	->	72	-0.76	0.46
76	->	73	-2.33	1.02
76	->	74	-1.40	0.42
76	->	75	-7.62	0.51
76	->	76	-104.94	3.04
76	->	77	-11.84	0.57
76	->	78	-0.84	0.27
76	->	79	-2.20	0.39
76	->	80	-3.85	1.44
76	->	81	-0.00	0.06
76	->	82	-0.05	0.02
76	->	83	-0.09	0.02
76	->	84	-0.02	0.01
76	->	85	-0.01	0.01
76	->	86	-0.02	0.01
76	->	87	-0.05	0.01
76	->	88	-0.03	0.00
76	->	89	-0.02	0.00
76	->	90	-0.01	0.00
76	->	91	-0.01	0.00
76	->	92	-0.01	0.00
76	->	93	-0.01	0.00
76	->	94	-0.01	0.00
76	->	95	-0.01	0.00
76	->	96	-0.01	0.00
76	->	97	-0.00	0.00
76	->	98	-0.00	0.00
76	->	99	-0.01	0.00
76	->	100	-0.02	0.00
76	->	101	-0.04	0.01
76	->	102	-0.12	0.07
76	->	103	-0.23	0.26
76	->	104	-0.11	0.06
76	->	105	-0.02	0.01
76	->	106	-0.02	0.00
76	->	107	-0.01	0.00
77	->	1	0.02	0.01
77	->	2	0.00	0.00
77	->	3	-0.00	0.00
77	->	4	0.00	0.00
77	->	5	-0.01	0.00
77	->	6	0.00	0.00
77	->	7	0.00	0.00
77	->	8	-0.00	0.00
77	->	9	-0.00	0.00
77	->	10	-0.01	0.00
77	->	11	-0.01	0.00
77	->	12	0.01	0.01
77	->	13	0.00	0.00
77	->	14	0.00	0.00

77 ->	15	0.00	0.00
77 ->	16	0.02	0.00
77 ->	17	-0.00	0.00
77 ->	18	0.00	0.00
77 ->	19	-0.02	0.00
77 ->	20	-0.00	0.00
77 ->	21	0.00	0.00
77 ->	22	0.02	0.00
77 ->	23	0.00	0.00
77 ->	24	0.00	0.00
77 ->	25	0.01	0.00
77 ->	26	0.00	0.00
77 ->	27	0.00	0.00
77 ->	28	0.00	0.00
77 ->	29	0.00	0.00
77 ->	30	-0.00	0.00
77 ->	31	0.01	0.00
77 ->	32	0.00	0.00
77 ->	33	-0.00	0.00
77 ->	34	-0.00	0.00
77 ->	35	-0.01	0.00
77 ->	36	0.00	0.00
77 ->	37	0.00	0.00
77 ->	38	-0.01	0.00
77 ->	39	0.00	0.00
77 ->	40	0.01	0.00
77 ->	41	-0.00	0.00
77 ->	42	0.00	0.00
77 ->	43	0.00	0.00
77 ->	44	0.03	0.01
77 ->	45	-0.00	0.00
77 ->	46	-0.04	0.01
77 ->	47	-0.00	0.00
77 ->	48	-0.00	0.00
77 ->	49	0.00	0.00
77 ->	50	0.00	0.00
77 ->	51	0.00	0.00
77 ->	52	0.00	0.00
77 ->	53	0.03	0.01
77 ->	54	-0.00	0.00
77 ->	55	0.00	0.00
77 ->	56	0.00	0.00
77 ->	57	0.00	0.00
77 ->	58	-0.02	0.00
77 ->	59	0.01	0.01
77 ->	60	-0.01	0.00
77 ->	61	-0.04	0.03
77 ->	62	-0.01	0.01
77 ->	63	-0.03	0.02
77 ->	64	-0.04	0.01

77	->	65	-0.01	0.00
77	->	66	-0.03	0.00
77	->	67	-0.00	0.00
77	->	68	0.01	0.01
77	->	69	-0.00	0.01
77	->	70	-0.05	0.02
77	->	71	-0.01	0.00
77	->	72	0.12	0.08
77	->	73	-1.92	1.42
77	->	74	-2.67	0.67
77	->	75	-1.33	0.22
77	->	76	-11.86	0.57
77	->	77	13.40	5.25
77	->	78	-6.45	0.46
77	->	79	-0.81	0.19
77	->	80	-1.88	0.46
77	->	81	-1.68	0.77
77	->	82	-0.06	0.04
77	->	83	-0.04	0.02
77	->	84	-0.03	0.02
77	->	85	-0.01	0.01
77	->	86	-0.02	0.02
77	->	87	-0.05	0.02
77	->	88	-0.01	0.01
77	->	89	-0.03	0.00
77	->	90	-0.02	0.00
77	->	91	-0.00	0.02
77	->	92	0.00	0.02
77	->	93	-0.02	0.01
77	->	94	-0.04	0.01
77	->	95	-0.03	0.01
77	->	96	-0.02	0.00
77	->	97	-0.00	0.00
77	->	98	-0.01	0.00
77	->	99	-0.01	0.00
77	->	100	-0.02	0.00
77	->	101	-0.06	0.04
77	->	102	-1.03	0.65
77	->	103	-7.86	4.50
77	->	104	-3.36	2.71
77	->	105	-0.07	0.02
77	->	106	-0.04	0.01
77	->	107	-0.01	0.00
78	->	1	0.01	0.01
78	->	2	0.00	0.00
78	->	3	-0.00	0.00
78	->	4	0.00	0.00
78	->	5	-0.01	0.00
78	->	6	0.00	0.00
78	->	7	-0.00	0.00

78	->	8	-0.00	0.00
78	->	9	-0.00	0.00
78	->	10	-0.01	0.00
78	->	11	-0.01	0.00
78	->	12	0.01	0.01
78	->	13	0.00	0.00
78	->	14	0.00	0.00
78	->	15	0.00	0.00
78	->	16	0.01	0.00
78	->	17	-0.00	0.00
78	->	18	-0.00	0.00
78	->	19	-0.01	0.00
78	->	20	-0.00	0.00
78	->	21	0.00	0.00
78	->	22	0.02	0.00
78	->	23	0.00	0.00
78	->	24	0.00	0.00
78	->	25	0.01	0.00
78	->	26	0.00	0.00
78	->	27	0.00	0.00
78	->	28	0.00	0.00
78	->	29	0.00	0.00
78	->	30	-0.00	0.00
78	->	31	0.02	0.00
78	->	32	0.00	0.00
78	->	33	-0.00	0.00
78	->	34	0.00	0.00
78	->	35	-0.01	0.00
78	->	36	0.01	0.00
78	->	37	0.00	0.00
78	->	38	-0.01	0.00
78	->	39	0.00	0.00
78	->	40	0.02	0.01
78	->	41	-0.00	0.00
78	->	42	0.00	0.00
78	->	43	0.00	0.00
78	->	44	0.02	0.00
78	->	45	-0.00	0.00
78	->	46	-0.03	0.00
78	->	47	-0.00	0.00
78	->	48	-0.00	0.00
78	->	49	0.00	0.00
78	->	50	-0.00	0.00
78	->	51	0.00	0.00
78	->	52	-0.00	0.00
78	->	53	0.02	0.01
78	->	54	-0.00	0.00
78	->	55	0.00	0.00
78	->	56	0.00	0.00
78	->	57	0.00	0.00

78	->	58	-0.02	0.00
78	->	59	0.04	0.01
78	->	60	-0.00	0.00
78	->	61	-0.01	0.00
78	->	62	0.00	0.01
78	->	63	-0.18	0.04
78	->	64	-0.03	0.01
78	->	65	-0.02	0.00
78	->	66	-0.08	0.01
78	->	67	0.00	0.00
78	->	68	0.14	0.08
78	->	69	0.04	0.01
78	->	70	-0.23	0.07
78	->	71	-0.00	0.01
78	->	72	0.32	0.18
78	->	73	-0.10	0.08
78	->	74	-2.20	0.63
78	->	75	-5.87	4.29
78	->	76	-0.84	0.26
78	->	77	-6.48	0.46
78	->	78	-101.77	3.35
78	->	79	-19.56	0.40
78	->	80	-0.74	0.22
78	->	81	-2.08	0.35
78	->	82	-1.74	1.02
78	->	83	-0.13	0.05
78	->	84	-0.03	0.01
78	->	85	-0.02	0.02
78	->	86	-1.22	1.54
78	->	87	-4.51	2.75
78	->	88	0.09	1.09
78	->	89	-0.14	0.14
78	->	90	-0.04	0.01
78	->	91	-0.03	0.01
78	->	92	-0.02	0.00
78	->	93	-0.02	0.00
78	->	94	-0.01	0.00
78	->	95	-0.01	0.00
78	->	96	-0.00	0.00
78	->	97	-0.00	0.00
78	->	98	-0.00	0.00
78	->	99	-0.01	0.00
78	->	100	-0.01	0.00
78	->	101	-0.02	0.00
78	->	102	-0.04	0.01
78	->	103	-0.10	0.04
78	->	104	-0.37	0.36
78	->	105	-1.98	1.46
78	->	106	-5.19	2.92
78	->	107	-0.23	0.37

79	->	1	0.00	0.00
79	->	2	0.00	0.00
79	->	3	0.00	0.00
79	->	4	0.00	0.00
79	->	5	-0.00	0.00
79	->	6	0.00	0.00
79	->	7	0.00	0.00
79	->	8	-0.00	0.00
79	->	9	0.00	0.00
79	->	10	-0.00	0.00
79	->	11	-0.00	0.00
79	->	12	0.00	0.00
79	->	13	0.00	0.00
79	->	14	0.00	0.00
79	->	15	0.00	0.00
79	->	16	0.00	0.00
79	->	17	-0.00	0.00
79	->	18	-0.00	0.00
79	->	19	-0.00	0.00
79	->	20	0.00	0.00
79	->	21	0.00	0.00
79	->	22	0.00	0.00
79	->	23	0.00	0.00
79	->	24	0.00	0.00
79	->	25	0.00	0.00
79	->	26	0.00	0.00
79	->	27	0.00	0.00
79	->	28	0.00	0.00
79	->	29	0.00	0.00
79	->	30	-0.00	0.00
79	->	31	0.00	0.00
79	->	32	0.00	0.00
79	->	33	-0.00	0.00
79	->	34	-0.00	0.00
79	->	35	-0.00	0.00
79	->	36	0.00	0.00
79	->	37	0.00	0.00
79	->	38	-0.00	0.00
79	->	39	0.00	0.00
79	->	40	0.00	0.00
79	->	41	-0.00	0.00
79	->	42	0.00	0.00
79	->	43	0.00	0.00
79	->	44	0.00	0.00
79	->	45	-0.00	0.00
79	->	46	-0.00	0.00
79	->	47	-0.00	0.00
79	->	48	-0.00	0.00
79	->	49	-0.00	0.00
79	->	50	-0.00	0.00

79	->	51	-0.00	0.00
79	->	52	-0.00	0.00
79	->	53	0.00	0.00
79	->	54	-0.00	0.00
79	->	55	0.00	0.00
79	->	56	0.00	0.00
79	->	57	-0.00	0.00
79	->	58	-0.00	0.00
79	->	59	-0.00	0.00
79	->	60	-0.00	0.00
79	->	61	-0.02	0.01
79	->	62	-0.09	0.02
79	->	63	2.95	0.87
79	->	64	-0.07	0.07
79	->	65	0.72	0.10
79	->	66	3.53	0.68
79	->	67	-0.08	0.05
79	->	68	-1.80	0.59
79	->	69	-0.02	0.01
79	->	70	-1.82	0.29
79	->	71	-0.00	0.00
79	->	72	-0.00	0.01
79	->	73	-0.04	0.03
79	->	74	-0.12	0.06
79	->	75	-3.49	0.55
79	->	76	-2.21	0.39
79	->	77	-0.82	0.19
79	->	78	-19.60	0.40
79	->	79	10.47	1.57
79	->	80	-4.69	0.47
79	->	81	-1.11	0.24
79	->	82	-3.58	0.80
79	->	83	17.76	10.28
79	->	84	-0.11	0.05
79	->	85	-0.03	0.03
79	->	86	-0.37	0.79
79	->	87	-0.99	2.25
79	->	88	-0.02	0.01
79	->	89	-0.01	0.00
79	->	90	-0.00	0.00
79	->	91	-0.00	0.00
79	->	92	-0.00	0.00
79	->	93	-0.00	0.00
79	->	94	-0.00	0.00
79	->	95	-0.00	0.00
79	->	96	-0.00	0.00
79	->	97	-0.00	0.00
79	->	98	-0.00	0.00
79	->	99	-0.00	0.00
79	->	100	-0.00	0.00

79	->	101	-0.00	0.00
79	->	102	-0.01	0.00
79	->	103	-0.01	0.01
79	->	104	-0.01	0.01
79	->	105	-0.01	0.00
79	->	106	-0.01	0.00
79	->	107	-0.00	0.00
80	->	1	0.00	0.00
80	->	2	0.00	0.00
80	->	3	0.00	0.00
80	->	4	0.00	0.00
80	->	5	-0.00	0.00
80	->	6	0.00	0.00
80	->	7	0.00	0.00
80	->	8	-0.00	0.00
80	->	9	0.00	0.00
80	->	10	-0.00	0.00
80	->	11	-0.00	0.00
80	->	12	0.00	0.00
80	->	13	0.00	0.00
80	->	14	0.00	0.00
80	->	15	0.00	0.00
80	->	16	0.00	0.00
80	->	17	0.00	0.00
80	->	18	0.00	0.00
80	->	19	-0.00	0.00
80	->	20	0.00	0.00
80	->	21	0.00	0.00
80	->	22	0.00	0.00
80	->	23	0.00	0.00
80	->	24	0.00	0.00
80	->	25	0.00	0.00
80	->	26	0.00	0.00
80	->	27	0.00	0.00
80	->	28	0.00	0.00
80	->	29	0.00	0.00
80	->	30	-0.00	0.00
80	->	31	0.00	0.00
80	->	32	0.00	0.00
80	->	33	0.00	0.00
80	->	34	0.00	0.00
80	->	35	-0.00	0.00
80	->	36	0.00	0.00
80	->	37	0.00	0.00
80	->	38	-0.00	0.00
80	->	39	0.00	0.00
80	->	40	0.00	0.00
80	->	41	0.00	0.00
80	->	42	0.00	0.00
80	->	43	0.00	0.00

80	->	44	0.00	0.00
80	->	45	-0.00	0.00
80	->	46	-0.00	0.00
80	->	47	0.00	0.00
80	->	48	0.00	0.00
80	->	49	-0.00	0.00
80	->	50	-0.00	0.00
80	->	51	0.00	0.00
80	->	52	0.00	0.00
80	->	53	0.00	0.00
80	->	54	0.00	0.00
80	->	55	0.00	0.00
80	->	56	0.00	0.00
80	->	57	0.00	0.00
80	->	58	-0.00	0.00
80	->	59	-0.00	0.00
80	->	60	-0.00	0.00
80	->	61	-0.01	0.01
80	->	62	-0.03	0.02
80	->	63	0.13	0.19
80	->	64	-0.19	0.18
80	->	65	-0.09	0.09
80	->	66	0.04	0.02
80	->	67	-0.01	0.00
80	->	68	-0.04	0.02
80	->	69	-0.01	0.01
80	->	70	-0.05	0.03
80	->	71	-0.00	0.00
80	->	72	-0.01	0.01
80	->	73	-0.02	0.01
80	->	74	-0.03	0.01
80	->	75	-0.17	0.05
80	->	76	-3.83	1.45
80	->	77	-1.89	0.46
80	->	78	-0.75	0.22
80	->	79	-4.72	0.47
80	->	80	-23.00	1.90
80	->	81	-15.31	0.43
80	->	82	-0.89	0.20
80	->	83	-2.77	0.64
80	->	84	-2.13	0.99
80	->	85	-0.04	0.05
80	->	86	-0.00	0.00
80	->	87	0.00	0.01
80	->	88	-0.00	0.00
80	->	89	-0.00	0.00
80	->	90	-0.00	0.00
80	->	91	-0.00	0.00
80	->	92	-0.00	0.00
80	->	93	-0.00	0.00

80	->	94	-0.00	0.00
80	->	95	-0.00	0.00
80	->	96	-0.00	0.00
80	->	97	-0.00	0.00
80	->	98	-0.00	0.00
80	->	99	-0.00	0.00
80	->	100	-0.00	0.00
80	->	101	-0.00	0.00
80	->	102	-0.01	0.00
80	->	103	-0.02	0.02
80	->	104	-0.03	0.02
80	->	105	-0.01	0.00
80	->	106	-0.00	0.00
80	->	107	-0.00	0.00
81	->	1	0.02	0.01
81	->	2	0.00	0.00
81	->	3	-0.00	0.00
81	->	4	0.00	0.00
81	->	5	-0.00	0.00
81	->	6	0.00	0.00
81	->	7	-0.00	0.00
81	->	8	-0.00	0.00
81	->	9	-0.00	0.00
81	->	10	-0.01	0.00
81	->	11	-0.01	0.00
81	->	12	0.01	0.01
81	->	13	0.00	0.00
81	->	14	0.00	0.00
81	->	15	0.00	0.00
81	->	16	0.01	0.00
81	->	17	-0.00	0.00
81	->	18	-0.00	0.00
81	->	19	-0.01	0.00
81	->	20	-0.00	0.00
81	->	21	0.00	0.00
81	->	22	0.01	0.01
81	->	23	0.00	0.00
81	->	24	0.00	0.00
81	->	25	0.01	0.00
81	->	26	0.00	0.00
81	->	27	0.00	0.00
81	->	28	0.00	0.00
81	->	29	0.00	0.00
81	->	30	-0.00	0.00
81	->	31	0.01	0.00
81	->	32	0.00	0.00
81	->	33	-0.00	0.00
81	->	34	0.00	0.00
81	->	35	-0.00	0.00
81	->	36	0.00	0.00

81	->	37	0.00	0.00
81	->	38	-0.01	0.00
81	->	39	0.00	0.00
81	->	40	0.01	0.00
81	->	41	-0.00	0.00
81	->	42	0.00	0.00
81	->	43	0.00	0.00
81	->	44	0.02	0.01
81	->	45	-0.00	0.00
81	->	46	-0.02	0.00
81	->	47	0.00	0.00
81	->	48	-0.00	0.00
81	->	49	0.00	0.00
81	->	50	0.00	0.00
81	->	51	0.00	0.00
81	->	52	0.00	0.00
81	->	53	0.01	0.00
81	->	54	0.00	0.00
81	->	55	0.00	0.00
81	->	56	0.00	0.00
81	->	57	0.00	0.00
81	->	58	-0.01	0.00
81	->	59	0.02	0.00
81	->	60	-0.00	0.00
81	->	61	-0.00	0.00
81	->	62	-0.01	0.00
81	->	63	-0.00	0.02
81	->	64	-0.03	0.01
81	->	65	-0.01	0.00
81	->	66	-0.03	0.01
81	->	67	-0.00	0.00
81	->	68	0.02	0.00
81	->	69	0.01	0.00
81	->	70	-0.00	0.00
81	->	71	0.00	0.00
81	->	72	0.04	0.01
81	->	73	-0.01	0.01
81	->	74	-0.12	0.04
81	->	75	-0.10	0.03
81	->	76	-0.00	0.06
81	->	77	-1.70	0.76
81	->	78	-2.11	0.35
81	->	79	-1.12	0.23
81	->	80	-15.35	0.43
81	->	81	7.82	3.97
81	->	82	-11.11	0.52
81	->	83	-0.68	0.26
81	->	84	-1.74	0.64
81	->	85	-0.62	0.68
81	->	86	-0.04	0.05

81	->	87	-0.05	0.02
81	->	88	-0.02	0.00
81	->	89	-0.02	0.00
81	->	90	-0.02	0.00
81	->	91	-0.01	0.00
81	->	92	-0.01	0.00
81	->	93	-0.02	0.01
81	->	94	-0.02	0.01
81	->	95	-0.01	0.01
81	->	96	-0.01	0.00
81	->	97	-0.00	0.00
81	->	98	-0.00	0.00
81	->	99	-0.01	0.00
81	->	100	-0.01	0.00
81	->	101	-0.02	0.01
81	->	102	-0.05	0.02
81	->	103	-0.56	0.62
81	->	104	-3.34	3.78
81	->	105	-0.34	0.77
81	->	106	-0.06	0.01
81	->	107	-0.01	0.00
82	->	1	0.00	0.00
82	->	2	0.00	0.00
82	->	3	0.00	0.00
82	->	4	0.00	0.00
82	->	5	-0.00	0.00
82	->	6	0.00	0.00
82	->	7	0.00	0.00
82	->	8	-0.00	0.00
82	->	9	0.00	0.00
82	->	10	-0.00	0.00
82	->	11	-0.00	0.00
82	->	12	0.00	0.00
82	->	13	0.00	0.00
82	->	14	0.00	0.00
82	->	15	0.00	0.00
82	->	16	0.00	0.00
82	->	17	-0.00	0.00
82	->	18	0.00	0.00
82	->	19	-0.00	0.00
82	->	20	0.00	0.00
82	->	21	0.00	0.00
82	->	22	0.00	0.00
82	->	23	0.00	0.00
82	->	24	0.00	0.00
82	->	25	0.00	0.00
82	->	26	0.00	0.00
82	->	27	0.00	0.00
82	->	28	0.00	0.00
82	->	29	0.00	0.00

82	->	30	0.00	0.00
82	->	31	0.00	0.00
82	->	32	0.00	0.00
82	->	33	0.00	0.00
82	->	34	0.00	0.00
82	->	35	-0.00	0.00
82	->	36	0.00	0.00
82	->	37	0.00	0.00
82	->	38	-0.00	0.00
82	->	39	0.00	0.00
82	->	40	0.00	0.00
82	->	41	0.00	0.00
82	->	42	0.00	0.00
82	->	43	0.00	0.00
82	->	44	0.00	0.00
82	->	45	-0.00	0.00
82	->	46	-0.00	0.00
82	->	47	0.00	0.00
82	->	48	0.00	0.00
82	->	49	0.00	0.00
82	->	50	-0.00	0.00
82	->	51	0.00	0.00
82	->	52	0.00	0.00
82	->	53	0.00	0.00
82	->	54	-0.00	0.00
82	->	55	0.00	0.00
82	->	56	0.00	0.00
82	->	57	-0.00	0.00
82	->	58	-0.00	0.00
82	->	59	0.00	0.00
82	->	60	-0.00	0.00
82	->	61	-0.00	0.00
82	->	62	-0.00	0.00
82	->	63	-0.07	0.02
82	->	64	-0.01	0.00
82	->	65	-0.04	0.01
82	->	66	-0.06	0.02
82	->	67	-0.00	0.00
82	->	68	-0.04	0.05
82	->	69	-0.00	0.00
82	->	70	-0.03	0.01
82	->	71	-0.00	0.00
82	->	72	-0.00	0.00
82	->	73	-0.00	0.00
82	->	74	-0.01	0.00
82	->	75	-0.05	0.02
82	->	76	-0.05	0.02
82	->	77	-0.06	0.04
82	->	78	-1.76	1.01
82	->	79	-3.59	0.82

82	->	80	-0.90	0.20
82	->	81	-11.20	0.53
82	->	82	17.54	1.94
82	->	83	-22.11	0.71
82	->	84	-0.85	0.33
82	->	85	-1.50	1.57
82	->	86	-0.55	0.85
82	->	87	-0.12	0.25
82	->	88	-0.01	0.00
82	->	89	-0.00	0.00
82	->	90	-0.00	0.00
82	->	91	-0.00	0.00
82	->	92	-0.00	0.00
82	->	93	-0.00	0.00
82	->	94	-0.00	0.00
82	->	95	-0.00	0.00
82	->	96	-0.00	0.00
82	->	97	-0.00	0.00
82	->	98	-0.00	0.00
82	->	99	-0.00	0.00
82	->	100	-0.00	0.00
82	->	101	-0.00	0.00
82	->	102	-0.00	0.00
82	->	103	-0.00	0.00
82	->	104	-0.01	0.00
82	->	105	-0.01	0.01
82	->	106	-0.01	0.00
82	->	107	-0.01	0.00
83	->	1	0.00	0.00
83	->	2	0.00	0.00
83	->	3	0.00	0.00
83	->	4	0.00	0.00
83	->	5	-0.00	0.00
83	->	6	0.00	0.00
83	->	7	0.00	0.00
83	->	8	-0.00	0.00
83	->	9	0.00	0.00
83	->	10	-0.00	0.00
83	->	11	-0.00	0.00
83	->	12	0.00	0.00
83	->	13	0.00	0.00
83	->	14	0.00	0.00
83	->	15	0.00	0.00
83	->	16	0.00	0.00
83	->	17	0.00	0.00
83	->	18	0.00	0.00
83	->	19	-0.00	0.00
83	->	20	0.00	0.00
83	->	21	0.00	0.00
83	->	22	0.00	0.00

83 ->	23	0.00	0.00
83 ->	24	0.00	0.00
83 ->	25	0.00	0.00
83 ->	26	0.00	0.00
83 ->	27	0.00	0.00
83 ->	28	0.00	0.00
83 ->	29	0.00	0.00
83 ->	30	0.00	0.00
83 ->	31	0.00	0.00
83 ->	32	0.00	0.00
83 ->	33	0.00	0.00
83 ->	34	0.00	0.00
83 ->	35	0.00	0.00
83 ->	36	-0.00	0.00
83 ->	37	-0.00	0.00
83 ->	38	0.00	0.00
83 ->	39	0.00	0.00
83 ->	40	0.00	0.00
83 ->	41	0.00	0.00
83 ->	42	0.00	0.00
83 ->	43	0.00	0.00
83 ->	44	0.00	0.00
83 ->	45	0.00	0.00
83 ->	46	-0.00	0.00
83 ->	47	0.00	0.00
83 ->	48	0.00	0.00
83 ->	49	0.00	0.00
83 ->	50	0.00	0.00
83 ->	51	0.00	0.00
83 ->	52	0.00	0.00
83 ->	53	-0.00	0.00
83 ->	54	0.00	0.00
83 ->	55	-0.00	0.00
83 ->	56	0.00	0.00
83 ->	57	0.00	0.00
83 ->	58	0.00	0.00
83 ->	59	-0.00	0.00
83 ->	60	-0.00	0.00
83 ->	61	-0.01	0.00
83 ->	62	-0.04	0.01
83 ->	63	2.28	0.30
83 ->	64	-0.20	0.14
83 ->	65	0.84	0.58
83 ->	66	2.09	0.29
83 ->	67	-0.02	0.03
83 ->	68	-0.23	0.07
83 ->	69	-0.01	0.00
83 ->	70	-0.00	0.01
83 ->	71	-0.00	0.00
83 ->	72	-0.00	0.00

83	->	73	-0.00	0.00
83	->	74	-0.01	0.00
83	->	75	-0.02	0.00
83	->	76	-0.09	0.02
83	->	77	-0.04	0.02
83	->	78	-0.13	0.05
83	->	79	17.75	10.28
83	->	80	-2.78	0.63
83	->	81	-0.69	0.26
83	->	82	-22.21	0.71
83	->	83	4.46	1.45
83	->	84	-8.24	0.49
83	->	85	-0.89	0.74
83	->	86	-0.00	0.01
83	->	87	0.02	0.05
83	->	88	-0.00	0.00
83	->	89	-0.00	0.00
83	->	90	-0.00	0.00
83	->	91	-0.00	0.00
83	->	92	-0.00	0.00
83	->	93	-0.00	0.00
83	->	94	-0.00	0.00
83	->	95	-0.00	0.00
83	->	96	-0.00	0.00
83	->	97	-0.00	0.00
83	->	98	-0.00	0.00
83	->	99	-0.00	0.00
83	->	100	0.00	0.00
83	->	101	-0.00	0.00
83	->	102	-0.00	0.00
83	->	103	-0.00	0.00
83	->	104	-0.01	0.00
83	->	105	-0.00	0.00
83	->	106	-0.00	0.00
83	->	107	-0.00	0.00
84	->	1	0.00	0.00
84	->	2	0.00	0.00
84	->	3	-0.00	0.00
84	->	4	0.00	0.00
84	->	5	0.00	0.00
84	->	6	0.00	0.00
84	->	7	0.00	0.00
84	->	8	0.00	0.00
84	->	9	0.00	0.00
84	->	10	-0.00	0.00
84	->	11	-0.00	0.00
84	->	12	0.00	0.00
84	->	13	0.00	0.00
84	->	14	0.00	0.00
84	->	15	0.00	0.00

84	->	16	0.00	0.00
84	->	17	0.00	0.00
84	->	18	0.00	0.00
84	->	19	-0.00	0.00
84	->	20	0.00	0.00
84	->	21	0.00	0.00
84	->	22	0.00	0.00
84	->	23	0.00	0.00
84	->	24	0.00	0.00
84	->	25	0.00	0.00
84	->	26	0.00	0.00
84	->	27	0.00	0.00
84	->	28	0.00	0.00
84	->	29	0.00	0.00
84	->	30	0.00	0.00
84	->	31	0.00	0.00
84	->	32	0.00	0.00
84	->	33	0.00	0.00
84	->	34	0.00	0.00
84	->	35	0.00	0.00
84	->	36	0.00	0.00
84	->	37	0.00	0.00
84	->	38	-0.00	0.00
84	->	39	0.00	0.00
84	->	40	0.00	0.00
84	->	41	0.00	0.00
84	->	42	0.00	0.00
84	->	43	0.00	0.00
84	->	44	0.00	0.00
84	->	45	0.00	0.00
84	->	46	-0.00	0.00
84	->	47	-0.00	0.00
84	->	48	0.00	0.00
84	->	49	0.00	0.00
84	->	50	0.00	0.00
84	->	51	0.00	0.00
84	->	52	0.00	0.00
84	->	53	0.00	0.00
84	->	54	0.00	0.00
84	->	55	-0.00	0.00
84	->	56	0.00	0.00
84	->	57	0.00	0.00
84	->	58	-0.00	0.00
84	->	59	0.00	0.00
84	->	60	-0.00	0.00
84	->	61	-0.00	0.00
84	->	62	-0.00	0.00
84	->	63	-0.00	0.01
84	->	64	-0.02	0.02
84	->	65	-0.08	0.20

84	->	66	-0.00	0.00
84	->	67	-0.00	0.00
84	->	68	-0.00	0.00
84	->	69	-0.00	0.00
84	->	70	-0.00	0.00
84	->	71	-0.00	0.00
84	->	72	-0.00	0.00
84	->	73	-0.00	0.00
84	->	74	-0.00	0.00
84	->	75	-0.00	0.00
84	->	76	-0.02	0.01
84	->	77	-0.03	0.02
84	->	78	-0.03	0.01
84	->	79	-0.11	0.05
84	->	80	-2.15	0.97
84	->	81	-1.75	0.63
84	->	82	-0.85	0.32
84	->	83	-8.38	0.49
84	->	84	17.49	1.56
84	->	85	-8.00	0.91
84	->	86	-0.00	0.00
84	->	87	-0.00	0.00
84	->	88	-0.00	0.00
84	->	89	-0.00	0.00
84	->	90	-0.00	0.00
84	->	91	0.00	0.00
84	->	92	-0.00	0.00
84	->	93	-0.00	0.00
84	->	94	-0.00	0.00
84	->	95	-0.00	0.00
84	->	96	-0.00	0.00
84	->	97	-0.00	0.00
84	->	98	-0.00	0.00
84	->	99	-0.00	0.00
84	->	100	-0.00	0.00
84	->	101	-0.00	0.00
84	->	102	-0.00	0.00
84	->	103	-0.00	0.00
84	->	104	-0.01	0.01
84	->	105	-0.00	0.00
84	->	106	-0.00	0.00
84	->	107	-0.00	0.00
85	->	1	0.00	0.00
85	->	2	-0.00	0.00
85	->	3	-0.00	0.00
85	->	4	-0.00	0.00
85	->	5	-0.00	0.00
85	->	6	0.00	0.00
85	->	7	0.00	0.00
85	->	8	-0.00	0.00

85	->	9	0.00	0.00
85	->	10	-0.00	0.00
85	->	11	-0.00	0.00
85	->	12	0.00	0.00
85	->	13	0.00	0.00
85	->	14	0.00	0.00
85	->	15	-0.00	0.00
85	->	16	0.00	0.00
85	->	17	0.00	0.00
85	->	18	-0.00	0.00
85	->	19	-0.00	0.00
85	->	20	0.00	0.00
85	->	21	0.00	0.00
85	->	22	0.00	0.00
85	->	23	0.00	0.00
85	->	24	0.00	0.00
85	->	25	0.00	0.00
85	->	26	0.00	0.00
85	->	27	0.00	0.00
85	->	28	0.00	0.00
85	->	29	0.00	0.00
85	->	30	0.00	0.00
85	->	31	0.00	0.00
85	->	32	0.00	0.00
85	->	33	0.00	0.00
85	->	34	-0.00	0.00
85	->	35	-0.00	0.00
85	->	36	0.00	0.00
85	->	37	0.00	0.00
85	->	38	-0.00	0.00
85	->	39	-0.00	0.00
85	->	40	0.00	0.00
85	->	41	-0.00	0.00
85	->	42	0.00	0.00
85	->	43	0.00	0.00
85	->	44	0.00	0.00
85	->	45	-0.00	0.00
85	->	46	-0.00	0.00
85	->	47	0.00	0.00
85	->	48	-0.00	0.00
85	->	49	0.00	0.00
85	->	50	0.00	0.00
85	->	51	0.00	0.00
85	->	52	-0.00	0.00
85	->	53	0.00	0.00
85	->	54	-0.00	0.00
85	->	55	0.00	0.00
85	->	56	0.00	0.00
85	->	57	-0.00	0.00
85	->	58	-0.00	0.00

85	->	59	0.00	0.00
85	->	60	0.00	0.00
85	->	61	-0.00	0.00
85	->	62	-0.00	0.00
85	->	63	-0.00	0.01
85	->	64	0.00	0.01
85	->	65	-0.03	0.07
85	->	66	-0.01	0.03
85	->	67	-0.00	0.00
85	->	68	0.00	0.01
85	->	69	-0.00	0.00
85	->	70	-0.00	0.00
85	->	71	0.00	0.00
85	->	72	0.00	0.01
85	->	73	-0.00	0.00
85	->	74	-0.00	0.01
85	->	75	-0.01	0.01
85	->	76	-0.01	0.01
85	->	77	-0.01	0.01
85	->	78	-0.02	0.02
85	->	79	-0.03	0.03
85	->	80	-0.04	0.05
85	->	81	-0.63	0.68
85	->	82	-1.51	1.57
85	->	83	-0.91	0.74
85	->	84	-8.07	0.91
85	->	85	-267.76	3.42
85	->	86	-0.01	0.02
85	->	87	-0.01	0.01
85	->	88	-0.00	0.00
85	->	89	-0.00	0.00
85	->	90	-0.00	0.00
85	->	91	-0.00	0.00
85	->	92	-0.00	0.00
85	->	93	-0.00	0.00
85	->	94	-0.00	0.00
85	->	95	-0.00	0.00
85	->	96	-0.00	0.00
85	->	97	-0.00	0.00
85	->	98	-0.00	0.00
85	->	99	-0.00	0.00
85	->	100	-0.00	0.00
85	->	101	-0.00	0.00
85	->	102	-0.00	0.01
85	->	103	-0.00	0.02
85	->	104	-0.02	0.07
85	->	105	-0.04	0.13
85	->	106	-0.01	0.02
85	->	107	-0.00	0.00
86	->	1	-0.00	0.00

86 ->	2	-0.00	0.00
86 ->	3	0.00	0.00
86 ->	4	-0.00	0.00
86 ->	5	0.00	0.00
86 ->	6	0.00	0.00
86 ->	7	0.00	0.00
86 ->	8	0.00	0.00
86 ->	9	0.00	0.00
86 ->	10	0.00	0.00
86 ->	11	0.00	0.00
86 ->	12	-0.00	0.00
86 ->	13	-0.00	0.00
86 ->	14	-0.00	0.00
86 ->	15	-0.00	0.00
86 ->	16	-0.00	0.00
86 ->	17	0.00	0.00
86 ->	18	0.00	0.00
86 ->	19	0.00	0.00
86 ->	20	0.00	0.00
86 ->	21	0.00	0.00
86 ->	22	-0.00	0.00
86 ->	23	-0.00	0.00
86 ->	24	0.00	0.00
86 ->	25	-0.00	0.00
86 ->	26	0.00	0.00
86 ->	27	-0.00	0.00
86 ->	28	-0.00	0.00
86 ->	29	-0.00	0.00
86 ->	30	0.00	0.00
86 ->	31	-0.00	0.00
86 ->	32	0.00	0.00
86 ->	33	0.00	0.00
86 ->	34	0.00	0.00
86 ->	35	0.00	0.00
86 ->	36	-0.00	0.00
86 ->	37	-0.00	0.00
86 ->	38	0.00	0.00
86 ->	39	-0.00	0.00
86 ->	40	-0.01	0.00
86 ->	41	0.00	0.00
86 ->	42	-0.00	0.00
86 ->	43	-0.00	0.00
86 ->	44	-0.00	0.00
86 ->	45	0.00	0.00
86 ->	46	0.00	0.00
86 ->	47	-0.00	0.00
86 ->	48	-0.00	0.00
86 ->	49	-0.00	0.00
86 ->	50	-0.00	0.00
86 ->	51	-0.00	0.00

86 ->	52	-0.00	0.00
86 ->	53	-0.01	0.01
86 ->	54	-0.00	0.00
86 ->	55	-0.00	0.00
86 ->	56	-0.00	0.00
86 ->	57	-0.00	0.00
86 ->	58	0.00	0.00
86 ->	59	-0.13	0.20
86 ->	60	-0.00	0.00
86 ->	61	-0.00	0.00
86 ->	62	-0.00	0.00
86 ->	63	0.02	0.02
86 ->	64	0.01	0.00
86 ->	65	-0.00	0.00
86 ->	66	0.03	0.05
86 ->	67	-0.01	0.01
86 ->	68	-1.89	1.19
86 ->	69	-0.05	0.05
86 ->	70	-0.11	0.22
86 ->	71	-0.00	0.00
86 ->	72	-0.03	0.01
86 ->	73	-0.00	0.00
86 ->	74	-0.00	0.01
86 ->	75	-0.08	0.09
86 ->	76	-0.02	0.01
86 ->	77	-0.02	0.02
86 ->	78	-1.21	1.52
86 ->	79	-0.36	0.78
86 ->	80	-0.00	0.00
86 ->	81	-0.04	0.05
86 ->	82	-0.54	0.85
86 ->	83	-0.00	0.01
86 ->	84	-0.00	0.00
86 ->	85	-0.01	0.02
86 ->	86	-86.08	2.64
86 ->	87	-35.31	1.42
86 ->	88	-0.31	0.07
86 ->	89	-0.03	0.01
86 ->	90	-0.00	0.00
86 ->	91	0.00	0.00
86 ->	92	0.00	0.00
86 ->	93	0.00	0.00
86 ->	94	0.00	0.00
86 ->	95	0.00	0.00
86 ->	96	0.00	0.00
86 ->	97	0.00	0.00
86 ->	98	0.00	0.00
86 ->	99	0.00	0.00
86 ->	100	0.00	0.00
86 ->	101	0.00	0.00

86	->	102	0.00	0.00
86	->	103	0.00	0.00
86	->	104	-0.00	0.00
86	->	105	-0.03	0.01
86	->	106	-0.18	0.07
86	->	107	-2.05	1.12
87	->	1	-0.01	0.00
87	->	2	-0.00	0.00
87	->	3	0.00	0.00
87	->	4	0.00	0.00
87	->	5	0.00	0.00
87	->	6	-0.00	0.00
87	->	7	0.00	0.00
87	->	8	0.00	0.00
87	->	9	0.00	0.00
87	->	10	0.00	0.00
87	->	11	0.00	0.00
87	->	12	-0.00	0.00
87	->	13	-0.00	0.00
87	->	14	-0.00	0.00
87	->	15	-0.00	0.00
87	->	16	-0.00	0.00
87	->	17	0.00	0.00
87	->	18	0.00	0.00
87	->	19	0.01	0.00
87	->	20	0.00	0.00
87	->	21	0.00	0.00
87	->	22	-0.01	0.00
87	->	23	-0.00	0.00
87	->	24	0.00	0.00
87	->	25	-0.01	0.00
87	->	26	-0.00	0.00
87	->	27	-0.00	0.00
87	->	28	-0.00	0.00
87	->	29	-0.00	0.00
87	->	30	0.00	0.00
87	->	31	-0.01	0.00
87	->	32	0.00	0.00
87	->	33	-0.00	0.00
87	->	34	-0.00	0.00
87	->	35	0.02	0.01
87	->	36	-0.01	0.00
87	->	37	-0.00	0.00
87	->	38	0.03	0.01
87	->	39	-0.00	0.00
87	->	40	-0.04	0.04
87	->	41	-0.00	0.00
87	->	42	-0.00	0.00
87	->	43	-0.00	0.00
87	->	44	-0.02	0.00

87	->	45	0.00	0.00
87	->	46	0.02	0.00
87	->	47	-0.01	0.01
87	->	48	0.00	0.00
87	->	49	-0.01	0.01
87	->	50	-0.01	0.01
87	->	51	-0.03	0.02
87	->	52	0.00	0.01
87	->	53	-0.10	0.08
87	->	54	-0.10	0.13
87	->	55	0.01	0.01
87	->	56	-0.01	0.01
87	->	57	-0.02	0.02
87	->	58	0.02	0.01
87	->	59	-1.34	1.65
87	->	60	-0.01	0.00
87	->	61	-0.01	0.00
87	->	62	-0.01	0.01
87	->	63	0.08	0.07
87	->	64	0.03	0.01
87	->	65	0.00	0.01
87	->	66	0.14	0.18
87	->	67	-0.01	0.01
87	->	68	-5.98	4.37
87	->	69	-0.16	0.12
87	->	70	-0.23	0.21
87	->	71	-0.08	0.11
87	->	72	-0.37	0.42
87	->	73	0.00	0.01
87	->	74	0.03	0.03
87	->	75	-0.89	0.81
87	->	76	-0.05	0.01
87	->	77	-0.05	0.02
87	->	78	-4.47	2.76
87	->	79	-0.98	2.22
87	->	80	0.00	0.01
87	->	81	-0.05	0.02
87	->	82	-0.12	0.24
87	->	83	0.02	0.05
87	->	84	-0.00	0.00
87	->	85	-0.01	0.01
87	->	86	-35.15	1.41
87	->	87	-171.18	4.57
87	->	88	-36.01	1.02
87	->	89	-0.30	0.07
87	->	90	-0.02	0.01
87	->	91	0.01	0.00
87	->	92	0.01	0.00
87	->	93	0.01	0.00
87	->	94	0.01	0.00

87	->	95	0.00	0.00
87	->	96	0.00	0.00
87	->	97	0.00	0.00
87	->	98	0.00	0.00
87	->	99	0.01	0.00
87	->	100	0.01	0.00
87	->	101	0.01	0.00
87	->	102	0.01	0.00
87	->	103	0.01	0.00
87	->	104	-0.01	0.01
87	->	105	-0.15	0.07
87	->	106	-1.19	0.64
87	->	107	-10.77	0.76
88	->	1	-0.01	0.00
88	->	2	-0.00	0.00
88	->	3	0.00	0.00
88	->	4	-0.00	0.00
88	->	5	0.01	0.00
88	->	6	-0.00	0.00
88	->	7	0.00	0.00
88	->	8	0.00	0.00
88	->	9	-0.00	0.00
88	->	10	0.01	0.00
88	->	11	0.00	0.00
88	->	12	-0.01	0.00
88	->	13	-0.00	0.00
88	->	14	-0.00	0.00
88	->	15	-0.00	0.00
88	->	16	-0.01	0.00
88	->	17	0.00	0.00
88	->	18	0.00	0.00
88	->	19	0.01	0.00
88	->	20	0.00	0.00
88	->	21	0.00	0.00
88	->	22	-0.02	0.00
88	->	23	-0.00	0.00
88	->	24	0.00	0.00
88	->	25	-0.02	0.00
88	->	26	-0.00	0.00
88	->	27	-0.00	0.00
88	->	28	-0.00	0.00
88	->	29	-0.00	0.00
88	->	30	0.00	0.00
88	->	31	-0.05	0.02
88	->	32	-0.00	0.00
88	->	33	-0.00	0.00
88	->	34	-0.00	0.01
88	->	35	0.06	0.02
88	->	36	-0.02	0.01
88	->	37	-0.01	0.01

88	->	38	0.09	0.04
88	->	39	-0.01	0.01
88	->	40	-0.53	0.54
88	->	41	-0.01	0.01
88	->	42	-0.01	0.03
88	->	43	-0.00	0.00
88	->	44	-0.03	0.01
88	->	45	0.00	0.00
88	->	46	0.02	0.01
88	->	47	-0.05	0.03
88	->	48	0.00	0.01
88	->	49	-0.02	0.01
88	->	50	-0.09	0.09
88	->	51	-0.39	0.32
88	->	52	0.01	0.02
88	->	53	-0.23	0.17
88	->	54	-1.30	1.36
88	->	55	0.03	0.03
88	->	56	-0.02	0.01
88	->	57	-0.05	0.11
88	->	58	0.03	0.02
88	->	59	-1.93	2.93
88	->	60	-0.01	0.00
88	->	61	-0.01	0.00
88	->	62	-0.00	0.00
88	->	63	0.02	0.01
88	->	64	0.01	0.00
88	->	65	0.00	0.00
88	->	66	0.02	0.01
88	->	67	-0.00	0.00
88	->	68	-0.34	0.54
88	->	69	-0.07	0.04
88	->	70	-0.12	0.07
88	->	71	-0.15	0.12
88	->	72	-2.18	1.30
88	->	73	-0.01	0.00
88	->	74	-0.51	1.00
88	->	75	-1.97	2.20
88	->	76	-0.03	0.00
88	->	77	-0.01	0.01
88	->	78	0.10	1.09
88	->	79	-0.02	0.01
88	->	80	-0.00	0.00
88	->	81	-0.02	0.00
88	->	82	-0.01	0.00
88	->	83	-0.00	0.00
88	->	84	-0.00	0.00
88	->	85	-0.00	0.00
88	->	86	-0.31	0.07
88	->	87	-35.83	1.02

88	->	88	-208.18	4.93
88	->	89	-33.64	1.18
88	->	90	-0.26	0.08
88	->	91	-0.00	0.01
88	->	92	0.01	0.00
88	->	93	0.01	0.00
88	->	94	0.00	0.00
88	->	95	0.00	0.00
88	->	96	0.00	0.00
88	->	97	0.00	0.00
88	->	98	0.01	0.00
88	->	99	0.01	0.00
88	->	100	0.01	0.00
88	->	101	0.01	0.00
88	->	102	0.00	0.00
88	->	103	-0.01	0.01
88	->	104	-0.11	0.06
88	->	105	-0.67	0.30
88	->	106	-11.45	0.78
88	->	107	0.17	0.13
89	->	1	-0.01	0.00
89	->	2	-0.00	0.00
89	->	3	0.00	0.00
89	->	4	-0.00	0.00
89	->	5	0.01	0.00
89	->	6	-0.00	0.00
89	->	7	-0.00	0.00
89	->	8	0.00	0.00
89	->	9	-0.00	0.00
89	->	10	0.01	0.00
89	->	11	0.00	0.00
89	->	12	-0.01	0.01
89	->	13	-0.00	0.00
89	->	14	-0.00	0.00
89	->	15	-0.00	0.00
89	->	16	-0.01	0.00
89	->	17	0.00	0.00
89	->	18	0.00	0.00
89	->	19	0.01	0.00
89	->	20	-0.00	0.00
89	->	21	-0.00	0.00
89	->	22	-0.02	0.00
89	->	23	-0.00	0.00
89	->	24	-0.00	0.00
89	->	25	-0.03	0.01
89	->	26	-0.01	0.01
89	->	27	-0.00	0.00
89	->	28	-0.00	0.00
89	->	29	-0.00	0.00
89	->	30	-0.00	0.00

89 ->	31	-0.24	0.24
89 ->	32	-0.00	0.00
89 ->	33	-0.00	0.00
89 ->	34	-0.01	0.01
89 ->	35	0.08	0.03
89 ->	36	-0.03	0.01
89 ->	37	-0.01	0.01
89 ->	38	0.13	0.09
89 ->	39	-0.01	0.01
89 ->	40	-4.20	3.49
89 ->	41	-0.04	0.03
89 ->	42	-0.32	0.26
89 ->	43	-0.01	0.01
89 ->	44	-0.05	0.02
89 ->	45	0.00	0.00
89 ->	46	0.03	0.01
89 ->	47	-0.19	0.08
89 ->	48	0.00	0.01
89 ->	49	-0.02	0.02
89 ->	50	-0.10	0.09
89 ->	51	-1.46	2.14
89 ->	52	0.02	0.03
89 ->	53	-0.09	0.08
89 ->	54	-0.32	0.77
89 ->	55	0.00	0.04
89 ->	56	-0.01	0.00
89 ->	57	-0.01	0.00
89 ->	58	0.02	0.01
89 ->	59	-0.07	0.03
89 ->	60	-0.00	0.00
89 ->	61	-0.00	0.00
89 ->	62	-0.00	0.00
89 ->	63	0.01	0.00
89 ->	64	0.01	0.00
89 ->	65	0.00	0.00
89 ->	66	0.02	0.00
89 ->	67	0.00	0.00
89 ->	68	-0.05	0.02
89 ->	69	-0.02	0.01
89 ->	70	-0.02	0.01
89 ->	71	-0.03	0.02
89 ->	72	-8.39	1.28
89 ->	73	-0.01	0.00
89 ->	74	0.42	0.16
89 ->	75	0.08	0.12
89 ->	76	-0.02	0.00
89 ->	77	-0.03	0.00
89 ->	78	-0.14	0.14
89 ->	79	-0.01	0.00
89 ->	80	-0.00	0.00

89	->	81	-0.02	0.00
89	->	82	-0.00	0.00
89	->	83	-0.00	0.00
89	->	84	-0.00	0.00
89	->	85	-0.00	0.00
89	->	86	-0.03	0.01
89	->	87	-0.30	0.07
89	->	88	-33.40	1.16
89	->	89	-170.04	4.75
89	->	90	-35.43	1.09
89	->	91	-0.33	0.09
89	->	92	-0.01	0.01
89	->	93	0.01	0.00
89	->	94	0.01	0.00
89	->	95	0.01	0.00
89	->	96	0.00	0.00
89	->	97	0.00	0.00
89	->	98	0.01	0.00
89	->	99	0.02	0.00
89	->	100	0.02	0.00
89	->	101	0.01	0.00
89	->	102	-0.00	0.01
89	->	103	-0.08	0.04
89	->	104	-1.78	0.60
89	->	105	-11.75	0.71
89	->	106	-4.51	0.90
89	->	107	-0.26	0.08
90	->	1	-0.02	0.01
90	->	2	0.00	0.00
90	->	3	-0.00	0.00
90	->	4	-0.00	0.00
90	->	5	0.02	0.00
90	->	6	-0.00	0.00
90	->	7	-0.00	0.00
90	->	8	0.01	0.00
90	->	9	-0.00	0.00
90	->	10	0.03	0.01
90	->	11	0.01	0.00
90	->	12	-0.04	0.04
90	->	13	-0.01	0.00
90	->	14	-0.00	0.00
90	->	15	-0.00	0.00
90	->	16	-0.02	0.00
90	->	17	0.00	0.00
90	->	18	0.00	0.00
90	->	19	0.02	0.00
90	->	20	0.00	0.00
90	->	21	-0.00	0.00
90	->	22	-0.11	0.03
90	->	23	-0.02	0.01

90	->	24	-0.00	0.00
90	->	25	-0.09	0.02
90	->	26	-0.18	0.12
90	->	27	0.00	0.01
90	->	28	-0.01	0.00
90	->	29	-0.01	0.01
90	->	30	-0.00	0.00
90	->	31	-1.28	1.60
90	->	32	-0.01	0.00
90	->	33	-0.01	0.00
90	->	34	-0.01	0.00
90	->	35	0.04	0.01
90	->	36	-0.02	0.00
90	->	37	-0.00	0.00
90	->	38	0.04	0.01
90	->	39	-0.00	0.00
90	->	40	-1.96	2.42
90	->	41	-0.05	0.04
90	->	42	-0.26	0.21
90	->	43	-0.03	0.05
90	->	44	-0.12	0.07
90	->	45	-0.00	0.00
90	->	46	-0.01	0.04
90	->	47	-1.48	0.59
90	->	48	-0.01	0.01
90	->	49	-0.01	0.00
90	->	50	-0.11	0.21
90	->	51	-0.04	0.03
90	->	52	-0.00	0.00
90	->	53	-0.03	0.01
90	->	54	-0.01	0.01
90	->	55	-0.00	0.00
90	->	56	-0.00	0.00
90	->	57	-0.00	0.00
90	->	58	0.01	0.00
90	->	59	-0.02	0.01
90	->	60	-0.00	0.00
90	->	61	-0.00	0.00
90	->	62	0.00	0.00
90	->	63	0.01	0.00
90	->	64	0.00	0.00
90	->	65	0.00	0.00
90	->	66	0.01	0.00
90	->	67	0.00	0.00
90	->	68	-0.02	0.01
90	->	69	-0.01	0.00
90	->	70	-0.01	0.00
90	->	71	-0.01	0.01
90	->	72	-2.05	0.97
90	->	73	-0.01	0.00

90	->	74	0.11	0.09
90	->	75	0.01	0.01
90	->	76	-0.01	0.00
90	->	77	-0.02	0.00
90	->	78	-0.04	0.01
90	->	79	-0.00	0.00
90	->	80	-0.00	0.00
90	->	81	-0.02	0.00
90	->	82	-0.00	0.00
90	->	83	-0.00	0.00
90	->	84	-0.00	0.00
90	->	85	-0.00	0.00
90	->	86	-0.00	0.00
90	->	87	-0.02	0.01
90	->	88	-0.26	0.08
90	->	89	-35.20	1.07
90	->	90	-92.61	3.43
90	->	91	-33.90	0.77
90	->	92	-0.16	0.06
90	->	93	0.00	0.01
90	->	94	0.01	0.00
90	->	95	0.01	0.00
90	->	96	0.00	0.00
90	->	97	0.00	0.00
90	->	98	0.01	0.00
90	->	99	0.02	0.00
90	->	100	0.01	0.00
90	->	101	-0.00	0.01
90	->	102	-0.04	0.02
90	->	103	-0.52	0.21
90	->	104	-6.11	0.54
90	->	105	-0.71	0.39
90	->	106	-0.26	0.10
90	->	107	-0.03	0.02
91	->	1	-0.08	0.05
91	->	2	-0.00	0.00
91	->	3	-0.00	0.00
91	->	4	-0.01	0.00
91	->	5	0.09	0.04
91	->	6	-0.00	0.00
91	->	7	-0.02	0.01
91	->	8	0.02	0.00
91	->	9	-0.01	0.01
91	->	10	0.14	0.07
91	->	11	0.02	0.01
91	->	12	-0.89	1.74
91	->	13	-0.03	0.01
91	->	14	0.02	0.03
91	->	15	0.00	0.00
91	->	16	-0.05	0.03

91	->	17	-0.00	0.00
91	->	18	-0.03	0.01
91	->	19	0.21	0.17
91	->	20	0.02	0.02
91	->	21	-0.06	0.03
91	->	22	-4.71	1.67
91	->	23	-1.03	0.55
91	->	24	0.02	0.02
91	->	25	-0.32	0.08
91	->	26	-2.91	0.83
91	->	27	0.07	0.05
91	->	28	-0.02	0.01
91	->	29	-0.03	0.02
91	->	30	-0.01	0.01
91	->	31	-0.32	0.23
91	->	32	-0.01	0.00
91	->	33	-0.01	0.00
91	->	34	-0.00	0.00
91	->	35	0.02	0.00
91	->	36	-0.01	0.00
91	->	37	-0.00	0.00
91	->	38	0.02	0.00
91	->	39	-0.00	0.00
91	->	40	-0.07	0.03
91	->	41	-0.02	0.01
91	->	42	-0.09	0.03
91	->	43	-0.14	0.10
91	->	44	-1.89	1.37
91	->	45	0.00	0.00
91	->	46	0.12	0.11
91	->	47	-2.97	0.61
91	->	48	0.00	0.00
91	->	49	-0.01	0.00
91	->	50	-0.02	0.01
91	->	51	-0.01	0.00
91	->	52	-0.00	0.00
91	->	53	-0.02	0.01
91	->	54	-0.00	0.00
91	->	55	-0.00	0.00
91	->	56	-0.00	0.00
91	->	57	-0.00	0.00
91	->	58	0.01	0.00
91	->	59	-0.02	0.00
91	->	60	-0.00	0.00
91	->	61	-0.00	0.00
91	->	62	0.00	0.00
91	->	63	0.01	0.00
91	->	64	0.00	0.00
91	->	65	0.00	0.00
91	->	66	0.01	0.00

91 ->	67	0.00	0.00
91 ->	68	-0.01	0.00
91 ->	69	-0.01	0.00
91 ->	70	-0.00	0.00
91 ->	71	-0.00	0.00
91 ->	72	-0.14	0.15
91 ->	73	-0.00	0.01
91 ->	74	-0.08	0.09
91 ->	75	0.01	0.01
91 ->	76	-0.01	0.00
91 ->	77	-0.00	0.02
91 ->	78	-0.03	0.01
91 ->	79	-0.00	0.00
91 ->	80	-0.00	0.00
91 ->	81	-0.01	0.00
91 ->	82	-0.00	0.00
91 ->	83	-0.00	0.00
91 ->	84	0.00	0.00
91 ->	85	-0.00	0.00
91 ->	86	0.00	0.00
91 ->	87	0.01	0.00
91 ->	88	-0.00	0.01
91 ->	89	-0.33	0.09
91 ->	90	-33.65	0.77
91 ->	91	-171.37	2.85
91 ->	92	-33.25	1.66
91 ->	93	-0.10	0.14
91 ->	94	-0.04	0.01
91 ->	95	0.00	0.00
91 ->	96	0.00	0.00
91 ->	97	0.00	0.00
91 ->	98	0.02	0.01
91 ->	99	0.02	0.01
91 ->	100	-0.00	0.01
91 ->	101	-0.10	0.05
91 ->	102	-1.11	0.53
91 ->	103	-11.39	0.81
91 ->	104	-3.48	0.72
91 ->	105	-0.22	0.10
91 ->	106	-0.03	0.02
91 ->	107	0.01	0.00
92 ->	1	-1.21	1.25
92 ->	2	-0.00	0.00
92 ->	3	-0.00	0.01
92 ->	4	-0.01	0.01
92 ->	5	0.22	0.11
92 ->	6	-0.01	0.01
92 ->	7	-0.03	0.03
92 ->	8	0.02	0.01
92 ->	9	-0.01	0.01

92	->	10	0.30	0.17
92	->	11	0.03	0.02
92	->	12	-3.52	4.79
92	->	13	-0.11	0.08
92	->	14	-0.43	0.45
92	->	15	0.01	0.02
92	->	16	-0.27	0.23
92	->	17	-0.01	0.01
92	->	18	-0.10	0.05
92	->	19	0.32	0.44
92	->	20	0.10	0.09
92	->	21	-0.12	0.05
92	->	22	-16.15	4.18
92	->	23	-6.18	3.16
92	->	24	0.11	0.07
92	->	25	-0.28	0.13
92	->	26	-1.06	0.44
92	->	27	0.20	0.14
92	->	28	-0.02	0.01
92	->	29	-0.01	0.00
92	->	30	-0.00	0.00
92	->	31	-0.06	0.01
92	->	32	-0.01	0.00
92	->	33	-0.00	0.00
92	->	34	-0.00	0.00
92	->	35	0.01	0.00
92	->	36	-0.01	0.00
92	->	37	-0.00	0.00
92	->	38	0.01	0.00
92	->	39	0.00	0.00
92	->	40	-0.03	0.01
92	->	41	-0.00	0.00
92	->	42	-0.02	0.01
92	->	43	-0.05	0.02
92	->	44	-6.53	1.54
92	->	45	0.00	0.01
92	->	46	0.37	0.25
92	->	47	-0.41	0.44
92	->	48	-0.00	0.00
92	->	49	-0.01	0.00
92	->	50	-0.01	0.00
92	->	51	-0.01	0.00
92	->	52	-0.00	0.00
92	->	53	-0.02	0.00
92	->	54	-0.00	0.00
92	->	55	-0.00	0.00
92	->	56	-0.00	0.00
92	->	57	-0.00	0.00
92	->	58	0.01	0.00
92	->	59	-0.01	0.00

92	->	60	-0.00	0.00
92	->	61	-0.00	0.00
92	->	62	0.00	0.00
92	->	63	0.00	0.00
92	->	64	0.00	0.00
92	->	65	0.00	0.00
92	->	66	0.00	0.00
92	->	67	0.00	0.00
92	->	68	-0.01	0.00
92	->	69	-0.00	0.00
92	->	70	-0.00	0.00
92	->	71	0.00	0.00
92	->	72	-0.02	0.03
92	->	73	-0.01	0.01
92	->	74	-0.03	0.03
92	->	75	0.01	0.00
92	->	76	-0.01	0.00
92	->	77	0.00	0.02
92	->	78	-0.02	0.00
92	->	79	-0.00	0.00
92	->	80	-0.00	0.00
92	->	81	-0.01	0.00
92	->	82	-0.00	0.00
92	->	83	-0.00	0.00
92	->	84	-0.00	0.00
92	->	85	-0.00	0.00
92	->	86	0.00	0.00
92	->	87	0.01	0.00
92	->	88	0.01	0.00
92	->	89	-0.01	0.01
92	->	90	-0.16	0.06
92	->	91	-33.12	1.63
92	->	92	-159.36	7.35
92	->	93	-32.62	2.65
92	->	94	-0.43	0.10
92	->	95	-0.01	0.01
92	->	96	0.00	0.00
92	->	97	0.00	0.00
92	->	98	0.02	0.01
92	->	99	0.01	0.01
92	->	100	-0.04	0.06
92	->	101	-1.03	0.34
92	->	102	-11.63	0.80
92	->	103	-3.52	0.89
92	->	104	-0.36	0.11
92	->	105	-0.03	0.02
92	->	106	0.01	0.00
92	->	107	0.01	0.00
93	->	1	-7.18	7.62
93	->	2	-0.01	0.00

93	->	3	0.00	0.01
93	->	4	-0.00	0.00
93	->	5	0.06	0.03
93	->	6	-0.00	0.00
93	->	7	-0.00	0.00
93	->	8	0.02	0.01
93	->	9	0.00	0.00
93	->	10	0.06	0.01
93	->	11	0.03	0.01
93	->	12	-1.83	2.93
93	->	13	-0.11	0.13
93	->	14	-0.65	0.65
93	->	15	0.01	0.09
93	->	16	-3.02	1.28
93	->	17	-0.02	0.01
93	->	18	-0.25	0.16
93	->	19	-0.26	1.31
93	->	20	0.06	0.09
93	->	21	-0.08	0.04
93	->	22	-8.46	6.50
93	->	23	-0.15	0.16
93	->	24	0.00	0.01
93	->	25	-0.07	0.02
93	->	26	-0.03	0.01
93	->	27	0.01	0.01
93	->	28	-0.00	0.00
93	->	29	-0.00	0.00
93	->	30	0.00	0.00
93	->	31	-0.03	0.00
93	->	32	-0.00	0.00
93	->	33	-0.00	0.00
93	->	34	-0.00	0.00
93	->	35	0.01	0.00
93	->	36	-0.00	0.00
93	->	37	-0.00	0.00
93	->	38	0.01	0.00
93	->	39	0.00	0.00
93	->	40	-0.01	0.00
93	->	41	-0.00	0.00
93	->	42	-0.01	0.00
93	->	43	-0.02	0.01
93	->	44	-4.23	1.94
93	->	45	-0.00	0.00
93	->	46	0.13	0.15
93	->	47	-0.05	0.03
93	->	48	-0.00	0.00
93	->	49	-0.00	0.00
93	->	50	-0.01	0.00
93	->	51	-0.00	0.00
93	->	52	-0.00	0.00

93	->	53	-0.01	0.00
93	->	54	-0.00	0.00
93	->	55	0.00	0.00
93	->	56	-0.00	0.00
93	->	57	-0.00	0.00
93	->	58	0.00	0.00
93	->	59	-0.01	0.00
93	->	60	-0.00	0.00
93	->	61	-0.00	0.00
93	->	62	0.00	0.00
93	->	63	0.00	0.00
93	->	64	0.00	0.00
93	->	65	0.00	0.00
93	->	66	0.00	0.00
93	->	67	-0.00	0.00
93	->	68	-0.01	0.00
93	->	69	-0.00	0.00
93	->	70	-0.00	0.00
93	->	71	0.00	0.00
93	->	72	-0.02	0.01
93	->	73	-0.01	0.01
93	->	74	0.00	0.01
93	->	75	0.01	0.00
93	->	76	-0.01	0.00
93	->	77	-0.02	0.01
93	->	78	-0.02	0.00
93	->	79	-0.00	0.00
93	->	80	-0.00	0.00
93	->	81	-0.02	0.01
93	->	82	-0.00	0.00
93	->	83	-0.00	0.00
93	->	84	-0.00	0.00
93	->	85	-0.00	0.00
93	->	86	0.00	0.00
93	->	87	0.01	0.00
93	->	88	0.01	0.00
93	->	89	0.01	0.00
93	->	90	0.00	0.01
93	->	91	-0.10	0.14
93	->	92	-32.46	2.60
93	->	93	-166.00	9.79
93	->	94	-35.11	0.96
93	->	95	-0.33	0.07
93	->	96	-0.03	0.01
93	->	97	-0.00	0.00
93	->	98	0.01	0.01
93	->	99	-0.05	0.02
93	->	100	-1.10	0.57
93	->	101	-11.46	0.73
93	->	102	-3.59	0.72

93	->	103	-0.36	0.08
93	->	104	-0.04	0.02
93	->	105	0.01	0.00
93	->	106	0.02	0.00
93	->	107	0.01	0.00
94	->	1	-0.79	1.01
94	->	2	-0.01	0.00
94	->	3	-0.01	0.00
94	->	4	-0.00	0.00
94	->	5	0.01	0.00
94	->	6	-0.00	0.00
94	->	7	-0.00	0.00
94	->	8	0.01	0.00
94	->	9	0.00	0.00
94	->	10	0.02	0.00
94	->	11	0.01	0.00
94	->	12	-0.05	0.03
94	->	13	-0.04	0.02
94	->	14	-0.10	0.05
94	->	15	-0.01	0.15
94	->	16	-11.71	2.06
94	->	17	-0.07	0.03
94	->	18	-2.57	1.26
94	->	19	-2.75	1.18
94	->	20	-0.07	0.03
94	->	21	0.04	0.05
94	->	22	-0.44	0.48
94	->	23	-0.01	0.01
94	->	24	-0.01	0.00
94	->	25	-0.01	0.01
94	->	26	-0.01	0.00
94	->	27	-0.00	0.00
94	->	28	-0.00	0.00
94	->	29	-0.00	0.00
94	->	30	-0.00	0.00
94	->	31	-0.01	0.00
94	->	32	-0.00	0.00
94	->	33	-0.00	0.00
94	->	34	0.00	0.00
94	->	35	0.00	0.00
94	->	36	-0.00	0.00
94	->	37	-0.00	0.00
94	->	38	0.00	0.00
94	->	39	0.00	0.00
94	->	40	-0.01	0.00
94	->	41	-0.00	0.00
94	->	42	-0.00	0.00
94	->	43	-0.00	0.00
94	->	44	-0.13	0.23
94	->	45	-0.01	0.00

94	->	46	0.02	0.02
94	->	47	-0.01	0.00
94	->	48	-0.00	0.00
94	->	49	-0.00	0.00
94	->	50	-0.00	0.00
94	->	51	-0.00	0.00
94	->	52	-0.00	0.00
94	->	53	-0.01	0.00
94	->	54	-0.00	0.00
94	->	55	-0.00	0.00
94	->	56	-0.00	0.00
94	->	57	-0.00	0.00
94	->	58	0.01	0.00
94	->	59	-0.01	0.00
94	->	60	-0.00	0.00
94	->	61	-0.00	0.00
94	->	62	0.00	0.00
94	->	63	0.01	0.00
94	->	64	0.01	0.00
94	->	65	0.00	0.00
94	->	66	0.00	0.00
94	->	67	-0.00	0.00
94	->	68	-0.01	0.00
94	->	69	-0.00	0.00
94	->	70	-0.00	0.00
94	->	71	0.00	0.00
94	->	72	-0.02	0.00
94	->	73	-0.00	0.00
94	->	74	0.02	0.00
94	->	75	0.01	0.00
94	->	76	-0.01	0.00
94	->	77	-0.04	0.01
94	->	78	-0.01	0.00
94	->	79	-0.00	0.00
94	->	80	-0.00	0.00
94	->	81	-0.02	0.01
94	->	82	-0.00	0.00
94	->	83	-0.00	0.00
94	->	84	-0.00	0.00
94	->	85	-0.00	0.00
94	->	86	0.00	0.00
94	->	87	0.01	0.00
94	->	88	0.00	0.00
94	->	89	0.01	0.00
94	->	90	0.01	0.00
94	->	91	-0.04	0.01
94	->	92	-0.43	0.10
94	->	93	-34.97	0.96
94	->	94	-204.19	3.81
94	->	95	-33.79	1.11

94	->	96	-0.13	0.07
94	->	97	-0.02	0.01
94	->	98	-0.25	0.07
94	->	99	-0.92	0.50
94	->	100	-11.25	1.12
94	->	101	0.29	0.22
94	->	102	0.01	0.06
94	->	103	0.00	0.02
94	->	104	0.02	0.00
94	->	105	0.02	0.00
94	->	106	0.01	0.00
94	->	107	0.00	0.00
95	->	1	-0.07	0.01
95	->	2	-0.00	0.00
95	->	3	-0.01	0.00
95	->	4	-0.00	0.00
95	->	5	0.01	0.00
95	->	6	-0.00	0.00
95	->	7	-0.00	0.00
95	->	8	0.00	0.00
95	->	9	0.00	0.00
95	->	10	0.01	0.00
95	->	11	0.01	0.00
95	->	12	-0.02	0.01
95	->	13	-0.01	0.00
95	->	14	-0.01	0.00
95	->	15	-0.01	0.01
95	->	16	-0.54	0.27
95	->	17	-0.01	0.01
95	->	18	-0.35	0.30
95	->	19	-0.15	0.07
95	->	20	-0.01	0.00
95	->	21	-0.00	0.01
95	->	22	-0.03	0.02
95	->	23	-0.01	0.00
95	->	24	-0.00	0.00
95	->	25	-0.02	0.00
95	->	26	-0.00	0.00
95	->	27	-0.00	0.00
95	->	28	-0.00	0.00
95	->	29	-0.00	0.00
95	->	30	0.00	0.00
95	->	31	-0.01	0.00
95	->	32	-0.00	0.00
95	->	33	-0.00	0.00
95	->	34	0.00	0.00
95	->	35	0.00	0.00
95	->	36	-0.00	0.00
95	->	37	-0.00	0.00
95	->	38	0.00	0.00

95	->	39	0.00	0.00
95	->	40	-0.00	0.00
95	->	41	-0.00	0.00
95	->	42	-0.00	0.00
95	->	43	-0.00	0.00
95	->	44	-0.04	0.02
95	->	45	-0.00	0.00
95	->	46	0.02	0.01
95	->	47	-0.00	0.00
95	->	48	-0.00	0.00
95	->	49	-0.00	0.00
95	->	50	-0.00	0.00
95	->	51	-0.00	0.00
95	->	52	-0.00	0.00
95	->	53	-0.02	0.00
95	->	54	-0.00	0.00
95	->	55	-0.00	0.00
95	->	56	-0.00	0.00
95	->	57	-0.00	0.00
95	->	58	0.01	0.00
95	->	59	-0.01	0.00
95	->	60	-0.00	0.00
95	->	61	-0.00	0.00
95	->	62	0.00	0.00
95	->	63	0.01	0.00
95	->	64	0.01	0.00
95	->	65	0.00	0.00
95	->	66	0.00	0.00
95	->	67	-0.00	0.00
95	->	68	-0.00	0.00
95	->	69	-0.00	0.00
95	->	70	-0.00	0.00
95	->	71	0.00	0.00
95	->	72	-0.02	0.00
95	->	73	-0.00	0.00
95	->	74	0.02	0.00
95	->	75	0.01	0.00
95	->	76	-0.01	0.00
95	->	77	-0.03	0.01
95	->	78	-0.01	0.00
95	->	79	-0.00	0.00
95	->	80	-0.00	0.00
95	->	81	-0.01	0.01
95	->	82	-0.00	0.00
95	->	83	-0.00	0.00
95	->	84	-0.00	0.00
95	->	85	-0.00	0.00
95	->	86	0.00	0.00
95	->	87	0.00	0.00
95	->	88	0.00	0.00

95	->	89	0.01	0.00
95	->	90	0.01	0.00
95	->	91	0.00	0.00
95	->	92	-0.01	0.01
95	->	93	-0.33	0.07
95	->	94	-33.50	1.10
95	->	95	-134.62	2.46
95	->	96	-35.11	0.76
95	->	97	-0.19	0.07
95	->	98	-2.69	0.53
95	->	99	-6.17	0.54
95	->	100	-2.51	0.63
95	->	101	-0.17	0.06
95	->	102	-0.04	0.02
95	->	103	0.01	0.00
95	->	104	0.02	0.00
95	->	105	0.01	0.00
95	->	106	0.01	0.00
95	->	107	0.00	0.00
96	->	1	-0.02	0.00
96	->	2	-0.00	0.00
96	->	3	-0.01	0.01
96	->	4	-0.00	0.00
96	->	5	0.00	0.00
96	->	6	-0.00	0.00
96	->	7	0.00	0.00
96	->	8	0.00	0.00
96	->	9	0.00	0.00
96	->	10	0.00	0.00
96	->	11	0.00	0.00
96	->	12	-0.01	0.00
96	->	13	-0.00	0.00
96	->	14	-0.00	0.00
96	->	15	-0.01	0.00
96	->	16	-0.12	0.06
96	->	17	-0.05	0.05
96	->	18	-0.12	0.06
96	->	19	0.01	0.01
96	->	20	-0.01	0.00
96	->	21	-0.01	0.00
96	->	22	-0.02	0.00
96	->	23	-0.00	0.00
96	->	24	-0.00	0.00
96	->	25	-0.02	0.00
96	->	26	-0.00	0.00
96	->	27	-0.00	0.00
96	->	28	-0.00	0.00
96	->	29	-0.00	0.00
96	->	30	0.00	0.00
96	->	31	-0.01	0.00

96	->	32	-0.00	0.00
96	->	33	-0.00	0.00
96	->	34	0.00	0.00
96	->	35	0.00	0.00
96	->	36	-0.00	0.00
96	->	37	-0.00	0.00
96	->	38	0.00	0.00
96	->	39	0.00	0.00
96	->	40	-0.00	0.00
96	->	41	-0.00	0.00
96	->	42	-0.00	0.00
96	->	43	-0.00	0.00
96	->	44	-0.03	0.01
96	->	45	-0.00	0.00
96	->	46	0.02	0.00
96	->	47	-0.00	0.00
96	->	48	-0.00	0.00
96	->	49	-0.00	0.00
96	->	50	-0.00	0.00
96	->	51	-0.00	0.00
96	->	52	-0.00	0.00
96	->	53	-0.01	0.00
96	->	54	-0.00	0.00
96	->	55	-0.00	0.00
96	->	56	-0.00	0.00
96	->	57	-0.00	0.00
96	->	58	0.01	0.00
96	->	59	-0.00	0.00
96	->	60	-0.00	0.00
96	->	61	-0.00	0.00
96	->	62	0.00	0.00
96	->	63	0.00	0.00
96	->	64	0.00	0.00
96	->	65	0.00	0.00
96	->	66	0.00	0.00
96	->	67	0.00	0.00
96	->	68	-0.00	0.00
96	->	69	-0.00	0.00
96	->	70	0.00	0.00
96	->	71	0.00	0.00
96	->	72	-0.01	0.00
96	->	73	-0.00	0.00
96	->	74	0.01	0.00
96	->	75	0.00	0.00
96	->	76	-0.01	0.00
96	->	77	-0.02	0.00
96	->	78	-0.00	0.00
96	->	79	-0.00	0.00
96	->	80	-0.00	0.00
96	->	81	-0.01	0.00

96	->	82	-0.00	0.00
96	->	83	-0.00	0.00
96	->	84	-0.00	0.00
96	->	85	-0.00	0.00
96	->	86	0.00	0.00
96	->	87	0.00	0.00
96	->	88	0.00	0.00
96	->	89	0.00	0.00
96	->	90	0.00	0.00
96	->	91	0.00	0.00
96	->	92	0.00	0.00
96	->	93	-0.03	0.01
96	->	94	-0.13	0.07
96	->	95	-34.88	0.76
96	->	96	-215.99	2.94
96	->	97	-2.86	1.66
96	->	98	-10.70	0.71
96	->	99	-0.38	0.12
96	->	100	-0.15	0.04
96	->	101	-0.01	0.02
96	->	102	0.01	0.01
96	->	103	0.01	0.00
96	->	104	0.01	0.00
96	->	105	0.00	0.00
96	->	106	0.00	0.00
96	->	107	0.00	0.00
97	->	1	-0.01	0.01
97	->	2	-0.00	0.00
97	->	3	-0.06	0.17
97	->	4	-0.00	0.00
97	->	5	0.00	0.00
97	->	6	-0.00	0.00
97	->	7	-0.00	0.00
97	->	8	0.00	0.00
97	->	9	-0.00	0.00
97	->	10	0.00	0.00
97	->	11	0.00	0.00
97	->	12	-0.00	0.00
97	->	13	-0.00	0.00
97	->	14	-0.01	0.00
97	->	15	-0.01	0.00
97	->	16	-0.08	0.05
97	->	17	-0.87	0.84
97	->	18	-0.08	0.06
97	->	19	-0.00	0.01
97	->	20	-0.03	0.03
97	->	21	-0.09	0.07
97	->	22	-0.02	0.01
97	->	23	-0.00	0.00
97	->	24	-0.01	0.01

97	->	25	-0.05	0.02
97	->	26	-0.00	0.00
97	->	27	-0.00	0.00
97	->	28	-0.00	0.00
97	->	29	-0.00	0.00
97	->	30	-0.00	0.00
97	->	31	-0.01	0.00
97	->	32	-0.00	0.00
97	->	33	-0.01	0.00
97	->	34	-0.00	0.00
97	->	35	0.00	0.00
97	->	36	-0.00	0.00
97	->	37	-0.00	0.00
97	->	38	0.00	0.00
97	->	39	0.00	0.00
97	->	40	-0.00	0.00
97	->	41	-0.00	0.00
97	->	42	-0.00	0.00
97	->	43	-0.00	0.00
97	->	44	-0.02	0.00
97	->	45	-0.00	0.00
97	->	46	0.01	0.00
97	->	47	-0.00	0.00
97	->	48	-0.00	0.00
97	->	49	-0.00	0.00
97	->	50	-0.00	0.00
97	->	51	-0.00	0.00
97	->	52	-0.00	0.00
97	->	53	-0.00	0.00
97	->	54	-0.00	0.00
97	->	55	-0.00	0.00
97	->	56	-0.00	0.00
97	->	57	-0.00	0.00
97	->	58	0.00	0.00
97	->	59	-0.00	0.00
97	->	60	-0.00	0.00
97	->	61	-0.00	0.00
97	->	62	0.00	0.00
97	->	63	0.00	0.00
97	->	64	0.00	0.00
97	->	65	0.00	0.00
97	->	66	0.00	0.00
97	->	67	0.00	0.00
97	->	68	-0.00	0.00
97	->	69	-0.00	0.00
97	->	70	-0.00	0.00
97	->	71	0.00	0.00
97	->	72	-0.00	0.00
97	->	73	-0.00	0.00
97	->	74	0.00	0.00

97	->	75	0.00	0.00
97	->	76	-0.00	0.00
97	->	77	-0.00	0.00
97	->	78	-0.00	0.00
97	->	79	-0.00	0.00
97	->	80	-0.00	0.00
97	->	81	-0.00	0.00
97	->	82	-0.00	0.00
97	->	83	-0.00	0.00
97	->	84	-0.00	0.00
97	->	85	-0.00	0.00
97	->	86	0.00	0.00
97	->	87	0.00	0.00
97	->	88	0.00	0.00
97	->	89	0.00	0.00
97	->	90	0.00	0.00
97	->	91	0.00	0.00
97	->	92	0.00	0.00
97	->	93	-0.00	0.00
97	->	94	-0.02	0.01
97	->	95	-0.19	0.07
97	->	96	-2.78	1.63
97	->	97	-49.91	2.32
97	->	98	-35.42	1.03
97	->	99	-0.24	0.06
97	->	100	-0.03	0.01
97	->	101	-0.00	0.00
97	->	102	0.00	0.00
97	->	103	0.00	0.00
97	->	104	0.00	0.00
97	->	105	0.00	0.00
97	->	106	0.00	0.00
97	->	107	0.00	0.00
98	->	1	-0.04	0.01
98	->	2	-0.00	0.00
98	->	3	-0.02	0.02
98	->	4	0.00	0.00
98	->	5	0.02	0.01
98	->	6	-0.01	0.00
98	->	7	-0.00	0.00
98	->	8	0.01	0.00
98	->	9	-0.00	0.00
98	->	10	0.02	0.00
98	->	11	0.01	0.00
98	->	12	-0.01	0.00
98	->	13	-0.02	0.01
98	->	14	-0.01	0.00
98	->	15	-0.01	0.01
98	->	16	-0.40	0.17
98	->	17	-2.28	1.23

98	->	18	-1.62	0.23
98	->	19	0.05	0.04
98	->	20	-0.11	0.05
98	->	21	-1.81	0.51
98	->	22	-0.14	0.05
98	->	23	-0.01	0.01
98	->	24	-0.05	0.02
98	->	25	-4.28	1.53
98	->	26	-0.00	0.00
98	->	27	-0.00	0.00
98	->	28	-0.00	0.00
98	->	29	-0.00	0.00
98	->	30	-0.01	0.01
98	->	31	-0.07	0.02
98	->	32	-0.05	0.02
98	->	33	-0.02	0.02
98	->	34	-0.00	0.00
98	->	35	0.01	0.00
98	->	36	-0.01	0.00
98	->	37	-0.00	0.00
98	->	38	0.01	0.00
98	->	39	0.00	0.00
98	->	40	-0.01	0.00
98	->	41	-0.01	0.00
98	->	42	-0.02	0.01
98	->	43	0.05	0.04
98	->	44	-0.46	0.13
98	->	45	0.05	0.05
98	->	46	0.10	0.04
98	->	47	0.01	0.01
98	->	48	-0.01	0.00
98	->	49	-0.01	0.00
98	->	50	-0.00	0.00
98	->	51	-0.00	0.00
98	->	52	-0.00	0.00
98	->	53	-0.01	0.00
98	->	54	-0.00	0.00
98	->	55	0.00	0.00
98	->	56	-0.00	0.00
98	->	57	-0.00	0.00
98	->	58	0.01	0.00
98	->	59	-0.00	0.00
98	->	60	-0.00	0.00
98	->	61	-0.00	0.00
98	->	62	0.00	0.00
98	->	63	0.00	0.00
98	->	64	0.00	0.00
98	->	65	0.00	0.00
98	->	66	0.00	0.00
98	->	67	0.00	0.00

98	->	68	-0.00	0.00
98	->	69	-0.00	0.00
98	->	70	-0.00	0.00
98	->	71	0.00	0.00
98	->	72	-0.01	0.00
98	->	73	-0.00	0.00
98	->	74	0.01	0.00
98	->	75	0.01	0.00
98	->	76	-0.00	0.00
98	->	77	-0.01	0.00
98	->	78	-0.00	0.00
98	->	79	-0.00	0.00
98	->	80	-0.00	0.00
98	->	81	-0.00	0.00
98	->	82	-0.00	0.00
98	->	83	-0.00	0.00
98	->	84	-0.00	0.00
98	->	85	-0.00	0.00
98	->	86	0.00	0.00
98	->	87	0.00	0.00
98	->	88	0.01	0.00
98	->	89	0.01	0.00
98	->	90	0.01	0.00
98	->	91	0.02	0.01
98	->	92	0.02	0.01
98	->	93	0.01	0.01
98	->	94	-0.25	0.07
98	->	95	-2.70	0.53
98	->	96	-10.74	0.70
98	->	97	-35.19	1.02
98	->	98	-175.64	2.81
98	->	99	-34.95	0.69
98	->	100	-0.17	0.09
98	->	101	-0.04	0.01
98	->	102	-0.00	0.00
98	->	103	0.00	0.00
98	->	104	0.01	0.00
98	->	105	0.00	0.00
98	->	106	0.00	0.00
98	->	107	0.00	0.00
99	->	1	-0.03	0.01
99	->	2	-0.00	0.00
99	->	3	-0.00	0.00
99	->	4	0.00	0.00
99	->	5	0.02	0.00
99	->	6	-0.00	0.00
99	->	7	-0.00	0.00
99	->	8	0.01	0.00
99	->	9	-0.00	0.00
99	->	10	0.01	0.00

99	->	11	0.01	0.00
99	->	12	-0.01	0.00
99	->	13	-0.01	0.00
99	->	14	-0.01	0.00
99	->	15	-0.00	0.00
99	->	16	-0.09	0.04
99	->	17	-0.12	0.05
99	->	18	-1.04	0.35
99	->	19	0.14	0.04
99	->	20	-0.03	0.01
99	->	21	-0.75	0.32
99	->	22	-0.32	0.14
99	->	23	-0.01	0.01
99	->	24	-0.02	0.01
99	->	25	-8.49	1.50
99	->	26	-0.01	0.00
99	->	27	-0.00	0.00
99	->	28	-0.00	0.00
99	->	29	-0.00	0.00
99	->	30	-0.01	0.01
99	->	31	-0.11	0.03
99	->	32	-0.18	0.09
99	->	33	-1.47	0.34
99	->	34	-0.00	0.00
99	->	35	0.02	0.00
99	->	36	-0.03	0.01
99	->	37	-0.00	0.00
99	->	38	0.01	0.00
99	->	39	-0.00	0.00
99	->	40	-0.02	0.00
99	->	41	-0.02	0.01
99	->	42	-0.04	0.01
99	->	43	0.13	0.08
99	->	44	-1.68	0.46
99	->	45	-1.60	0.34
99	->	46	0.04	0.06
99	->	47	-0.02	0.02
99	->	48	-0.04	0.01
99	->	49	-0.02	0.01
99	->	50	-0.01	0.00
99	->	51	-0.01	0.00
99	->	52	-0.01	0.00
99	->	53	-0.03	0.01
99	->	54	-0.00	0.00
99	->	55	-0.00	0.00
99	->	56	-0.00	0.00
99	->	57	-0.00	0.00
99	->	58	0.01	0.00
99	->	59	-0.01	0.00
99	->	60	-0.00	0.00

99	->	61	-0.00	0.00
99	->	62	0.00	0.00
99	->	63	0.00	0.00
99	->	64	0.00	0.00
99	->	65	0.00	0.00
99	->	66	0.00	0.00
99	->	67	0.00	0.00
99	->	68	-0.00	0.00
99	->	69	-0.01	0.00
99	->	70	-0.00	0.00
99	->	71	0.00	0.00
99	->	72	-0.02	0.00
99	->	73	-0.00	0.00
99	->	74	0.02	0.00
99	->	75	0.01	0.00
99	->	76	-0.01	0.00
99	->	77	-0.01	0.00
99	->	78	-0.01	0.00
99	->	79	-0.00	0.00
99	->	80	-0.00	0.00
99	->	81	-0.01	0.00
99	->	82	-0.00	0.00
99	->	83	-0.00	0.00
99	->	84	-0.00	0.00
99	->	85	-0.00	0.00
99	->	86	0.00	0.00
99	->	87	0.01	0.00
99	->	88	0.01	0.00
99	->	89	0.02	0.00
99	->	90	0.02	0.00
99	->	91	0.02	0.01
99	->	92	0.01	0.01
99	->	93	-0.05	0.02
99	->	94	-0.91	0.49
99	->	95	-6.18	0.54
99	->	96	-0.38	0.12
99	->	97	-0.24	0.06
99	->	98	-34.75	0.69
99	->	99	-85.31	2.75
99	->	100	-34.86	0.89
99	->	101	-0.21	0.04
99	->	102	-0.01	0.01
99	->	103	0.01	0.00
99	->	104	0.01	0.00
99	->	105	0.01	0.00
99	->	106	0.00	0.00
99	->	107	0.00	0.00
100	->	1	-0.01	0.01
100	->	2	-0.00	0.00
100	->	3	-0.00	0.00

100	->	4	0.00	0.00
100	->	5	0.01	0.00
100	->	6	-0.00	0.00
100	->	7	-0.00	0.00
100	->	8	0.00	0.00
100	->	9	-0.00	0.00
100	->	10	0.01	0.00
100	->	11	0.01	0.00
100	->	12	-0.01	0.00
100	->	13	-0.01	0.00
100	->	14	-0.01	0.00
100	->	15	-0.00	0.00
100	->	16	0.03	0.02
100	->	17	-0.00	0.01
100	->	18	-0.16	0.18
100	->	19	-0.09	0.08
100	->	20	-0.02	0.01
100	->	21	-0.10	0.05
100	->	22	-0.35	0.18
100	->	23	-0.00	0.01
100	->	24	-0.01	0.00
100	->	25	-0.22	0.06
100	->	26	-0.01	0.00
100	->	27	-0.00	0.00
100	->	28	-0.00	0.00
100	->	29	-0.00	0.00
100	->	30	0.00	0.00
100	->	31	-0.04	0.00
100	->	32	-0.01	0.00
100	->	33	-0.11	0.07
100	->	34	-0.00	0.00
100	->	35	0.03	0.00
100	->	36	-0.04	0.02
100	->	37	-0.00	0.00
100	->	38	0.02	0.00
100	->	39	-0.00	0.00
100	->	40	-0.02	0.00
100	->	41	-0.01	0.00
100	->	42	-0.02	0.00
100	->	43	-0.05	0.02
100	->	44	-5.16	2.06
100	->	45	-5.14	0.98
100	->	46	-1.50	0.48
100	->	47	-0.08	0.02
100	->	48	-0.10	0.02
100	->	49	-0.20	0.23
100	->	50	-0.02	0.02
100	->	51	-0.01	0.00
100	->	52	-0.02	0.01
100	->	53	-0.14	0.20

100	->	54	-0.00	0.00
100	->	55	-0.00	0.00
100	->	56	-0.00	0.00
100	->	57	-0.00	0.00
100	->	58	0.03	0.01
100	->	59	-0.02	0.01
100	->	60	-0.01	0.00
100	->	61	-0.00	0.00
100	->	62	0.00	0.00
100	->	63	0.01	0.00
100	->	64	0.01	0.00
100	->	65	0.00	0.00
100	->	66	0.01	0.00
100	->	67	0.00	0.00
100	->	68	-0.01	0.00
100	->	69	-0.01	0.00
100	->	70	-0.00	0.00
100	->	71	-0.00	0.00
100	->	72	-0.05	0.01
100	->	73	-0.01	0.00
100	->	74	0.02	0.00
100	->	75	0.02	0.00
100	->	76	-0.02	0.00
100	->	77	-0.02	0.00
100	->	78	-0.01	0.00
100	->	79	-0.00	0.00
100	->	80	-0.00	0.00
100	->	81	-0.01	0.00
100	->	82	-0.00	0.00
100	->	83	0.00	0.00
100	->	84	-0.00	0.00
100	->	85	-0.00	0.00
100	->	86	0.00	0.00
100	->	87	0.01	0.00
100	->	88	0.01	0.00
100	->	89	0.02	0.00
100	->	90	0.01	0.00
100	->	91	-0.00	0.01
100	->	92	-0.04	0.06
100	->	93	-1.10	0.57
100	->	94	-11.28	1.11
100	->	95	-2.51	0.63
100	->	96	-0.15	0.04
100	->	97	-0.03	0.01
100	->	98	-0.17	0.09
100	->	99	-34.67	0.89
100	->	100	-171.53	2.75
100	->	101	-36.17	0.70
100	->	102	-0.36	0.09
100	->	103	-0.04	0.01

100	->	104	0.00	0.00
100	->	105	0.01	0.00
100	->	106	0.00	0.00
100	->	107	0.00	0.00
101	->	1	-0.02	0.01
101	->	2	-0.00	0.00
101	->	3	-0.00	0.00
101	->	4	0.00	0.00
101	->	5	0.01	0.00
101	->	6	-0.00	0.00
101	->	7	0.00	0.00
101	->	8	0.00	0.00
101	->	9	0.00	0.00
101	->	10	0.01	0.00
101	->	11	0.00	0.00
101	->	12	-0.01	0.00
101	->	13	-0.01	0.00
101	->	14	-0.00	0.00
101	->	15	-0.00	0.00
101	->	16	-0.05	0.01
101	->	17	-0.00	0.00
101	->	18	-0.02	0.01
101	->	19	0.02	0.01
101	->	20	-0.00	0.00
101	->	21	-0.00	0.00
101	->	22	0.01	0.04
101	->	23	-0.01	0.00
101	->	24	-0.00	0.00
101	->	25	-0.02	0.01
101	->	26	-0.00	0.00
101	->	27	-0.00	0.00
101	->	28	-0.00	0.00
101	->	29	-0.00	0.00
101	->	30	0.00	0.00
101	->	31	-0.01	0.00
101	->	32	-0.00	0.00
101	->	33	-0.02	0.00
101	->	34	0.00	0.00
101	->	35	0.01	0.00
101	->	36	-0.02	0.01
101	->	37	-0.00	0.00
101	->	38	0.01	0.00
101	->	39	-0.00	0.00
101	->	40	-0.01	0.00
101	->	41	-0.00	0.00
101	->	42	-0.00	0.00
101	->	43	0.01	0.01
101	->	44	0.71	0.83
101	->	45	-0.09	0.03
101	->	46	-3.90	1.59

101	->	47	-0.20	0.07
101	->	48	-0.02	0.01
101	->	49	-0.13	0.11
101	->	50	-0.04	0.02
101	->	51	-0.01	0.00
101	->	52	-0.01	0.00
101	->	53	-0.37	1.21
101	->	54	-0.00	0.00
101	->	55	-0.00	0.00
101	->	56	-0.00	0.00
101	->	57	-0.00	0.00
101	->	58	0.03	0.01
101	->	59	-0.02	0.01
101	->	60	-0.02	0.01
101	->	61	-0.06	0.07
101	->	62	0.00	0.00
101	->	63	0.01	0.00
101	->	64	0.01	0.00
101	->	65	0.00	0.00
101	->	66	0.01	0.00
101	->	67	0.00	0.00
101	->	68	-0.01	0.00
101	->	69	-0.02	0.01
101	->	70	-0.00	0.00
101	->	71	-0.01	0.01
101	->	72	-0.09	0.05
101	->	73	-0.19	0.31
101	->	74	0.02	0.02
101	->	75	0.01	0.00
101	->	76	-0.04	0.01
101	->	77	-0.06	0.04
101	->	78	-0.02	0.00
101	->	79	-0.00	0.00
101	->	80	-0.00	0.00
101	->	81	-0.02	0.01
101	->	82	-0.00	0.00
101	->	83	-0.00	0.00
101	->	84	-0.00	0.00
101	->	85	-0.00	0.00
101	->	86	0.00	0.00
101	->	87	0.01	0.00
101	->	88	0.01	0.00
101	->	89	0.01	0.00
101	->	90	-0.00	0.01
101	->	91	-0.10	0.05
101	->	92	-1.04	0.34
101	->	93	-11.44	0.73
101	->	94	0.29	0.22
101	->	95	-0.17	0.07
101	->	96	-0.01	0.02

101	->	97	-0.00	0.00
101	->	98	-0.04	0.01
101	->	99	-0.21	0.04
101	->	100	-35.96	0.70
101	->	101	-214.80	2.92
101	->	102	-32.32	0.91
101	->	103	-0.12	0.03
101	->	104	-0.00	0.01
101	->	105	0.01	0.00
101	->	106	0.01	0.00
101	->	107	0.00	0.00
102	->	1	-0.03	0.01
102	->	2	-0.00	0.00
102	->	3	-0.00	0.00
102	->	4	0.00	0.00
102	->	5	0.01	0.00
102	->	6	-0.00	0.00
102	->	7	-0.00	0.00
102	->	8	0.00	0.00
102	->	9	0.00	0.00
102	->	10	0.01	0.00
102	->	11	0.01	0.00
102	->	12	-0.02	0.01
102	->	13	-0.01	0.00
102	->	14	-0.00	0.00
102	->	15	-0.00	0.00
102	->	16	-0.05	0.01
102	->	17	-0.00	0.00
102	->	18	-0.01	0.00
102	->	19	0.04	0.01
102	->	20	-0.00	0.00
102	->	21	-0.00	0.00
102	->	22	-0.07	0.04
102	->	23	-0.01	0.00
102	->	24	-0.00	0.00
102	->	25	-0.02	0.00
102	->	26	-0.00	0.00
102	->	27	-0.00	0.00
102	->	28	-0.00	0.00
102	->	29	-0.00	0.00
102	->	30	0.00	0.00
102	->	31	-0.01	0.00
102	->	32	-0.00	0.00
102	->	33	-0.00	0.00
102	->	34	-0.00	0.00
102	->	35	0.01	0.00
102	->	36	-0.01	0.00
102	->	37	-0.00	0.00
102	->	38	0.01	0.00
102	->	39	0.00	0.00

102	->	40	-0.01	0.00
102	->	41	-0.00	0.00
102	->	42	-0.00	0.00
102	->	43	-0.00	0.00
102	->	44	0.39	0.65
102	->	45	-0.01	0.01
102	->	46	-1.70	1.88
102	->	47	-0.18	0.16
102	->	48	-0.01	0.00
102	->	49	-0.01	0.01
102	->	50	-0.04	0.02
102	->	51	-0.01	0.00
102	->	52	-0.00	0.00
102	->	53	-0.07	0.06
102	->	54	-0.00	0.00
102	->	55	-0.00	0.00
102	->	56	-0.00	0.00
102	->	57	-0.00	0.00
102	->	58	0.02	0.01
102	->	59	-0.03	0.01
102	->	60	-0.01	0.01
102	->	61	-0.09	0.10
102	->	62	0.00	0.00
102	->	63	0.02	0.00
102	->	64	0.02	0.01
102	->	65	0.00	0.00
102	->	66	0.01	0.00
102	->	67	0.00	0.00
102	->	68	-0.02	0.01
102	->	69	-0.02	0.01
102	->	70	-0.01	0.01
102	->	71	-0.02	0.02
102	->	72	-0.30	0.25
102	->	73	-1.43	1.88
102	->	74	-0.17	0.28
102	->	75	0.01	0.01
102	->	76	-0.12	0.07
102	->	77	-1.03	0.65
102	->	78	-0.04	0.01
102	->	79	-0.01	0.00
102	->	80	-0.01	0.00
102	->	81	-0.05	0.02
102	->	82	-0.00	0.00
102	->	83	-0.00	0.00
102	->	84	-0.00	0.00
102	->	85	-0.00	0.01
102	->	86	0.00	0.00
102	->	87	0.01	0.00
102	->	88	0.00	0.00
102	->	89	-0.00	0.01

102	->	90	-0.04	0.02
102	->	91	-1.12	0.54
102	->	92	-11.60	0.80
102	->	93	-3.61	0.73
102	->	94	0.01	0.06
102	->	95	-0.04	0.02
102	->	96	0.01	0.01
102	->	97	0.00	0.00
102	->	98	-0.00	0.00
102	->	99	-0.01	0.01
102	->	100	-0.36	0.09
102	->	101	-32.06	0.90
102	->	102	-213.80	3.24
102	->	103	-32.08	0.84
102	->	104	-0.16	0.06
102	->	105	0.00	0.01
102	->	106	0.01	0.00
102	->	107	0.00	0.00
103	->	1	-0.04	0.02
103	->	2	-0.00	0.00
103	->	3	0.00	0.00
103	->	4	-0.00	0.00
103	->	5	0.01	0.00
103	->	6	-0.00	0.00
103	->	7	0.00	0.00
103	->	8	0.00	0.00
103	->	9	0.00	0.00
103	->	10	0.01	0.00
103	->	11	0.01	0.00
103	->	12	-0.03	0.01
103	->	13	-0.01	0.00
103	->	14	-0.00	0.00
103	->	15	-0.00	0.00
103	->	16	-0.04	0.01
103	->	17	-0.00	0.00
103	->	18	-0.00	0.00
103	->	19	0.03	0.01
103	->	20	-0.00	0.00
103	->	21	-0.00	0.00
103	->	22	-0.08	0.02
103	->	23	-0.01	0.00
103	->	24	-0.00	0.00
103	->	25	-0.01	0.00
103	->	26	-0.00	0.00
103	->	27	-0.00	0.00
103	->	28	-0.00	0.00
103	->	29	-0.00	0.00
103	->	30	0.00	0.00
103	->	31	-0.01	0.00
103	->	32	-0.00	0.00

103	->	33	-0.00	0.00
103	->	34	-0.00	0.00
103	->	35	0.01	0.00
103	->	36	-0.00	0.00
103	->	37	-0.00	0.00
103	->	38	0.01	0.00
103	->	39	-0.00	0.00
103	->	40	-0.01	0.00
103	->	41	-0.00	0.00
103	->	42	-0.00	0.00
103	->	43	-0.00	0.00
103	->	44	-0.11	0.13
103	->	45	-0.00	0.00
103	->	46	-0.11	0.15
103	->	47	-0.06	0.05
103	->	48	-0.00	0.00
103	->	49	-0.00	0.00
103	->	50	-0.03	0.01
103	->	51	-0.00	0.00
103	->	52	-0.00	0.00
103	->	53	-0.02	0.01
103	->	54	-0.00	0.00
103	->	55	-0.00	0.00
103	->	56	-0.00	0.00
103	->	57	-0.00	0.00
103	->	58	0.01	0.00
103	->	59	-0.02	0.00
103	->	60	-0.00	0.00
103	->	61	-0.02	0.01
103	->	62	0.00	0.00
103	->	63	0.03	0.01
103	->	64	0.02	0.01
103	->	65	0.00	0.00
103	->	66	0.02	0.00
103	->	67	-0.00	0.00
103	->	68	-0.02	0.01
103	->	69	-0.02	0.00
103	->	70	-0.01	0.01
103	->	71	-0.01	0.01
103	->	72	-0.57	0.62
103	->	73	-0.61	1.13
103	->	74	-1.82	0.82
103	->	75	-0.00	0.02
103	->	76	-0.23	0.26
103	->	77	-7.75	4.43
103	->	78	-0.10	0.04
103	->	79	-0.01	0.01
103	->	80	-0.02	0.02
103	->	81	-0.56	0.62
103	->	82	-0.00	0.00

103	->	83	-0.00	0.00
103	->	84	-0.00	0.00
103	->	85	-0.00	0.02
103	->	86	0.00	0.00
103	->	87	0.01	0.00
103	->	88	-0.01	0.01
103	->	89	-0.08	0.04
103	->	90	-0.52	0.21
103	->	91	-11.36	0.81
103	->	92	-3.54	0.90
103	->	93	-0.36	0.08
103	->	94	0.00	0.02
103	->	95	0.01	0.00
103	->	96	0.01	0.00
103	->	97	0.00	0.00
103	->	98	0.00	0.00
103	->	99	0.01	0.00
103	->	100	-0.04	0.01
103	->	101	-0.12	0.03
103	->	102	-31.86	0.83
103	->	103	-209.77	4.39
103	->	104	-32.79	0.99
103	->	105	-0.11	0.04
103	->	106	0.01	0.01
103	->	107	0.00	0.00
104	->	1	-0.03	0.01
104	->	2	-0.00	0.00
104	->	3	0.00	0.00
104	->	4	-0.00	0.00
104	->	5	0.01	0.00
104	->	6	-0.00	0.00
104	->	7	0.00	0.00
104	->	8	0.00	0.00
104	->	9	0.00	0.00
104	->	10	0.01	0.00
104	->	11	0.01	0.00
104	->	12	-0.03	0.02
104	->	13	-0.01	0.00
104	->	14	-0.00	0.00
104	->	15	-0.00	0.00
104	->	16	-0.03	0.00
104	->	17	0.00	0.00
104	->	18	-0.00	0.00
104	->	19	0.02	0.00
104	->	20	-0.00	0.00
104	->	21	-0.00	0.00
104	->	22	-0.05	0.01
104	->	23	-0.01	0.00
104	->	24	-0.00	0.00
104	->	25	-0.01	0.00

104	->	26	-0.01	0.00
104	->	27	-0.00	0.00
104	->	28	-0.00	0.00
104	->	29	-0.00	0.00
104	->	30	0.00	0.00
104	->	31	-0.01	0.00
104	->	32	-0.00	0.00
104	->	33	-0.00	0.00
104	->	34	-0.00	0.00
104	->	35	0.01	0.00
104	->	36	-0.00	0.00
104	->	37	-0.00	0.00
104	->	38	0.01	0.00
104	->	39	0.00	0.00
104	->	40	-0.01	0.00
104	->	41	-0.00	0.00
104	->	42	-0.00	0.00
104	->	43	-0.00	0.00
104	->	44	-0.05	0.03
104	->	45	-0.00	0.00
104	->	46	0.01	0.01
104	->	47	-0.04	0.03
104	->	48	-0.00	0.00
104	->	49	-0.00	0.00
104	->	50	-0.01	0.01
104	->	51	-0.01	0.00
104	->	52	-0.00	0.00
104	->	53	-0.02	0.00
104	->	54	-0.00	0.00
104	->	55	-0.00	0.00
104	->	56	-0.00	0.00
104	->	57	-0.00	0.00
104	->	58	0.01	0.00
104	->	59	-0.02	0.00
104	->	60	-0.00	0.00
104	->	61	-0.01	0.00
104	->	62	0.00	0.00
104	->	63	0.02	0.01
104	->	64	0.02	0.00
104	->	65	0.00	0.00
104	->	66	0.02	0.00
104	->	67	-0.00	0.00
104	->	68	-0.02	0.01
104	->	69	-0.01	0.00
104	->	70	-0.01	0.00
104	->	71	0.00	0.00
104	->	72	-0.12	0.95
104	->	73	-0.07	0.05
104	->	74	-2.87	1.49
104	->	75	-0.02	0.04

104	->	76	-0.11	0.06
104	->	77	-3.30	2.67
104	->	78	-0.37	0.36
104	->	79	-0.01	0.01
104	->	80	-0.03	0.02
104	->	81	-3.31	3.75
104	->	82	-0.01	0.00
104	->	83	-0.01	0.00
104	->	84	-0.01	0.01
104	->	85	-0.02	0.07
104	->	86	-0.00	0.00
104	->	87	-0.01	0.01
104	->	88	-0.11	0.06
104	->	89	-1.77	0.60
104	->	90	-6.12	0.54
104	->	91	-3.48	0.71
104	->	92	-0.36	0.11
104	->	93	-0.04	0.02
104	->	94	0.02	0.00
104	->	95	0.02	0.00
104	->	96	0.01	0.00
104	->	97	0.00	0.00
104	->	98	0.01	0.00
104	->	99	0.01	0.00
104	->	100	0.00	0.00
104	->	101	-0.00	0.01
104	->	102	-0.16	0.06
104	->	103	-32.58	0.96
104	->	104	-128.56	4.26
104	->	105	-34.30	0.74
104	->	106	-0.24	0.06
104	->	107	-0.01	0.01
105	->	1	-0.03	0.01
105	->	2	-0.00	0.00
105	->	3	0.00	0.00
105	->	4	-0.00	0.00
105	->	5	0.01	0.00
105	->	6	-0.00	0.00
105	->	7	0.00	0.00
105	->	8	0.01	0.00
105	->	9	0.00	0.00
105	->	10	0.02	0.00
105	->	11	0.01	0.01
105	->	12	-0.04	0.02
105	->	13	-0.01	0.00
105	->	14	-0.00	0.00
105	->	15	-0.00	0.00
105	->	16	-0.02	0.00
105	->	17	0.00	0.00
105	->	18	0.00	0.00

105	->	19	0.02	0.00
105	->	20	0.00	0.00
105	->	21	-0.00	0.00
105	->	22	-0.04	0.01
105	->	23	-0.00	0.00
105	->	24	-0.00	0.00
105	->	25	-0.01	0.00
105	->	26	-0.00	0.00
105	->	27	-0.00	0.00
105	->	28	-0.00	0.00
105	->	29	-0.00	0.00
105	->	30	0.00	0.00
105	->	31	-0.02	0.00
105	->	32	-0.00	0.00
105	->	33	-0.00	0.00
105	->	34	-0.00	0.00
105	->	35	0.01	0.00
105	->	36	-0.00	0.00
105	->	37	-0.00	0.00
105	->	38	0.01	0.00
105	->	39	0.00	0.00
105	->	40	-0.02	0.01
105	->	41	-0.00	0.00
105	->	42	-0.00	0.00
105	->	43	-0.00	0.00
105	->	44	-0.03	0.01
105	->	45	0.00	0.00
105	->	46	0.02	0.00
105	->	47	-0.01	0.00
105	->	48	-0.00	0.00
105	->	49	-0.00	0.00
105	->	50	-0.00	0.00
105	->	51	-0.00	0.00
105	->	52	-0.00	0.00
105	->	53	-0.01	0.00
105	->	54	-0.00	0.00
105	->	55	-0.00	0.00
105	->	56	-0.00	0.00
105	->	57	-0.00	0.00
105	->	58	0.00	0.00
105	->	59	-0.01	0.00
105	->	60	-0.00	0.00
105	->	61	-0.00	0.00
105	->	62	-0.00	0.00
105	->	63	0.01	0.00
105	->	64	0.01	0.00
105	->	65	0.00	0.00
105	->	66	0.01	0.00
105	->	67	-0.00	0.00
105	->	68	-0.02	0.00

105	->	69	-0.01	0.00
105	->	70	-0.01	0.00
105	->	71	-0.00	0.00
105	->	72	0.44	0.30
105	->	73	-0.01	0.00
105	->	74	-0.75	0.79
105	->	75	-0.21	0.33
105	->	76	-0.02	0.01
105	->	77	-0.07	0.02
105	->	78	-1.95	1.44
105	->	79	-0.01	0.00
105	->	80	-0.01	0.00
105	->	81	-0.33	0.75
105	->	82	-0.01	0.01
105	->	83	-0.00	0.00
105	->	84	-0.00	0.00
105	->	85	-0.04	0.12
105	->	86	-0.03	0.01
105	->	87	-0.15	0.07
105	->	88	-0.67	0.30
105	->	89	-11.70	0.70
105	->	90	-0.71	0.39
105	->	91	-0.22	0.10
105	->	92	-0.03	0.02
105	->	93	0.01	0.00
105	->	94	0.02	0.00
105	->	95	0.01	0.00
105	->	96	0.00	0.00
105	->	97	0.00	0.00
105	->	98	0.00	0.00
105	->	99	0.01	0.00
105	->	100	0.01	0.00
105	->	101	0.01	0.00
105	->	102	0.00	0.01
105	->	103	-0.11	0.04
105	->	104	-34.07	0.73
105	->	105	-216.61	3.24
105	->	106	-33.70	1.38
105	->	107	-0.16	0.05
106	->	1	-0.02	0.01
106	->	2	-0.00	0.00
106	->	3	0.00	0.00
106	->	4	-0.00	0.00
106	->	5	0.01	0.00
106	->	6	-0.00	0.00
106	->	7	0.00	0.00
106	->	8	0.01	0.00
106	->	9	0.00	0.00
106	->	10	0.02	0.01
106	->	11	0.01	0.01

106	->	12	-0.03	0.01
106	->	13	-0.01	0.00
106	->	14	-0.00	0.00
106	->	15	-0.00	0.00
106	->	16	-0.01	0.00
106	->	17	0.00	0.00
106	->	18	0.00	0.00
106	->	19	0.01	0.00
106	->	20	0.00	0.00
106	->	21	-0.00	0.00
106	->	22	-0.02	0.00
106	->	23	-0.00	0.00
106	->	24	-0.00	0.00
106	->	25	-0.01	0.00
106	->	26	-0.00	0.00
106	->	27	-0.00	0.00
106	->	28	-0.00	0.00
106	->	29	-0.00	0.00
106	->	30	0.00	0.00
106	->	31	-0.01	0.00
106	->	32	-0.00	0.00
106	->	33	-0.00	0.00
106	->	34	-0.00	0.00
106	->	35	0.00	0.00
106	->	36	-0.00	0.00
106	->	37	-0.00	0.00
106	->	38	0.00	0.00
106	->	39	-0.00	0.00
106	->	40	-0.01	0.01
106	->	41	-0.00	0.00
106	->	42	-0.00	0.00
106	->	43	-0.00	0.00
106	->	44	-0.02	0.00
106	->	45	0.00	0.00
106	->	46	0.01	0.00
106	->	47	-0.01	0.00
106	->	48	-0.00	0.00
106	->	49	-0.00	0.00
106	->	50	-0.00	0.00
106	->	51	-0.00	0.00
106	->	52	-0.00	0.00
106	->	53	-0.01	0.00
106	->	54	-0.00	0.00
106	->	55	-0.00	0.00
106	->	56	-0.00	0.00
106	->	57	-0.00	0.00
106	->	58	0.00	0.00
106	->	59	-0.01	0.00
106	->	60	-0.00	0.00
106	->	61	-0.00	0.00

106	->	62	0.00	0.00
106	->	63	0.01	0.00
106	->	64	0.01	0.00
106	->	65	0.00	0.00
106	->	66	0.01	0.00
106	->	67	-0.00	0.00
106	->	68	-0.02	0.01
106	->	69	-0.01	0.00
106	->	70	-0.00	0.00
106	->	71	-0.00	0.00
106	->	72	0.08	0.15
106	->	73	-0.00	0.00
106	->	74	0.02	0.03
106	->	75	0.10	0.19
106	->	76	-0.02	0.00
106	->	77	-0.04	0.01
106	->	78	-5.18	2.92
106	->	79	-0.01	0.00
106	->	80	-0.00	0.00
106	->	81	-0.06	0.01
106	->	82	-0.01	0.00
106	->	83	-0.00	0.00
106	->	84	-0.00	0.00
106	->	85	-0.01	0.02
106	->	86	-0.18	0.07
106	->	87	-1.19	0.64
106	->	88	-11.47	0.78
106	->	89	-4.51	0.89
106	->	90	-0.26	0.10
106	->	91	-0.03	0.02
106	->	92	0.01	0.00
106	->	93	0.02	0.00
106	->	94	0.01	0.00
106	->	95	0.01	0.00
106	->	96	0.00	0.00
106	->	97	0.00	0.00
106	->	98	0.00	0.00
106	->	99	0.00	0.00
106	->	100	0.00	0.00
106	->	101	0.01	0.00
106	->	102	0.01	0.00
106	->	103	0.01	0.01
106	->	104	-0.24	0.06
106	->	105	-33.42	1.37
106	->	106	-180.66	3.01
106	->	107	-36.25	0.95
107	->	1	-0.01	0.00
107	->	2	-0.00	0.00
107	->	3	0.00	0.00
107	->	4	-0.00	0.00

107	->	5	0.01	0.00
107	->	6	-0.00	0.00
107	->	7	0.00	0.00
107	->	8	0.00	0.00
107	->	9	0.00	0.00
107	->	10	0.01	0.00
107	->	11	0.01	0.00
107	->	12	-0.01	0.01
107	->	13	-0.00	0.00
107	->	14	-0.00	0.00
107	->	15	-0.00	0.00
107	->	16	-0.00	0.00
107	->	17	0.00	0.00
107	->	18	0.00	0.00
107	->	19	0.01	0.00
107	->	20	0.00	0.00
107	->	21	0.00	0.00
107	->	22	-0.01	0.00
107	->	23	-0.00	0.00
107	->	24	0.00	0.00
107	->	25	-0.01	0.00
107	->	26	-0.00	0.00
107	->	27	-0.00	0.00
107	->	28	-0.00	0.00
107	->	29	-0.00	0.00
107	->	30	0.00	0.00
107	->	31	-0.02	0.00
107	->	32	-0.00	0.00
107	->	33	0.00	0.00
107	->	34	-0.00	0.00
107	->	35	0.01	0.00
107	->	36	-0.00	0.00
107	->	37	-0.00	0.00
107	->	38	0.01	0.00
107	->	39	-0.00	0.00
107	->	40	-0.03	0.01
107	->	41	0.00	0.00
107	->	42	-0.00	0.00
107	->	43	-0.00	0.00
107	->	44	-0.01	0.00
107	->	45	0.00	0.00
107	->	46	0.01	0.00
107	->	47	-0.00	0.00
107	->	48	-0.00	0.00
107	->	49	-0.00	0.00
107	->	50	-0.00	0.00
107	->	51	-0.00	0.00
107	->	52	-0.00	0.00
107	->	53	-0.01	0.00
107	->	54	-0.00	0.00

107	->	55	-0.00	0.00
107	->	56	-0.00	0.00
107	->	57	-0.00	0.00
107	->	58	0.00	0.00
107	->	59	-0.01	0.00
107	->	60	-0.00	0.00
107	->	61	-0.00	0.00
107	->	62	-0.00	0.00
107	->	63	0.00	0.00
107	->	64	0.00	0.00
107	->	65	0.00	0.00
107	->	66	0.01	0.00
107	->	67	0.00	0.00
107	->	68	-0.02	0.00
107	->	69	-0.00	0.00
107	->	70	-0.00	0.00
107	->	71	-0.00	0.00
107	->	72	-0.05	0.01
107	->	73	-0.00	0.00
107	->	74	0.01	0.00
107	->	75	0.01	0.01
107	->	76	-0.01	0.00
107	->	77	-0.01	0.00
107	->	78	-0.22	0.37
107	->	79	-0.00	0.00
107	->	80	-0.00	0.00
107	->	81	-0.01	0.00
107	->	82	-0.01	0.00
107	->	83	-0.00	0.00
107	->	84	-0.00	0.00
107	->	85	-0.00	0.00
107	->	86	-2.12	1.14
107	->	87	-10.72	0.77
107	->	88	0.17	0.13
107	->	89	-0.26	0.08
107	->	90	-0.03	0.02
107	->	91	0.01	0.00
107	->	92	0.01	0.00
107	->	93	0.01	0.00
107	->	94	0.00	0.00
107	->	95	0.00	0.00
107	->	96	0.00	0.00
107	->	97	0.00	0.00
107	->	98	0.00	0.00
107	->	99	0.00	0.00
107	->	100	0.00	0.00
107	->	101	0.00	0.00
107	->	102	0.00	0.00
107	->	103	0.00	0.00
107	->	104	-0.01	0.01

107 ->	105	-0.16	0.05
107 ->	106	-36.00	0.96
107 ->	107	-216.16	2.68
