# Supplementary material for: Investigating dynamic and energetic determinants of protein nucleic acid recognition: analysis of the zinc finger zif268-DNA complexes
Source: BMC Struct Biol. 2010 Nov 24;10:42. doi: 10.1186/1472-6807-10-42 (PMC3002361; doi:10.1186/1472-6807-10-42)
Supplement: Additional file 5 — Average pair interaction energies for 1A1J Complex and their standard deviations. [file 1472-6807-10-42-S5.PDF]

=>> 1A1J

Residue ->	Residue	TGBTOT	SD
1 ->	1	-250.02	6.13
1 ->	2	-0.88	0.34
1 ->	3	-1.27	0.28
1 ->	4	-0.13	0.04
1 ->	5	-0.15	0.03
1 ->	6	-0.00	0.00
1 ->	7	-0.00	0.00
1 ->	8	-0.04	0.01
1 ->	9	-0.00	0.00
1 ->	10	-0.10	0.02
1 ->	11	-0.14	0.04
1 ->	12	0.65	0.82
1 ->	13	-4.71	1.72
1 ->	14	-3.75	1.15
1 ->	15	-4.01	1.38
1 ->	16	0.27	0.34
1 ->	17	-0.10	0.34
1 ->	18	0.01	0.01
1 ->	19	-0.33	0.25
1 ->	20	-0.05	0.03
1 ->	21	0.00	0.00
1 ->	22	0.17	0.14
1 ->	23	-0.01	0.01
1 ->	24	-0.00	0.00
1 ->	25	0.04	0.01
1 ->	26	0.00	0.00
1 ->	27	-0.01	0.01
1 ->	28	0.00	0.00
1 ->	29	0.00	0.00
1 ->	30	-0.00	0.00
1 ->	31	0.02	0.00
1 ->	32	0.00	0.00
1 ->	33	0.00	0.00
1 ->	34	-0.00	0.00
1 ->	35	-0.01	0.00
1 ->	36	0.00	0.00
1 ->	37	-0.00	0.00
1 ->	38	-0.01	0.00
1 ->	39	0.00	0.00
1 ->	40	0.01	0.00
1 ->	41	0.00	0.00
1 ->	42	0.00	0.00
1 ->	43	0.00	0.00
1 ->	44	0.05	0.01
1 ->	45	0.00	0.00
1 ->	46	-0.03	0.01

1 ->	47	0.00	0.00
1 ->	48	0.00	0.00
1 ->	49	0.00	0.00
1 ->	50	0.00	0.00
1 ->	51	0.00	0.00
1 ->	52	0.00	0.00
1 ->	53	0.01	0.00
1 ->	54	0.00	0.00
1 ->	55	0.00	0.00
1 ->	56	0.00	0.00
1 ->	57	0.00	0.00
1 ->	58	-0.00	0.00
1 ->	59	0.00	0.00
1 ->	60	0.00	0.00
1 ->	61	0.00	0.00
1 ->	62	-0.00	0.00
1 ->	63	-0.00	0.00
1 ->	64	-0.00	0.00
1 ->	65	0.00	0.00
1 ->	66	-0.00	0.00
1 ->	67	0.00	0.00
1 ->	68	0.00	0.00
1 ->	69	0.00	0.00
1 ->	70	0.00	0.00
1 ->	71	-0.00	0.00
1 ->	72	0.02	0.00
1 ->	73	0.00	0.00
1 ->	74	-0.02	0.00
1 ->	75	-0.01	0.00
1 ->	76	0.01	0.00
1 ->	77	0.02	0.01
1 ->	78	0.01	0.00
1 ->	79	0.00	0.00
1 ->	80	0.00	0.00
1 ->	81	0.02	0.01
1 ->	82	0.00	0.00
1 ->	83	0.00	0.00
1 ->	84	-0.00	0.00
1 ->	85	-0.00	0.00
1 ->	86	-0.01	0.00
1 ->	87	-0.01	0.00
1 ->	88	-0.01	0.00
1 ->	89	-0.02	0.00
1 ->	90	-0.06	0.03
1 ->	91	-1.23	0.91
1 ->	92	-8.50	6.28
1 ->	93	-1.14	1.24
1 ->	94	-0.11	0.02
1 ->	95	-0.04	0.01
1 ->	96	-0.01	0.01

1 ->	97	-0.02	0.01
1 ->	98	-0.02	0.00
1 ->	99	-0.02	0.00
1 ->	100	-0.02	0.00
1 ->	101	-0.04	0.01
1 ->	102	-0.05	0.01
1 ->	103	-0.04	0.01
1 ->	104	-0.03	0.01
1 ->	105	-0.02	0.01
1 ->	106	-0.01	0.00
2 ->	1	-0.89	0.34
2 ->	2	1.54	0.68
2 ->	3	-12.81	0.39
2 ->	4	-0.45	0.19
2 ->	5	-0.02	0.01
2 ->	6	-0.01	0.00
2 ->	7	-0.00	0.00
2 ->	8	0.00	0.00
2 ->	9	-0.00	0.00
2 ->	10	0.00	0.00
2 ->	11	-0.00	0.00
2 ->	12	-0.01	0.01
2 ->	13	-1.52	0.95
2 ->	14	-0.92	0.30
2 ->	15	-0.84	0.36
2 ->	16	-0.11	0.08
2 ->	17	-0.04	0.02
2 ->	18	-0.01	0.00
2 ->	19	-0.01	0.00
2 ->	20	-0.04	0.02
2 ->	21	-0.00	0.00
2 ->	22	-0.00	0.00
2 ->	23	-0.00	0.00
2 ->	24	-0.00	0.00
2 ->	25	-0.00	0.00
2 ->	26	-0.00	0.00
2 ->	27	-0.00	0.00
2 ->	28	-0.00	0.00
2 ->	29	-0.00	0.00
2 ->	30	-0.00	0.00
2 ->	31	-0.00	0.00
2 ->	32	0.00	0.00
2 ->	33	0.00	0.00
2 ->	34	-0.00	0.00
2 ->	35	-0.00	0.00
2 ->	36	0.00	0.00
2 ->	37	0.00	0.00
2 ->	38	-0.00	0.00
2 ->	39	0.00	0.00
2 ->	40	-0.00	0.00

2	->	41	0.00	0.00
2	->	42	0.00	0.00
2	->	43	0.00	0.00
2	->	44	0.00	0.00
2	->	45	0.00	0.00
2	->	46	-0.00	0.00
2	->	47	-0.00	0.00
2	->	48	0.00	0.00
2	->	49	0.00	0.00
2	->	50	0.00	0.00
2	->	51	0.00	0.00
2	->	52	0.00	0.00
2	->	53	0.00	0.00
2	->	54	0.00	0.00
2	->	55	0.00	0.00
2	->	56	0.00	0.00
2	->	57	0.00	0.00
2	->	58	-0.00	0.00
2	->	59	0.00	0.00
2	->	60	0.00	0.00
2	->	61	0.00	0.00
2	->	62	0.00	0.00
2	->	63	-0.00	0.00
2	->	64	0.00	0.00
2	->	65	0.00	0.00
2	->	66	-0.00	0.00
2	->	67	0.00	0.00
2	->	68	0.00	0.00
2	->	69	0.00	0.00
2	->	70	0.00	0.00
2	->	71	0.00	0.00
2	->	72	0.00	0.00
2	->	73	0.00	0.00
2	->	74	-0.00	0.00
2	->	75	-0.00	0.00
2	->	76	0.00	0.00
2	->	77	0.00	0.00
2	->	78	0.00	0.00
2	->	79	0.00	0.00
2	->	80	0.00	0.00
2	->	81	0.00	0.00
2	->	82	0.00	0.00
2	->	83	0.00	0.00
2	->	84	-0.00	0.00
2	->	85	0.00	0.00
2	->	86	-0.00	0.00
2	->	87	-0.00	0.00
2	->	88	-0.00	0.00
2	->	89	0.00	0.00
2	->	90	-0.00	0.00

2	->	91	-0.00	0.00
2	->	92	-0.01	0.00
2	->	93	-0.01	0.00
2	->	94	-0.01	0.00
2	->	95	-0.01	0.00
2	->	96	-0.00	0.01
2	->	97	-0.00	0.00
2	->	98	-0.00	0.00
2	->	99	-0.00	0.00
2	->	100	-0.00	0.00
2	->	101	-0.00	0.00
2	->	102	-0.00	0.00
2	->	103	-0.00	0.00
2	->	104	-0.00	0.00
2	->	105	0.00	0.00
2	->	106	0.00	0.00
3	->	1	-1.28	0.28
3	->	2	-12.92	0.39
3	->	3	19.57	1.90
3	->	4	-9.93	0.55
3	->	5	-0.51	0.10
3	->	6	-0.03	0.01
3	->	7	0.00	0.01
3	->	8	-0.00	0.00
3	->	9	-0.00	0.00
3	->	10	-0.00	0.01
3	->	11	-0.02	0.02
3	->	12	-0.23	0.06
3	->	13	-2.97	0.67
3	->	14	-3.41	0.86
3	->	15	-1.16	0.43
3	->	16	-2.05	1.01
3	->	17	-1.77	0.82
3	->	18	-0.04	0.02
3	->	19	-0.08	0.04
3	->	20	-1.70	0.42
3	->	21	-0.05	0.02
3	->	22	-0.01	0.01
3	->	23	-0.02	0.01
3	->	24	-0.02	0.01
3	->	25	-0.00	0.00
3	->	26	-0.00	0.00
3	->	27	-0.01	0.00
3	->	28	-0.00	0.00
3	->	29	0.00	0.00
3	->	30	-0.00	0.00
3	->	31	0.00	0.00
3	->	32	-0.00	0.00
3	->	33	-0.00	0.00
3	->	34	-0.00	0.00

3 ->	35	-0.00	0.00
3 ->	36	0.00	0.00
3 ->	37	0.00	0.00
3 ->	38	-0.00	0.00
3 ->	39	0.00	0.00
3 ->	40	0.00	0.00
3 ->	41	0.00	0.00
3 ->	42	-0.00	0.00
3 ->	43	-0.00	0.00
3 ->	44	-0.00	0.00
3 ->	45	-0.00	0.00
3 ->	46	-0.00	0.00
3 ->	47	-0.00	0.00
3 ->	48	0.00	0.00
3 ->	49	0.00	0.00
3 ->	50	-0.00	0.00
3 ->	51	0.00	0.00
3 ->	52	0.00	0.00
3 ->	53	-0.00	0.00
3 ->	54	0.00	0.00
3 ->	55	0.00	0.00
3 ->	56	0.00	0.00
3 ->	57	0.00	0.00
3 ->	58	0.00	0.00
3 ->	59	0.00	0.00
3 ->	60	0.00	0.00
3 ->	61	0.00	0.00
3 ->	62	0.00	0.00
3 ->	63	0.00	0.00
3 ->	64	0.00	0.00
3 ->	65	0.00	0.00
3 ->	66	0.00	0.00
3 ->	67	0.00	0.00
3 ->	68	-0.00	0.00
3 ->	69	-0.00	0.00
3 ->	70	0.00	0.00
3 ->	71	0.00	0.00
3 ->	72	-0.00	0.00
3 ->	73	-0.00	0.00
3 ->	74	0.00	0.00
3 ->	75	0.00	0.00
3 ->	76	-0.00	0.00
3 ->	77	-0.00	0.00
3 ->	78	-0.00	0.00
3 ->	79	0.00	0.00
3 ->	80	0.00	0.00
3 ->	81	-0.00	0.00
3 ->	82	0.00	0.00
3 ->	83	0.00	0.00
3 ->	84	0.00	0.00

3	->	85	0.00	0.00
3	->	86	0.00	0.00
3	->	87	0.00	0.00
3	->	88	0.00	0.00
3	->	89	-0.00	0.00
3	->	90	-0.00	0.00
3	->	91	-0.01	0.00
3	->	92	-0.00	0.01
3	->	93	-0.01	0.00
3	->	94	-0.01	0.01
3	->	95	-0.02	0.02
3	->	96	-0.03	0.11
3	->	97	-0.02	0.03
3	->	98	-0.00	0.00
3	->	99	-0.00	0.00
3	->	100	-0.00	0.00
3	->	101	-0.00	0.00
3	->	102	-0.00	0.00
3	->	103	0.00	0.00
3	->	104	0.00	0.00
3	->	105	0.00	0.00
3	->	106	0.00	0.00
4	->	1	-0.13	0.04
4	->	2	-0.48	0.20
4	->	3	-9.91	0.54
4	->	4	22.41	0.89
4	->	5	-12.31	0.63
4	->	6	-0.55	0.18
4	->	7	0.05	0.04
4	->	8	-0.04	0.07
4	->	9	-0.01	0.00
4	->	10	-0.05	0.03
4	->	11	-0.27	0.36
4	->	12	-0.36	0.58
4	->	13	-1.90	0.38
4	->	14	-0.56	0.38
4	->	15	-0.01	0.01
4	->	16	-0.02	0.01
4	->	17	-0.01	0.01
4	->	18	-0.00	0.00
4	->	19	-0.01	0.01
4	->	20	-1.09	0.35
4	->	21	-0.01	0.00
4	->	22	0.01	0.01
4	->	23	-0.07	0.03
4	->	24	-0.08	0.04
4	->	25	-0.01	0.00
4	->	26	-0.00	0.00
4	->	27	-0.06	0.02
4	->	28	-0.00	0.00

4 ->	29	-0.00	0.00
4 ->	30	-0.00	0.00
4 ->	31	-0.00	0.00
4 ->	32	-0.00	0.00
4 ->	33	0.00	0.00
4 ->	34	-0.00	0.00
4 ->	35	0.00	0.00
4 ->	36	-0.00	0.00
4 ->	37	0.00	0.00
4 ->	38	0.00	0.00
4 ->	39	0.00	0.00
4 ->	40	-0.00	0.00
4 ->	41	-0.00	0.00
4 ->	42	0.00	0.00
4 ->	43	0.00	0.00
4 ->	44	-0.00	0.00
4 ->	45	-0.00	0.00
4 ->	46	0.00	0.00
4 ->	47	0.00	0.00
4 ->	48	0.00	0.00
4 ->	49	0.00	0.00
4 ->	50	0.00	0.00
4 ->	51	0.00	0.00
4 ->	52	0.00	0.00
4 ->	53	-0.00	0.00
4 ->	54	0.00	0.00
4 ->	55	0.00	0.00
4 ->	56	0.00	0.00
4 ->	57	0.00	0.00
4 ->	58	0.00	0.00
4 ->	59	0.00	0.00
4 ->	60	0.00	0.00
4 ->	61	0.00	0.00
4 ->	62	0.00	0.00
4 ->	63	0.00	0.00
4 ->	64	0.00	0.00
4 ->	65	0.00	0.00
4 ->	66	0.00	0.00
4 ->	67	0.00	0.00
4 ->	68	0.00	0.00
4 ->	69	-0.00	0.00
4 ->	70	0.00	0.00
4 ->	71	0.00	0.00
4 ->	72	-0.00	0.00
4 ->	73	0.00	0.00
4 ->	74	-0.00	0.00
4 ->	75	0.00	0.00
4 ->	76	-0.00	0.00
4 ->	77	0.00	0.00
4 ->	78	0.00	0.00

4	->	79	0.00	0.00
4	->	80	0.00	0.00
4	->	81	0.00	0.00
4	->	82	0.00	0.00
4	->	83	0.00	0.00
4	->	84	0.00	0.00
4	->	85	0.00	0.00
4	->	86	0.00	0.00
4	->	87	0.00	0.00
4	->	88	0.00	0.00
4	->	89	-0.00	0.00
4	->	90	-0.00	0.00
4	->	91	-0.01	0.01
4	->	92	-0.01	0.00
4	->	93	-0.00	0.00
4	->	94	-0.00	0.00
4	->	95	-0.00	0.00
4	->	96	-0.00	0.00
4	->	97	0.00	0.00
4	->	98	-0.00	0.00
4	->	99	0.00	0.00
4	->	100	0.00	0.00
4	->	101	0.00	0.00
4	->	102	-0.00	0.00
4	->	103	-0.00	0.00
4	->	104	-0.00	0.00
4	->	105	-0.00	0.00
4	->	106	-0.00	0.00
5	->	1	-0.15	0.03
5	->	2	-0.02	0.01
5	->	3	-0.51	0.10
5	->	4	-12.36	0.63
5	->	5	-28.33	3.94
5	->	6	-1.51	0.53
5	->	7	-4.99	2.10
5	->	8	0.09	0.14
5	->	9	-0.03	0.09
5	->	10	4.66	2.76
5	->	11	-0.81	0.70
5	->	12	-0.92	0.89
5	->	13	-0.68	0.31
5	->	14	-0.60	0.33
5	->	15	0.02	0.01
5	->	16	-0.04	0.01
5	->	17	-0.00	0.00
5	->	18	-0.01	0.00
5	->	19	0.01	0.01
5	->	20	-0.93	0.23
5	->	21	-0.00	0.02
5	->	22	-0.16	0.03

5 ->	23	1.56	0.48
5 ->	24	-0.30	0.29
5 ->	25	-0.06	0.02
5 ->	26	-0.07	0.02
5 ->	27	2.64	1.11
5 ->	28	-0.02	0.01
5 ->	29	-0.01	0.00
5 ->	30	-0.00	0.00
5 ->	31	-0.02	0.00
5 ->	32	-0.00	0.00
5 ->	33	0.00	0.00
5 ->	34	-0.00	0.00
5 ->	35	0.00	0.00
5 ->	36	-0.00	0.00
5 ->	37	0.00	0.00
5 ->	38	0.00	0.00
5 ->	39	0.00	0.00
5 ->	40	-0.01	0.00
5 ->	41	-0.00	0.00
5 ->	42	-0.00	0.00
5 ->	43	-0.00	0.00
5 ->	44	-0.03	0.00
5 ->	45	-0.00	0.00
5 ->	46	0.02	0.00
5 ->	47	-0.00	0.00
5 ->	48	-0.00	0.00
5 ->	49	-0.00	0.00
5 ->	50	-0.00	0.00
5 ->	51	-0.00	0.00
5 ->	52	-0.00	0.00
5 ->	53	-0.00	0.00
5 ->	54	-0.00	0.00
5 ->	55	0.00	0.00
5 ->	56	-0.00	0.00
5 ->	57	-0.00	0.00
5 ->	58	0.00	0.00
5 ->	59	-0.00	0.00
5 ->	60	-0.00	0.00
5 ->	61	-0.00	0.00
5 ->	62	0.00	0.00
5 ->	63	0.00	0.00
5 ->	64	0.00	0.00
5 ->	65	-0.00	0.00
5 ->	66	0.00	0.00
5 ->	67	0.00	0.00
5 ->	68	-0.00	0.00
5 ->	69	-0.00	0.00
5 ->	70	-0.00	0.00
5 ->	71	0.00	0.00
5 ->	72	-0.01	0.00

5	->	73	-0.00	0.00
5	->	74	0.01	0.00
5	->	75	0.00	0.00
5	->	76	-0.00	0.00
5	->	77	-0.01	0.00
5	->	78	-0.01	0.00
5	->	79	-0.00	0.00
5	->	80	-0.00	0.00
5	->	81	-0.00	0.00
5	->	82	-0.00	0.00
5	->	83	-0.00	0.00
5	->	84	0.00	0.00
5	->	85	0.00	0.00
5	->	86	0.00	0.00
5	->	87	0.00	0.00
5	->	88	0.01	0.00
5	->	89	0.02	0.00
5	->	90	0.06	0.01
5	->	91	0.14	0.03
5	->	92	0.05	0.01
5	->	93	0.02	0.00
5	->	94	0.01	0.00
5	->	95	0.01	0.00
5	->	96	0.00	0.00
5	->	97	0.01	0.00
5	->	98	0.01	0.00
5	->	99	0.01	0.00
5	->	100	0.01	0.00
5	->	101	0.01	0.00
5	->	102	0.01	0.00
5	->	103	0.01	0.00
5	->	104	0.01	0.00
5	->	105	0.01	0.00
5	->	106	0.01	0.00
6	->	1	-0.00	0.00
6	->	2	-0.01	0.00
6	->	3	-0.03	0.01
6	->	4	-0.58	0.20
6	->	5	-1.61	0.52
6	->	6	1.11	0.62
6	->	7	-13.27	0.34
6	->	8	-0.40	0.14
6	->	9	-0.04	0.01
6	->	10	-0.07	0.05
6	->	11	-0.19	0.26
6	->	12	-0.03	0.03
6	->	13	-0.04	0.02
6	->	14	-0.04	0.02
6	->	15	-0.00	0.00
6	->	16	-0.00	0.00

6	->	17	-0.00	0.00
6	->	18	-0.00	0.00
6	->	19	-0.01	0.00
6	->	20	-0.39	0.23
6	->	21	-0.02	0.01
6	->	22	-0.01	0.00
6	->	23	-0.14	0.09
6	->	24	-1.32	0.33
6	->	25	-0.02	0.01
6	->	26	-0.01	0.00
6	->	27	-0.43	0.28
6	->	28	-0.09	0.16
6	->	29	-0.00	0.00
6	->	30	-0.01	0.00
6	->	31	0.00	0.00
6	->	32	-0.00	0.00
6	->	33	-0.00	0.00
6	->	34	0.00	0.00
6	->	35	-0.00	0.00
6	->	36	0.00	0.00
6	->	37	0.00	0.00
6	->	38	-0.00	0.00
6	->	39	-0.00	0.00
6	->	40	0.00	0.00
6	->	41	0.00	0.00
6	->	42	0.00	0.00
6	->	43	-0.00	0.00
6	->	44	0.00	0.00
6	->	45	0.00	0.00
6	->	46	-0.00	0.00
6	->	47	0.00	0.00
6	->	48	0.00	0.00
6	->	49	0.00	0.00
6	->	50	0.00	0.00
6	->	51	0.00	0.00
6	->	52	0.00	0.00
6	->	53	0.00	0.00
6	->	54	0.00	0.00
6	->	55	0.00	0.00
6	->	56	0.00	0.00
6	->	57	0.00	0.00
6	->	58	-0.00	0.00
6	->	59	0.00	0.00
6	->	60	0.00	0.00
6	->	61	0.00	0.00
6	->	62	0.00	0.00
6	->	63	-0.00	0.00
6	->	64	0.00	0.00
6	->	65	0.00	0.00
6	->	66	-0.00	0.00

6	->	67	0.00	0.00
6	->	68	0.00	0.00
6	->	69	0.00	0.00
6	->	70	0.00	0.00
6	->	71	0.00	0.00
6	->	72	0.00	0.00
6	->	73	0.00	0.00
6	->	74	-0.00	0.00
6	->	75	-0.00	0.00
6	->	76	0.00	0.00
6	->	77	0.00	0.00
6	->	78	0.00	0.00
6	->	79	0.00	0.00
6	->	80	0.00	0.00
6	->	81	0.00	0.00
6	->	82	0.00	0.00
6	->	83	0.00	0.00
6	->	84	-0.00	0.00
6	->	85	-0.00	0.00
6	->	86	-0.00	0.00
6	->	87	-0.00	0.00
6	->	88	-0.00	0.00
6	->	89	-0.00	0.00
6	->	90	-0.00	0.00
6	->	91	-0.01	0.00
6	->	92	-0.00	0.00
6	->	93	-0.00	0.00
6	->	94	-0.00	0.00
6	->	95	-0.00	0.00
6	->	96	-0.00	0.00
6	->	97	-0.00	0.00
6	->	98	-0.00	0.00
6	->	99	-0.00	0.00
6	->	100	-0.00	0.00
6	->	101	-0.00	0.00
6	->	102	-0.00	0.00
6	->	103	-0.00	0.00
6	->	104	-0.00	0.00
6	->	105	-0.00	0.00
6	->	106	-0.00	0.00
7	->	1	-0.00	0.00
7	->	2	-0.00	0.00
7	->	3	0.00	0.01
7	->	4	0.05	0.04
7	->	5	-5.07	2.09
7	->	6	-13.41	0.34
7	->	7	26.83	1.98
7	->	8	-11.29	0.48
7	->	9	-1.48	0.40
7	->	10	-2.07	1.27

7 ->	11	-0.47	0.41
7 ->	12	-0.05	0.03
7 ->	13	-0.01	0.01
7 ->	14	-0.04	0.02
7 ->	15	-0.00	0.00
7 ->	16	0.00	0.00
7 ->	17	-0.00	0.00
7 ->	18	0.00	0.00
7 ->	19	0.00	0.00
7 ->	20	-0.03	0.01
7 ->	21	-0.01	0.01
7 ->	22	0.02	0.01
7 ->	23	-0.35	0.17
7 ->	24	-0.16	0.18
7 ->	25	-0.00	0.01
7 ->	26	0.00	0.01
7 ->	27	-2.33	0.96
7 ->	28	-0.08	0.05
7 ->	29	-0.01	0.00
7 ->	30	-0.00	0.00
7 ->	31	-0.00	0.00
7 ->	32	-0.00	0.00
7 ->	33	-0.00	0.00
7 ->	34	0.00	0.00
7 ->	35	0.00	0.00
7 ->	36	0.00	0.00
7 ->	37	0.00	0.00
7 ->	38	-0.00	0.00
7 ->	39	0.00	0.00
7 ->	40	0.00	0.00
7 ->	41	0.00	0.00
7 ->	42	0.00	0.00
7 ->	43	-0.00	0.00
7 ->	44	-0.00	0.00
7 ->	45	-0.00	0.00
7 ->	46	-0.00	0.00
7 ->	47	0.00	0.00
7 ->	48	0.00	0.00
7 ->	49	0.00	0.00
7 ->	50	0.00	0.00
7 ->	51	0.00	0.00
7 ->	52	0.00	0.00
7 ->	53	0.00	0.00
7 ->	54	0.00	0.00
7 ->	55	0.00	0.00
7 ->	56	0.00	0.00
7 ->	57	0.00	0.00
7 ->	58	0.00	0.00
7 ->	59	0.00	0.00
7 ->	60	0.00	0.00

7	->	61	0.00	0.00
7	->	62	0.00	0.00
7	->	63	0.00	0.00
7	->	64	-0.00	0.00
7	->	65	0.00	0.00
7	->	66	0.00	0.00
7	->	67	0.00	0.00
7	->	68	0.00	0.00
7	->	69	0.00	0.00
7	->	70	-0.00	0.00
7	->	71	0.00	0.00
7	->	72	-0.00	0.00
7	->	73	0.00	0.00
7	->	74	-0.00	0.00
7	->	75	-0.00	0.00
7	->	76	0.00	0.00
7	->	77	0.00	0.00
7	->	78	0.00	0.00
7	->	79	0.00	0.00
7	->	80	0.00	0.00
7	->	81	0.00	0.00
7	->	82	0.00	0.00
7	->	83	0.00	0.00
7	->	84	-0.00	0.00
7	->	85	0.00	0.00
7	->	86	-0.00	0.00
7	->	87	-0.00	0.00
7	->	88	0.00	0.00
7	->	89	-0.00	0.00
7	->	90	-0.01	0.01
7	->	91	-0.02	0.01
7	->	92	-0.00	0.00
7	->	93	-0.00	0.00
7	->	94	-0.00	0.00
7	->	95	-0.00	0.00
7	->	96	-0.00	0.00
7	->	97	-0.00	0.00
7	->	98	-0.00	0.00
7	->	99	-0.00	0.00
7	->	100	-0.00	0.00
7	->	101	-0.00	0.00
7	->	102	-0.00	0.00
7	->	103	-0.00	0.00
7	->	104	-0.00	0.00
7	->	105	-0.00	0.00
7	->	106	0.00	0.00
8	->	1	-0.04	0.01
8	->	2	0.00	0.00
8	->	3	-0.00	0.00
8	->	4	-0.04	0.07

8 ->	5	0.08	0.15
8 ->	6	-0.42	0.15
8 ->	7	-11.30	0.49
8 ->	8	-70.79	1.49
8 ->	9	-18.56	0.45
8 ->	10	-0.52	0.15
8 ->	11	-0.54	0.50
8 ->	12	-0.05	0.02
8 ->	13	-0.11	0.20
8 ->	14	-0.00	0.00
8 ->	15	0.00	0.00
8 ->	16	-0.01	0.00
8 ->	17	0.00	0.00
8 ->	18	0.00	0.00
8 ->	19	0.01	0.00
8 ->	20	-0.00	0.00
8 ->	21	0.00	0.00
8 ->	22	-0.01	0.00
8 ->	23	-0.00	0.01
8 ->	24	-0.01	0.00
8 ->	25	-0.01	0.00
8 ->	26	-0.00	0.00
8 ->	27	-0.02	0.02
8 ->	28	-0.01	0.00
8 ->	29	-0.00	0.00
8 ->	30	-0.00	0.00
8 ->	31	-0.01	0.00
8 ->	32	-0.00	0.00
8 ->	33	0.00	0.00
8 ->	34	-0.00	0.00
8 ->	35	0.00	0.00
8 ->	36	-0.00	0.00
8 ->	37	0.00	0.00
8 ->	38	0.00	0.00
8 ->	39	0.00	0.00
8 ->	40	-0.00	0.00
8 ->	41	-0.00	0.00
8 ->	42	-0.00	0.00
8 ->	43	-0.00	0.00
8 ->	44	-0.01	0.00
8 ->	45	-0.00	0.00
8 ->	46	0.00	0.00
8 ->	47	-0.00	0.00
8 ->	48	-0.00	0.00
8 ->	49	-0.00	0.00
8 ->	50	-0.00	0.00
8 ->	51	-0.00	0.00
8 ->	52	-0.00	0.00
8 ->	53	-0.00	0.00
8 ->	54	-0.00	0.00

8 ->	55	0.00	0.00
8 ->	56	-0.00	0.00
8 ->	57	-0.00	0.00
8 ->	58	0.00	0.00
8 ->	59	-0.00	0.00
8 ->	60	-0.00	0.00
8 ->	61	0.00	0.00
8 ->	62	0.00	0.00
8 ->	63	0.00	0.00
8 ->	64	0.00	0.00
8 ->	65	-0.00	0.00
8 ->	66	0.00	0.00
8 ->	67	0.00	0.00
8 ->	68	-0.00	0.00
8 ->	69	-0.00	0.00
8 ->	70	-0.00	0.00
8 ->	71	0.00	0.00
8 ->	72	-0.00	0.00
8 ->	73	-0.00	0.00
8 ->	74	0.00	0.00
8 ->	75	0.00	0.00
8 ->	76	-0.00	0.00
8 ->	77	-0.00	0.00
8 ->	78	-0.00	0.00
8 ->	79	-0.00	0.00
8 ->	80	-0.00	0.00
8 ->	81	-0.00	0.00
8 ->	82	-0.00	0.00
8 ->	83	0.00	0.00
8 ->	84	0.00	0.00
8 ->	85	0.00	0.00
8 ->	86	0.00	0.00
8 ->	87	0.00	0.00
8 ->	88	0.00	0.00
8 ->	89	0.01	0.00
8 ->	90	0.01	0.00
8 ->	91	0.02	0.00
8 ->	92	0.02	0.00
8 ->	93	0.01	0.00
8 ->	94	0.01	0.00
8 ->	95	0.00	0.00
8 ->	96	0.00	0.00
8 ->	97	0.00	0.00
8 ->	98	0.00	0.00
8 ->	99	0.00	0.00
8 ->	100	0.00	0.00
8 ->	101	0.00	0.00
8 ->	102	0.00	0.00
8 ->	103	0.00	0.00
8 ->	104	0.01	0.00

8	->	105	0.01	0.00
8	->	106	0.00	0.00
9	->	1	-0.00	0.00
9	->	2	-0.00	0.00
9	->	3	-0.00	0.00
9	->	4	-0.01	0.00
9	->	5	-0.03	0.09
9	->	6	-0.04	0.01
9	->	7	-1.47	0.39
9	->	8	-18.64	0.45
9	->	9	14.85	1.80
9	->	10	-8.82	0.83
9	->	11	-0.47	0.21
9	->	12	-0.04	0.04
9	->	13	-0.01	0.01
9	->	14	-0.01	0.00
9	->	15	-0.00	0.00
9	->	16	-0.00	0.00
9	->	17	-0.00	0.00
9	->	18	-0.00	0.00
9	->	19	-0.00	0.00
9	->	20	-0.00	0.00
9	->	21	-0.00	0.00
9	->	22	-0.00	0.00
9	->	23	-0.03	0.03
9	->	24	-0.01	0.00
9	->	25	-0.00	0.00
9	->	26	-0.01	0.01
9	->	27	-0.22	0.22
9	->	28	-0.00	0.00
9	->	29	-0.00	0.00
9	->	30	-0.00	0.00
9	->	31	0.00	0.00
9	->	32	0.00	0.00
9	->	33	-0.00	0.00
9	->	34	0.00	0.00
9	->	35	-0.00	0.00
9	->	36	0.00	0.00
9	->	37	0.00	0.00
9	->	38	-0.00	0.00
9	->	39	-0.00	0.00
9	->	40	0.00	0.00
9	->	41	0.00	0.00
9	->	42	-0.00	0.00
9	->	43	0.00	0.00
9	->	44	-0.00	0.00
9	->	45	0.00	0.00
9	->	46	0.00	0.00
9	->	47	-0.00	0.00
9	->	48	0.00	0.00

9	->	49	0.00	0.00
9	->	50	0.00	0.00
9	->	51	-0.00	0.00
9	->	52	-0.00	0.00
9	->	53	-0.00	0.00
9	->	54	0.00	0.00
9	->	55	0.00	0.00
9	->	56	0.00	0.00
9	->	57	0.00	0.00
9	->	58	0.00	0.00
9	->	59	-0.00	0.00
9	->	60	0.00	0.00
9	->	61	0.00	0.00
9	->	62	0.00	0.00
9	->	63	-0.00	0.00
9	->	64	0.00	0.00
9	->	65	0.00	0.00
9	->	66	0.00	0.00
9	->	67	0.00	0.00
9	->	68	-0.00	0.00
9	->	69	-0.00	0.00
9	->	70	0.00	0.00
9	->	71	0.00	0.00
9	->	72	-0.00	0.00
9	->	73	0.00	0.00
9	->	74	0.00	0.00
9	->	75	0.00	0.00
9	->	76	-0.00	0.00
9	->	77	-0.00	0.00
9	->	78	-0.00	0.00
9	->	79	0.00	0.00
9	->	80	0.00	0.00
9	->	81	-0.00	0.00
9	->	82	0.00	0.00
9	->	83	0.00	0.00
9	->	84	0.00	0.00
9	->	85	0.00	0.00
9	->	86	0.00	0.00
9	->	87	-0.00	0.00
9	->	88	-0.00	0.00
9	->	89	-0.00	0.00
9	->	90	-0.00	0.00
9	->	91	-0.01	0.00
9	->	92	0.00	0.00
9	->	93	0.00	0.00
9	->	94	0.00	0.00
9	->	95	0.00	0.00
9	->	96	-0.00	0.00
9	->	97	-0.00	0.00
9	->	98	-0.00	0.00

9	->	99	0.00	0.00
9	->	100	0.00	0.00
9	->	101	0.00	0.00
9	->	102	0.00	0.00
9	->	103	0.00	0.00
9	->	104	0.00	0.00
9	->	105	0.00	0.00
9	->	106	0.00	0.00
10	->	1	-0.10	0.02
10	->	2	0.00	0.00
10	->	3	-0.00	0.01
10	->	4	-0.05	0.03
10	->	5	4.65	2.77
10	->	6	-0.07	0.05
10	->	7	-2.08	1.27
10	->	8	-0.54	0.14
10	->	9	-8.94	0.82
10	->	10	-27.62	9.09
10	->	11	-13.90	0.65
10	->	12	-2.23	3.05
10	->	13	-0.12	0.09
10	->	14	-0.03	0.05
10	->	15	-0.00	0.00
10	->	16	-0.03	0.00
10	->	17	0.00	0.00
10	->	18	0.00	0.00
10	->	19	0.04	0.01
10	->	20	-0.01	0.01
10	->	21	-0.00	0.00
10	->	22	-0.10	0.03
10	->	23	2.16	0.66
10	->	24	0.04	0.01
10	->	25	-0.05	0.01
10	->	26	-0.07	0.03
10	->	27	2.79	1.32
10	->	28	-0.01	0.00
10	->	29	-0.01	0.00
10	->	30	-0.00	0.00
10	->	31	-0.03	0.00
10	->	32	-0.00	0.00
10	->	33	0.00	0.00
10	->	34	-0.00	0.00
10	->	35	0.01	0.00
10	->	36	-0.00	0.00
10	->	37	0.00	0.00
10	->	38	0.01	0.00
10	->	39	0.00	0.00
10	->	40	-0.01	0.00
10	->	41	-0.00	0.00
10	->	42	-0.00	0.00

10 ->	43	-0.00	0.00
10 ->	44	-0.03	0.01
10 ->	45	-0.00	0.00
10 ->	46	0.02	0.00
10 ->	47	-0.00	0.00
10 ->	48	-0.00	0.00
10 ->	49	-0.00	0.00
10 ->	50	-0.00	0.00
10 ->	51	-0.00	0.00
10 ->	52	-0.00	0.00
10 ->	53	-0.01	0.00
10 ->	54	-0.00	0.00
10 ->	55	0.00	0.00
10 ->	56	-0.00	0.00
10 ->	57	-0.00	0.00
10 ->	58	0.00	0.00
10 ->	59	-0.00	0.00
10 ->	60	-0.00	0.00
10 ->	61	-0.00	0.00
10 ->	62	0.00	0.00
10 ->	63	0.00	0.00
10 ->	64	0.00	0.00
10 ->	65	-0.00	0.00
10 ->	66	0.00	0.00
10 ->	67	0.00	0.00
10 ->	68	-0.00	0.00
10 ->	69	-0.00	0.00
10 ->	70	-0.00	0.00
10 ->	71	0.00	0.00
10 ->	72	-0.01	0.00
10 ->	73	-0.00	0.00
10 ->	74	0.01	0.00
10 ->	75	0.01	0.00
10 ->	76	-0.00	0.00
10 ->	77	-0.01	0.00
10 ->	78	-0.01	0.00
10 ->	79	-0.00	0.00
10 ->	80	-0.00	0.00
10 ->	81	-0.01	0.00
10 ->	82	-0.00	0.00
10 ->	83	-0.00	0.00
10 ->	84	0.00	0.00
10 ->	85	0.00	0.00
10 ->	86	0.00	0.00
10 ->	87	0.01	0.00
10 ->	88	0.01	0.00
10 ->	89	0.03	0.00
10 ->	90	0.15	0.07
10 ->	91	0.37	0.18
10 ->	92	0.06	0.01

10	->	93	0.02	0.00
10	->	94	0.01	0.00
10	->	95	0.01	0.00
10	->	96	0.00	0.00
10	->	97	0.01	0.00
10	->	98	0.01	0.00
10	->	99	0.01	0.00
10	->	100	0.01	0.00
10	->	101	0.01	0.00
10	->	102	0.01	0.00
10	->	103	0.02	0.00
10	->	104	0.02	0.00
10	->	105	0.02	0.01
10	->	106	0.01	0.00
11	->	1	-0.14	0.04
11	->	2	-0.00	0.00
11	->	3	-0.02	0.02
11	->	4	-0.27	0.36
11	->	5	-0.83	0.72
11	->	6	-0.19	0.27
11	->	7	-0.48	0.41
11	->	8	-0.55	0.51
11	->	9	-0.47	0.21
11	->	10	-13.97	0.66
11	->	11	-33.12	3.24
11	->	12	-8.63	0.71
11	->	13	-1.22	1.61
11	->	14	-0.07	0.03
11	->	15	-0.00	0.00
11	->	16	-0.03	0.00
11	->	17	0.00	0.00
11	->	18	0.00	0.00
11	->	19	0.03	0.00
11	->	20	-0.02	0.01
11	->	21	-0.00	0.00
11	->	22	-0.03	0.01
11	->	23	0.04	0.05
11	->	24	-0.01	0.00
11	->	25	-0.02	0.00
11	->	26	-0.01	0.00
11	->	27	0.14	0.11
11	->	28	-0.00	0.00
11	->	29	-0.00	0.00
11	->	30	-0.00	0.00
11	->	31	-0.01	0.00
11	->	32	-0.00	0.00
11	->	33	0.00	0.00
11	->	34	-0.00	0.00
11	->	35	0.00	0.00
11	->	36	-0.00	0.00

11	->	37	0.00	0.00
11	->	38	0.00	0.00
11	->	39	0.00	0.00
11	->	40	-0.01	0.00
11	->	41	-0.00	0.00
11	->	42	-0.00	0.00
11	->	43	-0.00	0.00
11	->	44	-0.01	0.00
11	->	45	-0.00	0.00
11	->	46	0.01	0.00
11	->	47	-0.00	0.00
11	->	48	-0.00	0.00
11	->	49	-0.00	0.00
11	->	50	-0.00	0.00
11	->	51	-0.00	0.00
11	->	52	-0.00	0.00
11	->	53	-0.00	0.00
11	->	54	-0.00	0.00
11	->	55	-0.00	0.00
11	->	56	-0.00	0.00
11	->	57	-0.00	0.00
11	->	58	0.00	0.00
11	->	59	-0.00	0.00
11	->	60	-0.00	0.00
11	->	61	-0.00	0.00
11	->	62	0.00	0.00
11	->	63	0.00	0.00
11	->	64	0.00	0.00
11	->	65	-0.00	0.00
11	->	66	0.00	0.00
11	->	67	-0.00	0.00
11	->	68	-0.00	0.00
11	->	69	-0.00	0.00
11	->	70	-0.00	0.00
11	->	71	0.00	0.00
11	->	72	-0.01	0.00
11	->	73	-0.00	0.00
11	->	74	0.01	0.00
11	->	75	0.00	0.00
11	->	76	-0.00	0.00
11	->	77	-0.01	0.00
11	->	78	-0.01	0.00
11	->	79	-0.00	0.00
11	->	80	-0.00	0.00
11	->	81	-0.01	0.00
11	->	82	-0.00	0.00
11	->	83	-0.00	0.00
11	->	84	0.00	0.00
11	->	85	0.00	0.00
11	->	86	0.00	0.00

11	->	87	0.00	0.00
11	->	88	0.00	0.00
11	->	89	0.01	0.00
11	->	90	0.02	0.01
11	->	91	0.04	0.01
11	->	92	0.04	0.01
11	->	93	0.02	0.00
11	->	94	0.01	0.00
11	->	95	0.01	0.00
11	->	96	0.00	0.00
11	->	97	0.01	0.00
11	->	98	0.00	0.00
11	->	99	0.00	0.00
11	->	100	0.00	0.00
11	->	101	0.01	0.00
11	->	102	0.01	0.00
11	->	103	0.01	0.00
11	->	104	0.01	0.01
11	->	105	0.01	0.01
11	->	106	0.01	0.00
12	->	1	0.65	0.82
12	->	2	-0.01	0.01
12	->	3	-0.23	0.06
12	->	4	-0.36	0.58
12	->	5	-0.91	0.89
12	->	6	-0.03	0.03
12	->	7	-0.05	0.03
12	->	8	-0.05	0.02
12	->	9	-0.04	0.04
12	->	10	-2.28	3.08
12	->	11	-8.71	0.69
12	->	12	-108.41	3.22
12	->	13	-9.79	0.64
12	->	14	-2.49	1.10
12	->	15	-0.13	0.06
12	->	16	0.07	0.04
12	->	17	-0.01	0.00
12	->	18	0.00	0.00
12	->	19	-0.15	0.11
12	->	20	-0.08	0.04
12	->	21	-0.00	0.00
12	->	22	0.12	0.10
12	->	23	-0.27	0.21
12	->	24	-0.02	0.01
12	->	25	0.02	0.00
12	->	26	-0.01	0.01
12	->	27	-0.10	0.13
12	->	28	0.00	0.00
12	->	29	0.00	0.00
12	->	30	-0.00	0.00

12	->	31	0.02	0.00
12	->	32	0.00	0.00
12	->	33	-0.00	0.00
12	->	34	0.00	0.00
12	->	35	-0.00	0.00
12	->	36	0.00	0.00
12	->	37	-0.00	0.00
12	->	38	-0.00	0.00
12	->	39	-0.00	0.00
12	->	40	0.01	0.00
12	->	41	0.00	0.00
12	->	42	0.00	0.00
12	->	43	0.00	0.00
12	->	44	0.03	0.01
12	->	45	0.00	0.00
12	->	46	-0.02	0.00
12	->	47	0.00	0.00
12	->	48	0.00	0.00
12	->	49	0.00	0.00
12	->	50	0.00	0.00
12	->	51	0.00	0.00
12	->	52	0.00	0.00
12	->	53	0.01	0.00
12	->	54	0.00	0.00
12	->	55	-0.00	0.00
12	->	56	0.00	0.00
12	->	57	0.00	0.00
12	->	58	-0.00	0.00
12	->	59	0.00	0.00
12	->	60	0.00	0.00
12	->	61	0.00	0.00
12	->	62	-0.00	0.00
12	->	63	-0.00	0.00
12	->	64	-0.00	0.00
12	->	65	0.00	0.00
12	->	66	-0.00	0.00
12	->	67	-0.00	0.00
12	->	68	0.00	0.00
12	->	69	0.00	0.00
12	->	70	0.00	0.00
12	->	71	0.00	0.00
12	->	72	0.02	0.00
12	->	73	0.00	0.00
12	->	74	-0.02	0.00
12	->	75	-0.01	0.00
12	->	76	0.01	0.00
12	->	77	0.02	0.00
12	->	78	0.01	0.00
12	->	79	0.00	0.00
12	->	80	0.00	0.00

12	->	81	0.01	0.01
12	->	82	0.00	0.00
12	->	83	0.00	0.00
12	->	84	-0.00	0.00
12	->	85	-0.00	0.00
12	->	86	-0.00	0.00
12	->	87	-0.01	0.00
12	->	88	-0.01	0.00
12	->	89	-0.03	0.01
12	->	90	-0.19	0.13
12	->	91	-3.06	1.82
12	->	92	-5.25	3.15
12	->	93	-0.10	0.06
12	->	94	-0.03	0.01
12	->	95	-0.01	0.00
12	->	96	-0.00	0.00
12	->	97	-0.01	0.00
12	->	98	-0.01	0.00
12	->	99	-0.01	0.00
12	->	100	-0.01	0.00
12	->	101	-0.02	0.01
12	->	102	-0.04	0.01
12	->	103	-0.04	0.01
12	->	104	-0.04	0.02
12	->	105	-0.03	0.01
12	->	106	-0.01	0.00
13	->	1	-4.72	1.73
13	->	2	-1.55	0.95
13	->	3	-2.99	0.67
13	->	4	-1.93	0.38
13	->	5	-0.68	0.32
13	->	6	-0.04	0.02
13	->	7	-0.01	0.01
13	->	8	-0.11	0.21
13	->	9	-0.01	0.01
13	->	10	-0.12	0.09
13	->	11	-1.26	1.63
13	->	12	-9.84	0.64
13	->	13	-108.38	2.93
13	->	14	-12.25	0.42
13	->	15	-0.63	0.10
13	->	16	0.03	0.03
13	->	17	-0.04	0.01
13	->	18	0.01	0.01
13	->	19	-0.06	0.03
13	->	20	-0.09	0.04
13	->	21	-0.01	0.01
13	->	22	0.06	0.03
13	->	23	-0.04	0.02
13	->	24	-0.01	0.00

13 ->	25	0.02	0.00
13 ->	26	-0.00	0.00
13 ->	27	-0.02	0.01
13 ->	28	0.00	0.00
13 ->	29	0.00	0.00
13 ->	30	-0.00	0.00
13 ->	31	0.01	0.00
13 ->	32	0.00	0.00
13 ->	33	0.00	0.00
13 ->	34	0.00	0.00
13 ->	35	-0.00	0.00
13 ->	36	0.00	0.00
13 ->	37	-0.00	0.00
13 ->	38	-0.00	0.00
13 ->	39	-0.00	0.00
13 ->	40	0.00	0.00
13 ->	41	0.00	0.00
13 ->	42	0.00	0.00
13 ->	43	0.00	0.00
13 ->	44	0.01	0.00
13 ->	45	0.00	0.00
13 ->	46	-0.01	0.00
13 ->	47	0.00	0.00
13 ->	48	0.00	0.00
13 ->	49	0.00	0.00
13 ->	50	0.00	0.00
13 ->	51	0.00	0.00
13 ->	52	0.00	0.00
13 ->	53	0.00	0.00
13 ->	54	0.00	0.00
13 ->	55	0.00	0.00
13 ->	56	0.00	0.00
13 ->	57	0.00	0.00
13 ->	58	-0.00	0.00
13 ->	59	0.00	0.00
13 ->	60	0.00	0.00
13 ->	61	0.00	0.00
13 ->	62	-0.00	0.00
13 ->	63	-0.00	0.00
13 ->	64	-0.00	0.00
13 ->	65	0.00	0.00
13 ->	66	-0.00	0.00
13 ->	67	0.00	0.00
13 ->	68	0.00	0.00
13 ->	69	0.00	0.00
13 ->	70	0.00	0.00
13 ->	71	-0.00	0.00
13 ->	72	0.00	0.00
13 ->	73	0.00	0.00
13 ->	74	-0.00	0.00

13	->	75	-0.00	0.00
13	->	76	0.00	0.00
13	->	77	0.00	0.00
13	->	78	0.00	0.00
13	->	79	0.00	0.00
13	->	80	0.00	0.00
13	->	81	0.00	0.00
13	->	82	0.00	0.00
13	->	83	0.00	0.00
13	->	84	-0.00	0.00
13	->	85	-0.00	0.00
13	->	86	-0.00	0.00
13	->	87	-0.00	0.00
13	->	88	-0.00	0.00
13	->	89	-0.01	0.00
13	->	90	-0.02	0.01
13	->	91	-0.08	0.04
13	->	92	-0.06	0.12
13	->	93	-0.06	0.03
13	->	94	-0.02	0.00
13	->	95	-0.01	0.00
13	->	96	-0.01	0.00
13	->	97	-0.01	0.00
13	->	98	-0.01	0.00
13	->	99	-0.01	0.00
13	->	100	-0.00	0.00
13	->	101	-0.01	0.00
13	->	102	-0.01	0.00
13	->	103	-0.01	0.00
13	->	104	-0.01	0.00
13	->	105	-0.01	0.00
13	->	106	-0.00	0.00
14	->	1	-3.74	1.15
14	->	2	-0.91	0.30
14	->	3	-3.42	0.86
14	->	4	-0.56	0.38
14	->	5	-0.59	0.32
14	->	6	-0.04	0.02
14	->	7	-0.04	0.02
14	->	8	-0.00	0.00
14	->	9	-0.01	0.00
14	->	10	-0.04	0.05
14	->	11	-0.07	0.03
14	->	12	-2.47	1.06
14	->	13	-12.29	0.41
14	->	14	29.13	1.69
14	->	15	-17.09	0.36
14	->	16	-0.81	0.28
14	->	17	-0.19	0.04
14	->	18	-0.07	0.02

14 ->	19	-1.89	0.36
14 ->	20	-2.33	0.51
14 ->	21	-0.07	0.02
14 ->	22	-0.46	0.38
14 ->	23	-2.08	0.37
14 ->	24	-0.06	0.02
14 ->	25	-0.02	0.01
14 ->	26	-0.03	0.01
14 ->	27	-0.01	0.02
14 ->	28	-0.00	0.00
14 ->	29	-0.00	0.00
14 ->	30	-0.00	0.00
14 ->	31	-0.00	0.00
14 ->	32	-0.00	0.00
14 ->	33	-0.00	0.00
14 ->	34	0.00	0.00
14 ->	35	-0.00	0.00
14 ->	36	0.00	0.00
14 ->	37	0.00	0.00
14 ->	38	-0.00	0.00
14 ->	39	0.00	0.00
14 ->	40	0.00	0.00
14 ->	41	0.00	0.00
14 ->	42	-0.00	0.00
14 ->	43	-0.00	0.00
14 ->	44	-0.01	0.00
14 ->	45	-0.00	0.00
14 ->	46	-0.00	0.00
14 ->	47	-0.00	0.00
14 ->	48	0.00	0.00
14 ->	49	0.00	0.00
14 ->	50	0.00	0.00
14 ->	51	0.00	0.00
14 ->	52	0.00	0.00
14 ->	53	0.00	0.00
14 ->	54	0.00	0.00
14 ->	55	0.00	0.00
14 ->	56	0.00	0.00
14 ->	57	0.00	0.00
14 ->	58	-0.00	0.00
14 ->	59	0.00	0.00
14 ->	60	0.00	0.00
14 ->	61	0.00	0.00
14 ->	62	-0.00	0.00
14 ->	63	-0.00	0.00
14 ->	64	-0.00	0.00
14 ->	65	0.00	0.00
14 ->	66	-0.00	0.00
14 ->	67	0.00	0.00
14 ->	68	0.00	0.00

14 ->	69	0.00	0.00
14 ->	70	0.00	0.00
14 ->	71	0.00	0.00
14 ->	72	0.00	0.00
14 ->	73	0.00	0.00
14 ->	74	-0.00	0.00
14 ->	75	-0.00	0.00
14 ->	76	0.00	0.00
14 ->	77	0.00	0.00
14 ->	78	0.00	0.00
14 ->	79	0.00	0.00
14 ->	80	0.00	0.00
14 ->	81	0.00	0.00
14 ->	82	0.00	0.00
14 ->	83	0.00	0.00
14 ->	84	-0.00	0.00
14 ->	85	-0.00	0.00
14 ->	86	-0.00	0.00
14 ->	87	-0.00	0.00
14 ->	88	-0.00	0.00
14 ->	89	-0.00	0.00
14 ->	90	-0.01	0.02
14 ->	91	-0.81	0.70
14 ->	92	-0.67	0.43
14 ->	93	-0.19	0.15
14 ->	94	-0.02	0.01
14 ->	95	-0.01	0.01
14 ->	96	-0.00	0.00
14 ->	97	-0.01	0.00
14 ->	98	-0.00	0.00
14 ->	99	-0.00	0.00
14 ->	100	-0.00	0.00
14 ->	101	-0.00	0.00
14 ->	102	-0.00	0.00
14 ->	103	-0.00	0.00
14 ->	104	-0.00	0.00
14 ->	105	-0.00	0.00
14 ->	106	-0.00	0.00
15 ->	1	-3.95	1.37
15 ->	2	-0.82	0.35
15 ->	3	-1.14	0.43
15 ->	4	-0.01	0.01
15 ->	5	0.02	0.01
15 ->	6	-0.00	0.00
15 ->	7	-0.00	0.00
15 ->	8	0.00	0.00
15 ->	9	-0.00	0.00
15 ->	10	-0.00	0.00
15 ->	11	-0.00	0.00
15 ->	12	-0.13	0.06

15	->	13	-0.63	0.10
15	->	14	-17.07	0.37
15	->	15	20.13	2.48
15	->	16	-6.74	0.79
15	->	17	-0.30	0.08
15	->	18	-0.02	0.01
15	->	19	-0.40	0.33
15	->	20	-0.06	0.02
15	->	21	-0.01	0.00
15	->	22	-0.02	0.02
15	->	23	-0.01	0.00
15	->	24	-0.00	0.00
15	->	25	-0.00	0.00
15	->	26	-0.00	0.00
15	->	27	0.00	0.00
15	->	28	0.00	0.00
15	->	29	0.00	0.00
15	->	30	-0.00	0.00
15	->	31	0.00	0.00
15	->	32	0.00	0.00
15	->	33	0.00	0.00
15	->	34	0.00	0.00
15	->	35	-0.00	0.00
15	->	36	0.00	0.00
15	->	37	0.00	0.00
15	->	38	-0.00	0.00
15	->	39	0.00	0.00
15	->	40	0.00	0.00
15	->	41	-0.00	0.00
15	->	42	0.00	0.00
15	->	43	-0.00	0.00
15	->	44	0.00	0.00
15	->	45	0.00	0.00
15	->	46	-0.00	0.00
15	->	47	0.00	0.00
15	->	48	0.00	0.00
15	->	49	0.00	0.00
15	->	50	0.00	0.00
15	->	51	0.00	0.00
15	->	52	0.00	0.00
15	->	53	0.00	0.00
15	->	54	0.00	0.00
15	->	55	0.00	0.00
15	->	56	0.00	0.00
15	->	57	0.00	0.00
15	->	58	-0.00	0.00
15	->	59	0.00	0.00
15	->	60	0.00	0.00
15	->	61	0.00	0.00
15	->	62	-0.00	0.00

15	->	63	-0.00	0.00
15	->	64	-0.00	0.00
15	->	65	0.00	0.00
15	->	66	-0.00	0.00
15	->	67	0.00	0.00
15	->	68	0.00	0.00
15	->	69	0.00	0.00
15	->	70	0.00	0.00
15	->	71	-0.00	0.00
15	->	72	0.00	0.00
15	->	73	0.00	0.00
15	->	74	-0.00	0.00
15	->	75	-0.00	0.00
15	->	76	0.00	0.00
15	->	77	0.00	0.00
15	->	78	0.00	0.00
15	->	79	0.00	0.00
15	->	80	0.00	0.00
15	->	81	0.00	0.00
15	->	82	0.00	0.00
15	->	83	0.00	0.00
15	->	84	-0.00	0.00
15	->	85	-0.00	0.00
15	->	86	-0.00	0.00
15	->	87	-0.00	0.00
15	->	88	-0.00	0.00
15	->	89	-0.00	0.00
15	->	90	-0.00	0.00
15	->	91	0.01	0.02
15	->	92	-0.08	0.19
15	->	93	-1.30	2.15
15	->	94	-0.06	0.04
15	->	95	-0.04	0.04
15	->	96	-0.01	0.01
15	->	97	-0.00	0.01
15	->	98	-0.00	0.00
15	->	99	-0.00	0.00
15	->	100	-0.00	0.00
15	->	101	-0.00	0.00
15	->	102	-0.00	0.00
15	->	103	-0.00	0.00
15	->	104	-0.00	0.00
15	->	105	-0.00	0.00
15	->	106	-0.00	0.00
16	->	1	0.26	0.34
16	->	2	-0.11	0.08
16	->	3	-2.07	1.01
16	->	4	-0.02	0.01
16	->	5	-0.04	0.01
16	->	6	-0.00	0.00

16	->	7	0.00	0.00
16	->	8	-0.01	0.00
16	->	9	-0.00	0.00
16	->	10	-0.03	0.00
16	->	11	-0.03	0.00
16	->	12	0.07	0.04
16	->	13	0.03	0.03
16	->	14	-0.82	0.28
16	->	15	-6.89	0.81
16	->	16	-97.66	3.24
16	->	17	-21.48	0.46
16	->	18	-0.82	0.25
16	->	19	-12.46	2.24
16	->	20	-1.52	0.41
16	->	21	-0.04	0.04
16	->	22	0.33	0.23
16	->	23	-0.01	0.01
16	->	24	-0.01	0.00
16	->	25	0.04	0.01
16	->	26	-0.00	0.00
16	->	27	-0.00	0.00
16	->	28	0.00	0.00
16	->	29	0.00	0.00
16	->	30	-0.00	0.00
16	->	31	0.01	0.00
16	->	32	0.00	0.00
16	->	33	-0.00	0.00
16	->	34	-0.00	0.00
16	->	35	-0.01	0.00
16	->	36	0.01	0.00
16	->	37	-0.00	0.00
16	->	38	-0.00	0.00
16	->	39	-0.00	0.00
16	->	40	0.01	0.00
16	->	41	0.00	0.00
16	->	42	0.00	0.00
16	->	43	-0.00	0.00
16	->	44	0.07	0.01
16	->	45	-0.00	0.00
16	->	46	-0.04	0.00
16	->	47	-0.00	0.00
16	->	48	0.00	0.00
16	->	49	0.00	0.00
16	->	50	0.00	0.00
16	->	51	0.00	0.00
16	->	52	0.00	0.00
16	->	53	0.01	0.00
16	->	54	0.00	0.00
16	->	55	0.00	0.00
16	->	56	0.00	0.00

16	->	57	0.00	0.00
16	->	58	-0.01	0.00
16	->	59	0.01	0.00
16	->	60	0.00	0.00
16	->	61	0.00	0.00
16	->	62	-0.00	0.00
16	->	63	-0.00	0.00
16	->	64	-0.00	0.00
16	->	65	0.00	0.00
16	->	66	-0.00	0.00
16	->	67	0.00	0.00
16	->	68	0.00	0.00
16	->	69	0.00	0.00
16	->	70	0.00	0.00
16	->	71	-0.00	0.00
16	->	72	0.02	0.00
16	->	73	0.00	0.00
16	->	74	-0.02	0.00
16	->	75	-0.01	0.00
16	->	76	0.01	0.00
16	->	77	0.02	0.00
16	->	78	0.01	0.00
16	->	79	0.00	0.00
16	->	80	0.00	0.00
16	->	81	0.01	0.00
16	->	82	0.00	0.00
16	->	83	0.00	0.00
16	->	84	-0.00	0.00
16	->	85	-0.00	0.00
16	->	86	-0.00	0.00
16	->	87	-0.01	0.00
16	->	88	-0.01	0.00
16	->	89	-0.02	0.00
16	->	90	-0.04	0.01
16	->	91	-0.20	0.08
16	->	92	-2.43	0.42
16	->	93	-11.74	1.55
16	->	94	-2.42	1.42
16	->	95	-1.39	0.99
16	->	96	-0.08	0.09
16	->	97	-0.11	0.29
16	->	98	-0.04	0.03
16	->	99	-0.02	0.03
16	->	100	-0.05	0.01
16	->	101	-0.06	0.01
16	->	102	-0.05	0.01
16	->	103	-0.03	0.00
16	->	104	-0.02	0.00
16	->	105	-0.01	0.00
16	->	106	-0.00	0.00

17 ->	1	-0.10	0.33
17 ->	2	-0.04	0.02
17 ->	3	-1.80	0.85
17 ->	4	-0.01	0.01
17 ->	5	-0.00	0.00
17 ->	6	-0.00	0.00
17 ->	7	-0.00	0.00
17 ->	8	0.00	0.00
17 ->	9	-0.00	0.00
17 ->	10	0.00	0.00
17 ->	11	0.00	0.00
17 ->	12	-0.01	0.00
17 ->	13	-0.04	0.01
17 ->	14	-0.19	0.04
17 ->	15	-0.30	0.08
17 ->	16	-21.48	0.46
17 ->	17	19.88	1.32
17 ->	18	-9.20	0.45
17 ->	19	-0.73	0.13
17 ->	20	-2.23	0.34
17 ->	21	-4.00	1.17
17 ->	22	-0.16	0.08
17 ->	23	-0.07	0.03
17 ->	24	-0.06	0.02
17 ->	25	-0.03	0.01
17 ->	26	-0.00	0.00
17 ->	27	-0.00	0.00
17 ->	28	-0.00	0.00
17 ->	29	-0.00	0.00
17 ->	30	-0.00	0.00
17 ->	31	-0.00	0.00
17 ->	32	-0.00	0.00
17 ->	33	-0.00	0.00
17 ->	34	-0.00	0.00
17 ->	35	0.00	0.00
17 ->	36	-0.00	0.00
17 ->	37	-0.00	0.00
17 ->	38	0.00	0.00
17 ->	39	0.00	0.00
17 ->	40	-0.00	0.00
17 ->	41	-0.00	0.00
17 ->	42	0.00	0.00
17 ->	43	-0.00	0.00
17 ->	44	-0.00	0.00
17 ->	45	-0.00	0.00
17 ->	46	0.00	0.00
17 ->	47	-0.00	0.00
17 ->	48	-0.00	0.00
17 ->	49	-0.00	0.00
17 ->	50	-0.00	0.00

17	->	51	-0.00	0.00
17	->	52	-0.00	0.00
17	->	53	-0.00	0.00
17	->	54	-0.00	0.00
17	->	55	0.00	0.00
17	->	56	-0.00	0.00
17	->	57	0.00	0.00
17	->	58	0.00	0.00
17	->	59	-0.00	0.00
17	->	60	0.00	0.00
17	->	61	0.00	0.00
17	->	62	0.00	0.00
17	->	63	0.00	0.00
17	->	64	0.00	0.00
17	->	65	0.00	0.00
17	->	66	0.00	0.00
17	->	67	0.00	0.00
17	->	68	-0.00	0.00
17	->	69	-0.00	0.00
17	->	70	0.00	0.00
17	->	71	0.00	0.00
17	->	72	-0.00	0.00
17	->	73	-0.00	0.00
17	->	74	0.00	0.00
17	->	75	0.00	0.00
17	->	76	-0.00	0.00
17	->	77	-0.00	0.00
17	->	78	-0.00	0.00
17	->	79	-0.00	0.00
17	->	80	0.00	0.00
17	->	81	-0.00	0.00
17	->	82	-0.00	0.00
17	->	83	-0.00	0.00
17	->	84	0.00	0.00
17	->	85	0.00	0.00
17	->	86	0.00	0.00
17	->	87	0.00	0.00
17	->	88	0.00	0.00
17	->	89	0.00	0.00
17	->	90	0.00	0.00
17	->	91	-0.00	0.00
17	->	92	-0.01	0.01
17	->	93	-0.01	0.01
17	->	94	-0.03	0.02
17	->	95	-0.12	0.11
17	->	96	-0.31	0.50
17	->	97	-0.37	0.72
17	->	98	-0.02	0.02
17	->	99	-0.01	0.01
17	->	100	-0.00	0.00

17	->	101	-0.00	0.00
17	->	102	-0.00	0.00
17	->	103	0.00	0.00
17	->	104	0.00	0.00
17	->	105	0.00	0.00
17	->	106	0.00	0.00
18	->	1	0.01	0.01
18	->	2	-0.01	0.00
18	->	3	-0.04	0.02
18	->	4	-0.00	0.00
18	->	5	-0.01	0.00
18	->	6	-0.00	0.00
18	->	7	0.00	0.00
18	->	8	0.00	0.00
18	->	9	-0.00	0.00
18	->	10	0.00	0.00
18	->	11	0.00	0.00
18	->	12	0.00	0.00
18	->	13	0.01	0.01
18	->	14	-0.07	0.02
18	->	15	-0.02	0.01
18	->	16	-0.81	0.25
18	->	17	-9.23	0.45
18	->	18	23.45	0.87
18	->	19	-14.46	0.42
18	->	20	-0.94	0.17
18	->	21	-2.13	0.50
18	->	22	-2.58	0.76
18	->	23	-0.12	0.08
18	->	24	-0.04	0.03
18	->	25	-0.10	0.07
18	->	26	-0.01	0.00
18	->	27	-0.01	0.00
18	->	28	-0.00	0.00
18	->	29	-0.00	0.00
18	->	30	-0.00	0.00
18	->	31	-0.00	0.00
18	->	32	-0.00	0.00
18	->	33	-0.01	0.00
18	->	34	-0.00	0.00
18	->	35	0.00	0.00
18	->	36	-0.00	0.00
18	->	37	0.00	0.00
18	->	38	0.00	0.00
18	->	39	0.00	0.00
18	->	40	-0.00	0.00
18	->	41	0.00	0.00
18	->	42	0.00	0.00
18	->	43	-0.00	0.00
18	->	44	-0.00	0.02

18	->	45	-0.01	0.00
18	->	46	-0.01	0.01
18	->	47	-0.01	0.00
18	->	48	-0.00	0.00
18	->	49	-0.00	0.00
18	->	50	-0.00	0.00
18	->	51	-0.00	0.00
18	->	52	-0.00	0.00
18	->	53	-0.00	0.00
18	->	54	-0.00	0.00
18	->	55	0.00	0.00
18	->	56	-0.00	0.00
18	->	57	-0.00	0.00
18	->	58	0.00	0.00
18	->	59	-0.00	0.00
18	->	60	0.00	0.00
18	->	61	0.00	0.00
18	->	62	0.00	0.00
18	->	63	-0.00	0.00
18	->	64	-0.00	0.00
18	->	65	0.00	0.00
18	->	66	0.00	0.00
18	->	67	0.00	0.00
18	->	68	-0.00	0.00
18	->	69	0.00	0.00
18	->	70	-0.00	0.00
18	->	71	0.00	0.00
18	->	72	-0.00	0.00
18	->	73	0.00	0.00
18	->	74	0.00	0.00
18	->	75	0.00	0.00
18	->	76	0.00	0.00
18	->	77	0.00	0.00
18	->	78	-0.00	0.00
18	->	79	0.00	0.00
18	->	80	0.00	0.00
18	->	81	-0.00	0.00
18	->	82	0.00	0.00
18	->	83	0.00	0.00
18	->	84	0.00	0.00
18	->	85	0.00	0.00
18	->	86	0.00	0.00
18	->	87	0.00	0.00
18	->	88	0.00	0.00
18	->	89	-0.00	0.00
18	->	90	-0.01	0.01
18	->	91	-0.05	0.02
18	->	92	-0.09	0.04
18	->	93	-0.17	0.17
18	->	94	-0.38	0.30

18 ->	95	-0.52	0.44
18 ->	96	-0.33	0.46
18 ->	97	-0.53	0.64
18 ->	98	-0.38	0.28
18 ->	99	-0.06	0.07
18 ->	100	-0.01	0.00
18 ->	101	-0.01	0.00
18 ->	102	-0.00	0.00
18 ->	103	-0.00	0.00
18 ->	104	0.00	0.00
18 ->	105	0.00	0.00
18 ->	106	0.00	0.00
19 ->	1	-0.33	0.24
19 ->	2	-0.01	0.00
19 ->	3	-0.08	0.04
19 ->	4	-0.01	0.01
19 ->	5	0.01	0.01
19 ->	6	-0.01	0.00
19 ->	7	0.00	0.00
19 ->	8	0.01	0.00
19 ->	9	-0.00	0.00
19 ->	10	0.04	0.01
19 ->	11	0.03	0.00
19 ->	12	-0.14	0.11
19 ->	13	-0.06	0.03
19 ->	14	-1.90	0.35
19 ->	15	-0.40	0.32
19 ->	16	-12.35	2.21
19 ->	17	-0.73	0.13
19 ->	18	-14.52	0.43
19 ->	19	-12.40	2.55
19 ->	20	-8.10	0.47
19 ->	21	-1.22	0.26
19 ->	22	-6.38	3.82
19 ->	23	-2.88	0.53
19 ->	24	-0.13	0.06
19 ->	25	-0.16	0.04
19 ->	26	-0.04	0.02
19 ->	27	-0.02	0.00
19 ->	28	-0.01	0.00
19 ->	29	-0.00	0.00
19 ->	30	0.00	0.00
19 ->	31	-0.03	0.00
19 ->	32	-0.00	0.00
19 ->	33	-0.00	0.00
19 ->	34	0.00	0.00
19 ->	35	0.01	0.00
19 ->	36	-0.01	0.00
19 ->	37	0.00	0.00
19 ->	38	0.01	0.00

19	->	39	0.00	0.00
19	->	40	-0.01	0.00
19	->	41	-0.00	0.00
19	->	42	-0.00	0.00
19	->	43	-0.01	0.00
19	->	44	-0.20	0.06
19	->	45	-0.00	0.00
19	->	46	0.06	0.01
19	->	47	-0.01	0.00
19	->	48	-0.00	0.00
19	->	49	-0.00	0.00
19	->	50	-0.00	0.00
19	->	51	-0.00	0.00
19	->	52	-0.00	0.00
19	->	53	-0.02	0.00
19	->	54	-0.00	0.00
19	->	55	-0.00	0.00
19	->	56	-0.00	0.00
19	->	57	-0.00	0.00
19	->	58	0.01	0.00
19	->	59	-0.01	0.00
19	->	60	-0.00	0.00
19	->	61	-0.00	0.00
19	->	62	0.00	0.00
19	->	63	0.00	0.00
19	->	64	0.00	0.00
19	->	65	-0.00	0.00
19	->	66	0.00	0.00
19	->	67	-0.00	0.00
19	->	68	-0.00	0.00
19	->	69	-0.00	0.00
19	->	70	-0.00	0.00
19	->	71	0.00	0.00
19	->	72	-0.02	0.00
19	->	73	-0.00	0.00
19	->	74	0.02	0.00
19	->	75	0.01	0.00
19	->	76	-0.01	0.00
19	->	77	-0.02	0.00
19	->	78	-0.01	0.00
19	->	79	-0.00	0.00
19	->	80	-0.00	0.00
19	->	81	-0.01	0.00
19	->	82	-0.00	0.00
19	->	83	-0.00	0.00
19	->	84	0.00	0.00
19	->	85	0.00	0.00
19	->	86	0.01	0.00
19	->	87	0.01	0.00
19	->	88	0.01	0.00

19	->	89	0.03	0.00
19	->	90	0.03	0.04
19	->	91	-0.07	0.22
19	->	92	-0.65	0.48
19	->	93	-1.15	1.37
19	->	94	0.25	0.19
19	->	95	0.01	0.04
19	->	96	0.00	0.02
19	->	97	0.00	0.04
19	->	98	-0.07	0.12
19	->	99	0.02	0.05
19	->	100	0.02	0.01
19	->	101	0.04	0.01
19	->	102	0.03	0.00
19	->	103	0.02	0.00
19	->	104	0.02	0.00
19	->	105	0.01	0.00
19	->	106	0.01	0.00
20	->	1	-0.05	0.03
20	->	2	-0.04	0.02
20	->	3	-1.69	0.43
20	->	4	-1.11	0.35
20	->	5	-0.91	0.23
20	->	6	-0.39	0.22
20	->	7	-0.03	0.01
20	->	8	-0.00	0.00
20	->	9	-0.00	0.00
20	->	10	-0.01	0.01
20	->	11	-0.02	0.01
20	->	12	-0.08	0.04
20	->	13	-0.09	0.04
20	->	14	-2.38	0.53
20	->	15	-0.06	0.02
20	->	16	-1.54	0.41
20	->	17	-2.24	0.34
20	->	18	-0.95	0.17
20	->	19	-8.14	0.47
20	->	20	22.21	1.33
20	->	21	-6.38	0.57
20	->	22	-1.51	0.26
20	->	23	-2.85	0.47
20	->	24	-4.23	0.56
20	->	25	-0.17	0.07
20	->	26	-0.07	0.03
20	->	27	-0.08	0.03
20	->	28	-0.02	0.01
20	->	29	-0.00	0.00
20	->	30	-0.01	0.00
20	->	31	-0.00	0.00
20	->	32	-0.00	0.00

20 ->	33	-0.00	0.00
20 ->	34	-0.00	0.00
20 ->	35	0.00	0.00
20 ->	36	-0.00	0.00
20 ->	37	0.00	0.00
20 ->	38	0.00	0.00
20 ->	39	0.00	0.00
20 ->	40	-0.00	0.00
20 ->	41	0.00	0.00
20 ->	42	0.00	0.00
20 ->	43	-0.01	0.00
20 ->	44	-0.01	0.01
20 ->	45	-0.00	0.00
20 ->	46	-0.00	0.00
20 ->	47	-0.00	0.00
20 ->	48	-0.00	0.00
20 ->	49	-0.00	0.00
20 ->	50	-0.00	0.00
20 ->	51	-0.00	0.00
20 ->	52	-0.00	0.00
20 ->	53	-0.00	0.00
20 ->	54	-0.00	0.00
20 ->	55	0.00	0.00
20 ->	56	0.00	0.00
20 ->	57	-0.00	0.00
20 ->	58	0.00	0.00
20 ->	59	-0.00	0.00
20 ->	60	0.00	0.00
20 ->	61	0.00	0.00
20 ->	62	-0.00	0.00
20 ->	63	0.00	0.00
20 ->	64	0.00	0.00
20 ->	65	0.00	0.00
20 ->	66	0.00	0.00
20 ->	67	0.00	0.00
20 ->	68	-0.00	0.00
20 ->	69	-0.00	0.00
20 ->	70	-0.00	0.00
20 ->	71	-0.00	0.00
20 ->	72	-0.00	0.00
20 ->	73	0.00	0.00
20 ->	74	0.00	0.00
20 ->	75	0.00	0.00
20 ->	76	-0.00	0.00
20 ->	77	-0.00	0.00
20 ->	78	-0.00	0.00
20 ->	79	-0.00	0.00
20 ->	80	0.00	0.00
20 ->	81	-0.00	0.00
20 ->	82	0.00	0.00

20	->	83	-0.00	0.00
20	->	84	0.00	0.00
20	->	85	0.00	0.00
20	->	86	0.00	0.00
20	->	87	0.00	0.00
20	->	88	-0.00	0.00
20	->	89	-0.00	0.00
20	->	90	-0.00	0.01
20	->	91	0.04	0.06
20	->	92	0.02	0.02
20	->	93	-0.01	0.01
20	->	94	-0.01	0.00
20	->	95	-0.01	0.00
20	->	96	-0.02	0.02
20	->	97	-0.02	0.02
20	->	98	-0.01	0.00
20	->	99	-0.00	0.00
20	->	100	-0.00	0.00
20	->	101	-0.00	0.00
20	->	102	-0.00	0.00
20	->	103	-0.00	0.00
20	->	104	0.00	0.00
20	->	105	0.00	0.00
20	->	106	0.00	0.00
21	->	1	0.00	0.00
21	->	2	-0.00	0.00
21	->	3	-0.05	0.02
21	->	4	-0.01	0.00
21	->	5	-0.00	0.02
21	->	6	-0.02	0.01
21	->	7	-0.01	0.01
21	->	8	0.00	0.00
21	->	9	-0.00	0.00
21	->	10	-0.00	0.00
21	->	11	-0.00	0.00
21	->	12	-0.00	0.00
21	->	13	-0.01	0.01
21	->	14	-0.07	0.02
21	->	15	-0.01	0.00
21	->	16	-0.04	0.04
21	->	17	-4.00	1.17
21	->	18	-2.16	0.51
21	->	19	-1.23	0.26
21	->	20	-6.39	0.57
21	->	21	-20.60	1.94
21	->	22	-10.52	0.53
21	->	23	-0.80	0.15
21	->	24	-2.68	0.47
21	->	25	-3.04	1.01
21	->	26	-0.06	0.05

21 ->	27	-0.05	0.02
21 ->	28	-0.02	0.01
21 ->	29	-0.01	0.00
21 ->	30	-0.05	0.03
21 ->	31	-0.00	0.00
21 ->	32	-0.02	0.01
21 ->	33	-0.02	0.01
21 ->	34	-0.00	0.00
21 ->	35	0.00	0.00
21 ->	36	-0.00	0.00
21 ->	37	0.00	0.00
21 ->	38	0.00	0.00
21 ->	39	0.00	0.00
21 ->	40	-0.00	0.00
21 ->	41	0.00	0.00
21 ->	42	0.00	0.00
21 ->	43	-0.03	0.02
21 ->	44	0.03	0.02
21 ->	45	-0.01	0.00
21 ->	46	-0.02	0.01
21 ->	47	-0.01	0.00
21 ->	48	-0.00	0.00
21 ->	49	-0.00	0.00
21 ->	50	-0.00	0.00
21 ->	51	-0.00	0.00
21 ->	52	-0.00	0.00
21 ->	53	0.00	0.00
21 ->	54	0.00	0.00
21 ->	55	0.00	0.00
21 ->	56	0.00	0.00
21 ->	57	0.00	0.00
21 ->	58	-0.00	0.00
21 ->	59	-0.00	0.00
21 ->	60	0.00	0.00
21 ->	61	0.00	0.00
21 ->	62	0.00	0.00
21 ->	63	-0.00	0.00
21 ->	64	-0.00	0.00
21 ->	65	0.00	0.00
21 ->	66	-0.00	0.00
21 ->	67	0.00	0.00
21 ->	68	0.00	0.00
21 ->	69	0.00	0.00
21 ->	70	0.00	0.00
21 ->	71	0.00	0.00
21 ->	72	0.00	0.00
21 ->	73	0.00	0.00
21 ->	74	-0.00	0.00
21 ->	75	-0.00	0.00
21 ->	76	0.00	0.00

21	->	77	0.00	0.00
21	->	78	-0.00	0.00
21	->	79	0.00	0.00
21	->	80	0.00	0.00
21	->	81	0.00	0.00
21	->	82	0.00	0.00
21	->	83	0.00	0.00
21	->	84	-0.00	0.00
21	->	85	0.00	0.00
21	->	86	0.00	0.00
21	->	87	0.00	0.00
21	->	88	-0.00	0.00
21	->	89	-0.01	0.01
21	->	90	-0.03	0.02
21	->	91	-0.08	0.05
21	->	92	-0.02	0.01
21	->	93	-0.01	0.00
21	->	94	-0.01	0.01
21	->	95	-0.01	0.01
21	->	96	-0.17	0.33
21	->	97	-0.10	0.13
21	->	98	-0.03	0.02
21	->	99	-0.01	0.00
21	->	100	-0.00	0.00
21	->	101	-0.00	0.00
21	->	102	-0.00	0.00
21	->	103	-0.00	0.00
21	->	104	-0.00	0.00
21	->	105	0.00	0.00
21	->	106	0.00	0.00
22	->	1	0.17	0.14
22	->	2	-0.00	0.00
22	->	3	-0.01	0.01
22	->	4	0.01	0.01
22	->	5	-0.16	0.03
22	->	6	-0.01	0.00
22	->	7	0.02	0.01
22	->	8	-0.01	0.00
22	->	9	-0.00	0.00
22	->	10	-0.10	0.03
22	->	11	-0.03	0.01
22	->	12	0.12	0.10
22	->	13	0.06	0.03
22	->	14	-0.47	0.39
22	->	15	-0.02	0.02
22	->	16	0.33	0.23
22	->	17	-0.16	0.08
22	->	18	-2.62	0.74
22	->	19	-6.42	3.81
22	->	20	-1.52	0.26

22	->	21	-10.55	0.54
22	->	22	-96.84	3.68
22	->	23	-19.31	0.49
22	->	24	-0.70	0.18
22	->	25	-2.84	0.64
22	->	26	-1.77	0.93
22	->	27	-0.14	0.07
22	->	28	-0.03	0.02
22	->	29	-0.00	0.01
22	->	30	-0.04	0.01
22	->	31	0.03	0.02
22	->	32	-0.02	0.01
22	->	33	-0.04	0.02
22	->	34	-0.00	0.00
22	->	35	-0.02	0.00
22	->	36	0.01	0.00
22	->	37	-0.00	0.00
22	->	38	-0.01	0.00
22	->	39	-0.00	0.00
22	->	40	0.02	0.00
22	->	41	0.00	0.00
22	->	42	0.01	0.01
22	->	43	-0.14	0.10
22	->	44	-0.24	0.81
22	->	45	-0.07	0.06
22	->	46	-0.89	0.60
22	->	47	-0.12	0.05
22	->	48	-0.01	0.00
22	->	49	0.00	0.00
22	->	50	-0.00	0.00
22	->	51	0.00	0.00
22	->	52	0.00	0.00
22	->	53	0.03	0.01
22	->	54	0.00	0.00
22	->	55	0.00	0.00
22	->	56	0.00	0.00
22	->	57	0.00	0.00
22	->	58	-0.01	0.00
22	->	59	0.01	0.00
22	->	60	0.00	0.00
22	->	61	-0.00	0.00
22	->	62	-0.00	0.00
22	->	63	-0.01	0.00
22	->	64	-0.00	0.00
22	->	65	0.00	0.00
22	->	66	-0.00	0.00
22	->	67	-0.00	0.00
22	->	68	0.01	0.00
22	->	69	0.01	0.00
22	->	70	0.00	0.00

22	->	71	-0.00	0.00
22	->	72	0.04	0.01
22	->	73	0.00	0.00
22	->	74	-0.04	0.01
22	->	75	-0.02	0.00
22	->	76	0.01	0.00
22	->	77	0.02	0.00
22	->	78	0.02	0.00
22	->	79	0.00	0.00
22	->	80	0.00	0.00
22	->	81	0.01	0.00
22	->	82	0.00	0.00
22	->	83	0.00	0.00
22	->	84	-0.00	0.00
22	->	85	-0.00	0.00
22	->	86	-0.01	0.00
22	->	87	-0.02	0.00
22	->	88	-0.03	0.01
22	->	89	-0.16	0.07
22	->	90	-1.51	1.31
22	->	91	-7.60	6.14
22	->	92	-5.46	2.59
22	->	93	-0.26	0.39
22	->	94	-0.10	0.07
22	->	95	-0.04	0.01
22	->	96	-0.03	0.03
22	->	97	-0.09	0.05
22	->	98	-0.39	0.43
22	->	99	-2.19	2.44
22	->	100	0.20	0.24
22	->	101	-0.11	0.09
22	->	102	-0.07	0.03
22	->	103	-0.04	0.01
22	->	104	-0.03	0.01
22	->	105	-0.02	0.00
22	->	106	-0.01	0.00
23	->	1	-0.01	0.01
23	->	2	-0.00	0.00
23	->	3	-0.02	0.01
23	->	4	-0.07	0.03
23	->	5	1.54	0.49
23	->	6	-0.14	0.08
23	->	7	-0.35	0.17
23	->	8	-0.00	0.01
23	->	9	-0.03	0.03
23	->	10	2.09	0.66
23	->	11	0.04	0.05
23	->	12	-0.27	0.21
23	->	13	-0.04	0.02
23	->	14	-2.11	0.37

23	->	15	-0.01	0.00
23	->	16	-0.01	0.01
23	->	17	-0.07	0.03
23	->	18	-0.12	0.08
23	->	19	-2.90	0.53
23	->	20	-2.86	0.47
23	->	21	-0.81	0.15
23	->	22	-19.36	0.50
23	->	23	12.29	1.18
23	->	24	-10.05	0.57
23	->	25	-1.25	0.27
23	->	26	-2.95	0.85
23	->	27	22.42	13.39
23	->	28	-0.23	0.07
23	->	29	-0.04	0.03
23	->	30	-0.03	0.01
23	->	31	-0.01	0.00
23	->	32	-0.01	0.00
23	->	33	-0.00	0.00
23	->	34	-0.00	0.00
23	->	35	-0.00	0.00
23	->	36	0.00	0.00
23	->	37	0.00	0.00
23	->	38	-0.00	0.00
23	->	39	0.00	0.00
23	->	40	-0.00	0.00
23	->	41	-0.00	0.00
23	->	42	-0.00	0.00
23	->	43	-0.01	0.01
23	->	44	-0.05	0.02
23	->	45	0.00	0.00
23	->	46	-0.00	0.00
23	->	47	-0.00	0.00
23	->	48	-0.00	0.00
23	->	49	0.00	0.00
23	->	50	0.00	0.00
23	->	51	0.00	0.00
23	->	52	0.00	0.00
23	->	53	0.00	0.00
23	->	54	0.00	0.00
23	->	55	0.00	0.00
23	->	56	0.00	0.00
23	->	57	0.00	0.00
23	->	58	-0.00	0.00
23	->	59	0.00	0.00
23	->	60	0.00	0.00
23	->	61	0.00	0.00
23	->	62	-0.00	0.00
23	->	63	-0.00	0.00
23	->	64	-0.00	0.00

23	->	65	0.00	0.00
23	->	66	-0.00	0.00
23	->	67	0.00	0.00
23	->	68	0.00	0.00
23	->	69	0.00	0.00
23	->	70	0.00	0.00
23	->	71	-0.00	0.00
23	->	72	0.00	0.00
23	->	73	0.00	0.00
23	->	74	-0.00	0.00
23	->	75	-0.00	0.00
23	->	76	0.00	0.00
23	->	77	0.00	0.00
23	->	78	0.00	0.00
23	->	79	0.00	0.00
23	->	80	0.00	0.00
23	->	81	0.00	0.00
23	->	82	0.00	0.00
23	->	83	0.00	0.00
23	->	84	-0.00	0.00
23	->	85	-0.00	0.00
23	->	86	-0.00	0.00
23	->	87	-0.00	0.00
23	->	88	-0.00	0.00
23	->	89	-0.02	0.01
23	->	90	-0.91	0.23
23	->	91	-6.72	1.00
23	->	92	-0.06	0.02
23	->	93	-0.01	0.00
23	->	94	-0.01	0.00
23	->	95	-0.00	0.00
23	->	96	-0.00	0.00
23	->	97	-0.01	0.00
23	->	98	-0.00	0.00
23	->	99	-0.01	0.00
23	->	100	-0.01	0.00
23	->	101	-0.01	0.00
23	->	102	-0.01	0.00
23	->	103	-0.01	0.00
23	->	104	-0.00	0.00
23	->	105	-0.00	0.00
23	->	106	-0.00	0.00
24	->	1	-0.00	0.00
24	->	2	-0.00	0.00
24	->	3	-0.02	0.01
24	->	4	-0.08	0.05
24	->	5	-0.30	0.29
24	->	6	-1.33	0.34
24	->	7	-0.16	0.18
24	->	8	-0.01	0.00

24	->	9	-0.01	0.00
24	->	10	0.04	0.01
24	->	11	-0.01	0.00
24	->	12	-0.02	0.01
24	->	13	-0.01	0.00
24	->	14	-0.06	0.02
24	->	15	-0.00	0.00
24	->	16	-0.01	0.00
24	->	17	-0.06	0.02
24	->	18	-0.04	0.03
24	->	19	-0.13	0.06
24	->	20	-4.20	0.56
24	->	21	-2.71	0.46
24	->	22	-0.70	0.18
24	->	23	-10.08	0.57
24	->	24	24.39	1.72
24	->	25	-10.18	0.36
24	->	26	-1.12	0.22
24	->	27	-2.66	0.39
24	->	28	-5.39	0.86
24	->	29	-0.18	0.10
24	->	30	-0.56	0.29
24	->	31	-0.00	0.01
24	->	32	-0.01	0.00
24	->	33	-0.01	0.00
24	->	34	-0.00	0.00
24	->	35	0.00	0.00
24	->	36	0.00	0.00
24	->	37	0.00	0.00
24	->	38	0.00	0.00
24	->	39	0.00	0.00
24	->	40	-0.00	0.00
24	->	41	0.00	0.00
24	->	42	0.00	0.00
24	->	43	-0.02	0.01
24	->	44	-0.01	0.01
24	->	45	-0.00	0.00
24	->	46	-0.00	0.00
24	->	47	-0.00	0.00
24	->	48	-0.00	0.00
24	->	49	0.00	0.00
24	->	50	0.00	0.00
24	->	51	0.00	0.00
24	->	52	-0.00	0.00
24	->	53	0.00	0.00
24	->	54	-0.00	0.00
24	->	55	0.00	0.00
24	->	56	-0.00	0.00
24	->	57	0.00	0.00
24	->	58	-0.00	0.00

24	->	59	0.00	0.00
24	->	60	0.00	0.00
24	->	61	0.00	0.00
24	->	62	-0.00	0.00
24	->	63	-0.00	0.00
24	->	64	-0.00	0.00
24	->	65	0.00	0.00
24	->	66	-0.00	0.00
24	->	67	0.00	0.00
24	->	68	0.00	0.00
24	->	69	0.00	0.00
24	->	70	-0.00	0.00
24	->	71	-0.00	0.00
24	->	72	0.00	0.00
24	->	73	0.00	0.00
24	->	74	-0.00	0.00
24	->	75	0.00	0.00
24	->	76	0.00	0.00
24	->	77	0.00	0.00
24	->	78	-0.00	0.00
24	->	79	0.00	0.00
24	->	80	0.00	0.00
24	->	81	0.00	0.00
24	->	82	0.00	0.00
24	->	83	0.00	0.00
24	->	84	-0.00	0.00
24	->	85	0.00	0.00
24	->	86	0.00	0.00
24	->	87	0.00	0.00
24	->	88	-0.00	0.00
24	->	89	-0.01	0.01
24	->	90	-0.01	0.03
24	->	91	0.08	0.04
24	->	92	-0.00	0.00
24	->	93	-0.00	0.00
24	->	94	-0.00	0.00
24	->	95	-0.00	0.00
24	->	96	-0.01	0.01
24	->	97	-0.01	0.01
24	->	98	-0.00	0.00
24	->	99	-0.00	0.00
24	->	100	-0.00	0.00
24	->	101	-0.00	0.00
24	->	102	-0.00	0.00
24	->	103	-0.00	0.00
24	->	104	-0.00	0.00
24	->	105	0.00	0.00
24	->	106	0.00	0.00
25	->	1	0.04	0.01
25	->	2	-0.00	0.00

25	->	3	-0.00	0.00
25	->	4	-0.01	0.00
25	->	5	-0.06	0.02
25	->	6	-0.02	0.01
25	->	7	-0.00	0.01
25	->	8	-0.01	0.00
25	->	9	-0.00	0.00
25	->	10	-0.05	0.01
25	->	11	-0.02	0.00
25	->	12	0.02	0.00
25	->	13	0.02	0.00
25	->	14	-0.02	0.01
25	->	15	-0.00	0.00
25	->	16	0.04	0.01
25	->	17	-0.03	0.01
25	->	18	-0.10	0.07
25	->	19	-0.16	0.04
25	->	20	-0.17	0.07
25	->	21	-3.07	1.02
25	->	22	-2.84	0.63
25	->	23	-1.26	0.27
25	->	24	-10.21	0.36
25	->	25	-104.78	2.61
25	->	26	-12.61	0.42
25	->	27	-0.63	0.15
25	->	28	-1.43	0.35
25	->	29	-1.11	0.52
25	->	30	-4.47	0.78
25	->	31	0.05	0.07
25	->	32	-1.23	0.46
25	->	33	-0.89	0.40
25	->	34	-0.01	0.00
25	->	35	-0.03	0.00
25	->	36	0.02	0.00
25	->	37	-0.00	0.00
25	->	38	-0.02	0.00
25	->	39	-0.00	0.00
25	->	40	0.03	0.01
25	->	41	0.03	0.01
25	->	42	0.02	0.04
25	->	43	-2.97	2.08
25	->	44	-0.41	0.33
25	->	45	-0.23	0.16
25	->	46	-0.19	0.10
25	->	47	-0.03	0.04
25	->	48	-0.01	0.00
25	->	49	0.00	0.00
25	->	50	0.00	0.00
25	->	51	0.00	0.00
25	->	52	0.00	0.00

25	->	53	0.02	0.00
25	->	54	0.00	0.00
25	->	55	-0.00	0.00
25	->	56	0.00	0.00
25	->	57	0.00	0.00
25	->	58	-0.01	0.00
25	->	59	0.01	0.00
25	->	60	0.00	0.00
25	->	61	0.00	0.00
25	->	62	-0.00	0.00
25	->	63	-0.00	0.00
25	->	64	-0.00	0.00
25	->	65	0.00	0.00
25	->	66	-0.00	0.00
25	->	67	-0.00	0.00
25	->	68	0.00	0.00
25	->	69	0.00	0.00
25	->	70	0.00	0.00
25	->	71	-0.00	0.00
25	->	72	0.02	0.00
25	->	73	0.00	0.00
25	->	74	-0.02	0.00
25	->	75	-0.01	0.00
25	->	76	0.01	0.00
25	->	77	0.01	0.00
25	->	78	0.01	0.00
25	->	79	0.00	0.00
25	->	80	0.00	0.00
25	->	81	0.01	0.00
25	->	82	0.00	0.00
25	->	83	0.00	0.00
25	->	84	-0.00	0.00
25	->	85	-0.00	0.00
25	->	86	-0.01	0.00
25	->	87	-0.01	0.00
25	->	88	-0.04	0.01
25	->	89	-0.20	0.09
25	->	90	-0.56	0.30
25	->	91	-0.25	0.09
25	->	92	-0.06	0.01
25	->	93	-0.02	0.00
25	->	94	-0.03	0.01
25	->	95	-0.02	0.01
25	->	96	-0.09	0.15
25	->	97	-0.32	0.92
25	->	98	-0.11	0.09
25	->	99	-0.07	0.02
25	->	100	-0.02	0.00
25	->	101	-0.01	0.00
25	->	102	-0.02	0.00

25	->	103	-0.01	0.00
25	->	104	-0.01	0.00
25	->	105	-0.01	0.00
25	->	106	-0.01	0.00
26	->	1	0.00	0.00
26	->	2	-0.00	0.00
26	->	3	-0.00	0.00
26	->	4	-0.00	0.00
26	->	5	-0.07	0.02
26	->	6	-0.01	0.00
26	->	7	0.00	0.01
26	->	8	-0.00	0.00
26	->	9	-0.01	0.01
26	->	10	-0.07	0.03
26	->	11	-0.01	0.00
26	->	12	-0.01	0.01
26	->	13	-0.00	0.00
26	->	14	-0.03	0.01
26	->	15	-0.00	0.00
26	->	16	-0.00	0.00
26	->	17	-0.00	0.00
26	->	18	-0.01	0.00
26	->	19	-0.04	0.02
26	->	20	-0.07	0.03
26	->	21	-0.06	0.05
26	->	22	-1.80	0.92
26	->	23	-2.95	0.85
26	->	24	-1.12	0.22
26	->	25	-12.65	0.42
26	->	26	18.74	1.44
26	->	27	-21.82	0.40
26	->	28	-0.64	0.22
26	->	29	-1.19	0.36
26	->	30	-0.38	0.14
26	->	31	-0.13	0.10
26	->	32	-0.03	0.01
26	->	33	-0.00	0.00
26	->	34	-0.00	0.00
26	->	35	-0.00	0.00
26	->	36	0.00	0.00
26	->	37	0.00	0.00
26	->	38	0.00	0.00
26	->	39	-0.00	0.00
26	->	40	-0.01	0.00
26	->	41	-0.01	0.01
26	->	42	-0.02	0.01
26	->	43	-0.47	0.45
26	->	44	-0.08	0.06
26	->	45	0.00	0.00
26	->	46	-0.00	0.00

26 ->	47	-0.01	0.01
26 ->	48	-0.00	0.00
26 ->	49	-0.00	0.00
26 ->	50	-0.00	0.00
26 ->	51	-0.00	0.00
26 ->	52	0.00	0.00
26 ->	53	0.00	0.00
26 ->	54	0.00	0.00
26 ->	55	0.00	0.00
26 ->	56	0.00	0.00
26 ->	57	0.00	0.00
26 ->	58	-0.00	0.00
26 ->	59	0.00	0.00
26 ->	60	0.00	0.00
26 ->	61	0.00	0.00
26 ->	62	0.00	0.00
26 ->	63	-0.00	0.00
26 ->	64	-0.00	0.00
26 ->	65	0.00	0.00
26 ->	66	-0.00	0.00
26 ->	67	0.00	0.00
26 ->	68	0.00	0.00
26 ->	69	0.00	0.00
26 ->	70	0.00	0.00
26 ->	71	-0.00	0.00
26 ->	72	0.00	0.00
26 ->	73	0.00	0.00
26 ->	74	-0.00	0.00
26 ->	75	-0.00	0.00
26 ->	76	0.00	0.00
26 ->	77	0.00	0.00
26 ->	78	0.00	0.00
26 ->	79	0.00	0.00
26 ->	80	0.00	0.00
26 ->	81	0.00	0.00
26 ->	82	0.00	0.00
26 ->	83	0.00	0.00
26 ->	84	-0.00	0.00
26 ->	85	-0.00	0.00
26 ->	86	-0.00	0.00
26 ->	87	-0.00	0.00
26 ->	88	-0.01	0.00
26 ->	89	-0.21	0.07
26 ->	90	-3.32	0.52
26 ->	91	-0.94	0.34
26 ->	92	-0.02	0.00
26 ->	93	-0.00	0.00
26 ->	94	-0.00	0.00
26 ->	95	-0.00	0.00
26 ->	96	-0.00	0.00

26	->	97	-0.00	0.00
26	->	98	-0.00	0.00
26	->	99	-0.00	0.00
26	->	100	-0.00	0.00
26	->	101	-0.00	0.00
26	->	102	-0.00	0.00
26	->	103	-0.01	0.00
26	->	104	-0.00	0.00
26	->	105	-0.00	0.00
26	->	106	-0.00	0.00
27	->	1	-0.01	0.01
27	->	2	-0.00	0.00
27	->	3	-0.01	0.00
27	->	4	-0.06	0.02
27	->	5	2.62	1.11
27	->	6	-0.43	0.28
27	->	7	-2.31	0.94
27	->	8	-0.02	0.02
27	->	9	-0.23	0.22
27	->	10	2.76	1.33
27	->	11	0.15	0.11
27	->	12	-0.10	0.13
27	->	13	-0.02	0.01
27	->	14	-0.01	0.02
27	->	15	0.00	0.00
27	->	16	-0.00	0.00
27	->	17	-0.00	0.00
27	->	18	-0.01	0.00
27	->	19	-0.02	0.00
27	->	20	-0.08	0.03
27	->	21	-0.05	0.02
27	->	22	-0.14	0.07
27	->	23	22.41	13.39
27	->	24	-2.65	0.39
27	->	25	-0.63	0.15
27	->	26	-21.90	0.40
27	->	27	5.14	1.65
27	->	28	-4.85	0.39
27	->	29	-0.55	0.14
27	->	30	-0.03	0.04
27	->	31	-0.02	0.01
27	->	32	-0.00	0.00
27	->	33	-0.00	0.00
27	->	34	-0.00	0.00
27	->	35	-0.00	0.00
27	->	36	0.00	0.00
27	->	37	-0.00	0.00
27	->	38	-0.00	0.00
27	->	39	-0.00	0.00
27	->	40	-0.00	0.00

27	->	41	-0.00	0.00
27	->	42	-0.01	0.00
27	->	43	-0.01	0.01
27	->	44	-0.01	0.00
27	->	45	0.00	0.00
27	->	46	-0.00	0.00
27	->	47	-0.00	0.00
27	->	48	0.00	0.00
27	->	49	0.00	0.00
27	->	50	0.00	0.00
27	->	51	0.00	0.00
27	->	52	0.00	0.00
27	->	53	0.00	0.00
27	->	54	0.00	0.00
27	->	55	-0.00	0.00
27	->	56	0.00	0.00
27	->	57	0.00	0.00
27	->	58	-0.00	0.00
27	->	59	0.00	0.00
27	->	60	0.00	0.00
27	->	61	0.00	0.00
27	->	62	-0.00	0.00
27	->	63	-0.00	0.00
27	->	64	-0.00	0.00
27	->	65	0.00	0.00
27	->	66	-0.00	0.00
27	->	67	0.00	0.00
27	->	68	0.00	0.00
27	->	69	0.00	0.00
27	->	70	0.00	0.00
27	->	71	-0.00	0.00
27	->	72	0.00	0.00
27	->	73	0.00	0.00
27	->	74	-0.00	0.00
27	->	75	-0.00	0.00
27	->	76	0.00	0.00
27	->	77	0.00	0.00
27	->	78	0.00	0.00
27	->	79	0.00	0.00
27	->	80	0.00	0.00
27	->	81	0.00	0.00
27	->	82	0.00	0.00
27	->	83	0.00	0.00
27	->	84	0.00	0.00
27	->	85	-0.00	0.00
27	->	86	-0.00	0.00
27	->	87	-0.00	0.00
27	->	88	-0.00	0.00
27	->	89	0.02	0.01
27	->	90	0.05	0.04

27	->	91	0.10	0.06
27	->	92	-0.00	0.00
27	->	93	-0.00	0.00
27	->	94	-0.00	0.00
27	->	95	-0.00	0.00
27	->	96	-0.00	0.00
27	->	97	-0.00	0.00
27	->	98	-0.00	0.00
27	->	99	-0.00	0.00
27	->	100	-0.00	0.00
27	->	101	-0.00	0.00
27	->	102	-0.00	0.00
27	->	103	-0.00	0.00
27	->	104	-0.00	0.00
27	->	105	-0.00	0.00
27	->	106	-0.00	0.00
28	->	1	0.00	0.00
28	->	2	-0.00	0.00
28	->	3	-0.00	0.00
28	->	4	-0.00	0.00
28	->	5	-0.02	0.01
28	->	6	-0.09	0.16
28	->	7	-0.08	0.05
28	->	8	-0.01	0.00
28	->	9	-0.00	0.00
28	->	10	-0.01	0.00
28	->	11	-0.00	0.00
28	->	12	0.00	0.00
28	->	13	0.00	0.00
28	->	14	-0.00	0.00
28	->	15	0.00	0.00
28	->	16	0.00	0.00
28	->	17	-0.00	0.00
28	->	18	-0.00	0.00
28	->	19	-0.01	0.00
28	->	20	-0.02	0.01
28	->	21	-0.02	0.01
28	->	22	-0.03	0.02
28	->	23	-0.23	0.07
28	->	24	-5.40	0.86
28	->	25	-1.44	0.35
28	->	26	-0.66	0.22
28	->	27	-5.01	0.39
28	->	28	-22.60	1.42
28	->	29	-11.63	0.44
28	->	30	-3.65	1.33
28	->	31	-0.04	0.01
28	->	32	-0.02	0.00
28	->	33	-0.00	0.00
28	->	34	-0.00	0.00

28	->	35	0.00	0.00
28	->	36	-0.00	0.00
28	->	37	0.00	0.00
28	->	38	0.00	0.00
28	->	39	0.00	0.00
28	->	40	-0.00	0.00
28	->	41	-0.00	0.00
28	->	42	-0.00	0.00
28	->	43	-0.02	0.01
28	->	44	-0.00	0.00
28	->	45	-0.00	0.00
28	->	46	-0.00	0.00
28	->	47	-0.00	0.00
28	->	48	-0.00	0.00
28	->	49	0.00	0.00
28	->	50	0.00	0.00
28	->	51	0.00	0.00
28	->	52	0.00	0.00
28	->	53	0.00	0.00
28	->	54	0.00	0.00
28	->	55	0.00	0.00
28	->	56	-0.00	0.00
28	->	57	0.00	0.00
28	->	58	0.00	0.00
28	->	59	0.00	0.00
28	->	60	0.00	0.00
28	->	61	0.00	0.00
28	->	62	0.00	0.00
28	->	63	-0.00	0.00
28	->	64	-0.00	0.00
28	->	65	0.00	0.00
28	->	66	-0.00	0.00
28	->	67	0.00	0.00
28	->	68	0.00	0.00
28	->	69	0.00	0.00
28	->	70	0.00	0.00
28	->	71	0.00	0.00
28	->	72	0.00	0.00
28	->	73	0.00	0.00
28	->	74	-0.00	0.00
28	->	75	-0.00	0.00
28	->	76	0.00	0.00
28	->	77	0.00	0.00
28	->	78	0.00	0.00
28	->	79	0.00	0.00
28	->	80	0.00	0.00
28	->	81	0.00	0.00
28	->	82	0.00	0.00
28	->	83	0.00	0.00
28	->	84	-0.00	0.00

28	->	85	0.00	0.00
28	->	86	-0.00	0.00
28	->	87	0.00	0.00
28	->	88	-0.00	0.00
28	->	89	-0.01	0.00
28	->	90	-0.02	0.01
28	->	91	-0.02	0.01
28	->	92	-0.00	0.00
28	->	93	-0.00	0.00
28	->	94	-0.00	0.00
28	->	95	-0.00	0.00
28	->	96	-0.00	0.00
28	->	97	-0.00	0.00
28	->	98	-0.00	0.00
28	->	99	-0.00	0.00
28	->	100	-0.00	0.00
28	->	101	-0.00	0.00
28	->	102	-0.00	0.00
28	->	103	-0.00	0.00
28	->	104	-0.00	0.00
28	->	105	-0.00	0.00
28	->	106	-0.00	0.00
29	->	1	0.00	0.00
29	->	2	-0.00	0.00
29	->	3	0.00	0.00
29	->	4	-0.00	0.00
29	->	5	-0.01	0.00
29	->	6	-0.00	0.00
29	->	7	-0.01	0.00
29	->	8	-0.00	0.00
29	->	9	-0.00	0.00
29	->	10	-0.01	0.00
29	->	11	-0.00	0.00
29	->	12	0.00	0.00
29	->	13	0.00	0.00
29	->	14	-0.00	0.00
29	->	15	0.00	0.00
29	->	16	0.00	0.00
29	->	17	-0.00	0.00
29	->	18	-0.00	0.00
29	->	19	-0.00	0.00
29	->	20	-0.00	0.00
29	->	21	-0.01	0.00
29	->	22	-0.00	0.01
29	->	23	-0.04	0.03
29	->	24	-0.18	0.10
29	->	25	-1.12	0.52
29	->	26	-1.23	0.36
29	->	27	-0.56	0.14
29	->	28	-11.71	0.44

29	->	29	12.07	0.68
29	->	30	-14.56	0.39
29	->	31	-0.89	0.31
29	->	32	-0.03	0.00
29	->	33	-0.01	0.00
29	->	34	-0.00	0.00
29	->	35	0.00	0.00
29	->	36	-0.00	0.00
29	->	37	0.00	0.00
29	->	38	0.00	0.00
29	->	39	-0.00	0.00
29	->	40	-0.00	0.00
29	->	41	-0.00	0.01
29	->	42	0.00	0.01
29	->	43	-0.04	0.08
29	->	44	-0.01	0.00
29	->	45	-0.00	0.00
29	->	46	-0.00	0.00
29	->	47	-0.00	0.00
29	->	48	-0.00	0.00
29	->	49	0.00	0.00
29	->	50	0.00	0.00
29	->	51	0.00	0.00
29	->	52	0.00	0.00
29	->	53	0.00	0.00
29	->	54	0.00	0.00
29	->	55	0.00	0.00
29	->	56	-0.00	0.00
29	->	57	0.00	0.00
29	->	58	-0.00	0.00
29	->	59	0.00	0.00
29	->	60	0.00	0.00
29	->	61	0.00	0.00
29	->	62	0.00	0.00
29	->	63	-0.00	0.00
29	->	64	0.00	0.00
29	->	65	0.00	0.00
29	->	66	-0.00	0.00
29	->	67	0.00	0.00
29	->	68	0.00	0.00
29	->	69	0.00	0.00
29	->	70	0.00	0.00
29	->	71	0.00	0.00
29	->	72	0.00	0.00
29	->	73	0.00	0.00
29	->	74	-0.00	0.00
29	->	75	-0.00	0.00
29	->	76	0.00	0.00
29	->	77	0.00	0.00
29	->	78	0.00	0.00

29	->	79	0.00	0.00
29	->	80	0.00	0.00
29	->	81	0.00	0.00
29	->	82	0.00	0.00
29	->	83	0.00	0.00
29	->	84	-0.00	0.00
29	->	85	0.00	0.00
29	->	86	-0.00	0.00
29	->	87	-0.00	0.00
29	->	88	-0.00	0.00
29	->	89	-0.03	0.01
29	->	90	-0.06	0.03
29	->	91	-0.01	0.00
29	->	92	-0.00	0.00
29	->	93	-0.00	0.00
29	->	94	-0.00	0.00
29	->	95	-0.00	0.00
29	->	96	-0.00	0.00
29	->	97	-0.00	0.00
29	->	98	-0.00	0.00
29	->	99	-0.00	0.00
29	->	100	-0.00	0.00
29	->	101	-0.00	0.00
29	->	102	-0.00	0.00
29	->	103	-0.00	0.00
29	->	104	-0.00	0.00
29	->	105	-0.00	0.00
29	->	106	-0.00	0.00
30	->	1	-0.00	0.00
30	->	2	-0.00	0.00
30	->	3	-0.00	0.00
30	->	4	-0.00	0.00
30	->	5	-0.00	0.00
30	->	6	-0.01	0.00
30	->	7	-0.00	0.00
30	->	8	-0.00	0.00
30	->	9	-0.00	0.00
30	->	10	-0.00	0.00
30	->	11	-0.00	0.00
30	->	12	-0.00	0.00
30	->	13	-0.00	0.00
30	->	14	-0.00	0.00
30	->	15	-0.00	0.00
30	->	16	-0.00	0.00
30	->	17	-0.00	0.00
30	->	18	-0.00	0.00
30	->	19	0.00	0.00
30	->	20	-0.01	0.00
30	->	21	-0.05	0.03
30	->	22	-0.04	0.01

30 ->	23	-0.03	0.01
30 ->	24	-0.55	0.28
30 ->	25	-4.44	0.76
30 ->	26	-0.38	0.14
30 ->	27	-0.03	0.04
30 ->	28	-3.67	1.32
30 ->	29	-14.72	0.39
30 ->	30	-53.49	1.63
30 ->	31	-11.14	0.41
30 ->	32	-1.35	0.32
30 ->	33	-0.11	0.02
30 ->	34	-0.01	0.00
30 ->	35	-0.00	0.00
30 ->	36	-0.00	0.00
30 ->	37	0.00	0.00
30 ->	38	0.00	0.00
30 ->	39	-0.00	0.00
30 ->	40	0.00	0.00
30 ->	41	0.03	0.02
30 ->	42	-0.21	0.07
30 ->	43	-0.93	0.69
30 ->	44	-0.00	0.01
30 ->	45	-0.01	0.00
30 ->	46	0.00	0.00
30 ->	47	-0.00	0.00
30 ->	48	-0.00	0.00
30 ->	49	-0.00	0.00
30 ->	50	-0.00	0.00
30 ->	51	-0.00	0.00
30 ->	52	-0.00	0.00
30 ->	53	-0.00	0.00
30 ->	54	-0.00	0.00
30 ->	55	0.00	0.00
30 ->	56	-0.00	0.00
30 ->	57	0.00	0.00
30 ->	58	0.00	0.00
30 ->	59	-0.00	0.00
30 ->	60	-0.00	0.00
30 ->	61	0.00	0.00
30 ->	62	0.00	0.00
30 ->	63	0.00	0.00
30 ->	64	0.00	0.00
30 ->	65	-0.00	0.00
30 ->	66	0.00	0.00
30 ->	67	0.00	0.00
30 ->	68	-0.00	0.00
30 ->	69	-0.00	0.00
30 ->	70	-0.00	0.00
30 ->	71	0.00	0.00
30 ->	72	-0.00	0.00

30	->	73	-0.00	0.00
30	->	74	0.00	0.00
30	->	75	0.00	0.00
30	->	76	-0.00	0.00
30	->	77	-0.00	0.00
30	->	78	-0.00	0.00
30	->	79	-0.00	0.00
30	->	80	-0.00	0.00
30	->	81	-0.00	0.00
30	->	82	-0.00	0.00
30	->	83	0.00	0.00
30	->	84	0.00	0.00
30	->	85	0.00	0.00
30	->	86	0.00	0.00
30	->	87	0.00	0.00
30	->	88	-0.00	0.00
30	->	89	-0.01	0.01
30	->	90	0.01	0.02
30	->	91	-0.00	0.00
30	->	92	0.00	0.00
30	->	93	-0.00	0.00
30	->	94	0.00	0.00
30	->	95	0.00	0.00
30	->	96	-0.00	0.00
30	->	97	0.00	0.00
30	->	98	0.00	0.00
30	->	99	0.00	0.00
30	->	100	0.00	0.00
30	->	101	0.00	0.00
30	->	102	0.00	0.00
30	->	103	0.00	0.00
30	->	104	0.00	0.00
30	->	105	0.00	0.00
30	->	106	0.00	0.00
31	->	1	0.02	0.00
31	->	2	-0.00	0.00
31	->	3	0.00	0.00
31	->	4	-0.00	0.00
31	->	5	-0.02	0.00
31	->	6	0.00	0.00
31	->	7	-0.00	0.00
31	->	8	-0.01	0.00
31	->	9	0.00	0.00
31	->	10	-0.03	0.00
31	->	11	-0.01	0.00
31	->	12	0.02	0.00
31	->	13	0.01	0.00
31	->	14	-0.00	0.00
31	->	15	0.00	0.00
31	->	16	0.01	0.00

31	->	17	-0.00	0.00
31	->	18	-0.00	0.00
31	->	19	-0.03	0.00
31	->	20	-0.00	0.00
31	->	21	-0.00	0.00
31	->	22	0.03	0.02
31	->	23	-0.01	0.00
31	->	24	-0.00	0.01
31	->	25	0.05	0.07
31	->	26	-0.13	0.09
31	->	27	-0.02	0.01
31	->	28	-0.04	0.01
31	->	29	-0.94	0.33
31	->	30	-11.20	0.41
31	->	31	11.81	3.58
31	->	32	-0.30	0.35
31	->	33	-0.95	0.28
31	->	34	-0.13	0.06
31	->	35	-0.08	0.01
31	->	36	0.02	0.00
31	->	37	-0.00	0.00
31	->	38	-0.05	0.01
31	->	39	-0.01	0.01
31	->	40	0.39	0.42
31	->	41	-2.04	0.69
31	->	42	-3.17	0.40
31	->	43	-2.70	1.38
31	->	44	-0.08	0.05
31	->	45	-0.07	0.02
31	->	46	-0.07	0.01
31	->	47	-0.03	0.09
31	->	48	-0.03	0.01
31	->	49	-0.00	0.00
31	->	50	-0.00	0.00
31	->	51	-0.00	0.00
31	->	52	-0.00	0.00
31	->	53	0.02	0.00
31	->	54	0.00	0.00
31	->	55	-0.00	0.00
31	->	56	0.00	0.00
31	->	57	0.00	0.00
31	->	58	-0.01	0.00
31	->	59	0.01	0.00
31	->	60	0.00	0.00
31	->	61	-0.00	0.00
31	->	62	-0.00	0.00
31	->	63	-0.00	0.00
31	->	64	-0.00	0.00
31	->	65	0.00	0.00
31	->	66	-0.00	0.00

31	->	67	0.00	0.00
31	->	68	0.01	0.00
31	->	69	0.00	0.00
31	->	70	0.00	0.00
31	->	71	-0.00	0.00
31	->	72	0.03	0.00
31	->	73	0.00	0.00
31	->	74	-0.02	0.00
31	->	75	-0.02	0.00
31	->	76	0.01	0.00
31	->	77	0.01	0.00
31	->	78	0.02	0.00
31	->	79	0.00	0.00
31	->	80	0.00	0.00
31	->	81	0.01	0.00
31	->	82	0.00	0.00
31	->	83	0.00	0.00
31	->	84	-0.00	0.00
31	->	85	-0.00	0.00
31	->	86	-0.01	0.00
31	->	87	-0.04	0.01
31	->	88	-0.50	0.43
31	->	89	-3.38	2.94
31	->	90	-0.70	0.79
31	->	91	-0.05	0.01
31	->	92	-0.02	0.00
31	->	93	-0.01	0.00
31	->	94	-0.01	0.00
31	->	95	-0.00	0.00
31	->	96	-0.01	0.00
31	->	97	-0.02	0.01
31	->	98	-0.02	0.01
31	->	99	-0.03	0.00
31	->	100	-0.01	0.00
31	->	101	-0.01	0.00
31	->	102	-0.01	0.00
31	->	103	-0.01	0.00
31	->	104	-0.02	0.01
31	->	105	-0.02	0.01
31	->	106	-0.02	0.01
32	->	1	0.00	0.00
32	->	2	0.00	0.00
32	->	3	-0.00	0.00
32	->	4	-0.00	0.00
32	->	5	-0.00	0.00
32	->	6	-0.00	0.00
32	->	7	-0.00	0.00
32	->	8	-0.00	0.00
32	->	9	0.00	0.00
32	->	10	-0.00	0.00

32	->	11	-0.00	0.00
32	->	12	0.00	0.00
32	->	13	0.00	0.00
32	->	14	-0.00	0.00
32	->	15	0.00	0.00
32	->	16	0.00	0.00
32	->	17	-0.00	0.00
32	->	18	-0.00	0.00
32	->	19	-0.00	0.00
32	->	20	-0.00	0.00
32	->	21	-0.02	0.01
32	->	22	-0.02	0.01
32	->	23	-0.01	0.00
32	->	24	-0.01	0.00
32	->	25	-1.21	0.47
32	->	26	-0.03	0.01
32	->	27	-0.00	0.00
32	->	28	-0.02	0.00
32	->	29	-0.03	0.00
32	->	30	-1.38	0.32
32	->	31	-0.34	0.35
32	->	32	1.53	0.54
32	->	33	-10.59	0.45
32	->	34	-0.57	0.38
32	->	35	-0.02	0.00
32	->	36	-0.01	0.01
32	->	37	-0.00	0.00
32	->	38	0.00	0.00
32	->	39	-0.01	0.00
32	->	40	-0.02	0.01
32	->	41	-0.25	0.17
32	->	42	-0.84	0.21
32	->	43	-0.74	0.28
32	->	44	-0.16	0.08
32	->	45	-0.07	0.02
32	->	46	-0.02	0.00
32	->	47	-0.01	0.01
32	->	48	-0.04	0.01
32	->	49	-0.00	0.00
32	->	50	-0.00	0.00
32	->	51	-0.00	0.00
32	->	52	-0.00	0.00
32	->	53	-0.00	0.00
32	->	54	0.00	0.00
32	->	55	-0.00	0.00
32	->	56	-0.00	0.00
32	->	57	0.00	0.00
32	->	58	0.00	0.00
32	->	59	0.00	0.00
32	->	60	0.00	0.00

32	->	61	0.00	0.00
32	->	62	-0.00	0.00
32	->	63	-0.00	0.00
32	->	64	-0.00	0.00
32	->	65	0.00	0.00
32	->	66	-0.00	0.00
32	->	67	0.00	0.00
32	->	68	0.00	0.00
32	->	69	-0.00	0.00
32	->	70	0.00	0.00
32	->	71	-0.00	0.00
32	->	72	0.00	0.00
32	->	73	0.00	0.00
32	->	74	-0.00	0.00
32	->	75	-0.00	0.00
32	->	76	0.00	0.00
32	->	77	0.00	0.00
32	->	78	0.00	0.00
32	->	79	0.00	0.00
32	->	80	0.00	0.00
32	->	81	0.00	0.00
32	->	82	0.00	0.00
32	->	83	0.00	0.00
32	->	84	-0.00	0.00
32	->	85	0.00	0.00
32	->	86	-0.00	0.00
32	->	87	-0.00	0.00
32	->	88	-0.00	0.00
32	->	89	-0.01	0.00
32	->	90	-0.02	0.01
32	->	91	-0.01	0.00
32	->	92	-0.00	0.00
32	->	93	-0.00	0.00
32	->	94	-0.00	0.00
32	->	95	-0.00	0.00
32	->	96	-0.01	0.01
32	->	97	-0.02	0.03
32	->	98	-0.01	0.01
32	->	99	-0.00	0.00
32	->	100	-0.00	0.00
32	->	101	-0.00	0.00
32	->	102	-0.00	0.00
32	->	103	-0.00	0.00
32	->	104	-0.00	0.00
32	->	105	-0.00	0.00
32	->	106	-0.00	0.00
33	->	1	0.00	0.00
33	->	2	0.00	0.00
33	->	3	-0.00	0.00
33	->	4	0.00	0.00

33	->	5	0.00	0.00
33	->	6	-0.00	0.00
33	->	7	-0.00	0.00
33	->	8	0.00	0.00
33	->	9	-0.00	0.00
33	->	10	0.00	0.00
33	->	11	0.00	0.00
33	->	12	-0.00	0.00
33	->	13	0.00	0.00
33	->	14	-0.00	0.00
33	->	15	0.00	0.00
33	->	16	-0.00	0.00
33	->	17	-0.00	0.00
33	->	18	-0.01	0.00
33	->	19	-0.00	0.00
33	->	20	-0.00	0.00
33	->	21	-0.02	0.01
33	->	22	-0.04	0.02
33	->	23	-0.00	0.00
33	->	24	-0.01	0.00
33	->	25	-0.87	0.39
33	->	26	-0.00	0.00
33	->	27	-0.00	0.00
33	->	28	-0.00	0.00
33	->	29	-0.01	0.00
33	->	30	-0.11	0.02
33	->	31	-0.96	0.28
33	->	32	-10.73	0.44
33	->	33	27.79	0.98
33	->	34	-13.07	0.49
33	->	35	-0.44	0.05
33	->	36	-0.02	0.02
33	->	37	-0.01	0.00
33	->	38	-0.01	0.00
33	->	39	-0.02	0.01
33	->	40	-0.28	0.08
33	->	41	-2.65	0.72
33	->	42	-3.42	0.41
33	->	43	-0.85	0.20
33	->	44	-2.30	0.60
33	->	45	-2.23	0.38
33	->	46	0.01	0.02
33	->	47	-0.06	0.03
33	->	48	-1.27	0.26
33	->	49	-0.04	0.01
33	->	50	-0.01	0.00
33	->	51	-0.02	0.00
33	->	52	-0.02	0.00
33	->	53	-0.00	0.00
33	->	54	-0.00	0.00

33	->	55	-0.01	0.00
33	->	56	-0.00	0.00
33	->	57	0.00	0.00
33	->	58	-0.00	0.00
33	->	59	0.00	0.00
33	->	60	-0.00	0.00
33	->	61	-0.00	0.00
33	->	62	-0.00	0.00
33	->	63	-0.00	0.00
33	->	64	-0.00	0.00
33	->	65	0.00	0.00
33	->	66	-0.00	0.00
33	->	67	0.00	0.00
33	->	68	0.00	0.00
33	->	69	0.00	0.00
33	->	70	0.00	0.00
33	->	71	-0.00	0.00
33	->	72	-0.00	0.00
33	->	73	0.00	0.00
33	->	74	-0.00	0.00
33	->	75	-0.00	0.00
33	->	76	0.00	0.00
33	->	77	0.00	0.00
33	->	78	-0.00	0.00
33	->	79	0.00	0.00
33	->	80	0.00	0.00
33	->	81	-0.00	0.00
33	->	82	0.00	0.00
33	->	83	0.00	0.00
33	->	84	-0.00	0.00
33	->	85	0.00	0.00
33	->	86	-0.00	0.00
33	->	87	-0.00	0.00
33	->	88	-0.01	0.00
33	->	89	-0.01	0.01
33	->	90	-0.01	0.00
33	->	91	-0.00	0.00
33	->	92	-0.00	0.00
33	->	93	-0.00	0.00
33	->	94	-0.00	0.00
33	->	95	-0.00	0.00
33	->	96	-0.02	0.06
33	->	97	-0.13	0.27
33	->	98	-0.15	0.18
33	->	99	-0.03	0.01
33	->	100	-0.01	0.00
33	->	101	-0.00	0.00
33	->	102	-0.00	0.00
33	->	103	-0.00	0.00
33	->	104	-0.00	0.00

33	->	105	-0.00	0.00
33	->	106	0.00	0.00
34	->	1	-0.00	0.00
34	->	2	-0.00	0.00
34	->	3	-0.00	0.00
34	->	4	-0.00	0.00
34	->	5	-0.00	0.00
34	->	6	0.00	0.00
34	->	7	0.00	0.00
34	->	8	-0.00	0.00
34	->	9	0.00	0.00
34	->	10	-0.00	0.00
34	->	11	-0.00	0.00
34	->	12	0.00	0.00
34	->	13	0.00	0.00
34	->	14	0.00	0.00
34	->	15	0.00	0.00
34	->	16	-0.00	0.00
34	->	17	-0.00	0.00
34	->	18	-0.00	0.00
34	->	19	0.00	0.00
34	->	20	-0.00	0.00
34	->	21	-0.00	0.00
34	->	22	-0.00	0.00
34	->	23	-0.00	0.00
34	->	24	-0.00	0.00
34	->	25	-0.01	0.00
34	->	26	-0.00	0.00
34	->	27	-0.00	0.00
34	->	28	-0.00	0.00
34	->	29	-0.00	0.00
34	->	30	-0.01	0.00
34	->	31	-0.13	0.06
34	->	32	-0.59	0.40
34	->	33	-13.06	0.48
34	->	34	-55.73	2.43
34	->	35	-12.11	0.52
34	->	36	-0.66	0.86
34	->	37	-0.04	0.05
34	->	38	-0.14	0.06
34	->	39	-1.50	1.34
34	->	40	-1.75	0.80
34	->	41	-2.62	1.22
34	->	42	-0.92	0.26
34	->	43	-0.04	0.01
34	->	44	-0.03	0.01
34	->	45	-0.03	0.01
34	->	46	-0.00	0.01
34	->	47	0.01	0.01
34	->	48	-1.22	0.20

34	->	49	-0.01	0.01
34	->	50	-0.00	0.00
34	->	51	-0.09	0.03
34	->	52	-0.06	0.03
34	->	53	-0.01	0.00
34	->	54	-0.00	0.00
34	->	55	-0.06	0.01
34	->	56	-0.00	0.00
34	->	57	-0.00	0.00
34	->	58	0.00	0.00
34	->	59	-0.00	0.00
34	->	60	-0.00	0.00
34	->	61	0.00	0.00
34	->	62	0.00	0.00
34	->	63	0.00	0.00
34	->	64	0.00	0.00
34	->	65	0.00	0.00
34	->	66	0.00	0.00
34	->	67	0.00	0.00
34	->	68	0.00	0.00
34	->	69	-0.00	0.00
34	->	70	0.00	0.00
34	->	71	0.00	0.00
34	->	72	-0.00	0.00
34	->	73	-0.00	0.00
34	->	74	-0.00	0.00
34	->	75	-0.00	0.00
34	->	76	-0.00	0.00
34	->	77	0.00	0.00
34	->	78	0.00	0.00
34	->	79	0.00	0.00
34	->	80	0.00	0.00
34	->	81	0.00	0.00
34	->	82	0.00	0.00
34	->	83	-0.00	0.00
34	->	84	-0.00	0.00
34	->	85	-0.00	0.00
34	->	86	-0.00	0.00
34	->	87	-0.01	0.00
34	->	88	-0.02	0.01
34	->	89	-0.02	0.01
34	->	90	-0.00	0.00
34	->	91	-0.00	0.00
34	->	92	-0.00	0.00
34	->	93	0.00	0.00
34	->	94	0.00	0.00
34	->	95	0.00	0.00
34	->	96	-0.00	0.00
34	->	97	-0.00	0.00
34	->	98	-0.00	0.00

34	->	99	-0.00	0.00
34	->	100	-0.00	0.00
34	->	101	-0.00	0.00
34	->	102	-0.00	0.00
34	->	103	-0.00	0.00
34	->	104	-0.00	0.00
34	->	105	-0.00	0.00
34	->	106	-0.00	0.00
35	->	1	-0.01	0.00
35	->	2	-0.00	0.00
35	->	3	-0.00	0.00
35	->	4	0.00	0.00
35	->	5	0.00	0.00
35	->	6	-0.00	0.00
35	->	7	0.00	0.00
35	->	8	0.00	0.00
35	->	9	-0.00	0.00
35	->	10	0.01	0.00
35	->	11	0.00	0.00
35	->	12	-0.00	0.00
35	->	13	-0.00	0.00
35	->	14	-0.00	0.00
35	->	15	-0.00	0.00
35	->	16	-0.01	0.00
35	->	17	0.00	0.00
35	->	18	0.00	0.00
35	->	19	0.01	0.00
35	->	20	0.00	0.00
35	->	21	0.00	0.00
35	->	22	-0.02	0.00
35	->	23	-0.00	0.00
35	->	24	0.00	0.00
35	->	25	-0.03	0.00
35	->	26	-0.00	0.00
35	->	27	-0.00	0.00
35	->	28	0.00	0.00
35	->	29	0.00	0.00
35	->	30	-0.00	0.00
35	->	31	-0.08	0.01
35	->	32	-0.02	0.00
35	->	33	-0.44	0.05
35	->	34	-12.16	0.52
35	->	35	-24.65	2.55
35	->	36	-10.43	1.34
35	->	37	-0.52	3.70
35	->	38	2.73	0.64
35	->	39	-1.72	1.19
35	->	40	-2.26	0.93
35	->	41	-0.39	0.25
35	->	42	-0.61	0.18

35 ->	43	0.01	0.01
35 ->	44	-0.05	0.01
35 ->	45	-0.00	0.01
35 ->	46	0.04	0.01
35 ->	47	-0.12	0.04
35 ->	48	-1.09	0.22
35 ->	49	0.02	0.02
35 ->	50	-0.04	0.02
35 ->	51	2.36	0.78
35 ->	52	-0.46	0.32
35 ->	53	-0.06	0.02
35 ->	54	-0.07	0.04
35 ->	55	2.72	0.56
35 ->	56	-0.02	0.01
35 ->	57	-0.00	0.00
35 ->	58	0.03	0.01
35 ->	59	-0.03	0.00
35 ->	60	-0.00	0.00
35 ->	61	0.00	0.00
35 ->	62	0.00	0.00
35 ->	63	0.01	0.00
35 ->	64	0.00	0.00
35 ->	65	-0.00	0.00
35 ->	66	0.01	0.00
35 ->	67	0.00	0.00
35 ->	68	-0.01	0.00
35 ->	69	-0.01	0.00
35 ->	70	-0.00	0.00
35 ->	71	-0.00	0.00
35 ->	72	-0.03	0.00
35 ->	73	-0.00	0.00
35 ->	74	0.02	0.00
35 ->	75	0.02	0.00
35 ->	76	-0.01	0.00
35 ->	77	-0.01	0.00
35 ->	78	-0.01	0.00
35 ->	79	-0.00	0.00
35 ->	80	-0.00	0.00
35 ->	81	-0.01	0.00
35 ->	82	-0.00	0.00
35 ->	83	0.00	0.00
35 ->	84	0.00	0.00
35 ->	85	0.00	0.00
35 ->	86	0.02	0.00
35 ->	87	0.08	0.02
35 ->	88	0.13	0.04
35 ->	89	0.05	0.01
35 ->	90	0.03	0.00
35 ->	91	0.01	0.00
35 ->	92	0.01	0.00

35	->	93	0.00	0.00
35	->	94	0.00	0.00
35	->	95	0.00	0.00
35	->	96	0.00	0.00
35	->	97	0.02	0.00
35	->	98	0.03	0.01
35	->	99	0.03	0.01
35	->	100	0.02	0.00
35	->	101	0.01	0.00
35	->	102	0.01	0.00
35	->	103	0.01	0.00
35	->	104	0.01	0.00
35	->	105	0.00	0.00
35	->	106	0.01	0.00
36	->	1	0.00	0.00
36	->	2	0.00	0.00
36	->	3	0.00	0.00
36	->	4	-0.00	0.00
36	->	5	-0.00	0.00
36	->	6	0.00	0.00
36	->	7	0.00	0.00
36	->	8	-0.00	0.00
36	->	9	0.00	0.00
36	->	10	-0.00	0.00
36	->	11	-0.00	0.00
36	->	12	0.00	0.00
36	->	13	0.00	0.00
36	->	14	0.00	0.00
36	->	15	0.00	0.00
36	->	16	0.01	0.00
36	->	17	-0.00	0.00
36	->	18	-0.00	0.00
36	->	19	-0.01	0.00
36	->	20	-0.00	0.00
36	->	21	-0.00	0.00
36	->	22	0.01	0.00
36	->	23	0.00	0.00
36	->	24	0.00	0.00
36	->	25	0.02	0.00
36	->	26	0.00	0.00
36	->	27	0.00	0.00
36	->	28	-0.00	0.00
36	->	29	-0.00	0.00
36	->	30	-0.00	0.00
36	->	31	0.02	0.00
36	->	32	-0.01	0.01
36	->	33	-0.02	0.02
36	->	34	-0.68	0.86
36	->	35	-10.53	1.35
36	->	36	-115.53	2.56

36	->	37	-13.60	0.86
36	->	38	-0.83	0.22
36	->	39	-0.96	0.90
36	->	40	-0.01	0.02
36	->	41	-0.03	0.01
36	->	42	-0.03	0.01
36	->	43	-0.00	0.00
36	->	44	0.01	0.00
36	->	45	-0.01	0.01
36	->	46	-0.04	0.01
36	->	47	-0.01	0.00
36	->	48	-0.17	0.08
36	->	49	-0.04	0.03
36	->	50	-0.01	0.01
36	->	51	-0.13	0.05
36	->	52	-1.22	1.19
36	->	53	0.01	0.01
36	->	54	-0.00	0.01
36	->	55	-0.36	0.18
36	->	56	-0.21	0.30
36	->	57	-0.00	0.00
36	->	58	-0.06	0.05
36	->	59	0.02	0.01
36	->	60	0.00	0.00
36	->	61	-0.00	0.00
36	->	62	-0.00	0.00
36	->	63	-0.00	0.00
36	->	64	-0.00	0.00
36	->	65	0.00	0.00
36	->	66	-0.00	0.00
36	->	67	-0.00	0.00
36	->	68	0.01	0.00
36	->	69	0.01	0.01
36	->	70	-0.00	0.00
36	->	71	-0.00	0.00
36	->	72	0.02	0.00
36	->	73	0.00	0.00
36	->	74	-0.01	0.00
36	->	75	-0.02	0.01
36	->	76	0.01	0.00
36	->	77	0.01	0.00
36	->	78	0.01	0.00
36	->	79	0.00	0.00
36	->	80	0.00	0.00
36	->	81	0.00	0.00
36	->	82	0.00	0.00
36	->	83	-0.00	0.00
36	->	84	-0.00	0.00
36	->	85	-0.00	0.00
36	->	86	-0.01	0.00

36 ->	87	-0.03	0.01
36 ->	88	-0.04	0.01
36 ->	89	-0.02	0.00
36 ->	90	-0.02	0.00
36 ->	91	-0.01	0.00
36 ->	92	-0.01	0.00
36 ->	93	-0.00	0.00
36 ->	94	-0.00	0.00
36 ->	95	-0.00	0.00
36 ->	96	-0.00	0.00
36 ->	97	-0.03	0.01
36 ->	98	-0.04	0.02
36 ->	99	-0.04	0.02
36 ->	100	-0.02	0.01
36 ->	101	-0.01	0.00
36 ->	102	-0.00	0.00
36 ->	103	-0.00	0.00
36 ->	104	-0.00	0.00
36 ->	105	-0.00	0.00
36 ->	106	-0.00	0.00
37 ->	1	-0.00	0.00
37 ->	2	0.00	0.00
37 ->	3	0.00	0.00
37 ->	4	0.00	0.00
37 ->	5	0.00	0.00
37 ->	6	0.00	0.00
37 ->	7	0.00	0.00
37 ->	8	0.00	0.00
37 ->	9	0.00	0.00
37 ->	10	0.00	0.00
37 ->	11	0.00	0.00
37 ->	12	-0.00	0.00
37 ->	13	-0.00	0.00
37 ->	14	0.00	0.00
37 ->	15	0.00	0.00
37 ->	16	-0.00	0.00
37 ->	17	-0.00	0.00
37 ->	18	0.00	0.00
37 ->	19	0.00	0.00
37 ->	20	0.00	0.00
37 ->	21	0.00	0.00
37 ->	22	-0.00	0.00
37 ->	23	0.00	0.00
37 ->	24	0.00	0.00
37 ->	25	-0.00	0.00
37 ->	26	0.00	0.00
37 ->	27	-0.00	0.00
37 ->	28	0.00	0.00
37 ->	29	0.00	0.00
37 ->	30	0.00	0.00

37	->	31	-0.00	0.00
37	->	32	-0.00	0.00
37	->	33	-0.01	0.00
37	->	34	-0.04	0.05
37	->	35	-0.59	3.70
37	->	36	-13.73	0.84
37	->	37	20.93	3.10
37	->	38	-10.64	4.03
37	->	39	-0.80	0.37
37	->	40	-0.19	0.12
37	->	41	-0.01	0.01
37	->	42	-0.03	0.02
37	->	43	-0.00	0.00
37	->	44	-0.00	0.00
37	->	45	-0.00	0.00
37	->	46	-0.00	0.00
37	->	47	-0.00	0.01
37	->	48	-0.03	0.01
37	->	49	-0.01	0.00
37	->	50	-0.01	0.00
37	->	51	0.38	0.53
37	->	52	-0.24	0.30
37	->	53	-0.01	0.01
37	->	54	-0.03	0.01
37	->	55	-0.39	1.52
37	->	56	-0.12	0.20
37	->	57	-0.01	0.00
37	->	58	-0.01	0.00
37	->	59	-0.00	0.00
37	->	60	-0.00	0.00
37	->	61	-0.00	0.00
37	->	62	-0.00	0.00
37	->	63	0.00	0.00
37	->	64	0.00	0.00
37	->	65	0.00	0.00
37	->	66	0.00	0.00
37	->	67	0.00	0.00
37	->	68	-0.00	0.00
37	->	69	-0.00	0.00
37	->	70	-0.00	0.00
37	->	71	-0.00	0.00
37	->	72	-0.00	0.00
37	->	73	0.00	0.00
37	->	74	0.00	0.00
37	->	75	0.00	0.00
37	->	76	-0.00	0.00
37	->	77	-0.00	0.00
37	->	78	-0.00	0.00
37	->	79	-0.00	0.00
37	->	80	-0.00	0.00

37	->	81	-0.00	0.00
37	->	82	-0.00	0.00
37	->	83	-0.00	0.00
37	->	84	0.00	0.00
37	->	85	-0.00	0.00
37	->	86	-0.00	0.00
37	->	87	-0.00	0.01
37	->	88	-0.00	0.01
37	->	89	-0.00	0.00
37	->	90	0.00	0.00
37	->	91	0.00	0.00
37	->	92	0.00	0.00
37	->	93	0.00	0.00
37	->	94	0.00	0.00
37	->	95	0.00	0.00
37	->	96	0.00	0.00
37	->	97	0.00	0.00
37	->	98	-0.00	0.00
37	->	99	-0.00	0.00
37	->	100	-0.00	0.00
37	->	101	0.00	0.00
37	->	102	0.00	0.00
37	->	103	0.00	0.00
37	->	104	0.00	0.00
37	->	105	-0.00	0.00
37	->	106	0.00	0.00
38	->	1	-0.01	0.00
38	->	2	-0.00	0.00
38	->	3	-0.00	0.00
38	->	4	0.00	0.00
38	->	5	0.00	0.00
38	->	6	-0.00	0.00
38	->	7	-0.00	0.00
38	->	8	0.00	0.00
38	->	9	-0.00	0.00
38	->	10	0.01	0.00
38	->	11	0.00	0.00
38	->	12	-0.00	0.00
38	->	13	-0.00	0.00
38	->	14	-0.00	0.00
38	->	15	-0.00	0.00
38	->	16	-0.00	0.00
38	->	17	0.00	0.00
38	->	18	0.00	0.00
38	->	19	0.01	0.00
38	->	20	0.00	0.00
38	->	21	0.00	0.00
38	->	22	-0.01	0.00
38	->	23	-0.00	0.00
38	->	24	0.00	0.00

38	->	25	-0.02	0.00
38	->	26	0.00	0.00
38	->	27	-0.00	0.00
38	->	28	0.00	0.00
38	->	29	0.00	0.00
38	->	30	0.00	0.00
38	->	31	-0.05	0.01
38	->	32	0.00	0.00
38	->	33	-0.01	0.00
38	->	34	-0.14	0.06
38	->	35	2.73	0.65
38	->	36	-0.83	0.22
38	->	37	-10.61	4.08
38	->	38	-34.08	3.84
38	->	39	-11.85	0.67
38	->	40	-3.13	2.71
38	->	41	0.00	0.03
38	->	42	-0.02	0.05
38	->	43	0.00	0.00
38	->	44	-0.02	0.00
38	->	45	0.00	0.00
38	->	46	0.03	0.00
38	->	47	-0.02	0.01
38	->	48	-0.02	0.01
38	->	49	0.00	0.00
38	->	50	-0.01	0.01
38	->	51	2.63	0.95
38	->	52	0.04	0.02
38	->	53	-0.04	0.01
38	->	54	-0.05	0.03
38	->	55	2.49	0.75
38	->	56	-0.01	0.00
38	->	57	-0.00	0.00
38	->	58	0.03	0.00
38	->	59	-0.03	0.01
38	->	60	-0.00	0.00
38	->	61	0.00	0.00
38	->	62	-0.00	0.00
38	->	63	0.01	0.00
38	->	64	0.00	0.00
38	->	65	-0.00	0.00
38	->	66	0.01	0.00
38	->	67	0.00	0.00
38	->	68	-0.01	0.00
38	->	69	-0.01	0.00
38	->	70	-0.00	0.00
38	->	71	-0.00	0.00
38	->	72	-0.03	0.00
38	->	73	-0.00	0.00
38	->	74	0.02	0.00

38	->	75	0.03	0.00
38	->	76	-0.01	0.00
38	->	77	-0.01	0.00
38	->	78	-0.01	0.00
38	->	79	-0.00	0.00
38	->	80	-0.00	0.00
38	->	81	-0.01	0.00
38	->	82	-0.00	0.00
38	->	83	0.00	0.00
38	->	84	0.00	0.00
38	->	85	0.00	0.00
38	->	86	0.02	0.00
38	->	87	0.10	0.04
38	->	88	0.18	0.09
38	->	89	0.05	0.01
38	->	90	0.03	0.00
38	->	91	0.01	0.00
38	->	92	0.01	0.00
38	->	93	0.00	0.00
38	->	94	0.00	0.00
38	->	95	0.00	0.00
38	->	96	0.00	0.00
38	->	97	0.01	0.00
38	->	98	0.02	0.00
38	->	99	0.02	0.00
38	->	100	0.01	0.00
38	->	101	0.01	0.00
38	->	102	0.01	0.00
38	->	103	0.01	0.00
38	->	104	0.01	0.00
38	->	105	0.00	0.00
38	->	106	0.01	0.00
39	->	1	0.00	0.00
39	->	2	0.00	0.00
39	->	3	0.00	0.00
39	->	4	0.00	0.00
39	->	5	0.00	0.00
39	->	6	-0.00	0.00
39	->	7	0.00	0.00
39	->	8	0.00	0.00
39	->	9	-0.00	0.00
39	->	10	0.00	0.00
39	->	11	0.00	0.00
39	->	12	-0.00	0.00
39	->	13	-0.00	0.00
39	->	14	0.00	0.00
39	->	15	0.00	0.00
39	->	16	-0.00	0.00
39	->	17	0.00	0.00
39	->	18	0.00	0.00

39	->	19	0.00	0.00
39	->	20	0.00	0.00
39	->	21	0.00	0.00
39	->	22	-0.00	0.00
39	->	23	0.00	0.00
39	->	24	0.00	0.00
39	->	25	-0.00	0.00
39	->	26	-0.00	0.00
39	->	27	-0.00	0.00
39	->	28	0.00	0.00
39	->	29	-0.00	0.00
39	->	30	-0.00	0.00
39	->	31	-0.01	0.01
39	->	32	-0.01	0.00
39	->	33	-0.02	0.01
39	->	34	-1.48	1.33
39	->	35	-1.74	1.19
39	->	36	-0.94	0.88
39	->	37	-0.82	0.37
39	->	38	-11.94	0.69
39	->	39	11.39	1.89
39	->	40	-9.44	0.67
39	->	41	-0.54	0.28
39	->	42	-0.10	0.02
39	->	43	-0.01	0.00
39	->	44	-0.00	0.00
39	->	45	-0.00	0.00
39	->	46	-0.00	0.00
39	->	47	0.00	0.00
39	->	48	-0.02	0.01
39	->	49	-0.00	0.00
39	->	50	-0.00	0.00
39	->	51	-0.06	0.05
39	->	52	-0.01	0.01
39	->	53	-0.00	0.00
39	->	54	-0.00	0.00
39	->	55	-0.02	0.04
39	->	56	-0.00	0.00
39	->	57	0.00	0.00
39	->	58	-0.00	0.00
39	->	59	0.00	0.00
39	->	60	0.00	0.00
39	->	61	-0.00	0.00
39	->	62	0.00	0.00
39	->	63	-0.00	0.00
39	->	64	-0.00	0.00
39	->	65	0.00	0.00
39	->	66	-0.00	0.00
39	->	67	0.00	0.00
39	->	68	0.00	0.00

39	->	69	0.00	0.00
39	->	70	-0.00	0.00
39	->	71	0.00	0.00
39	->	72	-0.00	0.00
39	->	73	0.00	0.00
39	->	74	-0.00	0.00
39	->	75	-0.00	0.00
39	->	76	0.00	0.00
39	->	77	-0.00	0.00
39	->	78	0.00	0.00
39	->	79	0.00	0.00
39	->	80	0.00	0.00
39	->	81	0.00	0.00
39	->	82	0.00	0.00
39	->	83	0.00	0.00
39	->	84	-0.00	0.00
39	->	85	-0.00	0.00
39	->	86	-0.00	0.00
39	->	87	-0.00	0.00
39	->	88	-0.01	0.01
39	->	89	-0.01	0.00
39	->	90	-0.00	0.00
39	->	91	0.00	0.00
39	->	92	0.00	0.00
39	->	93	-0.00	0.00
39	->	94	0.00	0.00
39	->	95	0.00	0.00
39	->	96	-0.00	0.00
39	->	97	-0.00	0.00
39	->	98	-0.00	0.00
39	->	99	-0.00	0.00
39	->	100	-0.00	0.00
39	->	101	-0.00	0.00
39	->	102	-0.00	0.00
39	->	103	0.00	0.00
39	->	104	0.00	0.00
39	->	105	-0.00	0.00
39	->	106	-0.00	0.00
40	->	1	0.01	0.00
40	->	2	-0.00	0.00
40	->	3	0.00	0.00
40	->	4	-0.00	0.00
40	->	5	-0.01	0.00
40	->	6	0.00	0.00
40	->	7	0.00	0.00
40	->	8	-0.00	0.00
40	->	9	0.00	0.00
40	->	10	-0.01	0.00
40	->	11	-0.01	0.00
40	->	12	0.01	0.00

40	->	13	0.00	0.00
40	->	14	0.00	0.00
40	->	15	0.00	0.00
40	->	16	0.01	0.00
40	->	17	-0.00	0.00
40	->	18	-0.00	0.00
40	->	19	-0.01	0.00
40	->	20	-0.00	0.00
40	->	21	-0.00	0.00
40	->	22	0.02	0.00
40	->	23	-0.00	0.00
40	->	24	-0.00	0.00
40	->	25	0.03	0.01
40	->	26	-0.01	0.00
40	->	27	-0.00	0.00
40	->	28	-0.00	0.00
40	->	29	-0.00	0.00
40	->	30	0.00	0.00
40	->	31	0.39	0.42
40	->	32	-0.02	0.01
40	->	33	-0.28	0.08
40	->	34	-1.74	0.80
40	->	35	-2.25	0.94
40	->	36	-0.01	0.02
40	->	37	-0.19	0.13
40	->	38	-3.22	2.74
40	->	39	-9.51	0.67
40	->	40	-104.66	5.41
40	->	41	-15.85	0.68
40	->	42	-3.72	1.44
40	->	43	-0.14	0.08
40	->	44	0.02	0.01
40	->	45	-0.01	0.01
40	->	46	-0.04	0.01
40	->	47	0.02	0.03
40	->	48	-0.15	0.07
40	->	49	-0.01	0.00
40	->	50	-0.00	0.01
40	->	51	-0.93	0.70
40	->	52	-0.04	0.02
40	->	53	0.02	0.01
40	->	54	-0.01	0.01
40	->	55	-0.18	0.15
40	->	56	0.00	0.00
40	->	57	0.00	0.00
40	->	58	-0.02	0.00
40	->	59	0.02	0.01
40	->	60	0.00	0.00
40	->	61	-0.00	0.00
40	->	62	-0.00	0.00

40	->	63	-0.01	0.00
40	->	64	-0.00	0.00
40	->	65	0.00	0.00
40	->	66	-0.01	0.00
40	->	67	-0.00	0.00
40	->	68	0.01	0.00
40	->	69	0.01	0.00
40	->	70	0.00	0.00
40	->	71	-0.00	0.00
40	->	72	0.05	0.02
40	->	73	0.00	0.00
40	->	74	-0.03	0.01
40	->	75	-0.03	0.01
40	->	76	0.01	0.00
40	->	77	0.01	0.00
40	->	78	0.02	0.00
40	->	79	0.00	0.00
40	->	80	0.00	0.00
40	->	81	0.01	0.00
40	->	82	0.00	0.00
40	->	83	0.00	0.00
40	->	84	-0.00	0.00
40	->	85	-0.01	0.00
40	->	86	-0.03	0.01
40	->	87	-0.35	0.32
40	->	88	-4.87	3.17
40	->	89	-4.72	3.37
40	->	90	-0.08	0.02
40	->	91	-0.03	0.00
40	->	92	-0.01	0.00
40	->	93	-0.01	0.00
40	->	94	-0.00	0.00
40	->	95	-0.00	0.00
40	->	96	-0.00	0.00
40	->	97	-0.01	0.00
40	->	98	-0.01	0.00
40	->	99	-0.02	0.01
40	->	100	-0.01	0.00
40	->	101	-0.01	0.00
40	->	102	-0.01	0.00
40	->	103	-0.01	0.00
40	->	104	-0.02	0.00
40	->	105	-0.01	0.01
40	->	106	-0.03	0.01
41	->	1	0.00	0.00
41	->	2	0.00	0.00
41	->	3	0.00	0.00
41	->	4	-0.00	0.00
41	->	5	-0.00	0.00
41	->	6	0.00	0.00

41	->	7	0.00	0.00
41	->	8	-0.00	0.00
41	->	9	0.00	0.00
41	->	10	-0.00	0.00
41	->	11	-0.00	0.00
41	->	12	0.00	0.00
41	->	13	0.00	0.00
41	->	14	0.00	0.00
41	->	15	-0.00	0.00
41	->	16	0.00	0.00
41	->	17	-0.00	0.00
41	->	18	0.00	0.00
41	->	19	-0.00	0.00
41	->	20	0.00	0.00
41	->	21	0.00	0.00
41	->	22	0.00	0.00
41	->	23	-0.00	0.00
41	->	24	0.00	0.00
41	->	25	0.03	0.01
41	->	26	-0.01	0.01
41	->	27	-0.00	0.00
41	->	28	-0.00	0.00
41	->	29	-0.00	0.01
41	->	30	0.03	0.02
41	->	31	-2.04	0.69
41	->	32	-0.25	0.18
41	->	33	-2.65	0.72
41	->	34	-2.57	1.21
41	->	35	-0.39	0.24
41	->	36	-0.03	0.01
41	->	37	-0.01	0.01
41	->	38	0.00	0.03
41	->	39	-0.56	0.28
41	->	40	-15.82	0.67
41	->	41	-31.59	1.80
41	->	42	-8.58	0.39
41	->	43	-0.56	0.07
41	->	44	-0.02	0.03
41	->	45	-0.04	0.01
41	->	46	-0.02	0.01
41	->	47	0.04	0.03
41	->	48	-0.06	0.02
41	->	49	-0.01	0.00
41	->	50	-0.00	0.01
41	->	51	-0.02	0.01
41	->	52	-0.01	0.00
41	->	53	-0.00	0.00
41	->	54	-0.00	0.00
41	->	55	-0.01	0.01
41	->	56	-0.00	0.00

41	->	57	-0.00	0.00
41	->	58	0.00	0.00
41	->	59	-0.00	0.00
41	->	60	-0.00	0.00
41	->	61	0.00	0.00
41	->	62	0.00	0.00
41	->	63	0.00	0.00
41	->	64	0.00	0.00
41	->	65	0.00	0.00
41	->	66	0.00	0.00
41	->	67	0.00	0.00
41	->	68	-0.00	0.00
41	->	69	-0.00	0.00
41	->	70	0.00	0.00
41	->	71	0.00	0.00
41	->	72	-0.00	0.00
41	->	73	0.00	0.00
41	->	74	0.00	0.00
41	->	75	0.00	0.00
41	->	76	-0.00	0.00
41	->	77	0.00	0.00
41	->	78	-0.00	0.00
41	->	79	0.00	0.00
41	->	80	0.00	0.00
41	->	81	-0.00	0.00
41	->	82	-0.00	0.00
41	->	83	0.00	0.00
41	->	84	0.00	0.00
41	->	85	0.00	0.00
41	->	86	-0.00	0.00
41	->	87	-0.01	0.01
41	->	88	-0.06	0.02
41	->	89	-0.14	0.12
41	->	90	-0.03	0.02
41	->	91	-0.00	0.00
41	->	92	-0.00	0.00
41	->	93	-0.00	0.00
41	->	94	-0.00	0.00
41	->	95	-0.00	0.00
41	->	96	-0.00	0.00
41	->	97	-0.00	0.00
41	->	98	-0.00	0.00
41	->	99	-0.00	0.00
41	->	100	-0.00	0.00
41	->	101	-0.00	0.00
41	->	102	-0.00	0.00
41	->	103	-0.00	0.00
41	->	104	-0.00	0.00
41	->	105	-0.00	0.00
41	->	106	-0.00	0.00

42	->	1	0.00	0.00
42	->	2	0.00	0.00
42	->	3	-0.00	0.00
42	->	4	0.00	0.00
42	->	5	-0.00	0.00
42	->	6	0.00	0.00
42	->	7	0.00	0.00
42	->	8	-0.00	0.00
42	->	9	-0.00	0.00
42	->	10	-0.00	0.00
42	->	11	-0.00	0.00
42	->	12	0.00	0.00
42	->	13	0.00	0.00
42	->	14	-0.00	0.00
42	->	15	0.00	0.00
42	->	16	0.00	0.00
42	->	17	0.00	0.00
42	->	18	0.00	0.00
42	->	19	-0.00	0.00
42	->	20	0.00	0.00
42	->	21	0.00	0.00
42	->	22	0.01	0.01
42	->	23	-0.00	0.00
42	->	24	0.00	0.00
42	->	25	0.02	0.04
42	->	26	-0.02	0.01
42	->	27	-0.01	0.00
42	->	28	-0.00	0.00
42	->	29	0.00	0.01
42	->	30	-0.21	0.07
42	->	31	-3.15	0.40
42	->	32	-0.84	0.21
42	->	33	-3.43	0.41
42	->	34	-0.92	0.26
42	->	35	-0.59	0.18
42	->	36	-0.03	0.01
42	->	37	-0.03	0.02
42	->	38	-0.02	0.05
42	->	39	-0.10	0.02
42	->	40	-3.67	1.41
42	->	41	-8.66	0.39
42	->	42	28.85	1.08
42	->	43	-17.37	0.35
42	->	44	-1.02	0.18
42	->	45	-0.21	0.05
42	->	46	-0.11	0.03
42	->	47	-1.47	0.72
42	->	48	-2.88	0.52
42	->	49	-0.06	0.02
42	->	50	-0.08	0.03

42	->	51	-1.96	0.25
42	->	52	-0.08	0.04
42	->	53	-0.02	0.01
42	->	54	-0.02	0.01
42	->	55	0.00	0.02
42	->	56	-0.00	0.00
42	->	57	-0.00	0.00
42	->	58	-0.00	0.00
42	->	59	-0.00	0.00
42	->	60	-0.00	0.00
42	->	61	-0.00	0.00
42	->	62	-0.00	0.00
42	->	63	-0.00	0.00
42	->	64	-0.00	0.00
42	->	65	0.00	0.00
42	->	66	-0.00	0.00
42	->	67	0.00	0.00
42	->	68	0.00	0.00
42	->	69	0.00	0.00
42	->	70	-0.00	0.00
42	->	71	-0.00	0.00
42	->	72	-0.01	0.01
42	->	73	-0.00	0.00
42	->	74	-0.00	0.00
42	->	75	-0.00	0.00
42	->	76	0.00	0.00
42	->	77	0.00	0.00
42	->	78	0.00	0.00
42	->	79	0.00	0.00
42	->	80	0.00	0.00
42	->	81	0.00	0.00
42	->	82	0.00	0.00
42	->	83	0.00	0.00
42	->	84	-0.00	0.00
42	->	85	-0.00	0.00
42	->	86	-0.00	0.00
42	->	87	0.01	0.02
42	->	88	-0.79	0.47
42	->	89	-0.98	0.35
42	->	90	-0.22	0.09
42	->	91	-0.02	0.01
42	->	92	-0.00	0.00
42	->	93	-0.00	0.00
42	->	94	-0.00	0.00
42	->	95	-0.00	0.00
42	->	96	-0.00	0.00
42	->	97	-0.00	0.00
42	->	98	-0.01	0.00
42	->	99	-0.01	0.00
42	->	100	-0.00	0.00

42	->	101	-0.00	0.00
42	->	102	-0.00	0.00
42	->	103	-0.00	0.00
42	->	104	-0.00	0.00
42	->	105	-0.01	0.00
42	->	106	-0.00	0.00
43	->	1	0.00	0.00
43	->	2	0.00	0.00
43	->	3	-0.00	0.00
43	->	4	0.00	0.00
43	->	5	-0.00	0.00
43	->	6	-0.00	0.00
43	->	7	-0.00	0.00
43	->	8	-0.00	0.00
43	->	9	0.00	0.00
43	->	10	-0.00	0.00
43	->	11	-0.00	0.00
43	->	12	0.00	0.00
43	->	13	0.00	0.00
43	->	14	-0.00	0.00
43	->	15	-0.00	0.00
43	->	16	-0.00	0.00
43	->	17	-0.00	0.00
43	->	18	-0.00	0.00
43	->	19	-0.01	0.00
43	->	20	-0.01	0.00
43	->	21	-0.03	0.02
43	->	22	-0.14	0.10
43	->	23	-0.01	0.01
43	->	24	-0.02	0.01
43	->	25	-2.94	2.08
43	->	26	-0.47	0.45
43	->	27	-0.01	0.01
43	->	28	-0.02	0.01
43	->	29	-0.04	0.08
43	->	30	-0.95	0.69
43	->	31	-2.65	1.37
43	->	32	-0.72	0.27
43	->	33	-0.85	0.20
43	->	34	-0.04	0.01
43	->	35	0.01	0.01
43	->	36	-0.00	0.00
43	->	37	-0.00	0.00
43	->	38	0.00	0.00
43	->	39	-0.01	0.00
43	->	40	-0.14	0.08
43	->	41	-0.56	0.07
43	->	42	-17.35	0.35
43	->	43	23.73	1.67
43	->	44	-6.59	0.62

43	->	45	-0.22	0.06
43	->	46	-0.16	0.08
43	->	47	-0.71	0.77
43	->	48	-0.04	0.02
43	->	49	-0.00	0.00
43	->	50	-0.01	0.00
43	->	51	-0.01	0.00
43	->	52	-0.00	0.00
43	->	53	-0.00	0.00
43	->	54	-0.00	0.00
43	->	55	0.00	0.00
43	->	56	0.00	0.00
43	->	57	0.00	0.00
43	->	58	0.00	0.00
43	->	59	0.00	0.00
43	->	60	0.00	0.00
43	->	61	0.00	0.00
43	->	62	0.00	0.00
43	->	63	-0.00	0.00
43	->	64	-0.00	0.00
43	->	65	0.00	0.00
43	->	66	-0.00	0.00
43	->	67	0.00	0.00
43	->	68	0.00	0.00
43	->	69	0.00	0.00
43	->	70	0.00	0.00
43	->	71	0.00	0.00
43	->	72	0.00	0.00
43	->	73	-0.00	0.00
43	->	74	-0.00	0.00
43	->	75	-0.00	0.00
43	->	76	0.00	0.00
43	->	77	0.00	0.00
43	->	78	0.00	0.00
43	->	79	0.00	0.00
43	->	80	0.00	0.00
43	->	81	0.00	0.00
43	->	82	0.00	0.00
43	->	83	0.00	0.00
43	->	84	-0.00	0.00
43	->	85	-0.00	0.00
43	->	86	-0.00	0.00
43	->	87	-0.00	0.00
43	->	88	0.02	0.03
43	->	89	-0.83	0.50
43	->	90	-3.69	1.99
43	->	91	-0.05	0.02
43	->	92	-0.01	0.00
43	->	93	-0.00	0.00
43	->	94	0.00	0.00

43	->	95	-0.00	0.00
43	->	96	-0.00	0.00
43	->	97	-0.00	0.01
43	->	98	-0.01	0.01
43	->	99	-0.01	0.00
43	->	100	0.00	0.00
43	->	101	-0.00	0.00
43	->	102	-0.00	0.00
43	->	103	-0.00	0.00
43	->	104	-0.00	0.00
43	->	105	-0.00	0.00
43	->	106	-0.00	0.00
44	->	1	0.05	0.01
44	->	2	0.00	0.00
44	->	3	-0.00	0.00
44	->	4	-0.00	0.00
44	->	5	-0.03	0.00
44	->	6	0.00	0.00
44	->	7	-0.00	0.00
44	->	8	-0.01	0.00
44	->	9	-0.00	0.00
44	->	10	-0.03	0.01
44	->	11	-0.01	0.00
44	->	12	0.03	0.01
44	->	13	0.01	0.00
44	->	14	-0.01	0.00
44	->	15	0.00	0.00
44	->	16	0.07	0.01
44	->	17	-0.00	0.00
44	->	18	-0.00	0.02
44	->	19	-0.20	0.06
44	->	20	-0.01	0.01
44	->	21	0.03	0.02
44	->	22	-0.26	0.81
44	->	23	-0.05	0.02
44	->	24	-0.01	0.01
44	->	25	-0.43	0.35
44	->	26	-0.08	0.06
44	->	27	-0.01	0.00
44	->	28	-0.00	0.00
44	->	29	-0.01	0.00
44	->	30	-0.00	0.01
44	->	31	-0.08	0.05
44	->	32	-0.16	0.08
44	->	33	-2.32	0.61
44	->	34	-0.03	0.01
44	->	35	-0.05	0.01
44	->	36	0.01	0.00
44	->	37	-0.00	0.00
44	->	38	-0.02	0.00

44 ->	39	-0.00	0.00
44 ->	40	0.02	0.01
44 ->	41	-0.02	0.03
44 ->	42	-1.02	0.18
44 ->	43	-6.68	0.62
44 ->	44	-91.50	2.49
44 ->	45	-21.60	0.48
44 ->	46	-15.94	1.20
44 ->	47	-5.29	0.66
44 ->	48	-1.26	0.45
44 ->	49	0.02	0.06
44 ->	50	-0.09	0.04
44 ->	51	-0.01	0.01
44 ->	52	-0.00	0.00
44 ->	53	0.06	0.01
44 ->	54	-0.00	0.00
44 ->	55	-0.00	0.00
44 ->	56	0.00	0.00
44 ->	57	0.00	0.00
44 ->	58	-0.02	0.00
44 ->	59	0.02	0.00
44 ->	60	0.00	0.00
44 ->	61	-0.00	0.00
44 ->	62	-0.00	0.00
44 ->	63	-0.01	0.00
44 ->	64	-0.01	0.00
44 ->	65	0.00	0.00
44 ->	66	-0.01	0.00
44 ->	67	0.00	0.00
44 ->	68	0.01	0.00
44 ->	69	0.01	0.00
44 ->	70	0.00	0.00
44 ->	71	0.00	0.00
44 ->	72	0.13	0.04
44 ->	73	0.00	0.01
44 ->	74	-0.10	0.03
44 ->	75	-0.03	0.01
44 ->	76	0.02	0.00
44 ->	77	0.03	0.00
44 ->	78	0.03	0.00
44 ->	79	0.00	0.00
44 ->	80	0.00	0.00
44 ->	81	0.02	0.00
44 ->	82	0.00	0.00
44 ->	83	0.00	0.00
44 ->	84	-0.00	0.00
44 ->	85	-0.00	0.00
44 ->	86	-0.02	0.00
44 ->	87	-0.04	0.01
44 ->	88	-0.09	0.02

44	->	89	-0.48	0.26
44	->	90	-3.30	1.11
44	->	91	-8.96	1.04
44	->	92	-2.50	1.07
44	->	93	-0.09	0.06
44	->	94	-0.02	0.03
44	->	95	-0.02	0.01
44	->	96	-0.02	0.02
44	->	97	-0.12	0.06
44	->	98	-0.30	0.16
44	->	99	-0.60	0.21
44	->	100	1.08	0.64
44	->	101	0.36	0.48
44	->	102	-0.14	0.11
44	->	103	-0.07	0.03
44	->	104	-0.04	0.01
44	->	105	-0.02	0.00
44	->	106	-0.01	0.00
45	->	1	0.00	0.00
45	->	2	0.00	0.00
45	->	3	-0.00	0.00
45	->	4	-0.00	0.00
45	->	5	-0.00	0.00
45	->	6	0.00	0.00
45	->	7	-0.00	0.00
45	->	8	-0.00	0.00
45	->	9	0.00	0.00
45	->	10	-0.00	0.00
45	->	11	-0.00	0.00
45	->	12	0.00	0.00
45	->	13	0.00	0.00
45	->	14	-0.00	0.00
45	->	15	0.00	0.00
45	->	16	-0.00	0.00
45	->	17	-0.00	0.00
45	->	18	-0.01	0.00
45	->	19	-0.00	0.00
45	->	20	-0.00	0.00
45	->	21	-0.01	0.00
45	->	22	-0.07	0.06
45	->	23	0.00	0.00
45	->	24	-0.00	0.00
45	->	25	-0.23	0.17
45	->	26	0.00	0.00
45	->	27	0.00	0.00
45	->	28	-0.00	0.00
45	->	29	-0.00	0.00
45	->	30	-0.01	0.00
45	->	31	-0.07	0.02
45	->	32	-0.07	0.02

45	->	33	-2.28	0.39
45	->	34	-0.03	0.01
45	->	35	-0.00	0.01
45	->	36	-0.01	0.01
45	->	37	-0.00	0.00
45	->	38	0.00	0.00
45	->	39	-0.00	0.00
45	->	40	-0.01	0.01
45	->	41	-0.04	0.01
45	->	42	-0.21	0.05
45	->	43	-0.22	0.06
45	->	44	-21.61	0.48
45	->	45	21.87	2.36
45	->	46	-10.41	0.60
45	->	47	-1.01	0.20
45	->	48	-2.37	0.37
45	->	49	-2.73	1.32
45	->	50	-0.08	0.05
45	->	51	-0.06	0.04
45	->	52	-0.07	0.02
45	->	53	-0.02	0.01
45	->	54	-0.00	0.00
45	->	55	-0.00	0.00
45	->	56	-0.00	0.00
45	->	57	-0.00	0.00
45	->	58	0.00	0.00
45	->	59	-0.00	0.00
45	->	60	-0.00	0.00
45	->	61	-0.00	0.00
45	->	62	0.00	0.00
45	->	63	0.00	0.00
45	->	64	0.00	0.00
45	->	65	-0.00	0.00
45	->	66	0.00	0.00
45	->	67	0.00	0.00
45	->	68	-0.00	0.00
45	->	69	-0.00	0.00
45	->	70	0.00	0.00
45	->	71	-0.00	0.00
45	->	72	-0.00	0.00
45	->	73	-0.00	0.00
45	->	74	0.00	0.00
45	->	75	0.00	0.00
45	->	76	-0.00	0.00
45	->	77	-0.00	0.00
45	->	78	-0.00	0.00
45	->	79	-0.00	0.00
45	->	80	-0.00	0.00
45	->	81	-0.00	0.00
45	->	82	-0.00	0.00

45	->	83	0.00	0.00
45	->	84	0.00	0.00
45	->	85	0.00	0.00
45	->	86	0.00	0.00
45	->	87	-0.00	0.00
45	->	88	-0.00	0.00
45	->	89	0.00	0.01
45	->	90	0.01	0.01
45	->	91	-0.00	0.00
45	->	92	-0.00	0.00
45	->	93	-0.01	0.00
45	->	94	-0.01	0.01
45	->	95	-0.00	0.00
45	->	96	-0.01	0.02
45	->	97	-0.21	0.23
45	->	98	-1.69	1.67
45	->	99	-0.51	1.15
45	->	100	-0.02	0.02
45	->	101	-0.01	0.00
45	->	102	-0.00	0.00
45	->	103	-0.00	0.00
45	->	104	-0.00	0.00
45	->	105	-0.00	0.00
45	->	106	0.00	0.00
46	->	1	-0.03	0.01
46	->	2	-0.00	0.00
46	->	3	-0.00	0.00
46	->	4	0.00	0.00
46	->	5	0.02	0.00
46	->	6	-0.00	0.00
46	->	7	-0.00	0.00
46	->	8	0.00	0.00
46	->	9	0.00	0.00
46	->	10	0.02	0.00
46	->	11	0.01	0.00
46	->	12	-0.02	0.00
46	->	13	-0.01	0.00
46	->	14	-0.00	0.00
46	->	15	-0.00	0.00
46	->	16	-0.04	0.00
46	->	17	0.00	0.00
46	->	18	-0.01	0.01
46	->	19	0.06	0.01
46	->	20	-0.00	0.00
46	->	21	-0.02	0.01
46	->	22	-0.89	0.60
46	->	23	-0.00	0.00
46	->	24	-0.00	0.00
46	->	25	-0.19	0.10
46	->	26	-0.00	0.00

46	->	27	-0.00	0.00
46	->	28	-0.00	0.00
46	->	29	-0.00	0.00
46	->	30	0.00	0.00
46	->	31	-0.07	0.01
46	->	32	-0.02	0.00
46	->	33	0.01	0.02
46	->	34	-0.00	0.01
46	->	35	0.04	0.01
46	->	36	-0.04	0.01
46	->	37	-0.00	0.00
46	->	38	0.03	0.00
46	->	39	-0.00	0.00
46	->	40	-0.04	0.01
46	->	41	-0.02	0.01
46	->	42	-0.11	0.03
46	->	43	-0.16	0.08
46	->	44	-15.84	1.20
46	->	45	-10.48	0.60
46	->	46	-11.32	2.23
46	->	47	-13.02	0.59
46	->	48	-1.44	0.21
46	->	49	-2.76	0.54
46	->	50	-1.77	1.09
46	->	51	-0.14	0.09
46	->	52	-0.07	0.04
46	->	53	-0.19	0.04
46	->	54	-0.01	0.00
46	->	55	-0.01	0.00
46	->	56	-0.01	0.00
46	->	57	-0.00	0.00
46	->	58	0.03	0.01
46	->	59	-0.03	0.00
46	->	60	-0.01	0.00
46	->	61	-0.01	0.00
46	->	62	0.00	0.00
46	->	63	0.01	0.00
46	->	64	0.01	0.00
46	->	65	-0.00	0.00
46	->	66	0.01	0.00
46	->	67	0.00	0.00
46	->	68	-0.01	0.00
46	->	69	-0.02	0.00
46	->	70	-0.00	0.00
46	->	71	-0.01	0.01
46	->	72	-0.13	0.03
46	->	73	-0.01	0.00
46	->	74	0.06	0.01
46	->	75	0.04	0.01
46	->	76	-0.03	0.01

46	->	77	-0.03	0.01
46	->	78	-0.03	0.00
46	->	79	-0.00	0.00
46	->	80	-0.00	0.00
46	->	81	-0.02	0.00
46	->	82	-0.00	0.00
46	->	83	0.00	0.00
46	->	84	0.00	0.00
46	->	85	0.00	0.00
46	->	86	0.02	0.00
46	->	87	0.02	0.01
46	->	88	0.02	0.01
46	->	89	-0.06	0.10
46	->	90	0.20	0.15
46	->	91	0.47	0.14
46	->	92	0.25	0.18
46	->	93	-0.01	0.03
46	->	94	-0.01	0.02
46	->	95	0.01	0.00
46	->	96	0.01	0.01
46	->	97	0.06	0.05
46	->	98	-0.15	0.34
46	->	99	-1.57	0.43
46	->	100	-5.20	0.93
46	->	101	-0.62	0.32
46	->	102	-0.05	0.06
46	->	103	0.01	0.01
46	->	104	0.02	0.00
46	->	105	0.01	0.00
46	->	106	0.01	0.00
47	->	1	0.00	0.00
47	->	2	-0.00	0.00
47	->	3	-0.00	0.00
47	->	4	0.00	0.00
47	->	5	-0.00	0.00
47	->	6	0.00	0.00
47	->	7	0.00	0.00
47	->	8	-0.00	0.00
47	->	9	-0.00	0.00
47	->	10	-0.00	0.00
47	->	11	-0.00	0.00
47	->	12	0.00	0.00
47	->	13	0.00	0.00
47	->	14	-0.00	0.00
47	->	15	0.00	0.00
47	->	16	-0.00	0.00
47	->	17	-0.00	0.00
47	->	18	-0.01	0.00
47	->	19	-0.01	0.00
47	->	20	-0.00	0.00

47	->	21	-0.01	0.00
47	->	22	-0.12	0.05
47	->	23	-0.00	0.00
47	->	24	-0.00	0.00
47	->	25	-0.03	0.04
47	->	26	-0.01	0.01
47	->	27	-0.00	0.00
47	->	28	-0.00	0.00
47	->	29	-0.00	0.00
47	->	30	-0.00	0.00
47	->	31	-0.03	0.09
47	->	32	-0.01	0.01
47	->	33	-0.06	0.03
47	->	34	0.01	0.01
47	->	35	-0.12	0.04
47	->	36	-0.01	0.00
47	->	37	-0.00	0.01
47	->	38	-0.02	0.01
47	->	39	0.00	0.00
47	->	40	0.02	0.03
47	->	41	0.04	0.03
47	->	42	-1.48	0.72
47	->	43	-0.70	0.75
47	->	44	-5.21	0.66
47	->	45	-1.02	0.20
47	->	46	-13.08	0.59
47	->	47	21.27	1.15
47	->	48	-6.53	0.44
47	->	49	-0.94	0.17
47	->	50	-1.54	0.45
47	->	51	-1.60	0.62
47	->	52	-0.01	0.04
47	->	53	-0.03	0.03
47	->	54	-0.02	0.01
47	->	55	-0.04	0.01
47	->	56	-0.00	0.00
47	->	57	-0.00	0.00
47	->	58	0.00	0.00
47	->	59	-0.01	0.00
47	->	60	-0.00	0.00
47	->	61	-0.00	0.00
47	->	62	0.00	0.00
47	->	63	0.00	0.00
47	->	64	0.00	0.00
47	->	65	0.00	0.00
47	->	66	0.00	0.00
47	->	67	0.00	0.00
47	->	68	-0.00	0.00
47	->	69	-0.00	0.00
47	->	70	-0.00	0.00

47	->	71	-0.01	0.01
47	->	72	-0.18	0.10
47	->	73	-0.01	0.00
47	->	74	-0.00	0.01
47	->	75	-0.00	0.00
47	->	76	-0.00	0.00
47	->	77	-0.00	0.00
47	->	78	-0.00	0.00
47	->	79	-0.00	0.00
47	->	80	-0.00	0.00
47	->	81	0.00	0.00
47	->	82	-0.00	0.00
47	->	83	0.00	0.00
47	->	84	0.00	0.00
47	->	85	-0.00	0.00
47	->	86	-0.01	0.00
47	->	87	-0.06	0.02
47	->	88	-0.36	0.09
47	->	89	-3.90	0.60
47	->	90	-3.69	0.84
47	->	91	-0.48	0.28
47	->	92	-0.05	0.02
47	->	93	-0.01	0.00
47	->	94	-0.01	0.01
47	->	95	-0.00	0.00
47	->	96	-0.00	0.00
47	->	97	-0.00	0.01
47	->	98	0.01	0.05
47	->	99	-0.02	0.08
47	->	100	-0.27	0.09
47	->	101	-0.30	0.23
47	->	102	-0.11	0.09
47	->	103	-0.10	0.04
47	->	104	-0.02	0.01
47	->	105	-0.01	0.00
47	->	106	-0.00	0.00
48	->	1	0.00	0.00
48	->	2	0.00	0.00
48	->	3	0.00	0.00
48	->	4	0.00	0.00
48	->	5	-0.00	0.00
48	->	6	0.00	0.00
48	->	7	0.00	0.00
48	->	8	-0.00	0.00
48	->	9	0.00	0.00
48	->	10	-0.00	0.00
48	->	11	-0.00	0.00
48	->	12	0.00	0.00
48	->	13	0.00	0.00
48	->	14	0.00	0.00

48	->	15	0.00	0.00
48	->	16	0.00	0.00
48	->	17	-0.00	0.00
48	->	18	-0.00	0.00
48	->	19	-0.00	0.00
48	->	20	-0.00	0.00
48	->	21	-0.00	0.00
48	->	22	-0.01	0.00
48	->	23	-0.00	0.00
48	->	24	-0.00	0.00
48	->	25	-0.01	0.00
48	->	26	-0.00	0.00
48	->	27	0.00	0.00
48	->	28	-0.00	0.00
48	->	29	-0.00	0.00
48	->	30	-0.00	0.00
48	->	31	-0.03	0.01
48	->	32	-0.04	0.01
48	->	33	-1.26	0.27
48	->	34	-1.24	0.21
48	->	35	-1.08	0.23
48	->	36	-0.17	0.08
48	->	37	-0.03	0.01
48	->	38	-0.02	0.01
48	->	39	-0.02	0.01
48	->	40	-0.15	0.07
48	->	41	-0.06	0.02
48	->	42	-2.96	0.54
48	->	43	-0.04	0.02
48	->	44	-1.28	0.45
48	->	45	-2.39	0.37
48	->	46	-1.44	0.21
48	->	47	-6.59	0.45
48	->	48	22.71	1.52
48	->	49	-6.19	0.60
48	->	50	-0.88	0.14
48	->	51	-3.10	0.46
48	->	52	-3.91	0.77
48	->	53	-0.17	0.07
48	->	54	-0.07	0.03
48	->	55	-0.06	0.02
48	->	56	-0.02	0.01
48	->	57	-0.00	0.00
48	->	58	-0.00	0.00
48	->	59	-0.00	0.00
48	->	60	-0.00	0.00
48	->	61	-0.00	0.00
48	->	62	-0.00	0.00
48	->	63	0.00	0.00
48	->	64	0.00	0.00

48	->	65	-0.00	0.00
48	->	66	0.00	0.00
48	->	67	0.00	0.00
48	->	68	-0.00	0.00
48	->	69	-0.00	0.00
48	->	70	-0.00	0.00
48	->	71	-0.01	0.01
48	->	72	-0.02	0.01
48	->	73	-0.00	0.00
48	->	74	-0.00	0.00
48	->	75	0.00	0.00
48	->	76	-0.00	0.00
48	->	77	-0.00	0.00
48	->	78	-0.00	0.00
48	->	79	-0.00	0.00
48	->	80	-0.00	0.00
48	->	81	-0.00	0.00
48	->	82	-0.00	0.00
48	->	83	0.00	0.00
48	->	84	0.00	0.00
48	->	85	-0.00	0.00
48	->	86	-0.00	0.00
48	->	87	0.01	0.01
48	->	88	0.00	0.03
48	->	89	-0.03	0.03
48	->	90	0.00	0.01
48	->	91	-0.00	0.00
48	->	92	-0.00	0.00
48	->	93	-0.00	0.00
48	->	94	-0.00	0.00
48	->	95	-0.00	0.00
48	->	96	-0.00	0.00
48	->	97	-0.02	0.01
48	->	98	-0.09	0.06
48	->	99	-0.16	0.12
48	->	100	-0.02	0.01
48	->	101	-0.01	0.00
48	->	102	-0.00	0.00
48	->	103	-0.00	0.00
48	->	104	-0.00	0.00
48	->	105	-0.00	0.00
48	->	106	-0.00	0.00
49	->	1	0.00	0.00
49	->	2	0.00	0.00
49	->	3	0.00	0.00
49	->	4	0.00	0.00
49	->	5	-0.00	0.00
49	->	6	0.00	0.00
49	->	7	0.00	0.00
49	->	8	-0.00	0.00

49	->	9	0.00	0.00
49	->	10	-0.00	0.00
49	->	11	-0.00	0.00
49	->	12	0.00	0.00
49	->	13	0.00	0.00
49	->	14	0.00	0.00
49	->	15	0.00	0.00
49	->	16	0.00	0.00
49	->	17	-0.00	0.00
49	->	18	-0.00	0.00
49	->	19	-0.00	0.00
49	->	20	-0.00	0.00
49	->	21	-0.00	0.00
49	->	22	0.00	0.00
49	->	23	0.00	0.00
49	->	24	0.00	0.00
49	->	25	0.00	0.00
49	->	26	-0.00	0.00
49	->	27	0.00	0.00
49	->	28	0.00	0.00
49	->	29	0.00	0.00
49	->	30	-0.00	0.00
49	->	31	-0.00	0.00
49	->	32	-0.00	0.00
49	->	33	-0.04	0.01
49	->	34	-0.01	0.01
49	->	35	0.02	0.02
49	->	36	-0.04	0.03
49	->	37	-0.01	0.00
49	->	38	0.00	0.00
49	->	39	-0.00	0.00
49	->	40	-0.01	0.00
49	->	41	-0.01	0.00
49	->	42	-0.06	0.02
49	->	43	-0.00	0.00
49	->	44	0.02	0.06
49	->	45	-2.74	1.31
49	->	46	-2.78	0.54
49	->	47	-0.95	0.17
49	->	48	-6.20	0.60
49	->	49	-19.69	1.85
49	->	50	-8.01	0.47
49	->	51	-0.88	0.17
49	->	52	-2.87	0.48
49	->	53	-3.39	0.68
49	->	54	-0.14	0.06
49	->	55	-0.05	0.02
49	->	56	-0.03	0.02
49	->	57	-0.01	0.00
49	->	58	-0.03	0.03

49	->	59	-0.00	0.01
49	->	60	-0.02	0.01
49	->	61	-0.02	0.01
49	->	62	-0.00	0.00
49	->	63	0.00	0.00
49	->	64	0.00	0.00
49	->	65	0.00	0.00
49	->	66	0.00	0.00
49	->	67	0.00	0.00
49	->	68	-0.00	0.00
49	->	69	0.00	0.00
49	->	70	0.00	0.00
49	->	71	-0.06	0.05
49	->	72	0.06	0.03
49	->	73	-0.01	0.01
49	->	74	-0.02	0.01
49	->	75	-0.01	0.01
49	->	76	-0.00	0.00
49	->	77	-0.00	0.00
49	->	78	-0.00	0.00
49	->	79	-0.00	0.00
49	->	80	0.00	0.00
49	->	81	0.00	0.00
49	->	82	-0.00	0.00
49	->	83	0.00	0.00
49	->	84	-0.00	0.00
49	->	85	-0.00	0.00
49	->	86	-0.01	0.01
49	->	87	-0.04	0.02
49	->	88	-0.07	0.04
49	->	89	-0.02	0.01
49	->	90	-0.01	0.00
49	->	91	-0.01	0.00
49	->	92	-0.00	0.00
49	->	93	-0.00	0.00
49	->	94	-0.00	0.00
49	->	95	-0.00	0.00
49	->	96	-0.00	0.00
49	->	97	-0.01	0.00
49	->	98	-0.18	0.20
49	->	99	-2.03	2.12
49	->	100	-0.19	0.09
49	->	101	-0.01	0.00
49	->	102	-0.00	0.00
49	->	103	-0.00	0.00
49	->	104	-0.00	0.00
49	->	105	-0.00	0.00
49	->	106	-0.00	0.00
50	->	1	0.00	0.00
50	->	2	0.00	0.00

50	->	3	-0.00	0.00
50	->	4	0.00	0.00
50	->	5	-0.00	0.00
50	->	6	0.00	0.00
50	->	7	0.00	0.00
50	->	8	-0.00	0.00
50	->	9	0.00	0.00
50	->	10	-0.00	0.00
50	->	11	-0.00	0.00
50	->	12	0.00	0.00
50	->	13	0.00	0.00
50	->	14	0.00	0.00
50	->	15	0.00	0.00
50	->	16	0.00	0.00
50	->	17	-0.00	0.00
50	->	18	-0.00	0.00
50	->	19	-0.00	0.00
50	->	20	-0.00	0.00
50	->	21	-0.00	0.00
50	->	22	-0.00	0.00
50	->	23	0.00	0.00
50	->	24	0.00	0.00
50	->	25	0.00	0.00
50	->	26	-0.00	0.00
50	->	27	0.00	0.00
50	->	28	0.00	0.00
50	->	29	0.00	0.00
50	->	30	-0.00	0.00
50	->	31	-0.00	0.00
50	->	32	-0.00	0.00
50	->	33	-0.01	0.00
50	->	34	-0.00	0.00
50	->	35	-0.04	0.02
50	->	36	-0.01	0.01
50	->	37	-0.01	0.00
50	->	38	-0.01	0.01
50	->	39	-0.00	0.00
50	->	40	-0.00	0.01
50	->	41	-0.00	0.01
50	->	42	-0.08	0.03
50	->	43	-0.01	0.00
50	->	44	-0.09	0.04
50	->	45	-0.08	0.05
50	->	46	-1.79	1.08
50	->	47	-1.54	0.45
50	->	48	-0.89	0.14
50	->	49	-8.04	0.47
50	->	50	-21.91	1.71
50	->	51	-22.12	0.48
50	->	52	-0.93	0.20

50	->	53	-3.31	0.54
50	->	54	-1.35	0.91
50	->	55	-0.11	0.04
50	->	56	-0.04	0.02
50	->	57	-0.02	0.01
50	->	58	-0.02	0.02
50	->	59	-0.05	0.04
50	->	60	-0.03	0.02
50	->	61	-0.03	0.02
50	->	62	-0.00	0.00
50	->	63	-0.00	0.00
50	->	64	0.00	0.00
50	->	65	0.00	0.00
50	->	66	0.00	0.00
50	->	67	0.00	0.00
50	->	68	-0.00	0.00
50	->	69	-0.01	0.01
50	->	70	-0.03	0.02
50	->	71	-0.71	0.51
50	->	72	-1.54	0.44
50	->	73	-0.03	0.02
50	->	74	-0.03	0.02
50	->	75	-0.04	0.03
50	->	76	-0.00	0.00
50	->	77	-0.00	0.00
50	->	78	-0.00	0.00
50	->	79	-0.00	0.00
50	->	80	-0.00	0.00
50	->	81	0.00	0.00
50	->	82	0.00	0.00
50	->	83	0.00	0.00
50	->	84	-0.00	0.00
50	->	85	-0.00	0.00
50	->	86	-0.01	0.02
50	->	87	-0.15	0.10
50	->	88	-0.38	0.21
50	->	89	-0.37	0.20
50	->	90	-0.01	0.01
50	->	91	-0.01	0.00
50	->	92	-0.01	0.00
50	->	93	-0.00	0.00
50	->	94	-0.00	0.00
50	->	95	-0.00	0.00
50	->	96	-0.00	0.00
50	->	97	-0.00	0.00
50	->	98	0.00	0.01
50	->	99	-0.04	0.06
50	->	100	-0.10	0.07
50	->	101	-0.04	0.02
50	->	102	-0.02	0.01

50	->	103	-0.02	0.01
50	->	104	-0.01	0.00
50	->	105	-0.00	0.00
50	->	106	-0.00	0.00
51	->	1	0.00	0.00
51	->	2	0.00	0.00
51	->	3	0.00	0.00
51	->	4	0.00	0.00
51	->	5	-0.00	0.00
51	->	6	0.00	0.00
51	->	7	0.00	0.00
51	->	8	-0.00	0.00
51	->	9	-0.00	0.00
51	->	10	-0.00	0.00
51	->	11	-0.00	0.00
51	->	12	0.00	0.00
51	->	13	0.00	0.00
51	->	14	0.00	0.00
51	->	15	0.00	0.00
51	->	16	0.00	0.00
51	->	17	-0.00	0.00
51	->	18	-0.00	0.00
51	->	19	-0.00	0.00
51	->	20	-0.00	0.00
51	->	21	-0.00	0.00
51	->	22	0.00	0.00
51	->	23	0.00	0.00
51	->	24	0.00	0.00
51	->	25	0.00	0.00
51	->	26	-0.00	0.00
51	->	27	0.00	0.00
51	->	28	0.00	0.00
51	->	29	0.00	0.00
51	->	30	-0.00	0.00
51	->	31	-0.00	0.00
51	->	32	-0.00	0.00
51	->	33	-0.02	0.00
51	->	34	-0.09	0.03
51	->	35	2.34	0.78
51	->	36	-0.13	0.05
51	->	37	0.38	0.53
51	->	38	2.60	0.95
51	->	39	-0.06	0.05
51	->	40	-0.92	0.68
51	->	41	-0.02	0.01
51	->	42	-1.98	0.25
51	->	43	-0.01	0.00
51	->	44	-0.01	0.01
51	->	45	-0.06	0.04
51	->	46	-0.14	0.09

51	->	47	-1.63	0.63
51	->	48	-3.10	0.46
51	->	49	-0.88	0.17
51	->	50	-22.18	0.49
51	->	51	10.86	1.50
51	->	52	-10.31	0.53
51	->	53	-1.30	0.28
51	->	54	-3.16	1.11
51	->	55	16.01	8.76
51	->	56	-0.19	0.06
51	->	57	-0.03	0.02
51	->	58	-0.02	0.01
51	->	59	-0.02	0.01
51	->	60	-0.01	0.00
51	->	61	-0.00	0.00
51	->	62	-0.00	0.00
51	->	63	-0.00	0.00
51	->	64	-0.00	0.00
51	->	65	0.00	0.00
51	->	66	-0.00	0.00
51	->	67	0.00	0.00
51	->	68	-0.00	0.00
51	->	69	-0.00	0.00
51	->	70	-0.01	0.01
51	->	71	-0.04	0.03
51	->	72	-0.09	0.03
51	->	73	0.00	0.00
51	->	74	-0.00	0.00
51	->	75	-0.01	0.01
51	->	76	0.00	0.00
51	->	77	0.00	0.00
51	->	78	0.00	0.00
51	->	79	-0.00	0.00
51	->	80	0.00	0.00
51	->	81	0.00	0.00
51	->	82	0.00	0.00
51	->	83	0.00	0.00
51	->	84	-0.00	0.00
51	->	85	-0.00	0.00
51	->	86	-0.03	0.01
51	->	87	-0.87	0.36
51	->	88	-5.25	2.10
51	->	89	-0.08	0.02
51	->	90	-0.01	0.00
51	->	91	-0.01	0.00
51	->	92	-0.00	0.00
51	->	93	-0.00	0.00
51	->	94	-0.00	0.00
51	->	95	-0.00	0.00
51	->	96	-0.00	0.00

51	->	97	-0.00	0.00
51	->	98	-0.01	0.00
51	->	99	-0.02	0.01
51	->	100	-0.01	0.00
51	->	101	-0.00	0.00
51	->	102	-0.00	0.00
51	->	103	-0.01	0.00
51	->	104	-0.01	0.00
51	->	105	-0.01	0.00
51	->	106	-0.01	0.00
52	->	1	0.00	0.00
52	->	2	0.00	0.00
52	->	3	0.00	0.00
52	->	4	0.00	0.00
52	->	5	-0.00	0.00
52	->	6	0.00	0.00
52	->	7	0.00	0.00
52	->	8	-0.00	0.00
52	->	9	-0.00	0.00
52	->	10	-0.00	0.00
52	->	11	-0.00	0.00
52	->	12	0.00	0.00
52	->	13	0.00	0.00
52	->	14	0.00	0.00
52	->	15	0.00	0.00
52	->	16	0.00	0.00
52	->	17	-0.00	0.00
52	->	18	-0.00	0.00
52	->	19	-0.00	0.00
52	->	20	-0.00	0.00
52	->	21	-0.00	0.00
52	->	22	0.00	0.00
52	->	23	0.00	0.00
52	->	24	-0.00	0.00
52	->	25	0.00	0.00
52	->	26	0.00	0.00
52	->	27	0.00	0.00
52	->	28	0.00	0.00
52	->	29	0.00	0.00
52	->	30	-0.00	0.00
52	->	31	-0.00	0.00
52	->	32	-0.00	0.00
52	->	33	-0.02	0.00
52	->	34	-0.06	0.03
52	->	35	-0.47	0.32
52	->	36	-1.21	1.18
52	->	37	-0.24	0.30
52	->	38	0.04	0.02
52	->	39	-0.01	0.01
52	->	40	-0.04	0.02

52	->	41	-0.01	0.00
52	->	42	-0.08	0.04
52	->	43	-0.00	0.00
52	->	44	-0.00	0.00
52	->	45	-0.07	0.02
52	->	46	-0.07	0.04
52	->	47	-0.01	0.04
52	->	48	-3.88	0.77
52	->	49	-2.89	0.47
52	->	50	-0.94	0.20
52	->	51	-10.36	0.54
52	->	52	23.84	1.70
52	->	53	-10.23	0.50
52	->	54	-1.09	0.20
52	->	55	-3.04	0.67
52	->	56	-4.59	1.53
52	->	57	-0.17	0.08
52	->	58	-0.27	0.12
52	->	59	-0.01	0.01
52	->	60	-0.01	0.00
52	->	61	-0.01	0.00
52	->	62	-0.00	0.00
52	->	63	0.00	0.00
52	->	64	0.00	0.00
52	->	65	0.00	0.00
52	->	66	0.00	0.00
52	->	67	0.00	0.00
52	->	68	-0.00	0.00
52	->	69	-0.00	0.00
52	->	70	0.00	0.00
52	->	71	-0.02	0.01
52	->	72	-0.02	0.01
52	->	73	-0.00	0.00
52	->	74	-0.00	0.00
52	->	75	-0.00	0.00
52	->	76	-0.00	0.00
52	->	77	0.00	0.00
52	->	78	-0.00	0.00
52	->	79	-0.00	0.00
52	->	80	0.00	0.00
52	->	81	0.00	0.00
52	->	82	0.00	0.00
52	->	83	0.00	0.00
52	->	84	0.00	0.00
52	->	85	-0.00	0.00
52	->	86	-0.00	0.00
52	->	87	0.01	0.02
52	->	88	0.07	0.04
52	->	89	-0.01	0.00
52	->	90	-0.00	0.00

52	->	91	-0.00	0.00
52	->	92	-0.00	0.00
52	->	93	-0.00	0.00
52	->	94	-0.00	0.00
52	->	95	-0.00	0.00
52	->	96	-0.00	0.00
52	->	97	-0.01	0.00
52	->	98	-0.02	0.01
52	->	99	-0.05	0.03
52	->	100	-0.02	0.01
52	->	101	-0.00	0.00
52	->	102	-0.00	0.00
52	->	103	-0.00	0.00
52	->	104	-0.00	0.00
52	->	105	-0.00	0.00
52	->	106	-0.00	0.00
53	->	1	0.01	0.00
53	->	2	0.00	0.00
53	->	3	-0.00	0.00
53	->	4	-0.00	0.00
53	->	5	-0.00	0.00
53	->	6	0.00	0.00
53	->	7	0.00	0.00
53	->	8	-0.00	0.00
53	->	9	-0.00	0.00
53	->	10	-0.01	0.00
53	->	11	-0.00	0.00
53	->	12	0.01	0.00
53	->	13	0.00	0.00
53	->	14	0.00	0.00
53	->	15	0.00	0.00
53	->	16	0.01	0.00
53	->	17	-0.00	0.00
53	->	18	-0.00	0.00
53	->	19	-0.02	0.00
53	->	20	-0.00	0.00
53	->	21	0.00	0.00
53	->	22	0.03	0.01
53	->	23	0.00	0.00
53	->	24	0.00	0.00
53	->	25	0.02	0.00
53	->	26	0.00	0.00
53	->	27	0.00	0.00
53	->	28	0.00	0.00
53	->	29	0.00	0.00
53	->	30	-0.00	0.00
53	->	31	0.02	0.00
53	->	32	-0.00	0.00
53	->	33	-0.00	0.00
53	->	34	-0.01	0.00

53	->	35	-0.06	0.02
53	->	36	0.01	0.01
53	->	37	-0.01	0.01
53	->	38	-0.04	0.01
53	->	39	-0.00	0.00
53	->	40	0.02	0.01
53	->	41	-0.00	0.00
53	->	42	-0.02	0.01
53	->	43	-0.00	0.00
53	->	44	0.06	0.01
53	->	45	-0.02	0.01
53	->	46	-0.19	0.04
53	->	47	-0.03	0.03
53	->	48	-0.17	0.07
53	->	49	-3.44	0.69
53	->	50	-3.35	0.54
53	->	51	-1.31	0.28
53	->	52	-10.26	0.51
53	->	53	-101.57	3.11
53	->	54	-5.42	0.58
53	->	55	-0.71	0.21
53	->	56	-1.55	0.32
53	->	57	-1.23	0.43
53	->	58	-4.01	1.45
53	->	59	-0.05	0.37
53	->	60	-1.17	0.71
53	->	61	-0.51	0.32
53	->	62	-0.01	0.01
53	->	63	-0.03	0.01
53	->	64	-0.02	0.00
53	->	65	0.00	0.00
53	->	66	-0.02	0.00
53	->	67	-0.00	0.00
53	->	68	0.03	0.01
53	->	69	0.10	0.05
53	->	70	0.03	0.05
53	->	71	-3.34	2.25
53	->	72	-0.24	0.21
53	->	73	-0.14	0.10
53	->	74	-0.11	0.03
53	->	75	-0.18	0.09
53	->	76	0.04	0.02
53	->	77	0.03	0.01
53	->	78	0.03	0.00
53	->	79	0.00	0.00
53	->	80	0.00	0.00
53	->	81	0.02	0.00
53	->	82	0.00	0.00
53	->	83	-0.00	0.00
53	->	84	-0.01	0.00

53	->	85	-0.01	0.00
53	->	86	-0.13	0.08
53	->	87	-0.37	0.19
53	->	88	-0.14	0.06
53	->	89	-0.04	0.00
53	->	90	-0.03	0.00
53	->	91	-0.02	0.00
53	->	92	-0.01	0.00
53	->	93	-0.02	0.00
53	->	94	-0.01	0.00
53	->	95	-0.01	0.00
53	->	96	-0.01	0.00
53	->	97	-0.03	0.01
53	->	98	-0.12	0.06
53	->	99	-2.19	1.01
53	->	100	-7.85	2.27
53	->	101	-0.08	0.03
53	->	102	-0.02	0.00
53	->	103	-0.02	0.00
53	->	104	-0.01	0.00
53	->	105	-0.01	0.00
53	->	106	-0.01	0.00
54	->	1	0.00	0.00
54	->	2	0.00	0.00
54	->	3	0.00	0.00
54	->	4	0.00	0.00
54	->	5	-0.00	0.00
54	->	6	0.00	0.00
54	->	7	0.00	0.00
54	->	8	-0.00	0.00
54	->	9	0.00	0.00
54	->	10	-0.00	0.00
54	->	11	-0.00	0.00
54	->	12	0.00	0.00
54	->	13	0.00	0.00
54	->	14	0.00	0.00
54	->	15	0.00	0.00
54	->	16	0.00	0.00
54	->	17	-0.00	0.00
54	->	18	-0.00	0.00
54	->	19	-0.00	0.00
54	->	20	-0.00	0.00
54	->	21	0.00	0.00
54	->	22	0.00	0.00
54	->	23	0.00	0.00
54	->	24	-0.00	0.00
54	->	25	0.00	0.00
54	->	26	0.00	0.00
54	->	27	0.00	0.00
54	->	28	0.00	0.00

54	->	29	0.00	0.00
54	->	30	-0.00	0.00
54	->	31	0.00	0.00
54	->	32	0.00	0.00
54	->	33	-0.00	0.00
54	->	34	-0.00	0.00
54	->	35	-0.07	0.04
54	->	36	-0.00	0.01
54	->	37	-0.03	0.01
54	->	38	-0.05	0.03
54	->	39	-0.00	0.00
54	->	40	-0.01	0.01
54	->	41	-0.00	0.00
54	->	42	-0.02	0.01
54	->	43	-0.00	0.00
54	->	44	-0.00	0.00
54	->	45	-0.00	0.00
54	->	46	-0.01	0.00
54	->	47	-0.02	0.01
54	->	48	-0.07	0.03
54	->	49	-0.14	0.06
54	->	50	-1.37	0.90
54	->	51	-3.16	1.10
54	->	52	-1.10	0.20
54	->	53	-5.45	0.57
54	->	54	-23.62	1.66
54	->	55	-22.44	0.41
54	->	56	-0.61	0.14
54	->	57	-0.94	0.33
54	->	58	-0.21	0.15
54	->	59	-0.46	0.72
54	->	60	-0.02	0.01
54	->	61	-0.00	0.00
54	->	62	-0.00	0.00
54	->	63	0.00	0.00
54	->	64	0.00	0.00
54	->	65	0.00	0.00
54	->	66	0.00	0.00
54	->	67	-0.00	0.00
54	->	68	-0.01	0.01
54	->	69	-0.03	0.03
54	->	70	-0.06	0.06
54	->	71	-0.42	0.32
54	->	72	-0.09	0.05
54	->	73	0.00	0.00
54	->	74	-0.00	0.00
54	->	75	0.03	0.07
54	->	76	-0.00	0.00
54	->	77	0.00	0.00
54	->	78	-0.00	0.00

54	->	79	-0.00	0.00
54	->	80	0.00	0.00
54	->	81	0.00	0.00
54	->	82	-0.00	0.00
54	->	83	0.00	0.00
54	->	84	-0.00	0.00
54	->	85	-0.00	0.00
54	->	86	-0.06	0.10
54	->	87	-1.98	1.03
54	->	88	-0.30	0.45
54	->	89	-0.01	0.00
54	->	90	-0.00	0.00
54	->	91	-0.00	0.00
54	->	92	-0.00	0.00
54	->	93	-0.00	0.00
54	->	94	-0.00	0.00
54	->	95	-0.00	0.00
54	->	96	-0.00	0.00
54	->	97	-0.00	0.00
54	->	98	-0.00	0.00
54	->	99	-0.00	0.00
54	->	100	-0.00	0.00
54	->	101	-0.00	0.00
54	->	102	-0.00	0.00
54	->	103	-0.00	0.00
54	->	104	-0.00	0.00
54	->	105	-0.00	0.00
54	->	106	-0.00	0.00
55	->	1	0.00	0.00
55	->	2	0.00	0.00
55	->	3	0.00	0.00
55	->	4	0.00	0.00
55	->	5	0.00	0.00
55	->	6	0.00	0.00
55	->	7	0.00	0.00
55	->	8	0.00	0.00
55	->	9	0.00	0.00
55	->	10	0.00	0.00
55	->	11	-0.00	0.00
55	->	12	-0.00	0.00
55	->	13	0.00	0.00
55	->	14	0.00	0.00
55	->	15	0.00	0.00
55	->	16	0.00	0.00
55	->	17	0.00	0.00
55	->	18	0.00	0.00
55	->	19	-0.00	0.00
55	->	20	0.00	0.00
55	->	21	0.00	0.00
55	->	22	0.00	0.00

55	->	23	0.00	0.00
55	->	24	0.00	0.00
55	->	25	-0.00	0.00
55	->	26	0.00	0.00
55	->	27	-0.00	0.00
55	->	28	0.00	0.00
55	->	29	0.00	0.00
55	->	30	0.00	0.00
55	->	31	-0.00	0.00
55	->	32	-0.00	0.00
55	->	33	-0.01	0.00
55	->	34	-0.06	0.01
55	->	35	2.70	0.56
55	->	36	-0.36	0.18
55	->	37	-0.36	1.53
55	->	38	2.46	0.73
55	->	39	-0.02	0.04
55	->	40	-0.18	0.15
55	->	41	-0.01	0.01
55	->	42	0.00	0.02
55	->	43	0.00	0.00
55	->	44	-0.00	0.00
55	->	45	-0.00	0.00
55	->	46	-0.01	0.00
55	->	47	-0.04	0.01
55	->	48	-0.06	0.02
55	->	49	-0.05	0.02
55	->	50	-0.11	0.04
55	->	51	15.99	8.76
55	->	52	-3.02	0.67
55	->	53	-0.72	0.21
55	->	54	-22.54	0.41
55	->	55	4.19	1.35
55	->	56	-4.92	0.39
55	->	57	-0.50	0.12
55	->	58	-0.05	0.03
55	->	59	-0.06	0.04
55	->	60	-0.00	0.00
55	->	61	-0.00	0.00
55	->	62	-0.00	0.00
55	->	63	-0.00	0.00
55	->	64	-0.00	0.00
55	->	65	0.00	0.00
55	->	66	-0.00	0.00
55	->	67	-0.00	0.00
55	->	68	-0.00	0.00
55	->	69	-0.01	0.00
55	->	70	-0.01	0.00
55	->	71	-0.01	0.01
55	->	72	-0.01	0.00

55	->	73	-0.00	0.00
55	->	74	-0.00	0.00
55	->	75	-0.00	0.00
55	->	76	0.00	0.00
55	->	77	0.00	0.00
55	->	78	0.00	0.00
55	->	79	0.00	0.00
55	->	80	0.00	0.00
55	->	81	0.00	0.00
55	->	82	0.00	0.00
55	->	83	-0.00	0.00
55	->	84	-0.00	0.00
55	->	85	-0.00	0.00
55	->	86	0.01	0.01
55	->	87	0.05	0.03
55	->	88	0.05	0.04
55	->	89	-0.00	0.00
55	->	90	-0.00	0.00
55	->	91	-0.00	0.00
55	->	92	-0.00	0.00
55	->	93	-0.00	0.00
55	->	94	-0.00	0.00
55	->	95	-0.00	0.00
55	->	96	0.00	0.00
55	->	97	-0.00	0.00
55	->	98	-0.00	0.00
55	->	99	-0.00	0.00
55	->	100	-0.00	0.00
55	->	101	-0.00	0.00
55	->	102	-0.00	0.00
55	->	103	-0.00	0.00
55	->	104	-0.00	0.00
55	->	105	-0.00	0.00
55	->	106	-0.00	0.00
56	->	1	0.00	0.00
56	->	2	0.00	0.00
56	->	3	0.00	0.00
56	->	4	0.00	0.00
56	->	5	-0.00	0.00
56	->	6	0.00	0.00
56	->	7	0.00	0.00
56	->	8	-0.00	0.00
56	->	9	0.00	0.00
56	->	10	-0.00	0.00
56	->	11	-0.00	0.00
56	->	12	0.00	0.00
56	->	13	0.00	0.00
56	->	14	0.00	0.00
56	->	15	0.00	0.00
56	->	16	0.00	0.00

56 ->	17	-0.00	0.00
56 ->	18	-0.00	0.00
56 ->	19	-0.00	0.00
56 ->	20	0.00	0.00
56 ->	21	0.00	0.00
56 ->	22	0.00	0.00
56 ->	23	0.00	0.00
56 ->	24	-0.00	0.00
56 ->	25	0.00	0.00
56 ->	26	0.00	0.00
56 ->	27	0.00	0.00
56 ->	28	-0.00	0.00
56 ->	29	-0.00	0.00
56 ->	30	-0.00	0.00
56 ->	31	0.00	0.00
56 ->	32	-0.00	0.00
56 ->	33	-0.00	0.00
56 ->	34	-0.00	0.00
56 ->	35	-0.02	0.01
56 ->	36	-0.21	0.30
56 ->	37	-0.13	0.20
56 ->	38	-0.01	0.00
56 ->	39	-0.00	0.00
56 ->	40	0.00	0.00
56 ->	41	-0.00	0.00
56 ->	42	-0.00	0.00
56 ->	43	0.00	0.00
56 ->	44	0.00	0.00
56 ->	45	-0.00	0.00
56 ->	46	-0.01	0.00
56 ->	47	-0.00	0.00
56 ->	48	-0.02	0.01
56 ->	49	-0.03	0.02
56 ->	50	-0.04	0.02
56 ->	51	-0.19	0.06
56 ->	52	-4.59	1.52
56 ->	53	-1.56	0.33
56 ->	54	-0.62	0.14
56 ->	55	-5.08	0.39
56 ->	56	-23.98	2.29
56 ->	57	-11.64	0.43
56 ->	58	-2.41	0.93
56 ->	59	-0.04	0.03
56 ->	60	-0.01	0.00
56 ->	61	-0.00	0.00
56 ->	62	-0.00	0.00
56 ->	63	0.00	0.00
56 ->	64	0.00	0.00
56 ->	65	0.00	0.00
56 ->	66	0.00	0.00

56	->	67	0.00	0.00
56	->	68	-0.00	0.00
56	->	69	-0.01	0.00
56	->	70	-0.01	0.00
56	->	71	-0.02	0.00
56	->	72	-0.00	0.00
56	->	73	-0.00	0.00
56	->	74	-0.00	0.00
56	->	75	-0.00	0.00
56	->	76	-0.00	0.00
56	->	77	0.00	0.00
56	->	78	0.00	0.00
56	->	79	0.00	0.00
56	->	80	0.00	0.00
56	->	81	0.00	0.00
56	->	82	0.00	0.00
56	->	83	0.00	0.00
56	->	84	-0.00	0.00
56	->	85	-0.00	0.00
56	->	86	-0.01	0.00
56	->	87	-0.02	0.01
56	->	88	-0.01	0.00
56	->	89	-0.00	0.00
56	->	90	-0.00	0.00
56	->	91	-0.00	0.00
56	->	92	-0.00	0.00
56	->	93	-0.00	0.00
56	->	94	-0.00	0.00
56	->	95	-0.00	0.00
56	->	96	-0.00	0.00
56	->	97	-0.00	0.00
56	->	98	-0.00	0.00
56	->	99	-0.01	0.00
56	->	100	-0.00	0.00
56	->	101	-0.00	0.00
56	->	102	-0.00	0.00
56	->	103	-0.00	0.00
56	->	104	-0.00	0.00
56	->	105	-0.00	0.00
56	->	106	-0.00	0.00
57	->	1	0.00	0.00
57	->	2	0.00	0.00
57	->	3	0.00	0.00
57	->	4	0.00	0.00
57	->	5	-0.00	0.00
57	->	6	0.00	0.00
57	->	7	0.00	0.00
57	->	8	-0.00	0.00
57	->	9	0.00	0.00
57	->	10	-0.00	0.00

57	->	11	-0.00	0.00
57	->	12	0.00	0.00
57	->	13	0.00	0.00
57	->	14	0.00	0.00
57	->	15	0.00	0.00
57	->	16	0.00	0.00
57	->	17	0.00	0.00
57	->	18	-0.00	0.00
57	->	19	-0.00	0.00
57	->	20	-0.00	0.00
57	->	21	0.00	0.00
57	->	22	0.00	0.00
57	->	23	0.00	0.00
57	->	24	0.00	0.00
57	->	25	0.00	0.00
57	->	26	0.00	0.00
57	->	27	0.00	0.00
57	->	28	0.00	0.00
57	->	29	0.00	0.00
57	->	30	0.00	0.00
57	->	31	0.00	0.00
57	->	32	0.00	0.00
57	->	33	0.00	0.00
57	->	34	-0.00	0.00
57	->	35	-0.00	0.00
57	->	36	-0.00	0.00
57	->	37	-0.01	0.00
57	->	38	-0.00	0.00
57	->	39	0.00	0.00
57	->	40	0.00	0.00
57	->	41	-0.00	0.00
57	->	42	-0.00	0.00
57	->	43	0.00	0.00
57	->	44	0.00	0.00
57	->	45	-0.00	0.00
57	->	46	-0.00	0.00
57	->	47	-0.00	0.00
57	->	48	-0.00	0.00
57	->	49	-0.01	0.00
57	->	50	-0.02	0.01
57	->	51	-0.03	0.02
57	->	52	-0.17	0.08
57	->	53	-1.24	0.43
57	->	54	-0.98	0.34
57	->	55	-0.52	0.12
57	->	56	-11.71	0.43
57	->	57	12.24	0.59
57	->	58	-14.87	0.50
57	->	59	-1.14	0.40
57	->	60	-0.03	0.01

57	->	61	-0.01	0.00
57	->	62	-0.00	0.00
57	->	63	0.00	0.00
57	->	64	0.00	0.00
57	->	65	0.00	0.00
57	->	66	0.00	0.00
57	->	67	-0.00	0.00
57	->	68	-0.00	0.00
57	->	69	-0.03	0.04
57	->	70	-0.00	0.01
57	->	71	-0.03	0.01
57	->	72	-0.01	0.00
57	->	73	-0.00	0.00
57	->	74	-0.00	0.00
57	->	75	-0.01	0.00
57	->	76	-0.00	0.00
57	->	77	0.00	0.00
57	->	78	0.00	0.00
57	->	79	-0.00	0.00
57	->	80	0.00	0.00
57	->	81	0.00	0.00
57	->	82	0.00	0.00
57	->	83	0.00	0.00
57	->	84	0.00	0.00
57	->	85	-0.00	0.00
57	->	86	-0.01	0.01
57	->	87	-0.04	0.04
57	->	88	-0.01	0.00
57	->	89	-0.00	0.00
57	->	90	-0.00	0.00
57	->	91	-0.00	0.00
57	->	92	-0.00	0.00
57	->	93	-0.00	0.00
57	->	94	-0.00	0.00
57	->	95	-0.00	0.00
57	->	96	-0.00	0.00
57	->	97	-0.00	0.00
57	->	98	-0.00	0.00
57	->	99	-0.00	0.00
57	->	100	-0.00	0.00
57	->	101	-0.00	0.00
57	->	102	-0.00	0.00
57	->	103	-0.00	0.00
57	->	104	-0.00	0.00
57	->	105	-0.00	0.00
57	->	106	-0.00	0.00
58	->	1	-0.00	0.00
58	->	2	-0.00	0.00
58	->	3	0.00	0.00
58	->	4	0.00	0.00

58	->	5	0.00	0.00
58	->	6	-0.00	0.00
58	->	7	0.00	0.00
58	->	8	0.00	0.00
58	->	9	0.00	0.00
58	->	10	0.00	0.00
58	->	11	0.00	0.00
58	->	12	-0.00	0.00
58	->	13	-0.00	0.00
58	->	14	-0.00	0.00
58	->	15	-0.00	0.00
58	->	16	-0.01	0.00
58	->	17	0.00	0.00
58	->	18	0.00	0.00
58	->	19	0.01	0.00
58	->	20	0.00	0.00
58	->	21	-0.00	0.00
58	->	22	-0.01	0.00
58	->	23	-0.00	0.00
58	->	24	-0.00	0.00
58	->	25	-0.01	0.00
58	->	26	-0.00	0.00
58	->	27	-0.00	0.00
58	->	28	0.00	0.00
58	->	29	-0.00	0.00
58	->	30	0.00	0.00
58	->	31	-0.01	0.00
58	->	32	0.00	0.00
58	->	33	-0.00	0.00
58	->	34	0.00	0.00
58	->	35	0.03	0.01
58	->	36	-0.06	0.05
58	->	37	-0.01	0.00
58	->	38	0.03	0.00
58	->	39	-0.00	0.00
58	->	40	-0.02	0.00
58	->	41	0.00	0.00
58	->	42	-0.00	0.00
58	->	43	0.00	0.00
58	->	44	-0.02	0.00
58	->	45	0.00	0.00
58	->	46	0.03	0.01
58	->	47	0.00	0.00
58	->	48	-0.00	0.00
58	->	49	-0.03	0.03
58	->	50	-0.02	0.02
58	->	51	-0.02	0.01
58	->	52	-0.26	0.12
58	->	53	-3.99	1.43
58	->	54	-0.21	0.15

58 ->	55	-0.05	0.03
58 ->	56	-2.43	0.91
58 ->	57	-15.02	0.50
58 ->	58	-63.01	2.75
58 ->	59	-11.76	0.56
58 ->	60	-1.17	0.36
58 ->	61	-0.09	0.02
58 ->	62	-0.01	0.00
58 ->	63	0.02	0.00
58 ->	64	0.02	0.00
58 ->	65	-0.00	0.00
58 ->	66	0.02	0.00
58 ->	67	0.00	0.00
58 ->	68	-0.03	0.01
58 ->	69	-0.07	0.07
58 ->	70	-0.24	0.09
58 ->	71	-0.60	0.28
58 ->	72	-0.04	0.01
58 ->	73	-0.01	0.00
58 ->	74	0.03	0.01
58 ->	75	0.06	0.02
58 ->	76	-0.03	0.01
58 ->	77	-0.02	0.01
58 ->	78	-0.02	0.00
58 ->	79	-0.00	0.00
58 ->	80	-0.00	0.00
58 ->	81	-0.01	0.00
58 ->	82	-0.00	0.00
58 ->	83	0.00	0.00
58 ->	84	0.01	0.00
58 ->	85	0.00	0.00
58 ->	86	0.03	0.01
58 ->	87	0.04	0.02
58 ->	88	0.03	0.01
58 ->	89	0.01	0.00
58 ->	90	0.01	0.00
58 ->	91	0.01	0.00
58 ->	92	0.00	0.00
58 ->	93	0.01	0.00
58 ->	94	0.00	0.00
58 ->	95	0.00	0.00
58 ->	96	0.00	0.00
58 ->	97	0.01	0.01
58 ->	98	0.03	0.01
58 ->	99	0.04	0.02
58 ->	100	0.05	0.02
58 ->	101	0.02	0.00
58 ->	102	0.01	0.00
58 ->	103	0.01	0.00
58 ->	104	0.01	0.00

58	->	105	0.00	0.00
58	->	106	0.00	0.00
59	->	1	0.00	0.00
59	->	2	0.00	0.00
59	->	3	0.00	0.00
59	->	4	0.00	0.00
59	->	5	-0.00	0.00
59	->	6	0.00	0.00
59	->	7	0.00	0.00
59	->	8	-0.00	0.00
59	->	9	-0.00	0.00
59	->	10	-0.00	0.00
59	->	11	-0.00	0.00
59	->	12	0.00	0.00
59	->	13	0.00	0.00
59	->	14	0.00	0.00
59	->	15	0.00	0.00
59	->	16	0.01	0.00
59	->	17	-0.00	0.00
59	->	18	-0.00	0.00
59	->	19	-0.01	0.00
59	->	20	-0.00	0.00
59	->	21	-0.00	0.00
59	->	22	0.01	0.00
59	->	23	0.00	0.00
59	->	24	0.00	0.00
59	->	25	0.01	0.00
59	->	26	0.00	0.00
59	->	27	0.00	0.00
59	->	28	0.00	0.00
59	->	29	0.00	0.00
59	->	30	-0.00	0.00
59	->	31	0.01	0.00
59	->	32	0.00	0.00
59	->	33	0.00	0.00
59	->	34	-0.00	0.00
59	->	35	-0.03	0.00
59	->	36	0.02	0.01
59	->	37	-0.00	0.00
59	->	38	-0.03	0.01
59	->	39	0.00	0.00
59	->	40	0.02	0.01
59	->	41	-0.00	0.00
59	->	42	-0.00	0.00
59	->	43	0.00	0.00
59	->	44	0.02	0.00
59	->	45	-0.00	0.00
59	->	46	-0.03	0.00
59	->	47	-0.01	0.00
59	->	48	-0.00	0.00

59 ->	49	-0.00	0.01
59 ->	50	-0.05	0.04
59 ->	51	-0.02	0.01
59 ->	52	-0.01	0.01
59 ->	53	-0.06	0.38
59 ->	54	-0.47	0.72
59 ->	55	-0.06	0.04
59 ->	56	-0.04	0.03
59 ->	57	-1.21	0.42
59 ->	58	-11.81	0.57
59 ->	59	11.97	3.31
59 ->	60	-0.36	0.43
59 ->	61	-0.95	0.27
59 ->	62	-0.16	0.04
59 ->	63	-0.08	0.01
59 ->	64	-0.03	0.01
59 ->	65	-0.00	0.00
59 ->	66	-0.05	0.01
59 ->	67	-0.01	0.01
59 ->	68	0.12	0.10
59 ->	69	-2.10	0.77
59 ->	70	-3.28	0.53
59 ->	71	-1.82	0.32
59 ->	72	-0.05	0.04
59 ->	73	-0.06	0.02
59 ->	74	-0.07	0.01
59 ->	75	-0.75	0.46
59 ->	76	0.03	0.01
59 ->	77	0.01	0.01
59 ->	78	0.04	0.01
59 ->	79	-0.00	0.00
59 ->	80	-0.00	0.00
59 ->	81	0.02	0.00
59 ->	82	0.00	0.00
59 ->	83	-0.00	0.00
59 ->	84	-0.01	0.00
59 ->	85	-0.08	0.09
59 ->	86	-0.87	0.86
59 ->	87	-1.79	2.38
59 ->	88	-0.06	0.02
59 ->	89	-0.02	0.00
59 ->	90	-0.01	0.00
59 ->	91	-0.01	0.00
59 ->	92	-0.01	0.00
59 ->	93	-0.01	0.00
59 ->	94	-0.00	0.00
59 ->	95	-0.00	0.00
59 ->	96	-0.00	0.00
59 ->	97	-0.01	0.00
59 ->	98	-0.01	0.00

59	->	99	-0.05	0.02
59	->	100	-0.09	0.05
59	->	101	-0.03	0.01
59	->	102	-0.02	0.00
59	->	103	-0.01	0.00
59	->	104	-0.01	0.00
59	->	105	-0.01	0.00
59	->	106	-0.01	0.00
60	->	1	0.00	0.00
60	->	2	0.00	0.00
60	->	3	0.00	0.00
60	->	4	0.00	0.00
60	->	5	-0.00	0.00
60	->	6	0.00	0.00
60	->	7	0.00	0.00
60	->	8	-0.00	0.00
60	->	9	0.00	0.00
60	->	10	-0.00	0.00
60	->	11	-0.00	0.00
60	->	12	0.00	0.00
60	->	13	0.00	0.00
60	->	14	0.00	0.00
60	->	15	0.00	0.00
60	->	16	0.00	0.00
60	->	17	0.00	0.00
60	->	18	0.00	0.00
60	->	19	-0.00	0.00
60	->	20	0.00	0.00
60	->	21	0.00	0.00
60	->	22	0.00	0.00
60	->	23	0.00	0.00
60	->	24	0.00	0.00
60	->	25	0.00	0.00
60	->	26	0.00	0.00
60	->	27	0.00	0.00
60	->	28	0.00	0.00
60	->	29	0.00	0.00
60	->	30	-0.00	0.00
60	->	31	0.00	0.00
60	->	32	0.00	0.00
60	->	33	-0.00	0.00
60	->	34	-0.00	0.00
60	->	35	-0.00	0.00
60	->	36	0.00	0.00
60	->	37	-0.00	0.00
60	->	38	-0.00	0.00
60	->	39	0.00	0.00
60	->	40	0.00	0.00
60	->	41	-0.00	0.00
60	->	42	-0.00	0.00

60	->	43	0.00	0.00
60	->	44	0.00	0.00
60	->	45	-0.00	0.00
60	->	46	-0.01	0.00
60	->	47	-0.00	0.00
60	->	48	-0.00	0.00
60	->	49	-0.02	0.01
60	->	50	-0.03	0.02
60	->	51	-0.01	0.00
60	->	52	-0.01	0.00
60	->	53	-1.16	0.70
60	->	54	-0.02	0.01
60	->	55	-0.00	0.00
60	->	56	-0.01	0.00
60	->	57	-0.03	0.01
60	->	58	-1.20	0.37
60	->	59	-0.40	0.44
60	->	60	1.44	0.57
60	->	61	-10.46	0.56
60	->	62	-0.38	0.11
60	->	63	-0.02	0.00
60	->	64	-0.00	0.00
60	->	65	-0.00	0.00
60	->	66	0.00	0.00
60	->	67	-0.00	0.00
60	->	68	-0.02	0.01
60	->	69	-0.48	0.52
60	->	70	-0.84	0.15
60	->	71	-0.73	0.28
60	->	72	-0.14	0.07
60	->	73	-0.06	0.02
60	->	74	-0.01	0.00
60	->	75	-0.02	0.01
60	->	76	-0.04	0.01
60	->	77	-0.01	0.00
60	->	78	-0.00	0.00
60	->	79	-0.00	0.00
60	->	80	-0.00	0.00
60	->	81	-0.00	0.00
60	->	82	-0.00	0.00
60	->	83	-0.00	0.00
60	->	84	0.00	0.00
60	->	85	-0.00	0.00
60	->	86	-0.00	0.00
60	->	87	-0.01	0.00
60	->	88	-0.00	0.00
60	->	89	-0.00	0.00
60	->	90	-0.00	0.00
60	->	91	-0.00	0.00
60	->	92	-0.00	0.00

60	->	93	-0.00	0.00
60	->	94	-0.00	0.00
60	->	95	-0.00	0.00
60	->	96	-0.00	0.00
60	->	97	-0.00	0.00
60	->	98	-0.00	0.00
60	->	99	-0.04	0.03
60	->	100	-0.24	0.23
60	->	101	-0.02	0.01
60	->	102	-0.00	0.00
60	->	103	-0.00	0.00
60	->	104	-0.00	0.00
60	->	105	-0.00	0.00
60	->	106	-0.00	0.00
61	->	1	0.00	0.00
61	->	2	0.00	0.00
61	->	3	0.00	0.00
61	->	4	0.00	0.00
61	->	5	-0.00	0.00
61	->	6	0.00	0.00
61	->	7	0.00	0.00
61	->	8	0.00	0.00
61	->	9	0.00	0.00
61	->	10	-0.00	0.00
61	->	11	-0.00	0.00
61	->	12	0.00	0.00
61	->	13	0.00	0.00
61	->	14	0.00	0.00
61	->	15	0.00	0.00
61	->	16	0.00	0.00
61	->	17	0.00	0.00
61	->	18	0.00	0.00
61	->	19	-0.00	0.00
61	->	20	0.00	0.00
61	->	21	0.00	0.00
61	->	22	-0.00	0.00
61	->	23	0.00	0.00
61	->	24	0.00	0.00
61	->	25	0.00	0.00
61	->	26	0.00	0.00
61	->	27	0.00	0.00
61	->	28	0.00	0.00
61	->	29	0.00	0.00
61	->	30	0.00	0.00
61	->	31	-0.00	0.00
61	->	32	0.00	0.00
61	->	33	-0.00	0.00
61	->	34	0.00	0.00
61	->	35	0.00	0.00
61	->	36	-0.00	0.00

61 ->	37	-0.00	0.00
61 ->	38	0.00	0.00
61 ->	39	-0.00	0.00
61 ->	40	-0.00	0.00
61 ->	41	0.00	0.00
61 ->	42	-0.00	0.00
61 ->	43	0.00	0.00
61 ->	44	-0.00	0.00
61 ->	45	-0.00	0.00
61 ->	46	-0.01	0.00
61 ->	47	-0.00	0.00
61 ->	48	-0.00	0.00
61 ->	49	-0.02	0.01
61 ->	50	-0.03	0.02
61 ->	51	-0.00	0.00
61 ->	52	-0.01	0.00
61 ->	53	-0.50	0.32
61 ->	54	-0.00	0.00
61 ->	55	-0.00	0.00
61 ->	56	-0.00	0.00
61 ->	57	-0.01	0.00
61 ->	58	-0.09	0.02
61 ->	59	-0.96	0.27
61 ->	60	-10.60	0.55
61 ->	61	28.20	1.90
61 ->	62	-10.46	0.42
61 ->	63	-0.37	0.05
61 ->	64	-0.06	0.02
61 ->	65	-0.01	0.00
61 ->	66	-0.01	0.00
61 ->	67	-0.01	0.01
61 ->	68	-0.29	0.07
61 ->	69	-2.99	0.63
61 ->	70	-3.40	0.63
61 ->	71	-0.96	0.32
61 ->	72	-2.16	0.77
61 ->	73	-1.96	0.66
61 ->	74	0.01	0.02
61 ->	75	-0.15	0.06
61 ->	76	-1.33	0.69
61 ->	77	-0.04	0.02
61 ->	78	-0.01	0.00
61 ->	79	-0.02	0.01
61 ->	80	-0.01	0.01
61 ->	81	-0.00	0.00
61 ->	82	-0.00	0.00
61 ->	83	-0.01	0.00
61 ->	84	-0.00	0.00
61 ->	85	-0.00	0.00
61 ->	86	-0.01	0.00

61	->	87	-0.01	0.00
61	->	88	-0.00	0.00
61	->	89	-0.00	0.00
61	->	90	-0.00	0.00
61	->	91	-0.00	0.00
61	->	92	-0.01	0.00
61	->	93	-0.00	0.00
61	->	94	-0.00	0.00
61	->	95	-0.00	0.00
61	->	96	-0.00	0.00
61	->	97	-0.00	0.00
61	->	98	-0.00	0.00
61	->	99	-0.02	0.03
61	->	100	-1.28	0.83
61	->	101	-0.31	0.26
61	->	102	-0.02	0.01
61	->	103	-0.01	0.00
61	->	104	-0.00	0.00
61	->	105	-0.00	0.00
61	->	106	-0.00	0.00
62	->	1	-0.00	0.00
62	->	2	0.00	0.00
62	->	3	0.00	0.00
62	->	4	0.00	0.00
62	->	5	0.00	0.00
62	->	6	0.00	0.00
62	->	7	0.00	0.00
62	->	8	0.00	0.00
62	->	9	0.00	0.00
62	->	10	0.00	0.00
62	->	11	0.00	0.00
62	->	12	-0.00	0.00
62	->	13	-0.00	0.00
62	->	14	-0.00	0.00
62	->	15	-0.00	0.00
62	->	16	-0.00	0.00
62	->	17	0.00	0.00
62	->	18	0.00	0.00
62	->	19	0.00	0.00
62	->	20	-0.00	0.00
62	->	21	0.00	0.00
62	->	22	-0.00	0.00
62	->	23	-0.00	0.00
62	->	24	-0.00	0.00
62	->	25	-0.00	0.00
62	->	26	0.00	0.00
62	->	27	-0.00	0.00
62	->	28	0.00	0.00
62	->	29	0.00	0.00
62	->	30	0.00	0.00

62	->	31	-0.00	0.00
62	->	32	-0.00	0.00
62	->	33	-0.00	0.00
62	->	34	0.00	0.00
62	->	35	0.00	0.00
62	->	36	-0.00	0.00
62	->	37	-0.00	0.00
62	->	38	-0.00	0.00
62	->	39	0.00	0.00
62	->	40	-0.00	0.00
62	->	41	0.00	0.00
62	->	42	-0.00	0.00
62	->	43	0.00	0.00
62	->	44	-0.00	0.00
62	->	45	0.00	0.00
62	->	46	0.00	0.00
62	->	47	0.00	0.00
62	->	48	-0.00	0.00
62	->	49	-0.00	0.00
62	->	50	-0.00	0.00
62	->	51	-0.00	0.00
62	->	52	-0.00	0.00
62	->	53	-0.01	0.01
62	->	54	-0.00	0.00
62	->	55	-0.00	0.00
62	->	56	-0.00	0.00
62	->	57	-0.00	0.00
62	->	58	-0.01	0.00
62	->	59	-0.16	0.04
62	->	60	-0.40	0.12
62	->	61	-10.44	0.42
62	->	62	23.05	1.15
62	->	63	-11.46	0.66
62	->	64	-0.77	0.39
62	->	65	-0.02	0.03
62	->	66	-0.09	0.06
62	->	67	-0.03	0.14
62	->	68	-1.49	0.49
62	->	69	-1.37	0.34
62	->	70	-1.29	0.44
62	->	71	-0.02	0.01
62	->	72	-0.03	0.01
62	->	73	-0.01	0.01
62	->	74	0.00	0.00
62	->	75	-0.02	0.01
62	->	76	-2.42	1.52
62	->	77	-0.01	0.01
62	->	78	0.01	0.01
62	->	79	-0.11	0.05
62	->	80	-0.03	0.02

62	->	81	-0.01	0.00
62	->	82	-0.00	0.00
62	->	83	-0.04	0.01
62	->	84	-0.00	0.00
62	->	85	-0.00	0.00
62	->	86	-0.01	0.00
62	->	87	-0.00	0.00
62	->	88	-0.00	0.00
62	->	89	0.00	0.00
62	->	90	0.00	0.00
62	->	91	0.00	0.00
62	->	92	0.00	0.00
62	->	93	0.00	0.00
62	->	94	0.00	0.00
62	->	95	0.00	0.00
62	->	96	0.00	0.00
62	->	97	0.00	0.00
62	->	98	0.00	0.00
62	->	99	0.00	0.00
62	->	100	0.00	0.00
62	->	101	0.00	0.00
62	->	102	0.00	0.00
62	->	103	0.00	0.00
62	->	104	0.00	0.00
62	->	105	0.00	0.00
62	->	106	0.00	0.00
63	->	1	-0.00	0.00
63	->	2	-0.00	0.00
63	->	3	0.00	0.00
63	->	4	0.00	0.00
63	->	5	0.00	0.00
63	->	6	-0.00	0.00
63	->	7	0.00	0.00
63	->	8	0.00	0.00
63	->	9	-0.00	0.00
63	->	10	0.00	0.00
63	->	11	0.00	0.00
63	->	12	-0.00	0.00
63	->	13	-0.00	0.00
63	->	14	-0.00	0.00
63	->	15	-0.00	0.00
63	->	16	-0.00	0.00
63	->	17	0.00	0.00
63	->	18	-0.00	0.00
63	->	19	0.00	0.00
63	->	20	0.00	0.00
63	->	21	-0.00	0.00
63	->	22	-0.01	0.00
63	->	23	-0.00	0.00
63	->	24	-0.00	0.00

63	->	25	-0.00	0.00
63	->	26	-0.00	0.00
63	->	27	-0.00	0.00
63	->	28	-0.00	0.00
63	->	29	-0.00	0.00
63	->	30	0.00	0.00
63	->	31	-0.00	0.00
63	->	32	-0.00	0.00
63	->	33	-0.00	0.00
63	->	34	0.00	0.00
63	->	35	0.01	0.00
63	->	36	-0.00	0.00
63	->	37	0.00	0.00
63	->	38	0.01	0.00
63	->	39	-0.00	0.00
63	->	40	-0.01	0.00
63	->	41	0.00	0.00
63	->	42	-0.00	0.00
63	->	43	-0.00	0.00
63	->	44	-0.01	0.00
63	->	45	0.00	0.00
63	->	46	0.01	0.00
63	->	47	0.00	0.00
63	->	48	0.00	0.00
63	->	49	0.00	0.00
63	->	50	-0.00	0.00
63	->	51	-0.00	0.00
63	->	52	0.00	0.00
63	->	53	-0.03	0.01
63	->	54	0.00	0.00
63	->	55	-0.00	0.00
63	->	56	0.00	0.00
63	->	57	0.00	0.00
63	->	58	0.02	0.00
63	->	59	-0.08	0.01
63	->	60	-0.02	0.00
63	->	61	-0.37	0.05
63	->	62	-11.51	0.64
63	->	63	-31.09	2.61
63	->	64	-14.18	1.29
63	->	65	-2.35	1.80
63	->	66	0.87	1.23
63	->	67	-1.55	0.92
63	->	68	-2.39	0.84
63	->	69	-0.55	0.16
63	->	70	-0.85	0.31
63	->	71	0.00	0.01
63	->	72	-0.04	0.01
63	->	73	0.00	0.01
63	->	74	0.03	0.00

63	->	75	-0.01	0.02
63	->	76	-1.50	0.70
63	->	77	-0.03	0.02
63	->	78	-0.13	0.03
63	->	79	2.06	0.67
63	->	80	0.10	0.18
63	->	81	-0.02	0.01
63	->	82	-0.05	0.02
63	->	83	1.89	0.87
63	->	84	0.06	0.01
63	->	85	0.01	0.01
63	->	86	0.05	0.01
63	->	87	0.02	0.00
63	->	88	0.01	0.00
63	->	89	0.01	0.00
63	->	90	0.01	0.00
63	->	91	0.00	0.00
63	->	92	0.00	0.00
63	->	93	0.01	0.00
63	->	94	0.01	0.00
63	->	95	0.00	0.00
63	->	96	0.00	0.00
63	->	97	0.00	0.00
63	->	98	0.01	0.00
63	->	99	0.01	0.00
63	->	100	0.02	0.01
63	->	101	0.03	0.01
63	->	102	0.03	0.01
63	->	103	0.02	0.00
63	->	104	0.01	0.00
63	->	105	0.01	0.00
63	->	106	0.00	0.00
64	->	1	-0.00	0.00
64	->	2	0.00	0.00
64	->	3	0.00	0.00
64	->	4	0.00	0.00
64	->	5	0.00	0.00
64	->	6	0.00	0.00
64	->	7	-0.00	0.00
64	->	8	0.00	0.00
64	->	9	0.00	0.00
64	->	10	0.00	0.00
64	->	11	0.00	0.00
64	->	12	-0.00	0.00
64	->	13	-0.00	0.00
64	->	14	-0.00	0.00
64	->	15	-0.00	0.00
64	->	16	-0.00	0.00
64	->	17	0.00	0.00
64	->	18	-0.00	0.00

64 ->	19	0.00	0.00
64 ->	20	0.00	0.00
64 ->	21	-0.00	0.00
64 ->	22	-0.00	0.00
64 ->	23	-0.00	0.00
64 ->	24	-0.00	0.00
64 ->	25	-0.00	0.00
64 ->	26	-0.00	0.00
64 ->	27	-0.00	0.00
64 ->	28	-0.00	0.00
64 ->	29	0.00	0.00
64 ->	30	0.00	0.00
64 ->	31	-0.00	0.00
64 ->	32	-0.00	0.00
64 ->	33	-0.00	0.00
64 ->	34	0.00	0.00
64 ->	35	0.00	0.00
64 ->	36	-0.00	0.00
64 ->	37	0.00	0.00
64 ->	38	0.00	0.00
64 ->	39	-0.00	0.00
64 ->	40	-0.00	0.00
64 ->	41	0.00	0.00
64 ->	42	-0.00	0.00
64 ->	43	-0.00	0.00
64 ->	44	-0.01	0.00
64 ->	45	0.00	0.00
64 ->	46	0.01	0.00
64 ->	47	0.00	0.00
64 ->	48	0.00	0.00
64 ->	49	0.00	0.00
64 ->	50	0.00	0.00
64 ->	51	-0.00	0.00
64 ->	52	0.00	0.00
64 ->	53	-0.02	0.00
64 ->	54	0.00	0.00
64 ->	55	-0.00	0.00
64 ->	56	0.00	0.00
64 ->	57	0.00	0.00
64 ->	58	0.02	0.00
64 ->	59	-0.03	0.01
64 ->	60	-0.00	0.00
64 ->	61	-0.06	0.02
64 ->	62	-0.78	0.40
64 ->	63	-14.28	1.35
64 ->	64	-35.17	2.26
64 ->	65	-12.41	0.53
64 ->	66	-0.54	0.22
64 ->	67	-0.80	0.58
64 ->	68	-0.08	0.09

64	->	69	-0.16	0.13
64	->	70	-0.01	0.01
64	->	71	0.00	0.00
64	->	72	-0.02	0.00
64	->	73	-0.00	0.00
64	->	74	0.02	0.00
64	->	75	0.02	0.00
64	->	76	-0.94	1.61
64	->	77	-0.03	0.01
64	->	78	-0.02	0.01
64	->	79	-0.01	0.07
64	->	80	-0.03	0.06
64	->	81	-0.02	0.01
64	->	82	-0.01	0.00
64	->	83	-0.04	0.09
64	->	84	0.03	0.01
64	->	85	0.01	0.00
64	->	86	0.02	0.00
64	->	87	0.01	0.00
64	->	88	0.01	0.00
64	->	89	0.00	0.00
64	->	90	0.00	0.00
64	->	91	0.00	0.00
64	->	92	0.00	0.00
64	->	93	0.01	0.00
64	->	94	0.00	0.00
64	->	95	0.00	0.00
64	->	96	0.00	0.00
64	->	97	0.00	0.00
64	->	98	0.01	0.00
64	->	99	0.01	0.00
64	->	100	0.02	0.00
64	->	101	0.02	0.01
64	->	102	0.02	0.01
64	->	103	0.01	0.00
64	->	104	0.01	0.00
64	->	105	0.00	0.00
64	->	106	0.00	0.00
65	->	1	0.00	0.00
65	->	2	0.00	0.00
65	->	3	0.00	0.00
65	->	4	0.00	0.00
65	->	5	-0.00	0.00
65	->	6	0.00	0.00
65	->	7	0.00	0.00
65	->	8	-0.00	0.00
65	->	9	0.00	0.00
65	->	10	-0.00	0.00
65	->	11	-0.00	0.00
65	->	12	0.00	0.00

65 ->	13	0.00	0.00
65 ->	14	0.00	0.00
65 ->	15	0.00	0.00
65 ->	16	0.00	0.00
65 ->	17	0.00	0.00
65 ->	18	0.00	0.00
65 ->	19	-0.00	0.00
65 ->	20	0.00	0.00
65 ->	21	0.00	0.00
65 ->	22	0.00	0.00
65 ->	23	0.00	0.00
65 ->	24	0.00	0.00
65 ->	25	0.00	0.00
65 ->	26	0.00	0.00
65 ->	27	0.00	0.00
65 ->	28	0.00	0.00
65 ->	29	0.00	0.00
65 ->	30	-0.00	0.00
65 ->	31	0.00	0.00
65 ->	32	0.00	0.00
65 ->	33	0.00	0.00
65 ->	34	0.00	0.00
65 ->	35	-0.00	0.00
65 ->	36	0.00	0.00
65 ->	37	0.00	0.00
65 ->	38	-0.00	0.00
65 ->	39	0.00	0.00
65 ->	40	0.00	0.00
65 ->	41	0.00	0.00
65 ->	42	0.00	0.00
65 ->	43	0.00	0.00
65 ->	44	0.00	0.00
65 ->	45	-0.00	0.00
65 ->	46	-0.00	0.00
65 ->	47	0.00	0.00
65 ->	48	-0.00	0.00
65 ->	49	0.00	0.00
65 ->	50	0.00	0.00
65 ->	51	0.00	0.00
65 ->	52	0.00	0.00
65 ->	53	0.00	0.00
65 ->	54	0.00	0.00
65 ->	55	0.00	0.00
65 ->	56	0.00	0.00
65 ->	57	0.00	0.00
65 ->	58	-0.00	0.00
65 ->	59	-0.00	0.00
65 ->	60	-0.00	0.00
65 ->	61	-0.01	0.00
65 ->	62	-0.02	0.03

65	->	63	-2.41	1.81
65	->	64	-12.57	0.53
65	->	65	16.92	1.81
65	->	66	-14.10	0.58
65	->	67	-0.45	0.21
65	->	68	-0.04	0.01
65	->	69	-0.02	0.01
65	->	70	-0.02	0.01
65	->	71	-0.00	0.00
65	->	72	-0.00	0.00
65	->	73	-0.00	0.00
65	->	74	-0.00	0.00
65	->	75	-0.01	0.00
65	->	76	-0.03	0.03
65	->	77	-0.00	0.00
65	->	78	0.00	0.00
65	->	79	-0.21	0.14
65	->	80	-0.14	0.33
65	->	81	-0.01	0.01
65	->	82	-0.01	0.01
65	->	83	-1.30	1.66
65	->	84	-0.18	0.43
65	->	85	-0.00	0.00
65	->	86	-0.00	0.00
65	->	87	-0.00	0.00
65	->	88	-0.00	0.00
65	->	89	-0.00	0.00
65	->	90	-0.00	0.00
65	->	91	-0.00	0.00
65	->	92	-0.00	0.00
65	->	93	-0.00	0.00
65	->	94	-0.00	0.00
65	->	95	-0.00	0.00
65	->	96	-0.00	0.00
65	->	97	-0.00	0.00
65	->	98	-0.00	0.00
65	->	99	-0.00	0.00
65	->	100	-0.00	0.00
65	->	101	-0.00	0.00
65	->	102	-0.00	0.00
65	->	103	-0.00	0.00
65	->	104	-0.00	0.00
65	->	105	-0.00	0.00
65	->	106	-0.00	0.00
66	->	1	-0.00	0.00
66	->	2	-0.00	0.00
66	->	3	0.00	0.00
66	->	4	0.00	0.00
66	->	5	0.00	0.00
66	->	6	-0.00	0.00

66	->	7	0.00	0.00
66	->	8	0.00	0.00
66	->	9	0.00	0.00
66	->	10	0.00	0.00
66	->	11	0.00	0.00
66	->	12	-0.00	0.00
66	->	13	-0.00	0.00
66	->	14	-0.00	0.00
66	->	15	-0.00	0.00
66	->	16	-0.00	0.00
66	->	17	0.00	0.00
66	->	18	0.00	0.00
66	->	19	0.00	0.00
66	->	20	0.00	0.00
66	->	21	-0.00	0.00
66	->	22	-0.00	0.00
66	->	23	-0.00	0.00
66	->	24	-0.00	0.00
66	->	25	-0.00	0.00
66	->	26	-0.00	0.00
66	->	27	-0.00	0.00
66	->	28	-0.00	0.00
66	->	29	-0.00	0.00
66	->	30	0.00	0.00
66	->	31	-0.00	0.00
66	->	32	-0.00	0.00
66	->	33	-0.00	0.00
66	->	34	0.00	0.00
66	->	35	0.01	0.00
66	->	36	-0.00	0.00
66	->	37	0.00	0.00
66	->	38	0.01	0.00
66	->	39	-0.00	0.00
66	->	40	-0.01	0.00
66	->	41	0.00	0.00
66	->	42	-0.00	0.00
66	->	43	-0.00	0.00
66	->	44	-0.01	0.00
66	->	45	0.00	0.00
66	->	46	0.01	0.00
66	->	47	0.00	0.00
66	->	48	0.00	0.00
66	->	49	0.00	0.00
66	->	50	0.00	0.00
66	->	51	-0.00	0.00
66	->	52	0.00	0.00
66	->	53	-0.02	0.00
66	->	54	0.00	0.00
66	->	55	-0.00	0.00
66	->	56	0.00	0.00

66	->	57	0.00	0.00
66	->	58	0.02	0.00
66	->	59	-0.05	0.01
66	->	60	0.00	0.00
66	->	61	-0.01	0.00
66	->	62	-0.09	0.06
66	->	63	0.85	1.23
66	->	64	-0.55	0.22
66	->	65	-14.18	0.58
66	->	66	-39.75	2.76
66	->	67	-8.47	0.64
66	->	68	-2.44	1.47
66	->	69	-0.05	0.03
66	->	70	-0.01	0.03
66	->	71	-0.00	0.00
66	->	72	-0.02	0.00
66	->	73	0.00	0.00
66	->	74	0.03	0.00
66	->	75	0.04	0.01
66	->	76	-0.08	0.02
66	->	77	-0.03	0.00
66	->	78	-0.06	0.01
66	->	79	2.33	0.91
66	->	80	0.02	0.01
66	->	81	-0.03	0.00
66	->	82	-0.04	0.02
66	->	83	1.59	0.70
66	->	84	0.05	0.01
66	->	85	0.02	0.01
66	->	86	0.06	0.02
66	->	87	0.02	0.00
66	->	88	0.01	0.00
66	->	89	0.01	0.00
66	->	90	0.00	0.00
66	->	91	0.00	0.00
66	->	92	0.00	0.00
66	->	93	0.00	0.00
66	->	94	0.00	0.00
66	->	95	0.00	0.00
66	->	96	0.00	0.00
66	->	97	0.00	0.00
66	->	98	0.00	0.00
66	->	99	0.01	0.00
66	->	100	0.01	0.00
66	->	101	0.02	0.00
66	->	102	0.02	0.00
66	->	103	0.01	0.00
66	->	104	0.01	0.00
66	->	105	0.01	0.00
66	->	106	0.00	0.00

67	->	1	0.00	0.00
67	->	2	0.00	0.00
67	->	3	0.00	0.00
67	->	4	0.00	0.00
67	->	5	0.00	0.00
67	->	6	0.00	0.00
67	->	7	0.00	0.00
67	->	8	0.00	0.00
67	->	9	0.00	0.00
67	->	10	0.00	0.00
67	->	11	-0.00	0.00
67	->	12	-0.00	0.00
67	->	13	0.00	0.00
67	->	14	0.00	0.00
67	->	15	0.00	0.00
67	->	16	0.00	0.00
67	->	17	0.00	0.00
67	->	18	0.00	0.00
67	->	19	-0.00	0.00
67	->	20	0.00	0.00
67	->	21	0.00	0.00
67	->	22	-0.00	0.00
67	->	23	0.00	0.00
67	->	24	0.00	0.00
67	->	25	-0.00	0.00
67	->	26	0.00	0.00
67	->	27	0.00	0.00
67	->	28	0.00	0.00
67	->	29	0.00	0.00
67	->	30	0.00	0.00
67	->	31	0.00	0.00
67	->	32	0.00	0.00
67	->	33	0.00	0.00
67	->	34	0.00	0.00
67	->	35	0.00	0.00
67	->	36	-0.00	0.00
67	->	37	0.00	0.00
67	->	38	0.00	0.00
67	->	39	0.00	0.00
67	->	40	-0.00	0.00
67	->	41	0.00	0.00
67	->	42	0.00	0.00
67	->	43	0.00	0.00
67	->	44	0.00	0.00
67	->	45	0.00	0.00
67	->	46	0.00	0.00
67	->	47	0.00	0.00
67	->	48	0.00	0.00
67	->	49	0.00	0.00
67	->	50	0.00	0.00

67	->	51	0.00	0.00
67	->	52	0.00	0.00
67	->	53	-0.00	0.00
67	->	54	-0.00	0.00
67	->	55	-0.00	0.00
67	->	56	0.00	0.00
67	->	57	-0.00	0.00
67	->	58	0.00	0.00
67	->	59	-0.01	0.01
67	->	60	-0.00	0.00
67	->	61	-0.01	0.01
67	->	62	-0.03	0.14
67	->	63	-1.56	0.92
67	->	64	-0.81	0.60
67	->	65	-0.46	0.21
67	->	66	-8.57	0.63
67	->	67	12.64	1.45
67	->	68	-8.61	0.67
67	->	69	-0.84	0.57
67	->	70	-0.11	0.04
67	->	71	-0.00	0.00
67	->	72	-0.00	0.00
67	->	73	-0.00	0.00
67	->	74	-0.00	0.00
67	->	75	0.00	0.00
67	->	76	-0.01	0.01
67	->	77	-0.00	0.00
67	->	78	-0.00	0.00
67	->	79	-0.07	0.07
67	->	80	-0.00	0.00
67	->	81	-0.00	0.00
67	->	82	-0.00	0.00
67	->	83	-0.02	0.04
67	->	84	-0.00	0.00
67	->	85	-0.00	0.01
67	->	86	-0.00	0.00
67	->	87	-0.00	0.00
67	->	88	0.00	0.00
67	->	89	0.00	0.00
67	->	90	0.00	0.00
67	->	91	0.00	0.00
67	->	92	-0.00	0.00
67	->	93	0.00	0.00
67	->	94	-0.00	0.00
67	->	95	-0.00	0.00
67	->	96	0.00	0.00
67	->	97	0.00	0.00
67	->	98	0.00	0.00
67	->	99	0.00	0.00
67	->	100	-0.00	0.00

67	->	101	-0.00	0.00
67	->	102	-0.00	0.00
67	->	103	-0.00	0.00
67	->	104	0.00	0.00
67	->	105	0.00	0.00
67	->	106	0.00	0.00
68	->	1	0.00	0.00
68	->	2	0.00	0.00
68	->	3	-0.00	0.00
68	->	4	0.00	0.00
68	->	5	-0.00	0.00
68	->	6	0.00	0.00
68	->	7	0.00	0.00
68	->	8	-0.00	0.00
68	->	9	-0.00	0.00
68	->	10	-0.00	0.00
68	->	11	-0.00	0.00
68	->	12	0.00	0.00
68	->	13	0.00	0.00
68	->	14	0.00	0.00
68	->	15	0.00	0.00
68	->	16	0.00	0.00
68	->	17	-0.00	0.00
68	->	18	-0.00	0.00
68	->	19	-0.00	0.00
68	->	20	-0.00	0.00
68	->	21	0.00	0.00
68	->	22	0.01	0.00
68	->	23	0.00	0.00
68	->	24	0.00	0.00
68	->	25	0.00	0.00
68	->	26	0.00	0.00
68	->	27	0.00	0.00
68	->	28	0.00	0.00
68	->	29	0.00	0.00
68	->	30	-0.00	0.00
68	->	31	0.01	0.00
68	->	32	0.00	0.00
68	->	33	0.00	0.00
68	->	34	0.00	0.00
68	->	35	-0.01	0.00
68	->	36	0.01	0.00
68	->	37	-0.00	0.00
68	->	38	-0.01	0.00
68	->	39	0.00	0.00
68	->	40	0.01	0.00
68	->	41	-0.00	0.00
68	->	42	0.00	0.00
68	->	43	0.00	0.00
68	->	44	0.01	0.00

68	->	45	-0.00	0.00
68	->	46	-0.01	0.00
68	->	47	-0.00	0.00
68	->	48	-0.00	0.00
68	->	49	-0.00	0.00
68	->	50	-0.00	0.00
68	->	51	-0.00	0.00
68	->	52	-0.00	0.00
68	->	53	0.03	0.01
68	->	54	-0.01	0.01
68	->	55	-0.00	0.00
68	->	56	-0.00	0.00
68	->	57	-0.00	0.00
68	->	58	-0.03	0.01
68	->	59	0.12	0.10
68	->	60	-0.02	0.01
68	->	61	-0.29	0.07
68	->	62	-1.48	0.49
68	->	63	-2.39	0.85
68	->	64	-0.08	0.09
68	->	65	-0.04	0.01
68	->	66	-2.51	1.50
68	->	67	-8.73	0.67
68	->	68	-106.99	3.30
68	->	69	-13.18	0.98
68	->	70	-2.92	1.06
68	->	71	-0.06	0.05
68	->	72	0.03	0.01
68	->	73	-0.01	0.01
68	->	74	-0.04	0.01
68	->	75	-0.63	1.73
68	->	76	-0.06	0.04
68	->	77	0.01	0.01
68	->	78	0.07	0.05
68	->	79	-1.31	0.84
68	->	80	-0.03	0.01
68	->	81	0.02	0.01
68	->	82	-0.07	0.11
68	->	83	-0.16	0.12
68	->	84	-0.03	0.01
68	->	85	-1.69	1.78
68	->	86	-4.63	3.09
68	->	87	-0.11	0.09
68	->	88	-0.03	0.01
68	->	89	-0.01	0.00
68	->	90	-0.01	0.00
68	->	91	-0.00	0.00
68	->	92	-0.00	0.00
68	->	93	-0.00	0.00
68	->	94	-0.00	0.00

68	->	95	-0.00	0.00
68	->	96	-0.00	0.00
68	->	97	-0.00	0.00
68	->	98	-0.01	0.00
68	->	99	-0.01	0.00
68	->	100	-0.02	0.00
68	->	101	-0.02	0.01
68	->	102	-0.02	0.00
68	->	103	-0.02	0.00
68	->	104	-0.01	0.00
68	->	105	-0.02	0.00
68	->	106	-0.01	0.00
69	->	1	0.00	0.00
69	->	2	0.00	0.00
69	->	3	-0.00	0.00
69	->	4	-0.00	0.00
69	->	5	-0.00	0.00
69	->	6	0.00	0.00
69	->	7	0.00	0.00
69	->	8	-0.00	0.00
69	->	9	-0.00	0.00
69	->	10	-0.00	0.00
69	->	11	-0.00	0.00
69	->	12	0.00	0.00
69	->	13	0.00	0.00
69	->	14	0.00	0.00
69	->	15	0.00	0.00
69	->	16	0.00	0.00
69	->	17	-0.00	0.00
69	->	18	0.00	0.00
69	->	19	-0.00	0.00
69	->	20	-0.00	0.00
69	->	21	0.00	0.00
69	->	22	0.01	0.00
69	->	23	0.00	0.00
69	->	24	0.00	0.00
69	->	25	0.00	0.00
69	->	26	0.00	0.00
69	->	27	0.00	0.00
69	->	28	0.00	0.00
69	->	29	0.00	0.00
69	->	30	-0.00	0.00
69	->	31	0.00	0.00
69	->	32	-0.00	0.00
69	->	33	0.00	0.00
69	->	34	-0.00	0.00
69	->	35	-0.01	0.00
69	->	36	0.01	0.01
69	->	37	-0.00	0.00
69	->	38	-0.01	0.00

69	->	39	0.00	0.00
69	->	40	0.01	0.00
69	->	41	-0.00	0.00
69	->	42	0.00	0.00
69	->	43	0.00	0.00
69	->	44	0.01	0.00
69	->	45	-0.00	0.00
69	->	46	-0.02	0.00
69	->	47	-0.00	0.00
69	->	48	-0.00	0.00
69	->	49	0.00	0.00
69	->	50	-0.01	0.01
69	->	51	-0.00	0.00
69	->	52	-0.00	0.00
69	->	53	0.10	0.05
69	->	54	-0.03	0.03
69	->	55	-0.01	0.00
69	->	56	-0.01	0.00
69	->	57	-0.03	0.04
69	->	58	-0.07	0.07
69	->	59	-2.11	0.75
69	->	60	-0.49	0.53
69	->	61	-3.01	0.63
69	->	62	-1.41	0.34
69	->	63	-0.55	0.16
69	->	64	-0.16	0.14
69	->	65	-0.02	0.01
69	->	66	-0.05	0.03
69	->	67	-0.88	0.59
69	->	68	-13.17	0.99
69	->	69	9.40	2.71
69	->	70	-10.73	0.54
69	->	71	-0.40	0.06
69	->	72	-0.00	0.03
69	->	73	-0.04	0.02
69	->	74	-0.04	0.01
69	->	75	-0.40	0.19
69	->	76	0.08	0.05
69	->	77	-0.00	0.01
69	->	78	0.04	0.02
69	->	79	-0.02	0.01
69	->	80	-0.01	0.01
69	->	81	0.01	0.00
69	->	82	-0.00	0.00
69	->	83	-0.01	0.01
69	->	84	-0.01	0.00
69	->	85	-0.03	0.02
69	->	86	-0.14	0.07
69	->	87	-0.08	0.04
69	->	88	-0.02	0.00

69	->	89	-0.01	0.00
69	->	90	-0.01	0.00
69	->	91	-0.00	0.00
69	->	92	-0.00	0.00
69	->	93	-0.01	0.00
69	->	94	-0.00	0.00
69	->	95	-0.00	0.00
69	->	96	-0.00	0.00
69	->	97	-0.01	0.00
69	->	98	-0.01	0.00
69	->	99	-0.03	0.01
69	->	100	-0.05	0.02
69	->	101	-0.03	0.01
69	->	102	-0.01	0.00
69	->	103	-0.01	0.00
69	->	104	-0.01	0.00
69	->	105	-0.01	0.00
69	->	106	-0.00	0.00
70	->	1	0.00	0.00
70	->	2	0.00	0.00
70	->	3	0.00	0.00
70	->	4	0.00	0.00
70	->	5	-0.00	0.00
70	->	6	0.00	0.00
70	->	7	-0.00	0.00
70	->	8	-0.00	0.00
70	->	9	0.00	0.00
70	->	10	-0.00	0.00
70	->	11	-0.00	0.00
70	->	12	0.00	0.00
70	->	13	0.00	0.00
70	->	14	0.00	0.00
70	->	15	0.00	0.00
70	->	16	0.00	0.00
70	->	17	0.00	0.00
70	->	18	-0.00	0.00
70	->	19	-0.00	0.00
70	->	20	-0.00	0.00
70	->	21	0.00	0.00
70	->	22	0.00	0.00
70	->	23	0.00	0.00
70	->	24	-0.00	0.00
70	->	25	0.00	0.00
70	->	26	0.00	0.00
70	->	27	0.00	0.00
70	->	28	0.00	0.00
70	->	29	0.00	0.00
70	->	30	-0.00	0.00
70	->	31	0.00	0.00
70	->	32	0.00	0.00

70	->	33	0.00	0.00
70	->	34	0.00	0.00
70	->	35	-0.00	0.00
70	->	36	-0.00	0.00
70	->	37	-0.00	0.00
70	->	38	-0.00	0.00
70	->	39	-0.00	0.00
70	->	40	0.00	0.00
70	->	41	0.00	0.00
70	->	42	-0.00	0.00
70	->	43	0.00	0.00
70	->	44	0.00	0.00
70	->	45	0.00	0.00
70	->	46	-0.00	0.00
70	->	47	-0.00	0.00
70	->	48	-0.00	0.00
70	->	49	0.00	0.00
70	->	50	-0.03	0.02
70	->	51	-0.01	0.01
70	->	52	0.00	0.00
70	->	53	0.03	0.05
70	->	54	-0.06	0.06
70	->	55	-0.01	0.00
70	->	56	-0.01	0.00
70	->	57	-0.00	0.01
70	->	58	-0.24	0.09
70	->	59	-3.26	0.54
70	->	60	-0.83	0.15
70	->	61	-3.41	0.63
70	->	62	-1.29	0.44
70	->	63	-0.84	0.31
70	->	64	-0.01	0.01
70	->	65	-0.02	0.01
70	->	66	-0.01	0.03
70	->	67	-0.11	0.04
70	->	68	-2.88	1.04
70	->	69	-10.81	0.54
70	->	70	30.02	1.35
70	->	71	-9.76	0.29
70	->	72	-0.94	0.20
70	->	73	-0.21	0.06
70	->	74	-0.13	0.03
70	->	75	-4.85	1.16
70	->	76	-2.67	0.42
70	->	77	-0.06	0.02
70	->	78	-0.21	0.07
70	->	79	-1.93	0.49
70	->	80	-0.04	0.02
70	->	81	-0.01	0.01
70	->	82	-0.03	0.01

70	->	83	-0.02	0.01
70	->	84	-0.00	0.00
70	->	85	-0.05	0.06
70	->	86	-0.29	0.17
70	->	87	-0.19	0.11
70	->	88	-0.02	0.01
70	->	89	-0.01	0.00
70	->	90	-0.00	0.00
70	->	91	-0.00	0.00
70	->	92	-0.00	0.00
70	->	93	-0.00	0.00
70	->	94	-0.00	0.00
70	->	95	-0.00	0.00
70	->	96	-0.00	0.00
70	->	97	0.00	0.00
70	->	98	-0.00	0.00
70	->	99	-0.01	0.00
70	->	100	-0.02	0.02
70	->	101	-0.02	0.01
70	->	102	-0.01	0.00
70	->	103	-0.00	0.00
70	->	104	-0.00	0.00
70	->	105	-0.00	0.00
70	->	106	-0.00	0.00
71	->	1	-0.00	0.00
71	->	2	0.00	0.00
71	->	3	0.00	0.00
71	->	4	0.00	0.00
71	->	5	0.00	0.00
71	->	6	0.00	0.00
71	->	7	0.00	0.00
71	->	8	0.00	0.00
71	->	9	0.00	0.00
71	->	10	0.00	0.00
71	->	11	0.00	0.00
71	->	12	0.00	0.00
71	->	13	-0.00	0.00
71	->	14	0.00	0.00
71	->	15	-0.00	0.00
71	->	16	-0.00	0.00
71	->	17	0.00	0.00
71	->	18	0.00	0.00
71	->	19	0.00	0.00
71	->	20	-0.00	0.00
71	->	21	0.00	0.00
71	->	22	-0.00	0.00
71	->	23	-0.00	0.00
71	->	24	-0.00	0.00
71	->	25	-0.00	0.00
71	->	26	-0.00	0.00

71 ->	27	-0.00	0.00
71 ->	28	0.00	0.00
71 ->	29	0.00	0.00
71 ->	30	0.00	0.00
71 ->	31	-0.00	0.00
71 ->	32	-0.00	0.00
71 ->	33	-0.00	0.00
71 ->	34	0.00	0.00
71 ->	35	-0.00	0.00
71 ->	36	-0.00	0.00
71 ->	37	-0.00	0.00
71 ->	38	-0.00	0.00
71 ->	39	0.00	0.00
71 ->	40	-0.00	0.00
71 ->	41	0.00	0.00
71 ->	42	-0.00	0.00
71 ->	43	0.00	0.00
71 ->	44	0.00	0.00
71 ->	45	-0.00	0.00
71 ->	46	-0.01	0.01
71 ->	47	-0.01	0.01
71 ->	48	-0.01	0.01
71 ->	49	-0.06	0.05
71 ->	50	-0.71	0.51
71 ->	51	-0.04	0.03
71 ->	52	-0.02	0.01
71 ->	53	-3.29	2.24
71 ->	54	-0.42	0.32
71 ->	55	-0.01	0.01
71 ->	56	-0.02	0.00
71 ->	57	-0.03	0.01
71 ->	58	-0.61	0.29
71 ->	59	-1.78	0.32
71 ->	60	-0.72	0.28
71 ->	61	-0.95	0.32
71 ->	62	-0.02	0.01
71 ->	63	0.00	0.01
71 ->	64	0.00	0.00
71 ->	65	-0.00	0.00
71 ->	66	-0.00	0.00
71 ->	67	-0.00	0.00
71 ->	68	-0.06	0.05
71 ->	69	-0.40	0.06
71 ->	70	-9.76	0.29
71 ->	71	24.04	1.38
71 ->	72	-7.75	0.46
71 ->	73	-0.30	0.07
71 ->	74	-0.17	0.06
71 ->	75	-3.95	1.62
71 ->	76	-0.03	0.02

71	->	77	-0.01	0.00
71	->	78	0.00	0.01
71	->	79	-0.00	0.00
71	->	80	-0.00	0.00
71	->	81	0.00	0.00
71	->	82	-0.00	0.00
71	->	83	-0.00	0.00
71	->	84	-0.00	0.00
71	->	85	-0.00	0.00
71	->	86	-0.13	0.11
71	->	87	-0.38	0.28
71	->	88	-0.04	0.01
71	->	89	-0.01	0.00
71	->	90	-0.00	0.00
71	->	91	0.00	0.00
71	->	92	0.00	0.00
71	->	93	-0.00	0.00
71	->	94	0.00	0.00
71	->	95	0.00	0.00
71	->	96	0.00	0.00
71	->	97	0.00	0.00
71	->	98	-0.00	0.00
71	->	99	-0.01	0.01
71	->	100	0.01	0.05
71	->	101	-0.03	0.02
71	->	102	-0.01	0.00
71	->	103	0.01	0.01
71	->	104	-0.00	0.00
71	->	105	-0.00	0.00
71	->	106	-0.00	0.00
72	->	1	0.02	0.00
72	->	2	0.00	0.00
72	->	3	-0.00	0.00
72	->	4	-0.00	0.00
72	->	5	-0.01	0.00
72	->	6	0.00	0.00
72	->	7	-0.00	0.00
72	->	8	-0.00	0.00
72	->	9	-0.00	0.00
72	->	10	-0.01	0.00
72	->	11	-0.01	0.00
72	->	12	0.02	0.00
72	->	13	0.00	0.00
72	->	14	0.00	0.00
72	->	15	0.00	0.00
72	->	16	0.02	0.00
72	->	17	-0.00	0.00
72	->	18	-0.00	0.00
72	->	19	-0.02	0.00
72	->	20	-0.00	0.00

72	->	21	0.00	0.00
72	->	22	0.04	0.01
72	->	23	0.00	0.00
72	->	24	0.00	0.00
72	->	25	0.02	0.00
72	->	26	0.00	0.00
72	->	27	0.00	0.00
72	->	28	0.00	0.00
72	->	29	0.00	0.00
72	->	30	-0.00	0.00
72	->	31	0.03	0.00
72	->	32	0.00	0.00
72	->	33	-0.00	0.00
72	->	34	-0.00	0.00
72	->	35	-0.03	0.00
72	->	36	0.02	0.00
72	->	37	-0.00	0.00
72	->	38	-0.03	0.00
72	->	39	-0.00	0.00
72	->	40	0.05	0.02
72	->	41	-0.00	0.00
72	->	42	-0.01	0.01
72	->	43	0.00	0.00
72	->	44	0.13	0.04
72	->	45	-0.00	0.00
72	->	46	-0.13	0.03
72	->	47	-0.18	0.10
72	->	48	-0.02	0.01
72	->	49	0.06	0.03
72	->	50	-1.59	0.46
72	->	51	-0.09	0.03
72	->	52	-0.02	0.01
72	->	53	-0.24	0.22
72	->	54	-0.09	0.05
72	->	55	-0.01	0.00
72	->	56	-0.00	0.00
72	->	57	-0.01	0.00
72	->	58	-0.04	0.01
72	->	59	-0.05	0.04
72	->	60	-0.14	0.07
72	->	61	-2.18	0.77
72	->	62	-0.03	0.01
72	->	63	-0.04	0.01
72	->	64	-0.02	0.00
72	->	65	-0.00	0.00
72	->	66	-0.02	0.00
72	->	67	-0.00	0.00
72	->	68	0.03	0.01
72	->	69	-0.00	0.03
72	->	70	-0.94	0.20

72	->	71	-7.84	0.47
72	->	72	-92.13	2.48
72	->	73	-21.03	0.67
72	->	74	-14.97	1.31
72	->	75	-4.86	1.18
72	->	76	-0.96	0.47
72	->	77	0.11	0.05
72	->	78	0.30	0.13
72	->	79	-0.01	0.01
72	->	80	-0.01	0.01
72	->	81	0.04	0.01
72	->	82	-0.00	0.00
72	->	83	-0.00	0.00
72	->	84	-0.01	0.00
72	->	85	-0.03	0.01
72	->	86	-0.34	0.26
72	->	87	-2.63	0.84
72	->	88	-9.38	0.92
72	->	89	-2.38	0.62
72	->	90	-0.12	0.09
72	->	91	-0.01	0.02
72	->	92	0.00	0.02
72	->	93	-0.02	0.00
72	->	94	-0.01	0.00
72	->	95	-0.01	0.00
72	->	96	-0.00	0.00
72	->	97	-0.01	0.00
72	->	98	-0.03	0.01
72	->	99	-0.13	0.05
72	->	100	-0.34	0.29
72	->	101	-0.46	0.21
72	->	102	-0.17	0.23
72	->	103	0.60	0.75
72	->	104	0.37	0.20
72	->	105	0.18	0.18
72	->	106	-0.06	0.02
73	->	1	0.00	0.00
73	->	2	0.00	0.00
73	->	3	-0.00	0.00
73	->	4	0.00	0.00
73	->	5	-0.00	0.00
73	->	6	0.00	0.00
73	->	7	0.00	0.00
73	->	8	-0.00	0.00
73	->	9	0.00	0.00
73	->	10	-0.00	0.00
73	->	11	-0.00	0.00
73	->	12	0.00	0.00
73	->	13	0.00	0.00
73	->	14	0.00	0.00

73	->	15	0.00	0.00
73	->	16	0.00	0.00
73	->	17	-0.00	0.00
73	->	18	0.00	0.00
73	->	19	-0.00	0.00
73	->	20	0.00	0.00
73	->	21	0.00	0.00
73	->	22	0.00	0.00
73	->	23	0.00	0.00
73	->	24	0.00	0.00
73	->	25	0.00	0.00
73	->	26	0.00	0.00
73	->	27	0.00	0.00
73	->	28	0.00	0.00
73	->	29	0.00	0.00
73	->	30	-0.00	0.00
73	->	31	0.00	0.00
73	->	32	0.00	0.00
73	->	33	0.00	0.00
73	->	34	-0.00	0.00
73	->	35	-0.00	0.00
73	->	36	0.00	0.00
73	->	37	0.00	0.00
73	->	38	-0.00	0.00
73	->	39	0.00	0.00
73	->	40	0.00	0.00
73	->	41	0.00	0.00
73	->	42	-0.00	0.00
73	->	43	-0.00	0.00
73	->	44	0.00	0.01
73	->	45	-0.00	0.00
73	->	46	-0.01	0.00
73	->	47	-0.01	0.00
73	->	48	-0.00	0.00
73	->	49	-0.01	0.01
73	->	50	-0.03	0.02
73	->	51	0.00	0.00
73	->	52	-0.00	0.00
73	->	53	-0.14	0.10
73	->	54	0.00	0.00
73	->	55	-0.00	0.00
73	->	56	-0.00	0.00
73	->	57	-0.00	0.00
73	->	58	-0.01	0.00
73	->	59	-0.06	0.02
73	->	60	-0.06	0.02
73	->	61	-1.99	0.68
73	->	62	-0.01	0.01
73	->	63	0.00	0.01
73	->	64	-0.00	0.00

73	->	65	-0.00	0.00
73	->	66	0.00	0.00
73	->	67	-0.00	0.00
73	->	68	-0.01	0.01
73	->	69	-0.04	0.02
73	->	70	-0.21	0.06
73	->	71	-0.30	0.07
73	->	72	-21.03	0.67
73	->	73	24.77	2.95
73	->	74	-10.62	0.53
73	->	75	-0.86	0.23
73	->	76	-3.02	0.74
73	->	77	-1.93	0.88
73	->	78	-0.11	0.06
73	->	79	-0.05	0.03
73	->	80	-0.01	0.02
73	->	81	-0.01	0.00
73	->	82	-0.00	0.00
73	->	83	-0.00	0.00
73	->	84	0.00	0.00
73	->	85	-0.00	0.00
73	->	86	0.00	0.01
73	->	87	-0.01	0.00
73	->	88	-0.01	0.00
73	->	89	-0.01	0.00
73	->	90	-0.00	0.01
73	->	91	0.00	0.02
73	->	92	-0.01	0.02
73	->	93	-0.01	0.00
73	->	94	-0.00	0.00
73	->	95	-0.00	0.00
73	->	96	-0.00	0.00
73	->	97	-0.00	0.00
73	->	98	-0.01	0.00
73	->	99	-0.02	0.03
73	->	100	-1.06	0.61
73	->	101	-4.33	2.52
73	->	102	-0.21	0.19
73	->	103	-0.04	0.03
73	->	104	-0.00	0.00
73	->	105	-0.00	0.00
73	->	106	-0.00	0.00
74	->	1	-0.02	0.00
74	->	2	-0.00	0.00
74	->	3	0.00	0.00
74	->	4	-0.00	0.00
74	->	5	0.01	0.00
74	->	6	-0.00	0.00
74	->	7	-0.00	0.00
74	->	8	0.00	0.00

74	->	9	0.00	0.00
74	->	10	0.01	0.00
74	->	11	0.01	0.00
74	->	12	-0.02	0.00
74	->	13	-0.00	0.00
74	->	14	-0.00	0.00
74	->	15	-0.00	0.00
74	->	16	-0.02	0.00
74	->	17	0.00	0.00
74	->	18	0.00	0.00
74	->	19	0.02	0.00
74	->	20	0.00	0.00
74	->	21	-0.00	0.00
74	->	22	-0.04	0.01
74	->	23	-0.00	0.00
74	->	24	-0.00	0.00
74	->	25	-0.02	0.00
74	->	26	-0.00	0.00
74	->	27	-0.00	0.00
74	->	28	-0.00	0.00
74	->	29	-0.00	0.00
74	->	30	0.00	0.00
74	->	31	-0.02	0.00
74	->	32	-0.00	0.00
74	->	33	-0.00	0.00
74	->	34	-0.00	0.00
74	->	35	0.02	0.00
74	->	36	-0.01	0.00
74	->	37	0.00	0.00
74	->	38	0.02	0.00
74	->	39	-0.00	0.00
74	->	40	-0.03	0.01
74	->	41	0.00	0.00
74	->	42	-0.00	0.00
74	->	43	-0.00	0.00
74	->	44	-0.10	0.03
74	->	45	0.00	0.00
74	->	46	0.06	0.01
74	->	47	-0.00	0.01
74	->	48	-0.00	0.00
74	->	49	-0.02	0.01
74	->	50	-0.03	0.02
74	->	51	-0.00	0.00
74	->	52	-0.00	0.00
74	->	53	-0.11	0.03
74	->	54	-0.00	0.00
74	->	55	-0.00	0.00
74	->	56	-0.00	0.00
74	->	57	-0.00	0.00
74	->	58	0.03	0.01

74	->	59	-0.07	0.01
74	->	60	-0.01	0.00
74	->	61	0.01	0.02
74	->	62	0.00	0.00
74	->	63	0.03	0.00
74	->	64	0.02	0.00
74	->	65	-0.00	0.00
74	->	66	0.03	0.00
74	->	67	-0.00	0.00
74	->	68	-0.04	0.01
74	->	69	-0.04	0.01
74	->	70	-0.13	0.03
74	->	71	-0.17	0.06
74	->	72	-14.88	1.32
74	->	73	-10.67	0.53
74	->	74	-14.05	1.79
74	->	75	-13.34	0.51
74	->	76	-1.46	0.28
74	->	77	-2.78	0.45
74	->	78	-2.24	0.57
74	->	79	-0.09	0.07
74	->	80	-0.04	0.01
74	->	81	-0.13	0.06
74	->	82	-0.01	0.00
74	->	83	-0.01	0.00
74	->	84	0.02	0.00
74	->	85	-0.00	0.01
74	->	86	-0.00	0.03
74	->	87	-0.46	0.49
74	->	88	0.58	0.18
74	->	89	0.25	0.14
74	->	90	-0.01	0.06
74	->	91	-0.04	0.04
74	->	92	-0.02	0.02
74	->	93	0.02	0.00
74	->	94	0.01	0.00
74	->	95	0.01	0.00
74	->	96	0.00	0.00
74	->	97	0.01	0.00
74	->	98	0.02	0.00
74	->	99	0.04	0.01
74	->	100	0.03	0.04
74	->	101	-0.22	0.28
74	->	102	-2.24	1.25
74	->	103	-4.02	1.34
74	->	104	-0.65	0.29
74	->	105	0.02	0.04
74	->	106	0.01	0.00
75	->	1	-0.01	0.00
75	->	2	-0.00	0.00

75	->	3	0.00	0.00
75	->	4	0.00	0.00
75	->	5	0.00	0.00
75	->	6	-0.00	0.00
75	->	7	-0.00	0.00
75	->	8	0.00	0.00
75	->	9	0.00	0.00
75	->	10	0.01	0.00
75	->	11	0.00	0.00
75	->	12	-0.01	0.00
75	->	13	-0.00	0.00
75	->	14	-0.00	0.00
75	->	15	-0.00	0.00
75	->	16	-0.01	0.00
75	->	17	0.00	0.00
75	->	18	0.00	0.00
75	->	19	0.01	0.00
75	->	20	0.00	0.00
75	->	21	-0.00	0.00
75	->	22	-0.02	0.00
75	->	23	-0.00	0.00
75	->	24	0.00	0.00
75	->	25	-0.01	0.00
75	->	26	-0.00	0.00
75	->	27	-0.00	0.00
75	->	28	-0.00	0.00
75	->	29	-0.00	0.00
75	->	30	0.00	0.00
75	->	31	-0.02	0.00
75	->	32	-0.00	0.00
75	->	33	-0.00	0.00
75	->	34	-0.00	0.00
75	->	35	0.02	0.00
75	->	36	-0.02	0.01
75	->	37	0.00	0.00
75	->	38	0.03	0.00
75	->	39	-0.00	0.00
75	->	40	-0.03	0.01
75	->	41	0.00	0.00
75	->	42	-0.00	0.00
75	->	43	-0.00	0.00
75	->	44	-0.03	0.01
75	->	45	0.00	0.00
75	->	46	0.04	0.01
75	->	47	-0.00	0.00
75	->	48	0.00	0.00
75	->	49	-0.01	0.01
75	->	50	-0.04	0.03
75	->	51	-0.01	0.01
75	->	52	-0.00	0.00

75	->	53	-0.18	0.09
75	->	54	0.03	0.07
75	->	55	-0.00	0.00
75	->	56	-0.00	0.00
75	->	57	-0.01	0.00
75	->	58	0.06	0.02
75	->	59	-0.75	0.46
75	->	60	-0.02	0.01
75	->	61	-0.15	0.06
75	->	62	-0.02	0.01
75	->	63	-0.01	0.02
75	->	64	0.02	0.00
75	->	65	-0.01	0.00
75	->	66	0.04	0.01
75	->	67	0.00	0.00
75	->	68	-0.63	1.72
75	->	69	-0.40	0.19
75	->	70	-4.80	1.14
75	->	71	-3.89	1.59
75	->	72	-4.83	1.16
75	->	73	-0.87	0.23
75	->	74	-13.41	0.51
75	->	75	-44.93	3.49
75	->	76	-7.47	0.55
75	->	77	-1.26	0.21
75	->	78	-3.27	1.17
75	->	79	-2.95	0.74
75	->	80	-0.15	0.06
75	->	81	-0.09	0.02
75	->	82	-0.04	0.01
75	->	83	-0.02	0.00
75	->	84	0.02	0.00
75	->	85	-0.04	0.05
75	->	86	-0.69	0.51
75	->	87	-0.83	0.94
75	->	88	0.07	0.06
75	->	89	0.01	0.01
75	->	90	0.01	0.00
75	->	91	0.00	0.00
75	->	92	0.01	0.00
75	->	93	0.01	0.00
75	->	94	0.01	0.00
75	->	95	0.00	0.00
75	->	96	0.00	0.00
75	->	97	0.01	0.00
75	->	98	0.01	0.00
75	->	99	0.02	0.00
75	->	100	0.02	0.01
75	->	101	-0.01	0.03
75	->	102	-0.01	0.01

75	->	103	-0.02	0.03
75	->	104	-0.04	0.06
75	->	105	0.01	0.02
75	->	106	0.01	0.00
76	->	1	0.01	0.00
76	->	2	0.00	0.00
76	->	3	-0.00	0.00
76	->	4	-0.00	0.00
76	->	5	-0.00	0.00
76	->	6	0.00	0.00
76	->	7	0.00	0.00
76	->	8	-0.00	0.00
76	->	9	-0.00	0.00
76	->	10	-0.00	0.00
76	->	11	-0.00	0.00
76	->	12	0.01	0.00
76	->	13	0.00	0.00
76	->	14	0.00	0.00
76	->	15	0.00	0.00
76	->	16	0.01	0.00
76	->	17	-0.00	0.00
76	->	18	0.00	0.00
76	->	19	-0.01	0.00
76	->	20	-0.00	0.00
76	->	21	0.00	0.00
76	->	22	0.01	0.00
76	->	23	0.00	0.00
76	->	24	0.00	0.00
76	->	25	0.01	0.00
76	->	26	0.00	0.00
76	->	27	0.00	0.00
76	->	28	0.00	0.00
76	->	29	0.00	0.00
76	->	30	-0.00	0.00
76	->	31	0.01	0.00
76	->	32	0.00	0.00
76	->	33	0.00	0.00
76	->	34	-0.00	0.00
76	->	35	-0.01	0.00
76	->	36	0.01	0.00
76	->	37	-0.00	0.00
76	->	38	-0.01	0.00
76	->	39	0.00	0.00
76	->	40	0.01	0.00
76	->	41	-0.00	0.00
76	->	42	0.00	0.00
76	->	43	0.00	0.00
76	->	44	0.02	0.00
76	->	45	-0.00	0.00
76	->	46	-0.03	0.01

76	->	47	-0.00	0.00
76	->	48	-0.00	0.00
76	->	49	-0.00	0.00
76	->	50	-0.00	0.00
76	->	51	0.00	0.00
76	->	52	-0.00	0.00
76	->	53	0.04	0.02
76	->	54	-0.00	0.00
76	->	55	0.00	0.00
76	->	56	-0.00	0.00
76	->	57	-0.00	0.00
76	->	58	-0.03	0.01
76	->	59	0.03	0.01
76	->	60	-0.04	0.01
76	->	61	-1.33	0.69
76	->	62	-2.45	1.52
76	->	63	-1.50	0.70
76	->	64	-0.94	1.62
76	->	65	-0.03	0.03
76	->	66	-0.08	0.02
76	->	67	-0.01	0.01
76	->	68	-0.06	0.04
76	->	69	0.08	0.05
76	->	70	-2.71	0.42
76	->	71	-0.03	0.02
76	->	72	-0.97	0.47
76	->	73	-3.04	0.74
76	->	74	-1.47	0.28
76	->	75	-7.50	0.55
76	->	76	-106.33	2.49
76	->	77	-11.70	0.83
76	->	78	-0.82	0.26
76	->	79	-2.14	0.46
76	->	80	-3.00	1.43
76	->	81	-0.00	0.06
76	->	82	-0.04	0.02
76	->	83	-0.08	0.03
76	->	84	-0.05	0.02
76	->	85	-0.02	0.01
76	->	86	-0.05	0.02
76	->	87	-0.04	0.01
76	->	88	-0.02	0.00
76	->	89	-0.02	0.00
76	->	90	-0.01	0.00
76	->	91	-0.01	0.00
76	->	92	-0.01	0.00
76	->	93	-0.02	0.01
76	->	94	-0.01	0.01
76	->	95	-0.01	0.00
76	->	96	-0.00	0.00

76	->	97	-0.01	0.00
76	->	98	-0.01	0.00
76	->	99	-0.03	0.01
76	->	100	-0.18	0.33
76	->	101	-0.82	1.81
76	->	102	-0.28	0.19
76	->	103	-0.08	0.05
76	->	104	-0.02	0.00
76	->	105	-0.02	0.00
76	->	106	-0.01	0.00
77	->	1	0.02	0.01
77	->	2	0.00	0.00
77	->	3	-0.00	0.00
77	->	4	0.00	0.00
77	->	5	-0.01	0.00
77	->	6	0.00	0.00
77	->	7	0.00	0.00
77	->	8	-0.00	0.00
77	->	9	-0.00	0.00
77	->	10	-0.01	0.00
77	->	11	-0.01	0.00
77	->	12	0.02	0.00
77	->	13	0.00	0.00
77	->	14	0.00	0.00
77	->	15	0.00	0.00
77	->	16	0.02	0.00
77	->	17	-0.00	0.00
77	->	18	0.00	0.00
77	->	19	-0.02	0.00
77	->	20	-0.00	0.00
77	->	21	0.00	0.00
77	->	22	0.02	0.00
77	->	23	0.00	0.00
77	->	24	0.00	0.00
77	->	25	0.01	0.00
77	->	26	0.00	0.00
77	->	27	0.00	0.00
77	->	28	0.00	0.00
77	->	29	0.00	0.00
77	->	30	-0.00	0.00
77	->	31	0.01	0.00
77	->	32	0.00	0.00
77	->	33	0.00	0.00
77	->	34	0.00	0.00
77	->	35	-0.01	0.00
77	->	36	0.01	0.00
77	->	37	-0.00	0.00
77	->	38	-0.01	0.00
77	->	39	-0.00	0.00
77	->	40	0.01	0.00

77	->	41	0.00	0.00
77	->	42	0.00	0.00
77	->	43	0.00	0.00
77	->	44	0.03	0.00
77	->	45	-0.00	0.00
77	->	46	-0.03	0.01
77	->	47	-0.00	0.00
77	->	48	-0.00	0.00
77	->	49	-0.00	0.00
77	->	50	-0.00	0.00
77	->	51	0.00	0.00
77	->	52	0.00	0.00
77	->	53	0.03	0.01
77	->	54	0.00	0.00
77	->	55	0.00	0.00
77	->	56	0.00	0.00
77	->	57	0.00	0.00
77	->	58	-0.02	0.01
77	->	59	0.01	0.01
77	->	60	-0.01	0.00
77	->	61	-0.04	0.02
77	->	62	-0.01	0.01
77	->	63	-0.03	0.02
77	->	64	-0.03	0.01
77	->	65	-0.00	0.00
77	->	66	-0.03	0.00
77	->	67	-0.00	0.00
77	->	68	0.01	0.01
77	->	69	-0.00	0.01
77	->	70	-0.06	0.02
77	->	71	-0.01	0.00
77	->	72	0.11	0.05
77	->	73	-1.96	0.89
77	->	74	-2.80	0.45
77	->	75	-1.27	0.21
77	->	76	-11.74	0.85
77	->	77	11.99	3.19
77	->	78	-6.55	0.44
77	->	79	-0.87	0.20
77	->	80	-2.10	0.39
77	->	81	-1.40	0.72
77	->	82	-0.05	0.03
77	->	83	-0.04	0.02
77	->	84	-0.06	0.02
77	->	85	-0.03	0.02
77	->	86	-0.06	0.02
77	->	87	-0.02	0.01
77	->	88	-0.03	0.01
77	->	89	-0.02	0.00
77	->	90	-0.01	0.00

77	->	91	-0.01	0.01
77	->	92	-0.03	0.01
77	->	93	-0.04	0.01
77	->	94	-0.03	0.01
77	->	95	-0.01	0.00
77	->	96	-0.00	0.00
77	->	97	-0.01	0.00
77	->	98	-0.01	0.00
77	->	99	-0.02	0.01
77	->	100	-0.09	0.07
77	->	101	-1.12	0.80
77	->	102	-7.12	2.35
77	->	103	-2.60	2.70
77	->	104	-0.06	0.02
77	->	105	-0.04	0.01
77	->	106	-0.01	0.00
78	->	1	0.01	0.00
78	->	2	0.00	0.00
78	->	3	-0.00	0.00
78	->	4	0.00	0.00
78	->	5	-0.01	0.00
78	->	6	0.00	0.00
78	->	7	0.00	0.00
78	->	8	-0.00	0.00
78	->	9	-0.00	0.00
78	->	10	-0.01	0.00
78	->	11	-0.01	0.00
78	->	12	0.01	0.00
78	->	13	0.00	0.00
78	->	14	0.00	0.00
78	->	15	0.00	0.00
78	->	16	0.01	0.00
78	->	17	-0.00	0.00
78	->	18	-0.00	0.00
78	->	19	-0.01	0.00
78	->	20	-0.00	0.00
78	->	21	-0.00	0.00
78	->	22	0.02	0.00
78	->	23	0.00	0.00
78	->	24	-0.00	0.00
78	->	25	0.01	0.00
78	->	26	0.00	0.00
78	->	27	0.00	0.00
78	->	28	0.00	0.00
78	->	29	0.00	0.00
78	->	30	-0.00	0.00
78	->	31	0.02	0.00
78	->	32	0.00	0.00
78	->	33	-0.00	0.00
78	->	34	0.00	0.00

78	->	35	-0.01	0.00
78	->	36	0.01	0.00
78	->	37	-0.00	0.00
78	->	38	-0.01	0.00
78	->	39	0.00	0.00
78	->	40	0.02	0.00
78	->	41	-0.00	0.00
78	->	42	0.00	0.00
78	->	43	0.00	0.00
78	->	44	0.03	0.00
78	->	45	-0.00	0.00
78	->	46	-0.03	0.00
78	->	47	-0.00	0.00
78	->	48	-0.00	0.00
78	->	49	-0.00	0.00
78	->	50	-0.00	0.00
78	->	51	0.00	0.00
78	->	52	-0.00	0.00
78	->	53	0.03	0.00
78	->	54	-0.00	0.00
78	->	55	0.00	0.00
78	->	56	0.00	0.00
78	->	57	0.00	0.00
78	->	58	-0.02	0.00
78	->	59	0.04	0.01
78	->	60	-0.00	0.00
78	->	61	-0.01	0.00
78	->	62	0.01	0.01
78	->	63	-0.13	0.03
78	->	64	-0.02	0.01
78	->	65	0.00	0.00
78	->	66	-0.06	0.01
78	->	67	-0.00	0.00
78	->	68	0.07	0.05
78	->	69	0.04	0.02
78	->	70	-0.21	0.07
78	->	71	0.00	0.01
78	->	72	0.30	0.13
78	->	73	-0.11	0.06
78	->	74	-2.28	0.56
78	->	75	-3.29	1.18
78	->	76	-0.82	0.26
78	->	77	-6.59	0.43
78	->	78	-103.34	3.46
78	->	79	-19.63	0.42
78	->	80	-0.69	0.16
78	->	81	-2.19	0.50
78	->	82	-1.46	0.96
78	->	83	-0.12	0.05
78	->	84	-0.07	0.02

78	->	85	-1.33	1.49
78	->	86	-4.03	2.07
78	->	87	-0.03	0.62
78	->	88	-0.21	0.14
78	->	89	-0.04	0.01
78	->	90	-0.03	0.01
78	->	91	-0.02	0.00
78	->	92	-0.02	0.00
78	->	93	-0.01	0.00
78	->	94	-0.01	0.00
78	->	95	-0.00	0.00
78	->	96	-0.00	0.00
78	->	97	-0.01	0.00
78	->	98	-0.01	0.00
78	->	99	-0.02	0.00
78	->	100	-0.03	0.00
78	->	101	-0.05	0.01
78	->	102	-0.11	0.04
78	->	103	-0.34	0.44
78	->	104	-1.25	1.21
78	->	105	-5.68	2.36
78	->	106	-0.16	0.31
79	->	1	0.00	0.00
79	->	2	0.00	0.00
79	->	3	0.00	0.00
79	->	4	0.00	0.00
79	->	5	-0.00	0.00
79	->	6	0.00	0.00
79	->	7	0.00	0.00
79	->	8	-0.00	0.00
79	->	9	0.00	0.00
79	->	10	-0.00	0.00
79	->	11	-0.00	0.00
79	->	12	0.00	0.00
79	->	13	0.00	0.00
79	->	14	0.00	0.00
79	->	15	0.00	0.00
79	->	16	0.00	0.00
79	->	17	-0.00	0.00
79	->	18	0.00	0.00
79	->	19	-0.00	0.00
79	->	20	-0.00	0.00
79	->	21	0.00	0.00
79	->	22	0.00	0.00
79	->	23	0.00	0.00
79	->	24	0.00	0.00
79	->	25	0.00	0.00
79	->	26	0.00	0.00
79	->	27	0.00	0.00
79	->	28	0.00	0.00

79	->	29	0.00	0.00
79	->	30	-0.00	0.00
79	->	31	0.00	0.00
79	->	32	0.00	0.00
79	->	33	0.00	0.00
79	->	34	0.00	0.00
79	->	35	-0.00	0.00
79	->	36	0.00	0.00
79	->	37	-0.00	0.00
79	->	38	-0.00	0.00
79	->	39	0.00	0.00
79	->	40	0.00	0.00
79	->	41	0.00	0.00
79	->	42	0.00	0.00
79	->	43	0.00	0.00
79	->	44	0.00	0.00
79	->	45	-0.00	0.00
79	->	46	-0.00	0.00
79	->	47	-0.00	0.00
79	->	48	-0.00	0.00
79	->	49	-0.00	0.00
79	->	50	-0.00	0.00
79	->	51	-0.00	0.00
79	->	52	-0.00	0.00
79	->	53	0.00	0.00
79	->	54	-0.00	0.00
79	->	55	0.00	0.00
79	->	56	0.00	0.00
79	->	57	-0.00	0.00
79	->	58	-0.00	0.00
79	->	59	-0.00	0.00
79	->	60	-0.00	0.00
79	->	61	-0.02	0.01
79	->	62	-0.11	0.05
79	->	63	2.02	0.66
79	->	64	-0.01	0.07
79	->	65	-0.21	0.14
79	->	66	2.29	0.91
79	->	67	-0.07	0.06
79	->	68	-1.26	0.81
79	->	69	-0.02	0.01
79	->	70	-1.94	0.48
79	->	71	-0.00	0.00
79	->	72	-0.01	0.01
79	->	73	-0.05	0.03
79	->	74	-0.09	0.07
79	->	75	-2.98	0.74
79	->	76	-2.15	0.45
79	->	77	-0.88	0.20
79	->	78	-19.67	0.42

79	->	79	9.60	1.69
79	->	80	-4.93	0.61
79	->	81	-1.16	0.27
79	->	82	-3.85	0.97
79	->	83	25.16	13.50
79	->	84	-0.04	0.04
79	->	85	-0.21	0.23
79	->	86	-0.13	0.18
79	->	87	-0.02	0.01
79	->	88	-0.01	0.00
79	->	89	-0.00	0.00
79	->	90	-0.00	0.00
79	->	91	-0.00	0.00
79	->	92	-0.00	0.00
79	->	93	-0.00	0.00
79	->	94	-0.00	0.00
79	->	95	-0.00	0.00
79	->	96	-0.00	0.00
79	->	97	-0.00	0.00
79	->	98	-0.00	0.00
79	->	99	-0.00	0.00
79	->	100	-0.01	0.00
79	->	101	-0.01	0.01
79	->	102	-0.01	0.01
79	->	103	-0.01	0.00
79	->	104	-0.01	0.00
79	->	105	-0.01	0.00
79	->	106	-0.00	0.00
80	->	1	0.00	0.00
80	->	2	0.00	0.00
80	->	3	0.00	0.00
80	->	4	0.00	0.00
80	->	5	-0.00	0.00
80	->	6	0.00	0.00
80	->	7	0.00	0.00
80	->	8	-0.00	0.00
80	->	9	0.00	0.00
80	->	10	-0.00	0.00
80	->	11	-0.00	0.00
80	->	12	0.00	0.00
80	->	13	0.00	0.00
80	->	14	0.00	0.00
80	->	15	0.00	0.00
80	->	16	0.00	0.00
80	->	17	0.00	0.00
80	->	18	0.00	0.00
80	->	19	-0.00	0.00
80	->	20	0.00	0.00
80	->	21	0.00	0.00
80	->	22	0.00	0.00

80 ->	23	0.00	0.00
80 ->	24	0.00	0.00
80 ->	25	0.00	0.00
80 ->	26	0.00	0.00
80 ->	27	0.00	0.00
80 ->	28	0.00	0.00
80 ->	29	0.00	0.00
80 ->	30	-0.00	0.00
80 ->	31	0.00	0.00
80 ->	32	0.00	0.00
80 ->	33	0.00	0.00
80 ->	34	0.00	0.00
80 ->	35	-0.00	0.00
80 ->	36	0.00	0.00
80 ->	37	-0.00	0.00
80 ->	38	-0.00	0.00
80 ->	39	0.00	0.00
80 ->	40	0.00	0.00
80 ->	41	0.00	0.00
80 ->	42	0.00	0.00
80 ->	43	0.00	0.00
80 ->	44	0.00	0.00
80 ->	45	-0.00	0.00
80 ->	46	-0.00	0.00
80 ->	47	-0.00	0.00
80 ->	48	-0.00	0.00
80 ->	49	0.00	0.00
80 ->	50	-0.00	0.00
80 ->	51	0.00	0.00
80 ->	52	0.00	0.00
80 ->	53	0.00	0.00
80 ->	54	0.00	0.00
80 ->	55	0.00	0.00
80 ->	56	0.00	0.00
80 ->	57	0.00	0.00
80 ->	58	-0.00	0.00
80 ->	59	-0.00	0.00
80 ->	60	-0.00	0.00
80 ->	61	-0.01	0.01
80 ->	62	-0.03	0.02
80 ->	63	0.10	0.18
80 ->	64	-0.03	0.06
80 ->	65	-0.15	0.33
80 ->	66	0.02	0.01
80 ->	67	-0.00	0.00
80 ->	68	-0.03	0.01
80 ->	69	-0.01	0.01
80 ->	70	-0.04	0.02
80 ->	71	-0.00	0.00
80 ->	72	-0.01	0.01

80	->	73	-0.01	0.02
80	->	74	-0.04	0.01
80	->	75	-0.15	0.06
80	->	76	-2.98	1.43
80	->	77	-2.10	0.39
80	->	78	-0.69	0.15
80	->	79	-4.96	0.61
80	->	80	-23.43	2.09
80	->	81	-15.23	0.46
80	->	82	-0.74	0.25
80	->	83	-2.78	0.76
80	->	84	-1.09	1.00
80	->	85	-0.01	0.01
80	->	86	-0.01	0.00
80	->	87	-0.00	0.00
80	->	88	-0.00	0.00
80	->	89	-0.00	0.00
80	->	90	-0.00	0.00
80	->	91	-0.00	0.00
80	->	92	-0.00	0.00
80	->	93	-0.00	0.00
80	->	94	-0.00	0.00
80	->	95	-0.00	0.00
80	->	96	-0.00	0.00
80	->	97	-0.00	0.00
80	->	98	-0.00	0.00
80	->	99	-0.00	0.00
80	->	100	-0.00	0.00
80	->	101	-0.01	0.01
80	->	102	-0.03	0.02
80	->	103	-0.03	0.03
80	->	104	-0.01	0.00
80	->	105	-0.00	0.00
80	->	106	-0.00	0.00
81	->	1	0.02	0.01
81	->	2	0.00	0.00
81	->	3	-0.00	0.00
81	->	4	0.00	0.00
81	->	5	-0.00	0.00
81	->	6	0.00	0.00
81	->	7	0.00	0.00
81	->	8	-0.00	0.00
81	->	9	-0.00	0.00
81	->	10	-0.01	0.00
81	->	11	-0.01	0.00
81	->	12	0.01	0.01
81	->	13	0.00	0.00
81	->	14	0.00	0.00
81	->	15	0.00	0.00
81	->	16	0.01	0.00

81	->	17	-0.00	0.00
81	->	18	-0.00	0.00
81	->	19	-0.01	0.00
81	->	20	-0.00	0.00
81	->	21	0.00	0.00
81	->	22	0.01	0.00
81	->	23	0.00	0.00
81	->	24	0.00	0.00
81	->	25	0.01	0.00
81	->	26	0.00	0.00
81	->	27	0.00	0.00
81	->	28	0.00	0.00
81	->	29	0.00	0.00
81	->	30	-0.00	0.00
81	->	31	0.01	0.00
81	->	32	0.00	0.00
81	->	33	-0.00	0.00
81	->	34	0.00	0.00
81	->	35	-0.01	0.00
81	->	36	0.00	0.00
81	->	37	-0.00	0.00
81	->	38	-0.01	0.00
81	->	39	0.00	0.00
81	->	40	0.01	0.00
81	->	41	-0.00	0.00
81	->	42	0.00	0.00
81	->	43	0.00	0.00
81	->	44	0.02	0.00
81	->	45	-0.00	0.00
81	->	46	-0.02	0.00
81	->	47	0.00	0.00
81	->	48	-0.00	0.00
81	->	49	0.00	0.00
81	->	50	0.00	0.00
81	->	51	0.00	0.00
81	->	52	0.00	0.00
81	->	53	0.02	0.00
81	->	54	0.00	0.00
81	->	55	0.00	0.00
81	->	56	0.00	0.00
81	->	57	0.00	0.00
81	->	58	-0.01	0.00
81	->	59	0.02	0.00
81	->	60	-0.00	0.00
81	->	61	-0.00	0.00
81	->	62	-0.01	0.00
81	->	63	-0.02	0.01
81	->	64	-0.02	0.01
81	->	65	-0.01	0.01
81	->	66	-0.03	0.00

81 ->	67	-0.00	0.00
81 ->	68	0.02	0.01
81 ->	69	0.01	0.00
81 ->	70	-0.01	0.01
81 ->	71	0.00	0.00
81 ->	72	0.04	0.01
81 ->	73	-0.01	0.00
81 ->	74	-0.13	0.06
81 ->	75	-0.09	0.02
81 ->	76	-0.00	0.06
81 ->	77	-1.42	0.71
81 ->	78	-2.21	0.49
81 ->	79	-1.17	0.27
81 ->	80	-15.27	0.46
81 ->	81	5.84	2.86
81 ->	82	-11.52	0.63
81 ->	83	-0.55	0.39
81 ->	84	-1.24	0.74
81 ->	85	-0.07	0.08
81 ->	86	-0.07	0.03
81 ->	87	-0.02	0.00
81 ->	88	-0.02	0.01
81 ->	89	-0.02	0.00
81 ->	90	-0.01	0.00
81 ->	91	-0.01	0.00
81 ->	92	-0.02	0.00
81 ->	93	-0.02	0.00
81 ->	94	-0.01	0.00
81 ->	95	-0.00	0.00
81 ->	96	-0.00	0.00
81 ->	97	-0.00	0.00
81 ->	98	-0.01	0.00
81 ->	99	-0.01	0.00
81 ->	100	-0.02	0.01
81 ->	101	-0.05	0.02
81 ->	102	-0.31	0.41
81 ->	103	-1.74	2.79
81 ->	104	-0.44	0.98
81 ->	105	-0.08	0.03
81 ->	106	-0.02	0.01
82 ->	1	0.00	0.00
82 ->	2	0.00	0.00
82 ->	3	0.00	0.00
82 ->	4	0.00	0.00
82 ->	5	-0.00	0.00
82 ->	6	0.00	0.00
82 ->	7	0.00	0.00
82 ->	8	-0.00	0.00
82 ->	9	0.00	0.00
82 ->	10	-0.00	0.00

82	->	11	-0.00	0.00
82	->	12	0.00	0.00
82	->	13	0.00	0.00
82	->	14	0.00	0.00
82	->	15	0.00	0.00
82	->	16	0.00	0.00
82	->	17	-0.00	0.00
82	->	18	0.00	0.00
82	->	19	-0.00	0.00
82	->	20	0.00	0.00
82	->	21	0.00	0.00
82	->	22	0.00	0.00
82	->	23	0.00	0.00
82	->	24	0.00	0.00
82	->	25	0.00	0.00
82	->	26	0.00	0.00
82	->	27	0.00	0.00
82	->	28	0.00	0.00
82	->	29	0.00	0.00
82	->	30	-0.00	0.00
82	->	31	0.00	0.00
82	->	32	0.00	0.00
82	->	33	0.00	0.00
82	->	34	0.00	0.00
82	->	35	-0.00	0.00
82	->	36	0.00	0.00
82	->	37	-0.00	0.00
82	->	38	-0.00	0.00
82	->	39	0.00	0.00
82	->	40	0.00	0.00
82	->	41	-0.00	0.00
82	->	42	0.00	0.00
82	->	43	0.00	0.00
82	->	44	0.00	0.00
82	->	45	-0.00	0.00
82	->	46	-0.00	0.00
82	->	47	-0.00	0.00
82	->	48	-0.00	0.00
82	->	49	-0.00	0.00
82	->	50	0.00	0.00
82	->	51	0.00	0.00
82	->	52	0.00	0.00
82	->	53	0.00	0.00
82	->	54	-0.00	0.00
82	->	55	0.00	0.00
82	->	56	0.00	0.00
82	->	57	0.00	0.00
82	->	58	-0.00	0.00
82	->	59	0.00	0.00
82	->	60	-0.00	0.00

82	->	61	-0.00	0.00
82	->	62	-0.00	0.00
82	->	63	-0.05	0.02
82	->	64	-0.01	0.00
82	->	65	-0.01	0.01
82	->	66	-0.04	0.02
82	->	67	-0.00	0.00
82	->	68	-0.07	0.11
82	->	69	-0.00	0.00
82	->	70	-0.03	0.01
82	->	71	-0.00	0.00
82	->	72	-0.00	0.00
82	->	73	-0.00	0.00
82	->	74	-0.01	0.00
82	->	75	-0.04	0.01
82	->	76	-0.04	0.02
82	->	77	-0.05	0.03
82	->	78	-1.48	0.96
82	->	79	-3.86	0.98
82	->	80	-0.75	0.25
82	->	81	-11.62	0.63
82	->	82	16.87	1.24
82	->	83	-22.08	0.84
82	->	84	-0.89	0.26
82	->	85	-0.65	0.81
82	->	86	-0.05	0.06
82	->	87	-0.01	0.00
82	->	88	-0.00	0.00
82	->	89	-0.00	0.00
82	->	90	-0.00	0.00
82	->	91	-0.00	0.00
82	->	92	-0.00	0.00
82	->	93	-0.00	0.00
82	->	94	-0.00	0.00
82	->	95	-0.00	0.00
82	->	96	-0.00	0.00
82	->	97	-0.00	0.00
82	->	98	-0.00	0.00
82	->	99	-0.00	0.00
82	->	100	-0.00	0.00
82	->	101	-0.00	0.00
82	->	102	-0.00	0.00
82	->	103	-0.01	0.00
82	->	104	-0.01	0.00
82	->	105	-0.01	0.00
82	->	106	-0.00	0.00
83	->	1	0.00	0.00
83	->	2	0.00	0.00
83	->	3	0.00	0.00
83	->	4	0.00	0.00

83	->	5	-0.00	0.00
83	->	6	0.00	0.00
83	->	7	0.00	0.00
83	->	8	0.00	0.00
83	->	9	0.00	0.00
83	->	10	-0.00	0.00
83	->	11	-0.00	0.00
83	->	12	0.00	0.00
83	->	13	0.00	0.00
83	->	14	0.00	0.00
83	->	15	0.00	0.00
83	->	16	0.00	0.00
83	->	17	-0.00	0.00
83	->	18	0.00	0.00
83	->	19	-0.00	0.00
83	->	20	-0.00	0.00
83	->	21	0.00	0.00
83	->	22	0.00	0.00
83	->	23	0.00	0.00
83	->	24	0.00	0.00
83	->	25	0.00	0.00
83	->	26	0.00	0.00
83	->	27	0.00	0.00
83	->	28	0.00	0.00
83	->	29	0.00	0.00
83	->	30	0.00	0.00
83	->	31	0.00	0.00
83	->	32	0.00	0.00
83	->	33	0.00	0.00
83	->	34	-0.00	0.00
83	->	35	0.00	0.00
83	->	36	-0.00	0.00
83	->	37	-0.00	0.00
83	->	38	0.00	0.00
83	->	39	0.00	0.00
83	->	40	0.00	0.00
83	->	41	0.00	0.00
83	->	42	0.00	0.00
83	->	43	0.00	0.00
83	->	44	0.00	0.00
83	->	45	0.00	0.00
83	->	46	0.00	0.00
83	->	47	0.00	0.00
83	->	48	0.00	0.00
83	->	49	0.00	0.00
83	->	50	0.00	0.00
83	->	51	0.00	0.00
83	->	52	0.00	0.00
83	->	53	-0.00	0.00
83	->	54	0.00	0.00

83	->	55	-0.00	0.00
83	->	56	0.00	0.00
83	->	57	0.00	0.00
83	->	58	0.00	0.00
83	->	59	-0.00	0.00
83	->	60	-0.00	0.00
83	->	61	-0.01	0.00
83	->	62	-0.04	0.01
83	->	63	1.84	0.88
83	->	64	-0.04	0.09
83	->	65	-1.29	1.64
83	->	66	1.57	0.69
83	->	67	-0.02	0.04
83	->	68	-0.16	0.12
83	->	69	-0.01	0.01
83	->	70	-0.02	0.01
83	->	71	-0.00	0.00
83	->	72	-0.00	0.00
83	->	73	-0.00	0.00
83	->	74	-0.01	0.00
83	->	75	-0.02	0.00
83	->	76	-0.08	0.03
83	->	77	-0.04	0.02
83	->	78	-0.12	0.05
83	->	79	25.17	13.51
83	->	80	-2.80	0.75
83	->	81	-0.56	0.38
83	->	82	-22.19	0.84
83	->	83	3.26	2.14
83	->	84	-5.19	0.45
83	->	85	-0.01	0.01
83	->	86	-0.01	0.01
83	->	87	-0.00	0.00
83	->	88	-0.00	0.00
83	->	89	-0.00	0.00
83	->	90	-0.00	0.00
83	->	91	-0.00	0.00
83	->	92	-0.00	0.00
83	->	93	-0.00	0.00
83	->	94	-0.00	0.00
83	->	95	0.00	0.00
83	->	96	0.00	0.00
83	->	97	0.00	0.00
83	->	98	0.00	0.00
83	->	99	0.00	0.00
83	->	100	-0.00	0.00
83	->	101	-0.00	0.00
83	->	102	-0.00	0.00
83	->	103	-0.01	0.00
83	->	104	-0.00	0.00

83	->	105	-0.00	0.00
83	->	106	-0.00	0.00
84	->	1	-0.00	0.00
84	->	2	-0.00	0.00
84	->	3	0.00	0.00
84	->	4	0.00	0.00
84	->	5	0.00	0.00
84	->	6	-0.00	0.00
84	->	7	-0.00	0.00
84	->	8	0.00	0.00
84	->	9	0.00	0.00
84	->	10	0.00	0.00
84	->	11	0.00	0.00
84	->	12	-0.00	0.00
84	->	13	-0.00	0.00
84	->	14	-0.00	0.00
84	->	15	-0.00	0.00
84	->	16	-0.00	0.00
84	->	17	0.00	0.00
84	->	18	0.00	0.00
84	->	19	0.00	0.00
84	->	20	0.00	0.00
84	->	21	-0.00	0.00
84	->	22	-0.00	0.00
84	->	23	-0.00	0.00
84	->	24	-0.00	0.00
84	->	25	-0.00	0.00
84	->	26	-0.00	0.00
84	->	27	0.00	0.00
84	->	28	-0.00	0.00
84	->	29	-0.00	0.00
84	->	30	0.00	0.00
84	->	31	-0.00	0.00
84	->	32	-0.00	0.00
84	->	33	-0.00	0.00
84	->	34	-0.00	0.00
84	->	35	0.00	0.00
84	->	36	-0.00	0.00
84	->	37	0.00	0.00
84	->	38	0.00	0.00
84	->	39	-0.00	0.00
84	->	40	-0.00	0.00
84	->	41	0.00	0.00
84	->	42	-0.00	0.00
84	->	43	-0.00	0.00
84	->	44	-0.00	0.00
84	->	45	0.00	0.00
84	->	46	0.00	0.00
84	->	47	0.00	0.00
84	->	48	0.00	0.00

84	->	49	-0.00	0.00
84	->	50	-0.00	0.00
84	->	51	-0.00	0.00
84	->	52	0.00	0.00
84	->	53	-0.01	0.00
84	->	54	-0.00	0.00
84	->	55	-0.00	0.00
84	->	56	-0.00	0.00
84	->	57	0.00	0.00
84	->	58	0.01	0.00
84	->	59	-0.01	0.00
84	->	60	0.00	0.00
84	->	61	-0.00	0.00
84	->	62	-0.00	0.00
84	->	63	0.06	0.01
84	->	64	0.03	0.01
84	->	65	-0.18	0.43
84	->	66	0.05	0.01
84	->	67	-0.00	0.00
84	->	68	-0.03	0.01
84	->	69	-0.01	0.00
84	->	70	-0.00	0.00
84	->	71	-0.00	0.00
84	->	72	-0.01	0.00
84	->	73	0.00	0.00
84	->	74	0.02	0.00
84	->	75	0.02	0.00
84	->	76	-0.05	0.02
84	->	77	-0.06	0.02
84	->	78	-0.07	0.02
84	->	79	-0.04	0.04
84	->	80	-1.11	1.01
84	->	81	-1.26	0.75
84	->	82	-0.90	0.26
84	->	83	-5.29	0.45
84	->	84	-90.21	1.86
84	->	85	0.01	0.00
84	->	86	0.02	0.01
84	->	87	0.01	0.00
84	->	88	0.01	0.00
84	->	89	0.00	0.00
84	->	90	0.00	0.00
84	->	91	0.00	0.00
84	->	92	0.00	0.00
84	->	93	0.01	0.00
84	->	94	0.00	0.00
84	->	95	0.00	0.00
84	->	96	0.00	0.00
84	->	97	0.00	0.00
84	->	98	0.00	0.00

84	->	99	0.00	0.00
84	->	100	0.01	0.00
84	->	101	0.01	0.00
84	->	102	0.02	0.00
84	->	103	0.02	0.01
84	->	104	0.01	0.00
84	->	105	0.01	0.00
84	->	106	0.00	0.00
85	->	1	-0.00	0.00
85	->	2	0.00	0.00
85	->	3	0.00	0.00
85	->	4	0.00	0.00
85	->	5	0.00	0.00
85	->	6	-0.00	0.00
85	->	7	0.00	0.00
85	->	8	0.00	0.00
85	->	9	0.00	0.00
85	->	10	0.00	0.00
85	->	11	0.00	0.00
85	->	12	-0.00	0.00
85	->	13	-0.00	0.00
85	->	14	-0.00	0.00
85	->	15	-0.00	0.00
85	->	16	-0.00	0.00
85	->	17	0.00	0.00
85	->	18	0.00	0.00
85	->	19	0.00	0.00
85	->	20	0.00	0.00
85	->	21	0.00	0.00
85	->	22	-0.00	0.00
85	->	23	-0.00	0.00
85	->	24	0.00	0.00
85	->	25	-0.00	0.00
85	->	26	-0.00	0.00
85	->	27	-0.00	0.00
85	->	28	0.00	0.00
85	->	29	0.00	0.00
85	->	30	0.00	0.00
85	->	31	-0.00	0.00
85	->	32	0.00	0.00
85	->	33	0.00	0.00
85	->	34	-0.00	0.00
85	->	35	0.00	0.00
85	->	36	-0.00	0.00
85	->	37	-0.00	0.00
85	->	38	0.00	0.00
85	->	39	-0.00	0.00
85	->	40	-0.01	0.00
85	->	41	0.00	0.00
85	->	42	-0.00	0.00

85	->	43	-0.00	0.00
85	->	44	-0.00	0.00
85	->	45	0.00	0.00
85	->	46	0.00	0.00
85	->	47	-0.00	0.00
85	->	48	-0.00	0.00
85	->	49	-0.00	0.00
85	->	50	-0.00	0.00
85	->	51	-0.00	0.00
85	->	52	-0.00	0.00
85	->	53	-0.01	0.00
85	->	54	-0.00	0.00
85	->	55	-0.00	0.00
85	->	56	-0.00	0.00
85	->	57	-0.00	0.00
85	->	58	0.00	0.00
85	->	59	-0.08	0.09
85	->	60	-0.00	0.00
85	->	61	-0.00	0.00
85	->	62	-0.00	0.00
85	->	63	0.01	0.01
85	->	64	0.01	0.00
85	->	65	-0.00	0.00
85	->	66	0.02	0.01
85	->	67	-0.00	0.01
85	->	68	-1.70	1.79
85	->	69	-0.03	0.02
85	->	70	-0.05	0.06
85	->	71	-0.00	0.00
85	->	72	-0.03	0.01
85	->	73	-0.00	0.00
85	->	74	-0.00	0.01
85	->	75	-0.05	0.06
85	->	76	-0.02	0.01
85	->	77	-0.03	0.02
85	->	78	-1.31	1.45
85	->	79	-0.21	0.23
85	->	80	-0.01	0.01
85	->	81	-0.07	0.08
85	->	82	-0.64	0.80
85	->	83	-0.01	0.01
85	->	84	0.01	0.00
85	->	85	-85.88	2.97
85	->	86	-35.54	1.04
85	->	87	-0.25	0.06
85	->	88	-0.03	0.01
85	->	89	-0.00	0.00
85	->	90	0.00	0.00
85	->	91	0.00	0.00
85	->	92	0.00	0.00

85	->	93	0.00	0.00
85	->	94	0.00	0.00
85	->	95	0.00	0.00
85	->	96	0.00	0.00
85	->	97	0.00	0.00
85	->	98	0.00	0.00
85	->	99	0.00	0.00
85	->	100	0.00	0.00
85	->	101	0.00	0.00
85	->	102	0.00	0.00
85	->	103	-0.00	0.00
85	->	104	-0.03	0.01
85	->	105	-0.21	0.07
85	->	106	-2.13	1.57
86	->	1	-0.01	0.00
86	->	2	-0.00	0.00
86	->	3	0.00	0.00
86	->	4	0.00	0.00
86	->	5	0.00	0.00
86	->	6	-0.00	0.00
86	->	7	-0.00	0.00
86	->	8	0.00	0.00
86	->	9	0.00	0.00
86	->	10	0.00	0.00
86	->	11	0.00	0.00
86	->	12	-0.00	0.00
86	->	13	-0.00	0.00
86	->	14	-0.00	0.00
86	->	15	-0.00	0.00
86	->	16	-0.00	0.00
86	->	17	0.00	0.00
86	->	18	0.00	0.00
86	->	19	0.01	0.00
86	->	20	0.00	0.00
86	->	21	0.00	0.00
86	->	22	-0.01	0.00
86	->	23	-0.00	0.00
86	->	24	0.00	0.00
86	->	25	-0.01	0.00
86	->	26	-0.00	0.00
86	->	27	-0.00	0.00
86	->	28	-0.00	0.00
86	->	29	-0.00	0.00
86	->	30	0.00	0.00
86	->	31	-0.01	0.00
86	->	32	-0.00	0.00
86	->	33	-0.00	0.00
86	->	34	-0.00	0.00
86	->	35	0.02	0.00
86	->	36	-0.01	0.00

86	->	37	-0.00	0.00
86	->	38	0.02	0.00
86	->	39	-0.00	0.00
86	->	40	-0.03	0.01
86	->	41	-0.00	0.00
86	->	42	-0.00	0.00
86	->	43	-0.00	0.00
86	->	44	-0.02	0.00
86	->	45	0.00	0.00
86	->	46	0.02	0.00
86	->	47	-0.01	0.00
86	->	48	-0.00	0.00
86	->	49	-0.01	0.01
86	->	50	-0.01	0.02
86	->	51	-0.03	0.01
86	->	52	-0.00	0.00
86	->	53	-0.13	0.08
86	->	54	-0.06	0.10
86	->	55	0.01	0.01
86	->	56	-0.01	0.00
86	->	57	-0.01	0.01
86	->	58	0.03	0.01
86	->	59	-0.87	0.86
86	->	60	-0.00	0.00
86	->	61	-0.01	0.00
86	->	62	-0.01	0.00
86	->	63	0.05	0.01
86	->	64	0.02	0.00
86	->	65	-0.00	0.00
86	->	66	0.06	0.02
86	->	67	-0.00	0.00
86	->	68	-4.55	3.06
86	->	69	-0.14	0.07
86	->	70	-0.28	0.16
86	->	71	-0.13	0.11
86	->	72	-0.34	0.26
86	->	73	0.00	0.01
86	->	74	-0.00	0.03
86	->	75	-0.74	0.53
86	->	76	-0.05	0.02
86	->	77	-0.06	0.02
86	->	78	-3.98	2.06
86	->	79	-0.13	0.17
86	->	80	-0.01	0.00
86	->	81	-0.07	0.03
86	->	82	-0.05	0.06
86	->	83	-0.01	0.01
86	->	84	0.02	0.01
86	->	85	-35.38	1.03
86	->	86	-173.15	4.03

86	->	87	-35.98	0.91
86	->	88	-0.26	0.10
86	->	89	-0.02	0.01
86	->	90	0.01	0.00
86	->	91	0.01	0.00
86	->	92	0.00	0.00
86	->	93	0.00	0.00
86	->	94	0.00	0.00
86	->	95	0.00	0.00
86	->	96	0.00	0.00
86	->	97	0.00	0.00
86	->	98	0.01	0.00
86	->	99	0.01	0.00
86	->	100	0.01	0.00
86	->	101	0.01	0.00
86	->	102	0.01	0.00
86	->	103	-0.02	0.02
86	->	104	-0.13	0.06
86	->	105	-1.23	0.74
86	->	106	-10.67	0.74
87	->	1	-0.01	0.00
87	->	2	-0.00	0.00
87	->	3	0.00	0.00
87	->	4	0.00	0.00
87	->	5	0.00	0.00
87	->	6	-0.00	0.00
87	->	7	-0.00	0.00
87	->	8	0.00	0.00
87	->	9	-0.00	0.00
87	->	10	0.01	0.00
87	->	11	0.00	0.00
87	->	12	-0.01	0.00
87	->	13	-0.00	0.00
87	->	14	-0.00	0.00
87	->	15	-0.00	0.00
87	->	16	-0.01	0.00
87	->	17	0.00	0.00
87	->	18	0.00	0.00
87	->	19	0.01	0.00
87	->	20	0.00	0.00
87	->	21	0.00	0.00
87	->	22	-0.02	0.00
87	->	23	-0.00	0.00
87	->	24	0.00	0.00
87	->	25	-0.01	0.00
87	->	26	-0.00	0.00
87	->	27	-0.00	0.00
87	->	28	0.00	0.00
87	->	29	-0.00	0.00
87	->	30	0.00	0.00

87 ->	31	-0.04	0.01
87 ->	32	-0.00	0.00
87 ->	33	-0.00	0.00
87 ->	34	-0.01	0.00
87 ->	35	0.08	0.02
87 ->	36	-0.03	0.01
87 ->	37	-0.00	0.01
87 ->	38	0.10	0.04
87 ->	39	-0.00	0.00
87 ->	40	-0.35	0.32
87 ->	41	-0.01	0.01
87 ->	42	0.01	0.02
87 ->	43	-0.00	0.00
87 ->	44	-0.04	0.01
87 ->	45	-0.00	0.00
87 ->	46	0.02	0.01
87 ->	47	-0.06	0.02
87 ->	48	0.01	0.01
87 ->	49	-0.04	0.02
87 ->	50	-0.15	0.10
87 ->	51	-0.87	0.35
87 ->	52	0.01	0.02
87 ->	53	-0.37	0.19
87 ->	54	-1.94	1.02
87 ->	55	0.05	0.03
87 ->	56	-0.02	0.01
87 ->	57	-0.04	0.04
87 ->	58	0.04	0.02
87 ->	59	-1.76	2.36
87 ->	60	-0.01	0.00
87 ->	61	-0.01	0.00
87 ->	62	-0.00	0.00
87 ->	63	0.02	0.00
87 ->	64	0.01	0.00
87 ->	65	-0.00	0.00
87 ->	66	0.02	0.00
87 ->	67	-0.00	0.00
87 ->	68	-0.11	0.09
87 ->	69	-0.08	0.04
87 ->	70	-0.19	0.11
87 ->	71	-0.36	0.26
87 ->	72	-2.60	0.83
87 ->	73	-0.01	0.00
87 ->	74	-0.47	0.49
87 ->	75	-0.83	0.96
87 ->	76	-0.04	0.01
87 ->	77	-0.02	0.01
87 ->	78	-0.01	0.62
87 ->	79	-0.02	0.01
87 ->	80	-0.00	0.00

87	->	81	-0.02	0.00
87	->	82	-0.01	0.00
87	->	83	-0.00	0.00
87	->	84	0.01	0.00
87	->	85	-0.25	0.06
87	->	86	-35.77	0.88
87	->	87	-208.36	4.21
87	->	88	-32.47	1.48
87	->	89	-0.30	0.09
87	->	90	0.01	0.01
87	->	91	0.01	0.00
87	->	92	0.01	0.00
87	->	93	0.00	0.00
87	->	94	0.00	0.00
87	->	95	0.00	0.00
87	->	96	0.00	0.00
87	->	97	0.01	0.00
87	->	98	0.01	0.00
87	->	99	0.02	0.00
87	->	100	0.01	0.00
87	->	101	-0.00	0.00
87	->	102	-0.02	0.01
87	->	103	-0.13	0.06
87	->	104	-0.67	0.30
87	->	105	-11.21	0.86
87	->	106	0.20	0.14
88	->	1	-0.01	0.00
88	->	2	-0.00	0.00
88	->	3	0.00	0.00
88	->	4	0.00	0.00
88	->	5	0.01	0.00
88	->	6	-0.00	0.00
88	->	7	0.00	0.00
88	->	8	0.00	0.00
88	->	9	-0.00	0.00
88	->	10	0.01	0.00
88	->	11	0.00	0.00
88	->	12	-0.01	0.00
88	->	13	-0.00	0.00
88	->	14	-0.00	0.00
88	->	15	-0.00	0.00
88	->	16	-0.01	0.00
88	->	17	0.00	0.00
88	->	18	0.00	0.00
88	->	19	0.01	0.00
88	->	20	-0.00	0.00
88	->	21	-0.00	0.00
88	->	22	-0.03	0.01
88	->	23	-0.00	0.00
88	->	24	-0.00	0.00

88 ->	25	-0.04	0.01
88 ->	26	-0.01	0.00
88 ->	27	-0.00	0.00
88 ->	28	-0.00	0.00
88 ->	29	-0.00	0.00
88 ->	30	-0.00	0.00
88 ->	31	-0.50	0.43
88 ->	32	-0.00	0.00
88 ->	33	-0.01	0.00
88 ->	34	-0.02	0.01
88 ->	35	0.13	0.04
88 ->	36	-0.04	0.01
88 ->	37	-0.00	0.01
88 ->	38	0.18	0.09
88 ->	39	-0.01	0.01
88 ->	40	-4.86	3.15
88 ->	41	-0.06	0.02
88 ->	42	-0.80	0.48
88 ->	43	0.02	0.03
88 ->	44	-0.09	0.02
88 ->	45	-0.00	0.00
88 ->	46	0.02	0.01
88 ->	47	-0.36	0.09
88 ->	48	0.00	0.03
88 ->	49	-0.07	0.04
88 ->	50	-0.37	0.20
88 ->	51	-5.14	2.07
88 ->	52	0.07	0.04
88 ->	53	-0.14	0.06
88 ->	54	-0.30	0.44
88 ->	55	0.05	0.04
88 ->	56	-0.01	0.00
88 ->	57	-0.01	0.00
88 ->	58	0.03	0.01
88 ->	59	-0.06	0.02
88 ->	60	-0.00	0.00
88 ->	61	-0.00	0.00
88 ->	62	-0.00	0.00
88 ->	63	0.01	0.00
88 ->	64	0.01	0.00
88 ->	65	-0.00	0.00
88 ->	66	0.01	0.00
88 ->	67	0.00	0.00
88 ->	68	-0.03	0.01
88 ->	69	-0.02	0.00
88 ->	70	-0.02	0.01
88 ->	71	-0.04	0.01
88 ->	72	-9.37	0.91
88 ->	73	-0.01	0.00
88 ->	74	0.58	0.18

88	->	75	0.07	0.06
88	->	76	-0.02	0.00
88	->	77	-0.03	0.01
88	->	78	-0.21	0.14
88	->	79	-0.01	0.00
88	->	80	-0.00	0.00
88	->	81	-0.02	0.01
88	->	82	-0.00	0.00
88	->	83	-0.00	0.00
88	->	84	0.01	0.00
88	->	85	-0.03	0.01
88	->	86	-0.26	0.10
88	->	87	-32.29	1.46
88	->	88	-165.06	3.66
88	->	89	-34.48	0.76
88	->	90	-0.23	0.06
88	->	91	-0.01	0.01
88	->	92	0.01	0.00
88	->	93	0.01	0.00
88	->	94	0.01	0.00
88	->	95	0.00	0.00
88	->	96	0.00	0.00
88	->	97	0.01	0.00
88	->	98	0.02	0.00
88	->	99	0.02	0.00
88	->	100	0.01	0.00
88	->	101	-0.01	0.01
88	->	102	-0.10	0.04
88	->	103	-2.09	0.69
88	->	104	-11.50	0.71
88	->	105	-4.65	1.13
88	->	106	-0.25	0.07
89	->	1	-0.02	0.00
89	->	2	0.00	0.00
89	->	3	-0.00	0.00
89	->	4	-0.00	0.00
89	->	5	0.02	0.00
89	->	6	-0.00	0.00
89	->	7	-0.00	0.00
89	->	8	0.01	0.00
89	->	9	-0.00	0.00
89	->	10	0.03	0.00
89	->	11	0.01	0.00
89	->	12	-0.03	0.01
89	->	13	-0.01	0.00
89	->	14	-0.00	0.00
89	->	15	-0.00	0.00
89	->	16	-0.02	0.00
89	->	17	0.00	0.00
89	->	18	-0.00	0.00

89	->	19	0.03	0.00
89	->	20	-0.00	0.00
89	->	21	-0.01	0.01
89	->	22	-0.16	0.07
89	->	23	-0.02	0.01
89	->	24	-0.01	0.01
89	->	25	-0.20	0.09
89	->	26	-0.21	0.07
89	->	27	0.02	0.01
89	->	28	-0.01	0.00
89	->	29	-0.03	0.01
89	->	30	-0.01	0.01
89	->	31	-3.34	2.92
89	->	32	-0.01	0.00
89	->	33	-0.01	0.01
89	->	34	-0.02	0.01
89	->	35	0.05	0.01
89	->	36	-0.02	0.00
89	->	37	-0.00	0.00
89	->	38	0.05	0.01
89	->	39	-0.01	0.00
89	->	40	-4.65	3.34
89	->	41	-0.14	0.12
89	->	42	-0.93	0.35
89	->	43	-0.84	0.51
89	->	44	-0.48	0.26
89	->	45	0.00	0.01
89	->	46	-0.06	0.10
89	->	47	-3.93	0.61
89	->	48	-0.03	0.03
89	->	49	-0.02	0.01
89	->	50	-0.37	0.20
89	->	51	-0.08	0.02
89	->	52	-0.01	0.00
89	->	53	-0.04	0.00
89	->	54	-0.01	0.00
89	->	55	-0.00	0.00
89	->	56	-0.00	0.00
89	->	57	-0.00	0.00
89	->	58	0.01	0.00
89	->	59	-0.02	0.00
89	->	60	-0.00	0.00
89	->	61	-0.00	0.00
89	->	62	0.00	0.00
89	->	63	0.01	0.00
89	->	64	0.00	0.00
89	->	65	-0.00	0.00
89	->	66	0.01	0.00
89	->	67	0.00	0.00
89	->	68	-0.01	0.00

89	->	69	-0.01	0.00
89	->	70	-0.01	0.00
89	->	71	-0.01	0.00
89	->	72	-2.33	0.61
89	->	73	-0.01	0.00
89	->	74	0.26	0.14
89	->	75	0.01	0.01
89	->	76	-0.02	0.00
89	->	77	-0.02	0.00
89	->	78	-0.04	0.01
89	->	79	-0.00	0.00
89	->	80	-0.00	0.00
89	->	81	-0.02	0.00
89	->	82	-0.00	0.00
89	->	83	-0.00	0.00
89	->	84	0.00	0.00
89	->	85	-0.00	0.00
89	->	86	-0.02	0.01
89	->	87	-0.30	0.09
89	->	88	-34.31	0.76
89	->	89	-84.92	4.24
89	->	90	-33.19	1.11
89	->	91	-0.14	0.07
89	->	92	0.00	0.01
89	->	93	0.01	0.00
89	->	94	0.01	0.00
89	->	95	0.00	0.00
89	->	96	0.00	0.00
89	->	97	0.01	0.00
89	->	98	0.01	0.00
89	->	99	0.01	0.01
89	->	100	-0.01	0.01
89	->	101	-0.08	0.03
89	->	102	-0.71	0.30
89	->	103	-6.09	0.52
89	->	104	-0.59	0.32
89	->	105	-0.19	0.09
89	->	106	-0.02	0.02
90	->	1	-0.06	0.03
90	->	2	-0.00	0.00
90	->	3	-0.00	0.00
90	->	4	-0.00	0.00
90	->	5	0.06	0.01
90	->	6	-0.00	0.00
90	->	7	-0.01	0.01
90	->	8	0.01	0.00
90	->	9	-0.00	0.00
90	->	10	0.15	0.07
90	->	11	0.02	0.01
90	->	12	-0.19	0.13

90 ->	13	-0.02	0.01
90 ->	14	-0.01	0.02
90 ->	15	-0.00	0.00
90 ->	16	-0.04	0.01
90 ->	17	0.00	0.00
90 ->	18	-0.01	0.01
90 ->	19	0.03	0.04
90 ->	20	-0.00	0.01
90 ->	21	-0.03	0.02
90 ->	22	-1.52	1.32
90 ->	23	-0.91	0.23
90 ->	24	-0.01	0.03
90 ->	25	-0.56	0.29
90 ->	26	-3.27	0.51
90 ->	27	0.05	0.04
90 ->	28	-0.02	0.01
90 ->	29	-0.06	0.03
90 ->	30	0.01	0.02
90 ->	31	-0.69	0.78
90 ->	32	-0.02	0.01
90 ->	33	-0.01	0.00
90 ->	34	-0.00	0.00
90 ->	35	0.03	0.00
90 ->	36	-0.02	0.00
90 ->	37	0.00	0.00
90 ->	38	0.03	0.00
90 ->	39	-0.00	0.00
90 ->	40	-0.08	0.02
90 ->	41	-0.03	0.02
90 ->	42	-0.22	0.09
90 ->	43	-3.65	1.97
90 ->	44	-3.27	1.10
90 ->	45	0.01	0.01
90 ->	46	0.20	0.15
90 ->	47	-3.66	0.84
90 ->	48	0.00	0.01
90 ->	49	-0.01	0.00
90 ->	50	-0.01	0.01
90 ->	51	-0.01	0.00
90 ->	52	-0.00	0.00
90 ->	53	-0.03	0.00
90 ->	54	-0.00	0.00
90 ->	55	-0.00	0.00
90 ->	56	-0.00	0.00
90 ->	57	-0.00	0.00
90 ->	58	0.01	0.00
90 ->	59	-0.01	0.00
90 ->	60	-0.00	0.00
90 ->	61	-0.00	0.00
90 ->	62	0.00	0.00

90	->	63	0.01	0.00
90	->	64	0.00	0.00
90	->	65	-0.00	0.00
90	->	66	0.00	0.00
90	->	67	0.00	0.00
90	->	68	-0.01	0.00
90	->	69	-0.01	0.00
90	->	70	-0.00	0.00
90	->	71	-0.00	0.00
90	->	72	-0.12	0.09
90	->	73	-0.00	0.01
90	->	74	-0.01	0.06
90	->	75	0.01	0.00
90	->	76	-0.01	0.00
90	->	77	-0.01	0.00
90	->	78	-0.03	0.01
90	->	79	-0.00	0.00
90	->	80	-0.00	0.00
90	->	81	-0.01	0.00
90	->	82	-0.00	0.00
90	->	83	-0.00	0.00
90	->	84	0.00	0.00
90	->	85	0.00	0.00
90	->	86	0.01	0.00
90	->	87	0.01	0.01
90	->	88	-0.23	0.06
90	->	89	-33.02	1.09
90	->	90	-168.77	4.26
90	->	91	-33.21	0.98
90	->	92	-0.30	0.05
90	->	93	-0.03	0.01
90	->	94	0.01	0.00
90	->	95	0.01	0.00
90	->	96	0.00	0.00
90	->	97	0.02	0.01
90	->	98	0.01	0.00
90	->	99	0.01	0.01
90	->	100	-0.09	0.07
90	->	101	-1.12	0.49
90	->	102	-11.63	0.82
90	->	103	-3.33	0.63
90	->	104	-0.30	0.08
90	->	105	-0.04	0.02
90	->	106	0.01	0.00
91	->	1	-1.23	0.91
91	->	2	-0.00	0.00
91	->	3	-0.01	0.00
91	->	4	-0.01	0.01
91	->	5	0.14	0.03
91	->	6	-0.01	0.00

91	->	7	-0.02	0.01
91	->	8	0.02	0.00
91	->	9	-0.01	0.00
91	->	10	0.37	0.18
91	->	11	0.04	0.01
91	->	12	-3.07	1.82
91	->	13	-0.08	0.04
91	->	14	-0.83	0.71
91	->	15	0.01	0.02
91	->	16	-0.20	0.08
91	->	17	-0.00	0.00
91	->	18	-0.05	0.02
91	->	19	-0.08	0.22
91	->	20	0.04	0.06
91	->	21	-0.08	0.05
91	->	22	-7.59	6.14
91	->	23	-6.60	1.00
91	->	24	0.08	0.04
91	->	25	-0.25	0.09
91	->	26	-0.92	0.34
91	->	27	0.10	0.06
91	->	28	-0.02	0.01
91	->	29	-0.01	0.00
91	->	30	-0.00	0.00
91	->	31	-0.05	0.01
91	->	32	-0.01	0.00
91	->	33	-0.00	0.00
91	->	34	-0.00	0.00
91	->	35	0.01	0.00
91	->	36	-0.01	0.00
91	->	37	0.00	0.00
91	->	38	0.01	0.00
91	->	39	0.00	0.00
91	->	40	-0.03	0.00
91	->	41	-0.00	0.00
91	->	42	-0.02	0.01
91	->	43	-0.05	0.02
91	->	44	-8.94	1.03
91	->	45	-0.00	0.00
91	->	46	0.47	0.14
91	->	47	-0.48	0.28
91	->	48	-0.00	0.00
91	->	49	-0.01	0.00
91	->	50	-0.01	0.00
91	->	51	-0.01	0.00
91	->	52	-0.00	0.00
91	->	53	-0.02	0.00
91	->	54	-0.00	0.00
91	->	55	-0.00	0.00
91	->	56	-0.00	0.00

91	->	57	-0.00	0.00
91	->	58	0.01	0.00
91	->	59	-0.01	0.00
91	->	60	-0.00	0.00
91	->	61	-0.00	0.00
91	->	62	0.00	0.00
91	->	63	0.00	0.00
91	->	64	0.00	0.00
91	->	65	-0.00	0.00
91	->	66	0.00	0.00
91	->	67	0.00	0.00
91	->	68	-0.00	0.00
91	->	69	-0.00	0.00
91	->	70	-0.00	0.00
91	->	71	0.00	0.00
91	->	72	-0.01	0.02
91	->	73	0.00	0.02
91	->	74	-0.04	0.04
91	->	75	0.00	0.00
91	->	76	-0.01	0.00
91	->	77	-0.01	0.01
91	->	78	-0.02	0.00
91	->	79	-0.00	0.00
91	->	80	-0.00	0.00
91	->	81	-0.01	0.00
91	->	82	-0.00	0.00
91	->	83	-0.00	0.00
91	->	84	0.00	0.00
91	->	85	0.00	0.00
91	->	86	0.01	0.00
91	->	87	0.01	0.00
91	->	88	-0.01	0.01
91	->	89	-0.14	0.07
91	->	90	-33.08	0.96
91	->	91	-162.36	5.66
91	->	92	-33.12	1.03
91	->	93	-0.30	0.07
91	->	94	0.00	0.01
91	->	95	0.00	0.00
91	->	96	0.00	0.00
91	->	97	0.01	0.01
91	->	98	-0.00	0.01
91	->	99	0.00	0.03
91	->	100	-0.78	0.25
91	->	101	-11.78	0.73
91	->	102	-3.93	0.70
91	->	103	-0.35	0.07
91	->	104	-0.03	0.02
91	->	105	0.01	0.00
91	->	106	0.01	0.00

92	->	1	-8.47	6.26
92	->	2	-0.01	0.00
92	->	3	-0.00	0.01
92	->	4	-0.01	0.00
92	->	5	0.05	0.01
92	->	6	-0.00	0.00
92	->	7	-0.00	0.00
92	->	8	0.02	0.00
92	->	9	0.00	0.00
92	->	10	0.06	0.01
92	->	11	0.04	0.01
92	->	12	-5.19	3.14
92	->	13	-0.05	0.12
92	->	14	-0.65	0.43
92	->	15	-0.08	0.19
92	->	16	-2.45	0.42
92	->	17	-0.01	0.01
92	->	18	-0.09	0.04
92	->	19	-0.68	0.49
92	->	20	0.02	0.02
92	->	21	-0.02	0.01
92	->	22	-5.45	2.59
92	->	23	-0.06	0.02
92	->	24	-0.00	0.00
92	->	25	-0.06	0.01
92	->	26	-0.02	0.00
92	->	27	-0.00	0.00
92	->	28	-0.00	0.00
92	->	29	-0.00	0.00
92	->	30	0.00	0.00
92	->	31	-0.02	0.00
92	->	32	-0.00	0.00
92	->	33	-0.00	0.00
92	->	34	-0.00	0.00
92	->	35	0.01	0.00
92	->	36	-0.01	0.00
92	->	37	0.00	0.00
92	->	38	0.01	0.00
92	->	39	0.00	0.00
92	->	40	-0.01	0.00
92	->	41	-0.00	0.00
92	->	42	-0.00	0.00
92	->	43	-0.01	0.00
92	->	44	-2.48	1.07
92	->	45	-0.00	0.00
92	->	46	0.25	0.18
92	->	47	-0.05	0.02
92	->	48	-0.00	0.00
92	->	49	-0.00	0.00
92	->	50	-0.01	0.00

92	->	51	-0.00	0.00
92	->	52	-0.00	0.00
92	->	53	-0.01	0.00
92	->	54	-0.00	0.00
92	->	55	-0.00	0.00
92	->	56	-0.00	0.00
92	->	57	-0.00	0.00
92	->	58	0.00	0.00
92	->	59	-0.01	0.00
92	->	60	-0.00	0.00
92	->	61	-0.01	0.00
92	->	62	0.00	0.00
92	->	63	0.00	0.00
92	->	64	0.00	0.00
92	->	65	-0.00	0.00
92	->	66	0.00	0.00
92	->	67	-0.00	0.00
92	->	68	-0.00	0.00
92	->	69	-0.00	0.00
92	->	70	-0.00	0.00
92	->	71	0.00	0.00
92	->	72	0.00	0.02
92	->	73	-0.01	0.02
92	->	74	-0.02	0.02
92	->	75	0.01	0.00
92	->	76	-0.01	0.00
92	->	77	-0.03	0.01
92	->	78	-0.02	0.00
92	->	79	-0.00	0.00
92	->	80	-0.00	0.00
92	->	81	-0.02	0.00
92	->	82	-0.00	0.00
92	->	83	-0.00	0.00
92	->	84	0.00	0.00
92	->	85	0.00	0.00
92	->	86	0.00	0.00
92	->	87	0.01	0.00
92	->	88	0.01	0.00
92	->	89	0.00	0.01
92	->	90	-0.30	0.05
92	->	91	-32.99	1.01
92	->	92	-165.42	5.15
92	->	93	-34.74	0.96
92	->	94	-0.25	0.06
92	->	95	-0.02	0.01
92	->	96	-0.00	0.00
92	->	97	-0.00	0.01
92	->	98	-0.11	0.05
92	->	99	-0.36	0.51
92	->	100	-11.64	0.82

92	->	101	-4.34	0.63
92	->	102	-0.45	0.12
92	->	103	-0.05	0.03
92	->	104	0.01	0.00
92	->	105	0.02	0.00
92	->	106	0.01	0.00
93	->	1	-1.14	1.24
93	->	2	-0.01	0.00
93	->	3	-0.01	0.00
93	->	4	-0.00	0.00
93	->	5	0.02	0.00
93	->	6	-0.00	0.00
93	->	7	-0.00	0.00
93	->	8	0.01	0.00
93	->	9	0.00	0.00
93	->	10	0.02	0.00
93	->	11	0.02	0.00
93	->	12	-0.10	0.06
93	->	13	-0.06	0.03
93	->	14	-0.19	0.15
93	->	15	-1.29	2.14
93	->	16	-11.72	1.56
93	->	17	-0.01	0.01
93	->	18	-0.17	0.17
93	->	19	-1.18	1.38
93	->	20	-0.01	0.01
93	->	21	-0.01	0.00
93	->	22	-0.25	0.39
93	->	23	-0.01	0.00
93	->	24	-0.00	0.00
93	->	25	-0.02	0.00
93	->	26	-0.00	0.00
93	->	27	-0.00	0.00
93	->	28	-0.00	0.00
93	->	29	-0.00	0.00
93	->	30	-0.00	0.00
93	->	31	-0.01	0.00
93	->	32	-0.00	0.00
93	->	33	-0.00	0.00
93	->	34	0.00	0.00
93	->	35	0.00	0.00
93	->	36	-0.00	0.00
93	->	37	0.00	0.00
93	->	38	0.00	0.00
93	->	39	-0.00	0.00
93	->	40	-0.01	0.00
93	->	41	-0.00	0.00
93	->	42	-0.00	0.00
93	->	43	-0.00	0.00
93	->	44	-0.09	0.06

93	->	45	-0.01	0.00
93	->	46	-0.01	0.03
93	->	47	-0.01	0.00
93	->	48	-0.00	0.00
93	->	49	-0.00	0.00
93	->	50	-0.00	0.00
93	->	51	-0.00	0.00
93	->	52	-0.00	0.00
93	->	53	-0.02	0.00
93	->	54	-0.00	0.00
93	->	55	-0.00	0.00
93	->	56	-0.00	0.00
93	->	57	-0.00	0.00
93	->	58	0.01	0.00
93	->	59	-0.01	0.00
93	->	60	-0.00	0.00
93	->	61	-0.00	0.00
93	->	62	0.00	0.00
93	->	63	0.01	0.00
93	->	64	0.01	0.00
93	->	65	-0.00	0.00
93	->	66	0.00	0.00
93	->	67	0.00	0.00
93	->	68	-0.00	0.00
93	->	69	-0.01	0.00
93	->	70	-0.00	0.00
93	->	71	-0.00	0.00
93	->	72	-0.02	0.00
93	->	73	-0.01	0.00
93	->	74	0.02	0.00
93	->	75	0.01	0.00
93	->	76	-0.02	0.01
93	->	77	-0.04	0.01
93	->	78	-0.01	0.00
93	->	79	-0.00	0.00
93	->	80	-0.00	0.00
93	->	81	-0.02	0.00
93	->	82	-0.00	0.00
93	->	83	-0.00	0.00
93	->	84	0.01	0.00
93	->	85	0.00	0.00
93	->	86	0.00	0.00
93	->	87	0.00	0.00
93	->	88	0.01	0.00
93	->	89	0.01	0.00
93	->	90	-0.03	0.01
93	->	91	-0.30	0.07
93	->	92	-34.60	0.95
93	->	93	-207.94	3.05
93	->	94	-33.75	1.04

93	->	95	-0.26	0.06
93	->	96	-0.01	0.01
93	->	97	-0.05	0.04
93	->	98	-0.28	0.28
93	->	99	-11.25	0.77
93	->	100	0.24	0.19
93	->	101	-0.07	0.06
93	->	102	-0.02	0.02
93	->	103	0.01	0.00
93	->	104	0.01	0.00
93	->	105	0.01	0.00
93	->	106	0.00	0.00
94	->	1	-0.11	0.02
94	->	2	-0.01	0.00
94	->	3	-0.01	0.01
94	->	4	-0.00	0.00
94	->	5	0.01	0.00
94	->	6	-0.00	0.00
94	->	7	-0.00	0.00
94	->	8	0.01	0.00
94	->	9	0.00	0.00
94	->	10	0.01	0.00
94	->	11	0.01	0.00
94	->	12	-0.03	0.01
94	->	13	-0.02	0.00
94	->	14	-0.02	0.01
94	->	15	-0.06	0.04
94	->	16	-2.38	1.40
94	->	17	-0.03	0.02
94	->	18	-0.37	0.29
94	->	19	0.25	0.19
94	->	20	-0.01	0.00
94	->	21	-0.01	0.01
94	->	22	-0.10	0.07
94	->	23	-0.01	0.00
94	->	24	-0.00	0.00
94	->	25	-0.03	0.01
94	->	26	-0.00	0.00
94	->	27	-0.00	0.00
94	->	28	-0.00	0.00
94	->	29	-0.00	0.00
94	->	30	0.00	0.00
94	->	31	-0.01	0.00
94	->	32	-0.00	0.00
94	->	33	-0.00	0.00
94	->	34	0.00	0.00
94	->	35	0.00	0.00
94	->	36	-0.00	0.00
94	->	37	0.00	0.00
94	->	38	0.00	0.00

94	->	39	0.00	0.00
94	->	40	-0.00	0.00
94	->	41	-0.00	0.00
94	->	42	-0.00	0.00
94	->	43	0.00	0.00
94	->	44	-0.02	0.03
94	->	45	-0.01	0.01
94	->	46	-0.01	0.02
94	->	47	-0.01	0.01
94	->	48	-0.00	0.00
94	->	49	-0.00	0.00
94	->	50	-0.00	0.00
94	->	51	-0.00	0.00
94	->	52	-0.00	0.00
94	->	53	-0.01	0.00
94	->	54	-0.00	0.00
94	->	55	-0.00	0.00
94	->	56	-0.00	0.00
94	->	57	-0.00	0.00
94	->	58	0.00	0.00
94	->	59	-0.00	0.00
94	->	60	-0.00	0.00
94	->	61	-0.00	0.00
94	->	62	0.00	0.00
94	->	63	0.01	0.00
94	->	64	0.00	0.00
94	->	65	-0.00	0.00
94	->	66	0.00	0.00
94	->	67	-0.00	0.00
94	->	68	-0.00	0.00
94	->	69	-0.00	0.00
94	->	70	-0.00	0.00
94	->	71	0.00	0.00
94	->	72	-0.01	0.00
94	->	73	-0.00	0.00
94	->	74	0.01	0.00
94	->	75	0.01	0.00
94	->	76	-0.01	0.01
94	->	77	-0.03	0.01
94	->	78	-0.01	0.00
94	->	79	-0.00	0.00
94	->	80	-0.00	0.00
94	->	81	-0.01	0.00
94	->	82	-0.00	0.00
94	->	83	-0.00	0.00
94	->	84	0.00	0.00
94	->	85	0.00	0.00
94	->	86	0.00	0.00
94	->	87	0.00	0.00
94	->	88	0.01	0.00

94	->	89	0.01	0.00
94	->	90	0.01	0.00
94	->	91	0.00	0.01
94	->	92	-0.25	0.06
94	->	93	-33.46	1.04
94	->	94	-180.32	2.74
94	->	95	-36.14	0.99
94	->	96	-0.14	0.10
94	->	97	-1.23	0.71
94	->	98	-10.46	1.07
94	->	99	-2.81	0.76
94	->	100	-0.19	0.06
94	->	101	-0.03	0.02
94	->	102	0.01	0.00
94	->	103	0.02	0.00
94	->	104	0.01	0.00
94	->	105	0.01	0.00
94	->	106	0.00	0.00
95	->	1	-0.04	0.01
95	->	2	-0.01	0.00
95	->	3	-0.02	0.02
95	->	4	-0.00	0.00
95	->	5	0.01	0.00
95	->	6	-0.00	0.00
95	->	7	-0.00	0.00
95	->	8	0.00	0.00
95	->	9	0.00	0.00
95	->	10	0.01	0.00
95	->	11	0.01	0.00
95	->	12	-0.01	0.00
95	->	13	-0.01	0.00
95	->	14	-0.01	0.01
95	->	15	-0.04	0.04
95	->	16	-1.36	0.97
95	->	17	-0.11	0.10
95	->	18	-0.51	0.43
95	->	19	0.01	0.04
95	->	20	-0.01	0.00
95	->	21	-0.01	0.01
95	->	22	-0.04	0.01
95	->	23	-0.00	0.00
95	->	24	-0.00	0.00
95	->	25	-0.02	0.01
95	->	26	-0.00	0.00
95	->	27	-0.00	0.00
95	->	28	-0.00	0.00
95	->	29	-0.00	0.00
95	->	30	0.00	0.00
95	->	31	-0.00	0.00
95	->	32	-0.00	0.00

95	->	33	-0.00	0.00
95	->	34	0.00	0.00
95	->	35	0.00	0.00
95	->	36	-0.00	0.00
95	->	37	0.00	0.00
95	->	38	0.00	0.00
95	->	39	0.00	0.00
95	->	40	-0.00	0.00
95	->	41	-0.00	0.00
95	->	42	-0.00	0.00
95	->	43	-0.00	0.00
95	->	44	-0.02	0.01
95	->	45	-0.00	0.00
95	->	46	0.01	0.00
95	->	47	-0.00	0.00
95	->	48	-0.00	0.00
95	->	49	-0.00	0.00
95	->	50	-0.00	0.00
95	->	51	-0.00	0.00
95	->	52	-0.00	0.00
95	->	53	-0.01	0.00
95	->	54	-0.00	0.00
95	->	55	-0.00	0.00
95	->	56	-0.00	0.00
95	->	57	-0.00	0.00
95	->	58	0.00	0.00
95	->	59	-0.00	0.00
95	->	60	-0.00	0.00
95	->	61	-0.00	0.00
95	->	62	0.00	0.00
95	->	63	0.00	0.00
95	->	64	0.00	0.00
95	->	65	-0.00	0.00
95	->	66	0.00	0.00
95	->	67	-0.00	0.00
95	->	68	-0.00	0.00
95	->	69	-0.00	0.00
95	->	70	-0.00	0.00
95	->	71	0.00	0.00
95	->	72	-0.01	0.00
95	->	73	-0.00	0.00
95	->	74	0.01	0.00
95	->	75	0.00	0.00
95	->	76	-0.01	0.00
95	->	77	-0.01	0.00
95	->	78	-0.00	0.00
95	->	79	-0.00	0.00
95	->	80	-0.00	0.00
95	->	81	-0.00	0.00
95	->	82	-0.00	0.00

95	->	83	0.00	0.00
95	->	84	0.00	0.00
95	->	85	0.00	0.00
95	->	86	0.00	0.00
95	->	87	0.00	0.00
95	->	88	0.00	0.00
95	->	89	0.00	0.00
95	->	90	0.01	0.00
95	->	91	0.00	0.00
95	->	92	-0.02	0.01
95	->	93	-0.26	0.06
95	->	94	-35.91	1.00
95	->	95	-94.32	2.46
95	->	96	-2.59	2.15
95	->	97	-5.35	1.89
95	->	98	-1.20	1.92
95	->	99	-0.14	0.06
95	->	100	-0.00	0.01
95	->	101	0.01	0.00
95	->	102	0.01	0.00
95	->	103	0.01	0.00
95	->	104	0.00	0.00
95	->	105	0.00	0.00
95	->	106	0.00	0.00
96	->	1	-0.01	0.01
96	->	2	-0.00	0.01
96	->	3	-0.03	0.11
96	->	4	-0.00	0.00
96	->	5	0.00	0.00
96	->	6	-0.00	0.00
96	->	7	-0.00	0.00
96	->	8	0.00	0.00
96	->	9	-0.00	0.00
96	->	10	0.00	0.00
96	->	11	0.00	0.00
96	->	12	-0.00	0.00
96	->	13	-0.01	0.00
96	->	14	-0.00	0.00
96	->	15	-0.01	0.01
96	->	16	-0.08	0.09
96	->	17	-0.31	0.49
96	->	18	-0.32	0.44
96	->	19	0.00	0.02
96	->	20	-0.02	0.02
96	->	21	-0.17	0.32
96	->	22	-0.03	0.03
96	->	23	-0.00	0.00
96	->	24	-0.01	0.01
96	->	25	-0.10	0.15
96	->	26	-0.00	0.00

96	->	27	-0.00	0.00
96	->	28	-0.00	0.00
96	->	29	-0.00	0.00
96	->	30	-0.00	0.00
96	->	31	-0.01	0.00
96	->	32	-0.01	0.01
96	->	33	-0.02	0.06
96	->	34	-0.00	0.00
96	->	35	0.00	0.00
96	->	36	-0.00	0.00
96	->	37	0.00	0.00
96	->	38	0.00	0.00
96	->	39	-0.00	0.00
96	->	40	-0.00	0.00
96	->	41	-0.00	0.00
96	->	42	-0.00	0.00
96	->	43	-0.00	0.00
96	->	44	-0.02	0.02
96	->	45	-0.01	0.02
96	->	46	0.01	0.01
96	->	47	-0.00	0.00
96	->	48	-0.00	0.00
96	->	49	-0.00	0.00
96	->	50	-0.00	0.00
96	->	51	-0.00	0.00
96	->	52	-0.00	0.00
96	->	53	-0.01	0.00
96	->	54	-0.00	0.00
96	->	55	0.00	0.00
96	->	56	-0.00	0.00
96	->	57	-0.00	0.00
96	->	58	0.00	0.00
96	->	59	-0.00	0.00
96	->	60	-0.00	0.00
96	->	61	-0.00	0.00
96	->	62	0.00	0.00
96	->	63	0.00	0.00
96	->	64	0.00	0.00
96	->	65	-0.00	0.00
96	->	66	0.00	0.00
96	->	67	0.00	0.00
96	->	68	-0.00	0.00
96	->	69	-0.00	0.00
96	->	70	-0.00	0.00
96	->	71	0.00	0.00
96	->	72	-0.00	0.00
96	->	73	-0.00	0.00
96	->	74	0.00	0.00
96	->	75	0.00	0.00
96	->	76	-0.00	0.00

96	->	77	-0.00	0.00
96	->	78	-0.00	0.00
96	->	79	-0.00	0.00
96	->	80	-0.00	0.00
96	->	81	-0.00	0.00
96	->	82	-0.00	0.00
96	->	83	0.00	0.00
96	->	84	0.00	0.00
96	->	85	0.00	0.00
96	->	86	0.00	0.00
96	->	87	0.00	0.00
96	->	88	0.00	0.00
96	->	89	0.00	0.00
96	->	90	0.00	0.00
96	->	91	0.00	0.00
96	->	92	-0.00	0.00
96	->	93	-0.01	0.01
96	->	94	-0.14	0.10
96	->	95	-2.54	2.11
96	->	96	-48.61	3.09
96	->	97	-35.38	1.86
96	->	98	-0.51	0.77
96	->	99	-0.02	0.01
96	->	100	-0.00	0.00
96	->	101	0.00	0.00
96	->	102	0.00	0.00
96	->	103	0.00	0.00
96	->	104	0.00	0.00
96	->	105	0.00	0.00
96	->	106	0.00	0.00
97	->	1	-0.02	0.01
97	->	2	-0.00	0.00
97	->	3	-0.02	0.03
97	->	4	0.00	0.00
97	->	5	0.01	0.00
97	->	6	-0.00	0.00
97	->	7	-0.00	0.00
97	->	8	0.00	0.00
97	->	9	-0.00	0.00
97	->	10	0.01	0.00
97	->	11	0.01	0.00
97	->	12	-0.01	0.00
97	->	13	-0.01	0.00
97	->	14	-0.01	0.00
97	->	15	-0.00	0.01
97	->	16	-0.11	0.29
97	->	17	-0.37	0.72
97	->	18	-0.52	0.63
97	->	19	0.00	0.04
97	->	20	-0.02	0.02

97	->	21	-0.10	0.13
97	->	22	-0.09	0.05
97	->	23	-0.01	0.00
97	->	24	-0.01	0.01
97	->	25	-0.31	0.91
97	->	26	-0.00	0.00
97	->	27	-0.00	0.00
97	->	28	-0.00	0.00
97	->	29	-0.00	0.00
97	->	30	0.00	0.00
97	->	31	-0.02	0.01
97	->	32	-0.02	0.03
97	->	33	-0.13	0.27
97	->	34	-0.00	0.00
97	->	35	0.02	0.00
97	->	36	-0.03	0.01
97	->	37	0.00	0.00
97	->	38	0.01	0.00
97	->	39	-0.00	0.00
97	->	40	-0.01	0.00
97	->	41	-0.00	0.00
97	->	42	-0.00	0.00
97	->	43	-0.00	0.01
97	->	44	-0.12	0.06
97	->	45	-0.21	0.24
97	->	46	0.06	0.05
97	->	47	-0.00	0.01
97	->	48	-0.02	0.01
97	->	49	-0.01	0.00
97	->	50	-0.00	0.00
97	->	51	-0.00	0.00
97	->	52	-0.01	0.00
97	->	53	-0.03	0.01
97	->	54	-0.00	0.00
97	->	55	-0.00	0.00
97	->	56	-0.00	0.00
97	->	57	-0.00	0.00
97	->	58	0.01	0.01
97	->	59	-0.01	0.00
97	->	60	-0.00	0.00
97	->	61	-0.00	0.00
97	->	62	0.00	0.00
97	->	63	0.00	0.00
97	->	64	0.00	0.00
97	->	65	-0.00	0.00
97	->	66	0.00	0.00
97	->	67	0.00	0.00
97	->	68	-0.00	0.00
97	->	69	-0.01	0.00
97	->	70	0.00	0.00

97	->	71	0.00	0.00
97	->	72	-0.01	0.00
97	->	73	-0.00	0.00
97	->	74	0.01	0.00
97	->	75	0.01	0.00
97	->	76	-0.01	0.00
97	->	77	-0.01	0.00
97	->	78	-0.01	0.00
97	->	79	-0.00	0.00
97	->	80	-0.00	0.00
97	->	81	-0.00	0.00
97	->	82	-0.00	0.00
97	->	83	0.00	0.00
97	->	84	0.00	0.00
97	->	85	0.00	0.00
97	->	86	0.00	0.00
97	->	87	0.01	0.00
97	->	88	0.01	0.00
97	->	89	0.01	0.00
97	->	90	0.02	0.01
97	->	91	0.01	0.01
97	->	92	-0.00	0.01
97	->	93	-0.05	0.04
97	->	94	-1.22	0.71
97	->	95	-5.34	1.86
97	->	96	-35.13	1.83
97	->	97	-135.34	3.36
97	->	98	-34.97	1.21
97	->	99	-0.22	0.13
97	->	100	-0.00	0.01
97	->	101	0.01	0.00
97	->	102	0.01	0.00
97	->	103	0.00	0.00
97	->	104	0.00	0.00
97	->	105	0.00	0.00
97	->	106	0.00	0.00
98	->	1	-0.02	0.00
98	->	2	-0.00	0.00
98	->	3	-0.00	0.00
98	->	4	-0.00	0.00
98	->	5	0.01	0.00
98	->	6	-0.00	0.00
98	->	7	-0.00	0.00
98	->	8	0.00	0.00
98	->	9	-0.00	0.00
98	->	10	0.01	0.00
98	->	11	0.00	0.00
98	->	12	-0.01	0.00
98	->	13	-0.01	0.00
98	->	14	-0.00	0.00

98	->	15	-0.00	0.00
98	->	16	-0.04	0.03
98	->	17	-0.02	0.02
98	->	18	-0.38	0.28
98	->	19	-0.07	0.12
98	->	20	-0.01	0.00
98	->	21	-0.03	0.02
98	->	22	-0.39	0.43
98	->	23	-0.00	0.00
98	->	24	-0.00	0.00
98	->	25	-0.11	0.09
98	->	26	-0.00	0.00
98	->	27	-0.00	0.00
98	->	28	-0.00	0.00
98	->	29	-0.00	0.00
98	->	30	0.00	0.00
98	->	31	-0.02	0.01
98	->	32	-0.01	0.01
98	->	33	-0.15	0.17
98	->	34	-0.00	0.00
98	->	35	0.03	0.01
98	->	36	-0.04	0.02
98	->	37	-0.00	0.00
98	->	38	0.02	0.00
98	->	39	-0.00	0.00
98	->	40	-0.01	0.00
98	->	41	-0.00	0.00
98	->	42	-0.01	0.00
98	->	43	-0.01	0.01
98	->	44	-0.30	0.16
98	->	45	-1.70	1.66
98	->	46	-0.15	0.34
98	->	47	0.01	0.05
98	->	48	-0.09	0.06
98	->	49	-0.18	0.20
98	->	50	0.00	0.01
98	->	51	-0.01	0.00
98	->	52	-0.02	0.01
98	->	53	-0.12	0.06
98	->	54	-0.00	0.00
98	->	55	-0.00	0.00
98	->	56	-0.00	0.00
98	->	57	-0.00	0.00
98	->	58	0.03	0.01
98	->	59	-0.01	0.00
98	->	60	-0.00	0.00
98	->	61	-0.00	0.00
98	->	62	0.00	0.00
98	->	63	0.01	0.00
98	->	64	0.01	0.00

98	->	65	-0.00	0.00
98	->	66	0.00	0.00
98	->	67	0.00	0.00
98	->	68	-0.01	0.00
98	->	69	-0.01	0.00
98	->	70	-0.00	0.00
98	->	71	-0.00	0.00
98	->	72	-0.03	0.01
98	->	73	-0.01	0.00
98	->	74	0.02	0.00
98	->	75	0.01	0.00
98	->	76	-0.01	0.00
98	->	77	-0.01	0.00
98	->	78	-0.01	0.00
98	->	79	-0.00	0.00
98	->	80	-0.00	0.00
98	->	81	-0.01	0.00
98	->	82	-0.00	0.00
98	->	83	0.00	0.00
98	->	84	0.00	0.00
98	->	85	0.00	0.00
98	->	86	0.01	0.00
98	->	87	0.01	0.00
98	->	88	0.02	0.00
98	->	89	0.01	0.00
98	->	90	0.01	0.00
98	->	91	-0.00	0.01
98	->	92	-0.11	0.05
98	->	93	-0.28	0.28
98	->	94	-10.42	1.07
98	->	95	-1.20	1.92
98	->	96	-0.50	0.76
98	->	97	-34.76	1.20
98	->	98	-214.42	4.01
98	->	99	-33.97	1.25
98	->	100	-0.10	0.06
98	->	101	-0.00	0.01
98	->	102	0.01	0.00
98	->	103	0.01	0.00
98	->	104	0.00	0.00
98	->	105	0.00	0.00
98	->	106	0.00	0.00
99	->	1	-0.02	0.00
99	->	2	-0.00	0.00
99	->	3	-0.00	0.00
99	->	4	0.00	0.00
99	->	5	0.01	0.00
99	->	6	-0.00	0.00
99	->	7	-0.00	0.00
99	->	8	0.00	0.00

99	->	9	0.00	0.00
99	->	10	0.01	0.00
99	->	11	0.00	0.00
99	->	12	-0.01	0.00
99	->	13	-0.01	0.00
99	->	14	-0.00	0.00
99	->	15	-0.00	0.00
99	->	16	-0.02	0.03
99	->	17	-0.01	0.01
99	->	18	-0.06	0.07
99	->	19	0.02	0.05
99	->	20	-0.00	0.00
99	->	21	-0.01	0.00
99	->	22	-2.19	2.44
99	->	23	-0.01	0.00
99	->	24	-0.00	0.00
99	->	25	-0.07	0.02
99	->	26	-0.00	0.00
99	->	27	-0.00	0.00
99	->	28	-0.00	0.00
99	->	29	-0.00	0.00
99	->	30	0.00	0.00
99	->	31	-0.03	0.00
99	->	32	-0.00	0.00
99	->	33	-0.03	0.01
99	->	34	-0.00	0.00
99	->	35	0.03	0.01
99	->	36	-0.04	0.02
99	->	37	-0.00	0.00
99	->	38	0.02	0.00
99	->	39	-0.00	0.00
99	->	40	-0.02	0.01
99	->	41	-0.00	0.00
99	->	42	-0.01	0.00
99	->	43	-0.01	0.00
99	->	44	-0.60	0.21
99	->	45	-0.51	1.14
99	->	46	-1.54	0.43
99	->	47	-0.02	0.08
99	->	48	-0.16	0.12
99	->	49	-2.00	2.08
99	->	50	-0.04	0.06
99	->	51	-0.02	0.01
99	->	52	-0.05	0.03
99	->	53	-2.20	1.02
99	->	54	-0.00	0.00
99	->	55	-0.00	0.00
99	->	56	-0.01	0.00
99	->	57	-0.00	0.00
99	->	58	0.04	0.02

99	->	59	-0.05	0.02
99	->	60	-0.04	0.03
99	->	61	-0.02	0.03
99	->	62	0.00	0.00
99	->	63	0.01	0.00
99	->	64	0.01	0.00
99	->	65	-0.00	0.00
99	->	66	0.01	0.00
99	->	67	0.00	0.00
99	->	68	-0.01	0.00
99	->	69	-0.03	0.01
99	->	70	-0.01	0.00
99	->	71	-0.01	0.01
99	->	72	-0.13	0.05
99	->	73	-0.02	0.03
99	->	74	0.04	0.01
99	->	75	0.02	0.00
99	->	76	-0.03	0.01
99	->	77	-0.02	0.01
99	->	78	-0.02	0.00
99	->	79	-0.00	0.00
99	->	80	-0.00	0.00
99	->	81	-0.01	0.00
99	->	82	-0.00	0.00
99	->	83	0.00	0.00
99	->	84	0.00	0.00
99	->	85	0.00	0.00
99	->	86	0.01	0.00
99	->	87	0.02	0.00
99	->	88	0.02	0.00
99	->	89	0.01	0.01
99	->	90	0.01	0.01
99	->	91	0.00	0.03
99	->	92	-0.36	0.51
99	->	93	-11.28	0.77
99	->	94	-2.81	0.76
99	->	95	-0.14	0.06
99	->	96	-0.02	0.01
99	->	97	-0.22	0.13
99	->	98	-33.73	1.23
99	->	99	-173.05	3.73
99	->	100	-36.46	0.96
99	->	101	-0.41	0.09
99	->	102	-0.04	0.01
99	->	103	0.00	0.00
99	->	104	0.01	0.00
99	->	105	0.00	0.00
99	->	106	0.00	0.00
100	->	1	-0.02	0.00
100	->	2	-0.00	0.00

100	->	3	-0.00	0.00
100	->	4	0.00	0.00
100	->	5	0.01	0.00
100	->	6	-0.00	0.00
100	->	7	-0.00	0.00
100	->	8	0.00	0.00
100	->	9	0.00	0.00
100	->	10	0.01	0.00
100	->	11	0.00	0.00
100	->	12	-0.01	0.00
100	->	13	-0.00	0.00
100	->	14	-0.00	0.00
100	->	15	-0.00	0.00
100	->	16	-0.05	0.01
100	->	17	-0.00	0.00
100	->	18	-0.01	0.00
100	->	19	0.02	0.01
100	->	20	-0.00	0.00
100	->	21	-0.00	0.00
100	->	22	0.20	0.24
100	->	23	-0.01	0.00
100	->	24	-0.00	0.00
100	->	25	-0.02	0.00
100	->	26	-0.00	0.00
100	->	27	-0.00	0.00
100	->	28	-0.00	0.00
100	->	29	-0.00	0.00
100	->	30	0.00	0.00
100	->	31	-0.01	0.00
100	->	32	-0.00	0.00
100	->	33	-0.01	0.00
100	->	34	-0.00	0.00
100	->	35	0.02	0.00
100	->	36	-0.02	0.01
100	->	37	-0.00	0.00
100	->	38	0.01	0.00
100	->	39	-0.00	0.00
100	->	40	-0.01	0.00
100	->	41	-0.00	0.00
100	->	42	-0.00	0.00
100	->	43	0.00	0.00
100	->	44	1.09	0.64
100	->	45	-0.02	0.02
100	->	46	-5.23	0.93
100	->	47	-0.27	0.09
100	->	48	-0.02	0.01
100	->	49	-0.19	0.09
100	->	50	-0.09	0.07
100	->	51	-0.01	0.00
100	->	52	-0.02	0.01

100	->	53	-7.78	2.26
100	->	54	-0.00	0.00
100	->	55	-0.00	0.00
100	->	56	-0.00	0.00
100	->	57	-0.00	0.00
100	->	58	0.05	0.02
100	->	59	-0.09	0.05
100	->	60	-0.23	0.21
100	->	61	-1.30	0.84
100	->	62	0.00	0.00
100	->	63	0.02	0.01
100	->	64	0.02	0.00
100	->	65	-0.00	0.00
100	->	66	0.01	0.00
100	->	67	-0.00	0.00
100	->	68	-0.02	0.00
100	->	69	-0.05	0.02
100	->	70	-0.02	0.02
100	->	71	0.01	0.05
100	->	72	-0.34	0.29
100	->	73	-1.08	0.62
100	->	74	0.03	0.04
100	->	75	0.02	0.01
100	->	76	-0.18	0.33
100	->	77	-0.09	0.07
100	->	78	-0.03	0.00
100	->	79	-0.01	0.00
100	->	80	-0.00	0.00
100	->	81	-0.02	0.01
100	->	82	-0.00	0.00
100	->	83	-0.00	0.00
100	->	84	0.01	0.00
100	->	85	0.00	0.00
100	->	86	0.01	0.00
100	->	87	0.01	0.00
100	->	88	0.01	0.00
100	->	89	-0.01	0.01
100	->	90	-0.09	0.07
100	->	91	-0.78	0.25
100	->	92	-11.63	0.82
100	->	93	0.24	0.19
100	->	94	-0.19	0.06
100	->	95	-0.00	0.01
100	->	96	-0.00	0.00
100	->	97	-0.00	0.01
100	->	98	-0.10	0.06
100	->	99	-36.31	0.94
100	->	100	-205.39	3.59
100	->	101	-31.89	0.82
100	->	102	-0.13	0.04

100	->	103	-0.00	0.01
100	->	104	0.01	0.00
100	->	105	0.01	0.00
100	->	106	0.00	0.00
101	->	1	-0.04	0.01
101	->	2	-0.00	0.00
101	->	3	-0.00	0.00
101	->	4	0.00	0.00
101	->	5	0.01	0.00
101	->	6	-0.00	0.00
101	->	7	-0.00	0.00
101	->	8	0.00	0.00
101	->	9	0.00	0.00
101	->	10	0.01	0.00
101	->	11	0.01	0.00
101	->	12	-0.02	0.01
101	->	13	-0.01	0.00
101	->	14	-0.00	0.00
101	->	15	-0.00	0.00
101	->	16	-0.06	0.01
101	->	17	-0.00	0.00
101	->	18	-0.01	0.00
101	->	19	0.04	0.01
101	->	20	-0.00	0.00
101	->	21	-0.00	0.00
101	->	22	-0.11	0.09
101	->	23	-0.01	0.00
101	->	24	-0.00	0.00
101	->	25	-0.01	0.00
101	->	26	-0.00	0.00
101	->	27	-0.00	0.00
101	->	28	-0.00	0.00
101	->	29	-0.00	0.00
101	->	30	0.00	0.00
101	->	31	-0.01	0.00
101	->	32	-0.00	0.00
101	->	33	-0.00	0.00
101	->	34	-0.00	0.00
101	->	35	0.01	0.00
101	->	36	-0.01	0.00
101	->	37	0.00	0.00
101	->	38	0.01	0.00
101	->	39	-0.00	0.00
101	->	40	-0.01	0.00
101	->	41	-0.00	0.00
101	->	42	-0.00	0.00
101	->	43	-0.00	0.00
101	->	44	0.37	0.48
101	->	45	-0.01	0.00
101	->	46	-0.63	0.32

101 ->	47	-0.30	0.23
101 ->	48	-0.01	0.00
101 ->	49	-0.01	0.00
101 ->	50	-0.04	0.02
101 ->	51	-0.00	0.00
101 ->	52	-0.00	0.00
101 ->	53	-0.08	0.03
101 ->	54	-0.00	0.00
101 ->	55	-0.00	0.00
101 ->	56	-0.00	0.00
101 ->	57	-0.00	0.00
101 ->	58	0.02	0.00
101 ->	59	-0.03	0.01
101 ->	60	-0.02	0.01
101 ->	61	-0.30	0.25
101 ->	62	0.00	0.00
101 ->	63	0.03	0.01
101 ->	64	0.02	0.01
101 ->	65	-0.00	0.00
101 ->	66	0.02	0.00
101 ->	67	-0.00	0.00
101 ->	68	-0.02	0.01
101 ->	69	-0.03	0.01
101 ->	70	-0.02	0.01
101 ->	71	-0.03	0.02
101 ->	72	-0.46	0.21
101 ->	73	-4.31	2.52
101 ->	74	-0.22	0.28
101 ->	75	-0.01	0.03
101 ->	76	-0.81	1.79
101 ->	77	-1.13	0.80
101 ->	78	-0.05	0.01
101 ->	79	-0.01	0.01
101 ->	80	-0.01	0.01
101 ->	81	-0.05	0.02
101 ->	82	-0.00	0.00
101 ->	83	-0.00	0.00
101 ->	84	0.01	0.00
101 ->	85	0.00	0.00
101 ->	86	0.01	0.00
101 ->	87	-0.00	0.00
101 ->	88	-0.01	0.01
101 ->	89	-0.08	0.03
101 ->	90	-1.12	0.49
101 ->	91	-11.74	0.73
101 ->	92	-4.37	0.64
101 ->	93	-0.07	0.06
101 ->	94	-0.03	0.02
101 ->	95	0.01	0.00
101 ->	96	0.00	0.00

101	->	97	0.01	0.00
101	->	98	-0.00	0.01
101	->	99	-0.41	0.09
101	->	100	-31.67	0.81
101	->	101	-210.37	3.94
101	->	102	-31.52	0.94
101	->	103	-0.21	0.06
101	->	104	0.00	0.00
101	->	105	0.01	0.00
101	->	106	0.00	0.00
102	->	1	-0.05	0.01
102	->	2	-0.00	0.00
102	->	3	-0.00	0.00
102	->	4	-0.00	0.00
102	->	5	0.01	0.00
102	->	6	-0.00	0.00
102	->	7	-0.00	0.00
102	->	8	0.00	0.00
102	->	9	0.00	0.00
102	->	10	0.01	0.00
102	->	11	0.01	0.00
102	->	12	-0.04	0.01
102	->	13	-0.01	0.00
102	->	14	-0.00	0.00
102	->	15	-0.00	0.00
102	->	16	-0.05	0.01
102	->	17	-0.00	0.00
102	->	18	-0.00	0.00
102	->	19	0.03	0.00
102	->	20	-0.00	0.00
102	->	21	-0.00	0.00
102	->	22	-0.07	0.03
102	->	23	-0.01	0.00
102	->	24	-0.00	0.00
102	->	25	-0.02	0.00
102	->	26	-0.00	0.00
102	->	27	-0.00	0.00
102	->	28	-0.00	0.00
102	->	29	-0.00	0.00
102	->	30	0.00	0.00
102	->	31	-0.01	0.00
102	->	32	-0.00	0.00
102	->	33	-0.00	0.00
102	->	34	-0.00	0.00
102	->	35	0.01	0.00
102	->	36	-0.00	0.00
102	->	37	0.00	0.00
102	->	38	0.01	0.00
102	->	39	-0.00	0.00
102	->	40	-0.01	0.00

102 ->	41	-0.00	0.00
102 ->	42	-0.00	0.00
102 ->	43	-0.00	0.00
102 ->	44	-0.14	0.11
102 ->	45	-0.00	0.00
102 ->	46	-0.05	0.06
102 ->	47	-0.11	0.09
102 ->	48	-0.00	0.00
102 ->	49	-0.00	0.00
102 ->	50	-0.02	0.01
102 ->	51	-0.00	0.00
102 ->	52	-0.00	0.00
102 ->	53	-0.02	0.00
102 ->	54	-0.00	0.00
102 ->	55	-0.00	0.00
102 ->	56	-0.00	0.00
102 ->	57	-0.00	0.00
102 ->	58	0.01	0.00
102 ->	59	-0.02	0.00
102 ->	60	-0.00	0.00
102 ->	61	-0.02	0.01
102 ->	62	0.00	0.00
102 ->	63	0.03	0.01
102 ->	64	0.02	0.01
102 ->	65	-0.00	0.00
102 ->	66	0.02	0.00
102 ->	67	-0.00	0.00
102 ->	68	-0.02	0.00
102 ->	69	-0.01	0.00
102 ->	70	-0.01	0.00
102 ->	71	-0.01	0.00
102 ->	72	-0.17	0.23
102 ->	73	-0.21	0.19
102 ->	74	-2.24	1.26
102 ->	75	-0.01	0.01
102 ->	76	-0.28	0.19
102 ->	77	-7.01	2.31
102 ->	78	-0.11	0.04
102 ->	79	-0.01	0.01
102 ->	80	-0.03	0.02
102 ->	81	-0.31	0.41
102 ->	82	-0.00	0.00
102 ->	83	-0.00	0.00
102 ->	84	0.02	0.00
102 ->	85	0.00	0.00
102 ->	86	0.01	0.00
102 ->	87	-0.02	0.01
102 ->	88	-0.10	0.04
102 ->	89	-0.71	0.30
102 ->	90	-11.60	0.82

102	->	91	-3.95	0.71
102	->	92	-0.45	0.12
102	->	93	-0.02	0.02
102	->	94	0.01	0.00
102	->	95	0.01	0.00
102	->	96	0.00	0.00
102	->	97	0.01	0.00
102	->	98	0.01	0.00
102	->	99	-0.04	0.01
102	->	100	-0.13	0.04
102	->	101	-31.31	0.94
102	->	102	-208.84	3.10
102	->	103	-33.60	1.12
102	->	104	-0.13	0.04
102	->	105	0.01	0.01
102	->	106	0.00	0.00
103	->	1	-0.04	0.01
103	->	2	-0.00	0.00
103	->	3	0.00	0.00
103	->	4	-0.00	0.00
103	->	5	0.01	0.00
103	->	6	-0.00	0.00
103	->	7	-0.00	0.00
103	->	8	0.00	0.00
103	->	9	0.00	0.00
103	->	10	0.02	0.00
103	->	11	0.01	0.00
103	->	12	-0.04	0.01
103	->	13	-0.01	0.00
103	->	14	-0.00	0.00
103	->	15	-0.00	0.00
103	->	16	-0.03	0.00
103	->	17	0.00	0.00
103	->	18	-0.00	0.00
103	->	19	0.02	0.00
103	->	20	-0.00	0.00
103	->	21	-0.00	0.00
103	->	22	-0.04	0.01
103	->	23	-0.01	0.00
103	->	24	-0.00	0.00
103	->	25	-0.01	0.00
103	->	26	-0.01	0.00
103	->	27	-0.00	0.00
103	->	28	-0.00	0.00
103	->	29	-0.00	0.00
103	->	30	0.00	0.00
103	->	31	-0.01	0.00
103	->	32	-0.00	0.00
103	->	33	-0.00	0.00
103	->	34	-0.00	0.00

103 ->	35	0.01	0.00
103 ->	36	-0.00	0.00
103 ->	37	0.00	0.00
103 ->	38	0.01	0.00
103 ->	39	0.00	0.00
103 ->	40	-0.01	0.00
103 ->	41	-0.00	0.00
103 ->	42	-0.00	0.00
103 ->	43	-0.00	0.00
103 ->	44	-0.07	0.03
103 ->	45	-0.00	0.00
103 ->	46	0.01	0.01
103 ->	47	-0.10	0.04
103 ->	48	-0.00	0.00
103 ->	49	-0.00	0.00
103 ->	50	-0.02	0.01
103 ->	51	-0.01	0.00
103 ->	52	-0.00	0.00
103 ->	53	-0.02	0.00
103 ->	54	-0.00	0.00
103 ->	55	-0.00	0.00
103 ->	56	-0.00	0.00
103 ->	57	-0.00	0.00
103 ->	58	0.01	0.00
103 ->	59	-0.01	0.00
103 ->	60	-0.00	0.00
103 ->	61	-0.01	0.00
103 ->	62	0.00	0.00
103 ->	63	0.02	0.00
103 ->	64	0.01	0.00
103 ->	65	-0.00	0.00
103 ->	66	0.01	0.00
103 ->	67	-0.00	0.00
103 ->	68	-0.02	0.00
103 ->	69	-0.01	0.00
103 ->	70	-0.00	0.00
103 ->	71	0.01	0.01
103 ->	72	0.60	0.75
103 ->	73	-0.04	0.03
103 ->	74	-4.05	1.35
103 ->	75	-0.02	0.03
103 ->	76	-0.08	0.05
103 ->	77	-2.55	2.67
103 ->	78	-0.34	0.44
103 ->	79	-0.01	0.00
103 ->	80	-0.03	0.03
103 ->	81	-1.73	2.77
103 ->	82	-0.01	0.00
103 ->	83	-0.01	0.00
103 ->	84	0.02	0.01

103	->	85	-0.00	0.00
103	->	86	-0.02	0.02
103	->	87	-0.13	0.06
103	->	88	-2.08	0.69
103	->	89	-6.11	0.52
103	->	90	-3.30	0.63
103	->	91	-0.35	0.07
103	->	92	-0.05	0.03
103	->	93	0.01	0.00
103	->	94	0.02	0.00
103	->	95	0.01	0.00
103	->	96	0.00	0.00
103	->	97	0.00	0.00
103	->	98	0.01	0.00
103	->	99	0.00	0.00
103	->	100	-0.00	0.01
103	->	101	-0.21	0.06
103	->	102	-33.37	1.10
103	->	103	-130.20	4.69
103	->	104	-34.15	0.96
103	->	105	-0.28	0.07
103	->	106	-0.02	0.01
104	->	1	-0.03	0.01
104	->	2	-0.00	0.00
104	->	3	0.00	0.00
104	->	4	-0.00	0.00
104	->	5	0.01	0.00
104	->	6	-0.00	0.00
104	->	7	-0.00	0.00
104	->	8	0.01	0.00
104	->	9	0.00	0.00
104	->	10	0.02	0.00
104	->	11	0.01	0.01
104	->	12	-0.04	0.02
104	->	13	-0.01	0.00
104	->	14	-0.00	0.00
104	->	15	-0.00	0.00
104	->	16	-0.02	0.00
104	->	17	0.00	0.00
104	->	18	0.00	0.00
104	->	19	0.02	0.00
104	->	20	0.00	0.00
104	->	21	-0.00	0.00
104	->	22	-0.03	0.01
104	->	23	-0.00	0.00
104	->	24	-0.00	0.00
104	->	25	-0.01	0.00
104	->	26	-0.00	0.00
104	->	27	-0.00	0.00
104	->	28	-0.00	0.00

104 ->	29	-0.00	0.00
104 ->	30	0.00	0.00
104 ->	31	-0.02	0.01
104 ->	32	-0.00	0.00
104 ->	33	-0.00	0.00
104 ->	34	-0.00	0.00
104 ->	35	0.01	0.00
104 ->	36	-0.00	0.00
104 ->	37	0.00	0.00
104 ->	38	0.01	0.00
104 ->	39	0.00	0.00
104 ->	40	-0.02	0.00
104 ->	41	-0.00	0.00
104 ->	42	-0.00	0.00
104 ->	43	-0.00	0.00
104 ->	44	-0.04	0.01
104 ->	45	-0.00	0.00
104 ->	46	0.02	0.00
104 ->	47	-0.02	0.01
104 ->	48	-0.00	0.00
104 ->	49	-0.00	0.00
104 ->	50	-0.01	0.00
104 ->	51	-0.01	0.00
104 ->	52	-0.00	0.00
104 ->	53	-0.01	0.00
104 ->	54	-0.00	0.00
104 ->	55	-0.00	0.00
104 ->	56	-0.00	0.00
104 ->	57	-0.00	0.00
104 ->	58	0.01	0.00
104 ->	59	-0.01	0.00
104 ->	60	-0.00	0.00
104 ->	61	-0.00	0.00
104 ->	62	0.00	0.00
104 ->	63	0.01	0.00
104 ->	64	0.01	0.00
104 ->	65	-0.00	0.00
104 ->	66	0.01	0.00
104 ->	67	0.00	0.00
104 ->	68	-0.01	0.00
104 ->	69	-0.01	0.00
104 ->	70	-0.00	0.00
104 ->	71	-0.00	0.00
104 ->	72	0.37	0.21
104 ->	73	-0.00	0.00
104 ->	74	-0.65	0.29
104 ->	75	-0.04	0.06
104 ->	76	-0.02	0.00
104 ->	77	-0.06	0.02
104 ->	78	-1.24	1.20

104	->	79	-0.01	0.00
104	->	80	-0.01	0.00
104	->	81	-0.43	0.96
104	->	82	-0.01	0.00
104	->	83	-0.00	0.00
104	->	84	0.01	0.00
104	->	85	-0.03	0.01
104	->	86	-0.13	0.06
104	->	87	-0.66	0.30
104	->	88	-11.45	0.71
104	->	89	-0.59	0.33
104	->	90	-0.30	0.08
104	->	91	-0.03	0.02
104	->	92	0.01	0.00
104	->	93	0.01	0.00
104	->	94	0.01	0.00
104	->	95	0.00	0.00
104	->	96	0.00	0.00
104	->	97	0.00	0.00
104	->	98	0.00	0.00
104	->	99	0.01	0.00
104	->	100	0.01	0.00
104	->	101	0.00	0.00
104	->	102	-0.13	0.04
104	->	103	-33.91	0.95
104	->	104	-216.41	3.23
104	->	105	-34.33	1.11
104	->	106	-0.19	0.04
105	->	1	-0.02	0.01
105	->	2	0.00	0.00
105	->	3	0.00	0.00
105	->	4	-0.00	0.00
105	->	5	0.01	0.00
105	->	6	-0.00	0.00
105	->	7	-0.00	0.00
105	->	8	0.01	0.00
105	->	9	0.00	0.00
105	->	10	0.02	0.01
105	->	11	0.01	0.01
105	->	12	-0.03	0.01
105	->	13	-0.01	0.00
105	->	14	-0.00	0.00
105	->	15	-0.00	0.00
105	->	16	-0.01	0.00
105	->	17	0.00	0.00
105	->	18	0.00	0.00
105	->	19	0.01	0.00
105	->	20	0.00	0.00
105	->	21	0.00	0.00
105	->	22	-0.02	0.00

105	->	23	-0.00	0.00
105	->	24	0.00	0.00
105	->	25	-0.01	0.00
105	->	26	-0.00	0.00
105	->	27	-0.00	0.00
105	->	28	-0.00	0.00
105	->	29	-0.00	0.00
105	->	30	0.00	0.00
105	->	31	-0.02	0.01
105	->	32	-0.00	0.00
105	->	33	-0.00	0.00
105	->	34	-0.00	0.00
105	->	35	0.00	0.00
105	->	36	-0.00	0.00
105	->	37	-0.00	0.00
105	->	38	0.00	0.00
105	->	39	-0.00	0.00
105	->	40	-0.01	0.01
105	->	41	-0.00	0.00
105	->	42	-0.01	0.00
105	->	43	-0.00	0.00
105	->	44	-0.02	0.00
105	->	45	-0.00	0.00
105	->	46	0.01	0.00
105	->	47	-0.01	0.00
105	->	48	-0.00	0.00
105	->	49	-0.00	0.00
105	->	50	-0.00	0.00
105	->	51	-0.01	0.00
105	->	52	-0.00	0.00
105	->	53	-0.01	0.00
105	->	54	-0.00	0.00
105	->	55	-0.00	0.00
105	->	56	-0.00	0.00
105	->	57	-0.00	0.00
105	->	58	0.00	0.00
105	->	59	-0.01	0.00
105	->	60	-0.00	0.00
105	->	61	-0.00	0.00
105	->	62	0.00	0.00
105	->	63	0.01	0.00
105	->	64	0.00	0.00
105	->	65	-0.00	0.00
105	->	66	0.01	0.00
105	->	67	0.00	0.00
105	->	68	-0.02	0.00
105	->	69	-0.01	0.00
105	->	70	-0.00	0.00
105	->	71	-0.00	0.00
105	->	72	0.18	0.18

105	->	73	-0.00	0.00
105	->	74	0.02	0.04
105	->	75	0.01	0.02
105	->	76	-0.02	0.00
105	->	77	-0.04	0.01
105	->	78	-5.67	2.36
105	->	79	-0.01	0.00
105	->	80	-0.00	0.00
105	->	81	-0.08	0.03
105	->	82	-0.01	0.00
105	->	83	-0.00	0.00
105	->	84	0.01	0.00
105	->	85	-0.21	0.07
105	->	86	-1.23	0.74
105	->	87	-11.24	0.87
105	->	88	-4.64	1.13
105	->	89	-0.19	0.09
105	->	90	-0.04	0.02
105	->	91	0.01	0.00
105	->	92	0.02	0.00
105	->	93	0.01	0.00
105	->	94	0.01	0.00
105	->	95	0.00	0.00
105	->	96	0.00	0.00
105	->	97	0.00	0.00
105	->	98	0.00	0.00
105	->	99	0.00	0.00
105	->	100	0.01	0.00
105	->	101	0.01	0.00
105	->	102	0.01	0.01
105	->	103	-0.28	0.07
105	->	104	-34.03	1.09
105	->	105	-180.47	3.09
105	->	106	-36.15	1.10
106	->	1	-0.01	0.00
106	->	2	0.00	0.00
106	->	3	0.00	0.00
106	->	4	-0.00	0.00
106	->	5	0.01	0.00
106	->	6	-0.00	0.00
106	->	7	0.00	0.00
106	->	8	0.00	0.00
106	->	9	0.00	0.00
106	->	10	0.01	0.00
106	->	11	0.01	0.00
106	->	12	-0.01	0.00
106	->	13	-0.00	0.00
106	->	14	-0.00	0.00
106	->	15	-0.00	0.00
106	->	16	-0.00	0.00

106	->	17	0.00	0.00
106	->	18	0.00	0.00
106	->	19	0.01	0.00
106	->	20	0.00	0.00
106	->	21	0.00	0.00
106	->	22	-0.01	0.00
106	->	23	-0.00	0.00
106	->	24	0.00	0.00
106	->	25	-0.01	0.00
106	->	26	-0.00	0.00
106	->	27	-0.00	0.00
106	->	28	-0.00	0.00
106	->	29	-0.00	0.00
106	->	30	0.00	0.00
106	->	31	-0.02	0.01
106	->	32	-0.00	0.00
106	->	33	0.00	0.00
106	->	34	-0.00	0.00
106	->	35	0.01	0.00
106	->	36	-0.00	0.00
106	->	37	0.00	0.00
106	->	38	0.01	0.00
106	->	39	-0.00	0.00
106	->	40	-0.03	0.01
106	->	41	-0.00	0.00
106	->	42	-0.00	0.00
106	->	43	-0.00	0.00
106	->	44	-0.01	0.00
106	->	45	0.00	0.00
106	->	46	0.01	0.00
106	->	47	-0.00	0.00
106	->	48	-0.00	0.00
106	->	49	-0.00	0.00
106	->	50	-0.00	0.00
106	->	51	-0.01	0.00
106	->	52	-0.00	0.00
106	->	53	-0.01	0.00
106	->	54	-0.00	0.00
106	->	55	-0.00	0.00
106	->	56	-0.00	0.00
106	->	57	-0.00	0.00
106	->	58	0.00	0.00
106	->	59	-0.01	0.00
106	->	60	-0.00	0.00
106	->	61	-0.00	0.00
106	->	62	0.00	0.00
106	->	63	0.00	0.00
106	->	64	0.00	0.00
106	->	65	-0.00	0.00
106	->	66	0.00	0.00

106	->	67	0.00	0.00
106	->	68	-0.01	0.00
106	->	69	-0.00	0.00
106	->	70	-0.00	0.00
106	->	71	-0.00	0.00
106	->	72	-0.06	0.02
106	->	73	-0.00	0.00
106	->	74	0.01	0.00
106	->	75	0.01	0.00
106	->	76	-0.01	0.00
106	->	77	-0.01	0.00
106	->	78	-0.15	0.30
106	->	79	-0.00	0.00
106	->	80	-0.00	0.00
106	->	81	-0.02	0.01
106	->	82	-0.00	0.00
106	->	83	-0.00	0.00
106	->	84	0.00	0.00
106	->	85	-2.19	1.60
106	->	86	-10.62	0.74
106	->	87	0.20	0.14
106	->	88	-0.25	0.07
106	->	89	-0.02	0.02
106	->	90	0.01	0.00
106	->	91	0.01	0.00
106	->	92	0.01	0.00
106	->	93	0.00	0.00
106	->	94	0.00	0.00
106	->	95	0.00	0.00
106	->	96	0.00	0.00
106	->	97	0.00	0.00
106	->	98	0.00	0.00
106	->	99	0.00	0.00
106	->	100	0.00	0.00
106	->	101	0.00	0.00
106	->	102	0.00	0.00
106	->	103	-0.02	0.01
106	->	104	-0.19	0.04
106	->	105	-35.89	1.11
106	->	106	-215.96	2.56
