# Supplementary material for: Investigating dynamic and energetic determinants of protein nucleic acid recognition: analysis of the zinc finger zif268-DNA complexes
Source: BMC Struct Biol. 2010 Nov 24;10:42. doi: 10.1186/1472-6807-10-42 (PMC3002361; doi:10.1186/1472-6807-10-42)
Supplement: Additional file 6 — Average pair interaction energies for 1A1K Complex and their standard deviations. [file 1472-6807-10-42-S6.PDF]

=>> 1A1K

Residue ->	Residue	TGBTOT	SD
1 ->	1	-248.55	5.01
1 ->	2	-0.79	0.38
1 ->	3	-1.05	0.23
1 ->	4	-0.13	0.03
1 ->	5	-0.11	0.01
1 ->	6	-0.00	0.00
1 ->	7	-0.00	0.00
1 ->	8	-0.07	0.04
1 ->	9	-0.00	0.00
1 ->	10	-0.09	0.01
1 ->	11	-0.15	0.02
1 ->	12	-0.58	1.32
1 ->	13	-5.35	1.37
1 ->	14	-2.91	0.72
1 ->	15	-3.61	1.13
1 ->	16	0.03	0.08
1 ->	17	-0.05	0.01
1 ->	18	0.01	0.01
1 ->	19	-0.32	0.20
1 ->	20	-0.05	0.02
1 ->	21	0.00	0.00
1 ->	22	0.08	0.02
1 ->	23	0.00	0.00
1 ->	24	0.00	0.00
1 ->	25	0.03	0.00
1 ->	26	0.00	0.00
1 ->	27	-0.01	0.00
1 ->	28	0.00	0.00
1 ->	29	0.00	0.00
1 ->	30	0.00	0.00
1 ->	31	0.01	0.00
1 ->	32	0.00	0.00
1 ->	33	-0.00	0.00
1 ->	34	0.00	0.00
1 ->	35	-0.01	0.00
1 ->	36	0.00	0.00
1 ->	37	-0.00	0.00
1 ->	38	-0.01	0.00
1 ->	39	-0.00	0.00
1 ->	40	0.01	0.00
1 ->	41	0.00	0.00
1 ->	42	0.00	0.00
1 ->	43	0.00	0.00
1 ->	44	0.05	0.01
1 ->	45	0.00	0.00
1 ->	46	-0.03	0.00

1	->	47	0.00	0.00
1	->	48	0.00	0.00
1	->	49	0.00	0.00
1	->	50	0.00	0.00
1	->	51	0.00	0.00
1	->	52	0.00	0.00
1	->	53	0.01	0.00
1	->	54	0.00	0.00
1	->	55	0.00	0.00
1	->	56	0.00	0.00
1	->	57	0.00	0.00
1	->	58	-0.01	0.00
1	->	59	0.01	0.00
1	->	60	0.00	0.00
1	->	61	0.00	0.00
1	->	62	-0.00	0.00
1	->	63	-0.01	0.00
1	->	64	-0.00	0.00
1	->	65	-0.00	0.00
1	->	66	-0.00	0.00
1	->	67	0.00	0.00
1	->	68	0.01	0.00
1	->	69	0.00	0.00
1	->	70	0.00	0.00
1	->	71	-0.00	0.00
1	->	72	0.02	0.00
1	->	73	0.00	0.00
1	->	74	-0.02	0.00
1	->	75	-0.01	0.00
1	->	76	0.01	0.00
1	->	77	0.03	0.01
1	->	78	0.02	0.00
1	->	79	0.00	0.00
1	->	80	0.00	0.00
1	->	81	0.02	0.01
1	->	82	0.00	0.00
1	->	83	0.00	0.00
1	->	84	0.00	0.00
1	->	85	0.00	0.00
1	->	86	-0.00	0.00
1	->	87	-0.01	0.00
1	->	88	-0.01	0.00
1	->	89	-0.01	0.00
1	->	90	-0.03	0.01
1	->	91	-0.18	0.11
1	->	92	-2.35	1.61
1	->	93	-8.77	4.20
1	->	94	-0.18	0.13
1	->	95	-0.04	0.01
1	->	96	-0.01	0.00

1	->	97	-0.01	0.00
1	->	98	-0.04	0.01
1	->	99	-0.04	0.00
1	->	100	-0.02	0.00
1	->	101	-0.02	0.00
1	->	102	-0.04	0.00
1	->	103	-0.05	0.01
1	->	104	-0.05	0.01
1	->	105	-0.05	0.01
1	->	106	-0.03	0.01
1	->	107	-0.01	0.00
2	->	1	-0.79	0.37
2	->	2	1.47	0.69
2	->	3	-12.64	0.44
2	->	4	-0.36	0.14
2	->	5	-0.01	0.00
2	->	6	-0.00	0.00
2	->	7	-0.00	0.00
2	->	8	0.00	0.00
2	->	9	-0.00	0.00
2	->	10	-0.00	0.00
2	->	11	-0.00	0.00
2	->	12	-0.02	0.01
2	->	13	-1.80	0.85
2	->	14	-0.88	0.22
2	->	15	-0.85	0.35
2	->	16	-0.14	0.08
2	->	17	-0.04	0.01
2	->	18	-0.00	0.00
2	->	19	-0.01	0.00
2	->	20	-0.03	0.01
2	->	21	-0.00	0.00
2	->	22	-0.00	0.00
2	->	23	-0.00	0.00
2	->	24	-0.00	0.00
2	->	25	-0.00	0.00
2	->	26	-0.00	0.00
2	->	27	-0.00	0.00
2	->	28	-0.00	0.00
2	->	29	0.00	0.00
2	->	30	-0.00	0.00
2	->	31	-0.00	0.00
2	->	32	0.00	0.00
2	->	33	0.00	0.00
2	->	34	-0.00	0.00
2	->	35	-0.00	0.00
2	->	36	-0.00	0.00
2	->	37	0.00	0.00
2	->	38	0.00	0.00
2	->	39	0.00	0.00

2	->	40	0.00	0.00
2	->	41	0.00	0.00
2	->	42	0.00	0.00
2	->	43	-0.00	0.00
2	->	44	0.00	0.00
2	->	45	0.00	0.00
2	->	46	-0.00	0.00
2	->	47	0.00	0.00
2	->	48	0.00	0.00
2	->	49	0.00	0.00
2	->	50	0.00	0.00
2	->	51	0.00	0.00
2	->	52	0.00	0.00
2	->	53	0.00	0.00
2	->	54	0.00	0.00
2	->	55	0.00	0.00
2	->	56	0.00	0.00
2	->	57	0.00	0.00
2	->	58	-0.00	0.00
2	->	59	0.00	0.00
2	->	60	0.00	0.00
2	->	61	0.00	0.00
2	->	62	0.00	0.00
2	->	63	-0.00	0.00
2	->	64	-0.00	0.00
2	->	65	0.00	0.00
2	->	66	-0.00	0.00
2	->	67	0.00	0.00
2	->	68	0.00	0.00
2	->	69	0.00	0.00
2	->	70	0.00	0.00
2	->	71	0.00	0.00
2	->	72	0.00	0.00
2	->	73	0.00	0.00
2	->	74	-0.00	0.00
2	->	75	-0.00	0.00
2	->	76	0.00	0.00
2	->	77	0.00	0.00
2	->	78	0.00	0.00
2	->	79	0.00	0.00
2	->	80	0.00	0.00
2	->	81	0.00	0.00
2	->	82	0.00	0.00
2	->	83	0.00	0.00
2	->	84	0.00	0.00
2	->	85	0.00	0.00
2	->	86	0.00	0.00
2	->	87	-0.00	0.00
2	->	88	-0.00	0.00
2	->	89	-0.00	0.00

2	->	90	-0.00	0.00
2	->	91	-0.00	0.00
2	->	92	-0.01	0.00
2	->	93	-0.02	0.01
2	->	94	-0.01	0.01
2	->	95	-0.00	0.00
2	->	96	-0.00	0.00
2	->	97	-0.00	0.00
2	->	98	-0.00	0.00
2	->	99	-0.00	0.00
2	->	100	-0.00	0.00
2	->	101	-0.00	0.00
2	->	102	-0.00	0.00
2	->	103	-0.00	0.00
2	->	104	-0.00	0.00
2	->	105	-0.00	0.00
2	->	106	-0.00	0.00
2	->	107	-0.00	0.00
3	->	1	-1.06	0.23
3	->	2	-12.73	0.43
3	->	3	19.04	1.34
3	->	4	-9.86	0.42
3	->	5	-0.48	0.07
3	->	6	-0.03	0.01
3	->	7	0.00	0.01
3	->	8	-0.01	0.02
3	->	9	-0.00	0.00
3	->	10	-0.01	0.01
3	->	11	-0.01	0.00
3	->	12	-0.17	0.06
3	->	13	-3.30	0.81
3	->	14	-3.51	0.66
3	->	15	-1.13	0.30
3	->	16	-2.33	0.68
3	->	17	-1.69	0.60
3	->	18	-0.03	0.01
3	->	19	-0.07	0.02
3	->	20	-1.36	0.42
3	->	21	-0.03	0.01
3	->	22	-0.01	0.00
3	->	23	-0.02	0.01
3	->	24	-0.02	0.01
3	->	25	-0.00	0.00
3	->	26	-0.00	0.00
3	->	27	-0.01	0.00
3	->	28	-0.00	0.00
3	->	29	0.00	0.00
3	->	30	-0.00	0.00
3	->	31	0.00	0.00
3	->	32	-0.00	0.00

3 ->	33	-0.00	0.00
3 ->	34	-0.00	0.00
3 ->	35	-0.00	0.00
3 ->	36	0.00	0.00
3 ->	37	0.00	0.00
3 ->	38	0.00	0.00
3 ->	39	0.00	0.00
3 ->	40	-0.00	0.00
3 ->	41	0.00	0.00
3 ->	42	0.00	0.00
3 ->	43	-0.00	0.00
3 ->	44	-0.00	0.00
3 ->	45	-0.00	0.00
3 ->	46	-0.00	0.00
3 ->	47	-0.00	0.00
3 ->	48	0.00	0.00
3 ->	49	0.00	0.00
3 ->	50	-0.00	0.00
3 ->	51	0.00	0.00
3 ->	52	0.00	0.00
3 ->	53	-0.00	0.00
3 ->	54	0.00	0.00
3 ->	55	0.00	0.00
3 ->	56	0.00	0.00
3 ->	57	0.00	0.00
3 ->	58	0.00	0.00
3 ->	59	-0.00	0.00
3 ->	60	0.00	0.00
3 ->	61	0.00	0.00
3 ->	62	0.00	0.00
3 ->	63	0.00	0.00
3 ->	64	0.00	0.00
3 ->	65	0.00	0.00
3 ->	66	0.00	0.00
3 ->	67	0.00	0.00
3 ->	68	0.00	0.00
3 ->	69	-0.00	0.00
3 ->	70	0.00	0.00
3 ->	71	0.00	0.00
3 ->	72	-0.00	0.00
3 ->	73	-0.00	0.00
3 ->	74	0.00	0.00
3 ->	75	0.00	0.00
3 ->	76	-0.00	0.00
3 ->	77	-0.00	0.00
3 ->	78	-0.00	0.00
3 ->	79	0.00	0.00
3 ->	80	0.00	0.00
3 ->	81	-0.00	0.00
3 ->	82	0.00	0.00

3	->	83	0.00	0.00
3	->	84	0.00	0.00
3	->	85	0.00	0.00
3	->	86	0.00	0.00
3	->	87	0.00	0.00
3	->	88	0.00	0.00
3	->	89	0.00	0.00
3	->	90	-0.00	0.00
3	->	91	-0.00	0.00
3	->	92	-0.02	0.01
3	->	93	-0.02	0.01
3	->	94	-0.01	0.01
3	->	95	-0.01	0.01
3	->	96	-0.01	0.01
3	->	97	-0.03	0.04
3	->	98	-0.04	0.03
3	->	99	-0.01	0.00
3	->	100	-0.00	0.00
3	->	101	-0.00	0.00
3	->	102	-0.00	0.00
3	->	103	-0.00	0.00
3	->	104	-0.00	0.00
3	->	105	0.00	0.00
3	->	106	0.00	0.00
3	->	107	0.00	0.00
4	->	1	-0.13	0.03
4	->	2	-0.37	0.15
4	->	3	-9.83	0.42
4	->	4	22.51	0.73
4	->	5	-12.61	0.52
4	->	6	-0.38	0.13
4	->	7	0.04	0.05
4	->	8	-0.17	0.28
4	->	9	-0.01	0.01
4	->	10	-0.09	0.04
4	->	11	-0.03	0.02
4	->	12	-0.08	0.06
4	->	13	-2.13	0.41
4	->	14	-0.79	0.29
4	->	15	0.00	0.01
4	->	16	-0.03	0.01
4	->	17	-0.01	0.00
4	->	18	-0.00	0.00
4	->	19	-0.01	0.01
4	->	20	-1.19	0.31
4	->	21	-0.01	0.00
4	->	22	0.00	0.00
4	->	23	-0.09	0.03
4	->	24	-0.06	0.03
4	->	25	-0.01	0.00

4 ->	26	-0.00	0.00
4 ->	27	-0.06	0.01
4 ->	28	-0.00	0.00
4 ->	29	0.00	0.00
4 ->	30	-0.00	0.00
4 ->	31	0.00	0.00
4 ->	32	-0.00	0.00
4 ->	33	0.00	0.00
4 ->	34	0.00	0.00
4 ->	35	-0.00	0.00
4 ->	36	0.00	0.00
4 ->	37	0.00	0.00
4 ->	38	-0.00	0.00
4 ->	39	0.00	0.00
4 ->	40	0.00	0.00
4 ->	41	0.00	0.00
4 ->	42	0.00	0.00
4 ->	43	-0.00	0.00
4 ->	44	-0.00	0.00
4 ->	45	-0.00	0.00
4 ->	46	0.00	0.00
4 ->	47	0.00	0.00
4 ->	48	0.00	0.00
4 ->	49	-0.00	0.00
4 ->	50	0.00	0.00
4 ->	51	0.00	0.00
4 ->	52	0.00	0.00
4 ->	53	-0.00	0.00
4 ->	54	0.00	0.00
4 ->	55	0.00	0.00
4 ->	56	0.00	0.00
4 ->	57	0.00	0.00
4 ->	58	-0.00	0.00
4 ->	59	0.00	0.00
4 ->	60	0.00	0.00
4 ->	61	0.00	0.00
4 ->	62	0.00	0.00
4 ->	63	0.00	0.00
4 ->	64	0.00	0.00
4 ->	65	0.00	0.00
4 ->	66	0.00	0.00
4 ->	67	0.00	0.00
4 ->	68	0.00	0.00
4 ->	69	0.00	0.00
4 ->	70	0.00	0.00
4 ->	71	0.00	0.00
4 ->	72	0.00	0.00
4 ->	73	-0.00	0.00
4 ->	74	-0.00	0.00
4 ->	75	-0.00	0.00

4	->	76	0.00	0.00
4	->	77	0.00	0.00
4	->	78	0.00	0.00
4	->	79	0.00	0.00
4	->	80	0.00	0.00
4	->	81	0.00	0.00
4	->	82	0.00	0.00
4	->	83	0.00	0.00
4	->	84	0.00	0.00
4	->	85	0.00	0.00
4	->	86	0.00	0.00
4	->	87	0.00	0.00
4	->	88	-0.00	0.00
4	->	89	-0.00	0.00
4	->	90	-0.00	0.00
4	->	91	-0.01	0.00
4	->	92	-0.01	0.01
4	->	93	-0.00	0.00
4	->	94	-0.00	0.00
4	->	95	-0.00	0.00
4	->	96	0.00	0.00
4	->	97	-0.00	0.00
4	->	98	-0.00	0.00
4	->	99	-0.00	0.00
4	->	100	0.00	0.00
4	->	101	0.00	0.00
4	->	102	0.00	0.00
4	->	103	-0.00	0.00
4	->	104	-0.00	0.00
4	->	105	-0.00	0.00
4	->	106	-0.00	0.00
4	->	107	-0.00	0.00
5	->	1	-0.11	0.01
5	->	2	-0.01	0.00
5	->	3	-0.48	0.07
5	->	4	-12.67	0.52
5	->	5	-32.39	2.63
5	->	6	-1.33	0.58
5	->	7	-4.26	2.11
5	->	8	-0.06	0.31
5	->	9	0.02	0.10
5	->	10	1.23	0.25
5	->	11	0.08	0.01
5	->	12	-0.14	0.07
5	->	13	-0.35	0.07
5	->	14	-0.59	0.24
5	->	15	0.01	0.01
5	->	16	-0.04	0.01
5	->	17	-0.00	0.01
5	->	18	-0.01	0.00

5	->	19	0.01	0.01
5	->	20	-1.01	0.18
5	->	21	-0.01	0.01
5	->	22	-0.10	0.02
5	->	23	1.16	0.60
5	->	24	-0.50	0.28
5	->	25	-0.05	0.01
5	->	26	-0.05	0.02
5	->	27	1.40	0.35
5	->	28	-0.02	0.01
5	->	29	-0.00	0.00
5	->	30	-0.01	0.00
5	->	31	-0.02	0.00
5	->	32	-0.00	0.00
5	->	33	0.00	0.00
5	->	34	-0.00	0.00
5	->	35	0.01	0.00
5	->	36	-0.00	0.00
5	->	37	0.00	0.00
5	->	38	0.01	0.00
5	->	39	0.00	0.00
5	->	40	-0.01	0.00
5	->	41	-0.00	0.00
5	->	42	-0.00	0.00
5	->	43	-0.00	0.00
5	->	44	-0.03	0.00
5	->	45	-0.00	0.00
5	->	46	0.01	0.00
5	->	47	-0.00	0.00
5	->	48	-0.00	0.00
5	->	49	-0.00	0.00
5	->	50	-0.00	0.00
5	->	51	-0.00	0.00
5	->	52	-0.00	0.00
5	->	53	-0.00	0.00
5	->	54	-0.00	0.00
5	->	55	0.00	0.00
5	->	56	-0.00	0.00
5	->	57	-0.00	0.00
5	->	58	0.00	0.00
5	->	59	-0.00	0.00
5	->	60	-0.00	0.00
5	->	61	-0.00	0.00
5	->	62	0.00	0.00
5	->	63	0.00	0.00
5	->	64	0.00	0.00
5	->	65	0.00	0.00
5	->	66	0.00	0.00
5	->	67	0.00	0.00
5	->	68	-0.00	0.00

5	->	69	-0.00	0.00
5	->	70	-0.00	0.00
5	->	71	0.00	0.00
5	->	72	-0.01	0.00
5	->	73	-0.00	0.00
5	->	74	0.01	0.00
5	->	75	0.00	0.00
5	->	76	-0.00	0.00
5	->	77	-0.00	0.00
5	->	78	-0.01	0.00
5	->	79	-0.00	0.00
5	->	80	-0.00	0.00
5	->	81	-0.00	0.00
5	->	82	-0.00	0.00
5	->	83	-0.00	0.00
5	->	84	-0.00	0.00
5	->	85	-0.00	0.00
5	->	86	0.00	0.00
5	->	87	0.00	0.00
5	->	88	0.01	0.00
5	->	89	0.01	0.00
5	->	90	0.02	0.00
5	->	91	0.05	0.01
5	->	92	0.07	0.02
5	->	93	0.03	0.01
5	->	94	0.01	0.00
5	->	95	0.01	0.00
5	->	96	0.00	0.00
5	->	97	0.00	0.00
5	->	98	0.03	0.00
5	->	99	0.02	0.00
5	->	100	0.01	0.00
5	->	101	0.01	0.00
5	->	102	0.01	0.00
5	->	103	0.01	0.00
5	->	104	0.01	0.00
5	->	105	0.01	0.00
5	->	106	0.01	0.00
5	->	107	0.01	0.00
6	->	1	-0.00	0.00
6	->	2	-0.00	0.00
6	->	3	-0.03	0.01
6	->	4	-0.39	0.14
6	->	5	-1.43	0.57
6	->	6	0.77	0.70
6	->	7	-13.22	0.41
6	->	8	-0.41	0.19
6	->	9	-0.04	0.01
6	->	10	-0.03	0.01
6	->	11	-0.00	0.00

6 ->	12	-0.01	0.00
6 ->	13	-0.02	0.01
6 ->	14	-0.03	0.03
6 ->	15	-0.00	0.00
6 ->	16	-0.00	0.00
6 ->	17	-0.01	0.00
6 ->	18	-0.00	0.00
6 ->	19	-0.01	0.00
6 ->	20	-0.30	0.18
6 ->	21	-0.02	0.01
6 ->	22	-0.01	0.00
6 ->	23	-0.11	0.08
6 ->	24	-1.20	0.39
6 ->	25	-0.01	0.01
6 ->	26	-0.01	0.00
6 ->	27	-0.33	0.18
6 ->	28	-0.10	0.13
6 ->	29	-0.00	0.00
6 ->	30	-0.01	0.00
6 ->	31	0.00	0.00
6 ->	32	-0.00	0.00
6 ->	33	-0.00	0.00
6 ->	34	0.00	0.00
6 ->	35	-0.00	0.00
6 ->	36	0.00	0.00
6 ->	37	0.00	0.00
6 ->	38	-0.00	0.00
6 ->	39	-0.00	0.00
6 ->	40	0.00	0.00
6 ->	41	-0.00	0.00
6 ->	42	-0.00	0.00
6 ->	43	-0.00	0.00
6 ->	44	0.00	0.00
6 ->	45	0.00	0.00
6 ->	46	-0.00	0.00
6 ->	47	0.00	0.00
6 ->	48	0.00	0.00
6 ->	49	0.00	0.00
6 ->	50	0.00	0.00
6 ->	51	0.00	0.00
6 ->	52	0.00	0.00
6 ->	53	0.00	0.00
6 ->	54	0.00	0.00
6 ->	55	0.00	0.00
6 ->	56	0.00	0.00
6 ->	57	0.00	0.00
6 ->	58	-0.00	0.00
6 ->	59	0.00	0.00
6 ->	60	0.00	0.00
6 ->	61	0.00	0.00

6	->	62	0.00	0.00
6	->	63	-0.00	0.00
6	->	64	-0.00	0.00
6	->	65	0.00	0.00
6	->	66	0.00	0.00
6	->	67	0.00	0.00
6	->	68	0.00	0.00
6	->	69	0.00	0.00
6	->	70	0.00	0.00
6	->	71	0.00	0.00
6	->	72	0.00	0.00
6	->	73	0.00	0.00
6	->	74	-0.00	0.00
6	->	75	-0.00	0.00
6	->	76	0.00	0.00
6	->	77	0.00	0.00
6	->	78	0.00	0.00
6	->	79	0.00	0.00
6	->	80	0.00	0.00
6	->	81	0.00	0.00
6	->	82	0.00	0.00
6	->	83	0.00	0.00
6	->	84	0.00	0.00
6	->	85	0.00	0.00
6	->	86	-0.00	0.00
6	->	87	-0.00	0.00
6	->	88	-0.00	0.00
6	->	89	-0.00	0.00
6	->	90	-0.00	0.00
6	->	91	-0.00	0.00
6	->	92	-0.01	0.00
6	->	93	-0.00	0.00
6	->	94	-0.00	0.00
6	->	95	-0.00	0.00
6	->	96	-0.00	0.00
6	->	97	-0.00	0.00
6	->	98	-0.01	0.00
6	->	99	-0.00	0.00
6	->	100	-0.00	0.00
6	->	101	-0.00	0.00
6	->	102	-0.00	0.00
6	->	103	-0.00	0.00
6	->	104	-0.00	0.00
6	->	105	-0.00	0.00
6	->	106	-0.00	0.00
6	->	107	-0.00	0.00
7	->	1	-0.00	0.00
7	->	2	-0.00	0.00
7	->	3	0.00	0.01
7	->	4	0.04	0.05

7 ->	5	-4.35	2.12
7 ->	6	-13.35	0.41
7 ->	7	25.34	1.59
7 ->	8	-11.60	0.36
7 ->	9	-1.55	0.51
7 ->	10	-0.50	0.32
7 ->	11	-0.02	0.01
7 ->	12	-0.01	0.00
7 ->	13	-0.01	0.01
7 ->	14	-0.03	0.01
7 ->	15	-0.00	0.00
7 ->	16	-0.00	0.00
7 ->	17	-0.00	0.00
7 ->	18	-0.00	0.00
7 ->	19	0.00	0.00
7 ->	20	-0.02	0.01
7 ->	21	-0.00	0.00
7 ->	22	0.00	0.01
7 ->	23	-0.19	0.10
7 ->	24	-0.09	0.12
7 ->	25	-0.00	0.00
7 ->	26	-0.00	0.01
7 ->	27	-1.34	0.99
7 ->	28	-0.06	0.06
7 ->	29	-0.00	0.00
7 ->	30	-0.00	0.00
7 ->	31	-0.00	0.00
7 ->	32	-0.00	0.00
7 ->	33	-0.00	0.00
7 ->	34	0.00	0.00
7 ->	35	-0.00	0.00
7 ->	36	0.00	0.00
7 ->	37	0.00	0.00
7 ->	38	0.00	0.00
7 ->	39	0.00	0.00
7 ->	40	-0.00	0.00
7 ->	41	-0.00	0.00
7 ->	42	-0.00	0.00
7 ->	43	-0.00	0.00
7 ->	44	-0.00	0.00
7 ->	45	0.00	0.00
7 ->	46	-0.00	0.00
7 ->	47	0.00	0.00
7 ->	48	0.00	0.00
7 ->	49	0.00	0.00
7 ->	50	0.00	0.00
7 ->	51	0.00	0.00
7 ->	52	0.00	0.00
7 ->	53	0.00	0.00
7 ->	54	0.00	0.00

7 ->	55	0.00	0.00
7 ->	56	0.00	0.00
7 ->	57	0.00	0.00
7 ->	58	0.00	0.00
7 ->	59	0.00	0.00
7 ->	60	0.00	0.00
7 ->	61	0.00	0.00
7 ->	62	0.00	0.00
7 ->	63	0.00	0.00
7 ->	64	0.00	0.00
7 ->	65	0.00	0.00
7 ->	66	0.00	0.00
7 ->	67	0.00	0.00
7 ->	68	0.00	0.00
7 ->	69	0.00	0.00
7 ->	70	0.00	0.00
7 ->	71	0.00	0.00
7 ->	72	0.00	0.00
7 ->	73	0.00	0.00
7 ->	74	-0.00	0.00
7 ->	75	-0.00	0.00
7 ->	76	0.00	0.00
7 ->	77	0.00	0.00
7 ->	78	0.00	0.00
7 ->	79	0.00	0.00
7 ->	80	0.00	0.00
7 ->	81	0.00	0.00
7 ->	82	0.00	0.00
7 ->	83	0.00	0.00
7 ->	84	0.00	0.00
7 ->	85	0.00	0.00
7 ->	86	0.00	0.00
7 ->	87	0.00	0.00
7 ->	88	0.00	0.00
7 ->	89	0.00	0.00
7 ->	90	-0.00	0.00
7 ->	91	-0.00	0.00
7 ->	92	-0.00	0.00
7 ->	93	-0.00	0.00
7 ->	94	-0.00	0.00
7 ->	95	0.00	0.00
7 ->	96	-0.00	0.00
7 ->	97	-0.00	0.00
7 ->	98	-0.00	0.00
7 ->	99	-0.00	0.00
7 ->	100	-0.00	0.00
7 ->	101	-0.00	0.00
7 ->	102	-0.00	0.00
7 ->	103	-0.00	0.00
7 ->	104	0.00	0.00

7	->	105	0.00	0.00
7	->	106	0.00	0.00
7	->	107	0.00	0.00
8	->	1	-0.07	0.04
8	->	2	0.00	0.00
8	->	3	-0.01	0.02
8	->	4	-0.17	0.28
8	->	5	-0.07	0.32
8	->	6	-0.41	0.20
8	->	7	-11.62	0.36
8	->	8	-69.18	2.74
8	->	9	-18.43	0.62
8	->	10	-0.59	0.20
8	->	11	-0.03	0.15
8	->	12	-0.08	0.17
8	->	13	-0.84	1.85
8	->	14	-0.01	0.02
8	->	15	-0.00	0.00
8	->	16	-0.01	0.00
8	->	17	0.00	0.00
8	->	18	0.00	0.00
8	->	19	0.02	0.01
8	->	20	-0.01	0.01
8	->	21	-0.00	0.00
8	->	22	-0.01	0.00
8	->	23	-0.02	0.01
8	->	24	-0.01	0.00
8	->	25	-0.01	0.00
8	->	26	-0.00	0.00
8	->	27	-0.03	0.02
8	->	28	-0.00	0.00
8	->	29	-0.00	0.00
8	->	30	-0.00	0.00
8	->	31	-0.01	0.00
8	->	32	-0.00	0.00
8	->	33	0.00	0.00
8	->	34	-0.00	0.00
8	->	35	0.00	0.00
8	->	36	-0.00	0.00
8	->	37	0.00	0.00
8	->	38	0.00	0.00
8	->	39	0.00	0.00
8	->	40	-0.01	0.00
8	->	41	-0.00	0.00
8	->	42	-0.00	0.00
8	->	43	-0.00	0.00
8	->	44	-0.01	0.00
8	->	45	-0.00	0.00
8	->	46	0.01	0.00
8	->	47	-0.00	0.00

8	->	48	-0.00	0.00
8	->	49	-0.00	0.00
8	->	50	-0.00	0.00
8	->	51	-0.00	0.00
8	->	52	-0.00	0.00
8	->	53	-0.00	0.00
8	->	54	-0.00	0.00
8	->	55	0.00	0.00
8	->	56	-0.00	0.00
8	->	57	-0.00	0.00
8	->	58	0.00	0.00
8	->	59	-0.00	0.00
8	->	60	0.00	0.00
8	->	61	0.00	0.00
8	->	62	0.00	0.00
8	->	63	0.00	0.00
8	->	64	0.00	0.00
8	->	65	0.00	0.00
8	->	66	0.00	0.00
8	->	67	-0.00	0.00
8	->	68	-0.00	0.00
8	->	69	-0.00	0.00
8	->	70	-0.00	0.00
8	->	71	0.00	0.00
8	->	72	-0.00	0.00
8	->	73	-0.00	0.00
8	->	74	0.00	0.00
8	->	75	0.00	0.00
8	->	76	-0.00	0.00
8	->	77	-0.00	0.00
8	->	78	-0.00	0.00
8	->	79	-0.00	0.00
8	->	80	-0.00	0.00
8	->	81	-0.00	0.00
8	->	82	-0.00	0.00
8	->	83	0.00	0.00
8	->	84	-0.00	0.00
8	->	85	-0.00	0.00
8	->	86	0.00	0.00
8	->	87	0.00	0.00
8	->	88	0.00	0.00
8	->	89	0.00	0.00
8	->	90	0.01	0.00
8	->	91	0.02	0.00
8	->	92	0.03	0.01
8	->	93	0.02	0.01
8	->	94	0.01	0.00
8	->	95	0.00	0.00
8	->	96	0.00	0.00
8	->	97	0.00	0.00

8	->	98	0.01	0.00
8	->	99	0.01	0.00
8	->	100	0.00	0.00
8	->	101	0.00	0.00
8	->	102	0.00	0.00
8	->	103	0.00	0.00
8	->	104	0.00	0.00
8	->	105	0.01	0.00
8	->	106	0.01	0.00
8	->	107	0.00	0.00
9	->	1	-0.00	0.00
9	->	2	-0.00	0.00
9	->	3	-0.00	0.00
9	->	4	-0.01	0.01
9	->	5	0.02	0.10
9	->	6	-0.04	0.01
9	->	7	-1.55	0.51
9	->	8	-18.51	0.63
9	->	9	16.27	1.53
9	->	10	-9.62	0.80
9	->	11	-0.36	0.15
9	->	12	-0.04	0.03
9	->	13	-0.02	0.01
9	->	14	-0.01	0.00
9	->	15	-0.00	0.00
9	->	16	-0.00	0.00
9	->	17	0.00	0.00
9	->	18	-0.00	0.00
9	->	19	-0.00	0.00
9	->	20	-0.00	0.00
9	->	21	-0.00	0.00
9	->	22	-0.00	0.00
9	->	23	-0.01	0.04
9	->	24	-0.00	0.00
9	->	25	-0.00	0.00
9	->	26	-0.01	0.01
9	->	27	-0.08	0.08
9	->	28	-0.00	0.00
9	->	29	-0.00	0.00
9	->	30	-0.00	0.00
9	->	31	0.00	0.00
9	->	32	0.00	0.00
9	->	33	0.00	0.00
9	->	34	0.00	0.00
9	->	35	-0.00	0.00
9	->	36	0.00	0.00
9	->	37	0.00	0.00
9	->	38	-0.00	0.00
9	->	39	0.00	0.00
9	->	40	0.00	0.00

9	->	41	0.00	0.00
9	->	42	-0.00	0.00
9	->	43	-0.00	0.00
9	->	44	-0.00	0.00
9	->	45	0.00	0.00
9	->	46	0.00	0.00
9	->	47	0.00	0.00
9	->	48	0.00	0.00
9	->	49	0.00	0.00
9	->	50	-0.00	0.00
9	->	51	0.00	0.00
9	->	52	0.00	0.00
9	->	53	-0.00	0.00
9	->	54	0.00	0.00
9	->	55	-0.00	0.00
9	->	56	0.00	0.00
9	->	57	0.00	0.00
9	->	58	0.00	0.00
9	->	59	0.00	0.00
9	->	60	0.00	0.00
9	->	61	0.00	0.00
9	->	62	0.00	0.00
9	->	63	0.00	0.00
9	->	64	0.00	0.00
9	->	65	0.00	0.00
9	->	66	0.00	0.00
9	->	67	0.00	0.00
9	->	68	-0.00	0.00
9	->	69	-0.00	0.00
9	->	70	0.00	0.00
9	->	71	0.00	0.00
9	->	72	-0.00	0.00
9	->	73	0.00	0.00
9	->	74	0.00	0.00
9	->	75	0.00	0.00
9	->	76	-0.00	0.00
9	->	77	-0.00	0.00
9	->	78	-0.00	0.00
9	->	79	-0.00	0.00
9	->	80	0.00	0.00
9	->	81	-0.00	0.00
9	->	82	0.00	0.00
9	->	83	0.00	0.00
9	->	84	0.00	0.00
9	->	85	0.00	0.00
9	->	86	0.00	0.00
9	->	87	0.00	0.00
9	->	88	0.00	0.00
9	->	89	-0.00	0.00
9	->	90	-0.00	0.00

9	->	91	-0.00	0.00
9	->	92	-0.00	0.00
9	->	93	-0.00	0.00
9	->	94	0.00	0.00
9	->	95	0.00	0.00
9	->	96	0.00	0.00
9	->	97	-0.00	0.00
9	->	98	-0.00	0.00
9	->	99	-0.00	0.00
9	->	100	-0.00	0.00
9	->	101	0.00	0.00
9	->	102	0.00	0.00
9	->	103	0.00	0.00
9	->	104	0.00	0.00
9	->	105	0.00	0.00
9	->	106	0.00	0.00
9	->	107	0.00	0.00
10	->	1	-0.09	0.01
10	->	2	-0.00	0.00
10	->	3	-0.01	0.01
10	->	4	-0.09	0.04
10	->	5	1.21	0.26
10	->	6	-0.03	0.01
10	->	7	-0.51	0.32
10	->	8	-0.59	0.20
10	->	9	-9.69	0.80
10	->	10	-39.93	2.69
10	->	11	-14.20	0.30
10	->	12	-1.00	0.31
10	->	13	-0.20	0.08
10	->	14	-0.09	0.12
10	->	15	-0.00	0.00
10	->	16	-0.03	0.00
10	->	17	0.00	0.00
10	->	18	0.00	0.00
10	->	19	0.03	0.00
10	->	20	-0.01	0.01
10	->	21	-0.00	0.00
10	->	22	-0.06	0.01
10	->	23	1.80	0.89
10	->	24	0.02	0.01
10	->	25	-0.03	0.00
10	->	26	-0.04	0.01
10	->	27	1.36	0.26
10	->	28	-0.01	0.00
10	->	29	-0.00	0.00
10	->	30	-0.00	0.00
10	->	31	-0.02	0.01
10	->	32	-0.00	0.00
10	->	33	0.00	0.00

10 ->	34	-0.00	0.00
10 ->	35	0.01	0.00
10 ->	36	-0.00	0.00
10 ->	37	0.00	0.00
10 ->	38	0.01	0.00
10 ->	39	0.00	0.00
10 ->	40	-0.01	0.00
10 ->	41	-0.00	0.00
10 ->	42	-0.00	0.00
10 ->	43	-0.00	0.00
10 ->	44	-0.03	0.00
10 ->	45	-0.00	0.00
10 ->	46	0.02	0.00
10 ->	47	-0.00	0.00
10 ->	48	-0.00	0.00
10 ->	49	-0.00	0.00
10 ->	50	-0.00	0.00
10 ->	51	-0.00	0.00
10 ->	52	-0.00	0.00
10 ->	53	-0.01	0.00
10 ->	54	-0.00	0.00
10 ->	55	0.00	0.00
10 ->	56	-0.00	0.00
10 ->	57	-0.00	0.00
10 ->	58	0.00	0.00
10 ->	59	-0.00	0.00
10 ->	60	-0.00	0.00
10 ->	61	-0.00	0.00
10 ->	62	0.00	0.00
10 ->	63	0.00	0.00
10 ->	64	0.00	0.00
10 ->	65	0.00	0.00
10 ->	66	0.00	0.00
10 ->	67	0.00	0.00
10 ->	68	-0.00	0.00
10 ->	69	-0.00	0.00
10 ->	70	-0.00	0.00
10 ->	71	0.00	0.00
10 ->	72	-0.01	0.00
10 ->	73	-0.00	0.00
10 ->	74	0.01	0.00
10 ->	75	0.01	0.00
10 ->	76	-0.00	0.00
10 ->	77	-0.01	0.00
10 ->	78	-0.01	0.00
10 ->	79	-0.00	0.00
10 ->	80	-0.00	0.00
10 ->	81	-0.00	0.00
10 ->	82	-0.00	0.00
10 ->	83	-0.00	0.00

10	->	84	-0.00	0.00
10	->	85	-0.00	0.00
10	->	86	0.00	0.00
10	->	87	0.00	0.00
10	->	88	0.01	0.00
10	->	89	0.01	0.00
10	->	90	0.03	0.00
10	->	91	0.07	0.02
10	->	92	0.09	0.04
10	->	93	0.03	0.00
10	->	94	0.01	0.00
10	->	95	0.01	0.00
10	->	96	0.00	0.00
10	->	97	0.00	0.00
10	->	98	0.02	0.00
10	->	99	0.02	0.00
10	->	100	0.01	0.00
10	->	101	0.00	0.00
10	->	102	0.01	0.00
10	->	103	0.01	0.00
10	->	104	0.01	0.00
10	->	105	0.02	0.00
10	->	106	0.01	0.00
10	->	107	0.01	0.00
11	->	1	-0.15	0.02
11	->	2	-0.00	0.00
11	->	3	-0.01	0.00
11	->	4	-0.03	0.02
11	->	5	0.08	0.01
11	->	6	-0.00	0.00
11	->	7	-0.02	0.01
11	->	8	-0.03	0.16
11	->	9	-0.37	0.16
11	->	10	-14.32	0.29
11	->	11	-36.19	1.73
11	->	12	-8.44	0.51
11	->	13	-0.64	0.38
11	->	14	-0.04	0.02
11	->	15	-0.01	0.00
11	->	16	-0.02	0.00
11	->	17	0.00	0.00
11	->	18	0.00	0.00
11	->	19	0.03	0.01
11	->	20	-0.00	0.00
11	->	21	-0.00	0.00
11	->	22	-0.02	0.01
11	->	23	0.00	0.01
11	->	24	-0.00	0.00
11	->	25	-0.01	0.00
11	->	26	-0.00	0.00

11	->	27	0.01	0.00
11	->	28	-0.00	0.00
11	->	29	-0.00	0.00
11	->	30	-0.00	0.00
11	->	31	-0.01	0.00
11	->	32	-0.00	0.00
11	->	33	0.00	0.00
11	->	34	-0.00	0.00
11	->	35	0.00	0.00
11	->	36	-0.00	0.00
11	->	37	0.00	0.00
11	->	38	0.00	0.00
11	->	39	0.00	0.00
11	->	40	-0.01	0.00
11	->	41	0.00	0.00
11	->	42	-0.00	0.00
11	->	43	-0.00	0.00
11	->	44	-0.02	0.01
11	->	45	-0.00	0.00
11	->	46	0.01	0.00
11	->	47	-0.00	0.00
11	->	48	-0.00	0.00
11	->	49	-0.00	0.00
11	->	50	-0.00	0.00
11	->	51	-0.00	0.00
11	->	52	-0.00	0.00
11	->	53	-0.00	0.00
11	->	54	-0.00	0.00
11	->	55	-0.00	0.00
11	->	56	-0.00	0.00
11	->	57	-0.00	0.00
11	->	58	0.00	0.00
11	->	59	-0.00	0.00
11	->	60	-0.00	0.00
11	->	61	-0.00	0.00
11	->	62	0.00	0.00
11	->	63	0.00	0.00
11	->	64	0.00	0.00
11	->	65	0.00	0.00
11	->	66	0.00	0.00
11	->	67	-0.00	0.00
11	->	68	-0.00	0.00
11	->	69	-0.00	0.00
11	->	70	-0.00	0.00
11	->	71	-0.00	0.00
11	->	72	-0.01	0.00
11	->	73	-0.00	0.00
11	->	74	0.01	0.00
11	->	75	0.00	0.00
11	->	76	-0.00	0.00

11	->	77	-0.01	0.00
11	->	78	-0.01	0.00
11	->	79	-0.00	0.00
11	->	80	-0.00	0.00
11	->	81	-0.01	0.00
11	->	82	-0.00	0.00
11	->	83	-0.00	0.00
11	->	84	-0.00	0.00
11	->	85	-0.00	0.00
11	->	86	0.00	0.00
11	->	87	0.00	0.00
11	->	88	0.01	0.00
11	->	89	0.01	0.00
11	->	90	0.02	0.01
11	->	91	0.03	0.03
11	->	92	0.04	0.04
11	->	93	0.04	0.01
11	->	94	0.01	0.00
11	->	95	0.01	0.00
11	->	96	0.00	0.00
11	->	97	0.00	0.00
11	->	98	0.01	0.00
11	->	99	0.01	0.00
11	->	100	0.00	0.00
11	->	101	0.00	0.00
11	->	102	0.01	0.00
11	->	103	0.01	0.00
11	->	104	0.01	0.00
11	->	105	0.02	0.01
11	->	106	0.02	0.01
11	->	107	0.01	0.00
12	->	1	-0.59	1.33
12	->	2	-0.02	0.01
12	->	3	-0.17	0.06
12	->	4	-0.08	0.06
12	->	5	-0.14	0.06
12	->	6	-0.01	0.00
12	->	7	-0.01	0.00
12	->	8	-0.08	0.17
12	->	9	-0.04	0.03
12	->	10	-1.00	0.31
12	->	11	-8.56	0.52
12	->	12	-108.77	3.13
12	->	13	-10.01	1.11
12	->	14	-1.37	0.65
12	->	15	-0.28	0.30
12	->	16	0.05	0.03
12	->	17	-0.01	0.01
12	->	18	0.01	0.01
12	->	19	-0.15	0.11

12	->	20	-0.04	0.01
12	->	21	-0.00	0.00
12	->	22	0.06	0.02
12	->	23	-0.07	0.03
12	->	24	-0.01	0.00
12	->	25	0.02	0.00
12	->	26	-0.00	0.00
12	->	27	-0.02	0.01
12	->	28	0.00	0.00
12	->	29	0.00	0.00
12	->	30	-0.00	0.00
12	->	31	0.01	0.00
12	->	32	0.00	0.00
12	->	33	-0.00	0.00
12	->	34	0.00	0.00
12	->	35	-0.01	0.00
12	->	36	0.00	0.00
12	->	37	-0.00	0.00
12	->	38	-0.01	0.00
12	->	39	-0.00	0.00
12	->	40	0.01	0.00
12	->	41	0.00	0.00
12	->	42	0.00	0.00
12	->	43	0.00	0.00
12	->	44	0.04	0.01
12	->	45	0.00	0.00
12	->	46	-0.03	0.00
12	->	47	0.00	0.00
12	->	48	0.00	0.00
12	->	49	0.00	0.00
12	->	50	0.00	0.00
12	->	51	0.00	0.00
12	->	52	0.00	0.00
12	->	53	0.01	0.00
12	->	54	0.00	0.00
12	->	55	0.00	0.00
12	->	56	0.00	0.00
12	->	57	0.00	0.00
12	->	58	-0.00	0.00
12	->	59	0.00	0.00
12	->	60	0.00	0.00
12	->	61	0.00	0.00
12	->	62	-0.00	0.00
12	->	63	-0.00	0.00
12	->	64	-0.00	0.00
12	->	65	-0.00	0.00
12	->	66	-0.00	0.00
12	->	67	0.00	0.00
12	->	68	0.00	0.00
12	->	69	0.00	0.00

12 ->	70	0.00	0.00
12 ->	71	-0.00	0.00
12 ->	72	0.02	0.00
12 ->	73	0.00	0.00
12 ->	74	-0.02	0.00
12 ->	75	-0.01	0.00
12 ->	76	0.01	0.00
12 ->	77	0.02	0.01
12 ->	78	0.02	0.00
12 ->	79	0.00	0.00
12 ->	80	0.00	0.00
12 ->	81	0.02	0.00
12 ->	82	0.00	0.00
12 ->	83	0.00	0.00
12 ->	84	0.00	0.00
12 ->	85	0.00	0.00
12 ->	86	-0.00	0.00
12 ->	87	-0.01	0.00
12 ->	88	-0.01	0.00
12 ->	89	-0.01	0.00
12 ->	90	-0.06	0.04
12 ->	91	-1.67	1.73
12 ->	92	-6.63	2.45
12 ->	93	-2.61	3.36
12 ->	94	-0.05	0.01
12 ->	95	-0.02	0.00
12 ->	96	-0.01	0.00
12 ->	97	-0.00	0.00
12 ->	98	-0.02	0.00
12 ->	99	-0.02	0.00
12 ->	100	-0.01	0.00
12 ->	101	-0.01	0.00
12 ->	102	-0.02	0.01
12 ->	103	-0.04	0.01
12 ->	104	-0.05	0.02
12 ->	105	-0.06	0.02
12 ->	106	-0.04	0.01
12 ->	107	-0.02	0.01
13 ->	1	-5.35	1.37
13 ->	2	-1.83	0.86
13 ->	3	-3.31	0.81
13 ->	4	-2.15	0.41
13 ->	5	-0.35	0.07
13 ->	6	-0.02	0.01
13 ->	7	-0.01	0.01
13 ->	8	-0.86	1.88
13 ->	9	-0.02	0.01
13 ->	10	-0.21	0.09
13 ->	11	-0.68	0.40
13 ->	12	-10.10	1.10

13	->	13	-107.00	2.51
13	->	14	-12.63	0.38
13	->	15	-0.56	0.10
13	->	16	0.02	0.03
13	->	17	-0.03	0.01
13	->	18	0.01	0.01
13	->	19	-0.06	0.02
13	->	20	-0.07	0.02
13	->	21	-0.00	0.00
13	->	22	0.04	0.01
13	->	23	-0.04	0.01
13	->	24	-0.01	0.00
13	->	25	0.02	0.00
13	->	26	-0.00	0.00
13	->	27	-0.02	0.01
13	->	28	0.00	0.00
13	->	29	0.00	0.00
13	->	30	0.00	0.00
13	->	31	0.01	0.00
13	->	32	0.00	0.00
13	->	33	-0.00	0.00
13	->	34	0.00	0.00
13	->	35	-0.00	0.00
13	->	36	0.00	0.00
13	->	37	-0.00	0.00
13	->	38	-0.00	0.00
13	->	39	-0.00	0.00
13	->	40	0.00	0.00
13	->	41	0.00	0.00
13	->	42	0.00	0.00
13	->	43	-0.00	0.00
13	->	44	0.01	0.00
13	->	45	0.00	0.00
13	->	46	-0.01	0.00
13	->	47	0.00	0.00
13	->	48	0.00	0.00
13	->	49	0.00	0.00
13	->	50	0.00	0.00
13	->	51	0.00	0.00
13	->	52	0.00	0.00
13	->	53	0.00	0.00
13	->	54	0.00	0.00
13	->	55	0.00	0.00
13	->	56	0.00	0.00
13	->	57	0.00	0.00
13	->	58	-0.00	0.00
13	->	59	0.00	0.00
13	->	60	0.00	0.00
13	->	61	0.00	0.00
13	->	62	-0.00	0.00

13	->	63	-0.00	0.00
13	->	64	-0.00	0.00
13	->	65	-0.00	0.00
13	->	66	-0.00	0.00
13	->	67	0.00	0.00
13	->	68	0.00	0.00
13	->	69	0.00	0.00
13	->	70	0.00	0.00
13	->	71	-0.00	0.00
13	->	72	0.01	0.00
13	->	73	0.00	0.00
13	->	74	-0.00	0.00
13	->	75	-0.00	0.00
13	->	76	0.00	0.00
13	->	77	0.00	0.00
13	->	78	0.00	0.00
13	->	79	0.00	0.00
13	->	80	0.00	0.00
13	->	81	0.00	0.00
13	->	82	0.00	0.00
13	->	83	0.00	0.00
13	->	84	0.00	0.00
13	->	85	0.00	0.00
13	->	86	-0.00	0.00
13	->	87	-0.00	0.00
13	->	88	-0.00	0.00
13	->	89	-0.00	0.00
13	->	90	-0.01	0.00
13	->	91	-0.05	0.01
13	->	92	-0.16	0.05
13	->	93	-0.14	0.15
13	->	94	-0.03	0.01
13	->	95	-0.01	0.00
13	->	96	-0.00	0.00
13	->	97	-0.01	0.00
13	->	98	-0.02	0.00
13	->	99	-0.01	0.00
13	->	100	-0.01	0.00
13	->	101	-0.00	0.00
13	->	102	-0.01	0.00
13	->	103	-0.01	0.00
13	->	104	-0.01	0.00
13	->	105	-0.01	0.00
13	->	106	-0.01	0.00
13	->	107	-0.00	0.00
14	->	1	-2.90	0.73
14	->	2	-0.87	0.22
14	->	3	-3.51	0.67
14	->	4	-0.79	0.29
14	->	5	-0.57	0.24

14 ->	6	-0.03	0.03
14 ->	7	-0.03	0.01
14 ->	8	-0.01	0.02
14 ->	9	-0.01	0.00
14 ->	10	-0.10	0.12
14 ->	11	-0.04	0.02
14 ->	12	-1.40	0.63
14 ->	13	-12.69	0.38
14 ->	14	29.05	1.23
14 ->	15	-16.92	0.47
14 ->	16	-0.86	0.19
14 ->	17	-0.19	0.03
14 ->	18	-0.06	0.02
14 ->	19	-1.86	0.31
14 ->	20	-2.42	0.46
14 ->	21	-0.07	0.01
14 ->	22	-0.20	0.05
14 ->	23	-2.08	0.29
14 ->	24	-0.08	0.02
14 ->	25	-0.02	0.00
14 ->	26	-0.03	0.01
14 ->	27	-0.03	0.01
14 ->	28	-0.00	0.00
14 ->	29	-0.00	0.00
14 ->	30	-0.00	0.00
14 ->	31	-0.00	0.00
14 ->	32	-0.00	0.00
14 ->	33	-0.00	0.00
14 ->	34	-0.00	0.00
14 ->	35	-0.00	0.00
14 ->	36	0.00	0.00
14 ->	37	-0.00	0.00
14 ->	38	-0.00	0.00
14 ->	39	-0.00	0.00
14 ->	40	0.00	0.00
14 ->	41	-0.00	0.00
14 ->	42	-0.00	0.00
14 ->	43	-0.01	0.00
14 ->	44	-0.01	0.00
14 ->	45	-0.00	0.00
14 ->	46	-0.00	0.00
14 ->	47	-0.00	0.00
14 ->	48	0.00	0.00
14 ->	49	0.00	0.00
14 ->	50	0.00	0.00
14 ->	51	0.00	0.00
14 ->	52	0.00	0.00
14 ->	53	0.00	0.00
14 ->	54	0.00	0.00
14 ->	55	0.00	0.00

14	->	56	0.00	0.00
14	->	57	0.00	0.00
14	->	58	-0.00	0.00
14	->	59	0.00	0.00
14	->	60	0.00	0.00
14	->	61	0.00	0.00
14	->	62	-0.00	0.00
14	->	63	-0.00	0.00
14	->	64	-0.00	0.00
14	->	65	0.00	0.00
14	->	66	-0.00	0.00
14	->	67	0.00	0.00
14	->	68	0.00	0.00
14	->	69	0.00	0.00
14	->	70	0.00	0.00
14	->	71	0.00	0.00
14	->	72	0.00	0.00
14	->	73	0.00	0.00
14	->	74	-0.00	0.00
14	->	75	-0.00	0.00
14	->	76	0.00	0.00
14	->	77	0.00	0.00
14	->	78	0.00	0.00
14	->	79	0.00	0.00
14	->	80	0.00	0.00
14	->	81	0.00	0.00
14	->	82	0.00	0.00
14	->	83	0.00	0.00
14	->	84	0.00	0.00
14	->	85	0.00	0.00
14	->	86	-0.00	0.00
14	->	87	-0.00	0.00
14	->	88	-0.00	0.00
14	->	89	-0.00	0.00
14	->	90	-0.01	0.00
14	->	91	-0.07	0.04
14	->	92	-1.69	0.31
14	->	93	-1.12	0.79
14	->	94	-0.04	0.04
14	->	95	-0.00	0.00
14	->	96	-0.00	0.00
14	->	97	-0.01	0.00
14	->	98	-0.01	0.00
14	->	99	-0.01	0.00
14	->	100	-0.00	0.00
14	->	101	-0.00	0.00
14	->	102	-0.00	0.00
14	->	103	-0.00	0.00
14	->	104	-0.00	0.00
14	->	105	-0.00	0.00

14 ->	106	-0.00	0.00
14 ->	107	-0.00	0.00
15 ->	1	-3.56	1.11
15 ->	2	-0.83	0.33
15 ->	3	-1.11	0.30
15 ->	4	0.00	0.01
15 ->	5	0.01	0.01
15 ->	6	-0.00	0.00
15 ->	7	-0.00	0.00
15 ->	8	-0.00	0.00
15 ->	9	-0.00	0.00
15 ->	10	-0.00	0.00
15 ->	11	-0.01	0.00
15 ->	12	-0.28	0.30
15 ->	13	-0.56	0.10
15 ->	14	-16.93	0.47
15 ->	15	21.91	2.18
15 ->	16	-6.24	0.58
15 ->	17	-0.27	0.04
15 ->	18	-0.01	0.02
15 ->	19	-0.57	0.35
15 ->	20	-0.06	0.02
15 ->	21	-0.01	0.00
15 ->	22	-0.01	0.01
15 ->	23	-0.01	0.00
15 ->	24	-0.00	0.00
15 ->	25	0.00	0.00
15 ->	26	-0.00	0.00
15 ->	27	0.00	0.00
15 ->	28	0.00	0.00
15 ->	29	0.00	0.00
15 ->	30	-0.00	0.00
15 ->	31	0.00	0.00
15 ->	32	0.00	0.00
15 ->	33	0.00	0.00
15 ->	34	0.00	0.00
15 ->	35	-0.00	0.00
15 ->	36	0.00	0.00
15 ->	37	-0.00	0.00
15 ->	38	-0.00	0.00
15 ->	39	0.00	0.00
15 ->	40	0.00	0.00
15 ->	41	0.00	0.00
15 ->	42	0.00	0.00
15 ->	43	-0.00	0.00
15 ->	44	0.00	0.00
15 ->	45	0.00	0.00
15 ->	46	-0.00	0.00
15 ->	47	-0.00	0.00
15 ->	48	0.00	0.00

15	->	49	0.00	0.00
15	->	50	0.00	0.00
15	->	51	0.00	0.00
15	->	52	0.00	0.00
15	->	53	0.00	0.00
15	->	54	0.00	0.00
15	->	55	0.00	0.00
15	->	56	0.00	0.00
15	->	57	0.00	0.00
15	->	58	-0.00	0.00
15	->	59	0.00	0.00
15	->	60	0.00	0.00
15	->	61	0.00	0.00
15	->	62	0.00	0.00
15	->	63	-0.00	0.00
15	->	64	-0.00	0.00
15	->	65	0.00	0.00
15	->	66	-0.00	0.00
15	->	67	0.00	0.00
15	->	68	0.00	0.00
15	->	69	0.00	0.00
15	->	70	0.00	0.00
15	->	71	-0.00	0.00
15	->	72	0.00	0.00
15	->	73	0.00	0.00
15	->	74	-0.00	0.00
15	->	75	-0.00	0.00
15	->	76	0.00	0.00
15	->	77	0.00	0.00
15	->	78	0.00	0.00
15	->	79	0.00	0.00
15	->	80	0.00	0.00
15	->	81	0.00	0.00
15	->	82	0.00	0.00
15	->	83	0.00	0.00
15	->	84	0.00	0.00
15	->	85	0.00	0.00
15	->	86	-0.00	0.00
15	->	87	-0.00	0.00
15	->	88	-0.00	0.00
15	->	89	-0.00	0.00
15	->	90	-0.00	0.00
15	->	91	0.00	0.01
15	->	92	-0.17	0.19
15	->	93	-3.26	2.64
15	->	94	-0.46	1.00
15	->	95	-0.01	0.01
15	->	96	-0.01	0.00
15	->	97	-0.00	0.00
15	->	98	-0.01	0.01

15	->	99	-0.01	0.00
15	->	100	-0.00	0.00
15	->	101	-0.00	0.00
15	->	102	-0.00	0.00
15	->	103	-0.01	0.00
15	->	104	-0.00	0.00
15	->	105	-0.00	0.00
15	->	106	-0.00	0.00
15	->	107	-0.00	0.00
16	->	1	0.03	0.08
16	->	2	-0.14	0.08
16	->	3	-2.35	0.68
16	->	4	-0.03	0.01
16	->	5	-0.04	0.01
16	->	6	-0.00	0.00
16	->	7	-0.00	0.00
16	->	8	-0.01	0.00
16	->	9	-0.00	0.00
16	->	10	-0.03	0.00
16	->	11	-0.02	0.00
16	->	12	0.05	0.03
16	->	13	0.02	0.03
16	->	14	-0.86	0.19
16	->	15	-6.36	0.59
16	->	16	-95.09	3.69
16	->	17	-21.80	0.44
16	->	18	-1.19	0.47
16	->	19	-14.33	1.52
16	->	20	-1.36	0.46
16	->	21	-0.01	0.07
16	->	22	1.24	0.81
16	->	23	-0.01	0.01
16	->	24	-0.00	0.01
16	->	25	0.08	0.02
16	->	26	-0.00	0.00
16	->	27	-0.00	0.00
16	->	28	0.00	0.00
16	->	29	0.00	0.00
16	->	30	-0.00	0.00
16	->	31	0.01	0.00
16	->	32	0.00	0.00
16	->	33	-0.00	0.00
16	->	34	-0.00	0.00
16	->	35	-0.01	0.00
16	->	36	0.01	0.00
16	->	37	-0.00	0.00
16	->	38	-0.01	0.00
16	->	39	-0.00	0.00
16	->	40	0.01	0.00
16	->	41	0.00	0.00

16 ->	42	0.00	0.00
16 ->	43	-0.00	0.00
16 ->	44	0.28	0.13
16 ->	45	-0.02	0.01
16 ->	46	-0.14	0.07
16 ->	47	-0.01	0.01
16 ->	48	0.00	0.00
16 ->	49	0.00	0.00
16 ->	50	0.00	0.00
16 ->	51	0.00	0.00
16 ->	52	0.00	0.00
16 ->	53	0.02	0.00
16 ->	54	0.00	0.00
16 ->	55	0.00	0.00
16 ->	56	0.00	0.00
16 ->	57	0.00	0.00
16 ->	58	-0.01	0.00
16 ->	59	0.01	0.00
16 ->	60	0.00	0.00
16 ->	61	0.00	0.00
16 ->	62	-0.00	0.00
16 ->	63	-0.01	0.00
16 ->	64	-0.00	0.00
16 ->	65	-0.00	0.00
16 ->	66	-0.00	0.00
16 ->	67	0.00	0.00
16 ->	68	0.00	0.00
16 ->	69	0.01	0.00
16 ->	70	0.00	0.00
16 ->	71	-0.00	0.00
16 ->	72	0.02	0.00
16 ->	73	0.00	0.00
16 ->	74	-0.03	0.00
16 ->	75	-0.01	0.00
16 ->	76	0.02	0.01
16 ->	77	0.02	0.00
16 ->	78	0.01	0.00
16 ->	79	0.00	0.00
16 ->	80	0.00	0.00
16 ->	81	0.01	0.00
16 ->	82	0.00	0.00
16 ->	83	0.00	0.00
16 ->	84	0.00	0.00
16 ->	85	0.00	0.00
16 ->	86	-0.00	0.00
16 ->	87	-0.01	0.00
16 ->	88	-0.01	0.00
16 ->	89	-0.02	0.00
16 ->	90	-0.03	0.00
16 ->	91	-0.07	0.02

16 ->	92	-0.27	0.13
16 ->	93	-4.02	1.26
16 ->	94	-2.91	2.15
16 ->	95	-0.45	0.65
16 ->	96	-0.13	0.09
16 ->	97	-0.06	0.02
16 ->	98	-0.52	0.24
16 ->	99	-2.94	1.65
16 ->	100	-0.66	0.36
16 ->	101	-0.05	0.06
16 ->	102	-0.10	0.04
16 ->	103	-0.06	0.02
16 ->	104	-0.03	0.00
16 ->	105	-0.02	0.00
16 ->	106	-0.01	0.00
16 ->	107	-0.01	0.00
17 ->	1	-0.05	0.01
17 ->	2	-0.04	0.01
17 ->	3	-1.70	0.63
17 ->	4	-0.01	0.00
17 ->	5	-0.00	0.01
17 ->	6	-0.01	0.00
17 ->	7	-0.00	0.00
17 ->	8	0.00	0.00
17 ->	9	0.00	0.00
17 ->	10	0.00	0.00
17 ->	11	0.00	0.00
17 ->	12	-0.01	0.01
17 ->	13	-0.03	0.01
17 ->	14	-0.19	0.03
17 ->	15	-0.27	0.04
17 ->	16	-21.81	0.44
17 ->	17	20.25	2.33
17 ->	18	-9.45	0.42
17 ->	19	-0.65	0.17
17 ->	20	-2.35	0.32
17 ->	21	-2.61	1.36
17 ->	22	-0.11	0.07
17 ->	23	-0.07	0.04
17 ->	24	-0.07	0.03
17 ->	25	-0.02	0.01
17 ->	26	-0.00	0.00
17 ->	27	-0.00	0.00
17 ->	28	-0.00	0.00
17 ->	29	-0.00	0.00
17 ->	30	-0.00	0.00
17 ->	31	-0.00	0.00
17 ->	32	-0.00	0.00
17 ->	33	-0.00	0.00
17 ->	34	-0.00	0.00

17	->	35	0.00	0.00
17	->	36	-0.00	0.00
17	->	37	0.00	0.00
17	->	38	0.00	0.00
17	->	39	0.00	0.00
17	->	40	-0.00	0.00
17	->	41	0.00	0.00
17	->	42	-0.00	0.00
17	->	43	-0.00	0.00
17	->	44	-0.00	0.01
17	->	45	-0.00	0.00
17	->	46	-0.00	0.00
17	->	47	-0.00	0.00
17	->	48	-0.00	0.00
17	->	49	-0.00	0.00
17	->	50	-0.00	0.00
17	->	51	-0.00	0.00
17	->	52	-0.00	0.00
17	->	53	-0.00	0.00
17	->	54	-0.00	0.00
17	->	55	0.00	0.00
17	->	56	-0.00	0.00
17	->	57	0.00	0.00
17	->	58	0.00	0.00
17	->	59	-0.00	0.00
17	->	60	-0.00	0.00
17	->	61	0.00	0.00
17	->	62	0.00	0.00
17	->	63	0.00	0.00
17	->	64	-0.00	0.00
17	->	65	0.00	0.00
17	->	66	0.00	0.00
17	->	67	0.00	0.00
17	->	68	-0.00	0.00
17	->	69	-0.00	0.00
17	->	70	-0.00	0.00
17	->	71	0.00	0.00
17	->	72	-0.00	0.00
17	->	73	0.00	0.00
17	->	74	0.00	0.00
17	->	75	0.00	0.00
17	->	76	-0.00	0.00
17	->	77	-0.00	0.00
17	->	78	-0.00	0.00
17	->	79	-0.00	0.00
17	->	80	0.00	0.00
17	->	81	-0.00	0.00
17	->	82	0.00	0.00
17	->	83	0.00	0.00
17	->	84	0.00	0.00

17	->	85	0.00	0.00
17	->	86	0.00	0.00
17	->	87	0.00	0.00
17	->	88	0.00	0.00
17	->	89	0.00	0.00
17	->	90	0.00	0.00
17	->	91	-0.00	0.00
17	->	92	-0.01	0.00
17	->	93	-0.01	0.01
17	->	94	-0.01	0.01
17	->	95	-0.02	0.02
17	->	96	-0.04	0.05
17	->	97	-0.48	0.67
17	->	98	-1.41	1.35
17	->	99	-0.06	0.05
17	->	100	-0.01	0.01
17	->	101	-0.00	0.00
17	->	102	-0.00	0.00
17	->	103	-0.00	0.00
17	->	104	0.00	0.00
17	->	105	0.00	0.00
17	->	106	0.00	0.00
17	->	107	0.00	0.00
18	->	1	0.01	0.01
18	->	2	-0.00	0.00
18	->	3	-0.03	0.01
18	->	4	-0.00	0.00
18	->	5	-0.01	0.00
18	->	6	-0.00	0.00
18	->	7	-0.00	0.00
18	->	8	0.00	0.00
18	->	9	-0.00	0.00
18	->	10	0.00	0.00
18	->	11	0.00	0.00
18	->	12	0.01	0.01
18	->	13	0.01	0.01
18	->	14	-0.06	0.02
18	->	15	-0.01	0.02
18	->	16	-1.17	0.47
18	->	17	-9.49	0.42
18	->	18	24.15	0.88
18	->	19	-14.44	0.52
18	->	20	-0.93	0.15
18	->	21	-2.23	0.32
18	->	22	-2.43	0.78
18	->	23	-0.08	0.07
18	->	24	-0.04	0.03
18	->	25	-0.09	0.04
18	->	26	-0.00	0.00
18	->	27	-0.01	0.00

18	->	28	-0.00	0.00
18	->	29	-0.00	0.00
18	->	30	-0.00	0.00
18	->	31	-0.00	0.00
18	->	32	-0.00	0.00
18	->	33	-0.01	0.00
18	->	34	-0.00	0.00
18	->	35	0.00	0.00
18	->	36	-0.00	0.00
18	->	37	0.00	0.00
18	->	38	0.00	0.00
18	->	39	0.00	0.00
18	->	40	-0.00	0.00
18	->	41	0.00	0.00
18	->	42	-0.00	0.00
18	->	43	-0.01	0.01
18	->	44	0.01	0.02
18	->	45	-0.01	0.00
18	->	46	-0.02	0.01
18	->	47	-0.01	0.00
18	->	48	-0.00	0.00
18	->	49	-0.00	0.00
18	->	50	-0.00	0.00
18	->	51	-0.00	0.00
18	->	52	-0.00	0.00
18	->	53	-0.00	0.00
18	->	54	-0.00	0.00
18	->	55	0.00	0.00
18	->	56	-0.00	0.00
18	->	57	0.00	0.00
18	->	58	0.00	0.00
18	->	59	-0.00	0.00
18	->	60	-0.00	0.00
18	->	61	0.00	0.00
18	->	62	-0.00	0.00
18	->	63	-0.00	0.00
18	->	64	-0.00	0.00
18	->	65	0.00	0.00
18	->	66	-0.00	0.00
18	->	67	0.00	0.00
18	->	68	-0.00	0.00
18	->	69	0.00	0.00
18	->	70	0.00	0.00
18	->	71	0.00	0.00
18	->	72	-0.00	0.00
18	->	73	0.00	0.00
18	->	74	-0.00	0.00
18	->	75	0.00	0.00
18	->	76	0.00	0.00
18	->	77	0.00	0.00

18	->	78	-0.00	0.00
18	->	79	-0.00	0.00
18	->	80	0.00	0.00
18	->	81	0.00	0.00
18	->	82	-0.00	0.00
18	->	83	0.00	0.00
18	->	84	0.00	0.00
18	->	85	0.00	0.00
18	->	86	0.00	0.00
18	->	87	0.00	0.00
18	->	88	0.00	0.00
18	->	89	0.00	0.00
18	->	90	0.00	0.00
18	->	91	-0.02	0.01
18	->	92	-0.06	0.02
18	->	93	-0.18	0.05
18	->	94	-0.05	0.04
18	->	95	-0.08	0.06
18	->	96	-0.02	0.01
18	->	97	-0.13	0.05
18	->	98	-2.12	0.81
18	->	99	-0.63	0.44
18	->	100	-0.07	0.03
18	->	101	-0.00	0.00
18	->	102	-0.01	0.00
18	->	103	-0.00	0.00
18	->	104	-0.00	0.00
18	->	105	-0.00	0.00
18	->	106	0.00	0.00
18	->	107	0.00	0.00
19	->	1	-0.32	0.20
19	->	2	-0.01	0.00
19	->	3	-0.07	0.02
19	->	4	-0.01	0.01
19	->	5	0.01	0.01
19	->	6	-0.01	0.00
19	->	7	0.00	0.00
19	->	8	0.02	0.01
19	->	9	-0.00	0.00
19	->	10	0.03	0.00
19	->	11	0.03	0.01
19	->	12	-0.15	0.10
19	->	13	-0.06	0.02
19	->	14	-1.86	0.31
19	->	15	-0.56	0.35
19	->	16	-14.25	1.52
19	->	17	-0.66	0.17
19	->	18	-14.50	0.52
19	->	19	-8.80	3.15
19	->	20	-8.08	0.47

19	->	21	-1.35	0.33
19	->	22	-7.09	2.78
19	->	23	-2.44	0.71
19	->	24	-0.12	0.06
19	->	25	-0.18	0.05
19	->	26	-0.03	0.01
19	->	27	-0.02	0.00
19	->	28	-0.01	0.00
19	->	29	-0.00	0.00
19	->	30	-0.00	0.00
19	->	31	-0.02	0.00
19	->	32	-0.01	0.00
19	->	33	-0.00	0.00
19	->	34	-0.00	0.00
19	->	35	0.01	0.00
19	->	36	-0.01	0.00
19	->	37	0.00	0.00
19	->	38	0.01	0.00
19	->	39	0.00	0.00
19	->	40	-0.02	0.00
19	->	41	-0.00	0.00
19	->	42	-0.01	0.00
19	->	43	-0.02	0.01
19	->	44	-0.46	0.19
19	->	45	0.01	0.01
19	->	46	0.11	0.04
19	->	47	-0.01	0.01
19	->	48	-0.00	0.00
19	->	49	-0.00	0.00
19	->	50	-0.00	0.00
19	->	51	-0.00	0.00
19	->	52	-0.00	0.00
19	->	53	-0.02	0.00
19	->	54	-0.00	0.00
19	->	55	-0.00	0.00
19	->	56	-0.00	0.00
19	->	57	-0.00	0.00
19	->	58	0.01	0.00
19	->	59	-0.01	0.00
19	->	60	-0.00	0.00
19	->	61	-0.00	0.00
19	->	62	0.00	0.00
19	->	63	0.01	0.00
19	->	64	0.00	0.00
19	->	65	0.00	0.00
19	->	66	0.00	0.00
19	->	67	0.00	0.00
19	->	68	-0.00	0.00
19	->	69	-0.00	0.00
19	->	70	-0.00	0.00

19	->	71	0.00	0.00
19	->	72	-0.03	0.00
19	->	73	-0.00	0.00
19	->	74	0.03	0.00
19	->	75	0.01	0.00
19	->	76	-0.01	0.01
19	->	77	-0.02	0.00
19	->	78	-0.02	0.00
19	->	79	-0.00	0.00
19	->	80	-0.00	0.00
19	->	81	-0.01	0.00
19	->	82	-0.00	0.00
19	->	83	-0.00	0.00
19	->	84	-0.00	0.00
19	->	85	-0.00	0.00
19	->	86	0.00	0.00
19	->	87	0.01	0.00
19	->	88	0.01	0.00
19	->	89	0.02	0.00
19	->	90	0.03	0.00
19	->	91	-0.03	0.06
19	->	92	-2.91	0.73
19	->	93	-0.04	0.96
19	->	94	-0.14	0.23
19	->	95	-0.01	0.02
19	->	96	0.01	0.01
19	->	97	-0.01	0.01
19	->	98	0.05	0.06
19	->	99	0.17	0.07
19	->	100	0.08	0.04
19	->	101	-0.01	0.03
19	->	102	0.04	0.02
19	->	103	0.04	0.01
19	->	104	0.02	0.00
19	->	105	0.02	0.00
19	->	106	0.02	0.00
19	->	107	0.01	0.00
20	->	1	-0.05	0.02
20	->	2	-0.03	0.01
20	->	3	-1.36	0.42
20	->	4	-1.22	0.32
20	->	5	-1.00	0.19
20	->	6	-0.29	0.17
20	->	7	-0.02	0.01
20	->	8	-0.01	0.01
20	->	9	-0.00	0.00
20	->	10	-0.01	0.01
20	->	11	-0.00	0.00
20	->	12	-0.04	0.01
20	->	13	-0.07	0.02

20	->	14	-2.48	0.47
20	->	15	-0.06	0.02
20	->	16	-1.38	0.46
20	->	17	-2.37	0.32
20	->	18	-0.94	0.14
20	->	19	-8.13	0.48
20	->	20	22.34	1.48
20	->	21	-6.47	0.57
20	->	22	-1.39	0.25
20	->	23	-3.00	0.55
20	->	24	-4.30	0.47
20	->	25	-0.19	0.10
20	->	26	-0.06	0.02
20	->	27	-0.07	0.02
20	->	28	-0.02	0.00
20	->	29	-0.00	0.00
20	->	30	-0.01	0.01
20	->	31	-0.00	0.00
20	->	32	-0.00	0.00
20	->	33	-0.00	0.00
20	->	34	-0.00	0.00
20	->	35	0.00	0.00
20	->	36	-0.00	0.00
20	->	37	0.00	0.00
20	->	38	0.00	0.00
20	->	39	0.00	0.00
20	->	40	-0.00	0.00
20	->	41	-0.00	0.00
20	->	42	-0.00	0.00
20	->	43	-0.02	0.01
20	->	44	-0.02	0.01
20	->	45	-0.00	0.00
20	->	46	-0.00	0.00
20	->	47	-0.00	0.00
20	->	48	-0.00	0.00
20	->	49	-0.00	0.00
20	->	50	-0.00	0.00
20	->	51	-0.00	0.00
20	->	52	-0.00	0.00
20	->	53	-0.00	0.00
20	->	54	-0.00	0.00
20	->	55	0.00	0.00
20	->	56	-0.00	0.00
20	->	57	0.00	0.00
20	->	58	0.00	0.00
20	->	59	-0.00	0.00
20	->	60	-0.00	0.00
20	->	61	0.00	0.00
20	->	62	0.00	0.00
20	->	63	0.00	0.00

20	->	64	0.00	0.00
20	->	65	0.00	0.00
20	->	66	0.00	0.00
20	->	67	0.00	0.00
20	->	68	-0.00	0.00
20	->	69	-0.00	0.00
20	->	70	0.00	0.00
20	->	71	0.00	0.00
20	->	72	-0.00	0.00
20	->	73	0.00	0.00
20	->	74	0.00	0.00
20	->	75	0.00	0.00
20	->	76	0.00	0.00
20	->	77	0.00	0.00
20	->	78	-0.00	0.00
20	->	79	-0.00	0.00
20	->	80	0.00	0.00
20	->	81	0.00	0.00
20	->	82	0.00	0.00
20	->	83	0.00	0.00
20	->	84	0.00	0.00
20	->	85	0.00	0.00
20	->	86	0.00	0.00
20	->	87	0.00	0.00
20	->	88	0.00	0.00
20	->	89	-0.00	0.00
20	->	90	-0.00	0.00
20	->	91	0.01	0.01
20	->	92	-0.02	0.03
20	->	93	0.01	0.01
20	->	94	-0.01	0.00
20	->	95	-0.00	0.00
20	->	96	-0.00	0.00
20	->	97	-0.06	0.06
20	->	98	-0.14	0.07
20	->	99	-0.02	0.01
20	->	100	-0.00	0.00
20	->	101	-0.00	0.00
20	->	102	-0.00	0.00
20	->	103	-0.00	0.00
20	->	104	-0.00	0.00
20	->	105	-0.00	0.00
20	->	106	-0.00	0.00
20	->	107	0.00	0.00
21	->	1	0.00	0.00
21	->	2	-0.00	0.00
21	->	3	-0.03	0.01
21	->	4	-0.01	0.00
21	->	5	-0.01	0.01
21	->	6	-0.02	0.01

21	->	7	-0.00	0.00
21	->	8	-0.00	0.00
21	->	9	-0.00	0.00
21	->	10	-0.00	0.00
21	->	11	-0.00	0.00
21	->	12	-0.00	0.00
21	->	13	-0.00	0.00
21	->	14	-0.07	0.01
21	->	15	-0.01	0.00
21	->	16	-0.01	0.07
21	->	17	-2.62	1.36
21	->	18	-2.25	0.32
21	->	19	-1.36	0.33
21	->	20	-6.48	0.57
21	->	21	-20.18	2.02
21	->	22	-10.62	0.42
21	->	23	-0.77	0.20
21	->	24	-2.64	0.45
21	->	25	-3.73	0.54
21	->	26	-0.08	0.05
21	->	27	-0.06	0.02
21	->	28	-0.03	0.01
21	->	29	-0.01	0.00
21	->	30	-0.10	0.06
21	->	31	-0.00	0.00
21	->	32	-0.02	0.01
21	->	33	-0.02	0.01
21	->	34	-0.00	0.00
21	->	35	0.00	0.00
21	->	36	-0.00	0.00
21	->	37	0.00	0.00
21	->	38	0.00	0.00
21	->	39	0.00	0.00
21	->	40	-0.00	0.00
21	->	41	0.00	0.00
21	->	42	0.00	0.01
21	->	43	-0.20	0.09
21	->	44	0.06	0.03
21	->	45	-0.02	0.01
21	->	46	-0.03	0.01
21	->	47	-0.01	0.00
21	->	48	-0.00	0.00
21	->	49	-0.00	0.00
21	->	50	-0.00	0.00
21	->	51	0.00	0.00
21	->	52	-0.00	0.00
21	->	53	0.00	0.00
21	->	54	0.00	0.00
21	->	55	0.00	0.00
21	->	56	0.00	0.00

21	->	57	0.00	0.00
21	->	58	-0.00	0.00
21	->	59	0.00	0.00
21	->	60	0.00	0.00
21	->	61	0.00	0.00
21	->	62	0.00	0.00
21	->	63	-0.00	0.00
21	->	64	-0.00	0.00
21	->	65	0.00	0.00
21	->	66	-0.00	0.00
21	->	67	-0.00	0.00
21	->	68	0.00	0.00
21	->	69	0.00	0.00
21	->	70	0.00	0.00
21	->	71	-0.00	0.00
21	->	72	0.00	0.00
21	->	73	0.00	0.00
21	->	74	-0.00	0.00
21	->	75	-0.00	0.00
21	->	76	0.00	0.00
21	->	77	0.00	0.00
21	->	78	0.00	0.00
21	->	79	0.00	0.00
21	->	80	0.00	0.00
21	->	81	0.00	0.00
21	->	82	0.00	0.00
21	->	83	0.00	0.00
21	->	84	0.00	0.00
21	->	85	0.00	0.00
21	->	86	-0.00	0.00
21	->	87	-0.00	0.00
21	->	88	0.00	0.00
21	->	89	-0.00	0.00
21	->	90	-0.00	0.00
21	->	91	-0.02	0.01
21	->	92	-0.05	0.02
21	->	93	-0.03	0.01
21	->	94	-0.00	0.01
21	->	95	-0.00	0.00
21	->	96	-0.01	0.00
21	->	97	-0.57	0.68
21	->	98	-2.66	1.37
21	->	99	-0.22	0.09
21	->	100	-0.02	0.00
21	->	101	0.00	0.00
21	->	102	-0.00	0.00
21	->	103	-0.00	0.00
21	->	104	-0.00	0.00
21	->	105	-0.00	0.00
21	->	106	-0.00	0.00

21	->	107	0.00	0.00
22	->	1	0.08	0.02
22	->	2	-0.00	0.00
22	->	3	-0.01	0.00
22	->	4	0.00	0.00
22	->	5	-0.10	0.02
22	->	6	-0.01	0.00
22	->	7	0.00	0.01
22	->	8	-0.01	0.00
22	->	9	-0.00	0.00
22	->	10	-0.06	0.01
22	->	11	-0.02	0.01
22	->	12	0.06	0.02
22	->	13	0.04	0.01
22	->	14	-0.20	0.05
22	->	15	-0.01	0.01
22	->	16	1.24	0.82
22	->	17	-0.11	0.07
22	->	18	-2.46	0.78
22	->	19	-7.13	2.78
22	->	20	-1.39	0.25
22	->	21	-10.64	0.43
22	->	22	-97.10	2.82
22	->	23	-19.45	0.42
22	->	24	-0.66	0.20
22	->	25	-1.91	0.47
22	->	26	-1.43	0.68
22	->	27	-0.09	0.04
22	->	28	-0.02	0.01
22	->	29	-0.00	0.01
22	->	30	-0.03	0.01
22	->	31	0.01	0.00
22	->	32	-0.03	0.01
22	->	33	-0.05	0.02
22	->	34	-0.00	0.00
22	->	35	-0.02	0.00
22	->	36	0.01	0.00
22	->	37	-0.00	0.00
22	->	38	-0.02	0.00
22	->	39	-0.00	0.00
22	->	40	0.02	0.00
22	->	41	0.00	0.01
22	->	42	0.00	0.02
22	->	43	-1.66	0.79
22	->	44	-0.62	0.76
22	->	45	-0.21	0.12
22	->	46	-2.31	1.28
22	->	47	-0.21	0.07
22	->	48	-0.01	0.00
22	->	49	0.01	0.00

22	->	50	-0.00	0.00
22	->	51	0.00	0.00
22	->	52	0.00	0.00
22	->	53	0.03	0.01
22	->	54	0.00	0.00
22	->	55	-0.00	0.00
22	->	56	0.00	0.00
22	->	57	0.00	0.00
22	->	58	-0.01	0.00
22	->	59	0.01	0.00
22	->	60	0.00	0.00
22	->	61	-0.00	0.00
22	->	62	-0.00	0.00
22	->	63	-0.01	0.00
22	->	64	-0.01	0.00
22	->	65	-0.00	0.00
22	->	66	-0.01	0.00
22	->	67	-0.00	0.00
22	->	68	0.01	0.00
22	->	69	0.01	0.00
22	->	70	0.00	0.00
22	->	71	-0.00	0.00
22	->	72	0.05	0.01
22	->	73	0.00	0.00
22	->	74	-0.04	0.01
22	->	75	-0.02	0.00
22	->	76	0.02	0.01
22	->	77	0.02	0.00
22	->	78	0.02	0.00
22	->	79	0.00	0.00
22	->	80	0.00	0.00
22	->	81	0.01	0.00
22	->	82	0.00	0.00
22	->	83	0.00	0.00
22	->	84	0.00	0.00
22	->	85	0.00	0.00
22	->	86	-0.00	0.00
22	->	87	-0.01	0.00
22	->	88	-0.02	0.00
22	->	89	-0.04	0.00
22	->	90	-0.09	0.01
22	->	91	-0.35	0.11
22	->	92	-2.67	1.10
22	->	93	-8.22	1.31
22	->	94	0.20	0.29
22	->	95	-0.07	0.02
22	->	96	-0.03	0.01
22	->	97	-0.03	0.01
22	->	98	-0.88	0.43
22	->	99	-1.13	0.38

22	->	100	-2.38	1.04
22	->	101	0.61	0.42
22	->	102	-0.14	0.08
22	->	103	-0.07	0.02
22	->	104	-0.03	0.00
22	->	105	-0.03	0.00
22	->	106	-0.02	0.00
22	->	107	-0.01	0.00
23	->	1	0.00	0.00
23	->	2	-0.00	0.00
23	->	3	-0.02	0.01
23	->	4	-0.09	0.03
23	->	5	1.13	0.61
23	->	6	-0.11	0.07
23	->	7	-0.19	0.10
23	->	8	-0.02	0.01
23	->	9	-0.01	0.04
23	->	10	1.73	0.88
23	->	11	0.00	0.01
23	->	12	-0.07	0.03
23	->	13	-0.04	0.01
23	->	14	-2.11	0.28
23	->	15	-0.01	0.00
23	->	16	-0.01	0.01
23	->	17	-0.07	0.04
23	->	18	-0.08	0.07
23	->	19	-2.47	0.71
23	->	20	-3.00	0.56
23	->	21	-0.77	0.20
23	->	22	-19.51	0.43
23	->	23	9.38	1.21
23	->	24	-10.15	0.48
23	->	25	-1.30	0.28
23	->	26	-3.28	0.68
23	->	27	30.47	15.89
23	->	28	-0.22	0.07
23	->	29	-0.04	0.02
23	->	30	-0.03	0.02
23	->	31	-0.01	0.00
23	->	32	-0.01	0.00
23	->	33	-0.00	0.00
23	->	34	-0.00	0.00
23	->	35	-0.00	0.00
23	->	36	0.00	0.00
23	->	37	-0.00	0.00
23	->	38	-0.00	0.00
23	->	39	0.00	0.00
23	->	40	-0.00	0.00
23	->	41	-0.01	0.00
23	->	42	-0.02	0.01

23	->	43	-0.06	0.04
23	->	44	-0.05	0.02
23	->	45	0.00	0.00
23	->	46	-0.00	0.00
23	->	47	-0.00	0.00
23	->	48	-0.00	0.00
23	->	49	0.00	0.00
23	->	50	0.00	0.00
23	->	51	-0.00	0.00
23	->	52	0.00	0.00
23	->	53	0.00	0.00
23	->	54	0.00	0.00
23	->	55	0.00	0.00
23	->	56	0.00	0.00
23	->	57	0.00	0.00
23	->	58	-0.00	0.00
23	->	59	0.00	0.00
23	->	60	0.00	0.00
23	->	61	0.00	0.00
23	->	62	0.00	0.00
23	->	63	-0.00	0.00
23	->	64	-0.00	0.00
23	->	65	-0.00	0.00
23	->	66	-0.00	0.00
23	->	67	0.00	0.00
23	->	68	0.00	0.00
23	->	69	0.00	0.00
23	->	70	0.00	0.00
23	->	71	-0.00	0.00
23	->	72	0.00	0.00
23	->	73	0.00	0.00
23	->	74	-0.00	0.00
23	->	75	-0.00	0.00
23	->	76	0.00	0.00
23	->	77	0.00	0.00
23	->	78	0.00	0.00
23	->	79	0.00	0.00
23	->	80	0.00	0.00
23	->	81	0.00	0.00
23	->	82	0.00	0.00
23	->	83	0.00	0.00
23	->	84	0.00	0.00
23	->	85	0.00	0.00
23	->	86	-0.00	0.00
23	->	87	-0.00	0.00
23	->	88	-0.00	0.00
23	->	89	-0.00	0.00
23	->	90	-0.02	0.00
23	->	91	-0.18	0.17
23	->	92	-0.94	1.42

23	->	93	-0.02	0.01
23	->	94	-0.01	0.00
23	->	95	-0.00	0.00
23	->	96	-0.00	0.00
23	->	97	-0.01	0.01
23	->	98	-0.02	0.01
23	->	99	-0.00	0.01
23	->	100	-0.01	0.00
23	->	101	-0.00	0.00
23	->	102	-0.00	0.00
23	->	103	-0.00	0.00
23	->	104	-0.00	0.00
23	->	105	-0.00	0.00
23	->	106	-0.00	0.00
23	->	107	-0.00	0.00
24	->	1	0.00	0.00
24	->	2	-0.00	0.00
24	->	3	-0.02	0.01
24	->	4	-0.06	0.03
24	->	5	-0.49	0.28
24	->	6	-1.19	0.41
24	->	7	-0.09	0.12
24	->	8	-0.01	0.00
24	->	9	-0.00	0.00
24	->	10	0.02	0.01
24	->	11	-0.00	0.00
24	->	12	-0.01	0.00
24	->	13	-0.01	0.00
24	->	14	-0.08	0.02
24	->	15	-0.00	0.00
24	->	16	-0.00	0.01
24	->	17	-0.07	0.03
24	->	18	-0.04	0.03
24	->	19	-0.12	0.06
24	->	20	-4.28	0.46
24	->	21	-2.66	0.46
24	->	22	-0.67	0.20
24	->	23	-10.20	0.48
24	->	24	23.89	1.89
24	->	25	-9.97	0.48
24	->	26	-1.05	0.19
24	->	27	-2.48	0.42
24	->	28	-5.87	0.70
24	->	29	-0.20	0.10
24	->	30	-0.90	0.33
24	->	31	-0.01	0.00
24	->	32	-0.01	0.00
24	->	33	-0.01	0.00
24	->	34	-0.00	0.00
24	->	35	0.00	0.00

24 ->	36	-0.00	0.00
24 ->	37	0.00	0.00
24 ->	38	0.00	0.00
24 ->	39	0.00	0.00
24 ->	40	-0.00	0.00
24 ->	41	0.00	0.00
24 ->	42	0.00	0.00
24 ->	43	-0.03	0.02
24 ->	44	-0.01	0.01
24 ->	45	-0.01	0.00
24 ->	46	-0.00	0.00
24 ->	47	-0.00	0.00
24 ->	48	-0.00	0.00
24 ->	49	-0.00	0.00
24 ->	50	0.00	0.00
24 ->	51	0.00	0.00
24 ->	52	0.00	0.00
24 ->	53	0.00	0.00
24 ->	54	0.00	0.00
24 ->	55	0.00	0.00
24 ->	56	0.00	0.00
24 ->	57	0.00	0.00
24 ->	58	-0.00	0.00
24 ->	59	0.00	0.00
24 ->	60	0.00	0.00
24 ->	61	0.00	0.00
24 ->	62	0.00	0.00
24 ->	63	-0.00	0.00
24 ->	64	-0.00	0.00
24 ->	65	0.00	0.00
24 ->	66	0.00	0.00
24 ->	67	0.00	0.00
24 ->	68	0.00	0.00
24 ->	69	0.00	0.00
24 ->	70	0.00	0.00
24 ->	71	-0.00	0.00
24 ->	72	0.00	0.00
24 ->	73	0.00	0.00
24 ->	74	-0.00	0.00
24 ->	75	0.00	0.00
24 ->	76	0.00	0.00
24 ->	77	0.00	0.00
24 ->	78	0.00	0.00
24 ->	79	0.00	0.00
24 ->	80	0.00	0.00
24 ->	81	0.00	0.00
24 ->	82	0.00	0.00
24 ->	83	0.00	0.00
24 ->	84	0.00	0.00
24 ->	85	0.00	0.00

24 ->	86	0.00	0.00
24 ->	87	-0.00	0.00
24 ->	88	0.00	0.00
24 ->	89	-0.00	0.00
24 ->	90	-0.00	0.00
24 ->	91	0.00	0.00
24 ->	92	0.00	0.02
24 ->	93	-0.01	0.00
24 ->	94	-0.00	0.00
24 ->	95	-0.00	0.00
24 ->	96	-0.00	0.00
24 ->	97	-0.03	0.03
24 ->	98	-0.09	0.05
24 ->	99	-0.02	0.01
24 ->	100	-0.00	0.00
24 ->	101	-0.00	0.00
24 ->	102	-0.00	0.00
24 ->	103	-0.00	0.00
24 ->	104	-0.00	0.00
24 ->	105	-0.00	0.00
24 ->	106	-0.00	0.00
24 ->	107	0.00	0.00
25 ->	1	0.03	0.00
25 ->	2	-0.00	0.00
25 ->	3	-0.00	0.00
25 ->	4	-0.01	0.00
25 ->	5	-0.05	0.01
25 ->	6	-0.01	0.01
25 ->	7	-0.00	0.00
25 ->	8	-0.01	0.00
25 ->	9	-0.00	0.00
25 ->	10	-0.03	0.00
25 ->	11	-0.01	0.00
25 ->	12	0.02	0.00
25 ->	13	0.02	0.00
25 ->	14	-0.02	0.00
25 ->	15	0.00	0.00
25 ->	16	0.08	0.02
25 ->	17	-0.02	0.01
25 ->	18	-0.09	0.04
25 ->	19	-0.18	0.05
25 ->	20	-0.19	0.10
25 ->	21	-3.77	0.54
25 ->	22	-1.95	0.47
25 ->	23	-1.31	0.28
25 ->	24	-9.99	0.47
25 ->	25	-97.20	3.22
25 ->	26	-12.92	0.35
25 ->	27	-0.60	0.13
25 ->	28	-1.18	0.18

25 ->	29	-1.11	0.38
25 ->	30	-4.37	0.90
25 ->	31	0.09	0.05
25 ->	32	-1.55	0.34
25 ->	33	-0.73	0.24
25 ->	34	-0.02	0.01
25 ->	35	-0.03	0.00
25 ->	36	0.02	0.01
25 ->	37	-0.00	0.00
25 ->	38	-0.02	0.00
25 ->	39	-0.00	0.00
25 ->	40	0.04	0.01
25 ->	41	0.06	0.05
25 ->	42	0.05	0.06
25 ->	43	-5.22	0.63
25 ->	44	-0.18	0.25
25 ->	45	-0.11	0.20
25 ->	46	-0.27	0.10
25 ->	47	-0.02	0.01
25 ->	48	-0.01	0.01
25 ->	49	0.00	0.00
25 ->	50	0.00	0.00
25 ->	51	0.00	0.00
25 ->	52	0.00	0.00
25 ->	53	0.02	0.00
25 ->	54	0.00	0.00
25 ->	55	-0.00	0.00
25 ->	56	0.00	0.00
25 ->	57	0.00	0.00
25 ->	58	-0.01	0.00
25 ->	59	0.01	0.00
25 ->	60	0.00	0.00
25 ->	61	0.00	0.00
25 ->	62	-0.00	0.00
25 ->	63	-0.00	0.00
25 ->	64	-0.00	0.00
25 ->	65	-0.00	0.00
25 ->	66	-0.00	0.00
25 ->	67	0.00	0.00
25 ->	68	0.00	0.00
25 ->	69	0.00	0.00
25 ->	70	0.00	0.00
25 ->	71	-0.00	0.00
25 ->	72	0.02	0.00
25 ->	73	0.00	0.00
25 ->	74	-0.02	0.00
25 ->	75	-0.01	0.00
25 ->	76	0.01	0.00
25 ->	77	0.01	0.00
25 ->	78	0.01	0.00

25	->	79	0.00	0.00
25	->	80	0.00	0.00
25	->	81	0.01	0.00
25	->	82	0.00	0.00
25	->	83	0.00	0.00
25	->	84	0.00	0.00
25	->	85	0.00	0.00
25	->	86	-0.00	0.00
25	->	87	-0.01	0.00
25	->	88	-0.01	0.00
25	->	89	-0.03	0.00
25	->	90	-0.05	0.01
25	->	91	-0.08	0.03
25	->	92	-0.08	0.02
25	->	93	-0.05	0.01
25	->	94	-0.02	0.00
25	->	95	-0.02	0.00
25	->	96	-0.02	0.00
25	->	97	-0.31	0.26
25	->	98	-5.56	2.37
25	->	99	-10.32	2.75
25	->	100	-0.08	0.02
25	->	101	-0.01	0.01
25	->	102	-0.01	0.00
25	->	103	-0.01	0.00
25	->	104	-0.01	0.00
25	->	105	-0.01	0.00
25	->	106	-0.01	0.00
25	->	107	-0.01	0.00
26	->	1	0.00	0.00
26	->	2	-0.00	0.00
26	->	3	-0.00	0.00
26	->	4	-0.00	0.00
26	->	5	-0.05	0.02
26	->	6	-0.01	0.00
26	->	7	-0.00	0.01
26	->	8	-0.00	0.00
26	->	9	-0.01	0.01
26	->	10	-0.04	0.01
26	->	11	-0.00	0.00
26	->	12	-0.00	0.00
26	->	13	-0.00	0.00
26	->	14	-0.03	0.01
26	->	15	-0.00	0.00
26	->	16	-0.00	0.00
26	->	17	-0.00	0.00
26	->	18	-0.00	0.00
26	->	19	-0.03	0.01
26	->	20	-0.06	0.02
26	->	21	-0.08	0.05

26 ->	22	-1.47	0.68
26 ->	23	-3.29	0.68
26 ->	24	-1.05	0.19
26 ->	25	-12.97	0.36
26 ->	26	17.83	1.72
26 ->	27	-21.80	0.52
26 ->	28	-0.63	0.15
26 ->	29	-1.14	0.42
26 ->	30	-0.48	0.14
26 ->	31	-0.08	0.09
26 ->	32	-0.06	0.02
26 ->	33	-0.01	0.00
26 ->	34	-0.00	0.00
26 ->	35	-0.00	0.00
26 ->	36	-0.00	0.00
26 ->	37	-0.00	0.00
26 ->	38	-0.00	0.00
26 ->	39	-0.00	0.00
26 ->	40	-0.01	0.00
26 ->	41	-0.10	0.09
26 ->	42	-0.08	0.05
26 ->	43	-1.05	0.18
26 ->	44	-0.06	0.02
26 ->	45	0.00	0.00
26 ->	46	-0.00	0.00
26 ->	47	-0.01	0.01
26 ->	48	-0.00	0.00
26 ->	49	-0.00	0.00
26 ->	50	-0.00	0.00
26 ->	51	-0.00	0.00
26 ->	52	-0.00	0.00
26 ->	53	0.00	0.00
26 ->	54	0.00	0.00
26 ->	55	0.00	0.00
26 ->	56	0.00	0.00
26 ->	57	0.00	0.00
26 ->	58	-0.00	0.00
26 ->	59	0.00	0.00
26 ->	60	0.00	0.00
26 ->	61	0.00	0.00
26 ->	62	0.00	0.00
26 ->	63	-0.00	0.00
26 ->	64	-0.00	0.00
26 ->	65	0.00	0.00
26 ->	66	-0.00	0.00
26 ->	67	0.00	0.00
26 ->	68	0.00	0.00
26 ->	69	0.00	0.00
26 ->	70	0.00	0.00
26 ->	71	0.00	0.00

26 ->	72	0.00	0.00
26 ->	73	0.00	0.00
26 ->	74	-0.00	0.00
26 ->	75	-0.00	0.00
26 ->	76	0.00	0.00
26 ->	77	0.00	0.00
26 ->	78	0.00	0.00
26 ->	79	0.00	0.00
26 ->	80	0.00	0.00
26 ->	81	0.00	0.00
26 ->	82	0.00	0.00
26 ->	83	0.00	0.00
26 ->	84	0.00	0.00
26 ->	85	0.00	0.00
26 ->	86	-0.00	0.00
26 ->	87	-0.00	0.00
26 ->	88	-0.00	0.00
26 ->	89	-0.01	0.00
26 ->	90	-0.05	0.04
26 ->	91	-0.30	0.44
26 ->	92	-0.07	0.07
26 ->	93	-0.01	0.00
26 ->	94	-0.00	0.00
26 ->	95	-0.00	0.00
26 ->	96	-0.00	0.00
26 ->	97	-0.00	0.00
26 ->	98	0.00	0.00
26 ->	99	-0.00	0.01
26 ->	100	-0.00	0.00
26 ->	101	-0.00	0.00
26 ->	102	-0.00	0.00
26 ->	103	-0.00	0.00
26 ->	104	-0.00	0.00
26 ->	105	-0.00	0.00
26 ->	106	-0.00	0.00
26 ->	107	-0.00	0.00
27 ->	1	-0.01	0.00
27 ->	2	-0.00	0.00
27 ->	3	-0.01	0.00
27 ->	4	-0.06	0.01
27 ->	5	1.37	0.34
27 ->	6	-0.33	0.17
27 ->	7	-1.34	0.98
27 ->	8	-0.03	0.02
27 ->	9	-0.09	0.08
27 ->	10	1.30	0.26
27 ->	11	0.01	0.00
27 ->	12	-0.02	0.01
27 ->	13	-0.02	0.01
27 ->	14	-0.03	0.01

27 ->	15	0.00	0.00
27 ->	16	-0.00	0.00
27 ->	17	-0.00	0.00
27 ->	18	-0.01	0.00
27 ->	19	-0.02	0.00
27 ->	20	-0.07	0.02
27 ->	21	-0.06	0.02
27 ->	22	-0.09	0.04
27 ->	23	30.49	15.89
27 ->	24	-2.47	0.42
27 ->	25	-0.60	0.13
27 ->	26	-21.88	0.52
27 ->	27	3.09	1.59
27 ->	28	-4.84	0.46
27 ->	29	-0.52	0.13
27 ->	30	-0.04	0.03
27 ->	31	-0.01	0.01
27 ->	32	-0.01	0.00
27 ->	33	-0.00	0.00
27 ->	34	-0.00	0.00
27 ->	35	-0.00	0.00
27 ->	36	0.00	0.00
27 ->	37	-0.00	0.00
27 ->	38	-0.00	0.00
27 ->	39	-0.00	0.00
27 ->	40	-0.00	0.00
27 ->	41	-0.01	0.01
27 ->	42	-0.01	0.00
27 ->	43	-0.02	0.01
27 ->	44	-0.00	0.00
27 ->	45	0.00	0.00
27 ->	46	-0.00	0.00
27 ->	47	-0.00	0.00
27 ->	48	-0.00	0.00
27 ->	49	0.00	0.00
27 ->	50	0.00	0.00
27 ->	51	0.00	0.00
27 ->	52	0.00	0.00
27 ->	53	0.00	0.00
27 ->	54	0.00	0.00
27 ->	55	-0.00	0.00
27 ->	56	0.00	0.00
27 ->	57	0.00	0.00
27 ->	58	-0.00	0.00
27 ->	59	0.00	0.00
27 ->	60	0.00	0.00
27 ->	61	0.00	0.00
27 ->	62	0.00	0.00
27 ->	63	-0.00	0.00
27 ->	64	-0.00	0.00

27	->	65	0.00	0.00
27	->	66	-0.00	0.00
27	->	67	0.00	0.00
27	->	68	0.00	0.00
27	->	69	0.00	0.00
27	->	70	0.00	0.00
27	->	71	0.00	0.00
27	->	72	0.00	0.00
27	->	73	0.00	0.00
27	->	74	-0.00	0.00
27	->	75	-0.00	0.00
27	->	76	0.00	0.00
27	->	77	0.00	0.00
27	->	78	0.00	0.00
27	->	79	0.00	0.00
27	->	80	0.00	0.00
27	->	81	0.00	0.00
27	->	82	0.00	0.00
27	->	83	0.00	0.00
27	->	84	0.00	0.00
27	->	85	-0.00	0.00
27	->	86	0.00	0.00
27	->	87	-0.00	0.00
27	->	88	-0.00	0.00
27	->	89	-0.00	0.00
27	->	90	-0.01	0.00
27	->	91	-0.01	0.01
27	->	92	-0.00	0.02
27	->	93	-0.00	0.00
27	->	94	-0.00	0.00
27	->	95	-0.00	0.00
27	->	96	-0.00	0.00
27	->	97	-0.00	0.00
27	->	98	-0.01	0.00
27	->	99	-0.00	0.00
27	->	100	-0.00	0.00
27	->	101	-0.00	0.00
27	->	102	-0.00	0.00
27	->	103	-0.00	0.00
27	->	104	-0.00	0.00
27	->	105	-0.00	0.00
27	->	106	-0.00	0.00
27	->	107	-0.00	0.00
28	->	1	0.00	0.00
28	->	2	-0.00	0.00
28	->	3	-0.00	0.00
28	->	4	-0.00	0.00
28	->	5	-0.02	0.01
28	->	6	-0.10	0.13
28	->	7	-0.06	0.06

28 ->	8	-0.00	0.00
28 ->	9	-0.00	0.00
28 ->	10	-0.01	0.00
28 ->	11	-0.00	0.00
28 ->	12	0.00	0.00
28 ->	13	0.00	0.00
28 ->	14	-0.00	0.00
28 ->	15	0.00	0.00
28 ->	16	0.00	0.00
28 ->	17	-0.00	0.00
28 ->	18	-0.00	0.00
28 ->	19	-0.01	0.00
28 ->	20	-0.02	0.00
28 ->	21	-0.03	0.01
28 ->	22	-0.02	0.01
28 ->	23	-0.22	0.07
28 ->	24	-5.87	0.70
28 ->	25	-1.20	0.19
28 ->	26	-0.64	0.15
28 ->	27	-5.00	0.46
28 ->	28	-22.44	1.24
28 ->	29	-11.60	0.50
28 ->	30	-4.09	0.97
28 ->	31	-0.04	0.01
28 ->	32	-0.02	0.01
28 ->	33	-0.00	0.00
28 ->	34	-0.00	0.00
28 ->	35	0.00	0.00
28 ->	36	-0.00	0.00
28 ->	37	-0.00	0.00
28 ->	38	0.00	0.00
28 ->	39	-0.00	0.00
28 ->	40	-0.00	0.00
28 ->	41	-0.01	0.01
28 ->	42	-0.01	0.00
28 ->	43	-0.02	0.01
28 ->	44	0.00	0.00
28 ->	45	-0.00	0.00
28 ->	46	-0.00	0.00
28 ->	47	-0.00	0.00
28 ->	48	-0.00	0.00
28 ->	49	0.00	0.00
28 ->	50	0.00	0.00
28 ->	51	0.00	0.00
28 ->	52	0.00	0.00
28 ->	53	0.00	0.00
28 ->	54	0.00	0.00
28 ->	55	0.00	0.00
28 ->	56	0.00	0.00
28 ->	57	0.00	0.00

28 ->	58	0.00	0.00
28 ->	59	0.00	0.00
28 ->	60	0.00	0.00
28 ->	61	0.00	0.00
28 ->	62	0.00	0.00
28 ->	63	-0.00	0.00
28 ->	64	-0.00	0.00
28 ->	65	0.00	0.00
28 ->	66	-0.00	0.00
28 ->	67	0.00	0.00
28 ->	68	0.00	0.00
28 ->	69	0.00	0.00
28 ->	70	0.00	0.00
28 ->	71	0.00	0.00
28 ->	72	0.00	0.00
28 ->	73	0.00	0.00
28 ->	74	-0.00	0.00
28 ->	75	-0.00	0.00
28 ->	76	0.00	0.00
28 ->	77	0.00	0.00
28 ->	78	0.00	0.00
28 ->	79	0.00	0.00
28 ->	80	0.00	0.00
28 ->	81	0.00	0.00
28 ->	82	0.00	0.00
28 ->	83	0.00	0.00
28 ->	84	0.00	0.00
28 ->	85	0.00	0.00
28 ->	86	0.00	0.00
28 ->	87	-0.00	0.00
28 ->	88	-0.00	0.00
28 ->	89	-0.00	0.00
28 ->	90	-0.00	0.00
28 ->	91	-0.01	0.00
28 ->	92	-0.01	0.00
28 ->	93	-0.00	0.00
28 ->	94	-0.00	0.00
28 ->	95	-0.00	0.00
28 ->	96	-0.00	0.00
28 ->	97	-0.00	0.00
28 ->	98	-0.00	0.01
28 ->	99	-0.00	0.00
28 ->	100	-0.00	0.00
28 ->	101	-0.00	0.00
28 ->	102	-0.00	0.00
28 ->	103	-0.00	0.00
28 ->	104	-0.00	0.00
28 ->	105	-0.00	0.00
28 ->	106	-0.00	0.00
28 ->	107	-0.00	0.00

29	->	1	0.00	0.00
29	->	2	0.00	0.00
29	->	3	0.00	0.00
29	->	4	0.00	0.00
29	->	5	-0.00	0.00
29	->	6	-0.00	0.00
29	->	7	-0.00	0.00
29	->	8	-0.00	0.00
29	->	9	-0.00	0.00
29	->	10	-0.00	0.00
29	->	11	-0.00	0.00
29	->	12	0.00	0.00
29	->	13	0.00	0.00
29	->	14	-0.00	0.00
29	->	15	0.00	0.00
29	->	16	0.00	0.00
29	->	17	-0.00	0.00
29	->	18	-0.00	0.00
29	->	19	-0.00	0.00
29	->	20	-0.00	0.00
29	->	21	-0.01	0.00
29	->	22	-0.00	0.01
29	->	23	-0.04	0.02
29	->	24	-0.20	0.10
29	->	25	-1.11	0.38
29	->	26	-1.18	0.43
29	->	27	-0.53	0.13
29	->	28	-11.68	0.50
29	->	29	12.18	0.79
29	->	30	-14.54	0.46
29	->	31	-1.14	0.72
29	->	32	-0.03	0.00
29	->	33	-0.01	0.00
29	->	34	-0.01	0.01
29	->	35	-0.00	0.00
29	->	36	-0.00	0.00
29	->	37	-0.00	0.00
29	->	38	-0.00	0.00
29	->	39	-0.00	0.00
29	->	40	0.00	0.00
29	->	41	-0.04	0.10
29	->	42	-0.00	0.01
29	->	43	-0.00	0.02
29	->	44	-0.00	0.00
29	->	45	-0.00	0.00
29	->	46	-0.00	0.00
29	->	47	-0.00	0.00
29	->	48	-0.00	0.00
29	->	49	0.00	0.00
29	->	50	0.00	0.00

29	->	51	0.00	0.00
29	->	52	0.00	0.00
29	->	53	0.00	0.00
29	->	54	0.00	0.00
29	->	55	-0.00	0.00
29	->	56	0.00	0.00
29	->	57	-0.00	0.00
29	->	58	-0.00	0.00
29	->	59	0.00	0.00
29	->	60	0.00	0.00
29	->	61	0.00	0.00
29	->	62	0.00	0.00
29	->	63	-0.00	0.00
29	->	64	-0.00	0.00
29	->	65	0.00	0.00
29	->	66	-0.00	0.00
29	->	67	0.00	0.00
29	->	68	0.00	0.00
29	->	69	0.00	0.00
29	->	70	0.00	0.00
29	->	71	0.00	0.00
29	->	72	0.00	0.00
29	->	73	0.00	0.00
29	->	74	-0.00	0.00
29	->	75	-0.00	0.00
29	->	76	0.00	0.00
29	->	77	0.00	0.00
29	->	78	0.00	0.00
29	->	79	0.00	0.00
29	->	80	0.00	0.00
29	->	81	0.00	0.00
29	->	82	0.00	0.00
29	->	83	0.00	0.00
29	->	84	0.00	0.00
29	->	85	0.00	0.00
29	->	86	-0.00	0.00
29	->	87	-0.00	0.00
29	->	88	-0.00	0.00
29	->	89	-0.00	0.00
29	->	90	-0.00	0.00
29	->	91	-0.01	0.00
29	->	92	-0.00	0.00
29	->	93	-0.00	0.00
29	->	94	-0.00	0.00
29	->	95	-0.00	0.00
29	->	96	-0.00	0.00
29	->	97	-0.00	0.00
29	->	98	-0.00	0.00
29	->	99	-0.00	0.00
29	->	100	-0.00	0.00

29	->	101	-0.00	0.00
29	->	102	-0.00	0.00
29	->	103	-0.00	0.00
29	->	104	-0.00	0.00
29	->	105	-0.00	0.00
29	->	106	-0.00	0.00
29	->	107	-0.00	0.00
30	->	1	0.00	0.00
30	->	2	-0.00	0.00
30	->	3	-0.00	0.00
30	->	4	-0.00	0.00
30	->	5	-0.01	0.00
30	->	6	-0.01	0.00
30	->	7	-0.00	0.00
30	->	8	-0.00	0.00
30	->	9	-0.00	0.00
30	->	10	-0.00	0.00
30	->	11	-0.00	0.00
30	->	12	-0.00	0.00
30	->	13	0.00	0.00
30	->	14	-0.00	0.00
30	->	15	-0.00	0.00
30	->	16	-0.00	0.00
30	->	17	-0.00	0.00
30	->	18	-0.00	0.00
30	->	19	-0.00	0.00
30	->	20	-0.01	0.01
30	->	21	-0.10	0.06
30	->	22	-0.03	0.01
30	->	23	-0.03	0.02
30	->	24	-0.89	0.32
30	->	25	-4.33	0.88
30	->	26	-0.48	0.14
30	->	27	-0.04	0.03
30	->	28	-4.09	0.97
30	->	29	-14.68	0.47
30	->	30	-52.23	1.41
30	->	31	-11.07	0.41
30	->	32	-1.28	0.33
30	->	33	-0.10	0.03
30	->	34	-0.02	0.02
30	->	35	-0.00	0.00
30	->	36	-0.00	0.00
30	->	37	-0.00	0.00
30	->	38	0.00	0.00
30	->	39	-0.00	0.00
30	->	40	-0.01	0.01
30	->	41	-0.56	0.50
30	->	42	-0.10	0.09
30	->	43	-0.30	0.63

30 ->	44	-0.02	0.01
30 ->	45	-0.01	0.00
30 ->	46	0.00	0.00
30 ->	47	-0.00	0.00
30 ->	48	-0.00	0.00
30 ->	49	-0.00	0.00
30 ->	50	-0.00	0.00
30 ->	51	-0.00	0.00
30 ->	52	-0.00	0.00
30 ->	53	-0.00	0.00
30 ->	54	-0.00	0.00
30 ->	55	0.00	0.00
30 ->	56	-0.00	0.00
30 ->	57	-0.00	0.00
30 ->	58	0.00	0.00
30 ->	59	-0.00	0.00
30 ->	60	-0.00	0.00
30 ->	61	-0.00	0.00
30 ->	62	0.00	0.00
30 ->	63	0.00	0.00
30 ->	64	0.00	0.00
30 ->	65	0.00	0.00
30 ->	66	0.00	0.00
30 ->	67	0.00	0.00
30 ->	68	-0.00	0.00
30 ->	69	-0.00	0.00
30 ->	70	-0.00	0.00
30 ->	71	0.00	0.00
30 ->	72	-0.00	0.00
30 ->	73	-0.00	0.00
30 ->	74	0.00	0.00
30 ->	75	0.00	0.00
30 ->	76	-0.00	0.00
30 ->	77	-0.00	0.00
30 ->	78	-0.00	0.00
30 ->	79	-0.00	0.00
30 ->	80	0.00	0.00
30 ->	81	-0.00	0.00
30 ->	82	-0.00	0.00
30 ->	83	0.00	0.00
30 ->	84	0.00	0.00
30 ->	85	-0.00	0.00
30 ->	86	0.00	0.00
30 ->	87	0.00	0.00
30 ->	88	0.00	0.00
30 ->	89	0.00	0.00
30 ->	90	0.00	0.00
30 ->	91	0.00	0.00
30 ->	92	-0.00	0.00
30 ->	93	-0.00	0.00

30	->	94	-0.00	0.00
30	->	95	0.00	0.00
30	->	96	0.00	0.00
30	->	97	-0.01	0.01
30	->	98	-0.03	0.10
30	->	99	-0.01	0.01
30	->	100	0.00	0.00
30	->	101	-0.00	0.00
30	->	102	0.00	0.00
30	->	103	0.00	0.00
30	->	104	0.00	0.00
30	->	105	0.00	0.00
30	->	106	0.00	0.00
30	->	107	0.00	0.00
31	->	1	0.01	0.00
31	->	2	-0.00	0.00
31	->	3	0.00	0.00
31	->	4	0.00	0.00
31	->	5	-0.02	0.00
31	->	6	0.00	0.00
31	->	7	-0.00	0.00
31	->	8	-0.01	0.00
31	->	9	0.00	0.00
31	->	10	-0.02	0.01
31	->	11	-0.01	0.00
31	->	12	0.01	0.00
31	->	13	0.01	0.00
31	->	14	-0.00	0.00
31	->	15	0.00	0.00
31	->	16	0.01	0.00
31	->	17	-0.00	0.00
31	->	18	-0.00	0.00
31	->	19	-0.02	0.00
31	->	20	-0.00	0.00
31	->	21	-0.00	0.00
31	->	22	0.01	0.00
31	->	23	-0.01	0.00
31	->	24	-0.01	0.00
31	->	25	0.09	0.05
31	->	26	-0.08	0.08
31	->	27	-0.01	0.01
31	->	28	-0.04	0.01
31	->	29	-1.20	0.72
31	->	30	-11.14	0.41
31	->	31	6.64	2.04
31	->	32	-0.50	0.36
31	->	33	-0.81	0.45
31	->	34	-1.49	1.40
31	->	35	-0.10	0.02
31	->	36	0.02	0.01

31	->	37	-0.00	0.00
31	->	38	-0.05	0.01
31	->	39	-0.05	0.04
31	->	40	0.08	0.03
31	->	41	-2.31	0.89
31	->	42	-0.75	0.81
31	->	43	-0.21	0.56
31	->	44	0.02	0.04
31	->	45	-0.03	0.01
31	->	46	-0.04	0.01
31	->	47	0.00	0.00
31	->	48	-0.01	0.01
31	->	49	-0.00	0.00
31	->	50	0.00	0.00
31	->	51	-0.00	0.00
31	->	52	-0.00	0.00
31	->	53	0.01	0.00
31	->	54	0.00	0.00
31	->	55	-0.00	0.00
31	->	56	0.00	0.00
31	->	57	0.00	0.00
31	->	58	-0.01	0.00
31	->	59	0.01	0.00
31	->	60	0.00	0.00
31	->	61	-0.00	0.00
31	->	62	-0.00	0.00
31	->	63	-0.00	0.00
31	->	64	-0.00	0.00
31	->	65	-0.00	0.00
31	->	66	-0.00	0.00
31	->	67	-0.00	0.00
31	->	68	0.00	0.00
31	->	69	0.00	0.00
31	->	70	0.00	0.00
31	->	71	0.00	0.00
31	->	72	0.01	0.00
31	->	73	0.00	0.00
31	->	74	-0.01	0.00
31	->	75	-0.01	0.00
31	->	76	0.00	0.00
31	->	77	0.00	0.00
31	->	78	0.01	0.00
31	->	79	0.00	0.00
31	->	80	0.00	0.00
31	->	81	0.00	0.00
31	->	82	0.00	0.00
31	->	83	0.00	0.00
31	->	84	0.00	0.00
31	->	85	0.00	0.00
31	->	86	-0.00	0.00

31	->	87	-0.01	0.00
31	->	88	-0.02	0.00
31	->	89	-0.03	0.01
31	->	90	-0.05	0.03
31	->	91	-0.03	0.01
31	->	92	-0.02	0.01
31	->	93	-0.01	0.00
31	->	94	-0.00	0.00
31	->	95	-0.00	0.00
31	->	96	-0.00	0.00
31	->	97	-0.01	0.00
31	->	98	-0.06	0.02
31	->	99	-0.07	0.03
31	->	100	-0.02	0.00
31	->	101	-0.01	0.00
31	->	102	-0.00	0.00
31	->	103	-0.00	0.00
31	->	104	-0.00	0.00
31	->	105	-0.01	0.00
31	->	106	-0.00	0.00
31	->	107	-0.01	0.00
32	->	1	0.00	0.00
32	->	2	0.00	0.00
32	->	3	-0.00	0.00
32	->	4	-0.00	0.00
32	->	5	-0.00	0.00
32	->	6	-0.00	0.00
32	->	7	-0.00	0.00
32	->	8	-0.00	0.00
32	->	9	0.00	0.00
32	->	10	-0.00	0.00
32	->	11	-0.00	0.00
32	->	12	0.00	0.00
32	->	13	0.00	0.00
32	->	14	-0.00	0.00
32	->	15	0.00	0.00
32	->	16	0.00	0.00
32	->	17	-0.00	0.00
32	->	18	-0.00	0.00
32	->	19	-0.01	0.00
32	->	20	-0.00	0.00
32	->	21	-0.02	0.01
32	->	22	-0.03	0.01
32	->	23	-0.01	0.00
32	->	24	-0.01	0.00
32	->	25	-1.52	0.34
32	->	26	-0.06	0.02
32	->	27	-0.01	0.00
32	->	28	-0.02	0.01
32	->	29	-0.03	0.00

32 ->	30	-1.32	0.33
32 ->	31	-0.59	0.37
32 ->	32	2.30	0.64
32 ->	33	-9.98	0.51
32 ->	34	-0.46	0.23
32 ->	35	-0.02	0.01
32 ->	36	-0.01	0.00
32 ->	37	-0.00	0.00
32 ->	38	-0.00	0.00
32 ->	39	-0.01	0.00
32 ->	40	-0.01	0.00
32 ->	41	-0.43	0.38
32 ->	42	-0.95	0.22
32 ->	43	-0.88	0.30
32 ->	44	-0.28	0.12
32 ->	45	-0.09	0.02
32 ->	46	-0.02	0.01
32 ->	47	-0.00	0.00
32 ->	48	-0.04	0.01
32 ->	49	-0.01	0.00
32 ->	50	-0.00	0.00
32 ->	51	-0.00	0.00
32 ->	52	-0.00	0.00
32 ->	53	-0.00	0.00
32 ->	54	-0.00	0.00
32 ->	55	-0.00	0.00
32 ->	56	-0.00	0.00
32 ->	57	-0.00	0.00
32 ->	58	0.00	0.00
32 ->	59	-0.00	0.00
32 ->	60	-0.00	0.00
32 ->	61	0.00	0.00
32 ->	62	0.00	0.00
32 ->	63	0.00	0.00
32 ->	64	0.00	0.00
32 ->	65	0.00	0.00
32 ->	66	0.00	0.00
32 ->	67	0.00	0.00
32 ->	68	0.00	0.00
32 ->	69	-0.00	0.00
32 ->	70	0.00	0.00
32 ->	71	0.00	0.00
32 ->	72	0.00	0.00
32 ->	73	0.00	0.00
32 ->	74	-0.00	0.00
32 ->	75	-0.00	0.00
32 ->	76	-0.00	0.00
32 ->	77	0.00	0.00
32 ->	78	0.00	0.00
32 ->	79	0.00	0.00

32	->	80	0.00	0.00
32	->	81	0.00	0.00
32	->	82	0.00	0.00
32	->	83	0.00	0.00
32	->	84	0.00	0.00
32	->	85	0.00	0.00
32	->	86	0.00	0.00
32	->	87	-0.00	0.00
32	->	88	-0.00	0.00
32	->	89	-0.00	0.00
32	->	90	-0.01	0.00
32	->	91	-0.01	0.00
32	->	92	-0.00	0.00
32	->	93	-0.00	0.00
32	->	94	-0.00	0.00
32	->	95	-0.00	0.00
32	->	96	-0.00	0.00
32	->	97	-0.01	0.01
32	->	98	-0.09	0.04
32	->	99	-0.18	0.07
32	->	100	-0.01	0.00
32	->	101	-0.00	0.00
32	->	102	-0.00	0.00
32	->	103	-0.00	0.00
32	->	104	-0.00	0.00
32	->	105	-0.00	0.00
32	->	106	-0.00	0.00
32	->	107	-0.00	0.00
33	->	1	-0.00	0.00
33	->	2	0.00	0.00
33	->	3	-0.00	0.00
33	->	4	0.00	0.00
33	->	5	0.00	0.00
33	->	6	-0.00	0.00
33	->	7	-0.00	0.00
33	->	8	0.00	0.00
33	->	9	0.00	0.00
33	->	10	0.00	0.00
33	->	11	0.00	0.00
33	->	12	-0.00	0.00
33	->	13	-0.00	0.00
33	->	14	-0.00	0.00
33	->	15	0.00	0.00
33	->	16	-0.00	0.00
33	->	17	-0.00	0.00
33	->	18	-0.01	0.00
33	->	19	-0.00	0.00
33	->	20	-0.00	0.00
33	->	21	-0.02	0.01
33	->	22	-0.05	0.02

33	->	23	-0.00	0.00
33	->	24	-0.01	0.00
33	->	25	-0.72	0.23
33	->	26	-0.01	0.00
33	->	27	-0.00	0.00
33	->	28	-0.00	0.00
33	->	29	-0.01	0.00
33	->	30	-0.10	0.03
33	->	31	-0.82	0.45
33	->	32	-10.12	0.52
33	->	33	27.81	1.36
33	->	34	-13.34	0.58
33	->	35	-0.50	0.12
33	->	36	-0.03	0.01
33	->	37	-0.01	0.00
33	->	38	-0.01	0.00
33	->	39	-0.03	0.02
33	->	40	-0.21	0.08
33	->	41	-2.32	0.63
33	->	42	-3.06	0.46
33	->	43	-0.60	0.24
33	->	44	-2.26	0.44
33	->	45	-2.55	0.32
33	->	46	0.04	0.03
33	->	47	-0.08	0.04
33	->	48	-1.61	0.38
33	->	49	-0.06	0.02
33	->	50	-0.01	0.00
33	->	51	-0.02	0.01
33	->	52	-0.02	0.00
33	->	53	-0.00	0.00
33	->	54	-0.00	0.00
33	->	55	-0.01	0.00
33	->	56	-0.00	0.00
33	->	57	0.00	0.00
33	->	58	-0.00	0.00
33	->	59	0.00	0.00
33	->	60	-0.00	0.00
33	->	61	-0.00	0.00
33	->	62	0.00	0.00
33	->	63	-0.00	0.00
33	->	64	-0.00	0.00
33	->	65	0.00	0.00
33	->	66	-0.00	0.00
33	->	67	-0.00	0.00
33	->	68	-0.00	0.00
33	->	69	0.00	0.00
33	->	70	0.00	0.00
33	->	71	-0.00	0.00
33	->	72	-0.00	0.00

33	->	73	-0.00	0.00
33	->	74	0.00	0.00
33	->	75	0.00	0.00
33	->	76	0.00	0.00
33	->	77	-0.00	0.00
33	->	78	-0.00	0.00
33	->	79	-0.00	0.00
33	->	80	-0.00	0.00
33	->	81	-0.00	0.00
33	->	82	0.00	0.00
33	->	83	0.00	0.00
33	->	84	0.00	0.00
33	->	85	-0.00	0.00
33	->	86	0.00	0.00
33	->	87	-0.00	0.00
33	->	88	-0.00	0.00
33	->	89	-0.00	0.00
33	->	90	-0.00	0.00
33	->	91	-0.00	0.00
33	->	92	-0.00	0.00
33	->	93	-0.00	0.00
33	->	94	-0.00	0.00
33	->	95	-0.00	0.00
33	->	96	-0.00	0.00
33	->	97	-0.01	0.00
33	->	98	-0.03	0.04
33	->	99	-1.22	0.30
33	->	100	-0.06	0.03
33	->	101	-0.01	0.00
33	->	102	-0.00	0.00
33	->	103	-0.00	0.00
33	->	104	-0.00	0.00
33	->	105	0.00	0.00
33	->	106	-0.00	0.00
33	->	107	0.00	0.00
34	->	1	0.00	0.00
34	->	2	-0.00	0.00
34	->	3	-0.00	0.00
34	->	4	0.00	0.00
34	->	5	-0.00	0.00
34	->	6	0.00	0.00
34	->	7	0.00	0.00
34	->	8	-0.00	0.00
34	->	9	0.00	0.00
34	->	10	-0.00	0.00
34	->	11	-0.00	0.00
34	->	12	0.00	0.00
34	->	13	0.00	0.00
34	->	14	-0.00	0.00
34	->	15	0.00	0.00

34	->	16	-0.00	0.00
34	->	17	-0.00	0.00
34	->	18	-0.00	0.00
34	->	19	-0.00	0.00
34	->	20	-0.00	0.00
34	->	21	-0.00	0.00
34	->	22	-0.00	0.00
34	->	23	-0.00	0.00
34	->	24	-0.00	0.00
34	->	25	-0.02	0.01
34	->	26	-0.00	0.00
34	->	27	-0.00	0.00
34	->	28	-0.00	0.00
34	->	29	-0.01	0.01
34	->	30	-0.02	0.02
34	->	31	-1.46	1.38
34	->	32	-0.46	0.23
34	->	33	-13.32	0.57
34	->	34	-55.15	2.38
34	->	35	-12.17	0.76
34	->	36	-0.53	0.42
34	->	37	-0.07	0.05
34	->	38	-0.10	0.04
34	->	39	-1.70	1.35
34	->	40	-1.98	0.43
34	->	41	-1.91	0.82
34	->	42	-0.85	0.34
34	->	43	-0.04	0.01
34	->	44	-0.03	0.01
34	->	45	-0.02	0.01
34	->	46	-0.00	0.00
34	->	47	0.02	0.02
34	->	48	-1.09	0.37
34	->	49	-0.02	0.01
34	->	50	0.00	0.00
34	->	51	-0.09	0.02
34	->	52	-0.06	0.04
34	->	53	-0.01	0.00
34	->	54	-0.00	0.00
34	->	55	-0.05	0.01
34	->	56	-0.00	0.00
34	->	57	-0.00	0.00
34	->	58	0.00	0.00
34	->	59	-0.00	0.00
34	->	60	0.00	0.00
34	->	61	0.00	0.00
34	->	62	0.00	0.00
34	->	63	-0.00	0.00
34	->	64	0.00	0.00
34	->	65	0.00	0.00

34	->	66	-0.00	0.00
34	->	67	0.00	0.00
34	->	68	0.00	0.00
34	->	69	-0.00	0.00
34	->	70	0.00	0.00
34	->	71	0.00	0.00
34	->	72	-0.00	0.00
34	->	73	0.00	0.00
34	->	74	-0.00	0.00
34	->	75	-0.00	0.00
34	->	76	0.00	0.00
34	->	77	-0.00	0.00
34	->	78	0.00	0.00
34	->	79	0.00	0.00
34	->	80	0.00	0.00
34	->	81	0.00	0.00
34	->	82	-0.00	0.00
34	->	83	0.00	0.00
34	->	84	0.00	0.00
34	->	85	-0.00	0.00
34	->	86	-0.00	0.00
34	->	87	-0.00	0.00
34	->	88	-0.00	0.00
34	->	89	-0.01	0.01
34	->	90	-0.01	0.01
34	->	91	-0.00	0.00
34	->	92	-0.00	0.00
34	->	93	-0.00	0.00
34	->	94	-0.00	0.00
34	->	95	0.00	0.00
34	->	96	0.00	0.00
34	->	97	-0.00	0.00
34	->	98	0.00	0.00
34	->	99	-0.00	0.00
34	->	100	-0.00	0.00
34	->	101	0.00	0.00
34	->	102	-0.00	0.00
34	->	103	0.00	0.00
34	->	104	-0.00	0.00
34	->	105	-0.00	0.00
34	->	106	-0.00	0.00
34	->	107	-0.00	0.00
35	->	1	-0.01	0.00
35	->	2	-0.00	0.00
35	->	3	-0.00	0.00
35	->	4	-0.00	0.00
35	->	5	0.01	0.00
35	->	6	-0.00	0.00
35	->	7	-0.00	0.00
35	->	8	0.00	0.00

35	->	9	-0.00	0.00
35	->	10	0.01	0.00
35	->	11	0.00	0.00
35	->	12	-0.01	0.00
35	->	13	-0.00	0.00
35	->	14	-0.00	0.00
35	->	15	-0.00	0.00
35	->	16	-0.01	0.00
35	->	17	0.00	0.00
35	->	18	0.00	0.00
35	->	19	0.01	0.00
35	->	20	0.00	0.00
35	->	21	0.00	0.00
35	->	22	-0.02	0.00
35	->	23	-0.00	0.00
35	->	24	0.00	0.00
35	->	25	-0.03	0.00
35	->	26	-0.00	0.00
35	->	27	-0.00	0.00
35	->	28	0.00	0.00
35	->	29	-0.00	0.00
35	->	30	-0.00	0.00
35	->	31	-0.10	0.02
35	->	32	-0.02	0.01
35	->	33	-0.50	0.12
35	->	34	-12.24	0.76
35	->	35	-25.13	2.08
35	->	36	-10.10	0.86
35	->	37	2.36	1.43
35	->	38	2.85	0.51
35	->	39	-2.22	0.62
35	->	40	-2.86	0.32
35	->	41	-0.34	0.07
35	->	42	-0.55	0.23
35	->	43	0.00	0.01
35	->	44	-0.06	0.01
35	->	45	-0.00	0.01
35	->	46	0.04	0.01
35	->	47	-0.12	0.03
35	->	48	-1.11	0.24
35	->	49	0.03	0.03
35	->	50	-0.07	0.03
35	->	51	2.58	0.65
35	->	52	-0.45	0.33
35	->	53	-0.05	0.02
35	->	54	-0.04	0.05
35	->	55	2.40	0.36
35	->	56	-0.01	0.01
35	->	57	-0.00	0.00
35	->	58	0.03	0.01

35	->	59	-0.03	0.00
35	->	60	-0.00	0.00
35	->	61	0.00	0.00
35	->	62	0.00	0.00
35	->	63	0.01	0.00
35	->	64	0.00	0.00
35	->	65	0.00	0.00
35	->	66	0.01	0.00
35	->	67	0.00	0.00
35	->	68	-0.01	0.00
35	->	69	-0.01	0.00
35	->	70	-0.00	0.00
35	->	71	-0.00	0.00
35	->	72	-0.03	0.00
35	->	73	-0.00	0.00
35	->	74	0.02	0.00
35	->	75	0.02	0.00
35	->	76	-0.01	0.00
35	->	77	-0.01	0.00
35	->	78	-0.01	0.00
35	->	79	-0.00	0.00
35	->	80	-0.00	0.00
35	->	81	-0.00	0.00
35	->	82	-0.00	0.00
35	->	83	0.00	0.00
35	->	84	-0.00	0.00
35	->	85	-0.00	0.00
35	->	86	0.00	0.00
35	->	87	0.02	0.00
35	->	88	0.06	0.02
35	->	89	0.13	0.06
35	->	90	0.04	0.01
35	->	91	0.02	0.00
35	->	92	0.01	0.00
35	->	93	0.01	0.00
35	->	94	0.00	0.00
35	->	95	0.01	0.00
35	->	96	0.00	0.00
35	->	97	0.00	0.00
35	->	98	0.02	0.00
35	->	99	0.03	0.00
35	->	100	0.03	0.00
35	->	101	0.01	0.00
35	->	102	0.01	0.00
35	->	103	0.01	0.00
35	->	104	0.01	0.00
35	->	105	0.01	0.00
35	->	106	0.00	0.00
35	->	107	0.01	0.00
36	->	1	0.00	0.00

36 ->	2	-0.00	0.00
36 ->	3	0.00	0.00
36 ->	4	0.00	0.00
36 ->	5	-0.00	0.00
36 ->	6	0.00	0.00
36 ->	7	0.00	0.00
36 ->	8	-0.00	0.00
36 ->	9	0.00	0.00
36 ->	10	-0.00	0.00
36 ->	11	-0.00	0.00
36 ->	12	0.00	0.00
36 ->	13	0.00	0.00
36 ->	14	0.00	0.00
36 ->	15	0.00	0.00
36 ->	16	0.01	0.00
36 ->	17	-0.00	0.00
36 ->	18	-0.00	0.00
36 ->	19	-0.01	0.00
36 ->	20	-0.00	0.00
36 ->	21	-0.00	0.00
36 ->	22	0.01	0.00
36 ->	23	0.00	0.00
36 ->	24	-0.00	0.00
36 ->	25	0.02	0.01
36 ->	26	-0.00	0.00
36 ->	27	0.00	0.00
36 ->	28	-0.00	0.00
36 ->	29	-0.00	0.00
36 ->	30	-0.00	0.00
36 ->	31	0.02	0.01
36 ->	32	-0.01	0.00
36 ->	33	-0.03	0.01
36 ->	34	-0.55	0.43
36 ->	35	-10.18	0.90
36 ->	36	-115.50	2.19
36 ->	37	-14.18	0.89
36 ->	38	-1.00	0.36
36 ->	39	-1.23	0.79
36 ->	40	-0.01	0.02
36 ->	41	-0.03	0.01
36 ->	42	-0.03	0.01
36 ->	43	-0.00	0.00
36 ->	44	0.01	0.00
36 ->	45	-0.01	0.00
36 ->	46	-0.03	0.01
36 ->	47	-0.01	0.00
36 ->	48	-0.13	0.15
36 ->	49	-0.02	0.02
36 ->	50	-0.00	0.00
36 ->	51	-0.15	0.05

36	->	52	-0.44	0.67
36	->	53	0.02	0.01
36	->	54	-0.00	0.00
36	->	55	-0.32	0.11
36	->	56	-0.02	0.02
36	->	57	-0.00	0.00
36	->	58	-0.04	0.02
36	->	59	0.02	0.00
36	->	60	0.00	0.00
36	->	61	-0.00	0.00
36	->	62	-0.00	0.00
36	->	63	-0.00	0.00
36	->	64	-0.00	0.00
36	->	65	-0.00	0.00
36	->	66	-0.00	0.00
36	->	67	-0.00	0.00
36	->	68	0.01	0.00
36	->	69	0.01	0.00
36	->	70	-0.00	0.00
36	->	71	-0.00	0.00
36	->	72	0.01	0.00
36	->	73	0.00	0.00
36	->	74	-0.01	0.00
36	->	75	-0.01	0.00
36	->	76	0.01	0.00
36	->	77	0.00	0.00
36	->	78	0.01	0.00
36	->	79	0.00	0.00
36	->	80	0.00	0.00
36	->	81	0.00	0.00
36	->	82	0.00	0.00
36	->	83	-0.00	0.00
36	->	84	0.00	0.00
36	->	85	0.00	0.00
36	->	86	-0.00	0.00
36	->	87	-0.01	0.00
36	->	88	-0.03	0.01
36	->	89	-0.03	0.01
36	->	90	-0.02	0.00
36	->	91	-0.01	0.00
36	->	92	-0.01	0.00
36	->	93	-0.00	0.00
36	->	94	-0.00	0.00
36	->	95	-0.00	0.00
36	->	96	-0.00	0.00
36	->	97	-0.00	0.00
36	->	98	-0.01	0.00
36	->	99	-0.02	0.01
36	->	100	-0.03	0.02
36	->	101	-0.02	0.01

36	->	102	-0.01	0.00
36	->	103	-0.00	0.00
36	->	104	-0.00	0.00
36	->	105	-0.00	0.00
36	->	106	-0.00	0.00
36	->	107	-0.00	0.00
37	->	1	-0.00	0.00
37	->	2	0.00	0.00
37	->	3	0.00	0.00
37	->	4	0.00	0.00
37	->	5	0.00	0.00
37	->	6	0.00	0.00
37	->	7	0.00	0.00
37	->	8	0.00	0.00
37	->	9	0.00	0.00
37	->	10	0.00	0.00
37	->	11	0.00	0.00
37	->	12	-0.00	0.00
37	->	13	-0.00	0.00
37	->	14	-0.00	0.00
37	->	15	-0.00	0.00
37	->	16	-0.00	0.00
37	->	17	0.00	0.00
37	->	18	0.00	0.00
37	->	19	0.00	0.00
37	->	20	0.00	0.00
37	->	21	0.00	0.00
37	->	22	-0.00	0.00
37	->	23	-0.00	0.00
37	->	24	0.00	0.00
37	->	25	-0.00	0.00
37	->	26	-0.00	0.00
37	->	27	-0.00	0.00
37	->	28	-0.00	0.00
37	->	29	-0.00	0.00
37	->	30	-0.00	0.00
37	->	31	-0.00	0.00
37	->	32	-0.00	0.00
37	->	33	-0.01	0.00
37	->	34	-0.07	0.05
37	->	35	2.30	1.43
37	->	36	-14.28	0.86
37	->	37	22.61	1.80
37	->	38	-7.96	1.09
37	->	39	-0.75	0.30
37	->	40	-0.27	0.09
37	->	41	-0.01	0.00
37	->	42	-0.02	0.01
37	->	43	-0.00	0.00
37	->	44	-0.00	0.00

37	->	45	-0.00	0.00
37	->	46	0.00	0.00
37	->	47	-0.01	0.00
37	->	48	-0.03	0.01
37	->	49	-0.00	0.00
37	->	50	-0.01	0.00
37	->	51	0.79	0.10
37	->	52	-0.15	0.14
37	->	53	-0.01	0.00
37	->	54	-0.03	0.01
37	->	55	1.09	0.72
37	->	56	-0.06	0.07
37	->	57	-0.00	0.00
37	->	58	-0.00	0.00
37	->	59	-0.00	0.00
37	->	60	-0.00	0.00
37	->	61	0.00	0.00
37	->	62	0.00	0.00
37	->	63	0.00	0.00
37	->	64	0.00	0.00
37	->	65	0.00	0.00
37	->	66	0.00	0.00
37	->	67	0.00	0.00
37	->	68	-0.00	0.00
37	->	69	-0.00	0.00
37	->	70	-0.00	0.00
37	->	71	-0.00	0.00
37	->	72	-0.00	0.00
37	->	73	0.00	0.00
37	->	74	0.00	0.00
37	->	75	0.00	0.00
37	->	76	-0.00	0.00
37	->	77	-0.00	0.00
37	->	78	-0.00	0.00
37	->	79	-0.00	0.00
37	->	80	-0.00	0.00
37	->	81	-0.00	0.00
37	->	82	0.00	0.00
37	->	83	0.00	0.00
37	->	84	0.00	0.00
37	->	85	-0.00	0.00
37	->	86	-0.00	0.00
37	->	87	-0.00	0.00
37	->	88	-0.00	0.00
37	->	89	0.01	0.01
37	->	90	0.00	0.00
37	->	91	0.00	0.00
37	->	92	0.00	0.00
37	->	93	0.00	0.00
37	->	94	0.00	0.00

37	->	95	0.00	0.00
37	->	96	0.00	0.00
37	->	97	0.00	0.00
37	->	98	0.00	0.00
37	->	99	0.00	0.00
37	->	100	-0.00	0.00
37	->	101	0.00	0.00
37	->	102	0.00	0.00
37	->	103	0.00	0.00
37	->	104	0.00	0.00
37	->	105	0.00	0.00
37	->	106	0.00	0.00
37	->	107	0.00	0.00
38	->	1	-0.01	0.00
38	->	2	0.00	0.00
38	->	3	0.00	0.00
38	->	4	-0.00	0.00
38	->	5	0.01	0.00
38	->	6	-0.00	0.00
38	->	7	0.00	0.00
38	->	8	0.00	0.00
38	->	9	-0.00	0.00
38	->	10	0.01	0.00
38	->	11	0.00	0.00
38	->	12	-0.01	0.00
38	->	13	-0.00	0.00
38	->	14	-0.00	0.00
38	->	15	-0.00	0.00
38	->	16	-0.01	0.00
38	->	17	0.00	0.00
38	->	18	0.00	0.00
38	->	19	0.01	0.00
38	->	20	0.00	0.00
38	->	21	0.00	0.00
38	->	22	-0.02	0.00
38	->	23	-0.00	0.00
38	->	24	0.00	0.00
38	->	25	-0.02	0.00
38	->	26	-0.00	0.00
38	->	27	-0.00	0.00
38	->	28	0.00	0.00
38	->	29	-0.00	0.00
38	->	30	0.00	0.00
38	->	31	-0.05	0.01
38	->	32	-0.00	0.00
38	->	33	-0.01	0.00
38	->	34	-0.10	0.04
38	->	35	2.85	0.51
38	->	36	-1.00	0.36
38	->	37	-7.90	1.10

38 ->	38	-31.32	3.37
38 ->	39	-11.70	0.73
38 ->	40	-4.81	2.51
38 ->	41	0.02	0.03
38 ->	42	0.01	0.04
38 ->	43	-0.00	0.00
38 ->	44	-0.03	0.00
38 ->	45	0.00	0.00
38 ->	46	0.03	0.00
38 ->	47	-0.03	0.01
38 ->	48	-0.01	0.01
38 ->	49	0.00	0.00
38 ->	50	-0.03	0.01
38 ->	51	3.60	0.84
38 ->	52	0.05	0.02
38 ->	53	-0.04	0.01
38 ->	54	-0.01	0.04
38 ->	55	2.17	0.36
38 ->	56	-0.01	0.00
38 ->	57	-0.00	0.00
38 ->	58	0.03	0.00
38 ->	59	-0.03	0.00
38 ->	60	-0.00	0.00
38 ->	61	0.00	0.00
38 ->	62	0.00	0.00
38 ->	63	0.01	0.00
38 ->	64	0.00	0.00
38 ->	65	0.00	0.00
38 ->	66	0.01	0.00
38 ->	67	0.00	0.00
38 ->	68	-0.01	0.00
38 ->	69	-0.01	0.00
38 ->	70	-0.00	0.00
38 ->	71	-0.00	0.00
38 ->	72	-0.03	0.00
38 ->	73	-0.00	0.00
38 ->	74	0.02	0.00
38 ->	75	0.02	0.00
38 ->	76	-0.01	0.00
38 ->	77	-0.01	0.00
38 ->	78	-0.01	0.00
38 ->	79	-0.00	0.00
38 ->	80	-0.00	0.00
38 ->	81	-0.01	0.00
38 ->	82	-0.00	0.00
38 ->	83	0.00	0.00
38 ->	84	-0.00	0.00
38 ->	85	-0.00	0.00
38 ->	86	0.00	0.00
38 ->	87	0.02	0.00

38	->	88	0.12	0.04
38	->	89	0.33	0.21
38	->	90	0.05	0.01
38	->	91	0.03	0.00
38	->	92	0.01	0.00
38	->	93	0.01	0.00
38	->	94	0.00	0.00
38	->	95	0.00	0.00
38	->	96	0.00	0.00
38	->	97	0.00	0.00
38	->	98	0.01	0.00
38	->	99	0.02	0.00
38	->	100	0.02	0.00
38	->	101	0.01	0.00
38	->	102	0.01	0.00
38	->	103	0.01	0.00
38	->	104	0.01	0.00
38	->	105	0.01	0.00
38	->	106	0.00	0.00
38	->	107	0.01	0.00
39	->	1	-0.00	0.00
39	->	2	0.00	0.00
39	->	3	0.00	0.00
39	->	4	0.00	0.00
39	->	5	0.00	0.00
39	->	6	-0.00	0.00
39	->	7	0.00	0.00
39	->	8	0.00	0.00
39	->	9	0.00	0.00
39	->	10	0.00	0.00
39	->	11	0.00	0.00
39	->	12	-0.00	0.00
39	->	13	-0.00	0.00
39	->	14	-0.00	0.00
39	->	15	0.00	0.00
39	->	16	-0.00	0.00
39	->	17	0.00	0.00
39	->	18	0.00	0.00
39	->	19	0.00	0.00
39	->	20	0.00	0.00
39	->	21	0.00	0.00
39	->	22	-0.00	0.00
39	->	23	0.00	0.00
39	->	24	0.00	0.00
39	->	25	-0.00	0.00
39	->	26	-0.00	0.00
39	->	27	-0.00	0.00
39	->	28	-0.00	0.00
39	->	29	-0.00	0.00
39	->	30	-0.00	0.00

39 ->	31	-0.05	0.04
39 ->	32	-0.01	0.00
39 ->	33	-0.03	0.02
39 ->	34	-1.68	1.36
39 ->	35	-2.24	0.62
39 ->	36	-1.23	0.78
39 ->	37	-0.75	0.30
39 ->	38	-11.82	0.73
39 ->	39	11.58	1.39
39 ->	40	-9.58	0.41
39 ->	41	-0.47	0.12
39 ->	42	-0.10	0.02
39 ->	43	-0.01	0.00
39 ->	44	-0.00	0.00
39 ->	45	-0.00	0.00
39 ->	46	-0.00	0.00
39 ->	47	0.00	0.00
39 ->	48	-0.02	0.01
39 ->	49	-0.00	0.00
39 ->	50	-0.00	0.00
39 ->	51	-0.08	0.05
39 ->	52	-0.02	0.01
39 ->	53	-0.00	0.00
39 ->	54	-0.00	0.00
39 ->	55	-0.03	0.04
39 ->	56	-0.00	0.00
39 ->	57	0.00	0.00
39 ->	58	-0.00	0.00
39 ->	59	0.00	0.00
39 ->	60	0.00	0.00
39 ->	61	0.00	0.00
39 ->	62	0.00	0.00
39 ->	63	-0.00	0.00
39 ->	64	-0.00	0.00
39 ->	65	0.00	0.00
39 ->	66	-0.00	0.00
39 ->	67	-0.00	0.00
39 ->	68	0.00	0.00
39 ->	69	0.00	0.00
39 ->	70	0.00	0.00
39 ->	71	0.00	0.00
39 ->	72	0.00	0.00
39 ->	73	0.00	0.00
39 ->	74	-0.00	0.00
39 ->	75	-0.00	0.00
39 ->	76	0.00	0.00
39 ->	77	0.00	0.00
39 ->	78	0.00	0.00
39 ->	79	0.00	0.00
39 ->	80	0.00	0.00

39	->	81	0.00	0.00
39	->	82	0.00	0.00
39	->	83	0.00	0.00
39	->	84	0.00	0.00
39	->	85	0.00	0.00
39	->	86	-0.00	0.00
39	->	87	-0.00	0.00
39	->	88	-0.00	0.00
39	->	89	-0.01	0.01
39	->	90	-0.00	0.00
39	->	91	-0.00	0.00
39	->	92	0.00	0.00
39	->	93	0.00	0.00
39	->	94	0.00	0.00
39	->	95	-0.00	0.00
39	->	96	0.00	0.00
39	->	97	0.00	0.00
39	->	98	0.00	0.00
39	->	99	-0.00	0.00
39	->	100	-0.00	0.00
39	->	101	-0.00	0.00
39	->	102	0.00	0.00
39	->	103	-0.00	0.00
39	->	104	0.00	0.00
39	->	105	0.00	0.00
39	->	106	0.00	0.00
39	->	107	-0.00	0.00
40	->	1	0.01	0.00
40	->	2	0.00	0.00
40	->	3	-0.00	0.00
40	->	4	0.00	0.00
40	->	5	-0.01	0.00
40	->	6	0.00	0.00
40	->	7	-0.00	0.00
40	->	8	-0.01	0.00
40	->	9	0.00	0.00
40	->	10	-0.01	0.00
40	->	11	-0.01	0.00
40	->	12	0.01	0.00
40	->	13	0.00	0.00
40	->	14	0.00	0.00
40	->	15	0.00	0.00
40	->	16	0.01	0.00
40	->	17	-0.00	0.00
40	->	18	-0.00	0.00
40	->	19	-0.02	0.00
40	->	20	-0.00	0.00
40	->	21	-0.00	0.00
40	->	22	0.02	0.00
40	->	23	-0.00	0.00

40	->	24	-0.00	0.00
40	->	25	0.04	0.01
40	->	26	-0.01	0.00
40	->	27	-0.00	0.00
40	->	28	-0.00	0.00
40	->	29	0.00	0.00
40	->	30	-0.01	0.01
40	->	31	0.08	0.03
40	->	32	-0.01	0.00
40	->	33	-0.21	0.08
40	->	34	-1.98	0.43
40	->	35	-2.86	0.32
40	->	36	-0.01	0.02
40	->	37	-0.27	0.09
40	->	38	-4.93	2.52
40	->	39	-9.65	0.42
40	->	40	-103.02	3.37
40	->	41	-15.17	0.83
40	->	42	-3.59	0.63
40	->	43	-0.07	0.03
40	->	44	0.04	0.02
40	->	45	-0.02	0.00
40	->	46	-0.05	0.01
40	->	47	-0.00	0.03
40	->	48	-0.20	0.05
40	->	49	-0.01	0.01
40	->	50	0.02	0.02
40	->	51	-1.64	0.62
40	->	52	-0.07	0.03
40	->	53	0.03	0.01
40	->	54	-0.05	0.05
40	->	55	-0.34	0.16
40	->	56	0.00	0.00
40	->	57	0.00	0.00
40	->	58	-0.02	0.00
40	->	59	0.02	0.01
40	->	60	0.00	0.00
40	->	61	-0.00	0.00
40	->	62	-0.00	0.00
40	->	63	-0.01	0.00
40	->	64	-0.00	0.00
40	->	65	-0.00	0.00
40	->	66	-0.01	0.00
40	->	67	-0.00	0.00
40	->	68	0.01	0.00
40	->	69	0.01	0.00
40	->	70	0.00	0.00
40	->	71	-0.00	0.00
40	->	72	0.05	0.02
40	->	73	-0.00	0.00

40	->	74	-0.03	0.01
40	->	75	-0.03	0.00
40	->	76	0.01	0.00
40	->	77	0.01	0.00
40	->	78	0.02	0.00
40	->	79	0.00	0.00
40	->	80	0.00	0.00
40	->	81	0.01	0.00
40	->	82	0.00	0.00
40	->	83	0.00	0.00
40	->	84	0.00	0.00
40	->	85	0.00	0.00
40	->	86	-0.01	0.00
40	->	87	-0.04	0.01
40	->	88	-1.08	0.53
40	->	89	-8.47	3.51
40	->	90	-1.41	1.97
40	->	91	-0.07	0.02
40	->	92	-0.03	0.00
40	->	93	-0.01	0.00
40	->	94	-0.01	0.00
40	->	95	-0.00	0.00
40	->	96	-0.00	0.00
40	->	97	-0.00	0.00
40	->	98	-0.02	0.00
40	->	99	-0.03	0.01
40	->	100	-0.02	0.00
40	->	101	-0.01	0.00
40	->	102	-0.01	0.00
40	->	103	-0.01	0.00
40	->	104	-0.01	0.00
40	->	105	-0.02	0.00
40	->	106	-0.01	0.00
40	->	107	-0.03	0.00
41	->	1	0.00	0.00
41	->	2	0.00	0.00
41	->	3	0.00	0.00
41	->	4	0.00	0.00
41	->	5	-0.00	0.00
41	->	6	-0.00	0.00
41	->	7	-0.00	0.00
41	->	8	-0.00	0.00
41	->	9	0.00	0.00
41	->	10	-0.00	0.00
41	->	11	0.00	0.00
41	->	12	0.00	0.00
41	->	13	0.00	0.00
41	->	14	-0.00	0.00
41	->	15	0.00	0.00
41	->	16	0.00	0.00

41	->	17	0.00	0.00
41	->	18	0.00	0.00
41	->	19	-0.00	0.00
41	->	20	-0.00	0.00
41	->	21	0.00	0.00
41	->	22	0.00	0.01
41	->	23	-0.01	0.00
41	->	24	0.00	0.00
41	->	25	0.06	0.05
41	->	26	-0.10	0.08
41	->	27	-0.01	0.01
41	->	28	-0.01	0.01
41	->	29	-0.04	0.10
41	->	30	-0.57	0.50
41	->	31	-2.24	0.88
41	->	32	-0.41	0.36
41	->	33	-2.32	0.63
41	->	34	-1.90	0.82
41	->	35	-0.34	0.07
41	->	36	-0.03	0.01
41	->	37	-0.01	0.00
41	->	38	0.02	0.03
41	->	39	-0.49	0.12
41	->	40	-15.14	0.83
41	->	41	-32.84	2.17
41	->	42	-9.11	0.59
41	->	43	-0.66	0.21
41	->	44	0.01	0.04
41	->	45	-0.04	0.01
41	->	46	-0.03	0.01
41	->	47	0.06	0.03
41	->	48	-0.08	0.04
41	->	49	-0.01	0.01
41	->	50	0.00	0.01
41	->	51	-0.02	0.01
41	->	52	-0.01	0.00
41	->	53	-0.00	0.00
41	->	54	-0.00	0.00
41	->	55	-0.01	0.01
41	->	56	-0.00	0.00
41	->	57	0.00	0.00
41	->	58	0.00	0.00
41	->	59	-0.00	0.00
41	->	60	0.00	0.00
41	->	61	0.00	0.00
41	->	62	0.00	0.00
41	->	63	0.00	0.00
41	->	64	0.00	0.00
41	->	65	0.00	0.00
41	->	66	0.00	0.00

41	->	67	0.00	0.00
41	->	68	-0.00	0.00
41	->	69	-0.00	0.00
41	->	70	0.00	0.00
41	->	71	0.00	0.00
41	->	72	-0.00	0.00
41	->	73	-0.00	0.00
41	->	74	0.00	0.00
41	->	75	0.00	0.00
41	->	76	-0.00	0.00
41	->	77	-0.00	0.00
41	->	78	-0.00	0.00
41	->	79	-0.00	0.00
41	->	80	-0.00	0.00
41	->	81	-0.00	0.00
41	->	82	-0.00	0.00
41	->	83	0.00	0.00
41	->	84	0.00	0.00
41	->	85	-0.00	0.00
41	->	86	0.00	0.00
41	->	87	-0.00	0.00
41	->	88	-0.00	0.00
41	->	89	-0.03	0.01
41	->	90	-0.04	0.02
41	->	91	-0.01	0.01
41	->	92	-0.00	0.00
41	->	93	-0.00	0.00
41	->	94	-0.00	0.00
41	->	95	-0.00	0.00
41	->	96	-0.00	0.00
41	->	97	-0.00	0.00
41	->	98	-0.01	0.01
41	->	99	-0.02	0.01
41	->	100	-0.00	0.00
41	->	101	0.00	0.00
41	->	102	-0.00	0.00
41	->	103	-0.00	0.00
41	->	104	-0.00	0.00
41	->	105	0.00	0.00
41	->	106	-0.00	0.00
41	->	107	-0.00	0.00
42	->	1	0.00	0.00
42	->	2	0.00	0.00
42	->	3	0.00	0.00
42	->	4	0.00	0.00
42	->	5	-0.00	0.00
42	->	6	-0.00	0.00
42	->	7	-0.00	0.00
42	->	8	-0.00	0.00
42	->	9	-0.00	0.00

42 ->	10	-0.00	0.00
42 ->	11	-0.00	0.00
42 ->	12	0.00	0.00
42 ->	13	0.00	0.00
42 ->	14	-0.00	0.00
42 ->	15	0.00	0.00
42 ->	16	0.00	0.00
42 ->	17	-0.00	0.00
42 ->	18	-0.00	0.00
42 ->	19	-0.01	0.00
42 ->	20	-0.00	0.00
42 ->	21	0.00	0.01
42 ->	22	0.00	0.02
42 ->	23	-0.02	0.01
42 ->	24	0.00	0.00
42 ->	25	0.05	0.06
42 ->	26	-0.08	0.05
42 ->	27	-0.01	0.00
42 ->	28	-0.01	0.00
42 ->	29	-0.00	0.01
42 ->	30	-0.11	0.09
42 ->	31	-0.74	0.81
42 ->	32	-0.94	0.22
42 ->	33	-3.07	0.46
42 ->	34	-0.85	0.34
42 ->	35	-0.54	0.23
42 ->	36	-0.03	0.01
42 ->	37	-0.02	0.01
42 ->	38	0.01	0.04
42 ->	39	-0.10	0.02
42 ->	40	-3.54	0.61
42 ->	41	-9.24	0.60
42 ->	42	28.52	1.46
42 ->	43	-17.41	0.36
42 ->	44	-1.12	0.23
42 ->	45	-0.22	0.05
42 ->	46	-0.16	0.04
42 ->	47	-2.15	0.41
42 ->	48	-2.82	0.39
42 ->	49	-0.07	0.02
42 ->	50	-0.13	0.04
42 ->	51	-1.85	0.28
42 ->	52	-0.08	0.03
42 ->	53	-0.02	0.00
42 ->	54	-0.02	0.01
42 ->	55	-0.00	0.01
42 ->	56	-0.00	0.00
42 ->	57	-0.00	0.00
42 ->	58	-0.00	0.00
42 ->	59	-0.00	0.00

42	->	60	-0.00	0.00
42	->	61	-0.00	0.00
42	->	62	0.00	0.00
42	->	63	-0.00	0.00
42	->	64	-0.00	0.00
42	->	65	-0.00	0.00
42	->	66	-0.00	0.00
42	->	67	0.00	0.00
42	->	68	0.00	0.00
42	->	69	0.00	0.00
42	->	70	-0.00	0.00
42	->	71	-0.00	0.00
42	->	72	-0.01	0.00
42	->	73	-0.00	0.00
42	->	74	-0.00	0.00
42	->	75	-0.00	0.00
42	->	76	0.00	0.00
42	->	77	0.00	0.00
42	->	78	0.00	0.00
42	->	79	0.00	0.00
42	->	80	0.00	0.00
42	->	81	0.00	0.00
42	->	82	0.00	0.00
42	->	83	0.00	0.00
42	->	84	0.00	0.00
42	->	85	0.00	0.00
42	->	86	-0.00	0.00
42	->	87	-0.00	0.00
42	->	88	-0.01	0.01
42	->	89	-0.16	0.24
42	->	90	-0.26	0.19
42	->	91	-0.05	0.02
42	->	92	-0.02	0.00
42	->	93	-0.01	0.00
42	->	94	-0.00	0.00
42	->	95	-0.00	0.00
42	->	96	-0.00	0.00
42	->	97	-0.00	0.00
42	->	98	-0.03	0.01
42	->	99	-0.07	0.03
42	->	100	-0.01	0.00
42	->	101	-0.00	0.00
42	->	102	-0.00	0.00
42	->	103	-0.00	0.00
42	->	104	-0.00	0.00
42	->	105	-0.00	0.00
42	->	106	-0.00	0.00
42	->	107	-0.00	0.00
43	->	1	0.00	0.00
43	->	2	-0.00	0.00

43	->	3	-0.00	0.00
43	->	4	-0.00	0.00
43	->	5	-0.00	0.00
43	->	6	-0.00	0.00
43	->	7	-0.00	0.00
43	->	8	-0.00	0.00
43	->	9	-0.00	0.00
43	->	10	-0.00	0.00
43	->	11	-0.00	0.00
43	->	12	0.00	0.00
43	->	13	-0.00	0.00
43	->	14	-0.01	0.00
43	->	15	-0.00	0.00
43	->	16	-0.00	0.00
43	->	17	-0.00	0.00
43	->	18	-0.01	0.01
43	->	19	-0.02	0.01
43	->	20	-0.02	0.01
43	->	21	-0.20	0.09
43	->	22	-1.64	0.78
43	->	23	-0.06	0.04
43	->	24	-0.03	0.02
43	->	25	-5.18	0.63
43	->	26	-1.04	0.18
43	->	27	-0.02	0.01
43	->	28	-0.02	0.01
43	->	29	-0.00	0.02
43	->	30	-0.31	0.63
43	->	31	-0.21	0.56
43	->	32	-0.87	0.29
43	->	33	-0.59	0.23
43	->	34	-0.04	0.01
43	->	35	0.00	0.01
43	->	36	-0.00	0.00
43	->	37	-0.00	0.00
43	->	38	-0.00	0.00
43	->	39	-0.01	0.00
43	->	40	-0.07	0.03
43	->	41	-0.67	0.21
43	->	42	-17.41	0.35
43	->	43	21.69	1.57
43	->	44	-7.08	0.77
43	->	45	-0.16	0.05
43	->	46	-0.10	0.04
43	->	47	-0.15	0.08
43	->	48	-0.02	0.01
43	->	49	-0.00	0.00
43	->	50	-0.01	0.00
43	->	51	-0.01	0.00
43	->	52	-0.00	0.00

43	->	53	-0.00	0.00
43	->	54	-0.00	0.00
43	->	55	-0.00	0.00
43	->	56	0.00	0.00
43	->	57	0.00	0.00
43	->	58	0.00	0.00
43	->	59	-0.00	0.00
43	->	60	-0.00	0.00
43	->	61	-0.00	0.00
43	->	62	0.00	0.00
43	->	63	-0.00	0.00
43	->	64	0.00	0.00
43	->	65	0.00	0.00
43	->	66	0.00	0.00
43	->	67	0.00	0.00
43	->	68	0.00	0.00
43	->	69	-0.00	0.00
43	->	70	0.00	0.00
43	->	71	0.00	0.00
43	->	72	-0.00	0.00
43	->	73	-0.00	0.00
43	->	74	-0.00	0.00
43	->	75	-0.00	0.00
43	->	76	-0.00	0.00
43	->	77	-0.00	0.00
43	->	78	0.00	0.00
43	->	79	-0.00	0.00
43	->	80	-0.00	0.00
43	->	81	0.00	0.00
43	->	82	0.00	0.00
43	->	83	0.00	0.00
43	->	84	0.00	0.00
43	->	85	0.00	0.00
43	->	86	-0.00	0.00
43	->	87	-0.00	0.00
43	->	88	-0.00	0.00
43	->	89	-0.00	0.00
43	->	90	-0.02	0.01
43	->	91	-0.04	0.03
43	->	92	-0.02	0.01
43	->	93	-0.01	0.00
43	->	94	0.00	0.00
43	->	95	0.00	0.00
43	->	96	0.00	0.00
43	->	97	-0.00	0.00
43	->	98	0.07	0.05
43	->	99	0.05	0.12
43	->	100	-0.02	0.01
43	->	101	0.00	0.00
43	->	102	-0.00	0.00

43	->	103	-0.00	0.00
43	->	104	-0.00	0.00
43	->	105	-0.00	0.00
43	->	106	-0.00	0.00
43	->	107	-0.00	0.00
44	->	1	0.05	0.01
44	->	2	0.00	0.00
44	->	3	-0.00	0.00
44	->	4	-0.00	0.00
44	->	5	-0.03	0.00
44	->	6	0.00	0.00
44	->	7	-0.00	0.00
44	->	8	-0.01	0.00
44	->	9	-0.00	0.00
44	->	10	-0.03	0.00
44	->	11	-0.02	0.01
44	->	12	0.04	0.01
44	->	13	0.01	0.00
44	->	14	-0.01	0.00
44	->	15	0.00	0.00
44	->	16	0.28	0.13
44	->	17	-0.00	0.01
44	->	18	0.01	0.02
44	->	19	-0.46	0.19
44	->	20	-0.02	0.01
44	->	21	0.06	0.03
44	->	22	-0.65	0.76
44	->	23	-0.05	0.02
44	->	24	-0.01	0.01
44	->	25	-0.19	0.25
44	->	26	-0.06	0.02
44	->	27	-0.00	0.00
44	->	28	0.00	0.00
44	->	29	-0.00	0.00
44	->	30	-0.02	0.01
44	->	31	0.02	0.04
44	->	32	-0.28	0.12
44	->	33	-2.27	0.44
44	->	34	-0.03	0.01
44	->	35	-0.06	0.01
44	->	36	0.01	0.00
44	->	37	-0.00	0.00
44	->	38	-0.03	0.00
44	->	39	-0.00	0.00
44	->	40	0.04	0.02
44	->	41	0.01	0.04
44	->	42	-1.13	0.23
44	->	43	-7.19	0.78
44	->	44	-91.64	2.22
44	->	45	-21.62	0.64

44 ->	46	-16.92	1.41
44 ->	47	-4.89	0.71
44 ->	48	-0.99	0.26
44 ->	49	0.04	0.05
44 ->	50	-0.05	0.06
44 ->	51	0.00	0.02
44 ->	52	-0.00	0.01
44 ->	53	0.05	0.01
44 ->	54	-0.00	0.00
44 ->	55	-0.00	0.00
44 ->	56	0.00	0.00
44 ->	57	0.00	0.00
44 ->	58	-0.02	0.00
44 ->	59	0.02	0.00
44 ->	60	0.00	0.00
44 ->	61	-0.00	0.00
44 ->	62	-0.00	0.00
44 ->	63	-0.01	0.00
44 ->	64	-0.01	0.00
44 ->	65	-0.00	0.00
44 ->	66	-0.01	0.00
44 ->	67	0.00	0.00
44 ->	68	0.01	0.00
44 ->	69	0.01	0.00
44 ->	70	0.00	0.00
44 ->	71	0.00	0.00
44 ->	72	0.11	0.03
44 ->	73	-0.01	0.01
44 ->	74	-0.08	0.02
44 ->	75	-0.03	0.01
44 ->	76	0.02	0.01
44 ->	77	0.03	0.00
44 ->	78	0.03	0.00
44 ->	79	0.00	0.00
44 ->	80	0.00	0.00
44 ->	81	0.02	0.00
44 ->	82	0.00	0.00
44 ->	83	0.00	0.00
44 ->	84	0.00	0.00
44 ->	85	0.00	0.00
44 ->	86	-0.00	0.00
44 ->	87	-0.02	0.00
44 ->	88	-0.03	0.01
44 ->	89	-0.08	0.01
44 ->	90	-0.19	0.05
44 ->	91	-1.97	0.62
44 ->	92	-8.72	0.72
44 ->	93	-1.89	0.45
44 ->	94	0.01	0.07
44 ->	95	-0.04	0.01

44	->	96	-0.02	0.00
44	->	97	-0.02	0.01
44	->	98	-0.80	0.37
44	->	99	-3.04	1.93
44	->	100	-0.58	0.23
44	->	101	1.31	0.54
44	->	102	0.25	0.36
44	->	103	-0.09	0.08
44	->	104	-0.06	0.02
44	->	105	-0.04	0.00
44	->	106	-0.02	0.00
44	->	107	-0.01	0.00
45	->	1	0.00	0.00
45	->	2	0.00	0.00
45	->	3	-0.00	0.00
45	->	4	-0.00	0.00
45	->	5	-0.00	0.00
45	->	6	0.00	0.00
45	->	7	0.00	0.00
45	->	8	-0.00	0.00
45	->	9	0.00	0.00
45	->	10	-0.00	0.00
45	->	11	-0.00	0.00
45	->	12	0.00	0.00
45	->	13	0.00	0.00
45	->	14	-0.00	0.00
45	->	15	0.00	0.00
45	->	16	-0.02	0.01
45	->	17	-0.00	0.00
45	->	18	-0.01	0.00
45	->	19	0.01	0.01
45	->	20	-0.00	0.00
45	->	21	-0.02	0.01
45	->	22	-0.21	0.12
45	->	23	0.00	0.00
45	->	24	-0.01	0.00
45	->	25	-0.11	0.20
45	->	26	0.00	0.00
45	->	27	0.00	0.00
45	->	28	-0.00	0.00
45	->	29	-0.00	0.00
45	->	30	-0.01	0.00
45	->	31	-0.03	0.01
45	->	32	-0.09	0.02
45	->	33	-2.62	0.32
45	->	34	-0.02	0.01
45	->	35	-0.00	0.01
45	->	36	-0.01	0.00
45	->	37	-0.00	0.00
45	->	38	0.00	0.00

45	->	39	-0.00	0.00
45	->	40	-0.02	0.00
45	->	41	-0.04	0.01
45	->	42	-0.22	0.05
45	->	43	-0.16	0.05
45	->	44	-21.65	0.63
45	->	45	25.59	1.87
45	->	46	-10.32	0.40
45	->	47	-0.92	0.21
45	->	48	-2.03	0.35
45	->	49	-3.45	1.55
45	->	50	-0.11	0.05
45	->	51	-0.05	0.03
45	->	52	-0.05	0.02
45	->	53	-0.02	0.01
45	->	54	-0.00	0.00
45	->	55	-0.00	0.00
45	->	56	-0.00	0.00
45	->	57	-0.00	0.00
45	->	58	0.00	0.00
45	->	59	-0.00	0.00
45	->	60	-0.00	0.00
45	->	61	-0.00	0.00
45	->	62	0.00	0.00
45	->	63	0.00	0.00
45	->	64	0.00	0.00
45	->	65	0.00	0.00
45	->	66	0.00	0.00
45	->	67	0.00	0.00
45	->	68	-0.00	0.00
45	->	69	-0.00	0.00
45	->	70	0.00	0.00
45	->	71	-0.00	0.00
45	->	72	-0.01	0.00
45	->	73	-0.00	0.00
45	->	74	0.00	0.00
45	->	75	0.00	0.00
45	->	76	-0.00	0.00
45	->	77	-0.00	0.00
45	->	78	-0.00	0.00
45	->	79	-0.00	0.00
45	->	80	-0.00	0.00
45	->	81	-0.00	0.00
45	->	82	-0.00	0.00
45	->	83	0.00	0.00
45	->	84	-0.00	0.00
45	->	85	0.00	0.00
45	->	86	0.00	0.00
45	->	87	0.00	0.00
45	->	88	0.00	0.00

45	->	89	-0.00	0.00
45	->	90	-0.00	0.00
45	->	91	-0.00	0.00
45	->	92	-0.00	0.00
45	->	93	0.00	0.01
45	->	94	-0.01	0.01
45	->	95	-0.00	0.00
45	->	96	-0.00	0.00
45	->	97	-0.01	0.00
45	->	98	-0.33	0.48
45	->	99	-5.33	3.83
45	->	100	-3.06	1.88
45	->	101	-0.07	0.02
45	->	102	-0.01	0.00
45	->	103	-0.00	0.00
45	->	104	-0.00	0.00
45	->	105	-0.00	0.00
45	->	106	-0.00	0.00
45	->	107	0.00	0.00
46	->	1	-0.03	0.00
46	->	2	-0.00	0.00
46	->	3	-0.00	0.00
46	->	4	0.00	0.00
46	->	5	0.01	0.00
46	->	6	-0.00	0.00
46	->	7	-0.00	0.00
46	->	8	0.01	0.00
46	->	9	0.00	0.00
46	->	10	0.02	0.00
46	->	11	0.01	0.00
46	->	12	-0.03	0.00
46	->	13	-0.01	0.00
46	->	14	-0.00	0.00
46	->	15	-0.00	0.00
46	->	16	-0.14	0.07
46	->	17	-0.00	0.00
46	->	18	-0.02	0.01
46	->	19	0.11	0.04
46	->	20	-0.00	0.00
46	->	21	-0.03	0.01
46	->	22	-2.31	1.27
46	->	23	-0.00	0.00
46	->	24	-0.00	0.00
46	->	25	-0.27	0.10
46	->	26	-0.00	0.00
46	->	27	-0.00	0.00
46	->	28	-0.00	0.00
46	->	29	-0.00	0.00
46	->	30	0.00	0.00
46	->	31	-0.04	0.01

46	->	32	-0.02	0.01
46	->	33	0.04	0.03
46	->	34	-0.00	0.00
46	->	35	0.04	0.01
46	->	36	-0.03	0.01
46	->	37	0.00	0.00
46	->	38	0.03	0.00
46	->	39	-0.00	0.00
46	->	40	-0.05	0.01
46	->	41	-0.03	0.01
46	->	42	-0.16	0.04
46	->	43	-0.10	0.04
46	->	44	-16.87	1.41
46	->	45	-10.38	0.39
46	->	46	-8.98	1.83
46	->	47	-13.13	0.65
46	->	48	-1.46	0.20
46	->	49	-2.94	0.41
46	->	50	-2.12	0.90
46	->	51	-0.18	0.10
46	->	52	-0.07	0.03
46	->	53	-0.19	0.07
46	->	54	-0.02	0.00
46	->	55	-0.01	0.00
46	->	56	-0.01	0.00
46	->	57	-0.00	0.00
46	->	58	0.03	0.01
46	->	59	-0.03	0.00
46	->	60	-0.01	0.00
46	->	61	-0.01	0.01
46	->	62	0.00	0.00
46	->	63	0.01	0.00
46	->	64	0.01	0.00
46	->	65	0.00	0.00
46	->	66	0.01	0.00
46	->	67	0.00	0.00
46	->	68	-0.01	0.00
46	->	69	-0.02	0.00
46	->	70	-0.00	0.00
46	->	71	-0.01	0.01
46	->	72	-0.12	0.02
46	->	73	-0.01	0.01
46	->	74	0.06	0.01
46	->	75	0.03	0.00
46	->	76	-0.03	0.01
46	->	77	-0.03	0.01
46	->	78	-0.03	0.00
46	->	79	-0.00	0.00
46	->	80	-0.00	0.00
46	->	81	-0.02	0.00

46	->	82	-0.00	0.00
46	->	83	-0.00	0.00
46	->	84	-0.00	0.00
46	->	85	-0.00	0.00
46	->	86	0.00	0.00
46	->	87	0.02	0.00
46	->	88	0.02	0.00
46	->	89	0.03	0.01
46	->	90	-0.02	0.03
46	->	91	0.08	0.07
46	->	92	0.67	0.21
46	->	93	0.43	0.15
46	->	94	-0.04	0.05
46	->	95	0.02	0.00
46	->	96	0.02	0.00
46	->	97	0.01	0.00
46	->	98	0.30	0.15
46	->	99	0.44	0.45
46	->	100	-1.50	0.43
46	->	101	-5.59	1.14
46	->	102	-0.89	0.51
46	->	103	-0.05	0.07
46	->	104	0.01	0.01
46	->	105	0.02	0.00
46	->	106	0.01	0.00
46	->	107	0.01	0.00
47	->	1	0.00	0.00
47	->	2	0.00	0.00
47	->	3	-0.00	0.00
47	->	4	0.00	0.00
47	->	5	-0.00	0.00
47	->	6	0.00	0.00
47	->	7	0.00	0.00
47	->	8	-0.00	0.00
47	->	9	0.00	0.00
47	->	10	-0.00	0.00
47	->	11	-0.00	0.00
47	->	12	0.00	0.00
47	->	13	0.00	0.00
47	->	14	-0.00	0.00
47	->	15	-0.00	0.00
47	->	16	-0.01	0.01
47	->	17	-0.00	0.00
47	->	18	-0.01	0.00
47	->	19	-0.01	0.01
47	->	20	-0.00	0.00
47	->	21	-0.01	0.00
47	->	22	-0.21	0.07
47	->	23	-0.00	0.00
47	->	24	-0.00	0.00

47 ->	25	-0.02	0.01
47 ->	26	-0.01	0.01
47 ->	27	-0.00	0.00
47 ->	28	-0.00	0.00
47 ->	29	-0.00	0.00
47 ->	30	-0.00	0.00
47 ->	31	0.00	0.00
47 ->	32	-0.00	0.00
47 ->	33	-0.08	0.04
47 ->	34	0.02	0.02
47 ->	35	-0.12	0.03
47 ->	36	-0.01	0.00
47 ->	37	-0.01	0.00
47 ->	38	-0.03	0.01
47 ->	39	0.00	0.00
47 ->	40	-0.00	0.03
47 ->	41	0.06	0.03
47 ->	42	-2.19	0.42
47 ->	43	-0.15	0.08
47 ->	44	-4.83	0.71
47 ->	45	-0.92	0.21
47 ->	46	-13.19	0.66
47 ->	47	20.47	1.42
47 ->	48	-6.43	0.41
47 ->	49	-0.96	0.17
47 ->	50	-1.91	0.31
47 ->	51	-2.05	0.51
47 ->	52	-0.03	0.04
47 ->	53	-0.04	0.04
47 ->	54	-0.03	0.01
47 ->	55	-0.03	0.01
47 ->	56	-0.01	0.00
47 ->	57	-0.00	0.00
47 ->	58	0.00	0.00
47 ->	59	-0.01	0.00
47 ->	60	-0.00	0.00
47 ->	61	-0.00	0.00
47 ->	62	0.00	0.00
47 ->	63	0.00	0.00
47 ->	64	0.00	0.00
47 ->	65	0.00	0.00
47 ->	66	0.00	0.00
47 ->	67	0.00	0.00
47 ->	68	-0.00	0.00
47 ->	69	-0.00	0.00
47 ->	70	-0.00	0.00
47 ->	71	-0.01	0.01
47 ->	72	-0.12	0.07
47 ->	73	-0.00	0.00
47 ->	74	-0.00	0.01

47	->	75	-0.00	0.00
47	->	76	-0.00	0.00
47	->	77	-0.00	0.00
47	->	78	-0.00	0.00
47	->	79	-0.00	0.00
47	->	80	0.00	0.00
47	->	81	0.00	0.00
47	->	82	0.00	0.00
47	->	83	0.00	0.00
47	->	84	0.00	0.00
47	->	85	-0.00	0.00
47	->	86	-0.00	0.00
47	->	87	-0.01	0.00
47	->	88	-0.05	0.01
47	->	89	-0.23	0.07
47	->	90	-2.25	0.66
47	->	91	-3.19	0.73
47	->	92	-0.43	0.23
47	->	93	-0.03	0.02
47	->	94	-0.01	0.01
47	->	95	-0.00	0.00
47	->	96	-0.00	0.00
47	->	97	-0.00	0.00
47	->	98	0.01	0.01
47	->	99	-0.02	0.03
47	->	100	-0.07	0.04
47	->	101	-0.24	0.10
47	->	102	-0.19	0.13
47	->	103	-0.07	0.05
47	->	104	-0.06	0.03
47	->	105	-0.02	0.00
47	->	106	-0.01	0.00
47	->	107	-0.00	0.00
48	->	1	0.00	0.00
48	->	2	0.00	0.00
48	->	3	0.00	0.00
48	->	4	0.00	0.00
48	->	5	-0.00	0.00
48	->	6	0.00	0.00
48	->	7	0.00	0.00
48	->	8	-0.00	0.00
48	->	9	0.00	0.00
48	->	10	-0.00	0.00
48	->	11	-0.00	0.00
48	->	12	0.00	0.00
48	->	13	0.00	0.00
48	->	14	0.00	0.00
48	->	15	0.00	0.00
48	->	16	0.00	0.00
48	->	17	-0.00	0.00

48	->	18	-0.00	0.00
48	->	19	-0.00	0.00
48	->	20	-0.00	0.00
48	->	21	-0.00	0.00
48	->	22	-0.01	0.00
48	->	23	-0.00	0.00
48	->	24	-0.00	0.00
48	->	25	-0.01	0.01
48	->	26	-0.00	0.00
48	->	27	-0.00	0.00
48	->	28	-0.00	0.00
48	->	29	-0.00	0.00
48	->	30	-0.00	0.00
48	->	31	-0.01	0.01
48	->	32	-0.04	0.01
48	->	33	-1.61	0.40
48	->	34	-1.12	0.38
48	->	35	-1.10	0.25
48	->	36	-0.13	0.15
48	->	37	-0.03	0.01
48	->	38	-0.01	0.01
48	->	39	-0.02	0.01
48	->	40	-0.20	0.05
48	->	41	-0.08	0.04
48	->	42	-2.89	0.40
48	->	43	-0.02	0.01
48	->	44	-1.00	0.26
48	->	45	-2.05	0.35
48	->	46	-1.46	0.20
48	->	47	-6.48	0.41
48	->	48	22.33	0.99
48	->	49	-6.46	0.58
48	->	50	-1.00	0.15
48	->	51	-2.95	0.39
48	->	52	-4.13	0.55
48	->	53	-0.15	0.07
48	->	54	-0.07	0.04
48	->	55	-0.06	0.02
48	->	56	-0.02	0.01
48	->	57	-0.00	0.00
48	->	58	-0.00	0.01
48	->	59	-0.00	0.00
48	->	60	-0.00	0.00
48	->	61	-0.01	0.00
48	->	62	-0.00	0.00
48	->	63	0.00	0.00
48	->	64	0.00	0.00
48	->	65	0.00	0.00
48	->	66	0.00	0.00
48	->	67	0.00	0.00

48	->	68	-0.00	0.00
48	->	69	-0.00	0.00
48	->	70	0.00	0.00
48	->	71	-0.01	0.00
48	->	72	-0.01	0.01
48	->	73	-0.00	0.00
48	->	74	-0.00	0.00
48	->	75	0.00	0.00
48	->	76	-0.00	0.00
48	->	77	-0.00	0.00
48	->	78	-0.00	0.00
48	->	79	-0.00	0.00
48	->	80	0.00	0.00
48	->	81	-0.00	0.00
48	->	82	-0.00	0.00
48	->	83	0.00	0.00
48	->	84	0.00	0.00
48	->	85	0.00	0.00
48	->	86	-0.00	0.00
48	->	87	-0.00	0.00
48	->	88	-0.00	0.01
48	->	89	0.01	0.02
48	->	90	-0.01	0.01
48	->	91	-0.00	0.00
48	->	92	-0.00	0.00
48	->	93	-0.00	0.00
48	->	94	-0.00	0.00
48	->	95	-0.00	0.00
48	->	96	-0.00	0.00
48	->	97	-0.00	0.00
48	->	98	-0.02	0.01
48	->	99	-0.07	0.04
48	->	100	-0.08	0.02
48	->	101	-0.02	0.01
48	->	102	-0.01	0.00
48	->	103	-0.00	0.00
48	->	104	-0.00	0.00
48	->	105	-0.00	0.00
48	->	106	-0.00	0.00
48	->	107	-0.00	0.00
49	->	1	0.00	0.00
49	->	2	0.00	0.00
49	->	3	0.00	0.00
49	->	4	-0.00	0.00
49	->	5	-0.00	0.00
49	->	6	0.00	0.00
49	->	7	0.00	0.00
49	->	8	-0.00	0.00
49	->	9	0.00	0.00
49	->	10	-0.00	0.00

49 ->	11	-0.00	0.00
49 ->	12	0.00	0.00
49 ->	13	0.00	0.00
49 ->	14	0.00	0.00
49 ->	15	0.00	0.00
49 ->	16	0.00	0.00
49 ->	17	-0.00	0.00
49 ->	18	-0.00	0.00
49 ->	19	-0.00	0.00
49 ->	20	-0.00	0.00
49 ->	21	-0.00	0.00
49 ->	22	0.01	0.00
49 ->	23	0.00	0.00
49 ->	24	-0.00	0.00
49 ->	25	0.00	0.00
49 ->	26	-0.00	0.00
49 ->	27	0.00	0.00
49 ->	28	0.00	0.00
49 ->	29	0.00	0.00
49 ->	30	-0.00	0.00
49 ->	31	-0.00	0.00
49 ->	32	-0.01	0.00
49 ->	33	-0.06	0.02
49 ->	34	-0.02	0.01
49 ->	35	0.03	0.03
49 ->	36	-0.02	0.02
49 ->	37	-0.00	0.00
49 ->	38	0.00	0.00
49 ->	39	-0.00	0.00
49 ->	40	-0.01	0.01
49 ->	41	-0.01	0.01
49 ->	42	-0.07	0.02
49 ->	43	-0.00	0.00
49 ->	44	0.04	0.05
49 ->	45	-3.45	1.55
49 ->	46	-2.96	0.41
49 ->	47	-0.97	0.17
49 ->	48	-6.48	0.58
49 ->	49	-20.63	2.15
49 ->	50	-8.09	0.34
49 ->	51	-0.91	0.25
49 ->	52	-2.74	0.51
49 ->	53	-3.23	1.03
49 ->	54	-0.15	0.09
49 ->	55	-0.04	0.01
49 ->	56	-0.03	0.03
49 ->	57	-0.01	0.00
49 ->	58	-0.03	0.02
49 ->	59	-0.00	0.01
49 ->	60	-0.02	0.01

49	->	61	-0.03	0.01
49	->	62	-0.00	0.00
49	->	63	0.00	0.00
49	->	64	-0.00	0.00
49	->	65	0.00	0.00
49	->	66	0.00	0.00
49	->	67	0.00	0.00
49	->	68	-0.00	0.00
49	->	69	-0.00	0.00
49	->	70	0.00	0.00
49	->	71	-0.05	0.04
49	->	72	0.02	0.02
49	->	73	-0.02	0.01
49	->	74	-0.02	0.01
49	->	75	-0.00	0.00
49	->	76	-0.00	0.00
49	->	77	0.00	0.00
49	->	78	-0.00	0.00
49	->	79	0.00	0.00
49	->	80	0.00	0.00
49	->	81	0.00	0.00
49	->	82	0.00	0.00
49	->	83	0.00	0.00
49	->	84	0.00	0.00
49	->	85	0.00	0.00
49	->	86	-0.00	0.00
49	->	87	-0.00	0.01
49	->	88	-0.02	0.01
49	->	89	-0.03	0.02
49	->	90	-0.02	0.01
49	->	91	-0.01	0.00
49	->	92	-0.01	0.00
49	->	93	-0.00	0.00
49	->	94	-0.00	0.00
49	->	95	-0.00	0.00
49	->	96	-0.00	0.00
49	->	97	-0.00	0.00
49	->	98	-0.01	0.00
49	->	99	-0.03	0.02
49	->	100	-0.15	0.09
49	->	101	-0.12	0.08
49	->	102	-0.02	0.01
49	->	103	-0.00	0.00
49	->	104	-0.00	0.00
49	->	105	-0.00	0.00
49	->	106	-0.00	0.00
49	->	107	-0.00	0.00
50	->	1	0.00	0.00
50	->	2	0.00	0.00
50	->	3	-0.00	0.00

50	->	4	0.00	0.00
50	->	5	-0.00	0.00
50	->	6	0.00	0.00
50	->	7	0.00	0.00
50	->	8	-0.00	0.00
50	->	9	-0.00	0.00
50	->	10	-0.00	0.00
50	->	11	-0.00	0.00
50	->	12	0.00	0.00
50	->	13	0.00	0.00
50	->	14	0.00	0.00
50	->	15	0.00	0.00
50	->	16	0.00	0.00
50	->	17	-0.00	0.00
50	->	18	-0.00	0.00
50	->	19	-0.00	0.00
50	->	20	-0.00	0.00
50	->	21	-0.00	0.00
50	->	22	-0.00	0.00
50	->	23	0.00	0.00
50	->	24	0.00	0.00
50	->	25	0.00	0.00
50	->	26	-0.00	0.00
50	->	27	0.00	0.00
50	->	28	0.00	0.00
50	->	29	0.00	0.00
50	->	30	-0.00	0.00
50	->	31	0.00	0.00
50	->	32	-0.00	0.00
50	->	33	-0.01	0.00
50	->	34	0.00	0.00
50	->	35	-0.07	0.03
50	->	36	-0.00	0.00
50	->	37	-0.01	0.00
50	->	38	-0.03	0.01
50	->	39	-0.00	0.00
50	->	40	0.02	0.02
50	->	41	0.00	0.01
50	->	42	-0.13	0.04
50	->	43	-0.01	0.00
50	->	44	-0.05	0.06
50	->	45	-0.11	0.05
50	->	46	-2.12	0.89
50	->	47	-1.91	0.33
50	->	48	-1.01	0.15
50	->	49	-8.11	0.34
50	->	50	-21.71	1.70
50	->	51	-21.66	0.57
50	->	52	-0.88	0.14
50	->	53	-3.53	0.56

50	->	54	-2.40	1.41
50	->	55	-0.14	0.07
50	->	56	-0.04	0.02
50	->	57	-0.03	0.01
50	->	58	-0.01	0.01
50	->	59	-0.04	0.02
50	->	60	-0.03	0.01
50	->	61	-0.03	0.03
50	->	62	-0.00	0.00
50	->	63	0.00	0.00
50	->	64	0.00	0.00
50	->	65	0.00	0.00
50	->	66	0.00	0.00
50	->	67	0.00	0.00
50	->	68	-0.00	0.00
50	->	69	-0.01	0.00
50	->	70	-0.03	0.02
50	->	71	-0.43	0.27
50	->	72	-1.42	0.33
50	->	73	-0.00	0.03
50	->	74	-0.01	0.02
50	->	75	-0.02	0.01
50	->	76	-0.01	0.00
50	->	77	-0.00	0.00
50	->	78	-0.00	0.00
50	->	79	-0.00	0.00
50	->	80	-0.00	0.00
50	->	81	0.00	0.00
50	->	82	-0.00	0.00
50	->	83	0.00	0.00
50	->	84	-0.00	0.00
50	->	85	-0.00	0.00
50	->	86	-0.00	0.00
50	->	87	-0.02	0.01
50	->	88	-0.13	0.08
50	->	89	-0.31	0.21
50	->	90	-0.33	0.29
50	->	91	-0.03	0.01
50	->	92	-0.01	0.00
50	->	93	-0.01	0.00
50	->	94	-0.00	0.00
50	->	95	-0.00	0.00
50	->	96	-0.00	0.00
50	->	97	-0.00	0.00
50	->	98	-0.00	0.00
50	->	99	-0.01	0.01
50	->	100	-0.02	0.01
50	->	101	-0.04	0.03
50	->	102	-0.03	0.01
50	->	103	-0.03	0.02

50 ->	104	-0.02	0.01
50 ->	105	-0.01	0.00
50 ->	106	-0.00	0.00
50 ->	107	-0.00	0.00
51 ->	1	0.00	0.00
51 ->	2	0.00	0.00
51 ->	3	0.00	0.00
51 ->	4	0.00	0.00
51 ->	5	-0.00	0.00
51 ->	6	0.00	0.00
51 ->	7	0.00	0.00
51 ->	8	-0.00	0.00
51 ->	9	0.00	0.00
51 ->	10	-0.00	0.00
51 ->	11	-0.00	0.00
51 ->	12	0.00	0.00
51 ->	13	0.00	0.00
51 ->	14	0.00	0.00
51 ->	15	0.00	0.00
51 ->	16	0.00	0.00
51 ->	17	-0.00	0.00
51 ->	18	-0.00	0.00
51 ->	19	-0.00	0.00
51 ->	20	-0.00	0.00
51 ->	21	0.00	0.00
51 ->	22	0.00	0.00
51 ->	23	-0.00	0.00
51 ->	24	0.00	0.00
51 ->	25	0.00	0.00
51 ->	26	-0.00	0.00
51 ->	27	0.00	0.00
51 ->	28	0.00	0.00
51 ->	29	0.00	0.00
51 ->	30	-0.00	0.00
51 ->	31	-0.00	0.00
51 ->	32	-0.00	0.00
51 ->	33	-0.02	0.01
51 ->	34	-0.09	0.02
51 ->	35	2.55	0.65
51 ->	36	-0.15	0.05
51 ->	37	0.79	0.10
51 ->	38	3.57	0.84
51 ->	39	-0.08	0.05
51 ->	40	-1.60	0.61
51 ->	41	-0.02	0.01
51 ->	42	-1.86	0.28
51 ->	43	-0.01	0.00
51 ->	44	0.00	0.02
51 ->	45	-0.05	0.03
51 ->	46	-0.18	0.10

51 ->	47	-2.09	0.51
51 ->	48	-2.95	0.39
51 ->	49	-0.91	0.25
51 ->	50	-21.70	0.57
51 ->	51	11.44	1.52
51 ->	52	-10.40	0.52
51 ->	53	-1.20	0.27
51 ->	54	-3.38	1.64
51 ->	55	12.88	6.02
51 ->	56	-0.17	0.07
51 ->	57	-0.03	0.01
51 ->	58	-0.02	0.01
51 ->	59	-0.01	0.01
51 ->	60	-0.01	0.00
51 ->	61	-0.00	0.00
51 ->	62	-0.00	0.00
51 ->	63	-0.00	0.00
51 ->	64	-0.00	0.00
51 ->	65	-0.00	0.00
51 ->	66	-0.00	0.00
51 ->	67	0.00	0.00
51 ->	68	-0.00	0.00
51 ->	69	-0.00	0.00
51 ->	70	-0.01	0.00
51 ->	71	-0.02	0.01
51 ->	72	-0.06	0.02
51 ->	73	0.00	0.00
51 ->	74	-0.00	0.00
51 ->	75	-0.01	0.00
51 ->	76	0.00	0.00
51 ->	77	0.00	0.00
51 ->	78	0.00	0.00
51 ->	79	0.00	0.00
51 ->	80	0.00	0.00
51 ->	81	0.00	0.00
51 ->	82	0.00	0.00
51 ->	83	0.00	0.00
51 ->	84	0.00	0.00
51 ->	85	0.00	0.00
51 ->	86	-0.00	0.00
51 ->	87	-0.02	0.01
51 ->	88	-0.52	0.39
51 ->	89	-3.80	3.09
51 ->	90	-0.07	0.03
51 ->	91	-0.01	0.00
51 ->	92	-0.01	0.00
51 ->	93	-0.00	0.00
51 ->	94	-0.00	0.00
51 ->	95	-0.00	0.00
51 ->	96	-0.00	0.00

51	->	97	-0.00	0.00
51	->	98	-0.00	0.00
51	->	99	-0.01	0.00
51	->	100	-0.01	0.00
51	->	101	-0.01	0.00
51	->	102	-0.00	0.00
51	->	103	-0.00	0.00
51	->	104	-0.01	0.00
51	->	105	-0.00	0.00
51	->	106	-0.00	0.00
51	->	107	-0.00	0.00
52	->	1	0.00	0.00
52	->	2	0.00	0.00
52	->	3	0.00	0.00
52	->	4	0.00	0.00
52	->	5	-0.00	0.00
52	->	6	0.00	0.00
52	->	7	0.00	0.00
52	->	8	-0.00	0.00
52	->	9	0.00	0.00
52	->	10	-0.00	0.00
52	->	11	-0.00	0.00
52	->	12	0.00	0.00
52	->	13	0.00	0.00
52	->	14	0.00	0.00
52	->	15	0.00	0.00
52	->	16	0.00	0.00
52	->	17	-0.00	0.00
52	->	18	-0.00	0.00
52	->	19	-0.00	0.00
52	->	20	-0.00	0.00
52	->	21	-0.00	0.00
52	->	22	0.00	0.00
52	->	23	0.00	0.00
52	->	24	0.00	0.00
52	->	25	0.00	0.00
52	->	26	-0.00	0.00
52	->	27	0.00	0.00
52	->	28	0.00	0.00
52	->	29	0.00	0.00
52	->	30	-0.00	0.00
52	->	31	-0.00	0.00
52	->	32	-0.00	0.00
52	->	33	-0.02	0.00
52	->	34	-0.06	0.04
52	->	35	-0.46	0.33
52	->	36	-0.44	0.66
52	->	37	-0.15	0.14
52	->	38	0.05	0.02
52	->	39	-0.02	0.01

52	->	40	-0.07	0.03
52	->	41	-0.01	0.00
52	->	42	-0.08	0.03
52	->	43	-0.00	0.00
52	->	44	-0.00	0.01
52	->	45	-0.05	0.02
52	->	46	-0.07	0.03
52	->	47	-0.03	0.04
52	->	48	-4.11	0.55
52	->	49	-2.77	0.51
52	->	50	-0.88	0.14
52	->	51	-10.44	0.52
52	->	52	24.01	1.15
52	->	53	-10.30	0.48
52	->	54	-1.03	0.27
52	->	55	-3.00	0.77
52	->	56	-4.40	1.78
52	->	57	-0.17	0.08
52	->	58	-0.31	0.18
52	->	59	-0.01	0.01
52	->	60	-0.01	0.00
52	->	61	-0.01	0.00
52	->	62	-0.00	0.00
52	->	63	0.00	0.00
52	->	64	0.00	0.00
52	->	65	0.00	0.00
52	->	66	0.00	0.00
52	->	67	0.00	0.00
52	->	68	-0.00	0.00
52	->	69	-0.00	0.00
52	->	70	0.00	0.00
52	->	71	-0.02	0.01
52	->	72	-0.02	0.01
52	->	73	-0.00	0.00
52	->	74	-0.00	0.00
52	->	75	-0.00	0.00
52	->	76	-0.00	0.00
52	->	77	0.00	0.00
52	->	78	-0.00	0.00
52	->	79	-0.00	0.00
52	->	80	0.00	0.00
52	->	81	0.00	0.00
52	->	82	-0.00	0.00
52	->	83	0.00	0.00
52	->	84	0.00	0.00
52	->	85	0.00	0.00
52	->	86	-0.00	0.00
52	->	87	-0.00	0.00
52	->	88	0.00	0.02
52	->	89	0.07	0.06

52	->	90	-0.01	0.00
52	->	91	-0.00	0.00
52	->	92	-0.00	0.00
52	->	93	-0.00	0.00
52	->	94	-0.00	0.00
52	->	95	-0.00	0.00
52	->	96	-0.00	0.00
52	->	97	-0.00	0.00
52	->	98	-0.00	0.00
52	->	99	-0.01	0.00
52	->	100	-0.02	0.00
52	->	101	-0.01	0.00
52	->	102	-0.00	0.00
52	->	103	-0.00	0.00
52	->	104	-0.00	0.00
52	->	105	-0.00	0.00
52	->	106	-0.00	0.00
52	->	107	-0.00	0.00
53	->	1	0.01	0.00
53	->	2	0.00	0.00
53	->	3	-0.00	0.00
53	->	4	-0.00	0.00
53	->	5	-0.00	0.00
53	->	6	0.00	0.00
53	->	7	0.00	0.00
53	->	8	-0.00	0.00
53	->	9	-0.00	0.00
53	->	10	-0.01	0.00
53	->	11	-0.00	0.00
53	->	12	0.01	0.00
53	->	13	0.00	0.00
53	->	14	0.00	0.00
53	->	15	0.00	0.00
53	->	16	0.02	0.00
53	->	17	-0.00	0.00
53	->	18	-0.00	0.00
53	->	19	-0.02	0.00
53	->	20	-0.00	0.00
53	->	21	0.00	0.00
53	->	22	0.03	0.01
53	->	23	0.00	0.00
53	->	24	0.00	0.00
53	->	25	0.02	0.00
53	->	26	0.00	0.00
53	->	27	0.00	0.00
53	->	28	0.00	0.00
53	->	29	0.00	0.00
53	->	30	-0.00	0.00
53	->	31	0.01	0.00
53	->	32	-0.00	0.00

53	->	33	-0.00	0.00
53	->	34	-0.01	0.00
53	->	35	-0.05	0.02
53	->	36	0.02	0.01
53	->	37	-0.01	0.00
53	->	38	-0.04	0.01
53	->	39	-0.00	0.00
53	->	40	0.03	0.01
53	->	41	-0.00	0.00
53	->	42	-0.02	0.00
53	->	43	-0.00	0.00
53	->	44	0.05	0.01
53	->	45	-0.02	0.01
53	->	46	-0.19	0.07
53	->	47	-0.04	0.04
53	->	48	-0.15	0.07
53	->	49	-3.27	1.04
53	->	50	-3.55	0.55
53	->	51	-1.21	0.27
53	->	52	-10.33	0.48
53	->	53	-105.93	2.54
53	->	54	-5.06	0.49
53	->	55	-0.71	0.23
53	->	56	-1.50	0.22
53	->	57	-1.37	0.33
53	->	58	-4.26	2.58
53	->	59	-0.04	0.12
53	->	60	-1.39	0.55
53	->	61	-0.76	0.42
53	->	62	-0.01	0.00
53	->	63	-0.03	0.00
53	->	64	-0.02	0.00
53	->	65	-0.00	0.00
53	->	66	-0.02	0.00
53	->	67	-0.00	0.00
53	->	68	0.03	0.01
53	->	69	0.08	0.02
53	->	70	0.03	0.04
53	->	71	-3.15	1.85
53	->	72	-0.35	0.30
53	->	73	-0.17	0.13
53	->	74	-0.11	0.05
53	->	75	-0.08	0.07
53	->	76	0.05	0.02
53	->	77	0.03	0.01
53	->	78	0.03	0.01
53	->	79	0.00	0.00
53	->	80	0.00	0.00
53	->	81	0.02	0.00
53	->	82	0.00	0.00

53	->	83	-0.00	0.00
53	->	84	0.00	0.00
53	->	85	0.00	0.00
53	->	86	-0.01	0.01
53	->	87	-0.13	0.08
53	->	88	-0.39	0.27
53	->	89	-0.14	0.09
53	->	90	-0.04	0.01
53	->	91	-0.03	0.00
53	->	92	-0.02	0.00
53	->	93	-0.01	0.00
53	->	94	-0.02	0.00
53	->	95	-0.02	0.00
53	->	96	-0.01	0.00
53	->	97	-0.00	0.00
53	->	98	-0.02	0.00
53	->	99	-0.05	0.01
53	->	100	-0.22	0.24
53	->	101	-1.41	2.32
53	->	102	-0.10	0.05
53	->	103	-0.03	0.01
53	->	104	-0.02	0.00
53	->	105	-0.01	0.00
53	->	106	-0.01	0.00
53	->	107	-0.01	0.00
54	->	1	0.00	0.00
54	->	2	0.00	0.00
54	->	3	0.00	0.00
54	->	4	0.00	0.00
54	->	5	-0.00	0.00
54	->	6	0.00	0.00
54	->	7	0.00	0.00
54	->	8	-0.00	0.00
54	->	9	0.00	0.00
54	->	10	-0.00	0.00
54	->	11	-0.00	0.00
54	->	12	0.00	0.00
54	->	13	0.00	0.00
54	->	14	0.00	0.00
54	->	15	0.00	0.00
54	->	16	0.00	0.00
54	->	17	-0.00	0.00
54	->	18	-0.00	0.00
54	->	19	-0.00	0.00
54	->	20	-0.00	0.00
54	->	21	0.00	0.00
54	->	22	0.00	0.00
54	->	23	0.00	0.00
54	->	24	0.00	0.00
54	->	25	0.00	0.00

54	->	26	0.00	0.00
54	->	27	0.00	0.00
54	->	28	0.00	0.00
54	->	29	0.00	0.00
54	->	30	-0.00	0.00
54	->	31	0.00	0.00
54	->	32	-0.00	0.00
54	->	33	-0.00	0.00
54	->	34	-0.00	0.00
54	->	35	-0.04	0.05
54	->	36	-0.00	0.00
54	->	37	-0.03	0.01
54	->	38	-0.01	0.04
54	->	39	-0.00	0.00
54	->	40	-0.05	0.05
54	->	41	-0.00	0.00
54	->	42	-0.02	0.01
54	->	43	-0.00	0.00
54	->	44	-0.00	0.00
54	->	45	-0.00	0.00
54	->	46	-0.02	0.00
54	->	47	-0.03	0.01
54	->	48	-0.07	0.04
54	->	49	-0.15	0.09
54	->	50	-2.41	1.40
54	->	51	-3.38	1.64
54	->	52	-1.04	0.27
54	->	53	-5.11	0.49
54	->	54	-22.45	1.82
54	->	55	-22.30	0.42
54	->	56	-0.62	0.15
54	->	57	-0.83	0.30
54	->	58	-0.13	0.07
54	->	59	-0.15	0.11
54	->	60	-0.01	0.01
54	->	61	-0.00	0.00
54	->	62	-0.00	0.00
54	->	63	0.00	0.00
54	->	64	-0.00	0.00
54	->	65	0.00	0.00
54	->	66	0.00	0.00
54	->	67	0.00	0.00
54	->	68	-0.01	0.00
54	->	69	-0.02	0.01
54	->	70	-0.02	0.01
54	->	71	-0.31	0.27
54	->	72	-0.10	0.05
54	->	73	0.00	0.00
54	->	74	-0.01	0.00
54	->	75	-0.01	0.01

54	->	76	-0.00	0.00
54	->	77	0.00	0.00
54	->	78	-0.00	0.00
54	->	79	-0.00	0.00
54	->	80	0.00	0.00
54	->	81	0.00	0.00
54	->	82	0.00	0.00
54	->	83	0.00	0.00
54	->	84	0.00	0.00
54	->	85	0.00	0.00
54	->	86	-0.01	0.00
54	->	87	-0.20	0.13
54	->	88	-2.62	1.08
54	->	89	-0.41	0.86
54	->	90	-0.02	0.01
54	->	91	-0.01	0.00
54	->	92	-0.00	0.00
54	->	93	-0.00	0.00
54	->	94	-0.00	0.00
54	->	95	-0.00	0.00
54	->	96	-0.00	0.00
54	->	97	-0.00	0.00
54	->	98	-0.00	0.00
54	->	99	-0.00	0.00
54	->	100	-0.00	0.00
54	->	101	-0.00	0.00
54	->	102	-0.00	0.00
54	->	103	-0.00	0.00
54	->	104	-0.00	0.00
54	->	105	-0.00	0.00
54	->	106	-0.01	0.00
54	->	107	-0.00	0.00
55	->	1	0.00	0.00
55	->	2	0.00	0.00
55	->	3	0.00	0.00
55	->	4	0.00	0.00
55	->	5	0.00	0.00
55	->	6	0.00	0.00
55	->	7	0.00	0.00
55	->	8	0.00	0.00
55	->	9	-0.00	0.00
55	->	10	0.00	0.00
55	->	11	-0.00	0.00
55	->	12	0.00	0.00
55	->	13	0.00	0.00
55	->	14	0.00	0.00
55	->	15	0.00	0.00
55	->	16	0.00	0.00
55	->	17	0.00	0.00
55	->	18	0.00	0.00

55 ->	19	-0.00	0.00
55 ->	20	0.00	0.00
55 ->	21	0.00	0.00
55 ->	22	-0.00	0.00
55 ->	23	0.00	0.00
55 ->	24	0.00	0.00
55 ->	25	-0.00	0.00
55 ->	26	0.00	0.00
55 ->	27	-0.00	0.00
55 ->	28	0.00	0.00
55 ->	29	-0.00	0.00
55 ->	30	0.00	0.00
55 ->	31	-0.00	0.00
55 ->	32	-0.00	0.00
55 ->	33	-0.01	0.00
55 ->	34	-0.05	0.01
55 ->	35	2.37	0.36
55 ->	36	-0.32	0.11
55 ->	37	1.12	0.73
55 ->	38	2.16	0.36
55 ->	39	-0.03	0.04
55 ->	40	-0.34	0.16
55 ->	41	-0.01	0.01
55 ->	42	-0.00	0.01
55 ->	43	-0.00	0.00
55 ->	44	-0.00	0.00
55 ->	45	-0.00	0.00
55 ->	46	-0.01	0.00
55 ->	47	-0.03	0.01
55 ->	48	-0.06	0.02
55 ->	49	-0.04	0.01
55 ->	50	-0.14	0.07
55 ->	51	12.87	6.03
55 ->	52	-2.99	0.77
55 ->	53	-0.71	0.22
55 ->	54	-22.41	0.42
55 ->	55	4.21	1.12
55 ->	56	-5.03	0.43
55 ->	57	-0.47	0.10
55 ->	58	-0.04	0.02
55 ->	59	-0.03	0.02
55 ->	60	-0.00	0.00
55 ->	61	-0.00	0.00
55 ->	62	0.00	0.00
55 ->	63	-0.00	0.00
55 ->	64	-0.00	0.00
55 ->	65	-0.00	0.00
55 ->	66	-0.00	0.00
55 ->	67	0.00	0.00
55 ->	68	-0.00	0.00

55	->	69	-0.00	0.00
55	->	70	-0.01	0.00
55	->	71	-0.01	0.00
55	->	72	-0.01	0.00
55	->	73	-0.00	0.00
55	->	74	-0.00	0.00
55	->	75	-0.00	0.00
55	->	76	0.00	0.00
55	->	77	0.00	0.00
55	->	78	0.00	0.00
55	->	79	0.00	0.00
55	->	80	0.00	0.00
55	->	81	0.00	0.00
55	->	82	0.00	0.00
55	->	83	0.00	0.00
55	->	84	0.00	0.00
55	->	85	0.00	0.00
55	->	86	-0.00	0.00
55	->	87	0.01	0.01
55	->	88	0.03	0.07
55	->	89	0.08	0.09
55	->	90	-0.00	0.00
55	->	91	-0.00	0.00
55	->	92	-0.00	0.00
55	->	93	-0.00	0.00
55	->	94	-0.00	0.00
55	->	95	-0.00	0.00
55	->	96	-0.00	0.00
55	->	97	0.00	0.00
55	->	98	0.00	0.00
55	->	99	-0.00	0.00
55	->	100	-0.00	0.00
55	->	101	-0.00	0.00
55	->	102	-0.00	0.00
55	->	103	-0.00	0.00
55	->	104	-0.00	0.00
55	->	105	-0.00	0.00
55	->	106	-0.00	0.00
55	->	107	-0.00	0.00
56	->	1	0.00	0.00
56	->	2	0.00	0.00
56	->	3	0.00	0.00
56	->	4	0.00	0.00
56	->	5	-0.00	0.00
56	->	6	0.00	0.00
56	->	7	0.00	0.00
56	->	8	-0.00	0.00
56	->	9	0.00	0.00
56	->	10	-0.00	0.00
56	->	11	-0.00	0.00

56 ->	12	0.00	0.00
56 ->	13	0.00	0.00
56 ->	14	0.00	0.00
56 ->	15	0.00	0.00
56 ->	16	0.00	0.00
56 ->	17	-0.00	0.00
56 ->	18	-0.00	0.00
56 ->	19	-0.00	0.00
56 ->	20	-0.00	0.00
56 ->	21	0.00	0.00
56 ->	22	0.00	0.00
56 ->	23	0.00	0.00
56 ->	24	0.00	0.00
56 ->	25	0.00	0.00
56 ->	26	0.00	0.00
56 ->	27	0.00	0.00
56 ->	28	0.00	0.00
56 ->	29	0.00	0.00
56 ->	30	-0.00	0.00
56 ->	31	0.00	0.00
56 ->	32	-0.00	0.00
56 ->	33	-0.00	0.00
56 ->	34	-0.00	0.00
56 ->	35	-0.01	0.01
56 ->	36	-0.02	0.02
56 ->	37	-0.06	0.07
56 ->	38	-0.01	0.00
56 ->	39	-0.00	0.00
56 ->	40	0.00	0.00
56 ->	41	-0.00	0.00
56 ->	42	-0.00	0.00
56 ->	43	0.00	0.00
56 ->	44	0.00	0.00
56 ->	45	-0.00	0.00
56 ->	46	-0.01	0.00
56 ->	47	-0.01	0.00
56 ->	48	-0.02	0.01
56 ->	49	-0.03	0.03
56 ->	50	-0.04	0.02
56 ->	51	-0.18	0.07
56 ->	52	-4.41	1.77
56 ->	53	-1.51	0.22
56 ->	54	-0.63	0.15
56 ->	55	-5.18	0.43
56 ->	56	-24.52	2.04
56 ->	57	-11.74	0.37
56 ->	58	-2.75	1.30
56 ->	59	-0.03	0.02
56 ->	60	-0.02	0.00
56 ->	61	-0.00	0.00

56	->	62	-0.00	0.00
56	->	63	0.00	0.00
56	->	64	0.00	0.00
56	->	65	-0.00	0.00
56	->	66	0.00	0.00
56	->	67	0.00	0.00
56	->	68	-0.00	0.00
56	->	69	-0.01	0.00
56	->	70	-0.01	0.00
56	->	71	-0.02	0.01
56	->	72	-0.00	0.00
56	->	73	-0.00	0.00
56	->	74	-0.00	0.00
56	->	75	-0.00	0.00
56	->	76	-0.00	0.00
56	->	77	0.00	0.00
56	->	78	0.00	0.00
56	->	79	0.00	0.00
56	->	80	0.00	0.00
56	->	81	0.00	0.00
56	->	82	-0.00	0.00
56	->	83	0.00	0.00
56	->	84	-0.00	0.00
56	->	85	0.00	0.00
56	->	86	-0.00	0.00
56	->	87	-0.01	0.01
56	->	88	-0.03	0.01
56	->	89	-0.01	0.00
56	->	90	-0.00	0.00
56	->	91	-0.00	0.00
56	->	92	-0.00	0.00
56	->	93	-0.00	0.00
56	->	94	-0.00	0.00
56	->	95	-0.00	0.00
56	->	96	-0.00	0.00
56	->	97	-0.00	0.00
56	->	98	-0.00	0.00
56	->	99	-0.00	0.00
56	->	100	-0.00	0.00
56	->	101	-0.00	0.00
56	->	102	-0.00	0.00
56	->	103	-0.00	0.00
56	->	104	-0.00	0.00
56	->	105	-0.00	0.00
56	->	106	-0.00	0.00
56	->	107	-0.00	0.00
57	->	1	0.00	0.00
57	->	2	0.00	0.00
57	->	3	0.00	0.00
57	->	4	0.00	0.00

57	->	5	-0.00	0.00
57	->	6	0.00	0.00
57	->	7	0.00	0.00
57	->	8	-0.00	0.00
57	->	9	0.00	0.00
57	->	10	-0.00	0.00
57	->	11	-0.00	0.00
57	->	12	0.00	0.00
57	->	13	0.00	0.00
57	->	14	0.00	0.00
57	->	15	0.00	0.00
57	->	16	0.00	0.00
57	->	17	0.00	0.00
57	->	18	0.00	0.00
57	->	19	-0.00	0.00
57	->	20	0.00	0.00
57	->	21	0.00	0.00
57	->	22	0.00	0.00
57	->	23	0.00	0.00
57	->	24	0.00	0.00
57	->	25	0.00	0.00
57	->	26	0.00	0.00
57	->	27	0.00	0.00
57	->	28	0.00	0.00
57	->	29	-0.00	0.00
57	->	30	-0.00	0.00
57	->	31	0.00	0.00
57	->	32	-0.00	0.00
57	->	33	0.00	0.00
57	->	34	-0.00	0.00
57	->	35	-0.00	0.00
57	->	36	-0.00	0.00
57	->	37	-0.00	0.00
57	->	38	-0.00	0.00
57	->	39	0.00	0.00
57	->	40	0.00	0.00
57	->	41	0.00	0.00
57	->	42	-0.00	0.00
57	->	43	0.00	0.00
57	->	44	0.00	0.00
57	->	45	-0.00	0.00
57	->	46	-0.00	0.00
57	->	47	-0.00	0.00
57	->	48	-0.00	0.00
57	->	49	-0.01	0.00
57	->	50	-0.03	0.01
57	->	51	-0.03	0.01
57	->	52	-0.17	0.08
57	->	53	-1.39	0.33
57	->	54	-0.86	0.31

57	->	55	-0.49	0.10
57	->	56	-11.81	0.37
57	->	57	12.28	0.88
57	->	58	-15.06	0.49
57	->	59	-1.61	0.79
57	->	60	-0.03	0.00
57	->	61	-0.01	0.00
57	->	62	-0.00	0.00
57	->	63	0.00	0.00
57	->	64	0.00	0.00
57	->	65	0.00	0.00
57	->	66	0.00	0.00
57	->	67	-0.00	0.00
57	->	68	-0.00	0.00
57	->	69	-0.02	0.01
57	->	70	-0.00	0.01
57	->	71	-0.04	0.01
57	->	72	-0.01	0.00
57	->	73	-0.00	0.00
57	->	74	-0.00	0.00
57	->	75	-0.00	0.00
57	->	76	-0.00	0.00
57	->	77	0.00	0.00
57	->	78	0.00	0.00
57	->	79	-0.00	0.00
57	->	80	-0.00	0.00
57	->	81	0.00	0.00
57	->	82	0.00	0.00
57	->	83	0.00	0.00
57	->	84	0.00	0.00
57	->	85	-0.00	0.00
57	->	86	-0.00	0.00
57	->	87	-0.02	0.02
57	->	88	-0.07	0.07
57	->	89	-0.01	0.00
57	->	90	-0.00	0.00
57	->	91	-0.00	0.00
57	->	92	-0.00	0.00
57	->	93	-0.00	0.00
57	->	94	-0.00	0.00
57	->	95	-0.00	0.00
57	->	96	-0.00	0.00
57	->	97	-0.00	0.00
57	->	98	-0.00	0.00
57	->	99	-0.00	0.00
57	->	100	-0.00	0.00
57	->	101	-0.00	0.00
57	->	102	-0.00	0.00
57	->	103	-0.00	0.00
57	->	104	-0.00	0.00

57	->	105	-0.00	0.00
57	->	106	-0.00	0.00
57	->	107	-0.00	0.00
58	->	1	-0.01	0.00
58	->	2	-0.00	0.00
58	->	3	0.00	0.00
58	->	4	-0.00	0.00
58	->	5	0.00	0.00
58	->	6	-0.00	0.00
58	->	7	0.00	0.00
58	->	8	0.00	0.00
58	->	9	0.00	0.00
58	->	10	0.00	0.00
58	->	11	0.00	0.00
58	->	12	-0.00	0.00
58	->	13	-0.00	0.00
58	->	14	-0.00	0.00
58	->	15	-0.00	0.00
58	->	16	-0.01	0.00
58	->	17	0.00	0.00
58	->	18	0.00	0.00
58	->	19	0.01	0.00
58	->	20	0.00	0.00
58	->	21	-0.00	0.00
58	->	22	-0.01	0.00
58	->	23	-0.00	0.00
58	->	24	-0.00	0.00
58	->	25	-0.01	0.00
58	->	26	-0.00	0.00
58	->	27	-0.00	0.00
58	->	28	0.00	0.00
58	->	29	-0.00	0.00
58	->	30	0.00	0.00
58	->	31	-0.01	0.00
58	->	32	0.00	0.00
58	->	33	-0.00	0.00
58	->	34	0.00	0.00
58	->	35	0.03	0.01
58	->	36	-0.04	0.02
58	->	37	-0.00	0.00
58	->	38	0.03	0.00
58	->	39	-0.00	0.00
58	->	40	-0.02	0.00
58	->	41	0.00	0.00
58	->	42	-0.00	0.00
58	->	43	0.00	0.00
58	->	44	-0.02	0.00
58	->	45	0.00	0.00
58	->	46	0.03	0.01
58	->	47	0.00	0.00

58 ->	48	-0.00	0.01
58 ->	49	-0.03	0.02
58 ->	50	-0.01	0.01
58 ->	51	-0.02	0.01
58 ->	52	-0.31	0.17
58 ->	53	-4.24	2.55
58 ->	54	-0.13	0.07
58 ->	55	-0.04	0.02
58 ->	56	-2.76	1.27
58 ->	57	-15.19	0.49
58 ->	58	-62.00	3.63
58 ->	59	-11.74	0.54
58 ->	60	-1.36	0.54
58 ->	61	-0.10	0.03
58 ->	62	-0.01	0.00
58 ->	63	0.02	0.00
58 ->	64	0.01	0.00
58 ->	65	0.00	0.00
58 ->	66	0.02	0.00
58 ->	67	0.00	0.00
58 ->	68	-0.02	0.01
58 ->	69	-0.06	0.07
58 ->	70	-0.27	0.10
58 ->	71	-0.69	0.31
58 ->	72	-0.04	0.01
58 ->	73	-0.01	0.00
58 ->	74	0.03	0.01
58 ->	75	0.04	0.01
58 ->	76	-0.04	0.01
58 ->	77	-0.02	0.01
58 ->	78	-0.02	0.00
58 ->	79	-0.00	0.00
58 ->	80	-0.00	0.00
58 ->	81	-0.01	0.00
58 ->	82	-0.00	0.00
58 ->	83	0.00	0.00
58 ->	84	-0.00	0.00
58 ->	85	-0.00	0.00
58 ->	86	0.00	0.00
58 ->	87	0.03	0.01
58 ->	88	0.04	0.01
58 ->	89	0.03	0.01
58 ->	90	0.01	0.00
58 ->	91	0.01	0.00
58 ->	92	0.01	0.00
58 ->	93	0.00	0.00
58 ->	94	0.01	0.00
58 ->	95	0.01	0.00
58 ->	96	0.01	0.00
58 ->	97	0.00	0.00

58	->	98	0.01	0.00
58	->	99	0.02	0.01
58	->	100	0.03	0.01
58	->	101	0.04	0.01
58	->	102	0.02	0.00
58	->	103	0.01	0.00
58	->	104	0.01	0.00
58	->	105	0.01	0.00
58	->	106	0.00	0.00
58	->	107	0.00	0.00
59	->	1	0.01	0.00
59	->	2	0.00	0.00
59	->	3	-0.00	0.00
59	->	4	0.00	0.00
59	->	5	-0.00	0.00
59	->	6	0.00	0.00
59	->	7	0.00	0.00
59	->	8	-0.00	0.00
59	->	9	0.00	0.00
59	->	10	-0.00	0.00
59	->	11	-0.00	0.00
59	->	12	0.00	0.00
59	->	13	0.00	0.00
59	->	14	0.00	0.00
59	->	15	0.00	0.00
59	->	16	0.01	0.00
59	->	17	-0.00	0.00
59	->	18	-0.00	0.00
59	->	19	-0.01	0.00
59	->	20	-0.00	0.00
59	->	21	0.00	0.00
59	->	22	0.01	0.00
59	->	23	0.00	0.00
59	->	24	0.00	0.00
59	->	25	0.01	0.00
59	->	26	0.00	0.00
59	->	27	0.00	0.00
59	->	28	0.00	0.00
59	->	29	0.00	0.00
59	->	30	-0.00	0.00
59	->	31	0.01	0.00
59	->	32	-0.00	0.00
59	->	33	0.00	0.00
59	->	34	-0.00	0.00
59	->	35	-0.03	0.00
59	->	36	0.02	0.00
59	->	37	-0.00	0.00
59	->	38	-0.03	0.00
59	->	39	0.00	0.00
59	->	40	0.02	0.01

59 ->	41	-0.00	0.00
59 ->	42	-0.00	0.00
59 ->	43	-0.00	0.00
59 ->	44	0.02	0.00
59 ->	45	-0.00	0.00
59 ->	46	-0.03	0.00
59 ->	47	-0.01	0.00
59 ->	48	-0.00	0.00
59 ->	49	-0.00	0.01
59 ->	50	-0.04	0.02
59 ->	51	-0.01	0.01
59 ->	52	-0.01	0.01
59 ->	53	-0.04	0.12
59 ->	54	-0.15	0.11
59 ->	55	-0.03	0.02
59 ->	56	-0.03	0.02
59 ->	57	-1.68	0.79
59 ->	58	-11.79	0.55
59 ->	59	12.16	2.77
59 ->	60	-0.15	0.48
59 ->	61	-1.04	0.36
59 ->	62	-0.15	0.04
59 ->	63	-0.09	0.01
59 ->	64	-0.03	0.00
59 ->	65	-0.00	0.00
59 ->	66	-0.06	0.01
59 ->	67	-0.01	0.01
59 ->	68	0.19	0.28
59 ->	69	-2.25	0.88
59 ->	70	-3.16	0.48
59 ->	71	-1.68	0.41
59 ->	72	-0.06	0.06
59 ->	73	-0.07	0.02
59 ->	74	-0.07	0.01
59 ->	75	-0.65	1.64
59 ->	76	0.04	0.02
59 ->	77	0.01	0.00
59 ->	78	0.04	0.01
59 ->	79	-0.00	0.00
59 ->	80	-0.00	0.00
59 ->	81	0.02	0.00
59 ->	82	0.00	0.00
59 ->	83	-0.00	0.00
59 ->	84	0.00	0.00
59 ->	85	0.00	0.00
59 ->	86	-0.15	0.27
59 ->	87	-1.29	1.80
59 ->	88	-0.73	1.76
59 ->	89	-0.05	0.02
59 ->	90	-0.02	0.00

59	->	91	-0.01	0.00
59	->	92	-0.01	0.00
59	->	93	-0.01	0.00
59	->	94	-0.01	0.00
59	->	95	-0.01	0.00
59	->	96	-0.00	0.00
59	->	97	-0.00	0.00
59	->	98	-0.00	0.00
59	->	99	-0.01	0.00
59	->	100	-0.02	0.01
59	->	101	-0.04	0.03
59	->	102	-0.04	0.03
59	->	103	-0.02	0.00
59	->	104	-0.01	0.00
59	->	105	-0.01	0.00
59	->	106	-0.01	0.00
59	->	107	-0.01	0.00
60	->	1	0.00	0.00
60	->	2	0.00	0.00
60	->	3	0.00	0.00
60	->	4	0.00	0.00
60	->	5	-0.00	0.00
60	->	6	0.00	0.00
60	->	7	0.00	0.00
60	->	8	0.00	0.00
60	->	9	0.00	0.00
60	->	10	-0.00	0.00
60	->	11	-0.00	0.00
60	->	12	0.00	0.00
60	->	13	0.00	0.00
60	->	14	0.00	0.00
60	->	15	0.00	0.00
60	->	16	0.00	0.00
60	->	17	-0.00	0.00
60	->	18	-0.00	0.00
60	->	19	-0.00	0.00
60	->	20	-0.00	0.00
60	->	21	0.00	0.00
60	->	22	0.00	0.00
60	->	23	0.00	0.00
60	->	24	0.00	0.00
60	->	25	0.00	0.00
60	->	26	0.00	0.00
60	->	27	0.00	0.00
60	->	28	0.00	0.00
60	->	29	0.00	0.00
60	->	30	-0.00	0.00
60	->	31	0.00	0.00
60	->	32	-0.00	0.00
60	->	33	-0.00	0.00

60	->	34	0.00	0.00
60	->	35	-0.00	0.00
60	->	36	0.00	0.00
60	->	37	-0.00	0.00
60	->	38	-0.00	0.00
60	->	39	0.00	0.00
60	->	40	0.00	0.00
60	->	41	0.00	0.00
60	->	42	-0.00	0.00
60	->	43	-0.00	0.00
60	->	44	0.00	0.00
60	->	45	-0.00	0.00
60	->	46	-0.01	0.00
60	->	47	-0.00	0.00
60	->	48	-0.00	0.00
60	->	49	-0.02	0.01
60	->	50	-0.03	0.01
60	->	51	-0.01	0.00
60	->	52	-0.01	0.00
60	->	53	-1.39	0.55
60	->	54	-0.01	0.01
60	->	55	-0.00	0.00
60	->	56	-0.02	0.00
60	->	57	-0.03	0.00
60	->	58	-1.39	0.56
60	->	59	-0.19	0.48
60	->	60	1.80	0.54
60	->	61	-10.55	0.62
60	->	62	-0.37	0.21
60	->	63	-0.02	0.00
60	->	64	-0.00	0.00
60	->	65	-0.00	0.00
60	->	66	0.00	0.00
60	->	67	-0.00	0.00
60	->	68	-0.02	0.01
60	->	69	-0.72	0.87
60	->	70	-0.89	0.17
60	->	71	-0.77	0.18
60	->	72	-0.15	0.10
60	->	73	-0.06	0.02
60	->	74	-0.01	0.00
60	->	75	-0.01	0.01
60	->	76	-0.03	0.01
60	->	77	-0.01	0.00
60	->	78	-0.00	0.00
60	->	79	-0.00	0.00
60	->	80	-0.00	0.00
60	->	81	-0.00	0.00
60	->	82	-0.00	0.00
60	->	83	-0.00	0.00

60	->	84	0.00	0.00
60	->	85	0.00	0.00
60	->	86	-0.00	0.00
60	->	87	-0.01	0.00
60	->	88	-0.01	0.00
60	->	89	-0.00	0.00
60	->	90	-0.00	0.00
60	->	91	-0.00	0.00
60	->	92	-0.00	0.00
60	->	93	-0.00	0.00
60	->	94	-0.00	0.00
60	->	95	-0.00	0.00
60	->	96	-0.00	0.00
60	->	97	-0.00	0.00
60	->	98	-0.00	0.00
60	->	99	-0.00	0.00
60	->	100	-0.01	0.01
60	->	101	-0.07	0.09
60	->	102	-0.03	0.02
60	->	103	-0.00	0.00
60	->	104	-0.00	0.00
60	->	105	-0.00	0.00
60	->	106	-0.00	0.00
60	->	107	-0.00	0.00
61	->	1	0.00	0.00
61	->	2	0.00	0.00
61	->	3	0.00	0.00
61	->	4	0.00	0.00
61	->	5	-0.00	0.00
61	->	6	0.00	0.00
61	->	7	0.00	0.00
61	->	8	0.00	0.00
61	->	9	0.00	0.00
61	->	10	-0.00	0.00
61	->	11	-0.00	0.00
61	->	12	0.00	0.00
61	->	13	0.00	0.00
61	->	14	0.00	0.00
61	->	15	0.00	0.00
61	->	16	0.00	0.00
61	->	17	0.00	0.00
61	->	18	0.00	0.00
61	->	19	-0.00	0.00
61	->	20	0.00	0.00
61	->	21	0.00	0.00
61	->	22	-0.00	0.00
61	->	23	0.00	0.00
61	->	24	0.00	0.00
61	->	25	0.00	0.00
61	->	26	0.00	0.00

61	->	27	0.00	0.00
61	->	28	0.00	0.00
61	->	29	0.00	0.00
61	->	30	-0.00	0.00
61	->	31	-0.00	0.00
61	->	32	0.00	0.00
61	->	33	-0.00	0.00
61	->	34	0.00	0.00
61	->	35	0.00	0.00
61	->	36	-0.00	0.00
61	->	37	0.00	0.00
61	->	38	0.00	0.00
61	->	39	0.00	0.00
61	->	40	-0.00	0.00
61	->	41	0.00	0.00
61	->	42	-0.00	0.00
61	->	43	-0.00	0.00
61	->	44	-0.00	0.00
61	->	45	-0.00	0.00
61	->	46	-0.01	0.01
61	->	47	-0.00	0.00
61	->	48	-0.01	0.00
61	->	49	-0.03	0.01
61	->	50	-0.03	0.03
61	->	51	-0.00	0.00
61	->	52	-0.01	0.00
61	->	53	-0.76	0.42
61	->	54	-0.00	0.00
61	->	55	-0.00	0.00
61	->	56	-0.00	0.00
61	->	57	-0.01	0.00
61	->	58	-0.10	0.03
61	->	59	-1.06	0.36
61	->	60	-10.67	0.62
61	->	61	27.55	1.64
61	->	62	-10.64	0.50
61	->	63	-0.37	0.04
61	->	64	-0.05	0.01
61	->	65	-0.00	0.00
61	->	66	-0.01	0.00
61	->	67	-0.02	0.01
61	->	68	-0.30	0.07
61	->	69	-3.13	0.66
61	->	70	-3.54	0.54
61	->	71	-0.93	0.28
61	->	72	-1.73	0.74
61	->	73	-1.61	0.75
61	->	74	-0.01	0.01
61	->	75	-0.10	0.04
61	->	76	-1.41	0.89

61	->	77	-0.03	0.03
61	->	78	-0.01	0.00
61	->	79	-0.02	0.01
61	->	80	-0.01	0.01
61	->	81	-0.00	0.00
61	->	82	-0.00	0.00
61	->	83	-0.01	0.00
61	->	84	-0.00	0.00
61	->	85	-0.00	0.00
61	->	86	-0.00	0.00
61	->	87	-0.00	0.00
61	->	88	-0.01	0.00
61	->	89	-0.00	0.00
61	->	90	-0.00	0.00
61	->	91	-0.00	0.00
61	->	92	-0.00	0.00
61	->	93	-0.01	0.01
61	->	94	-0.00	0.00
61	->	95	-0.00	0.00
61	->	96	-0.00	0.00
61	->	97	-0.00	0.00
61	->	98	-0.00	0.00
61	->	99	-0.00	0.00
61	->	100	-0.03	0.03
61	->	101	-0.78	0.90
61	->	102	-0.05	0.18
61	->	103	-0.02	0.01
61	->	104	-0.01	0.00
61	->	105	-0.00	0.00
61	->	106	-0.00	0.00
61	->	107	-0.00	0.00
62	->	1	-0.00	0.00
62	->	2	0.00	0.00
62	->	3	0.00	0.00
62	->	4	0.00	0.00
62	->	5	0.00	0.00
62	->	6	0.00	0.00
62	->	7	0.00	0.00
62	->	8	0.00	0.00
62	->	9	0.00	0.00
62	->	10	0.00	0.00
62	->	11	0.00	0.00
62	->	12	-0.00	0.00
62	->	13	-0.00	0.00
62	->	14	-0.00	0.00
62	->	15	0.00	0.00
62	->	16	-0.00	0.00
62	->	17	0.00	0.00
62	->	18	-0.00	0.00
62	->	19	0.00	0.00

62	->	20	0.00	0.00
62	->	21	0.00	0.00
62	->	22	-0.00	0.00
62	->	23	0.00	0.00
62	->	24	0.00	0.00
62	->	25	-0.00	0.00
62	->	26	0.00	0.00
62	->	27	0.00	0.00
62	->	28	0.00	0.00
62	->	29	0.00	0.00
62	->	30	0.00	0.00
62	->	31	-0.00	0.00
62	->	32	0.00	0.00
62	->	33	0.00	0.00
62	->	34	0.00	0.00
62	->	35	0.00	0.00
62	->	36	-0.00	0.00
62	->	37	0.00	0.00
62	->	38	0.00	0.00
62	->	39	0.00	0.00
62	->	40	-0.00	0.00
62	->	41	0.00	0.00
62	->	42	0.00	0.00
62	->	43	0.00	0.00
62	->	44	-0.00	0.00
62	->	45	0.00	0.00
62	->	46	0.00	0.00
62	->	47	0.00	0.00
62	->	48	-0.00	0.00
62	->	49	-0.00	0.00
62	->	50	-0.00	0.00
62	->	51	-0.00	0.00
62	->	52	-0.00	0.00
62	->	53	-0.01	0.00
62	->	54	-0.00	0.00
62	->	55	0.00	0.00
62	->	56	-0.00	0.00
62	->	57	-0.00	0.00
62	->	58	-0.01	0.00
62	->	59	-0.15	0.04
62	->	60	-0.38	0.22
62	->	61	-10.61	0.49
62	->	62	22.78	1.27
62	->	63	-12.07	0.30
62	->	64	-0.39	0.11
62	->	65	-0.02	0.03
62	->	66	-0.15	0.06
62	->	67	-0.11	0.14
62	->	68	-1.19	0.58
62	->	69	-1.44	0.37

62	->	70	-1.18	0.42
62	->	71	-0.01	0.01
62	->	72	-0.03	0.01
62	->	73	-0.02	0.01
62	->	74	0.00	0.00
62	->	75	-0.01	0.01
62	->	76	-1.89	1.66
62	->	77	-0.01	0.01
62	->	78	0.00	0.01
62	->	79	-0.09	0.02
62	->	80	-0.04	0.02
62	->	81	-0.01	0.00
62	->	82	-0.00	0.00
62	->	83	-0.04	0.01
62	->	84	-0.00	0.00
62	->	85	-0.00	0.00
62	->	86	-0.00	0.00
62	->	87	-0.01	0.01
62	->	88	-0.00	0.00
62	->	89	-0.00	0.00
62	->	90	0.00	0.00
62	->	91	0.00	0.00
62	->	92	0.00	0.00
62	->	93	0.00	0.00
62	->	94	0.00	0.00
62	->	95	0.00	0.00
62	->	96	0.00	0.00
62	->	97	0.00	0.00
62	->	98	0.00	0.00
62	->	99	0.00	0.00
62	->	100	0.00	0.00
62	->	101	-0.00	0.00
62	->	102	0.01	0.01
62	->	103	0.00	0.00
62	->	104	0.00	0.00
62	->	105	0.00	0.00
62	->	106	0.00	0.00
62	->	107	0.00	0.00
63	->	1	-0.01	0.00
63	->	2	-0.00	0.00
63	->	3	0.00	0.00
63	->	4	0.00	0.00
63	->	5	0.00	0.00
63	->	6	-0.00	0.00
63	->	7	0.00	0.00
63	->	8	0.00	0.00
63	->	9	0.00	0.00
63	->	10	0.00	0.00
63	->	11	0.00	0.00
63	->	12	-0.00	0.00

63	->	13	-0.00	0.00
63	->	14	-0.00	0.00
63	->	15	-0.00	0.00
63	->	16	-0.01	0.00
63	->	17	0.00	0.00
63	->	18	-0.00	0.00
63	->	19	0.01	0.00
63	->	20	0.00	0.00
63	->	21	-0.00	0.00
63	->	22	-0.01	0.00
63	->	23	-0.00	0.00
63	->	24	-0.00	0.00
63	->	25	-0.00	0.00
63	->	26	-0.00	0.00
63	->	27	-0.00	0.00
63	->	28	-0.00	0.00
63	->	29	-0.00	0.00
63	->	30	0.00	0.00
63	->	31	-0.00	0.00
63	->	32	0.00	0.00
63	->	33	-0.00	0.00
63	->	34	-0.00	0.00
63	->	35	0.01	0.00
63	->	36	-0.00	0.00
63	->	37	0.00	0.00
63	->	38	0.01	0.00
63	->	39	-0.00	0.00
63	->	40	-0.01	0.00
63	->	41	0.00	0.00
63	->	42	-0.00	0.00
63	->	43	-0.00	0.00
63	->	44	-0.01	0.00
63	->	45	0.00	0.00
63	->	46	0.01	0.00
63	->	47	0.00	0.00
63	->	48	0.00	0.00
63	->	49	0.00	0.00
63	->	50	0.00	0.00
63	->	51	-0.00	0.00
63	->	52	0.00	0.00
63	->	53	-0.03	0.00
63	->	54	0.00	0.00
63	->	55	-0.00	0.00
63	->	56	0.00	0.00
63	->	57	0.00	0.00
63	->	58	0.02	0.00
63	->	59	-0.09	0.01
63	->	60	-0.02	0.00
63	->	61	-0.37	0.04
63	->	62	-12.12	0.30

63	->	63	-25.63	2.56
63	->	64	-15.29	1.10
63	->	65	0.87	1.25
63	->	66	3.06	0.50
63	->	67	-0.74	0.67
63	->	68	-2.17	0.73
63	->	69	-0.61	0.14
63	->	70	-0.98	0.30
63	->	71	0.01	0.01
63	->	72	-0.04	0.01
63	->	73	-0.00	0.01
63	->	74	0.03	0.01
63	->	75	-0.00	0.02
63	->	76	-1.51	1.12
63	->	77	-0.03	0.03
63	->	78	-0.17	0.05
63	->	79	3.21	1.00
63	->	80	-0.12	0.38
63	->	81	-0.01	0.02
63	->	82	-0.08	0.03
63	->	83	2.39	0.48
63	->	84	-0.00	0.01
63	->	85	-0.00	0.03
63	->	86	0.01	0.01
63	->	87	0.04	0.01
63	->	88	0.01	0.00
63	->	89	0.01	0.00
63	->	90	0.01	0.00
63	->	91	0.01	0.00
63	->	92	0.00	0.00
63	->	93	0.00	0.00
63	->	94	0.01	0.00
63	->	95	0.01	0.00
63	->	96	0.00	0.00
63	->	97	0.00	0.00
63	->	98	0.00	0.00
63	->	99	0.00	0.00
63	->	100	0.01	0.00
63	->	101	0.02	0.01
63	->	102	0.04	0.02
63	->	103	0.03	0.01
63	->	104	0.02	0.00
63	->	105	0.01	0.00
63	->	106	0.01	0.00
63	->	107	0.00	0.00
64	->	1	-0.00	0.00
64	->	2	-0.00	0.00
64	->	3	0.00	0.00
64	->	4	0.00	0.00
64	->	5	0.00	0.00

64	->	6	-0.00	0.00
64	->	7	0.00	0.00
64	->	8	0.00	0.00
64	->	9	0.00	0.00
64	->	10	0.00	0.00
64	->	11	0.00	0.00
64	->	12	-0.00	0.00
64	->	13	-0.00	0.00
64	->	14	-0.00	0.00
64	->	15	-0.00	0.00
64	->	16	-0.00	0.00
64	->	17	-0.00	0.00
64	->	18	-0.00	0.00
64	->	19	0.00	0.00
64	->	20	0.00	0.00
64	->	21	-0.00	0.00
64	->	22	-0.01	0.00
64	->	23	-0.00	0.00
64	->	24	-0.00	0.00
64	->	25	-0.00	0.00
64	->	26	-0.00	0.00
64	->	27	-0.00	0.00
64	->	28	-0.00	0.00
64	->	29	-0.00	0.00
64	->	30	0.00	0.00
64	->	31	-0.00	0.00
64	->	32	0.00	0.00
64	->	33	-0.00	0.00
64	->	34	0.00	0.00
64	->	35	0.00	0.00
64	->	36	-0.00	0.00
64	->	37	0.00	0.00
64	->	38	0.00	0.00
64	->	39	-0.00	0.00
64	->	40	-0.00	0.00
64	->	41	0.00	0.00
64	->	42	-0.00	0.00
64	->	43	0.00	0.00
64	->	44	-0.01	0.00
64	->	45	0.00	0.00
64	->	46	0.01	0.00
64	->	47	0.00	0.00
64	->	48	0.00	0.00
64	->	49	-0.00	0.00
64	->	50	0.00	0.00
64	->	51	-0.00	0.00
64	->	52	0.00	0.00
64	->	53	-0.02	0.00
64	->	54	-0.00	0.00
64	->	55	-0.00	0.00

64	->	56	0.00	0.00
64	->	57	0.00	0.00
64	->	58	0.01	0.00
64	->	59	-0.03	0.00
64	->	60	-0.00	0.00
64	->	61	-0.05	0.01
64	->	62	-0.40	0.11
64	->	63	-15.42	1.13
64	->	64	-35.23	1.72
64	->	65	-12.28	0.61
64	->	66	-0.29	0.22
64	->	67	-0.19	0.30
64	->	68	-0.04	0.02
64	->	69	-0.08	0.02
64	->	70	-0.03	0.03
64	->	71	0.00	0.00
64	->	72	-0.02	0.00
64	->	73	-0.00	0.00
64	->	74	0.02	0.00
64	->	75	0.02	0.01
64	->	76	-0.98	1.14
64	->	77	-0.04	0.01
64	->	78	-0.03	0.01
64	->	79	-0.09	0.06
64	->	80	-0.34	0.43
64	->	81	-0.03	0.01
64	->	82	-0.01	0.00
64	->	83	-0.21	0.12
64	->	84	-0.03	0.03
64	->	85	-0.01	0.07
64	->	86	0.00	0.00
64	->	87	0.02	0.00
64	->	88	0.01	0.00
64	->	89	0.01	0.00
64	->	90	0.00	0.00
64	->	91	0.00	0.00
64	->	92	0.00	0.00
64	->	93	0.00	0.00
64	->	94	0.01	0.00
64	->	95	0.01	0.00
64	->	96	0.01	0.00
64	->	97	0.00	0.00
64	->	98	0.00	0.00
64	->	99	0.00	0.00
64	->	100	0.01	0.00
64	->	101	0.02	0.01
64	->	102	0.03	0.01
64	->	103	0.03	0.01
64	->	104	0.02	0.00
64	->	105	0.01	0.00

64	->	106	0.01	0.00
64	->	107	0.00	0.00
65	->	1	-0.00	0.00
65	->	2	0.00	0.00
65	->	3	0.00	0.00
65	->	4	0.00	0.00
65	->	5	0.00	0.00
65	->	6	0.00	0.00
65	->	7	0.00	0.00
65	->	8	0.00	0.00
65	->	9	0.00	0.00
65	->	10	0.00	0.00
65	->	11	0.00	0.00
65	->	12	-0.00	0.00
65	->	13	-0.00	0.00
65	->	14	0.00	0.00
65	->	15	0.00	0.00
65	->	16	-0.00	0.00
65	->	17	0.00	0.00
65	->	18	0.00	0.00
65	->	19	0.00	0.00
65	->	20	0.00	0.00
65	->	21	0.00	0.00
65	->	22	-0.00	0.00
65	->	23	-0.00	0.00
65	->	24	0.00	0.00
65	->	25	-0.00	0.00
65	->	26	0.00	0.00
65	->	27	0.00	0.00
65	->	28	0.00	0.00
65	->	29	0.00	0.00
65	->	30	0.00	0.00
65	->	31	-0.00	0.00
65	->	32	0.00	0.00
65	->	33	0.00	0.00
65	->	34	0.00	0.00
65	->	35	0.00	0.00
65	->	36	-0.00	0.00
65	->	37	0.00	0.00
65	->	38	0.00	0.00
65	->	39	0.00	0.00
65	->	40	-0.00	0.00
65	->	41	0.00	0.00
65	->	42	-0.00	0.00
65	->	43	0.00	0.00
65	->	44	-0.00	0.00
65	->	45	0.00	0.00
65	->	46	0.00	0.00
65	->	47	0.00	0.00
65	->	48	0.00	0.00

65	->	49	0.00	0.00
65	->	50	0.00	0.00
65	->	51	-0.00	0.00
65	->	52	0.00	0.00
65	->	53	-0.00	0.00
65	->	54	0.00	0.00
65	->	55	-0.00	0.00
65	->	56	-0.00	0.00
65	->	57	0.00	0.00
65	->	58	0.00	0.00
65	->	59	-0.00	0.00
65	->	60	-0.00	0.00
65	->	61	-0.00	0.00
65	->	62	-0.02	0.03
65	->	63	0.80	1.26
65	->	64	-12.46	0.62
65	->	65	21.34	1.31
65	->	66	-8.71	1.28
65	->	67	-0.35	0.14
65	->	68	-0.21	0.05
65	->	69	-0.01	0.00
65	->	70	-0.04	0.02
65	->	71	-0.00	0.00
65	->	72	-0.00	0.00
65	->	73	-0.00	0.00
65	->	74	0.00	0.00
65	->	75	0.00	0.00
65	->	76	-0.03	0.02
65	->	77	-0.01	0.00
65	->	78	-0.01	0.00
65	->	79	0.67	0.11
65	->	80	-0.11	0.08
65	->	81	-0.01	0.00
65	->	82	-0.04	0.02
65	->	83	0.82	0.59
65	->	84	-0.03	0.02
65	->	85	-0.07	0.31
65	->	86	-0.00	0.00
65	->	87	0.00	0.00
65	->	88	0.00	0.00
65	->	89	0.00	0.00
65	->	90	0.00	0.00
65	->	91	0.00	0.00
65	->	92	0.00	0.00
65	->	93	0.00	0.00
65	->	94	0.00	0.00
65	->	95	0.00	0.00
65	->	96	0.00	0.00
65	->	97	0.00	0.00
65	->	98	0.00	0.00

65	->	99	0.00	0.00
65	->	100	0.00	0.00
65	->	101	-0.00	0.00
65	->	102	-0.00	0.00
65	->	103	0.00	0.00
65	->	104	0.00	0.00
65	->	105	0.00	0.00
65	->	106	0.00	0.00
65	->	107	0.00	0.00
66	->	1	-0.00	0.00
66	->	2	-0.00	0.00
66	->	3	0.00	0.00
66	->	4	0.00	0.00
66	->	5	0.00	0.00
66	->	6	0.00	0.00
66	->	7	0.00	0.00
66	->	8	0.00	0.00
66	->	9	0.00	0.00
66	->	10	0.00	0.00
66	->	11	0.00	0.00
66	->	12	-0.00	0.00
66	->	13	-0.00	0.00
66	->	14	-0.00	0.00
66	->	15	-0.00	0.00
66	->	16	-0.00	0.00
66	->	17	0.00	0.00
66	->	18	-0.00	0.00
66	->	19	0.00	0.00
66	->	20	0.00	0.00
66	->	21	-0.00	0.00
66	->	22	-0.01	0.00
66	->	23	-0.00	0.00
66	->	24	0.00	0.00
66	->	25	-0.00	0.00
66	->	26	-0.00	0.00
66	->	27	-0.00	0.00
66	->	28	-0.00	0.00
66	->	29	-0.00	0.00
66	->	30	0.00	0.00
66	->	31	-0.00	0.00
66	->	32	0.00	0.00
66	->	33	-0.00	0.00
66	->	34	-0.00	0.00
66	->	35	0.01	0.00
66	->	36	-0.00	0.00
66	->	37	0.00	0.00
66	->	38	0.01	0.00
66	->	39	-0.00	0.00
66	->	40	-0.01	0.00
66	->	41	0.00	0.00

66 ->	42	-0.00	0.00
66 ->	43	0.00	0.00
66 ->	44	-0.01	0.00
66 ->	45	0.00	0.00
66 ->	46	0.01	0.00
66 ->	47	0.00	0.00
66 ->	48	0.00	0.00
66 ->	49	0.00	0.00
66 ->	50	0.00	0.00
66 ->	51	-0.00	0.00
66 ->	52	0.00	0.00
66 ->	53	-0.02	0.00
66 ->	54	0.00	0.00
66 ->	55	-0.00	0.00
66 ->	56	0.00	0.00
66 ->	57	0.00	0.00
66 ->	58	0.02	0.00
66 ->	59	-0.06	0.01
66 ->	60	0.00	0.00
66 ->	61	-0.01	0.00
66 ->	62	-0.15	0.06
66 ->	63	3.07	0.49
66 ->	64	-0.30	0.23
66 ->	65	-8.64	1.27
66 ->	66	-32.32	2.13
66 ->	67	-8.89	0.37
66 ->	68	-3.52	0.61
66 ->	69	-0.04	0.03
66 ->	70	0.01	0.04
66 ->	71	-0.00	0.00
66 ->	72	-0.02	0.00
66 ->	73	0.00	0.00
66 ->	74	0.03	0.00
66 ->	75	0.05	0.01
66 ->	76	-0.09	0.03
66 ->	77	-0.03	0.01
66 ->	78	-0.07	0.01
66 ->	79	3.32	0.47
66 ->	80	0.03	0.01
66 ->	81	-0.03	0.01
66 ->	82	-0.06	0.03
66 ->	83	2.01	0.35
66 ->	84	-0.00	0.00
66 ->	85	-0.01	0.03
66 ->	86	0.01	0.01
66 ->	87	0.05	0.02
66 ->	88	0.02	0.00
66 ->	89	0.01	0.00
66 ->	90	0.01	0.00
66 ->	91	0.00	0.00

66	->	92	0.00	0.00
66	->	93	0.00	0.00
66	->	94	0.01	0.00
66	->	95	0.01	0.00
66	->	96	0.00	0.00
66	->	97	0.00	0.00
66	->	98	0.00	0.00
66	->	99	0.00	0.00
66	->	100	0.01	0.00
66	->	101	0.01	0.00
66	->	102	0.02	0.01
66	->	103	0.02	0.00
66	->	104	0.02	0.00
66	->	105	0.01	0.00
66	->	106	0.01	0.00
66	->	107	0.00	0.00
67	->	1	0.00	0.00
67	->	2	0.00	0.00
67	->	3	0.00	0.00
67	->	4	0.00	0.00
67	->	5	0.00	0.00
67	->	6	0.00	0.00
67	->	7	0.00	0.00
67	->	8	-0.00	0.00
67	->	9	0.00	0.00
67	->	10	0.00	0.00
67	->	11	-0.00	0.00
67	->	12	0.00	0.00
67	->	13	0.00	0.00
67	->	14	0.00	0.00
67	->	15	0.00	0.00
67	->	16	0.00	0.00
67	->	17	0.00	0.00
67	->	18	0.00	0.00
67	->	19	0.00	0.00
67	->	20	0.00	0.00
67	->	21	-0.00	0.00
67	->	22	-0.00	0.00
67	->	23	0.00	0.00
67	->	24	0.00	0.00
67	->	25	0.00	0.00
67	->	26	0.00	0.00
67	->	27	0.00	0.00
67	->	28	0.00	0.00
67	->	29	0.00	0.00
67	->	30	0.00	0.00
67	->	31	-0.00	0.00
67	->	32	0.00	0.00
67	->	33	-0.00	0.00
67	->	34	0.00	0.00

67 ->	35	0.00	0.00
67 ->	36	-0.00	0.00
67 ->	37	0.00	0.00
67 ->	38	0.00	0.00
67 ->	39	-0.00	0.00
67 ->	40	-0.00	0.00
67 ->	41	0.00	0.00
67 ->	42	0.00	0.00
67 ->	43	0.00	0.00
67 ->	44	0.00	0.00
67 ->	45	0.00	0.00
67 ->	46	0.00	0.00
67 ->	47	0.00	0.00
67 ->	48	0.00	0.00
67 ->	49	0.00	0.00
67 ->	50	0.00	0.00
67 ->	51	0.00	0.00
67 ->	52	0.00	0.00
67 ->	53	-0.00	0.00
67 ->	54	0.00	0.00
67 ->	55	0.00	0.00
67 ->	56	0.00	0.00
67 ->	57	-0.00	0.00
67 ->	58	0.00	0.00
67 ->	59	-0.01	0.01
67 ->	60	-0.00	0.00
67 ->	61	-0.02	0.01
67 ->	62	-0.11	0.14
67 ->	63	-0.74	0.67
67 ->	64	-0.19	0.31
67 ->	65	-0.35	0.14
67 ->	66	-8.96	0.38
67 ->	67	11.49	0.79
67 ->	68	-8.69	0.38
67 ->	69	-0.99	0.68
67 ->	70	-0.09	0.02
67 ->	71	-0.00	0.00
67 ->	72	-0.00	0.00
67 ->	73	-0.00	0.00
67 ->	74	-0.00	0.00
67 ->	75	-0.00	0.00
67 ->	76	-0.01	0.00
67 ->	77	-0.00	0.00
67 ->	78	0.00	0.00
67 ->	79	-0.07	0.03
67 ->	80	-0.01	0.00
67 ->	81	-0.00	0.00
67 ->	82	-0.00	0.00
67 ->	83	-0.02	0.02
67 ->	84	-0.00	0.00

67	->	85	-0.00	0.00
67	->	86	-0.00	0.01
67	->	87	-0.00	0.00
67	->	88	-0.00	0.00
67	->	89	0.00	0.00
67	->	90	0.00	0.00
67	->	91	0.00	0.00
67	->	92	0.00	0.00
67	->	93	-0.00	0.00
67	->	94	-0.00	0.00
67	->	95	0.00	0.00
67	->	96	-0.00	0.00
67	->	97	0.00	0.00
67	->	98	0.00	0.00
67	->	99	0.00	0.00
67	->	100	0.00	0.00
67	->	101	0.00	0.00
67	->	102	-0.00	0.00
67	->	103	-0.00	0.00
67	->	104	-0.00	0.00
67	->	105	-0.00	0.00
67	->	106	-0.00	0.00
67	->	107	0.00	0.00
68	->	1	0.01	0.00
68	->	2	0.00	0.00
68	->	3	0.00	0.00
68	->	4	0.00	0.00
68	->	5	-0.00	0.00
68	->	6	0.00	0.00
68	->	7	0.00	0.00
68	->	8	-0.00	0.00
68	->	9	-0.00	0.00
68	->	10	-0.00	0.00
68	->	11	-0.00	0.00
68	->	12	0.00	0.00
68	->	13	0.00	0.00
68	->	14	0.00	0.00
68	->	15	0.00	0.00
68	->	16	0.00	0.00
68	->	17	-0.00	0.00
68	->	18	-0.00	0.00
68	->	19	-0.00	0.00
68	->	20	-0.00	0.00
68	->	21	0.00	0.00
68	->	22	0.01	0.00
68	->	23	0.00	0.00
68	->	24	0.00	0.00
68	->	25	0.00	0.00
68	->	26	0.00	0.00
68	->	27	0.00	0.00

68	->	28	0.00	0.00
68	->	29	0.00	0.00
68	->	30	-0.00	0.00
68	->	31	0.00	0.00
68	->	32	0.00	0.00
68	->	33	-0.00	0.00
68	->	34	0.00	0.00
68	->	35	-0.01	0.00
68	->	36	0.01	0.00
68	->	37	-0.00	0.00
68	->	38	-0.01	0.00
68	->	39	0.00	0.00
68	->	40	0.01	0.00
68	->	41	-0.00	0.00
68	->	42	0.00	0.00
68	->	43	0.00	0.00
68	->	44	0.01	0.00
68	->	45	-0.00	0.00
68	->	46	-0.01	0.00
68	->	47	-0.00	0.00
68	->	48	-0.00	0.00
68	->	49	-0.00	0.00
68	->	50	-0.00	0.00
68	->	51	-0.00	0.00
68	->	52	-0.00	0.00
68	->	53	0.03	0.01
68	->	54	-0.01	0.00
68	->	55	-0.00	0.00
68	->	56	-0.00	0.00
68	->	57	-0.00	0.00
68	->	58	-0.02	0.01
68	->	59	0.18	0.28
68	->	60	-0.02	0.01
68	->	61	-0.30	0.07
68	->	62	-1.19	0.58
68	->	63	-2.17	0.74
68	->	64	-0.04	0.02
68	->	65	-0.22	0.05
68	->	66	-3.62	0.61
68	->	67	-8.83	0.39
68	->	68	-107.12	2.54
68	->	69	-13.23	0.94
68	->	70	-3.28	0.78
68	->	71	-0.07	0.04
68	->	72	0.03	0.01
68	->	73	-0.01	0.01
68	->	74	-0.05	0.01
68	->	75	-1.53	2.88
68	->	76	-0.07	0.05
68	->	77	0.01	0.01

68	->	78	0.09	0.04
68	->	79	-1.96	0.71
68	->	80	-0.04	0.02
68	->	81	0.03	0.01
68	->	82	-0.04	0.05
68	->	83	-0.23	0.06
68	->	84	-0.00	0.00
68	->	85	0.00	0.01
68	->	86	-1.78	1.64
68	->	87	-2.77	2.54
68	->	88	-0.06	0.02
68	->	89	-0.03	0.01
68	->	90	-0.01	0.00
68	->	91	-0.01	0.00
68	->	92	-0.01	0.00
68	->	93	-0.00	0.00
68	->	94	-0.00	0.00
68	->	95	-0.00	0.00
68	->	96	-0.00	0.00
68	->	97	-0.00	0.00
68	->	98	-0.00	0.00
68	->	99	-0.01	0.00
68	->	100	-0.01	0.00
68	->	101	-0.02	0.00
68	->	102	-0.02	0.01
68	->	103	-0.02	0.01
68	->	104	-0.02	0.00
68	->	105	-0.02	0.01
68	->	106	-0.02	0.01
68	->	107	-0.01	0.00
69	->	1	0.00	0.00
69	->	2	0.00	0.00
69	->	3	-0.00	0.00
69	->	4	0.00	0.00
69	->	5	-0.00	0.00
69	->	6	0.00	0.00
69	->	7	0.00	0.00
69	->	8	-0.00	0.00
69	->	9	-0.00	0.00
69	->	10	-0.00	0.00
69	->	11	-0.00	0.00
69	->	12	0.00	0.00
69	->	13	0.00	0.00
69	->	14	0.00	0.00
69	->	15	0.00	0.00
69	->	16	0.01	0.00
69	->	17	-0.00	0.00
69	->	18	0.00	0.00
69	->	19	-0.00	0.00
69	->	20	-0.00	0.00

69	->	21	0.00	0.00
69	->	22	0.01	0.00
69	->	23	0.00	0.00
69	->	24	0.00	0.00
69	->	25	0.00	0.00
69	->	26	0.00	0.00
69	->	27	0.00	0.00
69	->	28	0.00	0.00
69	->	29	0.00	0.00
69	->	30	-0.00	0.00
69	->	31	0.00	0.00
69	->	32	-0.00	0.00
69	->	33	0.00	0.00
69	->	34	-0.00	0.00
69	->	35	-0.01	0.00
69	->	36	0.01	0.00
69	->	37	-0.00	0.00
69	->	38	-0.01	0.00
69	->	39	0.00	0.00
69	->	40	0.01	0.00
69	->	41	-0.00	0.00
69	->	42	0.00	0.00
69	->	43	-0.00	0.00
69	->	44	0.01	0.00
69	->	45	-0.00	0.00
69	->	46	-0.02	0.00
69	->	47	-0.00	0.00
69	->	48	-0.00	0.00
69	->	49	-0.00	0.00
69	->	50	-0.01	0.00
69	->	51	-0.00	0.00
69	->	52	-0.00	0.00
69	->	53	0.08	0.02
69	->	54	-0.02	0.01
69	->	55	-0.00	0.00
69	->	56	-0.01	0.00
69	->	57	-0.02	0.01
69	->	58	-0.06	0.08
69	->	59	-2.27	0.87
69	->	60	-0.73	0.89
69	->	61	-3.15	0.66
69	->	62	-1.47	0.37
69	->	63	-0.61	0.14
69	->	64	-0.08	0.02
69	->	65	-0.01	0.00
69	->	66	-0.04	0.03
69	->	67	-1.05	0.68
69	->	68	-13.22	0.94
69	->	69	10.62	2.83
69	->	70	-10.75	0.60

69	->	71	-0.44	0.05
69	->	72	-0.01	0.03
69	->	73	-0.05	0.02
69	->	74	-0.05	0.01
69	->	75	-0.17	0.28
69	->	76	0.08	0.05
69	->	77	-0.00	0.01
69	->	78	0.04	0.01
69	->	79	-0.03	0.02
69	->	80	-0.01	0.00
69	->	81	0.01	0.00
69	->	82	-0.00	0.00
69	->	83	-0.01	0.01
69	->	84	-0.00	0.00
69	->	85	0.00	0.00
69	->	86	-0.04	0.05
69	->	87	-0.12	0.11
69	->	88	-0.04	0.02
69	->	89	-0.02	0.00
69	->	90	-0.01	0.00
69	->	91	-0.01	0.00
69	->	92	-0.00	0.00
69	->	93	-0.00	0.00
69	->	94	-0.01	0.00
69	->	95	-0.01	0.00
69	->	96	-0.00	0.00
69	->	97	-0.00	0.00
69	->	98	-0.00	0.00
69	->	99	-0.01	0.00
69	->	100	-0.02	0.01
69	->	101	-0.04	0.02
69	->	102	-0.05	0.03
69	->	103	-0.02	0.01
69	->	104	-0.01	0.00
69	->	105	-0.01	0.00
69	->	106	-0.01	0.00
69	->	107	-0.00	0.00
70	->	1	0.00	0.00
70	->	2	0.00	0.00
70	->	3	0.00	0.00
70	->	4	0.00	0.00
70	->	5	-0.00	0.00
70	->	6	0.00	0.00
70	->	7	0.00	0.00
70	->	8	-0.00	0.00
70	->	9	0.00	0.00
70	->	10	-0.00	0.00
70	->	11	-0.00	0.00
70	->	12	0.00	0.00
70	->	13	0.00	0.00

70 ->	14	0.00	0.00
70 ->	15	0.00	0.00
70 ->	16	0.00	0.00
70 ->	17	-0.00	0.00
70 ->	18	0.00	0.00
70 ->	19	-0.00	0.00
70 ->	20	0.00	0.00
70 ->	21	0.00	0.00
70 ->	22	0.00	0.00
70 ->	23	0.00	0.00
70 ->	24	0.00	0.00
70 ->	25	0.00	0.00
70 ->	26	0.00	0.00
70 ->	27	0.00	0.00
70 ->	28	0.00	0.00
70 ->	29	0.00	0.00
70 ->	30	-0.00	0.00
70 ->	31	0.00	0.00
70 ->	32	0.00	0.00
70 ->	33	0.00	0.00
70 ->	34	0.00	0.00
70 ->	35	-0.00	0.00
70 ->	36	-0.00	0.00
70 ->	37	-0.00	0.00
70 ->	38	-0.00	0.00
70 ->	39	0.00	0.00
70 ->	40	0.00	0.00
70 ->	41	0.00	0.00
70 ->	42	-0.00	0.00
70 ->	43	0.00	0.00
70 ->	44	0.00	0.00
70 ->	45	0.00	0.00
70 ->	46	-0.00	0.00
70 ->	47	-0.00	0.00
70 ->	48	0.00	0.00
70 ->	49	0.00	0.00
70 ->	50	-0.03	0.02
70 ->	51	-0.01	0.00
70 ->	52	0.00	0.00
70 ->	53	0.03	0.04
70 ->	54	-0.02	0.01
70 ->	55	-0.01	0.00
70 ->	56	-0.01	0.00
70 ->	57	-0.00	0.01
70 ->	58	-0.27	0.10
70 ->	59	-3.14	0.48
70 ->	60	-0.89	0.17
70 ->	61	-3.55	0.54
70 ->	62	-1.18	0.42
70 ->	63	-0.97	0.29

70	->	64	-0.03	0.03
70	->	65	-0.04	0.02
70	->	66	0.01	0.04
70	->	67	-0.09	0.02
70	->	68	-3.24	0.76
70	->	69	-10.82	0.60
70	->	70	30.19	1.13
70	->	71	-9.93	0.40
70	->	72	-1.04	0.19
70	->	73	-0.24	0.08
70	->	74	-0.13	0.03
70	->	75	-3.08	1.74
70	->	76	-2.85	0.37
70	->	77	-0.05	0.02
70	->	78	-0.19	0.06
70	->	79	-1.97	0.40
70	->	80	-0.08	0.06
70	->	81	-0.01	0.01
70	->	82	-0.03	0.01
70	->	83	-0.01	0.02
70	->	84	-0.00	0.00
70	->	85	-0.00	0.00
70	->	86	-0.09	0.19
70	->	87	-0.16	0.25
70	->	88	-0.08	0.07
70	->	89	-0.02	0.01
70	->	90	-0.01	0.00
70	->	91	-0.00	0.00
70	->	92	-0.00	0.00
70	->	93	-0.00	0.00
70	->	94	-0.00	0.00
70	->	95	-0.00	0.00
70	->	96	-0.00	0.00
70	->	97	-0.00	0.00
70	->	98	-0.00	0.00
70	->	99	-0.00	0.00
70	->	100	-0.00	0.00
70	->	101	-0.01	0.01
70	->	102	-0.02	0.02
70	->	103	-0.01	0.00
70	->	104	-0.01	0.00
70	->	105	-0.01	0.00
70	->	106	-0.00	0.00
70	->	107	-0.00	0.00
71	->	1	-0.00	0.00
71	->	2	0.00	0.00
71	->	3	0.00	0.00
71	->	4	0.00	0.00
71	->	5	0.00	0.00
71	->	6	0.00	0.00

71	->	7	0.00	0.00
71	->	8	0.00	0.00
71	->	9	0.00	0.00
71	->	10	0.00	0.00
71	->	11	-0.00	0.00
71	->	12	-0.00	0.00
71	->	13	-0.00	0.00
71	->	14	0.00	0.00
71	->	15	-0.00	0.00
71	->	16	-0.00	0.00
71	->	17	0.00	0.00
71	->	18	0.00	0.00
71	->	19	0.00	0.00
71	->	20	0.00	0.00
71	->	21	-0.00	0.00
71	->	22	-0.00	0.00
71	->	23	-0.00	0.00
71	->	24	-0.00	0.00
71	->	25	-0.00	0.00
71	->	26	0.00	0.00
71	->	27	0.00	0.00
71	->	28	0.00	0.00
71	->	29	0.00	0.00
71	->	30	0.00	0.00
71	->	31	0.00	0.00
71	->	32	0.00	0.00
71	->	33	-0.00	0.00
71	->	34	0.00	0.00
71	->	35	-0.00	0.00
71	->	36	-0.00	0.00
71	->	37	-0.00	0.00
71	->	38	-0.00	0.00
71	->	39	0.00	0.00
71	->	40	-0.00	0.00
71	->	41	0.00	0.00
71	->	42	-0.00	0.00
71	->	43	0.00	0.00
71	->	44	0.00	0.00
71	->	45	-0.00	0.00
71	->	46	-0.01	0.01
71	->	47	-0.01	0.01
71	->	48	-0.01	0.00
71	->	49	-0.05	0.04
71	->	50	-0.42	0.26
71	->	51	-0.02	0.01
71	->	52	-0.02	0.01
71	->	53	-3.11	1.85
71	->	54	-0.30	0.26
71	->	55	-0.01	0.00
71	->	56	-0.02	0.01

71	->	57	-0.04	0.01
71	->	58	-0.70	0.31
71	->	59	-1.64	0.41
71	->	60	-0.75	0.17
71	->	61	-0.92	0.28
71	->	62	-0.01	0.01
71	->	63	0.01	0.01
71	->	64	0.00	0.00
71	->	65	-0.00	0.00
71	->	66	-0.00	0.00
71	->	67	-0.00	0.00
71	->	68	-0.07	0.04
71	->	69	-0.44	0.05
71	->	70	-9.92	0.39
71	->	71	23.36	1.23
71	->	72	-7.84	0.54
71	->	73	-0.34	0.08
71	->	74	-0.18	0.07
71	->	75	-1.70	2.18
71	->	76	-0.02	0.02
71	->	77	-0.01	0.00
71	->	78	-0.00	0.01
71	->	79	-0.00	0.00
71	->	80	-0.00	0.00
71	->	81	0.00	0.00
71	->	82	-0.00	0.00
71	->	83	0.00	0.00
71	->	84	0.00	0.00
71	->	85	0.00	0.00
71	->	86	-0.00	0.00
71	->	87	-0.09	0.20
71	->	88	-0.15	0.22
71	->	89	-0.03	0.01
71	->	90	-0.01	0.00
71	->	91	-0.00	0.00
71	->	92	0.00	0.00
71	->	93	0.00	0.00
71	->	94	-0.00	0.00
71	->	95	0.00	0.00
71	->	96	0.00	0.00
71	->	97	0.00	0.00
71	->	98	0.00	0.00
71	->	99	0.00	0.00
71	->	100	-0.00	0.00
71	->	101	-0.02	0.01
71	->	102	-0.04	0.03
71	->	103	-0.01	0.01
71	->	104	0.00	0.00
71	->	105	-0.00	0.00
71	->	106	-0.00	0.00

71	->	107	-0.00	0.00
72	->	1	0.02	0.00
72	->	2	0.00	0.00
72	->	3	-0.00	0.00
72	->	4	0.00	0.00
72	->	5	-0.01	0.00
72	->	6	0.00	0.00
72	->	7	0.00	0.00
72	->	8	-0.00	0.00
72	->	9	-0.00	0.00
72	->	10	-0.01	0.00
72	->	11	-0.01	0.00
72	->	12	0.02	0.00
72	->	13	0.01	0.00
72	->	14	0.00	0.00
72	->	15	0.00	0.00
72	->	16	0.02	0.00
72	->	17	-0.00	0.00
72	->	18	-0.00	0.00
72	->	19	-0.03	0.00
72	->	20	-0.00	0.00
72	->	21	0.00	0.00
72	->	22	0.05	0.01
72	->	23	0.00	0.00
72	->	24	0.00	0.00
72	->	25	0.02	0.00
72	->	26	0.00	0.00
72	->	27	0.00	0.00
72	->	28	0.00	0.00
72	->	29	0.00	0.00
72	->	30	-0.00	0.00
72	->	31	0.01	0.00
72	->	32	0.00	0.00
72	->	33	-0.00	0.00
72	->	34	-0.00	0.00
72	->	35	-0.03	0.00
72	->	36	0.01	0.00
72	->	37	-0.00	0.00
72	->	38	-0.03	0.00
72	->	39	0.00	0.00
72	->	40	0.05	0.02
72	->	41	-0.00	0.00
72	->	42	-0.01	0.00
72	->	43	-0.00	0.00
72	->	44	0.11	0.03
72	->	45	-0.01	0.00
72	->	46	-0.12	0.02
72	->	47	-0.12	0.07
72	->	48	-0.01	0.01
72	->	49	0.02	0.02

72 ->	50	-1.46	0.34
72 ->	51	-0.06	0.02
72 ->	52	-0.02	0.01
72 ->	53	-0.36	0.31
72 ->	54	-0.09	0.05
72 ->	55	-0.01	0.00
72 ->	56	-0.00	0.00
72 ->	57	-0.01	0.00
72 ->	58	-0.04	0.01
72 ->	59	-0.06	0.06
72 ->	60	-0.15	0.10
72 ->	61	-1.75	0.74
72 ->	62	-0.03	0.01
72 ->	63	-0.04	0.01
72 ->	64	-0.02	0.00
72 ->	65	-0.00	0.00
72 ->	66	-0.02	0.00
72 ->	67	-0.00	0.00
72 ->	68	0.03	0.01
72 ->	69	-0.01	0.03
72 ->	70	-1.04	0.19
72 ->	71	-7.93	0.54
72 ->	72	-94.69	2.88
72 ->	73	-21.63	0.44
72 ->	74	-12.97	3.24
72 ->	75	-3.91	1.25
72 ->	76	-0.85	0.45
72 ->	77	0.09	0.05
72 ->	78	0.51	0.35
72 ->	79	-0.01	0.01
72 ->	80	-0.01	0.01
72 ->	81	0.04	0.01
72 ->	82	-0.00	0.00
72 ->	83	-0.00	0.00
72 ->	84	-0.00	0.00
72 ->	85	0.00	0.01
72 ->	86	-0.04	0.01
72 ->	87	-0.27	0.39
72 ->	88	-2.18	0.83
72 ->	89	-8.59	1.06
72 ->	90	-2.26	0.94
72 ->	91	-0.12	0.18
72 ->	92	-0.01	0.02
72 ->	93	-0.00	0.01
72 ->	94	-0.02	0.00
72 ->	95	-0.02	0.00
72 ->	96	-0.01	0.00
72 ->	97	-0.00	0.00
72 ->	98	-0.01	0.00
72 ->	99	-0.03	0.00

72	->	100	-0.04	0.01
72	->	101	-0.13	0.05
72	->	102	-0.50	0.35
72	->	103	-0.33	0.26
72	->	104	0.19	0.58
72	->	105	0.45	0.24
72	->	106	-0.06	0.18
72	->	107	-0.05	0.01
73	->	1	0.00	0.00
73	->	2	0.00	0.00
73	->	3	-0.00	0.00
73	->	4	-0.00	0.00
73	->	5	-0.00	0.00
73	->	6	0.00	0.00
73	->	7	0.00	0.00
73	->	8	-0.00	0.00
73	->	9	0.00	0.00
73	->	10	-0.00	0.00
73	->	11	-0.00	0.00
73	->	12	0.00	0.00
73	->	13	0.00	0.00
73	->	14	0.00	0.00
73	->	15	0.00	0.00
73	->	16	0.00	0.00
73	->	17	0.00	0.00
73	->	18	0.00	0.00
73	->	19	-0.00	0.00
73	->	20	0.00	0.00
73	->	21	0.00	0.00
73	->	22	0.00	0.00
73	->	23	0.00	0.00
73	->	24	0.00	0.00
73	->	25	0.00	0.00
73	->	26	0.00	0.00
73	->	27	0.00	0.00
73	->	28	0.00	0.00
73	->	29	0.00	0.00
73	->	30	-0.00	0.00
73	->	31	0.00	0.00
73	->	32	0.00	0.00
73	->	33	-0.00	0.00
73	->	34	0.00	0.00
73	->	35	-0.00	0.00
73	->	36	0.00	0.00
73	->	37	0.00	0.00
73	->	38	-0.00	0.00
73	->	39	0.00	0.00
73	->	40	-0.00	0.00
73	->	41	-0.00	0.00
73	->	42	-0.00	0.00

73	->	43	-0.00	0.00
73	->	44	-0.01	0.01
73	->	45	-0.00	0.00
73	->	46	-0.01	0.01
73	->	47	-0.00	0.00
73	->	48	-0.00	0.00
73	->	49	-0.02	0.01
73	->	50	-0.00	0.03
73	->	51	0.00	0.00
73	->	52	-0.00	0.00
73	->	53	-0.17	0.13
73	->	54	0.00	0.00
73	->	55	-0.00	0.00
73	->	56	-0.00	0.00
73	->	57	-0.00	0.00
73	->	58	-0.01	0.00
73	->	59	-0.07	0.02
73	->	60	-0.06	0.02
73	->	61	-1.64	0.77
73	->	62	-0.02	0.01
73	->	63	-0.00	0.01
73	->	64	-0.00	0.00
73	->	65	-0.00	0.00
73	->	66	0.00	0.00
73	->	67	-0.00	0.00
73	->	68	-0.01	0.01
73	->	69	-0.05	0.02
73	->	70	-0.24	0.08
73	->	71	-0.34	0.08
73	->	72	-21.63	0.44
73	->	73	23.02	3.04
73	->	74	-10.74	0.51
73	->	75	-0.79	0.18
73	->	76	-2.62	0.76
73	->	77	-1.69	1.09
73	->	78	-0.09	0.09
73	->	79	-0.04	0.03
73	->	80	-0.01	0.02
73	->	81	-0.01	0.00
73	->	82	-0.00	0.00
73	->	83	-0.00	0.00
73	->	84	-0.00	0.00
73	->	85	-0.00	0.00
73	->	86	-0.00	0.00
73	->	87	-0.00	0.01
73	->	88	-0.00	0.01
73	->	89	-0.01	0.01
73	->	90	-0.01	0.00
73	->	91	0.00	0.01
73	->	92	0.01	0.02

73	->	93	-0.02	0.03
73	->	94	-0.01	0.00
73	->	95	-0.01	0.00
73	->	96	-0.00	0.00
73	->	97	-0.00	0.00
73	->	98	-0.00	0.00
73	->	99	-0.00	0.00
73	->	100	-0.02	0.01
73	->	101	-0.67	0.61
73	->	102	-3.77	3.36
73	->	103	-0.49	0.41
73	->	104	-0.05	0.02
73	->	105	-0.01	0.00
73	->	106	-0.00	0.00
73	->	107	-0.00	0.00
74	->	1	-0.02	0.00
74	->	2	-0.00	0.00
74	->	3	0.00	0.00
74	->	4	-0.00	0.00
74	->	5	0.01	0.00
74	->	6	-0.00	0.00
74	->	7	-0.00	0.00
74	->	8	0.00	0.00
74	->	9	0.00	0.00
74	->	10	0.01	0.00
74	->	11	0.01	0.00
74	->	12	-0.02	0.00
74	->	13	-0.00	0.00
74	->	14	-0.00	0.00
74	->	15	-0.00	0.00
74	->	16	-0.03	0.00
74	->	17	0.00	0.00
74	->	18	-0.00	0.00
74	->	19	0.03	0.00
74	->	20	0.00	0.00
74	->	21	-0.00	0.00
74	->	22	-0.04	0.01
74	->	23	-0.00	0.00
74	->	24	-0.00	0.00
74	->	25	-0.02	0.00
74	->	26	-0.00	0.00
74	->	27	-0.00	0.00
74	->	28	-0.00	0.00
74	->	29	-0.00	0.00
74	->	30	0.00	0.00
74	->	31	-0.01	0.00
74	->	32	-0.00	0.00
74	->	33	0.00	0.00
74	->	34	-0.00	0.00
74	->	35	0.02	0.00

74	->	36	-0.01	0.00
74	->	37	0.00	0.00
74	->	38	0.02	0.00
74	->	39	-0.00	0.00
74	->	40	-0.03	0.01
74	->	41	0.00	0.00
74	->	42	-0.00	0.00
74	->	43	-0.00	0.00
74	->	44	-0.08	0.02
74	->	45	0.00	0.00
74	->	46	0.06	0.01
74	->	47	-0.00	0.01
74	->	48	-0.00	0.00
74	->	49	-0.02	0.01
74	->	50	-0.01	0.02
74	->	51	-0.00	0.00
74	->	52	-0.00	0.00
74	->	53	-0.11	0.05
74	->	54	-0.01	0.00
74	->	55	-0.00	0.00
74	->	56	-0.00	0.00
74	->	57	-0.00	0.00
74	->	58	0.03	0.01
74	->	59	-0.07	0.01
74	->	60	-0.01	0.00
74	->	61	-0.01	0.01
74	->	62	0.00	0.00
74	->	63	0.03	0.01
74	->	64	0.02	0.00
74	->	65	0.00	0.00
74	->	66	0.03	0.00
74	->	67	-0.00	0.00
74	->	68	-0.05	0.01
74	->	69	-0.05	0.01
74	->	70	-0.13	0.03
74	->	71	-0.18	0.07
74	->	72	-12.89	3.23
74	->	73	-10.80	0.52
74	->	74	-14.02	2.42
74	->	75	-13.39	0.81
74	->	76	-1.78	0.32
74	->	77	-2.68	0.50
74	->	78	-3.48	2.44
74	->	79	-0.14	0.07
74	->	80	-0.03	0.02
74	->	81	-0.11	0.04
74	->	82	-0.01	0.00
74	->	83	-0.01	0.00
74	->	84	-0.00	0.00
74	->	85	-0.00	0.01

74	->	86	0.00	0.01
74	->	87	0.02	0.02
74	->	88	-0.48	0.79
74	->	89	0.46	0.19
74	->	90	0.12	0.10
74	->	91	-0.09	0.07
74	->	92	-0.05	0.03
74	->	93	-0.01	0.02
74	->	94	0.02	0.00
74	->	95	0.02	0.00
74	->	96	0.01	0.00
74	->	97	0.00	0.00
74	->	98	0.01	0.00
74	->	99	0.02	0.00
74	->	100	0.02	0.01
74	->	101	0.03	0.02
74	->	102	-0.06	0.18
74	->	103	-2.12	0.54
74	->	104	-3.25	1.33
74	->	105	-0.82	0.59
74	->	106	0.05	0.05
74	->	107	0.01	0.00
75	->	1	-0.01	0.00
75	->	2	-0.00	0.00
75	->	3	0.00	0.00
75	->	4	-0.00	0.00
75	->	5	0.00	0.00
75	->	6	-0.00	0.00
75	->	7	-0.00	0.00
75	->	8	0.00	0.00
75	->	9	0.00	0.00
75	->	10	0.01	0.00
75	->	11	0.00	0.00
75	->	12	-0.01	0.00
75	->	13	-0.00	0.00
75	->	14	-0.00	0.00
75	->	15	-0.00	0.00
75	->	16	-0.01	0.00
75	->	17	0.00	0.00
75	->	18	0.00	0.00
75	->	19	0.01	0.00
75	->	20	0.00	0.00
75	->	21	-0.00	0.00
75	->	22	-0.02	0.00
75	->	23	-0.00	0.00
75	->	24	0.00	0.00
75	->	25	-0.01	0.00
75	->	26	-0.00	0.00
75	->	27	-0.00	0.00
75	->	28	-0.00	0.00

75	->	29	-0.00	0.00
75	->	30	0.00	0.00
75	->	31	-0.01	0.00
75	->	32	-0.00	0.00
75	->	33	0.00	0.00
75	->	34	-0.00	0.00
75	->	35	0.02	0.00
75	->	36	-0.01	0.00
75	->	37	0.00	0.00
75	->	38	0.02	0.00
75	->	39	-0.00	0.00
75	->	40	-0.03	0.00
75	->	41	0.00	0.00
75	->	42	-0.00	0.00
75	->	43	-0.00	0.00
75	->	44	-0.03	0.01
75	->	45	0.00	0.00
75	->	46	0.03	0.00
75	->	47	-0.00	0.00
75	->	48	0.00	0.00
75	->	49	-0.00	0.00
75	->	50	-0.02	0.01
75	->	51	-0.01	0.00
75	->	52	-0.00	0.00
75	->	53	-0.08	0.07
75	->	54	-0.01	0.01
75	->	55	-0.00	0.00
75	->	56	-0.00	0.00
75	->	57	-0.00	0.00
75	->	58	0.04	0.01
75	->	59	-0.65	1.63
75	->	60	-0.01	0.01
75	->	61	-0.10	0.04
75	->	62	-0.01	0.01
75	->	63	-0.00	0.02
75	->	64	0.02	0.01
75	->	65	0.00	0.00
75	->	66	0.05	0.01
75	->	67	-0.00	0.00
75	->	68	-1.51	2.85
75	->	69	-0.17	0.28
75	->	70	-3.06	1.71
75	->	71	-1.68	2.16
75	->	72	-3.89	1.25
75	->	73	-0.80	0.18
75	->	74	-13.47	0.82
75	->	75	-44.82	5.91
75	->	76	-7.54	0.56
75	->	77	-1.33	0.29
75	->	78	-9.47	6.15

75 ->	79	-3.46	0.64
75 ->	80	-0.15	0.05
75 ->	81	-0.11	0.04
75 ->	82	-0.06	0.03
75 ->	83	-0.02	0.00
75 ->	84	-0.00	0.00
75 ->	85	-0.01	0.02
75 ->	86	-0.08	0.10
75 ->	87	-0.08	0.62
75 ->	88	-2.95	3.03
75 ->	89	0.16	0.26
75 ->	90	0.00	0.02
75 ->	91	0.00	0.02
75 ->	92	0.00	0.00
75 ->	93	0.01	0.00
75 ->	94	0.01	0.00
75 ->	95	0.01	0.00
75 ->	96	0.00	0.00
75 ->	97	0.00	0.00
75 ->	98	0.01	0.00
75 ->	99	0.01	0.00
75 ->	100	0.01	0.00
75 ->	101	0.01	0.00
75 ->	102	-0.01	0.04
75 ->	103	-0.02	0.03
75 ->	104	-0.08	0.12
75 ->	105	-0.42	0.51
75 ->	106	0.21	0.24
75 ->	107	-0.00	0.02
76 ->	1	0.01	0.00
76 ->	2	0.00	0.00
76 ->	3	-0.00	0.00
76 ->	4	0.00	0.00
76 ->	5	-0.00	0.00
76 ->	6	0.00	0.00
76 ->	7	0.00	0.00
76 ->	8	-0.00	0.00
76 ->	9	-0.00	0.00
76 ->	10	-0.00	0.00
76 ->	11	-0.00	0.00
76 ->	12	0.01	0.00
76 ->	13	0.00	0.00
76 ->	14	0.00	0.00
76 ->	15	0.00	0.00
76 ->	16	0.02	0.01
76 ->	17	-0.00	0.00
76 ->	18	0.00	0.00
76 ->	19	-0.01	0.01
76 ->	20	0.00	0.00
76 ->	21	0.00	0.00

76	->	22	0.02	0.01
76	->	23	0.00	0.00
76	->	24	0.00	0.00
76	->	25	0.01	0.00
76	->	26	0.00	0.00
76	->	27	0.00	0.00
76	->	28	0.00	0.00
76	->	29	0.00	0.00
76	->	30	-0.00	0.00
76	->	31	0.00	0.00
76	->	32	-0.00	0.00
76	->	33	0.00	0.00
76	->	34	0.00	0.00
76	->	35	-0.01	0.00
76	->	36	0.01	0.00
76	->	37	-0.00	0.00
76	->	38	-0.01	0.00
76	->	39	0.00	0.00
76	->	40	0.01	0.00
76	->	41	-0.00	0.00
76	->	42	0.00	0.00
76	->	43	-0.00	0.00
76	->	44	0.02	0.01
76	->	45	-0.00	0.00
76	->	46	-0.03	0.01
76	->	47	-0.00	0.00
76	->	48	-0.00	0.00
76	->	49	-0.00	0.00
76	->	50	-0.01	0.00
76	->	51	0.00	0.00
76	->	52	-0.00	0.00
76	->	53	0.05	0.02
76	->	54	-0.00	0.00
76	->	55	0.00	0.00
76	->	56	-0.00	0.00
76	->	57	-0.00	0.00
76	->	58	-0.04	0.01
76	->	59	0.04	0.02
76	->	60	-0.03	0.01
76	->	61	-1.41	0.88
76	->	62	-1.92	1.67
76	->	63	-1.51	1.12
76	->	64	-0.98	1.13
76	->	65	-0.03	0.02
76	->	66	-0.09	0.03
76	->	67	-0.01	0.00
76	->	68	-0.07	0.05
76	->	69	0.08	0.05
76	->	70	-2.91	0.37
76	->	71	-0.02	0.02

76	->	72	-0.86	0.45
76	->	73	-2.65	0.76
76	->	74	-1.79	0.32
76	->	75	-7.57	0.56
76	->	76	-102.94	2.82
76	->	77	-12.08	0.88
76	->	78	-0.79	0.22
76	->	79	-2.17	0.40
76	->	80	-3.41	1.38
76	->	81	-0.02	0.06
76	->	82	-0.04	0.02
76	->	83	-0.07	0.02
76	->	84	-0.01	0.01
76	->	85	-0.01	0.01
76	->	86	-0.02	0.01
76	->	87	-0.04	0.01
76	->	88	-0.03	0.01
76	->	89	-0.02	0.01
76	->	90	-0.02	0.00
76	->	91	-0.01	0.00
76	->	92	-0.00	0.01
76	->	93	0.00	0.01
76	->	94	-0.03	0.01
76	->	95	-0.03	0.01
76	->	96	-0.01	0.01
76	->	97	-0.00	0.00
76	->	98	-0.01	0.00
76	->	99	-0.01	0.01
76	->	100	-0.03	0.02
76	->	101	-0.65	0.69
76	->	102	-5.33	5.85
76	->	103	-1.22	1.98
76	->	104	-0.10	0.04
76	->	105	-0.03	0.01
76	->	106	-0.02	0.00
76	->	107	-0.01	0.00
77	->	1	0.03	0.01
77	->	2	0.00	0.00
77	->	3	-0.00	0.00
77	->	4	0.00	0.00
77	->	5	-0.00	0.00
77	->	6	0.00	0.00
77	->	7	0.00	0.00
77	->	8	-0.00	0.00
77	->	9	-0.00	0.00
77	->	10	-0.01	0.00
77	->	11	-0.01	0.00
77	->	12	0.02	0.01
77	->	13	0.00	0.00
77	->	14	0.00	0.00

77	->	15	0.00	0.00
77	->	16	0.02	0.00
77	->	17	-0.00	0.00
77	->	18	0.00	0.00
77	->	19	-0.02	0.00
77	->	20	0.00	0.00
77	->	21	0.00	0.00
77	->	22	0.02	0.00
77	->	23	0.00	0.00
77	->	24	0.00	0.00
77	->	25	0.01	0.00
77	->	26	0.00	0.00
77	->	27	0.00	0.00
77	->	28	0.00	0.00
77	->	29	0.00	0.00
77	->	30	-0.00	0.00
77	->	31	0.00	0.00
77	->	32	0.00	0.00
77	->	33	-0.00	0.00
77	->	34	-0.00	0.00
77	->	35	-0.01	0.00
77	->	36	0.00	0.00
77	->	37	-0.00	0.00
77	->	38	-0.01	0.00
77	->	39	0.00	0.00
77	->	40	0.01	0.00
77	->	41	-0.00	0.00
77	->	42	0.00	0.00
77	->	43	-0.00	0.00
77	->	44	0.03	0.00
77	->	45	-0.00	0.00
77	->	46	-0.03	0.01
77	->	47	-0.00	0.00
77	->	48	-0.00	0.00
77	->	49	0.00	0.00
77	->	50	-0.00	0.00
77	->	51	0.00	0.00
77	->	52	0.00	0.00
77	->	53	0.03	0.01
77	->	54	0.00	0.00
77	->	55	0.00	0.00
77	->	56	0.00	0.00
77	->	57	0.00	0.00
77	->	58	-0.02	0.01
77	->	59	0.01	0.00
77	->	60	-0.01	0.00
77	->	61	-0.03	0.03
77	->	62	-0.01	0.01
77	->	63	-0.03	0.03
77	->	64	-0.04	0.01

77	->	65	-0.01	0.00
77	->	66	-0.03	0.01
77	->	67	-0.00	0.00
77	->	68	0.01	0.01
77	->	69	-0.00	0.01
77	->	70	-0.05	0.02
77	->	71	-0.01	0.00
77	->	72	0.09	0.05
77	->	73	-1.73	1.10
77	->	74	-2.71	0.50
77	->	75	-1.33	0.29
77	->	76	-12.11	0.91
77	->	77	12.57	3.81
77	->	78	-6.53	0.56
77	->	79	-0.88	0.20
77	->	80	-1.93	0.43
77	->	81	-1.63	0.56
77	->	82	-0.06	0.05
77	->	83	-0.03	0.02
77	->	84	-0.03	0.02
77	->	85	-0.01	0.01
77	->	86	-0.02	0.01
77	->	87	-0.04	0.01
77	->	88	-0.01	0.00
77	->	89	-0.03	0.00
77	->	90	-0.02	0.00
77	->	91	-0.01	0.01
77	->	92	-0.01	0.01
77	->	93	-0.03	0.01
77	->	94	-0.04	0.01
77	->	95	-0.03	0.01
77	->	96	-0.01	0.00
77	->	97	-0.00	0.00
77	->	98	-0.01	0.00
77	->	99	-0.01	0.00
77	->	100	-0.02	0.01
77	->	101	-0.06	0.04
77	->	102	-0.81	0.57
77	->	103	-6.62	3.14
77	->	104	-4.09	2.82
77	->	105	-0.10	0.11
77	->	106	-0.04	0.01
77	->	107	-0.01	0.00
78	->	1	0.02	0.00
78	->	2	0.00	0.00
78	->	3	-0.00	0.00
78	->	4	0.00	0.00
78	->	5	-0.01	0.00
78	->	6	0.00	0.00
78	->	7	0.00	0.00

78	->	8	-0.00	0.00
78	->	9	-0.00	0.00
78	->	10	-0.01	0.00
78	->	11	-0.01	0.00
78	->	12	0.02	0.00
78	->	13	0.00	0.00
78	->	14	0.00	0.00
78	->	15	0.00	0.00
78	->	16	0.01	0.00
78	->	17	-0.00	0.00
78	->	18	-0.00	0.00
78	->	19	-0.02	0.00
78	->	20	-0.00	0.00
78	->	21	0.00	0.00
78	->	22	0.02	0.00
78	->	23	0.00	0.00
78	->	24	0.00	0.00
78	->	25	0.01	0.00
78	->	26	0.00	0.00
78	->	27	0.00	0.00
78	->	28	0.00	0.00
78	->	29	0.00	0.00
78	->	30	-0.00	0.00
78	->	31	0.01	0.00
78	->	32	0.00	0.00
78	->	33	-0.00	0.00
78	->	34	0.00	0.00
78	->	35	-0.01	0.00
78	->	36	0.01	0.00
78	->	37	-0.00	0.00
78	->	38	-0.01	0.00
78	->	39	0.00	0.00
78	->	40	0.02	0.00
78	->	41	-0.00	0.00
78	->	42	0.00	0.00
78	->	43	0.00	0.00
78	->	44	0.03	0.00
78	->	45	-0.00	0.00
78	->	46	-0.03	0.00
78	->	47	-0.00	0.00
78	->	48	-0.00	0.00
78	->	49	-0.00	0.00
78	->	50	-0.00	0.00
78	->	51	0.00	0.00
78	->	52	-0.00	0.00
78	->	53	0.03	0.01
78	->	54	-0.00	0.00
78	->	55	0.00	0.00
78	->	56	0.00	0.00
78	->	57	0.00	0.00

78	->	58	-0.02	0.00
78	->	59	0.04	0.01
78	->	60	-0.00	0.00
78	->	61	-0.01	0.00
78	->	62	0.00	0.01
78	->	63	-0.17	0.05
78	->	64	-0.03	0.01
78	->	65	-0.01	0.00
78	->	66	-0.07	0.01
78	->	67	0.00	0.00
78	->	68	0.09	0.04
78	->	69	0.04	0.01
78	->	70	-0.19	0.06
78	->	71	-0.00	0.01
78	->	72	0.51	0.35
78	->	73	-0.09	0.09
78	->	74	-3.52	2.46
78	->	75	-9.54	6.19
78	->	76	-0.79	0.22
78	->	77	-6.57	0.57
78	->	78	-101.83	4.22
78	->	79	-19.50	0.41
78	->	80	-0.76	0.15
78	->	81	-2.21	0.49
78	->	82	-2.19	1.32
78	->	83	-0.13	0.05
78	->	84	-0.03	0.01
78	->	85	-0.02	0.01
78	->	86	-0.52	0.81
78	->	87	-2.81	2.46
78	->	88	0.45	1.04
78	->	89	-0.42	0.50
78	->	90	-0.05	0.04
78	->	91	-0.03	0.01
78	->	92	-0.02	0.00
78	->	93	-0.02	0.00
78	->	94	-0.01	0.00
78	->	95	-0.01	0.00
78	->	96	-0.00	0.00
78	->	97	-0.00	0.00
78	->	98	-0.01	0.00
78	->	99	-0.01	0.00
78	->	100	-0.01	0.00
78	->	101	-0.02	0.00
78	->	102	-0.05	0.01
78	->	103	-0.13	0.06
78	->	104	-0.30	0.16
78	->	105	-1.58	1.27
78	->	106	-5.45	2.94
78	->	107	-0.21	0.32

79	->	1	0.00	0.00
79	->	2	0.00	0.00
79	->	3	0.00	0.00
79	->	4	0.00	0.00
79	->	5	-0.00	0.00
79	->	6	0.00	0.00
79	->	7	0.00	0.00
79	->	8	-0.00	0.00
79	->	9	-0.00	0.00
79	->	10	-0.00	0.00
79	->	11	-0.00	0.00
79	->	12	0.00	0.00
79	->	13	0.00	0.00
79	->	14	0.00	0.00
79	->	15	0.00	0.00
79	->	16	0.00	0.00
79	->	17	-0.00	0.00
79	->	18	-0.00	0.00
79	->	19	-0.00	0.00
79	->	20	-0.00	0.00
79	->	21	0.00	0.00
79	->	22	0.00	0.00
79	->	23	0.00	0.00
79	->	24	0.00	0.00
79	->	25	0.00	0.00
79	->	26	0.00	0.00
79	->	27	0.00	0.00
79	->	28	0.00	0.00
79	->	29	0.00	0.00
79	->	30	-0.00	0.00
79	->	31	0.00	0.00
79	->	32	0.00	0.00
79	->	33	-0.00	0.00
79	->	34	0.00	0.00
79	->	35	-0.00	0.00
79	->	36	0.00	0.00
79	->	37	-0.00	0.00
79	->	38	-0.00	0.00
79	->	39	0.00	0.00
79	->	40	0.00	0.00
79	->	41	-0.00	0.00
79	->	42	0.00	0.00
79	->	43	-0.00	0.00
79	->	44	0.00	0.00
79	->	45	-0.00	0.00
79	->	46	-0.00	0.00
79	->	47	-0.00	0.00
79	->	48	-0.00	0.00
79	->	49	0.00	0.00
79	->	50	-0.00	0.00

79	->	51	0.00	0.00
79	->	52	-0.00	0.00
79	->	53	0.00	0.00
79	->	54	-0.00	0.00
79	->	55	0.00	0.00
79	->	56	0.00	0.00
79	->	57	-0.00	0.00
79	->	58	-0.00	0.00
79	->	59	-0.00	0.00
79	->	60	-0.00	0.00
79	->	61	-0.02	0.01
79	->	62	-0.09	0.02
79	->	63	3.18	1.00
79	->	64	-0.09	0.06
79	->	65	0.67	0.11
79	->	66	3.28	0.47
79	->	67	-0.07	0.03
79	->	68	-1.92	0.69
79	->	69	-0.03	0.02
79	->	70	-1.96	0.39
79	->	71	-0.00	0.00
79	->	72	-0.01	0.01
79	->	73	-0.04	0.03
79	->	74	-0.14	0.07
79	->	75	-3.48	0.63
79	->	76	-2.18	0.40
79	->	77	-0.88	0.20
79	->	78	-19.53	0.41
79	->	79	9.97	1.17
79	->	80	-5.15	0.41
79	->	81	-1.17	0.38
79	->	82	-3.55	0.73
79	->	83	17.40	9.68
79	->	84	-0.10	0.04
79	->	85	-0.03	0.03
79	->	86	-0.22	0.86
79	->	87	-0.04	0.02
79	->	88	-0.01	0.00
79	->	89	-0.01	0.00
79	->	90	-0.00	0.00
79	->	91	-0.00	0.00
79	->	92	-0.00	0.00
79	->	93	-0.00	0.00
79	->	94	-0.00	0.00
79	->	95	-0.00	0.00
79	->	96	-0.00	0.00
79	->	97	-0.00	0.00
79	->	98	-0.00	0.00
79	->	99	-0.00	0.00
79	->	100	-0.00	0.00

79	->	101	-0.00	0.00
79	->	102	-0.01	0.01
79	->	103	-0.02	0.01
79	->	104	-0.01	0.00
79	->	105	-0.01	0.00
79	->	106	-0.01	0.00
79	->	107	-0.00	0.00
80	->	1	0.00	0.00
80	->	2	0.00	0.00
80	->	3	0.00	0.00
80	->	4	0.00	0.00
80	->	5	-0.00	0.00
80	->	6	0.00	0.00
80	->	7	0.00	0.00
80	->	8	-0.00	0.00
80	->	9	0.00	0.00
80	->	10	-0.00	0.00
80	->	11	-0.00	0.00
80	->	12	0.00	0.00
80	->	13	0.00	0.00
80	->	14	0.00	0.00
80	->	15	0.00	0.00
80	->	16	0.00	0.00
80	->	17	0.00	0.00
80	->	18	0.00	0.00
80	->	19	-0.00	0.00
80	->	20	0.00	0.00
80	->	21	0.00	0.00
80	->	22	0.00	0.00
80	->	23	0.00	0.00
80	->	24	0.00	0.00
80	->	25	0.00	0.00
80	->	26	0.00	0.00
80	->	27	0.00	0.00
80	->	28	0.00	0.00
80	->	29	0.00	0.00
80	->	30	0.00	0.00
80	->	31	0.00	0.00
80	->	32	0.00	0.00
80	->	33	-0.00	0.00
80	->	34	0.00	0.00
80	->	35	-0.00	0.00
80	->	36	0.00	0.00
80	->	37	-0.00	0.00
80	->	38	-0.00	0.00
80	->	39	0.00	0.00
80	->	40	0.00	0.00
80	->	41	-0.00	0.00
80	->	42	0.00	0.00
80	->	43	-0.00	0.00

80	->	44	0.00	0.00
80	->	45	-0.00	0.00
80	->	46	-0.00	0.00
80	->	47	0.00	0.00
80	->	48	0.00	0.00
80	->	49	0.00	0.00
80	->	50	-0.00	0.00
80	->	51	0.00	0.00
80	->	52	0.00	0.00
80	->	53	0.00	0.00
80	->	54	0.00	0.00
80	->	55	0.00	0.00
80	->	56	0.00	0.00
80	->	57	-0.00	0.00
80	->	58	-0.00	0.00
80	->	59	-0.00	0.00
80	->	60	-0.00	0.00
80	->	61	-0.01	0.01
80	->	62	-0.04	0.02
80	->	63	-0.13	0.38
80	->	64	-0.34	0.42
80	->	65	-0.10	0.08
80	->	66	0.03	0.01
80	->	67	-0.01	0.00
80	->	68	-0.04	0.02
80	->	69	-0.01	0.00
80	->	70	-0.08	0.06
80	->	71	-0.00	0.00
80	->	72	-0.01	0.01
80	->	73	-0.01	0.02
80	->	74	-0.03	0.02
80	->	75	-0.15	0.05
80	->	76	-3.40	1.38
80	->	77	-1.92	0.44
80	->	78	-0.77	0.15
80	->	79	-5.19	0.42
80	->	80	-23.79	1.65
80	->	81	-15.10	0.59
80	->	82	-0.90	0.18
80	->	83	-2.78	0.82
80	->	84	-1.76	1.00
80	->	85	-0.03	0.04
80	->	86	-0.00	0.01
80	->	87	-0.00	0.00
80	->	88	-0.00	0.00
80	->	89	-0.00	0.00
80	->	90	-0.00	0.00
80	->	91	-0.00	0.00
80	->	92	-0.00	0.00
80	->	93	-0.00	0.00

80	->	94	-0.00	0.00
80	->	95	-0.00	0.00
80	->	96	-0.00	0.00
80	->	97	0.00	0.00
80	->	98	-0.00	0.00
80	->	99	-0.00	0.00
80	->	100	-0.00	0.00
80	->	101	-0.00	0.00
80	->	102	-0.01	0.00
80	->	103	-0.01	0.02
80	->	104	-0.02	0.01
80	->	105	-0.01	0.00
80	->	106	-0.00	0.00
80	->	107	-0.00	0.00
81	->	1	0.02	0.01
81	->	2	0.00	0.00
81	->	3	-0.00	0.00
81	->	4	0.00	0.00
81	->	5	-0.00	0.00
81	->	6	0.00	0.00
81	->	7	0.00	0.00
81	->	8	-0.00	0.00
81	->	9	-0.00	0.00
81	->	10	-0.00	0.00
81	->	11	-0.01	0.00
81	->	12	0.02	0.00
81	->	13	0.00	0.00
81	->	14	0.00	0.00
81	->	15	0.00	0.00
81	->	16	0.01	0.00
81	->	17	-0.00	0.00
81	->	18	0.00	0.00
81	->	19	-0.01	0.00
81	->	20	0.00	0.00
81	->	21	0.00	0.00
81	->	22	0.01	0.00
81	->	23	0.00	0.00
81	->	24	0.00	0.00
81	->	25	0.01	0.00
81	->	26	0.00	0.00
81	->	27	0.00	0.00
81	->	28	0.00	0.00
81	->	29	0.00	0.00
81	->	30	-0.00	0.00
81	->	31	0.00	0.00
81	->	32	0.00	0.00
81	->	33	-0.00	0.00
81	->	34	0.00	0.00
81	->	35	-0.00	0.00
81	->	36	0.00	0.00

81	->	37	-0.00	0.00
81	->	38	-0.01	0.00
81	->	39	0.00	0.00
81	->	40	0.01	0.00
81	->	41	-0.00	0.00
81	->	42	0.00	0.00
81	->	43	0.00	0.00
81	->	44	0.02	0.00
81	->	45	-0.00	0.00
81	->	46	-0.02	0.00
81	->	47	0.00	0.00
81	->	48	-0.00	0.00
81	->	49	0.00	0.00
81	->	50	0.00	0.00
81	->	51	0.00	0.00
81	->	52	0.00	0.00
81	->	53	0.02	0.00
81	->	54	0.00	0.00
81	->	55	0.00	0.00
81	->	56	0.00	0.00
81	->	57	0.00	0.00
81	->	58	-0.01	0.00
81	->	59	0.02	0.00
81	->	60	-0.00	0.00
81	->	61	-0.00	0.00
81	->	62	-0.01	0.00
81	->	63	-0.01	0.02
81	->	64	-0.03	0.01
81	->	65	-0.01	0.00
81	->	66	-0.03	0.01
81	->	67	-0.00	0.00
81	->	68	0.03	0.01
81	->	69	0.01	0.00
81	->	70	-0.01	0.01
81	->	71	0.00	0.00
81	->	72	0.04	0.01
81	->	73	-0.01	0.00
81	->	74	-0.11	0.04
81	->	75	-0.11	0.04
81	->	76	-0.02	0.06
81	->	77	-1.65	0.54
81	->	78	-2.23	0.49
81	->	79	-1.17	0.38
81	->	80	-15.15	0.58
81	->	81	6.80	3.68
81	->	82	-11.15	0.57
81	->	83	-0.65	0.30
81	->	84	-1.63	0.44
81	->	85	-0.70	0.99
81	->	86	-0.02	0.01

81	->	87	-0.04	0.02
81	->	88	-0.01	0.00
81	->	89	-0.02	0.00
81	->	90	-0.02	0.00
81	->	91	-0.01	0.00
81	->	92	-0.01	0.00
81	->	93	-0.02	0.01
81	->	94	-0.02	0.01
81	->	95	-0.01	0.00
81	->	96	-0.01	0.00
81	->	97	-0.00	0.00
81	->	98	-0.00	0.00
81	->	99	-0.01	0.00
81	->	100	-0.01	0.00
81	->	101	-0.02	0.00
81	->	102	-0.05	0.02
81	->	103	-0.34	0.43
81	->	104	-2.00	2.84
81	->	105	-0.52	1.20
81	->	106	-0.06	0.02
81	->	107	-0.01	0.00
82	->	1	0.00	0.00
82	->	2	0.00	0.00
82	->	3	0.00	0.00
82	->	4	0.00	0.00
82	->	5	-0.00	0.00
82	->	6	0.00	0.00
82	->	7	0.00	0.00
82	->	8	-0.00	0.00
82	->	9	0.00	0.00
82	->	10	-0.00	0.00
82	->	11	-0.00	0.00
82	->	12	0.00	0.00
82	->	13	0.00	0.00
82	->	14	0.00	0.00
82	->	15	0.00	0.00
82	->	16	0.00	0.00
82	->	17	0.00	0.00
82	->	18	-0.00	0.00
82	->	19	-0.00	0.00
82	->	20	0.00	0.00
82	->	21	0.00	0.00
82	->	22	0.00	0.00
82	->	23	0.00	0.00
82	->	24	0.00	0.00
82	->	25	0.00	0.00
82	->	26	0.00	0.00
82	->	27	0.00	0.00
82	->	28	0.00	0.00
82	->	29	0.00	0.00

82 ->	30	-0.00	0.00
82 ->	31	0.00	0.00
82 ->	32	0.00	0.00
82 ->	33	0.00	0.00
82 ->	34	-0.00	0.00
82 ->	35	-0.00	0.00
82 ->	36	0.00	0.00
82 ->	37	0.00	0.00
82 ->	38	-0.00	0.00
82 ->	39	0.00	0.00
82 ->	40	0.00	0.00
82 ->	41	-0.00	0.00
82 ->	42	0.00	0.00
82 ->	43	0.00	0.00
82 ->	44	0.00	0.00
82 ->	45	-0.00	0.00
82 ->	46	-0.00	0.00
82 ->	47	0.00	0.00
82 ->	48	-0.00	0.00
82 ->	49	0.00	0.00
82 ->	50	-0.00	0.00
82 ->	51	0.00	0.00
82 ->	52	-0.00	0.00
82 ->	53	0.00	0.00
82 ->	54	0.00	0.00
82 ->	55	0.00	0.00
82 ->	56	-0.00	0.00
82 ->	57	0.00	0.00
82 ->	58	-0.00	0.00
82 ->	59	0.00	0.00
82 ->	60	-0.00	0.00
82 ->	61	-0.00	0.00
82 ->	62	-0.00	0.00
82 ->	63	-0.08	0.03
82 ->	64	-0.01	0.00
82 ->	65	-0.04	0.02
82 ->	66	-0.06	0.03
82 ->	67	-0.00	0.00
82 ->	68	-0.04	0.05
82 ->	69	-0.00	0.00
82 ->	70	-0.03	0.01
82 ->	71	-0.00	0.00
82 ->	72	-0.00	0.00
82 ->	73	-0.00	0.00
82 ->	74	-0.01	0.00
82 ->	75	-0.06	0.03
82 ->	76	-0.04	0.02
82 ->	77	-0.06	0.05
82 ->	78	-2.19	1.30
82 ->	79	-3.57	0.73

82	->	80	-0.90	0.17
82	->	81	-11.22	0.58
82	->	82	18.40	2.30
82	->	83	-21.94	0.59
82	->	84	-0.78	0.23
82	->	85	-1.38	1.77
82	->	86	-0.09	0.24
82	->	87	-0.02	0.02
82	->	88	-0.01	0.00
82	->	89	-0.00	0.00
82	->	90	-0.00	0.00
82	->	91	-0.00	0.00
82	->	92	-0.00	0.00
82	->	93	-0.00	0.00
82	->	94	-0.00	0.00
82	->	95	-0.00	0.00
82	->	96	-0.00	0.00
82	->	97	-0.00	0.00
82	->	98	-0.00	0.00
82	->	99	-0.00	0.00
82	->	100	-0.00	0.00
82	->	101	-0.00	0.00
82	->	102	-0.00	0.00
82	->	103	-0.00	0.00
82	->	104	-0.01	0.01
82	->	105	-0.01	0.01
82	->	106	-0.01	0.01
82	->	107	-0.01	0.01
83	->	1	0.00	0.00
83	->	2	0.00	0.00
83	->	3	0.00	0.00
83	->	4	0.00	0.00
83	->	5	-0.00	0.00
83	->	6	0.00	0.00
83	->	7	0.00	0.00
83	->	8	0.00	0.00
83	->	9	0.00	0.00
83	->	10	-0.00	0.00
83	->	11	-0.00	0.00
83	->	12	0.00	0.00
83	->	13	0.00	0.00
83	->	14	0.00	0.00
83	->	15	0.00	0.00
83	->	16	0.00	0.00
83	->	17	0.00	0.00
83	->	18	0.00	0.00
83	->	19	-0.00	0.00
83	->	20	0.00	0.00
83	->	21	0.00	0.00
83	->	22	0.00	0.00

83	->	23	0.00	0.00
83	->	24	0.00	0.00
83	->	25	0.00	0.00
83	->	26	0.00	0.00
83	->	27	0.00	0.00
83	->	28	0.00	0.00
83	->	29	0.00	0.00
83	->	30	0.00	0.00
83	->	31	0.00	0.00
83	->	32	0.00	0.00
83	->	33	0.00	0.00
83	->	34	0.00	0.00
83	->	35	0.00	0.00
83	->	36	-0.00	0.00
83	->	37	0.00	0.00
83	->	38	0.00	0.00
83	->	39	0.00	0.00
83	->	40	0.00	0.00
83	->	41	0.00	0.00
83	->	42	0.00	0.00
83	->	43	0.00	0.00
83	->	44	0.00	0.00
83	->	45	0.00	0.00
83	->	46	-0.00	0.00
83	->	47	0.00	0.00
83	->	48	0.00	0.00
83	->	49	0.00	0.00
83	->	50	0.00	0.00
83	->	51	0.00	0.00
83	->	52	0.00	0.00
83	->	53	-0.00	0.00
83	->	54	0.00	0.00
83	->	55	0.00	0.00
83	->	56	0.00	0.00
83	->	57	0.00	0.00
83	->	58	0.00	0.00
83	->	59	-0.00	0.00
83	->	60	-0.00	0.00
83	->	61	-0.01	0.00
83	->	62	-0.04	0.01
83	->	63	2.37	0.48
83	->	64	-0.21	0.12
83	->	65	0.84	0.60
83	->	66	2.01	0.34
83	->	67	-0.02	0.02
83	->	68	-0.23	0.06
83	->	69	-0.01	0.01
83	->	70	-0.01	0.02
83	->	71	0.00	0.00
83	->	72	-0.00	0.00

83	->	73	-0.00	0.00
83	->	74	-0.01	0.00
83	->	75	-0.02	0.00
83	->	76	-0.07	0.02
83	->	77	-0.03	0.02
83	->	78	-0.13	0.05
83	->	79	17.38	9.68
83	->	80	-2.79	0.82
83	->	81	-0.66	0.29
83	->	82	-22.03	0.59
83	->	83	4.32	1.64
83	->	84	-8.34	0.58
83	->	85	-1.06	1.45
83	->	86	-0.01	0.00
83	->	87	-0.00	0.00
83	->	88	-0.00	0.00
83	->	89	-0.00	0.00
83	->	90	-0.00	0.00
83	->	91	-0.00	0.00
83	->	92	-0.00	0.00
83	->	93	-0.00	0.00
83	->	94	-0.00	0.00
83	->	95	-0.00	0.00
83	->	96	-0.00	0.00
83	->	97	0.00	0.00
83	->	98	-0.00	0.00
83	->	99	-0.00	0.00
83	->	100	0.00	0.00
83	->	101	-0.00	0.00
83	->	102	-0.00	0.00
83	->	103	-0.01	0.00
83	->	104	-0.01	0.00
83	->	105	-0.00	0.00
83	->	106	-0.00	0.00
83	->	107	-0.00	0.00
84	->	1	0.00	0.00
84	->	2	0.00	0.00
84	->	3	0.00	0.00
84	->	4	0.00	0.00
84	->	5	-0.00	0.00
84	->	6	0.00	0.00
84	->	7	0.00	0.00
84	->	8	-0.00	0.00
84	->	9	0.00	0.00
84	->	10	-0.00	0.00
84	->	11	-0.00	0.00
84	->	12	0.00	0.00
84	->	13	0.00	0.00
84	->	14	0.00	0.00
84	->	15	0.00	0.00

84	->	16	0.00	0.00
84	->	17	0.00	0.00
84	->	18	0.00	0.00
84	->	19	-0.00	0.00
84	->	20	0.00	0.00
84	->	21	0.00	0.00
84	->	22	0.00	0.00
84	->	23	0.00	0.00
84	->	24	0.00	0.00
84	->	25	0.00	0.00
84	->	26	0.00	0.00
84	->	27	0.00	0.00
84	->	28	0.00	0.00
84	->	29	0.00	0.00
84	->	30	0.00	0.00
84	->	31	0.00	0.00
84	->	32	0.00	0.00
84	->	33	0.00	0.00
84	->	34	0.00	0.00
84	->	35	-0.00	0.00
84	->	36	0.00	0.00
84	->	37	0.00	0.00
84	->	38	-0.00	0.00
84	->	39	0.00	0.00
84	->	40	0.00	0.00
84	->	41	0.00	0.00
84	->	42	0.00	0.00
84	->	43	0.00	0.00
84	->	44	0.00	0.00
84	->	45	-0.00	0.00
84	->	46	-0.00	0.00
84	->	47	0.00	0.00
84	->	48	0.00	0.00
84	->	49	0.00	0.00
84	->	50	-0.00	0.00
84	->	51	0.00	0.00
84	->	52	0.00	0.00
84	->	53	0.00	0.00
84	->	54	0.00	0.00
84	->	55	0.00	0.00
84	->	56	-0.00	0.00
84	->	57	0.00	0.00
84	->	58	-0.00	0.00
84	->	59	0.00	0.00
84	->	60	0.00	0.00
84	->	61	-0.00	0.00
84	->	62	-0.00	0.00
84	->	63	-0.00	0.01
84	->	64	-0.03	0.03
84	->	65	-0.03	0.02

84 ->	66	-0.00	0.00
84 ->	67	-0.00	0.00
84 ->	68	-0.00	0.00
84 ->	69	-0.00	0.00
84 ->	70	-0.00	0.00
84 ->	71	0.00	0.00
84 ->	72	-0.00	0.00
84 ->	73	-0.00	0.00
84 ->	74	-0.00	0.00
84 ->	75	-0.00	0.00
84 ->	76	-0.01	0.01
84 ->	77	-0.03	0.02
84 ->	78	-0.03	0.01
84 ->	79	-0.10	0.04
84 ->	80	-1.79	0.99
84 ->	81	-1.66	0.43
84 ->	82	-0.79	0.22
84 ->	83	-8.47	0.57
84 ->	84	16.97	1.98
84 ->	85	-8.26	0.81
84 ->	86	-0.00	0.00
84 ->	87	-0.00	0.00
84 ->	88	-0.00	0.00
84 ->	89	-0.00	0.00
84 ->	90	-0.00	0.00
84 ->	91	-0.00	0.00
84 ->	92	-0.00	0.00
84 ->	93	-0.00	0.00
84 ->	94	-0.00	0.00
84 ->	95	-0.00	0.00
84 ->	96	-0.00	0.00
84 ->	97	0.00	0.00
84 ->	98	-0.00	0.00
84 ->	99	-0.00	0.00
84 ->	100	-0.00	0.00
84 ->	101	-0.00	0.00
84 ->	102	-0.00	0.00
84 ->	103	-0.01	0.00
84 ->	104	-0.01	0.00
84 ->	105	-0.00	0.00
84 ->	106	-0.00	0.00
84 ->	107	-0.00	0.00
85 ->	1	0.00	0.00
85 ->	2	0.00	0.00
85 ->	3	0.00	0.00
85 ->	4	0.00	0.00
85 ->	5	-0.00	0.00
85 ->	6	0.00	0.00
85 ->	7	0.00	0.00
85 ->	8	-0.00	0.00

85	->	9	0.00	0.00
85	->	10	-0.00	0.00
85	->	11	-0.00	0.00
85	->	12	0.00	0.00
85	->	13	0.00	0.00
85	->	14	0.00	0.00
85	->	15	0.00	0.00
85	->	16	0.00	0.00
85	->	17	0.00	0.00
85	->	18	0.00	0.00
85	->	19	-0.00	0.00
85	->	20	0.00	0.00
85	->	21	0.00	0.00
85	->	22	0.00	0.00
85	->	23	0.00	0.00
85	->	24	0.00	0.00
85	->	25	0.00	0.00
85	->	26	0.00	0.00
85	->	27	-0.00	0.00
85	->	28	0.00	0.00
85	->	29	0.00	0.00
85	->	30	-0.00	0.00
85	->	31	0.00	0.00
85	->	32	0.00	0.00
85	->	33	-0.00	0.00
85	->	34	-0.00	0.00
85	->	35	-0.00	0.00
85	->	36	0.00	0.00
85	->	37	-0.00	0.00
85	->	38	-0.00	0.00
85	->	39	0.00	0.00
85	->	40	0.00	0.00
85	->	41	-0.00	0.00
85	->	42	0.00	0.00
85	->	43	0.00	0.00
85	->	44	0.00	0.00
85	->	45	0.00	0.00
85	->	46	-0.00	0.00
85	->	47	-0.00	0.00
85	->	48	0.00	0.00
85	->	49	0.00	0.00
85	->	50	-0.00	0.00
85	->	51	0.00	0.00
85	->	52	0.00	0.00
85	->	53	0.00	0.00
85	->	54	0.00	0.00
85	->	55	0.00	0.00
85	->	56	0.00	0.00
85	->	57	-0.00	0.00
85	->	58	-0.00	0.00

85	->	59	0.00	0.00
85	->	60	0.00	0.00
85	->	61	-0.00	0.00
85	->	62	-0.00	0.00
85	->	63	-0.00	0.03
85	->	64	-0.01	0.08
85	->	65	-0.07	0.30
85	->	66	-0.01	0.03
85	->	67	-0.00	0.00
85	->	68	0.00	0.01
85	->	69	0.00	0.00
85	->	70	-0.00	0.00
85	->	71	0.00	0.00
85	->	72	0.00	0.01
85	->	73	-0.00	0.00
85	->	74	-0.00	0.01
85	->	75	-0.01	0.02
85	->	76	-0.01	0.01
85	->	77	-0.01	0.01
85	->	78	-0.02	0.01
85	->	79	-0.03	0.03
85	->	80	-0.03	0.04
85	->	81	-0.71	0.99
85	->	82	-1.38	1.76
85	->	83	-1.08	1.44
85	->	84	-8.31	0.80
85	->	85	-268.20	2.73
85	->	86	-0.01	0.01
85	->	87	-0.00	0.01
85	->	88	-0.00	0.00
85	->	89	-0.00	0.00
85	->	90	-0.00	0.00
85	->	91	-0.00	0.00
85	->	92	-0.00	0.00
85	->	93	-0.00	0.00
85	->	94	-0.00	0.00
85	->	95	-0.00	0.00
85	->	96	0.00	0.00
85	->	97	0.00	0.00
85	->	98	-0.00	0.00
85	->	99	-0.00	0.00
85	->	100	-0.00	0.00
85	->	101	-0.00	0.00
85	->	102	-0.00	0.01
85	->	103	-0.00	0.02
85	->	104	-0.01	0.04
85	->	105	-0.02	0.04
85	->	106	-0.01	0.02
85	->	107	-0.00	0.00
86	->	1	-0.00	0.00

86	->	2	0.00	0.00
86	->	3	0.00	0.00
86	->	4	0.00	0.00
86	->	5	0.00	0.00
86	->	6	-0.00	0.00
86	->	7	0.00	0.00
86	->	8	0.00	0.00
86	->	9	0.00	0.00
86	->	10	0.00	0.00
86	->	11	0.00	0.00
86	->	12	-0.00	0.00
86	->	13	-0.00	0.00
86	->	14	-0.00	0.00
86	->	15	-0.00	0.00
86	->	16	-0.00	0.00
86	->	17	0.00	0.00
86	->	18	0.00	0.00
86	->	19	0.00	0.00
86	->	20	0.00	0.00
86	->	21	-0.00	0.00
86	->	22	-0.00	0.00
86	->	23	-0.00	0.00
86	->	24	0.00	0.00
86	->	25	-0.00	0.00
86	->	26	-0.00	0.00
86	->	27	0.00	0.00
86	->	28	0.00	0.00
86	->	29	-0.00	0.00
86	->	30	0.00	0.00
86	->	31	-0.00	0.00
86	->	32	0.00	0.00
86	->	33	0.00	0.00
86	->	34	-0.00	0.00
86	->	35	0.00	0.00
86	->	36	-0.00	0.00
86	->	37	-0.00	0.00
86	->	38	0.00	0.00
86	->	39	-0.00	0.00
86	->	40	-0.01	0.00
86	->	41	0.00	0.00
86	->	42	-0.00	0.00
86	->	43	-0.00	0.00
86	->	44	-0.00	0.00
86	->	45	0.00	0.00
86	->	46	0.00	0.00
86	->	47	-0.00	0.00
86	->	48	-0.00	0.00
86	->	49	-0.00	0.00
86	->	50	-0.00	0.00
86	->	51	-0.00	0.00

86	->	52	-0.00	0.00
86	->	53	-0.01	0.01
86	->	54	-0.01	0.00
86	->	55	-0.00	0.00
86	->	56	-0.00	0.00
86	->	57	-0.00	0.00
86	->	58	0.00	0.00
86	->	59	-0.15	0.27
86	->	60	-0.00	0.00
86	->	61	-0.00	0.00
86	->	62	-0.00	0.00
86	->	63	0.01	0.01
86	->	64	0.00	0.00
86	->	65	-0.00	0.00
86	->	66	0.01	0.01
86	->	67	-0.00	0.01
86	->	68	-1.78	1.64
86	->	69	-0.04	0.05
86	->	70	-0.09	0.20
86	->	71	-0.00	0.00
86	->	72	-0.04	0.01
86	->	73	-0.00	0.00
86	->	74	0.00	0.01
86	->	75	-0.08	0.11
86	->	76	-0.02	0.01
86	->	77	-0.02	0.01
86	->	78	-0.51	0.79
86	->	79	-0.22	0.85
86	->	80	-0.00	0.01
86	->	81	-0.02	0.01
86	->	82	-0.09	0.23
86	->	83	-0.01	0.00
86	->	84	-0.00	0.00
86	->	85	-0.01	0.01
86	->	86	-86.88	2.60
86	->	87	-35.59	1.14
86	->	88	-0.29	0.06
86	->	89	-0.03	0.01
86	->	90	-0.00	0.00
86	->	91	0.00	0.00
86	->	92	0.00	0.00
86	->	93	0.00	0.00
86	->	94	0.00	0.00
86	->	95	0.00	0.00
86	->	96	0.00	0.00
86	->	97	0.00	0.00
86	->	98	0.00	0.00
86	->	99	0.00	0.00
86	->	100	0.00	0.00
86	->	101	0.00	0.00

86	->	102	0.00	0.00
86	->	103	0.00	0.00
86	->	104	-0.00	0.00
86	->	105	-0.02	0.01
86	->	106	-0.18	0.06
86	->	107	-2.05	1.12
87	->	1	-0.01	0.00
87	->	2	-0.00	0.00
87	->	3	0.00	0.00
87	->	4	0.00	0.00
87	->	5	0.00	0.00
87	->	6	-0.00	0.00
87	->	7	0.00	0.00
87	->	8	0.00	0.00
87	->	9	0.00	0.00
87	->	10	0.00	0.00
87	->	11	0.00	0.00
87	->	12	-0.01	0.00
87	->	13	-0.00	0.00
87	->	14	-0.00	0.00
87	->	15	-0.00	0.00
87	->	16	-0.01	0.00
87	->	17	0.00	0.00
87	->	18	0.00	0.00
87	->	19	0.01	0.00
87	->	20	0.00	0.00
87	->	21	-0.00	0.00
87	->	22	-0.01	0.00
87	->	23	-0.00	0.00
87	->	24	-0.00	0.00
87	->	25	-0.01	0.00
87	->	26	-0.00	0.00
87	->	27	-0.00	0.00
87	->	28	-0.00	0.00
87	->	29	-0.00	0.00
87	->	30	0.00	0.00
87	->	31	-0.01	0.00
87	->	32	-0.00	0.00
87	->	33	-0.00	0.00
87	->	34	-0.00	0.00
87	->	35	0.02	0.00
87	->	36	-0.01	0.00
87	->	37	-0.00	0.00
87	->	38	0.02	0.00
87	->	39	-0.00	0.00
87	->	40	-0.04	0.01
87	->	41	-0.00	0.00
87	->	42	-0.00	0.00
87	->	43	-0.00	0.00
87	->	44	-0.02	0.00

87 ->	45	0.00	0.00
87 ->	46	0.02	0.00
87 ->	47	-0.01	0.00
87 ->	48	-0.00	0.00
87 ->	49	-0.00	0.01
87 ->	50	-0.02	0.01
87 ->	51	-0.02	0.01
87 ->	52	-0.00	0.00
87 ->	53	-0.13	0.08
87 ->	54	-0.20	0.13
87 ->	55	0.01	0.01
87 ->	56	-0.01	0.01
87 ->	57	-0.02	0.02
87 ->	58	0.03	0.01
87 ->	59	-1.27	1.76
87 ->	60	-0.01	0.00
87 ->	61	-0.00	0.00
87 ->	62	-0.01	0.01
87 ->	63	0.04	0.01
87 ->	64	0.02	0.00
87 ->	65	0.00	0.00
87 ->	66	0.05	0.02
87 ->	67	-0.00	0.00
87 ->	68	-2.72	2.50
87 ->	69	-0.12	0.10
87 ->	70	-0.16	0.24
87 ->	71	-0.09	0.19
87 ->	72	-0.27	0.39
87 ->	73	-0.00	0.01
87 ->	74	0.02	0.02
87 ->	75	-0.09	0.62
87 ->	76	-0.04	0.01
87 ->	77	-0.04	0.01
87 ->	78	-2.79	2.46
87 ->	79	-0.04	0.02
87 ->	80	-0.00	0.00
87 ->	81	-0.04	0.02
87 ->	82	-0.02	0.02
87 ->	83	-0.00	0.00
87 ->	84	-0.00	0.00
87 ->	85	-0.00	0.01
87 ->	86	-35.40	1.14
87 ->	87	-177.27	4.07
87 ->	88	-36.15	0.95
87 ->	89	-0.34	0.09
87 ->	90	-0.02	0.01
87 ->	91	0.01	0.00
87 ->	92	0.01	0.00
87 ->	93	0.00	0.00
87 ->	94	0.00	0.00

87	->	95	0.00	0.00
87	->	96	0.00	0.00
87	->	97	0.00	0.00
87	->	98	0.00	0.00
87	->	99	0.01	0.00
87	->	100	0.01	0.00
87	->	101	0.01	0.00
87	->	102	0.01	0.00
87	->	103	0.01	0.00
87	->	104	-0.00	0.01
87	->	105	-0.08	0.05
87	->	106	-1.03	0.59
87	->	107	-10.50	0.68
88	->	1	-0.01	0.00
88	->	2	-0.00	0.00
88	->	3	0.00	0.00
88	->	4	-0.00	0.00
88	->	5	0.01	0.00
88	->	6	-0.00	0.00
88	->	7	0.00	0.00
88	->	8	0.00	0.00
88	->	9	0.00	0.00
88	->	10	0.01	0.00
88	->	11	0.01	0.00
88	->	12	-0.01	0.00
88	->	13	-0.00	0.00
88	->	14	-0.00	0.00
88	->	15	-0.00	0.00
88	->	16	-0.01	0.00
88	->	17	0.00	0.00
88	->	18	0.00	0.00
88	->	19	0.01	0.00
88	->	20	0.00	0.00
88	->	21	0.00	0.00
88	->	22	-0.02	0.00
88	->	23	-0.00	0.00
88	->	24	0.00	0.00
88	->	25	-0.01	0.00
88	->	26	-0.00	0.00
88	->	27	-0.00	0.00
88	->	28	-0.00	0.00
88	->	29	-0.00	0.00
88	->	30	0.00	0.00
88	->	31	-0.02	0.00
88	->	32	-0.00	0.00
88	->	33	-0.00	0.00
88	->	34	-0.00	0.00
88	->	35	0.06	0.02
88	->	36	-0.03	0.01
88	->	37	-0.00	0.00

88	->	38	0.12	0.04
88	->	39	-0.00	0.00
88	->	40	-1.08	0.53
88	->	41	-0.00	0.00
88	->	42	-0.01	0.01
88	->	43	-0.00	0.00
88	->	44	-0.03	0.01
88	->	45	0.00	0.00
88	->	46	0.02	0.00
88	->	47	-0.05	0.01
88	->	48	-0.00	0.01
88	->	49	-0.02	0.01
88	->	50	-0.13	0.08
88	->	51	-0.52	0.39
88	->	52	0.00	0.02
88	->	53	-0.39	0.27
88	->	54	-2.57	1.06
88	->	55	0.03	0.06
88	->	56	-0.03	0.01
88	->	57	-0.07	0.06
88	->	58	0.04	0.01
88	->	59	-0.73	1.75
88	->	60	-0.01	0.00
88	->	61	-0.01	0.00
88	->	62	-0.00	0.00
88	->	63	0.01	0.00
88	->	64	0.01	0.00
88	->	65	0.00	0.00
88	->	66	0.02	0.00
88	->	67	-0.00	0.00
88	->	68	-0.06	0.02
88	->	69	-0.04	0.02
88	->	70	-0.08	0.07
88	->	71	-0.14	0.21
88	->	72	-2.15	0.81
88	->	73	-0.00	0.01
88	->	74	-0.48	0.79
88	->	75	-2.97	3.05
88	->	76	-0.03	0.01
88	->	77	-0.01	0.00
88	->	78	0.45	1.03
88	->	79	-0.01	0.00
88	->	80	-0.00	0.00
88	->	81	-0.01	0.00
88	->	82	-0.01	0.00
88	->	83	-0.00	0.00
88	->	84	-0.00	0.00
88	->	85	-0.00	0.00
88	->	86	-0.29	0.06
88	->	87	-35.97	0.94

88	->	88	-210.22	4.86
88	->	89	-31.97	1.13
88	->	90	-0.23	0.06
88	->	91	0.01	0.01
88	->	92	0.01	0.00
88	->	93	0.01	0.00
88	->	94	0.00	0.00
88	->	95	0.00	0.00
88	->	96	0.00	0.00
88	->	97	0.00	0.00
88	->	98	0.01	0.00
88	->	99	0.01	0.00
88	->	100	0.01	0.00
88	->	101	0.01	0.00
88	->	102	0.00	0.00
88	->	103	-0.01	0.01
88	->	104	-0.06	0.04
88	->	105	-0.29	0.21
88	->	106	-11.27	1.26
88	->	107	0.09	0.13
89	->	1	-0.01	0.00
89	->	2	-0.00	0.00
89	->	3	0.00	0.00
89	->	4	-0.00	0.00
89	->	5	0.01	0.00
89	->	6	-0.00	0.00
89	->	7	0.00	0.00
89	->	8	0.00	0.00
89	->	9	-0.00	0.00
89	->	10	0.01	0.00
89	->	11	0.01	0.00
89	->	12	-0.01	0.00
89	->	13	-0.00	0.00
89	->	14	-0.00	0.00
89	->	15	-0.00	0.00
89	->	16	-0.02	0.00
89	->	17	0.00	0.00
89	->	18	0.00	0.00
89	->	19	0.02	0.00
89	->	20	-0.00	0.00
89	->	21	-0.00	0.00
89	->	22	-0.04	0.00
89	->	23	-0.00	0.00
89	->	24	-0.00	0.00
89	->	25	-0.03	0.00
89	->	26	-0.01	0.00
89	->	27	-0.00	0.00
89	->	28	-0.00	0.00
89	->	29	-0.00	0.00
89	->	30	0.00	0.00

89	->	31	-0.03	0.01
89	->	32	-0.00	0.00
89	->	33	-0.00	0.00
89	->	34	-0.01	0.01
89	->	35	0.13	0.06
89	->	36	-0.03	0.01
89	->	37	0.01	0.01
89	->	38	0.33	0.21
89	->	39	-0.01	0.01
89	->	40	-8.43	3.49
89	->	41	-0.03	0.01
89	->	42	-0.17	0.25
89	->	43	-0.00	0.00
89	->	44	-0.08	0.01
89	->	45	-0.00	0.00
89	->	46	0.03	0.01
89	->	47	-0.23	0.07
89	->	48	0.01	0.02
89	->	49	-0.03	0.02
89	->	50	-0.31	0.21
89	->	51	-3.75	3.05
89	->	52	0.07	0.06
89	->	53	-0.14	0.09
89	->	54	-0.40	0.85
89	->	55	0.08	0.09
89	->	56	-0.01	0.00
89	->	57	-0.01	0.00
89	->	58	0.03	0.01
89	->	59	-0.05	0.02
89	->	60	-0.00	0.00
89	->	61	-0.00	0.00
89	->	62	-0.00	0.00
89	->	63	0.01	0.00
89	->	64	0.01	0.00
89	->	65	0.00	0.00
89	->	66	0.01	0.00
89	->	67	0.00	0.00
89	->	68	-0.03	0.01
89	->	69	-0.02	0.00
89	->	70	-0.02	0.01
89	->	71	-0.03	0.01
89	->	72	-8.58	1.06
89	->	73	-0.01	0.01
89	->	74	0.46	0.19
89	->	75	0.16	0.26
89	->	76	-0.02	0.01
89	->	77	-0.03	0.00
89	->	78	-0.42	0.49
89	->	79	-0.01	0.00
89	->	80	-0.00	0.00

89	->	81	-0.02	0.00
89	->	82	-0.00	0.00
89	->	83	-0.00	0.00
89	->	84	-0.00	0.00
89	->	85	-0.00	0.00
89	->	86	-0.03	0.01
89	->	87	-0.34	0.09
89	->	88	-31.80	1.12
89	->	89	-165.25	5.68
89	->	90	-35.49	0.96
89	->	91	-0.28	0.07
89	->	92	-0.01	0.01
89	->	93	0.01	0.00
89	->	94	0.01	0.00
89	->	95	0.01	0.00
89	->	96	0.00	0.00
89	->	97	0.00	0.00
89	->	98	0.01	0.00
89	->	99	0.02	0.00
89	->	100	0.02	0.00
89	->	101	0.01	0.00
89	->	102	-0.00	0.00
89	->	103	-0.07	0.03
89	->	104	-1.44	0.58
89	->	105	-11.61	0.63
89	->	106	-5.08	1.03
89	->	107	-0.26	0.06
90	->	1	-0.03	0.01
90	->	2	-0.00	0.00
90	->	3	-0.00	0.00
90	->	4	-0.00	0.00
90	->	5	0.02	0.00
90	->	6	-0.00	0.00
90	->	7	-0.00	0.00
90	->	8	0.01	0.00
90	->	9	-0.00	0.00
90	->	10	0.03	0.00
90	->	11	0.02	0.01
90	->	12	-0.06	0.04
90	->	13	-0.01	0.00
90	->	14	-0.01	0.00
90	->	15	-0.00	0.00
90	->	16	-0.03	0.00
90	->	17	0.00	0.00
90	->	18	0.00	0.00
90	->	19	0.03	0.00
90	->	20	-0.00	0.00
90	->	21	-0.00	0.00
90	->	22	-0.09	0.01
90	->	23	-0.02	0.00

90 ->	24	-0.00	0.00
90 ->	25	-0.05	0.01
90 ->	26	-0.05	0.04
90 ->	27	-0.01	0.00
90 ->	28	-0.00	0.00
90 ->	29	-0.00	0.00
90 ->	30	0.00	0.00
90 ->	31	-0.05	0.03
90 ->	32	-0.01	0.00
90 ->	33	-0.00	0.00
90 ->	34	-0.01	0.01
90 ->	35	0.04	0.01
90 ->	36	-0.02	0.00
90 ->	37	0.00	0.00
90 ->	38	0.05	0.01
90 ->	39	-0.00	0.00
90 ->	40	-1.38	1.94
90 ->	41	-0.04	0.02
90 ->	42	-0.24	0.18
90 ->	43	-0.02	0.01
90 ->	44	-0.19	0.05
90 ->	45	-0.00	0.00
90 ->	46	-0.02	0.03
90 ->	47	-2.25	0.68
90 ->	48	-0.01	0.01
90 ->	49	-0.02	0.01
90 ->	50	-0.33	0.29
90 ->	51	-0.07	0.03
90 ->	52	-0.01	0.00
90 ->	53	-0.04	0.01
90 ->	54	-0.02	0.01
90 ->	55	-0.00	0.00
90 ->	56	-0.00	0.00
90 ->	57	-0.00	0.00
90 ->	58	0.01	0.00
90 ->	59	-0.02	0.00
90 ->	60	-0.00	0.00
90 ->	61	-0.00	0.00
90 ->	62	0.00	0.00
90 ->	63	0.01	0.00
90 ->	64	0.00	0.00
90 ->	65	0.00	0.00
90 ->	66	0.01	0.00
90 ->	67	0.00	0.00
90 ->	68	-0.01	0.00
90 ->	69	-0.01	0.00
90 ->	70	-0.01	0.00
90 ->	71	-0.01	0.00
90 ->	72	-2.23	0.95
90 ->	73	-0.01	0.00

90	->	74	0.12	0.10
90	->	75	0.00	0.02
90	->	76	-0.02	0.00
90	->	77	-0.02	0.00
90	->	78	-0.05	0.04
90	->	79	-0.00	0.00
90	->	80	-0.00	0.00
90	->	81	-0.02	0.00
90	->	82	-0.00	0.00
90	->	83	-0.00	0.00
90	->	84	-0.00	0.00
90	->	85	-0.00	0.00
90	->	86	-0.00	0.00
90	->	87	-0.02	0.01
90	->	88	-0.23	0.06
90	->	89	-35.25	0.96
90	->	90	-93.69	2.93
90	->	91	-33.21	0.76
90	->	92	-0.18	0.05
90	->	93	0.00	0.01
90	->	94	0.01	0.00
90	->	95	0.01	0.00
90	->	96	0.00	0.00
90	->	97	0.00	0.00
90	->	98	0.01	0.00
90	->	99	0.02	0.00
90	->	100	0.01	0.00
90	->	101	-0.00	0.00
90	->	102	-0.05	0.02
90	->	103	-0.57	0.33
90	->	104	-5.98	0.66
90	->	105	-1.03	0.53
90	->	106	-0.30	0.11
90	->	107	-0.03	0.01
91	->	1	-0.18	0.11
91	->	2	-0.00	0.00
91	->	3	-0.00	0.00
91	->	4	-0.01	0.00
91	->	5	0.05	0.01
91	->	6	-0.00	0.00
91	->	7	-0.00	0.00
91	->	8	0.02	0.00
91	->	9	-0.00	0.00
91	->	10	0.07	0.02
91	->	11	0.03	0.03
91	->	12	-1.67	1.73
91	->	13	-0.05	0.01
91	->	14	-0.07	0.04
91	->	15	0.00	0.01
91	->	16	-0.07	0.02

91	->	17	-0.00	0.00
91	->	18	-0.02	0.01
91	->	19	-0.03	0.06
91	->	20	0.01	0.01
91	->	21	-0.02	0.01
91	->	22	-0.35	0.11
91	->	23	-0.18	0.17
91	->	24	0.00	0.00
91	->	25	-0.08	0.03
91	->	26	-0.28	0.43
91	->	27	-0.01	0.01
91	->	28	-0.01	0.00
91	->	29	-0.01	0.00
91	->	30	0.00	0.00
91	->	31	-0.03	0.01
91	->	32	-0.01	0.00
91	->	33	-0.00	0.00
91	->	34	-0.00	0.00
91	->	35	0.02	0.00
91	->	36	-0.01	0.00
91	->	37	0.00	0.00
91	->	38	0.03	0.00
91	->	39	-0.00	0.00
91	->	40	-0.07	0.02
91	->	41	-0.01	0.01
91	->	42	-0.05	0.02
91	->	43	-0.04	0.02
91	->	44	-1.97	0.61
91	->	45	-0.00	0.00
91	->	46	0.08	0.07
91	->	47	-3.17	0.72
91	->	48	-0.00	0.00
91	->	49	-0.01	0.00
91	->	50	-0.03	0.01
91	->	51	-0.01	0.00
91	->	52	-0.00	0.00
91	->	53	-0.03	0.00
91	->	54	-0.01	0.00
91	->	55	-0.00	0.00
91	->	56	-0.00	0.00
91	->	57	-0.00	0.00
91	->	58	0.01	0.00
91	->	59	-0.01	0.00
91	->	60	-0.00	0.00
91	->	61	-0.00	0.00
91	->	62	0.00	0.00
91	->	63	0.01	0.00
91	->	64	0.00	0.00
91	->	65	0.00	0.00
91	->	66	0.00	0.00

91	->	67	0.00	0.00
91	->	68	-0.01	0.00
91	->	69	-0.01	0.00
91	->	70	-0.00	0.00
91	->	71	-0.00	0.00
91	->	72	-0.12	0.18
91	->	73	0.00	0.01
91	->	74	-0.09	0.07
91	->	75	0.00	0.02
91	->	76	-0.01	0.00
91	->	77	-0.01	0.01
91	->	78	-0.03	0.01
91	->	79	-0.00	0.00
91	->	80	-0.00	0.00
91	->	81	-0.01	0.00
91	->	82	-0.00	0.00
91	->	83	-0.00	0.00
91	->	84	-0.00	0.00
91	->	85	-0.00	0.00
91	->	86	0.00	0.00
91	->	87	0.01	0.00
91	->	88	0.01	0.01
91	->	89	-0.28	0.07
91	->	90	-32.94	0.75
91	->	91	-175.34	3.27
91	->	92	-34.33	0.97
91	->	93	-0.34	0.08
91	->	94	-0.01	0.01
91	->	95	0.00	0.00
91	->	96	0.00	0.00
91	->	97	0.00	0.00
91	->	98	0.02	0.00
91	->	99	0.03	0.00
91	->	100	0.00	0.01
91	->	101	-0.08	0.03
91	->	102	-1.20	0.45
91	->	103	-11.33	0.63
91	->	104	-3.44	0.79
91	->	105	-0.27	0.07
91	->	106	-0.04	0.03
91	->	107	0.01	0.01
92	->	1	-2.35	1.62
92	->	2	-0.01	0.00
92	->	3	-0.02	0.01
92	->	4	-0.01	0.01
92	->	5	0.07	0.02
92	->	6	-0.01	0.00
92	->	7	-0.00	0.00
92	->	8	0.03	0.01
92	->	9	-0.00	0.00

92	->	10	0.09	0.04
92	->	11	0.04	0.04
92	->	12	-6.59	2.43
92	->	13	-0.16	0.05
92	->	14	-1.66	0.30
92	->	15	-0.17	0.19
92	->	16	-0.27	0.13
92	->	17	-0.01	0.00
92	->	18	-0.06	0.02
92	->	19	-2.98	0.74
92	->	20	-0.02	0.03
92	->	21	-0.05	0.02
92	->	22	-2.66	1.08
92	->	23	-0.91	1.40
92	->	24	0.00	0.02
92	->	25	-0.08	0.02
92	->	26	-0.07	0.07
92	->	27	-0.00	0.02
92	->	28	-0.01	0.00
92	->	29	-0.00	0.00
92	->	30	-0.00	0.00
92	->	31	-0.02	0.01
92	->	32	-0.00	0.00
92	->	33	-0.00	0.00
92	->	34	-0.00	0.00
92	->	35	0.01	0.00
92	->	36	-0.01	0.00
92	->	37	0.00	0.00
92	->	38	0.01	0.00
92	->	39	0.00	0.00
92	->	40	-0.03	0.00
92	->	41	-0.00	0.00
92	->	42	-0.02	0.00
92	->	43	-0.02	0.01
92	->	44	-8.69	0.72
92	->	45	-0.00	0.00
92	->	46	0.67	0.21
92	->	47	-0.43	0.23
92	->	48	-0.00	0.00
92	->	49	-0.01	0.00
92	->	50	-0.01	0.00
92	->	51	-0.01	0.00
92	->	52	-0.00	0.00
92	->	53	-0.02	0.00
92	->	54	-0.00	0.00
92	->	55	-0.00	0.00
92	->	56	-0.00	0.00
92	->	57	-0.00	0.00
92	->	58	0.01	0.00
92	->	59	-0.01	0.00

92	->	60	-0.00	0.00
92	->	61	-0.00	0.00
92	->	62	0.00	0.00
92	->	63	0.00	0.00
92	->	64	0.00	0.00
92	->	65	0.00	0.00
92	->	66	0.00	0.00
92	->	67	0.00	0.00
92	->	68	-0.01	0.00
92	->	69	-0.00	0.00
92	->	70	-0.00	0.00
92	->	71	0.00	0.00
92	->	72	-0.01	0.02
92	->	73	0.01	0.02
92	->	74	-0.05	0.03
92	->	75	0.00	0.00
92	->	76	-0.00	0.01
92	->	77	-0.01	0.01
92	->	78	-0.02	0.00
92	->	79	-0.00	0.00
92	->	80	-0.00	0.00
92	->	81	-0.01	0.00
92	->	82	-0.00	0.00
92	->	83	-0.00	0.00
92	->	84	-0.00	0.00
92	->	85	-0.00	0.00
92	->	86	0.00	0.00
92	->	87	0.01	0.00
92	->	88	0.01	0.00
92	->	89	-0.01	0.01
92	->	90	-0.18	0.05
92	->	91	-34.16	0.95
92	->	92	-165.21	3.29
92	->	93	-33.49	0.96
92	->	94	-0.32	0.08
92	->	95	-0.03	0.01
92	->	96	-0.00	0.00
92	->	97	0.00	0.00
92	->	98	0.02	0.01
92	->	99	0.01	0.01
92	->	100	-0.06	0.03
92	->	101	-1.08	0.41
92	->	102	-11.85	0.98
92	->	103	-3.43	0.73
92	->	104	-0.32	0.09
92	->	105	-0.03	0.01
92	->	106	0.02	0.00
92	->	107	0.01	0.00
93	->	1	-8.69	4.17
93	->	2	-0.02	0.01

93	->	3	-0.02	0.01
93	->	4	-0.00	0.00
93	->	5	0.03	0.01
93	->	6	-0.00	0.00
93	->	7	-0.00	0.00
93	->	8	0.02	0.01
93	->	9	-0.00	0.00
93	->	10	0.03	0.00
93	->	11	0.04	0.01
93	->	12	-2.58	3.34
93	->	13	-0.14	0.15
93	->	14	-1.12	0.79
93	->	15	-3.24	2.64
93	->	16	-4.01	1.26
93	->	17	-0.01	0.01
93	->	18	-0.18	0.05
93	->	19	-0.07	0.97
93	->	20	0.01	0.01
93	->	21	-0.03	0.01
93	->	22	-8.21	1.31
93	->	23	-0.02	0.01
93	->	24	-0.01	0.00
93	->	25	-0.05	0.01
93	->	26	-0.01	0.00
93	->	27	-0.00	0.00
93	->	28	-0.00	0.00
93	->	29	-0.00	0.00
93	->	30	-0.00	0.00
93	->	31	-0.01	0.00
93	->	32	-0.00	0.00
93	->	33	-0.00	0.00
93	->	34	-0.00	0.00
93	->	35	0.01	0.00
93	->	36	-0.00	0.00
93	->	37	0.00	0.00
93	->	38	0.01	0.00
93	->	39	0.00	0.00
93	->	40	-0.01	0.00
93	->	41	-0.00	0.00
93	->	42	-0.01	0.00
93	->	43	-0.01	0.00
93	->	44	-1.89	0.45
93	->	45	0.00	0.01
93	->	46	0.43	0.15
93	->	47	-0.03	0.02
93	->	48	-0.00	0.00
93	->	49	-0.00	0.00
93	->	50	-0.01	0.00
93	->	51	-0.00	0.00
93	->	52	-0.00	0.00

93 ->	53	-0.01	0.00
93 ->	54	-0.00	0.00
93 ->	55	-0.00	0.00
93 ->	56	-0.00	0.00
93 ->	57	-0.00	0.00
93 ->	58	0.00	0.00
93 ->	59	-0.01	0.00
93 ->	60	-0.00	0.00
93 ->	61	-0.01	0.01
93 ->	62	0.00	0.00
93 ->	63	0.00	0.00
93 ->	64	0.00	0.00
93 ->	65	0.00	0.00
93 ->	66	0.00	0.00
93 ->	67	-0.00	0.00
93 ->	68	-0.00	0.00
93 ->	69	-0.00	0.00
93 ->	70	-0.00	0.00
93 ->	71	0.00	0.00
93 ->	72	-0.00	0.01
93 ->	73	-0.02	0.03
93 ->	74	-0.01	0.02
93 ->	75	0.01	0.00
93 ->	76	0.00	0.01
93 ->	77	-0.03	0.01
93 ->	78	-0.02	0.00
93 ->	79	-0.00	0.00
93 ->	80	-0.00	0.00
93 ->	81	-0.02	0.01
93 ->	82	-0.00	0.00
93 ->	83	-0.00	0.00
93 ->	84	-0.00	0.00
93 ->	85	-0.00	0.00
93 ->	86	0.00	0.00
93 ->	87	0.00	0.00
93 ->	88	0.01	0.00
93 ->	89	0.01	0.00
93 ->	90	0.00	0.01
93 ->	91	-0.34	0.08
93 ->	92	-33.37	0.94
93 ->	93	-162.00	3.88
93 ->	94	-36.29	1.13
93 ->	95	-0.39	0.09
93 ->	96	-0.04	0.01
93 ->	97	-0.00	0.00
93 ->	98	0.01	0.02
93 ->	99	-0.07	0.05
93 ->	100	-0.05	0.37
93 ->	101	-11.97	0.77
93 ->	102	-4.02	0.88

93	->	103	-0.41	0.12
93	->	104	-0.04	0.02
93	->	105	0.01	0.00
93	->	106	0.02	0.00
93	->	107	0.01	0.00
94	->	1	-0.18	0.13
94	->	2	-0.01	0.01
94	->	3	-0.01	0.01
94	->	4	-0.00	0.00
94	->	5	0.01	0.00
94	->	6	-0.00	0.00
94	->	7	-0.00	0.00
94	->	8	0.01	0.00
94	->	9	0.00	0.00
94	->	10	0.01	0.00
94	->	11	0.01	0.00
94	->	12	-0.05	0.01
94	->	13	-0.03	0.01
94	->	14	-0.04	0.04
94	->	15	-0.45	0.98
94	->	16	-2.88	2.13
94	->	17	-0.01	0.01
94	->	18	-0.05	0.04
94	->	19	-0.14	0.23
94	->	20	-0.01	0.00
94	->	21	-0.00	0.01
94	->	22	0.20	0.29
94	->	23	-0.01	0.00
94	->	24	-0.00	0.00
94	->	25	-0.02	0.00
94	->	26	-0.00	0.00
94	->	27	-0.00	0.00
94	->	28	-0.00	0.00
94	->	29	-0.00	0.00
94	->	30	-0.00	0.00
94	->	31	-0.00	0.00
94	->	32	-0.00	0.00
94	->	33	-0.00	0.00
94	->	34	-0.00	0.00
94	->	35	0.00	0.00
94	->	36	-0.00	0.00
94	->	37	0.00	0.00
94	->	38	0.00	0.00
94	->	39	0.00	0.00
94	->	40	-0.01	0.00
94	->	41	-0.00	0.00
94	->	42	-0.00	0.00
94	->	43	0.00	0.00
94	->	44	0.01	0.07
94	->	45	-0.01	0.01

94	->	46	-0.04	0.05
94	->	47	-0.01	0.01
94	->	48	-0.00	0.00
94	->	49	-0.00	0.00
94	->	50	-0.00	0.00
94	->	51	-0.00	0.00
94	->	52	-0.00	0.00
94	->	53	-0.02	0.00
94	->	54	-0.00	0.00
94	->	55	-0.00	0.00
94	->	56	-0.00	0.00
94	->	57	-0.00	0.00
94	->	58	0.01	0.00
94	->	59	-0.01	0.00
94	->	60	-0.00	0.00
94	->	61	-0.00	0.00
94	->	62	0.00	0.00
94	->	63	0.01	0.00
94	->	64	0.01	0.00
94	->	65	0.00	0.00
94	->	66	0.01	0.00
94	->	67	-0.00	0.00
94	->	68	-0.00	0.00
94	->	69	-0.01	0.00
94	->	70	-0.00	0.00
94	->	71	-0.00	0.00
94	->	72	-0.02	0.00
94	->	73	-0.01	0.00
94	->	74	0.02	0.00
94	->	75	0.01	0.00
94	->	76	-0.03	0.01
94	->	77	-0.04	0.01
94	->	78	-0.01	0.00
94	->	79	-0.00	0.00
94	->	80	-0.00	0.00
94	->	81	-0.02	0.01
94	->	82	-0.00	0.00
94	->	83	-0.00	0.00
94	->	84	-0.00	0.00
94	->	85	-0.00	0.00
94	->	86	0.00	0.00
94	->	87	0.00	0.00
94	->	88	0.00	0.00
94	->	89	0.01	0.00
94	->	90	0.01	0.00
94	->	91	-0.01	0.01
94	->	92	-0.32	0.08
94	->	93	-36.05	1.10
94	->	94	-131.74	2.97
94	->	95	-34.70	0.77

94	->	96	-0.30	0.07
94	->	97	-0.02	0.01
94	->	98	-0.17	0.05
94	->	99	-2.35	0.57
94	->	100	-6.18	0.46
94	->	101	-0.77	0.33
94	->	102	-0.28	0.09
94	->	103	-0.07	0.05
94	->	104	0.02	0.01
94	->	105	0.01	0.00
94	->	106	0.01	0.00
94	->	107	0.00	0.00
95	->	1	-0.04	0.01
95	->	2	-0.00	0.00
95	->	3	-0.01	0.01
95	->	4	-0.00	0.00
95	->	5	0.01	0.00
95	->	6	-0.00	0.00
95	->	7	0.00	0.00
95	->	8	0.00	0.00
95	->	9	0.00	0.00
95	->	10	0.01	0.00
95	->	11	0.01	0.00
95	->	12	-0.02	0.00
95	->	13	-0.01	0.00
95	->	14	-0.00	0.00
95	->	15	-0.01	0.01
95	->	16	-0.42	0.64
95	->	17	-0.02	0.02
95	->	18	-0.08	0.07
95	->	19	-0.01	0.02
95	->	20	-0.00	0.00
95	->	21	-0.00	0.00
95	->	22	-0.07	0.02
95	->	23	-0.00	0.00
95	->	24	-0.00	0.00
95	->	25	-0.02	0.00
95	->	26	-0.00	0.00
95	->	27	-0.00	0.00
95	->	28	-0.00	0.00
95	->	29	-0.00	0.00
95	->	30	0.00	0.00
95	->	31	-0.00	0.00
95	->	32	-0.00	0.00
95	->	33	-0.00	0.00
95	->	34	0.00	0.00
95	->	35	0.01	0.00
95	->	36	-0.00	0.00
95	->	37	0.00	0.00
95	->	38	0.00	0.00

95	->	39	-0.00	0.00
95	->	40	-0.00	0.00
95	->	41	-0.00	0.00
95	->	42	-0.00	0.00
95	->	43	0.00	0.00
95	->	44	-0.04	0.01
95	->	45	-0.00	0.00
95	->	46	0.02	0.00
95	->	47	-0.00	0.00
95	->	48	-0.00	0.00
95	->	49	-0.00	0.00
95	->	50	-0.00	0.00
95	->	51	-0.00	0.00
95	->	52	-0.00	0.00
95	->	53	-0.02	0.00
95	->	54	-0.00	0.00
95	->	55	-0.00	0.00
95	->	56	-0.00	0.00
95	->	57	-0.00	0.00
95	->	58	0.01	0.00
95	->	59	-0.01	0.00
95	->	60	-0.00	0.00
95	->	61	-0.00	0.00
95	->	62	0.00	0.00
95	->	63	0.01	0.00
95	->	64	0.01	0.00
95	->	65	0.00	0.00
95	->	66	0.01	0.00
95	->	67	0.00	0.00
95	->	68	-0.00	0.00
95	->	69	-0.01	0.00
95	->	70	-0.00	0.00
95	->	71	0.00	0.00
95	->	72	-0.02	0.00
95	->	73	-0.01	0.00
95	->	74	0.02	0.00
95	->	75	0.01	0.00
95	->	76	-0.03	0.01
95	->	77	-0.03	0.01
95	->	78	-0.01	0.00
95	->	79	-0.00	0.00
95	->	80	-0.00	0.00
95	->	81	-0.01	0.00
95	->	82	-0.00	0.00
95	->	83	-0.00	0.00
95	->	84	-0.00	0.00
95	->	85	-0.00	0.00
95	->	86	0.00	0.00
95	->	87	0.00	0.00
95	->	88	0.00	0.00

95	->	89	0.01	0.00
95	->	90	0.01	0.00
95	->	91	0.00	0.00
95	->	92	-0.03	0.01
95	->	93	-0.39	0.09
95	->	94	-34.47	0.76
95	->	95	-218.59	3.03
95	->	96	-33.55	0.85
95	->	97	-0.09	0.07
95	->	98	-1.37	0.64
95	->	99	-10.92	0.70
95	->	100	-0.59	0.15
95	->	101	0.05	0.05
95	->	102	-0.02	0.05
95	->	103	0.01	0.01
95	->	104	0.02	0.00
95	->	105	0.01	0.00
95	->	106	0.01	0.00
95	->	107	0.00	0.00
96	->	1	-0.01	0.00
96	->	2	-0.00	0.00
96	->	3	-0.01	0.01
96	->	4	0.00	0.00
96	->	5	0.00	0.00
96	->	6	-0.00	0.00
96	->	7	-0.00	0.00
96	->	8	0.00	0.00
96	->	9	0.00	0.00
96	->	10	0.00	0.00
96	->	11	0.00	0.00
96	->	12	-0.01	0.00
96	->	13	-0.00	0.00
96	->	14	-0.00	0.00
96	->	15	-0.01	0.00
96	->	16	-0.13	0.09
96	->	17	-0.04	0.05
96	->	18	-0.02	0.01
96	->	19	0.01	0.01
96	->	20	-0.00	0.00
96	->	21	-0.01	0.00
96	->	22	-0.03	0.01
96	->	23	-0.00	0.00
96	->	24	-0.00	0.00
96	->	25	-0.02	0.00
96	->	26	-0.00	0.00
96	->	27	-0.00	0.00
96	->	28	-0.00	0.00
96	->	29	-0.00	0.00
96	->	30	0.00	0.00
96	->	31	-0.00	0.00

96	->	32	-0.00	0.00
96	->	33	-0.00	0.00
96	->	34	0.00	0.00
96	->	35	0.00	0.00
96	->	36	-0.00	0.00
96	->	37	0.00	0.00
96	->	38	0.00	0.00
96	->	39	0.00	0.00
96	->	40	-0.00	0.00
96	->	41	-0.00	0.00
96	->	42	-0.00	0.00
96	->	43	0.00	0.00
96	->	44	-0.02	0.00
96	->	45	-0.00	0.00
96	->	46	0.02	0.00
96	->	47	-0.00	0.00
96	->	48	-0.00	0.00
96	->	49	-0.00	0.00
96	->	50	-0.00	0.00
96	->	51	-0.00	0.00
96	->	52	-0.00	0.00
96	->	53	-0.01	0.00
96	->	54	-0.00	0.00
96	->	55	-0.00	0.00
96	->	56	-0.00	0.00
96	->	57	-0.00	0.00
96	->	58	0.01	0.00
96	->	59	-0.00	0.00
96	->	60	-0.00	0.00
96	->	61	-0.00	0.00
96	->	62	0.00	0.00
96	->	63	0.00	0.00
96	->	64	0.01	0.00
96	->	65	0.00	0.00
96	->	66	0.00	0.00
96	->	67	-0.00	0.00
96	->	68	-0.00	0.00
96	->	69	-0.00	0.00
96	->	70	-0.00	0.00
96	->	71	0.00	0.00
96	->	72	-0.01	0.00
96	->	73	-0.00	0.00
96	->	74	0.01	0.00
96	->	75	0.00	0.00
96	->	76	-0.01	0.01
96	->	77	-0.01	0.00
96	->	78	-0.00	0.00
96	->	79	-0.00	0.00
96	->	80	-0.00	0.00
96	->	81	-0.01	0.00

96	->	82	-0.00	0.00
96	->	83	-0.00	0.00
96	->	84	-0.00	0.00
96	->	85	0.00	0.00
96	->	86	0.00	0.00
96	->	87	0.00	0.00
96	->	88	0.00	0.00
96	->	89	0.00	0.00
96	->	90	0.00	0.00
96	->	91	0.00	0.00
96	->	92	-0.00	0.00
96	->	93	-0.04	0.01
96	->	94	-0.30	0.07
96	->	95	-33.29	0.86
96	->	96	-217.79	3.77
96	->	97	-1.36	1.75
96	->	98	-8.51	3.38
96	->	99	-1.92	0.79
96	->	100	-0.12	0.08
96	->	101	-0.01	0.04
96	->	102	0.01	0.01
96	->	103	0.01	0.00
96	->	104	0.01	0.00
96	->	105	0.00	0.00
96	->	106	0.00	0.00
96	->	107	0.00	0.00
97	->	1	-0.01	0.00
97	->	2	-0.00	0.00
97	->	3	-0.03	0.04
97	->	4	-0.00	0.00
97	->	5	0.00	0.00
97	->	6	-0.00	0.00
97	->	7	-0.00	0.00
97	->	8	0.00	0.00
97	->	9	-0.00	0.00
97	->	10	0.00	0.00
97	->	11	0.00	0.00
97	->	12	-0.00	0.00
97	->	13	-0.01	0.00
97	->	14	-0.01	0.00
97	->	15	-0.00	0.00
97	->	16	-0.06	0.02
97	->	17	-0.47	0.67
97	->	18	-0.13	0.05
97	->	19	-0.01	0.01
97	->	20	-0.06	0.06
97	->	21	-0.58	0.70
97	->	22	-0.03	0.01
97	->	23	-0.01	0.01
97	->	24	-0.03	0.03

97	->	25	-0.31	0.26
97	->	26	-0.00	0.00
97	->	27	-0.00	0.00
97	->	28	-0.00	0.00
97	->	29	-0.00	0.00
97	->	30	-0.01	0.01
97	->	31	-0.01	0.00
97	->	32	-0.01	0.01
97	->	33	-0.01	0.00
97	->	34	-0.00	0.00
97	->	35	0.00	0.00
97	->	36	-0.00	0.00
97	->	37	0.00	0.00
97	->	38	0.00	0.00
97	->	39	0.00	0.00
97	->	40	-0.00	0.00
97	->	41	-0.00	0.00
97	->	42	-0.00	0.00
97	->	43	-0.00	0.00
97	->	44	-0.02	0.01
97	->	45	-0.01	0.00
97	->	46	0.01	0.00
97	->	47	-0.00	0.00
97	->	48	-0.00	0.00
97	->	49	-0.00	0.00
97	->	50	-0.00	0.00
97	->	51	-0.00	0.00
97	->	52	-0.00	0.00
97	->	53	-0.00	0.00
97	->	54	-0.00	0.00
97	->	55	0.00	0.00
97	->	56	-0.00	0.00
97	->	57	-0.00	0.00
97	->	58	0.00	0.00
97	->	59	-0.00	0.00
97	->	60	-0.00	0.00
97	->	61	-0.00	0.00
97	->	62	0.00	0.00
97	->	63	0.00	0.00
97	->	64	0.00	0.00
97	->	65	0.00	0.00
97	->	66	0.00	0.00
97	->	67	0.00	0.00
97	->	68	-0.00	0.00
97	->	69	-0.00	0.00
97	->	70	-0.00	0.00
97	->	71	0.00	0.00
97	->	72	-0.00	0.00
97	->	73	-0.00	0.00
97	->	74	0.00	0.00

97	->	75	0.00	0.00
97	->	76	-0.00	0.00
97	->	77	-0.00	0.00
97	->	78	-0.00	0.00
97	->	79	-0.00	0.00
97	->	80	0.00	0.00
97	->	81	-0.00	0.00
97	->	82	-0.00	0.00
97	->	83	0.00	0.00
97	->	84	0.00	0.00
97	->	85	0.00	0.00
97	->	86	0.00	0.00
97	->	87	0.00	0.00
97	->	88	0.00	0.00
97	->	89	0.00	0.00
97	->	90	0.00	0.00
97	->	91	0.00	0.00
97	->	92	0.00	0.00
97	->	93	-0.00	0.00
97	->	94	-0.02	0.01
97	->	95	-0.09	0.07
97	->	96	-1.33	1.72
97	->	97	-50.62	2.77
97	->	98	-35.67	1.05
97	->	99	-0.65	0.90
97	->	100	-0.03	0.03
97	->	101	-0.00	0.00
97	->	102	0.00	0.00
97	->	103	0.00	0.00
97	->	104	0.00	0.00
97	->	105	0.00	0.00
97	->	106	0.00	0.00
97	->	107	0.00	0.00
98	->	1	-0.04	0.01
98	->	2	-0.00	0.00
98	->	3	-0.04	0.03
98	->	4	-0.00	0.00
98	->	5	0.03	0.00
98	->	6	-0.01	0.00
98	->	7	-0.00	0.00
98	->	8	0.01	0.00
98	->	9	-0.00	0.00
98	->	10	0.02	0.00
98	->	11	0.01	0.00
98	->	12	-0.02	0.00
98	->	13	-0.02	0.00
98	->	14	-0.01	0.00
98	->	15	-0.01	0.01
98	->	16	-0.52	0.24
98	->	17	-1.39	1.33

98	->	18	-2.09	0.81
98	->	19	0.05	0.06
98	->	20	-0.14	0.07
98	->	21	-2.63	1.36
98	->	22	-0.88	0.43
98	->	23	-0.02	0.01
98	->	24	-0.09	0.05
98	->	25	-5.56	2.34
98	->	26	0.00	0.00
98	->	27	-0.01	0.00
98	->	28	-0.00	0.01
98	->	29	-0.00	0.00
98	->	30	-0.02	0.11
98	->	31	-0.06	0.02
98	->	32	-0.09	0.04
98	->	33	-0.03	0.04
98	->	34	0.00	0.00
98	->	35	0.02	0.00
98	->	36	-0.01	0.00
98	->	37	0.00	0.00
98	->	38	0.01	0.00
98	->	39	0.00	0.00
98	->	40	-0.02	0.00
98	->	41	-0.01	0.01
98	->	42	-0.03	0.01
98	->	43	0.07	0.05
98	->	44	-0.80	0.37
98	->	45	-0.33	0.47
98	->	46	0.30	0.15
98	->	47	0.01	0.01
98	->	48	-0.02	0.01
98	->	49	-0.01	0.00
98	->	50	-0.00	0.00
98	->	51	-0.00	0.00
98	->	52	-0.00	0.00
98	->	53	-0.02	0.00
98	->	54	-0.00	0.00
98	->	55	0.00	0.00
98	->	56	-0.00	0.00
98	->	57	-0.00	0.00
98	->	58	0.01	0.00
98	->	59	-0.00	0.00
98	->	60	-0.00	0.00
98	->	61	-0.00	0.00
98	->	62	0.00	0.00
98	->	63	0.00	0.00
98	->	64	0.00	0.00
98	->	65	0.00	0.00
98	->	66	0.00	0.00
98	->	67	0.00	0.00

98	->	68	-0.00	0.00
98	->	69	-0.00	0.00
98	->	70	-0.00	0.00
98	->	71	0.00	0.00
98	->	72	-0.01	0.00
98	->	73	-0.00	0.00
98	->	74	0.01	0.00
98	->	75	0.01	0.00
98	->	76	-0.01	0.00
98	->	77	-0.01	0.00
98	->	78	-0.01	0.00
98	->	79	-0.00	0.00
98	->	80	-0.00	0.00
98	->	81	-0.00	0.00
98	->	82	-0.00	0.00
98	->	83	-0.00	0.00
98	->	84	-0.00	0.00
98	->	85	-0.00	0.00
98	->	86	0.00	0.00
98	->	87	0.00	0.00
98	->	88	0.01	0.00
98	->	89	0.01	0.00
98	->	90	0.01	0.00
98	->	91	0.02	0.00
98	->	92	0.02	0.01
98	->	93	0.01	0.02
98	->	94	-0.17	0.05
98	->	95	-1.37	0.64
98	->	96	-8.56	3.39
98	->	97	-35.49	1.07
98	->	98	-175.19	3.91
98	->	99	-33.78	2.25
98	->	100	-0.24	0.05
98	->	101	-0.05	0.02
98	->	102	0.00	0.00
98	->	103	0.01	0.00
98	->	104	0.01	0.00
98	->	105	0.01	0.00
98	->	106	0.00	0.00
98	->	107	0.00	0.00
99	->	1	-0.04	0.00
99	->	2	-0.00	0.00
99	->	3	-0.01	0.00
99	->	4	-0.00	0.00
99	->	5	0.02	0.00
99	->	6	-0.00	0.00
99	->	7	-0.00	0.00
99	->	8	0.01	0.00
99	->	9	-0.00	0.00
99	->	10	0.02	0.00

99	->	11	0.01	0.00
99	->	12	-0.02	0.00
99	->	13	-0.01	0.00
99	->	14	-0.01	0.00
99	->	15	-0.01	0.00
99	->	16	-2.92	1.64
99	->	17	-0.06	0.05
99	->	18	-0.62	0.44
99	->	19	0.17	0.07
99	->	20	-0.02	0.01
99	->	21	-0.22	0.09
99	->	22	-1.12	0.37
99	->	23	-0.00	0.01
99	->	24	-0.02	0.01
99	->	25	-10.26	2.74
99	->	26	-0.00	0.01
99	->	27	-0.00	0.00
99	->	28	-0.00	0.00
99	->	29	-0.00	0.00
99	->	30	-0.01	0.01
99	->	31	-0.07	0.03
99	->	32	-0.18	0.07
99	->	33	-1.22	0.29
99	->	34	-0.00	0.00
99	->	35	0.03	0.00
99	->	36	-0.02	0.01
99	->	37	0.00	0.00
99	->	38	0.02	0.00
99	->	39	-0.00	0.00
99	->	40	-0.03	0.01
99	->	41	-0.02	0.01
99	->	42	-0.07	0.03
99	->	43	0.06	0.12
99	->	44	-3.02	1.92
99	->	45	-5.33	3.81
99	->	46	0.44	0.45
99	->	47	-0.02	0.03
99	->	48	-0.07	0.04
99	->	49	-0.03	0.02
99	->	50	-0.01	0.01
99	->	51	-0.01	0.00
99	->	52	-0.01	0.00
99	->	53	-0.05	0.01
99	->	54	-0.00	0.00
99	->	55	-0.00	0.00
99	->	56	-0.00	0.00
99	->	57	-0.00	0.00
99	->	58	0.02	0.01
99	->	59	-0.01	0.00
99	->	60	-0.00	0.00

99	->	61	-0.00	0.00
99	->	62	0.00	0.00
99	->	63	0.00	0.00
99	->	64	0.00	0.00
99	->	65	0.00	0.00
99	->	66	0.00	0.00
99	->	67	0.00	0.00
99	->	68	-0.01	0.00
99	->	69	-0.01	0.00
99	->	70	-0.00	0.00
99	->	71	0.00	0.00
99	->	72	-0.03	0.00
99	->	73	-0.00	0.00
99	->	74	0.02	0.00
99	->	75	0.01	0.00
99	->	76	-0.01	0.01
99	->	77	-0.01	0.00
99	->	78	-0.01	0.00
99	->	79	-0.00	0.00
99	->	80	-0.00	0.00
99	->	81	-0.01	0.00
99	->	82	-0.00	0.00
99	->	83	-0.00	0.00
99	->	84	-0.00	0.00
99	->	85	-0.00	0.00
99	->	86	0.00	0.00
99	->	87	0.01	0.00
99	->	88	0.01	0.00
99	->	89	0.02	0.00
99	->	90	0.02	0.00
99	->	91	0.03	0.00
99	->	92	0.01	0.01
99	->	93	-0.07	0.05
99	->	94	-2.35	0.58
99	->	95	-10.95	0.70
99	->	96	-1.92	0.79
99	->	97	-0.65	0.91
99	->	98	-33.64	2.22
99	->	99	-163.68	4.77
99	->	100	-34.86	0.79
99	->	101	-0.47	0.07
99	->	102	-0.05	0.01
99	->	103	0.00	0.00
99	->	104	0.01	0.00
99	->	105	0.01	0.00
99	->	106	0.01	0.00
99	->	107	0.00	0.00
100	->	1	-0.02	0.00
100	->	2	-0.00	0.00
100	->	3	-0.00	0.00

100	->	4	0.00	0.00
100	->	5	0.01	0.00
100	->	6	-0.00	0.00
100	->	7	-0.00	0.00
100	->	8	0.00	0.00
100	->	9	-0.00	0.00
100	->	10	0.01	0.00
100	->	11	0.00	0.00
100	->	12	-0.01	0.00
100	->	13	-0.01	0.00
100	->	14	-0.00	0.00
100	->	15	-0.00	0.00
100	->	16	-0.66	0.36
100	->	17	-0.01	0.01
100	->	18	-0.07	0.03
100	->	19	0.08	0.04
100	->	20	-0.00	0.00
100	->	21	-0.02	0.00
100	->	22	-2.38	1.05
100	->	23	-0.01	0.00
100	->	24	-0.00	0.00
100	->	25	-0.08	0.02
100	->	26	-0.00	0.00
100	->	27	-0.00	0.00
100	->	28	-0.00	0.00
100	->	29	-0.00	0.00
100	->	30	0.00	0.00
100	->	31	-0.02	0.00
100	->	32	-0.01	0.00
100	->	33	-0.06	0.03
100	->	34	-0.00	0.00
100	->	35	0.03	0.00
100	->	36	-0.03	0.02
100	->	37	-0.00	0.00
100	->	38	0.02	0.00
100	->	39	-0.00	0.00
100	->	40	-0.02	0.00
100	->	41	-0.00	0.00
100	->	42	-0.01	0.00
100	->	43	-0.02	0.01
100	->	44	-0.57	0.23
100	->	45	-3.00	1.86
100	->	46	-1.48	0.43
100	->	47	-0.07	0.04
100	->	48	-0.08	0.02
100	->	49	-0.15	0.09
100	->	50	-0.02	0.01
100	->	51	-0.01	0.00
100	->	52	-0.02	0.00
100	->	53	-0.22	0.24

100	->	54	-0.00	0.00
100	->	55	-0.00	0.00
100	->	56	-0.00	0.00
100	->	57	-0.00	0.00
100	->	58	0.03	0.01
100	->	59	-0.02	0.01
100	->	60	-0.01	0.01
100	->	61	-0.03	0.03
100	->	62	0.00	0.00
100	->	63	0.01	0.00
100	->	64	0.01	0.00
100	->	65	0.00	0.00
100	->	66	0.01	0.00
100	->	67	0.00	0.00
100	->	68	-0.01	0.00
100	->	69	-0.02	0.01
100	->	70	-0.00	0.00
100	->	71	-0.00	0.00
100	->	72	-0.04	0.01
100	->	73	-0.02	0.01
100	->	74	0.02	0.01
100	->	75	0.01	0.00
100	->	76	-0.03	0.02
100	->	77	-0.02	0.01
100	->	78	-0.01	0.00
100	->	79	-0.00	0.00
100	->	80	-0.00	0.00
100	->	81	-0.01	0.00
100	->	82	-0.00	0.00
100	->	83	0.00	0.00
100	->	84	-0.00	0.00
100	->	85	-0.00	0.00
100	->	86	0.00	0.00
100	->	87	0.01	0.00
100	->	88	0.01	0.00
100	->	89	0.02	0.00
100	->	90	0.01	0.00
100	->	91	0.00	0.01
100	->	92	-0.06	0.03
100	->	93	-0.04	0.37
100	->	94	-6.19	0.46
100	->	95	-0.59	0.15
100	->	96	-0.12	0.07
100	->	97	-0.03	0.03
100	->	98	-0.24	0.05
100	->	99	-34.66	0.79
100	->	100	-92.25	2.81
100	->	101	-34.64	0.74
100	->	102	-0.28	0.05
100	->	103	-0.02	0.01

100	->	104	0.01	0.00
100	->	105	0.01	0.00
100	->	106	0.00	0.00
100	->	107	0.00	0.00
101	->	1	-0.02	0.00
101	->	2	-0.00	0.00
101	->	3	-0.00	0.00
101	->	4	0.00	0.00
101	->	5	0.01	0.00
101	->	6	-0.00	0.00
101	->	7	-0.00	0.00
101	->	8	0.00	0.00
101	->	9	0.00	0.00
101	->	10	0.00	0.00
101	->	11	0.00	0.00
101	->	12	-0.01	0.00
101	->	13	-0.00	0.00
101	->	14	-0.00	0.00
101	->	15	-0.00	0.00
101	->	16	-0.05	0.06
101	->	17	-0.00	0.00
101	->	18	-0.00	0.00
101	->	19	-0.01	0.03
101	->	20	-0.00	0.00
101	->	21	0.00	0.00
101	->	22	0.61	0.41
101	->	23	-0.00	0.00
101	->	24	-0.00	0.00
101	->	25	-0.01	0.01
101	->	26	-0.00	0.00
101	->	27	-0.00	0.00
101	->	28	-0.00	0.00
101	->	29	-0.00	0.00
101	->	30	-0.00	0.00
101	->	31	-0.01	0.00
101	->	32	-0.00	0.00
101	->	33	-0.01	0.00
101	->	34	0.00	0.00
101	->	35	0.01	0.00
101	->	36	-0.02	0.01
101	->	37	0.00	0.00
101	->	38	0.01	0.00
101	->	39	-0.00	0.00
101	->	40	-0.01	0.00
101	->	41	0.00	0.00
101	->	42	-0.00	0.00
101	->	43	0.00	0.00
101	->	44	1.30	0.54
101	->	45	-0.07	0.02
101	->	46	-5.63	1.14

101 ->	47	-0.24	0.11
101 ->	48	-0.02	0.01
101 ->	49	-0.12	0.07
101 ->	50	-0.04	0.03
101 ->	51	-0.01	0.00
101 ->	52	-0.01	0.00
101 ->	53	-1.39	2.31
101 ->	54	-0.00	0.00
101 ->	55	-0.00	0.00
101 ->	56	-0.00	0.00
101 ->	57	-0.00	0.00
101 ->	58	0.04	0.01
101 ->	59	-0.04	0.03
101 ->	60	-0.07	0.09
101 ->	61	-0.81	0.92
101 ->	62	-0.00	0.00
101 ->	63	0.02	0.01
101 ->	64	0.02	0.01
101 ->	65	-0.00	0.00
101 ->	66	0.01	0.00
101 ->	67	0.00	0.00
101 ->	68	-0.02	0.00
101 ->	69	-0.04	0.02
101 ->	70	-0.01	0.01
101 ->	71	-0.02	0.01
101 ->	72	-0.13	0.05
101 ->	73	-0.68	0.62
101 ->	74	0.03	0.02
101 ->	75	0.01	0.00
101 ->	76	-0.65	0.69
101 ->	77	-0.06	0.04
101 ->	78	-0.02	0.00
101 ->	79	-0.00	0.00
101 ->	80	-0.00	0.00
101 ->	81	-0.02	0.00
101 ->	82	-0.00	0.00
101 ->	83	-0.00	0.00
101 ->	84	-0.00	0.00
101 ->	85	-0.00	0.00
101 ->	86	0.00	0.00
101 ->	87	0.01	0.00
101 ->	88	0.01	0.00
101 ->	89	0.01	0.00
101 ->	90	-0.00	0.00
101 ->	91	-0.08	0.03
101 ->	92	-1.08	0.41
101 ->	93	-11.94	0.77
101 ->	94	-0.78	0.34
101 ->	95	0.05	0.05
101 ->	96	-0.01	0.04

101	->	97	-0.00	0.00
101	->	98	-0.05	0.02
101	->	99	-0.47	0.07
101	->	100	-34.44	0.74
101	->	101	-212.11	4.95
101	->	102	-31.65	1.29
101	->	103	-0.10	0.04
101	->	104	0.00	0.01
101	->	105	0.01	0.00
101	->	106	0.01	0.00
101	->	107	0.00	0.00
102	->	1	-0.04	0.00
102	->	2	-0.00	0.00
102	->	3	-0.00	0.00
102	->	4	0.00	0.00
102	->	5	0.01	0.00
102	->	6	-0.00	0.00
102	->	7	-0.00	0.00
102	->	8	0.00	0.00
102	->	9	0.00	0.00
102	->	10	0.01	0.00
102	->	11	0.01	0.00
102	->	12	-0.02	0.01
102	->	13	-0.01	0.00
102	->	14	-0.00	0.00
102	->	15	-0.00	0.00
102	->	16	-0.10	0.04
102	->	17	-0.00	0.00
102	->	18	-0.01	0.00
102	->	19	0.04	0.02
102	->	20	-0.00	0.00
102	->	21	-0.00	0.00
102	->	22	-0.14	0.08
102	->	23	-0.00	0.00
102	->	24	-0.00	0.00
102	->	25	-0.01	0.00
102	->	26	-0.00	0.00
102	->	27	-0.00	0.00
102	->	28	-0.00	0.00
102	->	29	-0.00	0.00
102	->	30	0.00	0.00
102	->	31	-0.00	0.00
102	->	32	-0.00	0.00
102	->	33	-0.00	0.00
102	->	34	-0.00	0.00
102	->	35	0.01	0.00
102	->	36	-0.01	0.00
102	->	37	0.00	0.00
102	->	38	0.01	0.00
102	->	39	0.00	0.00

102 ->	40	-0.01	0.00
102 ->	41	-0.00	0.00
102 ->	42	-0.00	0.00
102 ->	43	-0.00	0.00
102 ->	44	0.26	0.36
102 ->	45	-0.01	0.00
102 ->	46	-0.90	0.52
102 ->	47	-0.19	0.13
102 ->	48	-0.01	0.00
102 ->	49	-0.02	0.01
102 ->	50	-0.03	0.01
102 ->	51	-0.00	0.00
102 ->	52	-0.00	0.00
102 ->	53	-0.10	0.05
102 ->	54	-0.00	0.00
102 ->	55	-0.00	0.00
102 ->	56	-0.00	0.00
102 ->	57	-0.00	0.00
102 ->	58	0.02	0.00
102 ->	59	-0.04	0.03
102 ->	60	-0.03	0.02
102 ->	61	-0.05	0.17
102 ->	62	0.01	0.01
102 ->	63	0.04	0.02
102 ->	64	0.03	0.01
102 ->	65	-0.00	0.00
102 ->	66	0.02	0.01
102 ->	67	-0.00	0.00
102 ->	68	-0.02	0.01
102 ->	69	-0.05	0.03
102 ->	70	-0.02	0.02
102 ->	71	-0.04	0.03
102 ->	72	-0.50	0.35
102 ->	73	-3.77	3.35
102 ->	74	-0.06	0.18
102 ->	75	-0.01	0.04
102 ->	76	-5.30	5.81
102 ->	77	-0.82	0.57
102 ->	78	-0.05	0.01
102 ->	79	-0.01	0.01
102 ->	80	-0.01	0.00
102 ->	81	-0.05	0.02
102 ->	82	-0.00	0.00
102 ->	83	-0.00	0.00
102 ->	84	-0.00	0.00
102 ->	85	-0.00	0.01
102 ->	86	0.00	0.00
102 ->	87	0.01	0.00
102 ->	88	0.00	0.00
102 ->	89	-0.00	0.00

102	->	90	-0.05	0.02
102	->	91	-1.21	0.45
102	->	92	-11.82	0.98
102	->	93	-4.04	0.89
102	->	94	-0.28	0.09
102	->	95	-0.03	0.06
102	->	96	0.01	0.01
102	->	97	0.00	0.00
102	->	98	0.00	0.00
102	->	99	-0.05	0.01
102	->	100	-0.28	0.05
102	->	101	-31.42	1.25
102	->	102	-208.91	6.58
102	->	103	-31.70	0.98
102	->	104	-0.17	0.05
102	->	105	0.00	0.00
102	->	106	0.01	0.00
102	->	107	0.00	0.00
103	->	1	-0.05	0.01
103	->	2	-0.00	0.00
103	->	3	-0.00	0.00
103	->	4	-0.00	0.00
103	->	5	0.01	0.00
103	->	6	-0.00	0.00
103	->	7	-0.00	0.00
103	->	8	0.00	0.00
103	->	9	0.00	0.00
103	->	10	0.01	0.00
103	->	11	0.01	0.00
103	->	12	-0.04	0.01
103	->	13	-0.01	0.00
103	->	14	-0.00	0.00
103	->	15	-0.01	0.00
103	->	16	-0.06	0.02
103	->	17	-0.00	0.00
103	->	18	-0.00	0.00
103	->	19	0.04	0.01
103	->	20	-0.00	0.00
103	->	21	-0.00	0.00
103	->	22	-0.07	0.02
103	->	23	-0.00	0.00
103	->	24	-0.00	0.00
103	->	25	-0.01	0.00
103	->	26	-0.00	0.00
103	->	27	-0.00	0.00
103	->	28	-0.00	0.00
103	->	29	-0.00	0.00
103	->	30	0.00	0.00
103	->	31	-0.00	0.00
103	->	32	-0.00	0.00

103	->	33	-0.00	0.00
103	->	34	0.00	0.00
103	->	35	0.01	0.00
103	->	36	-0.00	0.00
103	->	37	0.00	0.00
103	->	38	0.01	0.00
103	->	39	-0.00	0.00
103	->	40	-0.01	0.00
103	->	41	-0.00	0.00
103	->	42	-0.00	0.00
103	->	43	-0.00	0.00
103	->	44	-0.09	0.08
103	->	45	-0.00	0.00
103	->	46	-0.05	0.07
103	->	47	-0.07	0.06
103	->	48	-0.00	0.00
103	->	49	-0.00	0.00
103	->	50	-0.03	0.02
103	->	51	-0.00	0.00
103	->	52	-0.00	0.00
103	->	53	-0.03	0.01
103	->	54	-0.00	0.00
103	->	55	-0.00	0.00
103	->	56	-0.00	0.00
103	->	57	-0.00	0.00
103	->	58	0.01	0.00
103	->	59	-0.02	0.00
103	->	60	-0.00	0.00
103	->	61	-0.02	0.01
103	->	62	0.00	0.00
103	->	63	0.03	0.01
103	->	64	0.03	0.01
103	->	65	0.00	0.00
103	->	66	0.02	0.00
103	->	67	-0.00	0.00
103	->	68	-0.02	0.01
103	->	69	-0.02	0.01
103	->	70	-0.01	0.00
103	->	71	-0.01	0.01
103	->	72	-0.32	0.25
103	->	73	-0.48	0.40
103	->	74	-2.10	0.55
103	->	75	-0.02	0.03
103	->	76	-1.20	1.95
103	->	77	-6.52	3.10
103	->	78	-0.13	0.06
103	->	79	-0.02	0.01
103	->	80	-0.01	0.02
103	->	81	-0.34	0.43
103	->	82	-0.00	0.00

103	->	83	-0.01	0.00
103	->	84	-0.01	0.00
103	->	85	-0.00	0.02
103	->	86	0.00	0.00
103	->	87	0.01	0.00
103	->	88	-0.01	0.01
103	->	89	-0.07	0.03
103	->	90	-0.58	0.33
103	->	91	-11.30	0.63
103	->	92	-3.44	0.74
103	->	93	-0.41	0.12
103	->	94	-0.07	0.05
103	->	95	0.01	0.01
103	->	96	0.01	0.00
103	->	97	0.00	0.00
103	->	98	0.01	0.00
103	->	99	0.00	0.00
103	->	100	-0.02	0.01
103	->	101	-0.10	0.04
103	->	102	-31.47	0.97
103	->	103	-208.56	4.98
103	->	104	-33.38	1.00
103	->	105	-0.10	0.03
103	->	106	0.01	0.01
103	->	107	0.00	0.00
104	->	1	-0.05	0.01
104	->	2	-0.00	0.00
104	->	3	-0.00	0.00
104	->	4	-0.00	0.00
104	->	5	0.01	0.00
104	->	6	-0.00	0.00
104	->	7	0.00	0.00
104	->	8	0.00	0.00
104	->	9	0.00	0.00
104	->	10	0.01	0.00
104	->	11	0.01	0.00
104	->	12	-0.05	0.02
104	->	13	-0.01	0.00
104	->	14	-0.00	0.00
104	->	15	-0.00	0.00
104	->	16	-0.03	0.00
104	->	17	0.00	0.00
104	->	18	-0.00	0.00
104	->	19	0.02	0.00
104	->	20	-0.00	0.00
104	->	21	-0.00	0.00
104	->	22	-0.03	0.00
104	->	23	-0.00	0.00
104	->	24	-0.00	0.00
104	->	25	-0.01	0.00

104	->	26	-0.00	0.00
104	->	27	-0.00	0.00
104	->	28	-0.00	0.00
104	->	29	-0.00	0.00
104	->	30	0.00	0.00
104	->	31	-0.00	0.00
104	->	32	-0.00	0.00
104	->	33	-0.00	0.00
104	->	34	-0.00	0.00
104	->	35	0.01	0.00
104	->	36	-0.00	0.00
104	->	37	0.00	0.00
104	->	38	0.01	0.00
104	->	39	0.00	0.00
104	->	40	-0.01	0.00
104	->	41	-0.00	0.00
104	->	42	-0.00	0.00
104	->	43	-0.00	0.00
104	->	44	-0.06	0.02
104	->	45	-0.00	0.00
104	->	46	0.01	0.01
104	->	47	-0.06	0.03
104	->	48	-0.00	0.00
104	->	49	-0.00	0.00
104	->	50	-0.02	0.01
104	->	51	-0.01	0.00
104	->	52	-0.00	0.00
104	->	53	-0.02	0.00
104	->	54	-0.00	0.00
104	->	55	-0.00	0.00
104	->	56	-0.00	0.00
104	->	57	-0.00	0.00
104	->	58	0.01	0.00
104	->	59	-0.01	0.00
104	->	60	-0.00	0.00
104	->	61	-0.01	0.00
104	->	62	0.00	0.00
104	->	63	0.02	0.00
104	->	64	0.02	0.00
104	->	65	0.00	0.00
104	->	66	0.02	0.00
104	->	67	-0.00	0.00
104	->	68	-0.02	0.00
104	->	69	-0.01	0.00
104	->	70	-0.01	0.00
104	->	71	0.00	0.00
104	->	72	0.20	0.58
104	->	73	-0.05	0.02
104	->	74	-3.27	1.34
104	->	75	-0.08	0.12

104	->	76	-0.10	0.04
104	->	77	-4.02	2.78
104	->	78	-0.30	0.16
104	->	79	-0.01	0.00
104	->	80	-0.02	0.01
104	->	81	-1.98	2.82
104	->	82	-0.01	0.01
104	->	83	-0.01	0.00
104	->	84	-0.01	0.00
104	->	85	-0.01	0.04
104	->	86	-0.00	0.00
104	->	87	-0.00	0.01
104	->	88	-0.06	0.04
104	->	89	-1.44	0.58
104	->	90	-6.00	0.66
104	->	91	-3.42	0.79
104	->	92	-0.32	0.09
104	->	93	-0.04	0.02
104	->	94	0.02	0.01
104	->	95	0.02	0.00
104	->	96	0.01	0.00
104	->	97	0.00	0.00
104	->	98	0.01	0.00
104	->	99	0.01	0.00
104	->	100	0.01	0.00
104	->	101	0.00	0.01
104	->	102	-0.17	0.05
104	->	103	-33.18	0.98
104	->	104	-127.95	2.58
104	->	105	-33.94	0.91
104	->	106	-0.24	0.06
104	->	107	-0.01	0.01
105	->	1	-0.05	0.01
105	->	2	-0.00	0.00
105	->	3	0.00	0.00
105	->	4	-0.00	0.00
105	->	5	0.01	0.00
105	->	6	-0.00	0.00
105	->	7	0.00	0.00
105	->	8	0.01	0.00
105	->	9	0.00	0.00
105	->	10	0.02	0.00
105	->	11	0.02	0.01
105	->	12	-0.06	0.02
105	->	13	-0.01	0.00
105	->	14	-0.00	0.00
105	->	15	-0.00	0.00
105	->	16	-0.02	0.00
105	->	17	0.00	0.00
105	->	18	-0.00	0.00

105 ->	19	0.02	0.00
105 ->	20	-0.00	0.00
105 ->	21	-0.00	0.00
105 ->	22	-0.03	0.00
105 ->	23	-0.00	0.00
105 ->	24	-0.00	0.00
105 ->	25	-0.01	0.00
105 ->	26	-0.00	0.00
105 ->	27	-0.00	0.00
105 ->	28	-0.00	0.00
105 ->	29	-0.00	0.00
105 ->	30	0.00	0.00
105 ->	31	-0.01	0.00
105 ->	32	-0.00	0.00
105 ->	33	0.00	0.00
105 ->	34	-0.00	0.00
105 ->	35	0.01	0.00
105 ->	36	-0.00	0.00
105 ->	37	0.00	0.00
105 ->	38	0.01	0.00
105 ->	39	0.00	0.00
105 ->	40	-0.02	0.00
105 ->	41	0.00	0.00
105 ->	42	-0.00	0.00
105 ->	43	-0.00	0.00
105 ->	44	-0.04	0.00
105 ->	45	-0.00	0.00
105 ->	46	0.02	0.00
105 ->	47	-0.02	0.00
105 ->	48	-0.00	0.00
105 ->	49	-0.00	0.00
105 ->	50	-0.01	0.00
105 ->	51	-0.00	0.00
105 ->	52	-0.00	0.00
105 ->	53	-0.01	0.00
105 ->	54	-0.00	0.00
105 ->	55	-0.00	0.00
105 ->	56	-0.00	0.00
105 ->	57	-0.00	0.00
105 ->	58	0.01	0.00
105 ->	59	-0.01	0.00
105 ->	60	-0.00	0.00
105 ->	61	-0.00	0.00
105 ->	62	0.00	0.00
105 ->	63	0.01	0.00
105 ->	64	0.01	0.00
105 ->	65	0.00	0.00
105 ->	66	0.01	0.00
105 ->	67	-0.00	0.00
105 ->	68	-0.02	0.01

105	->	69	-0.01	0.00
105	->	70	-0.01	0.00
105	->	71	-0.00	0.00
105	->	72	0.46	0.25
105	->	73	-0.01	0.00
105	->	74	-0.82	0.60
105	->	75	-0.41	0.51
105	->	76	-0.03	0.01
105	->	77	-0.10	0.11
105	->	78	-1.55	1.25
105	->	79	-0.01	0.00
105	->	80	-0.01	0.00
105	->	81	-0.51	1.18
105	->	82	-0.01	0.01
105	->	83	-0.00	0.00
105	->	84	-0.00	0.00
105	->	85	-0.02	0.04
105	->	86	-0.02	0.01
105	->	87	-0.08	0.05
105	->	88	-0.29	0.21
105	->	89	-11.55	0.63
105	->	90	-1.03	0.54
105	->	91	-0.27	0.07
105	->	92	-0.03	0.01
105	->	93	0.01	0.00
105	->	94	0.01	0.00
105	->	95	0.01	0.00
105	->	96	0.00	0.00
105	->	97	0.00	0.00
105	->	98	0.01	0.00
105	->	99	0.01	0.00
105	->	100	0.01	0.00
105	->	101	0.01	0.00
105	->	102	0.00	0.00
105	->	103	-0.10	0.03
105	->	104	-33.70	0.91
105	->	105	-217.77	2.49
105	->	106	-32.96	1.21
105	->	107	-0.15	0.05
106	->	1	-0.03	0.01
106	->	2	-0.00	0.00
106	->	3	0.00	0.00
106	->	4	-0.00	0.00
106	->	5	0.01	0.00
106	->	6	-0.00	0.00
106	->	7	0.00	0.00
106	->	8	0.01	0.00
106	->	9	0.00	0.00
106	->	10	0.01	0.00
106	->	11	0.02	0.01

106	->	12	-0.04	0.01
106	->	13	-0.01	0.00
106	->	14	-0.00	0.00
106	->	15	-0.00	0.00
106	->	16	-0.01	0.00
106	->	17	0.00	0.00
106	->	18	0.00	0.00
106	->	19	0.02	0.00
106	->	20	-0.00	0.00
106	->	21	-0.00	0.00
106	->	22	-0.02	0.00
106	->	23	-0.00	0.00
106	->	24	-0.00	0.00
106	->	25	-0.01	0.00
106	->	26	-0.00	0.00
106	->	27	-0.00	0.00
106	->	28	-0.00	0.00
106	->	29	-0.00	0.00
106	->	30	0.00	0.00
106	->	31	-0.00	0.00
106	->	32	-0.00	0.00
106	->	33	-0.00	0.00
106	->	34	-0.00	0.00
106	->	35	0.00	0.00
106	->	36	-0.00	0.00
106	->	37	0.00	0.00
106	->	38	0.00	0.00
106	->	39	0.00	0.00
106	->	40	-0.01	0.00
106	->	41	-0.00	0.00
106	->	42	-0.00	0.00
106	->	43	-0.00	0.00
106	->	44	-0.02	0.00
106	->	45	-0.00	0.00
106	->	46	0.01	0.00
106	->	47	-0.01	0.00
106	->	48	-0.00	0.00
106	->	49	-0.00	0.00
106	->	50	-0.00	0.00
106	->	51	-0.00	0.00
106	->	52	-0.00	0.00
106	->	53	-0.01	0.00
106	->	54	-0.01	0.00
106	->	55	-0.00	0.00
106	->	56	-0.00	0.00
106	->	57	-0.00	0.00
106	->	58	0.00	0.00
106	->	59	-0.01	0.00
106	->	60	-0.00	0.00
106	->	61	-0.00	0.00

106	->	62	0.00	0.00
106	->	63	0.01	0.00
106	->	64	0.01	0.00
106	->	65	0.00	0.00
106	->	66	0.01	0.00
106	->	67	-0.00	0.00
106	->	68	-0.02	0.01
106	->	69	-0.01	0.00
106	->	70	-0.00	0.00
106	->	71	-0.00	0.00
106	->	72	-0.06	0.18
106	->	73	-0.00	0.00
106	->	74	0.05	0.05
106	->	75	0.21	0.24
106	->	76	-0.02	0.00
106	->	77	-0.04	0.01
106	->	78	-5.44	2.94
106	->	79	-0.01	0.00
106	->	80	-0.00	0.00
106	->	81	-0.06	0.02
106	->	82	-0.01	0.01
106	->	83	-0.00	0.00
106	->	84	-0.00	0.00
106	->	85	-0.01	0.02
106	->	86	-0.18	0.06
106	->	87	-1.03	0.59
106	->	88	-11.29	1.26
106	->	89	-5.08	1.04
106	->	90	-0.30	0.11
106	->	91	-0.04	0.03
106	->	92	0.02	0.00
106	->	93	0.02	0.00
106	->	94	0.01	0.00
106	->	95	0.01	0.00
106	->	96	0.00	0.00
106	->	97	0.00	0.00
106	->	98	0.00	0.00
106	->	99	0.01	0.00
106	->	100	0.00	0.00
106	->	101	0.01	0.00
106	->	102	0.01	0.00
106	->	103	0.01	0.01
106	->	104	-0.24	0.06
106	->	105	-32.68	1.20
106	->	106	-180.93	3.50
106	->	107	-35.73	0.84
107	->	1	-0.01	0.00
107	->	2	-0.00	0.00
107	->	3	0.00	0.00
107	->	4	-0.00	0.00

107	->	5	0.01	0.00
107	->	6	-0.00	0.00
107	->	7	0.00	0.00
107	->	8	0.00	0.00
107	->	9	0.00	0.00
107	->	10	0.01	0.00
107	->	11	0.01	0.00
107	->	12	-0.02	0.01
107	->	13	-0.00	0.00
107	->	14	-0.00	0.00
107	->	15	-0.00	0.00
107	->	16	-0.01	0.00
107	->	17	0.00	0.00
107	->	18	0.00	0.00
107	->	19	0.01	0.00
107	->	20	0.00	0.00
107	->	21	0.00	0.00
107	->	22	-0.01	0.00
107	->	23	-0.00	0.00
107	->	24	0.00	0.00
107	->	25	-0.01	0.00
107	->	26	-0.00	0.00
107	->	27	-0.00	0.00
107	->	28	-0.00	0.00
107	->	29	-0.00	0.00
107	->	30	0.00	0.00
107	->	31	-0.01	0.00
107	->	32	-0.00	0.00
107	->	33	0.00	0.00
107	->	34	-0.00	0.00
107	->	35	0.01	0.00
107	->	36	-0.00	0.00
107	->	37	0.00	0.00
107	->	38	0.01	0.00
107	->	39	-0.00	0.00
107	->	40	-0.03	0.00
107	->	41	-0.00	0.00
107	->	42	-0.00	0.00
107	->	43	-0.00	0.00
107	->	44	-0.01	0.00
107	->	45	0.00	0.00
107	->	46	0.01	0.00
107	->	47	-0.00	0.00
107	->	48	-0.00	0.00
107	->	49	-0.00	0.00
107	->	50	-0.00	0.00
107	->	51	-0.00	0.00
107	->	52	-0.00	0.00
107	->	53	-0.01	0.00
107	->	54	-0.00	0.00

107	->	55	-0.00	0.00
107	->	56	-0.00	0.00
107	->	57	-0.00	0.00
107	->	58	0.00	0.00
107	->	59	-0.01	0.00
107	->	60	-0.00	0.00
107	->	61	-0.00	0.00
107	->	62	0.00	0.00
107	->	63	0.00	0.00
107	->	64	0.00	0.00
107	->	65	0.00	0.00
107	->	66	0.00	0.00
107	->	67	0.00	0.00
107	->	68	-0.01	0.00
107	->	69	-0.00	0.00
107	->	70	-0.00	0.00
107	->	71	-0.00	0.00
107	->	72	-0.05	0.01
107	->	73	-0.00	0.00
107	->	74	0.01	0.00
107	->	75	-0.00	0.02
107	->	76	-0.01	0.00
107	->	77	-0.01	0.00
107	->	78	-0.21	0.32
107	->	79	-0.00	0.00
107	->	80	-0.00	0.00
107	->	81	-0.01	0.00
107	->	82	-0.01	0.01
107	->	83	-0.00	0.00
107	->	84	-0.00	0.00
107	->	85	-0.00	0.00
107	->	86	-2.12	1.14
107	->	87	-10.45	0.68
107	->	88	0.09	0.13
107	->	89	-0.26	0.06
107	->	90	-0.03	0.01
107	->	91	0.01	0.01
107	->	92	0.01	0.00
107	->	93	0.01	0.00
107	->	94	0.00	0.00
107	->	95	0.00	0.00
107	->	96	0.00	0.00
107	->	97	0.00	0.00
107	->	98	0.00	0.00
107	->	99	0.00	0.00
107	->	100	0.00	0.00
107	->	101	0.00	0.00
107	->	102	0.00	0.00
107	->	103	0.00	0.00
107	->	104	-0.01	0.01

107 ->	105	-0.15	0.05
107 ->	106	-35.48	0.84
107 ->	107	-216.34	3.94
