# Supplementary material for: Investigating dynamic and energetic determinants of protein nucleic acid recognition: analysis of the zinc finger zif268-DNA complexes
Source: BMC Struct Biol. 2010 Nov 24;10:42. doi: 10.1186/1472-6807-10-42 (PMC3002361; doi:10.1186/1472-6807-10-42)
Supplement: Additional file 7 — Average pair interaction energies for 1A1L Complex and their standard deviations. [file 1472-6807-10-42-S7.PDF]

=>> 1A1L

Residue ->	Residue	TGBTOT	SD
1 ->	1	-239.30	7.07
1 ->	2	-0.86	0.37
1 ->	3	-1.18	0.19
1 ->	4	-0.13	0.03
1 ->	5	-0.13	0.02
1 ->	6	-0.00	0.00
1 ->	7	0.00	0.00
1 ->	8	-0.08	0.03
1 ->	9	-0.01	0.00
1 ->	10	-0.09	0.01
1 ->	11	-0.14	0.03
1 ->	12	1.10	0.48
1 ->	13	-3.85	1.59
1 ->	14	-3.04	1.10
1 ->	15	-4.18	1.64
1 ->	16	2.82	1.98
1 ->	17	-0.07	0.04
1 ->	18	-0.14	0.11
1 ->	19	-11.46	7.39
1 ->	20	-0.13	0.07
1 ->	21	0.01	0.02
1 ->	22	0.36	0.24
1 ->	23	0.02	0.03
1 ->	24	-0.01	0.01
1 ->	25	0.05	0.01
1 ->	26	0.00	0.00
1 ->	27	-0.01	0.00
1 ->	28	0.00	0.00
1 ->	29	0.00	0.00
1 ->	30	-0.00	0.00
1 ->	31	0.02	0.00
1 ->	32	0.00	0.00
1 ->	33	0.00	0.00
1 ->	34	0.00	0.00
1 ->	35	-0.01	0.00
1 ->	36	0.01	0.00
1 ->	37	-0.00	0.00
1 ->	38	-0.01	0.00
1 ->	39	-0.00	0.00
1 ->	40	0.01	0.00
1 ->	41	0.00	0.00
1 ->	42	0.00	0.00
1 ->	43	0.00	0.00
1 ->	44	0.09	0.04
1 ->	45	0.00	0.00
1 ->	46	-0.04	0.01

1 ->	47	0.00	0.00
1 ->	48	0.00	0.00
1 ->	49	0.00	0.00
1 ->	50	0.00	0.00
1 ->	51	0.00	0.00
1 ->	52	0.00	0.00
1 ->	53	0.02	0.00
1 ->	54	0.00	0.00
1 ->	55	0.00	0.00
1 ->	56	0.00	0.00
1 ->	57	0.00	0.00
1 ->	58	-0.01	0.00
1 ->	59	0.01	0.00
1 ->	60	0.00	0.00
1 ->	61	0.00	0.00
1 ->	62	-0.00	0.00
1 ->	63	-0.01	0.00
1 ->	64	-0.01	0.00
1 ->	65	-0.00	0.00
1 ->	66	-0.00	0.00
1 ->	67	0.00	0.00
1 ->	68	0.01	0.00
1 ->	69	0.00	0.00
1 ->	70	0.00	0.00
1 ->	71	-0.00	0.00
1 ->	72	0.03	0.01
1 ->	73	0.00	0.00
1 ->	74	-0.03	0.00
1 ->	75	-0.01	0.00
1 ->	76	0.01	0.00
1 ->	77	0.03	0.01
1 ->	78	0.02	0.00
1 ->	79	0.00	0.00
1 ->	80	0.00	0.00
1 ->	81	0.02	0.01
1 ->	82	0.00	0.00
1 ->	83	0.00	0.00
1 ->	84	0.00	0.00
1 ->	85	0.00	0.00
1 ->	86	-0.00	0.00
1 ->	87	-0.01	0.00
1 ->	88	-0.01	0.00
1 ->	89	-0.01	0.00
1 ->	90	-0.03	0.00
1 ->	91	-0.08	0.02
1 ->	92	-2.20	0.63
1 ->	93	-14.55	2.62
1 ->	94	-6.07	4.19
1 ->	95	-0.13	0.03
1 ->	96	-0.03	0.01

1	->	97	-0.01	0.00
1	->	98	-0.03	0.01
1	->	99	-0.03	0.00
1	->	100	-0.01	0.02
1	->	101	-0.04	0.01
1	->	102	-0.07	0.02
1	->	103	-0.07	0.02
1	->	104	-0.05	0.01
1	->	105	-0.04	0.00
1	->	106	-0.02	0.00
1	->	107	-0.01	0.00
2	->	1	-0.87	0.37
2	->	2	2.11	0.78
2	->	3	-13.02	0.54
2	->	4	-0.30	0.08
2	->	5	-0.01	0.01
2	->	6	-0.00	0.00
2	->	7	-0.00	0.00
2	->	8	0.00	0.01
2	->	9	-0.00	0.00
2	->	10	-0.00	0.00
2	->	11	-0.00	0.00
2	->	12	-0.02	0.01
2	->	13	-2.52	1.22
2	->	14	-1.14	0.26
2	->	15	-1.05	0.25
2	->	16	-0.09	0.06
2	->	17	-0.02	0.02
2	->	18	-0.01	0.00
2	->	19	-0.04	0.02
2	->	20	-0.03	0.01
2	->	21	-0.00	0.00
2	->	22	-0.00	0.00
2	->	23	-0.00	0.00
2	->	24	-0.00	0.00
2	->	25	-0.00	0.00
2	->	26	-0.00	0.00
2	->	27	-0.00	0.00
2	->	28	-0.00	0.00
2	->	29	-0.00	0.00
2	->	30	-0.00	0.00
2	->	31	-0.00	0.00
2	->	32	0.00	0.00
2	->	33	0.00	0.00
2	->	34	0.00	0.00
2	->	35	-0.00	0.00
2	->	36	0.00	0.00
2	->	37	0.00	0.00
2	->	38	-0.00	0.00
2	->	39	0.00	0.00

2 ->	40	-0.00	0.00
2 ->	41	0.00	0.00
2 ->	42	0.00	0.00
2 ->	43	-0.00	0.00
2 ->	44	0.00	0.00
2 ->	45	0.00	0.00
2 ->	46	-0.00	0.00
2 ->	47	0.00	0.00
2 ->	48	0.00	0.00
2 ->	49	0.00	0.00
2 ->	50	0.00	0.00
2 ->	51	0.00	0.00
2 ->	52	0.00	0.00
2 ->	53	0.00	0.00
2 ->	54	0.00	0.00
2 ->	55	0.00	0.00
2 ->	56	0.00	0.00
2 ->	57	0.00	0.00
2 ->	58	-0.00	0.00
2 ->	59	0.00	0.00
2 ->	60	0.00	0.00
2 ->	61	0.00	0.00
2 ->	62	0.00	0.00
2 ->	63	-0.00	0.00
2 ->	64	-0.00	0.00
2 ->	65	-0.00	0.00
2 ->	66	-0.00	0.00
2 ->	67	0.00	0.00
2 ->	68	0.00	0.00
2 ->	69	0.00	0.00
2 ->	70	0.00	0.00
2 ->	71	0.00	0.00
2 ->	72	0.00	0.00
2 ->	73	0.00	0.00
2 ->	74	-0.00	0.00
2 ->	75	-0.00	0.00
2 ->	76	0.00	0.00
2 ->	77	0.00	0.00
2 ->	78	0.00	0.00
2 ->	79	0.00	0.00
2 ->	80	0.00	0.00
2 ->	81	0.00	0.00
2 ->	82	0.00	0.00
2 ->	83	0.00	0.00
2 ->	84	0.00	0.00
2 ->	85	-0.00	0.00
2 ->	86	-0.00	0.00
2 ->	87	-0.00	0.00
2 ->	88	-0.00	0.00
2 ->	89	-0.00	0.00

2	->	90	-0.00	0.00
2	->	91	-0.00	0.00
2	->	92	-0.00	0.00
2	->	93	-0.01	0.01
2	->	94	-0.01	0.01
2	->	95	-0.01	0.00
2	->	96	-0.00	0.00
2	->	97	-0.00	0.00
2	->	98	-0.00	0.00
2	->	99	-0.00	0.00
2	->	100	-0.00	0.00
2	->	101	-0.00	0.00
2	->	102	-0.00	0.00
2	->	103	-0.00	0.00
2	->	104	-0.00	0.00
2	->	105	0.00	0.00
2	->	106	0.00	0.00
2	->	107	0.00	0.00
3	->	1	-1.18	0.19
3	->	2	-13.11	0.53
3	->	3	18.54	1.28
3	->	4	-9.93	0.53
3	->	5	-0.51	0.07
3	->	6	-0.03	0.00
3	->	7	0.01	0.01
3	->	8	-0.06	0.06
3	->	9	-0.01	0.00
3	->	10	-0.02	0.01
3	->	11	-0.01	0.00
3	->	12	-0.22	0.06
3	->	13	-3.45	0.39
3	->	14	-3.98	0.48
3	->	15	-1.48	0.36
3	->	16	-0.98	0.79
3	->	17	-0.68	0.81
3	->	18	-0.02	0.01
3	->	19	-0.09	0.06
3	->	20	-1.28	0.57
3	->	21	-0.03	0.01
3	->	22	-0.01	0.01
3	->	23	-0.03	0.01
3	->	24	-0.02	0.01
3	->	25	-0.00	0.00
3	->	26	-0.00	0.00
3	->	27	-0.01	0.00
3	->	28	-0.00	0.00
3	->	29	-0.00	0.00
3	->	30	-0.00	0.00
3	->	31	-0.00	0.00
3	->	32	-0.00	0.00

3 ->	33	-0.00	0.00
3 ->	34	-0.00	0.00
3 ->	35	-0.00	0.00
3 ->	36	0.00	0.00
3 ->	37	0.00	0.00
3 ->	38	0.00	0.00
3 ->	39	0.00	0.00
3 ->	40	-0.00	0.00
3 ->	41	0.00	0.00
3 ->	42	0.00	0.00
3 ->	43	-0.00	0.00
3 ->	44	-0.00	0.00
3 ->	45	-0.00	0.00
3 ->	46	0.00	0.00
3 ->	47	-0.00	0.00
3 ->	48	-0.00	0.00
3 ->	49	-0.00	0.00
3 ->	50	-0.00	0.00
3 ->	51	-0.00	0.00
3 ->	52	-0.00	0.00
3 ->	53	-0.00	0.00
3 ->	54	0.00	0.00
3 ->	55	-0.00	0.00
3 ->	56	0.00	0.00
3 ->	57	0.00	0.00
3 ->	58	-0.00	0.00
3 ->	59	0.00	0.00
3 ->	60	0.00	0.00
3 ->	61	0.00	0.00
3 ->	62	-0.00	0.00
3 ->	63	0.00	0.00
3 ->	64	0.00	0.00
3 ->	65	0.00	0.00
3 ->	66	0.00	0.00
3 ->	67	0.00	0.00
3 ->	68	-0.00	0.00
3 ->	69	0.00	0.00
3 ->	70	-0.00	0.00
3 ->	71	-0.00	0.00
3 ->	72	-0.00	0.00
3 ->	73	0.00	0.00
3 ->	74	0.00	0.00
3 ->	75	0.00	0.00
3 ->	76	-0.00	0.00
3 ->	77	-0.00	0.00
3 ->	78	-0.00	0.00
3 ->	79	-0.00	0.00
3 ->	80	-0.00	0.00
3 ->	81	-0.00	0.00
3 ->	82	0.00	0.00

3 ->	83	0.00	0.00
3 ->	84	0.00	0.00
3 ->	85	-0.00	0.00
3 ->	86	0.00	0.00
3 ->	87	0.00	0.00
3 ->	88	0.00	0.00
3 ->	89	0.00	0.00
3 ->	90	-0.00	0.00
3 ->	91	-0.00	0.00
3 ->	92	-0.00	0.01
3 ->	93	0.02	0.03
3 ->	94	-0.01	0.01
3 ->	95	-0.01	0.00
3 ->	96	-0.01	0.01
3 ->	97	-0.01	0.01
3 ->	98	-0.01	0.01
3 ->	99	-0.00	0.00
3 ->	100	-0.00	0.00
3 ->	101	-0.00	0.00
3 ->	102	-0.00	0.00
3 ->	103	-0.00	0.00
3 ->	104	0.00	0.00
3 ->	105	0.00	0.00
3 ->	106	0.00	0.00
3 ->	107	0.00	0.00
4 ->	1	-0.13	0.03
4 ->	2	-0.31	0.09
4 ->	3	-9.89	0.53
4 ->	4	22.70	1.15
4 ->	5	-12.69	0.49
4 ->	6	-0.32	0.06
4 ->	7	-0.01	0.13
4 ->	8	-0.68	0.48
4 ->	9	-0.03	0.02
4 ->	10	-0.15	0.06
4 ->	11	-0.02	0.01
4 ->	12	-0.12	0.06
4 ->	13	-2.10	0.50
4 ->	14	-0.89	0.36
4 ->	15	0.00	0.01
4 ->	16	-0.01	0.00
4 ->	17	-0.01	0.01
4 ->	18	-0.00	0.00
4 ->	19	0.00	0.01
4 ->	20	-0.98	0.31
4 ->	21	-0.02	0.01
4 ->	22	0.01	0.01
4 ->	23	-0.17	0.08
4 ->	24	-0.06	0.03
4 ->	25	-0.00	0.00

4	->	26	-0.00	0.00
4	->	27	-0.08	0.02
4	->	28	-0.00	0.00
4	->	29	0.00	0.00
4	->	30	-0.00	0.00
4	->	31	0.00	0.00
4	->	32	-0.00	0.00
4	->	33	-0.00	0.00
4	->	34	0.00	0.00
4	->	35	0.00	0.00
4	->	36	0.00	0.00
4	->	37	-0.00	0.00
4	->	38	-0.00	0.00
4	->	39	0.00	0.00
4	->	40	0.00	0.00
4	->	41	0.00	0.00
4	->	42	0.00	0.00
4	->	43	0.00	0.00
4	->	44	-0.00	0.00
4	->	45	-0.00	0.00
4	->	46	-0.00	0.00
4	->	47	0.00	0.00
4	->	48	0.00	0.00
4	->	49	0.00	0.00
4	->	50	-0.00	0.00
4	->	51	0.00	0.00
4	->	52	0.00	0.00
4	->	53	-0.00	0.00
4	->	54	0.00	0.00
4	->	55	0.00	0.00
4	->	56	0.00	0.00
4	->	57	0.00	0.00
4	->	58	-0.00	0.00
4	->	59	0.00	0.00
4	->	60	0.00	0.00
4	->	61	0.00	0.00
4	->	62	0.00	0.00
4	->	63	-0.00	0.00
4	->	64	-0.00	0.00
4	->	65	0.00	0.00
4	->	66	-0.00	0.00
4	->	67	0.00	0.00
4	->	68	0.00	0.00
4	->	69	0.00	0.00
4	->	70	0.00	0.00
4	->	71	0.00	0.00
4	->	72	0.00	0.00
4	->	73	0.00	0.00
4	->	74	-0.00	0.00
4	->	75	-0.00	0.00

4	->	76	0.00	0.00
4	->	77	0.00	0.00
4	->	78	0.00	0.00
4	->	79	0.00	0.00
4	->	80	0.00	0.00
4	->	81	0.00	0.00
4	->	82	0.00	0.00
4	->	83	0.00	0.00
4	->	84	0.00	0.00
4	->	85	0.00	0.00
4	->	86	0.00	0.00
4	->	87	-0.00	0.00
4	->	88	-0.00	0.00
4	->	89	-0.00	0.00
4	->	90	-0.00	0.00
4	->	91	-0.01	0.00
4	->	92	-0.02	0.01
4	->	93	-0.01	0.00
4	->	94	-0.00	0.00
4	->	95	-0.00	0.00
4	->	96	-0.00	0.00
4	->	97	-0.00	0.00
4	->	98	0.00	0.00
4	->	99	0.00	0.00
4	->	100	0.00	0.00
4	->	101	0.00	0.00
4	->	102	-0.00	0.00
4	->	103	-0.00	0.00
4	->	104	-0.00	0.00
4	->	105	-0.00	0.00
4	->	106	-0.00	0.00
4	->	107	-0.00	0.00
5	->	1	-0.13	0.02
5	->	2	-0.01	0.01
5	->	3	-0.51	0.07
5	->	4	-12.74	0.50
5	->	5	-29.21	3.08
5	->	6	-1.62	0.64
5	->	7	-6.34	1.25
5	->	8	-0.00	0.22
5	->	9	-0.03	0.06
5	->	10	1.74	0.68
5	->	11	0.07	0.01
5	->	12	-0.11	0.03
5	->	13	-0.35	0.08
5	->	14	-0.48	0.23
5	->	15	0.01	0.01
5	->	16	-0.03	0.00
5	->	17	-0.00	0.00
5	->	18	0.03	0.00

5 ->	19	0.01	0.01
5 ->	20	-1.06	0.24
5 ->	21	-0.01	0.01
5 ->	22	-0.14	0.04
5 ->	23	1.27	0.35
5 ->	24	-0.69	0.25
5 ->	25	-0.06	0.01
5 ->	26	-0.06	0.03
5 ->	27	2.07	1.17
5 ->	28	-0.02	0.01
5 ->	29	-0.00	0.00
5 ->	30	-0.01	0.00
5 ->	31	-0.02	0.00
5 ->	32	-0.00	0.00
5 ->	33	-0.00	0.00
5 ->	34	-0.00	0.00
5 ->	35	0.00	0.00
5 ->	36	-0.00	0.00
5 ->	37	0.00	0.00
5 ->	38	0.00	0.00
5 ->	39	0.00	0.00
5 ->	40	-0.01	0.00
5 ->	41	-0.00	0.00
5 ->	42	-0.00	0.00
5 ->	43	-0.00	0.00
5 ->	44	-0.02	0.00
5 ->	45	-0.00	0.00
5 ->	46	0.01	0.00
5 ->	47	-0.00	0.00
5 ->	48	-0.00	0.00
5 ->	49	-0.00	0.00
5 ->	50	-0.00	0.00
5 ->	51	-0.00	0.00
5 ->	52	-0.00	0.00
5 ->	53	-0.00	0.00
5 ->	54	-0.00	0.00
5 ->	55	0.00	0.00
5 ->	56	-0.00	0.00
5 ->	57	-0.00	0.00
5 ->	58	0.00	0.00
5 ->	59	-0.00	0.00
5 ->	60	-0.00	0.00
5 ->	61	-0.00	0.00
5 ->	62	0.00	0.00
5 ->	63	0.00	0.00
5 ->	64	0.00	0.00
5 ->	65	0.00	0.00
5 ->	66	0.00	0.00
5 ->	67	-0.00	0.00
5 ->	68	-0.00	0.00

5 ->	69	-0.00	0.00
5 ->	70	-0.00	0.00
5 ->	71	0.00	0.00
5 ->	72	-0.01	0.00
5 ->	73	-0.00	0.00
5 ->	74	0.01	0.00
5 ->	75	0.00	0.00
5 ->	76	-0.00	0.00
5 ->	77	-0.00	0.00
5 ->	78	-0.00	0.00
5 ->	79	-0.00	0.00
5 ->	80	-0.00	0.00
5 ->	81	-0.00	0.00
5 ->	82	-0.00	0.00
5 ->	83	-0.00	0.00
5 ->	84	-0.00	0.00
5 ->	85	-0.00	0.00
5 ->	86	0.00	0.00
5 ->	87	0.00	0.00
5 ->	88	0.00	0.00
5 ->	89	0.01	0.00
5 ->	90	0.02	0.00
5 ->	91	0.05	0.01
5 ->	92	0.10	0.04
5 ->	93	0.05	0.01
5 ->	94	0.02	0.00
5 ->	95	0.01	0.00
5 ->	96	0.00	0.00
5 ->	97	0.00	0.00
5 ->	98	0.01	0.00
5 ->	99	0.01	0.00
5 ->	100	0.01	0.00
5 ->	101	0.01	0.00
5 ->	102	0.01	0.00
5 ->	103	0.01	0.00
5 ->	104	0.01	0.00
5 ->	105	0.01	0.00
5 ->	106	0.01	0.00
5 ->	107	0.01	0.00
6 ->	1	-0.00	0.00
6 ->	2	-0.00	0.00
6 ->	3	-0.03	0.00
6 ->	4	-0.32	0.06
6 ->	5	-1.72	0.64
6 ->	6	0.30	0.45
6 ->	7	-13.06	0.40
6 ->	8	-0.49	0.16
6 ->	9	-0.03	0.01
6 ->	10	-0.05	0.03
6 ->	11	-0.00	0.00

6 ->	12	-0.01	0.00
6 ->	13	-0.02	0.00
6 ->	14	-0.02	0.01
6 ->	15	-0.00	0.00
6 ->	16	-0.00	0.00
6 ->	17	-0.00	0.00
6 ->	18	-0.01	0.00
6 ->	19	-0.01	0.00
6 ->	20	-0.21	0.14
6 ->	21	-0.01	0.01
6 ->	22	-0.00	0.00
6 ->	23	-0.08	0.03
6 ->	24	-0.93	0.35
6 ->	25	-0.01	0.01
6 ->	26	-0.01	0.00
6 ->	27	-0.24	0.19
6 ->	28	-0.03	0.04
6 ->	29	-0.00	0.00
6 ->	30	-0.00	0.00
6 ->	31	0.00	0.00
6 ->	32	-0.00	0.00
6 ->	33	-0.00	0.00
6 ->	34	0.00	0.00
6 ->	35	-0.00	0.00
6 ->	36	0.00	0.00
6 ->	37	0.00	0.00
6 ->	38	-0.00	0.00
6 ->	39	0.00	0.00
6 ->	40	0.00	0.00
6 ->	41	0.00	0.00
6 ->	42	-0.00	0.00
6 ->	43	-0.00	0.00
6 ->	44	0.00	0.00
6 ->	45	0.00	0.00
6 ->	46	-0.00	0.00
6 ->	47	0.00	0.00
6 ->	48	0.00	0.00
6 ->	49	0.00	0.00
6 ->	50	0.00	0.00
6 ->	51	0.00	0.00
6 ->	52	0.00	0.00
6 ->	53	0.00	0.00
6 ->	54	0.00	0.00
6 ->	55	0.00	0.00
6 ->	56	0.00	0.00
6 ->	57	0.00	0.00
6 ->	58	-0.00	0.00
6 ->	59	0.00	0.00
6 ->	60	0.00	0.00
6 ->	61	0.00	0.00

6	->	62	0.00	0.00
6	->	63	-0.00	0.00
6	->	64	0.00	0.00
6	->	65	0.00	0.00
6	->	66	-0.00	0.00
6	->	67	0.00	0.00
6	->	68	0.00	0.00
6	->	69	0.00	0.00
6	->	70	0.00	0.00
6	->	71	-0.00	0.00
6	->	72	0.00	0.00
6	->	73	0.00	0.00
6	->	74	-0.00	0.00
6	->	75	-0.00	0.00
6	->	76	0.00	0.00
6	->	77	0.00	0.00
6	->	78	0.00	0.00
6	->	79	0.00	0.00
6	->	80	0.00	0.00
6	->	81	0.00	0.00
6	->	82	0.00	0.00
6	->	83	0.00	0.00
6	->	84	0.00	0.00
6	->	85	0.00	0.00
6	->	86	0.00	0.00
6	->	87	-0.00	0.00
6	->	88	-0.00	0.00
6	->	89	-0.00	0.00
6	->	90	-0.00	0.00
6	->	91	-0.00	0.00
6	->	92	-0.01	0.00
6	->	93	-0.00	0.00
6	->	94	-0.00	0.00
6	->	95	-0.00	0.00
6	->	96	-0.00	0.00
6	->	97	-0.00	0.00
6	->	98	-0.00	0.00
6	->	99	-0.00	0.00
6	->	100	-0.00	0.00
6	->	101	-0.00	0.00
6	->	102	-0.00	0.00
6	->	103	-0.00	0.00
6	->	104	-0.00	0.00
6	->	105	-0.00	0.00
6	->	106	-0.00	0.00
6	->	107	-0.00	0.00
7	->	1	0.00	0.00
7	->	2	-0.00	0.00
7	->	3	0.01	0.01
7	->	4	-0.01	0.13

7 ->	5	-6.44	1.24
7 ->	6	-13.20	0.40
7 ->	7	25.98	1.47
7 ->	8	-11.37	0.52
7 ->	9	-1.36	0.26
7 ->	10	-1.39	0.79
7 ->	11	-0.04	0.02
7 ->	12	-0.01	0.00
7 ->	13	-0.01	0.02
7 ->	14	-0.04	0.01
7 ->	15	-0.00	0.00
7 ->	16	-0.00	0.00
7 ->	17	-0.00	0.00
7 ->	18	-0.00	0.00
7 ->	19	0.00	0.00
7 ->	20	-0.02	0.01
7 ->	21	-0.01	0.00
7 ->	22	0.01	0.01
7 ->	23	-0.28	0.16
7 ->	24	-0.06	0.03
7 ->	25	-0.00	0.00
7 ->	26	-0.00	0.01
7 ->	27	-1.23	1.33
7 ->	28	-0.02	0.02
7 ->	29	-0.00	0.00
7 ->	30	-0.00	0.00
7 ->	31	-0.00	0.00
7 ->	32	-0.00	0.00
7 ->	33	0.00	0.00
7 ->	34	0.00	0.00
7 ->	35	-0.00	0.00
7 ->	36	0.00	0.00
7 ->	37	0.00	0.00
7 ->	38	0.00	0.00
7 ->	39	0.00	0.00
7 ->	40	-0.00	0.00
7 ->	41	0.00	0.00
7 ->	42	0.00	0.00
7 ->	43	-0.00	0.00
7 ->	44	-0.00	0.00
7 ->	45	0.00	0.00
7 ->	46	-0.00	0.00
7 ->	47	0.00	0.00
7 ->	48	0.00	0.00
7 ->	49	0.00	0.00
7 ->	50	0.00	0.00
7 ->	51	0.00	0.00
7 ->	52	0.00	0.00
7 ->	53	0.00	0.00
7 ->	54	0.00	0.00

7 ->	55	0.00	0.00
7 ->	56	0.00	0.00
7 ->	57	0.00	0.00
7 ->	58	-0.00	0.00
7 ->	59	0.00	0.00
7 ->	60	0.00	0.00
7 ->	61	0.00	0.00
7 ->	62	0.00	0.00
7 ->	63	0.00	0.00
7 ->	64	0.00	0.00
7 ->	65	0.00	0.00
7 ->	66	0.00	0.00
7 ->	67	0.00	0.00
7 ->	68	0.00	0.00
7 ->	69	0.00	0.00
7 ->	70	0.00	0.00
7 ->	71	0.00	0.00
7 ->	72	-0.00	0.00
7 ->	73	0.00	0.00
7 ->	74	0.00	0.00
7 ->	75	0.00	0.00
7 ->	76	0.00	0.00
7 ->	77	0.00	0.00
7 ->	78	-0.00	0.00
7 ->	79	0.00	0.00
7 ->	80	0.00	0.00
7 ->	81	-0.00	0.00
7 ->	82	0.00	0.00
7 ->	83	0.00	0.00
7 ->	84	0.00	0.00
7 ->	85	-0.00	0.00
7 ->	86	0.00	0.00
7 ->	87	0.00	0.00
7 ->	88	0.00	0.00
7 ->	89	0.00	0.00
7 ->	90	-0.00	0.00
7 ->	91	-0.01	0.01
7 ->	92	-0.01	0.01
7 ->	93	-0.00	0.00
7 ->	94	-0.00	0.00
7 ->	95	-0.00	0.00
7 ->	96	-0.00	0.00
7 ->	97	-0.00	0.00
7 ->	98	-0.00	0.00
7 ->	99	-0.00	0.00
7 ->	100	-0.00	0.00
7 ->	101	0.00	0.00
7 ->	102	-0.00	0.00
7 ->	103	0.00	0.00
7 ->	104	0.00	0.00

7 ->	105	0.00	0.00
7 ->	106	0.00	0.00
7 ->	107	-0.00	0.00
8 ->	1	-0.08	0.03
8 ->	2	0.00	0.01
8 ->	3	-0.06	0.06
8 ->	4	-0.70	0.49
8 ->	5	-0.02	0.22
8 ->	6	-0.50	0.17
8 ->	7	-11.39	0.51
8 ->	8	-68.04	3.44
8 ->	9	-18.92	1.06
8 ->	10	-0.54	0.19
8 ->	11	0.01	0.02
8 ->	12	-0.07	0.02
8 ->	13	-2.61	2.77
8 ->	14	-0.02	0.01
8 ->	15	-0.00	0.00
8 ->	16	-0.02	0.01
8 ->	17	0.00	0.00
8 ->	18	0.01	0.00
8 ->	19	0.02	0.01
8 ->	20	-0.01	0.01
8 ->	21	-0.00	0.00
8 ->	22	-0.02	0.00
8 ->	23	-0.01	0.02
8 ->	24	-0.01	0.00
8 ->	25	-0.02	0.00
8 ->	26	-0.00	0.00
8 ->	27	0.01	0.02
8 ->	28	-0.00	0.00
8 ->	29	-0.00	0.00
8 ->	30	-0.00	0.00
8 ->	31	-0.01	0.00
8 ->	32	-0.00	0.00
8 ->	33	0.00	0.00
8 ->	34	-0.00	0.00
8 ->	35	0.00	0.00
8 ->	36	-0.00	0.00
8 ->	37	0.00	0.00
8 ->	38	0.00	0.00
8 ->	39	0.00	0.00
8 ->	40	-0.00	0.00
8 ->	41	-0.00	0.00
8 ->	42	-0.00	0.00
8 ->	43	-0.00	0.00
8 ->	44	-0.01	0.00
8 ->	45	-0.00	0.00
8 ->	46	0.01	0.00
8 ->	47	-0.00	0.00

8 ->	48	-0.00	0.00
8 ->	49	-0.00	0.00
8 ->	50	-0.00	0.00
8 ->	51	-0.00	0.00
8 ->	52	-0.00	0.00
8 ->	53	-0.00	0.00
8 ->	54	-0.00	0.00
8 ->	55	0.00	0.00
8 ->	56	-0.00	0.00
8 ->	57	-0.00	0.00
8 ->	58	0.00	0.00
8 ->	59	-0.00	0.00
8 ->	60	-0.00	0.00
8 ->	61	0.00	0.00
8 ->	62	0.00	0.00
8 ->	63	0.00	0.00
8 ->	64	0.00	0.00
8 ->	65	0.00	0.00
8 ->	66	0.00	0.00
8 ->	67	0.00	0.00
8 ->	68	-0.00	0.00
8 ->	69	-0.00	0.00
8 ->	70	-0.00	0.00
8 ->	71	-0.00	0.00
8 ->	72	-0.00	0.00
8 ->	73	-0.00	0.00
8 ->	74	0.00	0.00
8 ->	75	0.00	0.00
8 ->	76	-0.00	0.00
8 ->	77	-0.00	0.00
8 ->	78	-0.00	0.00
8 ->	79	-0.00	0.00
8 ->	80	-0.00	0.00
8 ->	81	-0.00	0.00
8 ->	82	-0.00	0.00
8 ->	83	-0.00	0.00
8 ->	84	0.00	0.00
8 ->	85	-0.00	0.00
8 ->	86	0.00	0.00
8 ->	87	0.00	0.00
8 ->	88	0.00	0.00
8 ->	89	0.00	0.00
8 ->	90	0.01	0.00
8 ->	91	0.01	0.00
8 ->	92	0.03	0.00
8 ->	93	0.03	0.01
8 ->	94	0.01	0.00
8 ->	95	0.01	0.00
8 ->	96	0.00	0.00
8 ->	97	0.00	0.00

8	->	98	0.01	0.00
8	->	99	0.00	0.00
8	->	100	0.00	0.00
8	->	101	0.00	0.00
8	->	102	0.00	0.00
8	->	103	0.01	0.00
8	->	104	0.01	0.00
8	->	105	0.01	0.00
8	->	106	0.01	0.00
8	->	107	0.00	0.00
9	->	1	-0.01	0.00
9	->	2	-0.00	0.00
9	->	3	-0.01	0.00
9	->	4	-0.03	0.02
9	->	5	-0.03	0.06
9	->	6	-0.03	0.01
9	->	7	-1.38	0.26
9	->	8	-19.02	1.08
9	->	9	14.08	1.95
9	->	10	-9.81	0.68
9	->	11	-0.31	0.07
9	->	12	-0.04	0.01
9	->	13	-0.06	0.05
9	->	14	-0.01	0.00
9	->	15	-0.00	0.00
9	->	16	-0.00	0.00
9	->	17	0.00	0.00
9	->	18	0.00	0.00
9	->	19	-0.00	0.00
9	->	20	-0.00	0.00
9	->	21	-0.00	0.00
9	->	22	-0.00	0.00
9	->	23	-0.03	0.01
9	->	24	-0.00	0.00
9	->	25	-0.00	0.00
9	->	26	-0.00	0.00
9	->	27	-0.04	0.03
9	->	28	-0.00	0.00
9	->	29	0.00	0.00
9	->	30	0.00	0.00
9	->	31	0.00	0.00
9	->	32	0.00	0.00
9	->	33	0.00	0.00
9	->	34	0.00	0.00
9	->	35	0.00	0.00
9	->	36	0.00	0.00
9	->	37	0.00	0.00
9	->	38	-0.00	0.00
9	->	39	0.00	0.00
9	->	40	-0.00	0.00

9	->	41	0.00	0.00
9	->	42	0.00	0.00
9	->	43	-0.00	0.00
9	->	44	-0.00	0.00
9	->	45	0.00	0.00
9	->	46	0.00	0.00
9	->	47	-0.00	0.00
9	->	48	0.00	0.00
9	->	49	0.00	0.00
9	->	50	-0.00	0.00
9	->	51	0.00	0.00
9	->	52	0.00	0.00
9	->	53	-0.00	0.00
9	->	54	-0.00	0.00
9	->	55	0.00	0.00
9	->	56	0.00	0.00
9	->	57	0.00	0.00
9	->	58	0.00	0.00
9	->	59	0.00	0.00
9	->	60	0.00	0.00
9	->	61	0.00	0.00
9	->	62	0.00	0.00
9	->	63	0.00	0.00
9	->	64	0.00	0.00
9	->	65	0.00	0.00
9	->	66	0.00	0.00
9	->	67	0.00	0.00
9	->	68	-0.00	0.00
9	->	69	-0.00	0.00
9	->	70	0.00	0.00
9	->	71	-0.00	0.00
9	->	72	-0.00	0.00
9	->	73	0.00	0.00
9	->	74	0.00	0.00
9	->	75	0.00	0.00
9	->	76	-0.00	0.00
9	->	77	-0.00	0.00
9	->	78	-0.00	0.00
9	->	79	0.00	0.00
9	->	80	0.00	0.00
9	->	81	-0.00	0.00
9	->	82	-0.00	0.00
9	->	83	0.00	0.00
9	->	84	0.00	0.00
9	->	85	-0.00	0.00
9	->	86	0.00	0.00
9	->	87	0.00	0.00
9	->	88	0.00	0.00
9	->	89	0.00	0.00
9	->	90	-0.00	0.00

9	->	91	-0.00	0.00
9	->	92	-0.00	0.00
9	->	93	0.00	0.00
9	->	94	0.00	0.00
9	->	95	0.00	0.00
9	->	96	0.00	0.00
9	->	97	-0.00	0.00
9	->	98	-0.00	0.00
9	->	99	0.00	0.00
9	->	100	0.00	0.00
9	->	101	0.00	0.00
9	->	102	0.00	0.00
9	->	103	0.00	0.00
9	->	104	0.00	0.00
9	->	105	0.00	0.00
9	->	106	0.00	0.00
9	->	107	0.00	0.00
10	->	1	-0.09	0.01
10	->	2	-0.00	0.00
10	->	3	-0.02	0.01
10	->	4	-0.14	0.06
10	->	5	1.73	0.67
10	->	6	-0.05	0.03
10	->	7	-1.35	0.78
10	->	8	-0.56	0.18
10	->	9	-9.89	0.67
10	->	10	-38.36	2.19
10	->	11	-14.25	0.37
10	->	12	-1.03	0.26
10	->	13	-0.35	0.20
10	->	14	-0.09	0.06
10	->	15	-0.00	0.00
10	->	16	-0.02	0.00
10	->	17	0.00	0.00
10	->	18	0.03	0.00
10	->	19	0.04	0.00
10	->	20	-0.01	0.00
10	->	21	0.00	0.00
10	->	22	-0.07	0.02
10	->	23	1.26	0.39
10	->	24	0.03	0.01
10	->	25	-0.04	0.00
10	->	26	-0.05	0.01
10	->	27	1.87	0.78
10	->	28	-0.01	0.00
10	->	29	-0.00	0.00
10	->	30	-0.00	0.00
10	->	31	-0.02	0.00
10	->	32	-0.00	0.00
10	->	33	0.00	0.00

10 ->	34	-0.00	0.00
10 ->	35	0.00	0.00
10 ->	36	-0.00	0.00
10 ->	37	0.00	0.00
10 ->	38	0.00	0.00
10 ->	39	0.00	0.00
10 ->	40	-0.01	0.00
10 ->	41	-0.00	0.00
10 ->	42	-0.00	0.00
10 ->	43	-0.00	0.00
10 ->	44	-0.02	0.00
10 ->	45	-0.00	0.00
10 ->	46	0.01	0.00
10 ->	47	-0.00	0.00
10 ->	48	-0.00	0.00
10 ->	49	-0.00	0.00
10 ->	50	-0.00	0.00
10 ->	51	-0.00	0.00
10 ->	52	-0.00	0.00
10 ->	53	-0.00	0.00
10 ->	54	-0.00	0.00
10 ->	55	0.00	0.00
10 ->	56	-0.00	0.00
10 ->	57	-0.00	0.00
10 ->	58	0.00	0.00
10 ->	59	-0.00	0.00
10 ->	60	-0.00	0.00
10 ->	61	-0.00	0.00
10 ->	62	0.00	0.00
10 ->	63	0.00	0.00
10 ->	64	0.00	0.00
10 ->	65	0.00	0.00
10 ->	66	0.00	0.00
10 ->	67	0.00	0.00
10 ->	68	-0.00	0.00
10 ->	69	-0.00	0.00
10 ->	70	-0.00	0.00
10 ->	71	-0.00	0.00
10 ->	72	-0.01	0.00
10 ->	73	-0.00	0.00
10 ->	74	0.01	0.00
10 ->	75	0.00	0.00
10 ->	76	-0.00	0.00
10 ->	77	-0.01	0.00
10 ->	78	-0.01	0.00
10 ->	79	-0.00	0.00
10 ->	80	-0.00	0.00
10 ->	81	-0.01	0.00
10 ->	82	-0.00	0.00
10 ->	83	-0.00	0.00

10 ->	84	-0.00	0.00
10 ->	85	-0.00	0.00
10 ->	86	0.00	0.00
10 ->	87	0.00	0.00
10 ->	88	0.01	0.00
10 ->	89	0.01	0.00
10 ->	90	0.02	0.00
10 ->	91	0.06	0.03
10 ->	92	0.11	0.06
10 ->	93	0.04	0.01
10 ->	94	0.02	0.00
10 ->	95	0.01	0.00
10 ->	96	0.00	0.00
10 ->	97	0.00	0.00
10 ->	98	0.01	0.00
10 ->	99	0.01	0.00
10 ->	100	0.01	0.00
10 ->	101	0.01	0.00
10 ->	102	0.01	0.00
10 ->	103	0.01	0.00
10 ->	104	0.01	0.00
10 ->	105	0.02	0.00
10 ->	106	0.01	0.00
10 ->	107	0.01	0.00
11 ->	1	-0.14	0.03
11 ->	2	-0.00	0.00
11 ->	3	-0.01	0.00
11 ->	4	-0.02	0.01
11 ->	5	0.07	0.01
11 ->	6	-0.00	0.00
11 ->	7	-0.04	0.02
11 ->	8	0.01	0.02
11 ->	9	-0.33	0.08
11 ->	10	-14.35	0.36
11 ->	11	-34.12	2.61
11 ->	12	-11.30	2.29
11 ->	13	-0.48	0.22
11 ->	14	-0.05	0.01
11 ->	15	-0.00	0.00
11 ->	16	-0.03	0.01
11 ->	17	0.00	0.00
11 ->	18	0.02	0.00
11 ->	19	0.04	0.01
11 ->	20	-0.00	0.00
11 ->	21	-0.00	0.00
11 ->	22	-0.03	0.01
11 ->	23	-0.03	0.04
11 ->	24	-0.00	0.00
11 ->	25	-0.02	0.00
11 ->	26	-0.01	0.01

11	->	27	0.01	0.01
11	->	28	-0.00	0.00
11	->	29	-0.00	0.00
11	->	30	0.00	0.00
11	->	31	-0.01	0.00
11	->	32	-0.00	0.00
11	->	33	0.00	0.00
11	->	34	-0.00	0.00
11	->	35	0.00	0.00
11	->	36	-0.00	0.00
11	->	37	0.00	0.00
11	->	38	0.00	0.00
11	->	39	0.00	0.00
11	->	40	-0.01	0.00
11	->	41	-0.00	0.00
11	->	42	-0.00	0.00
11	->	43	-0.00	0.00
11	->	44	-0.02	0.00
11	->	45	-0.00	0.00
11	->	46	0.01	0.00
11	->	47	-0.00	0.00
11	->	48	-0.00	0.00
11	->	49	-0.00	0.00
11	->	50	-0.00	0.00
11	->	51	-0.00	0.00
11	->	52	-0.00	0.00
11	->	53	-0.00	0.00
11	->	54	-0.00	0.00
11	->	55	-0.00	0.00
11	->	56	-0.00	0.00
11	->	57	-0.00	0.00
11	->	58	0.00	0.00
11	->	59	-0.00	0.00
11	->	60	-0.00	0.00
11	->	61	-0.00	0.00
11	->	62	0.00	0.00
11	->	63	0.00	0.00
11	->	64	0.00	0.00
11	->	65	0.00	0.00
11	->	66	0.00	0.00
11	->	67	0.00	0.00
11	->	68	-0.00	0.00
11	->	69	-0.00	0.00
11	->	70	-0.00	0.00
11	->	71	-0.00	0.00
11	->	72	-0.01	0.00
11	->	73	0.00	0.00
11	->	74	0.01	0.00
11	->	75	0.01	0.00
11	->	76	-0.00	0.00

11	->	77	-0.01	0.00
11	->	78	-0.01	0.00
11	->	79	-0.00	0.00
11	->	80	-0.00	0.00
11	->	81	-0.01	0.00
11	->	82	-0.00	0.00
11	->	83	-0.00	0.00
11	->	84	-0.00	0.00
11	->	85	-0.00	0.00
11	->	86	0.00	0.00
11	->	87	0.00	0.00
11	->	88	0.01	0.00
11	->	89	0.00	0.00
11	->	90	0.02	0.00
11	->	91	0.03	0.02
11	->	92	0.02	0.07
11	->	93	0.03	0.02
11	->	94	0.03	0.00
11	->	95	0.01	0.00
11	->	96	0.00	0.00
11	->	97	0.00	0.00
11	->	98	0.01	0.00
11	->	99	0.01	0.00
11	->	100	0.01	0.00
11	->	101	0.01	0.00
11	->	102	0.01	0.00
11	->	103	0.02	0.00
11	->	104	0.02	0.00
11	->	105	0.03	0.01
11	->	106	0.02	0.01
11	->	107	0.01	0.00
12	->	1	1.09	0.48
12	->	2	-0.02	0.01
12	->	3	-0.22	0.06
12	->	4	-0.11	0.06
12	->	5	-0.11	0.03
12	->	6	-0.01	0.00
12	->	7	-0.01	0.00
12	->	8	-0.07	0.02
12	->	9	-0.04	0.01
12	->	10	-1.04	0.27
12	->	11	-11.50	2.32
12	->	12	-107.89	2.94
12	->	13	-9.34	0.52
12	->	14	-1.91	0.65
12	->	15	-0.08	0.02
12	->	16	0.08	0.04
12	->	17	-0.01	0.00
12	->	18	-0.03	0.00
12	->	19	-0.23	0.10

12	->	20	-0.05	0.01
12	->	21	-0.00	0.00
12	->	22	0.08	0.02
12	->	23	-0.22	0.18
12	->	24	-0.02	0.01
12	->	25	0.02	0.01
12	->	26	-0.02	0.01
12	->	27	-0.04	0.01
12	->	28	0.00	0.00
12	->	29	0.00	0.00
12	->	30	-0.00	0.00
12	->	31	0.02	0.00
12	->	32	0.00	0.00
12	->	33	-0.00	0.00
12	->	34	0.00	0.00
12	->	35	-0.00	0.00
12	->	36	0.00	0.00
12	->	37	-0.00	0.00
12	->	38	-0.00	0.00
12	->	39	-0.00	0.00
12	->	40	0.01	0.00
12	->	41	0.00	0.00
12	->	42	0.00	0.00
12	->	43	0.00	0.00
12	->	44	0.04	0.01
12	->	45	0.00	0.00
12	->	46	-0.02	0.00
12	->	47	0.00	0.00
12	->	48	0.00	0.00
12	->	49	0.00	0.00
12	->	50	0.00	0.00
12	->	51	0.00	0.00
12	->	52	0.00	0.00
12	->	53	0.01	0.00
12	->	54	0.00	0.00
12	->	55	0.00	0.00
12	->	56	0.00	0.00
12	->	57	0.00	0.00
12	->	58	-0.00	0.00
12	->	59	0.01	0.00
12	->	60	0.00	0.00
12	->	61	0.00	0.00
12	->	62	-0.00	0.00
12	->	63	-0.00	0.00
12	->	64	-0.00	0.00
12	->	65	-0.00	0.00
12	->	66	-0.00	0.00
12	->	67	0.00	0.00
12	->	68	0.00	0.00
12	->	69	0.00	0.00

12	->	70	0.00	0.00
12	->	71	0.00	0.00
12	->	72	0.02	0.00
12	->	73	0.00	0.00
12	->	74	-0.02	0.01
12	->	75	-0.01	0.00
12	->	76	0.01	0.00
12	->	77	0.02	0.00
12	->	78	0.02	0.00
12	->	79	0.00	0.00
12	->	80	0.00	0.00
12	->	81	0.02	0.01
12	->	82	0.00	0.00
12	->	83	0.00	0.00
12	->	84	0.00	0.00
12	->	85	0.00	0.00
12	->	86	-0.00	0.00
12	->	87	-0.01	0.00
12	->	88	-0.01	0.00
12	->	89	-0.01	0.00
12	->	90	-0.03	0.01
12	->	91	-0.28	0.22
12	->	92	-6.43	1.95
12	->	93	-6.09	2.26
12	->	94	-0.11	0.03
12	->	95	-0.03	0.00
12	->	96	-0.01	0.00
12	->	97	-0.00	0.00
12	->	98	-0.01	0.00
12	->	99	-0.01	0.00
12	->	100	-0.01	0.00
12	->	101	-0.02	0.00
12	->	102	-0.03	0.01
12	->	103	-0.06	0.02
12	->	104	-0.08	0.04
12	->	105	-0.07	0.02
12	->	106	-0.04	0.01
12	->	107	-0.01	0.01
13	->	1	-3.85	1.60
13	->	2	-2.56	1.22
13	->	3	-3.46	0.39
13	->	4	-2.12	0.50
13	->	5	-0.35	0.08
13	->	6	-0.02	0.00
13	->	7	-0.01	0.02
13	->	8	-2.63	2.80
13	->	9	-0.06	0.05
13	->	10	-0.36	0.22
13	->	11	-0.49	0.24
13	->	12	-9.41	0.54

13 ->	13	-106.57	2.74
13 ->	14	-12.82	0.60
13 ->	15	-0.58	0.10
13 ->	16	0.08	0.05
13 ->	17	-0.02	0.01
13 ->	18	-0.03	0.01
13 ->	19	-0.45	0.12
13 ->	20	-0.09	0.02
13 ->	21	-0.01	0.01
13 ->	22	0.06	0.02
13 ->	23	-0.06	0.02
13 ->	24	-0.01	0.01
13 ->	25	0.02	0.00
13 ->	26	-0.00	0.00
13 ->	27	-0.03	0.01
13 ->	28	0.00	0.00
13 ->	29	0.00	0.00
13 ->	30	-0.00	0.00
13 ->	31	0.01	0.00
13 ->	32	0.00	0.00
13 ->	33	0.00	0.00
13 ->	34	0.00	0.00
13 ->	35	-0.00	0.00
13 ->	36	0.00	0.00
13 ->	37	-0.00	0.00
13 ->	38	-0.00	0.00
13 ->	39	-0.00	0.00
13 ->	40	0.00	0.00
13 ->	41	0.00	0.00
13 ->	42	0.00	0.00
13 ->	43	-0.00	0.00
13 ->	44	0.02	0.00
13 ->	45	0.00	0.00
13 ->	46	-0.01	0.00
13 ->	47	0.00	0.00
13 ->	48	0.00	0.00
13 ->	49	0.00	0.00
13 ->	50	0.00	0.00
13 ->	51	0.00	0.00
13 ->	52	0.00	0.00
13 ->	53	0.00	0.00
13 ->	54	0.00	0.00
13 ->	55	-0.00	0.00
13 ->	56	0.00	0.00
13 ->	57	0.00	0.00
13 ->	58	-0.00	0.00
13 ->	59	0.00	0.00
13 ->	60	0.00	0.00
13 ->	61	0.00	0.00
13 ->	62	-0.00	0.00

13	->	63	-0.00	0.00
13	->	64	-0.00	0.00
13	->	65	-0.00	0.00
13	->	66	-0.00	0.00
13	->	67	0.00	0.00
13	->	68	0.00	0.00
13	->	69	0.00	0.00
13	->	70	0.00	0.00
13	->	71	-0.00	0.00
13	->	72	0.01	0.00
13	->	73	0.00	0.00
13	->	74	-0.01	0.00
13	->	75	-0.00	0.00
13	->	76	0.00	0.00
13	->	77	0.01	0.00
13	->	78	0.00	0.00
13	->	79	0.00	0.00
13	->	80	0.00	0.00
13	->	81	0.00	0.00
13	->	82	0.00	0.00
13	->	83	0.00	0.00
13	->	84	0.00	0.00
13	->	85	0.00	0.00
13	->	86	-0.00	0.00
13	->	87	-0.00	0.00
13	->	88	-0.00	0.00
13	->	89	-0.00	0.00
13	->	90	-0.01	0.00
13	->	91	-0.03	0.01
13	->	92	-0.12	0.06
13	->	93	0.24	0.54
13	->	94	-0.09	0.03
13	->	95	-0.02	0.00
13	->	96	-0.01	0.00
13	->	97	-0.00	0.00
13	->	98	-0.01	0.00
13	->	99	-0.01	0.00
13	->	100	-0.01	0.00
13	->	101	-0.01	0.00
13	->	102	-0.01	0.00
13	->	103	-0.01	0.00
13	->	104	-0.01	0.00
13	->	105	-0.01	0.00
13	->	106	-0.01	0.00
13	->	107	-0.00	0.00
14	->	1	-3.03	1.10
14	->	2	-1.13	0.26
14	->	3	-4.00	0.48
14	->	4	-0.88	0.36
14	->	5	-0.46	0.22

14	->	6	-0.02	0.01
14	->	7	-0.04	0.01
14	->	8	-0.02	0.01
14	->	9	-0.01	0.00
14	->	10	-0.09	0.06
14	->	11	-0.05	0.01
14	->	12	-1.92	0.63
14	->	13	-12.86	0.59
14	->	14	30.24	1.38
14	->	15	-16.50	0.37
14	->	16	-0.27	0.25
14	->	17	-0.17	0.09
14	->	18	-0.09	0.03
14	->	19	-5.16	0.71
14	->	20	-2.42	0.46
14	->	21	-0.07	0.02
14	->	22	-0.33	0.19
14	->	23	-2.24	0.37
14	->	24	-0.06	0.02
14	->	25	-0.02	0.01
14	->	26	-0.04	0.01
14	->	27	-0.01	0.01
14	->	28	-0.00	0.00
14	->	29	-0.00	0.00
14	->	30	-0.00	0.00
14	->	31	-0.00	0.00
14	->	32	-0.00	0.00
14	->	33	-0.00	0.00
14	->	34	0.00	0.00
14	->	35	-0.00	0.00
14	->	36	0.00	0.00
14	->	37	-0.00	0.00
14	->	38	-0.00	0.00
14	->	39	0.00	0.00
14	->	40	0.00	0.00
14	->	41	0.00	0.00
14	->	42	-0.00	0.00
14	->	43	-0.00	0.00
14	->	44	-0.01	0.00
14	->	45	-0.00	0.00
14	->	46	-0.00	0.00
14	->	47	-0.00	0.00
14	->	48	0.00	0.00
14	->	49	0.00	0.00
14	->	50	0.00	0.00
14	->	51	0.00	0.00
14	->	52	0.00	0.00
14	->	53	0.00	0.00
14	->	54	0.00	0.00
14	->	55	0.00	0.00

14	->	56	0.00	0.00
14	->	57	0.00	0.00
14	->	58	-0.00	0.00
14	->	59	0.00	0.00
14	->	60	0.00	0.00
14	->	61	0.00	0.00
14	->	62	-0.00	0.00
14	->	63	-0.00	0.00
14	->	64	-0.00	0.00
14	->	65	0.00	0.00
14	->	66	-0.00	0.00
14	->	67	0.00	0.00
14	->	68	0.00	0.00
14	->	69	0.00	0.00
14	->	70	0.00	0.00
14	->	71	-0.00	0.00
14	->	72	0.00	0.00
14	->	73	0.00	0.00
14	->	74	-0.00	0.00
14	->	75	-0.00	0.00
14	->	76	0.00	0.00
14	->	77	0.00	0.00
14	->	78	0.00	0.00
14	->	79	0.00	0.00
14	->	80	0.00	0.00
14	->	81	0.00	0.00
14	->	82	0.00	0.00
14	->	83	0.00	0.00
14	->	84	0.00	0.00
14	->	85	0.00	0.00
14	->	86	-0.00	0.00
14	->	87	-0.00	0.00
14	->	88	-0.00	0.00
14	->	89	-0.00	0.00
14	->	90	-0.00	0.00
14	->	91	-0.01	0.02
14	->	92	-0.77	0.63
14	->	93	-0.49	0.36
14	->	94	-0.25	0.11
14	->	95	-0.01	0.01
14	->	96	-0.00	0.00
14	->	97	-0.00	0.00
14	->	98	-0.01	0.01
14	->	99	-0.00	0.00
14	->	100	-0.00	0.00
14	->	101	-0.00	0.00
14	->	102	-0.00	0.00
14	->	103	-0.00	0.00
14	->	104	-0.00	0.00
14	->	105	-0.00	0.00

14 ->	106	-0.00	0.00
14 ->	107	-0.00	0.00
15 ->	1	-4.11	1.64
15 ->	2	-1.03	0.24
15 ->	3	-1.47	0.37
15 ->	4	0.00	0.01
15 ->	5	0.01	0.01
15 ->	6	-0.00	0.00
15 ->	7	-0.00	0.00
15 ->	8	-0.00	0.00
15 ->	9	-0.00	0.00
15 ->	10	-0.00	0.00
15 ->	11	-0.00	0.00
15 ->	12	-0.08	0.02
15 ->	13	-0.58	0.10
15 ->	14	-16.52	0.38
15 ->	15	21.70	1.99
15 ->	16	-6.45	0.95
15 ->	17	-0.38	0.06
15 ->	18	-0.08	0.04
15 ->	19	-6.06	1.30
15 ->	20	-0.09	0.05
15 ->	21	-0.00	0.00
15 ->	22	0.01	0.02
15 ->	23	-0.01	0.00
15 ->	24	-0.00	0.00
15 ->	25	-0.00	0.00
15 ->	26	-0.00	0.00
15 ->	27	0.00	0.00
15 ->	28	0.00	0.00
15 ->	29	0.00	0.00
15 ->	30	-0.00	0.00
15 ->	31	0.00	0.00
15 ->	32	0.00	0.00
15 ->	33	-0.00	0.00
15 ->	34	0.00	0.00
15 ->	35	-0.00	0.00
15 ->	36	0.00	0.00
15 ->	37	-0.00	0.00
15 ->	38	-0.00	0.00
15 ->	39	0.00	0.00
15 ->	40	0.00	0.00
15 ->	41	0.00	0.00
15 ->	42	-0.00	0.00
15 ->	43	-0.00	0.00
15 ->	44	0.00	0.00
15 ->	45	0.00	0.00
15 ->	46	-0.00	0.00
15 ->	47	-0.00	0.00
15 ->	48	0.00	0.00

15	->	49	0.00	0.00
15	->	50	0.00	0.00
15	->	51	0.00	0.00
15	->	52	0.00	0.00
15	->	53	0.00	0.00
15	->	54	0.00	0.00
15	->	55	0.00	0.00
15	->	56	0.00	0.00
15	->	57	0.00	0.00
15	->	58	-0.00	0.00
15	->	59	0.00	0.00
15	->	60	0.00	0.00
15	->	61	0.00	0.00
15	->	62	-0.00	0.00
15	->	63	-0.00	0.00
15	->	64	-0.00	0.00
15	->	65	0.00	0.00
15	->	66	-0.00	0.00
15	->	67	0.00	0.00
15	->	68	0.00	0.00
15	->	69	0.00	0.00
15	->	70	0.00	0.00
15	->	71	-0.00	0.00
15	->	72	0.00	0.00
15	->	73	0.00	0.00
15	->	74	-0.00	0.00
15	->	75	-0.00	0.00
15	->	76	0.00	0.00
15	->	77	0.00	0.00
15	->	78	0.00	0.00
15	->	79	0.00	0.00
15	->	80	0.00	0.00
15	->	81	0.00	0.00
15	->	82	0.00	0.00
15	->	83	0.00	0.00
15	->	84	0.00	0.00
15	->	85	0.00	0.00
15	->	86	-0.00	0.00
15	->	87	-0.00	0.00
15	->	88	-0.00	0.00
15	->	89	-0.00	0.00
15	->	90	-0.00	0.00
15	->	91	-0.00	0.00
15	->	92	0.00	0.02
15	->	93	-0.05	0.12
15	->	94	-0.46	1.06
15	->	95	-0.05	0.03
15	->	96	-0.04	0.20
15	->	97	-0.01	0.00
15	->	98	-0.01	0.03

15	->	99	-0.00	0.00
15	->	100	0.00	0.00
15	->	101	-0.00	0.00
15	->	102	-0.00	0.00
15	->	103	-0.00	0.00
15	->	104	-0.00	0.00
15	->	105	-0.00	0.00
15	->	106	-0.00	0.00
15	->	107	-0.00	0.00
16	->	1	2.83	1.99
16	->	2	-0.09	0.06
16	->	3	-0.99	0.80
16	->	4	-0.01	0.00
16	->	5	-0.03	0.00
16	->	6	-0.00	0.00
16	->	7	-0.00	0.00
16	->	8	-0.02	0.01
16	->	9	-0.00	0.00
16	->	10	-0.02	0.00
16	->	11	-0.03	0.01
16	->	12	0.08	0.04
16	->	13	0.08	0.05
16	->	14	-0.27	0.25
16	->	15	-6.59	0.95
16	->	16	-96.73	2.76
16	->	17	-21.60	0.40
16	->	18	-1.98	1.03
16	->	19	-19.17	2.91
16	->	20	-1.33	0.58
16	->	21	-0.07	0.04
16	->	22	0.26	0.15
16	->	23	-0.02	0.01
16	->	24	-0.01	0.00
16	->	25	0.03	0.01
16	->	26	-0.00	0.00
16	->	27	-0.00	0.00
16	->	28	0.00	0.00
16	->	29	0.00	0.00
16	->	30	-0.00	0.00
16	->	31	0.01	0.00
16	->	32	0.00	0.00
16	->	33	-0.00	0.00
16	->	34	-0.00	0.00
16	->	35	-0.01	0.00
16	->	36	0.01	0.00
16	->	37	-0.00	0.00
16	->	38	-0.00	0.00
16	->	39	-0.00	0.00
16	->	40	0.01	0.00
16	->	41	0.00	0.00

16 ->	42	0.00	0.00
16 ->	43	-0.00	0.00
16 ->	44	0.08	0.03
16 ->	45	-0.00	0.00
16 ->	46	-0.04	0.01
16 ->	47	-0.00	0.00
16 ->	48	0.00	0.00
16 ->	49	0.00	0.00
16 ->	50	0.00	0.00
16 ->	51	0.00	0.00
16 ->	52	0.00	0.00
16 ->	53	0.02	0.00
16 ->	54	0.00	0.00
16 ->	55	0.00	0.00
16 ->	56	0.00	0.00
16 ->	57	0.00	0.00
16 ->	58	-0.01	0.00
16 ->	59	0.01	0.00
16 ->	60	0.00	0.00
16 ->	61	0.00	0.00
16 ->	62	-0.00	0.00
16 ->	63	-0.00	0.00
16 ->	64	-0.00	0.00
16 ->	65	-0.00	0.00
16 ->	66	-0.00	0.00
16 ->	67	-0.00	0.00
16 ->	68	0.00	0.00
16 ->	69	0.00	0.00
16 ->	70	0.00	0.00
16 ->	71	-0.00	0.00
16 ->	72	0.02	0.00
16 ->	73	0.00	0.00
16 ->	74	-0.02	0.00
16 ->	75	-0.01	0.00
16 ->	76	0.01	0.00
16 ->	77	0.02	0.00
16 ->	78	0.01	0.00
16 ->	79	0.00	0.00
16 ->	80	0.00	0.00
16 ->	81	0.01	0.00
16 ->	82	0.00	0.00
16 ->	83	0.00	0.00
16 ->	84	0.00	0.00
16 ->	85	0.00	0.00
16 ->	86	-0.00	0.00
16 ->	87	-0.00	0.00
16 ->	88	-0.01	0.00
16 ->	89	-0.01	0.00
16 ->	90	-0.02	0.00
16 ->	91	-0.03	0.01

16 ->	92	-0.21	0.07
16 ->	93	-2.21	0.72
16 ->	94	-11.52	2.02
16 ->	95	-1.99	0.86
16 ->	96	-0.27	0.40
16 ->	97	-0.06	0.04
16 ->	98	-0.25	0.60
16 ->	99	-0.09	0.10
16 ->	100	0.01	0.05
16 ->	101	-0.06	0.01
16 ->	102	-0.07	0.02
16 ->	103	-0.04	0.01
16 ->	104	-0.03	0.00
16 ->	105	-0.02	0.00
16 ->	106	-0.01	0.00
16 ->	107	-0.00	0.00
17 ->	1	-0.07	0.04
17 ->	2	-0.02	0.02
17 ->	3	-0.68	0.82
17 ->	4	-0.01	0.01
17 ->	5	-0.00	0.00
17 ->	6	-0.00	0.00
17 ->	7	-0.00	0.00
17 ->	8	0.00	0.00
17 ->	9	0.00	0.00
17 ->	10	0.00	0.00
17 ->	11	0.00	0.00
17 ->	12	-0.01	0.00
17 ->	13	-0.02	0.01
17 ->	14	-0.17	0.09
17 ->	15	-0.38	0.06
17 ->	16	-21.60	0.40
17 ->	17	18.50	1.76
17 ->	18	-10.69	0.53
17 ->	19	-0.65	0.19
17 ->	20	-1.92	0.69
17 ->	21	-3.17	1.90
17 ->	22	-0.12	0.09
17 ->	23	-0.04	0.02
17 ->	24	-0.05	0.03
17 ->	25	-0.02	0.02
17 ->	26	-0.00	0.00
17 ->	27	-0.00	0.00
17 ->	28	-0.00	0.00
17 ->	29	-0.00	0.00
17 ->	30	-0.00	0.00
17 ->	31	-0.00	0.00
17 ->	32	-0.00	0.00
17 ->	33	-0.00	0.00
17 ->	34	-0.00	0.00

17 ->	35	0.00	0.00
17 ->	36	-0.00	0.00
17 ->	37	0.00	0.00
17 ->	38	0.00	0.00
17 ->	39	0.00	0.00
17 ->	40	-0.00	0.00
17 ->	41	-0.00	0.00
17 ->	42	0.00	0.00
17 ->	43	-0.00	0.00
17 ->	44	-0.00	0.00
17 ->	45	-0.00	0.00
17 ->	46	-0.00	0.00
17 ->	47	-0.00	0.00
17 ->	48	-0.00	0.00
17 ->	49	-0.00	0.00
17 ->	50	-0.00	0.00
17 ->	51	-0.00	0.00
17 ->	52	0.00	0.00
17 ->	53	0.00	0.00
17 ->	54	-0.00	0.00
17 ->	55	0.00	0.00
17 ->	56	-0.00	0.00
17 ->	57	-0.00	0.00
17 ->	58	-0.00	0.00
17 ->	59	-0.00	0.00
17 ->	60	0.00	0.00
17 ->	61	0.00	0.00
17 ->	62	0.00	0.00
17 ->	63	0.00	0.00
17 ->	64	0.00	0.00
17 ->	65	0.00	0.00
17 ->	66	0.00	0.00
17 ->	67	0.00	0.00
17 ->	68	-0.00	0.00
17 ->	69	0.00	0.00
17 ->	70	-0.00	0.00
17 ->	71	-0.00	0.00
17 ->	72	-0.00	0.00
17 ->	73	0.00	0.00
17 ->	74	0.00	0.00
17 ->	75	0.00	0.00
17 ->	76	0.00	0.00
17 ->	77	-0.00	0.00
17 ->	78	-0.00	0.00
17 ->	79	0.00	0.00
17 ->	80	0.00	0.00
17 ->	81	-0.00	0.00
17 ->	82	0.00	0.00
17 ->	83	0.00	0.00
17 ->	84	0.00	0.00

17	->	85	0.00	0.00
17	->	86	0.00	0.00
17	->	87	0.00	0.00
17	->	88	0.00	0.00
17	->	89	0.00	0.00
17	->	90	0.00	0.00
17	->	91	-0.00	0.00
17	->	92	-0.00	0.00
17	->	93	-0.01	0.01
17	->	94	-0.01	0.01
17	->	95	-0.03	0.04
17	->	96	-0.07	0.14
17	->	97	-0.39	0.66
17	->	98	-0.45	0.91
17	->	99	-0.04	0.06
17	->	100	-0.01	0.01
17	->	101	-0.00	0.00
17	->	102	-0.00	0.00
17	->	103	-0.00	0.00
17	->	104	0.00	0.00
17	->	105	0.00	0.00
17	->	106	0.00	0.00
17	->	107	0.00	0.00
18	->	1	-0.14	0.11
18	->	2	-0.01	0.00
18	->	3	-0.02	0.01
18	->	4	-0.00	0.00
18	->	5	0.03	0.00
18	->	6	-0.01	0.00
18	->	7	-0.00	0.00
18	->	8	0.01	0.00
18	->	9	0.00	0.00
18	->	10	0.03	0.00
18	->	11	0.02	0.00
18	->	12	-0.03	0.00
18	->	13	-0.03	0.01
18	->	14	-0.09	0.03
18	->	15	-0.08	0.04
18	->	16	-1.98	1.02
18	->	17	-10.73	0.54
18	->	18	-19.85	3.59
18	->	19	-13.02	0.55
18	->	20	-1.04	0.20
18	->	21	-3.16	0.85
18	->	22	-7.44	5.06
18	->	23	-0.12	0.08
18	->	24	-0.07	0.03
18	->	25	-1.72	3.07
18	->	26	-0.01	0.00
18	->	27	-0.01	0.00

18 ->	28	-0.01	0.00
18 ->	29	-0.00	0.00
18 ->	30	0.00	0.00
18 ->	31	-0.04	0.01
18 ->	32	-0.01	0.01
18 ->	33	-0.03	0.04
18 ->	34	-0.00	0.00
18 ->	35	0.02	0.00
18 ->	36	-0.01	0.00
18 ->	37	0.00	0.00
18 ->	38	0.01	0.00
18 ->	39	0.00	0.00
18 ->	40	-0.02	0.00
18 ->	41	-0.00	0.00
18 ->	42	-0.01	0.01
18 ->	43	-0.01	0.02
18 ->	44	-0.52	0.55
18 ->	45	-0.02	0.06
18 ->	46	0.09	0.05
18 ->	47	-0.01	0.00
18 ->	48	-0.01	0.00
18 ->	49	-0.01	0.00
18 ->	50	-0.00	0.00
18 ->	51	-0.00	0.00
18 ->	52	-0.00	0.00
18 ->	53	-0.03	0.00
18 ->	54	-0.00	0.00
18 ->	55	-0.00	0.00
18 ->	56	-0.00	0.00
18 ->	57	-0.00	0.00
18 ->	58	0.01	0.00
18 ->	59	-0.01	0.00
18 ->	60	-0.00	0.00
18 ->	61	-0.00	0.00
18 ->	62	0.00	0.00
18 ->	63	0.00	0.00
18 ->	64	0.00	0.00
18 ->	65	0.00	0.00
18 ->	66	0.00	0.00
18 ->	67	0.00	0.00
18 ->	68	-0.00	0.00
18 ->	69	-0.01	0.00
18 ->	70	-0.00	0.00
18 ->	71	0.00	0.00
18 ->	72	-0.03	0.01
18 ->	73	-0.00	0.00
18 ->	74	0.02	0.00
18 ->	75	0.01	0.00
18 ->	76	-0.01	0.00
18 ->	77	-0.02	0.00

18	->	78	-0.01	0.00
18	->	79	-0.00	0.00
18	->	80	-0.00	0.00
18	->	81	-0.01	0.00
18	->	82	-0.00	0.00
18	->	83	-0.00	0.00
18	->	84	-0.00	0.00
18	->	85	-0.00	0.00
18	->	86	0.00	0.00
18	->	87	0.01	0.00
18	->	88	0.01	0.00
18	->	89	0.02	0.00
18	->	90	0.03	0.01
18	->	91	0.04	0.01
18	->	92	0.06	0.09
18	->	93	0.19	0.27
18	->	94	-2.98	2.37
18	->	95	-0.84	1.22
18	->	96	-0.14	0.28
18	->	97	-0.21	0.37
18	->	98	-0.52	0.80
18	->	99	-0.37	0.52
18	->	100	0.07	0.12
18	->	101	-0.01	0.03
18	->	102	0.01	0.01
18	->	103	0.02	0.00
18	->	104	0.01	0.00
18	->	105	0.01	0.00
18	->	106	0.01	0.00
18	->	107	0.01	0.00
19	->	1	-11.44	7.38
19	->	2	-0.04	0.02
19	->	3	-0.09	0.06
19	->	4	0.00	0.01
19	->	5	0.01	0.01
19	->	6	-0.01	0.00
19	->	7	0.00	0.00
19	->	8	0.02	0.01
19	->	9	-0.00	0.00
19	->	10	0.04	0.00
19	->	11	0.04	0.01
19	->	12	-0.23	0.10
19	->	13	-0.45	0.12
19	->	14	-5.16	0.70
19	->	15	-6.05	1.30
19	->	16	-19.13	2.92
19	->	17	-0.66	0.19
19	->	18	-13.08	0.56
19	->	19	-28.83	6.01
19	->	20	-8.43	0.52

19	->	21	-1.02	0.16
19	->	22	-4.72	0.99
19	->	23	-3.07	0.75
19	->	24	-0.11	0.08
19	->	25	-0.14	0.04
19	->	26	-0.04	0.01
19	->	27	-0.02	0.00
19	->	28	-0.01	0.00
19	->	29	-0.00	0.00
19	->	30	0.00	0.00
19	->	31	-0.02	0.00
19	->	32	-0.01	0.00
19	->	33	-0.00	0.00
19	->	34	-0.00	0.00
19	->	35	0.01	0.00
19	->	36	-0.01	0.00
19	->	37	0.00	0.00
19	->	38	0.01	0.00
19	->	39	0.00	0.00
19	->	40	-0.01	0.00
19	->	41	-0.00	0.00
19	->	42	-0.00	0.00
19	->	43	-0.01	0.00
19	->	44	-0.15	0.06
19	->	45	-0.01	0.00
19	->	46	0.04	0.01
19	->	47	-0.01	0.00
19	->	48	-0.00	0.00
19	->	49	-0.00	0.00
19	->	50	-0.00	0.00
19	->	51	-0.00	0.00
19	->	52	-0.00	0.00
19	->	53	-0.01	0.00
19	->	54	-0.00	0.00
19	->	55	-0.00	0.00
19	->	56	-0.00	0.00
19	->	57	-0.00	0.00
19	->	58	0.01	0.00
19	->	59	-0.01	0.00
19	->	60	-0.00	0.00
19	->	61	-0.00	0.00
19	->	62	0.00	0.00
19	->	63	0.00	0.00
19	->	64	0.00	0.00
19	->	65	0.00	0.00
19	->	66	0.00	0.00
19	->	67	-0.00	0.00
19	->	68	-0.00	0.00
19	->	69	-0.00	0.00
19	->	70	-0.00	0.00

19	->	71	0.00	0.00
19	->	72	-0.03	0.01
19	->	73	-0.00	0.00
19	->	74	0.02	0.00
19	->	75	0.01	0.00
19	->	76	-0.01	0.00
19	->	77	-0.02	0.00
19	->	78	-0.01	0.00
19	->	79	-0.00	0.00
19	->	80	-0.00	0.00
19	->	81	-0.01	0.00
19	->	82	-0.00	0.00
19	->	83	-0.00	0.00
19	->	84	-0.00	0.00
19	->	85	-0.00	0.00
19	->	86	0.00	0.00
19	->	87	0.01	0.00
19	->	88	0.01	0.00
19	->	89	0.01	0.00
19	->	90	0.02	0.00
19	->	91	0.00	0.02
19	->	92	0.08	0.16
19	->	93	-0.40	0.98
19	->	94	0.27	0.68
19	->	95	0.01	0.05
19	->	96	-0.00	0.02
19	->	97	-0.01	0.01
19	->	98	0.01	0.03
19	->	99	0.01	0.02
19	->	100	-0.04	0.04
19	->	101	0.02	0.01
19	->	102	0.04	0.01
19	->	103	0.03	0.00
19	->	104	0.02	0.00
19	->	105	0.02	0.00
19	->	106	0.01	0.00
19	->	107	0.01	0.00
20	->	1	-0.13	0.07
20	->	2	-0.03	0.01
20	->	3	-1.28	0.57
20	->	4	-1.01	0.32
20	->	5	-1.04	0.24
20	->	6	-0.21	0.14
20	->	7	-0.02	0.01
20	->	8	-0.01	0.01
20	->	9	-0.00	0.00
20	->	10	-0.01	0.00
20	->	11	-0.00	0.00
20	->	12	-0.05	0.01
20	->	13	-0.09	0.02

20 ->	14	-2.47	0.46
20 ->	15	-0.09	0.05
20 ->	16	-1.34	0.59
20 ->	17	-1.94	0.69
20 ->	18	-1.05	0.20
20 ->	19	-8.46	0.52
20 ->	20	21.59	1.38
20 ->	21	-6.15	0.62
20 ->	22	-1.42	0.25
20 ->	23	-2.78	0.40
20 ->	24	-3.88	0.65
20 ->	25	-0.16	0.08
20 ->	26	-0.06	0.02
20 ->	27	-0.06	0.02
20 ->	28	-0.02	0.00
20 ->	29	-0.00	0.00
20 ->	30	-0.01	0.00
20 ->	31	-0.00	0.00
20 ->	32	-0.00	0.00
20 ->	33	-0.01	0.00
20 ->	34	-0.00	0.00
20 ->	35	0.00	0.00
20 ->	36	-0.00	0.00
20 ->	37	0.00	0.00
20 ->	38	0.00	0.00
20 ->	39	0.00	0.00
20 ->	40	-0.00	0.00
20 ->	41	0.00	0.00
20 ->	42	-0.00	0.00
20 ->	43	-0.01	0.00
20 ->	44	-0.02	0.01
20 ->	45	-0.00	0.00
20 ->	46	-0.00	0.00
20 ->	47	-0.00	0.00
20 ->	48	-0.00	0.00
20 ->	49	-0.00	0.00
20 ->	50	-0.00	0.00
20 ->	51	-0.00	0.00
20 ->	52	-0.00	0.00
20 ->	53	-0.00	0.00
20 ->	54	-0.00	0.00
20 ->	55	0.00	0.00
20 ->	56	0.00	0.00
20 ->	57	0.00	0.00
20 ->	58	-0.00	0.00
20 ->	59	-0.00	0.00
20 ->	60	-0.00	0.00
20 ->	61	0.00	0.00
20 ->	62	0.00	0.00
20 ->	63	0.00	0.00

20	->	64	0.00	0.00
20	->	65	0.00	0.00
20	->	66	0.00	0.00
20	->	67	0.00	0.00
20	->	68	-0.00	0.00
20	->	69	0.00	0.00
20	->	70	0.00	0.00
20	->	71	-0.00	0.00
20	->	72	-0.00	0.00
20	->	73	0.00	0.00
20	->	74	0.00	0.00
20	->	75	0.00	0.00
20	->	76	0.00	0.00
20	->	77	0.00	0.00
20	->	78	-0.00	0.00
20	->	79	0.00	0.00
20	->	80	0.00	0.00
20	->	81	-0.00	0.00
20	->	82	0.00	0.00
20	->	83	0.00	0.00
20	->	84	0.00	0.00
20	->	85	0.00	0.00
20	->	86	0.00	0.00
20	->	87	0.00	0.00
20	->	88	0.00	0.00
20	->	89	-0.00	0.00
20	->	90	-0.00	0.00
20	->	91	-0.00	0.01
20	->	92	0.03	0.04
20	->	93	0.03	0.02
20	->	94	-0.01	0.01
20	->	95	-0.01	0.00
20	->	96	-0.00	0.00
20	->	97	-0.01	0.01
20	->	98	-0.01	0.01
20	->	99	-0.01	0.00
20	->	100	-0.01	0.00
20	->	101	-0.00	0.00
20	->	102	-0.00	0.00
20	->	103	-0.00	0.00
20	->	104	-0.00	0.00
20	->	105	-0.00	0.00
20	->	106	0.00	0.00
20	->	107	0.00	0.00
21	->	1	0.01	0.02
21	->	2	-0.00	0.00
21	->	3	-0.03	0.01
21	->	4	-0.02	0.01
21	->	5	-0.01	0.01
21	->	6	-0.01	0.01

21	->	7	-0.01	0.00
21	->	8	-0.00	0.00
21	->	9	-0.00	0.00
21	->	10	0.00	0.00
21	->	11	-0.00	0.00
21	->	12	-0.00	0.00
21	->	13	-0.01	0.01
21	->	14	-0.07	0.02
21	->	15	-0.00	0.00
21	->	16	-0.07	0.04
21	->	17	-3.17	1.89
21	->	18	-3.20	0.86
21	->	19	-1.03	0.16
21	->	20	-6.17	0.61
21	->	21	-20.50	2.28
21	->	22	-10.46	0.41
21	->	23	-0.88	0.17
21	->	24	-2.81	0.53
21	->	25	-3.48	0.65
21	->	26	-0.08	0.05
21	->	27	-0.06	0.03
21	->	28	-0.03	0.02
21	->	29	-0.01	0.01
21	->	30	-0.07	0.06
21	->	31	-0.00	0.00
21	->	32	-0.02	0.01
21	->	33	-0.03	0.02
21	->	34	-0.00	0.00
21	->	35	-0.00	0.00
21	->	36	0.00	0.00
21	->	37	0.00	0.00
21	->	38	0.00	0.00
21	->	39	0.00	0.00
21	->	40	-0.00	0.00
21	->	41	0.00	0.00
21	->	42	0.00	0.00
21	->	43	-0.06	0.03
21	->	44	0.03	0.02
21	->	45	-0.02	0.01
21	->	46	-0.01	0.01
21	->	47	-0.01	0.00
21	->	48	-0.00	0.00
21	->	49	-0.00	0.00
21	->	50	-0.00	0.00
21	->	51	-0.00	0.00
21	->	52	0.00	0.00
21	->	53	0.00	0.00
21	->	54	0.00	0.00
21	->	55	0.00	0.00
21	->	56	0.00	0.00

21	->	57	0.00	0.00
21	->	58	-0.00	0.00
21	->	59	0.00	0.00
21	->	60	0.00	0.00
21	->	61	0.00	0.00
21	->	62	-0.00	0.00
21	->	63	-0.00	0.00
21	->	64	-0.00	0.00
21	->	65	0.00	0.00
21	->	66	-0.00	0.00
21	->	67	0.00	0.00
21	->	68	0.00	0.00
21	->	69	0.00	0.00
21	->	70	0.00	0.00
21	->	71	-0.00	0.00
21	->	72	0.00	0.00
21	->	73	0.00	0.00
21	->	74	-0.00	0.00
21	->	75	-0.00	0.00
21	->	76	0.00	0.00
21	->	77	0.00	0.00
21	->	78	0.00	0.00
21	->	79	0.00	0.00
21	->	80	0.00	0.00
21	->	81	0.00	0.00
21	->	82	0.00	0.00
21	->	83	0.00	0.00
21	->	84	0.00	0.00
21	->	85	0.00	0.00
21	->	86	0.00	0.00
21	->	87	-0.00	0.00
21	->	88	-0.00	0.00
21	->	89	-0.00	0.00
21	->	90	-0.01	0.01
21	->	91	-0.04	0.02
21	->	92	-0.08	0.06
21	->	93	-0.03	0.01
21	->	94	-0.00	0.01
21	->	95	-0.01	0.00
21	->	96	-0.00	0.00
21	->	97	-0.02	0.02
21	->	98	-0.04	0.04
21	->	99	-0.02	0.02
21	->	100	-0.01	0.00
21	->	101	-0.00	0.00
21	->	102	-0.00	0.00
21	->	103	-0.00	0.00
21	->	104	-0.00	0.00
21	->	105	-0.00	0.00
21	->	106	0.00	0.00

21	->	107	0.00	0.00
22	->	1	0.36	0.24
22	->	2	-0.00	0.00
22	->	3	-0.01	0.01
22	->	4	0.01	0.01
22	->	5	-0.14	0.04
22	->	6	-0.00	0.00
22	->	7	0.01	0.01
22	->	8	-0.02	0.00
22	->	9	-0.00	0.00
22	->	10	-0.07	0.02
22	->	11	-0.03	0.01
22	->	12	0.08	0.02
22	->	13	0.06	0.02
22	->	14	-0.33	0.19
22	->	15	0.01	0.02
22	->	16	0.26	0.15
22	->	17	-0.12	0.09
22	->	18	-7.51	5.09
22	->	19	-4.75	0.98
22	->	20	-1.43	0.25
22	->	21	-10.48	0.40
22	->	22	-97.02	3.27
22	->	23	-19.54	0.42
22	->	24	-0.73	0.20
22	->	25	-2.75	0.67
22	->	26	-1.93	0.79
22	->	27	-0.15	0.05
22	->	28	-0.03	0.01
22	->	29	-0.01	0.01
22	->	30	-0.04	0.01
22	->	31	0.03	0.01
22	->	32	-0.03	0.01
22	->	33	-0.06	0.03
22	->	34	-0.00	0.00
22	->	35	-0.02	0.00
22	->	36	0.01	0.00
22	->	37	-0.00	0.00
22	->	38	-0.01	0.00
22	->	39	-0.00	0.00
22	->	40	0.02	0.00
22	->	41	0.01	0.00
22	->	42	0.02	0.02
22	->	43	-0.38	0.35
22	->	44	-0.63	0.70
22	->	45	-0.08	0.04
22	->	46	-0.48	0.36
22	->	47	-0.09	0.05
22	->	48	-0.01	0.01
22	->	49	0.00	0.00

22 ->	50	-0.00	0.00
22 ->	51	0.00	0.00
22 ->	52	0.00	0.00
22 ->	53	0.03	0.00
22 ->	54	0.00	0.00
22 ->	55	0.00	0.00
22 ->	56	0.00	0.00
22 ->	57	0.00	0.00
22 ->	58	-0.01	0.00
22 ->	59	0.01	0.00
22 ->	60	0.00	0.00
22 ->	61	-0.00	0.00
22 ->	62	-0.00	0.00
22 ->	63	-0.01	0.00
22 ->	64	-0.00	0.00
22 ->	65	-0.00	0.00
22 ->	66	-0.00	0.00
22 ->	67	0.00	0.00
22 ->	68	0.01	0.00
22 ->	69	0.01	0.00
22 ->	70	0.00	0.00
22 ->	71	-0.00	0.00
22 ->	72	0.06	0.02
22 ->	73	0.00	0.00
22 ->	74	-0.04	0.00
22 ->	75	-0.02	0.00
22 ->	76	0.02	0.00
22 ->	77	0.02	0.00
22 ->	78	0.02	0.01
22 ->	79	0.00	0.00
22 ->	80	0.00	0.00
22 ->	81	0.01	0.00
22 ->	82	0.00	0.00
22 ->	83	0.00	0.00
22 ->	84	0.00	0.00
22 ->	85	0.00	0.00
22 ->	86	-0.00	0.00
22 ->	87	-0.01	0.00
22 ->	88	-0.02	0.00
22 ->	89	-0.03	0.01
22 ->	90	-0.15	0.06
22 ->	91	-1.27	0.84
22 ->	92	-5.39	2.95
22 ->	93	-6.15	2.00
22 ->	94	-0.14	0.58
22 ->	95	-0.05	0.09
22 ->	96	-0.03	0.01
22 ->	97	-0.02	0.01
22 ->	98	-0.07	0.03
22 ->	99	-0.38	0.75

22	->	100	-1.22	1.71
22	->	101	0.15	0.19
22	->	102	-0.09	0.08
22	->	103	-0.07	0.03
22	->	104	-0.04	0.01
22	->	105	-0.03	0.00
22	->	106	-0.02	0.00
22	->	107	-0.01	0.00
23	->	1	0.02	0.03
23	->	2	-0.00	0.00
23	->	3	-0.03	0.01
23	->	4	-0.17	0.08
23	->	5	1.24	0.35
23	->	6	-0.08	0.03
23	->	7	-0.28	0.16
23	->	8	-0.01	0.02
23	->	9	-0.03	0.01
23	->	10	1.22	0.39
23	->	11	-0.03	0.04
23	->	12	-0.22	0.18
23	->	13	-0.06	0.02
23	->	14	-2.29	0.37
23	->	15	-0.01	0.00
23	->	16	-0.02	0.01
23	->	17	-0.04	0.02
23	->	18	-0.12	0.08
23	->	19	-3.10	0.75
23	->	20	-2.78	0.39
23	->	21	-0.89	0.17
23	->	22	-19.59	0.42
23	->	23	11.39	1.68
23	->	24	-10.16	0.59
23	->	25	-1.34	0.27
23	->	26	-3.23	0.55
23	->	27	30.32	18.32
23	->	28	-0.23	0.07
23	->	29	-0.03	0.02
23	->	30	-0.03	0.01
23	->	31	-0.01	0.00
23	->	32	-0.01	0.00
23	->	33	-0.00	0.00
23	->	34	-0.00	0.00
23	->	35	-0.00	0.00
23	->	36	0.00	0.00
23	->	37	-0.00	0.00
23	->	38	-0.00	0.00
23	->	39	0.00	0.00
23	->	40	-0.00	0.00
23	->	41	-0.00	0.00
23	->	42	-0.01	0.00

23	->	43	-0.01	0.01
23	->	44	-0.05	0.02
23	->	45	0.00	0.00
23	->	46	-0.00	0.00
23	->	47	-0.00	0.00
23	->	48	-0.00	0.00
23	->	49	0.00	0.00
23	->	50	0.00	0.00
23	->	51	0.00	0.00
23	->	52	0.00	0.00
23	->	53	0.00	0.00
23	->	54	0.00	0.00
23	->	55	0.00	0.00
23	->	56	0.00	0.00
23	->	57	0.00	0.00
23	->	58	-0.00	0.00
23	->	59	0.00	0.00
23	->	60	0.00	0.00
23	->	61	0.00	0.00
23	->	62	-0.00	0.00
23	->	63	-0.00	0.00
23	->	64	-0.00	0.00
23	->	65	-0.00	0.00
23	->	66	-0.00	0.00
23	->	67	0.00	0.00
23	->	68	0.00	0.00
23	->	69	0.00	0.00
23	->	70	0.00	0.00
23	->	71	-0.00	0.00
23	->	72	0.00	0.00
23	->	73	0.00	0.00
23	->	74	-0.00	0.00
23	->	75	-0.00	0.00
23	->	76	0.00	0.00
23	->	77	0.00	0.00
23	->	78	0.00	0.00
23	->	79	0.00	0.00
23	->	80	0.00	0.00
23	->	81	0.00	0.00
23	->	82	0.00	0.00
23	->	83	0.00	0.00
23	->	84	0.00	0.00
23	->	85	0.00	0.00
23	->	86	-0.00	0.00
23	->	87	-0.00	0.00
23	->	88	-0.00	0.00
23	->	89	-0.00	0.00
23	->	90	-0.01	0.01
23	->	91	-0.68	0.46
23	->	92	-4.95	3.10

23	->	93	-0.06	0.02
23	->	94	-0.01	0.00
23	->	95	-0.01	0.00
23	->	96	-0.00	0.00
23	->	97	-0.00	0.00
23	->	98	-0.01	0.00
23	->	99	-0.01	0.00
23	->	100	-0.01	0.00
23	->	101	-0.00	0.00
23	->	102	-0.00	0.00
23	->	103	-0.01	0.00
23	->	104	-0.01	0.00
23	->	105	-0.00	0.00
23	->	106	-0.00	0.00
23	->	107	-0.00	0.00
24	->	1	-0.01	0.01
24	->	2	-0.00	0.00
24	->	3	-0.02	0.01
24	->	4	-0.06	0.03
24	->	5	-0.68	0.25
24	->	6	-0.92	0.36
24	->	7	-0.06	0.03
24	->	8	-0.01	0.00
24	->	9	-0.00	0.00
24	->	10	0.03	0.01
24	->	11	-0.00	0.00
24	->	12	-0.02	0.01
24	->	13	-0.01	0.01
24	->	14	-0.06	0.02
24	->	15	-0.00	0.00
24	->	16	-0.01	0.00
24	->	17	-0.05	0.03
24	->	18	-0.07	0.03
24	->	19	-0.11	0.08
24	->	20	-3.87	0.64
24	->	21	-2.84	0.52
24	->	22	-0.74	0.20
24	->	23	-10.20	0.59
24	->	24	23.88	1.43
24	->	25	-10.23	0.48
24	->	26	-1.08	0.17
24	->	27	-2.88	0.41
24	->	28	-5.46	0.81
24	->	29	-0.17	0.10
24	->	30	-0.63	0.28
24	->	31	-0.00	0.00
24	->	32	-0.01	0.00
24	->	33	-0.01	0.00
24	->	34	-0.00	0.00
24	->	35	0.00	0.00

24	->	36	0.00	0.00
24	->	37	0.00	0.00
24	->	38	0.00	0.00
24	->	39	0.00	0.00
24	->	40	-0.00	0.00
24	->	41	0.00	0.00
24	->	42	0.00	0.00
24	->	43	-0.02	0.01
24	->	44	-0.01	0.01
24	->	45	-0.00	0.00
24	->	46	-0.00	0.00
24	->	47	-0.00	0.00
24	->	48	-0.00	0.00
24	->	49	0.00	0.00
24	->	50	0.00	0.00
24	->	51	0.00	0.00
24	->	52	-0.00	0.00
24	->	53	0.00	0.00
24	->	54	0.00	0.00
24	->	55	0.00	0.00
24	->	56	0.00	0.00
24	->	57	0.00	0.00
24	->	58	-0.00	0.00
24	->	59	-0.00	0.00
24	->	60	0.00	0.00
24	->	61	0.00	0.00
24	->	62	0.00	0.00
24	->	63	-0.00	0.00
24	->	64	-0.00	0.00
24	->	65	0.00	0.00
24	->	66	-0.00	0.00
24	->	67	0.00	0.00
24	->	68	-0.00	0.00
24	->	69	0.00	0.00
24	->	70	-0.00	0.00
24	->	71	0.00	0.00
24	->	72	0.00	0.00
24	->	73	0.00	0.00
24	->	74	-0.00	0.00
24	->	75	-0.00	0.00
24	->	76	0.00	0.00
24	->	77	0.00	0.00
24	->	78	0.00	0.00
24	->	79	0.00	0.00
24	->	80	0.00	0.00
24	->	81	0.00	0.00
24	->	82	0.00	0.00
24	->	83	0.00	0.00
24	->	84	0.00	0.00
24	->	85	0.00	0.00

24	->	86	0.00	0.00
24	->	87	0.00	0.00
24	->	88	0.00	0.00
24	->	89	-0.00	0.00
24	->	90	-0.00	0.01
24	->	91	0.00	0.02
24	->	92	0.08	0.06
24	->	93	-0.00	0.00
24	->	94	-0.00	0.00
24	->	95	-0.00	0.00
24	->	96	-0.00	0.00
24	->	97	-0.00	0.00
24	->	98	-0.01	0.00
24	->	99	-0.00	0.00
24	->	100	-0.00	0.00
24	->	101	-0.00	0.00
24	->	102	-0.00	0.00
24	->	103	-0.00	0.00
24	->	104	-0.00	0.00
24	->	105	-0.00	0.00
24	->	106	-0.00	0.00
24	->	107	0.00	0.00
25	->	1	0.05	0.01
25	->	2	-0.00	0.00
25	->	3	-0.00	0.00
25	->	4	-0.00	0.00
25	->	5	-0.06	0.01
25	->	6	-0.01	0.01
25	->	7	-0.00	0.00
25	->	8	-0.02	0.00
25	->	9	-0.00	0.00
25	->	10	-0.04	0.00
25	->	11	-0.02	0.00
25	->	12	0.02	0.01
25	->	13	0.02	0.00
25	->	14	-0.02	0.01
25	->	15	-0.00	0.00
25	->	16	0.03	0.01
25	->	17	-0.02	0.02
25	->	18	-1.74	3.11
25	->	19	-0.14	0.04
25	->	20	-0.16	0.08
25	->	21	-3.50	0.65
25	->	22	-2.75	0.65
25	->	23	-1.35	0.27
25	->	24	-10.27	0.48
25	->	25	-103.85	3.20
25	->	26	-12.70	0.46
25	->	27	-0.61	0.19
25	->	28	-1.54	0.30

25 ->	29	-1.29	0.42
25 ->	30	-5.13	1.66
25 ->	31	0.01	0.09
25 ->	32	-1.55	0.46
25 ->	33	-0.80	0.45
25 ->	34	-0.01	0.00
25 ->	35	-0.03	0.00
25 ->	36	0.02	0.00
25 ->	37	-0.00	0.00
25 ->	38	-0.02	0.00
25 ->	39	-0.00	0.00
25 ->	40	0.03	0.01
25 ->	41	0.03	0.01
25 ->	42	0.03	0.04
25 ->	43	-2.96	2.09
25 ->	44	-0.43	0.31
25 ->	45	-0.17	0.12
25 ->	46	-0.11	0.06
25 ->	47	-0.02	0.02
25 ->	48	-0.01	0.00
25 ->	49	0.00	0.00
25 ->	50	0.00	0.00
25 ->	51	0.00	0.00
25 ->	52	0.00	0.00
25 ->	53	0.02	0.00
25 ->	54	0.00	0.00
25 ->	55	-0.00	0.00
25 ->	56	0.00	0.00
25 ->	57	0.00	0.00
25 ->	58	-0.01	0.00
25 ->	59	0.01	0.00
25 ->	60	0.00	0.00
25 ->	61	0.00	0.00
25 ->	62	-0.00	0.00
25 ->	63	-0.00	0.00
25 ->	64	-0.00	0.00
25 ->	65	-0.00	0.00
25 ->	66	-0.00	0.00
25 ->	67	-0.00	0.00
25 ->	68	0.00	0.00
25 ->	69	0.00	0.00
25 ->	70	0.00	0.00
25 ->	71	-0.00	0.00
25 ->	72	0.03	0.01
25 ->	73	0.00	0.00
25 ->	74	-0.02	0.00
25 ->	75	-0.01	0.00
25 ->	76	0.01	0.00
25 ->	77	0.01	0.00
25 ->	78	0.01	0.00

25	->	79	0.00	0.00
25	->	80	0.00	0.00
25	->	81	0.01	0.00
25	->	82	0.00	0.00
25	->	83	0.00	0.00
25	->	84	0.00	0.00
25	->	85	0.00	0.00
25	->	86	-0.00	0.00
25	->	87	-0.01	0.00
25	->	88	-0.02	0.00
25	->	89	-0.03	0.01
25	->	90	-0.16	0.05
25	->	91	-0.52	0.17
25	->	92	-0.19	0.07
25	->	93	-0.06	0.01
25	->	94	-0.02	0.00
25	->	95	-0.02	0.00
25	->	96	-0.01	0.00
25	->	97	-0.02	0.04
25	->	98	-0.13	0.18
25	->	99	-0.11	0.09
25	->	100	-0.06	0.02
25	->	101	-0.02	0.00
25	->	102	-0.01	0.00
25	->	103	-0.01	0.00
25	->	104	-0.01	0.00
25	->	105	-0.01	0.00
25	->	106	-0.01	0.00
25	->	107	-0.01	0.00
26	->	1	0.00	0.00
26	->	2	-0.00	0.00
26	->	3	-0.00	0.00
26	->	4	-0.00	0.00
26	->	5	-0.06	0.03
26	->	6	-0.01	0.00
26	->	7	-0.00	0.01
26	->	8	-0.00	0.00
26	->	9	-0.00	0.00
26	->	10	-0.05	0.01
26	->	11	-0.01	0.01
26	->	12	-0.02	0.01
26	->	13	-0.00	0.00
26	->	14	-0.04	0.01
26	->	15	-0.00	0.00
26	->	16	-0.00	0.00
26	->	17	-0.00	0.00
26	->	18	-0.01	0.00
26	->	19	-0.04	0.01
26	->	20	-0.06	0.02
26	->	21	-0.08	0.05

26 ->	22	-1.96	0.78
26 ->	23	-3.24	0.55
26 ->	24	-1.09	0.17
26 ->	25	-12.74	0.46
26 ->	26	18.17	1.56
26 ->	27	-21.88	0.50
26 ->	28	-0.62	0.16
26 ->	29	-0.89	0.33
26 ->	30	-0.29	0.12
26 ->	31	-0.12	0.06
26 ->	32	-0.03	0.01
26 ->	33	-0.00	0.00
26 ->	34	-0.00	0.00
26 ->	35	-0.00	0.00
26 ->	36	0.00	0.00
26 ->	37	0.00	0.00
26 ->	38	0.00	0.00
26 ->	39	-0.00	0.00
26 ->	40	-0.01	0.00
26 ->	41	-0.02	0.00
26 ->	42	-0.02	0.01
26 ->	43	-0.50	0.33
26 ->	44	-0.07	0.04
26 ->	45	0.00	0.00
26 ->	46	-0.00	0.00
26 ->	47	-0.03	0.02
26 ->	48	-0.00	0.00
26 ->	49	-0.00	0.00
26 ->	50	-0.00	0.00
26 ->	51	-0.00	0.00
26 ->	52	0.00	0.00
26 ->	53	0.00	0.00
26 ->	54	0.00	0.00
26 ->	55	0.00	0.00
26 ->	56	0.00	0.00
26 ->	57	0.00	0.00
26 ->	58	-0.00	0.00
26 ->	59	0.00	0.00
26 ->	60	0.00	0.00
26 ->	61	0.00	0.00
26 ->	62	0.00	0.00
26 ->	63	-0.00	0.00
26 ->	64	-0.00	0.00
26 ->	65	-0.00	0.00
26 ->	66	-0.00	0.00
26 ->	67	0.00	0.00
26 ->	68	0.00	0.00
26 ->	69	0.00	0.00
26 ->	70	0.00	0.00
26 ->	71	-0.00	0.00

26	->	72	0.00	0.00
26	->	73	0.00	0.00
26	->	74	-0.00	0.00
26	->	75	-0.00	0.00
26	->	76	0.00	0.00
26	->	77	0.00	0.00
26	->	78	0.00	0.00
26	->	79	0.00	0.00
26	->	80	0.00	0.00
26	->	81	0.00	0.00
26	->	82	0.00	0.00
26	->	83	0.00	0.00
26	->	84	0.00	0.00
26	->	85	0.00	0.00
26	->	86	0.00	0.00
26	->	87	-0.00	0.00
26	->	88	-0.00	0.00
26	->	89	-0.01	0.00
26	->	90	-0.12	0.06
26	->	91	-2.69	0.56
26	->	92	-0.91	0.30
26	->	93	-0.02	0.00
26	->	94	-0.00	0.00
26	->	95	-0.00	0.00
26	->	96	-0.00	0.00
26	->	97	-0.00	0.00
26	->	98	-0.00	0.00
26	->	99	-0.00	0.00
26	->	100	-0.00	0.00
26	->	101	-0.00	0.00
26	->	102	-0.00	0.00
26	->	103	-0.00	0.00
26	->	104	-0.00	0.00
26	->	105	-0.00	0.00
26	->	106	-0.00	0.00
26	->	107	-0.00	0.00
27	->	1	-0.01	0.00
27	->	2	-0.00	0.00
27	->	3	-0.01	0.00
27	->	4	-0.08	0.02
27	->	5	2.03	1.18
27	->	6	-0.24	0.19
27	->	7	-1.23	1.31
27	->	8	0.01	0.02
27	->	9	-0.04	0.03
27	->	10	1.79	0.79
27	->	11	0.01	0.01
27	->	12	-0.04	0.01
27	->	13	-0.03	0.01
27	->	14	-0.01	0.01

27	->	15	0.00	0.00
27	->	16	-0.00	0.00
27	->	17	-0.00	0.00
27	->	18	-0.01	0.00
27	->	19	-0.02	0.00
27	->	20	-0.06	0.02
27	->	21	-0.06	0.03
27	->	22	-0.15	0.05
27	->	23	30.34	18.33
27	->	24	-2.87	0.41
27	->	25	-0.62	0.19
27	->	26	-21.97	0.50
27	->	27	3.55	1.70
27	->	28	-4.79	0.48
27	->	29	-0.51	0.11
27	->	30	-0.02	0.04
27	->	31	-0.02	0.01
27	->	32	-0.00	0.00
27	->	33	-0.00	0.00
27	->	34	-0.00	0.00
27	->	35	-0.00	0.00
27	->	36	0.00	0.00
27	->	37	-0.00	0.00
27	->	38	-0.00	0.00
27	->	39	-0.00	0.00
27	->	40	0.00	0.00
27	->	41	-0.00	0.00
27	->	42	-0.01	0.00
27	->	43	-0.01	0.01
27	->	44	-0.00	0.00
27	->	45	0.00	0.00
27	->	46	-0.00	0.00
27	->	47	-0.00	0.00
27	->	48	0.00	0.00
27	->	49	0.00	0.00
27	->	50	0.00	0.00
27	->	51	0.00	0.00
27	->	52	0.00	0.00
27	->	53	0.00	0.00
27	->	54	0.00	0.00
27	->	55	-0.00	0.00
27	->	56	0.00	0.00
27	->	57	0.00	0.00
27	->	58	-0.00	0.00
27	->	59	0.00	0.00
27	->	60	0.00	0.00
27	->	61	0.00	0.00
27	->	62	0.00	0.00
27	->	63	-0.00	0.00
27	->	64	-0.00	0.00

27	->	65	0.00	0.00
27	->	66	-0.00	0.00
27	->	67	0.00	0.00
27	->	68	0.00	0.00
27	->	69	0.00	0.00
27	->	70	0.00	0.00
27	->	71	0.00	0.00
27	->	72	0.00	0.00
27	->	73	0.00	0.00
27	->	74	-0.00	0.00
27	->	75	-0.00	0.00
27	->	76	0.00	0.00
27	->	77	0.00	0.00
27	->	78	0.00	0.00
27	->	79	0.00	0.00
27	->	80	0.00	0.00
27	->	81	0.00	0.00
27	->	82	0.00	0.00
27	->	83	0.00	0.00
27	->	84	0.00	0.00
27	->	85	0.00	0.00
27	->	86	-0.00	0.00
27	->	87	-0.00	0.00
27	->	88	-0.00	0.00
27	->	89	-0.00	0.00
27	->	90	0.01	0.01
27	->	91	0.04	0.04
27	->	92	0.04	0.07
27	->	93	-0.00	0.00
27	->	94	-0.00	0.00
27	->	95	-0.00	0.00
27	->	96	-0.00	0.00
27	->	97	-0.00	0.00
27	->	98	-0.00	0.00
27	->	99	-0.00	0.00
27	->	100	-0.00	0.00
27	->	101	-0.00	0.00
27	->	102	-0.00	0.00
27	->	103	-0.00	0.00
27	->	104	-0.00	0.00
27	->	105	-0.00	0.00
27	->	106	-0.00	0.00
27	->	107	-0.00	0.00
28	->	1	0.00	0.00
28	->	2	-0.00	0.00
28	->	3	-0.00	0.00
28	->	4	-0.00	0.00
28	->	5	-0.02	0.01
28	->	6	-0.03	0.04
28	->	7	-0.02	0.02

28	->	8	-0.00	0.00
28	->	9	-0.00	0.00
28	->	10	-0.01	0.00
28	->	11	-0.00	0.00
28	->	12	0.00	0.00
28	->	13	0.00	0.00
28	->	14	-0.00	0.00
28	->	15	0.00	0.00
28	->	16	0.00	0.00
28	->	17	-0.00	0.00
28	->	18	-0.01	0.00
28	->	19	-0.01	0.00
28	->	20	-0.02	0.00
28	->	21	-0.03	0.02
28	->	22	-0.03	0.01
28	->	23	-0.23	0.07
28	->	24	-5.47	0.81
28	->	25	-1.55	0.30
28	->	26	-0.63	0.16
28	->	27	-4.96	0.48
28	->	28	-22.74	1.42
28	->	29	-11.69	0.44
28	->	30	-3.99	1.24
28	->	31	-0.04	0.01
28	->	32	-0.02	0.00
28	->	33	-0.00	0.00
28	->	34	-0.00	0.00
28	->	35	0.00	0.00
28	->	36	-0.00	0.00
28	->	37	0.00	0.00
28	->	38	0.00	0.00
28	->	39	0.00	0.00
28	->	40	-0.00	0.00
28	->	41	-0.00	0.00
28	->	42	-0.00	0.00
28	->	43	-0.01	0.01
28	->	44	-0.00	0.00
28	->	45	-0.00	0.00
28	->	46	-0.00	0.00
28	->	47	-0.00	0.00
28	->	48	-0.00	0.00
28	->	49	0.00	0.00
28	->	50	0.00	0.00
28	->	51	0.00	0.00
28	->	52	-0.00	0.00
28	->	53	0.00	0.00
28	->	54	0.00	0.00
28	->	55	0.00	0.00
28	->	56	0.00	0.00
28	->	57	0.00	0.00

28	->	58	0.00	0.00
28	->	59	0.00	0.00
28	->	60	0.00	0.00
28	->	61	0.00	0.00
28	->	62	-0.00	0.00
28	->	63	-0.00	0.00
28	->	64	-0.00	0.00
28	->	65	-0.00	0.00
28	->	66	-0.00	0.00
28	->	67	0.00	0.00
28	->	68	0.00	0.00
28	->	69	0.00	0.00
28	->	70	0.00	0.00
28	->	71	-0.00	0.00
28	->	72	0.00	0.00
28	->	73	0.00	0.00
28	->	74	-0.00	0.00
28	->	75	-0.00	0.00
28	->	76	0.00	0.00
28	->	77	0.00	0.00
28	->	78	0.00	0.00
28	->	79	0.00	0.00
28	->	80	0.00	0.00
28	->	81	0.00	0.00
28	->	82	0.00	0.00
28	->	83	0.00	0.00
28	->	84	0.00	0.00
28	->	85	0.00	0.00
28	->	86	0.00	0.00
28	->	87	-0.00	0.00
28	->	88	0.00	0.00
28	->	89	-0.00	0.00
28	->	90	-0.01	0.00
28	->	91	-0.02	0.01
28	->	92	-0.01	0.00
28	->	93	-0.00	0.00
28	->	94	-0.00	0.00
28	->	95	-0.00	0.00
28	->	96	-0.00	0.00
28	->	97	-0.00	0.00
28	->	98	-0.00	0.00
28	->	99	-0.00	0.00
28	->	100	-0.00	0.00
28	->	101	-0.00	0.00
28	->	102	-0.00	0.00
28	->	103	-0.00	0.00
28	->	104	-0.00	0.00
28	->	105	-0.00	0.00
28	->	106	-0.00	0.00
28	->	107	-0.00	0.00

29	->	1	0.00	0.00
29	->	2	-0.00	0.00
29	->	3	-0.00	0.00
29	->	4	0.00	0.00
29	->	5	-0.00	0.00
29	->	6	-0.00	0.00
29	->	7	-0.00	0.00
29	->	8	-0.00	0.00
29	->	9	0.00	0.00
29	->	10	-0.00	0.00
29	->	11	-0.00	0.00
29	->	12	0.00	0.00
29	->	13	0.00	0.00
29	->	14	-0.00	0.00
29	->	15	0.00	0.00
29	->	16	0.00	0.00
29	->	17	-0.00	0.00
29	->	18	-0.00	0.00
29	->	19	-0.00	0.00
29	->	20	-0.00	0.00
29	->	21	-0.01	0.01
29	->	22	-0.01	0.01
29	->	23	-0.03	0.02
29	->	24	-0.17	0.10
29	->	25	-1.29	0.42
29	->	26	-0.93	0.34
29	->	27	-0.52	0.11
29	->	28	-11.76	0.44
29	->	29	12.10	0.61
29	->	30	-14.39	0.51
29	->	31	-0.94	0.57
29	->	32	-0.03	0.00
29	->	33	-0.01	0.00
29	->	34	-0.00	0.00
29	->	35	0.00	0.00
29	->	36	-0.00	0.00
29	->	37	0.00	0.00
29	->	38	0.00	0.00
29	->	39	-0.00	0.00
29	->	40	-0.00	0.00
29	->	41	-0.00	0.01
29	->	42	0.00	0.01
29	->	43	-0.01	0.02
29	->	44	-0.01	0.00
29	->	45	-0.00	0.00
29	->	46	-0.00	0.00
29	->	47	-0.00	0.00
29	->	48	-0.00	0.00
29	->	49	0.00	0.00
29	->	50	-0.00	0.00

29	->	51	0.00	0.00
29	->	52	0.00	0.00
29	->	53	0.00	0.00
29	->	54	0.00	0.00
29	->	55	0.00	0.00
29	->	56	-0.00	0.00
29	->	57	0.00	0.00
29	->	58	0.00	0.00
29	->	59	0.00	0.00
29	->	60	0.00	0.00
29	->	61	0.00	0.00
29	->	62	0.00	0.00
29	->	63	-0.00	0.00
29	->	64	-0.00	0.00
29	->	65	0.00	0.00
29	->	66	-0.00	0.00
29	->	67	0.00	0.00
29	->	68	0.00	0.00
29	->	69	0.00	0.00
29	->	70	0.00	0.00
29	->	71	0.00	0.00
29	->	72	0.00	0.00
29	->	73	0.00	0.00
29	->	74	-0.00	0.00
29	->	75	-0.00	0.00
29	->	76	0.00	0.00
29	->	77	0.00	0.00
29	->	78	0.00	0.00
29	->	79	0.00	0.00
29	->	80	0.00	0.00
29	->	81	0.00	0.00
29	->	82	0.00	0.00
29	->	83	0.00	0.00
29	->	84	0.00	0.00
29	->	85	0.00	0.00
29	->	86	-0.00	0.00
29	->	87	-0.00	0.00
29	->	88	-0.00	0.00
29	->	89	-0.00	0.00
29	->	90	-0.02	0.01
29	->	91	-0.04	0.02
29	->	92	-0.01	0.00
29	->	93	-0.00	0.00
29	->	94	-0.00	0.00
29	->	95	-0.00	0.00
29	->	96	-0.00	0.00
29	->	97	-0.00	0.00
29	->	98	-0.00	0.00
29	->	99	-0.00	0.00
29	->	100	-0.00	0.00

29	->	101	-0.00	0.00
29	->	102	-0.00	0.00
29	->	103	-0.00	0.00
29	->	104	-0.00	0.00
29	->	105	-0.00	0.00
29	->	106	-0.00	0.00
29	->	107	-0.00	0.00
30	->	1	-0.00	0.00
30	->	2	-0.00	0.00
30	->	3	-0.00	0.00
30	->	4	-0.00	0.00
30	->	5	-0.01	0.00
30	->	6	-0.00	0.00
30	->	7	-0.00	0.00
30	->	8	-0.00	0.00
30	->	9	0.00	0.00
30	->	10	-0.00	0.00
30	->	11	0.00	0.00
30	->	12	-0.00	0.00
30	->	13	-0.00	0.00
30	->	14	-0.00	0.00
30	->	15	-0.00	0.00
30	->	16	-0.00	0.00
30	->	17	-0.00	0.00
30	->	18	0.00	0.00
30	->	19	0.00	0.00
30	->	20	-0.01	0.00
30	->	21	-0.07	0.06
30	->	22	-0.04	0.01
30	->	23	-0.03	0.01
30	->	24	-0.62	0.28
30	->	25	-5.10	1.64
30	->	26	-0.29	0.12
30	->	27	-0.02	0.04
30	->	28	-4.01	1.24
30	->	29	-14.56	0.51
30	->	30	-53.20	2.26
30	->	31	-11.16	0.34
30	->	32	-1.32	0.40
30	->	33	-0.10	0.02
30	->	34	-0.01	0.00
30	->	35	-0.00	0.00
30	->	36	-0.00	0.00
30	->	37	0.00	0.00
30	->	38	0.00	0.00
30	->	39	-0.00	0.00
30	->	40	0.00	0.00
30	->	41	0.04	0.02
30	->	42	-0.20	0.06
30	->	43	-0.76	0.36

30 ->	44	-0.00	0.01
30 ->	45	-0.01	0.00
30 ->	46	0.00	0.00
30 ->	47	-0.01	0.00
30 ->	48	-0.01	0.00
30 ->	49	-0.00	0.00
30 ->	50	-0.00	0.00
30 ->	51	-0.00	0.00
30 ->	52	-0.00	0.00
30 ->	53	-0.00	0.00
30 ->	54	-0.00	0.00
30 ->	55	0.00	0.00
30 ->	56	-0.00	0.00
30 ->	57	-0.00	0.00
30 ->	58	0.00	0.00
30 ->	59	-0.00	0.00
30 ->	60	-0.00	0.00
30 ->	61	-0.00	0.00
30 ->	62	0.00	0.00
30 ->	63	0.00	0.00
30 ->	64	0.00	0.00
30 ->	65	0.00	0.00
30 ->	66	0.00	0.00
30 ->	67	0.00	0.00
30 ->	68	-0.00	0.00
30 ->	69	-0.00	0.00
30 ->	70	-0.00	0.00
30 ->	71	0.00	0.00
30 ->	72	-0.00	0.00
30 ->	73	-0.00	0.00
30 ->	74	0.00	0.00
30 ->	75	0.00	0.00
30 ->	76	-0.00	0.00
30 ->	77	-0.00	0.00
30 ->	78	-0.00	0.00
30 ->	79	-0.00	0.00
30 ->	80	-0.00	0.00
30 ->	81	-0.00	0.00
30 ->	82	-0.00	0.00
30 ->	83	0.00	0.00
30 ->	84	0.00	0.00
30 ->	85	-0.00	0.00
30 ->	86	0.00	0.00
30 ->	87	0.00	0.00
30 ->	88	0.00	0.00
30 ->	89	-0.00	0.00
30 ->	90	-0.01	0.01
30 ->	91	0.01	0.01
30 ->	92	-0.00	0.00
30 ->	93	0.00	0.00

30	->	94	-0.00	0.00
30	->	95	0.00	0.00
30	->	96	0.00	0.00
30	->	97	-0.00	0.00
30	->	98	0.00	0.00
30	->	99	0.00	0.00
30	->	100	0.00	0.00
30	->	101	0.00	0.00
30	->	102	0.00	0.00
30	->	103	0.00	0.00
30	->	104	0.00	0.00
30	->	105	0.00	0.00
30	->	106	-0.00	0.00
30	->	107	0.00	0.00
31	->	1	0.02	0.00
31	->	2	-0.00	0.00
31	->	3	-0.00	0.00
31	->	4	0.00	0.00
31	->	5	-0.02	0.00
31	->	6	0.00	0.00
31	->	7	-0.00	0.00
31	->	8	-0.01	0.00
31	->	9	0.00	0.00
31	->	10	-0.02	0.00
31	->	11	-0.01	0.00
31	->	12	0.02	0.00
31	->	13	0.01	0.00
31	->	14	-0.00	0.00
31	->	15	0.00	0.00
31	->	16	0.01	0.00
31	->	17	-0.00	0.00
31	->	18	-0.04	0.01
31	->	19	-0.02	0.00
31	->	20	-0.00	0.00
31	->	21	-0.00	0.00
31	->	22	0.03	0.01
31	->	23	-0.01	0.00
31	->	24	-0.00	0.00
31	->	25	0.00	0.09
31	->	26	-0.12	0.06
31	->	27	-0.02	0.01
31	->	28	-0.04	0.01
31	->	29	-0.99	0.58
31	->	30	-11.21	0.34
31	->	31	10.49	2.74
31	->	32	-0.21	0.44
31	->	33	-0.91	0.33
31	->	34	-0.11	0.03
31	->	35	-0.08	0.01
31	->	36	0.01	0.00

31	->	37	-0.00	0.00
31	->	38	-0.06	0.01
31	->	39	-0.01	0.01
31	->	40	0.27	0.23
31	->	41	-2.16	0.88
31	->	42	-3.29	0.41
31	->	43	-2.19	0.54
31	->	44	-0.08	0.04
31	->	45	-0.08	0.02
31	->	46	-0.06	0.01
31	->	47	0.00	0.02
31	->	48	-0.04	0.01
31	->	49	-0.00	0.00
31	->	50	-0.00	0.00
31	->	51	-0.00	0.00
31	->	52	-0.00	0.00
31	->	53	0.02	0.00
31	->	54	0.00	0.00
31	->	55	-0.00	0.00
31	->	56	0.00	0.00
31	->	57	0.00	0.00
31	->	58	-0.01	0.00
31	->	59	0.01	0.00
31	->	60	0.00	0.00
31	->	61	-0.00	0.00
31	->	62	-0.00	0.00
31	->	63	-0.00	0.00
31	->	64	-0.00	0.00
31	->	65	-0.00	0.00
31	->	66	-0.00	0.00
31	->	67	-0.00	0.00
31	->	68	0.01	0.00
31	->	69	0.00	0.00
31	->	70	0.00	0.00
31	->	71	0.00	0.00
31	->	72	0.03	0.00
31	->	73	0.00	0.00
31	->	74	-0.02	0.00
31	->	75	-0.02	0.00
31	->	76	0.01	0.00
31	->	77	0.01	0.00
31	->	78	0.02	0.00
31	->	79	0.00	0.00
31	->	80	0.00	0.00
31	->	81	0.01	0.00
31	->	82	0.00	0.00
31	->	83	0.00	0.00
31	->	84	0.00	0.00
31	->	85	0.00	0.00
31	->	86	-0.00	0.00

31	->	87	-0.01	0.00
31	->	88	-0.05	0.01
31	->	89	-0.36	0.38
31	->	90	-2.10	2.21
31	->	91	-0.52	1.08
31	->	92	-0.05	0.01
31	->	93	-0.02	0.00
31	->	94	-0.01	0.00
31	->	95	-0.01	0.00
31	->	96	-0.00	0.00
31	->	97	-0.01	0.00
31	->	98	-0.02	0.01
31	->	99	-0.03	0.01
31	->	100	-0.02	0.00
31	->	101	-0.01	0.00
31	->	102	-0.01	0.00
31	->	103	-0.01	0.00
31	->	104	-0.01	0.00
31	->	105	-0.02	0.00
31	->	106	-0.02	0.01
31	->	107	-0.02	0.01
32	->	1	0.00	0.00
32	->	2	0.00	0.00
32	->	3	-0.00	0.00
32	->	4	-0.00	0.00
32	->	5	-0.00	0.00
32	->	6	-0.00	0.00
32	->	7	-0.00	0.00
32	->	8	-0.00	0.00
32	->	9	0.00	0.00
32	->	10	-0.00	0.00
32	->	11	-0.00	0.00
32	->	12	0.00	0.00
32	->	13	0.00	0.00
32	->	14	-0.00	0.00
32	->	15	0.00	0.00
32	->	16	0.00	0.00
32	->	17	-0.00	0.00
32	->	18	-0.01	0.01
32	->	19	-0.01	0.00
32	->	20	-0.00	0.00
32	->	21	-0.02	0.01
32	->	22	-0.03	0.01
32	->	23	-0.01	0.00
32	->	24	-0.01	0.00
32	->	25	-1.54	0.47
32	->	26	-0.03	0.01
32	->	27	-0.00	0.00
32	->	28	-0.02	0.00
32	->	29	-0.03	0.00

32	->	30	-1.35	0.41
32	->	31	-0.25	0.45
32	->	32	1.72	0.66
32	->	33	-10.36	0.56
32	->	34	-0.49	0.16
32	->	35	-0.02	0.00
32	->	36	-0.01	0.00
32	->	37	-0.00	0.00
32	->	38	0.00	0.00
32	->	39	-0.01	0.00
32	->	40	-0.02	0.01
32	->	41	-0.32	0.21
32	->	42	-0.82	0.15
32	->	43	-0.73	0.24
32	->	44	-0.17	0.06
32	->	45	-0.06	0.02
32	->	46	-0.01	0.00
32	->	47	-0.01	0.00
32	->	48	-0.04	0.01
32	->	49	-0.00	0.00
32	->	50	-0.00	0.00
32	->	51	-0.00	0.00
32	->	52	-0.00	0.00
32	->	53	-0.00	0.00
32	->	54	0.00	0.00
32	->	55	-0.00	0.00
32	->	56	-0.00	0.00
32	->	57	-0.00	0.00
32	->	58	0.00	0.00
32	->	59	-0.00	0.00
32	->	60	0.00	0.00
32	->	61	0.00	0.00
32	->	62	0.00	0.00
32	->	63	-0.00	0.00
32	->	64	-0.00	0.00
32	->	65	0.00	0.00
32	->	66	-0.00	0.00
32	->	67	0.00	0.00
32	->	68	0.00	0.00
32	->	69	-0.00	0.00
32	->	70	0.00	0.00
32	->	71	-0.00	0.00
32	->	72	-0.00	0.00
32	->	73	0.00	0.00
32	->	74	-0.00	0.00
32	->	75	-0.00	0.00
32	->	76	0.00	0.00
32	->	77	0.00	0.00
32	->	78	0.00	0.00
32	->	79	0.00	0.00

32	->	80	0.00	0.00
32	->	81	0.00	0.00
32	->	82	0.00	0.00
32	->	83	0.00	0.00
32	->	84	0.00	0.00
32	->	85	0.00	0.00
32	->	86	-0.00	0.00
32	->	87	-0.00	0.00
32	->	88	-0.00	0.00
32	->	89	-0.00	0.00
32	->	90	-0.01	0.00
32	->	91	-0.01	0.00
32	->	92	-0.01	0.00
32	->	93	-0.00	0.00
32	->	94	-0.00	0.00
32	->	95	-0.00	0.00
32	->	96	-0.00	0.00
32	->	97	-0.00	0.01
32	->	98	-0.02	0.04
32	->	99	-0.01	0.01
32	->	100	-0.00	0.00
32	->	101	-0.00	0.00
32	->	102	-0.00	0.00
32	->	103	-0.00	0.00
32	->	104	-0.00	0.00
32	->	105	-0.00	0.00
32	->	106	-0.00	0.00
32	->	107	-0.00	0.00
33	->	1	0.00	0.00
33	->	2	0.00	0.00
33	->	3	-0.00	0.00
33	->	4	-0.00	0.00
33	->	5	-0.00	0.00
33	->	6	-0.00	0.00
33	->	7	0.00	0.00
33	->	8	0.00	0.00
33	->	9	0.00	0.00
33	->	10	0.00	0.00
33	->	11	0.00	0.00
33	->	12	-0.00	0.00
33	->	13	0.00	0.00
33	->	14	-0.00	0.00
33	->	15	-0.00	0.00
33	->	16	-0.00	0.00
33	->	17	-0.00	0.00
33	->	18	-0.03	0.04
33	->	19	-0.00	0.00
33	->	20	-0.01	0.00
33	->	21	-0.03	0.02
33	->	22	-0.06	0.03

33 ->	23	-0.00	0.00
33 ->	24	-0.01	0.00
33 ->	25	-0.79	0.44
33 ->	26	-0.00	0.00
33 ->	27	-0.00	0.00
33 ->	28	-0.00	0.00
33 ->	29	-0.01	0.00
33 ->	30	-0.10	0.02
33 ->	31	-0.92	0.33
33 ->	32	-10.50	0.55
33 ->	33	28.09	1.24
33 ->	34	-12.84	0.43
33 ->	35	-0.46	0.08
33 ->	36	-0.03	0.01
33 ->	37	-0.01	0.00
33 ->	38	-0.01	0.00
33 ->	39	-0.02	0.01
33 ->	40	-0.33	0.08
33 ->	41	-2.84	0.63
33 ->	42	-3.41	0.46
33 ->	43	-0.85	0.21
33 ->	44	-2.49	0.57
33 ->	45	-1.93	0.42
33 ->	46	0.00	0.02
33 ->	47	-0.08	0.03
33 ->	48	-1.38	0.35
33 ->	49	-0.04	0.01
33 ->	50	-0.01	0.00
33 ->	51	-0.02	0.00
33 ->	52	-0.02	0.00
33 ->	53	-0.00	0.00
33 ->	54	-0.00	0.00
33 ->	55	-0.01	0.00
33 ->	56	-0.00	0.00
33 ->	57	0.00	0.00
33 ->	58	-0.00	0.00
33 ->	59	0.00	0.00
33 ->	60	-0.00	0.00
33 ->	61	-0.00	0.00
33 ->	62	0.00	0.00
33 ->	63	-0.00	0.00
33 ->	64	-0.00	0.00
33 ->	65	0.00	0.00
33 ->	66	-0.00	0.00
33 ->	67	0.00	0.00
33 ->	68	0.00	0.00
33 ->	69	0.00	0.00
33 ->	70	-0.00	0.00
33 ->	71	-0.00	0.00
33 ->	72	-0.00	0.00

33	->	73	0.00	0.00
33	->	74	-0.00	0.00
33	->	75	-0.00	0.00
33	->	76	0.00	0.00
33	->	77	0.00	0.00
33	->	78	0.00	0.00
33	->	79	0.00	0.00
33	->	80	0.00	0.00
33	->	81	0.00	0.00
33	->	82	0.00	0.00
33	->	83	0.00	0.00
33	->	84	0.00	0.00
33	->	85	0.00	0.00
33	->	86	0.00	0.00
33	->	87	-0.00	0.00
33	->	88	-0.00	0.00
33	->	89	-0.01	0.01
33	->	90	-0.01	0.01
33	->	91	-0.01	0.00
33	->	92	-0.00	0.00
33	->	93	-0.00	0.00
33	->	94	-0.00	0.00
33	->	95	-0.00	0.00
33	->	96	-0.00	0.00
33	->	97	-0.01	0.01
33	->	98	-0.29	0.51
33	->	99	-0.42	0.40
33	->	100	-0.03	0.01
33	->	101	-0.01	0.00
33	->	102	-0.00	0.00
33	->	103	-0.00	0.00
33	->	104	-0.00	0.00
33	->	105	-0.00	0.00
33	->	106	-0.00	0.00
33	->	107	-0.00	0.00
34	->	1	0.00	0.00
34	->	2	0.00	0.00
34	->	3	-0.00	0.00
34	->	4	0.00	0.00
34	->	5	-0.00	0.00
34	->	6	0.00	0.00
34	->	7	0.00	0.00
34	->	8	-0.00	0.00
34	->	9	0.00	0.00
34	->	10	-0.00	0.00
34	->	11	-0.00	0.00
34	->	12	0.00	0.00
34	->	13	0.00	0.00
34	->	14	0.00	0.00
34	->	15	0.00	0.00

34 ->	16	-0.00	0.00
34 ->	17	-0.00	0.00
34 ->	18	-0.00	0.00
34 ->	19	-0.00	0.00
34 ->	20	-0.00	0.00
34 ->	21	-0.00	0.00
34 ->	22	-0.00	0.00
34 ->	23	-0.00	0.00
34 ->	24	-0.00	0.00
34 ->	25	-0.01	0.00
34 ->	26	-0.00	0.00
34 ->	27	-0.00	0.00
34 ->	28	-0.00	0.00
34 ->	29	-0.00	0.00
34 ->	30	-0.01	0.00
34 ->	31	-0.11	0.03
34 ->	32	-0.51	0.17
34 ->	33	-12.83	0.43
34 ->	34	-54.41	2.38
34 ->	35	-12.72	1.38
34 ->	36	-0.60	0.42
34 ->	37	-0.08	0.08
34 ->	38	-0.10	0.06
34 ->	39	-2.44	1.38
34 ->	40	-2.15	0.46
34 ->	41	-3.04	0.71
34 ->	42	-0.93	0.30
34 ->	43	-0.04	0.01
34 ->	44	-0.02	0.01
34 ->	45	-0.02	0.01
34 ->	46	-0.00	0.00
34 ->	47	0.03	0.01
34 ->	48	-1.07	0.32
34 ->	49	-0.01	0.00
34 ->	50	0.00	0.00
34 ->	51	-0.10	0.02
34 ->	52	-0.04	0.02
34 ->	53	-0.01	0.00
34 ->	54	-0.00	0.00
34 ->	55	-0.06	0.01
34 ->	56	-0.00	0.00
34 ->	57	-0.00	0.00
34 ->	58	0.00	0.00
34 ->	59	0.00	0.00
34 ->	60	-0.00	0.00
34 ->	61	0.00	0.00
34 ->	62	0.00	0.00
34 ->	63	0.00	0.00
34 ->	64	-0.00	0.00
34 ->	65	0.00	0.00

34	->	66	-0.00	0.00
34	->	67	0.00	0.00
34	->	68	0.00	0.00
34	->	69	-0.00	0.00
34	->	70	0.00	0.00
34	->	71	0.00	0.00
34	->	72	-0.00	0.00
34	->	73	-0.00	0.00
34	->	74	-0.00	0.00
34	->	75	-0.00	0.00
34	->	76	-0.00	0.00
34	->	77	0.00	0.00
34	->	78	0.00	0.00
34	->	79	0.00	0.00
34	->	80	0.00	0.00
34	->	81	0.00	0.00
34	->	82	0.00	0.00
34	->	83	0.00	0.00
34	->	84	0.00	0.00
34	->	85	0.00	0.00
34	->	86	-0.00	0.00
34	->	87	-0.00	0.00
34	->	88	-0.01	0.01
34	->	89	-0.03	0.01
34	->	90	-0.02	0.01
34	->	91	-0.00	0.00
34	->	92	-0.00	0.00
34	->	93	-0.00	0.00
34	->	94	0.00	0.00
34	->	95	-0.00	0.00
34	->	96	0.00	0.00
34	->	97	-0.00	0.00
34	->	98	-0.00	0.00
34	->	99	-0.00	0.00
34	->	100	-0.00	0.00
34	->	101	-0.00	0.00
34	->	102	-0.00	0.00
34	->	103	-0.00	0.00
34	->	104	-0.00	0.00
34	->	105	-0.00	0.00
34	->	106	-0.00	0.00
34	->	107	-0.00	0.00
35	->	1	-0.01	0.00
35	->	2	-0.00	0.00
35	->	3	-0.00	0.00
35	->	4	0.00	0.00
35	->	5	0.00	0.00
35	->	6	-0.00	0.00
35	->	7	-0.00	0.00
35	->	8	0.00	0.00

35	->	9	0.00	0.00
35	->	10	0.00	0.00
35	->	11	0.00	0.00
35	->	12	-0.00	0.00
35	->	13	-0.00	0.00
35	->	14	-0.00	0.00
35	->	15	-0.00	0.00
35	->	16	-0.01	0.00
35	->	17	0.00	0.00
35	->	18	0.02	0.00
35	->	19	0.01	0.00
35	->	20	0.00	0.00
35	->	21	-0.00	0.00
35	->	22	-0.02	0.00
35	->	23	-0.00	0.00
35	->	24	0.00	0.00
35	->	25	-0.03	0.00
35	->	26	-0.00	0.00
35	->	27	-0.00	0.00
35	->	28	0.00	0.00
35	->	29	0.00	0.00
35	->	30	-0.00	0.00
35	->	31	-0.08	0.01
35	->	32	-0.02	0.00
35	->	33	-0.46	0.08
35	->	34	-12.79	1.38
35	->	35	-24.23	2.89
35	->	36	-9.80	0.82
35	->	37	2.57	1.73
35	->	38	2.80	0.54
35	->	39	-2.46	0.43
35	->	40	-2.71	0.43
35	->	41	-0.41	0.09
35	->	42	-0.64	0.20
35	->	43	0.01	0.01
35	->	44	-0.05	0.01
35	->	45	0.00	0.01
35	->	46	0.03	0.01
35	->	47	-0.15	0.03
35	->	48	-1.09	0.23
35	->	49	0.01	0.02
35	->	50	-0.07	0.03
35	->	51	2.85	0.63
35	->	52	-0.35	0.29
35	->	53	-0.05	0.02
35	->	54	-0.04	0.06
35	->	55	2.39	0.36
35	->	56	-0.01	0.01
35	->	57	-0.00	0.00
35	->	58	0.03	0.01

35	->	59	-0.03	0.01
35	->	60	-0.00	0.00
35	->	61	0.00	0.00
35	->	62	0.00	0.00
35	->	63	0.01	0.00
35	->	64	0.00	0.00
35	->	65	0.00	0.00
35	->	66	0.01	0.00
35	->	67	0.00	0.00
35	->	68	-0.01	0.00
35	->	69	-0.01	0.00
35	->	70	-0.00	0.00
35	->	71	-0.00	0.00
35	->	72	-0.03	0.00
35	->	73	-0.00	0.00
35	->	74	0.02	0.00
35	->	75	0.02	0.00
35	->	76	-0.01	0.00
35	->	77	-0.01	0.00
35	->	78	-0.01	0.00
35	->	79	-0.00	0.00
35	->	80	-0.00	0.00
35	->	81	-0.01	0.00
35	->	82	-0.00	0.00
35	->	83	0.00	0.00
35	->	84	-0.00	0.00
35	->	85	-0.00	0.00
35	->	86	0.00	0.00
35	->	87	0.02	0.00
35	->	88	0.11	0.04
35	->	89	0.19	0.06
35	->	90	0.05	0.01
35	->	91	0.03	0.00
35	->	92	0.01	0.00
35	->	93	0.01	0.00
35	->	94	0.00	0.00
35	->	95	0.00	0.00
35	->	96	0.00	0.00
35	->	97	0.00	0.00
35	->	98	0.02	0.01
35	->	99	0.03	0.01
35	->	100	0.03	0.01
35	->	101	0.02	0.00
35	->	102	0.01	0.00
35	->	103	0.01	0.00
35	->	104	0.01	0.00
35	->	105	0.01	0.00
35	->	106	0.00	0.00
35	->	107	0.01	0.00
36	->	1	0.01	0.00

36	->	2	0.00	0.00
36	->	3	0.00	0.00
36	->	4	0.00	0.00
36	->	5	-0.00	0.00
36	->	6	0.00	0.00
36	->	7	0.00	0.00
36	->	8	-0.00	0.00
36	->	9	0.00	0.00
36	->	10	-0.00	0.00
36	->	11	-0.00	0.00
36	->	12	0.00	0.00
36	->	13	0.00	0.00
36	->	14	0.00	0.00
36	->	15	0.00	0.00
36	->	16	0.01	0.00
36	->	17	-0.00	0.00
36	->	18	-0.01	0.00
36	->	19	-0.01	0.00
36	->	20	-0.00	0.00
36	->	21	0.00	0.00
36	->	22	0.01	0.00
36	->	23	0.00	0.00
36	->	24	0.00	0.00
36	->	25	0.02	0.00
36	->	26	0.00	0.00
36	->	27	0.00	0.00
36	->	28	-0.00	0.00
36	->	29	-0.00	0.00
36	->	30	-0.00	0.00
36	->	31	0.01	0.00
36	->	32	-0.01	0.00
36	->	33	-0.03	0.01
36	->	34	-0.62	0.43
36	->	35	-9.86	0.84
36	->	36	-115.64	2.59
36	->	37	-14.42	1.08
36	->	38	-0.83	0.25
36	->	39	-1.11	0.80
36	->	40	-0.00	0.01
36	->	41	-0.03	0.01
36	->	42	-0.03	0.01
36	->	43	-0.00	0.00
36	->	44	0.01	0.00
36	->	45	-0.01	0.00
36	->	46	-0.03	0.01
36	->	47	-0.01	0.00
36	->	48	-0.17	0.13
36	->	49	-0.02	0.02
36	->	50	-0.00	0.00
36	->	51	-0.14	0.03

36	->	52	-0.71	0.82
36	->	53	0.02	0.01
36	->	54	-0.00	0.01
36	->	55	-0.30	0.08
36	->	56	-0.09	0.19
36	->	57	-0.00	0.00
36	->	58	-0.05	0.03
36	->	59	0.02	0.01
36	->	60	0.00	0.00
36	->	61	-0.00	0.00
36	->	62	-0.00	0.00
36	->	63	-0.01	0.00
36	->	64	-0.00	0.00
36	->	65	-0.00	0.00
36	->	66	-0.00	0.00
36	->	67	-0.00	0.00
36	->	68	0.01	0.00
36	->	69	0.01	0.00
36	->	70	-0.00	0.00
36	->	71	-0.00	0.00
36	->	72	0.02	0.00
36	->	73	0.00	0.00
36	->	74	-0.01	0.00
36	->	75	-0.01	0.00
36	->	76	0.01	0.00
36	->	77	0.01	0.00
36	->	78	0.01	0.00
36	->	79	0.00	0.00
36	->	80	0.00	0.00
36	->	81	0.00	0.00
36	->	82	0.00	0.00
36	->	83	-0.00	0.00
36	->	84	0.00	0.00
36	->	85	0.00	0.00
36	->	86	-0.00	0.00
36	->	87	-0.01	0.00
36	->	88	-0.03	0.01
36	->	89	-0.04	0.01
36	->	90	-0.02	0.00
36	->	91	-0.02	0.00
36	->	92	-0.01	0.00
36	->	93	-0.01	0.00
36	->	94	-0.00	0.00
36	->	95	-0.00	0.00
36	->	96	-0.00	0.00
36	->	97	-0.01	0.00
36	->	98	-0.03	0.01
36	->	99	-0.06	0.03
36	->	100	-0.05	0.02
36	->	101	-0.02	0.01

36	->	102	-0.01	0.00
36	->	103	-0.00	0.00
36	->	104	-0.00	0.00
36	->	105	-0.00	0.00
36	->	106	-0.00	0.00
36	->	107	-0.00	0.00
37	->	1	-0.00	0.00
37	->	2	0.00	0.00
37	->	3	0.00	0.00
37	->	4	-0.00	0.00
37	->	5	0.00	0.00
37	->	6	0.00	0.00
37	->	7	0.00	0.00
37	->	8	0.00	0.00
37	->	9	0.00	0.00
37	->	10	0.00	0.00
37	->	11	0.00	0.00
37	->	12	-0.00	0.00
37	->	13	-0.00	0.00
37	->	14	-0.00	0.00
37	->	15	-0.00	0.00
37	->	16	-0.00	0.00
37	->	17	0.00	0.00
37	->	18	0.00	0.00
37	->	19	0.00	0.00
37	->	20	0.00	0.00
37	->	21	0.00	0.00
37	->	22	-0.00	0.00
37	->	23	-0.00	0.00
37	->	24	0.00	0.00
37	->	25	-0.00	0.00
37	->	26	0.00	0.00
37	->	27	-0.00	0.00
37	->	28	0.00	0.00
37	->	29	0.00	0.00
37	->	30	0.00	0.00
37	->	31	-0.00	0.00
37	->	32	-0.00	0.00
37	->	33	-0.01	0.00
37	->	34	-0.08	0.08
37	->	35	2.50	1.74
37	->	36	-14.54	1.06
37	->	37	22.88	1.57
37	->	38	-7.90	0.99
37	->	39	-0.83	0.42
37	->	40	-0.23	0.06
37	->	41	-0.01	0.00
37	->	42	-0.02	0.01
37	->	43	-0.00	0.00
37	->	44	-0.00	0.00

37	->	45	-0.00	0.00
37	->	46	0.00	0.00
37	->	47	-0.01	0.00
37	->	48	-0.04	0.01
37	->	49	-0.01	0.00
37	->	50	-0.01	0.00
37	->	51	0.77	0.11
37	->	52	-0.32	0.34
37	->	53	-0.01	0.00
37	->	54	-0.03	0.01
37	->	55	0.47	0.76
37	->	56	-0.10	0.09
37	->	57	-0.01	0.00
37	->	58	-0.00	0.00
37	->	59	-0.00	0.00
37	->	60	-0.00	0.00
37	->	61	-0.00	0.00
37	->	62	0.00	0.00
37	->	63	0.00	0.00
37	->	64	0.00	0.00
37	->	65	0.00	0.00
37	->	66	0.00	0.00
37	->	67	0.00	0.00
37	->	68	-0.00	0.00
37	->	69	-0.00	0.00
37	->	70	-0.00	0.00
37	->	71	-0.00	0.00
37	->	72	-0.00	0.00
37	->	73	0.00	0.00
37	->	74	0.00	0.00
37	->	75	0.00	0.00
37	->	76	-0.00	0.00
37	->	77	-0.00	0.00
37	->	78	-0.00	0.00
37	->	79	-0.00	0.00
37	->	80	0.00	0.00
37	->	81	-0.00	0.00
37	->	82	0.00	0.00
37	->	83	0.00	0.00
37	->	84	0.00	0.00
37	->	85	-0.00	0.00
37	->	86	-0.00	0.00
37	->	87	-0.00	0.00
37	->	88	0.00	0.00
37	->	89	0.01	0.01
37	->	90	0.00	0.00
37	->	91	0.00	0.00
37	->	92	0.00	0.00
37	->	93	0.00	0.00
37	->	94	0.00	0.00

37	->	95	0.00	0.00
37	->	96	0.00	0.00
37	->	97	0.00	0.00
37	->	98	-0.00	0.00
37	->	99	-0.00	0.00
37	->	100	-0.00	0.00
37	->	101	-0.00	0.00
37	->	102	0.00	0.00
37	->	103	0.00	0.00
37	->	104	0.00	0.00
37	->	105	0.00	0.00
37	->	106	0.00	0.00
37	->	107	0.00	0.00
38	->	1	-0.01	0.00
38	->	2	-0.00	0.00
38	->	3	0.00	0.00
38	->	4	-0.00	0.00
38	->	5	0.00	0.00
38	->	6	-0.00	0.00
38	->	7	0.00	0.00
38	->	8	0.00	0.00
38	->	9	-0.00	0.00
38	->	10	0.00	0.00
38	->	11	0.00	0.00
38	->	12	-0.00	0.00
38	->	13	-0.00	0.00
38	->	14	-0.00	0.00
38	->	15	-0.00	0.00
38	->	16	-0.00	0.00
38	->	17	0.00	0.00
38	->	18	0.01	0.00
38	->	19	0.01	0.00
38	->	20	0.00	0.00
38	->	21	0.00	0.00
38	->	22	-0.01	0.00
38	->	23	-0.00	0.00
38	->	24	0.00	0.00
38	->	25	-0.02	0.00
38	->	26	0.00	0.00
38	->	27	-0.00	0.00
38	->	28	0.00	0.00
38	->	29	0.00	0.00
38	->	30	0.00	0.00
38	->	31	-0.06	0.01
38	->	32	0.00	0.00
38	->	33	-0.01	0.00
38	->	34	-0.10	0.06
38	->	35	2.80	0.54
38	->	36	-0.84	0.25
38	->	37	-7.83	1.00

38 ->	38	-33.55	1.87
38 ->	39	-11.83	0.56
38 ->	40	-3.12	0.69
38 ->	41	0.02	0.02
38 ->	42	0.02	0.04
38 ->	43	0.00	0.00
38 ->	44	-0.02	0.00
38 ->	45	0.00	0.00
38 ->	46	0.03	0.00
38 ->	47	-0.03	0.01
38 ->	48	-0.01	0.01
38 ->	49	-0.00	0.00
38 ->	50	-0.02	0.01
38 ->	51	3.32	0.73
38 ->	52	0.04	0.02
38 ->	53	-0.04	0.01
38 ->	54	-0.03	0.04
38 ->	55	2.02	0.29
38 ->	56	-0.01	0.00
38 ->	57	-0.00	0.00
38 ->	58	0.02	0.00
38 ->	59	-0.03	0.01
38 ->	60	-0.00	0.00
38 ->	61	0.00	0.00
38 ->	62	-0.00	0.00
38 ->	63	0.01	0.00
38 ->	64	0.00	0.00
38 ->	65	0.00	0.00
38 ->	66	0.01	0.00
38 ->	67	0.00	0.00
38 ->	68	-0.01	0.00
38 ->	69	-0.01	0.00
38 ->	70	-0.00	0.00
38 ->	71	-0.00	0.00
38 ->	72	-0.03	0.01
38 ->	73	-0.00	0.00
38 ->	74	0.02	0.00
38 ->	75	0.02	0.00
38 ->	76	-0.01	0.00
38 ->	77	-0.01	0.00
38 ->	78	-0.02	0.00
38 ->	79	-0.00	0.00
38 ->	80	-0.00	0.00
38 ->	81	-0.01	0.00
38 ->	82	-0.00	0.00
38 ->	83	0.00	0.00
38 ->	84	-0.00	0.00
38 ->	85	-0.00	0.00
38 ->	86	0.00	0.00
38 ->	87	0.03	0.00

38	->	88	0.13	0.04
38	->	89	0.29	0.11
38	->	90	0.05	0.01
38	->	91	0.03	0.00
38	->	92	0.01	0.00
38	->	93	0.01	0.00
38	->	94	0.00	0.00
38	->	95	0.00	0.00
38	->	96	0.00	0.00
38	->	97	0.00	0.00
38	->	98	0.01	0.00
38	->	99	0.02	0.00
38	->	100	0.02	0.00
38	->	101	0.01	0.00
38	->	102	0.01	0.00
38	->	103	0.00	0.00
38	->	104	0.01	0.00
38	->	105	0.01	0.00
38	->	106	0.00	0.00
38	->	107	0.01	0.00
39	->	1	-0.00	0.00
39	->	2	0.00	0.00
39	->	3	0.00	0.00
39	->	4	0.00	0.00
39	->	5	0.00	0.00
39	->	6	0.00	0.00
39	->	7	0.00	0.00
39	->	8	0.00	0.00
39	->	9	0.00	0.00
39	->	10	0.00	0.00
39	->	11	0.00	0.00
39	->	12	-0.00	0.00
39	->	13	-0.00	0.00
39	->	14	0.00	0.00
39	->	15	0.00	0.00
39	->	16	-0.00	0.00
39	->	17	0.00	0.00
39	->	18	0.00	0.00
39	->	19	0.00	0.00
39	->	20	0.00	0.00
39	->	21	0.00	0.00
39	->	22	-0.00	0.00
39	->	23	0.00	0.00
39	->	24	0.00	0.00
39	->	25	-0.00	0.00
39	->	26	-0.00	0.00
39	->	27	-0.00	0.00
39	->	28	0.00	0.00
39	->	29	-0.00	0.00
39	->	30	-0.00	0.00

39	->	31	-0.01	0.01
39	->	32	-0.01	0.00
39	->	33	-0.02	0.01
39	->	34	-2.42	1.39
39	->	35	-2.49	0.43
39	->	36	-1.10	0.79
39	->	37	-0.84	0.41
39	->	38	-11.95	0.55
39	->	39	12.31	1.13
39	->	40	-9.56	0.36
39	->	41	-0.53	0.22
39	->	42	-0.12	0.03
39	->	43	-0.01	0.00
39	->	44	-0.00	0.00
39	->	45	-0.00	0.00
39	->	46	-0.00	0.00
39	->	47	0.00	0.00
39	->	48	-0.02	0.01
39	->	49	-0.00	0.00
39	->	50	-0.00	0.00
39	->	51	-0.10	0.05
39	->	52	-0.02	0.01
39	->	53	-0.00	0.00
39	->	54	-0.00	0.00
39	->	55	-0.05	0.05
39	->	56	-0.00	0.00
39	->	57	0.00	0.00
39	->	58	-0.00	0.00
39	->	59	0.00	0.00
39	->	60	0.00	0.00
39	->	61	-0.00	0.00
39	->	62	0.00	0.00
39	->	63	-0.00	0.00
39	->	64	-0.00	0.00
39	->	65	0.00	0.00
39	->	66	-0.00	0.00
39	->	67	-0.00	0.00
39	->	68	0.00	0.00
39	->	69	0.00	0.00
39	->	70	-0.00	0.00
39	->	71	-0.00	0.00
39	->	72	0.00	0.00
39	->	73	-0.00	0.00
39	->	74	-0.00	0.00
39	->	75	-0.00	0.00
39	->	76	0.00	0.00
39	->	77	0.00	0.00
39	->	78	0.00	0.00
39	->	79	0.00	0.00
39	->	80	0.00	0.00

39	->	81	0.00	0.00
39	->	82	0.00	0.00
39	->	83	0.00	0.00
39	->	84	0.00	0.00
39	->	85	0.00	0.00
39	->	86	-0.00	0.00
39	->	87	-0.00	0.00
39	->	88	-0.01	0.00
39	->	89	-0.02	0.01
39	->	90	-0.01	0.00
39	->	91	-0.00	0.00
39	->	92	0.00	0.00
39	->	93	0.00	0.00
39	->	94	-0.00	0.00
39	->	95	0.00	0.00
39	->	96	-0.00	0.00
39	->	97	0.00	0.00
39	->	98	-0.00	0.00
39	->	99	-0.00	0.00
39	->	100	-0.00	0.00
39	->	101	-0.00	0.00
39	->	102	-0.00	0.00
39	->	103	-0.00	0.00
39	->	104	-0.00	0.00
39	->	105	0.00	0.00
39	->	106	-0.00	0.00
39	->	107	-0.00	0.00
40	->	1	0.01	0.00
40	->	2	-0.00	0.00
40	->	3	-0.00	0.00
40	->	4	0.00	0.00
40	->	5	-0.01	0.00
40	->	6	0.00	0.00
40	->	7	-0.00	0.00
40	->	8	-0.00	0.00
40	->	9	-0.00	0.00
40	->	10	-0.01	0.00
40	->	11	-0.01	0.00
40	->	12	0.01	0.00
40	->	13	0.00	0.00
40	->	14	0.00	0.00
40	->	15	0.00	0.00
40	->	16	0.01	0.00
40	->	17	-0.00	0.00
40	->	18	-0.02	0.00
40	->	19	-0.01	0.00
40	->	20	-0.00	0.00
40	->	21	-0.00	0.00
40	->	22	0.02	0.00
40	->	23	-0.00	0.00

40	->	24	-0.00	0.00
40	->	25	0.03	0.01
40	->	26	-0.01	0.00
40	->	27	0.00	0.00
40	->	28	-0.00	0.00
40	->	29	-0.00	0.00
40	->	30	0.00	0.00
40	->	31	0.26	0.23
40	->	32	-0.02	0.01
40	->	33	-0.33	0.08
40	->	34	-2.13	0.46
40	->	35	-2.71	0.44
40	->	36	-0.00	0.01
40	->	37	-0.23	0.06
40	->	38	-3.23	0.69
40	->	39	-9.61	0.37
40	->	40	-102.25	4.39
40	->	41	-16.56	1.87
40	->	42	-4.23	0.77
40	->	43	-0.18	0.13
40	->	44	0.03	0.01
40	->	45	-0.02	0.01
40	->	46	-0.04	0.01
40	->	47	-0.01	0.07
40	->	48	-0.20	0.06
40	->	49	-0.00	0.01
40	->	50	-0.00	0.04
40	->	51	-1.25	0.47
40	->	52	-0.04	0.02
40	->	53	0.03	0.01
40	->	54	-0.02	0.02
40	->	55	-0.20	0.07
40	->	56	0.00	0.00
40	->	57	0.00	0.00
40	->	58	-0.02	0.00
40	->	59	0.03	0.01
40	->	60	0.00	0.00
40	->	61	-0.00	0.00
40	->	62	-0.00	0.00
40	->	63	-0.01	0.00
40	->	64	-0.00	0.00
40	->	65	-0.00	0.00
40	->	66	-0.01	0.00
40	->	67	-0.00	0.00
40	->	68	0.01	0.00
40	->	69	0.01	0.00
40	->	70	0.00	0.00
40	->	71	0.00	0.00
40	->	72	0.04	0.01
40	->	73	-0.00	0.00

40	->	74	-0.03	0.00
40	->	75	-0.03	0.01
40	->	76	0.01	0.00
40	->	77	0.01	0.00
40	->	78	0.03	0.00
40	->	79	0.00	0.00
40	->	80	0.00	0.00
40	->	81	0.01	0.00
40	->	82	0.00	0.00
40	->	83	0.00	0.00
40	->	84	0.00	0.00
40	->	85	0.00	0.00
40	->	86	-0.01	0.00
40	->	87	-0.04	0.02
40	->	88	-0.88	0.79
40	->	89	-7.20	3.21
40	->	90	-4.40	2.83
40	->	91	-0.09	0.03
40	->	92	-0.03	0.00
40	->	93	-0.01	0.00
40	->	94	-0.01	0.00
40	->	95	-0.00	0.00
40	->	96	-0.00	0.00
40	->	97	-0.00	0.00
40	->	98	-0.01	0.00
40	->	99	-0.02	0.01
40	->	100	-0.02	0.01
40	->	101	-0.01	0.00
40	->	102	-0.01	0.00
40	->	103	-0.01	0.00
40	->	104	-0.01	0.00
40	->	105	-0.02	0.00
40	->	106	-0.01	0.01
40	->	107	-0.03	0.01
41	->	1	0.00	0.00
41	->	2	0.00	0.00
41	->	3	0.00	0.00
41	->	4	0.00	0.00
41	->	5	-0.00	0.00
41	->	6	0.00	0.00
41	->	7	0.00	0.00
41	->	8	-0.00	0.00
41	->	9	0.00	0.00
41	->	10	-0.00	0.00
41	->	11	-0.00	0.00
41	->	12	0.00	0.00
41	->	13	0.00	0.00
41	->	14	0.00	0.00
41	->	15	0.00	0.00
41	->	16	0.00	0.00

41	->	17	-0.00	0.00
41	->	18	-0.00	0.00
41	->	19	-0.00	0.00
41	->	20	0.00	0.00
41	->	21	0.00	0.00
41	->	22	0.01	0.00
41	->	23	-0.00	0.00
41	->	24	0.00	0.00
41	->	25	0.03	0.01
41	->	26	-0.02	0.00
41	->	27	-0.00	0.00
41	->	28	-0.00	0.00
41	->	29	-0.00	0.01
41	->	30	0.04	0.02
41	->	31	-2.15	0.88
41	->	32	-0.32	0.21
41	->	33	-2.84	0.63
41	->	34	-2.99	0.71
41	->	35	-0.41	0.09
41	->	36	-0.03	0.01
41	->	37	-0.01	0.00
41	->	38	0.02	0.02
41	->	39	-0.54	0.23
41	->	40	-16.54	1.88
41	->	41	-30.92	1.78
41	->	42	-8.57	0.46
41	->	43	-0.54	0.07
41	->	44	-0.02	0.03
41	->	45	-0.05	0.01
41	->	46	-0.01	0.01
41	->	47	0.06	0.04
41	->	48	-0.08	0.03
41	->	49	-0.00	0.01
41	->	50	-0.00	0.01
41	->	51	-0.01	0.01
41	->	52	-0.01	0.00
41	->	53	-0.00	0.00
41	->	54	-0.00	0.00
41	->	55	-0.01	0.01
41	->	56	-0.00	0.00
41	->	57	-0.00	0.00
41	->	58	0.00	0.00
41	->	59	-0.00	0.00
41	->	60	-0.00	0.00
41	->	61	0.00	0.00
41	->	62	0.00	0.00
41	->	63	0.00	0.00
41	->	64	0.00	0.00
41	->	65	0.00	0.00
41	->	66	0.00	0.00

41	->	67	0.00	0.00
41	->	68	-0.00	0.00
41	->	69	-0.00	0.00
41	->	70	0.00	0.00
41	->	71	0.00	0.00
41	->	72	0.00	0.00
41	->	73	-0.00	0.00
41	->	74	0.00	0.00
41	->	75	0.00	0.00
41	->	76	-0.00	0.00
41	->	77	0.00	0.00
41	->	78	-0.00	0.00
41	->	79	-0.00	0.00
41	->	80	0.00	0.00
41	->	81	-0.00	0.00
41	->	82	-0.00	0.00
41	->	83	0.00	0.00
41	->	84	-0.00	0.00
41	->	85	-0.00	0.00
41	->	86	0.00	0.00
41	->	87	-0.00	0.00
41	->	88	-0.02	0.01
41	->	89	-0.06	0.05
41	->	90	-0.13	0.09
41	->	91	-0.04	0.02
41	->	92	-0.00	0.00
41	->	93	-0.00	0.00
41	->	94	-0.00	0.00
41	->	95	-0.00	0.00
41	->	96	-0.00	0.00
41	->	97	-0.00	0.00
41	->	98	-0.00	0.00
41	->	99	-0.01	0.00
41	->	100	-0.00	0.00
41	->	101	-0.00	0.00
41	->	102	-0.00	0.00
41	->	103	-0.00	0.00
41	->	104	-0.00	0.00
41	->	105	-0.00	0.00
41	->	106	-0.00	0.00
41	->	107	-0.00	0.00
42	->	1	0.00	0.00
42	->	2	0.00	0.00
42	->	3	0.00	0.00
42	->	4	0.00	0.00
42	->	5	-0.00	0.00
42	->	6	-0.00	0.00
42	->	7	0.00	0.00
42	->	8	-0.00	0.00
42	->	9	0.00	0.00

42	->	10	-0.00	0.00
42	->	11	-0.00	0.00
42	->	12	0.00	0.00
42	->	13	0.00	0.00
42	->	14	-0.00	0.00
42	->	15	-0.00	0.00
42	->	16	0.00	0.00
42	->	17	0.00	0.00
42	->	18	-0.01	0.01
42	->	19	-0.00	0.00
42	->	20	-0.00	0.00
42	->	21	0.00	0.00
42	->	22	0.02	0.02
42	->	23	-0.01	0.00
42	->	24	0.00	0.00
42	->	25	0.03	0.04
42	->	26	-0.02	0.01
42	->	27	-0.01	0.00
42	->	28	-0.00	0.00
42	->	29	0.00	0.01
42	->	30	-0.20	0.06
42	->	31	-3.27	0.41
42	->	32	-0.82	0.15
42	->	33	-3.42	0.46
42	->	34	-0.93	0.30
42	->	35	-0.63	0.20
42	->	36	-0.03	0.01
42	->	37	-0.02	0.01
42	->	38	0.02	0.04
42	->	39	-0.12	0.03
42	->	40	-4.18	0.77
42	->	41	-8.63	0.47
42	->	42	29.86	1.28
42	->	43	-17.29	0.26
42	->	44	-1.00	0.19
42	->	45	-0.20	0.04
42	->	46	-0.09	0.02
42	->	47	-1.88	0.58
42	->	48	-2.58	0.36
42	->	49	-0.08	0.02
42	->	50	-0.10	0.05
42	->	51	-1.92	0.31
42	->	52	-0.08	0.03
42	->	53	-0.02	0.01
42	->	54	-0.01	0.02
42	->	55	0.01	0.01
42	->	56	-0.00	0.00
42	->	57	-0.00	0.00
42	->	58	-0.00	0.00
42	->	59	-0.00	0.00

42	->	60	-0.00	0.00
42	->	61	-0.00	0.00
42	->	62	0.00	0.00
42	->	63	-0.00	0.00
42	->	64	-0.00	0.00
42	->	65	-0.00	0.00
42	->	66	-0.00	0.00
42	->	67	0.00	0.00
42	->	68	0.00	0.00
42	->	69	0.00	0.00
42	->	70	-0.00	0.00
42	->	71	-0.00	0.00
42	->	72	-0.01	0.01
42	->	73	-0.00	0.00
42	->	74	-0.00	0.00
42	->	75	-0.00	0.00
42	->	76	0.00	0.00
42	->	77	0.00	0.00
42	->	78	0.00	0.00
42	->	79	0.00	0.00
42	->	80	0.00	0.00
42	->	81	0.00	0.00
42	->	82	0.00	0.00
42	->	83	0.00	0.00
42	->	84	0.00	0.00
42	->	85	0.00	0.00
42	->	86	-0.00	0.00
42	->	87	-0.01	0.00
42	->	88	0.02	0.04
42	->	89	-1.25	0.48
42	->	90	-1.27	0.34
42	->	91	-0.31	0.10
42	->	92	-0.02	0.01
42	->	93	-0.00	0.00
42	->	94	-0.00	0.00
42	->	95	-0.00	0.00
42	->	96	-0.00	0.00
42	->	97	-0.00	0.00
42	->	98	-0.01	0.00
42	->	99	-0.01	0.01
42	->	100	-0.01	0.00
42	->	101	-0.00	0.00
42	->	102	-0.00	0.00
42	->	103	-0.00	0.00
42	->	104	-0.00	0.00
42	->	105	-0.00	0.00
42	->	106	-0.01	0.00
42	->	107	-0.00	0.00
43	->	1	0.00	0.00
43	->	2	-0.00	0.00

43 ->	3	-0.00	0.00
43 ->	4	0.00	0.00
43 ->	5	-0.00	0.00
43 ->	6	-0.00	0.00
43 ->	7	-0.00	0.00
43 ->	8	-0.00	0.00
43 ->	9	-0.00	0.00
43 ->	10	-0.00	0.00
43 ->	11	-0.00	0.00
43 ->	12	0.00	0.00
43 ->	13	-0.00	0.00
43 ->	14	-0.00	0.00
43 ->	15	-0.00	0.00
43 ->	16	-0.00	0.00
43 ->	17	-0.00	0.00
43 ->	18	-0.01	0.02
43 ->	19	-0.01	0.00
43 ->	20	-0.01	0.00
43 ->	21	-0.06	0.03
43 ->	22	-0.37	0.35
43 ->	23	-0.01	0.01
43 ->	24	-0.02	0.01
43 ->	25	-2.93	2.09
43 ->	26	-0.50	0.32
43 ->	27	-0.01	0.01
43 ->	28	-0.01	0.01
43 ->	29	-0.01	0.02
43 ->	30	-0.77	0.36
43 ->	31	-2.14	0.53
43 ->	32	-0.72	0.23
43 ->	33	-0.84	0.21
43 ->	34	-0.04	0.01
43 ->	35	0.01	0.01
43 ->	36	-0.00	0.00
43 ->	37	-0.00	0.00
43 ->	38	0.00	0.00
43 ->	39	-0.01	0.00
43 ->	40	-0.18	0.13
43 ->	41	-0.54	0.07
43 ->	42	-17.28	0.26
43 ->	43	23.08	1.71
43 ->	44	-6.83	0.52
43 ->	45	-0.15	0.05
43 ->	46	-0.08	0.04
43 ->	47	-0.82	0.36
43 ->	48	-0.02	0.02
43 ->	49	-0.01	0.00
43 ->	50	-0.00	0.01
43 ->	51	-0.01	0.00
43 ->	52	-0.00	0.00

43	->	53	-0.00	0.00
43	->	54	-0.00	0.00
43	->	55	0.00	0.00
43	->	56	-0.00	0.00
43	->	57	0.00	0.00
43	->	58	0.00	0.00
43	->	59	0.00	0.00
43	->	60	-0.00	0.00
43	->	61	0.00	0.00
43	->	62	0.00	0.00
43	->	63	-0.00	0.00
43	->	64	-0.00	0.00
43	->	65	-0.00	0.00
43	->	66	-0.00	0.00
43	->	67	0.00	0.00
43	->	68	0.00	0.00
43	->	69	0.00	0.00
43	->	70	-0.00	0.00
43	->	71	0.00	0.00
43	->	72	-0.00	0.01
43	->	73	-0.00	0.00
43	->	74	-0.00	0.00
43	->	75	-0.00	0.00
43	->	76	0.00	0.00
43	->	77	0.00	0.00
43	->	78	0.00	0.00
43	->	79	0.00	0.00
43	->	80	0.00	0.00
43	->	81	0.00	0.00
43	->	82	0.00	0.00
43	->	83	0.00	0.00
43	->	84	0.00	0.00
43	->	85	0.00	0.00
43	->	86	-0.00	0.00
43	->	87	-0.00	0.00
43	->	88	0.00	0.01
43	->	89	0.01	0.02
43	->	90	-0.60	0.46
43	->	91	-3.73	1.58
43	->	92	-0.04	0.02
43	->	93	-0.01	0.00
43	->	94	-0.00	0.00
43	->	95	-0.00	0.00
43	->	96	-0.00	0.00
43	->	97	-0.00	0.00
43	->	98	-0.00	0.01
43	->	99	-0.01	0.01
43	->	100	-0.01	0.01
43	->	101	0.00	0.00
43	->	102	-0.00	0.00

43	->	103	-0.00	0.00
43	->	104	-0.00	0.00
43	->	105	-0.00	0.00
43	->	106	-0.01	0.00
43	->	107	-0.00	0.00
44	->	1	0.09	0.04
44	->	2	0.00	0.00
44	->	3	-0.00	0.00
44	->	4	-0.00	0.00
44	->	5	-0.02	0.00
44	->	6	0.00	0.00
44	->	7	-0.00	0.00
44	->	8	-0.01	0.00
44	->	9	-0.00	0.00
44	->	10	-0.02	0.00
44	->	11	-0.02	0.00
44	->	12	0.04	0.01
44	->	13	0.02	0.00
44	->	14	-0.01	0.00
44	->	15	0.00	0.00
44	->	16	0.08	0.03
44	->	17	-0.00	0.00
44	->	18	-0.53	0.55
44	->	19	-0.15	0.06
44	->	20	-0.02	0.01
44	->	21	0.03	0.02
44	->	22	-0.64	0.71
44	->	23	-0.05	0.02
44	->	24	-0.01	0.01
44	->	25	-0.44	0.33
44	->	26	-0.07	0.04
44	->	27	-0.00	0.00
44	->	28	-0.00	0.00
44	->	29	-0.01	0.00
44	->	30	-0.00	0.01
44	->	31	-0.08	0.04
44	->	32	-0.17	0.06
44	->	33	-2.51	0.57
44	->	34	-0.02	0.01
44	->	35	-0.05	0.01
44	->	36	0.01	0.00
44	->	37	-0.00	0.00
44	->	38	-0.02	0.00
44	->	39	-0.00	0.00
44	->	40	0.03	0.01
44	->	41	-0.02	0.03
44	->	42	-1.00	0.18
44	->	43	-6.93	0.52
44	->	44	-92.59	2.41
44	->	45	-21.50	0.61

44 ->	46	-8.46	5.09
44 ->	47	-4.67	0.72
44 ->	48	-1.02	0.45
44 ->	49	0.02	0.06
44 ->	50	-0.06	0.04
44 ->	51	-0.01	0.01
44 ->	52	0.00	0.01
44 ->	53	0.06	0.01
44 ->	54	-0.00	0.00
44 ->	55	-0.00	0.00
44 ->	56	0.00	0.00
44 ->	57	0.00	0.00
44 ->	58	-0.02	0.01
44 ->	59	0.02	0.00
44 ->	60	0.00	0.00
44 ->	61	-0.00	0.00
44 ->	62	-0.00	0.00
44 ->	63	-0.01	0.00
44 ->	64	-0.01	0.00
44 ->	65	-0.00	0.00
44 ->	66	-0.01	0.00
44 ->	67	-0.00	0.00
44 ->	68	0.01	0.00
44 ->	69	0.01	0.00
44 ->	70	0.00	0.00
44 ->	71	0.00	0.00
44 ->	72	0.45	0.44
44 ->	73	-0.01	0.01
44 ->	74	-0.09	0.03
44 ->	75	-0.04	0.01
44 ->	76	0.02	0.01
44 ->	77	0.03	0.01
44 ->	78	0.03	0.02
44 ->	79	0.00	0.00
44 ->	80	0.00	0.00
44 ->	81	0.02	0.00
44 ->	82	0.00	0.00
44 ->	83	0.00	0.00
44 ->	84	0.00	0.00
44 ->	85	0.00	0.00
44 ->	86	-0.00	0.00
44 ->	87	-0.02	0.00
44 ->	88	-0.05	0.01
44 ->	89	-0.07	0.03
44 ->	90	-0.49	0.14
44 ->	91	-5.96	2.31
44 ->	92	-8.42	1.46
44 ->	93	-2.93	1.46
44 ->	94	-0.11	0.11
44 ->	95	-0.05	0.02

44	->	96	-0.02	0.01
44	->	97	-0.02	0.01
44	->	98	-0.09	0.08
44	->	99	-0.25	0.20
44	->	100	-0.98	1.03
44	->	101	0.67	0.56
44	->	102	0.46	0.42
44	->	103	0.00	0.24
44	->	104	-0.09	0.04
44	->	105	-0.05	0.01
44	->	106	-0.02	0.00
44	->	107	-0.01	0.00
45	->	1	0.00	0.00
45	->	2	0.00	0.00
45	->	3	-0.00	0.00
45	->	4	-0.00	0.00
45	->	5	-0.00	0.00
45	->	6	0.00	0.00
45	->	7	0.00	0.00
45	->	8	-0.00	0.00
45	->	9	0.00	0.00
45	->	10	-0.00	0.00
45	->	11	-0.00	0.00
45	->	12	0.00	0.00
45	->	13	0.00	0.00
45	->	14	-0.00	0.00
45	->	15	0.00	0.00
45	->	16	-0.00	0.00
45	->	17	-0.00	0.00
45	->	18	-0.02	0.06
45	->	19	-0.01	0.00
45	->	20	-0.00	0.00
45	->	21	-0.02	0.01
45	->	22	-0.08	0.04
45	->	23	0.00	0.00
45	->	24	-0.00	0.00
45	->	25	-0.17	0.12
45	->	26	0.00	0.00
45	->	27	0.00	0.00
45	->	28	-0.00	0.00
45	->	29	-0.00	0.00
45	->	30	-0.01	0.00
45	->	31	-0.08	0.02
45	->	32	-0.06	0.02
45	->	33	-1.96	0.43
45	->	34	-0.02	0.01
45	->	35	0.00	0.01
45	->	36	-0.01	0.00
45	->	37	-0.00	0.00
45	->	38	0.00	0.00

45 ->	39	-0.00	0.00
45 ->	40	-0.02	0.01
45 ->	41	-0.05	0.01
45 ->	42	-0.20	0.04
45 ->	43	-0.15	0.05
45 ->	44	-21.50	0.61
45 ->	45	23.32	2.44
45 ->	46	-10.56	0.55
45 ->	47	-1.06	0.18
45 ->	48	-2.48	0.40
45 ->	49	-3.90	1.18
45 ->	50	-0.12	0.06
45 ->	51	-0.07	0.03
45 ->	52	-0.07	0.03
45 ->	53	-0.02	0.01
45 ->	54	-0.00	0.00
45 ->	55	-0.00	0.00
45 ->	56	-0.00	0.00
45 ->	57	-0.00	0.00
45 ->	58	0.00	0.00
45 ->	59	-0.00	0.00
45 ->	60	-0.00	0.00
45 ->	61	-0.00	0.00
45 ->	62	0.00	0.00
45 ->	63	0.00	0.00
45 ->	64	0.00	0.00
45 ->	65	0.00	0.00
45 ->	66	0.00	0.00
45 ->	67	0.00	0.00
45 ->	68	-0.00	0.00
45 ->	69	-0.00	0.00
45 ->	70	0.00	0.00
45 ->	71	-0.00	0.00
45 ->	72	0.01	0.03
45 ->	73	-0.00	0.00
45 ->	74	-0.00	0.00
45 ->	75	0.00	0.00
45 ->	76	-0.00	0.00
45 ->	77	-0.00	0.00
45 ->	78	-0.00	0.00
45 ->	79	-0.00	0.00
45 ->	80	-0.00	0.00
45 ->	81	-0.00	0.00
45 ->	82	-0.00	0.00
45 ->	83	0.00	0.00
45 ->	84	0.00	0.00
45 ->	85	-0.00	0.00
45 ->	86	0.00	0.00
45 ->	87	0.00	0.00
45 ->	88	-0.00	0.01

45	->	89	-0.01	0.01
45	->	90	-0.01	0.01
45	->	91	0.01	0.02
45	->	92	-0.02	0.02
45	->	93	-0.01	0.01
45	->	94	-0.01	0.01
45	->	95	-0.01	0.01
45	->	96	-0.00	0.00
45	->	97	-0.01	0.01
45	->	98	-0.29	0.35
45	->	99	-2.29	2.29
45	->	100	-0.60	0.48
45	->	101	-0.01	0.03
45	->	102	-0.00	0.01
45	->	103	-0.00	0.00
45	->	104	-0.00	0.00
45	->	105	-0.00	0.00
45	->	106	-0.00	0.00
45	->	107	0.00	0.00
46	->	1	-0.04	0.01
46	->	2	-0.00	0.00
46	->	3	0.00	0.00
46	->	4	-0.00	0.00
46	->	5	0.01	0.00
46	->	6	-0.00	0.00
46	->	7	-0.00	0.00
46	->	8	0.01	0.00
46	->	9	0.00	0.00
46	->	10	0.01	0.00
46	->	11	0.01	0.00
46	->	12	-0.02	0.00
46	->	13	-0.01	0.00
46	->	14	-0.00	0.00
46	->	15	-0.00	0.00
46	->	16	-0.04	0.01
46	->	17	-0.00	0.00
46	->	18	0.09	0.05
46	->	19	0.04	0.01
46	->	20	-0.00	0.00
46	->	21	-0.01	0.01
46	->	22	-0.48	0.36
46	->	23	-0.00	0.00
46	->	24	-0.00	0.00
46	->	25	-0.11	0.06
46	->	26	-0.00	0.00
46	->	27	-0.00	0.00
46	->	28	-0.00	0.00
46	->	29	-0.00	0.00
46	->	30	0.00	0.00
46	->	31	-0.06	0.01

46 ->	32	-0.01	0.00
46 ->	33	0.00	0.02
46 ->	34	-0.00	0.00
46 ->	35	0.03	0.01
46 ->	36	-0.03	0.01
46 ->	37	0.00	0.00
46 ->	38	0.03	0.00
46 ->	39	-0.00	0.00
46 ->	40	-0.04	0.01
46 ->	41	-0.01	0.01
46 ->	42	-0.09	0.02
46 ->	43	-0.08	0.04
46 ->	44	-8.42	5.06
46 ->	45	-10.61	0.55
46 ->	46	-12.83	4.11
46 ->	47	-12.88	0.50
46 ->	48	-1.31	0.20
46 ->	49	-3.31	0.60
46 ->	50	-2.61	1.28
46 ->	51	-0.18	0.10
46 ->	52	-0.07	0.03
46 ->	53	-0.27	0.09
46 ->	54	-0.01	0.01
46 ->	55	-0.01	0.00
46 ->	56	-0.01	0.00
46 ->	57	-0.00	0.00
46 ->	58	0.03	0.01
46 ->	59	-0.04	0.01
46 ->	60	-0.01	0.00
46 ->	61	-0.01	0.00
46 ->	62	0.00	0.00
46 ->	63	0.01	0.00
46 ->	64	0.01	0.00
46 ->	65	0.00	0.00
46 ->	66	0.01	0.00
46 ->	67	0.00	0.00
46 ->	68	-0.02	0.00
46 ->	69	-0.02	0.00
46 ->	70	-0.01	0.01
46 ->	71	-0.03	0.02
46 ->	72	-3.10	3.70
46 ->	73	0.01	0.02
46 ->	74	0.13	0.10
46 ->	75	0.05	0.01
46 ->	76	-0.03	0.00
46 ->	77	-0.04	0.01
46 ->	78	-0.05	0.02
46 ->	79	-0.00	0.00
46 ->	80	-0.00	0.00
46 ->	81	-0.02	0.01

46	->	82	-0.00	0.00
46	->	83	-0.00	0.00
46	->	84	-0.00	0.00
46	->	85	-0.00	0.00
46	->	86	0.00	0.00
46	->	87	0.02	0.00
46	->	88	0.02	0.02
46	->	89	-0.08	0.09
46	->	90	-0.33	0.22
46	->	91	0.09	0.20
46	->	92	0.46	0.28
46	->	93	0.07	0.17
46	->	94	0.01	0.03
46	->	95	0.02	0.01
46	->	96	0.02	0.00
46	->	97	0.01	0.00
46	->	98	0.03	0.04
46	->	99	-0.12	0.11
46	->	100	-1.34	0.87
46	->	101	-5.41	1.10
46	->	102	-2.62	2.08
46	->	103	-0.32	0.32
46	->	104	-0.01	0.03
46	->	105	0.02	0.00
46	->	106	0.02	0.00
46	->	107	0.01	0.00
47	->	1	0.00	0.00
47	->	2	0.00	0.00
47	->	3	-0.00	0.00
47	->	4	0.00	0.00
47	->	5	-0.00	0.00
47	->	6	0.00	0.00
47	->	7	0.00	0.00
47	->	8	-0.00	0.00
47	->	9	-0.00	0.00
47	->	10	-0.00	0.00
47	->	11	-0.00	0.00
47	->	12	0.00	0.00
47	->	13	0.00	0.00
47	->	14	-0.00	0.00
47	->	15	-0.00	0.00
47	->	16	-0.00	0.00
47	->	17	-0.00	0.00
47	->	18	-0.01	0.00
47	->	19	-0.01	0.00
47	->	20	-0.00	0.00
47	->	21	-0.01	0.00
47	->	22	-0.09	0.05
47	->	23	-0.00	0.00
47	->	24	-0.00	0.00

47	->	25	-0.02	0.02
47	->	26	-0.03	0.02
47	->	27	-0.00	0.00
47	->	28	-0.00	0.00
47	->	29	-0.00	0.00
47	->	30	-0.01	0.00
47	->	31	0.00	0.02
47	->	32	-0.01	0.00
47	->	33	-0.08	0.03
47	->	34	0.03	0.01
47	->	35	-0.15	0.03
47	->	36	-0.01	0.00
47	->	37	-0.01	0.00
47	->	38	-0.03	0.01
47	->	39	0.00	0.00
47	->	40	-0.01	0.07
47	->	41	0.06	0.04
47	->	42	-1.88	0.58
47	->	43	-0.82	0.36
47	->	44	-4.61	0.71
47	->	45	-1.07	0.18
47	->	46	-12.92	0.50
47	->	47	23.90	1.69
47	->	48	-6.61	0.46
47	->	49	-0.97	0.20
47	->	50	-1.59	0.63
47	->	51	-2.02	0.66
47	->	52	-0.01	0.04
47	->	53	-0.03	0.03
47	->	54	-0.02	0.01
47	->	55	-0.04	0.01
47	->	56	-0.00	0.00
47	->	57	-0.00	0.00
47	->	58	0.00	0.00
47	->	59	-0.01	0.00
47	->	60	-0.00	0.00
47	->	61	-0.00	0.00
47	->	62	0.00	0.00
47	->	63	0.00	0.00
47	->	64	0.00	0.00
47	->	65	0.00	0.00
47	->	66	0.00	0.00
47	->	67	0.00	0.00
47	->	68	-0.00	0.00
47	->	69	-0.00	0.00
47	->	70	-0.00	0.00
47	->	71	-0.02	0.01
47	->	72	-0.44	0.31
47	->	73	-0.00	0.00
47	->	74	0.02	0.02

47	->	75	0.00	0.00
47	->	76	-0.00	0.00
47	->	77	-0.00	0.00
47	->	78	-0.01	0.01
47	->	79	-0.00	0.00
47	->	80	-0.00	0.00
47	->	81	-0.00	0.00
47	->	82	-0.00	0.00
47	->	83	0.00	0.00
47	->	84	-0.00	0.00
47	->	85	-0.00	0.00
47	->	86	-0.00	0.00
47	->	87	-0.01	0.00
47	->	88	-0.04	0.06
47	->	89	-1.93	1.08
47	->	90	-4.74	0.74
47	->	91	-4.65	0.92
47	->	92	-0.18	0.29
47	->	93	-0.03	0.02
47	->	94	-0.01	0.00
47	->	95	-0.00	0.00
47	->	96	-0.00	0.00
47	->	97	-0.00	0.00
47	->	98	-0.01	0.01
47	->	99	-0.03	0.02
47	->	100	-0.04	0.03
47	->	101	-0.15	0.10
47	->	102	-0.17	0.15
47	->	103	-0.10	0.06
47	->	104	-0.06	0.04
47	->	105	-0.01	0.01
47	->	106	-0.02	0.01
47	->	107	-0.01	0.00
48	->	1	0.00	0.00
48	->	2	0.00	0.00
48	->	3	-0.00	0.00
48	->	4	0.00	0.00
48	->	5	-0.00	0.00
48	->	6	0.00	0.00
48	->	7	0.00	0.00
48	->	8	-0.00	0.00
48	->	9	0.00	0.00
48	->	10	-0.00	0.00
48	->	11	-0.00	0.00
48	->	12	0.00	0.00
48	->	13	0.00	0.00
48	->	14	0.00	0.00
48	->	15	0.00	0.00
48	->	16	0.00	0.00
48	->	17	-0.00	0.00

48 ->	18	-0.01	0.00
48 ->	19	-0.00	0.00
48 ->	20	-0.00	0.00
48 ->	21	-0.00	0.00
48 ->	22	-0.01	0.01
48 ->	23	-0.00	0.00
48 ->	24	-0.00	0.00
48 ->	25	-0.01	0.00
48 ->	26	-0.00	0.00
48 ->	27	0.00	0.00
48 ->	28	-0.00	0.00
48 ->	29	-0.00	0.00
48 ->	30	-0.01	0.00
48 ->	31	-0.04	0.01
48 ->	32	-0.04	0.01
48 ->	33	-1.37	0.35
48 ->	34	-1.10	0.33
48 ->	35	-1.07	0.24
48 ->	36	-0.17	0.12
48 ->	37	-0.04	0.01
48 ->	38	-0.01	0.01
48 ->	39	-0.02	0.01
48 ->	40	-0.20	0.07
48 ->	41	-0.08	0.03
48 ->	42	-2.64	0.37
48 ->	43	-0.02	0.02
48 ->	44	-1.04	0.45
48 ->	45	-2.49	0.40
48 ->	46	-1.31	0.20
48 ->	47	-6.66	0.46
48 ->	48	21.64	1.36
48 ->	49	-6.19	0.65
48 ->	50	-0.91	0.16
48 ->	51	-2.87	0.34
48 ->	52	-3.97	0.61
48 ->	53	-0.16	0.07
48 ->	54	-0.06	0.04
48 ->	55	-0.06	0.02
48 ->	56	-0.02	0.01
48 ->	57	-0.00	0.00
48 ->	58	-0.00	0.00
48 ->	59	-0.00	0.00
48 ->	60	-0.00	0.00
48 ->	61	-0.00	0.00
48 ->	62	-0.00	0.00
48 ->	63	0.00	0.00
48 ->	64	0.00	0.00
48 ->	65	0.00	0.00
48 ->	66	0.00	0.00
48 ->	67	0.00	0.00

48	->	68	-0.00	0.00
48	->	69	-0.00	0.00
48	->	70	0.00	0.00
48	->	71	-0.01	0.01
48	->	72	-0.01	0.03
48	->	73	-0.00	0.00
48	->	74	-0.00	0.00
48	->	75	-0.00	0.00
48	->	76	-0.00	0.00
48	->	77	-0.00	0.00
48	->	78	-0.00	0.00
48	->	79	-0.00	0.00
48	->	80	0.00	0.00
48	->	81	-0.00	0.00
48	->	82	-0.00	0.00
48	->	83	0.00	0.00
48	->	84	0.00	0.00
48	->	85	-0.00	0.00
48	->	86	-0.00	0.00
48	->	87	-0.00	0.00
48	->	88	0.03	0.04
48	->	89	-0.09	0.09
48	->	90	0.01	0.05
48	->	91	0.01	0.01
48	->	92	-0.00	0.00
48	->	93	-0.00	0.00
48	->	94	-0.00	0.00
48	->	95	-0.00	0.00
48	->	96	-0.00	0.00
48	->	97	-0.00	0.00
48	->	98	-0.03	0.02
48	->	99	-0.13	0.14
48	->	100	-0.14	0.12
48	->	101	-0.02	0.01
48	->	102	-0.00	0.00
48	->	103	-0.00	0.00
48	->	104	-0.00	0.00
48	->	105	-0.00	0.00
48	->	106	-0.00	0.00
48	->	107	-0.00	0.00
49	->	1	0.00	0.00
49	->	2	0.00	0.00
49	->	3	-0.00	0.00
49	->	4	0.00	0.00
49	->	5	-0.00	0.00
49	->	6	0.00	0.00
49	->	7	0.00	0.00
49	->	8	-0.00	0.00
49	->	9	0.00	0.00
49	->	10	-0.00	0.00

49	->	11	-0.00	0.00
49	->	12	0.00	0.00
49	->	13	0.00	0.00
49	->	14	0.00	0.00
49	->	15	0.00	0.00
49	->	16	0.00	0.00
49	->	17	-0.00	0.00
49	->	18	-0.01	0.00
49	->	19	-0.00	0.00
49	->	20	-0.00	0.00
49	->	21	-0.00	0.00
49	->	22	0.00	0.00
49	->	23	0.00	0.00
49	->	24	0.00	0.00
49	->	25	0.00	0.00
49	->	26	-0.00	0.00
49	->	27	0.00	0.00
49	->	28	0.00	0.00
49	->	29	0.00	0.00
49	->	30	-0.00	0.00
49	->	31	-0.00	0.00
49	->	32	-0.00	0.00
49	->	33	-0.04	0.01
49	->	34	-0.01	0.00
49	->	35	0.01	0.02
49	->	36	-0.02	0.02
49	->	37	-0.01	0.00
49	->	38	-0.00	0.00
49	->	39	-0.00	0.00
49	->	40	-0.00	0.01
49	->	41	-0.00	0.01
49	->	42	-0.08	0.02
49	->	43	-0.01	0.00
49	->	44	0.02	0.06
49	->	45	-3.90	1.17
49	->	46	-3.32	0.60
49	->	47	-0.98	0.20
49	->	48	-6.21	0.65
49	->	49	-18.34	2.00
49	->	50	-7.73	0.54
49	->	51	-0.78	0.16
49	->	52	-2.43	0.63
49	->	53	-3.73	0.82
49	->	54	-0.15	0.06
49	->	55	-0.05	0.02
49	->	56	-0.03	0.02
49	->	57	-0.01	0.00
49	->	58	-0.02	0.02
49	->	59	0.00	0.01
49	->	60	-0.02	0.01

49	->	61	-0.03	0.01
49	->	62	-0.00	0.00
49	->	63	0.00	0.00
49	->	64	0.00	0.00
49	->	65	0.00	0.00
49	->	66	0.00	0.00
49	->	67	0.00	0.00
49	->	68	-0.00	0.00
49	->	69	0.00	0.00
49	->	70	0.00	0.01
49	->	71	-0.13	0.10
49	->	72	0.00	0.14
49	->	73	-0.02	0.01
49	->	74	-0.03	0.02
49	->	75	-0.02	0.02
49	->	76	-0.00	0.00
49	->	77	-0.00	0.00
49	->	78	0.00	0.01
49	->	79	-0.00	0.00
49	->	80	-0.00	0.00
49	->	81	0.00	0.00
49	->	82	-0.00	0.00
49	->	83	0.00	0.00
49	->	84	0.00	0.00
49	->	85	-0.00	0.00
49	->	86	-0.00	0.00
49	->	87	-0.01	0.01
49	->	88	-0.07	0.04
49	->	89	-0.10	0.05
49	->	90	-0.04	0.02
49	->	91	-0.02	0.01
49	->	92	-0.01	0.01
49	->	93	-0.00	0.00
49	->	94	-0.00	0.00
49	->	95	-0.00	0.00
49	->	96	-0.00	0.00
49	->	97	-0.00	0.00
49	->	98	-0.01	0.01
49	->	99	-0.21	0.33
49	->	100	-1.38	1.42
49	->	101	-0.28	0.13
49	->	102	-0.01	0.01
49	->	103	0.00	0.01
49	->	104	0.00	0.00
49	->	105	-0.00	0.00
49	->	106	0.00	0.00
49	->	107	-0.00	0.00
50	->	1	0.00	0.00
50	->	2	0.00	0.00
50	->	3	-0.00	0.00

50	->	4	-0.00	0.00
50	->	5	-0.00	0.00
50	->	6	0.00	0.00
50	->	7	0.00	0.00
50	->	8	-0.00	0.00
50	->	9	-0.00	0.00
50	->	10	-0.00	0.00
50	->	11	-0.00	0.00
50	->	12	0.00	0.00
50	->	13	0.00	0.00
50	->	14	0.00	0.00
50	->	15	0.00	0.00
50	->	16	0.00	0.00
50	->	17	-0.00	0.00
50	->	18	-0.00	0.00
50	->	19	-0.00	0.00
50	->	20	-0.00	0.00
50	->	21	-0.00	0.00
50	->	22	-0.00	0.00
50	->	23	0.00	0.00
50	->	24	0.00	0.00
50	->	25	0.00	0.00
50	->	26	-0.00	0.00
50	->	27	0.00	0.00
50	->	28	0.00	0.00
50	->	29	-0.00	0.00
50	->	30	-0.00	0.00
50	->	31	-0.00	0.00
50	->	32	-0.00	0.00
50	->	33	-0.01	0.00
50	->	34	0.00	0.00
50	->	35	-0.07	0.03
50	->	36	-0.00	0.00
50	->	37	-0.01	0.00
50	->	38	-0.02	0.01
50	->	39	-0.00	0.00
50	->	40	-0.00	0.04
50	->	41	-0.00	0.01
50	->	42	-0.10	0.05
50	->	43	-0.00	0.01
50	->	44	-0.06	0.04
50	->	45	-0.12	0.06
50	->	46	-2.63	1.28
50	->	47	-1.60	0.63
50	->	48	-0.91	0.16
50	->	49	-7.75	0.54
50	->	50	-20.20	2.06
50	->	51	-21.85	0.61
50	->	52	-0.83	0.15
50	->	53	-3.09	0.69

50	->	54	-2.26	1.28
50	->	55	-0.14	0.07
50	->	56	-0.04	0.02
50	->	57	-0.02	0.02
50	->	58	-0.02	0.02
50	->	59	-0.10	0.09
50	->	60	-0.03	0.01
50	->	61	-0.02	0.01
50	->	62	-0.00	0.00
50	->	63	0.00	0.00
50	->	64	0.00	0.00
50	->	65	0.00	0.00
50	->	66	0.00	0.00
50	->	67	0.00	0.00
50	->	68	-0.00	0.00
50	->	69	-0.01	0.01
50	->	70	-0.04	0.01
50	->	71	-0.69	0.45
50	->	72	-1.99	0.79
50	->	73	-0.01	0.02
50	->	74	-0.01	0.03
50	->	75	-0.09	0.10
50	->	76	-0.01	0.00
50	->	77	-0.00	0.00
50	->	78	-0.01	0.01
50	->	79	-0.00	0.00
50	->	80	-0.00	0.00
50	->	81	0.00	0.00
50	->	82	-0.00	0.00
50	->	83	0.00	0.00
50	->	84	0.00	0.00
50	->	85	0.00	0.00
50	->	86	-0.00	0.00
50	->	87	-0.03	0.02
50	->	88	-0.56	0.46
50	->	89	-1.95	1.26
50	->	90	-0.49	0.21
50	->	91	-0.01	0.01
50	->	92	-0.01	0.00
50	->	93	-0.01	0.00
50	->	94	-0.00	0.00
50	->	95	-0.00	0.00
50	->	96	-0.00	0.00
50	->	97	-0.00	0.00
50	->	98	-0.00	0.00
50	->	99	-0.01	0.01
50	->	100	-0.02	0.08
50	->	101	-0.03	0.10
50	->	102	-0.03	0.02
50	->	103	-0.03	0.01

50	->	104	-0.02	0.01
50	->	105	-0.01	0.00
50	->	106	-0.01	0.02
50	->	107	-0.00	0.00
51	->	1	0.00	0.00
51	->	2	0.00	0.00
51	->	3	-0.00	0.00
51	->	4	0.00	0.00
51	->	5	-0.00	0.00
51	->	6	0.00	0.00
51	->	7	0.00	0.00
51	->	8	-0.00	0.00
51	->	9	0.00	0.00
51	->	10	-0.00	0.00
51	->	11	-0.00	0.00
51	->	12	0.00	0.00
51	->	13	0.00	0.00
51	->	14	0.00	0.00
51	->	15	0.00	0.00
51	->	16	0.00	0.00
51	->	17	-0.00	0.00
51	->	18	-0.00	0.00
51	->	19	-0.00	0.00
51	->	20	-0.00	0.00
51	->	21	-0.00	0.00
51	->	22	0.00	0.00
51	->	23	0.00	0.00
51	->	24	0.00	0.00
51	->	25	0.00	0.00
51	->	26	-0.00	0.00
51	->	27	0.00	0.00
51	->	28	0.00	0.00
51	->	29	0.00	0.00
51	->	30	-0.00	0.00
51	->	31	-0.00	0.00
51	->	32	-0.00	0.00
51	->	33	-0.02	0.00
51	->	34	-0.10	0.02
51	->	35	2.82	0.63
51	->	36	-0.14	0.03
51	->	37	0.77	0.11
51	->	38	3.28	0.74
51	->	39	-0.10	0.05
51	->	40	-1.24	0.45
51	->	41	-0.01	0.01
51	->	42	-1.93	0.31
51	->	43	-0.01	0.00
51	->	44	-0.01	0.01
51	->	45	-0.07	0.03
51	->	46	-0.18	0.10

51 ->	47	-2.05	0.66
51 ->	48	-2.87	0.34
51 ->	49	-0.79	0.16
51 ->	50	-21.89	0.62
51 ->	51	12.14	1.08
51 ->	52	-10.36	0.46
51 ->	53	-1.19	0.23
51 ->	54	-2.62	1.37
51 ->	55	14.24	7.03
51 ->	56	-0.18	0.07
51 ->	57	-0.02	0.01
51 ->	58	-0.02	0.01
51 ->	59	-0.03	0.04
51 ->	60	-0.01	0.00
51 ->	61	-0.00	0.00
51 ->	62	-0.00	0.00
51 ->	63	-0.00	0.00
51 ->	64	-0.00	0.00
51 ->	65	-0.00	0.00
51 ->	66	-0.00	0.00
51 ->	67	0.00	0.00
51 ->	68	-0.00	0.00
51 ->	69	-0.01	0.00
51 ->	70	-0.01	0.01
51 ->	71	-0.03	0.02
51 ->	72	-0.07	0.03
51 ->	73	0.00	0.00
51 ->	74	-0.00	0.00
51 ->	75	0.00	0.01
51 ->	76	-0.00	0.00
51 ->	77	0.00	0.00
51 ->	78	0.00	0.00
51 ->	79	-0.00	0.00
51 ->	80	0.00	0.00
51 ->	81	0.00	0.00
51 ->	82	0.00	0.00
51 ->	83	0.00	0.00
51 ->	84	0.00	0.00
51 ->	85	0.00	0.00
51 ->	86	-0.00	0.00
51 ->	87	-0.03	0.02
51 ->	88	-1.24	0.52
51 ->	89	-7.03	1.37
51 ->	90	-0.07	0.02
51 ->	91	-0.01	0.00
51 ->	92	-0.01	0.00
51 ->	93	-0.00	0.00
51 ->	94	-0.00	0.00
51 ->	95	-0.00	0.00
51 ->	96	-0.00	0.00

51	->	97	-0.00	0.00
51	->	98	-0.00	0.00
51	->	99	-0.01	0.01
51	->	100	-0.01	0.01
51	->	101	-0.00	0.00
51	->	102	-0.00	0.00
51	->	103	-0.00	0.00
51	->	104	-0.01	0.00
51	->	105	-0.00	0.00
51	->	106	-0.01	0.00
51	->	107	-0.01	0.00
52	->	1	0.00	0.00
52	->	2	0.00	0.00
52	->	3	-0.00	0.00
52	->	4	0.00	0.00
52	->	5	-0.00	0.00
52	->	6	0.00	0.00
52	->	7	0.00	0.00
52	->	8	-0.00	0.00
52	->	9	0.00	0.00
52	->	10	-0.00	0.00
52	->	11	-0.00	0.00
52	->	12	0.00	0.00
52	->	13	0.00	0.00
52	->	14	0.00	0.00
52	->	15	0.00	0.00
52	->	16	0.00	0.00
52	->	17	0.00	0.00
52	->	18	-0.00	0.00
52	->	19	-0.00	0.00
52	->	20	-0.00	0.00
52	->	21	0.00	0.00
52	->	22	0.00	0.00
52	->	23	0.00	0.00
52	->	24	-0.00	0.00
52	->	25	0.00	0.00
52	->	26	0.00	0.00
52	->	27	0.00	0.00
52	->	28	-0.00	0.00
52	->	29	0.00	0.00
52	->	30	-0.00	0.00
52	->	31	-0.00	0.00
52	->	32	-0.00	0.00
52	->	33	-0.02	0.00
52	->	34	-0.04	0.02
52	->	35	-0.36	0.29
52	->	36	-0.71	0.82
52	->	37	-0.32	0.34
52	->	38	0.04	0.02
52	->	39	-0.02	0.01

52 ->	40	-0.04	0.02
52 ->	41	-0.01	0.00
52 ->	42	-0.08	0.03
52 ->	43	-0.00	0.00
52 ->	44	0.00	0.01
52 ->	45	-0.07	0.03
52 ->	46	-0.07	0.03
52 ->	47	-0.01	0.04
52 ->	48	-3.96	0.60
52 ->	49	-2.44	0.63
52 ->	50	-0.84	0.15
52 ->	51	-10.41	0.46
52 ->	52	22.84	2.06
52 ->	53	-10.32	0.41
52 ->	54	-1.00	0.26
52 ->	55	-2.85	0.66
52 ->	56	-4.29	2.23
52 ->	57	-0.15	0.11
52 ->	58	-0.25	0.17
52 ->	59	0.00	0.02
52 ->	60	-0.01	0.00
52 ->	61	-0.01	0.01
52 ->	62	-0.00	0.00
52 ->	63	0.00	0.00
52 ->	64	0.00	0.00
52 ->	65	0.00	0.00
52 ->	66	0.00	0.00
52 ->	67	0.00	0.00
52 ->	68	-0.00	0.00
52 ->	69	0.00	0.00
52 ->	70	0.00	0.00
52 ->	71	-0.03	0.02
52 ->	72	-0.02	0.01
52 ->	73	-0.00	0.00
52 ->	74	-0.00	0.00
52 ->	75	-0.00	0.00
52 ->	76	-0.00	0.00
52 ->	77	0.00	0.00
52 ->	78	-0.00	0.00
52 ->	79	-0.00	0.00
52 ->	80	0.00	0.00
52 ->	81	0.00	0.00
52 ->	82	-0.00	0.00
52 ->	83	0.00	0.00
52 ->	84	0.00	0.00
52 ->	85	-0.00	0.00
52 ->	86	-0.00	0.00
52 ->	87	-0.00	0.00
52 ->	88	0.02	0.02
52 ->	89	0.08	0.06

52	->	90	-0.01	0.00
52	->	91	-0.00	0.00
52	->	92	-0.00	0.00
52	->	93	-0.00	0.00
52	->	94	-0.00	0.00
52	->	95	-0.00	0.00
52	->	96	-0.00	0.00
52	->	97	-0.00	0.00
52	->	98	-0.01	0.00
52	->	99	-0.03	0.02
52	->	100	-0.07	0.06
52	->	101	-0.02	0.01
52	->	102	-0.00	0.00
52	->	103	-0.00	0.00
52	->	104	-0.00	0.00
52	->	105	-0.00	0.00
52	->	106	-0.00	0.00
52	->	107	-0.00	0.00
53	->	1	0.02	0.00
53	->	2	0.00	0.00
53	->	3	-0.00	0.00
53	->	4	-0.00	0.00
53	->	5	-0.00	0.00
53	->	6	0.00	0.00
53	->	7	0.00	0.00
53	->	8	-0.00	0.00
53	->	9	-0.00	0.00
53	->	10	-0.00	0.00
53	->	11	-0.00	0.00
53	->	12	0.01	0.00
53	->	13	0.00	0.00
53	->	14	0.00	0.00
53	->	15	0.00	0.00
53	->	16	0.02	0.00
53	->	17	0.00	0.00
53	->	18	-0.03	0.00
53	->	19	-0.01	0.00
53	->	20	-0.00	0.00
53	->	21	0.00	0.00
53	->	22	0.03	0.00
53	->	23	0.00	0.00
53	->	24	0.00	0.00
53	->	25	0.02	0.00
53	->	26	0.00	0.00
53	->	27	0.00	0.00
53	->	28	0.00	0.00
53	->	29	0.00	0.00
53	->	30	-0.00	0.00
53	->	31	0.02	0.00
53	->	32	-0.00	0.00

53	->	33	-0.00	0.00
53	->	34	-0.01	0.00
53	->	35	-0.05	0.02
53	->	36	0.02	0.01
53	->	37	-0.01	0.00
53	->	38	-0.04	0.01
53	->	39	-0.00	0.00
53	->	40	0.03	0.01
53	->	41	-0.00	0.00
53	->	42	-0.02	0.01
53	->	43	-0.00	0.00
53	->	44	0.06	0.01
53	->	45	-0.02	0.01
53	->	46	-0.27	0.09
53	->	47	-0.03	0.03
53	->	48	-0.16	0.07
53	->	49	-3.79	0.83
53	->	50	-3.13	0.69
53	->	51	-1.20	0.23
53	->	52	-10.36	0.42
53	->	53	-100.21	2.82
53	->	54	-5.19	0.56
53	->	55	-0.67	0.21
53	->	56	-1.46	0.31
53	->	57	-1.03	0.65
53	->	58	-3.10	2.07
53	->	59	-0.23	0.50
53	->	60	-1.42	0.49
53	->	61	-0.82	0.39
53	->	62	-0.01	0.01
53	->	63	-0.03	0.00
53	->	64	-0.02	0.00
53	->	65	-0.00	0.00
53	->	66	-0.02	0.00
53	->	67	-0.00	0.00
53	->	68	0.03	0.01
53	->	69	0.10	0.03
53	->	70	0.04	0.08
53	->	71	-4.80	1.11
53	->	72	-0.46	0.23
53	->	73	-0.26	0.11
53	->	74	-0.16	0.05
53	->	75	-0.20	0.10
53	->	76	0.05	0.02
53	->	77	0.03	0.01
53	->	78	0.04	0.01
53	->	79	0.00	0.00
53	->	80	0.00	0.00
53	->	81	0.02	0.00
53	->	82	0.00	0.00

53	->	83	-0.00	0.00
53	->	84	0.00	0.00
53	->	85	0.00	0.00
53	->	86	-0.02	0.01
53	->	87	-0.13	0.06
53	->	88	-0.45	0.27
53	->	89	-0.24	0.17
53	->	90	-0.05	0.01
53	->	91	-0.03	0.00
53	->	92	-0.02	0.00
53	->	93	-0.01	0.00
53	->	94	-0.02	0.00
53	->	95	-0.02	0.00
53	->	96	-0.02	0.00
53	->	97	-0.01	0.00
53	->	98	-0.03	0.01
53	->	99	-0.15	0.11
53	->	100	-2.70	1.35
53	->	101	-9.01	2.43
53	->	102	-0.10	0.04
53	->	103	-0.02	0.01
53	->	104	-0.02	0.00
53	->	105	-0.01	0.00
53	->	106	-0.01	0.00
53	->	107	-0.01	0.00
54	->	1	0.00	0.00
54	->	2	0.00	0.00
54	->	3	0.00	0.00
54	->	4	0.00	0.00
54	->	5	-0.00	0.00
54	->	6	0.00	0.00
54	->	7	0.00	0.00
54	->	8	-0.00	0.00
54	->	9	-0.00	0.00
54	->	10	-0.00	0.00
54	->	11	-0.00	0.00
54	->	12	0.00	0.00
54	->	13	0.00	0.00
54	->	14	0.00	0.00
54	->	15	0.00	0.00
54	->	16	0.00	0.00
54	->	17	-0.00	0.00
54	->	18	-0.00	0.00
54	->	19	-0.00	0.00
54	->	20	-0.00	0.00
54	->	21	0.00	0.00
54	->	22	0.00	0.00
54	->	23	0.00	0.00
54	->	24	0.00	0.00
54	->	25	0.00	0.00

54 ->	26	0.00	0.00
54 ->	27	0.00	0.00
54 ->	28	0.00	0.00
54 ->	29	0.00	0.00
54 ->	30	-0.00	0.00
54 ->	31	0.00	0.00
54 ->	32	0.00	0.00
54 ->	33	-0.00	0.00
54 ->	34	-0.00	0.00
54 ->	35	-0.04	0.06
54 ->	36	-0.00	0.01
54 ->	37	-0.03	0.01
54 ->	38	-0.03	0.04
54 ->	39	-0.00	0.00
54 ->	40	-0.02	0.02
54 ->	41	-0.00	0.00
54 ->	42	-0.01	0.02
54 ->	43	-0.00	0.00
54 ->	44	-0.00	0.00
54 ->	45	-0.00	0.00
54 ->	46	-0.01	0.01
54 ->	47	-0.02	0.01
54 ->	48	-0.06	0.04
54 ->	49	-0.15	0.06
54 ->	50	-2.28	1.28
54 ->	51	-2.62	1.37
54 ->	52	-1.01	0.26
54 ->	53	-5.23	0.56
54 ->	54	-22.29	1.82
54 ->	55	-22.35	0.68
54 ->	56	-0.65	0.13
54 ->	57	-0.93	0.39
54 ->	58	-0.23	0.22
54 ->	59	-0.93	1.22
54 ->	60	-0.04	0.05
54 ->	61	-0.00	0.00
54 ->	62	-0.00	0.00
54 ->	63	-0.00	0.00
54 ->	64	0.00	0.00
54 ->	65	-0.00	0.00
54 ->	66	0.00	0.00
54 ->	67	-0.00	0.00
54 ->	68	-0.01	0.01
54 ->	69	-0.03	0.03
54 ->	70	-0.04	0.03
54 ->	71	-0.43	0.27
54 ->	72	-0.07	0.05
54 ->	73	0.00	0.00
54 ->	74	-0.00	0.00
54 ->	75	-0.01	0.04

54	->	76	-0.00	0.00
54	->	77	0.00	0.00
54	->	78	-0.00	0.00
54	->	79	-0.00	0.00
54	->	80	0.00	0.00
54	->	81	0.00	0.00
54	->	82	0.00	0.00
54	->	83	0.00	0.00
54	->	84	0.00	0.00
54	->	85	0.00	0.00
54	->	86	-0.01	0.01
54	->	87	-0.27	0.19
54	->	88	-2.99	1.59
54	->	89	-0.67	1.25
54	->	90	-0.01	0.00
54	->	91	-0.00	0.00
54	->	92	-0.00	0.00
54	->	93	-0.00	0.00
54	->	94	-0.00	0.00
54	->	95	-0.00	0.00
54	->	96	-0.00	0.00
54	->	97	-0.00	0.00
54	->	98	-0.00	0.00
54	->	99	-0.00	0.00
54	->	100	-0.00	0.00
54	->	101	-0.00	0.00
54	->	102	-0.00	0.00
54	->	103	-0.00	0.00
54	->	104	-0.00	0.00
54	->	105	-0.00	0.00
54	->	106	-0.01	0.00
54	->	107	-0.00	0.00
55	->	1	0.00	0.00
55	->	2	0.00	0.00
55	->	3	-0.00	0.00
55	->	4	0.00	0.00
55	->	5	0.00	0.00
55	->	6	0.00	0.00
55	->	7	0.00	0.00
55	->	8	0.00	0.00
55	->	9	0.00	0.00
55	->	10	0.00	0.00
55	->	11	-0.00	0.00
55	->	12	0.00	0.00
55	->	13	-0.00	0.00
55	->	14	0.00	0.00
55	->	15	0.00	0.00
55	->	16	0.00	0.00
55	->	17	0.00	0.00
55	->	18	-0.00	0.00

55 ->	19	-0.00	0.00
55 ->	20	0.00	0.00
55 ->	21	0.00	0.00
55 ->	22	0.00	0.00
55 ->	23	0.00	0.00
55 ->	24	0.00	0.00
55 ->	25	-0.00	0.00
55 ->	26	0.00	0.00
55 ->	27	-0.00	0.00
55 ->	28	0.00	0.00
55 ->	29	0.00	0.00
55 ->	30	0.00	0.00
55 ->	31	-0.00	0.00
55 ->	32	-0.00	0.00
55 ->	33	-0.01	0.00
55 ->	34	-0.06	0.01
55 ->	35	2.37	0.36
55 ->	36	-0.30	0.08
55 ->	37	0.50	0.76
55 ->	38	2.01	0.28
55 ->	39	-0.05	0.05
55 ->	40	-0.20	0.07
55 ->	41	-0.01	0.01
55 ->	42	0.01	0.01
55 ->	43	0.00	0.00
55 ->	44	-0.00	0.00
55 ->	45	-0.00	0.00
55 ->	46	-0.01	0.00
55 ->	47	-0.04	0.01
55 ->	48	-0.06	0.02
55 ->	49	-0.05	0.02
55 ->	50	-0.14	0.07
55 ->	51	14.22	7.03
55 ->	52	-2.84	0.66
55 ->	53	-0.68	0.21
55 ->	54	-22.46	0.69
55 ->	55	4.29	0.97
55 ->	56	-5.13	0.51
55 ->	57	-0.42	0.22
55 ->	58	-0.04	0.04
55 ->	59	-0.07	0.04
55 ->	60	-0.01	0.01
55 ->	61	-0.00	0.00
55 ->	62	-0.00	0.00
55 ->	63	-0.00	0.00
55 ->	64	-0.00	0.00
55 ->	65	-0.00	0.00
55 ->	66	-0.00	0.00
55 ->	67	-0.00	0.00
55 ->	68	-0.00	0.00

55	->	69	-0.01	0.00
55	->	70	-0.01	0.00
55	->	71	-0.01	0.00
55	->	72	-0.00	0.00
55	->	73	-0.00	0.00
55	->	74	-0.00	0.00
55	->	75	-0.00	0.00
55	->	76	0.00	0.00
55	->	77	0.00	0.00
55	->	78	0.00	0.00
55	->	79	0.00	0.00
55	->	80	0.00	0.00
55	->	81	0.00	0.00
55	->	82	0.00	0.00
55	->	83	-0.00	0.00
55	->	84	0.00	0.00
55	->	85	-0.00	0.00
55	->	86	-0.00	0.00
55	->	87	0.01	0.01
55	->	88	0.07	0.05
55	->	89	0.08	0.07
55	->	90	-0.00	0.00
55	->	91	-0.00	0.00
55	->	92	-0.00	0.00
55	->	93	-0.00	0.00
55	->	94	-0.00	0.00
55	->	95	-0.00	0.00
55	->	96	-0.00	0.00
55	->	97	-0.00	0.00
55	->	98	-0.00	0.00
55	->	99	-0.00	0.00
55	->	100	-0.00	0.00
55	->	101	-0.00	0.00
55	->	102	-0.00	0.00
55	->	103	-0.00	0.00
55	->	104	-0.00	0.00
55	->	105	-0.00	0.00
55	->	106	-0.00	0.00
55	->	107	-0.00	0.00
56	->	1	0.00	0.00
56	->	2	0.00	0.00
56	->	3	0.00	0.00
56	->	4	0.00	0.00
56	->	5	-0.00	0.00
56	->	6	0.00	0.00
56	->	7	0.00	0.00
56	->	8	-0.00	0.00
56	->	9	0.00	0.00
56	->	10	-0.00	0.00
56	->	11	-0.00	0.00

56	->	12	0.00	0.00
56	->	13	0.00	0.00
56	->	14	0.00	0.00
56	->	15	0.00	0.00
56	->	16	0.00	0.00
56	->	17	-0.00	0.00
56	->	18	-0.00	0.00
56	->	19	-0.00	0.00
56	->	20	0.00	0.00
56	->	21	0.00	0.00
56	->	22	0.00	0.00
56	->	23	0.00	0.00
56	->	24	0.00	0.00
56	->	25	0.00	0.00
56	->	26	0.00	0.00
56	->	27	0.00	0.00
56	->	28	0.00	0.00
56	->	29	-0.00	0.00
56	->	30	-0.00	0.00
56	->	31	0.00	0.00
56	->	32	-0.00	0.00
56	->	33	-0.00	0.00
56	->	34	-0.00	0.00
56	->	35	-0.01	0.01
56	->	36	-0.09	0.19
56	->	37	-0.10	0.09
56	->	38	-0.01	0.00
56	->	39	-0.00	0.00
56	->	40	0.00	0.00
56	->	41	-0.00	0.00
56	->	42	-0.00	0.00
56	->	43	-0.00	0.00
56	->	44	0.00	0.00
56	->	45	-0.00	0.00
56	->	46	-0.01	0.00
56	->	47	-0.00	0.00
56	->	48	-0.02	0.01
56	->	49	-0.03	0.02
56	->	50	-0.04	0.02
56	->	51	-0.18	0.07
56	->	52	-4.30	2.22
56	->	53	-1.46	0.30
56	->	54	-0.66	0.13
56	->	55	-5.28	0.50
56	->	56	-24.25	2.50
56	->	57	-11.69	0.42
56	->	58	-2.28	0.87
56	->	59	-0.13	0.20
56	->	60	-0.11	0.27
56	->	61	-0.00	0.00

56	->	62	-0.00	0.00
56	->	63	0.00	0.00
56	->	64	0.00	0.00
56	->	65	-0.00	0.00
56	->	66	0.00	0.00
56	->	67	-0.00	0.00
56	->	68	-0.00	0.00
56	->	69	-0.02	0.02
56	->	70	-0.01	0.01
56	->	71	-0.02	0.00
56	->	72	-0.00	0.00
56	->	73	-0.00	0.00
56	->	74	-0.00	0.00
56	->	75	-0.00	0.00
56	->	76	-0.00	0.00
56	->	77	0.00	0.00
56	->	78	0.00	0.00
56	->	79	0.00	0.00
56	->	80	0.00	0.00
56	->	81	0.00	0.00
56	->	82	0.00	0.00
56	->	83	0.00	0.00
56	->	84	0.00	0.00
56	->	85	0.00	0.00
56	->	86	-0.00	0.00
56	->	87	-0.01	0.00
56	->	88	-0.02	0.02
56	->	89	-0.01	0.01
56	->	90	-0.00	0.00
56	->	91	-0.00	0.00
56	->	92	-0.00	0.00
56	->	93	-0.00	0.00
56	->	94	-0.00	0.00
56	->	95	-0.00	0.00
56	->	96	-0.00	0.00
56	->	97	-0.00	0.00
56	->	98	-0.00	0.00
56	->	99	-0.00	0.00
56	->	100	-0.01	0.00
56	->	101	-0.00	0.00
56	->	102	-0.00	0.00
56	->	103	-0.00	0.00
56	->	104	-0.00	0.00
56	->	105	-0.00	0.00
56	->	106	-0.00	0.00
56	->	107	-0.00	0.00
57	->	1	0.00	0.00
57	->	2	0.00	0.00
57	->	3	0.00	0.00
57	->	4	0.00	0.00

57	->	5	-0.00	0.00
57	->	6	0.00	0.00
57	->	7	0.00	0.00
57	->	8	-0.00	0.00
57	->	9	0.00	0.00
57	->	10	-0.00	0.00
57	->	11	-0.00	0.00
57	->	12	0.00	0.00
57	->	13	0.00	0.00
57	->	14	0.00	0.00
57	->	15	0.00	0.00
57	->	16	0.00	0.00
57	->	17	-0.00	0.00
57	->	18	-0.00	0.00
57	->	19	-0.00	0.00
57	->	20	0.00	0.00
57	->	21	0.00	0.00
57	->	22	0.00	0.00
57	->	23	0.00	0.00
57	->	24	0.00	0.00
57	->	25	0.00	0.00
57	->	26	0.00	0.00
57	->	27	0.00	0.00
57	->	28	0.00	0.00
57	->	29	0.00	0.00
57	->	30	-0.00	0.00
57	->	31	0.00	0.00
57	->	32	-0.00	0.00
57	->	33	0.00	0.00
57	->	34	-0.00	0.00
57	->	35	-0.00	0.00
57	->	36	-0.00	0.00
57	->	37	-0.01	0.00
57	->	38	-0.00	0.00
57	->	39	0.00	0.00
57	->	40	0.00	0.00
57	->	41	-0.00	0.00
57	->	42	-0.00	0.00
57	->	43	0.00	0.00
57	->	44	0.00	0.00
57	->	45	-0.00	0.00
57	->	46	-0.00	0.00
57	->	47	-0.00	0.00
57	->	48	-0.00	0.00
57	->	49	-0.01	0.00
57	->	50	-0.02	0.02
57	->	51	-0.02	0.01
57	->	52	-0.15	0.11
57	->	53	-1.04	0.66
57	->	54	-0.97	0.41

57	->	55	-0.44	0.21
57	->	56	-11.76	0.42
57	->	57	12.15	0.85
57	->	58	-14.75	0.49
57	->	59	-0.98	0.48
57	->	60	-0.03	0.01
57	->	61	-0.01	0.00
57	->	62	-0.00	0.00
57	->	63	0.00	0.00
57	->	64	0.00	0.00
57	->	65	-0.00	0.00
57	->	66	0.00	0.00
57	->	67	-0.00	0.00
57	->	68	-0.00	0.00
57	->	69	-0.10	0.44
57	->	70	-0.00	0.01
57	->	71	-0.03	0.01
57	->	72	-0.01	0.00
57	->	73	-0.00	0.00
57	->	74	-0.00	0.00
57	->	75	-0.01	0.00
57	->	76	-0.00	0.00
57	->	77	0.00	0.00
57	->	78	0.00	0.00
57	->	79	-0.00	0.00
57	->	80	0.00	0.00
57	->	81	0.00	0.00
57	->	82	0.00	0.00
57	->	83	0.00	0.00
57	->	84	0.00	0.00
57	->	85	0.00	0.00
57	->	86	-0.00	0.00
57	->	87	-0.02	0.01
57	->	88	-0.04	0.03
57	->	89	-0.01	0.00
57	->	90	-0.00	0.00
57	->	91	-0.00	0.00
57	->	92	-0.00	0.00
57	->	93	-0.00	0.00
57	->	94	-0.00	0.00
57	->	95	-0.00	0.00
57	->	96	-0.00	0.00
57	->	97	-0.00	0.00
57	->	98	-0.00	0.00
57	->	99	-0.00	0.00
57	->	100	-0.00	0.00
57	->	101	-0.00	0.00
57	->	102	-0.00	0.00
57	->	103	-0.00	0.00
57	->	104	-0.00	0.00

57	->	105	-0.00	0.00
57	->	106	-0.00	0.00
57	->	107	-0.00	0.00
58	->	1	-0.01	0.00
58	->	2	-0.00	0.00
58	->	3	-0.00	0.00
58	->	4	-0.00	0.00
58	->	5	0.00	0.00
58	->	6	-0.00	0.00
58	->	7	-0.00	0.00
58	->	8	0.00	0.00
58	->	9	0.00	0.00
58	->	10	0.00	0.00
58	->	11	0.00	0.00
58	->	12	-0.00	0.00
58	->	13	-0.00	0.00
58	->	14	-0.00	0.00
58	->	15	-0.00	0.00
58	->	16	-0.01	0.00
58	->	17	-0.00	0.00
58	->	18	0.01	0.00
58	->	19	0.01	0.00
58	->	20	-0.00	0.00
58	->	21	-0.00	0.00
58	->	22	-0.01	0.00
58	->	23	-0.00	0.00
58	->	24	-0.00	0.00
58	->	25	-0.01	0.00
58	->	26	-0.00	0.00
58	->	27	-0.00	0.00
58	->	28	0.00	0.00
58	->	29	0.00	0.00
58	->	30	0.00	0.00
58	->	31	-0.01	0.00
58	->	32	0.00	0.00
58	->	33	-0.00	0.00
58	->	34	0.00	0.00
58	->	35	0.03	0.01
58	->	36	-0.05	0.03
58	->	37	-0.00	0.00
58	->	38	0.02	0.00
58	->	39	-0.00	0.00
58	->	40	-0.02	0.00
58	->	41	0.00	0.00
58	->	42	-0.00	0.00
58	->	43	0.00	0.00
58	->	44	-0.02	0.01
58	->	45	0.00	0.00
58	->	46	0.03	0.01
58	->	47	0.00	0.00

58	->	48	-0.00	0.00
58	->	49	-0.02	0.02
58	->	50	-0.02	0.02
58	->	51	-0.02	0.01
58	->	52	-0.25	0.17
58	->	53	-3.09	2.05
58	->	54	-0.23	0.22
58	->	55	-0.04	0.04
58	->	56	-2.30	0.86
58	->	57	-14.89	0.49
58	->	58	-62.74	2.69
58	->	59	-12.29	0.90
58	->	60	-1.22	0.53
58	->	61	-0.09	0.03
58	->	62	-0.02	0.01
58	->	63	0.02	0.01
58	->	64	0.02	0.01
58	->	65	0.00	0.00
58	->	66	0.02	0.01
58	->	67	0.00	0.00
58	->	68	-0.03	0.02
58	->	69	-1.15	2.31
58	->	70	-0.23	0.12
58	->	71	-0.54	0.38
58	->	72	-0.03	0.01
58	->	73	-0.01	0.00
58	->	74	0.02	0.01
58	->	75	0.05	0.02
58	->	76	-0.04	0.01
58	->	77	-0.02	0.01
58	->	78	-0.02	0.00
58	->	79	-0.00	0.00
58	->	80	-0.00	0.00
58	->	81	-0.01	0.00
58	->	82	-0.00	0.00
58	->	83	0.00	0.00
58	->	84	-0.00	0.00
58	->	85	-0.00	0.00
58	->	86	0.00	0.00
58	->	87	0.03	0.01
58	->	88	0.03	0.02
58	->	89	0.03	0.01
58	->	90	0.01	0.00
58	->	91	0.01	0.00
58	->	92	0.01	0.00
58	->	93	0.00	0.00
58	->	94	0.01	0.00
58	->	95	0.01	0.00
58	->	96	0.01	0.00
58	->	97	0.00	0.00

58	->	98	0.01	0.00
58	->	99	0.02	0.01
58	->	100	0.04	0.02
58	->	101	0.04	0.03
58	->	102	0.02	0.01
58	->	103	0.01	0.00
58	->	104	0.01	0.00
58	->	105	0.00	0.00
58	->	106	0.00	0.00
58	->	107	0.00	0.00
59	->	1	0.01	0.00
59	->	2	0.00	0.00
59	->	3	0.00	0.00
59	->	4	0.00	0.00
59	->	5	-0.00	0.00
59	->	6	0.00	0.00
59	->	7	0.00	0.00
59	->	8	-0.00	0.00
59	->	9	0.00	0.00
59	->	10	-0.00	0.00
59	->	11	-0.00	0.00
59	->	12	0.01	0.00
59	->	13	0.00	0.00
59	->	14	0.00	0.00
59	->	15	0.00	0.00
59	->	16	0.01	0.00
59	->	17	-0.00	0.00
59	->	18	-0.01	0.00
59	->	19	-0.01	0.00
59	->	20	-0.00	0.00
59	->	21	0.00	0.00
59	->	22	0.01	0.00
59	->	23	0.00	0.00
59	->	24	-0.00	0.00
59	->	25	0.01	0.00
59	->	26	0.00	0.00
59	->	27	0.00	0.00
59	->	28	0.00	0.00
59	->	29	0.00	0.00
59	->	30	-0.00	0.00
59	->	31	0.01	0.00
59	->	32	-0.00	0.00
59	->	33	0.00	0.00
59	->	34	0.00	0.00
59	->	35	-0.03	0.01
59	->	36	0.02	0.01
59	->	37	-0.00	0.00
59	->	38	-0.03	0.01
59	->	39	0.00	0.00
59	->	40	0.03	0.01

59 ->	41	-0.00	0.00
59 ->	42	-0.00	0.00
59 ->	43	0.00	0.00
59 ->	44	0.02	0.00
59 ->	45	-0.00	0.00
59 ->	46	-0.04	0.01
59 ->	47	-0.01	0.00
59 ->	48	-0.00	0.00
59 ->	49	0.00	0.01
59 ->	50	-0.10	0.09
59 ->	51	-0.03	0.04
59 ->	52	0.00	0.02
59 ->	53	-0.24	0.51
59 ->	54	-0.93	1.23
59 ->	55	-0.07	0.04
59 ->	56	-0.13	0.20
59 ->	57	-1.05	0.51
59 ->	58	-12.35	0.92
59 ->	59	14.58	4.23
59 ->	60	-0.21	0.45
59 ->	61	-0.97	0.25
59 ->	62	-0.15	0.04
59 ->	63	-0.09	0.01
59 ->	64	-0.03	0.00
59 ->	65	-0.00	0.00
59 ->	66	-0.06	0.01
59 ->	67	-0.01	0.00
59 ->	68	0.18	0.11
59 ->	69	-2.38	0.86
59 ->	70	-3.10	0.57
59 ->	71	-1.91	0.34
59 ->	72	-0.05	0.05
59 ->	73	-0.06	0.02
59 ->	74	-0.07	0.02
59 ->	75	-0.85	0.57
59 ->	76	0.03	0.02
59 ->	77	0.01	0.01
59 ->	78	0.07	0.02
59 ->	79	-0.00	0.00
59 ->	80	-0.00	0.00
59 ->	81	0.02	0.00
59 ->	82	0.00	0.00
59 ->	83	-0.00	0.00
59 ->	84	0.00	0.00
59 ->	85	0.00	0.00
59 ->	86	-0.20	0.30
59 ->	87	-1.81	2.27
59 ->	88	-2.15	2.99
59 ->	89	-0.12	0.09
59 ->	90	-0.03	0.01

59	->	91	-0.02	0.01
59	->	92	-0.01	0.00
59	->	93	-0.01	0.00
59	->	94	-0.01	0.00
59	->	95	-0.01	0.00
59	->	96	-0.00	0.00
59	->	97	-0.00	0.00
59	->	98	-0.01	0.00
59	->	99	-0.02	0.00
59	->	100	-0.06	0.03
59	->	101	-0.10	0.04
59	->	102	-0.04	0.01
59	->	103	-0.02	0.00
59	->	104	-0.02	0.00
59	->	105	-0.01	0.00
59	->	106	-0.01	0.00
59	->	107	-0.01	0.00
60	->	1	0.00	0.00
60	->	2	0.00	0.00
60	->	3	0.00	0.00
60	->	4	0.00	0.00
60	->	5	-0.00	0.00
60	->	6	0.00	0.00
60	->	7	0.00	0.00
60	->	8	-0.00	0.00
60	->	9	0.00	0.00
60	->	10	-0.00	0.00
60	->	11	-0.00	0.00
60	->	12	0.00	0.00
60	->	13	0.00	0.00
60	->	14	0.00	0.00
60	->	15	0.00	0.00
60	->	16	0.00	0.00
60	->	17	0.00	0.00
60	->	18	-0.00	0.00
60	->	19	-0.00	0.00
60	->	20	-0.00	0.00
60	->	21	0.00	0.00
60	->	22	0.00	0.00
60	->	23	0.00	0.00
60	->	24	0.00	0.00
60	->	25	0.00	0.00
60	->	26	0.00	0.00
60	->	27	0.00	0.00
60	->	28	0.00	0.00
60	->	29	0.00	0.00
60	->	30	-0.00	0.00
60	->	31	0.00	0.00
60	->	32	0.00	0.00
60	->	33	-0.00	0.00

60 ->	34	-0.00	0.00
60 ->	35	-0.00	0.00
60 ->	36	0.00	0.00
60 ->	37	-0.00	0.00
60 ->	38	-0.00	0.00
60 ->	39	0.00	0.00
60 ->	40	0.00	0.00
60 ->	41	-0.00	0.00
60 ->	42	-0.00	0.00
60 ->	43	-0.00	0.00
60 ->	44	0.00	0.00
60 ->	45	-0.00	0.00
60 ->	46	-0.01	0.00
60 ->	47	-0.00	0.00
60 ->	48	-0.00	0.00
60 ->	49	-0.02	0.01
60 ->	50	-0.03	0.01
60 ->	51	-0.01	0.00
60 ->	52	-0.01	0.00
60 ->	53	-1.39	0.49
60 ->	54	-0.04	0.05
60 ->	55	-0.01	0.01
60 ->	56	-0.12	0.28
60 ->	57	-0.03	0.01
60 ->	58	-1.25	0.54
60 ->	59	-0.24	0.45
60 ->	60	1.70	0.67
60 ->	61	-10.50	0.41
60 ->	62	-0.42	0.15
60 ->	63	-0.02	0.01
60 ->	64	-0.00	0.00
60 ->	65	-0.00	0.00
60 ->	66	0.00	0.00
60 ->	67	-0.00	0.00
60 ->	68	-0.02	0.01
60 ->	69	-0.61	0.61
60 ->	70	-0.97	0.17
60 ->	71	-0.83	0.21
60 ->	72	-0.15	0.06
60 ->	73	-0.06	0.02
60 ->	74	-0.01	0.00
60 ->	75	-0.02	0.01
60 ->	76	-0.04	0.02
60 ->	77	-0.01	0.00
60 ->	78	-0.00	0.00
60 ->	79	-0.00	0.00
60 ->	80	-0.00	0.00
60 ->	81	-0.00	0.00
60 ->	82	-0.00	0.00
60 ->	83	-0.00	0.00

60	->	84	-0.00	0.00
60	->	85	0.00	0.00
60	->	86	-0.00	0.00
60	->	87	-0.01	0.00
60	->	88	-0.01	0.00
60	->	89	-0.01	0.00
60	->	90	-0.00	0.00
60	->	91	-0.00	0.00
60	->	92	-0.00	0.00
60	->	93	-0.00	0.00
60	->	94	-0.00	0.00
60	->	95	-0.00	0.00
60	->	96	-0.00	0.00
60	->	97	-0.00	0.00
60	->	98	-0.00	0.00
60	->	99	-0.01	0.00
60	->	100	-0.04	0.02
60	->	101	-0.13	0.09
60	->	102	-0.02	0.01
60	->	103	-0.00	0.00
60	->	104	-0.00	0.00
60	->	105	-0.00	0.00
60	->	106	-0.00	0.00
60	->	107	-0.00	0.00
61	->	1	0.00	0.00
61	->	2	0.00	0.00
61	->	3	0.00	0.00
61	->	4	0.00	0.00
61	->	5	-0.00	0.00
61	->	6	0.00	0.00
61	->	7	0.00	0.00
61	->	8	0.00	0.00
61	->	9	0.00	0.00
61	->	10	-0.00	0.00
61	->	11	-0.00	0.00
61	->	12	0.00	0.00
61	->	13	0.00	0.00
61	->	14	0.00	0.00
61	->	15	0.00	0.00
61	->	16	0.00	0.00
61	->	17	0.00	0.00
61	->	18	-0.00	0.00
61	->	19	-0.00	0.00
61	->	20	0.00	0.00
61	->	21	0.00	0.00
61	->	22	-0.00	0.00
61	->	23	0.00	0.00
61	->	24	0.00	0.00
61	->	25	0.00	0.00
61	->	26	0.00	0.00

61	->	27	0.00	0.00
61	->	28	0.00	0.00
61	->	29	0.00	0.00
61	->	30	-0.00	0.00
61	->	31	-0.00	0.00
61	->	32	0.00	0.00
61	->	33	-0.00	0.00
61	->	34	0.00	0.00
61	->	35	0.00	0.00
61	->	36	-0.00	0.00
61	->	37	-0.00	0.00
61	->	38	0.00	0.00
61	->	39	-0.00	0.00
61	->	40	-0.00	0.00
61	->	41	0.00	0.00
61	->	42	-0.00	0.00
61	->	43	0.00	0.00
61	->	44	-0.00	0.00
61	->	45	-0.00	0.00
61	->	46	-0.01	0.00
61	->	47	-0.00	0.00
61	->	48	-0.00	0.00
61	->	49	-0.03	0.01
61	->	50	-0.02	0.01
61	->	51	-0.00	0.00
61	->	52	-0.01	0.01
61	->	53	-0.80	0.38
61	->	54	-0.00	0.00
61	->	55	-0.00	0.00
61	->	56	-0.00	0.00
61	->	57	-0.01	0.00
61	->	58	-0.09	0.03
61	->	59	-0.98	0.25
61	->	60	-10.63	0.40
61	->	61	27.81	1.60
61	->	62	-10.68	0.42
61	->	63	-0.40	0.07
61	->	64	-0.05	0.01
61	->	65	-0.01	0.00
61	->	66	-0.01	0.01
61	->	67	-0.02	0.01
61	->	68	-0.28	0.09
61	->	69	-3.07	0.81
61	->	70	-3.68	0.43
61	->	71	-0.91	0.24
61	->	72	-2.00	0.66
61	->	73	-1.90	0.69
61	->	74	0.01	0.02
61	->	75	-0.14	0.06
61	->	76	-1.37	0.76

61 ->	77	-0.05	0.04
61 ->	78	-0.01	0.01
61 ->	79	-0.02	0.01
61 ->	80	-0.02	0.01
61 ->	81	-0.00	0.00
61 ->	82	-0.00	0.00
61 ->	83	-0.01	0.00
61 ->	84	-0.00	0.00
61 ->	85	-0.00	0.00
61 ->	86	-0.00	0.00
61 ->	87	-0.00	0.00
61 ->	88	-0.01	0.00
61 ->	89	-0.01	0.00
61 ->	90	-0.00	0.00
61 ->	91	-0.00	0.00
61 ->	92	-0.00	0.00
61 ->	93	-0.01	0.00
61 ->	94	-0.00	0.00
61 ->	95	-0.00	0.00
61 ->	96	-0.00	0.00
61 ->	97	-0.00	0.00
61 ->	98	-0.00	0.00
61 ->	99	-0.01	0.00
61 ->	100	-0.02	0.04
61 ->	101	-1.28	0.79
61 ->	102	-0.14	0.18
61 ->	103	-0.01	0.01
61 ->	104	-0.01	0.00
61 ->	105	-0.00	0.00
61 ->	106	-0.00	0.00
61 ->	107	-0.00	0.00
62 ->	1	-0.00	0.00
62 ->	2	0.00	0.00
62 ->	3	-0.00	0.00
62 ->	4	0.00	0.00
62 ->	5	0.00	0.00
62 ->	6	0.00	0.00
62 ->	7	0.00	0.00
62 ->	8	0.00	0.00
62 ->	9	0.00	0.00
62 ->	10	0.00	0.00
62 ->	11	0.00	0.00
62 ->	12	-0.00	0.00
62 ->	13	-0.00	0.00
62 ->	14	-0.00	0.00
62 ->	15	-0.00	0.00
62 ->	16	-0.00	0.00
62 ->	17	0.00	0.00
62 ->	18	0.00	0.00
62 ->	19	0.00	0.00

62	->	20	0.00	0.00
62	->	21	-0.00	0.00
62	->	22	-0.00	0.00
62	->	23	-0.00	0.00
62	->	24	0.00	0.00
62	->	25	-0.00	0.00
62	->	26	0.00	0.00
62	->	27	0.00	0.00
62	->	28	-0.00	0.00
62	->	29	0.00	0.00
62	->	30	0.00	0.00
62	->	31	-0.00	0.00
62	->	32	0.00	0.00
62	->	33	0.00	0.00
62	->	34	0.00	0.00
62	->	35	0.00	0.00
62	->	36	-0.00	0.00
62	->	37	0.00	0.00
62	->	38	-0.00	0.00
62	->	39	0.00	0.00
62	->	40	-0.00	0.00
62	->	41	0.00	0.00
62	->	42	0.00	0.00
62	->	43	0.00	0.00
62	->	44	-0.00	0.00
62	->	45	0.00	0.00
62	->	46	0.00	0.00
62	->	47	0.00	0.00
62	->	48	-0.00	0.00
62	->	49	-0.00	0.00
62	->	50	-0.00	0.00
62	->	51	-0.00	0.00
62	->	52	-0.00	0.00
62	->	53	-0.01	0.01
62	->	54	-0.00	0.00
62	->	55	-0.00	0.00
62	->	56	-0.00	0.00
62	->	57	-0.00	0.00
62	->	58	-0.02	0.01
62	->	59	-0.15	0.04
62	->	60	-0.44	0.17
62	->	61	-10.66	0.42
62	->	62	22.99	1.10
62	->	63	-11.95	0.49
62	->	64	-0.42	0.11
62	->	65	-0.03	0.03
62	->	66	-0.15	0.05
62	->	67	-0.10	0.16
62	->	68	-1.21	0.54
62	->	69	-1.46	0.45

62	->	70	-0.93	0.37
62	->	71	-0.01	0.01
62	->	72	-0.03	0.01
62	->	73	-0.02	0.01
62	->	74	0.00	0.00
62	->	75	-0.01	0.01
62	->	76	-2.38	1.57
62	->	77	-0.01	0.01
62	->	78	0.00	0.01
62	->	79	-0.08	0.02
62	->	80	-0.03	0.02
62	->	81	-0.01	0.00
62	->	82	-0.00	0.00
62	->	83	-0.04	0.01
62	->	84	-0.00	0.00
62	->	85	-0.00	0.00
62	->	86	-0.01	0.01
62	->	87	-0.01	0.01
62	->	88	-0.00	0.00
62	->	89	-0.00	0.00
62	->	90	0.00	0.00
62	->	91	0.00	0.00
62	->	92	0.00	0.00
62	->	93	0.00	0.00
62	->	94	0.00	0.00
62	->	95	0.00	0.00
62	->	96	0.00	0.00
62	->	97	0.00	0.00
62	->	98	0.00	0.00
62	->	99	0.00	0.00
62	->	100	0.00	0.00
62	->	101	0.00	0.00
62	->	102	0.00	0.01
62	->	103	0.00	0.00
62	->	104	0.00	0.00
62	->	105	-0.00	0.00
62	->	106	0.00	0.00
62	->	107	0.00	0.00
63	->	1	-0.01	0.00
63	->	2	-0.00	0.00
63	->	3	0.00	0.00
63	->	4	-0.00	0.00
63	->	5	0.00	0.00
63	->	6	-0.00	0.00
63	->	7	0.00	0.00
63	->	8	0.00	0.00
63	->	9	0.00	0.00
63	->	10	0.00	0.00
63	->	11	0.00	0.00
63	->	12	-0.00	0.00

63 ->	13	-0.00	0.00
63 ->	14	-0.00	0.00
63 ->	15	-0.00	0.00
63 ->	16	-0.00	0.00
63 ->	17	0.00	0.00
63 ->	18	0.00	0.00
63 ->	19	0.00	0.00
63 ->	20	0.00	0.00
63 ->	21	-0.00	0.00
63 ->	22	-0.01	0.00
63 ->	23	-0.00	0.00
63 ->	24	-0.00	0.00
63 ->	25	-0.00	0.00
63 ->	26	-0.00	0.00
63 ->	27	-0.00	0.00
63 ->	28	-0.00	0.00
63 ->	29	-0.00	0.00
63 ->	30	0.00	0.00
63 ->	31	-0.00	0.00
63 ->	32	-0.00	0.00
63 ->	33	-0.00	0.00
63 ->	34	0.00	0.00
63 ->	35	0.01	0.00
63 ->	36	-0.01	0.00
63 ->	37	0.00	0.00
63 ->	38	0.01	0.00
63 ->	39	-0.00	0.00
63 ->	40	-0.01	0.00
63 ->	41	0.00	0.00
63 ->	42	-0.00	0.00
63 ->	43	-0.00	0.00
63 ->	44	-0.01	0.00
63 ->	45	0.00	0.00
63 ->	46	0.01	0.00
63 ->	47	0.00	0.00
63 ->	48	0.00	0.00
63 ->	49	0.00	0.00
63 ->	50	0.00	0.00
63 ->	51	-0.00	0.00
63 ->	52	0.00	0.00
63 ->	53	-0.03	0.00
63 ->	54	-0.00	0.00
63 ->	55	-0.00	0.00
63 ->	56	0.00	0.00
63 ->	57	0.00	0.00
63 ->	58	0.02	0.01
63 ->	59	-0.09	0.01
63 ->	60	-0.02	0.01
63 ->	61	-0.40	0.07
63 ->	62	-11.99	0.49

63	->	63	-26.89	1.72
63	->	64	-14.84	1.28
63	->	65	1.34	1.55
63	->	66	3.03	0.46
63	->	67	-0.71	0.64
63	->	68	-2.24	0.76
63	->	69	-0.61	0.14
63	->	70	-0.82	0.30
63	->	71	0.01	0.01
63	->	72	-0.04	0.01
63	->	73	-0.01	0.01
63	->	74	0.04	0.01
63	->	75	-0.01	0.02
63	->	76	-1.62	0.66
63	->	77	-0.02	0.03
63	->	78	-0.19	0.04
63	->	79	3.03	0.76
63	->	80	0.24	0.17
63	->	81	-0.01	0.02
63	->	82	-0.07	0.02
63	->	83	2.43	0.33
63	->	84	-0.00	0.01
63	->	85	0.00	0.01
63	->	86	0.03	0.03
63	->	87	0.04	0.01
63	->	88	0.01	0.00
63	->	89	0.01	0.00
63	->	90	0.01	0.00
63	->	91	0.01	0.00
63	->	92	0.00	0.00
63	->	93	0.00	0.00
63	->	94	0.01	0.00
63	->	95	0.01	0.00
63	->	96	0.00	0.00
63	->	97	0.00	0.00
63	->	98	0.00	0.00
63	->	99	0.01	0.00
63	->	100	0.01	0.00
63	->	101	0.03	0.00
63	->	102	0.04	0.01
63	->	103	0.03	0.01
63	->	104	0.02	0.00
63	->	105	0.01	0.00
63	->	106	0.01	0.00
63	->	107	0.00	0.00
64	->	1	-0.01	0.00
64	->	2	-0.00	0.00
64	->	3	0.00	0.00
64	->	4	-0.00	0.00
64	->	5	0.00	0.00

64	->	6	0.00	0.00
64	->	7	0.00	0.00
64	->	8	0.00	0.00
64	->	9	0.00	0.00
64	->	10	0.00	0.00
64	->	11	0.00	0.00
64	->	12	-0.00	0.00
64	->	13	-0.00	0.00
64	->	14	-0.00	0.00
64	->	15	-0.00	0.00
64	->	16	-0.00	0.00
64	->	17	0.00	0.00
64	->	18	0.00	0.00
64	->	19	0.00	0.00
64	->	20	0.00	0.00
64	->	21	-0.00	0.00
64	->	22	-0.00	0.00
64	->	23	-0.00	0.00
64	->	24	-0.00	0.00
64	->	25	-0.00	0.00
64	->	26	-0.00	0.00
64	->	27	-0.00	0.00
64	->	28	-0.00	0.00
64	->	29	-0.00	0.00
64	->	30	0.00	0.00
64	->	31	-0.00	0.00
64	->	32	-0.00	0.00
64	->	33	-0.00	0.00
64	->	34	-0.00	0.00
64	->	35	0.00	0.00
64	->	36	-0.00	0.00
64	->	37	0.00	0.00
64	->	38	0.00	0.00
64	->	39	-0.00	0.00
64	->	40	-0.00	0.00
64	->	41	0.00	0.00
64	->	42	-0.00	0.00
64	->	43	-0.00	0.00
64	->	44	-0.01	0.00
64	->	45	0.00	0.00
64	->	46	0.01	0.00
64	->	47	0.00	0.00
64	->	48	0.00	0.00
64	->	49	0.00	0.00
64	->	50	0.00	0.00
64	->	51	-0.00	0.00
64	->	52	0.00	0.00
64	->	53	-0.02	0.00
64	->	54	0.00	0.00
64	->	55	-0.00	0.00

64	->	56	0.00	0.00
64	->	57	0.00	0.00
64	->	58	0.02	0.01
64	->	59	-0.03	0.00
64	->	60	-0.00	0.00
64	->	61	-0.05	0.01
64	->	62	-0.43	0.11
64	->	63	-14.98	1.30
64	->	64	-35.22	1.55
64	->	65	-12.29	0.67
64	->	66	-0.30	0.27
64	->	67	-0.20	0.32
64	->	68	-0.04	0.02
64	->	69	-0.08	0.02
64	->	70	-0.02	0.02
64	->	71	0.00	0.00
64	->	72	-0.02	0.00
64	->	73	-0.00	0.00
64	->	74	0.02	0.00
64	->	75	0.02	0.00
64	->	76	-0.80	0.79
64	->	77	-0.04	0.02
64	->	78	-0.03	0.01
64	->	79	-0.07	0.06
64	->	80	-0.20	0.36
64	->	81	-0.03	0.01
64	->	82	-0.01	0.00
64	->	83	-0.21	0.14
64	->	84	-0.03	0.03
64	->	85	0.00	0.02
64	->	86	0.01	0.00
64	->	87	0.02	0.00
64	->	88	0.01	0.00
64	->	89	0.01	0.00
64	->	90	0.00	0.00
64	->	91	0.00	0.00
64	->	92	0.00	0.00
64	->	93	0.00	0.00
64	->	94	0.01	0.00
64	->	95	0.01	0.00
64	->	96	0.00	0.00
64	->	97	0.00	0.00
64	->	98	0.00	0.00
64	->	99	0.00	0.00
64	->	100	0.01	0.00
64	->	101	0.02	0.00
64	->	102	0.03	0.01
64	->	103	0.02	0.01
64	->	104	0.01	0.00
64	->	105	0.01	0.00

64	->	106	0.01	0.00
64	->	107	0.00	0.00
65	->	1	-0.00	0.00
65	->	2	-0.00	0.00
65	->	3	0.00	0.00
65	->	4	0.00	0.00
65	->	5	0.00	0.00
65	->	6	0.00	0.00
65	->	7	0.00	0.00
65	->	8	0.00	0.00
65	->	9	0.00	0.00
65	->	10	0.00	0.00
65	->	11	0.00	0.00
65	->	12	-0.00	0.00
65	->	13	-0.00	0.00
65	->	14	0.00	0.00
65	->	15	0.00	0.00
65	->	16	-0.00	0.00
65	->	17	0.00	0.00
65	->	18	0.00	0.00
65	->	19	0.00	0.00
65	->	20	0.00	0.00
65	->	21	0.00	0.00
65	->	22	-0.00	0.00
65	->	23	-0.00	0.00
65	->	24	0.00	0.00
65	->	25	-0.00	0.00
65	->	26	-0.00	0.00
65	->	27	0.00	0.00
65	->	28	-0.00	0.00
65	->	29	0.00	0.00
65	->	30	0.00	0.00
65	->	31	-0.00	0.00
65	->	32	0.00	0.00
65	->	33	0.00	0.00
65	->	34	0.00	0.00
65	->	35	0.00	0.00
65	->	36	-0.00	0.00
65	->	37	0.00	0.00
65	->	38	0.00	0.00
65	->	39	0.00	0.00
65	->	40	-0.00	0.00
65	->	41	0.00	0.00
65	->	42	-0.00	0.00
65	->	43	-0.00	0.00
65	->	44	-0.00	0.00
65	->	45	0.00	0.00
65	->	46	0.00	0.00
65	->	47	0.00	0.00
65	->	48	0.00	0.00

65 ->	49	0.00	0.00
65 ->	50	0.00	0.00
65 ->	51	-0.00	0.00
65 ->	52	0.00	0.00
65 ->	53	-0.00	0.00
65 ->	54	-0.00	0.00
65 ->	55	-0.00	0.00
65 ->	56	-0.00	0.00
65 ->	57	-0.00	0.00
65 ->	58	0.00	0.00
65 ->	59	-0.00	0.00
65 ->	60	-0.00	0.00
65 ->	61	-0.01	0.00
65 ->	62	-0.03	0.03
65 ->	63	1.26	1.56
65 ->	64	-12.47	0.68
65 ->	65	21.60	1.57
65 ->	66	-8.51	1.46
65 ->	67	-0.36	0.18
65 ->	68	-0.26	0.10
65 ->	69	-0.01	0.00
65 ->	70	-0.03	0.01
65 ->	71	-0.00	0.00
65 ->	72	-0.00	0.00
65 ->	73	-0.00	0.00
65 ->	74	0.00	0.00
65 ->	75	-0.00	0.00
65 ->	76	-0.04	0.02
65 ->	77	-0.01	0.00
65 ->	78	-0.02	0.00
65 ->	79	0.72	0.10
65 ->	80	-0.09	0.07
65 ->	81	-0.01	0.00
65 ->	82	-0.05	0.01
65 ->	83	0.64	0.67
65 ->	84	-0.06	0.15
65 ->	85	-0.04	0.16
65 ->	86	-0.00	0.00
65 ->	87	0.00	0.00
65 ->	88	0.00	0.00
65 ->	89	0.00	0.00
65 ->	90	0.00	0.00
65 ->	91	0.00	0.00
65 ->	92	0.00	0.00
65 ->	93	0.00	0.00
65 ->	94	0.00	0.00
65 ->	95	0.00	0.00
65 ->	96	0.00	0.00
65 ->	97	0.00	0.00
65 ->	98	0.00	0.00

65	->	99	0.00	0.00
65	->	100	0.00	0.00
65	->	101	0.00	0.00
65	->	102	0.00	0.00
65	->	103	0.00	0.00
65	->	104	0.00	0.00
65	->	105	0.00	0.00
65	->	106	0.00	0.00
65	->	107	0.00	0.00
66	->	1	-0.00	0.00
66	->	2	-0.00	0.00
66	->	3	0.00	0.00
66	->	4	-0.00	0.00
66	->	5	0.00	0.00
66	->	6	-0.00	0.00
66	->	7	0.00	0.00
66	->	8	0.00	0.00
66	->	9	0.00	0.00
66	->	10	0.00	0.00
66	->	11	0.00	0.00
66	->	12	-0.00	0.00
66	->	13	-0.00	0.00
66	->	14	-0.00	0.00
66	->	15	-0.00	0.00
66	->	16	-0.00	0.00
66	->	17	0.00	0.00
66	->	18	0.00	0.00
66	->	19	0.00	0.00
66	->	20	0.00	0.00
66	->	21	-0.00	0.00
66	->	22	-0.00	0.00
66	->	23	-0.00	0.00
66	->	24	-0.00	0.00
66	->	25	-0.00	0.00
66	->	26	-0.00	0.00
66	->	27	-0.00	0.00
66	->	28	-0.00	0.00
66	->	29	-0.00	0.00
66	->	30	0.00	0.00
66	->	31	-0.00	0.00
66	->	32	-0.00	0.00
66	->	33	-0.00	0.00
66	->	34	-0.00	0.00
66	->	35	0.01	0.00
66	->	36	-0.00	0.00
66	->	37	0.00	0.00
66	->	38	0.01	0.00
66	->	39	-0.00	0.00
66	->	40	-0.01	0.00
66	->	41	0.00	0.00

66 ->	42	-0.00	0.00
66 ->	43	-0.00	0.00
66 ->	44	-0.01	0.00
66 ->	45	0.00	0.00
66 ->	46	0.01	0.00
66 ->	47	0.00	0.00
66 ->	48	0.00	0.00
66 ->	49	0.00	0.00
66 ->	50	0.00	0.00
66 ->	51	-0.00	0.00
66 ->	52	0.00	0.00
66 ->	53	-0.02	0.00
66 ->	54	0.00	0.00
66 ->	55	-0.00	0.00
66 ->	56	0.00	0.00
66 ->	57	0.00	0.00
66 ->	58	0.02	0.01
66 ->	59	-0.06	0.01
66 ->	60	0.00	0.00
66 ->	61	-0.01	0.01
66 ->	62	-0.15	0.05
66 ->	63	3.04	0.46
66 ->	64	-0.31	0.28
66 ->	65	-8.44	1.46
66 ->	66	-30.72	3.64
66 ->	67	-8.90	0.46
66 ->	68	-4.17	2.00
66 ->	69	-0.03	0.03
66 ->	70	0.02	0.05
66 ->	71	-0.00	0.00
66 ->	72	-0.02	0.00
66 ->	73	0.00	0.00
66 ->	74	0.03	0.00
66 ->	75	0.04	0.01
66 ->	76	-0.10	0.02
66 ->	77	-0.03	0.01
66 ->	78	-0.09	0.02
66 ->	79	3.47	0.61
66 ->	80	0.04	0.02
66 ->	81	-0.03	0.01
66 ->	82	-0.07	0.03
66 ->	83	2.12	0.36
66 ->	84	-0.00	0.00
66 ->	85	-0.00	0.02
66 ->	86	0.04	0.09
66 ->	87	0.06	0.02
66 ->	88	0.02	0.00
66 ->	89	0.01	0.00
66 ->	90	0.01	0.00
66 ->	91	0.01	0.00

66	->	92	0.00	0.00
66	->	93	0.00	0.00
66	->	94	0.00	0.00
66	->	95	0.00	0.00
66	->	96	0.00	0.00
66	->	97	0.00	0.00
66	->	98	0.00	0.00
66	->	99	0.00	0.00
66	->	100	0.01	0.00
66	->	101	0.02	0.00
66	->	102	0.02	0.00
66	->	103	0.02	0.00
66	->	104	0.02	0.00
66	->	105	0.01	0.00
66	->	106	0.01	0.00
66	->	107	0.01	0.00
67	->	1	0.00	0.00
67	->	2	0.00	0.00
67	->	3	0.00	0.00
67	->	4	0.00	0.00
67	->	5	-0.00	0.00
67	->	6	0.00	0.00
67	->	7	0.00	0.00
67	->	8	0.00	0.00
67	->	9	0.00	0.00
67	->	10	0.00	0.00
67	->	11	0.00	0.00
67	->	12	0.00	0.00
67	->	13	0.00	0.00
67	->	14	0.00	0.00
67	->	15	0.00	0.00
67	->	16	-0.00	0.00
67	->	17	0.00	0.00
67	->	18	0.00	0.00
67	->	19	-0.00	0.00
67	->	20	0.00	0.00
67	->	21	0.00	0.00
67	->	22	0.00	0.00
67	->	23	0.00	0.00
67	->	24	0.00	0.00
67	->	25	-0.00	0.00
67	->	26	0.00	0.00
67	->	27	0.00	0.00
67	->	28	0.00	0.00
67	->	29	0.00	0.00
67	->	30	0.00	0.00
67	->	31	-0.00	0.00
67	->	32	0.00	0.00
67	->	33	0.00	0.00
67	->	34	0.00	0.00

67	->	35	0.00	0.00
67	->	36	-0.00	0.00
67	->	37	0.00	0.00
67	->	38	0.00	0.00
67	->	39	-0.00	0.00
67	->	40	-0.00	0.00
67	->	41	0.00	0.00
67	->	42	0.00	0.00
67	->	43	0.00	0.00
67	->	44	-0.00	0.00
67	->	45	0.00	0.00
67	->	46	0.00	0.00
67	->	47	0.00	0.00
67	->	48	0.00	0.00
67	->	49	0.00	0.00
67	->	50	0.00	0.00
67	->	51	0.00	0.00
67	->	52	0.00	0.00
67	->	53	-0.00	0.00
67	->	54	-0.00	0.00
67	->	55	-0.00	0.00
67	->	56	-0.00	0.00
67	->	57	-0.00	0.00
67	->	58	0.00	0.00
67	->	59	-0.01	0.00
67	->	60	-0.00	0.00
67	->	61	-0.02	0.01
67	->	62	-0.09	0.15
67	->	63	-0.71	0.64
67	->	64	-0.20	0.33
67	->	65	-0.36	0.19
67	->	66	-8.97	0.46
67	->	67	11.29	0.80
67	->	68	-8.71	0.48
67	->	69	-0.90	0.59
67	->	70	-0.10	0.02
67	->	71	-0.00	0.00
67	->	72	-0.00	0.00
67	->	73	-0.00	0.00
67	->	74	-0.00	0.00
67	->	75	0.00	0.00
67	->	76	-0.01	0.00
67	->	77	-0.00	0.00
67	->	78	0.00	0.00
67	->	79	-0.07	0.04
67	->	80	-0.01	0.00
67	->	81	-0.00	0.00
67	->	82	-0.00	0.00
67	->	83	-0.02	0.03
67	->	84	-0.00	0.00

67	->	85	-0.00	0.00
67	->	86	-0.01	0.01
67	->	87	-0.01	0.01
67	->	88	-0.00	0.00
67	->	89	0.00	0.00
67	->	90	0.00	0.00
67	->	91	0.00	0.00
67	->	92	0.00	0.00
67	->	93	-0.00	0.00
67	->	94	0.00	0.00
67	->	95	0.00	0.00
67	->	96	0.00	0.00
67	->	97	0.00	0.00
67	->	98	0.00	0.00
67	->	99	0.00	0.00
67	->	100	0.00	0.00
67	->	101	0.00	0.00
67	->	102	-0.00	0.00
67	->	103	-0.00	0.00
67	->	104	-0.00	0.00
67	->	105	-0.00	0.00
67	->	106	-0.00	0.00
67	->	107	-0.00	0.00
68	->	1	0.01	0.00
68	->	2	0.00	0.00
68	->	3	-0.00	0.00
68	->	4	0.00	0.00
68	->	5	-0.00	0.00
68	->	6	0.00	0.00
68	->	7	0.00	0.00
68	->	8	-0.00	0.00
68	->	9	-0.00	0.00
68	->	10	-0.00	0.00
68	->	11	-0.00	0.00
68	->	12	0.00	0.00
68	->	13	0.00	0.00
68	->	14	0.00	0.00
68	->	15	0.00	0.00
68	->	16	0.00	0.00
68	->	17	-0.00	0.00
68	->	18	-0.00	0.00
68	->	19	-0.00	0.00
68	->	20	-0.00	0.00
68	->	21	0.00	0.00
68	->	22	0.01	0.00
68	->	23	0.00	0.00
68	->	24	-0.00	0.00
68	->	25	0.00	0.00
68	->	26	0.00	0.00
68	->	27	0.00	0.00

68	->	28	0.00	0.00
68	->	29	0.00	0.00
68	->	30	-0.00	0.00
68	->	31	0.01	0.00
68	->	32	0.00	0.00
68	->	33	0.00	0.00
68	->	34	0.00	0.00
68	->	35	-0.01	0.00
68	->	36	0.01	0.00
68	->	37	-0.00	0.00
68	->	38	-0.01	0.00
68	->	39	0.00	0.00
68	->	40	0.01	0.00
68	->	41	-0.00	0.00
68	->	42	0.00	0.00
68	->	43	0.00	0.00
68	->	44	0.01	0.00
68	->	45	-0.00	0.00
68	->	46	-0.02	0.00
68	->	47	-0.00	0.00
68	->	48	-0.00	0.00
68	->	49	-0.00	0.00
68	->	50	-0.00	0.00
68	->	51	-0.00	0.00
68	->	52	-0.00	0.00
68	->	53	0.03	0.01
68	->	54	-0.01	0.01
68	->	55	-0.00	0.00
68	->	56	-0.00	0.00
68	->	57	-0.00	0.00
68	->	58	-0.03	0.02
68	->	59	0.17	0.11
68	->	60	-0.02	0.01
68	->	61	-0.28	0.09
68	->	62	-1.21	0.55
68	->	63	-2.25	0.77
68	->	64	-0.04	0.02
68	->	65	-0.26	0.11
68	->	66	-4.27	2.01
68	->	67	-8.86	0.49
68	->	68	-106.89	2.87
68	->	69	-12.59	0.89
68	->	70	-3.25	0.60
68	->	71	-0.07	0.02
68	->	72	0.02	0.01
68	->	73	-0.01	0.00
68	->	74	-0.05	0.01
68	->	75	-0.85	1.93
68	->	76	-0.07	0.06
68	->	77	0.01	0.01

68	->	78	0.09	0.03
68	->	79	-1.91	0.74
68	->	80	-0.04	0.01
68	->	81	0.02	0.01
68	->	82	-0.04	0.05
68	->	83	-0.28	0.15
68	->	84	-0.00	0.00
68	->	85	0.00	0.01
68	->	86	-2.34	2.14
68	->	87	-3.39	2.89
68	->	88	-0.08	0.03
68	->	89	-0.03	0.01
68	->	90	-0.01	0.00
68	->	91	-0.01	0.00
68	->	92	-0.01	0.00
68	->	93	-0.00	0.00
68	->	94	-0.00	0.00
68	->	95	-0.00	0.00
68	->	96	-0.00	0.00
68	->	97	-0.00	0.00
68	->	98	-0.00	0.00
68	->	99	-0.01	0.00
68	->	100	-0.01	0.00
68	->	101	-0.02	0.00
68	->	102	-0.02	0.01
68	->	103	-0.02	0.01
68	->	104	-0.02	0.01
68	->	105	-0.01	0.00
68	->	106	-0.02	0.01
68	->	107	-0.01	0.00
69	->	1	0.00	0.00
69	->	2	0.00	0.00
69	->	3	0.00	0.00
69	->	4	0.00	0.00
69	->	5	-0.00	0.00
69	->	6	0.00	0.00
69	->	7	0.00	0.00
69	->	8	-0.00	0.00
69	->	9	-0.00	0.00
69	->	10	-0.00	0.00
69	->	11	-0.00	0.00
69	->	12	0.00	0.00
69	->	13	0.00	0.00
69	->	14	0.00	0.00
69	->	15	0.00	0.00
69	->	16	0.00	0.00
69	->	17	0.00	0.00
69	->	18	-0.01	0.00
69	->	19	-0.00	0.00
69	->	20	0.00	0.00

69 ->	21	0.00	0.00
69 ->	22	0.01	0.00
69 ->	23	0.00	0.00
69 ->	24	0.00	0.00
69 ->	25	0.00	0.00
69 ->	26	0.00	0.00
69 ->	27	0.00	0.00
69 ->	28	0.00	0.00
69 ->	29	0.00	0.00
69 ->	30	-0.00	0.00
69 ->	31	0.00	0.00
69 ->	32	-0.00	0.00
69 ->	33	0.00	0.00
69 ->	34	-0.00	0.00
69 ->	35	-0.01	0.00
69 ->	36	0.01	0.00
69 ->	37	-0.00	0.00
69 ->	38	-0.01	0.00
69 ->	39	0.00	0.00
69 ->	40	0.01	0.00
69 ->	41	-0.00	0.00
69 ->	42	0.00	0.00
69 ->	43	0.00	0.00
69 ->	44	0.01	0.00
69 ->	45	-0.00	0.00
69 ->	46	-0.02	0.00
69 ->	47	-0.00	0.00
69 ->	48	-0.00	0.00
69 ->	49	0.00	0.00
69 ->	50	-0.01	0.01
69 ->	51	-0.01	0.00
69 ->	52	0.00	0.00
69 ->	53	0.10	0.03
69 ->	54	-0.03	0.03
69 ->	55	-0.01	0.00
69 ->	56	-0.02	0.02
69 ->	57	-0.10	0.45
69 ->	58	-1.17	2.36
69 ->	59	-2.38	0.86
69 ->	60	-0.62	0.63
69 ->	61	-3.09	0.81
69 ->	62	-1.49	0.46
69 ->	63	-0.61	0.14
69 ->	64	-0.08	0.02
69 ->	65	-0.01	0.00
69 ->	66	-0.03	0.03
69 ->	67	-0.95	0.59
69 ->	68	-12.59	0.89
69 ->	69	11.10	3.53
69 ->	70	-10.92	0.55

69	->	71	-0.43	0.05
69	->	72	-0.00	0.03
69	->	73	-0.04	0.02
69	->	74	-0.05	0.02
69	->	75	-0.29	0.17
69	->	76	0.10	0.05
69	->	77	-0.01	0.01
69	->	78	0.05	0.02
69	->	79	-0.02	0.02
69	->	80	-0.01	0.01
69	->	81	0.01	0.00
69	->	82	-0.00	0.00
69	->	83	-0.01	0.01
69	->	84	-0.00	0.00
69	->	85	-0.00	0.00
69	->	86	-0.05	0.04
69	->	87	-0.14	0.08
69	->	88	-0.06	0.03
69	->	89	-0.02	0.01
69	->	90	-0.01	0.00
69	->	91	-0.01	0.00
69	->	92	-0.00	0.00
69	->	93	-0.00	0.00
69	->	94	-0.01	0.00
69	->	95	-0.01	0.00
69	->	96	-0.00	0.00
69	->	97	-0.00	0.00
69	->	98	-0.00	0.00
69	->	99	-0.01	0.00
69	->	100	-0.03	0.01
69	->	101	-0.05	0.02
69	->	102	-0.04	0.02
69	->	103	-0.02	0.00
69	->	104	-0.01	0.00
69	->	105	-0.01	0.00
69	->	106	-0.01	0.00
69	->	107	-0.00	0.00
70	->	1	0.00	0.00
70	->	2	0.00	0.00
70	->	3	-0.00	0.00
70	->	4	0.00	0.00
70	->	5	-0.00	0.00
70	->	6	0.00	0.00
70	->	7	0.00	0.00
70	->	8	-0.00	0.00
70	->	9	0.00	0.00
70	->	10	-0.00	0.00
70	->	11	-0.00	0.00
70	->	12	0.00	0.00
70	->	13	0.00	0.00

70	->	14	0.00	0.00
70	->	15	0.00	0.00
70	->	16	0.00	0.00
70	->	17	-0.00	0.00
70	->	18	-0.00	0.00
70	->	19	-0.00	0.00
70	->	20	0.00	0.00
70	->	21	0.00	0.00
70	->	22	0.00	0.00
70	->	23	0.00	0.00
70	->	24	-0.00	0.00
70	->	25	0.00	0.00
70	->	26	0.00	0.00
70	->	27	0.00	0.00
70	->	28	0.00	0.00
70	->	29	0.00	0.00
70	->	30	-0.00	0.00
70	->	31	0.00	0.00
70	->	32	0.00	0.00
70	->	33	-0.00	0.00
70	->	34	0.00	0.00
70	->	35	-0.00	0.00
70	->	36	-0.00	0.00
70	->	37	-0.00	0.00
70	->	38	-0.00	0.00
70	->	39	-0.00	0.00
70	->	40	0.00	0.00
70	->	41	0.00	0.00
70	->	42	-0.00	0.00
70	->	43	-0.00	0.00
70	->	44	0.00	0.00
70	->	45	0.00	0.00
70	->	46	-0.01	0.01
70	->	47	-0.00	0.00
70	->	48	0.00	0.00
70	->	49	0.00	0.01
70	->	50	-0.04	0.01
70	->	51	-0.01	0.01
70	->	52	0.00	0.00
70	->	53	0.04	0.08
70	->	54	-0.04	0.03
70	->	55	-0.01	0.00
70	->	56	-0.01	0.01
70	->	57	-0.00	0.01
70	->	58	-0.23	0.12
70	->	59	-3.09	0.57
70	->	60	-0.97	0.16
70	->	61	-3.69	0.43
70	->	62	-0.93	0.37
70	->	63	-0.82	0.30

70	->	64	-0.02	0.02
70	->	65	-0.03	0.01
70	->	66	0.01	0.05
70	->	67	-0.10	0.02
70	->	68	-3.21	0.59
70	->	69	-10.99	0.54
70	->	70	30.20	1.10
70	->	71	-9.76	0.37
70	->	72	-0.93	0.25
70	->	73	-0.24	0.08
70	->	74	-0.14	0.04
70	->	75	-4.25	1.03
70	->	76	-2.73	0.49
70	->	77	-0.06	0.02
70	->	78	-0.25	0.10
70	->	79	-1.99	0.42
70	->	80	-0.05	0.02
70	->	81	-0.01	0.01
70	->	82	-0.03	0.01
70	->	83	-0.00	0.01
70	->	84	-0.00	0.00
70	->	85	-0.00	0.00
70	->	86	-0.24	0.38
70	->	87	-0.32	0.33
70	->	88	-0.09	0.05
70	->	89	-0.03	0.02
70	->	90	-0.01	0.00
70	->	91	-0.00	0.00
70	->	92	-0.00	0.00
70	->	93	-0.00	0.00
70	->	94	-0.00	0.00
70	->	95	-0.00	0.00
70	->	96	-0.00	0.00
70	->	97	0.00	0.00
70	->	98	-0.00	0.00
70	->	99	-0.00	0.00
70	->	100	-0.02	0.01
70	->	101	-0.04	0.02
70	->	102	-0.02	0.01
70	->	103	-0.01	0.00
70	->	104	-0.01	0.00
70	->	105	-0.01	0.00
70	->	106	-0.01	0.00
70	->	107	-0.00	0.00
71	->	1	-0.00	0.00
71	->	2	0.00	0.00
71	->	3	-0.00	0.00
71	->	4	0.00	0.00
71	->	5	0.00	0.00
71	->	6	-0.00	0.00

71 ->	7	0.00	0.00
71 ->	8	-0.00	0.00
71 ->	9	-0.00	0.00
71 ->	10	-0.00	0.00
71 ->	11	-0.00	0.00
71 ->	12	0.00	0.00
71 ->	13	-0.00	0.00
71 ->	14	-0.00	0.00
71 ->	15	-0.00	0.00
71 ->	16	-0.00	0.00
71 ->	17	-0.00	0.00
71 ->	18	0.00	0.00
71 ->	19	0.00	0.00
71 ->	20	-0.00	0.00
71 ->	21	-0.00	0.00
71 ->	22	-0.00	0.00
71 ->	23	-0.00	0.00
71 ->	24	0.00	0.00
71 ->	25	-0.00	0.00
71 ->	26	-0.00	0.00
71 ->	27	0.00	0.00
71 ->	28	-0.00	0.00
71 ->	29	0.00	0.00
71 ->	30	0.00	0.00
71 ->	31	0.00	0.00
71 ->	32	-0.00	0.00
71 ->	33	-0.00	0.00
71 ->	34	0.00	0.00
71 ->	35	-0.00	0.00
71 ->	36	-0.00	0.00
71 ->	37	-0.00	0.00
71 ->	38	-0.00	0.00
71 ->	39	-0.00	0.00
71 ->	40	0.00	0.00
71 ->	41	0.00	0.00
71 ->	42	-0.00	0.00
71 ->	43	0.00	0.00
71 ->	44	0.00	0.00
71 ->	45	-0.00	0.00
71 ->	46	-0.03	0.02
71 ->	47	-0.02	0.01
71 ->	48	-0.01	0.01
71 ->	49	-0.13	0.10
71 ->	50	-0.68	0.44
71 ->	51	-0.03	0.02
71 ->	52	-0.03	0.02
71 ->	53	-4.75	1.11
71 ->	54	-0.42	0.25
71 ->	55	-0.01	0.00
71 ->	56	-0.02	0.00

71	->	57	-0.03	0.01
71	->	58	-0.55	0.39
71	->	59	-1.88	0.34
71	->	60	-0.83	0.21
71	->	61	-0.91	0.24
71	->	62	-0.01	0.01
71	->	63	0.01	0.01
71	->	64	0.00	0.00
71	->	65	-0.00	0.00
71	->	66	-0.00	0.00
71	->	67	-0.00	0.00
71	->	68	-0.07	0.02
71	->	69	-0.43	0.05
71	->	70	-9.76	0.37
71	->	71	24.40	1.10
71	->	72	-7.73	0.47
71	->	73	-0.30	0.04
71	->	74	-0.18	0.09
71	->	75	-3.26	1.82
71	->	76	-0.03	0.02
71	->	77	-0.00	0.00
71	->	78	0.00	0.02
71	->	79	-0.00	0.00
71	->	80	-0.00	0.00
71	->	81	0.00	0.00
71	->	82	-0.00	0.00
71	->	83	0.00	0.00
71	->	84	0.00	0.00
71	->	85	0.00	0.00
71	->	86	-0.00	0.01
71	->	87	-0.04	0.03
71	->	88	-0.14	0.15
71	->	89	-0.06	0.03
71	->	90	-0.02	0.01
71	->	91	-0.00	0.00
71	->	92	0.00	0.00
71	->	93	0.00	0.00
71	->	94	-0.00	0.00
71	->	95	-0.00	0.00
71	->	96	0.00	0.00
71	->	97	0.00	0.00
71	->	98	0.00	0.00
71	->	99	-0.00	0.00
71	->	100	0.00	0.03
71	->	101	0.04	0.09
71	->	102	-0.03	0.02
71	->	103	-0.01	0.01
71	->	104	0.00	0.00
71	->	105	-0.00	0.00
71	->	106	-0.00	0.00

71 ->	107	-0.00	0.00
72 ->	1	0.03	0.01
72 ->	2	0.00	0.00
72 ->	3	-0.00	0.00
72 ->	4	0.00	0.00
72 ->	5	-0.01	0.00
72 ->	6	0.00	0.00
72 ->	7	-0.00	0.00
72 ->	8	-0.00	0.00
72 ->	9	-0.00	0.00
72 ->	10	-0.01	0.00
72 ->	11	-0.01	0.00
72 ->	12	0.02	0.00
72 ->	13	0.01	0.00
72 ->	14	0.00	0.00
72 ->	15	0.00	0.00
72 ->	16	0.02	0.00
72 ->	17	-0.00	0.00
72 ->	18	-0.03	0.01
72 ->	19	-0.03	0.01
72 ->	20	-0.00	0.00
72 ->	21	0.00	0.00
72 ->	22	0.06	0.02
72 ->	23	0.00	0.00
72 ->	24	0.00	0.00
72 ->	25	0.03	0.01
72 ->	26	0.00	0.00
72 ->	27	0.00	0.00
72 ->	28	0.00	0.00
72 ->	29	0.00	0.00
72 ->	30	-0.00	0.00
72 ->	31	0.03	0.00
72 ->	32	-0.00	0.00
72 ->	33	-0.00	0.00
72 ->	34	-0.00	0.00
72 ->	35	-0.03	0.00
72 ->	36	0.02	0.00
72 ->	37	-0.00	0.00
72 ->	38	-0.03	0.01
72 ->	39	0.00	0.00
72 ->	40	0.04	0.01
72 ->	41	0.00	0.00
72 ->	42	-0.01	0.01
72 ->	43	-0.00	0.01
72 ->	44	0.45	0.44
72 ->	45	0.01	0.03
72 ->	46	-3.12	3.71
72 ->	47	-0.44	0.31
72 ->	48	-0.01	0.03
72 ->	49	0.01	0.14

72	->	50	-2.04	0.79
72	->	51	-0.07	0.03
72	->	52	-0.02	0.01
72	->	53	-0.47	0.23
72	->	54	-0.07	0.05
72	->	55	-0.00	0.00
72	->	56	-0.00	0.00
72	->	57	-0.01	0.00
72	->	58	-0.03	0.01
72	->	59	-0.05	0.05
72	->	60	-0.15	0.06
72	->	61	-2.02	0.66
72	->	62	-0.03	0.01
72	->	63	-0.04	0.01
72	->	64	-0.02	0.00
72	->	65	-0.00	0.00
72	->	66	-0.02	0.00
72	->	67	-0.00	0.00
72	->	68	0.02	0.01
72	->	69	-0.00	0.03
72	->	70	-0.93	0.25
72	->	71	-7.79	0.48
72	->	72	-92.73	3.41
72	->	73	-21.63	0.55
72	->	74	-11.56	5.33
72	->	75	-4.54	2.84
72	->	76	-0.88	0.44
72	->	77	0.13	0.10
72	->	78	0.30	0.52
72	->	79	-0.01	0.01
72	->	80	-0.01	0.01
72	->	81	0.04	0.01
72	->	82	-0.00	0.00
72	->	83	-0.00	0.00
72	->	84	-0.00	0.00
72	->	85	0.00	0.01
72	->	86	-0.03	0.02
72	->	87	-0.22	0.28
72	->	88	-1.31	1.34
72	->	89	-5.07	3.25
72	->	90	-4.69	1.95
72	->	91	-0.83	0.96
72	->	92	-0.15	0.20
72	->	93	0.01	0.03
72	->	94	-0.04	0.01
72	->	95	-0.02	0.01
72	->	96	-0.01	0.00
72	->	97	-0.00	0.00
72	->	98	-0.02	0.00
72	->	99	-0.04	0.01

72	->	100	-0.52	0.39
72	->	101	-1.28	0.92
72	->	102	-0.62	0.35
72	->	103	-0.19	0.68
72	->	104	0.83	0.65
72	->	105	0.05	0.25
72	->	106	0.04	0.13
72	->	107	-0.03	0.02
73	->	1	0.00	0.00
73	->	2	0.00	0.00
73	->	3	0.00	0.00
73	->	4	0.00	0.00
73	->	5	-0.00	0.00
73	->	6	0.00	0.00
73	->	7	0.00	0.00
73	->	8	-0.00	0.00
73	->	9	0.00	0.00
73	->	10	-0.00	0.00
73	->	11	0.00	0.00
73	->	12	0.00	0.00
73	->	13	0.00	0.00
73	->	14	0.00	0.00
73	->	15	0.00	0.00
73	->	16	0.00	0.00
73	->	17	0.00	0.00
73	->	18	-0.00	0.00
73	->	19	-0.00	0.00
73	->	20	0.00	0.00
73	->	21	0.00	0.00
73	->	22	0.00	0.00
73	->	23	0.00	0.00
73	->	24	0.00	0.00
73	->	25	0.00	0.00
73	->	26	0.00	0.00
73	->	27	0.00	0.00
73	->	28	0.00	0.00
73	->	29	0.00	0.00
73	->	30	-0.00	0.00
73	->	31	0.00	0.00
73	->	32	0.00	0.00
73	->	33	0.00	0.00
73	->	34	-0.00	0.00
73	->	35	-0.00	0.00
73	->	36	0.00	0.00
73	->	37	0.00	0.00
73	->	38	-0.00	0.00
73	->	39	-0.00	0.00
73	->	40	-0.00	0.00
73	->	41	-0.00	0.00
73	->	42	-0.00	0.00

73	->	43	-0.00	0.00
73	->	44	-0.01	0.01
73	->	45	-0.00	0.00
73	->	46	0.01	0.02
73	->	47	-0.00	0.00
73	->	48	-0.00	0.00
73	->	49	-0.02	0.01
73	->	50	-0.01	0.02
73	->	51	0.00	0.00
73	->	52	-0.00	0.00
73	->	53	-0.26	0.11
73	->	54	0.00	0.00
73	->	55	-0.00	0.00
73	->	56	-0.00	0.00
73	->	57	-0.00	0.00
73	->	58	-0.01	0.00
73	->	59	-0.06	0.02
73	->	60	-0.06	0.02
73	->	61	-1.94	0.71
73	->	62	-0.02	0.01
73	->	63	-0.01	0.01
73	->	64	-0.00	0.00
73	->	65	-0.00	0.00
73	->	66	0.00	0.00
73	->	67	-0.00	0.00
73	->	68	-0.01	0.00
73	->	69	-0.04	0.02
73	->	70	-0.24	0.08
73	->	71	-0.30	0.04
73	->	72	-21.63	0.55
73	->	73	24.60	2.29
73	->	74	-10.59	0.56
73	->	75	-0.81	0.18
73	->	76	-2.94	0.69
73	->	77	-1.99	0.94
73	->	78	-0.12	0.09
73	->	79	-0.05	0.03
73	->	80	-0.02	0.02
73	->	81	-0.01	0.01
73	->	82	-0.00	0.00
73	->	83	-0.00	0.00
73	->	84	-0.00	0.00
73	->	85	-0.00	0.00
73	->	86	-0.00	0.00
73	->	87	-0.00	0.00
73	->	88	-0.01	0.01
73	->	89	-0.01	0.01
73	->	90	-0.01	0.01
73	->	91	0.00	0.00
73	->	92	0.00	0.01

73	->	93	-0.02	0.02
73	->	94	-0.01	0.00
73	->	95	-0.01	0.00
73	->	96	-0.00	0.00
73	->	97	-0.00	0.00
73	->	98	-0.00	0.00
73	->	99	-0.00	0.00
73	->	100	0.04	0.15
73	->	101	-1.30	0.94
73	->	102	-5.00	1.87
73	->	103	-0.24	0.22
73	->	104	-0.03	0.02
73	->	105	-0.01	0.00
73	->	106	-0.00	0.00
73	->	107	-0.00	0.00
74	->	1	-0.03	0.00
74	->	2	-0.00	0.00
74	->	3	0.00	0.00
74	->	4	-0.00	0.00
74	->	5	0.01	0.00
74	->	6	-0.00	0.00
74	->	7	0.00	0.00
74	->	8	0.00	0.00
74	->	9	0.00	0.00
74	->	10	0.01	0.00
74	->	11	0.01	0.00
74	->	12	-0.02	0.01
74	->	13	-0.01	0.00
74	->	14	-0.00	0.00
74	->	15	-0.00	0.00
74	->	16	-0.02	0.00
74	->	17	0.00	0.00
74	->	18	0.02	0.00
74	->	19	0.02	0.00
74	->	20	0.00	0.00
74	->	21	-0.00	0.00
74	->	22	-0.04	0.00
74	->	23	-0.00	0.00
74	->	24	-0.00	0.00
74	->	25	-0.02	0.00
74	->	26	-0.00	0.00
74	->	27	-0.00	0.00
74	->	28	-0.00	0.00
74	->	29	-0.00	0.00
74	->	30	0.00	0.00
74	->	31	-0.02	0.00
74	->	32	-0.00	0.00
74	->	33	-0.00	0.00
74	->	34	-0.00	0.00
74	->	35	0.02	0.00

74	->	36	-0.01	0.00
74	->	37	0.00	0.00
74	->	38	0.02	0.00
74	->	39	-0.00	0.00
74	->	40	-0.03	0.00
74	->	41	0.00	0.00
74	->	42	-0.00	0.00
74	->	43	-0.00	0.00
74	->	44	-0.09	0.03
74	->	45	-0.00	0.00
74	->	46	0.13	0.10
74	->	47	0.02	0.02
74	->	48	-0.00	0.00
74	->	49	-0.03	0.02
74	->	50	-0.01	0.03
74	->	51	-0.00	0.00
74	->	52	-0.00	0.00
74	->	53	-0.16	0.05
74	->	54	-0.00	0.00
74	->	55	-0.00	0.00
74	->	56	-0.00	0.00
74	->	57	-0.00	0.00
74	->	58	0.02	0.01
74	->	59	-0.07	0.02
74	->	60	-0.01	0.00
74	->	61	0.01	0.02
74	->	62	0.00	0.00
74	->	63	0.04	0.01
74	->	64	0.02	0.00
74	->	65	0.00	0.00
74	->	66	0.03	0.00
74	->	67	-0.00	0.00
74	->	68	-0.05	0.01
74	->	69	-0.05	0.02
74	->	70	-0.14	0.04
74	->	71	-0.18	0.09
74	->	72	-11.50	5.31
74	->	73	-10.63	0.56
74	->	74	-14.52	4.03
74	->	75	-13.46	0.70
74	->	76	-1.73	0.32
74	->	77	-3.12	0.90
74	->	78	-3.88	3.75
74	->	79	-0.11	0.06
74	->	80	-0.05	0.02
74	->	81	-0.16	0.13
74	->	82	-0.01	0.00
74	->	83	-0.01	0.00
74	->	84	-0.00	0.00
74	->	85	-0.01	0.01

74	->	86	-0.00	0.01
74	->	87	0.02	0.02
74	->	88	-0.21	0.24
74	->	89	0.43	0.28
74	->	90	0.30	0.19
74	->	91	-0.05	0.04
74	->	92	-0.05	0.04
74	->	93	-0.02	0.01
74	->	94	0.02	0.00
74	->	95	0.02	0.00
74	->	96	0.01	0.00
74	->	97	0.00	0.00
74	->	98	0.01	0.00
74	->	99	0.02	0.00
74	->	100	0.09	0.05
74	->	101	0.07	0.16
74	->	102	-0.56	0.81
74	->	103	-1.77	0.58
74	->	104	-3.45	1.49
74	->	105	-0.67	0.43
74	->	106	0.02	0.06
74	->	107	0.01	0.00
75	->	1	-0.01	0.00
75	->	2	-0.00	0.00
75	->	3	0.00	0.00
75	->	4	-0.00	0.00
75	->	5	0.00	0.00
75	->	6	-0.00	0.00
75	->	7	0.00	0.00
75	->	8	0.00	0.00
75	->	9	0.00	0.00
75	->	10	0.00	0.00
75	->	11	0.01	0.00
75	->	12	-0.01	0.00
75	->	13	-0.00	0.00
75	->	14	-0.00	0.00
75	->	15	-0.00	0.00
75	->	16	-0.01	0.00
75	->	17	0.00	0.00
75	->	18	0.01	0.00
75	->	19	0.01	0.00
75	->	20	0.00	0.00
75	->	21	-0.00	0.00
75	->	22	-0.02	0.00
75	->	23	-0.00	0.00
75	->	24	-0.00	0.00
75	->	25	-0.01	0.00
75	->	26	-0.00	0.00
75	->	27	-0.00	0.00
75	->	28	-0.00	0.00

75	->	29	-0.00	0.00
75	->	30	0.00	0.00
75	->	31	-0.02	0.00
75	->	32	-0.00	0.00
75	->	33	-0.00	0.00
75	->	34	-0.00	0.00
75	->	35	0.02	0.00
75	->	36	-0.01	0.00
75	->	37	0.00	0.00
75	->	38	0.02	0.00
75	->	39	-0.00	0.00
75	->	40	-0.03	0.01
75	->	41	0.00	0.00
75	->	42	-0.00	0.00
75	->	43	-0.00	0.00
75	->	44	-0.04	0.01
75	->	45	0.00	0.00
75	->	46	0.05	0.01
75	->	47	0.00	0.00
75	->	48	-0.00	0.00
75	->	49	-0.02	0.02
75	->	50	-0.09	0.10
75	->	51	0.00	0.01
75	->	52	-0.00	0.00
75	->	53	-0.20	0.10
75	->	54	-0.01	0.04
75	->	55	-0.00	0.00
75	->	56	-0.00	0.00
75	->	57	-0.01	0.00
75	->	58	0.05	0.02
75	->	59	-0.85	0.56
75	->	60	-0.02	0.01
75	->	61	-0.14	0.06
75	->	62	-0.01	0.01
75	->	63	-0.01	0.02
75	->	64	0.02	0.00
75	->	65	-0.00	0.00
75	->	66	0.04	0.01
75	->	67	0.00	0.00
75	->	68	-0.84	1.91
75	->	69	-0.29	0.17
75	->	70	-4.22	1.02
75	->	71	-3.20	1.80
75	->	72	-4.52	2.81
75	->	73	-0.82	0.18
75	->	74	-13.52	0.71
75	->	75	-46.21	3.87
75	->	76	-7.60	0.57
75	->	77	-1.29	0.20
75	->	78	-4.14	0.78

75	->	79	-3.26	0.76
75	->	80	-0.18	0.07
75	->	81	-0.10	0.03
75	->	82	-0.04	0.02
75	->	83	-0.02	0.01
75	->	84	-0.00	0.00
75	->	85	-0.01	0.01
75	->	86	-0.07	0.12
75	->	87	-0.22	0.52
75	->	88	-1.45	1.71
75	->	89	0.06	0.18
75	->	90	0.03	0.05
75	->	91	0.01	0.01
75	->	92	0.00	0.00
75	->	93	0.00	0.00
75	->	94	0.01	0.00
75	->	95	0.01	0.00
75	->	96	0.01	0.00
75	->	97	0.00	0.00
75	->	98	0.01	0.00
75	->	99	0.01	0.00
75	->	100	0.02	0.01
75	->	101	0.02	0.02
75	->	102	-0.01	0.03
75	->	103	-0.02	0.05
75	->	104	-0.09	0.42
75	->	105	-0.08	0.11
75	->	106	0.00	0.02
75	->	107	0.01	0.00
76	->	1	0.01	0.00
76	->	2	0.00	0.00
76	->	3	-0.00	0.00
76	->	4	0.00	0.00
76	->	5	-0.00	0.00
76	->	6	0.00	0.00
76	->	7	0.00	0.00
76	->	8	-0.00	0.00
76	->	9	-0.00	0.00
76	->	10	-0.00	0.00
76	->	11	-0.00	0.00
76	->	12	0.01	0.00
76	->	13	0.00	0.00
76	->	14	0.00	0.00
76	->	15	0.00	0.00
76	->	16	0.01	0.00
76	->	17	0.00	0.00
76	->	18	-0.01	0.00
76	->	19	-0.01	0.00
76	->	20	0.00	0.00
76	->	21	0.00	0.00

76 ->	22	0.02	0.00
76 ->	23	0.00	0.00
76 ->	24	0.00	0.00
76 ->	25	0.01	0.00
76 ->	26	0.00	0.00
76 ->	27	0.00	0.00
76 ->	28	0.00	0.00
76 ->	29	0.00	0.00
76 ->	30	-0.00	0.00
76 ->	31	0.01	0.00
76 ->	32	0.00	0.00
76 ->	33	0.00	0.00
76 ->	34	-0.00	0.00
76 ->	35	-0.01	0.00
76 ->	36	0.01	0.00
76 ->	37	-0.00	0.00
76 ->	38	-0.01	0.00
76 ->	39	0.00	0.00
76 ->	40	0.01	0.00
76 ->	41	-0.00	0.00
76 ->	42	0.00	0.00
76 ->	43	0.00	0.00
76 ->	44	0.02	0.01
76 ->	45	-0.00	0.00
76 ->	46	-0.03	0.00
76 ->	47	-0.00	0.00
76 ->	48	-0.00	0.00
76 ->	49	-0.00	0.00
76 ->	50	-0.01	0.00
76 ->	51	-0.00	0.00
76 ->	52	-0.00	0.00
76 ->	53	0.05	0.02
76 ->	54	-0.00	0.00
76 ->	55	0.00	0.00
76 ->	56	-0.00	0.00
76 ->	57	-0.00	0.00
76 ->	58	-0.04	0.01
76 ->	59	0.03	0.02
76 ->	60	-0.04	0.02
76 ->	61	-1.37	0.76
76 ->	62	-2.41	1.57
76 ->	63	-1.61	0.65
76 ->	64	-0.79	0.78
76 ->	65	-0.04	0.02
76 ->	66	-0.10	0.02
76 ->	67	-0.01	0.00
76 ->	68	-0.07	0.06
76 ->	69	0.10	0.05
76 ->	70	-2.78	0.49
76 ->	71	-0.03	0.02

76 ->	72	-0.89	0.44
76 ->	73	-2.97	0.68
76 ->	74	-1.73	0.32
76 ->	75	-7.64	0.56
76 ->	76	-104.50	3.05
76 ->	77	-11.94	0.79
76 ->	78	-0.86	0.25
76 ->	79	-2.14	0.34
76 ->	80	-3.51	1.55
76 ->	81	0.01	0.06
76 ->	82	-0.04	0.02
76 ->	83	-0.07	0.02
76 ->	84	-0.01	0.01
76 ->	85	-0.01	0.01
76 ->	86	-0.03	0.02
76 ->	87	-0.04	0.01
76 ->	88	-0.03	0.01
76 ->	89	-0.02	0.00
76 ->	90	-0.02	0.00
76 ->	91	-0.01	0.00
76 ->	92	-0.01	0.00
76 ->	93	-0.01	0.01
76 ->	94	-0.02	0.01
76 ->	95	-0.02	0.01
76 ->	96	-0.01	0.01
76 ->	97	-0.00	0.00
76 ->	98	-0.01	0.00
76 ->	99	-0.02	0.00
76 ->	100	-0.05	0.02
76 ->	101	-0.32	0.41
76 ->	102	-2.07	3.70
76 ->	103	-0.64	1.15
76 ->	104	-0.08	0.04
76 ->	105	-0.02	0.01
76 ->	106	-0.02	0.00
76 ->	107	-0.01	0.00
77 ->	1	0.03	0.01
77 ->	2	0.00	0.00
77 ->	3	-0.00	0.00
77 ->	4	0.00	0.00
77 ->	5	-0.00	0.00
77 ->	6	0.00	0.00
77 ->	7	0.00	0.00
77 ->	8	-0.00	0.00
77 ->	9	-0.00	0.00
77 ->	10	-0.01	0.00
77 ->	11	-0.01	0.00
77 ->	12	0.02	0.00
77 ->	13	0.01	0.00
77 ->	14	0.00	0.00

77	->	15	0.00	0.00
77	->	16	0.02	0.00
77	->	17	-0.00	0.00
77	->	18	-0.02	0.00
77	->	19	-0.02	0.00
77	->	20	0.00	0.00
77	->	21	0.00	0.00
77	->	22	0.02	0.00
77	->	23	0.00	0.00
77	->	24	0.00	0.00
77	->	25	0.01	0.00
77	->	26	0.00	0.00
77	->	27	0.00	0.00
77	->	28	0.00	0.00
77	->	29	0.00	0.00
77	->	30	-0.00	0.00
77	->	31	0.01	0.00
77	->	32	0.00	0.00
77	->	33	0.00	0.00
77	->	34	0.00	0.00
77	->	35	-0.01	0.00
77	->	36	0.01	0.00
77	->	37	-0.00	0.00
77	->	38	-0.01	0.00
77	->	39	0.00	0.00
77	->	40	0.01	0.00
77	->	41	0.00	0.00
77	->	42	0.00	0.00
77	->	43	0.00	0.00
77	->	44	0.03	0.01
77	->	45	-0.00	0.00
77	->	46	-0.04	0.01
77	->	47	-0.00	0.00
77	->	48	-0.00	0.00
77	->	49	-0.00	0.00
77	->	50	-0.00	0.00
77	->	51	0.00	0.00
77	->	52	0.00	0.00
77	->	53	0.03	0.01
77	->	54	0.00	0.00
77	->	55	0.00	0.00
77	->	56	0.00	0.00
77	->	57	0.00	0.00
77	->	58	-0.02	0.01
77	->	59	0.01	0.01
77	->	60	-0.01	0.00
77	->	61	-0.05	0.04
77	->	62	-0.01	0.01
77	->	63	-0.02	0.03
77	->	64	-0.04	0.02

77 ->	65	-0.01	0.00
77 ->	66	-0.03	0.01
77 ->	67	-0.00	0.00
77 ->	68	0.01	0.01
77 ->	69	-0.01	0.01
77 ->	70	-0.06	0.02
77 ->	71	-0.00	0.00
77 ->	72	0.13	0.10
77 ->	73	-2.03	0.95
77 ->	74	-3.14	0.90
77 ->	75	-1.30	0.20
77 ->	76	-11.98	0.82
77 ->	77	15.31	4.92
77 ->	78	-6.59	0.46
77 ->	79	-0.91	0.18
77 ->	80	-2.11	0.28
77 ->	81	-1.49	0.59
77 ->	82	-0.06	0.04
77 ->	83	-0.04	0.01
77 ->	84	-0.04	0.03
77 ->	85	-0.01	0.01
77 ->	86	-0.03	0.03
77 ->	87	-0.04	0.01
77 ->	88	-0.01	0.00
77 ->	89	-0.03	0.01
77 ->	90	-0.03	0.01
77 ->	91	-0.01	0.01
77 ->	92	-0.00	0.02
77 ->	93	-0.02	0.02
77 ->	94	-0.04	0.01
77 ->	95	-0.03	0.01
77 ->	96	-0.02	0.01
77 ->	97	-0.00	0.00
77 ->	98	-0.01	0.00
77 ->	99	-0.01	0.00
77 ->	100	-0.04	0.04
77 ->	101	-0.38	0.52
77 ->	102	-3.48	3.87
77 ->	103	-7.56	3.42
77 ->	104	-2.36	2.72
77 ->	105	-0.05	0.02
77 ->	106	-0.04	0.01
77 ->	107	-0.01	0.00
78 ->	1	0.02	0.00
78 ->	2	0.00	0.00
78 ->	3	-0.00	0.00
78 ->	4	0.00	0.00
78 ->	5	-0.00	0.00
78 ->	6	0.00	0.00
78 ->	7	-0.00	0.00

78	->	8	-0.00	0.00
78	->	9	-0.00	0.00
78	->	10	-0.01	0.00
78	->	11	-0.01	0.00
78	->	12	0.02	0.00
78	->	13	0.00	0.00
78	->	14	0.00	0.00
78	->	15	0.00	0.00
78	->	16	0.01	0.00
78	->	17	-0.00	0.00
78	->	18	-0.01	0.00
78	->	19	-0.01	0.00
78	->	20	-0.00	0.00
78	->	21	0.00	0.00
78	->	22	0.02	0.01
78	->	23	0.00	0.00
78	->	24	0.00	0.00
78	->	25	0.01	0.00
78	->	26	0.00	0.00
78	->	27	0.00	0.00
78	->	28	0.00	0.00
78	->	29	0.00	0.00
78	->	30	-0.00	0.00
78	->	31	0.02	0.00
78	->	32	0.00	0.00
78	->	33	0.00	0.00
78	->	34	0.00	0.00
78	->	35	-0.01	0.00
78	->	36	0.01	0.00
78	->	37	-0.00	0.00
78	->	38	-0.02	0.00
78	->	39	0.00	0.00
78	->	40	0.03	0.00
78	->	41	-0.00	0.00
78	->	42	0.00	0.00
78	->	43	0.00	0.00
78	->	44	0.03	0.02
78	->	45	-0.00	0.00
78	->	46	-0.05	0.02
78	->	47	-0.01	0.01
78	->	48	-0.00	0.00
78	->	49	0.00	0.01
78	->	50	-0.01	0.01
78	->	51	0.00	0.00
78	->	52	-0.00	0.00
78	->	53	0.04	0.01
78	->	54	-0.00	0.00
78	->	55	0.00	0.00
78	->	56	0.00	0.00
78	->	57	0.00	0.00

78	->	58	-0.02	0.00
78	->	59	0.07	0.02
78	->	60	-0.00	0.00
78	->	61	-0.01	0.01
78	->	62	0.00	0.01
78	->	63	-0.19	0.04
78	->	64	-0.03	0.01
78	->	65	-0.02	0.00
78	->	66	-0.09	0.02
78	->	67	0.00	0.00
78	->	68	0.09	0.03
78	->	69	0.05	0.02
78	->	70	-0.25	0.09
78	->	71	0.00	0.02
78	->	72	0.30	0.52
78	->	73	-0.12	0.09
78	->	74	-3.92	3.76
78	->	75	-4.17	0.77
78	->	76	-0.86	0.25
78	->	77	-6.62	0.46
78	->	78	-101.79	3.71
78	->	79	-19.55	0.49
78	->	80	-0.68	0.19
78	->	81	-2.04	0.60
78	->	82	-1.62	1.02
78	->	83	-0.13	0.05
78	->	84	-0.02	0.01
78	->	85	-0.02	0.02
78	->	86	-2.07	2.31
78	->	87	-5.35	3.25
78	->	88	-0.66	0.74
78	->	89	-0.26	0.45
78	->	90	-0.09	0.11
78	->	91	-0.02	0.02
78	->	92	-0.02	0.02
78	->	93	-0.02	0.01
78	->	94	-0.02	0.01
78	->	95	-0.01	0.00
78	->	96	-0.01	0.00
78	->	97	-0.00	0.00
78	->	98	-0.01	0.00
78	->	99	-0.01	0.00
78	->	100	-0.02	0.01
78	->	101	-0.05	0.03
78	->	102	-0.14	0.39
78	->	103	-0.61	1.94
78	->	104	-0.57	1.03
78	->	105	-0.87	0.79
78	->	106	-2.41	2.47
78	->	107	-0.03	0.22

79	->	1	0.00	0.00
79	->	2	0.00	0.00
79	->	3	-0.00	0.00
79	->	4	0.00	0.00
79	->	5	-0.00	0.00
79	->	6	0.00	0.00
79	->	7	0.00	0.00
79	->	8	-0.00	0.00
79	->	9	0.00	0.00
79	->	10	-0.00	0.00
79	->	11	-0.00	0.00
79	->	12	0.00	0.00
79	->	13	0.00	0.00
79	->	14	0.00	0.00
79	->	15	0.00	0.00
79	->	16	0.00	0.00
79	->	17	0.00	0.00
79	->	18	-0.00	0.00
79	->	19	-0.00	0.00
79	->	20	0.00	0.00
79	->	21	0.00	0.00
79	->	22	0.00	0.00
79	->	23	0.00	0.00
79	->	24	0.00	0.00
79	->	25	0.00	0.00
79	->	26	0.00	0.00
79	->	27	0.00	0.00
79	->	28	0.00	0.00
79	->	29	0.00	0.00
79	->	30	-0.00	0.00
79	->	31	0.00	0.00
79	->	32	0.00	0.00
79	->	33	0.00	0.00
79	->	34	0.00	0.00
79	->	35	-0.00	0.00
79	->	36	0.00	0.00
79	->	37	-0.00	0.00
79	->	38	-0.00	0.00
79	->	39	0.00	0.00
79	->	40	0.00	0.00
79	->	41	-0.00	0.00
79	->	42	0.00	0.00
79	->	43	0.00	0.00
79	->	44	0.00	0.00
79	->	45	-0.00	0.00
79	->	46	-0.00	0.00
79	->	47	-0.00	0.00
79	->	48	-0.00	0.00
79	->	49	-0.00	0.00
79	->	50	-0.00	0.00

79	->	51	-0.00	0.00
79	->	52	-0.00	0.00
79	->	53	0.00	0.00
79	->	54	-0.00	0.00
79	->	55	0.00	0.00
79	->	56	0.00	0.00
79	->	57	-0.00	0.00
79	->	58	-0.00	0.00
79	->	59	-0.00	0.00
79	->	60	-0.00	0.00
79	->	61	-0.02	0.01
79	->	62	-0.08	0.02
79	->	63	2.99	0.76
79	->	64	-0.07	0.06
79	->	65	0.71	0.10
79	->	66	3.44	0.61
79	->	67	-0.07	0.04
79	->	68	-1.87	0.72
79	->	69	-0.02	0.02
79	->	70	-1.99	0.42
79	->	71	-0.00	0.00
79	->	72	-0.01	0.01
79	->	73	-0.05	0.03
79	->	74	-0.11	0.06
79	->	75	-3.28	0.75
79	->	76	-2.15	0.34
79	->	77	-0.91	0.18
79	->	78	-19.59	0.49
79	->	79	10.33	1.32
79	->	80	-4.87	0.45
79	->	81	-1.24	0.25
79	->	82	-3.59	0.74
79	->	83	17.15	8.39
79	->	84	-0.11	0.04
79	->	85	-0.04	0.02
79	->	86	-0.98	1.79
79	->	87	-0.06	0.03
79	->	88	-0.01	0.00
79	->	89	-0.01	0.00
79	->	90	-0.00	0.00
79	->	91	-0.00	0.00
79	->	92	-0.00	0.00
79	->	93	-0.00	0.00
79	->	94	-0.00	0.00
79	->	95	-0.00	0.00
79	->	96	-0.00	0.00
79	->	97	-0.00	0.00
79	->	98	-0.00	0.00
79	->	99	-0.00	0.00
79	->	100	-0.00	0.00

79	->	101	-0.01	0.00
79	->	102	-0.01	0.01
79	->	103	-0.01	0.01
79	->	104	-0.01	0.01
79	->	105	-0.01	0.00
79	->	106	-0.01	0.00
79	->	107	-0.00	0.00
80	->	1	0.00	0.00
80	->	2	0.00	0.00
80	->	3	-0.00	0.00
80	->	4	0.00	0.00
80	->	5	-0.00	0.00
80	->	6	0.00	0.00
80	->	7	0.00	0.00
80	->	8	-0.00	0.00
80	->	9	0.00	0.00
80	->	10	-0.00	0.00
80	->	11	-0.00	0.00
80	->	12	0.00	0.00
80	->	13	0.00	0.00
80	->	14	0.00	0.00
80	->	15	0.00	0.00
80	->	16	0.00	0.00
80	->	17	0.00	0.00
80	->	18	-0.00	0.00
80	->	19	-0.00	0.00
80	->	20	0.00	0.00
80	->	21	0.00	0.00
80	->	22	0.00	0.00
80	->	23	0.00	0.00
80	->	24	0.00	0.00
80	->	25	0.00	0.00
80	->	26	0.00	0.00
80	->	27	0.00	0.00
80	->	28	0.00	0.00
80	->	29	0.00	0.00
80	->	30	-0.00	0.00
80	->	31	0.00	0.00
80	->	32	0.00	0.00
80	->	33	0.00	0.00
80	->	34	0.00	0.00
80	->	35	-0.00	0.00
80	->	36	0.00	0.00
80	->	37	0.00	0.00
80	->	38	-0.00	0.00
80	->	39	0.00	0.00
80	->	40	0.00	0.00
80	->	41	0.00	0.00
80	->	42	0.00	0.00
80	->	43	0.00	0.00

80 ->	44	0.00	0.00
80 ->	45	-0.00	0.00
80 ->	46	-0.00	0.00
80 ->	47	-0.00	0.00
80 ->	48	0.00	0.00
80 ->	49	-0.00	0.00
80 ->	50	-0.00	0.00
80 ->	51	0.00	0.00
80 ->	52	0.00	0.00
80 ->	53	0.00	0.00
80 ->	54	0.00	0.00
80 ->	55	0.00	0.00
80 ->	56	0.00	0.00
80 ->	57	0.00	0.00
80 ->	58	-0.00	0.00
80 ->	59	-0.00	0.00
80 ->	60	-0.00	0.00
80 ->	61	-0.02	0.01
80 ->	62	-0.03	0.02
80 ->	63	0.23	0.16
80 ->	64	-0.20	0.36
80 ->	65	-0.09	0.07
80 ->	66	0.04	0.02
80 ->	67	-0.01	0.00
80 ->	68	-0.04	0.01
80 ->	69	-0.01	0.01
80 ->	70	-0.05	0.02
80 ->	71	-0.00	0.00
80 ->	72	-0.01	0.01
80 ->	73	-0.02	0.02
80 ->	74	-0.05	0.02
80 ->	75	-0.18	0.07
80 ->	76	-3.48	1.56
80 ->	77	-2.12	0.27
80 ->	78	-0.69	0.19
80 ->	79	-4.90	0.45
80 ->	80	-22.21	1.66
80 ->	81	-15.07	0.44
80 ->	82	-0.88	0.16
80 ->	83	-2.79	0.74
80 ->	84	-2.20	1.07
80 ->	85	-0.06	0.05
80 ->	86	-0.01	0.01
80 ->	87	-0.00	0.00
80 ->	88	-0.00	0.00
80 ->	89	-0.00	0.00
80 ->	90	-0.00	0.00
80 ->	91	-0.00	0.00
80 ->	92	-0.00	0.00
80 ->	93	-0.00	0.00

80	->	94	-0.00	0.00
80	->	95	-0.00	0.00
80	->	96	-0.00	0.00
80	->	97	-0.00	0.00
80	->	98	-0.00	0.00
80	->	99	-0.00	0.00
80	->	100	-0.00	0.00
80	->	101	-0.00	0.00
80	->	102	-0.01	0.01
80	->	103	-0.03	0.02
80	->	104	-0.02	0.01
80	->	105	-0.00	0.00
80	->	106	-0.00	0.00
80	->	107	-0.00	0.00
81	->	1	0.02	0.01
81	->	2	0.00	0.00
81	->	3	-0.00	0.00
81	->	4	0.00	0.00
81	->	5	-0.00	0.00
81	->	6	0.00	0.00
81	->	7	-0.00	0.00
81	->	8	-0.00	0.00
81	->	9	-0.00	0.00
81	->	10	-0.01	0.00
81	->	11	-0.01	0.00
81	->	12	0.02	0.01
81	->	13	0.00	0.00
81	->	14	0.00	0.00
81	->	15	0.00	0.00
81	->	16	0.01	0.00
81	->	17	-0.00	0.00
81	->	18	-0.01	0.00
81	->	19	-0.01	0.00
81	->	20	-0.00	0.00
81	->	21	0.00	0.00
81	->	22	0.01	0.00
81	->	23	0.00	0.00
81	->	24	0.00	0.00
81	->	25	0.01	0.00
81	->	26	0.00	0.00
81	->	27	0.00	0.00
81	->	28	0.00	0.00
81	->	29	0.00	0.00
81	->	30	-0.00	0.00
81	->	31	0.01	0.00
81	->	32	0.00	0.00
81	->	33	0.00	0.00
81	->	34	0.00	0.00
81	->	35	-0.01	0.00
81	->	36	0.00	0.00

81 ->	37	-0.00	0.00
81 ->	38	-0.01	0.00
81 ->	39	0.00	0.00
81 ->	40	0.01	0.00
81 ->	41	-0.00	0.00
81 ->	42	0.00	0.00
81 ->	43	0.00	0.00
81 ->	44	0.02	0.00
81 ->	45	-0.00	0.00
81 ->	46	-0.02	0.01
81 ->	47	-0.00	0.00
81 ->	48	-0.00	0.00
81 ->	49	0.00	0.00
81 ->	50	0.00	0.00
81 ->	51	0.00	0.00
81 ->	52	0.00	0.00
81 ->	53	0.02	0.00
81 ->	54	0.00	0.00
81 ->	55	0.00	0.00
81 ->	56	0.00	0.00
81 ->	57	0.00	0.00
81 ->	58	-0.01	0.00
81 ->	59	0.02	0.00
81 ->	60	-0.00	0.00
81 ->	61	-0.00	0.00
81 ->	62	-0.01	0.00
81 ->	63	-0.01	0.02
81 ->	64	-0.03	0.01
81 ->	65	-0.01	0.00
81 ->	66	-0.03	0.01
81 ->	67	-0.00	0.00
81 ->	68	0.02	0.01
81 ->	69	0.01	0.00
81 ->	70	-0.01	0.01
81 ->	71	0.00	0.00
81 ->	72	0.04	0.01
81 ->	73	-0.01	0.01
81 ->	74	-0.16	0.13
81 ->	75	-0.10	0.03
81 ->	76	0.01	0.06
81 ->	77	-1.51	0.58
81 ->	78	-2.06	0.59
81 ->	79	-1.25	0.25
81 ->	80	-15.09	0.43
81 ->	81	7.30	3.82
81 ->	82	-11.35	0.50
81 ->	83	-0.70	0.27
81 ->	84	-1.77	0.47
81 ->	85	-1.04	1.41
81 ->	86	-0.04	0.05

81	->	87	-0.04	0.01
81	->	88	-0.02	0.00
81	->	89	-0.02	0.01
81	->	90	-0.02	0.00
81	->	91	-0.01	0.00
81	->	92	-0.01	0.00
81	->	93	-0.02	0.01
81	->	94	-0.02	0.01
81	->	95	-0.01	0.00
81	->	96	-0.01	0.00
81	->	97	-0.00	0.00
81	->	98	-0.00	0.00
81	->	99	-0.01	0.00
81	->	100	-0.01	0.00
81	->	101	-0.03	0.01
81	->	102	-0.08	0.07
81	->	103	-0.64	0.71
81	->	104	-2.56	3.03
81	->	105	-0.17	0.16
81	->	106	-0.06	0.02
81	->	107	-0.01	0.00
82	->	1	0.00	0.00
82	->	2	0.00	0.00
82	->	3	0.00	0.00
82	->	4	0.00	0.00
82	->	5	-0.00	0.00
82	->	6	0.00	0.00
82	->	7	0.00	0.00
82	->	8	-0.00	0.00
82	->	9	-0.00	0.00
82	->	10	-0.00	0.00
82	->	11	-0.00	0.00
82	->	12	0.00	0.00
82	->	13	0.00	0.00
82	->	14	0.00	0.00
82	->	15	0.00	0.00
82	->	16	0.00	0.00
82	->	17	0.00	0.00
82	->	18	-0.00	0.00
82	->	19	-0.00	0.00
82	->	20	0.00	0.00
82	->	21	0.00	0.00
82	->	22	0.00	0.00
82	->	23	0.00	0.00
82	->	24	0.00	0.00
82	->	25	0.00	0.00
82	->	26	0.00	0.00
82	->	27	0.00	0.00
82	->	28	0.00	0.00
82	->	29	0.00	0.00

82 ->	30	-0.00	0.00
82 ->	31	0.00	0.00
82 ->	32	0.00	0.00
82 ->	33	0.00	0.00
82 ->	34	0.00	0.00
82 ->	35	-0.00	0.00
82 ->	36	0.00	0.00
82 ->	37	0.00	0.00
82 ->	38	-0.00	0.00
82 ->	39	0.00	0.00
82 ->	40	0.00	0.00
82 ->	41	-0.00	0.00
82 ->	42	0.00	0.00
82 ->	43	0.00	0.00
82 ->	44	0.00	0.00
82 ->	45	-0.00	0.00
82 ->	46	-0.00	0.00
82 ->	47	-0.00	0.00
82 ->	48	-0.00	0.00
82 ->	49	-0.00	0.00
82 ->	50	-0.00	0.00
82 ->	51	0.00	0.00
82 ->	52	-0.00	0.00
82 ->	53	0.00	0.00
82 ->	54	0.00	0.00
82 ->	55	0.00	0.00
82 ->	56	0.00	0.00
82 ->	57	0.00	0.00
82 ->	58	-0.00	0.00
82 ->	59	0.00	0.00
82 ->	60	-0.00	0.00
82 ->	61	-0.00	0.00
82 ->	62	-0.00	0.00
82 ->	63	-0.07	0.02
82 ->	64	-0.01	0.00
82 ->	65	-0.05	0.01
82 ->	66	-0.07	0.03
82 ->	67	-0.00	0.00
82 ->	68	-0.04	0.05
82 ->	69	-0.00	0.00
82 ->	70	-0.03	0.01
82 ->	71	-0.00	0.00
82 ->	72	-0.00	0.00
82 ->	73	-0.00	0.00
82 ->	74	-0.01	0.00
82 ->	75	-0.04	0.02
82 ->	76	-0.04	0.02
82 ->	77	-0.06	0.04
82 ->	78	-1.64	1.01
82 ->	79	-3.61	0.74

82	->	80	-0.89	0.16
82	->	81	-11.43	0.50
82	->	82	18.15	2.19
82	->	83	-21.94	0.56
82	->	84	-0.84	0.32
82	->	85	-2.18	1.85
82	->	86	-0.46	0.81
82	->	87	-0.02	0.01
82	->	88	-0.01	0.00
82	->	89	-0.00	0.00
82	->	90	-0.00	0.00
82	->	91	-0.00	0.00
82	->	92	-0.00	0.00
82	->	93	-0.00	0.00
82	->	94	-0.00	0.00
82	->	95	-0.00	0.00
82	->	96	-0.00	0.00
82	->	97	-0.00	0.00
82	->	98	-0.00	0.00
82	->	99	-0.00	0.00
82	->	100	-0.00	0.00
82	->	101	-0.00	0.00
82	->	102	-0.00	0.00
82	->	103	-0.00	0.00
82	->	104	-0.01	0.01
82	->	105	-0.01	0.01
82	->	106	-0.01	0.01
82	->	107	-0.00	0.00
83	->	1	0.00	0.00
83	->	2	0.00	0.00
83	->	3	0.00	0.00
83	->	4	0.00	0.00
83	->	5	-0.00	0.00
83	->	6	0.00	0.00
83	->	7	0.00	0.00
83	->	8	-0.00	0.00
83	->	9	0.00	0.00
83	->	10	-0.00	0.00
83	->	11	-0.00	0.00
83	->	12	0.00	0.00
83	->	13	0.00	0.00
83	->	14	0.00	0.00
83	->	15	0.00	0.00
83	->	16	0.00	0.00
83	->	17	0.00	0.00
83	->	18	-0.00	0.00
83	->	19	-0.00	0.00
83	->	20	0.00	0.00
83	->	21	0.00	0.00
83	->	22	0.00	0.00

83	->	23	0.00	0.00
83	->	24	0.00	0.00
83	->	25	0.00	0.00
83	->	26	0.00	0.00
83	->	27	0.00	0.00
83	->	28	0.00	0.00
83	->	29	0.00	0.00
83	->	30	0.00	0.00
83	->	31	0.00	0.00
83	->	32	0.00	0.00
83	->	33	0.00	0.00
83	->	34	0.00	0.00
83	->	35	0.00	0.00
83	->	36	-0.00	0.00
83	->	37	0.00	0.00
83	->	38	0.00	0.00
83	->	39	0.00	0.00
83	->	40	0.00	0.00
83	->	41	0.00	0.00
83	->	42	0.00	0.00
83	->	43	0.00	0.00
83	->	44	0.00	0.00
83	->	45	0.00	0.00
83	->	46	-0.00	0.00
83	->	47	0.00	0.00
83	->	48	0.00	0.00
83	->	49	0.00	0.00
83	->	50	0.00	0.00
83	->	51	0.00	0.00
83	->	52	0.00	0.00
83	->	53	-0.00	0.00
83	->	54	0.00	0.00
83	->	55	-0.00	0.00
83	->	56	0.00	0.00
83	->	57	0.00	0.00
83	->	58	0.00	0.00
83	->	59	-0.00	0.00
83	->	60	-0.00	0.00
83	->	61	-0.01	0.00
83	->	62	-0.04	0.01
83	->	63	2.40	0.32
83	->	64	-0.21	0.14
83	->	65	0.67	0.67
83	->	66	2.12	0.35
83	->	67	-0.02	0.03
83	->	68	-0.28	0.14
83	->	69	-0.01	0.01
83	->	70	-0.00	0.01
83	->	71	0.00	0.00
83	->	72	-0.00	0.00

83 ->	73	-0.00	0.00
83 ->	74	-0.01	0.00
83 ->	75	-0.02	0.01
83 ->	76	-0.07	0.02
83 ->	77	-0.04	0.01
83 ->	78	-0.13	0.05
83 ->	79	17.14	8.39
83 ->	80	-2.79	0.74
83 ->	81	-0.71	0.27
83 ->	82	-22.02	0.56
83 ->	83	4.66	1.56
83 ->	84	-8.26	0.44
83 ->	85	-0.89	0.49
83 ->	86	0.00	0.03
83 ->	87	-0.00	0.00
83 ->	88	-0.00	0.00
83 ->	89	-0.00	0.00
83 ->	90	-0.00	0.00
83 ->	91	-0.00	0.00
83 ->	92	-0.00	0.00
83 ->	93	-0.00	0.00
83 ->	94	-0.00	0.00
83 ->	95	-0.00	0.00
83 ->	96	-0.00	0.00
83 ->	97	0.00	0.00
83 ->	98	0.00	0.00
83 ->	99	0.00	0.00
83 ->	100	0.00	0.00
83 ->	101	-0.00	0.00
83 ->	102	-0.00	0.00
83 ->	103	-0.00	0.00
83 ->	104	-0.01	0.00
83 ->	105	-0.00	0.00
83 ->	106	-0.00	0.00
83 ->	107	-0.00	0.00
84 ->	1	0.00	0.00
84 ->	2	0.00	0.00
84 ->	3	0.00	0.00
84 ->	4	0.00	0.00
84 ->	5	-0.00	0.00
84 ->	6	0.00	0.00
84 ->	7	0.00	0.00
84 ->	8	0.00	0.00
84 ->	9	0.00	0.00
84 ->	10	-0.00	0.00
84 ->	11	-0.00	0.00
84 ->	12	0.00	0.00
84 ->	13	0.00	0.00
84 ->	14	0.00	0.00
84 ->	15	0.00	0.00

84	->	16	0.00	0.00
84	->	17	0.00	0.00
84	->	18	-0.00	0.00
84	->	19	-0.00	0.00
84	->	20	0.00	0.00
84	->	21	0.00	0.00
84	->	22	0.00	0.00
84	->	23	0.00	0.00
84	->	24	0.00	0.00
84	->	25	0.00	0.00
84	->	26	0.00	0.00
84	->	27	0.00	0.00
84	->	28	0.00	0.00
84	->	29	0.00	0.00
84	->	30	0.00	0.00
84	->	31	0.00	0.00
84	->	32	0.00	0.00
84	->	33	0.00	0.00
84	->	34	0.00	0.00
84	->	35	-0.00	0.00
84	->	36	0.00	0.00
84	->	37	0.00	0.00
84	->	38	-0.00	0.00
84	->	39	0.00	0.00
84	->	40	0.00	0.00
84	->	41	-0.00	0.00
84	->	42	0.00	0.00
84	->	43	0.00	0.00
84	->	44	0.00	0.00
84	->	45	0.00	0.00
84	->	46	-0.00	0.00
84	->	47	-0.00	0.00
84	->	48	0.00	0.00
84	->	49	0.00	0.00
84	->	50	0.00	0.00
84	->	51	0.00	0.00
84	->	52	0.00	0.00
84	->	53	0.00	0.00
84	->	54	0.00	0.00
84	->	55	0.00	0.00
84	->	56	0.00	0.00
84	->	57	0.00	0.00
84	->	58	-0.00	0.00
84	->	59	0.00	0.00
84	->	60	-0.00	0.00
84	->	61	-0.00	0.00
84	->	62	-0.00	0.00
84	->	63	-0.00	0.01
84	->	64	-0.03	0.04
84	->	65	-0.06	0.15

84	->	66	-0.00	0.00
84	->	67	-0.00	0.00
84	->	68	-0.00	0.00
84	->	69	-0.00	0.00
84	->	70	-0.00	0.00
84	->	71	0.00	0.00
84	->	72	-0.00	0.00
84	->	73	-0.00	0.00
84	->	74	-0.00	0.00
84	->	75	-0.00	0.00
84	->	76	-0.01	0.01
84	->	77	-0.04	0.03
84	->	78	-0.02	0.01
84	->	79	-0.11	0.04
84	->	80	-2.22	1.06
84	->	81	-1.79	0.47
84	->	82	-0.85	0.32
84	->	83	-8.40	0.44
84	->	84	16.46	1.74
84	->	85	-7.77	0.66
84	->	86	-0.00	0.00
84	->	87	-0.00	0.00
84	->	88	-0.00	0.00
84	->	89	-0.00	0.00
84	->	90	-0.00	0.00
84	->	91	-0.00	0.00
84	->	92	-0.00	0.00
84	->	93	-0.00	0.00
84	->	94	-0.00	0.00
84	->	95	-0.00	0.00
84	->	96	-0.00	0.00
84	->	97	0.00	0.00
84	->	98	-0.00	0.00
84	->	99	-0.00	0.00
84	->	100	-0.00	0.00
84	->	101	-0.00	0.00
84	->	102	-0.00	0.00
84	->	103	-0.01	0.00
84	->	104	-0.01	0.00
84	->	105	-0.00	0.00
84	->	106	-0.00	0.00
84	->	107	-0.00	0.00
85	->	1	0.00	0.00
85	->	2	-0.00	0.00
85	->	3	-0.00	0.00
85	->	4	0.00	0.00
85	->	5	-0.00	0.00
85	->	6	0.00	0.00
85	->	7	-0.00	0.00
85	->	8	-0.00	0.00

85	->	9	-0.00	0.00
85	->	10	-0.00	0.00
85	->	11	-0.00	0.00
85	->	12	0.00	0.00
85	->	13	0.00	0.00
85	->	14	0.00	0.00
85	->	15	0.00	0.00
85	->	16	0.00	0.00
85	->	17	0.00	0.00
85	->	18	-0.00	0.00
85	->	19	-0.00	0.00
85	->	20	0.00	0.00
85	->	21	0.00	0.00
85	->	22	0.00	0.00
85	->	23	0.00	0.00
85	->	24	0.00	0.00
85	->	25	0.00	0.00
85	->	26	0.00	0.00
85	->	27	0.00	0.00
85	->	28	0.00	0.00
85	->	29	0.00	0.00
85	->	30	-0.00	0.00
85	->	31	0.00	0.00
85	->	32	0.00	0.00
85	->	33	0.00	0.00
85	->	34	0.00	0.00
85	->	35	-0.00	0.00
85	->	36	0.00	0.00
85	->	37	-0.00	0.00
85	->	38	-0.00	0.00
85	->	39	0.00	0.00
85	->	40	0.00	0.00
85	->	41	-0.00	0.00
85	->	42	0.00	0.00
85	->	43	0.00	0.00
85	->	44	0.00	0.00
85	->	45	-0.00	0.00
85	->	46	-0.00	0.00
85	->	47	-0.00	0.00
85	->	48	-0.00	0.00
85	->	49	-0.00	0.00
85	->	50	0.00	0.00
85	->	51	0.00	0.00
85	->	52	-0.00	0.00
85	->	53	0.00	0.00
85	->	54	0.00	0.00
85	->	55	-0.00	0.00
85	->	56	0.00	0.00
85	->	57	0.00	0.00
85	->	58	-0.00	0.00

85	->	59	0.00	0.00
85	->	60	0.00	0.00
85	->	61	-0.00	0.00
85	->	62	-0.00	0.00
85	->	63	0.00	0.01
85	->	64	0.00	0.02
85	->	65	-0.04	0.17
85	->	66	-0.00	0.02
85	->	67	-0.00	0.00
85	->	68	0.00	0.01
85	->	69	-0.00	0.00
85	->	70	-0.00	0.00
85	->	71	0.00	0.00
85	->	72	0.00	0.01
85	->	73	-0.00	0.00
85	->	74	-0.01	0.01
85	->	75	-0.01	0.01
85	->	76	-0.01	0.01
85	->	77	-0.01	0.01
85	->	78	-0.02	0.02
85	->	79	-0.04	0.02
85	->	80	-0.06	0.05
85	->	81	-1.06	1.39
85	->	82	-2.19	1.85
85	->	83	-0.92	0.51
85	->	84	-7.84	0.65
85	->	85	-266.65	3.02
85	->	86	-0.02	0.05
85	->	87	-0.01	0.01
85	->	88	-0.00	0.00
85	->	89	-0.00	0.00
85	->	90	-0.00	0.00
85	->	91	-0.00	0.00
85	->	92	-0.00	0.00
85	->	93	-0.00	0.00
85	->	94	-0.00	0.00
85	->	95	-0.00	0.00
85	->	96	-0.00	0.00
85	->	97	-0.00	0.00
85	->	98	-0.00	0.00
85	->	99	-0.00	0.00
85	->	100	-0.00	0.00
85	->	101	-0.00	0.00
85	->	102	-0.00	0.01
85	->	103	-0.01	0.03
85	->	104	-0.04	0.16
85	->	105	-0.04	0.15
85	->	106	-0.01	0.02
85	->	107	-0.00	0.00
86	->	1	-0.00	0.00

86 ->	2	-0.00	0.00
86 ->	3	0.00	0.00
86 ->	4	0.00	0.00
86 ->	5	0.00	0.00
86 ->	6	0.00	0.00
86 ->	7	0.00	0.00
86 ->	8	0.00	0.00
86 ->	9	0.00	0.00
86 ->	10	0.00	0.00
86 ->	11	0.00	0.00
86 ->	12	-0.00	0.00
86 ->	13	-0.00	0.00
86 ->	14	-0.00	0.00
86 ->	15	-0.00	0.00
86 ->	16	-0.00	0.00
86 ->	17	0.00	0.00
86 ->	18	0.00	0.00
86 ->	19	0.00	0.00
86 ->	20	0.00	0.00
86 ->	21	0.00	0.00
86 ->	22	-0.00	0.00
86 ->	23	-0.00	0.00
86 ->	24	0.00	0.00
86 ->	25	-0.00	0.00
86 ->	26	0.00	0.00
86 ->	27	-0.00	0.00
86 ->	28	0.00	0.00
86 ->	29	-0.00	0.00
86 ->	30	0.00	0.00
86 ->	31	-0.00	0.00
86 ->	32	-0.00	0.00
86 ->	33	0.00	0.00
86 ->	34	-0.00	0.00
86 ->	35	0.00	0.00
86 ->	36	-0.00	0.00
86 ->	37	-0.00	0.00
86 ->	38	0.00	0.00
86 ->	39	-0.00	0.00
86 ->	40	-0.01	0.00
86 ->	41	0.00	0.00
86 ->	42	-0.00	0.00
86 ->	43	-0.00	0.00
86 ->	44	-0.00	0.00
86 ->	45	0.00	0.00
86 ->	46	0.00	0.00
86 ->	47	-0.00	0.00
86 ->	48	-0.00	0.00
86 ->	49	-0.00	0.00
86 ->	50	-0.00	0.00
86 ->	51	-0.00	0.00

86	->	52	-0.00	0.00
86	->	53	-0.02	0.01
86	->	54	-0.01	0.01
86	->	55	-0.00	0.00
86	->	56	-0.00	0.00
86	->	57	-0.00	0.00
86	->	58	0.00	0.00
86	->	59	-0.20	0.30
86	->	60	-0.00	0.00
86	->	61	-0.00	0.00
86	->	62	-0.01	0.01
86	->	63	0.03	0.03
86	->	64	0.01	0.00
86	->	65	-0.00	0.00
86	->	66	0.04	0.08
86	->	67	-0.01	0.01
86	->	68	-2.34	2.14
86	->	69	-0.05	0.04
86	->	70	-0.25	0.39
86	->	71	-0.00	0.01
86	->	72	-0.03	0.02
86	->	73	-0.00	0.00
86	->	74	-0.00	0.01
86	->	75	-0.07	0.13
86	->	76	-0.03	0.02
86	->	77	-0.03	0.03
86	->	78	-2.05	2.28
86	->	79	-0.97	1.78
86	->	80	-0.01	0.01
86	->	81	-0.04	0.05
86	->	82	-0.45	0.80
86	->	83	0.00	0.03
86	->	84	-0.00	0.00
86	->	85	-0.02	0.05
86	->	86	-85.30	4.13
86	->	87	-34.44	2.44
86	->	88	-0.31	0.16
86	->	89	-0.03	0.01
86	->	90	-0.00	0.00
86	->	91	0.00	0.00
86	->	92	0.00	0.00
86	->	93	0.00	0.00
86	->	94	0.00	0.00
86	->	95	0.00	0.00
86	->	96	0.00	0.00
86	->	97	0.00	0.00
86	->	98	0.00	0.00
86	->	99	0.00	0.00
86	->	100	0.00	0.00
86	->	101	0.00	0.00

86	->	102	0.00	0.00
86	->	103	0.00	0.00
86	->	104	-0.01	0.01
86	->	105	-0.04	0.03
86	->	106	-0.27	0.17
86	->	107	-1.60	1.29
87	->	1	-0.01	0.00
87	->	2	-0.00	0.00
87	->	3	0.00	0.00
87	->	4	-0.00	0.00
87	->	5	0.00	0.00
87	->	6	-0.00	0.00
87	->	7	0.00	0.00
87	->	8	0.00	0.00
87	->	9	0.00	0.00
87	->	10	0.00	0.00
87	->	11	0.00	0.00
87	->	12	-0.01	0.00
87	->	13	-0.00	0.00
87	->	14	-0.00	0.00
87	->	15	-0.00	0.00
87	->	16	-0.00	0.00
87	->	17	0.00	0.00
87	->	18	0.01	0.00
87	->	19	0.01	0.00
87	->	20	0.00	0.00
87	->	21	-0.00	0.00
87	->	22	-0.01	0.00
87	->	23	-0.00	0.00
87	->	24	0.00	0.00
87	->	25	-0.01	0.00
87	->	26	-0.00	0.00
87	->	27	-0.00	0.00
87	->	28	-0.00	0.00
87	->	29	-0.00	0.00
87	->	30	0.00	0.00
87	->	31	-0.01	0.00
87	->	32	-0.00	0.00
87	->	33	-0.00	0.00
87	->	34	-0.00	0.00
87	->	35	0.02	0.00
87	->	36	-0.01	0.00
87	->	37	-0.00	0.00
87	->	38	0.03	0.00
87	->	39	-0.00	0.00
87	->	40	-0.04	0.02
87	->	41	-0.00	0.00
87	->	42	-0.01	0.00
87	->	43	-0.00	0.00
87	->	44	-0.02	0.00

87	->	45	0.00	0.00
87	->	46	0.02	0.00
87	->	47	-0.01	0.00
87	->	48	-0.00	0.00
87	->	49	-0.01	0.01
87	->	50	-0.03	0.02
87	->	51	-0.03	0.02
87	->	52	-0.00	0.00
87	->	53	-0.13	0.06
87	->	54	-0.27	0.19
87	->	55	0.01	0.01
87	->	56	-0.01	0.00
87	->	57	-0.02	0.01
87	->	58	0.03	0.01
87	->	59	-1.81	2.25
87	->	60	-0.01	0.00
87	->	61	-0.00	0.00
87	->	62	-0.01	0.01
87	->	63	0.04	0.01
87	->	64	0.02	0.00
87	->	65	0.00	0.00
87	->	66	0.06	0.02
87	->	67	-0.01	0.01
87	->	68	-3.33	2.85
87	->	69	-0.14	0.08
87	->	70	-0.31	0.32
87	->	71	-0.04	0.03
87	->	72	-0.22	0.28
87	->	73	-0.00	0.00
87	->	74	0.02	0.02
87	->	75	-0.25	0.53
87	->	76	-0.04	0.01
87	->	77	-0.04	0.01
87	->	78	-5.34	3.25
87	->	79	-0.06	0.03
87	->	80	-0.00	0.00
87	->	81	-0.04	0.01
87	->	82	-0.02	0.01
87	->	83	-0.00	0.00
87	->	84	-0.00	0.00
87	->	85	-0.01	0.01
87	->	86	-34.28	2.42
87	->	87	-174.90	4.22
87	->	88	-35.49	1.23
87	->	89	-0.30	0.12
87	->	90	-0.02	0.01
87	->	91	0.01	0.00
87	->	92	0.01	0.00
87	->	93	0.01	0.00
87	->	94	0.01	0.00

87	->	95	0.00	0.00
87	->	96	0.00	0.00
87	->	97	0.00	0.00
87	->	98	0.00	0.00
87	->	99	0.01	0.00
87	->	100	0.01	0.00
87	->	101	0.02	0.00
87	->	102	0.01	0.00
87	->	103	0.01	0.00
87	->	104	-0.01	0.01
87	->	105	-0.11	0.06
87	->	106	-2.01	1.76
87	->	107	-8.50	4.08
88	->	1	-0.01	0.00
88	->	2	-0.00	0.00
88	->	3	0.00	0.00
88	->	4	-0.00	0.00
88	->	5	0.00	0.00
88	->	6	-0.00	0.00
88	->	7	0.00	0.00
88	->	8	0.00	0.00
88	->	9	0.00	0.00
88	->	10	0.01	0.00
88	->	11	0.01	0.00
88	->	12	-0.01	0.00
88	->	13	-0.00	0.00
88	->	14	-0.00	0.00
88	->	15	-0.00	0.00
88	->	16	-0.01	0.00
88	->	17	0.00	0.00
88	->	18	0.01	0.00
88	->	19	0.01	0.00
88	->	20	0.00	0.00
88	->	21	-0.00	0.00
88	->	22	-0.02	0.00
88	->	23	-0.00	0.00
88	->	24	0.00	0.00
88	->	25	-0.02	0.00
88	->	26	-0.00	0.00
88	->	27	-0.00	0.00
88	->	28	0.00	0.00
88	->	29	-0.00	0.00
88	->	30	0.00	0.00
88	->	31	-0.05	0.01
88	->	32	-0.00	0.00
88	->	33	-0.00	0.00
88	->	34	-0.01	0.01
88	->	35	0.11	0.04
88	->	36	-0.03	0.01
88	->	37	0.00	0.00

88	->	38	0.13	0.04
88	->	39	-0.01	0.00
88	->	40	-0.88	0.78
88	->	41	-0.02	0.01
88	->	42	0.02	0.04
88	->	43	0.00	0.01
88	->	44	-0.05	0.01
88	->	45	-0.00	0.01
88	->	46	0.02	0.02
88	->	47	-0.04	0.06
88	->	48	0.03	0.04
88	->	49	-0.07	0.04
88	->	50	-0.57	0.47
88	->	51	-1.24	0.52
88	->	52	0.02	0.02
88	->	53	-0.45	0.27
88	->	54	-2.95	1.57
88	->	55	0.07	0.05
88	->	56	-0.02	0.02
88	->	57	-0.04	0.03
88	->	58	0.03	0.02
88	->	59	-2.13	2.98
88	->	60	-0.01	0.00
88	->	61	-0.01	0.00
88	->	62	-0.00	0.00
88	->	63	0.01	0.00
88	->	64	0.01	0.00
88	->	65	0.00	0.00
88	->	66	0.02	0.00
88	->	67	-0.00	0.00
88	->	68	-0.08	0.03
88	->	69	-0.06	0.03
88	->	70	-0.09	0.05
88	->	71	-0.13	0.13
88	->	72	-1.29	1.31
88	->	73	-0.01	0.01
88	->	74	-0.22	0.24
88	->	75	-1.47	1.73
88	->	76	-0.03	0.01
88	->	77	-0.01	0.00
88	->	78	-0.66	0.73
88	->	79	-0.01	0.00
88	->	80	-0.00	0.00
88	->	81	-0.02	0.00
88	->	82	-0.01	0.00
88	->	83	-0.00	0.00
88	->	84	-0.00	0.00
88	->	85	-0.00	0.00
88	->	86	-0.31	0.16
88	->	87	-35.32	1.23

88	->	88	-207.52	3.42
88	->	89	-32.97	1.49
88	->	90	-0.22	0.07
88	->	91	0.01	0.01
88	->	92	0.01	0.00
88	->	93	0.01	0.00
88	->	94	0.00	0.00
88	->	95	0.00	0.00
88	->	96	0.00	0.00
88	->	97	0.00	0.00
88	->	98	0.01	0.00
88	->	99	0.01	0.00
88	->	100	0.02	0.01
88	->	101	0.01	0.01
88	->	102	-0.00	0.00
88	->	103	-0.01	0.01
88	->	104	-0.06	0.04
88	->	105	-0.32	0.25
88	->	106	-11.25	1.64
88	->	107	0.12	0.13
89	->	1	-0.01	0.00
89	->	2	-0.00	0.00
89	->	3	0.00	0.00
89	->	4	-0.00	0.00
89	->	5	0.01	0.00
89	->	6	-0.00	0.00
89	->	7	0.00	0.00
89	->	8	0.00	0.00
89	->	9	0.00	0.00
89	->	10	0.01	0.00
89	->	11	0.00	0.00
89	->	12	-0.01	0.00
89	->	13	-0.00	0.00
89	->	14	-0.00	0.00
89	->	15	-0.00	0.00
89	->	16	-0.01	0.00
89	->	17	0.00	0.00
89	->	18	0.02	0.00
89	->	19	0.01	0.00
89	->	20	-0.00	0.00
89	->	21	-0.00	0.00
89	->	22	-0.03	0.01
89	->	23	-0.00	0.00
89	->	24	-0.00	0.00
89	->	25	-0.03	0.01
89	->	26	-0.01	0.00
89	->	27	-0.00	0.00
89	->	28	-0.00	0.00
89	->	29	-0.00	0.00
89	->	30	-0.00	0.00

89	->	31	-0.36	0.38
89	->	32	-0.00	0.00
89	->	33	-0.01	0.01
89	->	34	-0.03	0.01
89	->	35	0.19	0.06
89	->	36	-0.04	0.01
89	->	37	0.01	0.01
89	->	38	0.29	0.11
89	->	39	-0.02	0.01
89	->	40	-7.19	3.19
89	->	41	-0.06	0.05
89	->	42	-1.28	0.49
89	->	43	0.01	0.02
89	->	44	-0.07	0.03
89	->	45	-0.01	0.01
89	->	46	-0.08	0.09
89	->	47	-1.95	1.10
89	->	48	-0.09	0.09
89	->	49	-0.10	0.05
89	->	50	-1.95	1.27
89	->	51	-6.91	1.36
89	->	52	0.08	0.06
89	->	53	-0.24	0.17
89	->	54	-0.66	1.23
89	->	55	0.08	0.07
89	->	56	-0.01	0.01
89	->	57	-0.01	0.00
89	->	58	0.03	0.01
89	->	59	-0.12	0.09
89	->	60	-0.01	0.00
89	->	61	-0.01	0.00
89	->	62	-0.00	0.00
89	->	63	0.01	0.00
89	->	64	0.01	0.00
89	->	65	0.00	0.00
89	->	66	0.01	0.00
89	->	67	0.00	0.00
89	->	68	-0.03	0.01
89	->	69	-0.02	0.01
89	->	70	-0.03	0.02
89	->	71	-0.06	0.03
89	->	72	-5.04	3.26
89	->	73	-0.01	0.01
89	->	74	0.43	0.28
89	->	75	0.06	0.18
89	->	76	-0.02	0.00
89	->	77	-0.03	0.01
89	->	78	-0.26	0.45
89	->	79	-0.01	0.00
89	->	80	-0.00	0.00

89	->	81	-0.02	0.01
89	->	82	-0.00	0.00
89	->	83	-0.00	0.00
89	->	84	-0.00	0.00
89	->	85	-0.00	0.00
89	->	86	-0.03	0.01
89	->	87	-0.30	0.12
89	->	88	-32.79	1.48
89	->	89	-162.95	3.78
89	->	90	-33.73	0.85
89	->	91	-0.22	0.07
89	->	92	-0.02	0.01
89	->	93	0.01	0.00
89	->	94	0.00	0.00
89	->	95	0.01	0.00
89	->	96	0.00	0.00
89	->	97	0.00	0.00
89	->	98	0.01	0.00
89	->	99	0.02	0.00
89	->	100	0.02	0.01
89	->	101	0.01	0.00
89	->	102	-0.01	0.01
89	->	103	-0.13	0.04
89	->	104	-1.77	0.65
89	->	105	-11.43	0.78
89	->	106	-4.15	0.99
89	->	107	-0.16	0.23
90	->	1	-0.03	0.00
90	->	2	-0.00	0.00
90	->	3	-0.00	0.00
90	->	4	-0.00	0.00
90	->	5	0.02	0.00
90	->	6	-0.00	0.00
90	->	7	-0.00	0.00
90	->	8	0.01	0.00
90	->	9	-0.00	0.00
90	->	10	0.02	0.00
90	->	11	0.02	0.00
90	->	12	-0.03	0.01
90	->	13	-0.01	0.00
90	->	14	-0.00	0.00
90	->	15	-0.00	0.00
90	->	16	-0.02	0.00
90	->	17	0.00	0.00
90	->	18	0.03	0.01
90	->	19	0.02	0.00
90	->	20	-0.00	0.00
90	->	21	-0.01	0.01
90	->	22	-0.15	0.06
90	->	23	-0.01	0.01

90 ->	24	-0.00	0.01
90 ->	25	-0.16	0.05
90 ->	26	-0.12	0.06
90 ->	27	0.01	0.01
90 ->	28	-0.01	0.00
90 ->	29	-0.02	0.01
90 ->	30	-0.01	0.01
90 ->	31	-2.07	2.18
90 ->	32	-0.01	0.00
90 ->	33	-0.01	0.01
90 ->	34	-0.02	0.01
90 ->	35	0.05	0.01
90 ->	36	-0.02	0.00
90 ->	37	0.00	0.00
90 ->	38	0.05	0.01
90 ->	39	-0.01	0.00
90 ->	40	-4.33	2.81
90 ->	41	-0.13	0.09
90 ->	42	-1.23	0.33
90 ->	43	-0.61	0.47
90 ->	44	-0.49	0.14
90 ->	45	-0.01	0.01
90 ->	46	-0.32	0.22
90 ->	47	-4.78	0.73
90 ->	48	0.01	0.05
90 ->	49	-0.04	0.02
90 ->	50	-0.48	0.21
90 ->	51	-0.07	0.02
90 ->	52	-0.01	0.00
90 ->	53	-0.05	0.01
90 ->	54	-0.01	0.00
90 ->	55	-0.00	0.00
90 ->	56	-0.00	0.00
90 ->	57	-0.00	0.00
90 ->	58	0.01	0.00
90 ->	59	-0.03	0.01
90 ->	60	-0.00	0.00
90 ->	61	-0.00	0.00
90 ->	62	0.00	0.00
90 ->	63	0.01	0.00
90 ->	64	0.00	0.00
90 ->	65	0.00	0.00
90 ->	66	0.01	0.00
90 ->	67	0.00	0.00
90 ->	68	-0.01	0.00
90 ->	69	-0.01	0.00
90 ->	70	-0.01	0.00
90 ->	71	-0.02	0.01
90 ->	72	-4.67	1.96
90 ->	73	-0.01	0.01

90	->	74	0.30	0.19
90	->	75	0.03	0.05
90	->	76	-0.02	0.00
90	->	77	-0.03	0.01
90	->	78	-0.09	0.11
90	->	79	-0.00	0.00
90	->	80	-0.00	0.00
90	->	81	-0.02	0.00
90	->	82	-0.00	0.00
90	->	83	-0.00	0.00
90	->	84	-0.00	0.00
90	->	85	-0.00	0.00
90	->	86	-0.00	0.00
90	->	87	-0.02	0.01
90	->	88	-0.22	0.07
90	->	89	-33.62	0.82
90	->	90	-82.36	3.05
90	->	91	-32.95	0.79
90	->	92	-0.23	0.06
90	->	93	-0.01	0.01
90	->	94	0.00	0.00
90	->	95	0.01	0.00
90	->	96	0.00	0.00
90	->	97	0.00	0.00
90	->	98	0.01	0.00
90	->	99	0.01	0.00
90	->	100	0.01	0.01
90	->	101	-0.02	0.01
90	->	102	-0.10	0.05
90	->	103	-1.09	0.36
90	->	104	-6.28	0.50
90	->	105	-1.22	0.67
90	->	106	-0.32	0.16
90	->	107	-0.22	0.48
91	->	1	-0.08	0.02
91	->	2	-0.00	0.00
91	->	3	-0.00	0.00
91	->	4	-0.01	0.00
91	->	5	0.05	0.01
91	->	6	-0.00	0.00
91	->	7	-0.01	0.01
91	->	8	0.01	0.00
91	->	9	-0.00	0.00
91	->	10	0.06	0.03
91	->	11	0.03	0.02
91	->	12	-0.28	0.22
91	->	13	-0.03	0.01
91	->	14	-0.01	0.02
91	->	15	-0.00	0.00
91	->	16	-0.03	0.01

91 ->	17	-0.00	0.00
91 ->	18	0.04	0.01
91 ->	19	0.00	0.02
91 ->	20	-0.00	0.01
91 ->	21	-0.04	0.02
91 ->	22	-1.28	0.84
91 ->	23	-0.68	0.46
91 ->	24	0.00	0.02
91 ->	25	-0.52	0.17
91 ->	26	-2.65	0.54
91 ->	27	0.04	0.04
91 ->	28	-0.02	0.01
91 ->	29	-0.04	0.02
91 ->	30	0.01	0.01
91 ->	31	-0.52	1.08
91 ->	32	-0.01	0.00
91 ->	33	-0.01	0.00
91 ->	34	-0.00	0.00
91 ->	35	0.03	0.00
91 ->	36	-0.02	0.00
91 ->	37	0.00	0.00
91 ->	38	0.03	0.00
91 ->	39	-0.00	0.00
91 ->	40	-0.09	0.03
91 ->	41	-0.04	0.02
91 ->	42	-0.31	0.10
91 ->	43	-3.69	1.57
91 ->	44	-5.92	2.30
91 ->	45	0.01	0.02
91 ->	46	0.09	0.20
91 ->	47	-4.61	0.91
91 ->	48	0.01	0.01
91 ->	49	-0.02	0.01
91 ->	50	-0.01	0.01
91 ->	51	-0.01	0.00
91 ->	52	-0.00	0.00
91 ->	53	-0.03	0.00
91 ->	54	-0.00	0.00
91 ->	55	-0.00	0.00
91 ->	56	-0.00	0.00
91 ->	57	-0.00	0.00
91 ->	58	0.01	0.00
91 ->	59	-0.02	0.01
91 ->	60	-0.00	0.00
91 ->	61	-0.00	0.00
91 ->	62	0.00	0.00
91 ->	63	0.01	0.00
91 ->	64	0.00	0.00
91 ->	65	0.00	0.00
91 ->	66	0.01	0.00

91	->	67	0.00	0.00
91	->	68	-0.01	0.00
91	->	69	-0.01	0.00
91	->	70	-0.00	0.00
91	->	71	-0.00	0.00
91	->	72	-0.83	0.95
91	->	73	0.00	0.00
91	->	74	-0.05	0.04
91	->	75	0.01	0.01
91	->	76	-0.01	0.00
91	->	77	-0.01	0.01
91	->	78	-0.02	0.02
91	->	79	-0.00	0.00
91	->	80	-0.00	0.00
91	->	81	-0.01	0.00
91	->	82	-0.00	0.00
91	->	83	-0.00	0.00
91	->	84	-0.00	0.00
91	->	85	-0.00	0.00
91	->	86	0.00	0.00
91	->	87	0.01	0.00
91	->	88	0.01	0.01
91	->	89	-0.22	0.07
91	->	90	-32.79	0.79
91	->	91	-164.95	3.92
91	->	92	-33.61	0.81
91	->	93	-0.32	0.08
91	->	94	-0.04	0.02
91	->	95	0.01	0.00
91	->	96	0.00	0.00
91	->	97	0.00	0.00
91	->	98	0.02	0.01
91	->	99	0.02	0.00
91	->	100	0.01	0.01
91	->	101	-0.12	0.06
91	->	102	-1.12	0.45
91	->	103	-11.82	0.77
91	->	104	-3.02	0.54
91	->	105	-0.34	0.09
91	->	106	-0.04	0.02
91	->	107	-0.03	0.10
92	->	1	-2.20	0.62
92	->	2	-0.00	0.00
92	->	3	-0.00	0.01
92	->	4	-0.02	0.01
92	->	5	0.10	0.04
92	->	6	-0.01	0.00
92	->	7	-0.01	0.01
92	->	8	0.03	0.00
92	->	9	-0.00	0.00

92 ->	10	0.11	0.06
92 ->	11	0.02	0.07
92 ->	12	-6.41	1.95
92 ->	13	-0.12	0.06
92 ->	14	-0.79	0.64
92 ->	15	0.00	0.02
92 ->	16	-0.21	0.07
92 ->	17	-0.00	0.00
92 ->	18	0.06	0.09
92 ->	19	0.09	0.16
92 ->	20	0.03	0.04
92 ->	21	-0.08	0.06
92 ->	22	-5.37	2.94
92 ->	23	-4.85	3.05
92 ->	24	0.08	0.06
92 ->	25	-0.19	0.07
92 ->	26	-0.87	0.29
92 ->	27	0.05	0.07
92 ->	28	-0.01	0.00
92 ->	29	-0.01	0.00
92 ->	30	-0.00	0.00
92 ->	31	-0.05	0.01
92 ->	32	-0.01	0.00
92 ->	33	-0.00	0.00
92 ->	34	-0.00	0.00
92 ->	35	0.01	0.00
92 ->	36	-0.01	0.00
92 ->	37	0.00	0.00
92 ->	38	0.01	0.00
92 ->	39	0.00	0.00
92 ->	40	-0.03	0.00
92 ->	41	-0.00	0.00
92 ->	42	-0.02	0.01
92 ->	43	-0.04	0.02
92 ->	44	-8.40	1.45
92 ->	45	-0.02	0.02
92 ->	46	0.46	0.28
92 ->	47	-0.18	0.29
92 ->	48	-0.00	0.00
92 ->	49	-0.01	0.01
92 ->	50	-0.01	0.00
92 ->	51	-0.01	0.00
92 ->	52	-0.00	0.00
92 ->	53	-0.02	0.00
92 ->	54	-0.00	0.00
92 ->	55	-0.00	0.00
92 ->	56	-0.00	0.00
92 ->	57	-0.00	0.00
92 ->	58	0.01	0.00
92 ->	59	-0.01	0.00

92 ->	60	-0.00	0.00
92 ->	61	-0.00	0.00
92 ->	62	0.00	0.00
92 ->	63	0.00	0.00
92 ->	64	0.00	0.00
92 ->	65	0.00	0.00
92 ->	66	0.00	0.00
92 ->	67	0.00	0.00
92 ->	68	-0.01	0.00
92 ->	69	-0.00	0.00
92 ->	70	-0.00	0.00
92 ->	71	0.00	0.00
92 ->	72	-0.15	0.20
92 ->	73	0.00	0.01
92 ->	74	-0.05	0.04
92 ->	75	0.00	0.00
92 ->	76	-0.01	0.00
92 ->	77	-0.00	0.02
92 ->	78	-0.02	0.02
92 ->	79	-0.00	0.00
92 ->	80	-0.00	0.00
92 ->	81	-0.01	0.00
92 ->	82	-0.00	0.00
92 ->	83	-0.00	0.00
92 ->	84	-0.00	0.00
92 ->	85	-0.00	0.00
92 ->	86	0.00	0.00
92 ->	87	0.01	0.00
92 ->	88	0.01	0.00
92 ->	89	-0.02	0.01
92 ->	90	-0.23	0.06
92 ->	91	-33.47	0.82
92 ->	92	-163.05	4.87
92 ->	93	-32.26	0.91
92 ->	94	-0.29	0.08
92 ->	95	-0.01	0.01
92 ->	96	0.00	0.00
92 ->	97	0.00	0.00
92 ->	98	0.01	0.00
92 ->	99	0.00	0.01
92 ->	100	-0.04	0.05
92 ->	101	-1.00	0.38
92 ->	102	-11.82	0.91
92 ->	103	-3.64	1.13
92 ->	104	-0.29	0.10
92 ->	105	-0.03	0.01
92 ->	106	0.01	0.00
92 ->	107	0.01	0.01
93 ->	1	-14.49	2.63
93 ->	2	-0.01	0.01

93	->	3	0.02	0.03
93	->	4	-0.01	0.00
93	->	5	0.05	0.01
93	->	6	-0.00	0.00
93	->	7	-0.00	0.00
93	->	8	0.03	0.01
93	->	9	0.00	0.00
93	->	10	0.04	0.01
93	->	11	0.03	0.02
93	->	12	-6.00	2.26
93	->	13	0.24	0.54
93	->	14	-0.48	0.36
93	->	15	-0.05	0.12
93	->	16	-2.22	0.72
93	->	17	-0.01	0.01
93	->	18	0.19	0.27
93	->	19	-0.41	0.98
93	->	20	0.03	0.02
93	->	21	-0.03	0.01
93	->	22	-6.13	1.99
93	->	23	-0.06	0.02
93	->	24	-0.00	0.00
93	->	25	-0.06	0.01
93	->	26	-0.02	0.00
93	->	27	-0.00	0.00
93	->	28	-0.00	0.00
93	->	29	-0.00	0.00
93	->	30	0.00	0.00
93	->	31	-0.02	0.00
93	->	32	-0.00	0.00
93	->	33	-0.00	0.00
93	->	34	-0.00	0.00
93	->	35	0.01	0.00
93	->	36	-0.01	0.00
93	->	37	0.00	0.00
93	->	38	0.01	0.00
93	->	39	0.00	0.00
93	->	40	-0.01	0.00
93	->	41	-0.00	0.00
93	->	42	-0.00	0.00
93	->	43	-0.01	0.00
93	->	44	-2.92	1.46
93	->	45	-0.01	0.01
93	->	46	0.07	0.17
93	->	47	-0.03	0.02
93	->	48	-0.00	0.00
93	->	49	-0.00	0.00
93	->	50	-0.01	0.00
93	->	51	-0.00	0.00
93	->	52	-0.00	0.00

93	->	53	-0.01	0.00
93	->	54	-0.00	0.00
93	->	55	-0.00	0.00
93	->	56	-0.00	0.00
93	->	57	-0.00	0.00
93	->	58	0.00	0.00
93	->	59	-0.01	0.00
93	->	60	-0.00	0.00
93	->	61	-0.01	0.00
93	->	62	0.00	0.00
93	->	63	0.00	0.00
93	->	64	0.00	0.00
93	->	65	0.00	0.00
93	->	66	0.00	0.00
93	->	67	-0.00	0.00
93	->	68	-0.00	0.00
93	->	69	-0.00	0.00
93	->	70	-0.00	0.00
93	->	71	0.00	0.00
93	->	72	0.01	0.03
93	->	73	-0.02	0.02
93	->	74	-0.02	0.01
93	->	75	0.00	0.00
93	->	76	-0.01	0.01
93	->	77	-0.02	0.02
93	->	78	-0.02	0.01
93	->	79	-0.00	0.00
93	->	80	-0.00	0.00
93	->	81	-0.02	0.01
93	->	82	-0.00	0.00
93	->	83	-0.00	0.00
93	->	84	-0.00	0.00
93	->	85	-0.00	0.00
93	->	86	0.00	0.00
93	->	87	0.01	0.00
93	->	88	0.01	0.00
93	->	89	0.01	0.00
93	->	90	-0.01	0.01
93	->	91	-0.32	0.08
93	->	92	-32.16	0.91
93	->	93	-159.66	3.94
93	->	94	-34.61	0.78
93	->	95	-0.38	0.07
93	->	96	-0.03	0.01
93	->	97	-0.00	0.00
93	->	98	0.01	0.01
93	->	99	-0.02	0.02
93	->	100	-0.50	0.57
93	->	101	-11.53	0.88
93	->	102	-3.75	0.65

93	->	103	-0.35	0.11
93	->	104	-0.03	0.02
93	->	105	0.01	0.00
93	->	106	0.02	0.00
93	->	107	0.01	0.00
94	->	1	-6.02	4.17
94	->	2	-0.01	0.01
94	->	3	-0.01	0.01
94	->	4	-0.00	0.00
94	->	5	0.02	0.00
94	->	6	-0.00	0.00
94	->	7	-0.00	0.00
94	->	8	0.01	0.00
94	->	9	0.00	0.00
94	->	10	0.02	0.00
94	->	11	0.03	0.00
94	->	12	-0.11	0.03
94	->	13	-0.09	0.03
94	->	14	-0.25	0.11
94	->	15	-0.45	1.04
94	->	16	-11.50	2.01
94	->	17	-0.01	0.01
94	->	18	-3.01	2.39
94	->	19	0.28	0.68
94	->	20	-0.01	0.01
94	->	21	-0.00	0.01
94	->	22	-0.13	0.57
94	->	23	-0.01	0.00
94	->	24	-0.00	0.00
94	->	25	-0.02	0.00
94	->	26	-0.00	0.00
94	->	27	-0.00	0.00
94	->	28	-0.00	0.00
94	->	29	-0.00	0.00
94	->	30	-0.00	0.00
94	->	31	-0.01	0.00
94	->	32	-0.00	0.00
94	->	33	-0.00	0.00
94	->	34	0.00	0.00
94	->	35	0.00	0.00
94	->	36	-0.00	0.00
94	->	37	0.00	0.00
94	->	38	0.00	0.00
94	->	39	-0.00	0.00
94	->	40	-0.01	0.00
94	->	41	-0.00	0.00
94	->	42	-0.00	0.00
94	->	43	-0.00	0.00
94	->	44	-0.11	0.11
94	->	45	-0.01	0.01

94 ->	46	0.01	0.03
94 ->	47	-0.01	0.00
94 ->	48	-0.00	0.00
94 ->	49	-0.00	0.00
94 ->	50	-0.00	0.00
94 ->	51	-0.00	0.00
94 ->	52	-0.00	0.00
94 ->	53	-0.02	0.00
94 ->	54	-0.00	0.00
94 ->	55	-0.00	0.00
94 ->	56	-0.00	0.00
94 ->	57	-0.00	0.00
94 ->	58	0.01	0.00
94 ->	59	-0.01	0.00
94 ->	60	-0.00	0.00
94 ->	61	-0.00	0.00
94 ->	62	0.00	0.00
94 ->	63	0.01	0.00
94 ->	64	0.01	0.00
94 ->	65	0.00	0.00
94 ->	66	0.00	0.00
94 ->	67	0.00	0.00
94 ->	68	-0.00	0.00
94 ->	69	-0.01	0.00
94 ->	70	-0.00	0.00
94 ->	71	-0.00	0.00
94 ->	72	-0.04	0.01
94 ->	73	-0.01	0.00
94 ->	74	0.02	0.00
94 ->	75	0.01	0.00
94 ->	76	-0.02	0.01
94 ->	77	-0.04	0.01
94 ->	78	-0.02	0.01
94 ->	79	-0.00	0.00
94 ->	80	-0.00	0.00
94 ->	81	-0.02	0.01
94 ->	82	-0.00	0.00
94 ->	83	-0.00	0.00
94 ->	84	-0.00	0.00
94 ->	85	-0.00	0.00
94 ->	86	0.00	0.00
94 ->	87	0.01	0.00
94 ->	88	0.00	0.00
94 ->	89	0.00	0.00
94 ->	90	0.00	0.00
94 ->	91	-0.04	0.02
94 ->	92	-0.29	0.08
94 ->	93	-34.49	0.77
94 ->	94	-205.47	3.20
94 ->	95	-32.93	0.95

94 ->	96	-0.13	0.04
94 ->	97	-0.02	0.01
94 ->	98	-0.10	0.04
94 ->	99	-0.41	0.16
94 ->	100	-11.13	0.93
94 ->	101	0.24	0.26
94 ->	102	-0.06	0.08
94 ->	103	-0.01	0.02
94 ->	104	0.01	0.00
94 ->	105	0.01	0.00
94 ->	106	0.01	0.00
94 ->	107	0.00	0.00
95 ->	1	-0.13	0.03
95 ->	2	-0.01	0.00
95 ->	3	-0.01	0.00
95 ->	4	-0.00	0.00
95 ->	5	0.01	0.00
95 ->	6	-0.00	0.00
95 ->	7	-0.00	0.00
95 ->	8	0.01	0.00
95 ->	9	0.00	0.00
95 ->	10	0.01	0.00
95 ->	11	0.01	0.00
95 ->	12	-0.03	0.00
95 ->	13	-0.02	0.00
95 ->	14	-0.01	0.01
95 ->	15	-0.05	0.03
95 ->	16	-1.95	0.84
95 ->	17	-0.03	0.04
95 ->	18	-0.86	1.24
95 ->	19	0.01	0.05
95 ->	20	-0.01	0.00
95 ->	21	-0.01	0.00
95 ->	22	-0.05	0.10
95 ->	23	-0.01	0.00
95 ->	24	-0.00	0.00
95 ->	25	-0.02	0.00
95 ->	26	-0.00	0.00
95 ->	27	-0.00	0.00
95 ->	28	-0.00	0.00
95 ->	29	-0.00	0.00
95 ->	30	0.00	0.00
95 ->	31	-0.01	0.00
95 ->	32	-0.00	0.00
95 ->	33	-0.00	0.00
95 ->	34	-0.00	0.00
95 ->	35	0.00	0.00
95 ->	36	-0.00	0.00
95 ->	37	0.00	0.00
95 ->	38	0.00	0.00

95	->	39	0.00	0.00
95	->	40	-0.00	0.00
95	->	41	-0.00	0.00
95	->	42	-0.00	0.00
95	->	43	-0.00	0.00
95	->	44	-0.05	0.02
95	->	45	-0.01	0.01
95	->	46	0.02	0.01
95	->	47	-0.00	0.00
95	->	48	-0.00	0.00
95	->	49	-0.00	0.00
95	->	50	-0.00	0.00
95	->	51	-0.00	0.00
95	->	52	-0.00	0.00
95	->	53	-0.02	0.00
95	->	54	-0.00	0.00
95	->	55	-0.00	0.00
95	->	56	-0.00	0.00
95	->	57	-0.00	0.00
95	->	58	0.01	0.00
95	->	59	-0.01	0.00
95	->	60	-0.00	0.00
95	->	61	-0.00	0.00
95	->	62	0.00	0.00
95	->	63	0.01	0.00
95	->	64	0.01	0.00
95	->	65	0.00	0.00
95	->	66	0.00	0.00
95	->	67	0.00	0.00
95	->	68	-0.00	0.00
95	->	69	-0.01	0.00
95	->	70	-0.00	0.00
95	->	71	-0.00	0.00
95	->	72	-0.02	0.01
95	->	73	-0.01	0.00
95	->	74	0.02	0.00
95	->	75	0.01	0.00
95	->	76	-0.02	0.01
95	->	77	-0.03	0.01
95	->	78	-0.01	0.00
95	->	79	-0.00	0.00
95	->	80	-0.00	0.00
95	->	81	-0.01	0.00
95	->	82	-0.00	0.00
95	->	83	-0.00	0.00
95	->	84	-0.00	0.00
95	->	85	-0.00	0.00
95	->	86	0.00	0.00
95	->	87	0.00	0.00
95	->	88	0.00	0.00

95	->	89	0.01	0.00
95	->	90	0.01	0.00
95	->	91	0.01	0.00
95	->	92	-0.01	0.01
95	->	93	-0.38	0.07
95	->	94	-32.65	0.95
95	->	95	-135.55	2.63
95	->	96	-34.55	1.01
95	->	97	-0.19	0.10
95	->	98	-2.26	0.79
95	->	99	-5.68	0.75
95	->	100	-3.59	0.47
95	->	101	-0.14	0.06
95	->	102	-0.03	0.02
95	->	103	0.01	0.00
95	->	104	0.02	0.00
95	->	105	0.01	0.00
95	->	106	0.01	0.00
95	->	107	0.00	0.00
96	->	1	-0.03	0.01
96	->	2	-0.00	0.00
96	->	3	-0.01	0.01
96	->	4	-0.00	0.00
96	->	5	0.00	0.00
96	->	6	-0.00	0.00
96	->	7	-0.00	0.00
96	->	8	0.00	0.00
96	->	9	0.00	0.00
96	->	10	0.00	0.00
96	->	11	0.00	0.00
96	->	12	-0.01	0.00
96	->	13	-0.01	0.00
96	->	14	-0.00	0.00
96	->	15	-0.04	0.21
96	->	16	-0.27	0.39
96	->	17	-0.07	0.13
96	->	18	-0.14	0.28
96	->	19	-0.00	0.02
96	->	20	-0.00	0.00
96	->	21	-0.00	0.00
96	->	22	-0.03	0.01
96	->	23	-0.00	0.00
96	->	24	-0.00	0.00
96	->	25	-0.01	0.00
96	->	26	-0.00	0.00
96	->	27	-0.00	0.00
96	->	28	-0.00	0.00
96	->	29	-0.00	0.00
96	->	30	0.00	0.00
96	->	31	-0.00	0.00

96 ->	32	-0.00	0.00
96 ->	33	-0.00	0.00
96 ->	34	0.00	0.00
96 ->	35	0.00	0.00
96 ->	36	-0.00	0.00
96 ->	37	0.00	0.00
96 ->	38	0.00	0.00
96 ->	39	-0.00	0.00
96 ->	40	-0.00	0.00
96 ->	41	-0.00	0.00
96 ->	42	-0.00	0.00
96 ->	43	-0.00	0.00
96 ->	44	-0.02	0.01
96 ->	45	-0.00	0.00
96 ->	46	0.02	0.00
96 ->	47	-0.00	0.00
96 ->	48	-0.00	0.00
96 ->	49	-0.00	0.00
96 ->	50	-0.00	0.00
96 ->	51	-0.00	0.00
96 ->	52	-0.00	0.00
96 ->	53	-0.02	0.00
96 ->	54	-0.00	0.00
96 ->	55	-0.00	0.00
96 ->	56	-0.00	0.00
96 ->	57	-0.00	0.00
96 ->	58	0.01	0.00
96 ->	59	-0.00	0.00
96 ->	60	-0.00	0.00
96 ->	61	-0.00	0.00
96 ->	62	0.00	0.00
96 ->	63	0.00	0.00
96 ->	64	0.00	0.00
96 ->	65	0.00	0.00
96 ->	66	0.00	0.00
96 ->	67	0.00	0.00
96 ->	68	-0.00	0.00
96 ->	69	-0.00	0.00
96 ->	70	-0.00	0.00
96 ->	71	0.00	0.00
96 ->	72	-0.01	0.00
96 ->	73	-0.00	0.00
96 ->	74	0.01	0.00
96 ->	75	0.01	0.00
96 ->	76	-0.01	0.01
96 ->	77	-0.02	0.01
96 ->	78	-0.01	0.00
96 ->	79	-0.00	0.00
96 ->	80	-0.00	0.00
96 ->	81	-0.01	0.00

96	->	82	-0.00	0.00
96	->	83	-0.00	0.00
96	->	84	-0.00	0.00
96	->	85	-0.00	0.00
96	->	86	0.00	0.00
96	->	87	0.00	0.00
96	->	88	0.00	0.00
96	->	89	0.00	0.00
96	->	90	0.00	0.00
96	->	91	0.00	0.00
96	->	92	0.00	0.00
96	->	93	-0.03	0.01
96	->	94	-0.13	0.04
96	->	95	-34.30	1.01
96	->	96	-216.44	3.40
96	->	97	-2.79	1.83
96	->	98	-10.06	1.20
96	->	99	-0.56	0.49
96	->	100	-0.22	0.10
96	->	101	-0.01	0.02
96	->	102	0.01	0.01
96	->	103	0.01	0.00
96	->	104	0.01	0.00
96	->	105	0.00	0.00
96	->	106	0.00	0.00
96	->	107	0.00	0.00
97	->	1	-0.01	0.00
97	->	2	-0.00	0.00
97	->	3	-0.01	0.01
97	->	4	-0.00	0.00
97	->	5	0.00	0.00
97	->	6	-0.00	0.00
97	->	7	-0.00	0.00
97	->	8	0.00	0.00
97	->	9	-0.00	0.00
97	->	10	0.00	0.00
97	->	11	0.00	0.00
97	->	12	-0.00	0.00
97	->	13	-0.00	0.00
97	->	14	-0.00	0.00
97	->	15	-0.01	0.00
97	->	16	-0.06	0.04
97	->	17	-0.39	0.65
97	->	18	-0.20	0.36
97	->	19	-0.01	0.01
97	->	20	-0.01	0.01
97	->	21	-0.02	0.02
97	->	22	-0.02	0.01
97	->	23	-0.00	0.00
97	->	24	-0.00	0.00

97	->	25	-0.02	0.04
97	->	26	-0.00	0.00
97	->	27	-0.00	0.00
97	->	28	-0.00	0.00
97	->	29	-0.00	0.00
97	->	30	-0.00	0.00
97	->	31	-0.01	0.00
97	->	32	-0.00	0.01
97	->	33	-0.01	0.01
97	->	34	-0.00	0.00
97	->	35	0.00	0.00
97	->	36	-0.01	0.00
97	->	37	0.00	0.00
97	->	38	0.00	0.00
97	->	39	0.00	0.00
97	->	40	-0.00	0.00
97	->	41	-0.00	0.00
97	->	42	-0.00	0.00
97	->	43	-0.00	0.00
97	->	44	-0.02	0.01
97	->	45	-0.01	0.01
97	->	46	0.01	0.00
97	->	47	-0.00	0.00
97	->	48	-0.00	0.00
97	->	49	-0.00	0.00
97	->	50	-0.00	0.00
97	->	51	-0.00	0.00
97	->	52	-0.00	0.00
97	->	53	-0.01	0.00
97	->	54	-0.00	0.00
97	->	55	-0.00	0.00
97	->	56	-0.00	0.00
97	->	57	-0.00	0.00
97	->	58	0.00	0.00
97	->	59	-0.00	0.00
97	->	60	-0.00	0.00
97	->	61	-0.00	0.00
97	->	62	0.00	0.00
97	->	63	0.00	0.00
97	->	64	0.00	0.00
97	->	65	0.00	0.00
97	->	66	0.00	0.00
97	->	67	0.00	0.00
97	->	68	-0.00	0.00
97	->	69	-0.00	0.00
97	->	70	0.00	0.00
97	->	71	0.00	0.00
97	->	72	-0.00	0.00
97	->	73	-0.00	0.00
97	->	74	0.00	0.00

97 ->	75	0.00	0.00
97 ->	76	-0.00	0.00
97 ->	77	-0.00	0.00
97 ->	78	-0.00	0.00
97 ->	79	-0.00	0.00
97 ->	80	-0.00	0.00
97 ->	81	-0.00	0.00
97 ->	82	-0.00	0.00
97 ->	83	0.00	0.00
97 ->	84	0.00	0.00
97 ->	85	-0.00	0.00
97 ->	86	0.00	0.00
97 ->	87	0.00	0.00
97 ->	88	0.00	0.00
97 ->	89	0.00	0.00
97 ->	90	0.00	0.00
97 ->	91	0.00	0.00
97 ->	92	0.00	0.00
97 ->	93	-0.00	0.00
97 ->	94	-0.02	0.01
97 ->	95	-0.19	0.10
97 ->	96	-2.73	1.80
97 ->	97	-49.27	2.73
97 ->	98	-36.30	1.32
97 ->	99	-0.66	1.03
97 ->	100	-0.17	0.40
97 ->	101	-0.00	0.00
97 ->	102	0.00	0.00
97 ->	103	0.00	0.00
97 ->	104	0.00	0.00
97 ->	105	0.00	0.00
97 ->	106	0.00	0.00
97 ->	107	0.00	0.00
98 ->	1	-0.03	0.01
98 ->	2	-0.00	0.00
98 ->	3	-0.01	0.01
98 ->	4	0.00	0.00
98 ->	5	0.01	0.00
98 ->	6	-0.00	0.00
98 ->	7	-0.00	0.00
98 ->	8	0.01	0.00
98 ->	9	-0.00	0.00
98 ->	10	0.01	0.00
98 ->	11	0.01	0.00
98 ->	12	-0.01	0.00
98 ->	13	-0.01	0.00
98 ->	14	-0.01	0.01
98 ->	15	-0.01	0.03
98 ->	16	-0.24	0.59
98 ->	17	-0.45	0.90

98 ->	18	-0.52	0.81
98 ->	19	0.01	0.03
98 ->	20	-0.01	0.01
98 ->	21	-0.04	0.04
98 ->	22	-0.07	0.03
98 ->	23	-0.01	0.00
98 ->	24	-0.01	0.00
98 ->	25	-0.13	0.18
98 ->	26	-0.00	0.00
98 ->	27	-0.00	0.00
98 ->	28	-0.00	0.00
98 ->	29	-0.00	0.00
98 ->	30	0.00	0.00
98 ->	31	-0.02	0.01
98 ->	32	-0.02	0.04
98 ->	33	-0.29	0.51
98 ->	34	-0.00	0.00
98 ->	35	0.02	0.01
98 ->	36	-0.03	0.01
98 ->	37	-0.00	0.00
98 ->	38	0.01	0.00
98 ->	39	-0.00	0.00
98 ->	40	-0.01	0.00
98 ->	41	-0.00	0.00
98 ->	42	-0.01	0.00
98 ->	43	-0.00	0.01
98 ->	44	-0.09	0.08
98 ->	45	-0.30	0.36
98 ->	46	0.03	0.04
98 ->	47	-0.01	0.01
98 ->	48	-0.03	0.02
98 ->	49	-0.01	0.01
98 ->	50	-0.00	0.00
98 ->	51	-0.00	0.00
98 ->	52	-0.01	0.00
98 ->	53	-0.03	0.01
98 ->	54	-0.00	0.00
98 ->	55	-0.00	0.00
98 ->	56	-0.00	0.00
98 ->	57	-0.00	0.00
98 ->	58	0.01	0.00
98 ->	59	-0.01	0.00
98 ->	60	-0.00	0.00
98 ->	61	-0.00	0.00
98 ->	62	0.00	0.00
98 ->	63	0.00	0.00
98 ->	64	0.00	0.00
98 ->	65	0.00	0.00
98 ->	66	0.00	0.00
98 ->	67	0.00	0.00

98	->	68	-0.00	0.00
98	->	69	-0.00	0.00
98	->	70	-0.00	0.00
98	->	71	0.00	0.00
98	->	72	-0.02	0.00
98	->	73	-0.00	0.00
98	->	74	0.01	0.00
98	->	75	0.01	0.00
98	->	76	-0.01	0.00
98	->	77	-0.01	0.00
98	->	78	-0.01	0.00
98	->	79	-0.00	0.00
98	->	80	-0.00	0.00
98	->	81	-0.00	0.00
98	->	82	-0.00	0.00
98	->	83	0.00	0.00
98	->	84	-0.00	0.00
98	->	85	-0.00	0.00
98	->	86	0.00	0.00
98	->	87	0.00	0.00
98	->	88	0.01	0.00
98	->	89	0.01	0.00
98	->	90	0.01	0.00
98	->	91	0.02	0.01
98	->	92	0.01	0.00
98	->	93	0.01	0.01
98	->	94	-0.10	0.04
98	->	95	-2.27	0.80
98	->	96	-10.11	1.19
98	->	97	-36.04	1.32
98	->	98	-181.52	3.30
98	->	99	-35.85	1.19
98	->	100	-0.30	0.06
98	->	101	-0.02	0.01
98	->	102	0.00	0.00
98	->	103	0.00	0.00
98	->	104	0.00	0.00
98	->	105	0.00	0.00
98	->	106	0.00	0.00
98	->	107	0.00	0.00
99	->	1	-0.03	0.00
99	->	2	-0.00	0.00
99	->	3	-0.00	0.00
99	->	4	0.00	0.00
99	->	5	0.01	0.00
99	->	6	-0.00	0.00
99	->	7	-0.00	0.00
99	->	8	0.00	0.00
99	->	9	0.00	0.00
99	->	10	0.01	0.00

99 ->	11	0.01	0.00
99 ->	12	-0.01	0.00
99 ->	13	-0.01	0.00
99 ->	14	-0.00	0.00
99 ->	15	-0.00	0.00
99 ->	16	-0.09	0.10
99 ->	17	-0.04	0.06
99 ->	18	-0.38	0.53
99 ->	19	0.01	0.02
99 ->	20	-0.01	0.00
99 ->	21	-0.02	0.02
99 ->	22	-0.38	0.74
99 ->	23	-0.01	0.00
99 ->	24	-0.00	0.00
99 ->	25	-0.11	0.09
99 ->	26	-0.00	0.00
99 ->	27	-0.00	0.00
99 ->	28	-0.00	0.00
99 ->	29	-0.00	0.00
99 ->	30	0.00	0.00
99 ->	31	-0.03	0.01
99 ->	32	-0.01	0.01
99 ->	33	-0.41	0.40
99 ->	34	-0.00	0.00
99 ->	35	0.03	0.01
99 ->	36	-0.06	0.03
99 ->	37	-0.00	0.00
99 ->	38	0.02	0.00
99 ->	39	-0.00	0.00
99 ->	40	-0.02	0.01
99 ->	41	-0.01	0.00
99 ->	42	-0.01	0.01
99 ->	43	-0.01	0.01
99 ->	44	-0.24	0.19
99 ->	45	-2.29	2.28
99 ->	46	-0.12	0.11
99 ->	47	-0.03	0.02
99 ->	48	-0.13	0.14
99 ->	49	-0.22	0.33
99 ->	50	-0.01	0.01
99 ->	51	-0.01	0.01
99 ->	52	-0.03	0.02
99 ->	53	-0.15	0.11
99 ->	54	-0.00	0.00
99 ->	55	-0.00	0.00
99 ->	56	-0.00	0.00
99 ->	57	-0.00	0.00
99 ->	58	0.02	0.01
99 ->	59	-0.02	0.00
99 ->	60	-0.01	0.00

99	->	61	-0.01	0.00
99	->	62	0.00	0.00
99	->	63	0.01	0.00
99	->	64	0.00	0.00
99	->	65	0.00	0.00
99	->	66	0.00	0.00
99	->	67	0.00	0.00
99	->	68	-0.01	0.00
99	->	69	-0.01	0.00
99	->	70	-0.00	0.00
99	->	71	-0.00	0.00
99	->	72	-0.04	0.01
99	->	73	-0.00	0.00
99	->	74	0.02	0.00
99	->	75	0.01	0.00
99	->	76	-0.02	0.00
99	->	77	-0.01	0.00
99	->	78	-0.01	0.00
99	->	79	-0.00	0.00
99	->	80	-0.00	0.00
99	->	81	-0.01	0.00
99	->	82	-0.00	0.00
99	->	83	0.00	0.00
99	->	84	-0.00	0.00
99	->	85	-0.00	0.00
99	->	86	0.00	0.00
99	->	87	0.01	0.00
99	->	88	0.01	0.00
99	->	89	0.02	0.00
99	->	90	0.01	0.00
99	->	91	0.02	0.00
99	->	92	0.00	0.01
99	->	93	-0.02	0.02
99	->	94	-0.41	0.16
99	->	95	-5.67	0.75
99	->	96	-0.56	0.49
99	->	97	-0.66	1.03
99	->	98	-35.64	1.17
99	->	99	-94.83	3.67
99	->	100	-34.02	0.98
99	->	101	-0.18	0.06
99	->	102	-0.01	0.01
99	->	103	0.00	0.00
99	->	104	0.01	0.00
99	->	105	0.01	0.00
99	->	106	0.00	0.00
99	->	107	0.00	0.00
100	->	1	-0.01	0.02
100	->	2	-0.00	0.00
100	->	3	-0.00	0.00

100	->	4	0.00	0.00
100	->	5	0.01	0.00
100	->	6	-0.00	0.00
100	->	7	-0.00	0.00
100	->	8	0.00	0.00
100	->	9	0.00	0.00
100	->	10	0.01	0.00
100	->	11	0.01	0.00
100	->	12	-0.01	0.00
100	->	13	-0.01	0.00
100	->	14	-0.00	0.00
100	->	15	0.00	0.00
100	->	16	0.01	0.05
100	->	17	-0.01	0.01
100	->	18	0.07	0.12
100	->	19	-0.04	0.04
100	->	20	-0.01	0.00
100	->	21	-0.01	0.00
100	->	22	-1.22	1.71
100	->	23	-0.01	0.00
100	->	24	-0.00	0.00
100	->	25	-0.06	0.02
100	->	26	-0.00	0.00
100	->	27	-0.00	0.00
100	->	28	-0.00	0.00
100	->	29	-0.00	0.00
100	->	30	0.00	0.00
100	->	31	-0.02	0.00
100	->	32	-0.00	0.00
100	->	33	-0.03	0.01
100	->	34	-0.00	0.00
100	->	35	0.03	0.01
100	->	36	-0.05	0.02
100	->	37	-0.00	0.00
100	->	38	0.02	0.00
100	->	39	-0.00	0.00
100	->	40	-0.02	0.01
100	->	41	-0.00	0.00
100	->	42	-0.01	0.00
100	->	43	-0.01	0.01
100	->	44	-0.98	1.02
100	->	45	-0.59	0.47
100	->	46	-1.33	0.86
100	->	47	-0.04	0.03
100	->	48	-0.14	0.12
100	->	49	-1.37	1.39
100	->	50	-0.02	0.08
100	->	51	-0.01	0.01
100	->	52	-0.07	0.06
100	->	53	-2.71	1.35

100	->	54	-0.00	0.00
100	->	55	-0.00	0.00
100	->	56	-0.01	0.00
100	->	57	-0.00	0.00
100	->	58	0.04	0.02
100	->	59	-0.06	0.03
100	->	60	-0.04	0.02
100	->	61	-0.02	0.04
100	->	62	0.00	0.00
100	->	63	0.01	0.00
100	->	64	0.01	0.00
100	->	65	0.00	0.00
100	->	66	0.01	0.00
100	->	67	0.00	0.00
100	->	68	-0.01	0.00
100	->	69	-0.03	0.01
100	->	70	-0.02	0.01
100	->	71	0.00	0.03
100	->	72	-0.52	0.39
100	->	73	0.04	0.15
100	->	74	0.09	0.05
100	->	75	0.02	0.01
100	->	76	-0.05	0.02
100	->	77	-0.04	0.04
100	->	78	-0.02	0.01
100	->	79	-0.00	0.00
100	->	80	-0.00	0.00
100	->	81	-0.01	0.00
100	->	82	-0.00	0.00
100	->	83	0.00	0.00
100	->	84	-0.00	0.00
100	->	85	-0.00	0.00
100	->	86	0.00	0.00
100	->	87	0.01	0.00
100	->	88	0.02	0.01
100	->	89	0.02	0.01
100	->	90	0.01	0.01
100	->	91	0.01	0.01
100	->	92	-0.04	0.05
100	->	93	-0.50	0.57
100	->	94	-11.14	0.93
100	->	95	-3.60	0.47
100	->	96	-0.22	0.10
100	->	97	-0.16	0.38
100	->	98	-0.30	0.06
100	->	99	-33.76	1.00
100	->	100	-174.56	4.02
100	->	101	-35.88	1.03
100	->	102	-0.35	0.10
100	->	103	-0.04	0.01

100	->	104	0.00	0.00
100	->	105	0.01	0.00
100	->	106	0.00	0.00
100	->	107	0.00	0.00
101	->	1	-0.04	0.01
101	->	2	-0.00	0.00
101	->	3	-0.00	0.00
101	->	4	0.00	0.00
101	->	5	0.01	0.00
101	->	6	-0.00	0.00
101	->	7	0.00	0.00
101	->	8	0.00	0.00
101	->	9	0.00	0.00
101	->	10	0.01	0.00
101	->	11	0.01	0.00
101	->	12	-0.02	0.00
101	->	13	-0.01	0.00
101	->	14	-0.00	0.00
101	->	15	-0.00	0.00
101	->	16	-0.06	0.01
101	->	17	-0.00	0.00
101	->	18	-0.01	0.03
101	->	19	0.02	0.01
101	->	20	-0.00	0.00
101	->	21	-0.00	0.00
101	->	22	0.15	0.18
101	->	23	-0.00	0.00
101	->	24	-0.00	0.00
101	->	25	-0.02	0.00
101	->	26	-0.00	0.00
101	->	27	-0.00	0.00
101	->	28	-0.00	0.00
101	->	29	-0.00	0.00
101	->	30	0.00	0.00
101	->	31	-0.01	0.00
101	->	32	-0.00	0.00
101	->	33	-0.01	0.00
101	->	34	-0.00	0.00
101	->	35	0.02	0.00
101	->	36	-0.02	0.01
101	->	37	-0.00	0.00
101	->	38	0.01	0.00
101	->	39	-0.00	0.00
101	->	40	-0.01	0.00
101	->	41	-0.00	0.00
101	->	42	-0.00	0.00
101	->	43	0.00	0.00
101	->	44	0.66	0.56
101	->	45	-0.01	0.03
101	->	46	-5.48	1.09

101	->	47	-0.15	0.10
101	->	48	-0.02	0.01
101	->	49	-0.28	0.13
101	->	50	-0.03	0.10
101	->	51	-0.00	0.00
101	->	52	-0.02	0.01
101	->	53	-8.92	2.42
101	->	54	-0.00	0.00
101	->	55	-0.00	0.00
101	->	56	-0.00	0.00
101	->	57	-0.00	0.00
101	->	58	0.04	0.03
101	->	59	-0.10	0.04
101	->	60	-0.13	0.09
101	->	61	-1.29	0.80
101	->	62	0.00	0.00
101	->	63	0.03	0.00
101	->	64	0.02	0.00
101	->	65	0.00	0.00
101	->	66	0.02	0.00
101	->	67	0.00	0.00
101	->	68	-0.02	0.00
101	->	69	-0.05	0.02
101	->	70	-0.04	0.02
101	->	71	0.04	0.09
101	->	72	-1.27	0.91
101	->	73	-1.32	0.93
101	->	74	0.06	0.17
101	->	75	0.02	0.02
101	->	76	-0.32	0.41
101	->	77	-0.38	0.52
101	->	78	-0.05	0.03
101	->	79	-0.01	0.00
101	->	80	-0.00	0.00
101	->	81	-0.03	0.01
101	->	82	-0.00	0.00
101	->	83	-0.00	0.00
101	->	84	-0.00	0.00
101	->	85	-0.00	0.00
101	->	86	0.00	0.00
101	->	87	0.02	0.00
101	->	88	0.01	0.01
101	->	89	0.01	0.00
101	->	90	-0.02	0.01
101	->	91	-0.12	0.06
101	->	92	-1.00	0.38
101	->	93	-11.51	0.88
101	->	94	0.24	0.26
101	->	95	-0.14	0.06
101	->	96	-0.01	0.02

101	->	97	-0.00	0.00
101	->	98	-0.02	0.01
101	->	99	-0.18	0.06
101	->	100	-35.75	1.02
101	->	101	-203.65	4.57
101	->	102	-31.60	0.88
101	->	103	-0.14	0.06
101	->	104	-0.00	0.01
101	->	105	0.00	0.00
101	->	106	0.01	0.00
101	->	107	0.00	0.00
102	->	1	-0.07	0.02
102	->	2	-0.00	0.00
102	->	3	-0.00	0.00
102	->	4	-0.00	0.00
102	->	5	0.01	0.00
102	->	6	-0.00	0.00
102	->	7	-0.00	0.00
102	->	8	0.00	0.00
102	->	9	0.00	0.00
102	->	10	0.01	0.00
102	->	11	0.01	0.00
102	->	12	-0.03	0.01
102	->	13	-0.01	0.00
102	->	14	-0.00	0.00
102	->	15	-0.00	0.00
102	->	16	-0.07	0.02
102	->	17	-0.00	0.00
102	->	18	0.01	0.01
102	->	19	0.04	0.01
102	->	20	-0.00	0.00
102	->	21	-0.00	0.00
102	->	22	-0.09	0.08
102	->	23	-0.00	0.00
102	->	24	-0.00	0.00
102	->	25	-0.01	0.00
102	->	26	-0.00	0.00
102	->	27	-0.00	0.00
102	->	28	-0.00	0.00
102	->	29	-0.00	0.00
102	->	30	0.00	0.00
102	->	31	-0.01	0.00
102	->	32	-0.00	0.00
102	->	33	-0.00	0.00
102	->	34	-0.00	0.00
102	->	35	0.01	0.00
102	->	36	-0.01	0.00
102	->	37	0.00	0.00
102	->	38	0.01	0.00
102	->	39	-0.00	0.00

102 ->	40	-0.01	0.00
102 ->	41	-0.00	0.00
102 ->	42	-0.00	0.00
102 ->	43	-0.00	0.00
102 ->	44	0.46	0.42
102 ->	45	-0.00	0.01
102 ->	46	-2.65	2.09
102 ->	47	-0.17	0.15
102 ->	48	-0.00	0.00
102 ->	49	-0.01	0.01
102 ->	50	-0.03	0.02
102 ->	51	-0.00	0.00
102 ->	52	-0.00	0.00
102 ->	53	-0.10	0.04
102 ->	54	-0.00	0.00
102 ->	55	-0.00	0.00
102 ->	56	-0.00	0.00
102 ->	57	-0.00	0.00
102 ->	58	0.02	0.01
102 ->	59	-0.04	0.01
102 ->	60	-0.02	0.01
102 ->	61	-0.13	0.17
102 ->	62	0.00	0.01
102 ->	63	0.04	0.01
102 ->	64	0.03	0.01
102 ->	65	0.00	0.00
102 ->	66	0.02	0.00
102 ->	67	-0.00	0.00
102 ->	68	-0.02	0.01
102 ->	69	-0.04	0.02
102 ->	70	-0.02	0.01
102 ->	71	-0.03	0.02
102 ->	72	-0.62	0.35
102 ->	73	-4.97	1.86
102 ->	74	-0.57	0.83
102 ->	75	-0.01	0.03
102 ->	76	-2.06	3.68
102 ->	77	-3.46	3.84
102 ->	78	-0.15	0.39
102 ->	79	-0.01	0.01
102 ->	80	-0.01	0.01
102 ->	81	-0.08	0.07
102 ->	82	-0.00	0.00
102 ->	83	-0.00	0.00
102 ->	84	-0.00	0.00
102 ->	85	-0.00	0.01
102 ->	86	0.00	0.00
102 ->	87	0.01	0.00
102 ->	88	-0.00	0.00
102 ->	89	-0.01	0.01

102	->	90	-0.10	0.05
102	->	91	-1.13	0.45
102	->	92	-11.78	0.91
102	->	93	-3.78	0.65
102	->	94	-0.06	0.08
102	->	95	-0.03	0.02
102	->	96	0.01	0.01
102	->	97	0.00	0.00
102	->	98	0.00	0.00
102	->	99	-0.01	0.01
102	->	100	-0.35	0.10
102	->	101	-31.43	0.88
102	->	102	-206.50	4.12
102	->	103	-31.68	0.99
102	->	104	-0.19	0.05
102	->	105	-0.00	0.01
102	->	106	0.01	0.00
102	->	107	0.00	0.00
103	->	1	-0.07	0.02
103	->	2	-0.00	0.00
103	->	3	-0.00	0.00
103	->	4	-0.00	0.00
103	->	5	0.01	0.00
103	->	6	-0.00	0.00
103	->	7	0.00	0.00
103	->	8	0.01	0.00
103	->	9	0.00	0.00
103	->	10	0.01	0.00
103	->	11	0.02	0.00
103	->	12	-0.06	0.02
103	->	13	-0.01	0.00
103	->	14	-0.00	0.00
103	->	15	-0.00	0.00
103	->	16	-0.04	0.01
103	->	17	-0.00	0.00
103	->	18	0.02	0.00
103	->	19	0.03	0.00
103	->	20	-0.00	0.00
103	->	21	-0.00	0.00
103	->	22	-0.07	0.03
103	->	23	-0.01	0.00
103	->	24	-0.00	0.00
103	->	25	-0.01	0.00
103	->	26	-0.00	0.00
103	->	27	-0.00	0.00
103	->	28	-0.00	0.00
103	->	29	-0.00	0.00
103	->	30	0.00	0.00
103	->	31	-0.01	0.00
103	->	32	-0.00	0.00

103	->	33	-0.00	0.00
103	->	34	-0.00	0.00
103	->	35	0.01	0.00
103	->	36	-0.00	0.00
103	->	37	0.00	0.00
103	->	38	0.00	0.00
103	->	39	-0.00	0.00
103	->	40	-0.01	0.00
103	->	41	-0.00	0.00
103	->	42	-0.00	0.00
103	->	43	-0.00	0.00
103	->	44	0.00	0.24
103	->	45	-0.00	0.00
103	->	46	-0.32	0.32
103	->	47	-0.10	0.06
103	->	48	-0.00	0.00
103	->	49	0.00	0.01
103	->	50	-0.03	0.01
103	->	51	-0.00	0.00
103	->	52	-0.00	0.00
103	->	53	-0.02	0.01
103	->	54	-0.00	0.00
103	->	55	-0.00	0.00
103	->	56	-0.00	0.00
103	->	57	-0.00	0.00
103	->	58	0.01	0.00
103	->	59	-0.02	0.00
103	->	60	-0.00	0.00
103	->	61	-0.01	0.01
103	->	62	0.00	0.00
103	->	63	0.03	0.01
103	->	64	0.02	0.01
103	->	65	0.00	0.00
103	->	66	0.02	0.00
103	->	67	-0.00	0.00
103	->	68	-0.02	0.01
103	->	69	-0.02	0.00
103	->	70	-0.01	0.00
103	->	71	-0.01	0.01
103	->	72	-0.19	0.66
103	->	73	-0.24	0.22
103	->	74	-1.78	0.58
103	->	75	-0.02	0.04
103	->	76	-0.64	1.14
103	->	77	-7.44	3.38
103	->	78	-0.61	1.93
103	->	79	-0.01	0.01
103	->	80	-0.03	0.02
103	->	81	-0.64	0.71
103	->	82	-0.00	0.00

103	->	83	-0.00	0.00
103	->	84	-0.01	0.00
103	->	85	-0.01	0.03
103	->	86	0.00	0.00
103	->	87	0.01	0.00
103	->	88	-0.01	0.01
103	->	89	-0.13	0.04
103	->	90	-1.09	0.37
103	->	91	-11.79	0.77
103	->	92	-3.66	1.15
103	->	93	-0.35	0.11
103	->	94	-0.01	0.02
103	->	95	0.01	0.00
103	->	96	0.01	0.00
103	->	97	0.00	0.00
103	->	98	0.00	0.00
103	->	99	0.00	0.00
103	->	100	-0.04	0.01
103	->	101	-0.13	0.06
103	->	102	-31.49	0.98
103	->	103	-207.15	3.52
103	->	104	-33.17	0.84
103	->	105	-0.14	0.05
103	->	106	0.00	0.01
103	->	107	-0.00	0.01
104	->	1	-0.05	0.01
104	->	2	-0.00	0.00
104	->	3	0.00	0.00
104	->	4	-0.00	0.00
104	->	5	0.01	0.00
104	->	6	-0.00	0.00
104	->	7	0.00	0.00
104	->	8	0.01	0.00
104	->	9	0.00	0.00
104	->	10	0.01	0.00
104	->	11	0.02	0.00
104	->	12	-0.08	0.04
104	->	13	-0.01	0.00
104	->	14	-0.00	0.00
104	->	15	-0.00	0.00
104	->	16	-0.03	0.00
104	->	17	0.00	0.00
104	->	18	0.01	0.00
104	->	19	0.02	0.00
104	->	20	-0.00	0.00
104	->	21	-0.00	0.00
104	->	22	-0.04	0.01
104	->	23	-0.01	0.00
104	->	24	-0.00	0.00
104	->	25	-0.01	0.00

104 ->	26	-0.00	0.00
104 ->	27	-0.00	0.00
104 ->	28	-0.00	0.00
104 ->	29	-0.00	0.00
104 ->	30	0.00	0.00
104 ->	31	-0.01	0.00
104 ->	32	-0.00	0.00
104 ->	33	-0.00	0.00
104 ->	34	-0.00	0.00
104 ->	35	0.01	0.00
104 ->	36	-0.00	0.00
104 ->	37	0.00	0.00
104 ->	38	0.01	0.00
104 ->	39	-0.00	0.00
104 ->	40	-0.01	0.00
104 ->	41	-0.00	0.00
104 ->	42	-0.00	0.00
104 ->	43	-0.00	0.00
104 ->	44	-0.09	0.04
104 ->	45	-0.00	0.00
104 ->	46	-0.01	0.03
104 ->	47	-0.06	0.04
104 ->	48	-0.00	0.00
104 ->	49	0.00	0.00
104 ->	50	-0.02	0.01
104 ->	51	-0.01	0.00
104 ->	52	-0.00	0.00
104 ->	53	-0.02	0.00
104 ->	54	-0.00	0.00
104 ->	55	-0.00	0.00
104 ->	56	-0.00	0.00
104 ->	57	-0.00	0.00
104 ->	58	0.01	0.00
104 ->	59	-0.02	0.00
104 ->	60	-0.00	0.00
104 ->	61	-0.01	0.00
104 ->	62	0.00	0.00
104 ->	63	0.02	0.00
104 ->	64	0.01	0.00
104 ->	65	0.00	0.00
104 ->	66	0.02	0.00
104 ->	67	-0.00	0.00
104 ->	68	-0.02	0.01
104 ->	69	-0.01	0.00
104 ->	70	-0.01	0.00
104 ->	71	0.00	0.00
104 ->	72	0.82	0.65
104 ->	73	-0.03	0.02
104 ->	74	-3.50	1.51
104 ->	75	-0.09	0.42

104	->	76	-0.08	0.04
104	->	77	-2.31	2.67
104	->	78	-0.56	1.02
104	->	79	-0.01	0.01
104	->	80	-0.02	0.01
104	->	81	-2.53	3.01
104	->	82	-0.01	0.01
104	->	83	-0.01	0.00
104	->	84	-0.01	0.00
104	->	85	-0.04	0.16
104	->	86	-0.01	0.01
104	->	87	-0.01	0.01
104	->	88	-0.06	0.04
104	->	89	-1.77	0.65
104	->	90	-6.30	0.50
104	->	91	-3.00	0.54
104	->	92	-0.29	0.10
104	->	93	-0.03	0.02
104	->	94	0.01	0.00
104	->	95	0.02	0.00
104	->	96	0.01	0.00
104	->	97	0.00	0.00
104	->	98	0.00	0.00
104	->	99	0.01	0.00
104	->	100	0.00	0.00
104	->	101	-0.00	0.01
104	->	102	-0.19	0.05
104	->	103	-32.95	0.81
104	->	104	-129.07	3.96
104	->	105	-34.37	0.76
104	->	106	-0.29	0.07
104	->	107	-0.06	0.15
105	->	1	-0.04	0.00
105	->	2	0.00	0.00
105	->	3	0.00	0.00
105	->	4	-0.00	0.00
105	->	5	0.01	0.00
105	->	6	-0.00	0.00
105	->	7	0.00	0.00
105	->	8	0.01	0.00
105	->	9	0.00	0.00
105	->	10	0.02	0.00
105	->	11	0.03	0.01
105	->	12	-0.07	0.02
105	->	13	-0.01	0.00
105	->	14	-0.00	0.00
105	->	15	-0.00	0.00
105	->	16	-0.02	0.00
105	->	17	0.00	0.00
105	->	18	0.01	0.00

105 ->	19	0.02	0.00
105 ->	20	-0.00	0.00
105 ->	21	-0.00	0.00
105 ->	22	-0.03	0.00
105 ->	23	-0.00	0.00
105 ->	24	-0.00	0.00
105 ->	25	-0.01	0.00
105 ->	26	-0.00	0.00
105 ->	27	-0.00	0.00
105 ->	28	-0.00	0.00
105 ->	29	-0.00	0.00
105 ->	30	0.00	0.00
105 ->	31	-0.02	0.00
105 ->	32	-0.00	0.00
105 ->	33	-0.00	0.00
105 ->	34	-0.00	0.00
105 ->	35	0.01	0.00
105 ->	36	-0.00	0.00
105 ->	37	0.00	0.00
105 ->	38	0.01	0.00
105 ->	39	0.00	0.00
105 ->	40	-0.02	0.00
105 ->	41	-0.00	0.00
105 ->	42	-0.00	0.00
105 ->	43	-0.00	0.00
105 ->	44	-0.05	0.01
105 ->	45	-0.00	0.00
105 ->	46	0.02	0.00
105 ->	47	-0.01	0.01
105 ->	48	-0.00	0.00
105 ->	49	-0.00	0.00
105 ->	50	-0.01	0.00
105 ->	51	-0.00	0.00
105 ->	52	-0.00	0.00
105 ->	53	-0.01	0.00
105 ->	54	-0.00	0.00
105 ->	55	-0.00	0.00
105 ->	56	-0.00	0.00
105 ->	57	-0.00	0.00
105 ->	58	0.00	0.00
105 ->	59	-0.01	0.00
105 ->	60	-0.00	0.00
105 ->	61	-0.00	0.00
105 ->	62	-0.00	0.00
105 ->	63	0.01	0.00
105 ->	64	0.01	0.00
105 ->	65	0.00	0.00
105 ->	66	0.01	0.00
105 ->	67	-0.00	0.00
105 ->	68	-0.01	0.00

105	->	69	-0.01	0.00
105	->	70	-0.01	0.00
105	->	71	-0.00	0.00
105	->	72	0.05	0.25
105	->	73	-0.01	0.00
105	->	74	-0.68	0.44
105	->	75	-0.08	0.11
105	->	76	-0.02	0.01
105	->	77	-0.05	0.02
105	->	78	-0.85	0.78
105	->	79	-0.01	0.00
105	->	80	-0.00	0.00
105	->	81	-0.16	0.16
105	->	82	-0.01	0.01
105	->	83	-0.00	0.00
105	->	84	-0.00	0.00
105	->	85	-0.04	0.14
105	->	86	-0.04	0.03
105	->	87	-0.11	0.06
105	->	88	-0.32	0.24
105	->	89	-11.38	0.78
105	->	90	-1.22	0.67
105	->	91	-0.34	0.09
105	->	92	-0.03	0.01
105	->	93	0.01	0.00
105	->	94	0.01	0.00
105	->	95	0.01	0.00
105	->	96	0.00	0.00
105	->	97	0.00	0.00
105	->	98	0.00	0.00
105	->	99	0.01	0.00
105	->	100	0.01	0.00
105	->	101	0.00	0.00
105	->	102	-0.00	0.01
105	->	103	-0.14	0.05
105	->	104	-34.14	0.76
105	->	105	-217.75	2.47
105	->	106	-33.52	0.99
105	->	107	-0.86	1.91
106	->	1	-0.02	0.00
106	->	2	0.00	0.00
106	->	3	0.00	0.00
106	->	4	-0.00	0.00
106	->	5	0.01	0.00
106	->	6	-0.00	0.00
106	->	7	0.00	0.00
106	->	8	0.01	0.00
106	->	9	0.00	0.00
106	->	10	0.01	0.00
106	->	11	0.02	0.01

106 ->	12	-0.04	0.01
106 ->	13	-0.01	0.00
106 ->	14	-0.00	0.00
106 ->	15	-0.00	0.00
106 ->	16	-0.01	0.00
106 ->	17	0.00	0.00
106 ->	18	0.01	0.00
106 ->	19	0.01	0.00
106 ->	20	0.00	0.00
106 ->	21	0.00	0.00
106 ->	22	-0.02	0.00
106 ->	23	-0.00	0.00
106 ->	24	-0.00	0.00
106 ->	25	-0.01	0.00
106 ->	26	-0.00	0.00
106 ->	27	-0.00	0.00
106 ->	28	-0.00	0.00
106 ->	29	-0.00	0.00
106 ->	30	-0.00	0.00
106 ->	31	-0.02	0.01
106 ->	32	-0.00	0.00
106 ->	33	-0.00	0.00
106 ->	34	-0.00	0.00
106 ->	35	0.00	0.00
106 ->	36	-0.00	0.00
106 ->	37	0.00	0.00
106 ->	38	0.00	0.00
106 ->	39	-0.00	0.00
106 ->	40	-0.01	0.01
106 ->	41	-0.00	0.00
106 ->	42	-0.01	0.00
106 ->	43	-0.01	0.00
106 ->	44	-0.02	0.00
106 ->	45	-0.00	0.00
106 ->	46	0.02	0.00
106 ->	47	-0.02	0.01
106 ->	48	-0.00	0.00
106 ->	49	0.00	0.00
106 ->	50	-0.01	0.02
106 ->	51	-0.01	0.00
106 ->	52	-0.00	0.00
106 ->	53	-0.01	0.00
106 ->	54	-0.01	0.00
106 ->	55	-0.00	0.00
106 ->	56	-0.00	0.00
106 ->	57	-0.00	0.00
106 ->	58	0.00	0.00
106 ->	59	-0.01	0.00
106 ->	60	-0.00	0.00
106 ->	61	-0.00	0.00

106	->	62	0.00	0.00
106	->	63	0.01	0.00
106	->	64	0.01	0.00
106	->	65	0.00	0.00
106	->	66	0.01	0.00
106	->	67	-0.00	0.00
106	->	68	-0.02	0.01
106	->	69	-0.01	0.00
106	->	70	-0.01	0.00
106	->	71	-0.00	0.00
106	->	72	0.04	0.13
106	->	73	-0.00	0.00
106	->	74	0.02	0.06
106	->	75	0.00	0.02
106	->	76	-0.02	0.00
106	->	77	-0.04	0.01
106	->	78	-2.40	2.46
106	->	79	-0.01	0.00
106	->	80	-0.00	0.00
106	->	81	-0.06	0.02
106	->	82	-0.01	0.01
106	->	83	-0.00	0.00
106	->	84	-0.00	0.00
106	->	85	-0.01	0.02
106	->	86	-0.27	0.18
106	->	87	-2.02	1.79
106	->	88	-11.27	1.65
106	->	89	-4.15	0.98
106	->	90	-0.32	0.16
106	->	91	-0.04	0.02
106	->	92	0.01	0.00
106	->	93	0.02	0.00
106	->	94	0.01	0.00
106	->	95	0.01	0.00
106	->	96	0.00	0.00
106	->	97	0.00	0.00
106	->	98	0.00	0.00
106	->	99	0.00	0.00
106	->	100	0.00	0.00
106	->	101	0.01	0.00
106	->	102	0.01	0.00
106	->	103	0.00	0.01
106	->	104	-0.29	0.07
106	->	105	-33.22	0.99
106	->	106	-181.90	2.68
106	->	107	-35.33	1.62
107	->	1	-0.01	0.00
107	->	2	0.00	0.00
107	->	3	0.00	0.00
107	->	4	-0.00	0.00

107	->	5	0.01	0.00
107	->	6	-0.00	0.00
107	->	7	-0.00	0.00
107	->	8	0.00	0.00
107	->	9	0.00	0.00
107	->	10	0.01	0.00
107	->	11	0.01	0.00
107	->	12	-0.01	0.01
107	->	13	-0.00	0.00
107	->	14	-0.00	0.00
107	->	15	-0.00	0.00
107	->	16	-0.00	0.00
107	->	17	0.00	0.00
107	->	18	0.01	0.00
107	->	19	0.01	0.00
107	->	20	0.00	0.00
107	->	21	0.00	0.00
107	->	22	-0.01	0.00
107	->	23	-0.00	0.00
107	->	24	0.00	0.00
107	->	25	-0.01	0.00
107	->	26	-0.00	0.00
107	->	27	-0.00	0.00
107	->	28	-0.00	0.00
107	->	29	-0.00	0.00
107	->	30	0.00	0.00
107	->	31	-0.02	0.01
107	->	32	-0.00	0.00
107	->	33	-0.00	0.00
107	->	34	-0.00	0.00
107	->	35	0.01	0.00
107	->	36	-0.00	0.00
107	->	37	0.00	0.00
107	->	38	0.01	0.00
107	->	39	-0.00	0.00
107	->	40	-0.03	0.01
107	->	41	-0.00	0.00
107	->	42	-0.00	0.00
107	->	43	-0.00	0.00
107	->	44	-0.01	0.00
107	->	45	0.00	0.00
107	->	46	0.01	0.00
107	->	47	-0.01	0.00
107	->	48	-0.00	0.00
107	->	49	-0.00	0.00
107	->	50	-0.00	0.00
107	->	51	-0.01	0.00
107	->	52	-0.00	0.00
107	->	53	-0.01	0.00
107	->	54	-0.00	0.00

107 ->	55	-0.00	0.00
107 ->	56	-0.00	0.00
107 ->	57	-0.00	0.00
107 ->	58	0.00	0.00
107 ->	59	-0.01	0.00
107 ->	60	-0.00	0.00
107 ->	61	-0.00	0.00
107 ->	62	0.00	0.00
107 ->	63	0.00	0.00
107 ->	64	0.00	0.00
107 ->	65	0.00	0.00
107 ->	66	0.01	0.00
107 ->	67	-0.00	0.00
107 ->	68	-0.01	0.00
107 ->	69	-0.00	0.00
107 ->	70	-0.00	0.00
107 ->	71	-0.00	0.00
107 ->	72	-0.03	0.02
107 ->	73	-0.00	0.00
107 ->	74	0.01	0.00
107 ->	75	0.01	0.00
107 ->	76	-0.01	0.00
107 ->	77	-0.01	0.00
107 ->	78	-0.03	0.22
107 ->	79	-0.00	0.00
107 ->	80	-0.00	0.00
107 ->	81	-0.01	0.00
107 ->	82	-0.00	0.00
107 ->	83	-0.00	0.00
107 ->	84	-0.00	0.00
107 ->	85	-0.00	0.00
107 ->	86	-1.65	1.32
107 ->	87	-8.45	4.05
107 ->	88	0.12	0.13
107 ->	89	-0.16	0.22
107 ->	90	-0.22	0.48
107 ->	91	-0.03	0.10
107 ->	92	0.01	0.01
107 ->	93	0.01	0.00
107 ->	94	0.00	0.00
107 ->	95	0.00	0.00
107 ->	96	0.00	0.00
107 ->	97	0.00	0.00
107 ->	98	0.00	0.00
107 ->	99	0.00	0.00
107 ->	100	0.00	0.00
107 ->	101	0.00	0.00
107 ->	102	0.00	0.00
107 ->	103	-0.00	0.01
107 ->	104	-0.06	0.14

107 ->	105	-0.85	1.89
107 ->	106	-35.09	1.61
107 ->	107	-216.81	4.63
