# Supplementary material for: Investigating dynamic and energetic determinants of protein nucleic acid recognition: analysis of the zinc finger zif268-DNA complexes
Source: BMC Struct Biol. 2010 Nov 24;10:42. doi: 10.1186/1472-6807-10-42 (PMC3002361; doi:10.1186/1472-6807-10-42)
Supplement: Additional file 8 — Average pair interaction energies for 1AAY Complex and their standard deviations. [file 1472-6807-10-42-S8.PDF]

=>> 1AAY

Residue ->	Residue	TGBTOT	SD
1 ->	1	-248.44	6.53
1 ->	2	-1.00	0.39
1 ->	3	-1.09	0.23
1 ->	4	-0.13	0.03
1 ->	5	-0.12	0.02
1 ->	6	-0.00	0.00
1 ->	7	-0.00	0.00
1 ->	8	-0.05	0.03
1 ->	9	-0.01	0.00
1 ->	10	-0.09	0.01
1 ->	11	-0.16	0.06
1 ->	12	1.90	1.60
1 ->	13	-3.54	1.58
1 ->	14	-2.85	1.06
1 ->	15	-4.23	1.74
1 ->	16	-0.03	0.07
1 ->	17	-0.05	0.03
1 ->	18	-0.12	0.05
1 ->	19	-5.96	5.76
1 ->	20	-0.08	0.05
1 ->	21	0.00	0.01
1 ->	22	0.34	0.41
1 ->	23	0.00	0.01
1 ->	24	-0.00	0.00
1 ->	25	0.04	0.01
1 ->	26	0.00	0.00
1 ->	27	-0.01	0.00
1 ->	28	0.00	0.00
1 ->	29	0.00	0.00
1 ->	30	-0.00	0.00
1 ->	31	0.02	0.00
1 ->	32	0.00	0.00
1 ->	33	-0.00	0.00
1 ->	34	0.00	0.00
1 ->	35	-0.01	0.00
1 ->	36	0.00	0.00
1 ->	37	-0.00	0.00
1 ->	38	-0.01	0.00
1 ->	39	-0.00	0.00
1 ->	40	0.01	0.00
1 ->	41	0.00	0.00
1 ->	42	0.00	0.00
1 ->	43	-0.00	0.00
1 ->	44	0.07	0.02
1 ->	45	0.00	0.00
1 ->	46	-0.03	0.01

1	->	47	0.00	0.00
1	->	48	0.00	0.00
1	->	49	0.00	0.00
1	->	50	0.00	0.00
1	->	51	0.00	0.00
1	->	52	0.00	0.00
1	->	53	0.01	0.00
1	->	54	0.00	0.00
1	->	55	0.00	0.00
1	->	56	0.00	0.00
1	->	57	0.00	0.00
1	->	58	-0.01	0.00
1	->	59	0.01	0.00
1	->	60	0.00	0.00
1	->	61	0.00	0.00
1	->	62	-0.00	0.00
1	->	63	-0.01	0.00
1	->	64	-0.01	0.00
1	->	65	-0.00	0.00
1	->	66	-0.00	0.00
1	->	67	-0.00	0.00
1	->	68	0.01	0.00
1	->	69	0.00	0.00
1	->	70	0.00	0.00
1	->	71	-0.00	0.00
1	->	72	0.02	0.00
1	->	73	0.00	0.00
1	->	74	-0.02	0.00
1	->	75	-0.01	0.00
1	->	76	0.02	0.00
1	->	77	0.03	0.01
1	->	78	0.02	0.00
1	->	79	0.00	0.00
1	->	80	0.00	0.00
1	->	81	0.02	0.01
1	->	82	0.00	0.00
1	->	83	0.00	0.00
1	->	84	0.00	0.00
1	->	85	0.00	0.00
1	->	86	-0.00	0.00
1	->	87	-0.01	0.00
1	->	88	-0.01	0.00
1	->	89	-0.01	0.00
1	->	90	-0.03	0.00
1	->	91	-0.09	0.03
1	->	92	-1.24	0.79
1	->	93	-9.92	4.52
1	->	94	-2.96	2.41
1	->	95	-0.12	0.02
1	->	96	-0.03	0.00

1 ->	97	-0.01	0.00
1 ->	98	-0.03	0.01
1 ->	99	-0.02	0.00
1 ->	100	-0.01	0.01
1 ->	101	-0.03	0.00
1 ->	102	-0.05	0.01
1 ->	103	-0.06	0.01
1 ->	104	-0.05	0.01
1 ->	105	-0.05	0.01
1 ->	106	-0.03	0.00
1 ->	107	-0.01	0.00
2 ->	1	-1.00	0.39
2 ->	2	1.35	0.69
2 ->	3	-12.51	0.60
2 ->	4	-0.36	0.10
2 ->	5	-0.01	0.00
2 ->	6	-0.01	0.00
2 ->	7	-0.00	0.00
2 ->	8	-0.00	0.00
2 ->	9	-0.00	0.00
2 ->	10	-0.00	0.00
2 ->	11	-0.00	0.00
2 ->	12	-0.01	0.01
2 ->	13	-1.45	0.99
2 ->	14	-1.04	0.20
2 ->	15	-0.97	0.28
2 ->	16	-0.16	0.12
2 ->	17	-0.03	0.02
2 ->	18	-0.01	0.00
2 ->	19	-0.02	0.01
2 ->	20	-0.04	0.01
2 ->	21	-0.00	0.00
2 ->	22	-0.00	0.00
2 ->	23	-0.00	0.00
2 ->	24	-0.00	0.00
2 ->	25	-0.00	0.00
2 ->	26	-0.00	0.00
2 ->	27	-0.00	0.00
2 ->	28	-0.00	0.00
2 ->	29	-0.00	0.00
2 ->	30	-0.00	0.00
2 ->	31	0.00	0.00
2 ->	32	0.00	0.00
2 ->	33	0.00	0.00
2 ->	34	0.00	0.00
2 ->	35	-0.00	0.00
2 ->	36	0.00	0.00
2 ->	37	0.00	0.00
2 ->	38	0.00	0.00
2 ->	39	0.00	0.00

2	->	40	0.00	0.00
2	->	41	0.00	0.00
2	->	42	0.00	0.00
2	->	43	-0.00	0.00
2	->	44	0.00	0.00
2	->	45	0.00	0.00
2	->	46	-0.00	0.00
2	->	47	0.00	0.00
2	->	48	0.00	0.00
2	->	49	0.00	0.00
2	->	50	0.00	0.00
2	->	51	0.00	0.00
2	->	52	0.00	0.00
2	->	53	0.00	0.00
2	->	54	0.00	0.00
2	->	55	0.00	0.00
2	->	56	0.00	0.00
2	->	57	0.00	0.00
2	->	58	-0.00	0.00
2	->	59	0.00	0.00
2	->	60	0.00	0.00
2	->	61	0.00	0.00
2	->	62	-0.00	0.00
2	->	63	-0.00	0.00
2	->	64	-0.00	0.00
2	->	65	0.00	0.00
2	->	66	-0.00	0.00
2	->	67	0.00	0.00
2	->	68	0.00	0.00
2	->	69	0.00	0.00
2	->	70	0.00	0.00
2	->	71	-0.00	0.00
2	->	72	0.00	0.00
2	->	73	0.00	0.00
2	->	74	-0.00	0.00
2	->	75	-0.00	0.00
2	->	76	0.00	0.00
2	->	77	0.00	0.00
2	->	78	0.00	0.00
2	->	79	0.00	0.00
2	->	80	0.00	0.00
2	->	81	0.00	0.00
2	->	82	0.00	0.00
2	->	83	0.00	0.00
2	->	84	0.00	0.00
2	->	85	0.00	0.00
2	->	86	-0.00	0.00
2	->	87	-0.00	0.00
2	->	88	-0.00	0.00
2	->	89	-0.00	0.00

2	->	90	-0.00	0.00
2	->	91	-0.00	0.00
2	->	92	-0.00	0.00
2	->	93	-0.02	0.01
2	->	94	-0.02	0.01
2	->	95	-0.01	0.00
2	->	96	-0.00	0.00
2	->	97	-0.00	0.00
2	->	98	-0.00	0.00
2	->	99	-0.00	0.00
2	->	100	-0.00	0.00
2	->	101	-0.00	0.00
2	->	102	-0.00	0.00
2	->	103	-0.00	0.00
2	->	104	-0.00	0.00
2	->	105	-0.00	0.00
2	->	106	-0.00	0.00
2	->	107	0.00	0.00
3	->	1	-1.10	0.23
3	->	2	-12.64	0.59
3	->	3	18.79	1.55
3	->	4	-10.01	0.45
3	->	5	-0.48	0.08
3	->	6	-0.04	0.01
3	->	7	0.01	0.01
3	->	8	-0.02	0.02
3	->	9	-0.00	0.00
3	->	10	-0.02	0.01
3	->	11	-0.01	0.00
3	->	12	-0.22	0.07
3	->	13	-3.16	0.65
3	->	14	-3.92	0.51
3	->	15	-1.19	0.42
3	->	16	-1.77	0.82
3	->	17	-1.35	0.80
3	->	18	-0.02	0.02
3	->	19	-0.12	0.05
3	->	20	-1.56	0.44
3	->	21	-0.03	0.01
3	->	22	-0.01	0.01
3	->	23	-0.03	0.01
3	->	24	-0.02	0.01
3	->	25	-0.00	0.00
3	->	26	-0.00	0.00
3	->	27	-0.01	0.00
3	->	28	-0.00	0.00
3	->	29	0.00	0.00
3	->	30	-0.00	0.00
3	->	31	0.00	0.00
3	->	32	0.00	0.00

3 ->	33	-0.00	0.00
3 ->	34	0.00	0.00
3 ->	35	0.00	0.00
3 ->	36	0.00	0.00
3 ->	37	0.00	0.00
3 ->	38	0.00	0.00
3 ->	39	0.00	0.00
3 ->	40	-0.00	0.00
3 ->	41	0.00	0.00
3 ->	42	-0.00	0.00
3 ->	43	-0.00	0.00
3 ->	44	-0.00	0.00
3 ->	45	0.00	0.00
3 ->	46	0.00	0.00
3 ->	47	-0.00	0.00
3 ->	48	0.00	0.00
3 ->	49	-0.00	0.00
3 ->	50	-0.00	0.00
3 ->	51	-0.00	0.00
3 ->	52	-0.00	0.00
3 ->	53	-0.00	0.00
3 ->	54	0.00	0.00
3 ->	55	-0.00	0.00
3 ->	56	0.00	0.00
3 ->	57	0.00	0.00
3 ->	58	0.00	0.00
3 ->	59	-0.00	0.00
3 ->	60	0.00	0.00
3 ->	61	0.00	0.00
3 ->	62	0.00	0.00
3 ->	63	0.00	0.00
3 ->	64	0.00	0.00
3 ->	65	0.00	0.00
3 ->	66	0.00	0.00
3 ->	67	0.00	0.00
3 ->	68	-0.00	0.00
3 ->	69	-0.00	0.00
3 ->	70	-0.00	0.00
3 ->	71	0.00	0.00
3 ->	72	-0.00	0.00
3 ->	73	-0.00	0.00
3 ->	74	0.00	0.00
3 ->	75	0.00	0.00
3 ->	76	-0.00	0.00
3 ->	77	-0.00	0.00
3 ->	78	-0.00	0.00
3 ->	79	0.00	0.00
3 ->	80	0.00	0.00
3 ->	81	-0.00	0.00
3 ->	82	0.00	0.00

3	->	83	0.00	0.00
3	->	84	-0.00	0.00
3	->	85	-0.00	0.00
3	->	86	0.00	0.00
3	->	87	0.00	0.00
3	->	88	0.00	0.00
3	->	89	0.00	0.00
3	->	90	-0.00	0.00
3	->	91	-0.00	0.00
3	->	92	-0.01	0.01
3	->	93	-0.01	0.01
3	->	94	-0.01	0.00
3	->	95	-0.00	0.00
3	->	96	-0.00	0.00
3	->	97	-0.02	0.01
3	->	98	-0.01	0.00
3	->	99	-0.00	0.00
3	->	100	-0.00	0.00
3	->	101	-0.00	0.00
3	->	102	-0.00	0.00
3	->	103	-0.00	0.00
3	->	104	-0.00	0.00
3	->	105	0.00	0.00
3	->	106	0.00	0.00
3	->	107	0.00	0.00
4	->	1	-0.13	0.03
4	->	2	-0.38	0.11
4	->	3	-9.98	0.44
4	->	4	22.80	1.07
4	->	5	-12.63	0.39
4	->	6	-0.43	0.12
4	->	7	0.05	0.08
4	->	8	-0.23	0.35
4	->	9	-0.02	0.02
4	->	10	-0.14	0.06
4	->	11	-0.04	0.02
4	->	12	-0.21	0.20
4	->	13	-1.90	0.47
4	->	14	-0.88	0.44
4	->	15	-0.00	0.01
4	->	16	-0.02	0.01
4	->	17	-0.01	0.00
4	->	18	0.00	0.00
4	->	19	-0.01	0.01
4	->	20	-0.91	0.31
4	->	21	-0.01	0.01
4	->	22	0.01	0.01
4	->	23	-0.13	0.05
4	->	24	-0.05	0.03
4	->	25	-0.00	0.00

4 ->	26	-0.00	0.00
4 ->	27	-0.05	0.02
4 ->	28	-0.00	0.00
4 ->	29	-0.00	0.00
4 ->	30	-0.00	0.00
4 ->	31	0.00	0.00
4 ->	32	-0.00	0.00
4 ->	33	-0.00	0.00
4 ->	34	0.00	0.00
4 ->	35	0.00	0.00
4 ->	36	-0.00	0.00
4 ->	37	0.00	0.00
4 ->	38	-0.00	0.00
4 ->	39	0.00	0.00
4 ->	40	0.00	0.00
4 ->	41	0.00	0.00
4 ->	42	0.00	0.00
4 ->	43	0.00	0.00
4 ->	44	-0.00	0.00
4 ->	45	-0.00	0.00
4 ->	46	0.00	0.00
4 ->	47	0.00	0.00
4 ->	48	0.00	0.00
4 ->	49	-0.00	0.00
4 ->	50	0.00	0.00
4 ->	51	0.00	0.00
4 ->	52	0.00	0.00
4 ->	53	-0.00	0.00
4 ->	54	0.00	0.00
4 ->	55	0.00	0.00
4 ->	56	0.00	0.00
4 ->	57	0.00	0.00
4 ->	58	0.00	0.00
4 ->	59	0.00	0.00
4 ->	60	0.00	0.00
4 ->	61	0.00	0.00
4 ->	62	0.00	0.00
4 ->	63	-0.00	0.00
4 ->	64	-0.00	0.00
4 ->	65	0.00	0.00
4 ->	66	0.00	0.00
4 ->	67	0.00	0.00
4 ->	68	0.00	0.00
4 ->	69	0.00	0.00
4 ->	70	0.00	0.00
4 ->	71	0.00	0.00
4 ->	72	0.00	0.00
4 ->	73	0.00	0.00
4 ->	74	-0.00	0.00
4 ->	75	-0.00	0.00

4	->	76	0.00	0.00
4	->	77	0.00	0.00
4	->	78	0.00	0.00
4	->	79	0.00	0.00
4	->	80	0.00	0.00
4	->	81	0.00	0.00
4	->	82	0.00	0.00
4	->	83	0.00	0.00
4	->	84	0.00	0.00
4	->	85	0.00	0.00
4	->	86	-0.00	0.00
4	->	87	-0.00	0.00
4	->	88	0.00	0.00
4	->	89	-0.00	0.00
4	->	90	-0.00	0.00
4	->	91	-0.01	0.00
4	->	92	-0.02	0.01
4	->	93	-0.01	0.01
4	->	94	-0.00	0.00
4	->	95	-0.00	0.00
4	->	96	0.00	0.00
4	->	97	-0.00	0.00
4	->	98	0.00	0.00
4	->	99	0.00	0.00
4	->	100	0.00	0.00
4	->	101	0.00	0.00
4	->	102	-0.00	0.00
4	->	103	-0.00	0.00
4	->	104	-0.00	0.00
4	->	105	-0.00	0.00
4	->	106	-0.00	0.00
4	->	107	-0.00	0.00
5	->	1	-0.12	0.02
5	->	2	-0.01	0.00
5	->	3	-0.48	0.08
5	->	4	-12.68	0.39
5	->	5	-30.92	2.33
5	->	6	-1.64	0.58
5	->	7	-5.49	1.93
5	->	8	-0.11	0.35
5	->	9	-0.03	0.12
5	->	10	1.41	0.31
5	->	11	0.08	0.02
5	->	12	-0.33	0.32
5	->	13	-0.38	0.10
5	->	14	-0.62	0.34
5	->	15	0.01	0.00
5	->	16	-0.03	0.00
5	->	17	-0.00	0.00
5	->	18	0.03	0.00

5 ->	19	0.01	0.01
5 ->	20	-0.88	0.29
5 ->	21	-0.00	0.01
5 ->	22	-0.15	0.04
5 ->	23	1.86	0.96
5 ->	24	-0.37	0.38
5 ->	25	-0.05	0.01
5 ->	26	-0.05	0.01
5 ->	27	1.70	0.52
5 ->	28	-0.02	0.01
5 ->	29	-0.00	0.00
5 ->	30	-0.01	0.00
5 ->	31	-0.02	0.00
5 ->	32	-0.00	0.00
5 ->	33	0.00	0.00
5 ->	34	-0.00	0.00
5 ->	35	0.00	0.00
5 ->	36	-0.00	0.00
5 ->	37	0.00	0.00
5 ->	38	0.00	0.00
5 ->	39	0.00	0.00
5 ->	40	-0.01	0.00
5 ->	41	-0.00	0.00
5 ->	42	-0.00	0.00
5 ->	43	-0.00	0.00
5 ->	44	-0.03	0.00
5 ->	45	-0.00	0.00
5 ->	46	0.01	0.00
5 ->	47	-0.00	0.00
5 ->	48	-0.00	0.00
5 ->	49	-0.00	0.00
5 ->	50	-0.00	0.00
5 ->	51	-0.00	0.00
5 ->	52	-0.00	0.00
5 ->	53	-0.00	0.00
5 ->	54	-0.00	0.00
5 ->	55	0.00	0.00
5 ->	56	-0.00	0.00
5 ->	57	-0.00	0.00
5 ->	58	0.00	0.00
5 ->	59	-0.00	0.00
5 ->	60	-0.00	0.00
5 ->	61	-0.00	0.00
5 ->	62	0.00	0.00
5 ->	63	0.00	0.00
5 ->	64	0.00	0.00
5 ->	65	0.00	0.00
5 ->	66	0.00	0.00
5 ->	67	0.00	0.00
5 ->	68	-0.00	0.00

5	->	69	-0.00	0.00
5	->	70	-0.00	0.00
5	->	71	0.00	0.00
5	->	72	-0.01	0.00
5	->	73	-0.00	0.00
5	->	74	0.01	0.00
5	->	75	0.00	0.00
5	->	76	-0.00	0.00
5	->	77	-0.01	0.00
5	->	78	-0.01	0.00
5	->	79	-0.00	0.00
5	->	80	-0.00	0.00
5	->	81	-0.00	0.00
5	->	82	-0.00	0.00
5	->	83	0.00	0.00
5	->	84	-0.00	0.00
5	->	85	-0.00	0.00
5	->	86	0.00	0.00
5	->	87	0.00	0.00
5	->	88	0.00	0.00
5	->	89	0.01	0.00
5	->	90	0.02	0.00
5	->	91	0.06	0.03
5	->	92	0.11	0.06
5	->	93	0.05	0.01
5	->	94	0.02	0.00
5	->	95	0.01	0.00
5	->	96	0.01	0.00
5	->	97	0.00	0.00
5	->	98	0.02	0.00
5	->	99	0.01	0.00
5	->	100	0.01	0.00
5	->	101	0.01	0.00
5	->	102	0.01	0.00
5	->	103	0.01	0.00
5	->	104	0.01	0.00
5	->	105	0.01	0.00
5	->	106	0.01	0.00
5	->	107	0.01	0.00
6	->	1	-0.00	0.00
6	->	2	-0.01	0.00
6	->	3	-0.04	0.01
6	->	4	-0.44	0.13
6	->	5	-1.74	0.58
6	->	6	0.71	0.53
6	->	7	-13.03	0.46
6	->	8	-0.42	0.15
6	->	9	-0.04	0.01
6	->	10	-0.04	0.01
6	->	11	-0.00	0.00

6 ->	12	-0.01	0.01
6 ->	13	-0.02	0.01
6 ->	14	-0.06	0.06
6 ->	15	-0.00	0.00
6 ->	16	-0.00	0.00
6 ->	17	-0.01	0.00
6 ->	18	-0.01	0.00
6 ->	19	-0.01	0.00
6 ->	20	-0.46	0.28
6 ->	21	-0.02	0.01
6 ->	22	-0.01	0.00
6 ->	23	-0.17	0.13
6 ->	24	-1.22	0.29
6 ->	25	-0.01	0.01
6 ->	26	-0.01	0.00
6 ->	27	-0.23	0.12
6 ->	28	-0.03	0.03
6 ->	29	-0.00	0.00
6 ->	30	-0.00	0.00
6 ->	31	0.00	0.00
6 ->	32	-0.00	0.00
6 ->	33	-0.00	0.00
6 ->	34	0.00	0.00
6 ->	35	-0.00	0.00
6 ->	36	0.00	0.00
6 ->	37	-0.00	0.00
6 ->	38	-0.00	0.00
6 ->	39	-0.00	0.00
6 ->	40	0.00	0.00
6 ->	41	0.00	0.00
6 ->	42	-0.00	0.00
6 ->	43	-0.00	0.00
6 ->	44	0.00	0.00
6 ->	45	0.00	0.00
6 ->	46	-0.00	0.00
6 ->	47	0.00	0.00
6 ->	48	0.00	0.00
6 ->	49	0.00	0.00
6 ->	50	0.00	0.00
6 ->	51	0.00	0.00
6 ->	52	0.00	0.00
6 ->	53	0.00	0.00
6 ->	54	0.00	0.00
6 ->	55	0.00	0.00
6 ->	56	0.00	0.00
6 ->	57	0.00	0.00
6 ->	58	-0.00	0.00
6 ->	59	0.00	0.00
6 ->	60	0.00	0.00
6 ->	61	0.00	0.00

6 ->	62	-0.00	0.00
6 ->	63	-0.00	0.00
6 ->	64	-0.00	0.00
6 ->	65	0.00	0.00
6 ->	66	-0.00	0.00
6 ->	67	0.00	0.00
6 ->	68	0.00	0.00
6 ->	69	0.00	0.00
6 ->	70	0.00	0.00
6 ->	71	-0.00	0.00
6 ->	72	0.00	0.00
6 ->	73	0.00	0.00
6 ->	74	-0.00	0.00
6 ->	75	-0.00	0.00
6 ->	76	0.00	0.00
6 ->	77	0.00	0.00
6 ->	78	0.00	0.00
6 ->	79	0.00	0.00
6 ->	80	0.00	0.00
6 ->	81	0.00	0.00
6 ->	82	0.00	0.00
6 ->	83	0.00	0.00
6 ->	84	0.00	0.00
6 ->	85	0.00	0.00
6 ->	86	-0.00	0.00
6 ->	87	-0.00	0.00
6 ->	88	-0.00	0.00
6 ->	89	-0.00	0.00
6 ->	90	-0.00	0.00
6 ->	91	-0.00	0.00
6 ->	92	-0.00	0.00
6 ->	93	-0.00	0.00
6 ->	94	-0.00	0.00
6 ->	95	-0.00	0.00
6 ->	96	-0.00	0.00
6 ->	97	-0.00	0.00
6 ->	98	-0.00	0.00
6 ->	99	-0.00	0.00
6 ->	100	-0.00	0.00
6 ->	101	-0.00	0.00
6 ->	102	-0.00	0.00
6 ->	103	-0.00	0.00
6 ->	104	-0.00	0.00
6 ->	105	-0.00	0.00
6 ->	106	-0.00	0.00
6 ->	107	-0.00	0.00
7 ->	1	-0.00	0.00
7 ->	2	-0.00	0.00
7 ->	3	0.01	0.01
7 ->	4	0.05	0.08

7	->	5	-5.60	1.94
7	->	6	-13.17	0.46
7	->	7	25.70	1.52
7	->	8	-11.77	0.55
7	->	9	-1.25	0.47
7	->	10	-0.84	0.44
7	->	11	-0.03	0.01
7	->	12	-0.01	0.00
7	->	13	-0.01	0.01
7	->	14	-0.04	0.02
7	->	15	-0.00	0.00
7	->	16	-0.00	0.00
7	->	17	-0.00	0.00
7	->	18	-0.00	0.00
7	->	19	0.00	0.00
7	->	20	-0.02	0.01
7	->	21	-0.01	0.00
7	->	22	0.01	0.01
7	->	23	-0.25	0.11
7	->	24	-0.08	0.05
7	->	25	-0.00	0.00
7	->	26	-0.00	0.00
7	->	27	-0.95	0.92
7	->	28	-0.03	0.04
7	->	29	-0.00	0.00
7	->	30	-0.00	0.00
7	->	31	-0.00	0.00
7	->	32	-0.00	0.00
7	->	33	0.00	0.00
7	->	34	0.00	0.00
7	->	35	0.00	0.00
7	->	36	0.00	0.00
7	->	37	0.00	0.00
7	->	38	-0.00	0.00
7	->	39	-0.00	0.00
7	->	40	0.00	0.00
7	->	41	0.00	0.00
7	->	42	0.00	0.00
7	->	43	-0.00	0.00
7	->	44	-0.00	0.00
7	->	45	0.00	0.00
7	->	46	-0.00	0.00
7	->	47	-0.00	0.00
7	->	48	0.00	0.00
7	->	49	0.00	0.00
7	->	50	0.00	0.00
7	->	51	0.00	0.00
7	->	52	0.00	0.00
7	->	53	0.00	0.00
7	->	54	0.00	0.00

7 ->	55	0.00	0.00
7 ->	56	0.00	0.00
7 ->	57	0.00	0.00
7 ->	58	0.00	0.00
7 ->	59	0.00	0.00
7 ->	60	0.00	0.00
7 ->	61	0.00	0.00
7 ->	62	0.00	0.00
7 ->	63	-0.00	0.00
7 ->	64	0.00	0.00
7 ->	65	0.00	0.00
7 ->	66	0.00	0.00
7 ->	67	0.00	0.00
7 ->	68	-0.00	0.00
7 ->	69	0.00	0.00
7 ->	70	0.00	0.00
7 ->	71	0.00	0.00
7 ->	72	0.00	0.00
7 ->	73	0.00	0.00
7 ->	74	-0.00	0.00
7 ->	75	-0.00	0.00
7 ->	76	0.00	0.00
7 ->	77	0.00	0.00
7 ->	78	-0.00	0.00
7 ->	79	0.00	0.00
7 ->	80	0.00	0.00
7 ->	81	-0.00	0.00
7 ->	82	0.00	0.00
7 ->	83	0.00	0.00
7 ->	84	0.00	0.00
7 ->	85	-0.00	0.00
7 ->	86	0.00	0.00
7 ->	87	0.00	0.00
7 ->	88	0.00	0.00
7 ->	89	-0.00	0.00
7 ->	90	-0.00	0.00
7 ->	91	-0.01	0.01
7 ->	92	-0.01	0.01
7 ->	93	-0.00	0.00
7 ->	94	-0.00	0.00
7 ->	95	-0.00	0.00
7 ->	96	-0.00	0.00
7 ->	97	-0.00	0.00
7 ->	98	-0.00	0.00
7 ->	99	-0.00	0.00
7 ->	100	-0.00	0.00
7 ->	101	-0.00	0.00
7 ->	102	-0.00	0.00
7 ->	103	-0.00	0.00
7 ->	104	-0.00	0.00

7	->	105	-0.00	0.00
7	->	106	-0.00	0.00
7	->	107	0.00	0.00
8	->	1	-0.05	0.03
8	->	2	-0.00	0.00
8	->	3	-0.02	0.02
8	->	4	-0.23	0.36
8	->	5	-0.12	0.36
8	->	6	-0.43	0.16
8	->	7	-11.78	0.54
8	->	8	-69.66	2.86
8	->	9	-18.79	0.82
8	->	10	-0.66	0.23
8	->	11	-0.01	0.03
8	->	12	-0.05	0.02
8	->	13	-0.80	1.97
8	->	14	-0.01	0.01
8	->	15	-0.00	0.00
8	->	16	-0.01	0.00
8	->	17	0.00	0.00
8	->	18	0.01	0.00
8	->	19	0.02	0.00
8	->	20	-0.01	0.01
8	->	21	0.00	0.00
8	->	22	-0.02	0.00
8	->	23	-0.01	0.02
8	->	24	-0.01	0.00
8	->	25	-0.01	0.00
8	->	26	-0.00	0.00
8	->	27	-0.01	0.02
8	->	28	-0.00	0.00
8	->	29	-0.00	0.00
8	->	30	-0.00	0.00
8	->	31	-0.01	0.00
8	->	32	-0.00	0.00
8	->	33	0.00	0.00
8	->	34	-0.00	0.00
8	->	35	0.00	0.00
8	->	36	-0.00	0.00
8	->	37	0.00	0.00
8	->	38	0.00	0.00
8	->	39	0.00	0.00
8	->	40	-0.00	0.00
8	->	41	-0.00	0.00
8	->	42	-0.00	0.00
8	->	43	-0.00	0.00
8	->	44	-0.01	0.00
8	->	45	-0.00	0.00
8	->	46	0.00	0.00
8	->	47	-0.00	0.00

8	->	48	-0.00	0.00
8	->	49	-0.00	0.00
8	->	50	-0.00	0.00
8	->	51	-0.00	0.00
8	->	52	-0.00	0.00
8	->	53	-0.00	0.00
8	->	54	-0.00	0.00
8	->	55	0.00	0.00
8	->	56	-0.00	0.00
8	->	57	0.00	0.00
8	->	58	0.00	0.00
8	->	59	-0.00	0.00
8	->	60	-0.00	0.00
8	->	61	-0.00	0.00
8	->	62	0.00	0.00
8	->	63	0.00	0.00
8	->	64	0.00	0.00
8	->	65	0.00	0.00
8	->	66	0.00	0.00
8	->	67	0.00	0.00
8	->	68	-0.00	0.00
8	->	69	-0.00	0.00
8	->	70	-0.00	0.00
8	->	71	0.00	0.00
8	->	72	-0.00	0.00
8	->	73	-0.00	0.00
8	->	74	0.00	0.00
8	->	75	0.00	0.00
8	->	76	-0.00	0.00
8	->	77	-0.00	0.00
8	->	78	-0.00	0.00
8	->	79	-0.00	0.00
8	->	80	-0.00	0.00
8	->	81	-0.00	0.00
8	->	82	-0.00	0.00
8	->	83	-0.00	0.00
8	->	84	0.00	0.00
8	->	85	-0.00	0.00
8	->	86	0.00	0.00
8	->	87	0.00	0.00
8	->	88	0.00	0.00
8	->	89	0.00	0.00
8	->	90	0.01	0.00
8	->	91	0.01	0.00
8	->	92	0.02	0.00
8	->	93	0.02	0.00
8	->	94	0.01	0.00
8	->	95	0.01	0.00
8	->	96	0.00	0.00
8	->	97	0.00	0.00

8	->	98	0.01	0.00
8	->	99	0.00	0.00
8	->	100	0.00	0.00
8	->	101	0.00	0.00
8	->	102	0.00	0.00
8	->	103	0.00	0.00
8	->	104	0.01	0.00
8	->	105	0.01	0.00
8	->	106	0.01	0.00
8	->	107	0.01	0.00
9	->	1	-0.01	0.00
9	->	2	-0.00	0.00
9	->	3	-0.00	0.00
9	->	4	-0.02	0.02
9	->	5	-0.03	0.12
9	->	6	-0.04	0.01
9	->	7	-1.26	0.47
9	->	8	-18.89	0.84
9	->	9	14.93	1.36
9	->	10	-9.53	0.69
9	->	11	-0.37	0.18
9	->	12	-0.04	0.01
9	->	13	-0.04	0.06
9	->	14	-0.01	0.01
9	->	15	-0.00	0.00
9	->	16	-0.00	0.00
9	->	17	-0.00	0.00
9	->	18	0.00	0.00
9	->	19	-0.00	0.00
9	->	20	-0.00	0.00
9	->	21	-0.00	0.00
9	->	22	-0.00	0.00
9	->	23	-0.02	0.04
9	->	24	-0.00	0.00
9	->	25	-0.00	0.00
9	->	26	-0.01	0.01
9	->	27	-0.10	0.10
9	->	28	-0.00	0.00
9	->	29	-0.00	0.00
9	->	30	-0.00	0.00
9	->	31	0.00	0.00
9	->	32	0.00	0.00
9	->	33	-0.00	0.00
9	->	34	0.00	0.00
9	->	35	-0.00	0.00
9	->	36	0.00	0.00
9	->	37	-0.00	0.00
9	->	38	-0.00	0.00
9	->	39	-0.00	0.00
9	->	40	0.00	0.00

9	->	41	0.00	0.00
9	->	42	0.00	0.00
9	->	43	0.00	0.00
9	->	44	0.00	0.00
9	->	45	0.00	0.00
9	->	46	0.00	0.00
9	->	47	-0.00	0.00
9	->	48	0.00	0.00
9	->	49	0.00	0.00
9	->	50	0.00	0.00
9	->	51	0.00	0.00
9	->	52	0.00	0.00
9	->	53	0.00	0.00
9	->	54	0.00	0.00
9	->	55	0.00	0.00
9	->	56	0.00	0.00
9	->	57	0.00	0.00
9	->	58	0.00	0.00
9	->	59	0.00	0.00
9	->	60	0.00	0.00
9	->	61	0.00	0.00
9	->	62	0.00	0.00
9	->	63	0.00	0.00
9	->	64	0.00	0.00
9	->	65	0.00	0.00
9	->	66	0.00	0.00
9	->	67	0.00	0.00
9	->	68	-0.00	0.00
9	->	69	0.00	0.00
9	->	70	0.00	0.00
9	->	71	0.00	0.00
9	->	72	-0.00	0.00
9	->	73	0.00	0.00
9	->	74	0.00	0.00
9	->	75	0.00	0.00
9	->	76	-0.00	0.00
9	->	77	-0.00	0.00
9	->	78	-0.00	0.00
9	->	79	0.00	0.00
9	->	80	0.00	0.00
9	->	81	-0.00	0.00
9	->	82	0.00	0.00
9	->	83	0.00	0.00
9	->	84	0.00	0.00
9	->	85	-0.00	0.00
9	->	86	0.00	0.00
9	->	87	0.00	0.00
9	->	88	-0.00	0.00
9	->	89	-0.00	0.00
9	->	90	-0.00	0.00

9	->	91	-0.01	0.00
9	->	92	-0.00	0.00
9	->	93	-0.00	0.00
9	->	94	0.00	0.00
9	->	95	0.00	0.00
9	->	96	0.00	0.00
9	->	97	-0.00	0.00
9	->	98	-0.00	0.00
9	->	99	-0.00	0.00
9	->	100	-0.00	0.00
9	->	101	0.00	0.00
9	->	102	0.00	0.00
9	->	103	0.00	0.00
9	->	104	0.00	0.00
9	->	105	0.00	0.00
9	->	106	0.00	0.00
9	->	107	-0.00	0.00
10	->	1	-0.09	0.01
10	->	2	-0.00	0.00
10	->	3	-0.02	0.01
10	->	4	-0.14	0.06
10	->	5	1.40	0.31
10	->	6	-0.04	0.01
10	->	7	-0.84	0.44
10	->	8	-0.67	0.22
10	->	9	-9.60	0.69
10	->	10	-37.94	2.45
10	->	11	-14.14	0.36
10	->	12	-1.52	0.51
10	->	13	-0.45	0.50
10	->	14	-0.14	0.16
10	->	15	-0.00	0.00
10	->	16	-0.02	0.00
10	->	17	0.00	0.00
10	->	18	0.03	0.00
10	->	19	0.04	0.01
10	->	20	-0.01	0.01
10	->	21	-0.00	0.00
10	->	22	-0.09	0.03
10	->	23	2.13	0.93
10	->	24	0.03	0.01
10	->	25	-0.04	0.00
10	->	26	-0.05	0.01
10	->	27	1.38	0.23
10	->	28	-0.01	0.00
10	->	29	-0.00	0.00
10	->	30	-0.00	0.00
10	->	31	-0.02	0.01
10	->	32	-0.00	0.00
10	->	33	0.00	0.00

10 ->	34	-0.00	0.00
10 ->	35	0.01	0.00
10 ->	36	-0.00	0.00
10 ->	37	0.00	0.00
10 ->	38	0.01	0.00
10 ->	39	0.00	0.00
10 ->	40	-0.01	0.00
10 ->	41	-0.00	0.00
10 ->	42	-0.00	0.00
10 ->	43	-0.00	0.00
10 ->	44	-0.02	0.00
10 ->	45	-0.00	0.00
10 ->	46	0.01	0.00
10 ->	47	-0.00	0.00
10 ->	48	-0.00	0.00
10 ->	49	-0.00	0.00
10 ->	50	-0.00	0.00
10 ->	51	-0.00	0.00
10 ->	52	-0.00	0.00
10 ->	53	-0.01	0.00
10 ->	54	-0.00	0.00
10 ->	55	0.00	0.00
10 ->	56	-0.00	0.00
10 ->	57	-0.00	0.00
10 ->	58	0.00	0.00
10 ->	59	-0.00	0.00
10 ->	60	-0.00	0.00
10 ->	61	-0.00	0.00
10 ->	62	0.00	0.00
10 ->	63	0.00	0.00
10 ->	64	0.00	0.00
10 ->	65	0.00	0.00
10 ->	66	0.00	0.00
10 ->	67	0.00	0.00
10 ->	68	-0.00	0.00
10 ->	69	-0.00	0.00
10 ->	70	-0.00	0.00
10 ->	71	0.00	0.00
10 ->	72	-0.01	0.00
10 ->	73	-0.00	0.00
10 ->	74	0.01	0.00
10 ->	75	0.00	0.00
10 ->	76	-0.00	0.00
10 ->	77	-0.01	0.00
10 ->	78	-0.01	0.00
10 ->	79	-0.00	0.00
10 ->	80	-0.00	0.00
10 ->	81	-0.01	0.00
10 ->	82	-0.00	0.00
10 ->	83	-0.00	0.00

10	->	84	-0.00	0.00
10	->	85	-0.00	0.00
10	->	86	0.00	0.00
10	->	87	0.00	0.00
10	->	88	0.01	0.00
10	->	89	0.01	0.00
10	->	90	0.03	0.01
10	->	91	0.09	0.05
10	->	92	0.17	0.10
10	->	93	0.05	0.02
10	->	94	0.02	0.00
10	->	95	0.01	0.00
10	->	96	0.00	0.00
10	->	97	0.00	0.00
10	->	98	0.01	0.00
10	->	99	0.01	0.00
10	->	100	0.01	0.00
10	->	101	0.01	0.00
10	->	102	0.01	0.00
10	->	103	0.01	0.00
10	->	104	0.01	0.00
10	->	105	0.02	0.01
10	->	106	0.02	0.01
10	->	107	0.01	0.00
11	->	1	-0.16	0.06
11	->	2	-0.00	0.00
11	->	3	-0.01	0.00
11	->	4	-0.04	0.02
11	->	5	0.08	0.02
11	->	6	-0.00	0.00
11	->	7	-0.03	0.01
11	->	8	-0.01	0.04
11	->	9	-0.39	0.19
11	->	10	-14.24	0.35
11	->	11	-35.84	1.94
11	->	12	-9.03	1.01
11	->	13	-1.16	1.04
11	->	14	-0.04	0.02
11	->	15	-0.00	0.00
11	->	16	-0.02	0.00
11	->	17	0.00	0.00
11	->	18	0.02	0.00
11	->	19	0.04	0.01
11	->	20	-0.00	0.00
11	->	21	-0.00	0.00
11	->	22	-0.03	0.01
11	->	23	-0.00	0.03
11	->	24	-0.00	0.00
11	->	25	-0.02	0.00
11	->	26	-0.01	0.00

11	->	27	0.01	0.01
11	->	28	-0.00	0.00
11	->	29	-0.00	0.00
11	->	30	-0.00	0.00
11	->	31	-0.01	0.00
11	->	32	-0.00	0.00
11	->	33	0.00	0.00
11	->	34	-0.00	0.00
11	->	35	0.00	0.00
11	->	36	-0.00	0.00
11	->	37	0.00	0.00
11	->	38	0.00	0.00
11	->	39	0.00	0.00
11	->	40	-0.01	0.00
11	->	41	-0.00	0.00
11	->	42	-0.00	0.00
11	->	43	-0.00	0.00
11	->	44	-0.01	0.00
11	->	45	-0.00	0.00
11	->	46	0.01	0.00
11	->	47	-0.00	0.00
11	->	48	-0.00	0.00
11	->	49	-0.00	0.00
11	->	50	-0.00	0.00
11	->	51	-0.00	0.00
11	->	52	-0.00	0.00
11	->	53	-0.00	0.00
11	->	54	-0.00	0.00
11	->	55	-0.00	0.00
11	->	56	-0.00	0.00
11	->	57	-0.00	0.00
11	->	58	0.00	0.00
11	->	59	-0.00	0.00
11	->	60	-0.00	0.00
11	->	61	-0.00	0.00
11	->	62	0.00	0.00
11	->	63	0.00	0.00
11	->	64	0.00	0.00
11	->	65	0.00	0.00
11	->	66	0.00	0.00
11	->	67	-0.00	0.00
11	->	68	-0.00	0.00
11	->	69	-0.00	0.00
11	->	70	-0.00	0.00
11	->	71	0.00	0.00
11	->	72	-0.01	0.00
11	->	73	-0.00	0.00
11	->	74	0.01	0.00
11	->	75	0.00	0.00
11	->	76	-0.00	0.00

11	->	77	-0.01	0.00
11	->	78	-0.01	0.00
11	->	79	-0.00	0.00
11	->	80	-0.00	0.00
11	->	81	-0.01	0.00
11	->	82	-0.00	0.00
11	->	83	-0.00	0.00
11	->	84	0.00	0.00
11	->	85	-0.00	0.00
11	->	86	0.00	0.00
11	->	87	0.00	0.00
11	->	88	0.00	0.00
11	->	89	0.00	0.00
11	->	90	0.01	0.00
11	->	91	0.02	0.01
11	->	92	0.03	0.02
11	->	93	0.04	0.02
11	->	94	0.02	0.00
11	->	95	0.01	0.00
11	->	96	0.00	0.00
11	->	97	0.00	0.00
11	->	98	0.01	0.00
11	->	99	0.00	0.00
11	->	100	0.00	0.00
11	->	101	0.01	0.00
11	->	102	0.01	0.00
11	->	103	0.01	0.00
11	->	104	0.02	0.01
11	->	105	0.03	0.01
11	->	106	0.02	0.01
11	->	107	0.01	0.00
12	->	1	1.89	1.60
12	->	2	-0.01	0.01
12	->	3	-0.22	0.07
12	->	4	-0.20	0.20
12	->	5	-0.33	0.31
12	->	6	-0.01	0.01
12	->	7	-0.01	0.00
12	->	8	-0.05	0.02
12	->	9	-0.04	0.01
12	->	10	-1.53	0.52
12	->	11	-9.17	1.04
12	->	12	-102.57	6.76
12	->	13	-10.06	1.73
12	->	14	-2.73	1.47
12	->	15	-0.16	0.12
12	->	16	0.03	0.02
12	->	17	-0.01	0.01
12	->	18	-0.04	0.01
12	->	19	-2.30	4.23

12	->	20	-0.10	0.07
12	->	21	-0.00	0.01
12	->	22	0.79	1.15
12	->	23	-0.53	0.45
12	->	24	-0.03	0.02
12	->	25	0.03	0.02
12	->	26	-0.03	0.03
12	->	27	-0.07	0.05
12	->	28	0.00	0.00
12	->	29	0.00	0.00
12	->	30	-0.00	0.00
12	->	31	0.02	0.01
12	->	32	0.00	0.00
12	->	33	-0.00	0.00
12	->	34	0.00	0.00
12	->	35	-0.01	0.00
12	->	36	0.00	0.00
12	->	37	-0.00	0.00
12	->	38	-0.01	0.00
12	->	39	-0.00	0.00
12	->	40	0.01	0.00
12	->	41	0.00	0.00
12	->	42	0.00	0.00
12	->	43	-0.00	0.01
12	->	44	0.06	0.03
12	->	45	-0.00	0.00
12	->	46	-0.03	0.01
12	->	47	0.00	0.00
12	->	48	0.00	0.00
12	->	49	0.00	0.00
12	->	50	0.00	0.00
12	->	51	0.00	0.00
12	->	52	0.00	0.00
12	->	53	0.01	0.00
12	->	54	0.00	0.00
12	->	55	0.00	0.00
12	->	56	0.00	0.00
12	->	57	0.00	0.00
12	->	58	-0.00	0.00
12	->	59	0.00	0.00
12	->	60	0.00	0.00
12	->	61	0.00	0.00
12	->	62	-0.00	0.00
12	->	63	-0.00	0.00
12	->	64	-0.00	0.00
12	->	65	-0.00	0.00
12	->	66	-0.00	0.00
12	->	67	0.00	0.00
12	->	68	0.00	0.00
12	->	69	0.00	0.00

12	->	70	0.00	0.00
12	->	71	-0.00	0.00
12	->	72	0.02	0.00
12	->	73	0.00	0.00
12	->	74	-0.02	0.00
12	->	75	-0.01	0.00
12	->	76	0.01	0.00
12	->	77	0.02	0.00
12	->	78	0.02	0.00
12	->	79	0.00	0.00
12	->	80	0.00	0.00
12	->	81	0.02	0.01
12	->	82	0.00	0.00
12	->	83	0.00	0.00
12	->	84	0.00	0.00
12	->	85	0.00	0.00
12	->	86	-0.00	0.00
12	->	87	-0.01	0.00
12	->	88	-0.01	0.00
12	->	89	-0.01	0.00
12	->	90	-0.05	0.02
12	->	91	-1.01	0.87
12	->	92	-8.42	4.95
12	->	93	-7.93	3.51
12	->	94	-0.08	0.04
12	->	95	-0.03	0.01
12	->	96	-0.01	0.00
12	->	97	-0.00	0.00
12	->	98	-0.02	0.01
12	->	99	-0.01	0.00
12	->	100	-0.01	0.01
12	->	101	-0.02	0.00
12	->	102	-0.03	0.01
12	->	103	-0.05	0.01
12	->	104	-0.06	0.03
12	->	105	-0.07	0.03
12	->	106	-0.04	0.01
12	->	107	-0.02	0.01
13	->	1	-3.54	1.58
13	->	2	-1.48	1.01
13	->	3	-3.17	0.65
13	->	4	-1.94	0.47
13	->	5	-0.38	0.11
13	->	6	-0.02	0.01
13	->	7	-0.01	0.01
13	->	8	-0.80	1.99
13	->	9	-0.04	0.06
13	->	10	-0.46	0.51
13	->	11	-1.20	1.04
13	->	12	-10.12	1.71

13 ->	13	-107.37	3.05
13 ->	14	-12.56	0.56
13 ->	15	-0.61	0.11
13 ->	16	-0.01	0.02
13 ->	17	-0.03	0.01
13 ->	18	-0.03	0.01
13 ->	19	-0.26	0.18
13 ->	20	-0.07	0.02
13 ->	21	-0.01	0.01
13 ->	22	0.07	0.03
13 ->	23	-0.05	0.02
13 ->	24	-0.01	0.00
13 ->	25	0.02	0.00
13 ->	26	-0.00	0.00
13 ->	27	-0.02	0.00
13 ->	28	0.00	0.00
13 ->	29	0.00	0.00
13 ->	30	0.00	0.00
13 ->	31	0.01	0.00
13 ->	32	0.00	0.00
13 ->	33	0.00	0.00
13 ->	34	0.00	0.00
13 ->	35	-0.00	0.00
13 ->	36	0.00	0.00
13 ->	37	-0.00	0.00
13 ->	38	-0.00	0.00
13 ->	39	-0.00	0.00
13 ->	40	0.00	0.00
13 ->	41	0.00	0.00
13 ->	42	0.00	0.00
13 ->	43	-0.00	0.00
13 ->	44	0.02	0.00
13 ->	45	0.00	0.00
13 ->	46	-0.01	0.00
13 ->	47	0.00	0.00
13 ->	48	0.00	0.00
13 ->	49	0.00	0.00
13 ->	50	0.00	0.00
13 ->	51	0.00	0.00
13 ->	52	0.00	0.00
13 ->	53	0.00	0.00
13 ->	54	0.00	0.00
13 ->	55	-0.00	0.00
13 ->	56	0.00	0.00
13 ->	57	0.00	0.00
13 ->	58	-0.00	0.00
13 ->	59	0.00	0.00
13 ->	60	0.00	0.00
13 ->	61	0.00	0.00
13 ->	62	-0.00	0.00

13	->	63	-0.00	0.00
13	->	64	-0.00	0.00
13	->	65	-0.00	0.00
13	->	66	-0.00	0.00
13	->	67	0.00	0.00
13	->	68	0.00	0.00
13	->	69	0.00	0.00
13	->	70	0.00	0.00
13	->	71	-0.00	0.00
13	->	72	0.00	0.00
13	->	73	0.00	0.00
13	->	74	-0.00	0.00
13	->	75	-0.00	0.00
13	->	76	0.00	0.00
13	->	77	0.01	0.00
13	->	78	0.00	0.00
13	->	79	0.00	0.00
13	->	80	0.00	0.00
13	->	81	0.00	0.00
13	->	82	0.00	0.00
13	->	83	0.00	0.00
13	->	84	0.00	0.00
13	->	85	0.00	0.00
13	->	86	-0.00	0.00
13	->	87	-0.00	0.00
13	->	88	-0.00	0.00
13	->	89	-0.00	0.00
13	->	90	-0.01	0.00
13	->	91	-0.04	0.02
13	->	92	-0.12	0.07
13	->	93	-0.15	0.42
13	->	94	-0.07	0.02
13	->	95	-0.02	0.00
13	->	96	-0.01	0.00
13	->	97	-0.00	0.00
13	->	98	-0.01	0.00
13	->	99	-0.01	0.00
13	->	100	-0.01	0.00
13	->	101	-0.01	0.00
13	->	102	-0.01	0.00
13	->	103	-0.01	0.00
13	->	104	-0.01	0.00
13	->	105	-0.01	0.00
13	->	106	-0.01	0.00
13	->	107	-0.00	0.00
14	->	1	-2.84	1.06
14	->	2	-1.04	0.20
14	->	3	-3.93	0.51
14	->	4	-0.88	0.44
14	->	5	-0.60	0.33

14	->	6	-0.06	0.06
14	->	7	-0.04	0.02
14	->	8	-0.01	0.01
14	->	9	-0.01	0.01
14	->	10	-0.14	0.16
14	->	11	-0.04	0.02
14	->	12	-2.71	1.45
14	->	13	-12.61	0.56
14	->	14	30.03	1.13
14	->	15	-16.90	0.42
14	->	16	-0.72	0.14
14	->	17	-0.16	0.03
14	->	18	-0.12	0.03
14	->	19	-4.09	1.70
14	->	20	-2.52	0.57
14	->	21	-0.06	0.02
14	->	22	-0.50	0.31
14	->	23	-2.16	0.37
14	->	24	-0.07	0.04
14	->	25	-0.02	0.00
14	->	26	-0.04	0.01
14	->	27	-0.01	0.01
14	->	28	-0.00	0.00
14	->	29	-0.00	0.00
14	->	30	-0.00	0.00
14	->	31	0.00	0.00
14	->	32	-0.00	0.00
14	->	33	-0.00	0.00
14	->	34	0.00	0.00
14	->	35	-0.00	0.00
14	->	36	0.00	0.00
14	->	37	-0.00	0.00
14	->	38	-0.00	0.00
14	->	39	0.00	0.00
14	->	40	0.00	0.00
14	->	41	-0.00	0.00
14	->	42	-0.00	0.00
14	->	43	-0.00	0.00
14	->	44	-0.01	0.01
14	->	45	-0.00	0.00
14	->	46	-0.00	0.00
14	->	47	-0.00	0.00
14	->	48	0.00	0.00
14	->	49	0.00	0.00
14	->	50	0.00	0.00
14	->	51	0.00	0.00
14	->	52	0.00	0.00
14	->	53	0.00	0.00
14	->	54	0.00	0.00
14	->	55	0.00	0.00

14	->	56	0.00	0.00
14	->	57	0.00	0.00
14	->	58	-0.00	0.00
14	->	59	0.00	0.00
14	->	60	0.00	0.00
14	->	61	0.00	0.00
14	->	62	-0.00	0.00
14	->	63	-0.00	0.00
14	->	64	-0.00	0.00
14	->	65	0.00	0.00
14	->	66	-0.00	0.00
14	->	67	-0.00	0.00
14	->	68	0.00	0.00
14	->	69	0.00	0.00
14	->	70	0.00	0.00
14	->	71	0.00	0.00
14	->	72	0.00	0.00
14	->	73	0.00	0.00
14	->	74	-0.00	0.00
14	->	75	-0.00	0.00
14	->	76	0.00	0.00
14	->	77	0.00	0.00
14	->	78	0.00	0.00
14	->	79	0.00	0.00
14	->	80	0.00	0.00
14	->	81	0.00	0.00
14	->	82	0.00	0.00
14	->	83	0.00	0.00
14	->	84	0.00	0.00
14	->	85	0.00	0.00
14	->	86	-0.00	0.00
14	->	87	-0.00	0.00
14	->	88	-0.00	0.00
14	->	89	-0.00	0.00
14	->	90	-0.00	0.00
14	->	91	-0.01	0.03
14	->	92	-0.71	0.81
14	->	93	-0.56	0.49
14	->	94	-0.20	0.09
14	->	95	-0.01	0.00
14	->	96	-0.00	0.00
14	->	97	-0.01	0.00
14	->	98	-0.01	0.00
14	->	99	-0.00	0.00
14	->	100	-0.00	0.00
14	->	101	-0.00	0.00
14	->	102	-0.00	0.00
14	->	103	-0.00	0.00
14	->	104	-0.00	0.00
14	->	105	-0.00	0.00

14	->	106	-0.00	0.00
14	->	107	-0.00	0.00
15	->	1	-4.18	1.74
15	->	2	-0.94	0.27
15	->	3	-1.18	0.42
15	->	4	-0.00	0.01
15	->	5	0.01	0.00
15	->	6	-0.00	0.00
15	->	7	-0.00	0.00
15	->	8	-0.00	0.00
15	->	9	-0.00	0.00
15	->	10	-0.00	0.00
15	->	11	-0.00	0.00
15	->	12	-0.16	0.12
15	->	13	-0.61	0.11
15	->	14	-16.92	0.42
15	->	15	22.54	2.69
15	->	16	-6.18	0.72
15	->	17	-0.29	0.05
15	->	18	-0.14	0.08
15	->	19	-3.89	2.91
15	->	20	-0.07	0.03
15	->	21	-0.00	0.00
15	->	22	-0.01	0.04
15	->	23	-0.01	0.00
15	->	24	-0.00	0.00
15	->	25	-0.00	0.00
15	->	26	-0.00	0.00
15	->	27	0.00	0.00
15	->	28	0.00	0.00
15	->	29	0.00	0.00
15	->	30	-0.00	0.00
15	->	31	0.00	0.00
15	->	32	0.00	0.00
15	->	33	0.00	0.00
15	->	34	0.00	0.00
15	->	35	-0.00	0.00
15	->	36	0.00	0.00
15	->	37	0.00	0.00
15	->	38	-0.00	0.00
15	->	39	-0.00	0.00
15	->	40	0.00	0.00
15	->	41	0.00	0.00
15	->	42	0.00	0.00
15	->	43	-0.00	0.00
15	->	44	0.00	0.01
15	->	45	0.00	0.00
15	->	46	-0.00	0.00
15	->	47	-0.00	0.00
15	->	48	0.00	0.00

15	->	49	0.00	0.00
15	->	50	0.00	0.00
15	->	51	0.00	0.00
15	->	52	0.00	0.00
15	->	53	0.00	0.00
15	->	54	0.00	0.00
15	->	55	0.00	0.00
15	->	56	0.00	0.00
15	->	57	0.00	0.00
15	->	58	-0.00	0.00
15	->	59	0.00	0.00
15	->	60	0.00	0.00
15	->	61	0.00	0.00
15	->	62	0.00	0.00
15	->	63	-0.00	0.00
15	->	64	-0.00	0.00
15	->	65	0.00	0.00
15	->	66	-0.00	0.00
15	->	67	-0.00	0.00
15	->	68	0.00	0.00
15	->	69	0.00	0.00
15	->	70	0.00	0.00
15	->	71	-0.00	0.00
15	->	72	0.00	0.00
15	->	73	0.00	0.00
15	->	74	-0.00	0.00
15	->	75	-0.00	0.00
15	->	76	0.00	0.00
15	->	77	0.00	0.00
15	->	78	0.00	0.00
15	->	79	0.00	0.00
15	->	80	0.00	0.00
15	->	81	0.00	0.00
15	->	82	0.00	0.00
15	->	83	0.00	0.00
15	->	84	0.00	0.00
15	->	85	0.00	0.00
15	->	86	-0.00	0.00
15	->	87	-0.00	0.00
15	->	88	-0.00	0.00
15	->	89	-0.00	0.00
15	->	90	-0.00	0.00
15	->	91	-0.00	0.01
15	->	92	0.01	0.02
15	->	93	-0.58	0.58
15	->	94	-2.58	1.88
15	->	95	-0.04	0.02
15	->	96	-0.01	0.01
15	->	97	-0.01	0.01
15	->	98	-0.00	0.00

15	->	99	-0.00	0.00
15	->	100	-0.00	0.00
15	->	101	-0.00	0.00
15	->	102	-0.00	0.00
15	->	103	-0.00	0.00
15	->	104	-0.00	0.00
15	->	105	-0.00	0.00
15	->	106	-0.00	0.00
15	->	107	-0.00	0.00
16	->	1	-0.03	0.07
16	->	2	-0.16	0.12
16	->	3	-1.80	0.82
16	->	4	-0.02	0.01
16	->	5	-0.03	0.00
16	->	6	-0.00	0.00
16	->	7	-0.00	0.00
16	->	8	-0.01	0.00
16	->	9	-0.00	0.00
16	->	10	-0.02	0.00
16	->	11	-0.02	0.00
16	->	12	0.03	0.02
16	->	13	-0.01	0.02
16	->	14	-0.72	0.14
16	->	15	-6.30	0.73
16	->	16	-97.11	3.18
16	->	17	-21.64	0.46
16	->	18	-11.11	5.07
16	->	19	-4.16	1.93
16	->	20	-1.52	0.42
16	->	21	-0.08	0.06
16	->	22	0.17	0.12
16	->	23	-0.02	0.01
16	->	24	-0.00	0.00
16	->	25	0.04	0.01
16	->	26	-0.00	0.00
16	->	27	-0.00	0.00
16	->	28	0.00	0.00
16	->	29	0.00	0.00
16	->	30	-0.00	0.00
16	->	31	0.01	0.00
16	->	32	0.00	0.00
16	->	33	-0.00	0.00
16	->	34	0.00	0.00
16	->	35	-0.01	0.00
16	->	36	0.01	0.00
16	->	37	-0.00	0.00
16	->	38	-0.01	0.00
16	->	39	-0.00	0.00
16	->	40	0.01	0.00
16	->	41	0.00	0.00

16	->	42	0.00	0.00
16	->	43	-0.00	0.00
16	->	44	0.25	0.22
16	->	45	-0.00	0.00
16	->	46	-0.06	0.02
16	->	47	-0.00	0.00
16	->	48	0.00	0.00
16	->	49	0.00	0.00
16	->	50	0.00	0.00
16	->	51	0.00	0.00
16	->	52	0.00	0.00
16	->	53	0.02	0.00
16	->	54	0.00	0.00
16	->	55	0.00	0.00
16	->	56	0.00	0.00
16	->	57	0.00	0.00
16	->	58	-0.01	0.00
16	->	59	0.01	0.00
16	->	60	0.00	0.00
16	->	61	0.00	0.00
16	->	62	-0.00	0.00
16	->	63	-0.00	0.00
16	->	64	-0.00	0.00
16	->	65	-0.00	0.00
16	->	66	-0.00	0.00
16	->	67	-0.00	0.00
16	->	68	0.00	0.00
16	->	69	0.01	0.00
16	->	70	-0.00	0.00
16	->	71	-0.00	0.00
16	->	72	0.02	0.00
16	->	73	0.00	0.00
16	->	74	-0.01	0.00
16	->	75	-0.01	0.00
16	->	76	0.01	0.01
16	->	77	0.02	0.00
16	->	78	0.01	0.00
16	->	79	0.00	0.00
16	->	80	0.00	0.00
16	->	81	0.01	0.00
16	->	82	0.00	0.00
16	->	83	0.00	0.00
16	->	84	0.00	0.00
16	->	85	0.00	0.00
16	->	86	-0.00	0.00
16	->	87	-0.00	0.00
16	->	88	-0.01	0.00
16	->	89	-0.01	0.00
16	->	90	-0.02	0.00
16	->	91	-0.04	0.02

16	->	92	-0.07	0.02
16	->	93	-0.23	0.08
16	->	94	-2.87	0.93
16	->	95	-8.50	0.89
16	->	96	-1.80	0.77
16	->	97	-0.38	0.45
16	->	98	-0.34	1.16
16	->	99	0.41	0.31
16	->	100	0.27	0.16
16	->	101	-0.07	0.03
16	->	102	-0.05	0.01
16	->	103	-0.03	0.00
16	->	104	-0.02	0.00
16	->	105	-0.01	0.00
16	->	106	-0.01	0.00
16	->	107	-0.00	0.00
17	->	1	-0.05	0.03
17	->	2	-0.03	0.02
17	->	3	-1.36	0.82
17	->	4	-0.01	0.00
17	->	5	-0.00	0.00
17	->	6	-0.01	0.00
17	->	7	-0.00	0.00
17	->	8	0.00	0.00
17	->	9	-0.00	0.00
17	->	10	0.00	0.00
17	->	11	0.00	0.00
17	->	12	-0.01	0.01
17	->	13	-0.03	0.01
17	->	14	-0.16	0.03
17	->	15	-0.29	0.05
17	->	16	-21.65	0.46
17	->	17	19.76	1.57
17	->	18	-10.58	0.57
17	->	19	-0.83	0.17
17	->	20	-2.43	0.28
17	->	21	-3.81	1.26
17	->	22	-0.17	0.11
17	->	23	-0.07	0.04
17	->	24	-0.07	0.03
17	->	25	-0.03	0.02
17	->	26	-0.00	0.00
17	->	27	-0.00	0.00
17	->	28	-0.00	0.00
17	->	29	-0.00	0.00
17	->	30	-0.00	0.00
17	->	31	-0.00	0.00
17	->	32	-0.00	0.00
17	->	33	-0.00	0.00
17	->	34	-0.00	0.00

17 ->	35	0.00	0.00
17 ->	36	-0.00	0.00
17 ->	37	0.00	0.00
17 ->	38	0.00	0.00
17 ->	39	0.00	0.00
17 ->	40	-0.00	0.00
17 ->	41	-0.00	0.00
17 ->	42	-0.00	0.00
17 ->	43	-0.00	0.00
17 ->	44	-0.00	0.02
17 ->	45	-0.00	0.00
17 ->	46	-0.00	0.00
17 ->	47	-0.00	0.00
17 ->	48	-0.00	0.00
17 ->	49	-0.00	0.00
17 ->	50	-0.00	0.00
17 ->	51	-0.00	0.00
17 ->	52	-0.00	0.00
17 ->	53	-0.00	0.00
17 ->	54	-0.00	0.00
17 ->	55	0.00	0.00
17 ->	56	-0.00	0.00
17 ->	57	0.00	0.00
17 ->	58	0.00	0.00
17 ->	59	-0.00	0.00
17 ->	60	-0.00	0.00
17 ->	61	-0.00	0.00
17 ->	62	0.00	0.00
17 ->	63	0.00	0.00
17 ->	64	0.00	0.00
17 ->	65	0.00	0.00
17 ->	66	0.00	0.00
17 ->	67	0.00	0.00
17 ->	68	-0.00	0.00
17 ->	69	-0.00	0.00
17 ->	70	0.00	0.00
17 ->	71	-0.00	0.00
17 ->	72	-0.00	0.00
17 ->	73	-0.00	0.00
17 ->	74	0.00	0.00
17 ->	75	0.00	0.00
17 ->	76	-0.00	0.00
17 ->	77	-0.00	0.00
17 ->	78	-0.00	0.00
17 ->	79	-0.00	0.00
17 ->	80	0.00	0.00
17 ->	81	-0.00	0.00
17 ->	82	-0.00	0.00
17 ->	83	0.00	0.00
17 ->	84	0.00	0.00

17	->	85	-0.00	0.00
17	->	86	0.00	0.00
17	->	87	0.00	0.00
17	->	88	0.00	0.00
17	->	89	0.00	0.00
17	->	90	0.00	0.00
17	->	91	0.00	0.01
17	->	92	0.00	0.01
17	->	93	-0.01	0.01
17	->	94	-0.01	0.01
17	->	95	-0.01	0.00
17	->	96	-0.01	0.00
17	->	97	-0.99	1.25
17	->	98	-0.10	0.05
17	->	99	-0.01	0.01
17	->	100	-0.00	0.00
17	->	101	-0.00	0.00
17	->	102	-0.00	0.00
17	->	103	-0.00	0.00
17	->	104	0.00	0.00
17	->	105	0.00	0.00
17	->	106	0.00	0.00
17	->	107	0.00	0.00
18	->	1	-0.12	0.05
18	->	2	-0.01	0.00
18	->	3	-0.02	0.02
18	->	4	0.00	0.00
18	->	5	0.03	0.00
18	->	6	-0.01	0.00
18	->	7	-0.00	0.00
18	->	8	0.01	0.00
18	->	9	0.00	0.00
18	->	10	0.03	0.00
18	->	11	0.02	0.00
18	->	12	-0.04	0.01
18	->	13	-0.03	0.01
18	->	14	-0.12	0.03
18	->	15	-0.14	0.08
18	->	16	-11.05	5.05
18	->	17	-10.62	0.57
18	->	18	-14.17	4.06
18	->	19	-13.58	0.76
18	->	20	-1.14	0.21
18	->	21	-2.60	0.81
18	->	22	-2.81	0.92
18	->	23	-0.09	0.06
18	->	24	-0.05	0.03
18	->	25	-0.29	0.17
18	->	26	-0.01	0.00
18	->	27	-0.01	0.00

18 ->	28	-0.01	0.00
18 ->	29	-0.00	0.00
18 ->	30	0.00	0.00
18 ->	31	-0.03	0.01
18 ->	32	-0.01	0.00
18 ->	33	-0.01	0.01
18 ->	34	-0.00	0.00
18 ->	35	0.01	0.00
18 ->	36	-0.01	0.00
18 ->	37	0.00	0.00
18 ->	38	0.01	0.00
18 ->	39	0.00	0.00
18 ->	40	-0.01	0.00
18 ->	41	-0.00	0.00
18 ->	42	-0.00	0.00
18 ->	43	-0.01	0.01
18 ->	44	-4.08	5.45
18 ->	45	-0.01	0.01
18 ->	46	0.06	0.02
18 ->	47	-0.01	0.01
18 ->	48	-0.00	0.00
18 ->	49	-0.00	0.00
18 ->	50	-0.00	0.00
18 ->	51	-0.00	0.00
18 ->	52	-0.00	0.00
18 ->	53	-0.02	0.00
18 ->	54	-0.00	0.00
18 ->	55	-0.00	0.00
18 ->	56	-0.00	0.00
18 ->	57	-0.00	0.00
18 ->	58	0.01	0.00
18 ->	59	-0.01	0.00
18 ->	60	-0.00	0.00
18 ->	61	-0.00	0.00
18 ->	62	0.00	0.00
18 ->	63	0.00	0.00
18 ->	64	0.00	0.00
18 ->	65	0.00	0.00
18 ->	66	0.00	0.00
18 ->	67	0.00	0.00
18 ->	68	-0.00	0.00
18 ->	69	-0.00	0.00
18 ->	70	-0.00	0.00
18 ->	71	0.00	0.00
18 ->	72	-0.02	0.00
18 ->	73	-0.00	0.00
18 ->	74	0.01	0.00
18 ->	75	0.01	0.00
18 ->	76	-0.01	0.00
18 ->	77	-0.01	0.00

18	->	78	-0.01	0.00
18	->	79	-0.00	0.00
18	->	80	-0.00	0.00
18	->	81	-0.01	0.00
18	->	82	-0.00	0.00
18	->	83	-0.00	0.00
18	->	84	-0.00	0.00
18	->	85	-0.00	0.00
18	->	86	0.00	0.00
18	->	87	0.01	0.00
18	->	88	0.01	0.00
18	->	89	0.01	0.00
18	->	90	0.02	0.00
18	->	91	0.05	0.05
18	->	92	0.04	0.04
18	->	93	0.03	0.08
18	->	94	-2.30	2.22
18	->	95	0.45	0.24
18	->	96	0.07	0.09
18	->	97	-1.28	1.30
18	->	98	-2.79	1.51
18	->	99	-1.65	1.76
18	->	100	0.02	0.10
18	->	101	0.01	0.01
18	->	102	0.02	0.00
18	->	103	0.02	0.00
18	->	104	0.01	0.00
18	->	105	0.01	0.00
18	->	106	0.01	0.00
18	->	107	0.00	0.00
19	->	1	-5.94	5.73
19	->	2	-0.02	0.01
19	->	3	-0.12	0.05
19	->	4	-0.01	0.01
19	->	5	0.01	0.01
19	->	6	-0.01	0.00
19	->	7	0.00	0.00
19	->	8	0.02	0.00
19	->	9	-0.00	0.00
19	->	10	0.04	0.01
19	->	11	0.04	0.01
19	->	12	-2.30	4.22
19	->	13	-0.26	0.18
19	->	14	-4.10	1.70
19	->	15	-3.86	2.89
19	->	16	-4.15	1.92
19	->	17	-0.84	0.17
19	->	18	-13.65	0.77
19	->	19	-36.01	5.23
19	->	20	-8.38	0.42

19 ->	21	-1.00	0.21
19 ->	22	-7.56	5.04
19 ->	23	-2.80	1.07
19 ->	24	-0.10	0.07
19 ->	25	-0.15	0.05
19 ->	26	-0.04	0.02
19 ->	27	-0.02	0.01
19 ->	28	-0.01	0.00
19 ->	29	-0.00	0.00
19 ->	30	0.00	0.00
19 ->	31	-0.02	0.00
19 ->	32	-0.00	0.00
19 ->	33	-0.00	0.00
19 ->	34	-0.00	0.00
19 ->	35	0.01	0.00
19 ->	36	-0.01	0.00
19 ->	37	0.00	0.00
19 ->	38	0.01	0.00
19 ->	39	0.00	0.00
19 ->	40	-0.01	0.00
19 ->	41	-0.00	0.00
19 ->	42	-0.00	0.00
19 ->	43	-0.01	0.01
19 ->	44	-0.58	0.65
19 ->	45	-0.00	0.00
19 ->	46	0.05	0.03
19 ->	47	-0.01	0.01
19 ->	48	-0.00	0.00
19 ->	49	-0.00	0.00
19 ->	50	-0.00	0.00
19 ->	51	-0.00	0.00
19 ->	52	-0.00	0.00
19 ->	53	-0.02	0.00
19 ->	54	-0.00	0.00
19 ->	55	-0.00	0.00
19 ->	56	-0.00	0.00
19 ->	57	-0.00	0.00
19 ->	58	0.01	0.00
19 ->	59	-0.01	0.00
19 ->	60	-0.00	0.00
19 ->	61	-0.00	0.00
19 ->	62	0.00	0.00
19 ->	63	0.00	0.00
19 ->	64	0.00	0.00
19 ->	65	0.00	0.00
19 ->	66	0.00	0.00
19 ->	67	0.00	0.00
19 ->	68	-0.00	0.00
19 ->	69	-0.00	0.00
19 ->	70	-0.00	0.00

19	->	71	0.00	0.00
19	->	72	-0.02	0.00
19	->	73	-0.00	0.00
19	->	74	0.02	0.00
19	->	75	0.01	0.00
19	->	76	-0.01	0.01
19	->	77	-0.02	0.00
19	->	78	-0.01	0.00
19	->	79	-0.00	0.00
19	->	80	-0.00	0.00
19	->	81	-0.01	0.00
19	->	82	-0.00	0.00
19	->	83	-0.00	0.00
19	->	84	-0.00	0.00
19	->	85	-0.00	0.00
19	->	86	0.00	0.00
19	->	87	0.01	0.00
19	->	88	0.01	0.00
19	->	89	0.01	0.00
19	->	90	0.02	0.00
19	->	91	0.11	0.14
19	->	92	0.42	0.53
19	->	93	-2.00	2.01
19	->	94	-1.15	1.62
19	->	95	0.07	0.04
19	->	96	0.01	0.01
19	->	97	-0.04	0.05
19	->	98	0.00	0.03
19	->	99	-0.04	0.04
19	->	100	-0.01	0.05
19	->	101	0.01	0.02
19	->	102	0.03	0.01
19	->	103	0.03	0.01
19	->	104	0.02	0.01
19	->	105	0.02	0.01
19	->	106	0.02	0.00
19	->	107	0.01	0.00
20	->	1	-0.08	0.05
20	->	2	-0.04	0.01
20	->	3	-1.56	0.45
20	->	4	-0.95	0.32
20	->	5	-0.86	0.29
20	->	6	-0.45	0.27
20	->	7	-0.02	0.01
20	->	8	-0.01	0.01
20	->	9	-0.00	0.00
20	->	10	-0.01	0.01
20	->	11	-0.00	0.00
20	->	12	-0.10	0.07
20	->	13	-0.07	0.02

20 ->	14	-2.57	0.58
20 ->	15	-0.07	0.03
20 ->	16	-1.54	0.42
20 ->	17	-2.44	0.28
20 ->	18	-1.14	0.21
20 ->	19	-8.42	0.42
20 ->	20	22.05	1.31
20 ->	21	-6.16	0.50
20 ->	22	-1.52	0.26
20 ->	23	-2.97	0.48
20 ->	24	-3.90	0.76
20 ->	25	-0.16	0.08
20 ->	26	-0.06	0.02
20 ->	27	-0.06	0.03
20 ->	28	-0.02	0.01
20 ->	29	-0.00	0.00
20 ->	30	-0.01	0.00
20 ->	31	-0.00	0.00
20 ->	32	-0.00	0.00
20 ->	33	-0.00	0.00
20 ->	34	-0.00	0.00
20 ->	35	0.00	0.00
20 ->	36	-0.00	0.00
20 ->	37	0.00	0.00
20 ->	38	0.00	0.00
20 ->	39	0.00	0.00
20 ->	40	-0.00	0.00
20 ->	41	0.00	0.00
20 ->	42	-0.00	0.00
20 ->	43	-0.01	0.01
20 ->	44	-0.01	0.02
20 ->	45	-0.00	0.00
20 ->	46	-0.00	0.00
20 ->	47	-0.00	0.00
20 ->	48	-0.00	0.00
20 ->	49	0.00	0.00
20 ->	50	-0.00	0.00
20 ->	51	-0.00	0.00
20 ->	52	-0.00	0.00
20 ->	53	-0.00	0.00
20 ->	54	0.00	0.00
20 ->	55	0.00	0.00
20 ->	56	-0.00	0.00
20 ->	57	0.00	0.00
20 ->	58	0.00	0.00
20 ->	59	-0.00	0.00
20 ->	60	0.00	0.00
20 ->	61	0.00	0.00
20 ->	62	0.00	0.00
20 ->	63	0.00	0.00

20	->	64	0.00	0.00
20	->	65	0.00	0.00
20	->	66	0.00	0.00
20	->	67	0.00	0.00
20	->	68	-0.00	0.00
20	->	69	0.00	0.00
20	->	70	0.00	0.00
20	->	71	0.00	0.00
20	->	72	-0.00	0.00
20	->	73	0.00	0.00
20	->	74	0.00	0.00
20	->	75	0.00	0.00
20	->	76	0.00	0.00
20	->	77	-0.00	0.00
20	->	78	-0.00	0.00
20	->	79	0.00	0.00
20	->	80	0.00	0.00
20	->	81	-0.00	0.00
20	->	82	0.00	0.00
20	->	83	0.00	0.00
20	->	84	0.00	0.00
20	->	85	-0.00	0.00
20	->	86	0.00	0.00
20	->	87	0.00	0.00
20	->	88	0.00	0.00
20	->	89	-0.00	0.00
20	->	90	-0.00	0.00
20	->	91	0.02	0.03
20	->	92	0.08	0.07
20	->	93	0.01	0.03
20	->	94	-0.02	0.01
20	->	95	-0.00	0.00
20	->	96	-0.00	0.00
20	->	97	-0.06	0.08
20	->	98	-0.05	0.04
20	->	99	-0.01	0.00
20	->	100	-0.01	0.00
20	->	101	-0.00	0.00
20	->	102	-0.00	0.00
20	->	103	-0.00	0.00
20	->	104	-0.00	0.00
20	->	105	0.00	0.00
20	->	106	0.00	0.00
20	->	107	0.00	0.00
21	->	1	0.00	0.01
21	->	2	-0.00	0.00
21	->	3	-0.03	0.01
21	->	4	-0.01	0.01
21	->	5	-0.00	0.01
21	->	6	-0.02	0.01

21	->	7	-0.01	0.00
21	->	8	0.00	0.00
21	->	9	-0.00	0.00
21	->	10	-0.00	0.00
21	->	11	-0.00	0.00
21	->	12	-0.00	0.01
21	->	13	-0.01	0.01
21	->	14	-0.06	0.02
21	->	15	-0.00	0.00
21	->	16	-0.08	0.06
21	->	17	-3.82	1.26
21	->	18	-2.62	0.82
21	->	19	-1.01	0.21
21	->	20	-6.18	0.50
21	->	21	-19.54	1.97
21	->	22	-10.60	0.56
21	->	23	-0.94	0.19
21	->	24	-2.93	0.46
21	->	25	-3.61	0.76
21	->	26	-0.10	0.05
21	->	27	-0.07	0.03
21	->	28	-0.03	0.03
21	->	29	-0.01	0.00
21	->	30	-0.07	0.04
21	->	31	-0.00	0.00
21	->	32	-0.01	0.01
21	->	33	-0.02	0.01
21	->	34	-0.00	0.00
21	->	35	0.00	0.00
21	->	36	-0.00	0.00
21	->	37	0.00	0.00
21	->	38	0.00	0.00
21	->	39	0.00	0.00
21	->	40	-0.00	0.00
21	->	41	0.00	0.00
21	->	42	0.00	0.00
21	->	43	-0.04	0.03
21	->	44	0.08	0.11
21	->	45	-0.01	0.00
21	->	46	-0.01	0.00
21	->	47	-0.01	0.00
21	->	48	-0.00	0.00
21	->	49	-0.00	0.00
21	->	50	-0.00	0.00
21	->	51	-0.00	0.00
21	->	52	-0.00	0.00
21	->	53	0.00	0.00
21	->	54	-0.00	0.00
21	->	55	0.00	0.00
21	->	56	0.00	0.00

21	->	57	0.00	0.00
21	->	58	-0.00	0.00
21	->	59	0.00	0.00
21	->	60	-0.00	0.00
21	->	61	0.00	0.00
21	->	62	0.00	0.00
21	->	63	-0.00	0.00
21	->	64	-0.00	0.00
21	->	65	0.00	0.00
21	->	66	0.00	0.00
21	->	67	0.00	0.00
21	->	68	0.00	0.00
21	->	69	0.00	0.00
21	->	70	0.00	0.00
21	->	71	-0.00	0.00
21	->	72	0.00	0.00
21	->	73	0.00	0.00
21	->	74	-0.00	0.00
21	->	75	-0.00	0.00
21	->	76	0.00	0.00
21	->	77	0.00	0.00
21	->	78	-0.00	0.00
21	->	79	-0.00	0.00
21	->	80	0.00	0.00
21	->	81	0.00	0.00
21	->	82	0.00	0.00
21	->	83	0.00	0.00
21	->	84	0.00	0.00
21	->	85	0.00	0.00
21	->	86	0.00	0.00
21	->	87	0.00	0.00
21	->	88	0.00	0.00
21	->	89	-0.00	0.00
21	->	90	-0.00	0.00
21	->	91	-0.04	0.03
21	->	92	-0.10	0.07
21	->	93	-0.03	0.01
21	->	94	-0.00	0.01
21	->	95	-0.01	0.00
21	->	96	-0.00	0.00
21	->	97	-0.93	1.27
21	->	98	-0.48	0.62
21	->	99	-0.01	0.01
21	->	100	-0.01	0.00
21	->	101	-0.00	0.00
21	->	102	-0.00	0.00
21	->	103	-0.00	0.00
21	->	104	-0.00	0.00
21	->	105	-0.00	0.00
21	->	106	0.00	0.00

21 ->	107	0.00	0.00
22 ->	1	0.34	0.41
22 ->	2	-0.00	0.00
22 ->	3	-0.01	0.01
22 ->	4	0.01	0.01
22 ->	5	-0.15	0.04
22 ->	6	-0.01	0.00
22 ->	7	0.01	0.01
22 ->	8	-0.02	0.00
22 ->	9	-0.00	0.00
22 ->	10	-0.09	0.03
22 ->	11	-0.03	0.01
22 ->	12	0.79	1.15
22 ->	13	0.07	0.03
22 ->	14	-0.50	0.31
22 ->	15	-0.01	0.04
22 ->	16	0.17	0.12
22 ->	17	-0.17	0.11
22 ->	18	-2.85	0.92
22 ->	19	-7.60	5.05
22 ->	20	-1.53	0.26
22 ->	21	-10.62	0.56
22 ->	22	-94.87	5.06
22 ->	23	-19.17	0.65
22 ->	24	-0.77	0.19
22 ->	25	-2.61	0.57
22 ->	26	-2.16	0.69
22 ->	27	-0.15	0.07
22 ->	28	-0.02	0.02
22 ->	29	-0.00	0.01
22 ->	30	-0.04	0.02
22 ->	31	0.03	0.02
22 ->	32	-0.02	0.01
22 ->	33	-0.04	0.02
22 ->	34	-0.00	0.00
22 ->	35	-0.02	0.00
22 ->	36	0.01	0.00
22 ->	37	-0.00	0.00
22 ->	38	-0.01	0.00
22 ->	39	-0.00	0.00
22 ->	40	0.02	0.00
22 ->	41	0.01	0.01
22 ->	42	0.03	0.02
22 ->	43	-0.70	0.67
22 ->	44	-0.45	0.94
22 ->	45	-0.06	0.03
22 ->	46	-0.29	0.24
22 ->	47	-0.27	0.19
22 ->	48	-0.01	0.01
22 ->	49	0.00	0.00

22	->	50	0.00	0.00
22	->	51	0.00	0.00
22	->	52	0.00	0.00
22	->	53	0.02	0.00
22	->	54	0.00	0.00
22	->	55	0.00	0.00
22	->	56	0.00	0.00
22	->	57	0.00	0.00
22	->	58	-0.01	0.00
22	->	59	0.01	0.00
22	->	60	0.00	0.00
22	->	61	0.00	0.00
22	->	62	-0.00	0.00
22	->	63	-0.01	0.00
22	->	64	-0.00	0.00
22	->	65	-0.00	0.00
22	->	66	-0.00	0.00
22	->	67	-0.00	0.00
22	->	68	0.01	0.00
22	->	69	0.01	0.00
22	->	70	0.00	0.00
22	->	71	-0.00	0.00
22	->	72	0.03	0.00
22	->	73	0.00	0.00
22	->	74	-0.03	0.01
22	->	75	-0.01	0.00
22	->	76	0.02	0.00
22	->	77	0.02	0.00
22	->	78	0.02	0.00
22	->	79	0.00	0.00
22	->	80	0.00	0.00
22	->	81	0.01	0.00
22	->	82	0.00	0.00
22	->	83	0.00	0.00
22	->	84	0.00	0.00
22	->	85	0.00	0.00
22	->	86	-0.00	0.00
22	->	87	-0.01	0.00
22	->	88	-0.02	0.00
22	->	89	-0.03	0.01
22	->	90	-0.14	0.08
22	->	91	-3.41	2.78
22	->	92	-11.13	6.42
22	->	93	-5.51	2.79
22	->	94	-0.18	0.40
22	->	95	-0.11	0.08
22	->	96	-0.03	0.01
22	->	97	-0.04	0.03
22	->	98	-0.20	0.17
22	->	99	-0.17	0.37

22	->	100	-1.21	1.96
22	->	101	0.09	0.17
22	->	102	-0.06	0.03
22	->	103	-0.08	0.03
22	->	104	-0.05	0.02
22	->	105	-0.04	0.01
22	->	106	-0.02	0.00
22	->	107	-0.01	0.00
23	->	1	0.00	0.01
23	->	2	-0.00	0.00
23	->	3	-0.03	0.01
23	->	4	-0.13	0.05
23	->	5	1.83	0.97
23	->	6	-0.17	0.13
23	->	7	-0.25	0.11
23	->	8	-0.01	0.02
23	->	9	-0.02	0.04
23	->	10	2.07	0.93
23	->	11	-0.00	0.03
23	->	12	-0.51	0.43
23	->	13	-0.05	0.02
23	->	14	-2.19	0.36
23	->	15	-0.01	0.00
23	->	16	-0.02	0.01
23	->	17	-0.07	0.04
23	->	18	-0.09	0.06
23	->	19	-2.83	1.07
23	->	20	-2.97	0.48
23	->	21	-0.95	0.19
23	->	22	-19.21	0.65
23	->	23	11.67	1.54
23	->	24	-10.09	0.63
23	->	25	-1.22	0.24
23	->	26	-3.29	0.48
23	->	27	24.83	13.88
23	->	28	-0.24	0.07
23	->	29	-0.03	0.02
23	->	30	-0.03	0.02
23	->	31	-0.01	0.00
23	->	32	-0.01	0.00
23	->	33	-0.00	0.00
23	->	34	-0.00	0.00
23	->	35	-0.00	0.00
23	->	36	0.00	0.00
23	->	37	-0.00	0.00
23	->	38	-0.00	0.00
23	->	39	0.00	0.00
23	->	40	-0.00	0.00
23	->	41	-0.00	0.00
23	->	42	-0.01	0.00

23	->	43	-0.01	0.01
23	->	44	-0.05	0.03
23	->	45	0.00	0.00
23	->	46	-0.00	0.00
23	->	47	-0.00	0.00
23	->	48	-0.00	0.00
23	->	49	0.00	0.00
23	->	50	0.00	0.00
23	->	51	0.00	0.00
23	->	52	0.00	0.00
23	->	53	0.00	0.00
23	->	54	0.00	0.00
23	->	55	0.00	0.00
23	->	56	0.00	0.00
23	->	57	0.00	0.00
23	->	58	-0.00	0.00
23	->	59	0.00	0.00
23	->	60	0.00	0.00
23	->	61	0.00	0.00
23	->	62	0.00	0.00
23	->	63	-0.00	0.00
23	->	64	-0.00	0.00
23	->	65	0.00	0.00
23	->	66	-0.00	0.00
23	->	67	0.00	0.00
23	->	68	0.00	0.00
23	->	69	0.00	0.00
23	->	70	0.00	0.00
23	->	71	-0.00	0.00
23	->	72	0.00	0.00
23	->	73	0.00	0.00
23	->	74	-0.00	0.00
23	->	75	-0.00	0.00
23	->	76	0.00	0.00
23	->	77	0.00	0.00
23	->	78	0.00	0.00
23	->	79	0.00	0.00
23	->	80	0.00	0.00
23	->	81	0.00	0.00
23	->	82	0.00	0.00
23	->	83	0.00	0.00
23	->	84	0.00	0.00
23	->	85	0.00	0.00
23	->	86	-0.00	0.00
23	->	87	-0.00	0.00
23	->	88	-0.00	0.00
23	->	89	-0.00	0.00
23	->	90	-0.02	0.02
23	->	91	-0.66	0.58
23	->	92	-3.89	3.77

23	->	93	-0.06	0.03
23	->	94	-0.01	0.00
23	->	95	-0.01	0.00
23	->	96	-0.00	0.00
23	->	97	-0.01	0.01
23	->	98	-0.01	0.01
23	->	99	-0.00	0.00
23	->	100	-0.01	0.00
23	->	101	-0.00	0.00
23	->	102	-0.00	0.00
23	->	103	-0.00	0.00
23	->	104	-0.01	0.00
23	->	105	-0.01	0.00
23	->	106	-0.00	0.00
23	->	107	-0.00	0.00
24	->	1	-0.00	0.00
24	->	2	-0.00	0.00
24	->	3	-0.02	0.01
24	->	4	-0.05	0.03
24	->	5	-0.37	0.37
24	->	6	-1.22	0.30
24	->	7	-0.08	0.05
24	->	8	-0.01	0.00
24	->	9	-0.00	0.00
24	->	10	0.03	0.01
24	->	11	-0.00	0.00
24	->	12	-0.03	0.02
24	->	13	-0.01	0.00
24	->	14	-0.07	0.04
24	->	15	-0.00	0.00
24	->	16	-0.00	0.00
24	->	17	-0.07	0.03
24	->	18	-0.05	0.03
24	->	19	-0.11	0.07
24	->	20	-3.89	0.75
24	->	21	-2.95	0.46
24	->	22	-0.77	0.19
24	->	23	-10.14	0.63
24	->	24	23.91	1.65
24	->	25	-10.18	0.44
24	->	26	-1.11	0.15
24	->	27	-2.91	0.52
24	->	28	-5.52	1.03
24	->	29	-0.15	0.07
24	->	30	-0.58	0.28
24	->	31	-0.01	0.01
24	->	32	-0.01	0.00
24	->	33	-0.01	0.00
24	->	34	-0.00	0.00
24	->	35	0.00	0.00

24 ->	36	-0.00	0.00
24 ->	37	0.00	0.00
24 ->	38	0.00	0.00
24 ->	39	0.00	0.00
24 ->	40	-0.00	0.00
24 ->	41	0.00	0.00
24 ->	42	-0.00	0.00
24 ->	43	-0.02	0.02
24 ->	44	-0.00	0.01
24 ->	45	-0.00	0.00
24 ->	46	-0.00	0.00
24 ->	47	-0.00	0.00
24 ->	48	-0.00	0.00
24 ->	49	0.00	0.00
24 ->	50	0.00	0.00
24 ->	51	0.00	0.00
24 ->	52	0.00	0.00
24 ->	53	0.00	0.00
24 ->	54	0.00	0.00
24 ->	55	0.00	0.00
24 ->	56	0.00	0.00
24 ->	57	0.00	0.00
24 ->	58	-0.00	0.00
24 ->	59	0.00	0.00
24 ->	60	0.00	0.00
24 ->	61	0.00	0.00
24 ->	62	0.00	0.00
24 ->	63	-0.00	0.00
24 ->	64	-0.00	0.00
24 ->	65	0.00	0.00
24 ->	66	-0.00	0.00
24 ->	67	0.00	0.00
24 ->	68	-0.00	0.00
24 ->	69	0.00	0.00
24 ->	70	0.00	0.00
24 ->	71	0.00	0.00
24 ->	72	-0.00	0.00
24 ->	73	0.00	0.00
24 ->	74	0.00	0.00
24 ->	75	0.00	0.00
24 ->	76	0.00	0.00
24 ->	77	0.00	0.00
24 ->	78	-0.00	0.00
24 ->	79	0.00	0.00
24 ->	80	0.00	0.00
24 ->	81	0.00	0.00
24 ->	82	-0.00	0.00
24 ->	83	0.00	0.00
24 ->	84	0.00	0.00
24 ->	85	0.00	0.00

24	->	86	0.00	0.00
24	->	87	0.00	0.00
24	->	88	0.00	0.00
24	->	89	-0.00	0.00
24	->	90	-0.00	0.00
24	->	91	0.01	0.03
24	->	92	0.06	0.06
24	->	93	-0.00	0.00
24	->	94	-0.00	0.00
24	->	95	-0.00	0.00
24	->	96	-0.00	0.00
24	->	97	-0.02	0.02
24	->	98	-0.03	0.04
24	->	99	-0.00	0.00
24	->	100	-0.00	0.00
24	->	101	-0.00	0.00
24	->	102	-0.00	0.00
24	->	103	-0.00	0.00
24	->	104	-0.00	0.00
24	->	105	-0.00	0.00
24	->	106	0.00	0.00
24	->	107	0.00	0.00
25	->	1	0.04	0.01
25	->	2	-0.00	0.00
25	->	3	-0.00	0.00
25	->	4	-0.00	0.00
25	->	5	-0.05	0.01
25	->	6	-0.01	0.01
25	->	7	-0.00	0.00
25	->	8	-0.01	0.00
25	->	9	-0.00	0.00
25	->	10	-0.04	0.00
25	->	11	-0.02	0.00
25	->	12	0.03	0.02
25	->	13	0.02	0.00
25	->	14	-0.02	0.00
25	->	15	-0.00	0.00
25	->	16	0.04	0.01
25	->	17	-0.03	0.02
25	->	18	-0.29	0.17
25	->	19	-0.15	0.05
25	->	20	-0.16	0.08
25	->	21	-3.63	0.75
25	->	22	-2.62	0.56
25	->	23	-1.22	0.24
25	->	24	-10.21	0.44
25	->	25	-103.93	3.14
25	->	26	-12.80	0.47
25	->	27	-0.62	0.23
25	->	28	-1.49	0.38

25	->	29	-1.23	0.47
25	->	30	-5.26	1.14
25	->	31	0.02	0.09
25	->	32	-1.00	0.50
25	->	33	-0.55	0.38
25	->	34	-0.01	0.01
25	->	35	-0.03	0.00
25	->	36	0.02	0.00
25	->	37	-0.00	0.00
25	->	38	-0.02	0.00
25	->	39	-0.00	0.00
25	->	40	0.02	0.01
25	->	41	0.02	0.02
25	->	42	0.01	0.04
25	->	43	-2.69	2.33
25	->	44	-0.25	0.36
25	->	45	-0.11	0.08
25	->	46	-0.09	0.04
25	->	47	-0.05	0.03
25	->	48	-0.01	0.00
25	->	49	0.00	0.00
25	->	50	0.00	0.00
25	->	51	0.00	0.00
25	->	52	0.00	0.00
25	->	53	0.02	0.00
25	->	54	0.00	0.00
25	->	55	-0.00	0.00
25	->	56	0.00	0.00
25	->	57	0.00	0.00
25	->	58	-0.01	0.00
25	->	59	0.01	0.00
25	->	60	0.00	0.00
25	->	61	0.00	0.00
25	->	62	-0.00	0.00
25	->	63	-0.00	0.00
25	->	64	-0.00	0.00
25	->	65	-0.00	0.00
25	->	66	-0.00	0.00
25	->	67	-0.00	0.00
25	->	68	0.00	0.00
25	->	69	0.00	0.00
25	->	70	0.00	0.00
25	->	71	-0.00	0.00
25	->	72	0.02	0.00
25	->	73	0.00	0.00
25	->	74	-0.01	0.00
25	->	75	-0.01	0.00
25	->	76	0.01	0.00
25	->	77	0.01	0.00
25	->	78	0.01	0.00

25	->	79	0.00	0.00
25	->	80	0.00	0.00
25	->	81	0.00	0.00
25	->	82	0.00	0.00
25	->	83	0.00	0.00
25	->	84	0.00	0.00
25	->	85	0.00	0.00
25	->	86	-0.00	0.00
25	->	87	-0.01	0.00
25	->	88	-0.01	0.00
25	->	89	-0.03	0.00
25	->	90	-0.12	0.03
25	->	91	-0.35	0.13
25	->	92	-0.21	0.10
25	->	93	-0.05	0.01
25	->	94	-0.02	0.00
25	->	95	-0.02	0.00
25	->	96	-0.01	0.00
25	->	97	-0.38	0.47
25	->	98	-3.27	4.00
25	->	99	-0.21	0.20
25	->	100	-0.06	0.01
25	->	101	-0.02	0.00
25	->	102	-0.01	0.00
25	->	103	-0.01	0.00
25	->	104	-0.01	0.00
25	->	105	-0.01	0.00
25	->	106	-0.01	0.00
25	->	107	-0.01	0.00
26	->	1	0.00	0.00
26	->	2	-0.00	0.00
26	->	3	-0.00	0.00
26	->	4	-0.00	0.00
26	->	5	-0.05	0.01
26	->	6	-0.01	0.00
26	->	7	-0.00	0.00
26	->	8	-0.00	0.00
26	->	9	-0.01	0.01
26	->	10	-0.05	0.01
26	->	11	-0.01	0.00
26	->	12	-0.03	0.03
26	->	13	-0.00	0.00
26	->	14	-0.04	0.01
26	->	15	-0.00	0.00
26	->	16	-0.00	0.00
26	->	17	-0.00	0.00
26	->	18	-0.01	0.00
26	->	19	-0.04	0.02
26	->	20	-0.06	0.02
26	->	21	-0.10	0.05

26	->	22	-2.19	0.68
26	->	23	-3.29	0.49
26	->	24	-1.11	0.15
26	->	25	-12.84	0.47
26	->	26	18.29	1.37
26	->	27	-21.86	0.55
26	->	28	-0.64	0.15
26	->	29	-0.99	0.39
26	->	30	-0.45	0.18
26	->	31	-0.19	0.23
26	->	32	-0.03	0.02
26	->	33	-0.01	0.00
26	->	34	-0.00	0.00
26	->	35	-0.00	0.00
26	->	36	0.00	0.00
26	->	37	-0.00	0.00
26	->	38	0.00	0.00
26	->	39	-0.00	0.00
26	->	40	-0.01	0.00
26	->	41	-0.03	0.04
26	->	42	-0.03	0.01
26	->	43	-0.50	0.34
26	->	44	-0.06	0.03
26	->	45	0.00	0.00
26	->	46	-0.00	0.00
26	->	47	-0.03	0.04
26	->	48	-0.00	0.00
26	->	49	-0.00	0.00
26	->	50	-0.00	0.00
26	->	51	-0.00	0.00
26	->	52	0.00	0.00
26	->	53	0.00	0.00
26	->	54	0.00	0.00
26	->	55	0.00	0.00
26	->	56	0.00	0.00
26	->	57	0.00	0.00
26	->	58	-0.00	0.00
26	->	59	0.00	0.00
26	->	60	0.00	0.00
26	->	61	0.00	0.00
26	->	62	-0.00	0.00
26	->	63	-0.00	0.00
26	->	64	-0.00	0.00
26	->	65	0.00	0.00
26	->	66	0.00	0.00
26	->	67	0.00	0.00
26	->	68	0.00	0.00
26	->	69	0.00	0.00
26	->	70	0.00	0.00
26	->	71	-0.00	0.00

26	->	72	0.00	0.00
26	->	73	0.00	0.00
26	->	74	-0.00	0.00
26	->	75	-0.00	0.00
26	->	76	0.00	0.00
26	->	77	0.00	0.00
26	->	78	0.00	0.00
26	->	79	0.00	0.00
26	->	80	0.00	0.00
26	->	81	0.00	0.00
26	->	82	0.00	0.00
26	->	83	0.00	0.00
26	->	84	0.00	0.00
26	->	85	0.00	0.00
26	->	86	-0.00	0.00
26	->	87	-0.00	0.00
26	->	88	-0.00	0.00
26	->	89	-0.01	0.00
26	->	90	-0.21	0.14
26	->	91	-3.11	1.17
26	->	92	-0.71	0.38
26	->	93	-0.02	0.01
26	->	94	-0.00	0.00
26	->	95	-0.00	0.00
26	->	96	-0.00	0.00
26	->	97	-0.00	0.00
26	->	98	-0.00	0.00
26	->	99	-0.00	0.00
26	->	100	-0.01	0.00
26	->	101	-0.00	0.00
26	->	102	-0.00	0.00
26	->	103	-0.00	0.00
26	->	104	-0.00	0.00
26	->	105	-0.00	0.00
26	->	106	-0.00	0.00
26	->	107	-0.00	0.00
27	->	1	-0.01	0.00
27	->	2	-0.00	0.00
27	->	3	-0.01	0.00
27	->	4	-0.05	0.02
27	->	5	1.68	0.52
27	->	6	-0.22	0.12
27	->	7	-0.95	0.91
27	->	8	-0.01	0.02
27	->	9	-0.11	0.11
27	->	10	1.33	0.23
27	->	11	0.01	0.01
27	->	12	-0.07	0.05
27	->	13	-0.02	0.00
27	->	14	-0.01	0.01

27	->	15	0.00	0.00
27	->	16	-0.00	0.00
27	->	17	-0.00	0.00
27	->	18	-0.01	0.00
27	->	19	-0.02	0.01
27	->	20	-0.06	0.03
27	->	21	-0.07	0.03
27	->	22	-0.15	0.07
27	->	23	24.86	13.88
27	->	24	-2.90	0.52
27	->	25	-0.63	0.23
27	->	26	-21.94	0.56
27	->	27	2.94	1.63
27	->	28	-4.87	0.38
27	->	29	-0.55	0.11
27	->	30	-0.04	0.03
27	->	31	-0.03	0.01
27	->	32	-0.00	0.00
27	->	33	-0.00	0.00
27	->	34	-0.00	0.00
27	->	35	-0.00	0.00
27	->	36	0.00	0.00
27	->	37	-0.00	0.00
27	->	38	-0.00	0.00
27	->	39	-0.00	0.00
27	->	40	-0.00	0.00
27	->	41	-0.01	0.00
27	->	42	-0.01	0.00
27	->	43	-0.01	0.01
27	->	44	-0.00	0.00
27	->	45	0.00	0.00
27	->	46	-0.00	0.00
27	->	47	-0.00	0.00
27	->	48	0.00	0.00
27	->	49	0.00	0.00
27	->	50	0.00	0.00
27	->	51	0.00	0.00
27	->	52	0.00	0.00
27	->	53	0.00	0.00
27	->	54	0.00	0.00
27	->	55	-0.00	0.00
27	->	56	0.00	0.00
27	->	57	0.00	0.00
27	->	58	-0.00	0.00
27	->	59	0.00	0.00
27	->	60	0.00	0.00
27	->	61	0.00	0.00
27	->	62	0.00	0.00
27	->	63	-0.00	0.00
27	->	64	-0.00	0.00

27	->	65	0.00	0.00
27	->	66	-0.00	0.00
27	->	67	0.00	0.00
27	->	68	0.00	0.00
27	->	69	0.00	0.00
27	->	70	0.00	0.00
27	->	71	0.00	0.00
27	->	72	0.00	0.00
27	->	73	0.00	0.00
27	->	74	-0.00	0.00
27	->	75	-0.00	0.00
27	->	76	0.00	0.00
27	->	77	0.00	0.00
27	->	78	0.00	0.00
27	->	79	0.00	0.00
27	->	80	0.00	0.00
27	->	81	0.00	0.00
27	->	82	0.00	0.00
27	->	83	0.00	0.00
27	->	84	0.00	0.00
27	->	85	-0.00	0.00
27	->	86	-0.00	0.00
27	->	87	-0.00	0.00
27	->	88	-0.00	0.00
27	->	89	-0.00	0.00
27	->	90	0.01	0.01
27	->	91	0.03	0.04
27	->	92	0.06	0.09
27	->	93	-0.00	0.00
27	->	94	-0.00	0.00
27	->	95	-0.00	0.00
27	->	96	-0.00	0.00
27	->	97	-0.00	0.00
27	->	98	-0.00	0.00
27	->	99	-0.00	0.00
27	->	100	-0.00	0.00
27	->	101	-0.00	0.00
27	->	102	-0.00	0.00
27	->	103	-0.00	0.00
27	->	104	-0.00	0.00
27	->	105	-0.00	0.00
27	->	106	-0.00	0.00
27	->	107	-0.00	0.00
28	->	1	0.00	0.00
28	->	2	-0.00	0.00
28	->	3	-0.00	0.00
28	->	4	-0.00	0.00
28	->	5	-0.02	0.01
28	->	6	-0.03	0.03
28	->	7	-0.03	0.04

28	->	8	-0.00	0.00
28	->	9	-0.00	0.00
28	->	10	-0.01	0.00
28	->	11	-0.00	0.00
28	->	12	0.00	0.00
28	->	13	0.00	0.00
28	->	14	-0.00	0.00
28	->	15	0.00	0.00
28	->	16	0.00	0.00
28	->	17	-0.00	0.00
28	->	18	-0.01	0.00
28	->	19	-0.01	0.00
28	->	20	-0.02	0.01
28	->	21	-0.03	0.03
28	->	22	-0.02	0.02
28	->	23	-0.24	0.07
28	->	24	-5.53	1.03
28	->	25	-1.51	0.39
28	->	26	-0.65	0.15
28	->	27	-5.03	0.38
28	->	28	-22.33	1.57
28	->	29	-11.57	0.36
28	->	30	-3.69	1.22
28	->	31	-0.05	0.02
28	->	32	-0.01	0.00
28	->	33	-0.00	0.00
28	->	34	-0.00	0.00
28	->	35	0.00	0.00
28	->	36	-0.00	0.00
28	->	37	0.00	0.00
28	->	38	0.00	0.00
28	->	39	-0.00	0.00
28	->	40	-0.00	0.00
28	->	41	-0.00	0.00
28	->	42	-0.00	0.00
28	->	43	-0.01	0.01
28	->	44	-0.00	0.00
28	->	45	-0.00	0.00
28	->	46	-0.00	0.00
28	->	47	-0.00	0.00
28	->	48	-0.00	0.00
28	->	49	0.00	0.00
28	->	50	0.00	0.00
28	->	51	0.00	0.00
28	->	52	0.00	0.00
28	->	53	0.00	0.00
28	->	54	0.00	0.00
28	->	55	0.00	0.00
28	->	56	0.00	0.00
28	->	57	0.00	0.00

28	->	58	-0.00	0.00
28	->	59	0.00	0.00
28	->	60	0.00	0.00
28	->	61	0.00	0.00
28	->	62	0.00	0.00
28	->	63	-0.00	0.00
28	->	64	-0.00	0.00
28	->	65	0.00	0.00
28	->	66	-0.00	0.00
28	->	67	0.00	0.00
28	->	68	0.00	0.00
28	->	69	0.00	0.00
28	->	70	0.00	0.00
28	->	71	-0.00	0.00
28	->	72	0.00	0.00
28	->	73	0.00	0.00
28	->	74	-0.00	0.00
28	->	75	-0.00	0.00
28	->	76	0.00	0.00
28	->	77	0.00	0.00
28	->	78	0.00	0.00
28	->	79	0.00	0.00
28	->	80	0.00	0.00
28	->	81	0.00	0.00
28	->	82	0.00	0.00
28	->	83	0.00	0.00
28	->	84	0.00	0.00
28	->	85	0.00	0.00
28	->	86	-0.00	0.00
28	->	87	-0.00	0.00
28	->	88	0.00	0.00
28	->	89	-0.00	0.00
28	->	90	-0.01	0.00
28	->	91	-0.02	0.01
28	->	92	-0.01	0.00
28	->	93	-0.00	0.00
28	->	94	-0.00	0.00
28	->	95	-0.00	0.00
28	->	96	-0.00	0.00
28	->	97	-0.00	0.00
28	->	98	-0.00	0.00
28	->	99	-0.00	0.00
28	->	100	-0.00	0.00
28	->	101	-0.00	0.00
28	->	102	-0.00	0.00
28	->	103	-0.00	0.00
28	->	104	-0.00	0.00
28	->	105	-0.00	0.00
28	->	106	-0.00	0.00
28	->	107	-0.00	0.00

29	->	1	0.00	0.00
29	->	2	-0.00	0.00
29	->	3	0.00	0.00
29	->	4	-0.00	0.00
29	->	5	-0.00	0.00
29	->	6	-0.00	0.00
29	->	7	-0.00	0.00
29	->	8	-0.00	0.00
29	->	9	-0.00	0.00
29	->	10	-0.00	0.00
29	->	11	-0.00	0.00
29	->	12	0.00	0.00
29	->	13	0.00	0.00
29	->	14	-0.00	0.00
29	->	15	0.00	0.00
29	->	16	0.00	0.00
29	->	17	-0.00	0.00
29	->	18	-0.00	0.00
29	->	19	-0.00	0.00
29	->	20	-0.00	0.00
29	->	21	-0.01	0.00
29	->	22	-0.00	0.01
29	->	23	-0.03	0.02
29	->	24	-0.15	0.07
29	->	25	-1.24	0.47
29	->	26	-1.03	0.39
29	->	27	-0.56	0.11
29	->	28	-11.64	0.36
29	->	29	12.23	0.69
29	->	30	-14.31	0.53
29	->	31	-1.17	0.51
29	->	32	-0.03	0.01
29	->	33	-0.01	0.00
29	->	34	-0.00	0.00
29	->	35	0.00	0.00
29	->	36	-0.00	0.00
29	->	37	0.00	0.00
29	->	38	0.00	0.00
29	->	39	-0.00	0.00
29	->	40	-0.00	0.00
29	->	41	-0.01	0.01
29	->	42	-0.00	0.01
29	->	43	-0.02	0.02
29	->	44	-0.01	0.00
29	->	45	-0.00	0.00
29	->	46	-0.00	0.00
29	->	47	-0.00	0.00
29	->	48	-0.00	0.00
29	->	49	0.00	0.00
29	->	50	0.00	0.00

29	->	51	-0.00	0.00
29	->	52	0.00	0.00
29	->	53	0.00	0.00
29	->	54	0.00	0.00
29	->	55	0.00	0.00
29	->	56	0.00	0.00
29	->	57	0.00	0.00
29	->	58	-0.00	0.00
29	->	59	0.00	0.00
29	->	60	0.00	0.00
29	->	61	0.00	0.00
29	->	62	0.00	0.00
29	->	63	-0.00	0.00
29	->	64	-0.00	0.00
29	->	65	0.00	0.00
29	->	66	-0.00	0.00
29	->	67	0.00	0.00
29	->	68	0.00	0.00
29	->	69	0.00	0.00
29	->	70	0.00	0.00
29	->	71	-0.00	0.00
29	->	72	0.00	0.00
29	->	73	0.00	0.00
29	->	74	-0.00	0.00
29	->	75	-0.00	0.00
29	->	76	0.00	0.00
29	->	77	0.00	0.00
29	->	78	0.00	0.00
29	->	79	0.00	0.00
29	->	80	0.00	0.00
29	->	81	0.00	0.00
29	->	82	0.00	0.00
29	->	83	0.00	0.00
29	->	84	0.00	0.00
29	->	85	0.00	0.00
29	->	86	0.00	0.00
29	->	87	-0.00	0.00
29	->	88	-0.00	0.00
29	->	89	-0.00	0.00
29	->	90	-0.02	0.02
29	->	91	-0.05	0.04
29	->	92	-0.01	0.00
29	->	93	-0.00	0.00
29	->	94	-0.00	0.00
29	->	95	-0.00	0.00
29	->	96	-0.00	0.00
29	->	97	-0.00	0.00
29	->	98	-0.00	0.00
29	->	99	-0.00	0.00
29	->	100	-0.00	0.00

29	->	101	-0.00	0.00
29	->	102	-0.00	0.00
29	->	103	-0.00	0.00
29	->	104	-0.00	0.00
29	->	105	-0.00	0.00
29	->	106	-0.00	0.00
29	->	107	-0.00	0.00
30	->	1	-0.00	0.00
30	->	2	-0.00	0.00
30	->	3	-0.00	0.00
30	->	4	-0.00	0.00
30	->	5	-0.01	0.00
30	->	6	-0.00	0.00
30	->	7	-0.00	0.00
30	->	8	-0.00	0.00
30	->	9	-0.00	0.00
30	->	10	-0.00	0.00
30	->	11	-0.00	0.00
30	->	12	-0.00	0.00
30	->	13	0.00	0.00
30	->	14	-0.00	0.00
30	->	15	-0.00	0.00
30	->	16	-0.00	0.00
30	->	17	-0.00	0.00
30	->	18	0.00	0.00
30	->	19	0.00	0.00
30	->	20	-0.01	0.00
30	->	21	-0.07	0.04
30	->	22	-0.04	0.02
30	->	23	-0.03	0.02
30	->	24	-0.57	0.27
30	->	25	-5.19	1.12
30	->	26	-0.45	0.17
30	->	27	-0.04	0.03
30	->	28	-3.71	1.21
30	->	29	-14.45	0.54
30	->	30	-53.47	1.73
30	->	31	-11.06	0.31
30	->	32	-1.22	0.30
30	->	33	-0.10	0.03
30	->	34	-0.01	0.01
30	->	35	-0.00	0.00
30	->	36	-0.00	0.00
30	->	37	0.00	0.00
30	->	38	0.00	0.00
30	->	39	-0.00	0.00
30	->	40	0.00	0.00
30	->	41	-0.10	0.22
30	->	42	-0.15	0.10
30	->	43	-0.65	0.70

30 ->	44	-0.01	0.01
30 ->	45	-0.01	0.00
30 ->	46	0.00	0.00
30 ->	47	-0.01	0.00
30 ->	48	-0.01	0.00
30 ->	49	-0.00	0.00
30 ->	50	-0.00	0.00
30 ->	51	-0.00	0.00
30 ->	52	-0.00	0.00
30 ->	53	-0.00	0.00
30 ->	54	-0.00	0.00
30 ->	55	0.00	0.00
30 ->	56	-0.00	0.00
30 ->	57	-0.00	0.00
30 ->	58	0.00	0.00
30 ->	59	-0.00	0.00
30 ->	60	-0.00	0.00
30 ->	61	0.00	0.00
30 ->	62	0.00	0.00
30 ->	63	0.00	0.00
30 ->	64	0.00	0.00
30 ->	65	0.00	0.00
30 ->	66	0.00	0.00
30 ->	67	0.00	0.00
30 ->	68	-0.00	0.00
30 ->	69	-0.00	0.00
30 ->	70	-0.00	0.00
30 ->	71	0.00	0.00
30 ->	72	-0.00	0.00
30 ->	73	-0.00	0.00
30 ->	74	0.00	0.00
30 ->	75	0.00	0.00
30 ->	76	-0.00	0.00
30 ->	77	-0.00	0.00
30 ->	78	-0.00	0.00
30 ->	79	-0.00	0.00
30 ->	80	-0.00	0.00
30 ->	81	-0.00	0.00
30 ->	82	-0.00	0.00
30 ->	83	0.00	0.00
30 ->	84	0.00	0.00
30 ->	85	0.00	0.00
30 ->	86	0.00	0.00
30 ->	87	0.00	0.00
30 ->	88	0.00	0.00
30 ->	89	-0.00	0.00
30 ->	90	-0.01	0.01
30 ->	91	-0.01	0.01
30 ->	92	-0.00	0.00
30 ->	93	0.00	0.00

30	->	94	-0.00	0.00
30	->	95	0.00	0.00
30	->	96	0.00	0.00
30	->	97	-0.00	0.00
30	->	98	-0.01	0.02
30	->	99	-0.00	0.00
30	->	100	0.00	0.00
30	->	101	0.00	0.00
30	->	102	0.00	0.00
30	->	103	0.00	0.00
30	->	104	0.00	0.00
30	->	105	0.00	0.00
30	->	106	-0.00	0.00
30	->	107	0.00	0.00
31	->	1	0.02	0.00
31	->	2	0.00	0.00
31	->	3	0.00	0.00
31	->	4	0.00	0.00
31	->	5	-0.02	0.00
31	->	6	0.00	0.00
31	->	7	-0.00	0.00
31	->	8	-0.01	0.00
31	->	9	0.00	0.00
31	->	10	-0.02	0.01
31	->	11	-0.01	0.00
31	->	12	0.02	0.01
31	->	13	0.01	0.00
31	->	14	0.00	0.00
31	->	15	0.00	0.00
31	->	16	0.01	0.00
31	->	17	-0.00	0.00
31	->	18	-0.03	0.01
31	->	19	-0.02	0.00
31	->	20	-0.00	0.00
31	->	21	-0.00	0.00
31	->	22	0.03	0.02
31	->	23	-0.01	0.00
31	->	24	-0.01	0.01
31	->	25	0.02	0.09
31	->	26	-0.19	0.23
31	->	27	-0.03	0.01
31	->	28	-0.05	0.02
31	->	29	-1.24	0.52
31	->	30	-11.13	0.31
31	->	31	11.07	3.49
31	->	32	-0.31	0.39
31	->	33	-0.85	0.26
31	->	34	-1.17	1.57
31	->	35	-0.09	0.02
31	->	36	0.02	0.00

31	->	37	-0.00	0.00
31	->	38	-0.06	0.01
31	->	39	-0.03	0.03
31	->	40	0.16	0.10
31	->	41	-2.34	0.73
31	->	42	-2.26	1.46
31	->	43	-1.79	1.44
31	->	44	-0.04	0.06
31	->	45	-0.06	0.03
31	->	46	-0.05	0.02
31	->	47	-0.04	0.05
31	->	48	-0.03	0.02
31	->	49	-0.00	0.00
31	->	50	0.00	0.00
31	->	51	-0.00	0.00
31	->	52	-0.00	0.00
31	->	53	0.02	0.01
31	->	54	0.00	0.00
31	->	55	-0.00	0.00
31	->	56	0.00	0.00
31	->	57	0.00	0.00
31	->	58	-0.01	0.00
31	->	59	0.01	0.00
31	->	60	0.00	0.00
31	->	61	0.00	0.00
31	->	62	-0.00	0.00
31	->	63	-0.00	0.00
31	->	64	-0.00	0.00
31	->	65	-0.00	0.00
31	->	66	-0.00	0.00
31	->	67	-0.00	0.00
31	->	68	0.01	0.00
31	->	69	0.00	0.00
31	->	70	0.00	0.00
31	->	71	-0.00	0.00
31	->	72	0.03	0.01
31	->	73	0.00	0.00
31	->	74	-0.02	0.01
31	->	75	-0.01	0.01
31	->	76	0.01	0.00
31	->	77	0.01	0.00
31	->	78	0.01	0.00
31	->	79	0.00	0.00
31	->	80	0.00	0.00
31	->	81	0.01	0.00
31	->	82	0.00	0.00
31	->	83	0.00	0.00
31	->	84	0.00	0.00
31	->	85	0.00	0.00
31	->	86	-0.00	0.00

31 ->	87	-0.01	0.00
31 ->	88	-0.04	0.02
31 ->	89	-0.46	0.43
31 ->	90	-3.56	3.58
31 ->	91	-0.53	0.47
31 ->	92	-0.05	0.01
31 ->	93	-0.02	0.01
31 ->	94	-0.01	0.00
31 ->	95	-0.01	0.00
31 ->	96	-0.00	0.00
31 ->	97	-0.01	0.00
31 ->	98	-0.04	0.02
31 ->	99	-0.04	0.02
31 ->	100	-0.02	0.01
31 ->	101	-0.01	0.00
31 ->	102	-0.01	0.00
31 ->	103	-0.01	0.00
31 ->	104	-0.01	0.00
31 ->	105	-0.02	0.01
31 ->	106	-0.02	0.01
31 ->	107	-0.02	0.01
32 ->	1	0.00	0.00
32 ->	2	0.00	0.00
32 ->	3	0.00	0.00
32 ->	4	-0.00	0.00
32 ->	5	-0.00	0.00
32 ->	6	-0.00	0.00
32 ->	7	-0.00	0.00
32 ->	8	-0.00	0.00
32 ->	9	0.00	0.00
32 ->	10	-0.00	0.00
32 ->	11	-0.00	0.00
32 ->	12	0.00	0.00
32 ->	13	0.00	0.00
32 ->	14	-0.00	0.00
32 ->	15	0.00	0.00
32 ->	16	0.00	0.00
32 ->	17	-0.00	0.00
32 ->	18	-0.01	0.00
32 ->	19	-0.00	0.00
32 ->	20	-0.00	0.00
32 ->	21	-0.01	0.01
32 ->	22	-0.02	0.01
32 ->	23	-0.01	0.00
32 ->	24	-0.01	0.00
32 ->	25	-1.00	0.49
32 ->	26	-0.03	0.02
32 ->	27	-0.00	0.00
32 ->	28	-0.01	0.00
32 ->	29	-0.03	0.01

32	->	30	-1.24	0.31
32	->	31	-0.36	0.39
32	->	32	1.62	0.61
32	->	33	-10.43	0.49
32	->	34	-0.75	0.38
32	->	35	-0.02	0.01
32	->	36	-0.01	0.01
32	->	37	-0.00	0.00
32	->	38	0.00	0.00
32	->	39	-0.01	0.00
32	->	40	-0.02	0.01
32	->	41	-0.34	0.29
32	->	42	-0.66	0.35
32	->	43	-0.55	0.38
32	->	44	-0.12	0.07
32	->	45	-0.07	0.02
32	->	46	-0.02	0.01
32	->	47	-0.01	0.01
32	->	48	-0.05	0.02
32	->	49	-0.00	0.00
32	->	50	-0.00	0.00
32	->	51	-0.00	0.00
32	->	52	-0.00	0.00
32	->	53	-0.00	0.00
32	->	54	-0.00	0.00
32	->	55	-0.00	0.00
32	->	56	-0.00	0.00
32	->	57	-0.00	0.00
32	->	58	0.00	0.00
32	->	59	-0.00	0.00
32	->	60	-0.00	0.00
32	->	61	0.00	0.00
32	->	62	-0.00	0.00
32	->	63	-0.00	0.00
32	->	64	-0.00	0.00
32	->	65	0.00	0.00
32	->	66	-0.00	0.00
32	->	67	0.00	0.00
32	->	68	-0.00	0.00
32	->	69	-0.00	0.00
32	->	70	0.00	0.00
32	->	71	0.00	0.00
32	->	72	-0.00	0.00
32	->	73	0.00	0.00
32	->	74	-0.00	0.00
32	->	75	-0.00	0.00
32	->	76	0.00	0.00
32	->	77	0.00	0.00
32	->	78	0.00	0.00
32	->	79	0.00	0.00

32	->	80	0.00	0.00
32	->	81	0.00	0.00
32	->	82	0.00	0.00
32	->	83	0.00	0.00
32	->	84	0.00	0.00
32	->	85	0.00	0.00
32	->	86	0.00	0.00
32	->	87	0.00	0.00
32	->	88	-0.00	0.00
32	->	89	-0.00	0.00
32	->	90	-0.01	0.00
32	->	91	-0.01	0.01
32	->	92	-0.01	0.00
32	->	93	-0.00	0.00
32	->	94	-0.00	0.00
32	->	95	-0.00	0.00
32	->	96	-0.00	0.00
32	->	97	-0.01	0.01
32	->	98	-0.04	0.03
32	->	99	-0.03	0.02
32	->	100	-0.01	0.00
32	->	101	-0.00	0.00
32	->	102	-0.00	0.00
32	->	103	-0.00	0.00
32	->	104	-0.00	0.00
32	->	105	-0.00	0.00
32	->	106	-0.00	0.00
32	->	107	-0.00	0.00
33	->	1	-0.00	0.00
33	->	2	0.00	0.00
33	->	3	-0.00	0.00
33	->	4	-0.00	0.00
33	->	5	0.00	0.00
33	->	6	-0.00	0.00
33	->	7	0.00	0.00
33	->	8	0.00	0.00
33	->	9	-0.00	0.00
33	->	10	0.00	0.00
33	->	11	0.00	0.00
33	->	12	-0.00	0.00
33	->	13	0.00	0.00
33	->	14	-0.00	0.00
33	->	15	0.00	0.00
33	->	16	-0.00	0.00
33	->	17	-0.00	0.00
33	->	18	-0.01	0.01
33	->	19	-0.00	0.00
33	->	20	-0.00	0.00
33	->	21	-0.02	0.01
33	->	22	-0.04	0.02

33	->	23	-0.00	0.00
33	->	24	-0.01	0.00
33	->	25	-0.54	0.37
33	->	26	-0.01	0.00
33	->	27	-0.00	0.00
33	->	28	-0.00	0.00
33	->	29	-0.01	0.00
33	->	30	-0.10	0.03
33	->	31	-0.87	0.26
33	->	32	-10.55	0.47
33	->	33	27.51	1.22
33	->	34	-13.09	0.41
33	->	35	-0.47	0.09
33	->	36	-0.03	0.01
33	->	37	-0.01	0.00
33	->	38	-0.01	0.00
33	->	39	-0.03	0.02
33	->	40	-0.23	0.08
33	->	41	-2.40	0.49
33	->	42	-3.27	0.56
33	->	43	-0.86	0.23
33	->	44	-2.78	0.46
33	->	45	-2.20	0.35
33	->	46	0.02	0.03
33	->	47	-0.14	0.05
33	->	48	-1.73	0.35
33	->	49	-0.04	0.01
33	->	50	-0.01	0.00
33	->	51	-0.02	0.01
33	->	52	-0.02	0.01
33	->	53	-0.00	0.00
33	->	54	-0.00	0.00
33	->	55	-0.01	0.00
33	->	56	-0.00	0.00
33	->	57	0.00	0.00
33	->	58	-0.00	0.00
33	->	59	-0.00	0.00
33	->	60	-0.00	0.00
33	->	61	-0.00	0.00
33	->	62	0.00	0.00
33	->	63	0.00	0.00
33	->	64	0.00	0.00
33	->	65	0.00	0.00
33	->	66	-0.00	0.00
33	->	67	0.00	0.00
33	->	68	-0.00	0.00
33	->	69	0.00	0.00
33	->	70	-0.00	0.00
33	->	71	-0.00	0.00
33	->	72	-0.00	0.00

33	->	73	-0.00	0.00
33	->	74	0.00	0.00
33	->	75	0.00	0.00
33	->	76	0.00	0.00
33	->	77	-0.00	0.00
33	->	78	-0.00	0.00
33	->	79	-0.00	0.00
33	->	80	-0.00	0.00
33	->	81	-0.00	0.00
33	->	82	0.00	0.00
33	->	83	0.00	0.00
33	->	84	0.00	0.00
33	->	85	-0.00	0.00
33	->	86	0.00	0.00
33	->	87	-0.00	0.00
33	->	88	-0.00	0.00
33	->	89	-0.01	0.00
33	->	90	-0.01	0.01
33	->	91	-0.01	0.01
33	->	92	-0.00	0.00
33	->	93	-0.00	0.00
33	->	94	-0.00	0.00
33	->	95	-0.00	0.00
33	->	96	-0.00	0.00
33	->	97	-0.01	0.00
33	->	98	-0.12	0.18
33	->	99	-0.48	0.37
33	->	100	-0.03	0.01
33	->	101	-0.01	0.00
33	->	102	-0.00	0.00
33	->	103	-0.00	0.00
33	->	104	-0.00	0.00
33	->	105	-0.00	0.00
33	->	106	-0.00	0.00
33	->	107	0.00	0.00
34	->	1	0.00	0.00
34	->	2	0.00	0.00
34	->	3	0.00	0.00
34	->	4	0.00	0.00
34	->	5	-0.00	0.00
34	->	6	0.00	0.00
34	->	7	0.00	0.00
34	->	8	-0.00	0.00
34	->	9	0.00	0.00
34	->	10	-0.00	0.00
34	->	11	-0.00	0.00
34	->	12	0.00	0.00
34	->	13	0.00	0.00
34	->	14	0.00	0.00
34	->	15	0.00	0.00

34 ->	16	0.00	0.00
34 ->	17	-0.00	0.00
34 ->	18	-0.00	0.00
34 ->	19	-0.00	0.00
34 ->	20	-0.00	0.00
34 ->	21	-0.00	0.00
34 ->	22	-0.00	0.00
34 ->	23	-0.00	0.00
34 ->	24	-0.00	0.00
34 ->	25	-0.01	0.01
34 ->	26	-0.00	0.00
34 ->	27	-0.00	0.00
34 ->	28	-0.00	0.00
34 ->	29	-0.00	0.00
34 ->	30	-0.01	0.01
34 ->	31	-1.16	1.55
34 ->	32	-0.78	0.40
34 ->	33	-13.09	0.41
34 ->	34	-55.64	2.31
34 ->	35	-11.88	0.42
34 ->	36	-0.42	0.18
34 ->	37	-0.05	0.03
34 ->	38	-0.10	0.05
34 ->	39	-1.98	1.43
34 ->	40	-2.00	0.45
34 ->	41	-2.79	1.23
34 ->	42	-0.82	0.28
34 ->	43	-0.04	0.01
34 ->	44	-0.02	0.01
34 ->	45	-0.02	0.01
34 ->	46	-0.00	0.00
34 ->	47	0.02	0.01
34 ->	48	-1.19	0.24
34 ->	49	-0.01	0.00
34 ->	50	0.00	0.00
34 ->	51	-0.10	0.02
34 ->	52	-0.05	0.02
34 ->	53	-0.00	0.00
34 ->	54	-0.00	0.00
34 ->	55	-0.05	0.01
34 ->	56	-0.00	0.00
34 ->	57	0.00	0.00
34 ->	58	0.00	0.00
34 ->	59	0.00	0.00
34 ->	60	-0.00	0.00
34 ->	61	0.00	0.00
34 ->	62	0.00	0.00
34 ->	63	-0.00	0.00
34 ->	64	-0.00	0.00
34 ->	65	-0.00	0.00

34	->	66	-0.00	0.00
34	->	67	0.00	0.00
34	->	68	0.00	0.00
34	->	69	-0.00	0.00
34	->	70	0.00	0.00
34	->	71	0.00	0.00
34	->	72	0.00	0.00
34	->	73	-0.00	0.00
34	->	74	-0.00	0.00
34	->	75	-0.00	0.00
34	->	76	0.00	0.00
34	->	77	0.00	0.00
34	->	78	0.00	0.00
34	->	79	0.00	0.00
34	->	80	0.00	0.00
34	->	81	0.00	0.00
34	->	82	0.00	0.00
34	->	83	0.00	0.00
34	->	84	0.00	0.00
34	->	85	0.00	0.00
34	->	86	-0.00	0.00
34	->	87	-0.00	0.00
34	->	88	-0.01	0.01
34	->	89	-0.03	0.01
34	->	90	-0.03	0.02
34	->	91	-0.01	0.00
34	->	92	-0.00	0.00
34	->	93	-0.00	0.00
34	->	94	-0.00	0.00
34	->	95	-0.00	0.00
34	->	96	0.00	0.00
34	->	97	-0.00	0.00
34	->	98	-0.00	0.00
34	->	99	-0.00	0.00
34	->	100	-0.00	0.00
34	->	101	-0.00	0.00
34	->	102	-0.00	0.00
34	->	103	-0.00	0.00
34	->	104	-0.00	0.00
34	->	105	-0.00	0.00
34	->	106	-0.00	0.00
34	->	107	-0.00	0.00
35	->	1	-0.01	0.00
35	->	2	-0.00	0.00
35	->	3	0.00	0.00
35	->	4	0.00	0.00
35	->	5	0.00	0.00
35	->	6	-0.00	0.00
35	->	7	0.00	0.00
35	->	8	0.00	0.00

35	->	9	-0.00	0.00
35	->	10	0.01	0.00
35	->	11	0.00	0.00
35	->	12	-0.01	0.00
35	->	13	-0.00	0.00
35	->	14	-0.00	0.00
35	->	15	-0.00	0.00
35	->	16	-0.01	0.00
35	->	17	0.00	0.00
35	->	18	0.01	0.00
35	->	19	0.01	0.00
35	->	20	0.00	0.00
35	->	21	0.00	0.00
35	->	22	-0.02	0.00
35	->	23	-0.00	0.00
35	->	24	0.00	0.00
35	->	25	-0.03	0.00
35	->	26	-0.00	0.00
35	->	27	-0.00	0.00
35	->	28	0.00	0.00
35	->	29	0.00	0.00
35	->	30	-0.00	0.00
35	->	31	-0.09	0.02
35	->	32	-0.02	0.01
35	->	33	-0.47	0.09
35	->	34	-11.95	0.41
35	->	35	-24.85	2.46
35	->	36	-10.28	1.14
35	->	37	2.27	1.38
35	->	38	3.10	0.58
35	->	39	-2.20	0.70
35	->	40	-2.74	0.45
35	->	41	-0.37	0.12
35	->	42	-0.57	0.25
35	->	43	0.01	0.01
35	->	44	-0.04	0.01
35	->	45	0.00	0.01
35	->	46	0.04	0.01
35	->	47	-0.13	0.03
35	->	48	-1.10	0.22
35	->	49	0.02	0.02
35	->	50	-0.07	0.03
35	->	51	2.90	0.66
35	->	52	-0.30	0.27
35	->	53	-0.06	0.02
35	->	54	-0.07	0.05
35	->	55	2.49	0.43
35	->	56	-0.02	0.01
35	->	57	-0.00	0.00
35	->	58	0.03	0.01

35	->	59	-0.03	0.00
35	->	60	-0.00	0.00
35	->	61	0.00	0.00
35	->	62	0.00	0.00
35	->	63	0.01	0.00
35	->	64	0.00	0.00
35	->	65	0.00	0.00
35	->	66	0.01	0.00
35	->	67	0.00	0.00
35	->	68	-0.01	0.00
35	->	69	-0.01	0.00
35	->	70	-0.00	0.00
35	->	71	-0.00	0.00
35	->	72	-0.04	0.01
35	->	73	-0.00	0.00
35	->	74	0.02	0.00
35	->	75	0.02	0.00
35	->	76	-0.01	0.00
35	->	77	-0.01	0.00
35	->	78	-0.01	0.00
35	->	79	-0.00	0.00
35	->	80	-0.00	0.00
35	->	81	-0.01	0.00
35	->	82	-0.00	0.00
35	->	83	0.00	0.00
35	->	84	-0.00	0.00
35	->	85	-0.00	0.00
35	->	86	0.00	0.00
35	->	87	0.02	0.01
35	->	88	0.09	0.03
35	->	89	0.15	0.06
35	->	90	0.04	0.01
35	->	91	0.03	0.00
35	->	92	0.01	0.00
35	->	93	0.01	0.00
35	->	94	0.00	0.00
35	->	95	0.00	0.00
35	->	96	0.00	0.00
35	->	97	0.00	0.00
35	->	98	0.02	0.00
35	->	99	0.03	0.00
35	->	100	0.03	0.00
35	->	101	0.02	0.00
35	->	102	0.01	0.00
35	->	103	0.01	0.00
35	->	104	0.01	0.00
35	->	105	0.01	0.00
35	->	106	0.00	0.00
35	->	107	0.01	0.00
36	->	1	0.00	0.00

36	->	2	0.00	0.00
36	->	3	0.00	0.00
36	->	4	-0.00	0.00
36	->	5	-0.00	0.00
36	->	6	0.00	0.00
36	->	7	0.00	0.00
36	->	8	-0.00	0.00
36	->	9	0.00	0.00
36	->	10	-0.00	0.00
36	->	11	-0.00	0.00
36	->	12	0.00	0.00
36	->	13	0.00	0.00
36	->	14	0.00	0.00
36	->	15	0.00	0.00
36	->	16	0.01	0.00
36	->	17	-0.00	0.00
36	->	18	-0.01	0.00
36	->	19	-0.01	0.00
36	->	20	-0.00	0.00
36	->	21	-0.00	0.00
36	->	22	0.01	0.00
36	->	23	0.00	0.00
36	->	24	-0.00	0.00
36	->	25	0.02	0.00
36	->	26	0.00	0.00
36	->	27	0.00	0.00
36	->	28	-0.00	0.00
36	->	29	-0.00	0.00
36	->	30	-0.00	0.00
36	->	31	0.02	0.00
36	->	32	-0.01	0.01
36	->	33	-0.03	0.01
36	->	34	-0.44	0.19
36	->	35	-10.36	1.16
36	->	36	-115.97	2.45
36	->	37	-13.99	1.11
36	->	38	-0.87	0.30
36	->	39	-0.96	0.75
36	->	40	0.00	0.02
36	->	41	-0.02	0.01
36	->	42	-0.03	0.01
36	->	43	-0.00	0.00
36	->	44	0.01	0.00
36	->	45	-0.01	0.00
36	->	46	-0.03	0.01
36	->	47	-0.01	0.00
36	->	48	-0.17	0.12
36	->	49	-0.02	0.02
36	->	50	-0.00	0.00
36	->	51	-0.15	0.05

36	->	52	-0.59	0.90
36	->	53	0.02	0.01
36	->	54	-0.00	0.00
36	->	55	-0.31	0.13
36	->	56	-0.06	0.16
36	->	57	-0.00	0.00
36	->	58	-0.04	0.03
36	->	59	0.02	0.01
36	->	60	0.00	0.00
36	->	61	-0.00	0.00
36	->	62	-0.00	0.00
36	->	63	-0.00	0.00
36	->	64	-0.00	0.00
36	->	65	-0.00	0.00
36	->	66	-0.00	0.00
36	->	67	-0.00	0.00
36	->	68	0.01	0.00
36	->	69	0.01	0.00
36	->	70	-0.00	0.00
36	->	71	-0.00	0.00
36	->	72	0.02	0.00
36	->	73	0.00	0.00
36	->	74	-0.01	0.00
36	->	75	-0.01	0.00
36	->	76	0.01	0.00
36	->	77	0.00	0.00
36	->	78	0.01	0.00
36	->	79	0.00	0.00
36	->	80	0.00	0.00
36	->	81	0.00	0.00
36	->	82	0.00	0.00
36	->	83	-0.00	0.00
36	->	84	0.00	0.00
36	->	85	0.00	0.00
36	->	86	-0.00	0.00
36	->	87	-0.01	0.00
36	->	88	-0.03	0.01
36	->	89	-0.04	0.01
36	->	90	-0.02	0.00
36	->	91	-0.02	0.00
36	->	92	-0.01	0.00
36	->	93	-0.00	0.00
36	->	94	-0.00	0.00
36	->	95	-0.00	0.00
36	->	96	-0.00	0.00
36	->	97	-0.00	0.00
36	->	98	-0.02	0.00
36	->	99	-0.03	0.01
36	->	100	-0.03	0.01
36	->	101	-0.02	0.01

36	->	102	-0.01	0.00
36	->	103	-0.00	0.00
36	->	104	-0.00	0.00
36	->	105	-0.00	0.00
36	->	106	-0.00	0.00
36	->	107	-0.00	0.00
37	->	1	-0.00	0.00
37	->	2	0.00	0.00
37	->	3	0.00	0.00
37	->	4	0.00	0.00
37	->	5	0.00	0.00
37	->	6	-0.00	0.00
37	->	7	0.00	0.00
37	->	8	0.00	0.00
37	->	9	-0.00	0.00
37	->	10	0.00	0.00
37	->	11	0.00	0.00
37	->	12	-0.00	0.00
37	->	13	-0.00	0.00
37	->	14	-0.00	0.00
37	->	15	0.00	0.00
37	->	16	-0.00	0.00
37	->	17	0.00	0.00
37	->	18	0.00	0.00
37	->	19	0.00	0.00
37	->	20	0.00	0.00
37	->	21	0.00	0.00
37	->	22	-0.00	0.00
37	->	23	-0.00	0.00
37	->	24	0.00	0.00
37	->	25	-0.00	0.00
37	->	26	-0.00	0.00
37	->	27	-0.00	0.00
37	->	28	0.00	0.00
37	->	29	0.00	0.00
37	->	30	0.00	0.00
37	->	31	-0.00	0.00
37	->	32	-0.00	0.00
37	->	33	-0.01	0.00
37	->	34	-0.05	0.03
37	->	35	2.20	1.38
37	->	36	-14.11	1.10
37	->	37	22.06	1.47
37	->	38	-8.16	1.10
37	->	39	-0.80	0.45
37	->	40	-0.28	0.14
37	->	41	-0.01	0.00
37	->	42	-0.02	0.01
37	->	43	-0.00	0.00
37	->	44	-0.00	0.00

37	->	45	-0.00	0.00
37	->	46	0.00	0.00
37	->	47	-0.01	0.00
37	->	48	-0.03	0.01
37	->	49	-0.01	0.00
37	->	50	-0.01	0.00
37	->	51	0.77	0.12
37	->	52	-0.13	0.13
37	->	53	-0.01	0.00
37	->	54	-0.03	0.01
37	->	55	0.78	0.63
37	->	56	-0.04	0.02
37	->	57	-0.00	0.00
37	->	58	-0.00	0.00
37	->	59	-0.00	0.00
37	->	60	-0.00	0.00
37	->	61	0.00	0.00
37	->	62	0.00	0.00
37	->	63	0.00	0.00
37	->	64	0.00	0.00
37	->	65	0.00	0.00
37	->	66	0.00	0.00
37	->	67	0.00	0.00
37	->	68	-0.00	0.00
37	->	69	-0.00	0.00
37	->	70	-0.00	0.00
37	->	71	-0.00	0.00
37	->	72	-0.00	0.00
37	->	73	-0.00	0.00
37	->	74	0.00	0.00
37	->	75	0.00	0.00
37	->	76	-0.00	0.00
37	->	77	-0.00	0.00
37	->	78	-0.00	0.00
37	->	79	-0.00	0.00
37	->	80	-0.00	0.00
37	->	81	-0.00	0.00
37	->	82	-0.00	0.00
37	->	83	0.00	0.00
37	->	84	0.00	0.00
37	->	85	-0.00	0.00
37	->	86	-0.00	0.00
37	->	87	-0.00	0.00
37	->	88	-0.00	0.01
37	->	89	0.01	0.01
37	->	90	0.00	0.00
37	->	91	0.00	0.00
37	->	92	0.00	0.00
37	->	93	0.00	0.00
37	->	94	0.00	0.00

37	->	95	0.00	0.00
37	->	96	0.00	0.00
37	->	97	0.00	0.00
37	->	98	0.00	0.00
37	->	99	0.00	0.00
37	->	100	0.00	0.00
37	->	101	0.00	0.00
37	->	102	0.00	0.00
37	->	103	0.00	0.00
37	->	104	0.00	0.00
37	->	105	0.00	0.00
37	->	106	-0.00	0.00
37	->	107	0.00	0.00
38	->	1	-0.01	0.00
38	->	2	0.00	0.00
38	->	3	0.00	0.00
38	->	4	-0.00	0.00
38	->	5	0.00	0.00
38	->	6	-0.00	0.00
38	->	7	-0.00	0.00
38	->	8	0.00	0.00
38	->	9	-0.00	0.00
38	->	10	0.01	0.00
38	->	11	0.00	0.00
38	->	12	-0.01	0.00
38	->	13	-0.00	0.00
38	->	14	-0.00	0.00
38	->	15	-0.00	0.00
38	->	16	-0.01	0.00
38	->	17	0.00	0.00
38	->	18	0.01	0.00
38	->	19	0.01	0.00
38	->	20	0.00	0.00
38	->	21	0.00	0.00
38	->	22	-0.01	0.00
38	->	23	-0.00	0.00
38	->	24	0.00	0.00
38	->	25	-0.02	0.00
38	->	26	0.00	0.00
38	->	27	-0.00	0.00
38	->	28	0.00	0.00
38	->	29	0.00	0.00
38	->	30	0.00	0.00
38	->	31	-0.06	0.01
38	->	32	0.00	0.00
38	->	33	-0.01	0.00
38	->	34	-0.10	0.05
38	->	35	3.10	0.58
38	->	36	-0.87	0.30
38	->	37	-8.10	1.10

38	->	38	-28.65	4.98
38	->	39	-11.72	0.73
38	->	40	-6.75	4.04
38	->	41	0.01	0.03
38	->	42	0.00	0.04
38	->	43	0.00	0.00
38	->	44	-0.02	0.00
38	->	45	0.00	0.00
38	->	46	0.03	0.00
38	->	47	-0.03	0.01
38	->	48	-0.01	0.01
38	->	49	0.00	0.00
38	->	50	-0.03	0.01
38	->	51	3.83	0.98
38	->	52	0.06	0.02
38	->	53	-0.05	0.01
38	->	54	-0.04	0.06
38	->	55	2.27	0.45
38	->	56	-0.01	0.00
38	->	57	-0.00	0.00
38	->	58	0.02	0.00
38	->	59	-0.03	0.01
38	->	60	-0.00	0.00
38	->	61	0.00	0.00
38	->	62	0.00	0.00
38	->	63	0.01	0.00
38	->	64	0.00	0.00
38	->	65	0.00	0.00
38	->	66	0.01	0.00
38	->	67	0.00	0.00
38	->	68	-0.01	0.00
38	->	69	-0.01	0.00
38	->	70	-0.00	0.00
38	->	71	-0.00	0.00
38	->	72	-0.05	0.01
38	->	73	-0.00	0.00
38	->	74	0.02	0.01
38	->	75	0.02	0.00
38	->	76	-0.01	0.00
38	->	77	-0.01	0.00
38	->	78	-0.02	0.00
38	->	79	-0.00	0.00
38	->	80	-0.00	0.00
38	->	81	-0.01	0.00
38	->	82	-0.00	0.00
38	->	83	0.00	0.00
38	->	84	-0.00	0.00
38	->	85	-0.00	0.00
38	->	86	0.00	0.00
38	->	87	0.04	0.02

38	->	88	0.20	0.07
38	->	89	0.47	0.20
38	->	90	0.06	0.03
38	->	91	0.03	0.01
38	->	92	0.01	0.00
38	->	93	0.01	0.00
38	->	94	0.00	0.00
38	->	95	0.00	0.00
38	->	96	0.00	0.00
38	->	97	0.00	0.00
38	->	98	0.01	0.00
38	->	99	0.02	0.00
38	->	100	0.02	0.00
38	->	101	0.01	0.00
38	->	102	0.01	0.00
38	->	103	0.01	0.00
38	->	104	0.01	0.00
38	->	105	0.01	0.00
38	->	106	0.00	0.00
38	->	107	0.01	0.00
39	->	1	-0.00	0.00
39	->	2	0.00	0.00
39	->	3	0.00	0.00
39	->	4	0.00	0.00
39	->	5	0.00	0.00
39	->	6	-0.00	0.00
39	->	7	-0.00	0.00
39	->	8	0.00	0.00
39	->	9	-0.00	0.00
39	->	10	0.00	0.00
39	->	11	0.00	0.00
39	->	12	-0.00	0.00
39	->	13	-0.00	0.00
39	->	14	0.00	0.00
39	->	15	-0.00	0.00
39	->	16	-0.00	0.00
39	->	17	0.00	0.00
39	->	18	0.00	0.00
39	->	19	0.00	0.00
39	->	20	0.00	0.00
39	->	21	0.00	0.00
39	->	22	-0.00	0.00
39	->	23	0.00	0.00
39	->	24	0.00	0.00
39	->	25	-0.00	0.00
39	->	26	-0.00	0.00
39	->	27	-0.00	0.00
39	->	28	-0.00	0.00
39	->	29	-0.00	0.00
39	->	30	-0.00	0.00

39	->	31	-0.03	0.03
39	->	32	-0.01	0.00
39	->	33	-0.03	0.02
39	->	34	-1.97	1.43
39	->	35	-2.23	0.70
39	->	36	-0.96	0.74
39	->	37	-0.80	0.45
39	->	38	-11.83	0.74
39	->	39	11.46	1.30
39	->	40	-9.67	0.47
39	->	41	-0.50	0.32
39	->	42	-0.11	0.03
39	->	43	-0.01	0.00
39	->	44	-0.00	0.00
39	->	45	-0.00	0.00
39	->	46	-0.00	0.00
39	->	47	0.00	0.00
39	->	48	-0.02	0.01
39	->	49	-0.00	0.00
39	->	50	-0.00	0.00
39	->	51	-0.10	0.06
39	->	52	-0.01	0.01
39	->	53	-0.00	0.00
39	->	54	-0.00	0.00
39	->	55	-0.03	0.04
39	->	56	-0.00	0.00
39	->	57	0.00	0.00
39	->	58	-0.00	0.00
39	->	59	0.00	0.00
39	->	60	0.00	0.00
39	->	61	-0.00	0.00
39	->	62	0.00	0.00
39	->	63	-0.00	0.00
39	->	64	-0.00	0.00
39	->	65	0.00	0.00
39	->	66	-0.00	0.00
39	->	67	0.00	0.00
39	->	68	0.00	0.00
39	->	69	0.00	0.00
39	->	70	0.00	0.00
39	->	71	0.00	0.00
39	->	72	-0.00	0.00
39	->	73	0.00	0.00
39	->	74	-0.00	0.00
39	->	75	-0.00	0.00
39	->	76	0.00	0.00
39	->	77	0.00	0.00
39	->	78	0.00	0.00
39	->	79	0.00	0.00
39	->	80	0.00	0.00

39	->	81	0.00	0.00
39	->	82	0.00	0.00
39	->	83	0.00	0.00
39	->	84	0.00	0.00
39	->	85	0.00	0.00
39	->	86	-0.00	0.00
39	->	87	-0.00	0.00
39	->	88	-0.01	0.00
39	->	89	-0.03	0.02
39	->	90	-0.02	0.02
39	->	91	-0.00	0.00
39	->	92	-0.00	0.00
39	->	93	0.00	0.00
39	->	94	-0.00	0.00
39	->	95	0.00	0.00
39	->	96	-0.00	0.00
39	->	97	0.00	0.00
39	->	98	0.00	0.00
39	->	99	-0.00	0.00
39	->	100	-0.00	0.00
39	->	101	-0.00	0.00
39	->	102	-0.00	0.00
39	->	103	0.00	0.00
39	->	104	-0.00	0.00
39	->	105	0.00	0.00
39	->	106	-0.00	0.00
39	->	107	-0.00	0.00
40	->	1	0.01	0.00
40	->	2	0.00	0.00
40	->	3	-0.00	0.00
40	->	4	0.00	0.00
40	->	5	-0.01	0.00
40	->	6	0.00	0.00
40	->	7	0.00	0.00
40	->	8	-0.00	0.00
40	->	9	0.00	0.00
40	->	10	-0.01	0.00
40	->	11	-0.01	0.00
40	->	12	0.01	0.00
40	->	13	0.00	0.00
40	->	14	0.00	0.00
40	->	15	0.00	0.00
40	->	16	0.01	0.00
40	->	17	-0.00	0.00
40	->	18	-0.01	0.00
40	->	19	-0.01	0.00
40	->	20	-0.00	0.00
40	->	21	-0.00	0.00
40	->	22	0.02	0.00
40	->	23	-0.00	0.00

40	->	24	-0.00	0.00
40	->	25	0.02	0.01
40	->	26	-0.01	0.00
40	->	27	-0.00	0.00
40	->	28	-0.00	0.00
40	->	29	-0.00	0.00
40	->	30	0.00	0.00
40	->	31	0.16	0.10
40	->	32	-0.02	0.01
40	->	33	-0.23	0.08
40	->	34	-1.99	0.45
40	->	35	-2.74	0.45
40	->	36	0.00	0.02
40	->	37	-0.29	0.14
40	->	38	-6.88	4.04
40	->	39	-9.75	0.48
40	->	40	-104.51	2.51
40	->	41	-14.92	0.56
40	->	42	-2.77	0.51
40	->	43	-0.07	0.03
40	->	44	0.01	0.01
40	->	45	-0.01	0.00
40	->	46	-0.03	0.01
40	->	47	0.01	0.02
40	->	48	-0.15	0.06
40	->	49	-0.01	0.00
40	->	50	0.01	0.01
40	->	51	-1.47	0.82
40	->	52	-0.05	0.02
40	->	53	0.02	0.01
40	->	54	-0.01	0.03
40	->	55	-0.36	0.27
40	->	56	-0.00	0.00
40	->	57	0.00	0.00
40	->	58	-0.02	0.00
40	->	59	0.02	0.01
40	->	60	0.00	0.00
40	->	61	-0.00	0.00
40	->	62	-0.00	0.00
40	->	63	-0.01	0.00
40	->	64	-0.00	0.00
40	->	65	-0.00	0.00
40	->	66	-0.01	0.00
40	->	67	-0.00	0.00
40	->	68	0.01	0.00
40	->	69	0.01	0.00
40	->	70	0.00	0.00
40	->	71	-0.00	0.00
40	->	72	0.05	0.01
40	->	73	0.00	0.00

40	->	74	-0.03	0.01
40	->	75	-0.03	0.01
40	->	76	0.01	0.00
40	->	77	0.01	0.00
40	->	78	0.02	0.00
40	->	79	0.00	0.00
40	->	80	0.00	0.00
40	->	81	0.01	0.00
40	->	82	0.00	0.00
40	->	83	0.00	0.00
40	->	84	0.00	0.00
40	->	85	0.00	0.00
40	->	86	-0.01	0.00
40	->	87	-0.06	0.05
40	->	88	-0.81	0.68
40	->	89	-6.18	3.18
40	->	90	-3.09	3.40
40	->	91	-0.07	0.03
40	->	92	-0.02	0.01
40	->	93	-0.01	0.00
40	->	94	-0.01	0.00
40	->	95	-0.00	0.00
40	->	96	-0.00	0.00
40	->	97	-0.00	0.00
40	->	98	-0.01	0.00
40	->	99	-0.01	0.00
40	->	100	-0.02	0.00
40	->	101	-0.01	0.00
40	->	102	-0.01	0.00
40	->	103	-0.01	0.00
40	->	104	-0.01	0.00
40	->	105	-0.02	0.00
40	->	106	-0.01	0.01
40	->	107	-0.03	0.01
41	->	1	0.00	0.00
41	->	2	0.00	0.00
41	->	3	0.00	0.00
41	->	4	0.00	0.00
41	->	5	-0.00	0.00
41	->	6	0.00	0.00
41	->	7	0.00	0.00
41	->	8	-0.00	0.00
41	->	9	0.00	0.00
41	->	10	-0.00	0.00
41	->	11	-0.00	0.00
41	->	12	0.00	0.00
41	->	13	0.00	0.00
41	->	14	-0.00	0.00
41	->	15	0.00	0.00
41	->	16	0.00	0.00

41	->	17	-0.00	0.00
41	->	18	-0.00	0.00
41	->	19	-0.00	0.00
41	->	20	0.00	0.00
41	->	21	0.00	0.00
41	->	22	0.01	0.01
41	->	23	-0.00	0.00
41	->	24	0.00	0.00
41	->	25	0.02	0.02
41	->	26	-0.04	0.04
41	->	27	-0.01	0.00
41	->	28	-0.00	0.00
41	->	29	-0.01	0.01
41	->	30	-0.10	0.22
41	->	31	-2.31	0.70
41	->	32	-0.34	0.28
41	->	33	-2.40	0.49
41	->	34	-2.74	1.21
41	->	35	-0.37	0.12
41	->	36	-0.02	0.01
41	->	37	-0.01	0.00
41	->	38	0.01	0.03
41	->	39	-0.51	0.33
41	->	40	-14.90	0.56
41	->	41	-32.27	1.92
41	->	42	-9.01	0.55
41	->	43	-0.59	0.12
41	->	44	-0.04	0.03
41	->	45	-0.05	0.01
41	->	46	-0.02	0.01
41	->	47	0.04	0.03
41	->	48	-0.10	0.03
41	->	49	-0.01	0.01
41	->	50	0.00	0.01
41	->	51	-0.02	0.01
41	->	52	-0.01	0.01
41	->	53	-0.00	0.00
41	->	54	-0.00	0.00
41	->	55	-0.01	0.00
41	->	56	-0.00	0.00
41	->	57	-0.00	0.00
41	->	58	0.00	0.00
41	->	59	-0.00	0.00
41	->	60	-0.00	0.00
41	->	61	0.00	0.00
41	->	62	0.00	0.00
41	->	63	0.00	0.00
41	->	64	0.00	0.00
41	->	65	0.00	0.00
41	->	66	0.00	0.00

41	->	67	0.00	0.00
41	->	68	-0.00	0.00
41	->	69	-0.00	0.00
41	->	70	0.00	0.00
41	->	71	0.00	0.00
41	->	72	-0.00	0.00
41	->	73	-0.00	0.00
41	->	74	0.00	0.00
41	->	75	0.00	0.00
41	->	76	-0.00	0.00
41	->	77	-0.00	0.00
41	->	78	-0.00	0.00
41	->	79	-0.00	0.00
41	->	80	-0.00	0.00
41	->	81	-0.00	0.00
41	->	82	0.00	0.00
41	->	83	0.00	0.00
41	->	84	0.00	0.00
41	->	85	0.00	0.00
41	->	86	-0.00	0.00
41	->	87	-0.00	0.00
41	->	88	-0.01	0.01
41	->	89	-0.10	0.04
41	->	90	-0.30	0.34
41	->	91	-0.17	0.24
41	->	92	-0.01	0.01
41	->	93	-0.00	0.00
41	->	94	-0.00	0.00
41	->	95	-0.00	0.00
41	->	96	-0.00	0.00
41	->	97	-0.00	0.00
41	->	98	-0.01	0.00
41	->	99	-0.01	0.01
41	->	100	-0.00	0.00
41	->	101	-0.00	0.00
41	->	102	-0.00	0.00
41	->	103	-0.00	0.00
41	->	104	-0.00	0.00
41	->	105	-0.00	0.00
41	->	106	-0.00	0.00
41	->	107	-0.00	0.00
42	->	1	0.00	0.00
42	->	2	0.00	0.00
42	->	3	-0.00	0.00
42	->	4	0.00	0.00
42	->	5	-0.00	0.00
42	->	6	-0.00	0.00
42	->	7	0.00	0.00
42	->	8	-0.00	0.00
42	->	9	0.00	0.00

42	->	10	-0.00	0.00
42	->	11	-0.00	0.00
42	->	12	0.00	0.00
42	->	13	0.00	0.00
42	->	14	-0.00	0.00
42	->	15	0.00	0.00
42	->	16	0.00	0.00
42	->	17	-0.00	0.00
42	->	18	-0.00	0.00
42	->	19	-0.00	0.00
42	->	20	-0.00	0.00
42	->	21	0.00	0.00
42	->	22	0.03	0.02
42	->	23	-0.01	0.00
42	->	24	-0.00	0.00
42	->	25	0.01	0.04
42	->	26	-0.03	0.01
42	->	27	-0.01	0.00
42	->	28	-0.00	0.00
42	->	29	-0.00	0.01
42	->	30	-0.15	0.10
42	->	31	-2.25	1.45
42	->	32	-0.65	0.35
42	->	33	-3.28	0.56
42	->	34	-0.82	0.28
42	->	35	-0.56	0.25
42	->	36	-0.03	0.02
42	->	37	-0.02	0.01
42	->	38	0.00	0.04
42	->	39	-0.11	0.03
42	->	40	-2.74	0.50
42	->	41	-9.12	0.56
42	->	42	28.69	1.34
42	->	43	-17.22	0.30
42	->	44	-0.95	0.21
42	->	45	-0.18	0.05
42	->	46	-0.12	0.03
42	->	47	-2.28	0.41
42	->	48	-2.52	0.39
42	->	49	-0.07	0.02
42	->	50	-0.15	0.06
42	->	51	-2.16	0.33
42	->	52	-0.07	0.03
42	->	53	-0.02	0.01
42	->	54	-0.02	0.02
42	->	55	-0.00	0.01
42	->	56	-0.00	0.00
42	->	57	-0.00	0.00
42	->	58	-0.00	0.00
42	->	59	-0.00	0.00

42	->	60	-0.00	0.00
42	->	61	-0.00	0.00
42	->	62	-0.00	0.00
42	->	63	-0.00	0.00
42	->	64	-0.00	0.00
42	->	65	-0.00	0.00
42	->	66	-0.00	0.00
42	->	67	0.00	0.00
42	->	68	0.00	0.00
42	->	69	0.00	0.00
42	->	70	-0.00	0.00
42	->	71	-0.00	0.00
42	->	72	-0.03	0.04
42	->	73	-0.00	0.00
42	->	74	-0.01	0.00
42	->	75	-0.00	0.00
42	->	76	0.00	0.00
42	->	77	0.00	0.00
42	->	78	0.00	0.00
42	->	79	0.00	0.00
42	->	80	0.00	0.00
42	->	81	0.00	0.00
42	->	82	0.00	0.00
42	->	83	0.00	0.00
42	->	84	0.00	0.00
42	->	85	0.00	0.00
42	->	86	-0.00	0.00
42	->	87	-0.01	0.00
42	->	88	-0.02	0.04
42	->	89	-0.87	0.70
42	->	90	-1.08	0.47
42	->	91	-0.27	0.19
42	->	92	-0.04	0.03
42	->	93	-0.01	0.00
42	->	94	-0.00	0.00
42	->	95	-0.00	0.00
42	->	96	-0.00	0.00
42	->	97	-0.00	0.00
42	->	98	-0.01	0.01
42	->	99	-0.02	0.02
42	->	100	-0.01	0.00
42	->	101	-0.00	0.00
42	->	102	-0.00	0.00
42	->	103	-0.01	0.00
42	->	104	-0.01	0.01
42	->	105	-0.01	0.00
42	->	106	-0.01	0.00
42	->	107	-0.00	0.00
43	->	1	-0.00	0.00
43	->	2	-0.00	0.00

43 ->	3	-0.00	0.00
43 ->	4	0.00	0.00
43 ->	5	-0.00	0.00
43 ->	6	-0.00	0.00
43 ->	7	-0.00	0.00
43 ->	8	-0.00	0.00
43 ->	9	0.00	0.00
43 ->	10	-0.00	0.00
43 ->	11	-0.00	0.00
43 ->	12	-0.00	0.01
43 ->	13	-0.00	0.00
43 ->	14	-0.00	0.00
43 ->	15	-0.00	0.00
43 ->	16	-0.00	0.00
43 ->	17	-0.00	0.00
43 ->	18	-0.01	0.01
43 ->	19	-0.01	0.01
43 ->	20	-0.01	0.01
43 ->	21	-0.04	0.03
43 ->	22	-0.70	0.66
43 ->	23	-0.01	0.01
43 ->	24	-0.02	0.02
43 ->	25	-2.67	2.33
43 ->	26	-0.49	0.34
43 ->	27	-0.01	0.01
43 ->	28	-0.01	0.01
43 ->	29	-0.02	0.02
43 ->	30	-0.67	0.70
43 ->	31	-1.77	1.41
43 ->	32	-0.53	0.37
43 ->	33	-0.86	0.23
43 ->	34	-0.04	0.01
43 ->	35	0.01	0.01
43 ->	36	-0.00	0.00
43 ->	37	-0.00	0.00
43 ->	38	0.00	0.00
43 ->	39	-0.01	0.00
43 ->	40	-0.07	0.03
43 ->	41	-0.60	0.12
43 ->	42	-17.23	0.30
43 ->	43	22.77	1.41
43 ->	44	-6.44	0.57
43 ->	45	-0.21	0.04
43 ->	46	-0.16	0.09
43 ->	47	-1.51	0.72
43 ->	48	-0.04	0.02
43 ->	49	-0.00	0.00
43 ->	50	-0.01	0.00
43 ->	51	-0.01	0.00
43 ->	52	-0.00	0.00

43	->	53	-0.00	0.00
43	->	54	-0.00	0.00
43	->	55	0.00	0.00
43	->	56	0.00	0.00
43	->	57	0.00	0.00
43	->	58	-0.00	0.00
43	->	59	0.00	0.00
43	->	60	0.00	0.00
43	->	61	-0.00	0.00
43	->	62	-0.00	0.00
43	->	63	-0.00	0.00
43	->	64	-0.00	0.00
43	->	65	-0.00	0.00
43	->	66	-0.00	0.00
43	->	67	0.00	0.00
43	->	68	0.00	0.00
43	->	69	0.00	0.00
43	->	70	0.00	0.00
43	->	71	-0.00	0.00
43	->	72	-0.00	0.00
43	->	73	-0.00	0.00
43	->	74	-0.00	0.00
43	->	75	-0.00	0.00
43	->	76	0.00	0.00
43	->	77	0.00	0.00
43	->	78	0.00	0.00
43	->	79	0.00	0.00
43	->	80	0.00	0.00
43	->	81	0.00	0.00
43	->	82	0.00	0.00
43	->	83	0.00	0.00
43	->	84	0.00	0.00
43	->	85	0.00	0.00
43	->	86	-0.00	0.00
43	->	87	-0.00	0.00
43	->	88	-0.00	0.01
43	->	89	0.01	0.04
43	->	90	-0.40	0.43
43	->	91	-2.59	1.98
43	->	92	-0.08	0.07
43	->	93	-0.02	0.01
43	->	94	-0.00	0.00
43	->	95	-0.00	0.00
43	->	96	-0.00	0.00
43	->	97	-0.00	0.00
43	->	98	-0.01	0.02
43	->	99	-0.03	0.03
43	->	100	-0.01	0.01
43	->	101	0.00	0.01
43	->	102	-0.00	0.00

43	->	103	-0.01	0.00
43	->	104	-0.00	0.00
43	->	105	-0.00	0.00
43	->	106	-0.00	0.00
43	->	107	-0.00	0.00
44	->	1	0.07	0.02
44	->	2	0.00	0.00
44	->	3	-0.00	0.00
44	->	4	-0.00	0.00
44	->	5	-0.03	0.00
44	->	6	0.00	0.00
44	->	7	-0.00	0.00
44	->	8	-0.01	0.00
44	->	9	0.00	0.00
44	->	10	-0.02	0.00
44	->	11	-0.01	0.00
44	->	12	0.06	0.03
44	->	13	0.02	0.00
44	->	14	-0.01	0.01
44	->	15	0.00	0.01
44	->	16	0.25	0.22
44	->	17	-0.00	0.02
44	->	18	-4.09	5.45
44	->	19	-0.58	0.65
44	->	20	-0.01	0.02
44	->	21	0.08	0.12
44	->	22	-0.44	0.93
44	->	23	-0.05	0.03
44	->	24	-0.00	0.01
44	->	25	-0.27	0.37
44	->	26	-0.06	0.03
44	->	27	-0.00	0.00
44	->	28	-0.00	0.00
44	->	29	-0.01	0.00
44	->	30	-0.01	0.01
44	->	31	-0.04	0.06
44	->	32	-0.12	0.07
44	->	33	-2.81	0.46
44	->	34	-0.02	0.01
44	->	35	-0.04	0.01
44	->	36	0.01	0.00
44	->	37	-0.00	0.00
44	->	38	-0.02	0.00
44	->	39	-0.00	0.00
44	->	40	0.01	0.01
44	->	41	-0.04	0.03
44	->	42	-0.95	0.21
44	->	43	-6.54	0.57
44	->	44	-93.35	3.30
44	->	45	-21.47	0.53

44 ->	46	-11.52	7.03
44 ->	47	-4.99	0.93
44 ->	48	-1.21	0.42
44 ->	49	-0.01	0.06
44 ->	50	-0.04	0.04
44 ->	51	-0.01	0.01
44 ->	52	-0.00	0.01
44 ->	53	0.05	0.02
44 ->	54	-0.00	0.00
44 ->	55	-0.00	0.00
44 ->	56	0.00	0.00
44 ->	57	0.00	0.00
44 ->	58	-0.02	0.01
44 ->	59	0.01	0.00
44 ->	60	0.00	0.00
44 ->	61	-0.00	0.00
44 ->	62	-0.00	0.00
44 ->	63	-0.01	0.00
44 ->	64	-0.01	0.00
44 ->	65	-0.00	0.00
44 ->	66	-0.01	0.00
44 ->	67	-0.00	0.00
44 ->	68	0.01	0.00
44 ->	69	0.01	0.00
44 ->	70	0.00	0.00
44 ->	71	-0.00	0.00
44 ->	72	0.07	0.03
44 ->	73	-0.00	0.01
44 ->	74	-0.05	0.03
44 ->	75	-0.02	0.01
44 ->	76	0.02	0.01
44 ->	77	0.02	0.01
44 ->	78	0.02	0.00
44 ->	79	0.00	0.00
44 ->	80	0.00	0.00
44 ->	81	0.01	0.00
44 ->	82	0.00	0.00
44 ->	83	0.00	0.00
44 ->	84	0.00	0.00
44 ->	85	0.00	0.00
44 ->	86	-0.00	0.00
44 ->	87	-0.01	0.00
44 ->	88	-0.03	0.01
44 ->	89	-0.05	0.02
44 ->	90	-0.15	0.12
44 ->	91	-1.29	1.51
44 ->	92	-4.71	3.91
44 ->	93	-3.34	3.07
44 ->	94	0.07	0.61
44 ->	95	-0.11	0.12

44	->	96	-0.03	0.01
44	->	97	-0.06	0.10
44	->	98	-0.92	1.41
44	->	99	-1.30	1.49
44	->	100	-2.18	1.41
44	->	101	0.74	0.95
44	->	102	0.10	0.42
44	->	103	-0.12	0.10
44	->	104	-0.06	0.04
44	->	105	-0.03	0.02
44	->	106	-0.02	0.01
44	->	107	-0.01	0.00
45	->	1	0.00	0.00
45	->	2	0.00	0.00
45	->	3	0.00	0.00
45	->	4	-0.00	0.00
45	->	5	-0.00	0.00
45	->	6	0.00	0.00
45	->	7	0.00	0.00
45	->	8	-0.00	0.00
45	->	9	0.00	0.00
45	->	10	-0.00	0.00
45	->	11	-0.00	0.00
45	->	12	-0.00	0.00
45	->	13	0.00	0.00
45	->	14	-0.00	0.00
45	->	15	0.00	0.00
45	->	16	-0.00	0.00
45	->	17	-0.00	0.00
45	->	18	-0.01	0.01
45	->	19	-0.00	0.00
45	->	20	-0.00	0.00
45	->	21	-0.01	0.00
45	->	22	-0.06	0.03
45	->	23	0.00	0.00
45	->	24	-0.00	0.00
45	->	25	-0.11	0.08
45	->	26	0.00	0.00
45	->	27	0.00	0.00
45	->	28	-0.00	0.00
45	->	29	-0.00	0.00
45	->	30	-0.01	0.00
45	->	31	-0.06	0.03
45	->	32	-0.07	0.02
45	->	33	-2.26	0.36
45	->	34	-0.02	0.01
45	->	35	0.00	0.01
45	->	36	-0.01	0.00
45	->	37	-0.00	0.00
45	->	38	0.00	0.00

45	->	39	-0.00	0.00
45	->	40	-0.01	0.00
45	->	41	-0.05	0.01
45	->	42	-0.18	0.05
45	->	43	-0.21	0.04
45	->	44	-21.48	0.52
45	->	45	23.62	2.37
45	->	46	-10.31	0.59
45	->	47	-1.04	0.19
45	->	48	-2.35	0.35
45	->	49	-2.84	1.53
45	->	50	-0.11	0.04
45	->	51	-0.06	0.03
45	->	52	-0.06	0.03
45	->	53	-0.02	0.01
45	->	54	-0.00	0.00
45	->	55	-0.00	0.00
45	->	56	-0.00	0.00
45	->	57	-0.00	0.00
45	->	58	0.00	0.00
45	->	59	-0.00	0.00
45	->	60	-0.00	0.00
45	->	61	-0.00	0.00
45	->	62	0.00	0.00
45	->	63	0.00	0.00
45	->	64	0.00	0.00
45	->	65	0.00	0.00
45	->	66	0.00	0.00
45	->	67	0.00	0.00
45	->	68	-0.00	0.00
45	->	69	-0.00	0.00
45	->	70	0.00	0.00
45	->	71	-0.00	0.00
45	->	72	-0.01	0.00
45	->	73	-0.00	0.00
45	->	74	0.00	0.00
45	->	75	0.00	0.00
45	->	76	-0.00	0.00
45	->	77	-0.00	0.00
45	->	78	-0.00	0.00
45	->	79	-0.00	0.00
45	->	80	-0.00	0.00
45	->	81	-0.00	0.00
45	->	82	0.00	0.00
45	->	83	0.00	0.00
45	->	84	0.00	0.00
45	->	85	-0.00	0.00
45	->	86	0.00	0.00
45	->	87	0.00	0.00
45	->	88	0.00	0.00

45	->	89	-0.00	0.00
45	->	90	-0.00	0.01
45	->	91	0.00	0.01
45	->	92	-0.00	0.01
45	->	93	-0.00	0.00
45	->	94	-0.01	0.00
45	->	95	-0.01	0.01
45	->	96	-0.00	0.00
45	->	97	-0.01	0.00
45	->	98	-0.31	0.33
45	->	99	-3.66	2.84
45	->	100	-1.12	1.83
45	->	101	-0.04	0.03
45	->	102	-0.01	0.00
45	->	103	-0.00	0.00
45	->	104	-0.00	0.00
45	->	105	-0.00	0.00
45	->	106	-0.00	0.00
45	->	107	0.00	0.00
46	->	1	-0.03	0.01
46	->	2	-0.00	0.00
46	->	3	0.00	0.00
46	->	4	0.00	0.00
46	->	5	0.01	0.00
46	->	6	-0.00	0.00
46	->	7	-0.00	0.00
46	->	8	0.00	0.00
46	->	9	0.00	0.00
46	->	10	0.01	0.00
46	->	11	0.01	0.00
46	->	12	-0.03	0.01
46	->	13	-0.01	0.00
46	->	14	-0.00	0.00
46	->	15	-0.00	0.00
46	->	16	-0.06	0.02
46	->	17	-0.00	0.00
46	->	18	0.06	0.02
46	->	19	0.05	0.03
46	->	20	-0.00	0.00
46	->	21	-0.01	0.00
46	->	22	-0.29	0.24
46	->	23	-0.00	0.00
46	->	24	-0.00	0.00
46	->	25	-0.09	0.04
46	->	26	-0.00	0.00
46	->	27	-0.00	0.00
46	->	28	-0.00	0.00
46	->	29	-0.00	0.00
46	->	30	0.00	0.00
46	->	31	-0.05	0.02

46	->	32	-0.02	0.01
46	->	33	0.02	0.03
46	->	34	-0.00	0.00
46	->	35	0.04	0.01
46	->	36	-0.03	0.01
46	->	37	0.00	0.00
46	->	38	0.03	0.00
46	->	39	-0.00	0.00
46	->	40	-0.03	0.01
46	->	41	-0.02	0.01
46	->	42	-0.12	0.03
46	->	43	-0.16	0.09
46	->	44	-11.48	6.99
46	->	45	-10.37	0.59
46	->	46	-15.96	7.26
46	->	47	-13.17	0.41
46	->	48	-1.28	0.18
46	->	49	-2.93	0.47
46	->	50	-2.23	1.14
46	->	51	-0.15	0.09
46	->	52	-0.07	0.03
46	->	53	-0.27	0.15
46	->	54	-0.01	0.00
46	->	55	-0.01	0.00
46	->	56	-0.01	0.00
46	->	57	-0.00	0.00
46	->	58	0.04	0.01
46	->	59	-0.03	0.01
46	->	60	-0.01	0.00
46	->	61	-0.01	0.01
46	->	62	0.00	0.00
46	->	63	0.01	0.00
46	->	64	0.01	0.00
46	->	65	0.00	0.00
46	->	66	0.01	0.00
46	->	67	0.00	0.00
46	->	68	-0.02	0.00
46	->	69	-0.02	0.00
46	->	70	-0.00	0.00
46	->	71	-0.01	0.01
46	->	72	-0.11	0.04
46	->	73	-0.01	0.01
46	->	74	0.05	0.02
46	->	75	0.03	0.01
46	->	76	-0.04	0.01
46	->	77	-0.03	0.00
46	->	78	-0.03	0.00
46	->	79	-0.00	0.00
46	->	80	-0.00	0.00
46	->	81	-0.01	0.00

46 ->	82	-0.00	0.00
46 ->	83	0.00	0.00
46 ->	84	-0.00	0.00
46 ->	85	-0.00	0.00
46 ->	86	0.00	0.00
46 ->	87	0.02	0.00
46 ->	88	0.02	0.01
46 ->	89	0.03	0.01
46 ->	90	-0.04	0.06
46 ->	91	0.09	0.15
46 ->	92	0.46	0.35
46 ->	93	0.25	0.29
46 ->	94	-0.00	0.03
46 ->	95	-0.01	0.02
46 ->	96	0.01	0.00
46 ->	97	0.01	0.00
46 ->	98	0.08	0.06
46 ->	99	-0.06	0.12
46 ->	100	-1.50	0.67
46 ->	101	-3.60	1.99
46 ->	102	-0.69	0.58
46 ->	103	-0.04	0.07
46 ->	104	0.01	0.01
46 ->	105	0.02	0.00
46 ->	106	0.01	0.00
46 ->	107	0.01	0.00
47 ->	1	0.00	0.00
47 ->	2	0.00	0.00
47 ->	3	-0.00	0.00
47 ->	4	0.00	0.00
47 ->	5	-0.00	0.00
47 ->	6	0.00	0.00
47 ->	7	-0.00	0.00
47 ->	8	-0.00	0.00
47 ->	9	-0.00	0.00
47 ->	10	-0.00	0.00
47 ->	11	-0.00	0.00
47 ->	12	0.00	0.00
47 ->	13	0.00	0.00
47 ->	14	-0.00	0.00
47 ->	15	-0.00	0.00
47 ->	16	-0.00	0.00
47 ->	17	-0.00	0.00
47 ->	18	-0.01	0.01
47 ->	19	-0.01	0.01
47 ->	20	-0.00	0.00
47 ->	21	-0.01	0.00
47 ->	22	-0.26	0.19
47 ->	23	-0.00	0.00
47 ->	24	-0.00	0.00

47	->	25	-0.05	0.03
47	->	26	-0.03	0.04
47	->	27	-0.00	0.00
47	->	28	-0.00	0.00
47	->	29	-0.00	0.00
47	->	30	-0.01	0.00
47	->	31	-0.04	0.05
47	->	32	-0.01	0.01
47	->	33	-0.14	0.05
47	->	34	0.02	0.01
47	->	35	-0.13	0.03
47	->	36	-0.01	0.00
47	->	37	-0.01	0.00
47	->	38	-0.03	0.01
47	->	39	0.00	0.00
47	->	40	0.01	0.02
47	->	41	0.04	0.03
47	->	42	-2.27	0.42
47	->	43	-1.49	0.70
47	->	44	-4.92	0.91
47	->	45	-1.05	0.19
47	->	46	-13.24	0.41
47	->	47	20.43	1.58
47	->	48	-6.35	0.52
47	->	49	-0.91	0.16
47	->	50	-1.74	0.46
47	->	51	-1.70	0.54
47	->	52	-0.01	0.03
47	->	53	-0.02	0.02
47	->	54	-0.02	0.01
47	->	55	-0.03	0.01
47	->	56	-0.00	0.00
47	->	57	-0.00	0.00
47	->	58	0.00	0.00
47	->	59	-0.00	0.00
47	->	60	-0.00	0.00
47	->	61	-0.00	0.00
47	->	62	-0.00	0.00
47	->	63	0.00	0.00
47	->	64	-0.00	0.00
47	->	65	0.00	0.00
47	->	66	0.00	0.00
47	->	67	0.00	0.00
47	->	68	-0.00	0.00
47	->	69	-0.00	0.00
47	->	70	-0.00	0.00
47	->	71	-0.01	0.00
47	->	72	-0.04	0.02
47	->	73	-0.00	0.01
47	->	74	-0.01	0.02

47	->	75	-0.00	0.00
47	->	76	-0.00	0.00
47	->	77	0.00	0.00
47	->	78	-0.00	0.00
47	->	79	-0.00	0.00
47	->	80	0.00	0.00
47	->	81	0.00	0.00
47	->	82	-0.00	0.00
47	->	83	0.00	0.00
47	->	84	0.00	0.00
47	->	85	-0.00	0.00
47	->	86	-0.00	0.00
47	->	87	-0.00	0.00
47	->	88	-0.05	0.02
47	->	89	-0.22	0.12
47	->	90	-1.88	1.45
47	->	91	-2.33	0.83
47	->	92	-1.47	1.72
47	->	93	-0.11	0.11
47	->	94	-0.02	0.01
47	->	95	-0.01	0.00
47	->	96	-0.00	0.00
47	->	97	-0.00	0.00
47	->	98	-0.00	0.01
47	->	99	-0.01	0.02
47	->	100	-0.05	0.03
47	->	101	-0.16	0.06
47	->	102	-0.08	0.09
47	->	103	-0.14	0.19
47	->	104	-0.06	0.04
47	->	105	-0.01	0.00
47	->	106	-0.01	0.00
47	->	107	-0.00	0.00
48	->	1	0.00	0.00
48	->	2	0.00	0.00
48	->	3	0.00	0.00
48	->	4	0.00	0.00
48	->	5	-0.00	0.00
48	->	6	0.00	0.00
48	->	7	0.00	0.00
48	->	8	-0.00	0.00
48	->	9	0.00	0.00
48	->	10	-0.00	0.00
48	->	11	-0.00	0.00
48	->	12	0.00	0.00
48	->	13	0.00	0.00
48	->	14	0.00	0.00
48	->	15	0.00	0.00
48	->	16	0.00	0.00
48	->	17	-0.00	0.00

48 ->	18	-0.00	0.00
48 ->	19	-0.00	0.00
48 ->	20	-0.00	0.00
48 ->	21	-0.00	0.00
48 ->	22	-0.01	0.01
48 ->	23	-0.00	0.00
48 ->	24	-0.00	0.00
48 ->	25	-0.01	0.00
48 ->	26	-0.00	0.00
48 ->	27	0.00	0.00
48 ->	28	-0.00	0.00
48 ->	29	-0.00	0.00
48 ->	30	-0.01	0.00
48 ->	31	-0.03	0.02
48 ->	32	-0.05	0.02
48 ->	33	-1.72	0.35
48 ->	34	-1.21	0.24
48 ->	35	-1.10	0.23
48 ->	36	-0.17	0.13
48 ->	37	-0.03	0.01
48 ->	38	-0.01	0.01
48 ->	39	-0.02	0.01
48 ->	40	-0.15	0.06
48 ->	41	-0.10	0.03
48 ->	42	-2.58	0.39
48 ->	43	-0.04	0.02
48 ->	44	-1.22	0.43
48 ->	45	-2.37	0.35
48 ->	46	-1.28	0.18
48 ->	47	-6.40	0.53
48 ->	48	22.17	1.08
48 ->	49	-6.23	0.52
48 ->	50	-0.99	0.19
48 ->	51	-3.06	0.61
48 ->	52	-4.13	0.66
48 ->	53	-0.20	0.08
48 ->	54	-0.08	0.05
48 ->	55	-0.06	0.03
48 ->	56	-0.02	0.01
48 ->	57	-0.00	0.00
48 ->	58	-0.00	0.01
48 ->	59	-0.00	0.00
48 ->	60	-0.00	0.00
48 ->	61	-0.00	0.00
48 ->	62	-0.00	0.00
48 ->	63	0.00	0.00
48 ->	64	0.00	0.00
48 ->	65	0.00	0.00
48 ->	66	0.00	0.00
48 ->	67	0.00	0.00

48	->	68	-0.00	0.00
48	->	69	-0.00	0.00
48	->	70	-0.00	0.00
48	->	71	-0.01	0.00
48	->	72	-0.02	0.01
48	->	73	-0.00	0.00
48	->	74	0.00	0.00
48	->	75	0.00	0.00
48	->	76	-0.00	0.00
48	->	77	-0.00	0.00
48	->	78	-0.00	0.00
48	->	79	-0.00	0.00
48	->	80	0.00	0.00
48	->	81	-0.00	0.00
48	->	82	-0.00	0.00
48	->	83	0.00	0.00
48	->	84	0.00	0.00
48	->	85	-0.00	0.00
48	->	86	-0.00	0.00
48	->	87	-0.00	0.00
48	->	88	0.01	0.01
48	->	89	0.01	0.03
48	->	90	-0.03	0.04
48	->	91	0.00	0.01
48	->	92	0.00	0.00
48	->	93	-0.00	0.00
48	->	94	-0.00	0.00
48	->	95	-0.00	0.00
48	->	96	-0.00	0.00
48	->	97	-0.00	0.00
48	->	98	-0.01	0.01
48	->	99	-0.06	0.03
48	->	100	-0.07	0.03
48	->	101	-0.02	0.01
48	->	102	-0.01	0.00
48	->	103	-0.00	0.00
48	->	104	-0.00	0.00
48	->	105	-0.00	0.00
48	->	106	-0.00	0.00
48	->	107	-0.00	0.00
49	->	1	0.00	0.00
49	->	2	0.00	0.00
49	->	3	-0.00	0.00
49	->	4	-0.00	0.00
49	->	5	-0.00	0.00
49	->	6	0.00	0.00
49	->	7	0.00	0.00
49	->	8	-0.00	0.00
49	->	9	0.00	0.00
49	->	10	-0.00	0.00

49 ->	11	-0.00	0.00
49 ->	12	0.00	0.00
49 ->	13	0.00	0.00
49 ->	14	0.00	0.00
49 ->	15	0.00	0.00
49 ->	16	0.00	0.00
49 ->	17	-0.00	0.00
49 ->	18	-0.00	0.00
49 ->	19	-0.00	0.00
49 ->	20	0.00	0.00
49 ->	21	-0.00	0.00
49 ->	22	0.00	0.00
49 ->	23	0.00	0.00
49 ->	24	0.00	0.00
49 ->	25	0.00	0.00
49 ->	26	-0.00	0.00
49 ->	27	0.00	0.00
49 ->	28	0.00	0.00
49 ->	29	0.00	0.00
49 ->	30	-0.00	0.00
49 ->	31	-0.00	0.00
49 ->	32	-0.00	0.00
49 ->	33	-0.04	0.01
49 ->	34	-0.01	0.00
49 ->	35	0.02	0.02
49 ->	36	-0.02	0.02
49 ->	37	-0.01	0.00
49 ->	38	0.00	0.00
49 ->	39	-0.00	0.00
49 ->	40	-0.01	0.00
49 ->	41	-0.01	0.01
49 ->	42	-0.07	0.02
49 ->	43	-0.00	0.00
49 ->	44	-0.01	0.06
49 ->	45	-2.84	1.52
49 ->	46	-2.94	0.48
49 ->	47	-0.92	0.16
49 ->	48	-6.24	0.52
49 ->	49	-21.09	2.36
49 ->	50	-7.90	0.52
49 ->	51	-0.91	0.15
49 ->	52	-2.76	0.48
49 ->	53	-3.32	0.77
49 ->	54	-0.15	0.08
49 ->	55	-0.05	0.02
49 ->	56	-0.03	0.02
49 ->	57	-0.01	0.00
49 ->	58	-0.03	0.02
49 ->	59	-0.00	0.00
49 ->	60	-0.02	0.01

49	->	61	-0.02	0.01
49	->	62	-0.00	0.00
49	->	63	0.00	0.00
49	->	64	-0.00	0.00
49	->	65	0.00	0.00
49	->	66	0.00	0.00
49	->	67	0.00	0.00
49	->	68	-0.00	0.00
49	->	69	0.00	0.00
49	->	70	0.00	0.00
49	->	71	-0.04	0.02
49	->	72	0.02	0.02
49	->	73	-0.01	0.01
49	->	74	-0.01	0.01
49	->	75	-0.00	0.00
49	->	76	0.00	0.00
49	->	77	-0.00	0.00
49	->	78	-0.00	0.00
49	->	79	0.00	0.00
49	->	80	-0.00	0.00
49	->	81	0.00	0.00
49	->	82	0.00	0.00
49	->	83	0.00	0.00
49	->	84	0.00	0.00
49	->	85	0.00	0.00
49	->	86	-0.00	0.00
49	->	87	-0.00	0.00
49	->	88	-0.02	0.02
49	->	89	-0.03	0.02
49	->	90	-0.02	0.01
49	->	91	-0.01	0.00
49	->	92	-0.01	0.00
49	->	93	-0.00	0.00
49	->	94	-0.00	0.00
49	->	95	-0.00	0.00
49	->	96	-0.00	0.00
49	->	97	-0.00	0.00
49	->	98	-0.01	0.00
49	->	99	-0.05	0.03
49	->	100	-0.27	0.12
49	->	101	-0.21	0.16
49	->	102	-0.02	0.01
49	->	103	-0.00	0.00
49	->	104	-0.00	0.00
49	->	105	-0.00	0.00
49	->	106	-0.00	0.00
49	->	107	-0.00	0.00
50	->	1	0.00	0.00
50	->	2	0.00	0.00
50	->	3	-0.00	0.00

50	->	4	0.00	0.00
50	->	5	-0.00	0.00
50	->	6	0.00	0.00
50	->	7	0.00	0.00
50	->	8	-0.00	0.00
50	->	9	0.00	0.00
50	->	10	-0.00	0.00
50	->	11	-0.00	0.00
50	->	12	0.00	0.00
50	->	13	0.00	0.00
50	->	14	0.00	0.00
50	->	15	0.00	0.00
50	->	16	0.00	0.00
50	->	17	-0.00	0.00
50	->	18	-0.00	0.00
50	->	19	-0.00	0.00
50	->	20	-0.00	0.00
50	->	21	-0.00	0.00
50	->	22	0.00	0.00
50	->	23	0.00	0.00
50	->	24	0.00	0.00
50	->	25	0.00	0.00
50	->	26	-0.00	0.00
50	->	27	0.00	0.00
50	->	28	0.00	0.00
50	->	29	0.00	0.00
50	->	30	-0.00	0.00
50	->	31	0.00	0.00
50	->	32	-0.00	0.00
50	->	33	-0.01	0.00
50	->	34	0.00	0.00
50	->	35	-0.07	0.03
50	->	36	-0.00	0.00
50	->	37	-0.01	0.00
50	->	38	-0.03	0.01
50	->	39	-0.00	0.00
50	->	40	0.01	0.01
50	->	41	0.00	0.01
50	->	42	-0.15	0.06
50	->	43	-0.01	0.00
50	->	44	-0.04	0.04
50	->	45	-0.11	0.04
50	->	46	-2.24	1.14
50	->	47	-1.76	0.47
50	->	48	-1.00	0.19
50	->	49	-7.92	0.52
50	->	50	-21.72	1.79
50	->	51	-21.87	0.43
50	->	52	-0.85	0.16
50	->	53	-3.65	0.94

50	->	54	-1.83	1.09
50	->	55	-0.11	0.05
50	->	56	-0.03	0.02
50	->	57	-0.02	0.01
50	->	58	-0.01	0.02
50	->	59	-0.04	0.01
50	->	60	-0.03	0.02
50	->	61	-0.04	0.06
50	->	62	-0.00	0.00
50	->	63	0.00	0.00
50	->	64	0.00	0.00
50	->	65	0.00	0.00
50	->	66	0.00	0.00
50	->	67	0.00	0.00
50	->	68	-0.00	0.00
50	->	69	-0.01	0.00
50	->	70	-0.03	0.02
50	->	71	-0.49	0.43
50	->	72	-1.32	0.36
50	->	73	-0.03	0.04
50	->	74	-0.03	0.03
50	->	75	-0.01	0.01
50	->	76	-0.01	0.01
50	->	77	-0.00	0.00
50	->	78	-0.00	0.00
50	->	79	-0.00	0.00
50	->	80	-0.00	0.00
50	->	81	0.00	0.00
50	->	82	-0.00	0.00
50	->	83	0.00	0.00
50	->	84	0.00	0.00
50	->	85	0.00	0.00
50	->	86	-0.00	0.00
50	->	87	-0.01	0.01
50	->	88	-0.11	0.08
50	->	89	-0.30	0.26
50	->	90	-0.34	0.33
50	->	91	-0.03	0.02
50	->	92	-0.01	0.01
50	->	93	-0.01	0.00
50	->	94	-0.00	0.00
50	->	95	-0.00	0.00
50	->	96	-0.00	0.00
50	->	97	-0.00	0.00
50	->	98	-0.00	0.00
50	->	99	-0.01	0.00
50	->	100	-0.02	0.02
50	->	101	-0.02	0.03
50	->	102	-0.07	0.05
50	->	103	-0.04	0.05

50	->	104	-0.02	0.02
50	->	105	-0.01	0.00
50	->	106	-0.00	0.00
50	->	107	-0.00	0.00
51	->	1	0.00	0.00
51	->	2	0.00	0.00
51	->	3	-0.00	0.00
51	->	4	0.00	0.00
51	->	5	-0.00	0.00
51	->	6	0.00	0.00
51	->	7	0.00	0.00
51	->	8	-0.00	0.00
51	->	9	0.00	0.00
51	->	10	-0.00	0.00
51	->	11	-0.00	0.00
51	->	12	0.00	0.00
51	->	13	0.00	0.00
51	->	14	0.00	0.00
51	->	15	0.00	0.00
51	->	16	0.00	0.00
51	->	17	-0.00	0.00
51	->	18	-0.00	0.00
51	->	19	-0.00	0.00
51	->	20	-0.00	0.00
51	->	21	-0.00	0.00
51	->	22	0.00	0.00
51	->	23	0.00	0.00
51	->	24	0.00	0.00
51	->	25	0.00	0.00
51	->	26	-0.00	0.00
51	->	27	0.00	0.00
51	->	28	0.00	0.00
51	->	29	-0.00	0.00
51	->	30	-0.00	0.00
51	->	31	-0.00	0.00
51	->	32	-0.00	0.00
51	->	33	-0.02	0.01
51	->	34	-0.10	0.02
51	->	35	2.87	0.66
51	->	36	-0.15	0.05
51	->	37	0.77	0.12
51	->	38	3.80	0.99
51	->	39	-0.10	0.06
51	->	40	-1.44	0.80
51	->	41	-0.02	0.01
51	->	42	-2.18	0.34
51	->	43	-0.01	0.00
51	->	44	-0.01	0.01
51	->	45	-0.06	0.03
51	->	46	-0.15	0.09

51	->	47	-1.74	0.54
51	->	48	-3.06	0.60
51	->	49	-0.92	0.15
51	->	50	-21.91	0.44
51	->	51	11.86	1.67
51	->	52	-10.24	0.58
51	->	53	-1.30	0.28
51	->	54	-3.47	1.32
51	->	55	14.07	8.51
51	->	56	-0.19	0.06
51	->	57	-0.03	0.02
51	->	58	-0.02	0.01
51	->	59	-0.01	0.00
51	->	60	-0.01	0.00
51	->	61	-0.00	0.00
51	->	62	-0.00	0.00
51	->	63	-0.00	0.00
51	->	64	-0.00	0.00
51	->	65	0.00	0.00
51	->	66	-0.00	0.00
51	->	67	0.00	0.00
51	->	68	-0.00	0.00
51	->	69	-0.00	0.00
51	->	70	-0.01	0.00
51	->	71	-0.03	0.02
51	->	72	-0.09	0.04
51	->	73	0.00	0.00
51	->	74	-0.01	0.00
51	->	75	-0.01	0.00
51	->	76	0.00	0.00
51	->	77	0.00	0.00
51	->	78	0.00	0.00
51	->	79	-0.00	0.00
51	->	80	0.00	0.00
51	->	81	0.00	0.00
51	->	82	0.00	0.00
51	->	83	0.00	0.00
51	->	84	0.00	0.00
51	->	85	0.00	0.00
51	->	86	-0.00	0.00
51	->	87	-0.03	0.01
51	->	88	-0.73	0.52
51	->	89	-4.46	3.07
51	->	90	-0.08	0.05
51	->	91	-0.02	0.00
51	->	92	-0.01	0.00
51	->	93	-0.00	0.00
51	->	94	-0.00	0.00
51	->	95	-0.00	0.00
51	->	96	-0.00	0.00

51	->	97	-0.00	0.00
51	->	98	-0.00	0.00
51	->	99	-0.01	0.00
51	->	100	-0.01	0.00
51	->	101	-0.01	0.00
51	->	102	-0.01	0.00
51	->	103	-0.01	0.00
51	->	104	-0.01	0.00
51	->	105	-0.01	0.00
51	->	106	-0.01	0.00
51	->	107	-0.00	0.00
52	->	1	0.00	0.00
52	->	2	0.00	0.00
52	->	3	-0.00	0.00
52	->	4	0.00	0.00
52	->	5	-0.00	0.00
52	->	6	0.00	0.00
52	->	7	0.00	0.00
52	->	8	-0.00	0.00
52	->	9	0.00	0.00
52	->	10	-0.00	0.00
52	->	11	-0.00	0.00
52	->	12	0.00	0.00
52	->	13	0.00	0.00
52	->	14	0.00	0.00
52	->	15	0.00	0.00
52	->	16	0.00	0.00
52	->	17	-0.00	0.00
52	->	18	-0.00	0.00
52	->	19	-0.00	0.00
52	->	20	-0.00	0.00
52	->	21	-0.00	0.00
52	->	22	0.00	0.00
52	->	23	0.00	0.00
52	->	24	0.00	0.00
52	->	25	0.00	0.00
52	->	26	0.00	0.00
52	->	27	0.00	0.00
52	->	28	0.00	0.00
52	->	29	0.00	0.00
52	->	30	-0.00	0.00
52	->	31	-0.00	0.00
52	->	32	-0.00	0.00
52	->	33	-0.02	0.01
52	->	34	-0.05	0.02
52	->	35	-0.31	0.27
52	->	36	-0.58	0.89
52	->	37	-0.12	0.12
52	->	38	0.06	0.02
52	->	39	-0.01	0.01

52	->	40	-0.05	0.02
52	->	41	-0.01	0.01
52	->	42	-0.07	0.03
52	->	43	-0.00	0.00
52	->	44	-0.00	0.01
52	->	45	-0.06	0.03
52	->	46	-0.07	0.03
52	->	47	-0.01	0.03
52	->	48	-4.10	0.65
52	->	49	-2.78	0.48
52	->	50	-0.86	0.16
52	->	51	-10.29	0.58
52	->	52	23.45	1.77
52	->	53	-10.31	0.56
52	->	54	-1.14	0.22
52	->	55	-3.04	0.69
52	->	56	-4.68	1.46
52	->	57	-0.17	0.07
52	->	58	-0.25	0.13
52	->	59	-0.01	0.01
52	->	60	-0.01	0.00
52	->	61	-0.01	0.00
52	->	62	-0.00	0.00
52	->	63	0.00	0.00
52	->	64	0.00	0.00
52	->	65	0.00	0.00
52	->	66	0.00	0.00
52	->	67	0.00	0.00
52	->	68	-0.00	0.00
52	->	69	-0.00	0.00
52	->	70	-0.00	0.00
52	->	71	-0.02	0.01
52	->	72	-0.02	0.01
52	->	73	-0.00	0.00
52	->	74	-0.00	0.00
52	->	75	-0.00	0.00
52	->	76	-0.00	0.00
52	->	77	0.00	0.00
52	->	78	-0.00	0.00
52	->	79	0.00	0.00
52	->	80	0.00	0.00
52	->	81	-0.00	0.00
52	->	82	-0.00	0.00
52	->	83	0.00	0.00
52	->	84	0.00	0.00
52	->	85	0.00	0.00
52	->	86	-0.00	0.00
52	->	87	0.00	0.01
52	->	88	0.01	0.02
52	->	89	0.06	0.05

52	->	90	-0.01	0.00
52	->	91	-0.01	0.00
52	->	92	-0.00	0.00
52	->	93	-0.00	0.00
52	->	94	-0.00	0.00
52	->	95	-0.00	0.00
52	->	96	-0.00	0.00
52	->	97	-0.00	0.00
52	->	98	-0.00	0.00
52	->	99	-0.01	0.00
52	->	100	-0.02	0.01
52	->	101	-0.01	0.01
52	->	102	-0.00	0.00
52	->	103	-0.00	0.00
52	->	104	-0.00	0.00
52	->	105	-0.00	0.00
52	->	106	-0.00	0.00
52	->	107	-0.00	0.00
53	->	1	0.01	0.00
53	->	2	0.00	0.00
53	->	3	-0.00	0.00
53	->	4	-0.00	0.00
53	->	5	-0.00	0.00
53	->	6	0.00	0.00
53	->	7	0.00	0.00
53	->	8	-0.00	0.00
53	->	9	0.00	0.00
53	->	10	-0.01	0.00
53	->	11	-0.00	0.00
53	->	12	0.01	0.00
53	->	13	0.00	0.00
53	->	14	0.00	0.00
53	->	15	0.00	0.00
53	->	16	0.02	0.00
53	->	17	-0.00	0.00
53	->	18	-0.02	0.00
53	->	19	-0.02	0.00
53	->	20	-0.00	0.00
53	->	21	0.00	0.00
53	->	22	0.02	0.00
53	->	23	0.00	0.00
53	->	24	0.00	0.00
53	->	25	0.02	0.00
53	->	26	0.00	0.00
53	->	27	0.00	0.00
53	->	28	0.00	0.00
53	->	29	0.00	0.00
53	->	30	-0.00	0.00
53	->	31	0.02	0.01
53	->	32	-0.00	0.00

53	->	33	-0.00	0.00
53	->	34	-0.00	0.00
53	->	35	-0.06	0.02
53	->	36	0.02	0.01
53	->	37	-0.01	0.00
53	->	38	-0.05	0.01
53	->	39	-0.00	0.00
53	->	40	0.02	0.01
53	->	41	-0.00	0.00
53	->	42	-0.02	0.01
53	->	43	-0.00	0.00
53	->	44	0.05	0.02
53	->	45	-0.02	0.01
53	->	46	-0.28	0.15
53	->	47	-0.02	0.02
53	->	48	-0.20	0.08
53	->	49	-3.35	0.78
53	->	50	-3.67	0.93
53	->	51	-1.31	0.27
53	->	52	-10.33	0.57
53	->	53	-105.14	2.41
53	->	54	-5.11	0.61
53	->	55	-0.70	0.22
53	->	56	-1.49	0.29
53	->	57	-1.19	0.33
53	->	58	-4.46	2.04
53	->	59	0.02	0.12
53	->	60	-1.33	0.55
53	->	61	-0.56	0.41
53	->	62	-0.01	0.00
53	->	63	-0.03	0.00
53	->	64	-0.02	0.00
53	->	65	-0.00	0.00
53	->	66	-0.02	0.00
53	->	67	-0.00	0.00
53	->	68	0.03	0.01
53	->	69	0.08	0.02
53	->	70	0.03	0.04
53	->	71	-2.57	1.81
53	->	72	-0.32	0.35
53	->	73	-0.11	0.09
53	->	74	-0.09	0.04
53	->	75	-0.14	0.10
53	->	76	0.04	0.01
53	->	77	0.02	0.01
53	->	78	0.03	0.01
53	->	79	0.00	0.00
53	->	80	0.00	0.00
53	->	81	0.01	0.00
53	->	82	0.00	0.00

53	->	83	-0.00	0.00
53	->	84	0.00	0.00
53	->	85	0.00	0.00
53	->	86	-0.01	0.00
53	->	87	-0.09	0.06
53	->	88	-0.30	0.27
53	->	89	-0.13	0.11
53	->	90	-0.04	0.02
53	->	91	-0.03	0.01
53	->	92	-0.02	0.00
53	->	93	-0.01	0.00
53	->	94	-0.02	0.00
53	->	95	-0.01	0.00
53	->	96	-0.01	0.00
53	->	97	-0.00	0.00
53	->	98	-0.02	0.01
53	->	99	-0.07	0.02
53	->	100	-0.56	0.53
53	->	101	-2.99	3.21
53	->	102	-0.28	0.88
53	->	103	-0.03	0.01
53	->	104	-0.02	0.00
53	->	105	-0.01	0.00
53	->	106	-0.01	0.00
53	->	107	-0.01	0.00
54	->	1	0.00	0.00
54	->	2	0.00	0.00
54	->	3	0.00	0.00
54	->	4	0.00	0.00
54	->	5	-0.00	0.00
54	->	6	0.00	0.00
54	->	7	0.00	0.00
54	->	8	-0.00	0.00
54	->	9	0.00	0.00
54	->	10	-0.00	0.00
54	->	11	-0.00	0.00
54	->	12	0.00	0.00
54	->	13	0.00	0.00
54	->	14	0.00	0.00
54	->	15	0.00	0.00
54	->	16	0.00	0.00
54	->	17	-0.00	0.00
54	->	18	-0.00	0.00
54	->	19	-0.00	0.00
54	->	20	0.00	0.00
54	->	21	-0.00	0.00
54	->	22	0.00	0.00
54	->	23	0.00	0.00
54	->	24	0.00	0.00
54	->	25	0.00	0.00

54 ->	26	0.00	0.00
54 ->	27	0.00	0.00
54 ->	28	0.00	0.00
54 ->	29	0.00	0.00
54 ->	30	-0.00	0.00
54 ->	31	0.00	0.00
54 ->	32	-0.00	0.00
54 ->	33	-0.00	0.00
54 ->	34	-0.00	0.00
54 ->	35	-0.07	0.05
54 ->	36	-0.00	0.00
54 ->	37	-0.03	0.01
54 ->	38	-0.04	0.06
54 ->	39	-0.00	0.00
54 ->	40	-0.01	0.03
54 ->	41	-0.00	0.00
54 ->	42	-0.02	0.02
54 ->	43	-0.00	0.00
54 ->	44	-0.00	0.00
54 ->	45	-0.00	0.00
54 ->	46	-0.01	0.00
54 ->	47	-0.02	0.01
54 ->	48	-0.08	0.05
54 ->	49	-0.15	0.08
54 ->	50	-1.84	1.08
54 ->	51	-3.46	1.33
54 ->	52	-1.15	0.22
54 ->	53	-5.15	0.60
54 ->	54	-22.76	2.26
54 ->	55	-22.32	0.45
54 ->	56	-0.57	0.13
54 ->	57	-0.96	0.37
54 ->	58	-0.19	0.12
54 ->	59	-0.19	0.13
54 ->	60	-0.02	0.01
54 ->	61	-0.00	0.00
54 ->	62	-0.00	0.00
54 ->	63	0.00	0.00
54 ->	64	0.00	0.00
54 ->	65	0.00	0.00
54 ->	66	0.00	0.00
54 ->	67	0.00	0.00
54 ->	68	-0.01	0.01
54 ->	69	-0.02	0.01
54 ->	70	-0.04	0.04
54 ->	71	-0.47	0.43
54 ->	72	-0.29	0.33
54 ->	73	0.00	0.01
54 ->	74	-0.00	0.01
54 ->	75	-0.02	0.03

54	->	76	-0.00	0.00
54	->	77	-0.00	0.00
54	->	78	-0.00	0.00
54	->	79	-0.00	0.00
54	->	80	0.00	0.00
54	->	81	0.00	0.00
54	->	82	-0.00	0.00
54	->	83	0.00	0.00
54	->	84	0.00	0.00
54	->	85	0.00	0.00
54	->	86	-0.01	0.00
54	->	87	-0.15	0.14
54	->	88	-1.98	1.52
54	->	89	-0.53	1.18
54	->	90	-0.02	0.00
54	->	91	-0.01	0.00
54	->	92	-0.00	0.00
54	->	93	-0.00	0.00
54	->	94	-0.00	0.00
54	->	95	-0.00	0.00
54	->	96	-0.00	0.00
54	->	97	-0.00	0.00
54	->	98	-0.00	0.00
54	->	99	-0.00	0.00
54	->	100	-0.00	0.00
54	->	101	-0.00	0.00
54	->	102	-0.00	0.00
54	->	103	-0.00	0.00
54	->	104	-0.00	0.00
54	->	105	-0.00	0.00
54	->	106	-0.00	0.00
54	->	107	-0.00	0.00
55	->	1	0.00	0.00
55	->	2	0.00	0.00
55	->	3	-0.00	0.00
55	->	4	0.00	0.00
55	->	5	0.00	0.00
55	->	6	0.00	0.00
55	->	7	0.00	0.00
55	->	8	0.00	0.00
55	->	9	0.00	0.00
55	->	10	0.00	0.00
55	->	11	-0.00	0.00
55	->	12	0.00	0.00
55	->	13	-0.00	0.00
55	->	14	0.00	0.00
55	->	15	0.00	0.00
55	->	16	0.00	0.00
55	->	17	0.00	0.00
55	->	18	-0.00	0.00

55 ->	19	-0.00	0.00
55 ->	20	0.00	0.00
55 ->	21	0.00	0.00
55 ->	22	0.00	0.00
55 ->	23	0.00	0.00
55 ->	24	0.00	0.00
55 ->	25	-0.00	0.00
55 ->	26	0.00	0.00
55 ->	27	-0.00	0.00
55 ->	28	0.00	0.00
55 ->	29	0.00	0.00
55 ->	30	0.00	0.00
55 ->	31	-0.00	0.00
55 ->	32	-0.00	0.00
55 ->	33	-0.01	0.00
55 ->	34	-0.05	0.01
55 ->	35	2.47	0.43
55 ->	36	-0.31	0.13
55 ->	37	0.80	0.63
55 ->	38	2.27	0.45
55 ->	39	-0.03	0.04
55 ->	40	-0.36	0.27
55 ->	41	-0.01	0.00
55 ->	42	-0.00	0.01
55 ->	43	0.00	0.00
55 ->	44	-0.00	0.00
55 ->	45	-0.00	0.00
55 ->	46	-0.01	0.00
55 ->	47	-0.03	0.01
55 ->	48	-0.06	0.03
55 ->	49	-0.05	0.02
55 ->	50	-0.11	0.05
55 ->	51	14.06	8.50
55 ->	52	-3.02	0.69
55 ->	53	-0.71	0.21
55 ->	54	-22.43	0.46
55 ->	55	3.91	1.13
55 ->	56	-4.99	0.39
55 ->	57	-0.49	0.16
55 ->	58	-0.04	0.02
55 ->	59	-0.04	0.02
55 ->	60	-0.00	0.00
55 ->	61	-0.00	0.00
55 ->	62	-0.00	0.00
55 ->	63	-0.00	0.00
55 ->	64	-0.00	0.00
55 ->	65	-0.00	0.00
55 ->	66	-0.00	0.00
55 ->	67	-0.00	0.00
55 ->	68	-0.00	0.00

55	->	69	-0.00	0.00
55	->	70	-0.01	0.00
55	->	71	-0.02	0.01
55	->	72	-0.01	0.01
55	->	73	-0.00	0.00
55	->	74	-0.00	0.00
55	->	75	-0.00	0.00
55	->	76	0.00	0.00
55	->	77	0.00	0.00
55	->	78	0.00	0.00
55	->	79	0.00	0.00
55	->	80	0.00	0.00
55	->	81	0.00	0.00
55	->	82	0.00	0.00
55	->	83	-0.00	0.00
55	->	84	0.00	0.00
55	->	85	0.00	0.00
55	->	86	-0.00	0.00
55	->	87	0.00	0.03
55	->	88	-0.03	0.38
55	->	89	0.06	0.06
55	->	90	-0.00	0.00
55	->	91	-0.00	0.00
55	->	92	-0.00	0.00
55	->	93	-0.00	0.00
55	->	94	-0.00	0.00
55	->	95	-0.00	0.00
55	->	96	-0.00	0.00
55	->	97	0.00	0.00
55	->	98	-0.00	0.00
55	->	99	-0.00	0.00
55	->	100	-0.00	0.00
55	->	101	-0.00	0.00
55	->	102	-0.00	0.00
55	->	103	-0.00	0.00
55	->	104	-0.00	0.00
55	->	105	-0.00	0.00
55	->	106	-0.00	0.00
55	->	107	-0.00	0.00
56	->	1	0.00	0.00
56	->	2	0.00	0.00
56	->	3	0.00	0.00
56	->	4	0.00	0.00
56	->	5	-0.00	0.00
56	->	6	0.00	0.00
56	->	7	0.00	0.00
56	->	8	-0.00	0.00
56	->	9	0.00	0.00
56	->	10	-0.00	0.00
56	->	11	-0.00	0.00

56	->	12	0.00	0.00
56	->	13	0.00	0.00
56	->	14	0.00	0.00
56	->	15	0.00	0.00
56	->	16	0.00	0.00
56	->	17	-0.00	0.00
56	->	18	-0.00	0.00
56	->	19	-0.00	0.00
56	->	20	-0.00	0.00
56	->	21	0.00	0.00
56	->	22	0.00	0.00
56	->	23	0.00	0.00
56	->	24	0.00	0.00
56	->	25	0.00	0.00
56	->	26	0.00	0.00
56	->	27	0.00	0.00
56	->	28	0.00	0.00
56	->	29	0.00	0.00
56	->	30	-0.00	0.00
56	->	31	0.00	0.00
56	->	32	-0.00	0.00
56	->	33	-0.00	0.00
56	->	34	-0.00	0.00
56	->	35	-0.02	0.01
56	->	36	-0.06	0.16
56	->	37	-0.04	0.02
56	->	38	-0.01	0.00
56	->	39	-0.00	0.00
56	->	40	-0.00	0.00
56	->	41	-0.00	0.00
56	->	42	-0.00	0.00
56	->	43	0.00	0.00
56	->	44	0.00	0.00
56	->	45	-0.00	0.00
56	->	46	-0.01	0.00
56	->	47	-0.00	0.00
56	->	48	-0.02	0.01
56	->	49	-0.03	0.02
56	->	50	-0.03	0.02
56	->	51	-0.19	0.06
56	->	52	-4.68	1.46
56	->	53	-1.50	0.29
56	->	54	-0.58	0.13
56	->	55	-5.14	0.39
56	->	56	-24.64	1.90
56	->	57	-11.55	0.53
56	->	58	-2.22	1.14
56	->	59	-0.04	0.02
56	->	60	-0.02	0.00
56	->	61	-0.00	0.00

56	->	62	-0.00	0.00
56	->	63	0.00	0.00
56	->	64	0.00	0.00
56	->	65	0.00	0.00
56	->	66	0.00	0.00
56	->	67	0.00	0.00
56	->	68	-0.00	0.00
56	->	69	-0.01	0.00
56	->	70	-0.00	0.00
56	->	71	-0.02	0.01
56	->	72	-0.00	0.00
56	->	73	-0.00	0.00
56	->	74	-0.00	0.00
56	->	75	-0.00	0.00
56	->	76	-0.00	0.00
56	->	77	0.00	0.00
56	->	78	0.00	0.00
56	->	79	0.00	0.00
56	->	80	0.00	0.00
56	->	81	0.00	0.00
56	->	82	0.00	0.00
56	->	83	0.00	0.00
56	->	84	0.00	0.00
56	->	85	-0.00	0.00
56	->	86	-0.00	0.00
56	->	87	-0.01	0.00
56	->	88	-0.02	0.01
56	->	89	-0.01	0.00
56	->	90	-0.00	0.00
56	->	91	-0.00	0.00
56	->	92	-0.00	0.00
56	->	93	-0.00	0.00
56	->	94	-0.00	0.00
56	->	95	-0.00	0.00
56	->	96	-0.00	0.00
56	->	97	-0.00	0.00
56	->	98	-0.00	0.00
56	->	99	-0.00	0.00
56	->	100	-0.00	0.00
56	->	101	-0.00	0.00
56	->	102	-0.00	0.00
56	->	103	-0.00	0.00
56	->	104	-0.00	0.00
56	->	105	-0.00	0.00
56	->	106	-0.00	0.00
56	->	107	-0.00	0.00
57	->	1	0.00	0.00
57	->	2	0.00	0.00
57	->	3	0.00	0.00
57	->	4	0.00	0.00

57 ->	5	-0.00	0.00
57 ->	6	0.00	0.00
57 ->	7	0.00	0.00
57 ->	8	0.00	0.00
57 ->	9	0.00	0.00
57 ->	10	-0.00	0.00
57 ->	11	-0.00	0.00
57 ->	12	0.00	0.00
57 ->	13	0.00	0.00
57 ->	14	0.00	0.00
57 ->	15	0.00	0.00
57 ->	16	0.00	0.00
57 ->	17	0.00	0.00
57 ->	18	-0.00	0.00
57 ->	19	-0.00	0.00
57 ->	20	0.00	0.00
57 ->	21	0.00	0.00
57 ->	22	0.00	0.00
57 ->	23	0.00	0.00
57 ->	24	0.00	0.00
57 ->	25	0.00	0.00
57 ->	26	0.00	0.00
57 ->	27	0.00	0.00
57 ->	28	0.00	0.00
57 ->	29	0.00	0.00
57 ->	30	-0.00	0.00
57 ->	31	0.00	0.00
57 ->	32	-0.00	0.00
57 ->	33	0.00	0.00
57 ->	34	0.00	0.00
57 ->	35	-0.00	0.00
57 ->	36	-0.00	0.00
57 ->	37	-0.00	0.00
57 ->	38	-0.00	0.00
57 ->	39	0.00	0.00
57 ->	40	0.00	0.00
57 ->	41	-0.00	0.00
57 ->	42	-0.00	0.00
57 ->	43	0.00	0.00
57 ->	44	0.00	0.00
57 ->	45	-0.00	0.00
57 ->	46	-0.00	0.00
57 ->	47	-0.00	0.00
57 ->	48	-0.00	0.00
57 ->	49	-0.01	0.00
57 ->	50	-0.02	0.01
57 ->	51	-0.03	0.02
57 ->	52	-0.17	0.07
57 ->	53	-1.20	0.33
57 ->	54	-1.00	0.38

57 ->	55	-0.51	0.15
57 ->	56	-11.63	0.53
57 ->	57	12.20	0.63
57 ->	58	-15.03	0.41
57 ->	59	-1.39	0.63
57 ->	60	-0.03	0.00
57 ->	61	-0.01	0.00
57 ->	62	-0.00	0.00
57 ->	63	0.00	0.00
57 ->	64	0.00	0.00
57 ->	65	0.00	0.00
57 ->	66	0.00	0.00
57 ->	67	-0.00	0.00
57 ->	68	-0.00	0.00
57 ->	69	-0.02	0.02
57 ->	70	-0.00	0.01
57 ->	71	-0.04	0.01
57 ->	72	-0.01	0.00
57 ->	73	-0.00	0.00
57 ->	74	-0.00	0.00
57 ->	75	-0.01	0.00
57 ->	76	-0.00	0.00
57 ->	77	0.00	0.00
57 ->	78	0.00	0.00
57 ->	79	-0.00	0.00
57 ->	80	0.00	0.00
57 ->	81	0.00	0.00
57 ->	82	0.00	0.00
57 ->	83	0.00	0.00
57 ->	84	0.00	0.00
57 ->	85	-0.00	0.00
57 ->	86	-0.00	0.00
57 ->	87	-0.01	0.01
57 ->	88	-0.04	0.04
57 ->	89	-0.01	0.00
57 ->	90	-0.00	0.00
57 ->	91	-0.00	0.00
57 ->	92	-0.00	0.00
57 ->	93	-0.00	0.00
57 ->	94	-0.00	0.00
57 ->	95	-0.00	0.00
57 ->	96	-0.00	0.00
57 ->	97	-0.00	0.00
57 ->	98	-0.00	0.00
57 ->	99	-0.00	0.00
57 ->	100	-0.00	0.00
57 ->	101	-0.00	0.00
57 ->	102	-0.00	0.00
57 ->	103	-0.00	0.00
57 ->	104	-0.00	0.00

57	->	105	-0.00	0.00
57	->	106	-0.00	0.00
57	->	107	-0.00	0.00
58	->	1	-0.01	0.00
58	->	2	-0.00	0.00
58	->	3	0.00	0.00
58	->	4	0.00	0.00
58	->	5	0.00	0.00
58	->	6	-0.00	0.00
58	->	7	0.00	0.00
58	->	8	0.00	0.00
58	->	9	0.00	0.00
58	->	10	0.00	0.00
58	->	11	0.00	0.00
58	->	12	-0.00	0.00
58	->	13	-0.00	0.00
58	->	14	-0.00	0.00
58	->	15	-0.00	0.00
58	->	16	-0.01	0.00
58	->	17	0.00	0.00
58	->	18	0.01	0.00
58	->	19	0.01	0.00
58	->	20	0.00	0.00
58	->	21	-0.00	0.00
58	->	22	-0.01	0.00
58	->	23	-0.00	0.00
58	->	24	-0.00	0.00
58	->	25	-0.01	0.00
58	->	26	-0.00	0.00
58	->	27	-0.00	0.00
58	->	28	-0.00	0.00
58	->	29	-0.00	0.00
58	->	30	0.00	0.00
58	->	31	-0.01	0.00
58	->	32	0.00	0.00
58	->	33	-0.00	0.00
58	->	34	0.00	0.00
58	->	35	0.03	0.01
58	->	36	-0.04	0.03
58	->	37	-0.00	0.00
58	->	38	0.02	0.00
58	->	39	-0.00	0.00
58	->	40	-0.02	0.00
58	->	41	0.00	0.00
58	->	42	-0.00	0.00
58	->	43	-0.00	0.00
58	->	44	-0.02	0.01
58	->	45	0.00	0.00
58	->	46	0.04	0.01
58	->	47	0.00	0.00

58	->	48	-0.00	0.01
58	->	49	-0.03	0.02
58	->	50	-0.01	0.02
58	->	51	-0.02	0.01
58	->	52	-0.25	0.13
58	->	53	-4.43	2.01
58	->	54	-0.19	0.12
58	->	55	-0.04	0.02
58	->	56	-2.24	1.11
58	->	57	-15.16	0.41
58	->	58	-61.77	3.02
58	->	59	-11.89	0.83
58	->	60	-1.41	0.41
58	->	61	-0.10	0.02
58	->	62	-0.01	0.00
58	->	63	0.02	0.00
58	->	64	0.01	0.00
58	->	65	0.00	0.00
58	->	66	0.02	0.00
58	->	67	0.00	0.00
58	->	68	-0.03	0.01
58	->	69	-0.06	0.09
58	->	70	-0.29	0.05
58	->	71	-0.70	0.24
58	->	72	-0.04	0.01
58	->	73	-0.01	0.00
58	->	74	0.03	0.00
58	->	75	0.05	0.01
58	->	76	-0.04	0.01
58	->	77	-0.02	0.00
58	->	78	-0.02	0.00
58	->	79	-0.00	0.00
58	->	80	-0.00	0.00
58	->	81	-0.01	0.00
58	->	82	-0.00	0.00
58	->	83	0.00	0.00
58	->	84	-0.00	0.00
58	->	85	-0.00	0.00
58	->	86	0.00	0.00
58	->	87	0.02	0.01
58	->	88	0.03	0.02
58	->	89	0.03	0.01
58	->	90	0.01	0.00
58	->	91	0.01	0.00
58	->	92	0.01	0.00
58	->	93	0.00	0.00
58	->	94	0.01	0.00
58	->	95	0.01	0.00
58	->	96	0.01	0.00
58	->	97	0.00	0.00

58	->	98	0.01	0.00
58	->	99	0.02	0.01
58	->	100	0.04	0.02
58	->	101	0.05	0.02
58	->	102	0.03	0.01
58	->	103	0.01	0.00
58	->	104	0.01	0.00
58	->	105	0.01	0.00
58	->	106	0.00	0.00
58	->	107	0.00	0.00
59	->	1	0.01	0.00
59	->	2	0.00	0.00
59	->	3	-0.00	0.00
59	->	4	0.00	0.00
59	->	5	-0.00	0.00
59	->	6	0.00	0.00
59	->	7	0.00	0.00
59	->	8	-0.00	0.00
59	->	9	0.00	0.00
59	->	10	-0.00	0.00
59	->	11	-0.00	0.00
59	->	12	0.00	0.00
59	->	13	0.00	0.00
59	->	14	0.00	0.00
59	->	15	0.00	0.00
59	->	16	0.01	0.00
59	->	17	-0.00	0.00
59	->	18	-0.01	0.00
59	->	19	-0.01	0.00
59	->	20	-0.00	0.00
59	->	21	0.00	0.00
59	->	22	0.01	0.00
59	->	23	0.00	0.00
59	->	24	0.00	0.00
59	->	25	0.01	0.00
59	->	26	0.00	0.00
59	->	27	0.00	0.00
59	->	28	0.00	0.00
59	->	29	0.00	0.00
59	->	30	-0.00	0.00
59	->	31	0.01	0.00
59	->	32	-0.00	0.00
59	->	33	-0.00	0.00
59	->	34	0.00	0.00
59	->	35	-0.03	0.00
59	->	36	0.02	0.01
59	->	37	-0.00	0.00
59	->	38	-0.03	0.01
59	->	39	0.00	0.00
59	->	40	0.02	0.01

59 ->	41	-0.00	0.00
59 ->	42	-0.00	0.00
59 ->	43	0.00	0.00
59 ->	44	0.01	0.00
59 ->	45	-0.00	0.00
59 ->	46	-0.03	0.01
59 ->	47	-0.00	0.00
59 ->	48	-0.00	0.00
59 ->	49	-0.00	0.00
59 ->	50	-0.04	0.01
59 ->	51	-0.01	0.00
59 ->	52	-0.01	0.01
59 ->	53	0.02	0.12
59 ->	54	-0.19	0.13
59 ->	55	-0.04	0.02
59 ->	56	-0.04	0.02
59 ->	57	-1.47	0.63
59 ->	58	-11.94	0.85
59 ->	59	11.16	3.20
59 ->	60	-0.28	0.42
59 ->	61	-0.98	0.26
59 ->	62	-0.15	0.03
59 ->	63	-0.09	0.01
59 ->	64	-0.03	0.00
59 ->	65	-0.00	0.00
59 ->	66	-0.06	0.01
59 ->	67	-0.01	0.01
59 ->	68	0.08	0.24
59 ->	69	-2.26	0.76
59 ->	70	-3.26	0.44
59 ->	71	-1.83	0.33
59 ->	72	-0.05	0.06
59 ->	73	-0.05	0.02
59 ->	74	-0.07	0.02
59 ->	75	-0.55	0.41
59 ->	76	0.04	0.01
59 ->	77	0.01	0.00
59 ->	78	0.04	0.01
59 ->	79	-0.00	0.00
59 ->	80	-0.00	0.00
59 ->	81	0.02	0.00
59 ->	82	0.00	0.00
59 ->	83	-0.00	0.00
59 ->	84	0.00	0.00
59 ->	85	0.00	0.00
59 ->	86	-0.09	0.22
59 ->	87	-0.76	1.64
59 ->	88	-0.79	1.53
59 ->	89	-0.05	0.02
59 ->	90	-0.02	0.00

59	->	91	-0.01	0.00
59	->	92	-0.01	0.00
59	->	93	-0.01	0.00
59	->	94	-0.01	0.00
59	->	95	-0.01	0.00
59	->	96	-0.00	0.00
59	->	97	-0.00	0.00
59	->	98	-0.01	0.00
59	->	99	-0.01	0.00
59	->	100	-0.03	0.01
59	->	101	-0.06	0.02
59	->	102	-0.05	0.02
59	->	103	-0.02	0.01
59	->	104	-0.02	0.00
59	->	105	-0.01	0.00
59	->	106	-0.01	0.00
59	->	107	-0.01	0.00
60	->	1	0.00	0.00
60	->	2	0.00	0.00
60	->	3	0.00	0.00
60	->	4	0.00	0.00
60	->	5	-0.00	0.00
60	->	6	0.00	0.00
60	->	7	0.00	0.00
60	->	8	-0.00	0.00
60	->	9	0.00	0.00
60	->	10	-0.00	0.00
60	->	11	-0.00	0.00
60	->	12	0.00	0.00
60	->	13	0.00	0.00
60	->	14	0.00	0.00
60	->	15	0.00	0.00
60	->	16	0.00	0.00
60	->	17	-0.00	0.00
60	->	18	-0.00	0.00
60	->	19	-0.00	0.00
60	->	20	0.00	0.00
60	->	21	-0.00	0.00
60	->	22	0.00	0.00
60	->	23	0.00	0.00
60	->	24	0.00	0.00
60	->	25	0.00	0.00
60	->	26	0.00	0.00
60	->	27	0.00	0.00
60	->	28	0.00	0.00
60	->	29	0.00	0.00
60	->	30	-0.00	0.00
60	->	31	0.00	0.00
60	->	32	-0.00	0.00
60	->	33	-0.00	0.00

60	->	34	-0.00	0.00
60	->	35	-0.00	0.00
60	->	36	0.00	0.00
60	->	37	-0.00	0.00
60	->	38	-0.00	0.00
60	->	39	0.00	0.00
60	->	40	0.00	0.00
60	->	41	-0.00	0.00
60	->	42	-0.00	0.00
60	->	43	0.00	0.00
60	->	44	0.00	0.00
60	->	45	-0.00	0.00
60	->	46	-0.01	0.00
60	->	47	-0.00	0.00
60	->	48	-0.00	0.00
60	->	49	-0.02	0.01
60	->	50	-0.03	0.02
60	->	51	-0.01	0.00
60	->	52	-0.01	0.00
60	->	53	-1.32	0.55
60	->	54	-0.02	0.01
60	->	55	-0.00	0.00
60	->	56	-0.02	0.00
60	->	57	-0.03	0.00
60	->	58	-1.45	0.43
60	->	59	-0.32	0.42
60	->	60	1.64	0.74
60	->	61	-10.27	0.56
60	->	62	-0.32	0.08
60	->	63	-0.02	0.00
60	->	64	-0.00	0.00
60	->	65	-0.00	0.00
60	->	66	0.00	0.00
60	->	67	-0.00	0.00
60	->	68	-0.02	0.01
60	->	69	-0.74	0.89
60	->	70	-0.93	0.16
60	->	71	-0.83	0.18
60	->	72	-0.12	0.07
60	->	73	-0.04	0.02
60	->	74	-0.01	0.00
60	->	75	-0.02	0.01
60	->	76	-0.03	0.02
60	->	77	-0.00	0.00
60	->	78	-0.00	0.00
60	->	79	-0.00	0.00
60	->	80	-0.00	0.00
60	->	81	-0.00	0.00
60	->	82	-0.00	0.00
60	->	83	-0.00	0.00

60	->	84	0.00	0.00
60	->	85	0.00	0.00
60	->	86	-0.00	0.00
60	->	87	-0.01	0.00
60	->	88	-0.01	0.00
60	->	89	-0.00	0.00
60	->	90	-0.00	0.00
60	->	91	-0.00	0.00
60	->	92	-0.00	0.00
60	->	93	-0.00	0.00
60	->	94	-0.00	0.00
60	->	95	-0.00	0.00
60	->	96	-0.00	0.00
60	->	97	-0.00	0.00
60	->	98	-0.00	0.00
60	->	99	-0.00	0.00
60	->	100	-0.02	0.02
60	->	101	-0.10	0.12
60	->	102	-0.04	0.04
60	->	103	-0.00	0.00
60	->	104	-0.00	0.00
60	->	105	-0.00	0.00
60	->	106	-0.00	0.00
60	->	107	-0.00	0.00
61	->	1	0.00	0.00
61	->	2	0.00	0.00
61	->	3	0.00	0.00
61	->	4	0.00	0.00
61	->	5	-0.00	0.00
61	->	6	0.00	0.00
61	->	7	0.00	0.00
61	->	8	-0.00	0.00
61	->	9	0.00	0.00
61	->	10	-0.00	0.00
61	->	11	-0.00	0.00
61	->	12	0.00	0.00
61	->	13	0.00	0.00
61	->	14	0.00	0.00
61	->	15	0.00	0.00
61	->	16	0.00	0.00
61	->	17	-0.00	0.00
61	->	18	-0.00	0.00
61	->	19	-0.00	0.00
61	->	20	0.00	0.00
61	->	21	0.00	0.00
61	->	22	0.00	0.00
61	->	23	0.00	0.00
61	->	24	0.00	0.00
61	->	25	0.00	0.00
61	->	26	0.00	0.00

61	->	27	0.00	0.00
61	->	28	0.00	0.00
61	->	29	0.00	0.00
61	->	30	0.00	0.00
61	->	31	0.00	0.00
61	->	32	0.00	0.00
61	->	33	-0.00	0.00
61	->	34	0.00	0.00
61	->	35	0.00	0.00
61	->	36	-0.00	0.00
61	->	37	0.00	0.00
61	->	38	0.00	0.00
61	->	39	-0.00	0.00
61	->	40	-0.00	0.00
61	->	41	0.00	0.00
61	->	42	-0.00	0.00
61	->	43	-0.00	0.00
61	->	44	-0.00	0.00
61	->	45	-0.00	0.00
61	->	46	-0.01	0.01
61	->	47	-0.00	0.00
61	->	48	-0.00	0.00
61	->	49	-0.02	0.01
61	->	50	-0.04	0.06
61	->	51	-0.00	0.00
61	->	52	-0.01	0.00
61	->	53	-0.56	0.41
61	->	54	-0.00	0.00
61	->	55	-0.00	0.00
61	->	56	-0.00	0.00
61	->	57	-0.01	0.00
61	->	58	-0.10	0.02
61	->	59	-0.99	0.26
61	->	60	-10.40	0.56
61	->	61	27.37	1.61
61	->	62	-10.89	0.44
61	->	63	-0.38	0.05
61	->	64	-0.05	0.01
61	->	65	-0.01	0.00
61	->	66	-0.01	0.00
61	->	67	-0.01	0.00
61	->	68	-0.30	0.06
61	->	69	-3.12	0.70
61	->	70	-3.80	0.49
61	->	71	-0.96	0.36
61	->	72	-1.40	0.79
61	->	73	-1.20	0.72
61	->	74	-0.00	0.02
61	->	75	-0.09	0.04
61	->	76	-1.45	0.76

61	->	77	-0.02	0.02
61	->	78	-0.01	0.00
61	->	79	-0.02	0.00
61	->	80	-0.01	0.00
61	->	81	-0.00	0.00
61	->	82	-0.00	0.00
61	->	83	-0.01	0.00
61	->	84	-0.00	0.00
61	->	85	-0.00	0.00
61	->	86	-0.00	0.00
61	->	87	-0.00	0.00
61	->	88	-0.01	0.00
61	->	89	-0.00	0.00
61	->	90	-0.00	0.00
61	->	91	-0.00	0.00
61	->	92	-0.01	0.00
61	->	93	-0.01	0.01
61	->	94	-0.00	0.00
61	->	95	-0.00	0.00
61	->	96	-0.00	0.00
61	->	97	-0.00	0.00
61	->	98	-0.00	0.00
61	->	99	-0.00	0.00
61	->	100	-0.03	0.04
61	->	101	-0.86	1.02
61	->	102	-0.51	0.57
61	->	103	-0.05	0.09
61	->	104	-0.01	0.01
61	->	105	-0.00	0.00
61	->	106	-0.00	0.00
61	->	107	-0.00	0.00
62	->	1	-0.00	0.00
62	->	2	-0.00	0.00
62	->	3	0.00	0.00
62	->	4	0.00	0.00
62	->	5	0.00	0.00
62	->	6	-0.00	0.00
62	->	7	0.00	0.00
62	->	8	0.00	0.00
62	->	9	0.00	0.00
62	->	10	0.00	0.00
62	->	11	0.00	0.00
62	->	12	-0.00	0.00
62	->	13	-0.00	0.00
62	->	14	-0.00	0.00
62	->	15	0.00	0.00
62	->	16	-0.00	0.00
62	->	17	0.00	0.00
62	->	18	0.00	0.00
62	->	19	0.00	0.00

62	->	20	0.00	0.00
62	->	21	0.00	0.00
62	->	22	-0.00	0.00
62	->	23	0.00	0.00
62	->	24	0.00	0.00
62	->	25	-0.00	0.00
62	->	26	-0.00	0.00
62	->	27	0.00	0.00
62	->	28	0.00	0.00
62	->	29	0.00	0.00
62	->	30	0.00	0.00
62	->	31	-0.00	0.00
62	->	32	-0.00	0.00
62	->	33	0.00	0.00
62	->	34	0.00	0.00
62	->	35	0.00	0.00
62	->	36	-0.00	0.00
62	->	37	0.00	0.00
62	->	38	0.00	0.00
62	->	39	0.00	0.00
62	->	40	-0.00	0.00
62	->	41	0.00	0.00
62	->	42	-0.00	0.00
62	->	43	-0.00	0.00
62	->	44	-0.00	0.00
62	->	45	0.00	0.00
62	->	46	0.00	0.00
62	->	47	-0.00	0.00
62	->	48	-0.00	0.00
62	->	49	-0.00	0.00
62	->	50	-0.00	0.00
62	->	51	-0.00	0.00
62	->	52	-0.00	0.00
62	->	53	-0.01	0.00
62	->	54	-0.00	0.00
62	->	55	-0.00	0.00
62	->	56	-0.00	0.00
62	->	57	-0.00	0.00
62	->	58	-0.01	0.00
62	->	59	-0.15	0.03
62	->	60	-0.34	0.09
62	->	61	-10.87	0.44
62	->	62	22.97	0.98
62	->	63	-11.96	0.45
62	->	64	-0.41	0.08
62	->	65	-0.02	0.03
62	->	66	-0.18	0.07
62	->	67	-0.07	0.13
62	->	68	-1.19	0.49
62	->	69	-1.37	0.46

62	->	70	-1.16	0.38
62	->	71	-0.01	0.01
62	->	72	-0.02	0.01
62	->	73	-0.01	0.01
62	->	74	0.00	0.00
62	->	75	-0.01	0.01
62	->	76	-1.97	1.62
62	->	77	-0.01	0.01
62	->	78	0.00	0.00
62	->	79	-0.09	0.02
62	->	80	-0.03	0.01
62	->	81	-0.00	0.00
62	->	82	-0.00	0.00
62	->	83	-0.04	0.01
62	->	84	-0.00	0.00
62	->	85	-0.00	0.00
62	->	86	-0.00	0.00
62	->	87	-0.00	0.00
62	->	88	-0.00	0.00
62	->	89	0.00	0.00
62	->	90	0.00	0.00
62	->	91	0.00	0.00
62	->	92	0.00	0.00
62	->	93	0.00	0.00
62	->	94	0.00	0.00
62	->	95	0.00	0.00
62	->	96	0.00	0.00
62	->	97	0.00	0.00
62	->	98	0.00	0.00
62	->	99	0.00	0.00
62	->	100	0.00	0.00
62	->	101	0.00	0.00
62	->	102	0.00	0.01
62	->	103	0.00	0.00
62	->	104	0.00	0.00
62	->	105	0.00	0.00
62	->	106	0.00	0.00
62	->	107	0.00	0.00
63	->	1	-0.01	0.00
63	->	2	-0.00	0.00
63	->	3	0.00	0.00
63	->	4	-0.00	0.00
63	->	5	0.00	0.00
63	->	6	-0.00	0.00
63	->	7	-0.00	0.00
63	->	8	0.00	0.00
63	->	9	0.00	0.00
63	->	10	0.00	0.00
63	->	11	0.00	0.00
63	->	12	-0.00	0.00

63	->	13	-0.00	0.00
63	->	14	-0.00	0.00
63	->	15	-0.00	0.00
63	->	16	-0.00	0.00
63	->	17	0.00	0.00
63	->	18	0.00	0.00
63	->	19	0.00	0.00
63	->	20	0.00	0.00
63	->	21	-0.00	0.00
63	->	22	-0.01	0.00
63	->	23	-0.00	0.00
63	->	24	-0.00	0.00
63	->	25	-0.00	0.00
63	->	26	-0.00	0.00
63	->	27	-0.00	0.00
63	->	28	-0.00	0.00
63	->	29	-0.00	0.00
63	->	30	0.00	0.00
63	->	31	-0.00	0.00
63	->	32	-0.00	0.00
63	->	33	0.00	0.00
63	->	34	-0.00	0.00
63	->	35	0.01	0.00
63	->	36	-0.00	0.00
63	->	37	0.00	0.00
63	->	38	0.01	0.00
63	->	39	-0.00	0.00
63	->	40	-0.01	0.00
63	->	41	0.00	0.00
63	->	42	-0.00	0.00
63	->	43	-0.00	0.00
63	->	44	-0.01	0.00
63	->	45	0.00	0.00
63	->	46	0.01	0.00
63	->	47	0.00	0.00
63	->	48	0.00	0.00
63	->	49	0.00	0.00
63	->	50	0.00	0.00
63	->	51	-0.00	0.00
63	->	52	0.00	0.00
63	->	53	-0.03	0.00
63	->	54	0.00	0.00
63	->	55	-0.00	0.00
63	->	56	0.00	0.00
63	->	57	0.00	0.00
63	->	58	0.02	0.00
63	->	59	-0.09	0.01
63	->	60	-0.02	0.00
63	->	61	-0.38	0.05
63	->	62	-12.01	0.46

63	->	63	-27.25	1.94
63	->	64	-15.02	1.33
63	->	65	0.80	1.17
63	->	66	2.98	0.45
63	->	67	-0.47	0.49
63	->	68	-2.21	0.74
63	->	69	-0.63	0.14
63	->	70	-0.98	0.28
63	->	71	0.01	0.01
63	->	72	-0.04	0.01
63	->	73	-0.01	0.01
63	->	74	0.04	0.01
63	->	75	-0.00	0.02
63	->	76	-1.27	0.71
63	->	77	-0.03	0.02
63	->	78	-0.14	0.03
63	->	79	3.08	0.61
63	->	80	0.14	0.18
63	->	81	-0.01	0.02
63	->	82	-0.06	0.02
63	->	83	2.30	0.30
63	->	84	-0.00	0.01
63	->	85	-0.00	0.04
63	->	86	0.01	0.00
63	->	87	0.04	0.01
63	->	88	0.01	0.00
63	->	89	0.01	0.00
63	->	90	0.01	0.00
63	->	91	0.01	0.00
63	->	92	0.00	0.00
63	->	93	0.00	0.00
63	->	94	0.01	0.00
63	->	95	0.01	0.00
63	->	96	0.01	0.00
63	->	97	0.00	0.00
63	->	98	0.00	0.00
63	->	99	0.01	0.00
63	->	100	0.01	0.00
63	->	101	0.02	0.01
63	->	102	0.04	0.02
63	->	103	0.04	0.01
63	->	104	0.02	0.00
63	->	105	0.01	0.00
63	->	106	0.01	0.00
63	->	107	0.00	0.00
64	->	1	-0.01	0.00
64	->	2	-0.00	0.00
64	->	3	0.00	0.00
64	->	4	-0.00	0.00
64	->	5	0.00	0.00

64 ->	6	-0.00	0.00
64 ->	7	0.00	0.00
64 ->	8	0.00	0.00
64 ->	9	0.00	0.00
64 ->	10	0.00	0.00
64 ->	11	0.00	0.00
64 ->	12	-0.00	0.00
64 ->	13	-0.00	0.00
64 ->	14	-0.00	0.00
64 ->	15	-0.00	0.00
64 ->	16	-0.00	0.00
64 ->	17	0.00	0.00
64 ->	18	0.00	0.00
64 ->	19	0.00	0.00
64 ->	20	0.00	0.00
64 ->	21	-0.00	0.00
64 ->	22	-0.00	0.00
64 ->	23	-0.00	0.00
64 ->	24	-0.00	0.00
64 ->	25	-0.00	0.00
64 ->	26	-0.00	0.00
64 ->	27	-0.00	0.00
64 ->	28	-0.00	0.00
64 ->	29	-0.00	0.00
64 ->	30	0.00	0.00
64 ->	31	-0.00	0.00
64 ->	32	-0.00	0.00
64 ->	33	0.00	0.00
64 ->	34	-0.00	0.00
64 ->	35	0.00	0.00
64 ->	36	-0.00	0.00
64 ->	37	0.00	0.00
64 ->	38	0.00	0.00
64 ->	39	-0.00	0.00
64 ->	40	-0.00	0.00
64 ->	41	0.00	0.00
64 ->	42	-0.00	0.00
64 ->	43	-0.00	0.00
64 ->	44	-0.01	0.00
64 ->	45	0.00	0.00
64 ->	46	0.01	0.00
64 ->	47	-0.00	0.00
64 ->	48	0.00	0.00
64 ->	49	-0.00	0.00
64 ->	50	0.00	0.00
64 ->	51	-0.00	0.00
64 ->	52	0.00	0.00
64 ->	53	-0.02	0.00
64 ->	54	0.00	0.00
64 ->	55	-0.00	0.00

64 ->	56	0.00	0.00
64 ->	57	0.00	0.00
64 ->	58	0.01	0.00
64 ->	59	-0.03	0.00
64 ->	60	-0.00	0.00
64 ->	61	-0.05	0.01
64 ->	62	-0.41	0.08
64 ->	63	-15.17	1.35
64 ->	64	-35.31	2.24
64 ->	65	-12.24	0.52
64 ->	66	-0.24	0.16
64 ->	67	-0.10	0.23
64 ->	68	-0.05	0.02
64 ->	69	-0.08	0.02
64 ->	70	-0.03	0.02
64 ->	71	0.00	0.00
64 ->	72	-0.02	0.00
64 ->	73	-0.00	0.00
64 ->	74	0.02	0.00
64 ->	75	0.02	0.00
64 ->	76	-0.91	1.48
64 ->	77	-0.04	0.01
64 ->	78	-0.03	0.01
64 ->	79	-0.07	0.06
64 ->	80	-0.17	0.22
64 ->	81	-0.03	0.01
64 ->	82	-0.01	0.00
64 ->	83	-0.20	0.12
64 ->	84	-0.03	0.04
64 ->	85	-0.32	1.50
64 ->	86	0.00	0.00
64 ->	87	0.01	0.00
64 ->	88	0.01	0.00
64 ->	89	0.01	0.00
64 ->	90	0.00	0.00
64 ->	91	0.00	0.00
64 ->	92	0.00	0.00
64 ->	93	0.00	0.00
64 ->	94	0.01	0.00
64 ->	95	0.01	0.00
64 ->	96	0.01	0.00
64 ->	97	0.00	0.00
64 ->	98	0.00	0.00
64 ->	99	0.00	0.00
64 ->	100	0.01	0.00
64 ->	101	0.02	0.01
64 ->	102	0.03	0.01
64 ->	103	0.03	0.01
64 ->	104	0.02	0.00
64 ->	105	0.01	0.00

64	->	106	0.01	0.00
64	->	107	0.00	0.00
65	->	1	-0.00	0.00
65	->	2	0.00	0.00
65	->	3	0.00	0.00
65	->	4	0.00	0.00
65	->	5	0.00	0.00
65	->	6	0.00	0.00
65	->	7	0.00	0.00
65	->	8	0.00	0.00
65	->	9	0.00	0.00
65	->	10	0.00	0.00
65	->	11	0.00	0.00
65	->	12	-0.00	0.00
65	->	13	-0.00	0.00
65	->	14	0.00	0.00
65	->	15	0.00	0.00
65	->	16	-0.00	0.00
65	->	17	0.00	0.00
65	->	18	0.00	0.00
65	->	19	0.00	0.00
65	->	20	0.00	0.00
65	->	21	0.00	0.00
65	->	22	-0.00	0.00
65	->	23	0.00	0.00
65	->	24	0.00	0.00
65	->	25	-0.00	0.00
65	->	26	0.00	0.00
65	->	27	0.00	0.00
65	->	28	0.00	0.00
65	->	29	0.00	0.00
65	->	30	0.00	0.00
65	->	31	-0.00	0.00
65	->	32	0.00	0.00
65	->	33	0.00	0.00
65	->	34	-0.00	0.00
65	->	35	0.00	0.00
65	->	36	-0.00	0.00
65	->	37	0.00	0.00
65	->	38	0.00	0.00
65	->	39	0.00	0.00
65	->	40	-0.00	0.00
65	->	41	0.00	0.00
65	->	42	-0.00	0.00
65	->	43	-0.00	0.00
65	->	44	-0.00	0.00
65	->	45	0.00	0.00
65	->	46	0.00	0.00
65	->	47	0.00	0.00
65	->	48	0.00	0.00

65	->	49	0.00	0.00
65	->	50	0.00	0.00
65	->	51	0.00	0.00
65	->	52	0.00	0.00
65	->	53	-0.00	0.00
65	->	54	0.00	0.00
65	->	55	-0.00	0.00
65	->	56	0.00	0.00
65	->	57	0.00	0.00
65	->	58	0.00	0.00
65	->	59	-0.00	0.00
65	->	60	-0.00	0.00
65	->	61	-0.01	0.00
65	->	62	-0.02	0.03
65	->	63	0.72	1.17
65	->	64	-12.43	0.52
65	->	65	21.78	1.70
65	->	66	-9.08	1.16
65	->	67	-0.28	0.07
65	->	68	-0.22	0.06
65	->	69	-0.01	0.00
65	->	70	-0.03	0.01
65	->	71	-0.00	0.00
65	->	72	-0.00	0.00
65	->	73	-0.00	0.00
65	->	74	0.00	0.00
65	->	75	0.00	0.00
65	->	76	-0.03	0.02
65	->	77	-0.01	0.00
65	->	78	-0.01	0.00
65	->	79	0.70	0.11
65	->	80	-0.09	0.09
65	->	81	-0.01	0.00
65	->	82	-0.04	0.01
65	->	83	0.82	0.59
65	->	84	-0.07	0.11
65	->	85	-0.12	0.50
65	->	86	-0.00	0.00
65	->	87	0.00	0.00
65	->	88	0.00	0.00
65	->	89	0.00	0.00
65	->	90	0.00	0.00
65	->	91	0.00	0.00
65	->	92	0.00	0.00
65	->	93	0.00	0.00
65	->	94	0.00	0.00
65	->	95	0.00	0.00
65	->	96	0.00	0.00
65	->	97	0.00	0.00
65	->	98	0.00	0.00

65	->	99	0.00	0.00
65	->	100	0.00	0.00
65	->	101	0.00	0.00
65	->	102	-0.00	0.00
65	->	103	0.00	0.00
65	->	104	0.00	0.00
65	->	105	0.00	0.00
65	->	106	0.00	0.00
65	->	107	0.00	0.00
66	->	1	-0.00	0.00
66	->	2	-0.00	0.00
66	->	3	0.00	0.00
66	->	4	0.00	0.00
66	->	5	0.00	0.00
66	->	6	-0.00	0.00
66	->	7	0.00	0.00
66	->	8	0.00	0.00
66	->	9	0.00	0.00
66	->	10	0.00	0.00
66	->	11	0.00	0.00
66	->	12	-0.00	0.00
66	->	13	-0.00	0.00
66	->	14	-0.00	0.00
66	->	15	-0.00	0.00
66	->	16	-0.00	0.00
66	->	17	0.00	0.00
66	->	18	0.00	0.00
66	->	19	0.00	0.00
66	->	20	0.00	0.00
66	->	21	0.00	0.00
66	->	22	-0.00	0.00
66	->	23	-0.00	0.00
66	->	24	-0.00	0.00
66	->	25	-0.00	0.00
66	->	26	0.00	0.00
66	->	27	-0.00	0.00
66	->	28	-0.00	0.00
66	->	29	-0.00	0.00
66	->	30	0.00	0.00
66	->	31	-0.00	0.00
66	->	32	-0.00	0.00
66	->	33	-0.00	0.00
66	->	34	-0.00	0.00
66	->	35	0.01	0.00
66	->	36	-0.00	0.00
66	->	37	0.00	0.00
66	->	38	0.01	0.00
66	->	39	-0.00	0.00
66	->	40	-0.01	0.00
66	->	41	0.00	0.00

66 ->	42	-0.00	0.00
66 ->	43	-0.00	0.00
66 ->	44	-0.01	0.00
66 ->	45	0.00	0.00
66 ->	46	0.01	0.00
66 ->	47	0.00	0.00
66 ->	48	0.00	0.00
66 ->	49	0.00	0.00
66 ->	50	0.00	0.00
66 ->	51	-0.00	0.00
66 ->	52	0.00	0.00
66 ->	53	-0.02	0.00
66 ->	54	0.00	0.00
66 ->	55	-0.00	0.00
66 ->	56	0.00	0.00
66 ->	57	0.00	0.00
66 ->	58	0.02	0.00
66 ->	59	-0.06	0.01
66 ->	60	0.00	0.00
66 ->	61	-0.01	0.00
66 ->	62	-0.18	0.07
66 ->	63	3.00	0.44
66 ->	64	-0.24	0.16
66 ->	65	-9.00	1.16
66 ->	66	-30.79	2.37
66 ->	67	-8.81	0.38
66 ->	68	-3.50	0.54
66 ->	69	-0.02	0.03
66 ->	70	0.04	0.04
66 ->	71	-0.00	0.00
66 ->	72	-0.02	0.00
66 ->	73	0.00	0.00
66 ->	74	0.03	0.00
66 ->	75	0.05	0.01
66 ->	76	-0.10	0.03
66 ->	77	-0.03	0.01
66 ->	78	-0.07	0.01
66 ->	79	3.57	0.72
66 ->	80	0.03	0.01
66 ->	81	-0.03	0.01
66 ->	82	-0.05	0.02
66 ->	83	2.05	0.32
66 ->	84	-0.00	0.00
66 ->	85	-0.00	0.03
66 ->	86	0.01	0.01
66 ->	87	0.04	0.01
66 ->	88	0.01	0.00
66 ->	89	0.01	0.00
66 ->	90	0.01	0.00
66 ->	91	0.00	0.00

66	->	92	0.00	0.00
66	->	93	0.00	0.00
66	->	94	0.01	0.00
66	->	95	0.00	0.00
66	->	96	0.00	0.00
66	->	97	0.00	0.00
66	->	98	0.00	0.00
66	->	99	0.00	0.00
66	->	100	0.01	0.00
66	->	101	0.02	0.00
66	->	102	0.02	0.01
66	->	103	0.02	0.00
66	->	104	0.02	0.00
66	->	105	0.01	0.00
66	->	106	0.01	0.00
66	->	107	0.00	0.00
67	->	1	-0.00	0.00
67	->	2	0.00	0.00
67	->	3	0.00	0.00
67	->	4	0.00	0.00
67	->	5	0.00	0.00
67	->	6	0.00	0.00
67	->	7	0.00	0.00
67	->	8	0.00	0.00
67	->	9	0.00	0.00
67	->	10	0.00	0.00
67	->	11	-0.00	0.00
67	->	12	0.00	0.00
67	->	13	0.00	0.00
67	->	14	-0.00	0.00
67	->	15	-0.00	0.00
67	->	16	-0.00	0.00
67	->	17	0.00	0.00
67	->	18	0.00	0.00
67	->	19	0.00	0.00
67	->	20	0.00	0.00
67	->	21	0.00	0.00
67	->	22	-0.00	0.00
67	->	23	0.00	0.00
67	->	24	0.00	0.00
67	->	25	-0.00	0.00
67	->	26	0.00	0.00
67	->	27	0.00	0.00
67	->	28	0.00	0.00
67	->	29	0.00	0.00
67	->	30	0.00	0.00
67	->	31	-0.00	0.00
67	->	32	0.00	0.00
67	->	33	0.00	0.00
67	->	34	0.00	0.00

67 ->	35	0.00	0.00
67 ->	36	-0.00	0.00
67 ->	37	0.00	0.00
67 ->	38	0.00	0.00
67 ->	39	0.00	0.00
67 ->	40	-0.00	0.00
67 ->	41	0.00	0.00
67 ->	42	0.00	0.00
67 ->	43	0.00	0.00
67 ->	44	-0.00	0.00
67 ->	45	0.00	0.00
67 ->	46	0.00	0.00
67 ->	47	0.00	0.00
67 ->	48	0.00	0.00
67 ->	49	0.00	0.00
67 ->	50	0.00	0.00
67 ->	51	0.00	0.00
67 ->	52	0.00	0.00
67 ->	53	-0.00	0.00
67 ->	54	0.00	0.00
67 ->	55	-0.00	0.00
67 ->	56	0.00	0.00
67 ->	57	-0.00	0.00
67 ->	58	0.00	0.00
67 ->	59	-0.01	0.01
67 ->	60	-0.00	0.00
67 ->	61	-0.01	0.00
67 ->	62	-0.07	0.13
67 ->	63	-0.47	0.49
67 ->	64	-0.10	0.24
67 ->	65	-0.28	0.07
67 ->	66	-8.86	0.37
67 ->	67	10.99	0.83
67 ->	68	-8.64	0.41
67 ->	69	-1.01	0.71
67 ->	70	-0.08	0.01
67 ->	71	-0.00	0.00
67 ->	72	-0.00	0.00
67 ->	73	-0.00	0.00
67 ->	74	-0.00	0.00
67 ->	75	-0.00	0.00
67 ->	76	-0.01	0.00
67 ->	77	-0.00	0.00
67 ->	78	-0.00	0.00
67 ->	79	-0.06	0.03
67 ->	80	-0.00	0.00
67 ->	81	-0.00	0.00
67 ->	82	-0.00	0.00
67 ->	83	-0.01	0.01
67 ->	84	-0.00	0.00

67	->	85	-0.00	0.00
67	->	86	-0.01	0.02
67	->	87	-0.00	0.01
67	->	88	0.00	0.00
67	->	89	0.00	0.00
67	->	90	0.00	0.00
67	->	91	0.00	0.00
67	->	92	0.00	0.00
67	->	93	-0.00	0.00
67	->	94	0.00	0.00
67	->	95	0.00	0.00
67	->	96	0.00	0.00
67	->	97	0.00	0.00
67	->	98	0.00	0.00
67	->	99	0.00	0.00
67	->	100	0.00	0.00
67	->	101	0.00	0.00
67	->	102	-0.00	0.00
67	->	103	-0.00	0.00
67	->	104	-0.00	0.00
67	->	105	-0.00	0.00
67	->	106	-0.00	0.00
67	->	107	-0.00	0.00
68	->	1	0.01	0.00
68	->	2	0.00	0.00
68	->	3	-0.00	0.00
68	->	4	0.00	0.00
68	->	5	-0.00	0.00
68	->	6	0.00	0.00
68	->	7	-0.00	0.00
68	->	8	-0.00	0.00
68	->	9	-0.00	0.00
68	->	10	-0.00	0.00
68	->	11	-0.00	0.00
68	->	12	0.00	0.00
68	->	13	0.00	0.00
68	->	14	0.00	0.00
68	->	15	0.00	0.00
68	->	16	0.00	0.00
68	->	17	-0.00	0.00
68	->	18	-0.00	0.00
68	->	19	-0.00	0.00
68	->	20	-0.00	0.00
68	->	21	0.00	0.00
68	->	22	0.01	0.00
68	->	23	0.00	0.00
68	->	24	-0.00	0.00
68	->	25	0.00	0.00
68	->	26	0.00	0.00
68	->	27	0.00	0.00

68	->	28	0.00	0.00
68	->	29	0.00	0.00
68	->	30	-0.00	0.00
68	->	31	0.01	0.00
68	->	32	-0.00	0.00
68	->	33	-0.00	0.00
68	->	34	0.00	0.00
68	->	35	-0.01	0.00
68	->	36	0.01	0.00
68	->	37	-0.00	0.00
68	->	38	-0.01	0.00
68	->	39	0.00	0.00
68	->	40	0.01	0.00
68	->	41	-0.00	0.00
68	->	42	0.00	0.00
68	->	43	0.00	0.00
68	->	44	0.01	0.00
68	->	45	-0.00	0.00
68	->	46	-0.02	0.00
68	->	47	-0.00	0.00
68	->	48	-0.00	0.00
68	->	49	-0.00	0.00
68	->	50	-0.00	0.00
68	->	51	-0.00	0.00
68	->	52	-0.00	0.00
68	->	53	0.03	0.01
68	->	54	-0.01	0.01
68	->	55	-0.00	0.00
68	->	56	-0.00	0.00
68	->	57	-0.00	0.00
68	->	58	-0.03	0.01
68	->	59	0.08	0.24
68	->	60	-0.02	0.01
68	->	61	-0.30	0.06
68	->	62	-1.19	0.49
68	->	63	-2.22	0.75
68	->	64	-0.05	0.02
68	->	65	-0.22	0.06
68	->	66	-3.60	0.54
68	->	67	-8.79	0.40
68	->	68	-106.90	3.17
68	->	69	-13.53	1.21
68	->	70	-3.70	0.78
68	->	71	-0.08	0.03
68	->	72	0.04	0.03
68	->	73	-0.01	0.00
68	->	74	-0.06	0.01
68	->	75	-2.71	3.66
68	->	76	-0.07	0.05
68	->	77	0.01	0.01

68	->	78	0.09	0.04
68	->	79	-2.23	0.64
68	->	80	-0.04	0.01
68	->	81	0.03	0.01
68	->	82	-0.04	0.04
68	->	83	-0.25	0.06
68	->	84	-0.00	0.00
68	->	85	-0.00	0.01
68	->	86	-0.88	1.48
68	->	87	-1.67	1.97
68	->	88	-0.06	0.02
68	->	89	-0.03	0.01
68	->	90	-0.01	0.00
68	->	91	-0.01	0.00
68	->	92	-0.01	0.00
68	->	93	-0.00	0.00
68	->	94	-0.01	0.00
68	->	95	-0.00	0.00
68	->	96	-0.00	0.00
68	->	97	-0.00	0.00
68	->	98	-0.00	0.00
68	->	99	-0.01	0.00
68	->	100	-0.01	0.00
68	->	101	-0.02	0.00
68	->	102	-0.02	0.01
68	->	103	-0.02	0.01
68	->	104	-0.02	0.00
68	->	105	-0.01	0.01
68	->	106	-0.02	0.02
68	->	107	-0.01	0.01
69	->	1	0.00	0.00
69	->	2	0.00	0.00
69	->	3	-0.00	0.00
69	->	4	0.00	0.00
69	->	5	-0.00	0.00
69	->	6	0.00	0.00
69	->	7	0.00	0.00
69	->	8	-0.00	0.00
69	->	9	0.00	0.00
69	->	10	-0.00	0.00
69	->	11	-0.00	0.00
69	->	12	0.00	0.00
69	->	13	0.00	0.00
69	->	14	0.00	0.00
69	->	15	0.00	0.00
69	->	16	0.01	0.00
69	->	17	-0.00	0.00
69	->	18	-0.00	0.00
69	->	19	-0.00	0.00
69	->	20	0.00	0.00

69	->	21	0.00	0.00
69	->	22	0.01	0.00
69	->	23	0.00	0.00
69	->	24	0.00	0.00
69	->	25	0.00	0.00
69	->	26	0.00	0.00
69	->	27	0.00	0.00
69	->	28	0.00	0.00
69	->	29	0.00	0.00
69	->	30	-0.00	0.00
69	->	31	0.00	0.00
69	->	32	-0.00	0.00
69	->	33	0.00	0.00
69	->	34	-0.00	0.00
69	->	35	-0.01	0.00
69	->	36	0.01	0.00
69	->	37	-0.00	0.00
69	->	38	-0.01	0.00
69	->	39	0.00	0.00
69	->	40	0.01	0.00
69	->	41	-0.00	0.00
69	->	42	0.00	0.00
69	->	43	0.00	0.00
69	->	44	0.01	0.00
69	->	45	-0.00	0.00
69	->	46	-0.02	0.00
69	->	47	-0.00	0.00
69	->	48	-0.00	0.00
69	->	49	0.00	0.00
69	->	50	-0.01	0.00
69	->	51	-0.00	0.00
69	->	52	-0.00	0.00
69	->	53	0.08	0.02
69	->	54	-0.02	0.01
69	->	55	-0.00	0.00
69	->	56	-0.01	0.00
69	->	57	-0.02	0.02
69	->	58	-0.06	0.09
69	->	59	-2.28	0.75
69	->	60	-0.75	0.91
69	->	61	-3.14	0.70
69	->	62	-1.40	0.47
69	->	63	-0.63	0.14
69	->	64	-0.08	0.02
69	->	65	-0.01	0.00
69	->	66	-0.02	0.03
69	->	67	-1.07	0.71
69	->	68	-13.52	1.22
69	->	69	11.09	2.56
69	->	70	-10.61	0.56

69 ->	71	-0.42	0.06
69 ->	72	-0.01	0.03
69 ->	73	-0.04	0.01
69 ->	74	-0.04	0.01
69 ->	75	-0.24	0.18
69 ->	76	0.11	0.07
69 ->	77	-0.00	0.01
69 ->	78	0.03	0.01
69 ->	79	-0.02	0.01
69 ->	80	-0.01	0.00
69 ->	81	0.01	0.00
69 ->	82	-0.00	0.00
69 ->	83	-0.01	0.01
69 ->	84	-0.00	0.00
69 ->	85	-0.00	0.00
69 ->	86	-0.03	0.04
69 ->	87	-0.09	0.11
69 ->	88	-0.04	0.02
69 ->	89	-0.02	0.00
69 ->	90	-0.01	0.00
69 ->	91	-0.01	0.00
69 ->	92	-0.00	0.00
69 ->	93	-0.00	0.00
69 ->	94	-0.01	0.00
69 ->	95	-0.01	0.00
69 ->	96	-0.01	0.00
69 ->	97	-0.00	0.00
69 ->	98	-0.00	0.00
69 ->	99	-0.01	0.00
69 ->	100	-0.02	0.01
69 ->	101	-0.04	0.02
69 ->	102	-0.05	0.02
69 ->	103	-0.02	0.01
69 ->	104	-0.01	0.00
69 ->	105	-0.01	0.00
69 ->	106	-0.01	0.00
69 ->	107	-0.00	0.00
70 ->	1	0.00	0.00
70 ->	2	0.00	0.00
70 ->	3	-0.00	0.00
70 ->	4	0.00	0.00
70 ->	5	-0.00	0.00
70 ->	6	0.00	0.00
70 ->	7	0.00	0.00
70 ->	8	-0.00	0.00
70 ->	9	0.00	0.00
70 ->	10	-0.00	0.00
70 ->	11	-0.00	0.00
70 ->	12	0.00	0.00
70 ->	13	0.00	0.00

70	->	14	0.00	0.00
70	->	15	0.00	0.00
70	->	16	-0.00	0.00
70	->	17	0.00	0.00
70	->	18	-0.00	0.00
70	->	19	-0.00	0.00
70	->	20	0.00	0.00
70	->	21	0.00	0.00
70	->	22	0.00	0.00
70	->	23	0.00	0.00
70	->	24	0.00	0.00
70	->	25	0.00	0.00
70	->	26	0.00	0.00
70	->	27	0.00	0.00
70	->	28	0.00	0.00
70	->	29	0.00	0.00
70	->	30	-0.00	0.00
70	->	31	0.00	0.00
70	->	32	0.00	0.00
70	->	33	-0.00	0.00
70	->	34	0.00	0.00
70	->	35	-0.00	0.00
70	->	36	-0.00	0.00
70	->	37	-0.00	0.00
70	->	38	-0.00	0.00
70	->	39	0.00	0.00
70	->	40	0.00	0.00
70	->	41	0.00	0.00
70	->	42	-0.00	0.00
70	->	43	0.00	0.00
70	->	44	0.00	0.00
70	->	45	0.00	0.00
70	->	46	-0.00	0.00
70	->	47	-0.00	0.00
70	->	48	-0.00	0.00
70	->	49	0.00	0.00
70	->	50	-0.03	0.02
70	->	51	-0.01	0.00
70	->	52	-0.00	0.00
70	->	53	0.03	0.04
70	->	54	-0.04	0.04
70	->	55	-0.01	0.00
70	->	56	-0.00	0.00
70	->	57	-0.00	0.01
70	->	58	-0.29	0.05
70	->	59	-3.24	0.45
70	->	60	-0.92	0.16
70	->	61	-3.82	0.50
70	->	62	-1.16	0.37
70	->	63	-0.98	0.28

70	->	64	-0.03	0.02
70	->	65	-0.03	0.01
70	->	66	0.04	0.04
70	->	67	-0.08	0.01
70	->	68	-3.65	0.77
70	->	69	-10.67	0.56
70	->	70	30.22	1.28
70	->	71	-9.85	0.37
70	->	72	-1.03	0.21
70	->	73	-0.25	0.07
70	->	74	-0.12	0.03
70	->	75	-3.31	1.42
70	->	76	-3.23	0.55
70	->	77	-0.05	0.02
70	->	78	-0.17	0.05
70	->	79	-1.78	0.36
70	->	80	-0.06	0.04
70	->	81	-0.00	0.00
70	->	82	-0.03	0.01
70	->	83	-0.01	0.01
70	->	84	-0.00	0.00
70	->	85	-0.00	0.00
70	->	86	-0.03	0.04
70	->	87	-0.11	0.17
70	->	88	-0.07	0.09
70	->	89	-0.01	0.01
70	->	90	-0.00	0.00
70	->	91	-0.00	0.00
70	->	92	-0.00	0.00
70	->	93	-0.00	0.00
70	->	94	-0.00	0.00
70	->	95	-0.00	0.00
70	->	96	-0.00	0.00
70	->	97	0.00	0.00
70	->	98	0.00	0.00
70	->	99	-0.00	0.00
70	->	100	-0.00	0.00
70	->	101	-0.01	0.01
70	->	102	-0.02	0.01
70	->	103	-0.01	0.01
70	->	104	-0.01	0.00
70	->	105	-0.00	0.00
70	->	106	-0.00	0.00
70	->	107	-0.00	0.00
71	->	1	-0.00	0.00
71	->	2	-0.00	0.00
71	->	3	0.00	0.00
71	->	4	0.00	0.00
71	->	5	0.00	0.00
71	->	6	-0.00	0.00

71	->	7	0.00	0.00
71	->	8	0.00	0.00
71	->	9	0.00	0.00
71	->	10	0.00	0.00
71	->	11	0.00	0.00
71	->	12	-0.00	0.00
71	->	13	-0.00	0.00
71	->	14	0.00	0.00
71	->	15	-0.00	0.00
71	->	16	-0.00	0.00
71	->	17	-0.00	0.00
71	->	18	0.00	0.00
71	->	19	0.00	0.00
71	->	20	0.00	0.00
71	->	21	-0.00	0.00
71	->	22	-0.00	0.00
71	->	23	-0.00	0.00
71	->	24	0.00	0.00
71	->	25	-0.00	0.00
71	->	26	-0.00	0.00
71	->	27	0.00	0.00
71	->	28	-0.00	0.00
71	->	29	-0.00	0.00
71	->	30	0.00	0.00
71	->	31	-0.00	0.00
71	->	32	0.00	0.00
71	->	33	-0.00	0.00
71	->	34	0.00	0.00
71	->	35	-0.00	0.00
71	->	36	-0.00	0.00
71	->	37	-0.00	0.00
71	->	38	-0.00	0.00
71	->	39	0.00	0.00
71	->	40	-0.00	0.00
71	->	41	0.00	0.00
71	->	42	-0.00	0.00
71	->	43	-0.00	0.00
71	->	44	-0.00	0.00
71	->	45	-0.00	0.00
71	->	46	-0.01	0.01
71	->	47	-0.01	0.00
71	->	48	-0.01	0.00
71	->	49	-0.04	0.02
71	->	50	-0.47	0.41
71	->	51	-0.03	0.02
71	->	52	-0.02	0.01
71	->	53	-2.54	1.81
71	->	54	-0.46	0.42
71	->	55	-0.02	0.01
71	->	56	-0.02	0.01

71	->	57	-0.04	0.01
71	->	58	-0.72	0.24
71	->	59	-1.80	0.32
71	->	60	-0.82	0.17
71	->	61	-0.96	0.36
71	->	62	-0.01	0.01
71	->	63	0.01	0.01
71	->	64	0.00	0.00
71	->	65	-0.00	0.00
71	->	66	-0.00	0.00
71	->	67	-0.00	0.00
71	->	68	-0.08	0.03
71	->	69	-0.42	0.06
71	->	70	-9.85	0.36
71	->	71	23.35	1.31
71	->	72	-7.77	0.47
71	->	73	-0.36	0.09
71	->	74	-0.16	0.08
71	->	75	-2.02	2.08
71	->	76	-0.03	0.03
71	->	77	-0.01	0.00
71	->	78	0.00	0.01
71	->	79	-0.00	0.00
71	->	80	-0.00	0.00
71	->	81	0.00	0.00
71	->	82	-0.00	0.00
71	->	83	-0.00	0.00
71	->	84	-0.00	0.00
71	->	85	0.00	0.00
71	->	86	-0.00	0.00
71	->	87	-0.04	0.04
71	->	88	-0.13	0.20
71	->	89	-0.02	0.01
71	->	90	-0.01	0.01
71	->	91	0.00	0.00
71	->	92	0.00	0.00
71	->	93	0.00	0.00
71	->	94	0.00	0.00
71	->	95	0.00	0.00
71	->	96	0.00	0.00
71	->	97	0.00	0.00
71	->	98	0.00	0.00
71	->	99	0.00	0.00
71	->	100	-0.00	0.00
71	->	101	-0.02	0.01
71	->	102	-0.04	0.02
71	->	103	-0.02	0.01
71	->	104	-0.00	0.01
71	->	105	-0.00	0.00
71	->	106	-0.00	0.00

71	->	107	-0.00	0.00
72	->	1	0.02	0.00
72	->	2	0.00	0.00
72	->	3	-0.00	0.00
72	->	4	0.00	0.00
72	->	5	-0.01	0.00
72	->	6	0.00	0.00
72	->	7	0.00	0.00
72	->	8	-0.00	0.00
72	->	9	-0.00	0.00
72	->	10	-0.01	0.00
72	->	11	-0.01	0.00
72	->	12	0.02	0.00
72	->	13	0.00	0.00
72	->	14	0.00	0.00
72	->	15	0.00	0.00
72	->	16	0.02	0.00
72	->	17	-0.00	0.00
72	->	18	-0.02	0.00
72	->	19	-0.02	0.00
72	->	20	-0.00	0.00
72	->	21	0.00	0.00
72	->	22	0.03	0.00
72	->	23	0.00	0.00
72	->	24	-0.00	0.00
72	->	25	0.02	0.00
72	->	26	0.00	0.00
72	->	27	0.00	0.00
72	->	28	0.00	0.00
72	->	29	0.00	0.00
72	->	30	-0.00	0.00
72	->	31	0.03	0.01
72	->	32	-0.00	0.00
72	->	33	-0.00	0.00
72	->	34	0.00	0.00
72	->	35	-0.04	0.01
72	->	36	0.02	0.00
72	->	37	-0.00	0.00
72	->	38	-0.05	0.01
72	->	39	-0.00	0.00
72	->	40	0.05	0.01
72	->	41	-0.00	0.00
72	->	42	-0.03	0.04
72	->	43	-0.00	0.00
72	->	44	0.07	0.03
72	->	45	-0.01	0.00
72	->	46	-0.11	0.04
72	->	47	-0.04	0.02
72	->	48	-0.02	0.01
72	->	49	0.02	0.02

72	->	50	-1.36	0.37
72	->	51	-0.09	0.04
72	->	52	-0.02	0.01
72	->	53	-0.32	0.36
72	->	54	-0.29	0.33
72	->	55	-0.01	0.01
72	->	56	-0.00	0.00
72	->	57	-0.01	0.00
72	->	58	-0.04	0.01
72	->	59	-0.05	0.06
72	->	60	-0.12	0.07
72	->	61	-1.42	0.79
72	->	62	-0.02	0.01
72	->	63	-0.04	0.01
72	->	64	-0.02	0.00
72	->	65	-0.00	0.00
72	->	66	-0.02	0.00
72	->	67	-0.00	0.00
72	->	68	0.04	0.03
72	->	69	-0.01	0.03
72	->	70	-1.02	0.21
72	->	71	-7.86	0.47
72	->	72	-96.02	3.38
72	->	73	-21.39	0.56
72	->	74	-10.90	5.27
72	->	75	-3.85	2.19
72	->	76	-1.04	0.43
72	->	77	0.06	0.04
72	->	78	0.26	0.17
72	->	79	-0.01	0.01
72	->	80	-0.01	0.01
72	->	81	0.03	0.01
72	->	82	-0.00	0.00
72	->	83	-0.00	0.00
72	->	84	-0.00	0.00
72	->	85	0.00	0.01
72	->	86	-0.04	0.01
72	->	87	-0.24	0.16
72	->	88	-2.38	0.87
72	->	89	-8.33	1.35
72	->	90	-1.94	1.17
72	->	91	-0.16	0.22
72	->	92	-0.02	0.02
72	->	93	-0.02	0.01
72	->	94	-0.02	0.00
72	->	95	-0.01	0.00
72	->	96	-0.01	0.00
72	->	97	-0.00	0.00
72	->	98	-0.01	0.00
72	->	99	-0.02	0.00

72	->	100	-0.05	0.02
72	->	101	-0.14	0.07
72	->	102	-0.34	0.18
72	->	103	-0.34	0.21
72	->	104	-0.30	0.84
72	->	105	0.43	0.36
72	->	106	0.10	0.15
72	->	107	-0.05	0.01
73	->	1	0.00	0.00
73	->	2	0.00	0.00
73	->	3	-0.00	0.00
73	->	4	0.00	0.00
73	->	5	-0.00	0.00
73	->	6	0.00	0.00
73	->	7	0.00	0.00
73	->	8	-0.00	0.00
73	->	9	0.00	0.00
73	->	10	-0.00	0.00
73	->	11	-0.00	0.00
73	->	12	0.00	0.00
73	->	13	0.00	0.00
73	->	14	0.00	0.00
73	->	15	0.00	0.00
73	->	16	0.00	0.00
73	->	17	-0.00	0.00
73	->	18	-0.00	0.00
73	->	19	-0.00	0.00
73	->	20	0.00	0.00
73	->	21	0.00	0.00
73	->	22	0.00	0.00
73	->	23	0.00	0.00
73	->	24	0.00	0.00
73	->	25	0.00	0.00
73	->	26	0.00	0.00
73	->	27	0.00	0.00
73	->	28	0.00	0.00
73	->	29	0.00	0.00
73	->	30	-0.00	0.00
73	->	31	0.00	0.00
73	->	32	0.00	0.00
73	->	33	-0.00	0.00
73	->	34	-0.00	0.00
73	->	35	-0.00	0.00
73	->	36	0.00	0.00
73	->	37	-0.00	0.00
73	->	38	-0.00	0.00
73	->	39	0.00	0.00
73	->	40	0.00	0.00
73	->	41	-0.00	0.00
73	->	42	-0.00	0.00

73	->	43	-0.00	0.00
73	->	44	-0.00	0.01
73	->	45	-0.00	0.00
73	->	46	-0.01	0.01
73	->	47	-0.00	0.01
73	->	48	-0.00	0.00
73	->	49	-0.01	0.01
73	->	50	-0.03	0.05
73	->	51	0.00	0.00
73	->	52	-0.00	0.00
73	->	53	-0.11	0.09
73	->	54	0.00	0.01
73	->	55	-0.00	0.00
73	->	56	-0.00	0.00
73	->	57	-0.00	0.00
73	->	58	-0.01	0.00
73	->	59	-0.05	0.02
73	->	60	-0.04	0.02
73	->	61	-1.22	0.74
73	->	62	-0.01	0.01
73	->	63	-0.01	0.01
73	->	64	-0.00	0.00
73	->	65	-0.00	0.00
73	->	66	0.00	0.00
73	->	67	-0.00	0.00
73	->	68	-0.01	0.00
73	->	69	-0.04	0.01
73	->	70	-0.25	0.07
73	->	71	-0.37	0.10
73	->	72	-21.39	0.56
73	->	73	23.33	2.05
73	->	74	-10.74	0.50
73	->	75	-0.75	0.14
73	->	76	-3.15	0.90
73	->	77	-1.97	0.90
73	->	78	-0.08	0.05
73	->	79	-0.05	0.03
73	->	80	-0.02	0.01
73	->	81	-0.01	0.00
73	->	82	-0.00	0.00
73	->	83	-0.00	0.00
73	->	84	-0.00	0.00
73	->	85	-0.00	0.00
73	->	86	-0.00	0.00
73	->	87	-0.01	0.01
73	->	88	-0.00	0.01
73	->	89	-0.02	0.01
73	->	90	-0.01	0.00
73	->	91	0.00	0.02
73	->	92	-0.02	0.03

73	->	93	-0.02	0.02
73	->	94	-0.01	0.00
73	->	95	-0.00	0.00
73	->	96	-0.00	0.00
73	->	97	-0.00	0.00
73	->	98	-0.00	0.00
73	->	99	-0.00	0.00
73	->	100	-0.01	0.02
73	->	101	-0.30	0.40
73	->	102	-2.57	2.12
73	->	103	-1.51	1.66
73	->	104	-0.25	0.38
73	->	105	-0.00	0.01
73	->	106	-0.00	0.00
73	->	107	-0.00	0.00
74	->	1	-0.02	0.00
74	->	2	-0.00	0.00
74	->	3	0.00	0.00
74	->	4	-0.00	0.00
74	->	5	0.01	0.00
74	->	6	-0.00	0.00
74	->	7	-0.00	0.00
74	->	8	0.00	0.00
74	->	9	0.00	0.00
74	->	10	0.01	0.00
74	->	11	0.01	0.00
74	->	12	-0.02	0.00
74	->	13	-0.00	0.00
74	->	14	-0.00	0.00
74	->	15	-0.00	0.00
74	->	16	-0.01	0.00
74	->	17	0.00	0.00
74	->	18	0.01	0.00
74	->	19	0.02	0.00
74	->	20	0.00	0.00
74	->	21	-0.00	0.00
74	->	22	-0.03	0.01
74	->	23	-0.00	0.00
74	->	24	0.00	0.00
74	->	25	-0.01	0.00
74	->	26	-0.00	0.00
74	->	27	-0.00	0.00
74	->	28	-0.00	0.00
74	->	29	-0.00	0.00
74	->	30	0.00	0.00
74	->	31	-0.02	0.01
74	->	32	-0.00	0.00
74	->	33	0.00	0.00
74	->	34	-0.00	0.00
74	->	35	0.02	0.00

74 ->	36	-0.01	0.00
74 ->	37	0.00	0.00
74 ->	38	0.02	0.01
74 ->	39	-0.00	0.00
74 ->	40	-0.03	0.01
74 ->	41	0.00	0.00
74 ->	42	-0.01	0.00
74 ->	43	-0.00	0.00
74 ->	44	-0.05	0.03
74 ->	45	0.00	0.00
74 ->	46	0.05	0.02
74 ->	47	-0.01	0.02
74 ->	48	0.00	0.00
74 ->	49	-0.01	0.01
74 ->	50	-0.04	0.03
74 ->	51	-0.01	0.00
74 ->	52	-0.00	0.00
74 ->	53	-0.09	0.04
74 ->	54	-0.00	0.01
74 ->	55	-0.00	0.00
74 ->	56	-0.00	0.00
74 ->	57	-0.00	0.00
74 ->	58	0.03	0.00
74 ->	59	-0.07	0.02
74 ->	60	-0.01	0.00
74 ->	61	-0.00	0.02
74 ->	62	0.00	0.00
74 ->	63	0.04	0.01
74 ->	64	0.02	0.00
74 ->	65	0.00	0.00
74 ->	66	0.03	0.00
74 ->	67	-0.00	0.00
74 ->	68	-0.06	0.01
74 ->	69	-0.04	0.01
74 ->	70	-0.12	0.03
74 ->	71	-0.16	0.08
74 ->	72	-10.83	5.24
74 ->	73	-10.78	0.50
74 ->	74	-15.10	3.46
74 ->	75	-13.21	0.61
74 ->	76	-1.71	0.39
74 ->	77	-2.54	0.48
74 ->	78	-4.78	3.65
74 ->	79	-0.14	0.07
74 ->	80	-0.04	0.02
74 ->	81	-0.11	0.05
74 ->	82	-0.01	0.00
74 ->	83	-0.01	0.00
74 ->	84	-0.00	0.00
74 ->	85	-0.00	0.01

74	->	86	-0.01	0.02
74	->	87	0.02	0.09
74	->	88	-1.79	2.08
74	->	89	0.38	0.34
74	->	90	0.08	0.12
74	->	91	-0.05	0.07
74	->	92	-0.03	0.03
74	->	93	0.01	0.01
74	->	94	0.02	0.00
74	->	95	0.01	0.00
74	->	96	0.01	0.00
74	->	97	0.00	0.00
74	->	98	0.01	0.00
74	->	99	0.01	0.00
74	->	100	0.02	0.01
74	->	101	0.04	0.02
74	->	102	-0.03	0.13
74	->	103	-1.30	1.18
74	->	104	-2.47	1.47
74	->	105	-2.19	1.78
74	->	106	0.09	0.18
74	->	107	0.00	0.02
75	->	1	-0.01	0.00
75	->	2	-0.00	0.00
75	->	3	0.00	0.00
75	->	4	-0.00	0.00
75	->	5	0.00	0.00
75	->	6	-0.00	0.00
75	->	7	-0.00	0.00
75	->	8	0.00	0.00
75	->	9	0.00	0.00
75	->	10	0.00	0.00
75	->	11	0.00	0.00
75	->	12	-0.01	0.00
75	->	13	-0.00	0.00
75	->	14	-0.00	0.00
75	->	15	-0.00	0.00
75	->	16	-0.01	0.00
75	->	17	0.00	0.00
75	->	18	0.01	0.00
75	->	19	0.01	0.00
75	->	20	0.00	0.00
75	->	21	-0.00	0.00
75	->	22	-0.01	0.00
75	->	23	-0.00	0.00
75	->	24	0.00	0.00
75	->	25	-0.01	0.00
75	->	26	-0.00	0.00
75	->	27	-0.00	0.00
75	->	28	-0.00	0.00

75	->	29	-0.00	0.00
75	->	30	0.00	0.00
75	->	31	-0.01	0.01
75	->	32	-0.00	0.00
75	->	33	0.00	0.00
75	->	34	-0.00	0.00
75	->	35	0.02	0.00
75	->	36	-0.01	0.00
75	->	37	0.00	0.00
75	->	38	0.02	0.00
75	->	39	-0.00	0.00
75	->	40	-0.03	0.01
75	->	41	0.00	0.00
75	->	42	-0.00	0.00
75	->	43	-0.00	0.00
75	->	44	-0.02	0.01
75	->	45	0.00	0.00
75	->	46	0.03	0.01
75	->	47	-0.00	0.00
75	->	48	0.00	0.00
75	->	49	-0.00	0.00
75	->	50	-0.01	0.01
75	->	51	-0.01	0.00
75	->	52	-0.00	0.00
75	->	53	-0.14	0.10
75	->	54	-0.02	0.03
75	->	55	-0.00	0.00
75	->	56	-0.00	0.00
75	->	57	-0.01	0.00
75	->	58	0.05	0.01
75	->	59	-0.55	0.41
75	->	60	-0.02	0.01
75	->	61	-0.09	0.04
75	->	62	-0.01	0.01
75	->	63	-0.00	0.02
75	->	64	0.02	0.00
75	->	65	0.00	0.00
75	->	66	0.05	0.01
75	->	67	-0.00	0.00
75	->	68	-2.67	3.61
75	->	69	-0.24	0.18
75	->	70	-3.29	1.40
75	->	71	-1.99	2.05
75	->	72	-3.82	2.17
75	->	73	-0.76	0.14
75	->	74	-13.28	0.62
75	->	75	-49.87	4.71
75	->	76	-7.64	0.54
75	->	77	-1.24	0.18
75	->	78	-3.72	1.06

75	->	79	-3.31	0.69
75	->	80	-0.17	0.07
75	->	81	-0.08	0.02
75	->	82	-0.04	0.02
75	->	83	-0.02	0.01
75	->	84	-0.00	0.00
75	->	85	-0.00	0.01
75	->	86	-0.07	0.09
75	->	87	-0.27	0.53
75	->	88	-0.55	1.42
75	->	89	0.05	0.07
75	->	90	0.02	0.03
75	->	91	0.01	0.01
75	->	92	0.00	0.00
75	->	93	0.01	0.00
75	->	94	0.01	0.00
75	->	95	0.01	0.00
75	->	96	0.00	0.00
75	->	97	0.00	0.00
75	->	98	0.01	0.00
75	->	99	0.01	0.00
75	->	100	0.02	0.00
75	->	101	0.02	0.01
75	->	102	0.00	0.02
75	->	103	-0.01	0.03
75	->	104	-0.00	0.06
75	->	105	-0.09	0.17
75	->	106	0.02	0.02
75	->	107	0.01	0.01
76	->	1	0.02	0.00
76	->	2	0.00	0.00
76	->	3	-0.00	0.00
76	->	4	0.00	0.00
76	->	5	-0.00	0.00
76	->	6	0.00	0.00
76	->	7	0.00	0.00
76	->	8	-0.00	0.00
76	->	9	-0.00	0.00
76	->	10	-0.00	0.00
76	->	11	-0.00	0.00
76	->	12	0.01	0.00
76	->	13	0.00	0.00
76	->	14	0.00	0.00
76	->	15	0.00	0.00
76	->	16	0.01	0.01
76	->	17	-0.00	0.00
76	->	18	-0.01	0.00
76	->	19	-0.01	0.01
76	->	20	0.00	0.00
76	->	21	0.00	0.00

76 ->	22	0.02	0.00
76 ->	23	0.00	0.00
76 ->	24	0.00	0.00
76 ->	25	0.01	0.00
76 ->	26	0.00	0.00
76 ->	27	0.00	0.00
76 ->	28	0.00	0.00
76 ->	29	0.00	0.00
76 ->	30	-0.00	0.00
76 ->	31	0.01	0.00
76 ->	32	0.00	0.00
76 ->	33	0.00	0.00
76 ->	34	0.00	0.00
76 ->	35	-0.01	0.00
76 ->	36	0.01	0.00
76 ->	37	-0.00	0.00
76 ->	38	-0.01	0.00
76 ->	39	0.00	0.00
76 ->	40	0.01	0.00
76 ->	41	-0.00	0.00
76 ->	42	0.00	0.00
76 ->	43	0.00	0.00
76 ->	44	0.02	0.01
76 ->	45	-0.00	0.00
76 ->	46	-0.04	0.01
76 ->	47	-0.00	0.00
76 ->	48	-0.00	0.00
76 ->	49	0.00	0.00
76 ->	50	-0.01	0.01
76 ->	51	0.00	0.00
76 ->	52	-0.00	0.00
76 ->	53	0.04	0.01
76 ->	54	-0.00	0.00
76 ->	55	0.00	0.00
76 ->	56	-0.00	0.00
76 ->	57	-0.00	0.00
76 ->	58	-0.04	0.01
76 ->	59	0.04	0.01
76 ->	60	-0.03	0.02
76 ->	61	-1.46	0.76
76 ->	62	-2.00	1.61
76 ->	63	-1.27	0.70
76 ->	64	-0.91	1.50
76 ->	65	-0.03	0.02
76 ->	66	-0.10	0.03
76 ->	67	-0.01	0.00
76 ->	68	-0.07	0.05
76 ->	69	0.11	0.07
76 ->	70	-3.30	0.56
76 ->	71	-0.03	0.03

76 ->	72	-1.06	0.43
76 ->	73	-3.18	0.90
76 ->	74	-1.71	0.39
76 ->	75	-7.68	0.53
76 ->	76	-103.07	4.42
76 ->	77	-11.69	0.55
76 ->	78	-0.82	0.26
76 ->	79	-2.16	0.42
76 ->	80	-2.90	0.99
76 ->	81	-0.01	0.04
76 ->	82	-0.04	0.02
76 ->	83	-0.07	0.02
76 ->	84	-0.01	0.01
76 ->	85	-0.00	0.01
76 ->	86	-0.02	0.01
76 ->	87	-0.04	0.01
76 ->	88	-0.02	0.01
76 ->	89	-0.02	0.00
76 ->	90	-0.02	0.00
76 ->	91	-0.01	0.00
76 ->	92	-0.00	0.01
76 ->	93	-0.01	0.01
76 ->	94	-0.03	0.01
76 ->	95	-0.02	0.01
76 ->	96	-0.01	0.01
76 ->	97	-0.00	0.00
76 ->	98	-0.01	0.00
76 ->	99	-0.02	0.01
76 ->	100	-0.04	0.03
76 ->	101	-0.51	0.65
76 ->	102	-3.77	4.87
76 ->	103	-1.50	2.48
76 ->	104	-0.14	0.08
76 ->	105	-0.02	0.01
76 ->	106	-0.02	0.00
76 ->	107	-0.01	0.00
77 ->	1	0.03	0.01
77 ->	2	0.00	0.00
77 ->	3	-0.00	0.00
77 ->	4	0.00	0.00
77 ->	5	-0.01	0.00
77 ->	6	0.00	0.00
77 ->	7	0.00	0.00
77 ->	8	-0.00	0.00
77 ->	9	-0.00	0.00
77 ->	10	-0.01	0.00
77 ->	11	-0.01	0.00
77 ->	12	0.02	0.00
77 ->	13	0.01	0.00
77 ->	14	0.00	0.00

77	->	15	0.00	0.00
77	->	16	0.02	0.00
77	->	17	-0.00	0.00
77	->	18	-0.01	0.00
77	->	19	-0.02	0.00
77	->	20	-0.00	0.00
77	->	21	0.00	0.00
77	->	22	0.02	0.00
77	->	23	0.00	0.00
77	->	24	0.00	0.00
77	->	25	0.01	0.00
77	->	26	0.00	0.00
77	->	27	0.00	0.00
77	->	28	0.00	0.00
77	->	29	0.00	0.00
77	->	30	-0.00	0.00
77	->	31	0.01	0.00
77	->	32	0.00	0.00
77	->	33	-0.00	0.00
77	->	34	0.00	0.00
77	->	35	-0.01	0.00
77	->	36	0.00	0.00
77	->	37	-0.00	0.00
77	->	38	-0.01	0.00
77	->	39	0.00	0.00
77	->	40	0.01	0.00
77	->	41	-0.00	0.00
77	->	42	0.00	0.00
77	->	43	0.00	0.00
77	->	44	0.02	0.01
77	->	45	-0.00	0.00
77	->	46	-0.03	0.00
77	->	47	0.00	0.00
77	->	48	-0.00	0.00
77	->	49	-0.00	0.00
77	->	50	-0.00	0.00
77	->	51	0.00	0.00
77	->	52	0.00	0.00
77	->	53	0.02	0.01
77	->	54	-0.00	0.00
77	->	55	0.00	0.00
77	->	56	0.00	0.00
77	->	57	0.00	0.00
77	->	58	-0.02	0.00
77	->	59	0.01	0.00
77	->	60	-0.00	0.00
77	->	61	-0.02	0.02
77	->	62	-0.01	0.01
77	->	63	-0.03	0.02
77	->	64	-0.04	0.01

77	->	65	-0.01	0.00
77	->	66	-0.03	0.01
77	->	67	-0.00	0.00
77	->	68	0.01	0.01
77	->	69	-0.00	0.01
77	->	70	-0.05	0.02
77	->	71	-0.01	0.00
77	->	72	0.06	0.04
77	->	73	-2.01	0.91
77	->	74	-2.57	0.48
77	->	75	-1.24	0.18
77	->	76	-11.72	0.56
77	->	77	13.47	3.59
77	->	78	-6.75	0.43
77	->	79	-0.84	0.22
77	->	80	-2.09	0.51
77	->	81	-1.65	0.54
77	->	82	-0.05	0.03
77	->	83	-0.03	0.02
77	->	84	-0.03	0.02
77	->	85	-0.01	0.02
77	->	86	-0.02	0.01
77	->	87	-0.05	0.01
77	->	88	-0.01	0.01
77	->	89	-0.03	0.00
77	->	90	-0.02	0.00
77	->	91	-0.01	0.01
77	->	92	-0.01	0.02
77	->	93	-0.03	0.01
77	->	94	-0.03	0.01
77	->	95	-0.03	0.01
77	->	96	-0.01	0.00
77	->	97	-0.00	0.00
77	->	98	-0.01	0.00
77	->	99	-0.01	0.00
77	->	100	-0.02	0.00
77	->	101	-0.05	0.02
77	->	102	-0.73	0.56
77	->	103	-6.37	4.22
77	->	104	-5.33	2.91
77	->	105	-0.22	0.28
77	->	106	-0.05	0.02
77	->	107	-0.01	0.00
78	->	1	0.02	0.00
78	->	2	0.00	0.00
78	->	3	-0.00	0.00
78	->	4	0.00	0.00
78	->	5	-0.01	0.00
78	->	6	0.00	0.00
78	->	7	-0.00	0.00

78	->	8	-0.00	0.00
78	->	9	-0.00	0.00
78	->	10	-0.01	0.00
78	->	11	-0.01	0.00
78	->	12	0.02	0.00
78	->	13	0.00	0.00
78	->	14	0.00	0.00
78	->	15	0.00	0.00
78	->	16	0.01	0.00
78	->	17	-0.00	0.00
78	->	18	-0.01	0.00
78	->	19	-0.01	0.00
78	->	20	-0.00	0.00
78	->	21	-0.00	0.00
78	->	22	0.02	0.00
78	->	23	0.00	0.00
78	->	24	-0.00	0.00
78	->	25	0.01	0.00
78	->	26	0.00	0.00
78	->	27	0.00	0.00
78	->	28	0.00	0.00
78	->	29	0.00	0.00
78	->	30	-0.00	0.00
78	->	31	0.01	0.00
78	->	32	0.00	0.00
78	->	33	-0.00	0.00
78	->	34	0.00	0.00
78	->	35	-0.01	0.00
78	->	36	0.01	0.00
78	->	37	-0.00	0.00
78	->	38	-0.02	0.00
78	->	39	0.00	0.00
78	->	40	0.02	0.00
78	->	41	-0.00	0.00
78	->	42	0.00	0.00
78	->	43	0.00	0.00
78	->	44	0.02	0.00
78	->	45	-0.00	0.00
78	->	46	-0.03	0.00
78	->	47	-0.00	0.00
78	->	48	-0.00	0.00
78	->	49	-0.00	0.00
78	->	50	-0.00	0.00
78	->	51	0.00	0.00
78	->	52	-0.00	0.00
78	->	53	0.03	0.01
78	->	54	-0.00	0.00
78	->	55	0.00	0.00
78	->	56	0.00	0.00
78	->	57	0.00	0.00

78	->	58	-0.02	0.00
78	->	59	0.04	0.01
78	->	60	-0.00	0.00
78	->	61	-0.01	0.00
78	->	62	0.00	0.00
78	->	63	-0.14	0.03
78	->	64	-0.03	0.01
78	->	65	-0.01	0.00
78	->	66	-0.07	0.01
78	->	67	-0.00	0.00
78	->	68	0.09	0.04
78	->	69	0.03	0.01
78	->	70	-0.17	0.05
78	->	71	0.00	0.01
78	->	72	0.26	0.17
78	->	73	-0.08	0.05
78	->	74	-4.83	3.66
78	->	75	-3.75	1.06
78	->	76	-0.83	0.26
78	->	77	-6.78	0.43
78	->	78	-103.39	2.87
78	->	79	-19.63	0.35
78	->	80	-0.71	0.15
78	->	81	-2.02	0.48
78	->	82	-1.57	0.83
78	->	83	-0.12	0.04
78	->	84	-0.02	0.01
78	->	85	-0.02	0.01
78	->	86	-1.41	1.52
78	->	87	-4.68	2.69
78	->	88	-0.14	0.72
78	->	89	-0.14	0.12
78	->	90	-0.04	0.01
78	->	91	-0.03	0.01
78	->	92	-0.02	0.01
78	->	93	-0.02	0.00
78	->	94	-0.01	0.00
78	->	95	-0.01	0.00
78	->	96	-0.00	0.00
78	->	97	-0.00	0.00
78	->	98	-0.01	0.00
78	->	99	-0.01	0.00
78	->	100	-0.01	0.00
78	->	101	-0.02	0.01
78	->	102	-0.04	0.01
78	->	103	-0.09	0.02
78	->	104	-0.22	0.14
78	->	105	-1.04	1.31
78	->	106	-3.68	3.03
78	->	107	-0.08	0.42

79	->	1	0.00	0.00
79	->	2	0.00	0.00
79	->	3	0.00	0.00
79	->	4	0.00	0.00
79	->	5	-0.00	0.00
79	->	6	0.00	0.00
79	->	7	0.00	0.00
79	->	8	-0.00	0.00
79	->	9	0.00	0.00
79	->	10	-0.00	0.00
79	->	11	-0.00	0.00
79	->	12	0.00	0.00
79	->	13	0.00	0.00
79	->	14	0.00	0.00
79	->	15	0.00	0.00
79	->	16	0.00	0.00
79	->	17	-0.00	0.00
79	->	18	-0.00	0.00
79	->	19	-0.00	0.00
79	->	20	0.00	0.00
79	->	21	-0.00	0.00
79	->	22	0.00	0.00
79	->	23	0.00	0.00
79	->	24	0.00	0.00
79	->	25	0.00	0.00
79	->	26	0.00	0.00
79	->	27	0.00	0.00
79	->	28	0.00	0.00
79	->	29	0.00	0.00
79	->	30	-0.00	0.00
79	->	31	0.00	0.00
79	->	32	0.00	0.00
79	->	33	-0.00	0.00
79	->	34	0.00	0.00
79	->	35	-0.00	0.00
79	->	36	0.00	0.00
79	->	37	-0.00	0.00
79	->	38	-0.00	0.00
79	->	39	0.00	0.00
79	->	40	0.00	0.00
79	->	41	-0.00	0.00
79	->	42	0.00	0.00
79	->	43	0.00	0.00
79	->	44	0.00	0.00
79	->	45	-0.00	0.00
79	->	46	-0.00	0.00
79	->	47	-0.00	0.00
79	->	48	-0.00	0.00
79	->	49	0.00	0.00
79	->	50	-0.00	0.00

79	->	51	-0.00	0.00
79	->	52	0.00	0.00
79	->	53	0.00	0.00
79	->	54	-0.00	0.00
79	->	55	0.00	0.00
79	->	56	0.00	0.00
79	->	57	-0.00	0.00
79	->	58	-0.00	0.00
79	->	59	-0.00	0.00
79	->	60	-0.00	0.00
79	->	61	-0.02	0.00
79	->	62	-0.09	0.02
79	->	63	3.05	0.61
79	->	64	-0.07	0.06
79	->	65	0.70	0.11
79	->	66	3.54	0.73
79	->	67	-0.06	0.03
79	->	68	-2.17	0.63
79	->	69	-0.02	0.01
79	->	70	-1.78	0.36
79	->	71	-0.00	0.00
79	->	72	-0.01	0.01
79	->	73	-0.05	0.03
79	->	74	-0.14	0.07
79	->	75	-3.33	0.68
79	->	76	-2.17	0.42
79	->	77	-0.85	0.22
79	->	78	-19.69	0.35
79	->	79	10.02	1.10
79	->	80	-4.85	0.47
79	->	81	-1.17	0.22
79	->	82	-3.50	0.86
79	->	83	17.46	8.64
79	->	84	-0.10	0.05
79	->	85	-0.03	0.02
79	->	86	-0.04	0.06
79	->	87	-0.04	0.02
79	->	88	-0.01	0.00
79	->	89	-0.01	0.00
79	->	90	-0.00	0.00
79	->	91	-0.00	0.00
79	->	92	-0.00	0.00
79	->	93	-0.00	0.00
79	->	94	-0.00	0.00
79	->	95	-0.00	0.00
79	->	96	-0.00	0.00
79	->	97	-0.00	0.00
79	->	98	-0.00	0.00
79	->	99	-0.00	0.00
79	->	100	-0.00	0.00

79	->	101	-0.00	0.00
79	->	102	-0.01	0.01
79	->	103	-0.02	0.01
79	->	104	-0.01	0.01
79	->	105	-0.01	0.00
79	->	106	-0.01	0.00
79	->	107	-0.00	0.00
80	->	1	0.00	0.00
80	->	2	0.00	0.00
80	->	3	0.00	0.00
80	->	4	0.00	0.00
80	->	5	-0.00	0.00
80	->	6	0.00	0.00
80	->	7	0.00	0.00
80	->	8	-0.00	0.00
80	->	9	0.00	0.00
80	->	10	-0.00	0.00
80	->	11	-0.00	0.00
80	->	12	0.00	0.00
80	->	13	0.00	0.00
80	->	14	0.00	0.00
80	->	15	0.00	0.00
80	->	16	0.00	0.00
80	->	17	0.00	0.00
80	->	18	-0.00	0.00
80	->	19	-0.00	0.00
80	->	20	0.00	0.00
80	->	21	0.00	0.00
80	->	22	0.00	0.00
80	->	23	0.00	0.00
80	->	24	0.00	0.00
80	->	25	0.00	0.00
80	->	26	0.00	0.00
80	->	27	0.00	0.00
80	->	28	0.00	0.00
80	->	29	0.00	0.00
80	->	30	-0.00	0.00
80	->	31	0.00	0.00
80	->	32	0.00	0.00
80	->	33	-0.00	0.00
80	->	34	0.00	0.00
80	->	35	-0.00	0.00
80	->	36	0.00	0.00
80	->	37	-0.00	0.00
80	->	38	-0.00	0.00
80	->	39	0.00	0.00
80	->	40	0.00	0.00
80	->	41	-0.00	0.00
80	->	42	0.00	0.00
80	->	43	0.00	0.00

80	->	44	0.00	0.00
80	->	45	-0.00	0.00
80	->	46	-0.00	0.00
80	->	47	0.00	0.00
80	->	48	0.00	0.00
80	->	49	-0.00	0.00
80	->	50	-0.00	0.00
80	->	51	0.00	0.00
80	->	52	0.00	0.00
80	->	53	0.00	0.00
80	->	54	0.00	0.00
80	->	55	0.00	0.00
80	->	56	0.00	0.00
80	->	57	0.00	0.00
80	->	58	-0.00	0.00
80	->	59	-0.00	0.00
80	->	60	-0.00	0.00
80	->	61	-0.01	0.00
80	->	62	-0.03	0.01
80	->	63	0.14	0.18
80	->	64	-0.17	0.21
80	->	65	-0.09	0.09
80	->	66	0.03	0.01
80	->	67	-0.00	0.00
80	->	68	-0.04	0.01
80	->	69	-0.01	0.00
80	->	70	-0.06	0.04
80	->	71	-0.00	0.00
80	->	72	-0.01	0.01
80	->	73	-0.02	0.01
80	->	74	-0.04	0.02
80	->	75	-0.17	0.07
80	->	76	-2.87	0.99
80	->	77	-2.09	0.51
80	->	78	-0.72	0.15
80	->	79	-4.88	0.47
80	->	80	-22.95	2.06
80	->	81	-15.31	0.46
80	->	82	-0.87	0.18
80	->	83	-2.80	0.57
80	->	84	-1.97	1.16
80	->	85	-0.06	0.06
80	->	86	-0.00	0.00
80	->	87	-0.00	0.00
80	->	88	-0.00	0.00
80	->	89	-0.00	0.00
80	->	90	-0.00	0.00
80	->	91	-0.00	0.00
80	->	92	-0.00	0.00
80	->	93	-0.00	0.00

80	->	94	-0.00	0.00
80	->	95	-0.00	0.00
80	->	96	-0.00	0.00
80	->	97	-0.00	0.00
80	->	98	-0.00	0.00
80	->	99	-0.00	0.00
80	->	100	-0.00	0.00
80	->	101	-0.00	0.00
80	->	102	-0.01	0.01
80	->	103	-0.04	0.04
80	->	104	-0.02	0.02
80	->	105	-0.01	0.00
80	->	106	-0.00	0.00
80	->	107	-0.00	0.00
81	->	1	0.02	0.01
81	->	2	0.00	0.00
81	->	3	-0.00	0.00
81	->	4	0.00	0.00
81	->	5	-0.00	0.00
81	->	6	0.00	0.00
81	->	7	-0.00	0.00
81	->	8	-0.00	0.00
81	->	9	-0.00	0.00
81	->	10	-0.01	0.00
81	->	11	-0.01	0.00
81	->	12	0.02	0.01
81	->	13	0.00	0.00
81	->	14	0.00	0.00
81	->	15	0.00	0.00
81	->	16	0.01	0.00
81	->	17	-0.00	0.00
81	->	18	-0.01	0.00
81	->	19	-0.01	0.00
81	->	20	-0.00	0.00
81	->	21	0.00	0.00
81	->	22	0.01	0.00
81	->	23	0.00	0.00
81	->	24	0.00	0.00
81	->	25	0.00	0.00
81	->	26	0.00	0.00
81	->	27	0.00	0.00
81	->	28	0.00	0.00
81	->	29	0.00	0.00
81	->	30	-0.00	0.00
81	->	31	0.01	0.00
81	->	32	0.00	0.00
81	->	33	-0.00	0.00
81	->	34	0.00	0.00
81	->	35	-0.01	0.00
81	->	36	0.00	0.00

81	->	37	-0.00	0.00
81	->	38	-0.01	0.00
81	->	39	0.00	0.00
81	->	40	0.01	0.00
81	->	41	-0.00	0.00
81	->	42	0.00	0.00
81	->	43	0.00	0.00
81	->	44	0.01	0.00
81	->	45	-0.00	0.00
81	->	46	-0.01	0.00
81	->	47	0.00	0.00
81	->	48	-0.00	0.00
81	->	49	0.00	0.00
81	->	50	0.00	0.00
81	->	51	0.00	0.00
81	->	52	-0.00	0.00
81	->	53	0.01	0.00
81	->	54	0.00	0.00
81	->	55	0.00	0.00
81	->	56	0.00	0.00
81	->	57	0.00	0.00
81	->	58	-0.01	0.00
81	->	59	0.02	0.00
81	->	60	-0.00	0.00
81	->	61	-0.00	0.00
81	->	62	-0.00	0.00
81	->	63	-0.01	0.02
81	->	64	-0.03	0.01
81	->	65	-0.01	0.00
81	->	66	-0.03	0.01
81	->	67	-0.00	0.00
81	->	68	0.03	0.01
81	->	69	0.01	0.00
81	->	70	-0.00	0.00
81	->	71	0.00	0.00
81	->	72	0.03	0.01
81	->	73	-0.01	0.00
81	->	74	-0.11	0.05
81	->	75	-0.08	0.02
81	->	76	-0.01	0.04
81	->	77	-1.67	0.53
81	->	78	-2.05	0.48
81	->	79	-1.18	0.22
81	->	80	-15.35	0.46
81	->	81	5.76	3.13
81	->	82	-11.44	0.49
81	->	83	-0.64	0.20
81	->	84	-1.50	0.70
81	->	85	-0.96	1.16
81	->	86	-0.04	0.05

81	->	87	-0.05	0.01
81	->	88	-0.02	0.00
81	->	89	-0.02	0.00
81	->	90	-0.02	0.00
81	->	91	-0.01	0.00
81	->	92	-0.01	0.00
81	->	93	-0.02	0.01
81	->	94	-0.01	0.01
81	->	95	-0.01	0.00
81	->	96	-0.00	0.00
81	->	97	-0.00	0.00
81	->	98	-0.00	0.00
81	->	99	-0.00	0.00
81	->	100	-0.01	0.00
81	->	101	-0.02	0.01
81	->	102	-0.04	0.02
81	->	103	-0.17	0.26
81	->	104	-0.84	1.88
81	->	105	-0.18	0.22
81	->	106	-0.07	0.02
81	->	107	-0.01	0.00
82	->	1	0.00	0.00
82	->	2	0.00	0.00
82	->	3	0.00	0.00
82	->	4	0.00	0.00
82	->	5	-0.00	0.00
82	->	6	0.00	0.00
82	->	7	0.00	0.00
82	->	8	-0.00	0.00
82	->	9	0.00	0.00
82	->	10	-0.00	0.00
82	->	11	-0.00	0.00
82	->	12	0.00	0.00
82	->	13	0.00	0.00
82	->	14	0.00	0.00
82	->	15	0.00	0.00
82	->	16	0.00	0.00
82	->	17	-0.00	0.00
82	->	18	-0.00	0.00
82	->	19	-0.00	0.00
82	->	20	0.00	0.00
82	->	21	0.00	0.00
82	->	22	0.00	0.00
82	->	23	0.00	0.00
82	->	24	-0.00	0.00
82	->	25	0.00	0.00
82	->	26	0.00	0.00
82	->	27	0.00	0.00
82	->	28	0.00	0.00
82	->	29	0.00	0.00

82	->	30	-0.00	0.00
82	->	31	0.00	0.00
82	->	32	0.00	0.00
82	->	33	0.00	0.00
82	->	34	0.00	0.00
82	->	35	-0.00	0.00
82	->	36	0.00	0.00
82	->	37	-0.00	0.00
82	->	38	-0.00	0.00
82	->	39	0.00	0.00
82	->	40	0.00	0.00
82	->	41	0.00	0.00
82	->	42	0.00	0.00
82	->	43	0.00	0.00
82	->	44	0.00	0.00
82	->	45	0.00	0.00
82	->	46	-0.00	0.00
82	->	47	-0.00	0.00
82	->	48	-0.00	0.00
82	->	49	0.00	0.00
82	->	50	-0.00	0.00
82	->	51	0.00	0.00
82	->	52	-0.00	0.00
82	->	53	0.00	0.00
82	->	54	-0.00	0.00
82	->	55	0.00	0.00
82	->	56	0.00	0.00
82	->	57	0.00	0.00
82	->	58	-0.00	0.00
82	->	59	0.00	0.00
82	->	60	-0.00	0.00
82	->	61	-0.00	0.00
82	->	62	-0.00	0.00
82	->	63	-0.06	0.02
82	->	64	-0.01	0.00
82	->	65	-0.04	0.01
82	->	66	-0.05	0.02
82	->	67	-0.00	0.00
82	->	68	-0.04	0.04
82	->	69	-0.00	0.00
82	->	70	-0.03	0.01
82	->	71	-0.00	0.00
82	->	72	-0.00	0.00
82	->	73	-0.00	0.00
82	->	74	-0.01	0.00
82	->	75	-0.04	0.02
82	->	76	-0.04	0.02
82	->	77	-0.05	0.03
82	->	78	-1.60	0.82
82	->	79	-3.51	0.86

82	->	80	-0.88	0.18
82	->	81	-11.52	0.49
82	->	82	17.35	1.49
82	->	83	-22.07	0.57
82	->	84	-0.89	0.37
82	->	85	-1.41	1.37
82	->	86	-0.05	0.07
82	->	87	-0.02	0.01
82	->	88	-0.00	0.00
82	->	89	-0.00	0.00
82	->	90	-0.00	0.00
82	->	91	-0.00	0.00
82	->	92	-0.00	0.00
82	->	93	-0.00	0.00
82	->	94	-0.00	0.00
82	->	95	-0.00	0.00
82	->	96	-0.00	0.00
82	->	97	-0.00	0.00
82	->	98	-0.00	0.00
82	->	99	-0.00	0.00
82	->	100	-0.00	0.00
82	->	101	-0.00	0.00
82	->	102	-0.00	0.00
82	->	103	-0.00	0.00
82	->	104	-0.01	0.00
82	->	105	-0.01	0.00
82	->	106	-0.01	0.00
82	->	107	-0.00	0.00
83	->	1	0.00	0.00
83	->	2	0.00	0.00
83	->	3	0.00	0.00
83	->	4	0.00	0.00
83	->	5	0.00	0.00
83	->	6	0.00	0.00
83	->	7	0.00	0.00
83	->	8	-0.00	0.00
83	->	9	0.00	0.00
83	->	10	-0.00	0.00
83	->	11	-0.00	0.00
83	->	12	0.00	0.00
83	->	13	0.00	0.00
83	->	14	0.00	0.00
83	->	15	0.00	0.00
83	->	16	0.00	0.00
83	->	17	0.00	0.00
83	->	18	-0.00	0.00
83	->	19	-0.00	0.00
83	->	20	0.00	0.00
83	->	21	0.00	0.00
83	->	22	0.00	0.00

83	->	23	0.00	0.00
83	->	24	0.00	0.00
83	->	25	0.00	0.00
83	->	26	0.00	0.00
83	->	27	0.00	0.00
83	->	28	0.00	0.00
83	->	29	0.00	0.00
83	->	30	0.00	0.00
83	->	31	0.00	0.00
83	->	32	0.00	0.00
83	->	33	0.00	0.00
83	->	34	0.00	0.00
83	->	35	0.00	0.00
83	->	36	-0.00	0.00
83	->	37	0.00	0.00
83	->	38	0.00	0.00
83	->	39	0.00	0.00
83	->	40	0.00	0.00
83	->	41	0.00	0.00
83	->	42	0.00	0.00
83	->	43	0.00	0.00
83	->	44	0.00	0.00
83	->	45	0.00	0.00
83	->	46	0.00	0.00
83	->	47	0.00	0.00
83	->	48	0.00	0.00
83	->	49	0.00	0.00
83	->	50	0.00	0.00
83	->	51	0.00	0.00
83	->	52	0.00	0.00
83	->	53	-0.00	0.00
83	->	54	0.00	0.00
83	->	55	-0.00	0.00
83	->	56	0.00	0.00
83	->	57	0.00	0.00
83	->	58	0.00	0.00
83	->	59	-0.00	0.00
83	->	60	-0.00	0.00
83	->	61	-0.01	0.00
83	->	62	-0.04	0.01
83	->	63	2.28	0.30
83	->	64	-0.20	0.12
83	->	65	0.85	0.60
83	->	66	2.05	0.32
83	->	67	-0.01	0.01
83	->	68	-0.25	0.06
83	->	69	-0.01	0.01
83	->	70	-0.01	0.01
83	->	71	-0.00	0.00
83	->	72	-0.00	0.00

83	->	73	-0.00	0.00
83	->	74	-0.01	0.00
83	->	75	-0.02	0.01
83	->	76	-0.07	0.02
83	->	77	-0.03	0.02
83	->	78	-0.12	0.04
83	->	79	17.45	8.64
83	->	80	-2.81	0.57
83	->	81	-0.65	0.20
83	->	82	-22.17	0.57
83	->	83	4.89	1.83
83	->	84	-8.25	0.56
83	->	85	-0.83	0.73
83	->	86	-0.01	0.00
83	->	87	-0.00	0.00
83	->	88	-0.00	0.00
83	->	89	-0.00	0.00
83	->	90	-0.00	0.00
83	->	91	-0.00	0.00
83	->	92	-0.00	0.00
83	->	93	-0.00	0.00
83	->	94	-0.00	0.00
83	->	95	-0.00	0.00
83	->	96	-0.00	0.00
83	->	97	0.00	0.00
83	->	98	-0.00	0.00
83	->	99	0.00	0.00
83	->	100	0.00	0.00
83	->	101	-0.00	0.00
83	->	102	-0.00	0.00
83	->	103	-0.00	0.00
83	->	104	-0.01	0.00
83	->	105	-0.00	0.00
83	->	106	-0.00	0.00
83	->	107	-0.00	0.00
84	->	1	0.00	0.00
84	->	2	0.00	0.00
84	->	3	-0.00	0.00
84	->	4	0.00	0.00
84	->	5	-0.00	0.00
84	->	6	0.00	0.00
84	->	7	0.00	0.00
84	->	8	0.00	0.00
84	->	9	0.00	0.00
84	->	10	-0.00	0.00
84	->	11	0.00	0.00
84	->	12	0.00	0.00
84	->	13	0.00	0.00
84	->	14	0.00	0.00
84	->	15	0.00	0.00

84	->	16	0.00	0.00
84	->	17	0.00	0.00
84	->	18	-0.00	0.00
84	->	19	-0.00	0.00
84	->	20	0.00	0.00
84	->	21	0.00	0.00
84	->	22	0.00	0.00
84	->	23	0.00	0.00
84	->	24	0.00	0.00
84	->	25	0.00	0.00
84	->	26	0.00	0.00
84	->	27	0.00	0.00
84	->	28	0.00	0.00
84	->	29	0.00	0.00
84	->	30	0.00	0.00
84	->	31	0.00	0.00
84	->	32	0.00	0.00
84	->	33	0.00	0.00
84	->	34	0.00	0.00
84	->	35	-0.00	0.00
84	->	36	0.00	0.00
84	->	37	0.00	0.00
84	->	38	-0.00	0.00
84	->	39	0.00	0.00
84	->	40	0.00	0.00
84	->	41	0.00	0.00
84	->	42	0.00	0.00
84	->	43	0.00	0.00
84	->	44	0.00	0.00
84	->	45	0.00	0.00
84	->	46	-0.00	0.00
84	->	47	0.00	0.00
84	->	48	0.00	0.00
84	->	49	0.00	0.00
84	->	50	0.00	0.00
84	->	51	0.00	0.00
84	->	52	0.00	0.00
84	->	53	0.00	0.00
84	->	54	0.00	0.00
84	->	55	0.00	0.00
84	->	56	0.00	0.00
84	->	57	0.00	0.00
84	->	58	-0.00	0.00
84	->	59	0.00	0.00
84	->	60	0.00	0.00
84	->	61	-0.00	0.00
84	->	62	-0.00	0.00
84	->	63	-0.00	0.01
84	->	64	-0.03	0.04
84	->	65	-0.07	0.11

84	->	66	-0.00	0.00
84	->	67	-0.00	0.00
84	->	68	-0.00	0.00
84	->	69	-0.00	0.00
84	->	70	-0.00	0.00
84	->	71	-0.00	0.00
84	->	72	-0.00	0.00
84	->	73	-0.00	0.00
84	->	74	-0.00	0.00
84	->	75	-0.00	0.00
84	->	76	-0.01	0.01
84	->	77	-0.03	0.02
84	->	78	-0.02	0.01
84	->	79	-0.10	0.05
84	->	80	-1.99	1.15
84	->	81	-1.52	0.71
84	->	82	-0.91	0.36
84	->	83	-8.39	0.55
84	->	84	16.87	1.80
84	->	85	-8.45	1.36
84	->	86	-0.00	0.00
84	->	87	-0.00	0.00
84	->	88	-0.00	0.00
84	->	89	-0.00	0.00
84	->	90	-0.00	0.00
84	->	91	-0.00	0.00
84	->	92	-0.00	0.00
84	->	93	-0.00	0.00
84	->	94	-0.00	0.00
84	->	95	-0.00	0.00
84	->	96	-0.00	0.00
84	->	97	0.00	0.00
84	->	98	-0.00	0.00
84	->	99	-0.00	0.00
84	->	100	-0.00	0.00
84	->	101	-0.00	0.00
84	->	102	-0.00	0.00
84	->	103	-0.01	0.00
84	->	104	-0.01	0.00
84	->	105	-0.00	0.00
84	->	106	-0.00	0.00
84	->	107	-0.00	0.00
85	->	1	0.00	0.00
85	->	2	0.00	0.00
85	->	3	-0.00	0.00
85	->	4	0.00	0.00
85	->	5	-0.00	0.00
85	->	6	0.00	0.00
85	->	7	-0.00	0.00
85	->	8	-0.00	0.00

85	->	9	-0.00	0.00
85	->	10	-0.00	0.00
85	->	11	-0.00	0.00
85	->	12	0.00	0.00
85	->	13	0.00	0.00
85	->	14	0.00	0.00
85	->	15	0.00	0.00
85	->	16	0.00	0.00
85	->	17	-0.00	0.00
85	->	18	-0.00	0.00
85	->	19	-0.00	0.00
85	->	20	-0.00	0.00
85	->	21	0.00	0.00
85	->	22	0.00	0.00
85	->	23	0.00	0.00
85	->	24	0.00	0.00
85	->	25	0.00	0.00
85	->	26	0.00	0.00
85	->	27	-0.00	0.00
85	->	28	0.00	0.00
85	->	29	0.00	0.00
85	->	30	0.00	0.00
85	->	31	0.00	0.00
85	->	32	0.00	0.00
85	->	33	-0.00	0.00
85	->	34	0.00	0.00
85	->	35	-0.00	0.00
85	->	36	0.00	0.00
85	->	37	-0.00	0.00
85	->	38	-0.00	0.00
85	->	39	0.00	0.00
85	->	40	0.00	0.00
85	->	41	0.00	0.00
85	->	42	0.00	0.00
85	->	43	0.00	0.00
85	->	44	0.00	0.00
85	->	45	-0.00	0.00
85	->	46	-0.00	0.00
85	->	47	-0.00	0.00
85	->	48	-0.00	0.00
85	->	49	0.00	0.00
85	->	50	0.00	0.00
85	->	51	0.00	0.00
85	->	52	0.00	0.00
85	->	53	0.00	0.00
85	->	54	0.00	0.00
85	->	55	0.00	0.00
85	->	56	-0.00	0.00
85	->	57	-0.00	0.00
85	->	58	-0.00	0.00

85	->	59	0.00	0.00
85	->	60	0.00	0.00
85	->	61	-0.00	0.00
85	->	62	-0.00	0.00
85	->	63	-0.00	0.04
85	->	64	-0.32	1.52
85	->	65	-0.12	0.50
85	->	66	-0.00	0.03
85	->	67	-0.00	0.00
85	->	68	-0.00	0.01
85	->	69	-0.00	0.00
85	->	70	-0.00	0.00
85	->	71	0.00	0.00
85	->	72	0.00	0.01
85	->	73	-0.00	0.00
85	->	74	-0.00	0.01
85	->	75	-0.00	0.01
85	->	76	-0.00	0.01
85	->	77	-0.01	0.02
85	->	78	-0.02	0.01
85	->	79	-0.03	0.02
85	->	80	-0.06	0.06
85	->	81	-0.97	1.16
85	->	82	-1.42	1.38
85	->	83	-0.84	0.73
85	->	84	-8.52	1.36
85	->	85	-267.36	2.80
85	->	86	-0.00	0.01
85	->	87	-0.00	0.01
85	->	88	-0.00	0.00
85	->	89	-0.00	0.00
85	->	90	-0.00	0.00
85	->	91	-0.00	0.00
85	->	92	-0.00	0.00
85	->	93	-0.00	0.00
85	->	94	-0.00	0.00
85	->	95	-0.00	0.00
85	->	96	-0.00	0.00
85	->	97	-0.00	0.00
85	->	98	-0.00	0.00
85	->	99	-0.00	0.00
85	->	100	-0.00	0.00
85	->	101	-0.00	0.00
85	->	102	-0.00	0.01
85	->	103	-0.00	0.02
85	->	104	-0.01	0.03
85	->	105	-0.02	0.04
85	->	106	-0.01	0.02
85	->	107	-0.00	0.01
86	->	1	-0.00	0.00

86 ->	2	-0.00	0.00
86 ->	3	0.00	0.00
86 ->	4	-0.00	0.00
86 ->	5	0.00	0.00
86 ->	6	-0.00	0.00
86 ->	7	0.00	0.00
86 ->	8	0.00	0.00
86 ->	9	0.00	0.00
86 ->	10	0.00	0.00
86 ->	11	0.00	0.00
86 ->	12	-0.00	0.00
86 ->	13	-0.00	0.00
86 ->	14	-0.00	0.00
86 ->	15	-0.00	0.00
86 ->	16	-0.00	0.00
86 ->	17	0.00	0.00
86 ->	18	0.00	0.00
86 ->	19	0.00	0.00
86 ->	20	0.00	0.00
86 ->	21	0.00	0.00
86 ->	22	-0.00	0.00
86 ->	23	-0.00	0.00
86 ->	24	0.00	0.00
86 ->	25	-0.00	0.00
86 ->	26	-0.00	0.00
86 ->	27	-0.00	0.00
86 ->	28	-0.00	0.00
86 ->	29	0.00	0.00
86 ->	30	0.00	0.00
86 ->	31	-0.00	0.00
86 ->	32	0.00	0.00
86 ->	33	0.00	0.00
86 ->	34	-0.00	0.00
86 ->	35	0.00	0.00
86 ->	36	-0.00	0.00
86 ->	37	-0.00	0.00
86 ->	38	0.00	0.00
86 ->	39	-0.00	0.00
86 ->	40	-0.01	0.00
86 ->	41	-0.00	0.00
86 ->	42	-0.00	0.00
86 ->	43	-0.00	0.00
86 ->	44	-0.00	0.00
86 ->	45	0.00	0.00
86 ->	46	0.00	0.00
86 ->	47	-0.00	0.00
86 ->	48	-0.00	0.00
86 ->	49	-0.00	0.00
86 ->	50	-0.00	0.00
86 ->	51	-0.00	0.00

86	->	52	-0.00	0.00
86	->	53	-0.01	0.00
86	->	54	-0.01	0.00
86	->	55	-0.00	0.00
86	->	56	-0.00	0.00
86	->	57	-0.00	0.00
86	->	58	0.00	0.00
86	->	59	-0.09	0.22
86	->	60	-0.00	0.00
86	->	61	-0.00	0.00
86	->	62	-0.00	0.00
86	->	63	0.01	0.00
86	->	64	0.00	0.00
86	->	65	-0.00	0.00
86	->	66	0.01	0.01
86	->	67	-0.01	0.02
86	->	68	-0.88	1.50
86	->	69	-0.03	0.04
86	->	70	-0.03	0.04
86	->	71	-0.00	0.00
86	->	72	-0.04	0.01
86	->	73	-0.00	0.00
86	->	74	-0.01	0.02
86	->	75	-0.07	0.10
86	->	76	-0.02	0.01
86	->	77	-0.02	0.01
86	->	78	-1.40	1.50
86	->	79	-0.04	0.06
86	->	80	-0.00	0.00
86	->	81	-0.04	0.05
86	->	82	-0.05	0.06
86	->	83	-0.01	0.00
86	->	84	-0.00	0.00
86	->	85	-0.00	0.01
86	->	86	-87.35	2.83
86	->	87	-35.53	1.02
86	->	88	-0.29	0.05
86	->	89	-0.03	0.01
86	->	90	-0.00	0.00
86	->	91	0.00	0.00
86	->	92	0.00	0.00
86	->	93	0.00	0.00
86	->	94	0.00	0.00
86	->	95	0.00	0.00
86	->	96	0.00	0.00
86	->	97	0.00	0.00
86	->	98	0.00	0.00
86	->	99	0.00	0.00
86	->	100	0.00	0.00
86	->	101	0.00	0.00

86	->	102	0.00	0.00
86	->	103	0.00	0.00
86	->	104	-0.00	0.00
86	->	105	-0.02	0.01
86	->	106	-0.18	0.09
86	->	107	-2.06	1.14
87	->	1	-0.01	0.00
87	->	2	-0.00	0.00
87	->	3	0.00	0.00
87	->	4	-0.00	0.00
87	->	5	0.00	0.00
87	->	6	-0.00	0.00
87	->	7	0.00	0.00
87	->	8	0.00	0.00
87	->	9	0.00	0.00
87	->	10	0.00	0.00
87	->	11	0.00	0.00
87	->	12	-0.01	0.00
87	->	13	-0.00	0.00
87	->	14	-0.00	0.00
87	->	15	-0.00	0.00
87	->	16	-0.00	0.00
87	->	17	0.00	0.00
87	->	18	0.01	0.00
87	->	19	0.01	0.00
87	->	20	0.00	0.00
87	->	21	0.00	0.00
87	->	22	-0.01	0.00
87	->	23	-0.00	0.00
87	->	24	0.00	0.00
87	->	25	-0.01	0.00
87	->	26	-0.00	0.00
87	->	27	-0.00	0.00
87	->	28	-0.00	0.00
87	->	29	-0.00	0.00
87	->	30	0.00	0.00
87	->	31	-0.01	0.00
87	->	32	0.00	0.00
87	->	33	-0.00	0.00
87	->	34	-0.00	0.00
87	->	35	0.02	0.01
87	->	36	-0.01	0.00
87	->	37	-0.00	0.00
87	->	38	0.04	0.02
87	->	39	-0.00	0.00
87	->	40	-0.06	0.05
87	->	41	-0.00	0.00
87	->	42	-0.01	0.00
87	->	43	-0.00	0.00
87	->	44	-0.01	0.00

87	->	45	0.00	0.00
87	->	46	0.02	0.00
87	->	47	-0.00	0.00
87	->	48	-0.00	0.00
87	->	49	-0.00	0.00
87	->	50	-0.01	0.01
87	->	51	-0.03	0.01
87	->	52	0.00	0.01
87	->	53	-0.09	0.06
87	->	54	-0.15	0.14
87	->	55	0.00	0.03
87	->	56	-0.01	0.00
87	->	57	-0.01	0.01
87	->	58	0.02	0.01
87	->	59	-0.75	1.61
87	->	60	-0.01	0.00
87	->	61	-0.00	0.00
87	->	62	-0.00	0.00
87	->	63	0.04	0.01
87	->	64	0.01	0.00
87	->	65	0.00	0.00
87	->	66	0.04	0.01
87	->	67	-0.00	0.01
87	->	68	-1.64	1.94
87	->	69	-0.09	0.10
87	->	70	-0.11	0.16
87	->	71	-0.04	0.04
87	->	72	-0.24	0.16
87	->	73	-0.01	0.01
87	->	74	0.02	0.09
87	->	75	-0.29	0.55
87	->	76	-0.04	0.01
87	->	77	-0.05	0.01
87	->	78	-4.66	2.70
87	->	79	-0.04	0.02
87	->	80	-0.00	0.00
87	->	81	-0.05	0.01
87	->	82	-0.02	0.01
87	->	83	-0.00	0.00
87	->	84	-0.00	0.00
87	->	85	-0.00	0.01
87	->	86	-35.34	1.02
87	->	87	-177.28	3.62
87	->	88	-36.13	0.86
87	->	89	-0.34	0.09
87	->	90	-0.02	0.01
87	->	91	0.01	0.00
87	->	92	0.01	0.00
87	->	93	0.01	0.00
87	->	94	0.01	0.00

87	->	95	0.00	0.00
87	->	96	0.00	0.00
87	->	97	0.00	0.00
87	->	98	0.00	0.00
87	->	99	0.01	0.00
87	->	100	0.01	0.00
87	->	101	0.01	0.00
87	->	102	0.01	0.00
87	->	103	0.01	0.00
87	->	104	-0.01	0.01
87	->	105	-0.12	0.05
87	->	106	-0.95	0.63
87	->	107	-10.60	0.79
88	->	1	-0.01	0.00
88	->	2	-0.00	0.00
88	->	3	0.00	0.00
88	->	4	0.00	0.00
88	->	5	0.00	0.00
88	->	6	-0.00	0.00
88	->	7	0.00	0.00
88	->	8	0.00	0.00
88	->	9	-0.00	0.00
88	->	10	0.01	0.00
88	->	11	0.00	0.00
88	->	12	-0.01	0.00
88	->	13	-0.00	0.00
88	->	14	-0.00	0.00
88	->	15	-0.00	0.00
88	->	16	-0.01	0.00
88	->	17	0.00	0.00
88	->	18	0.01	0.00
88	->	19	0.01	0.00
88	->	20	0.00	0.00
88	->	21	0.00	0.00
88	->	22	-0.02	0.00
88	->	23	-0.00	0.00
88	->	24	0.00	0.00
88	->	25	-0.01	0.00
88	->	26	-0.00	0.00
88	->	27	-0.00	0.00
88	->	28	0.00	0.00
88	->	29	-0.00	0.00
88	->	30	0.00	0.00
88	->	31	-0.04	0.02
88	->	32	-0.00	0.00
88	->	33	-0.00	0.00
88	->	34	-0.01	0.01
88	->	35	0.09	0.03
88	->	36	-0.03	0.01
88	->	37	-0.00	0.01

88	->	38	0.20	0.07
88	->	39	-0.01	0.00
88	->	40	-0.81	0.67
88	->	41	-0.01	0.01
88	->	42	-0.02	0.04
88	->	43	-0.00	0.01
88	->	44	-0.03	0.01
88	->	45	0.00	0.00
88	->	46	0.02	0.01
88	->	47	-0.05	0.02
88	->	48	0.01	0.01
88	->	49	-0.02	0.02
88	->	50	-0.11	0.08
88	->	51	-0.73	0.52
88	->	52	0.01	0.02
88	->	53	-0.30	0.27
88	->	54	-1.93	1.49
88	->	55	-0.03	0.37
88	->	56	-0.02	0.01
88	->	57	-0.04	0.04
88	->	58	0.03	0.02
88	->	59	-0.78	1.51
88	->	60	-0.01	0.00
88	->	61	-0.01	0.00
88	->	62	-0.00	0.00
88	->	63	0.01	0.00
88	->	64	0.01	0.00
88	->	65	0.00	0.00
88	->	66	0.01	0.00
88	->	67	0.00	0.00
88	->	68	-0.06	0.02
88	->	69	-0.04	0.02
88	->	70	-0.07	0.09
88	->	71	-0.13	0.19
88	->	72	-2.35	0.86
88	->	73	-0.00	0.01
88	->	74	-1.81	2.10
88	->	75	-0.56	1.44
88	->	76	-0.02	0.01
88	->	77	-0.01	0.01
88	->	78	-0.14	0.72
88	->	79	-0.01	0.00
88	->	80	-0.00	0.00
88	->	81	-0.02	0.00
88	->	82	-0.00	0.00
88	->	83	-0.00	0.00
88	->	84	-0.00	0.00
88	->	85	-0.00	0.00
88	->	86	-0.29	0.05
88	->	87	-35.94	0.85

88	->	88	-209.72	5.13
88	->	89	-32.78	1.31
88	->	90	-0.24	0.06
88	->	91	0.00	0.01
88	->	92	0.01	0.00
88	->	93	0.01	0.00
88	->	94	0.00	0.00
88	->	95	0.00	0.00
88	->	96	0.00	0.00
88	->	97	0.00	0.00
88	->	98	0.01	0.00
88	->	99	0.01	0.00
88	->	100	0.01	0.00
88	->	101	0.01	0.00
88	->	102	0.00	0.00
88	->	103	-0.01	0.01
88	->	104	-0.10	0.05
88	->	105	-0.51	0.32
88	->	106	-11.44	0.86
88	->	107	0.17	0.20
89	->	1	-0.01	0.00
89	->	2	-0.00	0.00
89	->	3	0.00	0.00
89	->	4	-0.00	0.00
89	->	5	0.01	0.00
89	->	6	-0.00	0.00
89	->	7	-0.00	0.00
89	->	8	0.00	0.00
89	->	9	-0.00	0.00
89	->	10	0.01	0.00
89	->	11	0.00	0.00
89	->	12	-0.01	0.00
89	->	13	-0.00	0.00
89	->	14	-0.00	0.00
89	->	15	-0.00	0.00
89	->	16	-0.01	0.00
89	->	17	0.00	0.00
89	->	18	0.01	0.00
89	->	19	0.01	0.00
89	->	20	-0.00	0.00
89	->	21	-0.00	0.00
89	->	22	-0.03	0.01
89	->	23	-0.00	0.00
89	->	24	-0.00	0.00
89	->	25	-0.03	0.00
89	->	26	-0.01	0.00
89	->	27	-0.00	0.00
89	->	28	-0.00	0.00
89	->	29	-0.00	0.00
89	->	30	-0.00	0.00

89	->	31	-0.46	0.43
89	->	32	-0.00	0.00
89	->	33	-0.01	0.00
89	->	34	-0.03	0.01
89	->	35	0.15	0.06
89	->	36	-0.04	0.01
89	->	37	0.01	0.01
89	->	38	0.47	0.21
89	->	39	-0.03	0.02
89	->	40	-6.12	3.14
89	->	41	-0.10	0.05
89	->	42	-0.89	0.71
89	->	43	0.01	0.04
89	->	44	-0.05	0.02
89	->	45	-0.00	0.00
89	->	46	0.03	0.01
89	->	47	-0.22	0.12
89	->	48	0.01	0.03
89	->	49	-0.03	0.02
89	->	50	-0.30	0.26
89	->	51	-4.38	3.02
89	->	52	0.06	0.05
89	->	53	-0.13	0.11
89	->	54	-0.52	1.17
89	->	55	0.06	0.06
89	->	56	-0.01	0.00
89	->	57	-0.01	0.00
89	->	58	0.03	0.01
89	->	59	-0.05	0.02
89	->	60	-0.00	0.00
89	->	61	-0.00	0.00
89	->	62	0.00	0.00
89	->	63	0.01	0.00
89	->	64	0.01	0.00
89	->	65	0.00	0.00
89	->	66	0.01	0.00
89	->	67	0.00	0.00
89	->	68	-0.03	0.01
89	->	69	-0.02	0.00
89	->	70	-0.01	0.01
89	->	71	-0.02	0.01
89	->	72	-8.32	1.35
89	->	73	-0.02	0.01
89	->	74	0.37	0.34
89	->	75	0.05	0.07
89	->	76	-0.02	0.00
89	->	77	-0.03	0.00
89	->	78	-0.14	0.12
89	->	79	-0.01	0.00
89	->	80	-0.00	0.00

89	->	81	-0.02	0.00
89	->	82	-0.00	0.00
89	->	83	-0.00	0.00
89	->	84	-0.00	0.00
89	->	85	-0.00	0.00
89	->	86	-0.03	0.01
89	->	87	-0.34	0.09
89	->	88	-32.59	1.29
89	->	89	-165.55	4.32
89	->	90	-34.85	1.12
89	->	91	-0.30	0.09
89	->	92	-0.02	0.02
89	->	93	0.01	0.00
89	->	94	0.01	0.00
89	->	95	0.00	0.00
89	->	96	0.00	0.00
89	->	97	0.00	0.00
89	->	98	0.01	0.00
89	->	99	0.02	0.00
89	->	100	0.02	0.00
89	->	101	0.01	0.00
89	->	102	-0.00	0.00
89	->	103	-0.07	0.03
89	->	104	-1.77	0.94
89	->	105	-11.66	0.62
89	->	106	-4.51	1.06
89	->	107	-0.26	0.07
90	->	1	-0.03	0.00
90	->	2	-0.00	0.00
90	->	3	-0.00	0.00
90	->	4	-0.00	0.00
90	->	5	0.02	0.00
90	->	6	-0.00	0.00
90	->	7	-0.00	0.00
90	->	8	0.01	0.00
90	->	9	-0.00	0.00
90	->	10	0.03	0.01
90	->	11	0.01	0.00
90	->	12	-0.05	0.02
90	->	13	-0.01	0.00
90	->	14	-0.00	0.00
90	->	15	-0.00	0.00
90	->	16	-0.02	0.00
90	->	17	0.00	0.00
90	->	18	0.02	0.00
90	->	19	0.02	0.00
90	->	20	-0.00	0.00
90	->	21	-0.00	0.00
90	->	22	-0.14	0.08
90	->	23	-0.02	0.02

90	->	24	-0.00	0.00
90	->	25	-0.12	0.03
90	->	26	-0.21	0.14
90	->	27	0.01	0.01
90	->	28	-0.01	0.00
90	->	29	-0.02	0.02
90	->	30	-0.01	0.01
90	->	31	-3.50	3.53
90	->	32	-0.01	0.00
90	->	33	-0.01	0.01
90	->	34	-0.03	0.02
90	->	35	0.04	0.01
90	->	36	-0.02	0.00
90	->	37	0.00	0.00
90	->	38	0.06	0.03
90	->	39	-0.02	0.02
90	->	40	-3.03	3.33
90	->	41	-0.30	0.35
90	->	42	-1.05	0.47
90	->	43	-0.41	0.44
90	->	44	-0.15	0.12
90	->	45	-0.00	0.01
90	->	46	-0.04	0.06
90	->	47	-1.87	1.46
90	->	48	-0.03	0.04
90	->	49	-0.02	0.01
90	->	50	-0.33	0.32
90	->	51	-0.08	0.05
90	->	52	-0.01	0.00
90	->	53	-0.04	0.02
90	->	54	-0.02	0.00
90	->	55	-0.00	0.00
90	->	56	-0.00	0.00
90	->	57	-0.00	0.00
90	->	58	0.01	0.00
90	->	59	-0.02	0.00
90	->	60	-0.00	0.00
90	->	61	-0.00	0.00
90	->	62	0.00	0.00
90	->	63	0.01	0.00
90	->	64	0.00	0.00
90	->	65	0.00	0.00
90	->	66	0.01	0.00
90	->	67	0.00	0.00
90	->	68	-0.01	0.00
90	->	69	-0.01	0.00
90	->	70	-0.00	0.00
90	->	71	-0.01	0.01
90	->	72	-1.92	1.17
90	->	73	-0.01	0.00

90 ->	74	0.08	0.12
90 ->	75	0.02	0.03
90 ->	76	-0.02	0.00
90 ->	77	-0.02	0.00
90 ->	78	-0.04	0.01
90 ->	79	-0.00	0.00
90 ->	80	-0.00	0.00
90 ->	81	-0.02	0.00
90 ->	82	-0.00	0.00
90 ->	83	-0.00	0.00
90 ->	84	-0.00	0.00
90 ->	85	-0.00	0.00
90 ->	86	-0.00	0.00
90 ->	87	-0.02	0.01
90 ->	88	-0.24	0.06
90 ->	89	-34.65	1.10
90 ->	90	-87.83	4.08
90 ->	91	-33.32	1.04
90 ->	92	-0.12	0.06
90 ->	93	0.01	0.00
90 ->	94	0.01	0.00
90 ->	95	0.01	0.00
90 ->	96	0.00	0.00
90 ->	97	0.00	0.00
90 ->	98	0.02	0.00
90 ->	99	0.01	0.00
90 ->	100	0.01	0.00
90 ->	101	-0.00	0.01
90 ->	102	-0.03	0.02
90 ->	103	-0.39	0.22
90 ->	104	-5.78	0.76
90 ->	105	-0.71	0.64
90 ->	106	-0.23	0.12
90 ->	107	-0.03	0.02
91 ->	1	-0.09	0.03
91 ->	2	-0.00	0.00
91 ->	3	-0.00	0.00
91 ->	4	-0.01	0.00
91 ->	5	0.06	0.03
91 ->	6	-0.00	0.00
91 ->	7	-0.01	0.01
91 ->	8	0.01	0.00
91 ->	9	-0.01	0.00
91 ->	10	0.09	0.05
91 ->	11	0.02	0.01
91 ->	12	-1.01	0.87
91 ->	13	-0.04	0.02
91 ->	14	-0.00	0.03
91 ->	15	-0.00	0.01
91 ->	16	-0.04	0.02

91	->	17	0.00	0.01
91	->	18	0.05	0.05
91	->	19	0.11	0.14
91	->	20	0.02	0.03
91	->	21	-0.04	0.03
91	->	22	-3.41	2.78
91	->	23	-0.66	0.58
91	->	24	0.01	0.03
91	->	25	-0.35	0.13
91	->	26	-3.04	1.12
91	->	27	0.03	0.04
91	->	28	-0.02	0.01
91	->	29	-0.05	0.04
91	->	30	-0.01	0.01
91	->	31	-0.52	0.46
91	->	32	-0.01	0.01
91	->	33	-0.01	0.01
91	->	34	-0.01	0.00
91	->	35	0.03	0.00
91	->	36	-0.02	0.00
91	->	37	0.00	0.00
91	->	38	0.03	0.01
91	->	39	-0.00	0.00
91	->	40	-0.07	0.03
91	->	41	-0.16	0.24
91	->	42	-0.27	0.19
91	->	43	-2.58	1.97
91	->	44	-1.28	1.50
91	->	45	0.00	0.01
91	->	46	0.09	0.15
91	->	47	-2.33	0.82
91	->	48	0.00	0.01
91	->	49	-0.01	0.00
91	->	50	-0.03	0.02
91	->	51	-0.02	0.00
91	->	52	-0.01	0.00
91	->	53	-0.03	0.01
91	->	54	-0.01	0.00
91	->	55	-0.00	0.00
91	->	56	-0.00	0.00
91	->	57	-0.00	0.00
91	->	58	0.01	0.00
91	->	59	-0.01	0.00
91	->	60	-0.00	0.00
91	->	61	-0.00	0.00
91	->	62	0.00	0.00
91	->	63	0.01	0.00
91	->	64	0.00	0.00
91	->	65	0.00	0.00
91	->	66	0.00	0.00

91	->	67	0.00	0.00
91	->	68	-0.01	0.00
91	->	69	-0.01	0.00
91	->	70	-0.00	0.00
91	->	71	0.00	0.00
91	->	72	-0.16	0.22
91	->	73	0.00	0.02
91	->	74	-0.05	0.07
91	->	75	0.01	0.01
91	->	76	-0.01	0.00
91	->	77	-0.01	0.01
91	->	78	-0.03	0.01
91	->	79	-0.00	0.00
91	->	80	-0.00	0.00
91	->	81	-0.01	0.00
91	->	82	-0.00	0.00
91	->	83	-0.00	0.00
91	->	84	-0.00	0.00
91	->	85	-0.00	0.00
91	->	86	0.00	0.00
91	->	87	0.01	0.00
91	->	88	0.00	0.01
91	->	89	-0.30	0.09
91	->	90	-33.10	1.01
91	->	91	-169.88	3.31
91	->	92	-33.08	0.94
91	->	93	-0.07	0.16
91	->	94	-0.03	0.01
91	->	95	0.01	0.00
91	->	96	0.00	0.00
91	->	97	0.00	0.00
91	->	98	0.03	0.01
91	->	99	0.01	0.01
91	->	100	0.01	0.01
91	->	101	-0.12	0.08
91	->	102	-0.84	0.47
91	->	103	-11.55	0.62
91	->	104	-4.18	0.78
91	->	105	-0.27	0.08
91	->	106	-0.03	0.03
91	->	107	0.01	0.01
92	->	1	-1.24	0.79
92	->	2	-0.00	0.00
92	->	3	-0.01	0.01
92	->	4	-0.02	0.01
92	->	5	0.11	0.06
92	->	6	-0.00	0.00
92	->	7	-0.01	0.01
92	->	8	0.02	0.00
92	->	9	-0.00	0.00

92	->	10	0.17	0.10
92	->	11	0.03	0.02
92	->	12	-8.36	4.91
92	->	13	-0.12	0.07
92	->	14	-0.73	0.83
92	->	15	0.01	0.02
92	->	16	-0.07	0.02
92	->	17	0.00	0.01
92	->	18	0.04	0.04
92	->	19	0.41	0.53
92	->	20	0.08	0.07
92	->	21	-0.10	0.07
92	->	22	-11.10	6.40
92	->	23	-3.86	3.75
92	->	24	0.06	0.06
92	->	25	-0.21	0.10
92	->	26	-0.68	0.37
92	->	27	0.06	0.09
92	->	28	-0.01	0.00
92	->	29	-0.01	0.00
92	->	30	-0.00	0.00
92	->	31	-0.05	0.01
92	->	32	-0.01	0.00
92	->	33	-0.00	0.00
92	->	34	-0.00	0.00
92	->	35	0.01	0.00
92	->	36	-0.01	0.00
92	->	37	0.00	0.00
92	->	38	0.01	0.00
92	->	39	-0.00	0.00
92	->	40	-0.02	0.01
92	->	41	-0.01	0.01
92	->	42	-0.04	0.03
92	->	43	-0.08	0.07
92	->	44	-4.70	3.90
92	->	45	-0.00	0.01
92	->	46	0.46	0.35
92	->	47	-1.46	1.71
92	->	48	0.00	0.00
92	->	49	-0.01	0.00
92	->	50	-0.01	0.01
92	->	51	-0.01	0.00
92	->	52	-0.00	0.00
92	->	53	-0.02	0.00
92	->	54	-0.00	0.00
92	->	55	-0.00	0.00
92	->	56	-0.00	0.00
92	->	57	-0.00	0.00
92	->	58	0.01	0.00
92	->	59	-0.01	0.00

92	->	60	-0.00	0.00
92	->	61	-0.01	0.00
92	->	62	0.00	0.00
92	->	63	0.00	0.00
92	->	64	0.00	0.00
92	->	65	0.00	0.00
92	->	66	0.00	0.00
92	->	67	0.00	0.00
92	->	68	-0.01	0.00
92	->	69	-0.00	0.00
92	->	70	-0.00	0.00
92	->	71	0.00	0.00
92	->	72	-0.02	0.02
92	->	73	-0.02	0.03
92	->	74	-0.03	0.03
92	->	75	0.00	0.00
92	->	76	-0.00	0.01
92	->	77	-0.01	0.02
92	->	78	-0.02	0.01
92	->	79	-0.00	0.00
92	->	80	-0.00	0.00
92	->	81	-0.01	0.00
92	->	82	-0.00	0.00
92	->	83	-0.00	0.00
92	->	84	-0.00	0.00
92	->	85	-0.00	0.00
92	->	86	0.00	0.00
92	->	87	0.01	0.00
92	->	88	0.01	0.00
92	->	89	-0.02	0.02
92	->	90	-0.12	0.06
92	->	91	-32.96	0.92
92	->	92	-159.31	5.75
92	->	93	-32.48	1.16
92	->	94	-0.44	0.09
92	->	95	-0.01	0.01
92	->	96	0.00	0.00
92	->	97	0.00	0.00
92	->	98	0.02	0.01
92	->	99	-0.01	0.01
92	->	100	-0.02	0.05
92	->	101	-1.82	0.78
92	->	102	-11.88	0.64
92	->	103	-4.07	1.00
92	->	104	-0.43	0.13
92	->	105	-0.04	0.03
92	->	106	0.01	0.01
92	->	107	0.01	0.00
93	->	1	-9.90	4.49
93	->	2	-0.02	0.01

93	->	3	-0.01	0.01
93	->	4	-0.01	0.01
93	->	5	0.05	0.01
93	->	6	-0.00	0.00
93	->	7	-0.00	0.00
93	->	8	0.02	0.00
93	->	9	-0.00	0.00
93	->	10	0.05	0.02
93	->	11	0.04	0.02
93	->	12	-7.85	3.51
93	->	13	-0.15	0.42
93	->	14	-0.55	0.47
93	->	15	-0.59	0.58
93	->	16	-0.23	0.08
93	->	17	-0.01	0.01
93	->	18	0.03	0.08
93	->	19	-2.05	2.03
93	->	20	0.01	0.03
93	->	21	-0.03	0.01
93	->	22	-5.50	2.78
93	->	23	-0.06	0.03
93	->	24	-0.00	0.00
93	->	25	-0.05	0.01
93	->	26	-0.02	0.01
93	->	27	-0.00	0.00
93	->	28	-0.00	0.00
93	->	29	-0.00	0.00
93	->	30	0.00	0.00
93	->	31	-0.02	0.01
93	->	32	-0.00	0.00
93	->	33	-0.00	0.00
93	->	34	-0.00	0.00
93	->	35	0.01	0.00
93	->	36	-0.00	0.00
93	->	37	0.00	0.00
93	->	38	0.01	0.00
93	->	39	0.00	0.00
93	->	40	-0.01	0.00
93	->	41	-0.00	0.00
93	->	42	-0.01	0.00
93	->	43	-0.02	0.01
93	->	44	-3.33	3.07
93	->	45	-0.00	0.00
93	->	46	0.25	0.29
93	->	47	-0.11	0.11
93	->	48	-0.00	0.00
93	->	49	-0.00	0.00
93	->	50	-0.01	0.00
93	->	51	-0.00	0.00
93	->	52	-0.00	0.00

93	->	53	-0.01	0.00
93	->	54	-0.00	0.00
93	->	55	-0.00	0.00
93	->	56	-0.00	0.00
93	->	57	-0.00	0.00
93	->	58	0.00	0.00
93	->	59	-0.01	0.00
93	->	60	-0.00	0.00
93	->	61	-0.01	0.01
93	->	62	0.00	0.00
93	->	63	0.00	0.00
93	->	64	0.00	0.00
93	->	65	0.00	0.00
93	->	66	0.00	0.00
93	->	67	-0.00	0.00
93	->	68	-0.00	0.00
93	->	69	-0.00	0.00
93	->	70	-0.00	0.00
93	->	71	0.00	0.00
93	->	72	-0.02	0.01
93	->	73	-0.02	0.02
93	->	74	0.01	0.01
93	->	75	0.01	0.00
93	->	76	-0.01	0.01
93	->	77	-0.03	0.01
93	->	78	-0.02	0.00
93	->	79	-0.00	0.00
93	->	80	-0.00	0.00
93	->	81	-0.02	0.01
93	->	82	-0.00	0.00
93	->	83	-0.00	0.00
93	->	84	-0.00	0.00
93	->	85	-0.00	0.00
93	->	86	0.00	0.00
93	->	87	0.01	0.00
93	->	88	0.01	0.00
93	->	89	0.01	0.00
93	->	90	0.01	0.00
93	->	91	-0.07	0.16
93	->	92	-32.34	1.14
93	->	93	-162.95	3.27
93	->	94	-36.36	0.94
93	->	95	-0.33	0.07
93	->	96	-0.02	0.01
93	->	97	-0.01	0.01
93	->	98	-0.01	0.01
93	->	99	-0.10	0.04
93	->	100	-0.39	0.86
93	->	101	-11.31	1.35
93	->	102	-2.73	0.87

93 ->	103	-0.36	0.13
93 ->	104	-0.05	0.05
93 ->	105	0.02	0.01
93 ->	106	0.02	0.00
93 ->	107	0.01	0.00
94 ->	1	-2.91	2.38
94 ->	2	-0.02	0.01
94 ->	3	-0.01	0.00
94 ->	4	-0.00	0.00
94 ->	5	0.02	0.00
94 ->	6	-0.00	0.00
94 ->	7	-0.00	0.00
94 ->	8	0.01	0.00
94 ->	9	0.00	0.00
94 ->	10	0.02	0.00
94 ->	11	0.02	0.00
94 ->	12	-0.08	0.04
94 ->	13	-0.07	0.02
94 ->	14	-0.20	0.09
94 ->	15	-2.52	1.85
94 ->	16	-2.85	0.93
94 ->	17	-0.01	0.01
94 ->	18	-2.31	2.23
94 ->	19	-1.16	1.64
94 ->	20	-0.02	0.01
94 ->	21	-0.00	0.01
94 ->	22	-0.18	0.40
94 ->	23	-0.01	0.00
94 ->	24	-0.00	0.00
94 ->	25	-0.02	0.00
94 ->	26	-0.00	0.00
94 ->	27	-0.00	0.00
94 ->	28	-0.00	0.00
94 ->	29	-0.00	0.00
94 ->	30	-0.00	0.00
94 ->	31	-0.01	0.00
94 ->	32	-0.00	0.00
94 ->	33	-0.00	0.00
94 ->	34	-0.00	0.00
94 ->	35	0.00	0.00
94 ->	36	-0.00	0.00
94 ->	37	0.00	0.00
94 ->	38	0.00	0.00
94 ->	39	-0.00	0.00
94 ->	40	-0.01	0.00
94 ->	41	-0.00	0.00
94 ->	42	-0.00	0.00
94 ->	43	-0.00	0.00
94 ->	44	0.07	0.61
94 ->	45	-0.01	0.00

94	->	46	-0.00	0.03
94	->	47	-0.02	0.01
94	->	48	-0.00	0.00
94	->	49	-0.00	0.00
94	->	50	-0.00	0.00
94	->	51	-0.00	0.00
94	->	52	-0.00	0.00
94	->	53	-0.02	0.00
94	->	54	-0.00	0.00
94	->	55	-0.00	0.00
94	->	56	-0.00	0.00
94	->	57	-0.00	0.00
94	->	58	0.01	0.00
94	->	59	-0.01	0.00
94	->	60	-0.00	0.00
94	->	61	-0.00	0.00
94	->	62	0.00	0.00
94	->	63	0.01	0.00
94	->	64	0.01	0.00
94	->	65	0.00	0.00
94	->	66	0.01	0.00
94	->	67	0.00	0.00
94	->	68	-0.01	0.00
94	->	69	-0.01	0.00
94	->	70	-0.00	0.00
94	->	71	0.00	0.00
94	->	72	-0.02	0.00
94	->	73	-0.01	0.00
94	->	74	0.02	0.00
94	->	75	0.01	0.00
94	->	76	-0.03	0.01
94	->	77	-0.03	0.01
94	->	78	-0.01	0.00
94	->	79	-0.00	0.00
94	->	80	-0.00	0.00
94	->	81	-0.01	0.01
94	->	82	-0.00	0.00
94	->	83	-0.00	0.00
94	->	84	-0.00	0.00
94	->	85	-0.00	0.00
94	->	86	0.00	0.00
94	->	87	0.01	0.00
94	->	88	0.00	0.00
94	->	89	0.01	0.00
94	->	90	0.01	0.00
94	->	91	-0.03	0.01
94	->	92	-0.44	0.09
94	->	93	-36.19	0.93
94	->	94	-209.30	3.89
94	->	95	-32.88	0.90

94	->	96	-0.28	0.11
94	->	97	-0.02	0.02
94	->	98	-0.15	0.08
94	->	99	-0.44	0.20
94	->	100	-11.47	0.87
94	->	101	0.29	0.25
94	->	102	0.04	0.06
94	->	103	0.00	0.02
94	->	104	0.01	0.00
94	->	105	0.02	0.00
94	->	106	0.01	0.00
94	->	107	0.00	0.00
95	->	1	-0.12	0.02
95	->	2	-0.01	0.00
95	->	3	-0.00	0.00
95	->	4	-0.00	0.00
95	->	5	0.01	0.00
95	->	6	-0.00	0.00
95	->	7	-0.00	0.00
95	->	8	0.01	0.00
95	->	9	0.00	0.00
95	->	10	0.01	0.00
95	->	11	0.01	0.00
95	->	12	-0.03	0.01
95	->	13	-0.02	0.00
95	->	14	-0.01	0.00
95	->	15	-0.04	0.02
95	->	16	-8.48	0.89
95	->	17	-0.01	0.00
95	->	18	0.45	0.24
95	->	19	0.07	0.04
95	->	20	-0.00	0.00
95	->	21	-0.01	0.00
95	->	22	-0.11	0.08
95	->	23	-0.01	0.00
95	->	24	-0.00	0.00
95	->	25	-0.02	0.00
95	->	26	-0.00	0.00
95	->	27	-0.00	0.00
95	->	28	-0.00	0.00
95	->	29	-0.00	0.00
95	->	30	0.00	0.00
95	->	31	-0.01	0.00
95	->	32	-0.00	0.00
95	->	33	-0.00	0.00
95	->	34	-0.00	0.00
95	->	35	0.00	0.00
95	->	36	-0.00	0.00
95	->	37	0.00	0.00
95	->	38	0.00	0.00

95	->	39	0.00	0.00
95	->	40	-0.00	0.00
95	->	41	-0.00	0.00
95	->	42	-0.00	0.00
95	->	43	-0.00	0.00
95	->	44	-0.11	0.12
95	->	45	-0.01	0.01
95	->	46	-0.01	0.02
95	->	47	-0.01	0.00
95	->	48	-0.00	0.00
95	->	49	-0.00	0.00
95	->	50	-0.00	0.00
95	->	51	-0.00	0.00
95	->	52	-0.00	0.00
95	->	53	-0.01	0.00
95	->	54	-0.00	0.00
95	->	55	-0.00	0.00
95	->	56	-0.00	0.00
95	->	57	-0.00	0.00
95	->	58	0.01	0.00
95	->	59	-0.01	0.00
95	->	60	-0.00	0.00
95	->	61	-0.00	0.00
95	->	62	0.00	0.00
95	->	63	0.01	0.00
95	->	64	0.01	0.00
95	->	65	0.00	0.00
95	->	66	0.00	0.00
95	->	67	0.00	0.00
95	->	68	-0.00	0.00
95	->	69	-0.01	0.00
95	->	70	-0.00	0.00
95	->	71	0.00	0.00
95	->	72	-0.01	0.00
95	->	73	-0.00	0.00
95	->	74	0.01	0.00
95	->	75	0.01	0.00
95	->	76	-0.02	0.01
95	->	77	-0.03	0.01
95	->	78	-0.01	0.00
95	->	79	-0.00	0.00
95	->	80	-0.00	0.00
95	->	81	-0.01	0.00
95	->	82	-0.00	0.00
95	->	83	-0.00	0.00
95	->	84	-0.00	0.00
95	->	85	-0.00	0.00
95	->	86	0.00	0.00
95	->	87	0.00	0.00
95	->	88	0.00	0.00

95	->	89	0.00	0.00
95	->	90	0.01	0.00
95	->	91	0.01	0.00
95	->	92	-0.01	0.01
95	->	93	-0.33	0.07
95	->	94	-32.63	0.90
95	->	95	-177.55	2.76
95	->	96	-36.07	0.91
95	->	97	-0.11	0.06
95	->	98	-1.88	0.79
95	->	99	-11.48	0.79
95	->	100	-4.79	1.04
95	->	101	-0.38	0.13
95	->	102	-0.05	0.05
95	->	103	0.01	0.00
95	->	104	0.02	0.00
95	->	105	0.01	0.00
95	->	106	0.01	0.00
95	->	107	0.00	0.00
96	->	1	-0.03	0.00
96	->	2	-0.00	0.00
96	->	3	-0.00	0.00
96	->	4	0.00	0.00
96	->	5	0.01	0.00
96	->	6	-0.00	0.00
96	->	7	-0.00	0.00
96	->	8	0.00	0.00
96	->	9	0.00	0.00
96	->	10	0.00	0.00
96	->	11	0.00	0.00
96	->	12	-0.01	0.00
96	->	13	-0.01	0.00
96	->	14	-0.00	0.00
96	->	15	-0.01	0.01
96	->	16	-1.77	0.77
96	->	17	-0.01	0.00
96	->	18	0.07	0.10
96	->	19	0.01	0.01
96	->	20	-0.00	0.00
96	->	21	-0.00	0.00
96	->	22	-0.03	0.01
96	->	23	-0.00	0.00
96	->	24	-0.00	0.00
96	->	25	-0.01	0.00
96	->	26	-0.00	0.00
96	->	27	-0.00	0.00
96	->	28	-0.00	0.00
96	->	29	-0.00	0.00
96	->	30	0.00	0.00
96	->	31	-0.00	0.00

96	->	32	-0.00	0.00
96	->	33	-0.00	0.00
96	->	34	0.00	0.00
96	->	35	0.00	0.00
96	->	36	-0.00	0.00
96	->	37	0.00	0.00
96	->	38	0.00	0.00
96	->	39	-0.00	0.00
96	->	40	-0.00	0.00
96	->	41	-0.00	0.00
96	->	42	-0.00	0.00
96	->	43	-0.00	0.00
96	->	44	-0.03	0.01
96	->	45	-0.00	0.00
96	->	46	0.01	0.00
96	->	47	-0.00	0.00
96	->	48	-0.00	0.00
96	->	49	-0.00	0.00
96	->	50	-0.00	0.00
96	->	51	-0.00	0.00
96	->	52	-0.00	0.00
96	->	53	-0.01	0.00
96	->	54	-0.00	0.00
96	->	55	-0.00	0.00
96	->	56	-0.00	0.00
96	->	57	-0.00	0.00
96	->	58	0.01	0.00
96	->	59	-0.00	0.00
96	->	60	-0.00	0.00
96	->	61	-0.00	0.00
96	->	62	0.00	0.00
96	->	63	0.01	0.00
96	->	64	0.01	0.00
96	->	65	0.00	0.00
96	->	66	0.00	0.00
96	->	67	0.00	0.00
96	->	68	-0.00	0.00
96	->	69	-0.01	0.00
96	->	70	-0.00	0.00
96	->	71	0.00	0.00
96	->	72	-0.01	0.00
96	->	73	-0.00	0.00
96	->	74	0.01	0.00
96	->	75	0.00	0.00
96	->	76	-0.01	0.01
96	->	77	-0.01	0.00
96	->	78	-0.00	0.00
96	->	79	-0.00	0.00
96	->	80	-0.00	0.00
96	->	81	-0.00	0.00

96	->	82	-0.00	0.00
96	->	83	-0.00	0.00
96	->	84	-0.00	0.00
96	->	85	-0.00	0.00
96	->	86	0.00	0.00
96	->	87	0.00	0.00
96	->	88	0.00	0.00
96	->	89	0.00	0.00
96	->	90	0.00	0.00
96	->	91	0.00	0.00
96	->	92	0.00	0.00
96	->	93	-0.02	0.01
96	->	94	-0.27	0.11
96	->	95	-35.81	0.91
96	->	96	-96.18	2.49
96	->	97	-1.08	1.05
96	->	98	-5.76	0.59
96	->	99	-0.46	0.26
96	->	100	-0.22	0.09
96	->	101	-0.05	0.06
96	->	102	0.00	0.01
96	->	103	0.01	0.00
96	->	104	0.01	0.00
96	->	105	0.00	0.00
96	->	106	0.00	0.00
96	->	107	0.00	0.00
97	->	1	-0.01	0.00
97	->	2	-0.00	0.00
97	->	3	-0.02	0.01
97	->	4	-0.00	0.00
97	->	5	0.00	0.00
97	->	6	-0.00	0.00
97	->	7	-0.00	0.00
97	->	8	0.00	0.00
97	->	9	-0.00	0.00
97	->	10	0.00	0.00
97	->	11	0.00	0.00
97	->	12	-0.00	0.00
97	->	13	-0.00	0.00
97	->	14	-0.01	0.00
97	->	15	-0.01	0.01
97	->	16	-0.38	0.43
97	->	17	-0.98	1.25
97	->	18	-1.28	1.30
97	->	19	-0.04	0.05
97	->	20	-0.06	0.08
97	->	21	-0.94	1.28
97	->	22	-0.04	0.03
97	->	23	-0.01	0.01
97	->	24	-0.02	0.02

97 ->	25	-0.38	0.47
97 ->	26	-0.00	0.00
97 ->	27	-0.00	0.00
97 ->	28	-0.00	0.00
97 ->	29	-0.00	0.00
97 ->	30	-0.00	0.00
97 ->	31	-0.01	0.00
97 ->	32	-0.01	0.01
97 ->	33	-0.01	0.00
97 ->	34	-0.00	0.00
97 ->	35	0.00	0.00
97 ->	36	-0.00	0.00
97 ->	37	0.00	0.00
97 ->	38	0.00	0.00
97 ->	39	0.00	0.00
97 ->	40	-0.00	0.00
97 ->	41	-0.00	0.00
97 ->	42	-0.00	0.00
97 ->	43	-0.00	0.00
97 ->	44	-0.07	0.10
97 ->	45	-0.01	0.00
97 ->	46	0.01	0.00
97 ->	47	-0.00	0.00
97 ->	48	-0.00	0.00
97 ->	49	-0.00	0.00
97 ->	50	-0.00	0.00
97 ->	51	-0.00	0.00
97 ->	52	-0.00	0.00
97 ->	53	-0.00	0.00
97 ->	54	-0.00	0.00
97 ->	55	0.00	0.00
97 ->	56	-0.00	0.00
97 ->	57	-0.00	0.00
97 ->	58	0.00	0.00
97 ->	59	-0.00	0.00
97 ->	60	-0.00	0.00
97 ->	61	-0.00	0.00
97 ->	62	0.00	0.00
97 ->	63	0.00	0.00
97 ->	64	0.00	0.00
97 ->	65	0.00	0.00
97 ->	66	0.00	0.00
97 ->	67	0.00	0.00
97 ->	68	-0.00	0.00
97 ->	69	-0.00	0.00
97 ->	70	0.00	0.00
97 ->	71	0.00	0.00
97 ->	72	-0.00	0.00
97 ->	73	-0.00	0.00
97 ->	74	0.00	0.00

97	->	75	0.00	0.00
97	->	76	-0.00	0.00
97	->	77	-0.00	0.00
97	->	78	-0.00	0.00
97	->	79	-0.00	0.00
97	->	80	-0.00	0.00
97	->	81	-0.00	0.00
97	->	82	-0.00	0.00
97	->	83	0.00	0.00
97	->	84	0.00	0.00
97	->	85	-0.00	0.00
97	->	86	0.00	0.00
97	->	87	0.00	0.00
97	->	88	0.00	0.00
97	->	89	0.00	0.00
97	->	90	0.00	0.00
97	->	91	0.00	0.00
97	->	92	0.00	0.00
97	->	93	-0.01	0.01
97	->	94	-0.02	0.02
97	->	95	-0.11	0.06
97	->	96	-1.05	1.03
97	->	97	-49.55	2.88
97	->	98	-34.96	1.06
97	->	99	-0.26	0.08
97	->	100	-0.03	0.01
97	->	101	-0.00	0.00
97	->	102	0.00	0.00
97	->	103	0.00	0.00
97	->	104	0.00	0.00
97	->	105	0.00	0.00
97	->	106	0.00	0.00
97	->	107	0.00	0.00
98	->	1	-0.03	0.01
98	->	2	-0.00	0.00
98	->	3	-0.01	0.00
98	->	4	0.00	0.00
98	->	5	0.02	0.00
98	->	6	-0.00	0.00
98	->	7	-0.00	0.00
98	->	8	0.01	0.00
98	->	9	-0.00	0.00
98	->	10	0.01	0.00
98	->	11	0.01	0.00
98	->	12	-0.02	0.01
98	->	13	-0.01	0.00
98	->	14	-0.01	0.00
98	->	15	-0.00	0.00
98	->	16	-0.33	1.14
98	->	17	-0.10	0.05

98 ->	18	-2.80	1.52
98 ->	19	0.00	0.03
98 ->	20	-0.05	0.04
98 ->	21	-0.47	0.61
98 ->	22	-0.20	0.17
98 ->	23	-0.01	0.01
98 ->	24	-0.03	0.04
98 ->	25	-3.23	3.96
98 ->	26	-0.00	0.00
98 ->	27	-0.00	0.00
98 ->	28	-0.00	0.00
98 ->	29	-0.00	0.00
98 ->	30	-0.01	0.02
98 ->	31	-0.04	0.02
98 ->	32	-0.04	0.03
98 ->	33	-0.12	0.19
98 ->	34	-0.00	0.00
98 ->	35	0.02	0.00
98 ->	36	-0.02	0.00
98 ->	37	0.00	0.00
98 ->	38	0.01	0.00
98 ->	39	0.00	0.00
98 ->	40	-0.01	0.00
98 ->	41	-0.01	0.00
98 ->	42	-0.01	0.01
98 ->	43	-0.01	0.02
98 ->	44	-0.92	1.41
98 ->	45	-0.31	0.33
98 ->	46	0.08	0.06
98 ->	47	-0.00	0.01
98 ->	48	-0.01	0.01
98 ->	49	-0.01	0.00
98 ->	50	-0.00	0.00
98 ->	51	-0.00	0.00
98 ->	52	-0.00	0.00
98 ->	53	-0.02	0.01
98 ->	54	-0.00	0.00
98 ->	55	-0.00	0.00
98 ->	56	-0.00	0.00
98 ->	57	-0.00	0.00
98 ->	58	0.01	0.00
98 ->	59	-0.01	0.00
98 ->	60	-0.00	0.00
98 ->	61	-0.00	0.00
98 ->	62	0.00	0.00
98 ->	63	0.00	0.00
98 ->	64	0.00	0.00
98 ->	65	0.00	0.00
98 ->	66	0.00	0.00
98 ->	67	0.00	0.00

98	->	68	-0.00	0.00
98	->	69	-0.00	0.00
98	->	70	0.00	0.00
98	->	71	0.00	0.00
98	->	72	-0.01	0.00
98	->	73	-0.00	0.00
98	->	74	0.01	0.00
98	->	75	0.01	0.00
98	->	76	-0.01	0.00
98	->	77	-0.01	0.00
98	->	78	-0.01	0.00
98	->	79	-0.00	0.00
98	->	80	-0.00	0.00
98	->	81	-0.00	0.00
98	->	82	-0.00	0.00
98	->	83	-0.00	0.00
98	->	84	-0.00	0.00
98	->	85	-0.00	0.00
98	->	86	0.00	0.00
98	->	87	0.00	0.00
98	->	88	0.01	0.00
98	->	89	0.01	0.00
98	->	90	0.02	0.00
98	->	91	0.03	0.01
98	->	92	0.02	0.01
98	->	93	-0.01	0.01
98	->	94	-0.15	0.08
98	->	95	-1.87	0.78
98	->	96	-5.79	0.58
98	->	97	-34.78	1.03
98	->	98	-130.63	4.65
98	->	99	-34.05	1.16
98	->	100	-0.30	0.07
98	->	101	-0.01	0.01
98	->	102	0.01	0.00
98	->	103	0.01	0.00
98	->	104	0.01	0.00
98	->	105	0.01	0.00
98	->	106	0.00	0.00
98	->	107	0.00	0.00
99	->	1	-0.02	0.00
99	->	2	-0.00	0.00
99	->	3	-0.00	0.00
99	->	4	0.00	0.00
99	->	5	0.01	0.00
99	->	6	-0.00	0.00
99	->	7	-0.00	0.00
99	->	8	0.00	0.00
99	->	9	-0.00	0.00
99	->	10	0.01	0.00

99	->	11	0.00	0.00
99	->	12	-0.01	0.00
99	->	13	-0.01	0.00
99	->	14	-0.00	0.00
99	->	15	-0.00	0.00
99	->	16	0.42	0.31
99	->	17	-0.01	0.01
99	->	18	-1.66	1.77
99	->	19	-0.04	0.04
99	->	20	-0.01	0.00
99	->	21	-0.01	0.01
99	->	22	-0.17	0.36
99	->	23	-0.00	0.00
99	->	24	-0.00	0.00
99	->	25	-0.21	0.20
99	->	26	-0.00	0.00
99	->	27	-0.00	0.00
99	->	28	-0.00	0.00
99	->	29	-0.00	0.00
99	->	30	-0.00	0.00
99	->	31	-0.04	0.02
99	->	32	-0.03	0.02
99	->	33	-0.46	0.35
99	->	34	-0.00	0.00
99	->	35	0.03	0.00
99	->	36	-0.03	0.01
99	->	37	0.00	0.00
99	->	38	0.02	0.00
99	->	39	-0.00	0.00
99	->	40	-0.01	0.00
99	->	41	-0.01	0.01
99	->	42	-0.02	0.02
99	->	43	-0.03	0.03
99	->	44	-1.29	1.48
99	->	45	-3.65	2.82
99	->	46	-0.06	0.12
99	->	47	-0.01	0.02
99	->	48	-0.06	0.03
99	->	49	-0.05	0.03
99	->	50	-0.01	0.00
99	->	51	-0.01	0.00
99	->	52	-0.01	0.00
99	->	53	-0.07	0.02
99	->	54	-0.00	0.00
99	->	55	-0.00	0.00
99	->	56	-0.00	0.00
99	->	57	-0.00	0.00
99	->	58	0.02	0.01
99	->	59	-0.01	0.00
99	->	60	-0.00	0.00

99	->	61	-0.00	0.00
99	->	62	0.00	0.00
99	->	63	0.01	0.00
99	->	64	0.00	0.00
99	->	65	0.00	0.00
99	->	66	0.00	0.00
99	->	67	0.00	0.00
99	->	68	-0.01	0.00
99	->	69	-0.01	0.00
99	->	70	-0.00	0.00
99	->	71	0.00	0.00
99	->	72	-0.02	0.00
99	->	73	-0.00	0.00
99	->	74	0.01	0.00
99	->	75	0.01	0.00
99	->	76	-0.02	0.01
99	->	77	-0.01	0.00
99	->	78	-0.01	0.00
99	->	79	-0.00	0.00
99	->	80	-0.00	0.00
99	->	81	-0.00	0.00
99	->	82	-0.00	0.00
99	->	83	0.00	0.00
99	->	84	-0.00	0.00
99	->	85	-0.00	0.00
99	->	86	0.00	0.00
99	->	87	0.01	0.00
99	->	88	0.01	0.00
99	->	89	0.02	0.00
99	->	90	0.01	0.00
99	->	91	0.01	0.01
99	->	92	-0.01	0.01
99	->	93	-0.10	0.04
99	->	94	-0.44	0.20
99	->	95	-11.44	0.80
99	->	96	-0.46	0.26
99	->	97	-0.26	0.08
99	->	98	-33.85	1.14
99	->	99	-211.71	5.48
99	->	100	-34.07	0.77
99	->	101	-0.15	0.04
99	->	102	0.00	0.00
99	->	103	0.01	0.00
99	->	104	0.01	0.00
99	->	105	0.01	0.00
99	->	106	0.00	0.00
99	->	107	0.00	0.00
100	->	1	-0.01	0.01
100	->	2	-0.00	0.00
100	->	3	-0.00	0.00

100	->	4	0.00	0.00
100	->	5	0.01	0.00
100	->	6	-0.00	0.00
100	->	7	-0.00	0.00
100	->	8	0.00	0.00
100	->	9	-0.00	0.00
100	->	10	0.01	0.00
100	->	11	0.00	0.00
100	->	12	-0.01	0.01
100	->	13	-0.01	0.00
100	->	14	-0.00	0.00
100	->	15	-0.00	0.00
100	->	16	0.27	0.16
100	->	17	-0.00	0.00
100	->	18	0.02	0.10
100	->	19	-0.01	0.05
100	->	20	-0.01	0.00
100	->	21	-0.01	0.00
100	->	22	-1.20	1.95
100	->	23	-0.01	0.00
100	->	24	-0.00	0.00
100	->	25	-0.06	0.01
100	->	26	-0.01	0.00
100	->	27	-0.00	0.00
100	->	28	-0.00	0.00
100	->	29	-0.00	0.00
100	->	30	0.00	0.00
100	->	31	-0.02	0.01
100	->	32	-0.01	0.00
100	->	33	-0.03	0.01
100	->	34	-0.00	0.00
100	->	35	0.03	0.00
100	->	36	-0.03	0.01
100	->	37	0.00	0.00
100	->	38	0.02	0.00
100	->	39	-0.00	0.00
100	->	40	-0.02	0.00
100	->	41	-0.00	0.00
100	->	42	-0.01	0.00
100	->	43	-0.01	0.01
100	->	44	-2.15	1.40
100	->	45	-1.10	1.81
100	->	46	-1.49	0.68
100	->	47	-0.05	0.03
100	->	48	-0.07	0.03
100	->	49	-0.26	0.12
100	->	50	-0.02	0.02
100	->	51	-0.01	0.00
100	->	52	-0.02	0.01
100	->	53	-0.56	0.53

100	->	54	-0.00	0.00
100	->	55	-0.00	0.00
100	->	56	-0.00	0.00
100	->	57	-0.00	0.00
100	->	58	0.04	0.02
100	->	59	-0.03	0.01
100	->	60	-0.02	0.02
100	->	61	-0.03	0.04
100	->	62	0.00	0.00
100	->	63	0.01	0.00
100	->	64	0.01	0.00
100	->	65	0.00	0.00
100	->	66	0.01	0.00
100	->	67	0.00	0.00
100	->	68	-0.01	0.00
100	->	69	-0.02	0.01
100	->	70	-0.00	0.00
100	->	71	-0.00	0.00
100	->	72	-0.05	0.02
100	->	73	-0.01	0.02
100	->	74	0.02	0.01
100	->	75	0.02	0.00
100	->	76	-0.04	0.03
100	->	77	-0.02	0.00
100	->	78	-0.01	0.00
100	->	79	-0.00	0.00
100	->	80	-0.00	0.00
100	->	81	-0.01	0.00
100	->	82	-0.00	0.00
100	->	83	0.00	0.00
100	->	84	-0.00	0.00
100	->	85	-0.00	0.00
100	->	86	0.00	0.00
100	->	87	0.01	0.00
100	->	88	0.01	0.00
100	->	89	0.02	0.00
100	->	90	0.01	0.00
100	->	91	0.01	0.01
100	->	92	-0.02	0.05
100	->	93	-0.38	0.86
100	->	94	-11.50	0.87
100	->	95	-4.78	1.04
100	->	96	-0.22	0.09
100	->	97	-0.03	0.01
100	->	98	-0.30	0.07
100	->	99	-33.81	0.76
100	->	100	-176.03	3.00
100	->	101	-35.72	1.02
100	->	102	-0.39	0.09
100	->	103	-0.04	0.01

100	->	104	0.00	0.00
100	->	105	0.01	0.00
100	->	106	0.00	0.00
100	->	107	0.00	0.00
101	->	1	-0.03	0.00
101	->	2	-0.00	0.00
101	->	3	-0.00	0.00
101	->	4	0.00	0.00
101	->	5	0.01	0.00
101	->	6	-0.00	0.00
101	->	7	-0.00	0.00
101	->	8	0.00	0.00
101	->	9	0.00	0.00
101	->	10	0.01	0.00
101	->	11	0.01	0.00
101	->	12	-0.02	0.00
101	->	13	-0.01	0.00
101	->	14	-0.00	0.00
101	->	15	-0.00	0.00
101	->	16	-0.07	0.03
101	->	17	-0.00	0.00
101	->	18	0.01	0.01
101	->	19	0.01	0.02
101	->	20	-0.00	0.00
101	->	21	-0.00	0.00
101	->	22	0.09	0.17
101	->	23	-0.00	0.00
101	->	24	-0.00	0.00
101	->	25	-0.02	0.00
101	->	26	-0.00	0.00
101	->	27	-0.00	0.00
101	->	28	-0.00	0.00
101	->	29	-0.00	0.00
101	->	30	0.00	0.00
101	->	31	-0.01	0.00
101	->	32	-0.00	0.00
101	->	33	-0.01	0.00
101	->	34	-0.00	0.00
101	->	35	0.02	0.00
101	->	36	-0.02	0.01
101	->	37	0.00	0.00
101	->	38	0.01	0.00
101	->	39	-0.00	0.00
101	->	40	-0.01	0.00
101	->	41	-0.00	0.00
101	->	42	-0.00	0.00
101	->	43	0.00	0.01
101	->	44	0.73	0.95
101	->	45	-0.04	0.03
101	->	46	-3.65	1.99

101	->	47	-0.16	0.06
101	->	48	-0.02	0.01
101	->	49	-0.20	0.14
101	->	50	-0.02	0.03
101	->	51	-0.01	0.00
101	->	52	-0.01	0.01
101	->	53	-2.95	3.19
101	->	54	-0.00	0.00
101	->	55	-0.00	0.00
101	->	56	-0.00	0.00
101	->	57	-0.00	0.00
101	->	58	0.05	0.02
101	->	59	-0.06	0.02
101	->	60	-0.10	0.11
101	->	61	-0.88	1.05
101	->	62	0.00	0.00
101	->	63	0.02	0.01
101	->	64	0.02	0.01
101	->	65	0.00	0.00
101	->	66	0.02	0.00
101	->	67	0.00	0.00
101	->	68	-0.02	0.00
101	->	69	-0.04	0.02
101	->	70	-0.01	0.01
101	->	71	-0.02	0.01
101	->	72	-0.14	0.07
101	->	73	-0.30	0.41
101	->	74	0.04	0.02
101	->	75	0.02	0.01
101	->	76	-0.51	0.65
101	->	77	-0.05	0.02
101	->	78	-0.02	0.01
101	->	79	-0.00	0.00
101	->	80	-0.00	0.00
101	->	81	-0.02	0.01
101	->	82	-0.00	0.00
101	->	83	-0.00	0.00
101	->	84	-0.00	0.00
101	->	85	-0.00	0.00
101	->	86	0.00	0.00
101	->	87	0.01	0.00
101	->	88	0.01	0.00
101	->	89	0.01	0.00
101	->	90	-0.00	0.01
101	->	91	-0.12	0.08
101	->	92	-1.82	0.78
101	->	93	-11.28	1.35
101	->	94	0.29	0.25
101	->	95	-0.38	0.13
101	->	96	-0.05	0.06

101	->	97	-0.00	0.00
101	->	98	-0.01	0.01
101	->	99	-0.15	0.04
101	->	100	-35.54	1.00
101	->	101	-210.94	4.33
101	->	102	-32.08	0.76
101	->	103	-0.13	0.04
101	->	104	-0.00	0.01
101	->	105	0.01	0.00
101	->	106	0.01	0.00
101	->	107	0.00	0.00
102	->	1	-0.05	0.01
102	->	2	-0.00	0.00
102	->	3	-0.00	0.00
102	->	4	-0.00	0.00
102	->	5	0.01	0.00
102	->	6	-0.00	0.00
102	->	7	-0.00	0.00
102	->	8	0.00	0.00
102	->	9	0.00	0.00
102	->	10	0.01	0.00
102	->	11	0.01	0.00
102	->	12	-0.03	0.01
102	->	13	-0.01	0.00
102	->	14	-0.00	0.00
102	->	15	-0.00	0.00
102	->	16	-0.05	0.01
102	->	17	-0.00	0.00
102	->	18	0.02	0.00
102	->	19	0.03	0.01
102	->	20	-0.00	0.00
102	->	21	-0.00	0.00
102	->	22	-0.06	0.03
102	->	23	-0.00	0.00
102	->	24	-0.00	0.00
102	->	25	-0.01	0.00
102	->	26	-0.00	0.00
102	->	27	-0.00	0.00
102	->	28	-0.00	0.00
102	->	29	-0.00	0.00
102	->	30	0.00	0.00
102	->	31	-0.01	0.00
102	->	32	-0.00	0.00
102	->	33	-0.00	0.00
102	->	34	-0.00	0.00
102	->	35	0.01	0.00
102	->	36	-0.01	0.00
102	->	37	0.00	0.00
102	->	38	0.01	0.00
102	->	39	-0.00	0.00

102	->	40	-0.01	0.00
102	->	41	-0.00	0.00
102	->	42	-0.00	0.00
102	->	43	-0.00	0.00
102	->	44	0.10	0.42
102	->	45	-0.01	0.00
102	->	46	-0.70	0.59
102	->	47	-0.08	0.09
102	->	48	-0.01	0.00
102	->	49	-0.02	0.01
102	->	50	-0.07	0.05
102	->	51	-0.01	0.00
102	->	52	-0.00	0.00
102	->	53	-0.28	0.88
102	->	54	-0.00	0.00
102	->	55	-0.00	0.00
102	->	56	-0.00	0.00
102	->	57	-0.00	0.00
102	->	58	0.03	0.01
102	->	59	-0.05	0.02
102	->	60	-0.04	0.04
102	->	61	-0.50	0.58
102	->	62	0.00	0.01
102	->	63	0.04	0.02
102	->	64	0.03	0.01
102	->	65	-0.00	0.00
102	->	66	0.02	0.01
102	->	67	-0.00	0.00
102	->	68	-0.02	0.01
102	->	69	-0.05	0.02
102	->	70	-0.02	0.01
102	->	71	-0.04	0.02
102	->	72	-0.34	0.18
102	->	73	-2.60	2.11
102	->	74	-0.03	0.13
102	->	75	0.00	0.02
102	->	76	-3.75	4.85
102	->	77	-0.73	0.56
102	->	78	-0.04	0.01
102	->	79	-0.01	0.01
102	->	80	-0.01	0.01
102	->	81	-0.04	0.02
102	->	82	-0.00	0.00
102	->	83	-0.00	0.00
102	->	84	-0.00	0.00
102	->	85	-0.00	0.01
102	->	86	0.00	0.00
102	->	87	0.01	0.00
102	->	88	0.00	0.00
102	->	89	-0.00	0.00

102	->	90	-0.03	0.02
102	->	91	-0.84	0.48
102	->	92	-11.85	0.63
102	->	93	-2.74	0.88
102	->	94	0.04	0.06
102	->	95	-0.05	0.05
102	->	96	0.00	0.01
102	->	97	0.00	0.00
102	->	98	0.01	0.00
102	->	99	0.00	0.00
102	->	100	-0.39	0.09
102	->	101	-31.85	0.76
102	->	102	-210.08	4.68
102	->	103	-31.86	1.16
102	->	104	-0.16	0.06
102	->	105	0.00	0.00
102	->	106	0.01	0.00
102	->	107	0.00	0.00
103	->	1	-0.06	0.01
103	->	2	-0.00	0.00
103	->	3	-0.00	0.00
103	->	4	-0.00	0.00
103	->	5	0.01	0.00
103	->	6	-0.00	0.00
103	->	7	-0.00	0.00
103	->	8	0.00	0.00
103	->	9	0.00	0.00
103	->	10	0.01	0.00
103	->	11	0.01	0.00
103	->	12	-0.05	0.01
103	->	13	-0.01	0.00
103	->	14	-0.00	0.00
103	->	15	-0.00	0.00
103	->	16	-0.03	0.00
103	->	17	-0.00	0.00
103	->	18	0.02	0.00
103	->	19	0.03	0.01
103	->	20	-0.00	0.00
103	->	21	-0.00	0.00
103	->	22	-0.08	0.03
103	->	23	-0.00	0.00
103	->	24	-0.00	0.00
103	->	25	-0.01	0.00
103	->	26	-0.00	0.00
103	->	27	-0.00	0.00
103	->	28	-0.00	0.00
103	->	29	-0.00	0.00
103	->	30	0.00	0.00
103	->	31	-0.01	0.00
103	->	32	-0.00	0.00

103	->	33	-0.00	0.00
103	->	34	-0.00	0.00
103	->	35	0.01	0.00
103	->	36	-0.00	0.00
103	->	37	0.00	0.00
103	->	38	0.01	0.00
103	->	39	0.00	0.00
103	->	40	-0.01	0.00
103	->	41	-0.00	0.00
103	->	42	-0.01	0.00
103	->	43	-0.01	0.00
103	->	44	-0.12	0.10
103	->	45	-0.00	0.00
103	->	46	-0.04	0.07
103	->	47	-0.14	0.19
103	->	48	-0.00	0.00
103	->	49	-0.00	0.00
103	->	50	-0.04	0.05
103	->	51	-0.01	0.00
103	->	52	-0.00	0.00
103	->	53	-0.03	0.01
103	->	54	-0.00	0.00
103	->	55	-0.00	0.00
103	->	56	-0.00	0.00
103	->	57	-0.00	0.00
103	->	58	0.01	0.00
103	->	59	-0.02	0.01
103	->	60	-0.00	0.00
103	->	61	-0.05	0.09
103	->	62	0.00	0.00
103	->	63	0.04	0.01
103	->	64	0.03	0.01
103	->	65	0.00	0.00
103	->	66	0.02	0.00
103	->	67	-0.00	0.00
103	->	68	-0.02	0.01
103	->	69	-0.02	0.01
103	->	70	-0.01	0.01
103	->	71	-0.02	0.01
103	->	72	-0.34	0.21
103	->	73	-1.49	1.66
103	->	74	-1.28	1.17
103	->	75	-0.01	0.03
103	->	76	-1.48	2.47
103	->	77	-6.26	4.15
103	->	78	-0.09	0.02
103	->	79	-0.02	0.01
103	->	80	-0.04	0.04
103	->	81	-0.17	0.26
103	->	82	-0.00	0.00

103	->	83	-0.00	0.00
103	->	84	-0.01	0.00
103	->	85	-0.00	0.02
103	->	86	0.00	0.00
103	->	87	0.01	0.00
103	->	88	-0.01	0.01
103	->	89	-0.07	0.03
103	->	90	-0.40	0.22
103	->	91	-11.52	0.62
103	->	92	-4.09	1.01
103	->	93	-0.36	0.13
103	->	94	0.00	0.02
103	->	95	0.01	0.00
103	->	96	0.01	0.00
103	->	97	0.00	0.00
103	->	98	0.01	0.00
103	->	99	0.01	0.00
103	->	100	-0.04	0.01
103	->	101	-0.13	0.04
103	->	102	-31.66	1.15
103	->	103	-208.88	4.07
103	->	104	-32.71	1.00
103	->	105	-0.11	0.04
103	->	106	0.01	0.01
103	->	107	0.00	0.00
104	->	1	-0.05	0.01
104	->	2	-0.00	0.00
104	->	3	-0.00	0.00
104	->	4	-0.00	0.00
104	->	5	0.01	0.00
104	->	6	-0.00	0.00
104	->	7	-0.00	0.00
104	->	8	0.01	0.00
104	->	9	0.00	0.00
104	->	10	0.01	0.00
104	->	11	0.02	0.01
104	->	12	-0.06	0.03
104	->	13	-0.01	0.00
104	->	14	-0.00	0.00
104	->	15	-0.00	0.00
104	->	16	-0.02	0.00
104	->	17	0.00	0.00
104	->	18	0.01	0.00
104	->	19	0.02	0.01
104	->	20	-0.00	0.00
104	->	21	-0.00	0.00
104	->	22	-0.05	0.02
104	->	23	-0.01	0.00
104	->	24	-0.00	0.00
104	->	25	-0.01	0.00

104 ->	26	-0.00	0.00
104 ->	27	-0.00	0.00
104 ->	28	-0.00	0.00
104 ->	29	-0.00	0.00
104 ->	30	0.00	0.00
104 ->	31	-0.01	0.00
104 ->	32	-0.00	0.00
104 ->	33	-0.00	0.00
104 ->	34	-0.00	0.00
104 ->	35	0.01	0.00
104 ->	36	-0.00	0.00
104 ->	37	0.00	0.00
104 ->	38	0.01	0.00
104 ->	39	-0.00	0.00
104 ->	40	-0.01	0.00
104 ->	41	-0.00	0.00
104 ->	42	-0.01	0.01
104 ->	43	-0.00	0.00
104 ->	44	-0.06	0.04
104 ->	45	-0.00	0.00
104 ->	46	0.01	0.01
104 ->	47	-0.06	0.04
104 ->	48	-0.00	0.00
104 ->	49	-0.00	0.00
104 ->	50	-0.02	0.02
104 ->	51	-0.01	0.00
104 ->	52	-0.00	0.00
104 ->	53	-0.02	0.00
104 ->	54	-0.00	0.00
104 ->	55	-0.00	0.00
104 ->	56	-0.00	0.00
104 ->	57	-0.00	0.00
104 ->	58	0.01	0.00
104 ->	59	-0.02	0.00
104 ->	60	-0.00	0.00
104 ->	61	-0.01	0.01
104 ->	62	0.00	0.00
104 ->	63	0.02	0.00
104 ->	64	0.02	0.00
104 ->	65	0.00	0.00
104 ->	66	0.02	0.00
104 ->	67	-0.00	0.00
104 ->	68	-0.02	0.00
104 ->	69	-0.01	0.00
104 ->	70	-0.01	0.00
104 ->	71	-0.00	0.01
104 ->	72	-0.29	0.82
104 ->	73	-0.25	0.37
104 ->	74	-2.47	1.48
104 ->	75	-0.00	0.06

104 ->	76	-0.14	0.08
104 ->	77	-5.24	2.89
104 ->	78	-0.22	0.14
104 ->	79	-0.01	0.01
104 ->	80	-0.02	0.02
104 ->	81	-0.84	1.86
104 ->	82	-0.01	0.00
104 ->	83	-0.01	0.00
104 ->	84	-0.01	0.00
104 ->	85	-0.01	0.03
104 ->	86	-0.00	0.00
104 ->	87	-0.01	0.01
104 ->	88	-0.10	0.05
104 ->	89	-1.76	0.93
104 ->	90	-5.80	0.75
104 ->	91	-4.17	0.79
104 ->	92	-0.43	0.13
104 ->	93	-0.05	0.05
104 ->	94	0.01	0.00
104 ->	95	0.02	0.00
104 ->	96	0.01	0.00
104 ->	97	0.00	0.00
104 ->	98	0.01	0.00
104 ->	99	0.01	0.00
104 ->	100	0.00	0.00
104 ->	101	-0.00	0.01
104 ->	102	-0.16	0.06
104 ->	103	-32.49	0.98
104 ->	104	-129.22	3.65
104 ->	105	-34.24	0.99
104 ->	106	-0.26	0.07
104 ->	107	-0.01	0.01
105 ->	1	-0.05	0.01
105 ->	2	-0.00	0.00
105 ->	3	0.00	0.00
105 ->	4	-0.00	0.00
105 ->	5	0.01	0.00
105 ->	6	-0.00	0.00
105 ->	7	-0.00	0.00
105 ->	8	0.01	0.00
105 ->	9	0.00	0.00
105 ->	10	0.02	0.01
105 ->	11	0.03	0.01
105 ->	12	-0.07	0.03
105 ->	13	-0.01	0.00
105 ->	14	-0.00	0.00
105 ->	15	-0.00	0.00
105 ->	16	-0.01	0.00
105 ->	17	0.00	0.00
105 ->	18	0.01	0.00

105	->	19	0.02	0.01
105	->	20	0.00	0.00
105	->	21	-0.00	0.00
105	->	22	-0.04	0.01
105	->	23	-0.01	0.00
105	->	24	-0.00	0.00
105	->	25	-0.01	0.00
105	->	26	-0.00	0.00
105	->	27	-0.00	0.00
105	->	28	-0.00	0.00
105	->	29	-0.00	0.00
105	->	30	0.00	0.00
105	->	31	-0.02	0.01
105	->	32	-0.00	0.00
105	->	33	-0.00	0.00
105	->	34	-0.00	0.00
105	->	35	0.01	0.00
105	->	36	-0.00	0.00
105	->	37	0.00	0.00
105	->	38	0.01	0.00
105	->	39	0.00	0.00
105	->	40	-0.02	0.00
105	->	41	-0.00	0.00
105	->	42	-0.01	0.00
105	->	43	-0.00	0.00
105	->	44	-0.03	0.02
105	->	45	-0.00	0.00
105	->	46	0.02	0.00
105	->	47	-0.01	0.00
105	->	48	-0.00	0.00
105	->	49	-0.00	0.00
105	->	50	-0.01	0.00
105	->	51	-0.01	0.00
105	->	52	-0.00	0.00
105	->	53	-0.01	0.00
105	->	54	-0.00	0.00
105	->	55	-0.00	0.00
105	->	56	-0.00	0.00
105	->	57	-0.00	0.00
105	->	58	0.01	0.00
105	->	59	-0.01	0.00
105	->	60	-0.00	0.00
105	->	61	-0.00	0.00
105	->	62	0.00	0.00
105	->	63	0.01	0.00
105	->	64	0.01	0.00
105	->	65	0.00	0.00
105	->	66	0.01	0.00
105	->	67	-0.00	0.00
105	->	68	-0.01	0.01

105	->	69	-0.01	0.00
105	->	70	-0.00	0.00
105	->	71	-0.00	0.00
105	->	72	0.44	0.36
105	->	73	-0.00	0.01
105	->	74	-2.21	1.79
105	->	75	-0.09	0.16
105	->	76	-0.02	0.01
105	->	77	-0.21	0.27
105	->	78	-1.04	1.30
105	->	79	-0.01	0.00
105	->	80	-0.01	0.00
105	->	81	-0.18	0.21
105	->	82	-0.01	0.00
105	->	83	-0.00	0.00
105	->	84	-0.00	0.00
105	->	85	-0.02	0.04
105	->	86	-0.02	0.01
105	->	87	-0.12	0.05
105	->	88	-0.51	0.31
105	->	89	-11.61	0.63
105	->	90	-0.71	0.64
105	->	91	-0.27	0.08
105	->	92	-0.03	0.03
105	->	93	0.02	0.01
105	->	94	0.02	0.00
105	->	95	0.01	0.00
105	->	96	0.00	0.00
105	->	97	0.00	0.00
105	->	98	0.01	0.00
105	->	99	0.01	0.00
105	->	100	0.01	0.00
105	->	101	0.01	0.00
105	->	102	0.00	0.00
105	->	103	-0.11	0.04
105	->	104	-34.00	0.99
105	->	105	-216.02	2.88
105	->	106	-34.07	1.22
105	->	107	-0.18	0.05
106	->	1	-0.03	0.00
106	->	2	-0.00	0.00
106	->	3	0.00	0.00
106	->	4	-0.00	0.00
106	->	5	0.01	0.00
106	->	6	-0.00	0.00
106	->	7	-0.00	0.00
106	->	8	0.01	0.00
106	->	9	0.00	0.00
106	->	10	0.02	0.01
106	->	11	0.02	0.01

106 ->	12	-0.04	0.01
106 ->	13	-0.01	0.00
106 ->	14	-0.00	0.00
106 ->	15	-0.00	0.00
106 ->	16	-0.01	0.00
106 ->	17	0.00	0.00
106 ->	18	0.01	0.00
106 ->	19	0.02	0.00
106 ->	20	0.00	0.00
106 ->	21	0.00	0.00
106 ->	22	-0.02	0.00
106 ->	23	-0.00	0.00
106 ->	24	0.00	0.00
106 ->	25	-0.01	0.00
106 ->	26	-0.00	0.00
106 ->	27	-0.00	0.00
106 ->	28	-0.00	0.00
106 ->	29	-0.00	0.00
106 ->	30	-0.00	0.00
106 ->	31	-0.02	0.01
106 ->	32	-0.00	0.00
106 ->	33	-0.00	0.00
106 ->	34	-0.00	0.00
106 ->	35	0.00	0.00
106 ->	36	-0.00	0.00
106 ->	37	-0.00	0.00
106 ->	38	0.00	0.00
106 ->	39	-0.00	0.00
106 ->	40	-0.01	0.01
106 ->	41	-0.00	0.00
106 ->	42	-0.01	0.00
106 ->	43	-0.00	0.00
106 ->	44	-0.02	0.01
106 ->	45	-0.00	0.00
106 ->	46	0.01	0.00
106 ->	47	-0.01	0.00
106 ->	48	-0.00	0.00
106 ->	49	-0.00	0.00
106 ->	50	-0.00	0.00
106 ->	51	-0.01	0.00
106 ->	52	-0.00	0.00
106 ->	53	-0.01	0.00
106 ->	54	-0.00	0.00
106 ->	55	-0.00	0.00
106 ->	56	-0.00	0.00
106 ->	57	-0.00	0.00
106 ->	58	0.00	0.00
106 ->	59	-0.01	0.00
106 ->	60	-0.00	0.00
106 ->	61	-0.00	0.00

106	->	62	0.00	0.00
106	->	63	0.01	0.00
106	->	64	0.01	0.00
106	->	65	0.00	0.00
106	->	66	0.01	0.00
106	->	67	-0.00	0.00
106	->	68	-0.02	0.02
106	->	69	-0.01	0.00
106	->	70	-0.00	0.00
106	->	71	-0.00	0.00
106	->	72	0.10	0.15
106	->	73	-0.00	0.00
106	->	74	0.09	0.18
106	->	75	0.02	0.02
106	->	76	-0.02	0.00
106	->	77	-0.05	0.02
106	->	78	-3.67	3.04
106	->	79	-0.01	0.00
106	->	80	-0.00	0.00
106	->	81	-0.07	0.02
106	->	82	-0.01	0.00
106	->	83	-0.00	0.00
106	->	84	-0.00	0.00
106	->	85	-0.01	0.02
106	->	86	-0.18	0.09
106	->	87	-0.95	0.63
106	->	88	-11.46	0.86
106	->	89	-4.51	1.06
106	->	90	-0.23	0.12
106	->	91	-0.03	0.03
106	->	92	0.01	0.01
106	->	93	0.02	0.00
106	->	94	0.01	0.00
106	->	95	0.01	0.00
106	->	96	0.00	0.00
106	->	97	0.00	0.00
106	->	98	0.00	0.00
106	->	99	0.00	0.00
106	->	100	0.00	0.00
106	->	101	0.01	0.00
106	->	102	0.01	0.00
106	->	103	0.01	0.01
106	->	104	-0.26	0.07
106	->	105	-33.78	1.22
106	->	106	-180.31	2.97
106	->	107	-36.50	0.81
107	->	1	-0.01	0.00
107	->	2	0.00	0.00
107	->	3	0.00	0.00
107	->	4	-0.00	0.00

107	->	5	0.01	0.00
107	->	6	-0.00	0.00
107	->	7	0.00	0.00
107	->	8	0.01	0.00
107	->	9	-0.00	0.00
107	->	10	0.01	0.00
107	->	11	0.01	0.00
107	->	12	-0.02	0.01
107	->	13	-0.00	0.00
107	->	14	-0.00	0.00
107	->	15	-0.00	0.00
107	->	16	-0.00	0.00
107	->	17	0.00	0.00
107	->	18	0.00	0.00
107	->	19	0.01	0.00
107	->	20	0.00	0.00
107	->	21	0.00	0.00
107	->	22	-0.01	0.00
107	->	23	-0.00	0.00
107	->	24	0.00	0.00
107	->	25	-0.01	0.00
107	->	26	-0.00	0.00
107	->	27	-0.00	0.00
107	->	28	-0.00	0.00
107	->	29	-0.00	0.00
107	->	30	0.00	0.00
107	->	31	-0.02	0.01
107	->	32	-0.00	0.00
107	->	33	0.00	0.00
107	->	34	-0.00	0.00
107	->	35	0.01	0.00
107	->	36	-0.00	0.00
107	->	37	0.00	0.00
107	->	38	0.01	0.00
107	->	39	-0.00	0.00
107	->	40	-0.03	0.01
107	->	41	-0.00	0.00
107	->	42	-0.00	0.00
107	->	43	-0.00	0.00
107	->	44	-0.01	0.00
107	->	45	0.00	0.00
107	->	46	0.01	0.00
107	->	47	-0.00	0.00
107	->	48	-0.00	0.00
107	->	49	-0.00	0.00
107	->	50	-0.00	0.00
107	->	51	-0.00	0.00
107	->	52	-0.00	0.00
107	->	53	-0.01	0.00
107	->	54	-0.00	0.00

107	->	55	-0.00	0.00
107	->	56	-0.00	0.00
107	->	57	-0.00	0.00
107	->	58	0.00	0.00
107	->	59	-0.01	0.00
107	->	60	-0.00	0.00
107	->	61	-0.00	0.00
107	->	62	0.00	0.00
107	->	63	0.00	0.00
107	->	64	0.00	0.00
107	->	65	0.00	0.00
107	->	66	0.00	0.00
107	->	67	-0.00	0.00
107	->	68	-0.01	0.01
107	->	69	-0.00	0.00
107	->	70	-0.00	0.00
107	->	71	-0.00	0.00
107	->	72	-0.05	0.01
107	->	73	-0.00	0.00
107	->	74	0.00	0.02
107	->	75	0.01	0.01
107	->	76	-0.01	0.00
107	->	77	-0.01	0.00
107	->	78	-0.08	0.42
107	->	79	-0.00	0.00
107	->	80	-0.00	0.00
107	->	81	-0.01	0.00
107	->	82	-0.00	0.00
107	->	83	-0.00	0.00
107	->	84	-0.00	0.00
107	->	85	-0.00	0.01
107	->	86	-2.12	1.16
107	->	87	-10.55	0.80
107	->	88	0.17	0.20
107	->	89	-0.26	0.07
107	->	90	-0.03	0.02
107	->	91	0.01	0.01
107	->	92	0.01	0.00
107	->	93	0.01	0.00
107	->	94	0.00	0.00
107	->	95	0.00	0.00
107	->	96	0.00	0.00
107	->	97	0.00	0.00
107	->	98	0.00	0.00
107	->	99	0.00	0.00
107	->	100	0.00	0.00
107	->	101	0.00	0.00
107	->	102	0.00	0.00
107	->	103	0.00	0.00
107	->	104	-0.01	0.01

107 ->	105	-0.18	0.05
107 ->	106	-36.25	0.82
107 ->	107	-215.77	2.75
